# Land Use Land Cover Labeling of GLOBE Images Using a Deep Learning Fusion Model

**DOI:** 10.3390/s22186895

**Published:** 2022-09-13

**Authors:** Sergio Manzanarez, Vidya Manian, Marvin Santos

**Affiliations:** Department of Electrical and Computer Engineering, University of Puerto Rico, Mayaguez, PR 00681, USA

**Keywords:** land use land cover classification, image segmentation, fusion model, GLOBE database, deep learning, DeepLab, UNet

## Abstract

Most of the land use land cover classification methods presented in the literature have been conducted using satellite remote sensing images. High-resolution aerial imagery is now being used for land cover classification. The Global Learning and Observations to Benefit, the Environment land cover image database, is created by citizen scientists worldwide who use their handheld cameras to take a set of six images per land cover site. These images have clutter due to man-made objects, and the pixel uncertainties result in incorrect labels. The problem of accurate labeling of these land cover images is addressed. An integrated architecture that combines Unet and DeepLabV3 for initial segmentation, followed by a weighted fusion model that combines the segmentation labels, is presented. The land cover images with labels are used for training the deep learning models. The fusion model combines the labels of five images taken from the north, south, east, west, and down directions to assign a unique label to the image sets. 2916 GLOBE images have been labeled with land cover classes using the integrated model with minimal human-in-the-loop annotation. The validation step shows that our architecture of labeling the images results in 90.97% label accuracy. Our fusion model can be used for labeling large databases of land cover classes from RGB images.

## 1. Introduction

Land cover refers to the biological material on the surface of the Earth; on the other hand, it describes how people utilize the land and socio-economic activity. It also shows how people use the landscape for development, agricultural, or mixed uses. Thus, the Land Use Land Cover (LULC) refers to classifying the human activities or elements covering the Earth using scientific and statistical methods.

LULC classification methods are broadly categorized as pixel-based or object-based methods. Traditionally, the LULC was conducted manually, later moved to digital technology using the GIS technology, and recently, the Artificial Intelligence (AI) revolution has led to the emergence of new algorithms. A review of machine learning methods using feature extraction for supervised and unsupervised LULC classification is given in [1]. The AI methods for classification follow hard classification performed by traditional Machine Learning (ML) methods, which can be supervised, unsupervised, or semi-supervised; the second approach is soft classification. Recently, Deep Learning (DL) architectures are being widely used with satellite remote sensing images. High level feature extraction using the Deep Boltzman Machine (DBM) and low-level feature extraction using Principal Component Analysis (PCA) and Random Forest (RF) are compared in [2] for land cover classification from multispectral Lidar data. A hierarchical clustering method is Bag of Words (BoW), which is used to represent unstructured data as words. In [3], the BoW and Probabilistic Topic Model are used for extracting latent features for scene classification. The conventional neural networks with a few layers such as AlexNet, CaffeNet, and VGGNet have depth limitations that cannot gather semantic information, making them unsuitable for LULC classification. Moreover, a particular deep network is not versatile enough to tackle all LULC labeling problems. Hence, AlexNet, Inception-v3, and ResNet18 are combined in [4] with a new fully connected layer and a softmax layer to generate the LC labels. A transfer learning approach is presented in [5], where a pretrained network is used both in the feature descriptor and classifier stages. This approach is applied to aerial orthophotographs. A comparative study of transfer learning-based deep networks are given in [6]. The model performance is improved using enhancement techniques such as early stopping, gradient clipping, adaptive learning rates, and data augmentation. The model is applied for the LULC classification of satellite imagery.

The same scene in an image of a land cover may have complex spatial and structural patterns. In contrast, different land-use types may have a similar reflectance spectrum and texture. It becomes challenging to obtain a precise classification of the land use type due to high intraclass heterogeneity, and low interclass diversity [4]. We observe from the above that LULC classification is conducted on satellite imagery, and high resolution aerial photographs. Whereas, the GLOBE database is composed of land cover images taken by handheld cameras and have a higher resolution than a satellite or aerial image. Hence, we propose a segmentation pipeline that is novel in the following ways for labeling of these images:

1. The images are preprocessed using a pretrained DeepLabV3 architecture for removing man-made objects with a mask. Four architectures: DeepLabV3, DeepLabV3+, UNet, and UNet+ are used for segmentation.
2. The segmented directional images are combined in a decision block using a weighted fusion method to generate a label for the most common land cover class of the group of images.

In this paper, we present an integrated deep learning model for segmenting directional LULC images, combining the segmented output for generating unique labels to groups of images. The rest of the paper is organized as follows. Section 2 presents the preprocessing methods applied to the GLOBE images, architecture of the integrated UNet, UNet++, DeepLabV3, DeepLabV3+ models, and final labeling stage. Section 3 presents the results, while Section 4 discusses the results and compares them with those of the state-of-the-art methods. The conclusions are provided in Section 5.

## 2. Materials and Methods

This paper uses CNN-based UNet, Unet++, DeepLabV3, and DeepLabV3+ for semantic segmentation on the GLOBE Dataset.

### 2.1. GLOBE Image Database

The Global Learning and Observations to Benefit the Environment (GLOBE) application allows the public who are called citizen scientists to make observations of the environment using their camera and post the images in the GLOBE database. It is an open dataset available to researchers, students, and the scientific community for performing their investigation on different domains, such as the atmosphere, biosphere, hydrosphere, and pedosphere. The visualization tool [7] includes biometry with tree height data, land cover photos, lilac phenology, and carbon cycle with stored carbon and tree diversity. The atmosphere domain has data on air temperature, aerosols, clouds, precipitation, humidity, surface ozone, surface temperatures, and water vapor. GLOBE Observer (GO) land cover data collection began in September 2018. The observers voluntarily assign the land cover types to the images and are not accurate. The observers use the GO app to upload their photos. The GLOBE land cover data aims to verify remote sensing land cover classifications. The GO summary data consists of the following information: Land cover id, data source, measured at location, overall land cover classification, field notes, description of the six photos taken at the north, south, east, west, up, and down directions.

**Figure 1:**
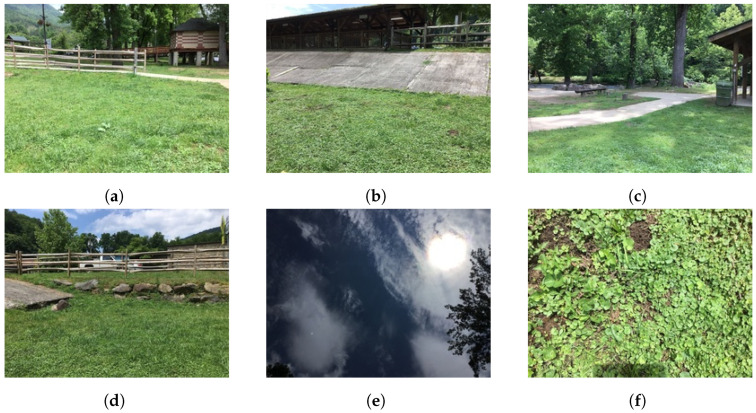
Sample images from GLOBE Visualization tool. (**a**) North, (**b**) South, (**c**) East, (**d**) West, (**e**) Up, (**f**) Down.

The series of six photos taken by observers are quality checked. The citizen scientists who capture the photos are kept anonymous. Many applications of the GLOBE land cover data are listed in [8]. The photos in Figure 1 are taken at latitude 35.47° and longitude −83.32°. The volunteers do not always label the image with the land cover type, and many labels are erroneously given, as they do not have experience in land cover classification. However, this is an endeavor that is beneficial for many applications such as urban planing [9,10] and environmental protection [11].

### 2.2. Image Preprocessing

Since GLOBE is an international science and education program that allows students, teachers, citizen scientists, and the public worldwide to participate in data collection from anywhere on the planet, the collaborators contribute by taking images in the four cardinal points and the directions up and down. These images are varied, from blurry to out of focus and with different sizes, so we created a simple database of RGB images, taken from the Globe Visualization tool, fully manually annotated and diverse to train the models for accurate Land cover semantic segmentation. Data pre-processing involves image resizing, normalization, and data augmentation. The dataset has different sizes, so image resizing is required. We resize the image to 512 × 512 and zero pad if necessary. Image pixel values are normalized between 0 and 1 by dividing each pixel by 255. A deep learning model with many parameters should be trained on a relatively large proportional dataset for good performance. Data augmentation is a successful approach to improving a model performance. Hence, it increases variation in the image dataset [12]. Various data augmentation techniques are used such as size crop, rotation, horizontal and vertical flip, zoom, brightness, and contrast. Table 1 shows some transformations applied to the image dataset to create more training samples.

#### Annotations and Classes

**Figure 2:**
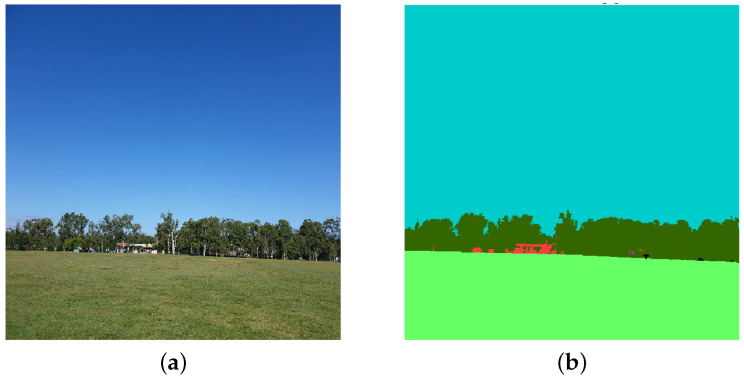
Example of manual labeling image of GLOBE dataset. (**a**) Original Image, (**b**) image labeling.

The GLOBE database does not have ground truth. Hence, we annotated the images and created a ground truth for training and validation. The trained model can now be used without human in the loop for labeling new images. The images are annotated using 12 classes: Grass, Building, Tree, Road, Sky, Soil, Bare Rock, Sand, Sidewalk, Water, Gravel, Blue Mountain as shown in Table 2. Annotations were made manually with Labelme [13] using polygon shape as shown in Figure 2.

### 2.3. CNN for Semantic Segmentation

**Figure 3:**
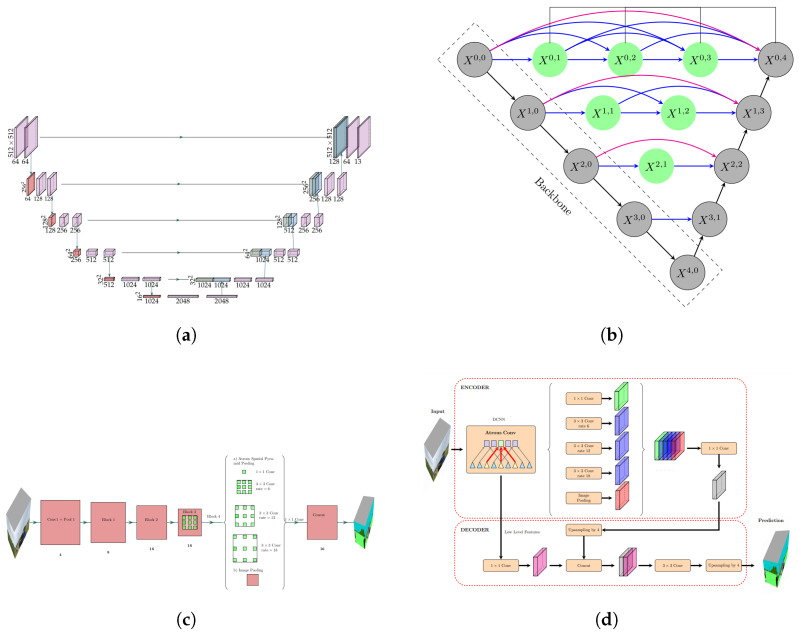
Architectures used for the semantic segmentation stage. (**a**) U-Net architecture (Ronneberger) et al. [18]), (**b**) U-Net++ architecture (Zhou et al. in [19]), (**c**) DeeplabV3. (Liang-Chieh Chen et al. [20]), (**d**) DeeplabV3+ (Liang-Chieh Chen et al. [21]).

We work with CNN-based models due to their high performance in high-resolution images for per-pixel semantic labeling (or image classification) [14]. Specifically, this paper focuses on semantic segmentation using deep learning architectures, as it has more advantages than traditional image processing techniques [15,16]. Semantic segmentation deep learning models can generally be viewed as an encoder network followed by a decoder network. An encoder maps the input into a code, usually a pre-trained classification network such as VGG/Resnet, and a decoder maps the code to reconstruct the input. In semantic segmentation of LULC images, each pixel is given a unique label. One of the first Deep Convolutional Neural Networks (DCNNs) used for semantic segmentation is the Fully Convolutional Network (FCN) [17]. The FCN network model is an extension of the classical CNN but is a network consisting of only convolutional layers. Various more advanced FCN-based approaches have been proposed, such as U-Net, DeepLab, DeeplabV3, U-Net++, SegNet, and DeeplabV3+. This paper focuses on four deep learning semantic segmention architectures: U-Net, DeepLabV3, U-Net++, and DepLabV3+. U-Net is an image segmentation model proposed by Ronneberger et al. [18], which is characterized by symmetrical U-shape architecture consisting of the symmetric contracting path and expansive path, as shown in Figure 3a. U-Net modifies and extends the FCN; the main idea is to make FCN maintain the high-level features in the early layer of the decoder. To this end, they use concatenation for fusing decoder blocks by skipping connections to localize the segmentations. Similarly, Unet++, introduced by Zhou et al. in [19], is an architecture for semantic segmentation based on both nested and dense skip connections. The decoder of Unet++ is more complex than in Unet. In Figure 3b, black blocks refer to the original U-Net, while the green show dense convolution blocks on the skip pathways.

DeepLabV3 is a semantic segmentation model designed and open-sourced by Google that outperforms DeepLabV1 and DeepLabV2. In DeepLab models, input feature maps become smaller while traversing through the convolutional and pooling layers of the network by using Atrous convolutions and Atrous Spatial Pyramid Pooling (ASPP) modules [20,22]. DeepLab-v3+ is an extended model based on the DeepLab-v3 model by adding a decoder module to refine the segmentation results, especially along object boundaries. The depth-wise separable convolution is applied to Atrous spatial pyramid pooling and decoder modules, resulting in a faster and stronger encoder-decoder network for semantic segmentation [21].

### 2.4. Workflow for Supervised Classification

**Figure 4:**
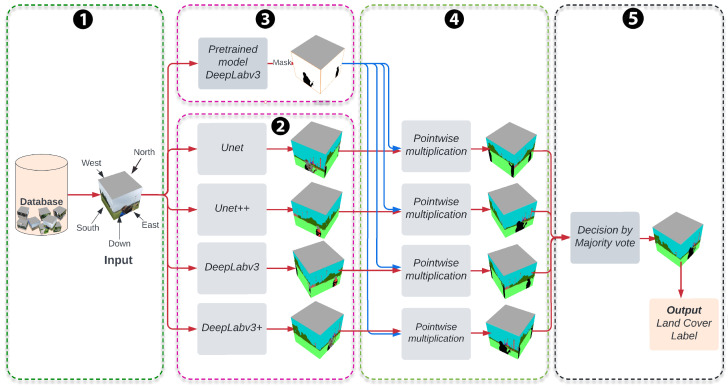
Block diagram for the land cover classification framework. The arrows represent the input and output for each stage, and the stage are represented by the blocks.

Figure 4 illustrates the proposed labeling procedure workflow. The workflow has five stages; ① the first is the preprocessing stage, which corresponds to our annotated datasets, and then data augmentation. ② After this, four deep learning semantic segmentation models were trained, UNet, UNet++, DeepLabV3, and DeepLabV3+; implementation of these models follows [23]. ③ We use DeepLabV3 [20] architecture to remove some man-made objects that appear in GLOBE images. In general, the pre-trained DeepLabV3 with Resnet50 as a backbone model is used to detect man-made objects in the third stage; for example, most of the down direction images have the feet of the photographer, which are removed using DeepLabV3. This pre-trained model has been trained on a subset of Common Objects in the Context dataset (COCO dataset) [24], on the 20 categories present in the Pascal VOC dataset, and was implemented on PyTorch [25]. Those 20 categories on Pascal VOC are the aeroplane, bicycle, bird, boat, bottle, bus, car, cat, chair, cow, diningtable, dog, horse, motorbike, person, pottedplant, sheep, sofa, train, and tvmonitor. ④ In the fourth step, we remove these man-made objects by pointwise multiplication of the mask generated in step three and each segmented image generated by each semantic segmentation model.

⑤ The last step is the final fusion step that combines the segmentation results of the four models. The postprocessing step builds a new classifier by taking a majority vote of the four segmentation models in the previous step. If we denote the segmentations models as $M_{i}$ for i∈{1,2,3,4}. For each pixel *x*, each model $M_{i}$ generates a probability vector $P_{i}\left(x\right)=\left(p_{i,1}\left(x\right),p_{i,2}\left(x\right),\cdots ,p_{i,13}\left(x\right)\right)$, where $p_{i,j}\left(x\right)$ represent the probability of the pixel *x* belonging to class *j*. These models predict class label for *x* as $C_{i}\left(x\right)={\mathrm{argmax}}_{j=1,2,\cdots ,13}\left(P_{i}\left(x\right)\right)$. Next, the final segmentation model predicts a class label for each *x* as $C\left(x\right)=\mathrm{mode}\{C_{1}\left(x\right),C_{2}\left(x\right),C_{3}\left(x\right),$$C_{4}\left(x\right)\}$. If the mode is not unique, we randomly select one of the four models. Finally, the percentage of LC label from the five directional images is divided by the number of pixels of each class and the total number of pixels of the directional images. We do not use the Up image from GLOBE (see Figure 1) because it does not improve the information for land cover classes, in most images there are only sky and/or clouds. We also consider the perspective of the five images taken by weighting the down directional image. If $p_{j}$ represents the percent of land cover class *j* in the directional image and if $I_{1},I_{2},I_{3},I_{4}$ and $I_{5}$ represents the North, South, East, West, and Down images, respectively, then

$$p_{j}=\frac{\sum_{k=1}^{5}\sum_{\begin{array}{c} x\in I_{k},\\ C(I_{k}\left(x\right))=j \end{array}}w_{k}}{\sum_{k=1}^{5}\sum_{x\in I_{k}}w_{k}}$$

### 2.5. Supervised Semantic Segmentation

**Figure 5:**
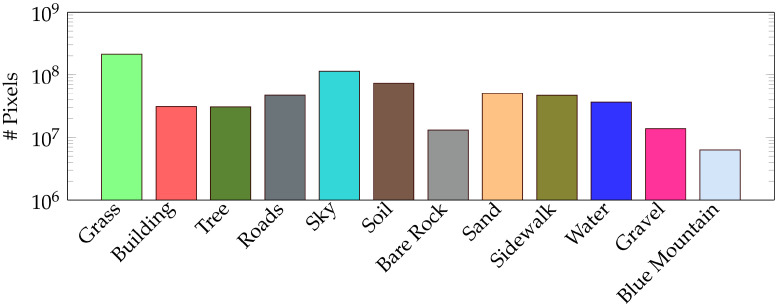
Histogram of the class distribution on the training dataset. The *y*-axis is in logarithmic scale.

For training semantic segmentation models, we use the Adam optimizer with a learning rate of 0.001. The cost function is essential in the training and validation stages. However, there are many of them, and it is impossible to choose one of them as the best [26] for the segmentation task. As shown in Figure 5, our dataset is highly unbalanced. Hence, the Dice Loss function [27] is selected for its high performance.

(1) $$\mathrm{Dice}\;\mathrm{Coeff}=2\frac{y\cap y_{pred}}{y+y_{pred}},\;\mathrm{Dice}\;\mathrm{Loss}=1-\mathrm{Dice}\;\mathrm{Coeff}$$

All our models are trained for 1000 epochs, with a batch size of seven. The validation metrics are the mean intersection over unit and labeling accuracy. Training the four models with 600 images takes four days in Google Colab.

A total of 600 images are used for training the networks, and 100 images for the validation for 13 land cover classes. The output labels are verified for accuracy with testing images from existing correctly labeled GLOBE images. The integrated model is used to assign LC labels to 2915 GLOBE images. Supplementary Table S1 gives the GPS positions and the links to download the five directional images for the database of 2916 LC images. The Supplementary Table S2 gives the predicted labels for the 2916 groups of five image database, totaling the fusion method’s use of 14,580 individual images. Four labels are assigned to each of the 2915 group of five images ordered with higher to lower probability of the LC class found in the image as Label 1, Label 2, Label 3, and Label 4. Land cover label 1 is the most encountered LC class in the five directional images, followed by the other three labels.

In order to standardize the image acquisition and sharing of LULC images around the world, certain requirements can be imposed on the citizen scientists for acquisition and sharing of LULC images such as (a) use of certain models of cameras, (b) imaging distance, (c) imaging time during the day, and (d) weather conditions such as cloud cover during the day, so that the illumination is uniform for all the acquired images.

## 3. Results

**Figure 6:**
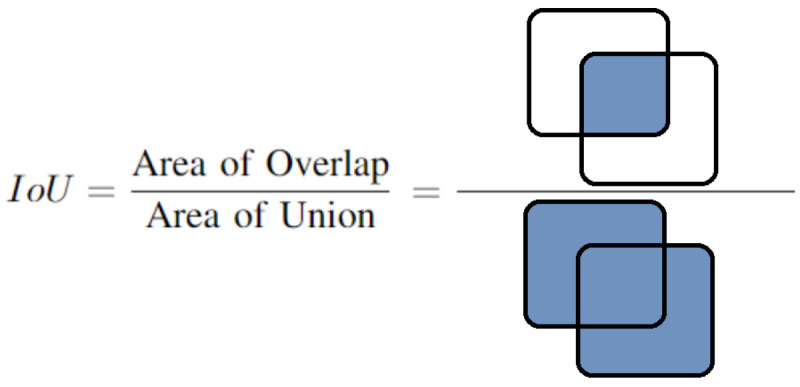
Intersection over union.

This section presents and discusses the results of applying the proposed method for labeling and classifying the GLOBE images. To evaluate and analyze the performance of UNet, UNet++, DeepLabV3, DeepLabV3+, and the fusion method proposed, we use Intersection over Union (IoU), also known as the Jaccard index shown in Figure 6. This metric is defined as the similarity of two regions based on their overlap.

In Table 3, we list and compare the segmentation results obtained on the validation set using the IOU metric for UNet, UNet++, DeepLabV3, DeepLabV3+, and the proposed method. The results demonstrate that our method improves the IOU. The challenging classes such as Building, Sidewalk, and Road also have good accuracies.

The IOU of our fusion model increased accuracy by 1.91% on average compared to the the best of the four individual models. However, if we look at the soil class, the IOU increased by 2.94%. This result demonstrates that our method performs better than the existing models for segmenting directional, rotated and translated land cover imagery.

**Figure 7:**
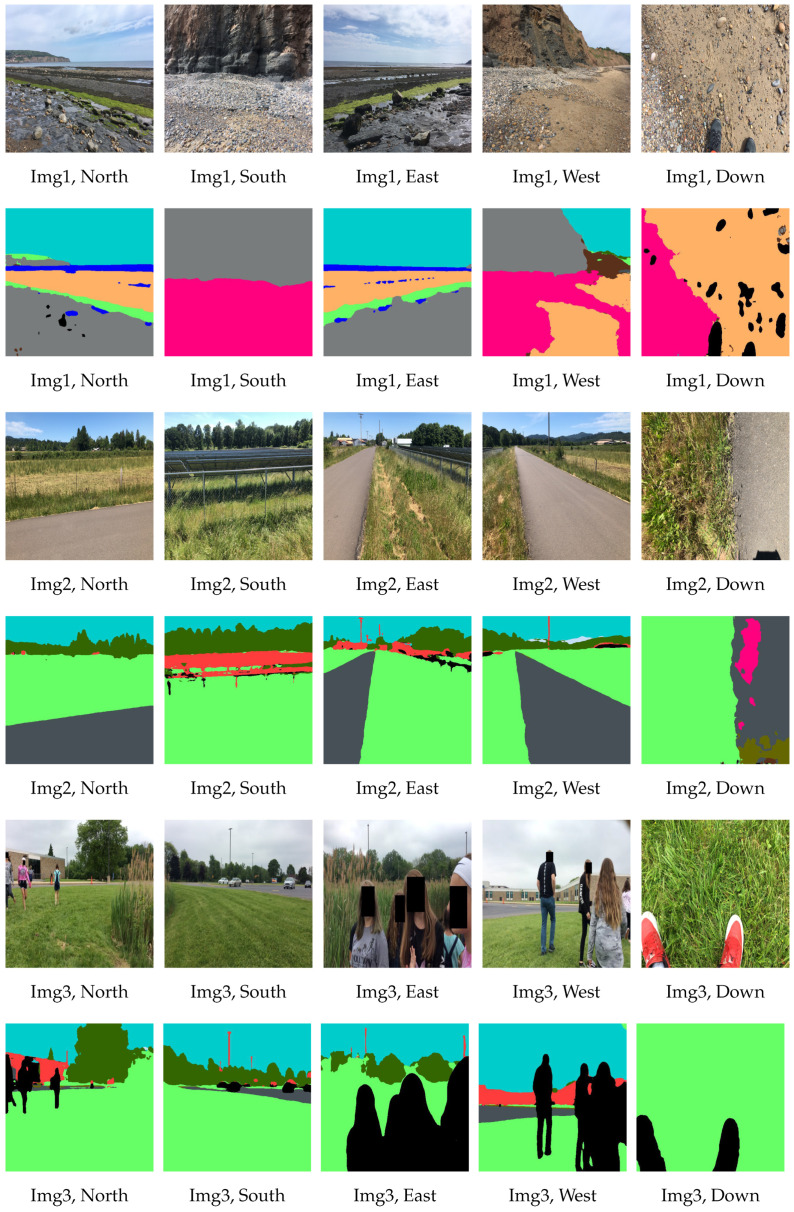
Three sets of five directional GLOBE images and their respective segmented images with the fusion model.

For the image sets in Figure 7, the land cover class labels obtained with our fusion model is given in Table 4.

**Figure 8:**
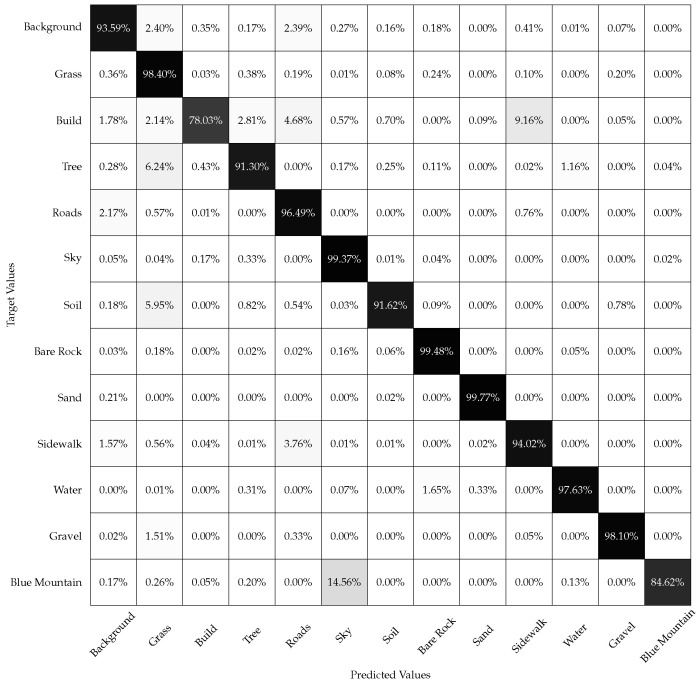
Confusion matrix for semantic segmentation stage in fusion method.

The confusion matrix with land cover labeling accuracies for the 13 classes using the fusion model is given in Figure 8. The classification accuracy is calculated as the number of pixels correctly classified over the total number of pixels for each land cover class multiplied by 100. The model obtained an Overall Accuracy (OA) of 90.97%. The black diagnol entries have accuracies closer to 100%. The off-diagnoal entries have lower accuracies indicating better performance of the fusion model.

## 4. Discussion

The GLOBE images are obtained from handheld cameras; hence, they have a lot of variability in each land cover class. The proposed fusion model has a validation mIOU of 90.97%. The method has difficulty discriminating between the buildings, road, sidewalk classes, and grass and tree classes.

**Figure 9:**
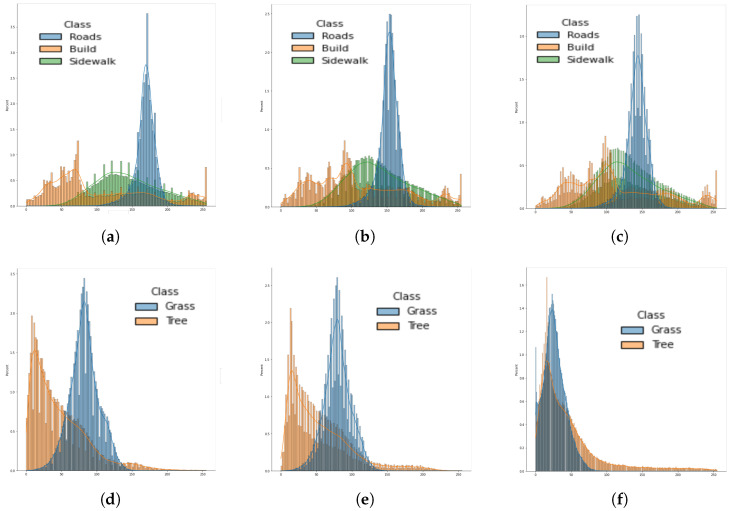
Histogram of ten images containing Road, Build, Sidewalk and ten that includes Grass and Tree. (**a**) Red Band, (**b**) Green Band, (**c**) Blue Band, (**d**) Red Band, (**e**) Green band, (**f**) Blue Band.

Figure 9 shows the difference in the mean intensity values for each class from images taken from different GPS locations. It can be observed that the building class has higher variability, and hence the accuracy in the confusion matrix is lower for this class.

The images are complex, as they are high-resolution RGB images taken from urban and rural areas with a high amount of detail and man-made objects. The images need a lot of preprocessing to remove the noise and variability due to multiple classes and be assigned a land cover label. Our fusion architecture is scalable and can be expanded to a larger application to include more land cover classes. We have developed the fusion architecture in GoogleColab, which runs the four models in parallel using the Graphical Processing Unit (GPU). The current architecture uses four models, but the fusion can be conducted with three models. At least three models are necessary for majority voting. We obtained good results by fusing four models.

Each of the architectures perform well for a particular land cover class, and not for the other. For example, Unet performs well for water and is useful for removing background objects, and DeepLabV3+ performs well for grass and sand classes. Hence, it is necessary to check the accuracies individually for each land cover class.

## 5. Conclusions

A fusion method combining four deep learning architectures is used for labeling high resolution remote sensing imagery from the GLOBE database. DeepLabV3+ is used to remove man-made objects from the images. A weighted averaging is used in the decision block to combine the segmentation results of the four architectures for generating land cover labels with probabilities. The accuracy of labeling achieved is 90.97% for the validation dataset. The GLOBE database does not have ground truth, and our proposed method uses minimal human in the loop based annotation. Our method successfully labeled 2916 image groups. A total of 20% of the images are used for training, and 7% for validation. This work provides a tool for the labeling of land cover images acquired by the public from different parts of the world with different camera settings as well as random image captures from humans with no fixed rotation, translation, or scaling parameters. Despite the variability of the images in the GLOBE database, our labeling tool assigns labels with minimal human in the loop annotation.

## Figures and Tables

**Table 1:** Data augmentation.

Augmentation	Unet	Unet+	DeepLabv3	DeepLabv3+
Crop	(450,512)	(450,512)	(450,512)	(450,512)
Rotation	90${}^{\circ }$, 180${}^{\circ }$ and 270${}^{\circ }$	90${}^{\circ }$, 180${}^{\circ }$ and 270${}^{\circ }$	90${}^{\circ }$, 180${}^{\circ }$ and 270${}^{\circ }$	90${}^{\circ }$, 180${}^{\circ }$ and 270${}^{\circ }$
Brightness and Contrast	0 to 0.05	0 to 0.05	0 to 0.05	0 to 0.05
Flip *x* axis	yes	yes	yes	yes
Flip *y* axis	yes	yes	yes	yes
Median Blur	3	3	3	3

**Table 2:** Semantic class used for train segmentation models stage.

Class ID	Name		Description
1	Grass	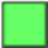	High grass, low grass, grassland and herbaceous.
2	Building	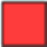	Building, houses and bridges.
3	Tree	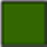	Trees or shrubs, that are part of a forest, include stem and main wooden axis of a tree and the tree crown.
4	Roads	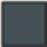	Surfaces that are made of asphalt used for vehicular traffic, including parking lots, but only those made of asphalt.
5	Sky	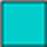	Open sky, cloud, without leaves of trees or any other occlusions
6	Soil	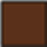	Surfaces that are made of dry or wet soil e.g., soil, dirt roads, muddy or bare soil.
7	Bare Rock	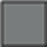	Bare rock places, including rocks.
8	Sand	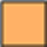	Beach sand or river sand.
9	Sidewalk	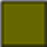	Pedestrian traffic places that are made of concrete.
10	Water	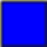	Flowing and stagnant water.
11	Gravel	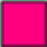	Medium or small gravel used for construction or filling of pedestrian or vehicular paths.
12	Blue Mountain	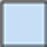	Only mountains look like blue due to the far viewing distance.
0	Background	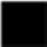	Area not classified to any class. It can include e.g., man-made objects, persons, cars, and all objects excluded from above. In general all unlabeled pixels are defined with the label “undefined”.

**Table 3:** The Intersection over unit metric for segmentation on GLOBE validation dataset.

	UNet	UNet++	DeepLabV3	DeepLabV3+	Fusion
Grass	0.8496	0.9018	0.9083	0.8842	0.9102
Building	0.7344	0.7400	0.6390	0.7315	0.7656
Tree	0.7825	0.8148	0.8712	**0.8906**	0.8787
Roads	0.7668	0.8611	0.8592	0.8277	0.8905
Sky	0.9086	0.9802	0.9623	0.9721	0.9791
Soil	0.7947	0.8280	0.7526	0.8067	0.9092
Bare Rock	0.9576	0.9247	0.8653	0.9735	0.9813
Sand	0.7926	0.8221	0.9968	0.9967	0.9891
Sidewalk	0.7466	0.8903	0.8795	**0.8970**	0.8966
Water	0.9349	0.8605	0.8665	0.9330	0.9413
Gravel	0.7563	0.5810	0.4314	0.9494	0.9350
Blue Mountain	0.6635	0.9285	0.8206	0.8249	0.8394
Backgroud	0.8335	0.8814	0.6960	0.7799	0.8026
mean IOU	0.8073	0.8444	0.8211	0.8906	0.9097

**Table 4:** Land cover and location of the two directional images.

	% Land Cover				Location	
Img1	Bare Rock 33.20%	Gravel 22.40%	Sky 19.06%	Sand 18.99%	Latitude 54.4206	Longitude −0.523
Img2	Grass 49.43%	Roads 24.04%	Sky 11.58%	Tree 9.25%	Latitude 44.5668	Longitude −123.2958
Img3	Grass 45.84%	Sky 22.75%	Background 18.57%	Tree 8.40%	Latitude 42.0859	Longitude −83.2099

## Data Availability

The Biosphere Land Cover Classification image datasets are available at https://vis.globe.gov/GLOBE/ (accessed on 31 March 2022).

## Supplementary Materials

The following supporting information can be downloaded at: https://www.mdpi.com/article/10.3390/s22186895/s1, Table S1: 2915 GLOBE images with GPS positions and links for downloading the five directional images. Table S2: Predicted land cover labels using the fusion method. Labels are ordered from higher to lower probability.
