# Supplementary material for: Land Use Land Cover Labeling of GLOBE Images Using a Deep Learning Fusion Model
Source: Sensors (Basel). 2022 Sep 13;22(18):6895. doi: 10.3390/s22186895 (PMC9503776; doi:10.3390/s22186895)
Supplement: Supplementary file 1 [file sensors-22-06895-s001.zip › Table S2.pdf]

Supplement Table S2. Predicted land cover labels using the fusion method. Labels are ordered from higher to lower probability.

	Background	Grass	Building	Tree	Roads	Sky	Soil	Bare Rock	Sand	Sidewalk	Water	Gravel	Blue Mountain		**Label 1**	**Label 2**	**Label 3**	Label 4
GV_1	8.02	3.32	40.84	35.25	9.18	0.91	0.00	0.00	0.00	2.48	0.00	0.00	0.00		Building	Tree	Roads	Background
GV_2	1.43	0.81	0.01	2.39	0.00	46.75	0.00	0.00	41.93	0.00	6.67	0.00	0.00		Sky	Sand	Water	Tree
GV_3	2.22	36.43	3.73	9.47	44.33	1.30	0.05	2.47	0.00	0.00	0.00	0.00	0.00		Roads	Grass	Tree	Building
GV_4	8.69	2.65	31.03	23.73	15.41	0.71	0.05	0.00	0.01	17.55	0.00	0.17	0.00		Building	Tree	Sidewalk	Roads
GV_5	8.34	17.07	8.31	31.58	26.31	2.35	0.00	0.02	0.00	5.28	0.01	0.64	0.09		Tree	Roads	Grass	Background
GV_6	7.84	7.81	15.09	36.55	26.02	1.39	0.04	0.00	0.00	5.19	0.00	0.07	0.00		Tree	Roads	Building	Background
GV_7	1.02	2.27	0.00	0.07	0.03	19.06	0.92	33.20	18.99	0.00	2.04	22.40	0.00		Bare Rock	Gravel	Sky	Sand
GV_8	0.99	25.67	10.47	35.60	14.07	3.41	0.03	0.01	0.00	9.71	0.00	0.04	0.00		Tree	Grass	Roads	Building
GV_9	5.83	27.59	14.62	21.28	19.41	6.20	0.00	0.35	0.00	4.48	0.23	0.01	0.00		Grass	Tree	Roads	Building
GV_10	2.57	7.05	51.58	4.05	23.18	4.21	0.23	0.00	0.00	7.12	0.00	0.02	0.00		Building	Roads	Sidewalk	Grass
GV_11	7.14	24.44	11.90	32.16	13.57	2.36	0.73	0.30	0.00	5.38	0.00	2.00	0.01		Tree	Grass	Roads	Building
GV_12	9.30	20.50	10.47	5.24	3.01	11.28	5.50	19.27	0.06	0.27	13.09	2.01	0.00		Grass	Bare Rock	Water	Sky
GV_13	2.48	19.15	2.94	45.99	3.26	7.27	0.21	0.10	0.00	17.58	0.00	1.00	0.00		Tree	Grass	Sidewalk	Sky
GV_14	3.30	11.99	5.95	32.53	33.80	4.04	0.19	1.83	0.00	6.30	0.00	0.05	0.00		Roads	Tree	Grass	Sidewalk
GV_15	1.99	17.53	7.97	36.03	19.47	1.23	0.00	0.00	0.00	15.04	0.06	0.58	0.10		Tree	Roads	Grass	Sidewalk
GV_16	20.56	2.51	2.29	58.13	15.52	0.36	0.35	0.00	0.00	0.18	0.00	0.11	0.00		Tree	Background	Roads	Grass
GV_17	19.08	20.82	3.11	6.02	0.01	31.94	1.55	0.00	0.00	17.47	0.00	0.00	0.00		Sky	Grass	Background	Sidewalk
GV_18	3.83	38.45	13.18	13.47	1.58	15.04	3.21	0.06	0.06	11.05	0.04	0.02	0.00		Grass	Sky	Tree	Building
GV_19	0.13	38.78	0.20	4.11	25.80	9.01	0.38	0.00	0.00	0.00	0.00	21.53	0.06		Grass	Roads	Gravel	Sky
GV_20	2.02	36.09	0.11	4.62	30.88	7.17	0.20	0.00	0.00	0.00	0.00	18.86	0.05		Grass	Roads	Gravel	Sky
GV_21	0.76	49.43	3.71	9.25	24.04	11.58	0.03	0.00	0.00	0.52	0.00	0.53	0.15		Grass	Roads	Sky	Tree
GV_22	8.30	9.70	15.41	11.80	5.20	21.44	1.12	15.12	6.74	3.99	1.15	0.01	0.03		Sky	Building	Bare Rock	Tree
GV_23	0.61	55.04	4.39	14.71	16.05	8.83	0.00	0.00	0.00	0.38	0.00	0.00	0.00		Grass	Roads	Tree	Sky
GV_24	1.84	63.53	10.21	12.93	0.45	10.83	0.00	0.00	0.00	0.20	0.00	0.00	0.00		Grass	Tree	Sky	Building
GV_25	1.05	40.54	12.11	16.53	5.62	15.59	0.15	0.00	5.68	0.54	1.22	0.98	0.00		Grass	Tree	Sky	Building
GV_26	2.54	25.21	33.88	8.35	0.05	22.20	0.13	0.14	0.00	7.20	0.00	0.00	0.31		Building	Grass	Sky	Tree
GV_27	1.45	61.33	2.29	26.91	1.80	2.07	0.16	0.21	0.00	1.52	0.02	2.23	0.00		Grass	Tree	Building	Gravel
GV_28	0.01	38.81	0.12	7.03	0.00	54.03	0.00	0.00	0.00	0.00	0.00	0.00	0.00		Sky	Grass	Tree	Building
GV_29	1.19	43.24	9.07	5.44	25.01	15.76	0.00	0.00	0.00	0.08	0.00	0.00	0.20		Grass	Roads	Sky	Building
GV_30	1.49	13.09	13.00	3.84	35.46	12.26	0.05	0.00	0.00	19.13	0.00	1.69	0.00		Roads	Sidewalk	Grass	Building
GV_31	1.10	46.79	1.78	9.93	31.98	8.12	0.00	0.00	0.00	0.11	0.00	0.00	0.18		Grass	Roads	Tree	Sky
GV_32	2.27	41.01	8.40	7.86	34.19	5.60	0.00	0.00	0.00	0.15	0.00	0.25	0.28		Grass	Roads	Building	Tree
GV_33	1.04	47.46	2.91	9.24	27.98	10.76	0.16	0.00	0.00	0.18	0.00	0.10	0.16		Grass	Roads	Sky	Tree
GV_34	2.03	45.36	4.61	12.78	5.94	28.52	0.02	0.00	0.00	0.74	0.00	0.01	0.00		Grass	Sky	Tree	Roads
GV_35	5.54	86.81	3.55	3.24	0.02	0.00	0.25	0.04	0.00	0.54	0.00	0.01	0.00		Grass	Background	Building	Tree
GV_36	0.71	75.95	0.48	16.68	0.00	6.04	0.03	0.02	0.00	0.00	0.00	0.00	0.09		Grass	Tree	Sky	Background
GV_37	1.32	48.84	0.56	18.20	1.48	28.64	0.12	0.05	0.00	0.00	0.00	0.57	0.23		Grass	Sky	Tree	Roads
GV_38	26.60	11.13	0.00	62.17	0.00	0.11	0.00	0.00	0.00	0.00	0.00	0.00	0.00		Tree	Background	Grass	Sky
GV_39	0.14	71.47	0.13	3.33	3.61	19.44	0.28	0.00	0.00	1.57	0.00	0.02	0.00		Grass	Sky	Roads	Tree
GV_40	0.07	83.72	0.00	2.82	0.04	13.19	0.00	0.00	0.00	0.15	0.00	0.00	0.00		Grass	Sky	Tree	Sidewalk
GV_41	0.29	58.43	0.77	17.43	2.23	20.79	0.05	0.00	0.00	0.01	0.00	0.00	0.00		Grass	Sky	Tree	Roads
GV_42	0.45	41.51	24.02	5.84	19.03	6.03	0.06	0.03	0.00	2.95	0.00	0.07	0.00		Grass	Building	Roads	Sky
GV_43	1.63	21.15	9.36	20.74	21.14	16.27	2.34	0.27	0.00	2.14	0.00	4.96	0.00		Grass	Roads	Tree	Sky
GV_44	0.25	50.13	4.59	23.37	1.18	4.14	1.51	0.00	0.00	14.82	0.00	0.02	0.00		Grass	Tree	Sidewalk	Building
GV_45	3.07	40.73	2.57	16.46	8.18	0.71	2.53	0.02	0.00	21.46	0.00	4.27	0.00		Grass	Sidewalk	Tree	Roads
GV_46	0.91	56.58	1.09	18.50	9.48	2.20	3.30	0.02	0.00	3.69	0.00	4.22	0.00		Grass	Tree	Roads	Gravel
GV_47	0.21	36.56	0.00	11.81	1.35	48.74	0.00	0.00	0.00	1.31	0.00	0.02	0.00		Sky	Grass	Tree	Roads
GV_48	4.02	59.80	2.34	22.57	0.45	10.29	0.02	0.00	0.00	0.43	0.00	0.03	0.05		Grass	Tree	Sky	Background
GV_49	4.70	20.61	1.96	20.59	40.21	1.40	0.28	0.08	0.38	5.69	0.08	4.03	0.00		Roads	Grass	Tree	Sidewalk
GV_50	24.63	13.97	9.83	11.35	37.17	0.34	0.00	0.00	0.00	2.36	0.00	0.04	0.30		Roads	Background	Grass	Tree
GV_51	18.57	45.84	2.37	8.40	1.88	22.75	0.00	0.00	0.00	0.17	0.00	0.00	0.01		Grass	Sky	Background	Tree
GV_52	0.09	55.71	0.08	4.66	6.08	24.07	5.37	0.00	0.00	0.00	0.00	3.94	0.00		Grass	Sky	Roads	Soil
GV_53	4.73	61.48	6.33	12.35	2.45	6.56	0.00	0.00	0.00	5.92	0.00	0.13	0.04		Grass	Tree	Sky	Building
GV_54	21.53	3.20	3.67	20.80	11.12	34.43	0.07	0.00	0.00	5.11	0.00	0.00	0.08		Sky	Background	Tree	Roads
GV_55	1.55	55.95	10.88	17.67	0.33	13.34	0.00	0.00	0.00	0.22	0.00	0.00	0.06		Grass	Tree	Sky	Building
GV_56	17.53	4.16	27.12	3.87	43.78	2.03	0.21	0.01	0.00	1.28	0.00	0.00	0.00		Roads	Building	Background	Grass
GV_57	0.01	48.48	0.00	0.47	26.36	0.00	0.39	11.07	0.00	13.21	0.00	0.00	0.00		Grass	Roads	Sidewalk	Bare Rock

GV_58	0.59	47.95	0.01	48.79	0.00	2.65	0.00	0.00	0.00	0.00	0.00	0.00	0.00		Tree	Grass	Sky	Background
GV_59	3.36	96.02	0.00	0.00	0.00	0.00	0.61	0.00	0.00	0.00	0.00	0.00	0.00		Grass	Background	Soil	Water
GV_60	0.80	79.48	0.04	0.01	0.00	16.34	0.79	0.00	0.00	0.00	0.01	0.00	2.52		Grass	Sky	Blue Mountain	Background
GV_61	1.13	75.57	0.00	0.00	0.00	20.55	1.28	0.01	0.00	0.00	0.00	0.00	1.47		Grass	Sky	Blue Mountain	Soil
GV_62	0.10	19.62	4.70	43.66	0.08	15.80	0.01	0.00	0.00	16.03	0.00	0.00	0.00		Tree	Grass	Sidewalk	Sky
GV_63	4.15	30.36	7.35	9.54	0.01	25.01	23.23	0.27	0.00	0.04	0.00	0.02	0.00		Grass	Sky	Soil	Tree
GV_64	1.55	53.24	0.07	0.06	3.40	13.98	25.05	0.00	0.00	0.00	0.01	0.77	1.89		Grass	Soil	Sky	Roads
GV_65	0.84	90.77	0.00	0.01	0.00	5.46	0.61	0.00	0.00	0.00	0.00	0.00	2.31		Grass	Sky	Blue Mountain	Background
GV_66	0.92	64.42	0.00	0.26	5.39	7.92	20.50	0.00	0.00	0.00	0.00	0.58	0.00		Grass	Soil	Sky	Roads
GV_67	1.35	42.50	0.03	7.02	6.79	7.61	0.82	0.00	0.00	0.00	0.00	33.74	0.15		Grass	Gravel	Sky	Tree
GV_68	2.40	49.53	0.06	0.00	8.18	8.13	29.54	0.00	0.00	0.00	0.00	0.00	2.17		Grass	Soil	Roads	Sky
GV_69	1.59	66.74	0.00	0.00	0.42	1.21	28.92	0.01	0.00	0.00	0.49	0.00	0.62		Grass	Soil	Background	Sky
GV_70	3.09	68.59	0.00	0.00	4.83	3.56	19.31	0.00	0.00	0.01	0.00	0.00	0.62		Grass	Soil	Roads	Sky
GV_71	3.13	0.94	5.23	9.62	68.48	5.97	0.14	0.08	0.00	6.42	0.00	0.00	0.00		Roads	Tree	Sidewalk	Sky
GV_72	5.21	29.13	8.80	6.64	38.65	1.07	0.10	0.00	0.00	10.39	0.00	0.00	0.00		Roads	Grass	Sidewalk	Building
GV_73	8.25	15.95	15.74	41.93	9.84	0.98	0.11	0.03	0.00	5.95	0.05	1.16	0.00		Tree	Grass	Building	Roads
GV_74	10.88	14.31	7.91	43.39	9.58	0.99	1.30	0.34	0.00	10.12	0.00	1.18	0.00		Tree	Grass	Background	Sidewalk
GV_75	12.27	11.23	11.95	22.21	34.32	2.96	0.12	0.00	0.00	4.93	0.00	0.00	0.00		Roads	Tree	Background	Building
GV_76	0.65	0.33	0.09	16.25	0.00	29.30	0.00	0.01	33.65	0.00	19.60	0.00	0.12		Sand	Sky	Water	Tree
GV_77	1.03	15.60	9.88	14.01	23.59	11.00	3.71	0.00	0.00	20.44	0.00	0.73	0.00		Roads	Sidewalk	Grass	Tree
GV_78	0.34	11.50	8.19	17.54	33.08	13.50	0.02	0.01	0.00	14.95	0.00	0.88	0.00		Roads	Tree	Sidewalk	Sky
GV_79	3.13	7.74	12.96	29.27	17.83	0.40	0.07	2.51	0.00	26.01	0.00	0.09	0.00		Tree	Sidewalk	Roads	Building
GV_80	0.78	22.01	10.04	31.48	13.94	7.35	1.74	0.40	0.00	10.47	0.00	1.78	0.00		Tree	Grass	Roads	Sidewalk
GV_81	4.68	60.44	7.84	10.40	3.15	1.71	5.18	0.00	0.10	6.45	0.00	0.05	0.00		Grass	Tree	Building	Sidewalk
GV_82	0.93	17.02	0.03	80.61	0.00	0.34	0.91	0.02	0.00	0.00	0.12	0.02	0.00		Tree	Grass	Background	Soil
GV_83	0.69	49.42	3.75	21.02	9.66	8.97	1.38	0.00	0.01	0.72	0.00	4.27	0.11		Grass	Tree	Roads	Sky
GV_84	4.57	47.83	6.70	7.71	1.14	18.78	0.07	0.16	4.42	4.85	3.47	0.00	0.29		Grass	Sky	Tree	Building
GV_85	3.53	36.08	2.51	25.84	13.49	8.87	8.21	0.00	0.00	0.29	0.00	1.14	0.03		Grass	Tree	Roads	Sky
GV_86	2.86	2.34	39.37	8.49	16.18	17.07	0.01	0.69	0.00	12.82	0.00	0.15	0.03		Building	Sky	Roads	Sidewalk
GV_87	2.40	20.60	7.13	22.94	19.49	16.33	0.92	0.79	0.00	8.96	0.02	0.40	0.01		Tree	Grass	Roads	Sky
GV_88	0.02	75.38	0.12	7.11	0.00	17.37	0.00	0.00	0.00	0.00	0.00	0.00	0.00		Grass	Sky	Tree	Building
GV_89	2.56	29.56	2.17	31.49	9.52	21.06	0.00	0.01	0.00	3.63	0.00	0.00	0.00		Tree	Grass	Sky	Roads
GV_90	2.20	1.10	0.90	4.06	0.02	24.43	57.51	0.09	0.00	0.00	0.00	0.00	9.69		Soil	Sky	Blue Mountain	Tree
GV_91	1.88	0.08	0.00	1.42	0.01	44.62	0.00	0.00	45.57	0.00	6.42	0.00	0.00		Sand	Sky	Water	Background
GV_92	1.07	0.37	0.00	1.55	0.00	0.62	0.00	0.00	92.96	0.00	3.42	0.00	0.00		Sand	Water	Tree	Background
GV_93	0.38	8.68	0.00	0.01	45.03	3.67	0.02	0.00	35.68	0.57	5.96	0.00	0.00		Roads	Sand	Grass	Water
GV_94	2.81	49.52	7.94	21.37	0.10	18.12	0.02	0.00	0.00	0.06	0.00	0.00	0.06		Grass	Tree	Sky	Building
GV_95	1.51	36.02	12.07	30.95	8.55	6.54	0.61	0.04	0.00	1.43	0.00	2.29	0.00		Grass	Tree	Building	Roads
GV_96	3.31	32.68	0.82	57.60	0.00	5.10	0.05	0.01	0.00	0.36	0.00	0.05	0.00		Tree	Grass	Sky	Background
GV_97	0.81	35.96	17.97	24.58	0.01	20.49	0.00	0.00	0.00	0.19	0.00	0.00	0.00		Grass	Tree	Sky	Building
GV_98	6.75	30.73	10.85	29.83	8.03	10.36	1.16	0.06	0.00	1.71	0.00	0.52	0.00		Grass	Tree	Building	Sky
GV_99	3.92	45.65	19.27	15.68	6.91	6.00	1.87	0.01	0.00	0.62	0.00	0.07	0.00		Grass	Building	Tree	Roads
GV_100	1.64	56.96	15.73	22.26	0.00	3.22	0.00	0.01	0.00	0.18	0.00	0.00	0.00		Grass	Tree	Building	Sky
GV_101	0.75	26.16	0.00	46.94	0.96	11.48	1.67	1.68	6.39	0.00	1.09	2.65	0.22		Tree	Grass	Sky	Sand
GV_102	0.06	37.09	0.00	54.74	0.12	2.25	2.12	0.04	0.00	0.00	0.00	3.58	0.00		Tree	Grass	Gravel	Sky
GV_103	2.91	10.44	0.25	63.21	0.02	5.26	15.73	0.15	0.00	0.00	0.00	2.02	0.00		Tree	Soil	Grass	Sky
GV_104	0.26	55.38	0.07	20.34	0.32	9.46	7.04	0.08	2.78	0.00	0.01	4.14	0.13		Grass	Tree	Sky	Soil
GV_105	0.39	45.16	0.10	12.13	26.94	10.89	0.32	0.00	0.00	0.99	0.03	2.42	0.62		Grass	Roads	Tree	Sky
GV_106	27.68	2.56	0.09	0.00	19.66	0.35	0.07	0.91	0.08	48.26	0.28	0.06	0.00		Sidewalk	Background	Roads	Grass
GV_107	0.21	6.15	0.01	19.43	0.60	18.60	32.19	4.16	1.01	7.32	10.19	0.12	0.00		Soil	Tree	Sky	Water
GV_108	4.03	36.22	5.98	27.36	0.43	25.37	0.02	0.00	0.00	0.32	0.00	0.28	0.00		Grass	Tree	Sky	Building
GV_109	0.57	62.54	0.01	18.00	3.37	0.06	3.36	0.16	0.00	0.00	0.03	11.90	0.00		Grass	Tree	Gravel	Roads
GV_110	0.57	25.83	0.30	37.56	2.05	14.89	15.51	0.04	0.00	0.31	0.30	2.63	0.02		Tree	Grass	Soil	Sky
GV_111	2.08	28.77	2.19	20.11	19.06	15.87	3.49	0.04	0.00	0.31	0.01	8.05	0.00		Grass	Tree	Roads	Sky
GV_112	0.66	26.70	1.17	16.16	26.61	15.30	0.28	0.01	1.79	7.94	0.02	3.30	0.04		Grass	Roads	Tree	Sky
GV_113	0.73	12.32	0.18	72.15	0.02	2.36	9.70	0.00	0.00	0.00	0.00	2.54	0.00		Tree	Grass	Soil	Gravel
GV_114	0.41	13.20	0.03	60.58	1.06	0.38	17.26	0.25	0.00	0.00	0.00	6.84	0.00		Tree	Soil	Grass	Gravel
GV_115	0.17	50.32	0.00	21.07	13.88	1.20	12.36	0.02	0.00	0.00	0.00	0.98	0.00		Grass	Tree	Roads	Soil
GV_116	0.21	21.97	1.89	10.93	25.52	20.55	9.92	0.31	0.00	0.85	0.22	6.59	1.04		Roads	Grass	Sky	Tree

GV_117	6.00	14.60	7.12	11.53	39.94	16.67	0.28	1.04	0.00	2.62	0.00	0.06	0.14		Roads	Sky	Grass	Tree
GV_118	0.19	73.63	0.00	2.88	0.19	7.49	13.47	0.01	0.00	0.15	0.00	1.96	0.04		Grass	Soil	Sky	Tree
GV_119	2.54	44.14	5.09	12.62	18.75	10.89	3.00	0.04	0.00	0.84	0.14	1.14	0.81		Grass	Roads	Tree	Sky
GV_120	0.63	33.17	1.10	5.42	40.87	17.80	0.06	0.00	0.00	0.46	0.00	0.00	0.50		Roads	Grass	Sky	Tree
GV_121	0.13	41.78	0.71	23.44	12.31	10.15	0.20	0.00	2.42	3.10	0.00	4.65	1.11		Grass	Tree	Roads	Sky
GV_122	0.03	15.99	0.00	83.26	0.00	0.17	0.40	0.02	0.00	0.00	0.00	0.12	0.00		Tree	Grass	Soil	Sky
GV_123	1.47	63.68	0.02	26.29	0.39	0.12	0.92	0.10	0.00	1.11	0.00	5.88	0.00		Grass	Tree	Gravel	Background
GV_124	0.00	36.73	0.02	14.90	26.94	8.35	0.30	0.06	0.00	12.08	0.00	0.60	0.02		Grass	Roads	Tree	Sidewalk
GV_125	1.46	22.84	0.30	10.58	28.54	6.12	11.26	8.61	0.32	0.05	8.35	1.56	0.00		Roads	Grass	Soil	Tree
GV_126	0.10	6.12	0.08	18.93	59.26	13.16	0.03	0.00	0.16	1.82	0.00	0.35	0.00		Roads	Tree	Sky	Grass
GV_127	2.10	10.81	0.05	36.28	34.79	10.84	0.52	0.06	0.00	1.38	0.01	3.16	0.00		Tree	Roads	Sky	Grass
GV_128	0.24	56.74	0.01	12.52	0.17	2.78	12.90	0.00	0.00	0.78	0.01	13.85	0.01		Grass	Gravel	Soil	Tree
GV_129	7.21	3.30	9.24	23.05	47.00	9.74	0.05	0.01	0.00	0.35	0.01	0.02	0.03		Roads	Tree	Sky	Building
GV_130	1.32	21.86	6.08	17.54	42.71	5.31	0.02	0.02	0.02	3.27	0.00	1.84	0.00		Roads	Grass	Tree	Building
GV_131	0.53	7.53	0.00	75.78	1.01	0.44	10.14	0.01	0.00	0.11	0.00	4.45	0.00		Tree	Soil	Grass	Gravel
GV_132	0.65	72.23	3.29	16.84	1.92	4.19	0.65	0.00	0.00	0.22	0.00	0.01	0.00		Grass	Tree	Sky	Building
GV_133	0.29	60.29	1.00	22.58	0.14	14.08	0.28	0.00	0.00	1.34	0.00	0.00	0.00		Grass	Tree	Sky	Sidewalk
GV_134	3.23	38.90	2.00	30.42	5.23	6.77	0.34	0.00	0.01	12.97	0.09	0.03	0.01		Grass	Tree	Sidewalk	Sky
GV_135	11.21	1.85	5.46	1.24	15.12	20.37	40.37	0.49	0.00	0.30	0.00	0.13	3.45		Soil	Sky	Roads	Background
GV_136	0.53	67.17	1.65	5.83	0.00	23.30	0.00	0.02	0.00	0.00	0.01	0.16	1.34		Grass	Sky	Tree	Building
GV_137	0.58	61.22	0.29	11.88	0.83	11.84	11.84	0.09	0.00	0.12	0.00	0.15	1.15		Grass	Tree	Sky	Soil
GV_138	0.60	50.74	0.04	24.25	6.04	1.46	8.98	1.35	0.00	0.20	0.15	6.17	0.00		Grass	Tree	Soil	Gravel
GV_139	0.11	66.33	0.02	22.38	0.00	9.23	0.69	0.02	0.00	0.00	0.00	0.06	1.16		Grass	Tree	Sky	Blue Mountain
GV_140	0.44	63.33	0.65	29.00	0.02	5.22	0.81	0.08	0.00	0.00	0.00	0.46	0.00		Grass	Tree	Sky	Soil
GV_141	2.10	20.31	0.36	28.98	2.73	19.90	25.15	0.00	0.00	0.37	0.00	0.11	0.01		Tree	Soil	Grass	Sky
GV_142	9.21	17.57	13.63	7.04	22.09	11.60	0.24	0.23	0.00	18.32	0.00	0.07	0.00		Roads	Sidewalk	Grass	Building
GV_143	1.73	22.54	0.55	40.03	20.70	3.09	9.64	0.11	0.00	0.36	0.05	1.20	0.00		Tree	Grass	Roads	Soil
GV_144	3.52	27.28	10.31	18.25	24.06	5.75	0.98	0.13	0.00	1.94	0.00	7.74	0.04		Grass	Roads	Tree	Building
GV_145	0.13	32.87	1.64	30.82	14.51	8.38	0.00	0.00	0.00	11.65	0.00	0.00	0.00		Grass	Tree	Roads	Sidewalk
GV_146	1.14	37.58	1.94	32.68	22.55	1.21	0.10	0.02	0.00	2.79	0.00	0.01	0.00		Grass	Tree	Roads	Sidewalk
GV_147	0.32	52.18	0.09	33.10	13.23	0.24	0.21	0.00	0.00	0.00	0.59	0.02	0.00		Grass	Tree	Roads	Water
GV_148	0.32	57.12	0.33	20.59	15.53	2.64	0.10	0.00	0.00	3.37	0.00	0.00	0.00		Grass	Tree	Roads	Sidewalk
GV_149	0.06	27.51	0.01	16.72	41.93	4.61	0.01	0.01	0.00	8.84	0.00	0.02	0.29		Roads	Grass	Tree	Sidewalk
GV_150	15.97	18.84	0.00	64.56	0.00	0.27	0.11	0.00	0.00	0.21	0.00	0.02	0.00		Tree	Grass	Background	Sky
GV_151	0.42	54.57	8.80	12.29	6.20	11.36	0.00	0.00	0.00	6.36	0.00	0.01	0.00		Grass	Tree	Sky	Building
GV_152	2.56	29.05	19.49	9.33	11.73	12.55	0.13	0.01	0.02	15.00	0.00	0.13	0.00		Grass	Building	Sidewalk	Sky
GV_153	1.91	45.12	20.64	7.60	2.78	12.18	0.00	0.00	0.00	9.69	0.00	0.07	0.00		Grass	Building	Sky	Sidewalk
GV_154	0.47	31.58	16.77	14.35	6.75	15.06	0.54	0.02	0.00	12.88	0.00	1.57	0.00		Grass	Building	Sky	Tree
GV_155	1.44	20.40	16.57	22.69	24.53	7.15	0.07	0.01	0.00	6.84	0.01	0.29	0.00		Roads	Tree	Grass	Building
GV_156	1.22	25.45	9.24	31.39	14.92	7.07	4.57	1.53	0.00	2.88	0.00	1.72	0.00		Tree	Grass	Roads	Building
GV_157	7.92	17.70	5.36	10.42	47.60	5.85	0.07	0.00	0.00	5.06	0.00	0.00	0.00		Roads	Grass	Tree	Background
GV_158	2.99	35.68	2.77	1.30	9.89	0.06	2.89	0.00	0.42	43.68	0.00	0.33	0.00		Sidewalk	Grass	Roads	Background
GV_159	5.05	53.37	11.58	22.92	3.24	2.56	0.85	0.00	0.00	0.41	0.00	0.03	0.00		Grass	Tree	Building	Background
GV_160	0.24	38.21	0.36	49.12	3.07	2.33	3.51	0.02	0.00	0.00	0.00	3.13	0.00		Tree	Grass	Soil	Gravel
GV_161	0.01	39.94	0.51	50.36	4.93	0.21	2.10	0.06	0.00	0.00	0.00	1.88	0.00		Tree	Grass	Roads	Soil
GV_162	0.14	99.85	0.00	0.00	0.00	0.00	0.00	0.00	0.00	0.00	0.00	0.00	0.00		Grass	Background	Soil	Sky
GV_163	0.18	99.19	0.02	0.02	0.00	0.13	0.42	0.02	0.00	0.00	0.01	0.00	0.01		Grass	Soil	Background	Sky
GV_164	1.79	96.47	0.02	0.00	0.04	0.00	0.50	0.00	0.00	1.19	0.00	0.00	0.00		Grass	Background	Sidewalk	Soil
GV_165	1.35	63.05	0.26	19.48	1.14	13.24	0.76	0.18	0.00	0.44	0.00	0.03	0.07		Grass	Tree	Sky	Background
GV_166	1.31	64.66	3.57	27.31	0.00	3.15	0.00	0.01	0.00	0.00	0.00	0.00	0.00		Grass	Tree	Building	Sky
GV_167	0.94	21.57	14.37	23.55	0.25	38.79	0.00	0.02	0.00	0.51	0.00	0.00	0.00		Sky	Tree	Grass	Building
GV_168	14.82	15.44	9.78	24.04	5.91	15.58	0.42	0.02	4.89	8.37	0.00	0.73	0.00		Tree	Sky	Grass	Background
GV_169	3.21	34.40	10.54	25.26	2.62	16.70	0.14	0.00	0.00	7.07	0.00	0.05	0.00		Grass	Tree	Sky	Building
GV_170	3.88	72.50	9.40	7.11	4.45	1.74	0.24	0.00	0.00	0.64	0.00	0.03	0.00		Grass	Building	Tree	Roads
GV_171	3.51	35.88	20.69	5.58	6.31	18.35	0.00	0.00	0.00	9.67	0.00	0.00	0.00		Grass	Building	Sky	Sidewalk
GV_172	0.63	45.20	4.24	25.83	6.75	11.28	3.29	0.02	0.01	1.92	0.02	0.82	0.00		Grass	Tree	Sky	Roads
GV_173	10.66	78.46	4.26	1.73	0.80	0.86	0.00	0.00	0.00	3.22	0.00	0.01	0.00		Grass	Background	Building	Sidewalk
GV_174	13.20	15.67	11.55	17.76	11.93	18.57	0.13	0.09	0.05	10.34	0.15	0.55	0.01		Sky	Tree	Grass	Background
GV_175	0.30	35.47	0.05	11.65	12.64	34.59	0.25	0.00	0.00	0.52	0.01	4.52	0.00		Grass	Sky	Roads	Tree

GV_176	1.20	46.92	0.66	19.35	0.66	27.42	3.39	0.00	0.03	0.29	0.00	0.09	0.00		Grass	Sky	Tree	Soil
GV_177	0.49	62.58	0.95	8.38	0.00	2.40	25.20	0.00	0.00	0.00	0.00	0.00	0.00		Grass	Soil	Tree	Sky
GV_178	1.18	34.01	8.41	46.33	1.37	5.85	1.02	0.01	0.00	1.75	0.00	0.06	0.00		Tree	Grass	Building	Sky
GV_179	1.31	37.53	7.16	44.42	0.46	4.59	1.13	0.00	0.00	3.37	0.00	0.03	0.00		Tree	Grass	Building	Sky
GV_180	1.96	22.89	3.45	53.87	0.69	14.42	2.43	0.04	0.00	0.26	0.00	0.00	0.00		Tree	Grass	Sky	Building
GV_181	2.75	51.57	11.02	11.65	0.00	22.88	0.00	0.01	0.00	0.12	0.00	0.00	0.00		Grass	Sky	Tree	Building
GV_182	3.39	58.51	1.49	23.99	5.23	6.70	0.07	0.00	0.00	0.29	0.00	0.31	0.00		Grass	Tree	Sky	Roads
GV_183	3.65	9.49	7.50	10.98	41.62	11.80	0.01	0.05	1.94	12.96	0.00	0.00	0.00		Roads	Sidewalk	Sky	Tree
GV_184	0.32	68.26	0.70	10.40	1.34	9.34	0.20	0.02	8.33	0.02	0.73	0.34	0.00		Grass	Tree	Sky	Sand
GV_185	0.86	56.39	18.60	20.30	0.02	3.64	0.02	0.00	0.00	0.15	0.00	0.00	0.00		Grass	Tree	Building	Sky
GV_186	1.08	61.24	0.57	24.44	0.00	12.64	0.01	0.00	0.00	0.01	0.00	0.00	0.00		Grass	Tree	Sky	Background
GV_187	4.04	8.38	0.45	12.61	20.46	20.86	12.84	19.70	0.01	0.18	0.09	0.30	0.07		Sky	Roads	Bare Rock	Soil
GV_188	2.79	4.80	3.07	15.28	16.46	7.73	2.45	45.62	0.46	0.71	0.59	0.03	0.00		Bare Rock	Roads	Tree	Sky
GV_189	1.40	64.60	1.04	4.31	0.32	8.17	12.28	0.26	2.57	4.57	0.00	0.35	0.13		Grass	Soil	Sky	Sidewalk
GV_190	12.76	16.16	14.68	18.69	8.30	16.58	0.32	0.68	4.49	7.11	0.02	0.22	0.00		Tree	Sky	Grass	Building
GV_191	0.08	93.96	0.03	0.00	5.60	0.13	0.17	0.00	0.00	0.00	0.00	0.02	0.00		Grass	Roads	Soil	Sky
GV_192	2.53	11.12	14.29	25.50	0.67	43.77	0.01	0.03	0.00	1.94	0.00	0.13	0.01		Sky	Tree	Building	Grass
GV_193	3.50	17.08	5.71	52.53	0.06	20.67	0.00	0.01	0.00	0.42	0.00	0.03	0.00		Tree	Sky	Grass	Building
GV_194	0.03	51.15	0.25	43.56	1.50	3.38	0.01	0.08	0.00	0.00	0.00	0.04	0.00		Grass	Tree	Sky	Roads
GV_195	0.00	56.36	0.02	36.51	0.00	3.41	1.93	0.00	0.00	0.00	0.00	1.77	0.00		Grass	Tree	Sky	Soil
GV_196	22.82	8.57	9.23	37.54	2.26	16.63	0.53	0.01	0.04	1.81	0.18	0.13	0.24		Tree	Background	Sky	Building
GV_197	14.46	14.38	0.11	33.14	13.14	13.94	0.87	0.00	6.54	1.35	0.22	1.65	0.18		Tree	Background	Grass	Sky
GV_198	1.34	36.60	14.03	22.99	0.00	25.02	0.01	0.01	0.00	0.00	0.00	0.00	0.00		Grass	Sky	Tree	Building
GV_199	5.66	33.42	19.54	26.04	3.52	10.79	0.03	0.00	0.00	0.98	0.00	0.00	0.00		Grass	Tree	Building	Sky
GV_200	2.61	34.94	18.68	19.67	4.56	19.07	0.01	0.00	0.00	0.46	0.00	0.00	0.01		Grass	Tree	Sky	Building
GV_201	4.43	18.87	19.48	30.16	0.21	26.24	0.06	0.01	0.00	0.52	0.00	0.00	0.02		Tree	Sky	Building	Grass
GV_202	2.11	25.32	21.43	21.05	4.82	24.79	0.00	0.01	0.00	0.47	0.00	0.00	0.01		Grass	Sky	Building	Tree
GV_203	0.84	11.98	7.82	27.93	0.02	50.72	0.02	0.39	0.00	0.18	0.00	0.00	0.09		Sky	Tree	Grass	Building
GV_204	2.66	49.72	11.40	25.78	1.05	9.09	0.00	0.00	0.00	0.30	0.00	0.00	0.00		Grass	Tree	Building	Sky
GV_205	2.85	11.62	8.62	35.31	0.57	40.75	0.00	0.09	0.00	0.12	0.00	0.00	0.08		Sky	Tree	Grass	Building
GV_206	2.10	45.83	2.63	34.99	1.83	7.01	0.33	0.06	0.00	0.16	0.00	5.05	0.00		Grass	Tree	Sky	Gravel
GV_207	1.06	37.82	5.56	18.73	0.01	34.38	0.18	0.00	0.00	0.04	0.00	1.94	0.30		Grass	Sky	Tree	Building
GV_208	0.28	64.54	0.01	4.71	5.41	23.32	0.32	0.04	0.05	1.09	0.22	0.00	0.00		Grass	Sky	Roads	Tree
GV_209	2.10	58.87	19.96	14.59	1.65	2.15	0.00	0.01	0.00	0.68	0.00	0.00	0.00		Grass	Building	Tree	Sky
GV_210	2.08	44.50	27.81	16.43	3.65	5.48	0.03	0.00	0.00	0.02	0.00	0.00	0.00		Grass	Building	Tree	Sky
GV_211	0.43	77.01	12.75	8.49	0.28	0.98	0.00	0.00	0.00	0.06	0.00	0.00	0.00		Grass	Building	Tree	Sky
GV_212	1.26	68.15	16.73	6.81	1.90	4.89	0.00	0.00	0.00	0.23	0.00	0.00	0.03		Grass	Building	Tree	Sky
GV_213	10.31	15.15	0.56	18.88	5.84	7.44	11.01	5.77	7.43	1.44	10.03	1.94	4.20		Tree	Grass	Soil	Background
GV_214	1.90	38.14	32.99	3.22	3.09	13.82	0.03	0.00	0.00	6.78	0.00	0.04	0.00		Grass	Building	Sky	Sidewalk
GV_215	6.39	31.69	0.06	23.06	1.02	0.01	36.33	0.02	0.00	0.31	0.00	1.12	0.00		Soil	Grass	Tree	Background
GV_216	8.68	33.63	0.08	34.25	1.39	0.53	11.84	0.00	0.00	2.92	0.00	6.69	0.00		Tree	Grass	Soil	Background
GV_217	7.10	32.83	0.74	0.87	0.18	13.89	21.94	22.08	0.00	0.00	0.08	0.29	0.00		Grass	Bare Rock	Soil	Sky
GV_218	1.81	28.93	13.14	6.19	14.41	8.00	15.06	1.19	0.02	10.80	0.00	0.45	0.00		Grass	Soil	Roads	Building
GV_219	0.00	66.54	0.05	6.85	0.00	26.55	0.00	0.00	0.00	0.00	0.00	0.00	0.00		Grass	Sky	Tree	Building
GV_220	12.87	8.57	16.27	25.93	17.33	18.31	0.02	0.00	0.00	0.63	0.00	0.00	0.06		Tree	Sky	Roads	Building
GV_221	2.24	52.37	7.12	29.95	1.16	4.08	0.70	0.07	0.00	0.40	0.00	1.91	0.00		Grass	Tree	Building	Sky
GV_222	0.08	14.03	0.06	20.69	13.28	30.92	6.00	1.74	0.00	0.00	0.00	13.11	0.09		Sky	Tree	Grass	Roads
GV_223	1.90	48.22	9.08	28.22	0.02	12.31	0.00	0.01	0.00	0.24	0.00	0.00	0.00		Grass	Tree	Sky	Building
GV_224	4.49	38.59	10.51	23.15	7.79	4.09	1.57	0.00	0.04	6.20	0.00	3.57	0.00		Grass	Tree	Building	Roads
GV_225	0.03	70.86	0.01	21.48	0.02	3.23	1.41	0.00	0.00	0.15	2.55	0.27	0.00		Grass	Tree	Sky	Water
GV_226	0.90	53.62	1.78	14.39	1.68	11.70	9.50	5.34	0.00	0.00	0.04	1.05	0.00		Grass	Tree	Sky	Soil
GV_227	0.84	64.03	2.64	16.12	3.44	12.73	0.14	0.00	0.00	0.00	0.01	0.04	0.00		Grass	Tree	Sky	Roads
GV_228	1.96	39.70	8.62	19.58	6.25	3.83	10.56	6.69	0.00	0.83	0.01	1.94	0.02		Grass	Tree	Soil	Building
GV_229	0.72	30.29	17.78	3.92	20.74	20.04	0.00	0.01	0.00	6.47	0.00	0.02	0.00		Grass	Roads	Sky	Building
GV_230	3.34	45.63	3.78	24.12	5.46	15.66	0.63	0.00	0.01	0.03	0.00	1.22	0.11		Grass	Tree	Sky	Roads
GV_231	0.79	47.15	3.98	9.36	10.24	19.37	5.64	0.00	0.60	0.09	1.61	0.99	0.17		Grass	Sky	Roads	Tree
GV_232	1.61	55.11	1.43	32.88	1.93	4.81	0.84	0.00	0.45	0.21	0.00	0.73	0.00		Grass	Tree	Sky	Roads
GV_233	1.19	17.99	18.13	11.39	22.39	17.78	0.03	0.04	0.00	11.03	0.00	0.01	0.01		Roads	Building	Grass	Sky
GV_234	0.72	42.54	22.59	10.48	3.26	18.15	0.00	0.00	0.00	2.20	0.00	0.06	0.00		Grass	Building	Sky	Tree

GV_235	0.50	39.18	9.26	9.42	7.13	11.27	4.23	13.02	0.00	0.74	0.46	4.79	0.00		Grass	Bare Rock	Sky	Tree
GV_236	3.90	49.69	9.75	15.77	6.35	8.77	1.22	0.53	0.16	3.82	0.00	0.05	0.00		Grass	Tree	Building	Sky
GV_237	0.49	26.18	15.92	9.00	20.43	26.29	0.00	0.00	0.00	0.76	0.83	0.00	0.10		Sky	Grass	Roads	Building
GV_238	0.18	68.79	2.41	7.49	0.53	20.52	0.00	0.00	0.00	0.06	0.00	0.03	0.00		Grass	Sky	Tree	Building
GV_239	0.11	41.65	2.12	32.34	14.14	3.06	0.10	0.00	0.00	0.01	0.00	6.47	0.00		Grass	Tree	Roads	Gravel
GV_240	1.09	7.40	38.13	25.60	0.42	11.17	1.07	7.15	0.00	7.96	0.00	0.01	0.00		Building	Tree	Sky	Sidewalk
GV_241	5.77	46.57	8.66	24.16	4.00	4.08	4.47	0.01	0.00	1.87	0.01	0.39	0.00		Grass	Tree	Building	Background
GV_242	6.03	9.11	31.23	29.45	6.30	5.54	0.82	4.31	0.01	6.35	0.00	0.84	0.00		Building	Tree	Grass	Sidewalk
GV_243	1.10	55.96	4.51	10.85	3.63	21.06	2.45	0.01	0.00	0.00	0.00	0.05	0.39		Grass	Sky	Tree	Building
GV_244	2.72	32.56	0.02	32.85	11.03	19.65	0.01	0.01	0.00	0.31	0.00	0.83	0.00		Tree	Grass	Sky	Roads
GV_245	3.29	2.28	11.73	9.04	47.83	11.24	0.44	0.14	2.15	10.99	0.61	0.09	0.17		Roads	Building	Sky	Sidewalk
GV_246	0.19	42.47	0.19	26.70	2.04	14.48	3.89	6.41	3.60	0.03	0.00	0.00	0.00		Grass	Tree	Sky	Bare Rock
GV_247	3.27	45.94	5.31	13.67	19.18	7.89	0.06	0.00	0.00	4.61	0.00	0.04	0.03		Grass	Roads	Tree	Sky
GV_248	0.01	94.10	0.04	0.00	0.00	5.83	0.00	0.00	0.00	0.00	0.01	0.00	0.01		Grass	Sky	Building	Background
GV_249	4.71	37.47	1.71	28.89	9.21	5.40	1.01	0.05	0.00	11.52	0.00	0.02	0.00		Grass	Tree	Sidewalk	Roads
GV_250	4.28	26.52	7.62	1.91	20.10	0.10	28.80	2.51	2.19	5.09	0.77	0.09	0.00		Soil	Grass	Roads	Building
GV_251	0.70	31.47	0.02	17.64	2.75	46.68	0.00	0.01	0.00	0.20	0.00	0.47	0.07		Sky	Grass	Tree	Roads
GV_252	1.72	42.05	0.76	7.19	13.10	3.29	0.51	7.62	1.37	22.28	0.07	0.04	0.00		Grass	Sidewalk	Roads	Bare Rock
GV_253	3.59	16.99	0.07	53.37	17.90	4.21	0.29	0.01	0.00	0.15	0.00	3.42	0.00		Tree	Roads	Grass	Sky
GV_254	0.96	47.72	0.02	0.42	8.10	15.85	13.68	0.14	0.00	0.01	0.01	11.92	1.18		Grass	Sky	Soil	Gravel
GV_255	4.56	65.50	7.53	7.95	10.93	1.15	1.76	0.02	0.00	0.31	0.00	0.28	0.00		Grass	Roads	Tree	Building
GV_256	4.56	65.50	7.53	7.95	10.93	1.15	1.76	0.02	0.00	0.31	0.00	0.28	0.00		Grass	Roads	Tree	Building
GV_257	4.56	65.50	7.53	7.95	10.93	1.15	1.76	0.02	0.00	0.31	0.00	0.28	0.00		Grass	Roads	Tree	Building
GV_258	0.50	53.87	1.36	17.54	0.00	20.98	0.00	0.00	0.00	5.75	0.00	0.00	0.00		Grass	Sky	Tree	Sidewalk
GV_259	1.48	92.32	0.26	5.10	0.21	0.03	0.59	0.02	0.00	0.00	0.00	0.00	0.00		Grass	Tree	Background	Soil
GV_260	1.29	75.57	0.05	13.27	0.33	0.16	9.18	0.02	0.00	0.12	0.00	0.01	0.00		Grass	Tree	Soil	Background
GV_261	6.45	25.55	29.12	25.54	4.44	5.02	0.05	0.34	0.05	1.69	0.00	1.75	0.00		Building	Grass	Tree	Background
GV_262	18.87	13.37	27.05	24.12	5.61	2.82	0.04	0.04	0.00	8.09	0.00	0.00	0.00		Building	Tree	Background	Grass
GV_263	1.54	30.21	8.05	17.68	0.00	42.48	0.02	0.00	0.00	0.03	0.00	0.00	0.00		Sky	Grass	Tree	Building
GV_264	1.29	47.50	8.41	20.90	0.01	21.47	0.00	0.01	0.00	0.42	0.00	0.00	0.00		Grass	Sky	Tree	Building
GV_265	9.62	4.05	29.38	13.60	23.58	1.88	0.25	0.00	0.00	17.23	0.00	0.40	0.00		Building	Roads	Sidewalk	Tree
GV_266	15.99	0.84	22.60	6.39	46.51	0.63	0.07	0.12	0.00	4.46	0.00	2.40	0.00		Roads	Building	Background	Tree
GV_267	15.32	7.73	32.59	6.19	15.49	7.30	0.10	0.80	8.34	6.05	0.07	0.00	0.00		Building	Roads	Background	Sand
GV_268	1.64	96.20	0.87	0.56	0.73	0.01	0.00	0.00	0.00	0.00	0.00	0.00	0.00		Grass	Background	Building	Roads
GV_269	1.85	29.93	5.10	29.16	15.77	6.11	0.66	0.02	0.01	0.39	10.00	0.98	0.00		Grass	Tree	Roads	Water
GV_270	0.19	0.48	52.23	8.72	0.00	14.28	0.00	0.02	0.00	0.01	0.00	24.06	0.01		Building	Gravel	Sky	Tree
GV_271	4.21	27.64	13.46	30.00	4.52	14.21	0.09	2.22	0.00	0.38	0.02	0.03	3.21		Tree	Grass	Sky	Building
GV_272	14.92	15.42	16.57	30.00	12.42	1.67	0.64	0.06	0.00	7.38	0.01	0.90	0.00		Tree	Building	Grass	Background
GV_273	0.72	82.51	0.00	0.03	13.46	0.00	1.95	0.01	0.06	0.02	0.02	1.19	0.00		Grass	Roads	Soil	Gravel
GV_274	0.28	86.92	0.34	1.18	2.07	0.00	7.28	0.01	0.00	0.00	0.00	1.92	0.00		Grass	Soil	Roads	Gravel
GV_275	4.26	78.70	5.16	1.95	5.39	0.07	1.97	1.41	0.00	1.03	0.00	0.07	0.00		Grass	Roads	Building	Background
GV_276	0.60	55.52	0.00	19.49	11.34	0.00	7.33	0.10	0.00	0.16	0.04	5.42	0.00		Grass	Tree	Roads	Soil
GV_277	0.85	52.79	0.02	22.69	19.89	1.03	2.67	0.00	0.00	0.00	0.00	0.06	0.00		Grass	Tree	Roads	Soil
GV_278	0.60	56.92	0.00	25.01	0.00	0.20	16.99	0.12	0.12	0.00	0.03	0.00	0.00		Grass	Tree	Soil	Background
GV_279	0.47	90.27	0.00	6.51	0.20	0.00	2.52	0.02	0.00	0.00	0.00	0.01	0.00		Grass	Tree	Soil	Background
GV_280	0.24	73.61	0.03	7.39	7.12	8.75	2.07	0.01	0.30	0.17	0.05	0.26	0.00		Grass	Sky	Tree	Roads
GV_281	0.11	71.36	1.47	7.85	10.35	8.17	0.13	0.06	0.00	0.29	0.06	0.15	0.00		Grass	Roads	Sky	Tree
GV_282	0.36	65.79	1.55	6.22	13.04	9.25	0.44	0.01	0.00	0.24	3.05	0.05	0.00		Grass	Roads	Sky	Tree
GV_283	0.09	41.23	0.01	11.04	22.34	24.08	0.02	0.00	1.01	0.03	0.06	0.08	0.02		Grass	Sky	Roads	Tree
GV_284	1.67	37.36	0.92	17.10	28.38	11.66	0.28	0.01	1.14	0.07	0.29	1.12	0.00		Grass	Roads	Tree	Sky
GV_285	0.42	67.49	0.13	3.57	6.63	2.42	11.30	0.03	0.95	4.28	2.42	0.35	0.01		Grass	Soil	Roads	Sidewalk
GV_286	0.02	60.97	0.00	1.78	15.28	10.04	0.89	0.00	5.84	2.22	2.88	0.09	0.00		Grass	Roads	Sky	Sand
GV_287	0.90	40.17	0.13	3.51	0.03	17.92	26.00	4.17	0.03	0.00	0.67	6.19	0.27		Grass	Soil	Sky	Gravel
GV_288	0.65	71.13	0.00	4.50	7.59	11.45	3.99	0.00	0.03	0.28	0.06	0.31	0.00		Grass	Sky	Roads	Tree
GV_289	0.03	60.70	0.01	4.79	6.49	8.18	11.27	0.00	0.00	8.50	0.00	0.05	0.00		Grass	Soil	Sidewalk	Sky
GV_290	0.01	84.70	0.00	0.00	0.14	0.00	15.01	0.00	0.00	0.00	0.01	0.14	0.00		Grass	Soil	Gravel	Roads
GV_291	0.01	60.19	0.07	1.09	33.16	3.90	0.00	0.00	0.00	0.94	0.60	0.05	0.00		Grass	Roads	Sky	Tree
GV_292	0.74	46.44	0.00	0.10	3.44	0.24	44.90	0.88	0.12	2.55	0.57	0.02	0.00		Grass	Soil	Roads	Sidewalk
GV_293	0.55	33.63	0.06	2.32	24.19	1.92	2.90	0.08	0.70	19.75	13.79	0.11	0.00		Grass	Roads	Sidewalk	Water

GV_294	0.06	40.80	0.03	2.74	35.76	10.60	0.78	0.03	0.11	6.68	1.58	0.82	0.00		Grass	Roads	Sky	Sidewalk
GV_295	0.08	95.45	0.14	0.00	2.09	0.10	0.02	0.00	0.00	2.03	0.09	0.00	0.00		Grass	Roads	Sidewalk	Building
GV_296	0.12	66.94	0.24	2.60	0.09	0.90	28.37	0.00	0.00	0.70	0.01	0.00	0.01		Grass	Soil	Tree	Sky
GV_297	0.07	81.09	0.00	0.08	1.01	1.28	15.94	0.04	0.00	0.16	0.08	0.25	0.00		Grass	Soil	Sky	Roads
GV_298	0.07	68.63	0.00	0.95	0.26	1.94	28.02	0.01	0.01	0.01	0.10	0.00	0.00		Grass	Soil	Sky	Tree
GV_299	0.67	45.49	1.28	0.02	32.08	4.20	0.53	0.02	0.01	14.70	1.00	0.00	0.00		Grass	Roads	Sidewalk	Sky
GV_300	0.27	30.99	0.00	5.97	18.81	3.23	17.83	0.22	3.85	3.52	12.39	2.92	0.00		Grass	Roads	Soil	Water
GV_301	0.00	39.41	0.00	0.00	47.71	0.27	6.18	0.57	0.47	0.63	4.18	0.58	0.00		Roads	Grass	Soil	Water
GV_302	0.95	45.94	2.13	1.00	26.84	5.92	0.05	0.00	0.00	17.16	0.00	0.00	0.00		Grass	Roads	Sidewalk	Sky
GV_303	0.09	68.41	0.00	0.75	13.59	0.44	12.14	0.00	0.00	4.38	0.00	0.20	0.00		Grass	Roads	Soil	Sidewalk
GV_304	0.34	65.54	3.59	0.03	15.06	11.27	0.44	0.10	0.00	3.55	0.01	0.07	0.00		Grass	Roads	Sky	Building
GV_305	2.65	57.30	4.08	0.88	19.38	8.57	2.34	0.00	0.00	3.28	1.52	0.00	0.00		Grass	Roads	Sky	Building
GV_306	1.68	72.73	0.00	1.22	18.79	4.63	0.20	0.11	0.00	0.64	0.00	0.00	0.00		Grass	Roads	Sky	Background
GV_307	0.06	60.11	0.47	1.40	12.82	5.76	0.41	0.00	0.00	18.91	0.05	0.01	0.00		Grass	Sidewalk	Roads	Sky
GV_308	1.10	50.66	12.94	1.34	24.03	6.64	0.22	0.03	0.76	1.93	0.03	0.32	0.00		Grass	Roads	Building	Sky
GV_309	0.07	28.48	0.04	1.65	50.85	0.84	0.04	0.03	1.03	14.27	2.18	0.51	0.00		Roads	Grass	Sidewalk	Water
GV_310	0.68	44.57	5.25	5.14	24.76	1.60	6.81	0.02	0.08	9.94	1.11	0.04	0.00		Grass	Roads	Sidewalk	Soil
GV_311	0.69	67.97	12.49	0.03	4.51	0.00	12.10	0.00	0.00	2.20	0.01	0.00	0.00		Grass	Building	Soil	Roads
GV_312	1.67	93.08	0.28	0.08	4.44	0.13	0.30	0.02	0.00	0.00	0.00	0.00	0.00		Grass	Roads	Background	Soil
GV_313	0.83	68.41	0.05	1.11	13.43	0.32	6.01	0.13	0.61	7.15	1.95	0.01	0.00		Grass	Roads	Sidewalk	Soil
GV_314	2.04	38.74	6.28	1.63	11.89	0.04	0.61	0.03	16.90	14.46	7.34	0.03	0.00		Grass	Sand	Sidewalk	Roads
GV_315	0.04	54.86	0.04	0.32	23.34	2.24	1.61	0.00	9.31	5.78	2.35	0.12	0.00		Grass	Roads	Sand	Sidewalk
GV_316	0.00	74.63	0.01	0.45	21.80	1.68	0.92	0.00	0.00	0.33	0.18	0.00	0.00		Grass	Roads	Sky	Soil
GV_317	0.04	39.88	0.04	0.43	24.17	0.57	8.78	0.00	11.13	13.60	1.34	0.02	0.00		Grass	Roads	Sidewalk	Sand
GV_318	0.07	40.09	0.21	0.84	20.24	0.36	10.51	0.01	6.67	18.62	2.38	0.00	0.00		Grass	Roads	Sidewalk	Soil
GV_319	0.02	93.91	0.01	1.99	0.86	1.50	1.27	0.00	0.00	0.43	0.00	0.00	0.00		Grass	Tree	Sky	Soil
GV_320	0.21	95.84	0.00	1.40	0.15	2.39	0.01	0.00	0.00	0.00	0.00	0.00	0.00		Grass	Sky	Tree	Background
GV_321	0.04	91.76	0.00	2.18	1.54	2.53	1.89	0.06	0.00	0.00	0.00	0.00	0.00		Grass	Sky	Tree	Soil
GV_322	0.07	94.67	0.21	2.02	0.06	2.97	0.00	0.00	0.00	0.00	0.00	0.00	0.00		Grass	Sky	Tree	Building
GV_323	0.27	94.14	0.00	0.39	3.37	1.38	0.26	0.17	0.00	0.02	0.00	0.00	0.00		Grass	Roads	Sky	Tree
GV_324	0.85	36.49	9.49	0.98	17.72	0.01	0.11	0.00	8.78	23.98	1.58	0.00	0.00		Grass	Sidewalk	Roads	Building
GV_325	0.37	62.54	1.44	1.65	20.10	0.25	0.28	0.00	0.00	13.34	0.00	0.04	0.00		Grass	Roads	Sidewalk	Tree
GV_326	1.05	38.03	11.21	1.88	17.46	0.01	0.89	0.00	8.45	19.82	1.21	0.00	0.00		Grass	Sidewalk	Roads	Building
GV_327	0.07	86.31	0.30	0.00	6.99	0.74	5.32	0.00	0.00	0.24	0.00	0.02	0.00		Grass	Roads	Soil	Sky
GV_328	0.03	40.42	0.11	3.39	16.85	1.73	2.07	0.00	8.20	26.67	0.41	0.10	0.02		Grass	Sidewalk	Roads	Sand
GV_329	0.00	62.85	0.00	0.11	7.16	0.10	6.23	0.00	1.01	21.76	0.76	0.01	0.00		Grass	Sidewalk	Roads	Soil
GV_330	0.27	53.70	0.13	0.96	38.72	0.56	0.00	0.00	0.04	4.48	1.13	0.00	0.00		Grass	Roads	Sidewalk	Water
GV_331	0.04	72.01	0.04	1.50	6.37	1.60	17.95	0.09	0.01	0.37	0.02	0.00	0.00		Grass	Soil	Roads	Sky
GV_332	0.64	35.54	0.64	0.12	40.55	0.78	4.35	0.00	0.86	15.59	0.95	0.01	0.00		Roads	Grass	Sidewalk	Soil
GV_333	0.15	65.08	0.17	0.34	27.13	3.10	0.00	0.00	0.00	4.02	0.00	0.00	0.00		Grass	Roads	Sidewalk	Sky
GV_334	0.58	64.61	0.07	0.01	23.51	3.28	2.12	0.00	0.00	5.83	0.00	0.00	0.00		Grass	Roads	Sidewalk	Sky
GV_335	1.47	38.73	9.73	2.03	31.50	2.18	0.01	0.39	0.00	13.85	0.11	0.00	0.01		Grass	Roads	Sidewalk	Building
GV_336	0.55	72.99	0.15	1.61	15.22	5.61	3.38	0.20	0.00	0.13	0.02	0.00	0.15		Grass	Roads	Sky	Soil
GV_337	1.42	50.99	0.93	1.91	42.60	0.10	0.46	0.03	0.28	1.15	0.12	0.01	0.00		Grass	Roads	Tree	Background
GV_338	0.30	49.81	0.21	5.25	18.98	3.27	1.87	0.03	10.59	9.43	0.02	0.25	0.00		Grass	Roads	Sand	Sidewalk
GV_339	0.11	61.77	0.02	0.02	26.97	0.01	1.50	0.07	0.00	9.53	0.00	0.00	0.00		Grass	Roads	Sidewalk	Soil
GV_340	1.35	76.67	0.00	0.62	8.65	0.96	5.44	0.01	0.00	6.24	0.01	0.05	0.00		Grass	Roads	Sidewalk	Soil
GV_341	0.17	48.92	0.29	1.19	42.53	0.47	0.05	0.00	0.81	2.79	2.40	0.36	0.00		Grass	Roads	Sidewalk	Water
GV_342	0.03	51.67	1.24	0.22	42.28	2.18	0.11	0.00	0.02	1.78	0.47	0.00	0.00		Grass	Roads	Sky	Sidewalk
GV_343	0.50	31.56	2.61	0.24	52.04	0.62	1.88	0.00	2.99	7.26	0.31	0.00	0.00		Roads	Grass	Sidewalk	Sand
GV_344	0.99	38.93	2.95	0.07	47.60	1.06	5.94	0.07	0.00	1.58	0.54	0.28	0.00		Roads	Grass	Soil	Building
GV_345	0.12	50.33	0.59	0.01	43.30	1.28	1.89	0.01	0.04	1.21	1.19	0.00	0.00		Grass	Roads	Soil	Sky
GV_346	0.18	80.43	0.02	0.04	17.33	0.27	0.46	0.00	0.01	1.26	0.00	0.00	0.00		Grass	Roads	Sidewalk	Soil
GV_347	0.05	66.17	0.33	0.35	7.12	2.64	16.52	0.00	0.02	6.08	0.52	0.18	0.00		Grass	Soil	Roads	Sidewalk
GV_348	0.01	53.90	0.00	0.00	32.41	0.35	5.98	0.02	0.00	6.58	0.74	0.00	0.00		Grass	Roads	Sidewalk	Soil
GV_349	1.78	42.17	0.04	1.85	42.72	1.95	0.49	0.21	0.70	0.52	7.55	0.01	0.00		Roads	Grass	Water	Sky
GV_350	0.02	49.36	0.01	5.74	35.37	0.46	0.20	0.02	0.25	7.31	1.01	0.25	0.00		Grass	Roads	Sidewalk	Tree
GV_351	0.75	85.93	0.26	0.30	3.27	2.28	3.55	0.02	0.08	3.45	0.10	0.00	0.00		Grass	Soil	Sidewalk	Roads
GV_352	0.99	64.75	4.91	1.56	9.67	3.72	0.26	0.00	0.00	14.13	0.00	0.00	0.00		Grass	Sidewalk	Roads	Building

GV_353	0.55	65.46	0.06	0.03	14.64	14.16	0.00	0.04	0.00	4.41	0.66	0.00	0.00		Grass	Roads	Sky	Sidewalk
GV_354	0.26	53.40	0.46	0.01	28.39	3.61	0.01	0.00	0.00	0.78	13.06	0.02	0.00		Grass	Roads	Water	Sky
GV_355	2.72	55.29	2.67	0.03	29.48	4.94	0.52	0.00	0.00	2.98	1.01	0.35	0.00		Grass	Roads	Sky	Sidewalk
GV_356	0.17	42.66	0.21	2.15	31.91	12.49	0.91	0.01	0.00	9.07	0.38	0.00	0.04		Grass	Roads	Sky	Sidewalk
GV_357	0.09	36.07	0.00	0.21	37.16	1.26	4.98	0.00	0.00	20.21	0.00	0.01	0.00		Roads	Grass	Sidewalk	Soil
GV_358	1.02	47.84	0.47	3.43	39.38	2.13	0.35	0.00	0.24	5.07	0.08	0.00	0.00		Grass	Roads	Sidewalk	Tree
GV_359	0.68	49.58	3.00	2.42	23.84	13.58	0.55	0.02	0.00	6.33	0.00	0.00	0.00		Grass	Roads	Sky	Sidewalk
GV_360	0.04	70.08	0.06	0.27	6.41	2.94	12.23	0.00	0.01	6.61	1.30	0.05	0.00		Grass	Soil	Sidewalk	Roads
GV_361	0.01	90.57	0.00	0.00	1.03	1.63	5.78	0.01	0.00	0.83	0.04	0.00	0.10		Grass	Soil	Sky	Roads
GV_362	13.01	38.22	3.62	0.76	35.66	0.12	0.22	0.00	0.00	7.57	0.84	0.00	0.00		Grass	Roads	Background	Sidewalk
GV_363	0.07	67.04	0.00	0.01	16.90	0.06	6.82	0.00	3.17	4.89	1.04	0.00	0.00		Grass	Roads	Soil	Sidewalk
GV_364	1.34	88.88	0.01	0.00	1.81	0.09	5.32	0.01	0.72	1.82	0.00	0.00	0.00		Grass	Soil	Sidewalk	Roads
GV_365	8.91	79.59	3.22	0.89	3.15	0.19	3.58	0.01	0.00	0.46	0.00	0.00	0.00		Grass	Background	Soil	Building
GV_366	0.19	98.74	0.00	0.00	0.17	0.01	0.84	0.00	0.00	0.04	0.00	0.00	0.00		Grass	Soil	Background	Roads
GV_367	1.32	56.87	0.01	0.00	37.48	0.04	1.89	0.01	0.83	1.30	0.25	0.00	0.00		Grass	Roads	Soil	Background
GV_368	1.57	67.75	0.03	0.08	15.22	0.00	3.08	0.00	0.00	12.27	0.00	0.01	0.00		Grass	Roads	Sidewalk	Soil
GV_369	1.16	66.93	2.82	0.01	16.39	7.01	1.73	0.04	0.36	3.27	0.26	0.03	0.00		Grass	Roads	Sky	Sidewalk
GV_370	0.00	96.79	0.00	0.08	1.38	0.64	0.12	0.00	0.00	0.99	0.00	0.00	0.00		Grass	Roads	Sidewalk	Sky
GV_371	0.02	98.05	0.00	0.00	1.00	0.00	0.82	0.00	0.00	0.10	0.00	0.00	0.00		Grass	Roads	Soil	Sidewalk
GV_372	0.00	99.02	0.00	0.07	0.00	0.42	0.44	0.00	0.00	0.00	0.00	0.00	0.06		Grass	Soil	Sky	Tree
GV_373	0.43	97.13	0.00	0.00	1.95	0.01	0.23	0.00	0.00	0.23	0.00	0.00	0.00		Grass	Roads	Background	Soil
GV_374	4.26	66.71	0.88	0.44	7.78	2.06	17.17	0.00	0.00	0.68	0.00	0.02	0.00		Grass	Soil	Roads	Background
GV_375	0.05	98.40	0.00	0.00	1.07	0.13	0.01	0.00	0.00	0.34	0.00	0.00	0.00		Grass	Roads	Sidewalk	Sky
GV_376	0.01	99.80	0.00	0.00	0.01	0.10	0.03	0.01	0.04	0.00	0.00	0.00	0.00		Grass	Sky	Sand	Soil
GV_377	0.15	99.62	0.00	0.00	0.00	0.00	0.00	0.00	0.22	0.00	0.00	0.00	0.00		Grass	Sand	Background	Water
GV_378	0.60	60.33	0.10	0.00	32.12	0.03	3.50	0.20	0.29	0.99	1.84	0.00	0.00		Grass	Roads	Soil	Water
GV_379	0.00	44.18	0.00	0.00	32.29	0.08	0.62	0.00	0.06	22.43	0.33	0.00	0.00		Grass	Roads	Sidewalk	Soil
GV_380	0.35	82.35	0.80	4.64	0.03	11.79	0.00	0.02	0.00	0.02	0.00	0.00	0.00		Grass	Sky	Tree	Building
GV_381	3.55	66.60	9.68	17.49	0.04	1.29	0.03	0.00	0.00	1.32	0.00	0.00	0.00		Grass	Tree	Building	Background
GV_382	0.59	40.91	0.45	4.80	19.36	25.45	3.19	2.31	0.09	2.44	0.00	0.20	0.21		Grass	Sky	Roads	Tree
GV_383	1.16	10.56	0.65	75.71	0.07	0.28	2.88	0.11	0.00	0.03	0.01	8.53	0.00		Tree	Grass	Gravel	Soil
GV_384	10.72	5.98	17.75	28.03	12.74	2.69	5.11	0.13	2.42	14.34	0.00	0.09	0.00		Tree	Building	Sidewalk	Roads
GV_385	0.72	81.46	0.09	8.81	1.57	6.58	0.13	0.00	0.00	0.19	0.23	0.00	0.22		Grass	Tree	Sky	Roads
GV_386	0.61	36.98	4.53	19.63	0.00	38.20	0.00	0.00	0.00	0.04	0.00	0.00	0.00		Sky	Grass	Tree	Building
GV_387	8.58	3.28	35.58	10.65	12.95	4.09	0.29	0.52	0.01	21.73	0.00	2.31	0.00		Building	Sidewalk	Roads	Tree
GV_388	1.55	33.11	5.19	28.63	22.41	8.38	0.02	0.00	0.00	0.62	0.00	0.08	0.00		Grass	Tree	Roads	Sky
GV_389	6.67	17.71	1.25	16.89	16.67	38.76	0.00	0.73	0.01	0.14	0.05	0.00	1.11		Sky	Grass	Tree	Roads
GV_390	0.00	4.73	0.00	5.16	0.05	27.57	11.64	50.64	0.13	0.05	0.01	0.00	0.01		Bare Rock	Sky	Soil	Tree
GV_391	8.04	28.42	0.20	60.86	0.59	0.62	0.73	0.02	0.00	0.05	0.07	0.40	0.00		Tree	Grass	Background	Soil
GV_392	4.49	32.68	0.05	58.34	0.00	4.39	0.03	0.00	0.00	0.00	0.00	0.02	0.00		Tree	Grass	Background	Sky
GV_393	0.12	23.04	0.01	74.66	0.00	1.39	0.07	0.00	0.00	0.00	0.00	0.71	0.00		Tree	Grass	Sky	Gravel
GV_394	0.46	46.58	0.90	19.28	0.01	3.66	21.23	6.21	0.00	0.01	0.00	1.62	0.03		Grass	Soil	Tree	Bare Rock
GV_395	0.77	51.42	0.83	6.63	15.30	14.89	2.71	0.31	1.07	1.22	2.28	1.94	0.62		Grass	Roads	Sky	Tree
GV_396	9.60	20.27	3.00	19.18	13.06	19.78	2.82	0.34	0.00	0.58	0.99	9.23	1.15		Grass	Sky	Tree	Roads
GV_397	3.49	9.82	1.44	56.21	16.35	5.52	4.10	0.40	0.00	0.34	0.00	2.34	0.00		Tree	Roads	Grass	Sky
GV_398	0.84	10.91	0.02	72.28	0.00	0.85	1.17	0.00	0.00	0.00	0.03	13.90	0.00		Tree	Gravel	Grass	Soil
GV_399	0.47	35.72	0.01	7.60	0.29	9.24	26.43	7.05	6.91	0.00	0.33	5.95	0.01		Grass	Soil	Sky	Tree
GV_400	0.91	6.03	20.25	23.38	12.43	35.80	0.09	0.06	0.00	0.80	0.04	0.22	0.00		Sky	Tree	Building	Roads
GV_401	3.57	10.98	12.78	17.25	1.35	12.11	0.14	1.79	0.36	39.47	0.14	0.00	0.05		Sidewalk	Tree	Building	Sky
GV_402	0.47	10.96	0.47	0.50	44.06	0.65	0.10	2.01	1.25	37.87	1.67	0.00	0.00		Roads	Sidewalk	Grass	Bare Rock
GV_403	11.36	43.02	6.04	3.24	13.79	0.92	0.02	0.04	0.72	20.39	0.43	0.02	0.00		Grass	Sidewalk	Roads	Background
GV_404	1.18	24.11	0.13	47.18	15.95	1.48	9.64	0.34	0.00	0.00	0.00	0.00	0.01		Tree	Grass	Roads	Soil
GV_405	2.15	38.31	13.40	23.09	6.61	8.57	0.00	0.00	0.00	7.83	0.00	0.00	0.02		Grass	Tree	Building	Sky
GV_406	0.79	2.08	37.32	19.68	0.77	29.36	2.90	0.52	0.01	6.54	0.00	0.00	0.03		Building	Sky	Tree	Sidewalk
GV_407	6.84	15.27	16.25	24.64	9.07	18.73	1.24	0.86	0.00	6.74	0.00	0.21	0.15		Tree	Sky	Building	Grass
GV_408	10.93	12.40	21.91	26.86	11.12	2.21	0.11	0.00	0.00	14.35	0.00	0.12	0.00		Tree	Building	Sidewalk	Grass
GV_409	4.34	42.53	25.42	15.18	3.53	6.76	0.24	0.40	0.00	1.59	0.00	0.01	0.00		Grass	Building	Tree	Sky
GV_410	2.83	36.28	11.63	8.72	7.15	31.13	0.34	0.03	0.00	0.37	0.00	1.48	0.02		Grass	Sky	Building	Tree
GV_411	2.69	32.63	12.48	8.73	12.17	28.93	0.35	0.04	0.00	0.54	0.00	1.31	0.12		Grass	Sky	Building	Roads

GV_412	2.29	34.26	14.51	7.84	7.98	29.11	0.23	0.08	0.00	0.24	0.00	3.42	0.04		Grass	Sky	Building	Roads
GV_413	0.91	89.94	4.09	4.50	0.36	0.16	0.00	0.00	0.00	0.03	0.00	0.00	0.00		Grass	Tree	Building	Background
GV_414	2.32	16.62	1.40	56.30	12.63	7.53	1.32	0.26	0.00	0.18	0.00	1.45	0.00		Tree	Grass	Roads	Sky
GV_415	0.56	46.26	0.08	14.05	0.01	12.85	22.12	0.20	0.00	0.00	0.00	2.15	1.72		Grass	Soil	Tree	Sky
GV_416	5.40	30.87	5.25	11.43	0.18	3.29	13.31	22.51	0.00	0.18	7.55	0.03	0.00		Grass	Bare Rock	Soil	Tree
GV_417	0.88	62.55	0.93	14.81	0.04	19.91	0.86	0.00	0.00	0.01	0.00	0.01	0.00		Grass	Sky	Tree	Building
GV_418	0.65	85.47	0.49	9.39	0.34	3.59	0.06	0.00	0.00	0.00	0.00	0.02	0.00		Grass	Tree	Sky	Background
GV_419	3.84	69.88	11.05	7.17	0.15	2.98	0.00	0.00	0.00	4.86	0.00	0.07	0.00		Grass	Building	Tree	Sidewalk
GV_420	11.95	6.60	5.37	52.35	9.34	5.41	0.06	0.00	0.00	4.90	0.20	3.81	0.00		Tree	Background	Roads	Grass
GV_421	12.17	1.86	28.65	22.06	17.69	7.06	0.37	9.83	0.00	0.28	0.00	0.03	0.00		Building	Tree	Roads	Background
GV_422	12.42	25.24	18.71	17.53	0.98	20.76	2.15	0.06	0.00	0.01	0.00	2.14	0.00		Grass	Sky	Building	Tree
GV_423	4.71	38.16	1.85	45.77	2.24	4.58	2.20	0.19	0.04	0.00	0.09	0.13	0.03		Tree	Grass	Background	Sky
GV_424	1.72	18.40	0.00	0.54	1.78	0.61	71.90	0.00	4.44	0.51	0.00	0.08	0.00		Soil	Grass	Sand	Roads
GV_425	1.20	43.68	10.53	28.80	1.16	12.69	0.00	0.00	0.00	1.94	0.00	0.00	0.00		Grass	Tree	Sky	Building
GV_426	0.54	99.43	0.00	0.00	0.00	0.00	0.00	0.00	0.00	0.00	0.00	0.02	0.00		Grass	Background	Gravel	Water
GV_427	1.19	98.81	0.00	0.00	0.00	0.00	0.00	0.00	0.00	0.00	0.00	0.00	0.00		Grass	Background	Building	Building
GV_428	2.90	97.10	0.00	0.00	0.00	0.00	0.00	0.00	0.00	0.00	0.00	0.00	0.00		Grass	Background	Tree	Soil
GV_429	1.11	43.55	9.96	32.77	0.20	11.08	0.00	0.00	0.00	1.33	0.00	0.00	0.00		Grass	Tree	Sky	Building
GV_430	3.83	96.17	0.00	0.00	0.00	0.00	0.00	0.00	0.00	0.00	0.00	0.00	0.00		Grass	Background	Building	Building
GV_431	8.70	28.06	23.64	4.58	1.10	29.92	1.18	2.58	0.00	0.12	0.00	0.02	0.08		Sky	Grass	Building	Background
GV_432	2.35	23.46	5.39	35.28	0.55	24.46	5.52	0.01	0.03	2.76	0.00	0.17	0.02		Tree	Sky	Grass	Soil
GV_433	4.40	19.52	6.27	10.42	18.96	39.44	0.06	0.56	0.01	0.24	0.00	0.01	0.11		Sky	Grass	Roads	Tree
GV_434	0.58	53.35	0.02	10.32	0.01	18.21	0.17	11.29	0.00	0.01	0.00	0.00	6.05		Grass	Sky	Bare Rock	Tree
GV_435	0.14	40.42	0.14	15.87	1.19	26.18	2.54	0.04	0.47	6.61	0.93	5.47	0.00		Grass	Sky	Tree	Sidewalk
GV_436	2.62	18.09	0.84	56.81	0.09	16.17	4.51	0.06	0.03	0.07	0.01	0.61	0.09		Tree	Grass	Sky	Soil
GV_437	1.51	7.07	0.06	77.08	0.06	1.53	11.41	0.60	0.00	0.01	0.01	0.67	0.00		Tree	Soil	Grass	Sky
GV_438	1.77	75.10	3.70	10.74	0.55	8.08	0.01	0.00	0.00	0.05	0.00	0.00	0.00		Grass	Tree	Sky	Building
GV_439	4.22	37.12	10.34	19.59	16.57	10.38	0.76	0.09	0.03	0.51	0.13	0.23	0.01		Grass	Tree	Roads	Sky
GV_440	0.69	55.20	0.38	6.69	13.65	22.83	0.19	0.01	0.00	0.03	0.00	0.31	0.01		Grass	Sky	Roads	Tree
GV_441	4.57	6.61	33.34	21.57	13.23	16.98	0.19	0.82	0.00	1.49	0.00	1.20	0.00		Building	Tree	Sky	Roads
GV_442	2.35	19.85	20.73	10.05	18.49	18.06	5.21	0.03	0.00	3.38	0.00	1.85	0.00		Building	Grass	Roads	Sky
GV_443	2.39	8.60	19.11	23.64	4.60	19.78	3.86	1.63	0.02	15.63	0.00	0.41	0.33		Tree	Sky	Building	Sidewalk
GV_444	0.06	44.02	0.33	17.17	5.60	27.16	0.96	0.03	0.00	4.41	0.00	0.24	0.03		Grass	Sky	Tree	Roads
GV_445	0.57	36.87	0.42	13.21	13.17	20.68	8.09	0.00	0.59	5.57	0.00	0.82	0.01		Grass	Sky	Tree	Roads
GV_446	0.17	67.33	0.03	4.36	5.96	16.31	1.04	0.00	0.00	1.28	3.44	0.00	0.08		Grass	Sky	Roads	Tree
GV_447	0.26	77.32	0.45	14.84	2.14	4.96	0.01	0.00	0.00	0.01	0.00	0.00	0.00		Grass	Tree	Sky	Roads
GV_448	6.84	11.53	4.18	27.98	5.44	5.12	14.02	11.63	1.82	8.76	0.00	2.67	0.00		Tree	Soil	Bare Rock	Grass
GV_449	1.67	91.08	4.43	2.04	0.48	0.00	0.12	0.00	0.00	0.18	0.00	0.00	0.00		Grass	Building	Tree	Background
GV_450	2.70	77.22	1.22	12.17	0.08	6.60	0.02	0.00	0.00	0.00	0.00	0.00	0.00		Grass	Tree	Sky	Background
GV_451	0.19	14.63	0.07	4.36	0.00	34.82	34.75	11.04	0.02	0.00	0.00	0.03	0.09		Sky	Soil	Grass	Bare Rock
GV_452	1.77	51.70	1.33	15.79	0.00	29.02	0.30	0.04	0.00	0.00	0.00	0.00	0.03		Grass	Sky	Tree	Background
GV_453	0.95	36.15	1.18	19.27	0.00	22.52	10.16	9.76	0.00	0.00	0.00	0.02	0.00		Grass	Sky	Tree	Soil
GV_454	1.43	29.27	0.92	11.10	0.46	34.40	8.73	13.34	0.00	0.00	0.15	0.00	0.20		Sky	Grass	Bare Rock	Tree
GV_455	0.60	36.22	0.05	21.05	0.00	23.37	17.91	0.07	0.00	0.70	0.00	0.00	0.03		Grass	Sky	Tree	Soil
GV_456	0.37	45.82	0.95	18.10	0.00	20.30	3.39	11.07	0.00	0.00	0.00	0.00	0.01		Grass	Sky	Tree	Bare Rock
GV_457	0.40	52.07	0.07	22.16	0.00	25.29	0.00	0.01	0.00	0.00	0.00	0.00	0.00		Grass	Sky	Tree	Background
GV_458	2.58	41.22	15.49	16.82	10.91	3.25	3.99	0.03	0.00	4.03	0.28	1.40	0.00		Grass	Tree	Building	Roads
GV_459	0.72	17.59	0.00	64.23	0.26	0.07	13.48	2.52	0.00	0.00	0.00	1.12	0.00		Tree	Grass	Soil	Bare Rock
GV_460	0.18	5.45	0.36	81.90	9.41	0.02	2.58	0.10	0.00	0.00	0.00	0.00	0.00		Tree	Roads	Grass	Soil
GV_461	0.22	2.05	0.12	85.06	0.58	0.02	11.23	0.00	0.00	0.00	0.00	0.72	0.00		Tree	Soil	Grass	Gravel
GV_462	0.36	3.58	0.00	84.95	0.00	0.10	10.99	0.00	0.00	0.00	0.00	0.01	0.00		Tree	Soil	Grass	Background
GV_463	0.39	21.42	0.00	72.38	0.14	0.16	5.31	0.00	0.00	0.00	0.01	0.19	0.00		Tree	Grass	Soil	Background
GV_464	0.57	29.33	0.00	58.01	0.00	0.10	11.62	0.12	0.00	0.00	0.00	0.25	0.00		Tree	Grass	Soil	Background
GV_465	0.00	21.38	0.00	78.09	0.00	0.00	0.52	0.00	0.00	0.00	0.00	0.01	0.00		Tree	Grass	Soil	Gravel
GV_466	2.85	29.51	0.02	46.48	11.23	2.57	2.55	3.33	0.00	0.06	0.00	1.40	0.00		Tree	Grass	Roads	Bare Rock
GV_467	2.35	36.21	0.27	23.60	21.87	10.39	0.43	2.46	0.00	0.13	0.00	1.96	0.33		Grass	Tree	Roads	Sky
GV_468	0.13	42.53	0.00	41.61	13.74	0.10	0.01	0.00	0.00	0.02	0.01	1.85	0.00		Grass	Tree	Roads	Gravel
GV_469	0.45	8.44	0.00	65.78	12.57	0.05	11.68	0.01	0.00	0.00	0.00	1.02	0.00		Tree	Roads	Soil	Grass
GV_470	0.91	40.47	0.02	42.80	0.02	0.02	1.03	0.12	0.00	0.00	11.21	3.40	0.00		Tree	Grass	Water	Gravel

GV_471	6.11	12.76	0.24	45.10	0.90	6.90	5.03	14.94	0.02	0.02	0.33	7.53	0.13		Tree	Bare Rock	Grass	Gravel
GV_472	1.25	14.12	0.01	67.41	1.62	1.25	0.33	0.01	0.00	0.00	0.00	14.00	0.00		Tree	Grass	Gravel	Roads
GV_473	2.37	30.53	0.94	20.00	3.48	10.29	27.39	2.16	0.00	0.82	0.00	0.23	1.78		Grass	Soil	Tree	Sky
GV_474	1.42	2.04	0.00	5.46	6.88	16.47	8.32	43.55	0.77	10.30	3.88	0.02	0.87		Bare Rock	Sky	Sidewalk	Soil
GV_475	2.08	1.68	0.05	5.97	19.67	17.22	28.42	22.45	0.00	0.04	0.03	0.39	2.01		Soil	Bare Rock	Roads	Sky
GV_476	3.45	14.07	21.60	18.15	15.63	10.61	6.20	0.37	0.16	7.15	0.11	2.49	0.01		Building	Tree	Roads	Grass
GV_477	11.88	0.34	6.85	3.48	57.91	18.03	0.01	1.20	0.00	0.06	0.00	0.00	0.23		Roads	Sky	Background	Building
GV_478	0.99	16.09	2.86	46.71	6.83	4.50	0.47	0.00	0.00	0.13	0.00	21.42	0.01		Tree	Gravel	Grass	Roads
GV_479	0.70	4.59	4.04	66.88	12.73	0.44	8.93	0.00	0.00	0.09	0.00	1.58	0.00		Tree	Roads	Soil	Grass
GV_480	2.91	13.35	0.05	50.50	10.39	0.47	1.16	0.20	0.00	0.02	0.00	20.95	0.00		Tree	Gravel	Grass	Roads
GV_481	7.74	6.34	0.27	67.06	4.07	0.33	1.63	3.24	0.01	0.01	0.05	9.26	0.00		Tree	Gravel	Background	Grass
GV_482	2.81	12.23	0.01	72.66	0.86	0.15	10.15	0.02	0.00	0.00	0.00	1.12	0.00		Tree	Grass	Soil	Background
GV_483	1.16	31.53	0.01	55.09	9.95	0.37	0.37	0.00	0.00	0.05	0.00	1.45	0.00		Tree	Grass	Roads	Gravel
GV_484	1.20	4.77	0.04	78.53	10.69	0.04	4.73	0.00	0.00	0.00	0.00	0.00	0.00		Tree	Roads	Grass	Soil
GV_485	1.00	10.85	0.00	75.70	0.00	0.03	7.56	0.06	0.00	0.00	0.00	4.80	0.00		Tree	Grass	Soil	Gravel
GV_486	1.05	17.51	0.03	71.21	2.62	0.38	1.08	0.03	0.00	0.00	0.02	6.08	0.00		Tree	Grass	Gravel	Roads
GV_487	4.81	18.14	0.40	13.30	4.98	20.57	0.93	21.25	0.00	0.41	1.02	12.25	1.94		Bare Rock	Sky	Grass	Tree
GV_488	3.55	19.68	0.47	39.57	8.64	7.66	3.38	7.66	0.00	0.00	0.35	7.84	1.20		Tree	Grass	Roads	Gravel
GV_489	1.08	5.85	0.00	73.22	0.01	0.07	17.81	0.00	0.00	0.00	0.00	1.97	0.00		Tree	Soil	Grass	Gravel
GV_490	0.04	10.15	0.99	39.55	35.21	5.39	0.04	0.00	0.13	8.46	0.01	0.02	0.00		Tree	Roads	Grass	Sidewalk
GV_491	0.13	9.99	1.16	29.05	43.86	2.83	0.00	0.00	0.00	12.98	0.00	0.00	0.00		Roads	Tree	Sidewalk	Grass
GV_492	0.12	5.55	2.61	25.39	48.22	9.48	0.00	0.00	0.00	8.60	0.00	0.00	0.04		Roads	Tree	Sky	Sidewalk
GV_493	0.23	33.88	0.34	42.11	12.01	3.20	0.01	0.02	0.59	2.78	0.35	4.51	0.00		Tree	Grass	Roads	Gravel
GV_494	0.19	15.46	0.01	50.79	16.30	8.66	0.09	0.00	0.44	5.18	0.00	2.86	0.02		Tree	Roads	Grass	Sky
GV_495	0.28	17.64	0.05	73.97	0.01	0.11	7.42	0.19	0.00	0.00	0.00	0.33	0.00		Tree	Grass	Soil	Gravel
GV_496	8.20	34.18	0.04	46.39	0.30	0.04	3.93	0.10	0.00	0.09	0.00	6.71	0.02		Tree	Grass	Background	Gravel
GV_497	9.21	19.72	4.61	2.31	24.74	12.40	18.04	4.42	0.00	0.88	0.01	1.60	2.07		Roads	Grass	Soil	Sky
GV_498	3.09	11.61	0.82	6.95	7.28	11.69	47.02	8.91	0.00	0.00	0.14	1.50	0.99		Soil	Sky	Grass	Bare Rock
GV_499	0.23	3.19	0.01	2.34	30.99	7.86	12.55	39.42	0.00	0.01	0.01	1.00	2.39		Bare Rock	Roads	Soil	Sky
GV_500	0.76	8.88	0.01	7.38	5.29	5.30	66.73	0.37	0.00	0.00	0.01	2.78	2.48		Soil	Grass	Tree	Sky
GV_501	1.92	14.46	0.08	55.49	0.76	2.31	22.49	0.36	0.00	0.07	0.02	2.05	0.00		Tree	Soil	Grass	Sky
GV_502	3.17	14.22	1.71	10.15	7.15	10.12	28.82	20.54	1.18	0.80	0.03	0.63	1.49		Soil	Bare Rock	Grass	Tree
GV_503	0.83	1.13	0.01	0.91	2.88	7.53	47.54	34.53	0.00	0.19	0.23	1.10	3.13		Soil	Bare Rock	Sky	Blue Mountain
GV_504	2.37	5.67	0.14	11.20	1.71	11.52	43.60	20.10	0.00	0.01	1.17	0.20	2.32		Soil	Bare Rock	Sky	Tree
GV_505	2.30	7.85	0.49	5.47	13.75	7.82	43.42	14.01	0.04	0.18	1.25	2.82	0.62		Soil	Bare Rock	Roads	Grass
GV_506	1.75	9.38	1.25	28.37	36.42	10.51	0.83	0.04	0.06	11.07	0.00	0.32	0.00		Roads	Tree	Sidewalk	Sky
GV_507	3.63	4.24	0.01	52.24	0.95	0.03	37.51	0.34	0.00	0.00	0.00	1.05	0.00		Tree	Soil	Grass	Background
GV_508	0.19	7.48	0.03	64.90	0.66	0.01	20.19	0.00	0.00	0.00	0.02	6.53	0.00		Tree	Soil	Grass	Gravel
GV_509	0.29	15.59	0.00	55.33	1.89	0.03	19.76	0.02	0.00	0.00	0.37	6.72	0.00		Tree	Soil	Grass	Gravel
GV_510	0.55	31.40	0.19	28.52	13.11	4.94	15.33	0.03	0.07	4.80	0.00	0.95	0.10		Grass	Tree	Soil	Roads
GV_511	0.13	4.74	0.00	84.04	0.00	0.00	10.88	0.16	0.00	0.00	0.00	0.03	0.00		Tree	Soil	Grass	Bare Rock
GV_512	0.42	3.75	0.24	19.62	61.10	9.79	0.38	1.98	0.00	0.82	0.00	0.01	1.89		Roads	Tree	Sky	Grass
GV_513	1.13	30.54	0.03	64.02	0.88	0.01	2.69	0.02	0.00	0.00	0.00	0.67	0.00		Tree	Grass	Soil	Background
GV_514	0.67	6.10	0.03	12.66	10.08	4.96	48.10	5.87	0.00	0.00	1.26	9.56	0.72		Soil	Tree	Roads	Gravel
GV_515	0.21	6.90	0.07	10.89	16.49	5.56	48.43	8.94	0.00	0.02	0.96	0.58	0.97		Soil	Roads	Tree	Bare Rock
GV_516	0.16	46.37	0.01	16.85	1.30	3.14	26.84	0.12	0.00	0.00	0.01	3.90	1.31		Grass	Soil	Tree	Gravel
GV_517	0.23	23.34	0.00	27.42	0.56	1.43	30.84	0.24	0.00	0.00	0.00	15.94	0.00		Soil	Tree	Grass	Gravel
GV_518	0.16	22.87	0.00	36.52	0.21	2.31	27.24	0.12	0.00	0.08	0.00	10.49	0.00		Tree	Soil	Grass	Gravel
GV_519	0.28	27.92	0.13	37.04	0.49	2.05	15.40	6.76	0.00	0.00	0.00	9.94	0.00		Tree	Grass	Soil	Gravel
GV_520	0.79	12.91	0.01	18.06	0.01	6.17	58.50	0.18	0.00	0.00	0.00	2.48	0.91		Soil	Tree	Grass	Sky
GV_521	0.36	20.80	0.02	14.16	13.10	5.26	40.28	0.06	0.11	0.01	0.00	4.26	1.57		Soil	Grass	Tree	Roads
GV_522	0.49	4.49	0.02	7.69	1.04	6.22	40.20	29.05	0.19	0.00	1.06	7.97	1.57		Soil	Bare Rock	Gravel	Tree
GV_523	0.28	15.90	0.00	71.75	0.00	0.17	10.70	0.00	0.00	0.00	0.00	1.19	0.00		Tree	Grass	Soil	Gravel
GV_524	1.11	25.31	0.20	68.35	0.79	0.07	2.50	0.26	0.00	0.03	0.04	1.34	0.00		Tree	Grass	Soil	Gravel
GV_525	0.27	30.73	0.00	58.79	1.04	0.04	5.51	2.14	0.00	0.00	0.00	1.47	0.00		Tree	Grass	Soil	Bare Rock
GV_526	0.40	22.66	0.03	73.68	0.32	0.03	0.43	0.70	0.00	1.61	0.00	0.14	0.00		Tree	Grass	Sidewalk	Bare Rock
GV_527	1.05	23.15	0.35	65.65	0.18	0.00	1.90	2.90	0.00	0.02	0.01	4.80	0.00		Tree	Grass	Gravel	Bare Rock
GV_528	0.15	20.36	0.00	76.52	0.04	0.21	0.70	0.72	0.00	0.00	0.00	1.30	0.00		Tree	Grass	Gravel	Bare Rock
GV_529	0.10	23.99	0.00	66.23	0.00	0.02	8.86	0.03	0.00	0.00	0.00	0.76	0.00		Tree	Grass	Soil	Gravel

GV_530	1.80	29.13	0.00	60.01	0.56	0.74	3.55	0.01	0.00	0.38	0.00	3.81	0.01		Tree	Grass	Gravel	Soil
GV_531	0.58	21.59	0.00	62.50	1.11	0.04	7.51	1.65	0.02	0.00	0.07	4.94	0.00		Tree	Grass	Soil	Gravel
GV_532	0.97	58.08	0.00	24.04	0.16	1.09	4.41	3.62	0.99	0.43	0.00	6.22	0.00		Grass	Tree	Gravel	Soil
GV_533	0.42	14.02	0.00	70.92	0.44	0.31	1.81	9.37	0.00	0.00	0.00	2.70	0.00		Tree	Grass	Bare Rock	Gravel
GV_534	0.28	9.19	0.00	78.25	0.00	0.20	10.29	1.30	0.00	0.00	0.01	0.47	0.00		Tree	Soil	Grass	Bare Rock
GV_535	0.35	40.23	0.00	46.88	0.03	0.04	10.52	0.89	0.00	0.03	0.00	1.02	0.00		Tree	Grass	Soil	Gravel
GV_536	0.05	22.76	0.00	65.53	1.31	0.03	8.59	0.01	0.00	0.00	0.00	1.72	0.00		Tree	Grass	Soil	Gravel
GV_537	0.08	4.41	0.00	87.46	0.12	0.00	7.52	0.00	0.00	0.00	0.00	0.41	0.00		Tree	Soil	Grass	Gravel
GV_538	1.19	45.12	0.05	47.97	1.33	0.03	2.79	0.06	0.00	0.09	0.00	1.38	0.00		Tree	Grass	Soil	Gravel
GV_539	0.13	20.24	0.00	74.59	0.97	0.07	3.41	0.02	0.00	0.00	0.00	0.56	0.00		Tree	Grass	Soil	Roads
GV_540	4.52	19.00	0.01	60.90	0.01	0.01	15.10	0.00	0.00	0.00	0.00	0.44	0.00		Tree	Grass	Soil	Background
GV_541	0.27	73.89	0.04	25.35	0.00	0.41	0.01	0.00	0.00	0.00	0.00	0.02	0.00		Grass	Tree	Sky	Background
GV_542	0.62	31.42	0.01	61.55	0.00	0.00	5.94	0.01	0.00	0.00	0.01	0.43	0.00		Tree	Grass	Soil	Background
GV_543	4.32	32.13	0.01	52.70	0.64	1.72	7.31	0.21	0.10	0.20	0.36	0.30	0.00		Tree	Grass	Soil	Background
GV_544	1.58	34.77	0.01	55.73	0.12	0.07	5.93	0.00	0.00	0.00	0.07	1.71	0.00		Tree	Grass	Soil	Gravel
GV_545	1.23	13.15	0.00	63.98	0.11	0.39	19.51	0.11	0.00	0.00	0.06	1.46	0.00		Tree	Soil	Grass	Gravel
GV_546	2.76	7.55	0.00	79.61	0.13	0.08	9.40	0.07	0.00	0.00	0.00	0.38	0.00		Tree	Soil	Grass	Background
GV_547	1.83	62.79	0.06	21.43	1.06	0.16	11.65	0.72	0.00	0.00	0.01	0.29	0.00		Grass	Tree	Soil	Background
GV_548	1.86	28.16	0.01	61.36	0.37	0.06	7.00	0.17	0.00	0.07	0.00	0.93	0.00		Tree	Grass	Soil	Background
GV_549	0.27	55.25	0.09	32.07	0.01	1.15	8.66	0.66	0.00	0.00	0.01	1.80	0.02		Grass	Tree	Soil	Gravel
GV_550	1.89	36.31	0.06	53.72	0.85	0.31	5.49	0.03	0.00	0.00	0.00	1.35	0.00		Tree	Grass	Soil	Background
GV_551	1.70	32.96	0.00	62.51	0.00	0.55	2.19	0.01	0.00	0.00	0.00	0.08	0.00		Tree	Grass	Soil	Background
GV_552	0.53	68.03	0.25	28.39	0.00	1.82	0.54	0.01	0.00	0.00	0.00	0.43	0.00		Grass	Tree	Sky	Soil
GV_553	2.02	40.77	0.05	45.33	0.00	6.38	4.66	0.05	0.00	0.01	0.32	0.41	0.00		Tree	Grass	Sky	Soil
GV_554	0.36	63.88	0.06	24.30	0.51	3.15	7.21	0.22	0.00	0.00	0.00	0.29	0.02		Grass	Tree	Soil	Sky
GV_555	1.11	66.32	6.13	23.88	0.78	1.58	0.04	0.01	0.00	0.08	0.00	0.06	0.00		Grass	Tree	Building	Sky
GV_556	0.46	59.33	0.20	35.70	1.49	2.73	0.05	0.00	0.00	0.00	0.00	0.00	0.03		Grass	Tree	Sky	Roads
GV_557	0.66	51.66	0.13	41.88	0.00	5.32	0.07	0.03	0.00	0.00	0.00	0.16	0.08		Grass	Tree	Sky	Background
GV_558	0.41	56.68	1.01	35.21	0.00	4.44	1.97	0.01	0.00	0.00	0.00	0.26	0.00		Grass	Tree	Sky	Soil
GV_559	0.07	63.50	0.49	29.46	0.00	6.20	0.00	0.00	0.00	0.00	0.00	0.00	0.28		Grass	Tree	Sky	Building
GV_560	0.08	62.17	0.33	34.75	0.00	2.43	0.23	0.00	0.00	0.00	0.00	0.01	0.01		Grass	Tree	Sky	Building
GV_561	0.57	57.02	0.40	32.46	1.45	4.71	2.71	0.03	0.00	0.00	0.00	0.63	0.02		Grass	Tree	Sky	Soil
GV_562	4.46	16.18	0.01	68.39	0.14	0.03	10.48	0.00	0.00	0.00	0.01	0.30	0.00		Tree	Grass	Soil	Background
GV_563	0.18	80.64	1.03	5.84	0.00	12.27	0.04	0.00	0.00	0.00	0.00	0.00	0.00		Grass	Sky	Tree	Building
GV_564	2.42	17.52	16.07	14.04	41.88	7.72	0.02	0.01	0.00	0.32	0.00	0.01	0.00		Roads	Grass	Building	Tree
GV_565	2.18	15.88	3.25	25.09	36.51	11.02	0.33	0.06	0.00	5.64	0.00	0.01	0.04		Roads	Tree	Grass	Sky
GV_566	0.46	34.60	0.00	41.73	0.00	18.10	4.01	0.16	0.00	0.00	0.00	0.89	0.02		Tree	Grass	Sky	Soil
GV_567	0.41	43.80	7.33	21.24	5.87	8.80	0.02	0.00	0.00	12.30	0.00	0.24	0.00		Grass	Tree	Sidewalk	Sky
GV_568	0.50	5.36	0.07	1.94	3.70	17.07	65.72	1.74	0.21	3.21	0.16	0.02	0.31		Soil	Sky	Grass	Roads
GV_569	3.71	23.55	17.53	33.46	14.95	3.63	0.24	0.00	0.00	2.70	0.00	0.24	0.00		Tree	Grass	Building	Roads
GV_570	0.25	12.04	0.09	2.46	8.29	10.57	43.98	3.71	16.61	1.77	0.18	0.00	0.04		Soil	Sand	Grass	Sky
GV_571	6.49	2.15	0.12	2.84	55.71	9.40	0.32	0.37	21.54	0.09	0.93	0.00	0.04		Roads	Sand	Sky	Background
GV_572	1.03	71.42	1.71	4.16	1.81	10.35	0.98	0.00	0.00	8.43	0.01	0.00	0.10		Grass	Sky	Sidewalk	Tree
GV_573	2.13	38.35	12.35	34.05	6.24	1.91	2.30	0.24	0.00	1.13	0.00	1.31	0.00		Grass	Tree	Building	Roads
GV_574	7.47	42.92	10.49	25.25	3.76	0.52	1.19	0.06	0.00	8.02	0.01	0.32	0.00		Grass	Tree	Building	Sidewalk
GV_575	5.40	21.10	20.26	8.44	30.27	9.20	0.43	0.66	0.00	3.73	0.00	0.50	0.00		Roads	Grass	Building	Sky
GV_576	3.19	27.52	4.22	11.23	26.44	19.02	0.02	0.01	0.00	8.34	0.00	0.00	0.00		Grass	Roads	Sky	Tree
GV_577	3.35	30.61	4.66	12.54	18.82	12.66	1.04	0.01	0.00	15.52	0.00	0.78	0.00		Grass	Roads	Sidewalk	Sky
GV_578	3.44	32.88	3.90	34.53	16.47	0.24	1.88	0.05	0.15	5.97	0.14	0.34	0.00		Tree	Grass	Roads	Sidewalk
GV_579	1.20	42.33	1.37	48.67	0.00	0.14	5.73	0.02	0.00	0.00	0.00	0.54	0.00		Tree	Grass	Soil	Building
GV_580	0.65	40.28	0.09	32.78	8.47	1.72	1.95	6.83	0.00	6.91	0.00	0.31	0.00		Grass	Tree	Roads	Sidewalk
GV_581	0.84	31.01	0.01	42.27	8.29	0.15	11.91	0.00	0.00	0.75	0.00	4.76	0.00		Tree	Grass	Soil	Roads
GV_582	1.69	15.04	0.18	46.88	14.44	1.33	5.25	0.71	0.01	3.89	0.00	10.57	0.00		Tree	Grass	Roads	Gravel
GV_583	1.54	37.11	0.00	44.62	2.82	0.08	6.65	0.03	0.00	1.67	0.00	5.49	0.00		Tree	Grass	Soil	Gravel
GV_584	0.35	65.71	0.27	20.87	5.47	4.35	0.07	0.07	0.00	0.10	0.00	2.74	0.00		Grass	Tree	Roads	Sky
GV_585	4.43	34.59	3.95	41.46	0.31	5.93	1.77	0.35	0.00	6.17	0.00	1.01	0.01		Tree	Grass	Sidewalk	Sky
GV_586	6.93	44.10	0.84	38.48	1.96	0.31	5.61	0.33	0.00	0.57	0.00	0.85	0.00		Grass	Tree	Background	Soil
GV_587	0.32	68.93	0.02	18.52	0.33	1.94	5.32	0.05	0.00	0.00	0.00	4.56	0.01		Grass	Tree	Soil	Gravel
GV_588	0.15	83.54	0.00	2.89	11.86	0.35	0.29	0.02	0.00	0.00	0.00	0.91	0.00		Grass	Roads	Tree	Gravel

GV_589	2.01	42.54	3.33	15.92	19.76	14.35	0.50	0.04	0.00	0.85	0.00	0.70	0.00		Grass	Roads	Tree	Sky
GV_590	5.06	26.95	25.04	24.34	4.47	6.94	0.38	1.50	0.00	4.47	0.00	0.84	0.00		Grass	Building	Tree	Sky
GV_591	2.26	47.42	0.00	39.92	1.04	3.34	0.69	0.00	0.00	3.17	0.05	2.10	0.00		Grass	Tree	Sky	Sidewalk
GV_592	11.63	16.57	3.41	53.57	3.59	1.45	2.83	0.05	0.00	6.35	0.00	0.55	0.00		Tree	Grass	Background	Sidewalk
GV_593	0.26	9.90	0.00	52.86	0.28	0.86	32.23	0.09	0.00	0.04	0.00	3.45	0.00		Tree	Soil	Grass	Gravel
GV_594	0.75	11.15	0.00	71.17	0.63	0.12	15.43	0.27	0.00	0.03	0.00	0.44	0.00		Tree	Soil	Grass	Background
GV_595	3.11	25.70	0.35	49.76	0.08	0.98	15.97	0.02	0.00	0.00	0.00	4.02	0.00		Tree	Grass	Soil	Gravel
GV_596	13.24	11.20	7.77	54.35	0.24	0.92	2.27	7.12	0.01	0.86	0.03	1.99	0.00		Tree	Background	Grass	Building
GV_597	4.09	31.60	3.26	30.95	0.10	0.02	14.22	0.12	0.00	0.00	11.23	4.40	0.00		Grass	Tree	Soil	Water
GV_598	5.71	44.84	0.16	36.17	10.27	0.63	1.80	0.30	0.00	0.00	0.00	0.12	0.00		Grass	Tree	Roads	Background
GV_599	22.24	21.86	0.01	36.67	0.07	0.18	8.67	0.01	0.42	0.00	0.00	9.87	0.00		Tree	Background	Grass	Gravel
GV_600	1.02	40.18	19.32	29.25	1.01	0.58	4.91	0.00	0.00	0.83	0.00	2.91	0.00		Grass	Tree	Building	Soil
GV_601	3.64	20.42	3.48	48.72	8.69	0.25	1.78	1.03	0.00	8.58	0.04	3.38	0.00		Tree	Grass	Roads	Sidewalk
GV_602	1.37	48.59	1.44	29.00	1.50	0.83	0.20	0.07	0.00	1.11	0.00	15.89	0.00		Grass	Tree	Gravel	Roads
GV_603	0.02	60.02	0.15	25.06	0.17	0.83	2.35	0.00	0.00	0.00	0.00	11.40	0.00		Grass	Tree	Gravel	Soil
GV_604	1.17	43.56	0.00	43.05	0.32	0.22	0.60	0.00	0.00	0.69	0.00	10.39	0.00		Grass	Tree	Gravel	Background
GV_605	2.63	51.33	1.47	20.05	6.16	9.93	5.03	0.00	0.00	3.39	0.00	0.01	0.00		Grass	Tree	Sky	Roads
GV_606	0.93	70.13	0.03	22.06	0.02	5.33	0.23	0.00	0.00	0.11	0.00	1.15	0.00		Grass	Tree	Sky	Gravel
GV_607	0.21	17.23	4.24	26.59	36.81	7.00	0.14	0.05	0.00	6.69	0.00	1.05	0.00		Roads	Tree	Grass	Sky
GV_608	6.04	7.08	17.13	26.71	30.79	6.99	0.00	0.00	0.00	5.19	0.00	0.06	0.00		Roads	Tree	Building	Grass
GV_609	1.72	37.82	0.30	10.60	34.46	10.74	4.29	0.00	0.00	0.02	0.01	0.04	0.01		Grass	Roads	Sky	Tree
GV_610	1.83	17.52	7.14	12.57	6.83	8.88	45.05	0.11	0.00	0.00	0.00	0.06	0.00		Soil	Grass	Tree	Sky
GV_611	4.51	14.48	21.05	2.42	32.11	18.42	5.89	0.25	0.00	0.36	0.00	0.51	0.00		Roads	Building	Sky	Grass
GV_612	1.97	17.71	1.53	10.09	25.27	16.54	18.64	0.95	3.97	1.36	0.85	1.09	0.02		Roads	Soil	Grass	Sky
GV_613	4.90	1.68	21.86	5.48	32.47	12.57	5.88	1.70	0.02	6.57	2.76	4.04	0.08		Roads	Building	Sky	Sidewalk
GV_614	7.16	25.13	6.18	10.17	19.36	9.86	1.97	4.30	0.01	4.06	7.93	3.66	0.21		Grass	Roads	Tree	Sky
GV_615	5.01	9.87	21.95	2.69	11.56	10.22	1.28	4.36	0.14	14.32	17.86	0.61	0.12		Building	Water	Sidewalk	Roads
GV_616	5.63	27.01	8.02	11.48	27.65	11.99	2.74	0.02	0.00	0.50	0.09	4.85	0.01		Roads	Grass	Sky	Tree
GV_617	7.69	24.30	11.42	7.38	13.12	11.51	0.83	0.82	0.57	2.08	19.96	0.26	0.06		Grass	Water	Roads	Sky
GV_618	10.75	4.15	43.40	9.40	5.47	6.25	2.71	11.11	0.00	6.60	0.00	0.15	0.00		Building	Bare Rock	Background	Tree
GV_619	7.82	3.88	71.44	1.84	2.37	3.75	0.00	0.11	0.00	8.72	0.00	0.06	0.00		Building	Sidewalk	Background	Grass
GV_620	7.69	48.85	11.55	21.24	0.10	6.91	0.35	1.70	0.00	1.26	0.06	0.24	0.02		Grass	Tree	Building	Background
GV_621	3.10	12.46	19.28	20.35	33.91	5.06	0.08	0.01	0.00	5.32	0.00	0.44	0.00		Roads	Tree	Building	Grass
GV_622	2.28	62.29	9.27	17.05	0.07	7.75	0.09	0.00	0.00	1.17	0.00	0.02	0.01		Grass	Tree	Building	Sky
GV_623	3.26	44.90	0.83	19.36	5.68	2.20	15.90	4.93	0.00	2.81	0.00	0.13	0.00		Grass	Tree	Soil	Roads
GV_624	2.99	26.78	7.33	21.21	22.08	12.52	4.63	0.31	0.00	1.41	0.00	0.65	0.10		Grass	Roads	Tree	Sky
GV_625	8.67	0.53	35.07	1.70	25.86	9.55	9.30	0.35	0.01	7.60	0.00	1.37	0.00		Building	Roads	Sky	Soil
GV_626	9.21	0.26	24.47	2.64	41.68	18.76	0.01	0.39	0.00	2.58	0.00	0.01	0.00		Roads	Building	Sky	Background
GV_627	13.25	10.00	29.87	0.77	26.30	11.83	0.02	0.67	0.00	6.85	0.00	0.44	0.00		Building	Roads	Background	Sky
GV_628	1.15	30.41	3.85	30.55	10.96	3.15	8.11	0.03	3.96	7.76	0.02	0.05	0.00		Tree	Grass	Roads	Soil
GV_629	2.90	5.87	8.52	8.90	50.19	16.16	1.86	0.78	0.00	0.26	0.00	4.55	0.00		Roads	Sky	Tree	Building
GV_630	6.90	4.47	13.70	7.21	42.71	11.82	2.10	7.89	0.06	2.59	0.00	0.53	0.00		Roads	Building	Sky	Bare Rock
GV_631	0.06	14.19	0.59	36.62	44.10	4.16	0.00	0.00	0.00	0.04	0.00	0.24	0.00		Roads	Tree	Grass	Sky
GV_632	0.66	47.48	0.07	23.10	21.54	6.90	0.18	0.07	0.00	0.00	0.00	0.02	0.00		Grass	Tree	Roads	Sky
GV_633	0.64	59.37	0.10	36.96	0.02	2.32	0.58	0.01	0.00	0.00	0.00	0.00	0.00		Grass	Tree	Sky	Background
GV_634	0.18	63.24	0.00	23.82	0.14	0.06	12.40	0.12	0.00	0.00	0.00	0.04	0.00		Grass	Tree	Soil	Background
GV_635	0.73	67.89	0.00	24.08	1.53	0.00	4.47	0.60	0.00	0.00	0.01	0.70	0.00		Grass	Tree	Soil	Roads
GV_636	1.09	60.54	0.01	18.62	6.82	5.52	0.44	0.21	0.00	5.20	0.01	1.54	0.00		Grass	Tree	Roads	Sky
GV_637	0.42	69.12	0.60	18.12	0.03	8.06	0.12	0.36	0.00	0.00	3.18	0.00	0.00		Grass	Tree	Sky	Water
GV_638	0.40	62.88	0.14	16.15	3.72	5.57	9.28	0.09	0.00	0.00	0.00	1.76	0.00		Grass	Tree	Soil	Sky
GV_639	0.01	48.07	0.01	41.08	0.10	0.04	2.13	0.00	0.00	0.00	7.76	0.81	0.00		Grass	Tree	Water	Soil
GV_640	0.00	64.86	0.00	31.93	0.00	0.02	2.66	0.00	0.00	0.00	0.00	0.53	0.00		Grass	Tree	Soil	Gravel
GV_641	1.28	22.75	0.20	24.67	20.13	8.26	5.00	0.27	0.00	0.07	0.00	17.37	0.00		Tree	Grass	Roads	Gravel
GV_642	3.72	53.08	14.33	15.32	0.85	12.22	0.01	0.00	0.00	0.46	0.00	0.00	0.00		Grass	Tree	Building	Sky
GV_643	7.24	56.18	9.03	16.38	0.39	10.33	0.03	0.00	0.00	0.43	0.00	0.00	0.00		Grass	Tree	Sky	Building
GV_644	10.31	14.64	13.25	20.33	21.24	14.50	0.29	0.12	0.10	5.19	0.00	0.02	0.00		Roads	Tree	Grass	Sky
GV_645	0.80	25.30	0.14	17.18	0.37	56.06	0.00	0.00	0.00	0.03	0.00	0.00	0.13		Sky	Grass	Tree	Background
GV_646	0.05	57.45	0.09	13.01	0.21	22.43	0.01	0.00	0.35	1.67	2.45	0.00	2.29		Grass	Sky	Tree	Water
GV_647	4.01	34.73	14.34	10.67	2.60	16.36	0.09	0.00	0.00	16.21	0.00	0.99	0.00		Grass	Sky	Sidewalk	Building

GV_648	0.87	66.54	0.26	30.34	0.00	1.45	0.08	0.37	0.00	0.00	0.00	0.09	0.00		Grass	Tree	Sky	Background
GV_649	3.48	85.17	0.91	8.68	0.56	0.83	0.34	0.00	0.00	0.00	0.01	0.00	0.02		Grass	Tree	Background	Building
GV_650	4.36	38.25	5.27	39.84	2.68	5.19	0.21	0.06	0.00	0.46	0.00	3.68	0.00		Tree	Grass	Building	Sky
GV_651	9.86	24.50	19.02	25.67	3.46	12.16	0.01	0.01	0.00	5.14	0.00	0.17	0.00		Tree	Grass	Building	Sky
GV_652	0.62	69.63	0.00	0.00	15.25	0.01	9.31	0.00	0.00	1.13	0.63	3.40	0.00		Grass	Roads	Soil	Gravel
GV_653	3.61	33.78	2.67	7.10	14.05	4.93	24.79	2.57	0.04	0.31	0.00	6.12	0.04		Grass	Soil	Roads	Tree
GV_654	12.18	6.69	8.42	32.67	14.55	10.78	0.04	0.02	0.00	14.64	0.00	0.01	0.00		Tree	Sidewalk	Roads	Background
GV_655	3.18	24.44	2.64	19.31	4.46	17.71	0.22	0.16	0.02	26.93	0.15	0.01	0.78		Sidewalk	Grass	Tree	Sky
GV_656	6.25	11.36	39.13	18.40	3.34	14.45	0.04	1.51	0.00	5.48	0.00	0.04	0.00		Building	Tree	Sky	Grass
GV_657	1.78	60.85	2.99	24.94	0.32	8.59	0.00	0.00	0.00	0.06	0.00	0.12	0.34		Grass	Tree	Sky	Building
GV_658	0.41	44.32	3.86	32.62	1.62	5.88	10.13	0.66	0.00	0.01	0.00	0.26	0.21		Grass	Tree	Soil	Sky
GV_659	1.62	62.93	1.52	5.67	0.58	27.06	0.05	0.00	0.00	0.38	0.14	0.00	0.04		Grass	Sky	Tree	Background
GV_660	0.22	49.68	0.14	35.97	0.30	11.00	0.95	0.60	0.00	0.20	0.00	0.91	0.03		Grass	Tree	Sky	Soil
GV_661	8.37	9.08	24.64	9.96	36.98	7.24	0.28	0.01	0.00	3.43	0.00	0.00	0.00		Roads	Building	Tree	Grass
GV_662	2.59	14.81	33.05	3.34	0.84	41.38	0.00	2.02	0.00	1.92	0.00	0.00	0.04		Sky	Building	Grass	Tree
GV_663	0.92	54.24	5.43	35.04	0.01	4.33	0.00	0.00	0.00	0.03	0.00	0.00	0.00		Grass	Tree	Building	Sky
GV_664	1.46	30.99	1.46	17.22	11.42	16.08	1.15	1.76	1.08	0.94	6.45	8.54	1.46		Grass	Tree	Sky	Roads
GV_665	2.56	39.09	3.76	31.69	10.48	8.73	2.11	0.16	0.00	0.74	0.01	0.34	0.33		Grass	Tree	Roads	Sky
GV_666	2.38	53.50	7.17	3.49	9.61	1.02	0.15	0.07	0.15	22.47	0.00	0.00	0.00		Grass	Sidewalk	Roads	Building
GV_667	1.00	1.45	0.07	10.90	25.79	21.95	2.61	0.58	23.19	1.73	3.83	0.02	6.88		Roads	Sand	Sky	Tree
GV_668	7.19	37.65	5.02	18.58	14.47	5.93	2.07	0.01	0.00	0.09	0.00	8.53	0.47		Grass	Tree	Roads	Gravel
GV_669	0.26	52.24	1.11	24.03	6.90	8.14	0.74	0.14	1.38	1.38	1.57	1.06	1.05		Grass	Tree	Sky	Roads
GV_670	0.64	55.81	0.66	32.24	0.31	5.77	1.08	0.00	0.00	2.68	0.00	0.34	0.46		Grass	Tree	Sky	Sidewalk
GV_671	0.38	59.12	0.59	27.17	2.24	8.47	0.21	0.00	0.00	0.30	0.00	0.02	1.48		Grass	Tree	Sky	Roads
GV_672	0.32	67.72	0.95	18.52	3.27	4.95	1.39	0.00	0.01	0.19	0.60	1.65	0.43		Grass	Tree	Sky	Roads
GV_673	0.40	50.55	1.41	19.07	9.14	4.38	0.44	0.18	0.00	12.79	0.00	0.13	1.52		Grass	Tree	Sidewalk	Roads
GV_674	2.04	16.56	18.70	26.73	16.24	5.44	0.39	1.69	0.00	9.05	0.02	3.13	0.00		Tree	Building	Grass	Roads
GV_675	0.79	32.45	0.46	26.64	19.98	10.91	4.05	0.13	0.00	3.58	0.00	0.35	0.66		Grass	Tree	Roads	Sky
GV_676	0.40	36.67	0.01	22.96	9.06	9.75	5.89	6.43	0.01	0.67	0.27	6.71	1.17		Grass	Tree	Sky	Roads
GV_677	0.79	29.32	0.45	31.29	11.50	4.77	6.48	5.80	0.00	3.13	0.00	6.46	0.00		Tree	Grass	Roads	Soil
GV_678	1.37	58.83	1.12	29.67	2.88	3.97	0.03	0.01	0.00	1.91	0.00	0.18	0.02		Grass	Tree	Sky	Roads
GV_679	2.23	42.79	3.58	22.17	2.61	6.52	18.08	0.88	0.00	0.01	0.06	1.07	0.01		Grass	Tree	Soil	Sky
GV_680	2.21	56.31	1.33	29.12	1.97	4.46	1.89	0.08	0.01	0.04	0.07	2.00	0.51		Grass	Tree	Sky	Background
GV_681	2.38	26.66	0.57	5.92	34.53	21.43	2.89	4.40	0.00	1.15	0.05	0.01	0.01		Roads	Grass	Sky	Tree
GV_682	4.58	18.40	2.69	2.39	30.08	36.01	3.56	1.46	0.00	0.05	0.00	0.76	0.01		Sky	Roads	Grass	Background
GV_683	1.72	59.31	0.02	3.06	18.65	7.12	0.89	0.48	0.67	6.65	1.14	0.23	0.06		Grass	Roads	Sky	Sidewalk
GV_684	5.74	32.40	0.32	4.17	29.04	6.99	9.77	1.83	0.00	5.08	0.01	4.38	0.29		Grass	Roads	Soil	Sky
GV_685	2.14	36.09	0.25	19.75	30.35	1.37	9.40	0.01	0.00	0.29	0.00	0.35	0.01		Grass	Roads	Tree	Soil
GV_686	2.90	48.98	3.05	25.29	0.14	10.54	7.99	0.17	0.17	0.71	0.03	0.01	0.00		Grass	Tree	Sky	Soil
GV_687	3.63	18.52	1.01	16.34	32.58	26.48	0.05	0.10	0.00	1.17	0.00	0.09	0.03		Roads	Sky	Grass	Tree
GV_688	2.72	37.06	3.82	36.96	2.14	2.00	5.77	0.07	6.54	2.28	0.00	0.64	0.00		Grass	Tree	Sand	Soil
GV_689	9.10	20.31	1.67	3.37	30.68	29.37	0.04	0.12	0.00	4.44	0.00	0.38	0.52		Roads	Sky	Grass	Background
GV_690	2.73	37.10	9.10	36.86	1.20	5.25	0.42	0.28	0.00	3.26	0.00	3.82	0.00		Grass	Tree	Building	Sky
GV_691	7.99	13.20	10.24	17.93	3.12	29.82	0.14	7.36	0.00	9.95	0.00	0.14	0.11		Sky	Tree	Grass	Building
GV_692	16.14	13.06	10.41	9.41	15.35	29.04	0.00	0.02	0.00	6.57	0.00	0.00	0.00		Sky	Background	Roads	Grass
GV_693	0.56	54.09	8.52	21.20	6.64	7.46	0.21	0.00	0.00	0.99	0.00	0.13	0.21		Grass	Tree	Building	Sky
GV_694	1.13	28.02	0.19	39.36	19.36	5.58	5.97	0.14	0.00	0.00	0.00	0.22	0.03		Tree	Grass	Roads	Soil
GV_695	0.55	19.93	0.23	46.54	17.31	0.16	0.22	0.00	0.00	6.33	0.00	8.74	0.00		Tree	Grass	Roads	Gravel
GV_696	4.00	11.08	0.61	61.44	14.82	0.06	5.22	0.04	0.00	0.02	0.00	2.70	0.00		Tree	Roads	Grass	Soil
GV_697	0.09	21.29	0.01	67.19	7.40	0.73	0.13	0.05	0.00	0.00	0.00	3.11	0.00		Tree	Grass	Roads	Gravel
GV_698	0.65	27.14	0.29	49.06	0.59	0.01	7.74	0.00	0.00	0.02	12.25	2.25	0.00		Tree	Grass	Water	Soil
GV_699	1.34	42.15	0.05	33.90	2.91	5.81	0.64	0.54	0.00	0.00	0.00	12.64	0.03		Grass	Tree	Gravel	Sky
GV_700	0.80	46.19	0.03	41.50	0.00	1.72	4.68	0.04	0.00	0.00	3.23	1.81	0.00		Grass	Tree	Soil	Water
GV_701	7.38	1.60	14.19	7.90	33.75	4.39	0.11	1.13	0.00	29.31	0.00	0.24	0.00		Roads	Sidewalk	Building	Tree
GV_702	1.31	63.26	1.39	4.01	16.68	11.47	0.00	0.01	0.00	0.04	0.00	0.09	1.73		Grass	Roads	Sky	Tree
GV_703	2.88	50.16	0.24	1.79	12.20	5.72	5.63	0.23	0.17	5.51	15.33	0.10	0.03		Grass	Water	Roads	Sky
GV_704	1.34	66.91	10.39	14.39	0.89	1.53	0.78	0.00	0.00	3.75	0.01	0.00	0.00		Grass	Tree	Building	Sidewalk
GV_705	2.49	78.45	1.82	13.48	0.03	3.52	0.00	0.00	0.00	0.21	0.00	0.00	0.00		Grass	Tree	Sky	Background
GV_706	1.39	16.03	7.11	28.12	30.41	1.13	0.00	0.07	0.00	15.73	0.00	0.00	0.00		Roads	Tree	Grass	Sidewalk

GV_707	1.24	46.31	0.05	8.01	13.87	7.51	11.36	0.53	0.27	0.18	10.49	0.06	0.11		Grass	Roads	Soil	Water
GV_708	1.76	76.17	1.36	2.80	0.25	17.23	0.00	0.00	0.00	0.32	0.00	0.00	0.11		Grass	Sky	Tree	Background
GV_709	2.46	68.25	1.43	5.30	0.91	14.69	2.93	0.00	0.00	2.45	0.37	1.14	0.07		Grass	Sky	Tree	Soil
GV_710	0.05	84.43	0.01	5.21	0.62	8.32	0.66	0.00	0.00	0.45	0.00	0.13	0.13		Grass	Sky	Tree	Soil
GV_711	2.56	3.50	15.35	3.95	41.03	2.45	0.15	0.13	1.33	21.45	0.00	8.09	0.00		Roads	Sidewalk	Building	Gravel
GV_712	7.13	19.64	9.24	0.33	41.90	17.56	0.33	0.56	0.00	2.75	0.00	0.57	0.00		Roads	Grass	Sky	Building
GV_713	5.59	5.20	2.22	19.71	29.28	6.77	9.27	13.10	5.51	2.68	0.09	0.59	0.00		Roads	Tree	Bare Rock	Soil
GV_714	5.43	12.32	2.30	35.58	14.26	6.38	1.67	4.17	0.05	16.66	0.00	1.18	0.00		Tree	Sidewalk	Roads	Grass
GV_715	6.82	25.39	2.59	23.53	24.64	4.63	0.31	0.03	1.74	9.42	0.19	0.72	0.00		Grass	Roads	Tree	Sidewalk
GV_716	8.28	42.01	0.20	26.54	19.38	1.51	1.34	0.00	0.00	0.21	0.00	0.50	0.00		Grass	Tree	Roads	Background
GV_717	5.45	25.76	12.90	15.91	16.91	13.12	1.88	1.09	0.00	3.59	0.02	3.29	0.07		Grass	Roads	Tree	Sky
GV_718	2.26	35.02	2.21	7.71	0.03	0.01	0.01	0.18	0.00	52.12	0.00	0.47	0.00		Sidewalk	Grass	Tree	Background
GV_719	5.59	21.55	2.98	28.67	20.37	5.51	4.74	2.88	0.00	2.90	0.12	4.68	0.00		Tree	Grass	Roads	Background
GV_720	2.69	36.77	2.81	20.78	7.28	11.24	1.34	0.71	0.03	8.57	1.01	6.33	0.43		Grass	Tree	Sky	Sidewalk
GV_721	1.27	7.70	0.41	10.65	48.24	1.94	9.46	6.18	0.00	13.86	0.08	0.19	0.02		Roads	Sidewalk	Tree	Soil
GV_722	8.51	4.79	2.68	46.04	10.24	14.47	0.17	0.41	2.97	8.27	1.05	0.40	0.01		Tree	Sky	Roads	Background
GV_723	2.99	29.81	1.58	13.53	15.64	6.22	22.10	3.75	0.00	2.11	0.05	2.20	0.01		Grass	Soil	Roads	Tree
GV_724	1.88	49.15	2.91	13.57	18.86	10.37	0.88	0.11	0.00	0.52	0.03	1.71	0.00		Grass	Roads	Tree	Sky
GV_725	0.64	63.88	3.92	23.70	0.43	6.17	1.08	0.00	0.00	0.18	0.00	0.00	0.00		Grass	Tree	Sky	Building
GV_726	12.38	38.10	5.96	18.36	0.20	8.05	6.59	0.18	10.13	0.01	0.00	0.04	0.00		Grass	Tree	Background	Sand
GV_727	1.04	42.22	8.49	16.83	18.18	8.44	3.00	0.05	0.01	0.20	0.01	1.53	0.00		Grass	Roads	Tree	Building
GV_728	2.77	22.38	7.96	5.55	16.27	15.02	8.62	14.48	0.15	6.27	0.06	0.43	0.03		Grass	Roads	Sky	Bare Rock
GV_729	2.14	28.25	6.06	9.23	27.93	8.49	0.59	3.41	0.03	13.60	0.14	0.12	0.02		Grass	Roads	Sidewalk	Tree
GV_730	3.49	95.91	0.02	0.43	0.06	0.00	0.02	0.01	0.00	0.06	0.00	0.00	0.00		Grass	Background	Tree	Sidewalk
GV_731	5.29	32.60	27.39	23.82	2.11	4.45	0.33	0.04	0.00	3.68	0.00	0.29	0.00		Grass	Building	Tree	Background
GV_732	2.89	31.73	24.27	25.85	7.97	1.21	0.07	0.04	0.00	4.83	0.00	1.13	0.00		Grass	Tree	Building	Roads
GV_733	22.00	25.33	18.06	11.89	11.45	1.33	2.87	2.72	0.00	3.44	0.91	0.00	0.00		Grass	Background	Building	Tree
GV_734	1.21	37.84	8.73	13.71	10.99	0.01	0.78	0.01	17.01	9.35	0.35	0.00	0.00		Grass	Sand	Tree	Roads
GV_735	1.66	20.81	9.99	41.17	13.62	4.83	1.24	0.00	0.00	3.70	0.00	2.97	0.00		Tree	Grass	Roads	Building
GV_736	3.11	2.18	18.87	3.47	60.45	3.77	0.23	0.82	0.00	4.82	0.00	2.26	0.00		Roads	Building	Sidewalk	Sky
GV_737	0.98	40.31	3.57	40.47	0.65	7.18	0.06	0.11	0.00	2.31	0.00	4.36	0.00		Tree	Grass	Sky	Gravel
GV_738	3.74	96.26	0.00	0.00	0.00	0.00	0.00	0.00	0.00	0.00	0.00	0.00	0.00		Grass	Background	Soil	Tree
GV_739	1.91	39.99	0.91	49.09	3.04	0.68	0.44	0.14	0.00	3.58	0.00	0.21	0.00		Tree	Grass	Sidewalk	Roads
GV_740	1.59	88.79	1.25	6.74	0.00	0.00	0.00	0.00	0.00	1.62	0.00	0.00	0.00		Grass	Tree	Sidewalk	Background
GV_741	1.34	60.48	1.97	16.27	0.47	0.65	6.19	3.99	1.79	0.13	0.52	6.20	0.00		Grass	Tree	Gravel	Soil
GV_742	0.24	40.78	0.25	0.01	4.93	0.03	0.19	0.00	0.00	53.25	0.00	0.33	0.00		Sidewalk	Grass	Roads	Gravel
GV_743	5.68	58.57	0.16	30.49	0.00	4.85	0.00	0.02	0.00	0.22	0.00	0.00	0.00		Grass	Tree	Background	Sky
GV_744	3.14	50.52	1.24	29.79	3.58	2.86	3.64	0.58	0.14	0.84	0.00	3.66	0.01		Grass	Tree	Gravel	Soil
GV_745	1.26	65.13	1.66	21.38	1.96	6.19	0.58	0.66	0.00	0.70	0.01	0.45	0.03		Grass	Tree	Sky	Roads
GV_746	5.00	10.96	4.76	21.36	44.69	1.17	0.11	0.04	0.00	6.90	0.00	4.99	0.00		Roads	Tree	Grass	Sidewalk
GV_747	3.26	53.36	1.19	18.18	9.14	4.86	6.84	2.35	0.00	0.03	0.01	0.72	0.07		Grass	Tree	Roads	Soil
GV_748	5.27	6.44	0.72	19.88	50.05	7.55	7.94	0.09	0.00	0.04	0.00	1.76	0.25		Roads	Tree	Soil	Sky
GV_749	0.56	29.76	0.15	14.30	19.92	18.74	2.97	1.81	0.00	0.20	0.61	10.50	0.48		Grass	Roads	Sky	Tree
GV_750	8.09	8.70	5.10	9.16	11.63	32.66	10.27	9.23	0.91	1.67	0.06	0.05	2.48		Sky	Roads	Soil	Bare Rock
GV_751	8.31	7.74	34.13	6.33	2.48	18.20	6.78	13.69	0.01	1.25	0.21	0.75	0.11		Building	Sky	Bare Rock	Background
GV_752	7.11	8.01	0.84	2.83	77.09	2.29	0.08	1.36	0.04	0.30	0.05	0.00	0.00		Roads	Grass	Background	Tree
GV_753	1.08	10.93	2.95	11.44	66.14	6.12	0.38	0.05	0.00	0.01	0.00	0.88	0.02		Roads	Tree	Grass	Sky
GV_754	1.15	31.23	3.94	3.57	23.14	15.28	18.90	2.57	0.00	0.10	0.00	0.08	0.05		Grass	Roads	Soil	Sky
GV_755	6.80	15.39	1.32	4.88	45.82	18.65	3.85	2.56	0.01	0.09	0.00	0.30	0.33		Roads	Sky	Grass	Background
GV_756	1.45	35.81	0.01	0.02	44.03	12.13	0.80	0.00	5.25	0.00	0.45	0.05	0.00		Roads	Grass	Sky	Sand
GV_757	0.26	39.47	0.16	0.11	41.51	13.32	1.84	0.00	2.75	0.01	0.00	0.56	0.00		Roads	Grass	Sky	Sand
GV_758	0.28	44.00	14.07	16.01	2.50	8.66	0.00	0.22	0.00	14.14	0.00	0.10	0.01		Grass	Tree	Sidewalk	Building
GV_759	0.60	63.40	0.08	2.33	18.43	6.60	0.49	3.78	2.10	0.26	1.28	0.31	0.35		Grass	Roads	Sky	Bare Rock
GV_760	0.47	38.64	0.01	10.78	20.42	6.55	4.34	3.61	14.09	0.00	0.32	0.62	0.14		Grass	Roads	Sand	Tree
GV_761	2.97	41.85	1.72	11.20	27.02	2.84	6.29	0.77	0.87	0.02	4.01	0.45	0.00		Grass	Roads	Tree	Soil
GV_762	0.39	28.78	1.00	18.62	30.06	7.33	2.59	0.61	0.00	9.44	0.00	1.19	0.00		Roads	Grass	Tree	Sidewalk
GV_763	1.76	52.98	0.26	30.11	0.12	5.13	8.34	0.07	0.00	0.00	0.00	0.18	1.06		Grass	Tree	Soil	Sky
GV_764	1.06	46.06	0.40	38.08	1.32	8.00	3.69	0.24	0.00	0.00	0.14	0.89	0.12		Grass	Tree	Sky	Soil
GV_765	3.93	26.88	0.12	49.21	5.01	1.18	10.65	0.62	0.00	0.00	0.00	2.40	0.00		Tree	Grass	Soil	Roads

GV_766	23.02	15.65	0.18	44.98	7.04	2.24	0.76	2.64	0.14	0.94	0.01	2.38	0.00		Tree	Background	Grass	Roads
GV_767	4.09	28.98	0.28	48.80	4.41	1.88	7.08	0.04	0.00	0.00	0.00	4.44	0.00		Tree	Grass	Soil	Gravel
GV_768	10.00	58.57	0.68	4.34	2.39	3.92	17.48	0.35	0.08	0.33	0.01	1.86	0.00		Grass	Soil	Background	Tree
GV_769	2.59	35.30	8.83	19.05	5.99	11.39	7.84	0.46	0.14	1.37	0.41	6.56	0.07		Grass	Tree	Sky	Building
GV_770	1.63	35.74	0.13	32.26	18.70	2.05	5.96	2.46	0.00	0.24	0.00	0.83	0.00		Grass	Tree	Roads	Soil
GV_771	0.04	13.36	0.29	64.52	20.47	0.28	0.02	0.01	0.00	0.25	0.00	0.76	0.00		Tree	Roads	Grass	Gravel
GV_772	3.40	8.15	18.81	13.52	38.09	14.55	0.01	0.54	0.00	2.24	0.01	0.01	0.66		Roads	Building	Sky	Tree
GV_773	3.94	3.03	2.05	13.71	52.30	9.92	1.50	8.93	0.56	0.00	0.03	3.25	0.77		Roads	Tree	Sky	Bare Rock
GV_774	0.90	8.22	6.07	4.83	37.89	6.69	4.67	27.68	0.02	1.35	0.00	1.43	0.24		Roads	Bare Rock	Grass	Sky
GV_775	1.95	36.10	0.05	34.40	10.45	4.50	10.67	0.42	0.00	0.00	0.00	1.47	0.00		Grass	Tree	Soil	Roads
GV_776	6.74	67.06	0.29	10.99	11.47	2.47	0.73	0.01	0.00	0.00	0.00	0.25	0.00		Grass	Roads	Tree	Background
GV_777	0.04	77.57	0.87	7.05	0.00	14.04	0.14	0.00	0.00	0.00	0.00	0.29	0.00		Grass	Sky	Tree	Building
GV_778	1.29	29.13	2.25	35.22	11.66	9.00	6.33	2.58	0.00	0.24	0.00	2.30	0.00		Tree	Grass	Roads	Sky
GV_779	2.36	44.63	5.04	27.09	7.03	11.65	0.35	0.30	0.04	0.52	0.01	0.78	0.19		Grass	Tree	Sky	Roads
GV_780	0.74	36.93	0.08	15.70	5.93	16.35	6.17	0.06	3.18	0.00	11.16	3.69	0.00		Grass	Sky	Tree	Water
GV_781	0.25	62.63	0.91	18.16	0.03	6.11	3.41	8.44	0.00	0.00	0.02	0.02	0.01		Grass	Tree	Bare Rock	Sky
GV_782	1.89	40.47	6.81	48.37	0.00	1.63	0.80	0.00	0.00	0.03	0.00	0.00	0.00		Tree	Grass	Building	Background
GV_783	1.45	34.63	6.97	6.42	5.37	9.06	34.38	0.00	0.00	0.77	0.00	0.95	0.00		Grass	Soil	Sky	Building
GV_784	0.34	18.08	10.40	5.29	1.54	17.22	46.84	0.26	0.00	0.02	0.00	0.00	0.00		Soil	Grass	Sky	Building
GV_785	1.26	26.68	0.46	3.24	53.56	12.34	0.06	1.75	0.00	0.00	0.00	0.63	0.01		Roads	Grass	Sky	Tree
GV_786	1.41	52.18	0.64	27.28	0.14	4.16	7.62	0.05	1.08	0.24	0.15	4.92	0.14		Grass	Tree	Soil	Gravel
GV_787	6.57	22.18	2.10	3.46	31.38	18.79	3.34	4.64	0.00	7.12	0.11	0.30	0.00		Roads	Grass	Sky	Sidewalk
GV_788	2.40	20.25	2.29	11.95	5.71	13.96	0.08	1.17	0.19	0.59	41.33	0.04	0.04		Water	Grass	Sky	Tree
GV_789	3.95	7.63	6.29	14.56	29.11	12.23	12.72	0.40	0.00	0.66	0.00	12.46	0.00		Roads	Tree	Soil	Gravel
GV_790	0.17	81.44	0.02	1.38	0.00	12.10	0.93	3.30	0.00	0.01	0.00	0.48	0.18		Grass	Sky	Bare Rock	Tree
GV_791	0.14	52.22	0.01	3.10	11.39	8.38	22.09	0.06	0.00	0.03	0.01	2.56	0.00		Grass	Soil	Roads	Sky
GV_792	0.27	91.85	0.02	2.60	0.00	2.53	2.61	0.02	0.10	0.00	0.00	0.00	0.00		Grass	Soil	Tree	Sky
GV_793	2.78	17.01	6.26	1.05	20.15	7.95	28.30	7.71	0.00	0.24	0.60	7.16	0.80		Soil	Roads	Grass	Sky
GV_794	0.88	34.96	0.41	0.05	50.54	8.22	1.28	2.75	0.00	0.09	0.01	0.63	0.17		Roads	Grass	Sky	Bare Rock
GV_795	0.12	39.23	0.19	23.56	25.36	7.31	0.48	2.95	0.00	0.00	0.00	0.80	0.00		Grass	Roads	Tree	Sky
GV_796	1.18	5.55	0.20	0.18	41.75	10.20	12.93	23.14	0.27	0.04	0.33	3.32	0.92		Roads	Bare Rock	Soil	Sky
GV_797	0.07	34.14	0.08	0.03	43.49	4.74	3.25	6.52	0.00	0.01	0.01	7.64	0.01		Roads	Grass	Gravel	Bare Rock
GV_798	0.71	0.93	0.08	0.16	18.41	5.49	1.08	49.23	0.00	3.45	0.00	20.45	0.02		Bare Rock	Gravel	Roads	Sky
GV_799	1.45	50.36	0.36	0.76	9.99	8.09	21.72	6.81	0.00	0.00	0.06	0.36	0.06		Grass	Soil	Roads	Sky
GV_800	0.99	39.51	0.01	0.65	2.31	1.65	23.63	12.00	8.24	1.19	0.77	9.06	0.00		Grass	Soil	Bare Rock	Gravel
GV_801	1.23	68.55	0.23	4.33	1.99	3.01	9.77	3.80	3.60	0.01	3.14	0.34	0.00		Grass	Soil	Tree	Bare Rock
GV_802	1.02	32.54	0.14	5.33	43.91	3.61	0.41	10.30	0.00	0.17	0.49	2.08	0.00		Roads	Grass	Bare Rock	Tree
GV_803	1.06	50.50	0.34	19.65	9.36	6.37	5.02	0.52	0.00	3.29	0.00	3.90	0.00		Grass	Tree	Roads	Sky
GV_804	1.13	19.36	35.85	4.15	7.12	10.90	6.92	0.57	0.12	12.88	0.01	0.33	0.68		Building	Grass	Sidewalk	Sky
GV_805	4.03	57.13	0.10	34.25	0.10	0.44	1.23	1.80	0.04	0.14	0.00	0.75	0.00		Grass	Tree	Background	Bare Rock
GV_806	0.17	1.32	17.95	13.67	30.64	9.58	0.18	0.74	0.00	25.72	0.00	0.00	0.04		Roads	Sidewalk	Building	Tree
GV_807	1.75	37.77	1.88	22.70	1.08	14.55	5.44	14.75	0.00	0.05	0.00	0.00	0.03		Grass	Tree	Bare Rock	Sky
GV_808	2.61	5.06	4.31	22.34	25.15	9.43	1.03	7.89	0.00	20.79	0.00	1.22	0.17		Roads	Tree	Sidewalk	Sky
GV_809	10.48	3.20	16.53	15.90	14.69	5.36	9.42	17.80	0.03	5.64	0.00	0.94	0.00		Bare Rock	Building	Tree	Roads
GV_810	3.52	21.29	3.49	53.61	4.55	2.66	8.48	0.01	0.00	0.23	0.00	2.13	0.01		Tree	Grass	Soil	Roads
GV_811	3.50	41.79	0.70	30.10	2.43	5.73	12.09	0.27	0.00	0.16	0.00	1.16	2.07		Grass	Tree	Soil	Sky
GV_812	1.85	24.57	1.66	58.79	0.74	1.08	9.59	0.37	0.00	0.00	0.01	1.34	0.00		Tree	Grass	Soil	Background
GV_813	3.68	39.53	0.24	23.48	8.03	8.16	10.28	0.72	0.00	0.05	0.62	4.98	0.23		Grass	Tree	Soil	Sky
GV_814	4.44	12.68	0.00	27.21	8.22	1.74	29.82	14.82	0.90	0.13	0.01	0.04	0.00		Soil	Tree	Bare Rock	Grass
GV_815	2.77	46.53	0.04	30.25	8.50	2.88	3.44	1.59	0.00	0.00	0.03	3.98	0.00		Grass	Tree	Roads	Gravel
GV_816	1.34	27.84	0.14	51.24	9.30	0.88	4.30	0.20	0.02	0.00	0.00	4.72	0.01		Tree	Grass	Roads	Gravel
GV_817	2.99	51.48	3.79	21.38	6.63	7.81	2.65	1.92	0.00	0.50	0.01	0.65	0.19		Grass	Tree	Sky	Roads
GV_818	4.07	38.69	0.00	42.55	5.77	2.78	1.07	0.18	0.50	1.96	0.00	2.43	0.00		Tree	Grass	Roads	Background
GV_819	4.92	32.36	2.66	34.85	15.45	6.00	1.69	1.07	0.00	0.10	0.20	0.70	0.00		Tree	Grass	Roads	Sky
GV_820	5.10	53.95	1.66	31.30	0.80	4.86	0.00	0.13	0.00	0.34	0.83	0.48	0.56		Grass	Tree	Background	Sky
GV_821	6.70	26.67	9.59	38.35	0.59	3.42	0.01	0.05	0.00	14.57	0.00	0.05	0.00		Tree	Grass	Sidewalk	Building
GV_822	7.49	17.61	1.45	18.19	21.01	23.41	0.70	1.56	0.08	3.59	4.39	0.44	0.09		Sky	Roads	Tree	Grass
GV_823	3.21	34.79	0.22	16.34	2.50	23.37	1.57	2.03	0.03	0.00	13.39	0.74	1.82		Grass	Sky	Tree	Water
GV_824	2.50	44.60	0.23	46.34	0.01	1.68	0.04	0.00	0.00	0.11	0.00	4.50	0.00		Tree	Grass	Gravel	Background

GV_825	2.64	52.24	3.66	27.30	2.84	2.14	0.01	0.03	0.01	9.04	0.01	0.02	0.07		Grass	Tree	Sidewalk	Building
GV_826	8.18	14.81	1.95	18.18	19.78	27.64	3.43	0.44	0.20	3.71	0.24	0.23	1.19		Sky	Roads	Tree	Grass
GV_827	2.46	42.59	0.61	12.33	12.18	24.59	0.35	0.78	2.09	0.33	0.50	0.54	0.64		Grass	Sky	Tree	Roads
GV_828	2.76	10.04	21.11	19.41	14.23	26.36	0.00	0.01	0.00	5.67	0.39	0.00	0.02		Sky	Building	Tree	Roads
GV_829	1.10	34.44	0.09	32.00	12.04	5.85	6.81	0.00	0.00	0.01	0.06	7.57	0.03		Grass	Tree	Roads	Gravel
GV_830	0.37	57.63	0.77	33.24	2.07	5.14	0.27	0.13	0.00	0.05	0.06	0.27	0.00		Grass	Tree	Sky	Roads
GV_831	1.91	76.43	2.39	7.70	0.26	7.32	0.01	0.30	0.00	0.31	0.95	2.41	0.00		Grass	Tree	Sky	Gravel
GV_832	3.16	43.59	0.39	27.71	3.43	12.84	2.94	0.00	0.44	0.01	3.67	1.26	0.56		Grass	Tree	Sky	Water
GV_833	1.04	24.58	0.03	28.12	6.02	15.23	6.11	0.89	0.64	0.00	15.11	2.11	0.12		Tree	Grass	Sky	Water
GV_834	2.79	43.04	0.13	51.25	0.00	2.42	0.11	0.24	0.00	0.00	0.00	0.02	0.00		Tree	Grass	Background	Sky
GV_835	2.63	64.50	0.64	16.13	1.43	13.57	0.06	0.01	0.00	0.11	0.47	0.03	0.42		Grass	Tree	Sky	Background
GV_836	19.32	8.92	10.92	17.77	0.77	30.83	0.00	0.00	0.00	0.88	7.07	0.00	3.50		Sky	Background	Tree	Building
GV_837	4.06	10.89	0.34	21.82	22.86	7.05	13.20	16.37	0.02	0.06	0.00	3.33	0.00		Roads	Tree	Bare Rock	Soil
GV_838	1.64	26.13	4.34	7.56	11.15	17.52	23.88	0.05	6.50	0.68	0.00	0.55	0.00		Grass	Soil	Sky	Roads
GV_839	0.90	63.94	5.37	7.46	0.03	22.25	0.00	0.00	0.00	0.06	0.00	0.00	0.00		Grass	Sky	Tree	Building
GV_840	1.21	8.50	0.49	20.83	44.66	4.62	8.27	0.32	0.48	0.53	9.66	0.43	0.01		Roads	Tree	Water	Grass
GV_841	1.37	69.86	0.55	17.42	0.02	7.20	0.96	2.18	0.00	0.00	0.21	0.22	0.02		Grass	Tree	Sky	Bare Rock
GV_842	0.26	18.02	0.01	0.14	35.60	11.68	24.67	0.38	0.00	0.00	0.00	9.21	0.03		Roads	Soil	Grass	Sky
GV_843	1.40	20.70	0.62	0.04	47.98	12.24	12.69	0.15	0.00	0.00	1.30	2.13	0.74		Roads	Grass	Soil	Sky
GV_844	0.44	45.03	0.02	1.44	32.26	9.48	5.40	1.34	0.18	0.00	0.00	4.16	0.25		Grass	Roads	Sky	Soil
GV_845	0.34	87.44	0.01	0.00	0.00	11.62	0.23	0.36	0.00	0.00	0.00	0.00	0.00		Grass	Sky	Bare Rock	Background
GV_846	0.16	89.27	0.00	0.60	0.00	8.89	0.22	0.81	0.00	0.00	0.00	0.02	0.01		Grass	Sky	Bare Rock	Tree
GV_847	1.93	15.40	0.20	10.75	25.99	9.39	9.63	2.64	0.03	0.03	5.50	18.43	0.07		Roads	Gravel	Grass	Tree
GV_848	0.69	15.71	0.03	2.39	64.76	4.41	4.49	4.19	0.00	1.99	0.00	1.35	0.00		Roads	Grass	Soil	Sky
GV_849	0.07	51.59	0.07	4.14	24.58	8.82	9.39	0.46	0.00	0.02	0.00	0.86	0.00		Grass	Roads	Soil	Sky
GV_850	0.63	22.49	0.13	1.72	34.94	4.42	12.65	2.46	0.00	0.04	0.00	20.51	0.00		Roads	Grass	Gravel	Soil
GV_851	0.85	22.15	0.29	4.05	0.41	5.37	4.23	23.95	2.72	1.95	31.84	2.16	0.02		Water	Bare Rock	Grass	Sky
GV_852	0.48	4.40	0.01	2.29	1.02	6.69	0.97	45.79	5.07	0.05	32.63	0.59	0.00		Bare Rock	Water	Sky	Sand
GV_853	1.36	25.42	0.33	4.75	35.16	7.05	14.31	3.02	0.00	0.03	0.44	7.80	0.34		Roads	Grass	Soil	Gravel
GV_854	0.74	21.19	0.36	0.01	45.03	13.51	6.10	5.37	0.00	0.18	0.03	7.18	0.29		Roads	Grass	Sky	Gravel
GV_855	1.16	16.12	0.00	0.00	17.78	8.52	38.59	16.46	0.01	0.08	1.01	0.14	0.12		Soil	Roads	Bare Rock	Grass
GV_856	0.23	31.19	0.00	0.00	45.76	9.49	0.00	13.33	0.00	0.00	0.00	0.00	0.00		Roads	Grass	Bare Rock	Sky
GV_857	0.03	57.57	0.19	6.34	5.40	5.18	7.37	15.56	0.00	0.12	0.00	2.20	0.04		Grass	Bare Rock	Soil	Tree
GV_858	0.76	58.69	0.74	0.94	2.50	11.82	18.41	4.05	2.00	0.01	0.01	0.03	0.06		Grass	Soil	Sky	Bare Rock
GV_859	5.25	42.49	0.86	21.41	6.77	5.29	0.33	12.85	0.42	2.52	0.86	0.93	0.00		Grass	Tree	Bare Rock	Roads
GV_860	3.53	17.61	12.97	6.21	26.14	12.41	0.78	8.44	0.00	9.49	0.10	2.27	0.05		Roads	Grass	Building	Sky
GV_861	2.28	2.48	37.24	8.41	35.36	4.52	0.24	0.91	0.00	8.53	0.00	0.03	0.00		Building	Roads	Sidewalk	Tree
GV_862	1.72	9.85	23.71	4.87	42.79	13.13	3.70	0.01	0.04	0.18	0.00	0.00	0.00		Roads	Building	Sky	Grass
GV_863	0.75	43.54	0.87	4.05	33.21	14.35	0.92	0.19	0.00	2.07	0.00	0.02	0.03		Grass	Roads	Sky	Tree
GV_864	0.01	48.82	0.06	0.00	38.34	12.77	0.00	0.00	0.00	0.00	0.00	0.00	0.00		Grass	Roads	Sky	Building
GV_865	0.10	40.02	1.03	0.05	46.50	11.22	0.49	0.04	0.00	0.01	0.00	0.54	0.00		Roads	Grass	Sky	Building
GV_866	2.36	19.32	1.44	18.68	40.98	9.40	5.38	0.00	0.00	0.11	0.00	2.32	0.00		Roads	Grass	Tree	Sky
GV_867	0.01	51.08	0.00	0.07	2.45	11.40	34.48	0.28	0.00	0.00	0.00	0.23	0.00		Grass	Soil	Sky	Roads
GV_868	0.58	26.11	0.00	0.24	3.14	7.63	60.96	1.27	0.01	0.00	0.02	0.05	0.00		Soil	Grass	Sky	Roads
GV_869	0.01	81.83	0.00	0.26	1.63	11.55	4.61	0.07	0.00	0.00	0.00	0.04	0.00		Grass	Sky	Soil	Roads
GV_870	0.30	34.79	0.00	0.30	6.66	8.17	39.92	7.93	0.00	0.00	0.00	1.87	0.05		Soil	Grass	Sky	Bare Rock
GV_871	0.09	67.06	0.00	5.57	12.32	13.76	0.65	0.01	0.04	0.00	0.21	0.20	0.09		Grass	Sky	Roads	Tree
GV_872	12.87	30.80	4.83	3.89	23.74	11.18	3.10	8.05	0.00	0.71	0.00	0.77	0.06		Grass	Roads	Background	Sky
GV_873	0.04	85.42	0.00	2.11	0.24	9.00	0.85	1.63	0.00	0.00	0.00	0.54	0.16		Grass	Sky	Tree	Bare Rock
GV_874	0.69	14.98	0.00	0.02	2.98	15.92	47.56	12.81	0.04	0.00	0.00	4.99	0.01		Soil	Sky	Grass	Bare Rock
GV_875	0.17	43.39	0.00	0.13	22.24	7.51	22.01	0.08	0.00	0.00	0.00	4.39	0.08		Grass	Roads	Soil	Sky
GV_876	0.07	37.18	0.00	2.51	1.48	8.49	48.52	0.01	0.00	0.01	0.02	1.30	0.41		Soil	Grass	Sky	Tree
GV_877	0.03	34.98	0.05	0.00	31.54	10.10	22.34	0.25	0.00	0.03	0.00	0.68	0.00		Grass	Roads	Soil	Sky
GV_878	0.09	77.16	2.82	0.01	0.00	19.92	0.00	0.00	0.00	0.00	0.00	0.00	0.00		Grass	Sky	Building	Background
GV_879	0.04	91.18	0.05	0.01	0.00	8.56	0.07	0.00	0.00	0.00	0.00	0.07	0.02		Grass	Sky	Gravel	Soil
GV_880	0.01	45.42	0.02	0.02	36.33	9.66	5.63	0.04	0.00	0.00	0.00	2.85	0.00		Grass	Roads	Sky	Soil
GV_881	0.12	50.24	0.00	1.11	17.26	10.37	9.25	6.36	0.00	0.00	0.04	5.20	0.05		Grass	Roads	Sky	Soil
GV_882	0.96	36.93	1.25	0.24	18.51	14.25	27.00	0.01	0.00	0.00	0.00	0.67	0.18		Grass	Soil	Roads	Sky
GV_883	0.15	23.63	0.12	0.52	3.72	12.74	56.58	0.10	0.00	0.00	0.00	2.37	0.07		Soil	Grass	Sky	Roads

GV_884	0.21	14.07	0.12	0.15	0.40	8.99	75.80	0.05	0.00	0.00	0.00	0.00	0.21		Soil	Grass	Sky	Roads
GV_885	0.29	23.63	0.33	0.29	1.62	8.08	65.52	0.06	0.00	0.00	0.00	0.00	0.19		Soil	Grass	Sky	Roads
GV_886	0.45	66.02	0.09	6.95	0.39	10.88	13.19	1.68	0.01	0.00	0.00	0.33	0.00		Grass	Soil	Sky	Tree
GV_887	3.71	13.49	0.52	20.28	16.69	12.36	5.55	0.34	0.26	0.02	17.66	9.07	0.05		Tree	Water	Roads	Grass
GV_888	2.17	45.74	1.48	46.93	0.10	3.06	0.00	0.00	0.00	0.02	0.00	0.50	0.00		Tree	Grass	Sky	Background
GV_889	2.44	46.59	0.67	21.38	2.51	26.10	0.00	0.05	0.00	0.05	0.09	0.04	0.08		Grass	Sky	Tree	Roads
GV_890	1.26	70.98	0.88	13.49	0.00	12.73	0.03	0.00	0.00	0.00	0.00	0.00	0.63		Grass	Tree	Sky	Background
GV_891	0.03	82.22	0.00	3.91	0.01	9.98	3.74	0.00	0.00	0.00	0.00	0.11	0.00		Grass	Sky	Tree	Soil
GV_892	0.17	70.84	0.43	26.05	0.00	2.52	0.00	0.00	0.00	0.00	0.00	0.00	0.00		Grass	Tree	Sky	Building
GV_893	0.49	39.85	0.39	13.57	17.80	18.19	0.70	0.00	0.00	0.00	8.93	0.00	0.07		Grass	Sky	Roads	Tree
GV_894	2.06	43.86	1.24	22.85	3.76	9.72	0.59	0.00	0.16	0.09	15.64	0.00	0.02		Grass	Tree	Water	Sky
GV_895	1.12	22.39	18.87	6.23	7.41	14.13	24.51	0.28	0.00	2.15	0.00	2.92	0.00		Soil	Grass	Building	Sky
GV_896	44.98	29.81	2.32	7.07	0.17	15.63	0.00	0.00	0.00	0.01	0.00	0.00	0.00		Background	Grass	Sky	Tree
GV_897	0.50	51.15	0.00	0.80	1.17	2.65	8.76	5.97	0.38	0.00	16.91	11.71	0.00		Grass	Water	Gravel	Soil
GV_898	0.09	33.52	0.21	9.59	34.14	16.92	3.32	1.95	0.00	0.07	0.00	0.19	0.00		Roads	Grass	Sky	Tree
GV_899	21.15	17.85	10.11	21.07	0.82	25.34	2.65	0.00	0.00	0.35	0.00	0.66	0.00		Sky	Background	Tree	Grass
GV_900	1.91	85.33	0.08	9.19	0.10	0.27	0.07	0.00	0.00	0.00	2.63	0.01	0.39		Grass	Tree	Water	Background
GV_901	1.95	87.46	0.20	1.24	3.76	1.67	3.58	0.02	0.00	0.06	0.03	0.02	0.01		Grass	Roads	Soil	Background
GV_902	3.55	27.75	0.01	0.24	14.37	0.49	50.10	0.61	0.28	1.42	0.00	1.17	0.00		Soil	Grass	Roads	Background
GV_903	1.54	13.96	0.14	2.31	37.40	0.19	25.06	0.17	0.21	10.92	0.21	7.87	0.00		Roads	Soil	Grass	Sidewalk
GV_904	3.10	32.55	0.15	4.97	6.43	0.99	44.16	5.89	0.34	0.03	0.00	1.39	0.00		Soil	Grass	Roads	Bare Rock
GV_905	2.38	62.17	0.03	1.52	2.97	0.00	29.54	0.22	0.71	0.14	0.00	0.33	0.00		Grass	Soil	Roads	Background
GV_906	3.52	63.47	3.51	19.39	0.07	8.32	0.22	0.81	0.44	0.04	0.01	0.21	0.00		Grass	Tree	Sky	Background
GV_907	8.86	23.57	19.91	8.74	9.92	14.61	0.27	0.51	0.27	13.11	0.07	0.02	0.12		Grass	Building	Sky	Sidewalk
GV_908	1.04	51.29	0.00	6.99	0.00	19.87	12.32	7.58	0.38	0.00	0.00	0.05	0.47		Grass	Sky	Soil	Bare Rock
GV_909	0.13	63.70	0.32	23.97	1.20	0.00	0.09	0.00	0.00	10.54	0.00	0.07	0.00		Grass	Tree	Sidewalk	Roads
GV_910	0.09	81.23	0.11	12.76	0.06	5.69	0.00	0.00	0.00	0.00	0.00	0.00	0.06		Grass	Tree	Sky	Building
GV_911	1.62	54.54	0.10	17.57	2.73	0.07	11.29	1.18	0.00	0.00	0.14	10.61	0.14		Grass	Tree	Soil	Gravel
GV_912	2.26	34.83	3.04	29.56	10.08	0.33	6.07	0.05	0.00	0.18	0.27	13.33	0.00		Grass	Tree	Gravel	Roads
GV_913	0.05	58.76	0.02	28.74	3.45	3.13	0.89	0.11	0.00	2.42	0.00	2.42	0.00		Grass	Tree	Roads	Sky
GV_914	1.27	37.17	0.15	46.18	0.27	0.18	10.08	0.01	0.00	0.09	0.02	4.58	0.00		Tree	Grass	Soil	Gravel
GV_915	0.51	55.86	0.00	38.62	0.95	0.06	3.96	0.02	0.00	0.00	0.00	0.01	0.00		Grass	Tree	Soil	Roads
GV_916	4.60	46.55	0.00	43.77	0.00	0.05	2.79	0.43	0.00	0.00	0.05	1.77	0.00		Grass	Tree	Background	Soil
GV_917	0.04	10.43	0.00	81.61	0.00	0.02	6.22	0.27	0.00	0.00	0.00	1.41	0.00		Tree	Grass	Soil	Gravel
GV_918	0.35	31.95	0.11	50.51	0.01	0.22	10.87	0.00	0.00	0.00	0.28	5.70	0.00		Tree	Grass	Soil	Gravel
GV_919	0.30	52.71	0.04	31.12	0.01	0.01	0.73	5.86	0.00	0.00	0.01	9.22	0.00		Grass	Tree	Gravel	Bare Rock
GV_920	0.03	18.60	0.00	53.61	4.46	2.16	1.42	0.00	0.00	1.80	0.00	17.91	0.00		Tree	Grass	Gravel	Roads
GV_921	0.43	40.09	0.05	49.26	1.10	1.11	0.62	0.00	0.48	0.10	5.21	1.54	0.00		Tree	Grass	Water	Gravel
GV_922	0.10	31.34	0.01	68.40	0.00	0.00	0.14	0.00	0.00	0.00	0.00	0.01	0.00		Tree	Grass	Soil	Background
GV_923	2.52	52.62	2.40	32.58	0.05	1.00	1.11	1.24	0.00	0.00	0.00	6.47	0.00		Grass	Tree	Gravel	Background
GV_924	0.18	53.05	0.06	26.47	0.84	9.49	7.53	0.19	0.00	0.08	0.00	2.11	0.00		Grass	Tree	Sky	Soil
GV_925	0.14	46.69	0.00	52.33	0.00	0.03	0.08	0.00	0.00	0.00	0.00	0.72	0.00		Tree	Grass	Gravel	Background
GV_926	0.01	42.32	0.01	57.09	0.00	0.12	0.22	0.00	0.00	0.00	0.00	0.23	0.00		Tree	Grass	Gravel	Soil
GV_927	2.22	40.44	0.01	52.04	0.00	0.00	0.78	0.15	0.00	0.00	0.00	4.35	0.00		Tree	Grass	Gravel	Background
GV_928	2.04	43.41	0.01	42.32	0.00	0.00	0.13	0.09	0.00	0.00	11.95	0.04	0.00		Grass	Tree	Water	Background
GV_929	0.02	22.46	0.00	66.96	0.00	0.04	7.22	0.00	0.00	0.00	0.00	3.31	0.00		Tree	Grass	Soil	Gravel
GV_930	0.12	13.06	0.94	80.65	0.00	0.01	5.21	0.00	0.00	0.00	0.00	0.01	0.00		Tree	Grass	Soil	Building
GV_931	0.90	19.75	0.00	71.24	0.38	0.55	0.56	0.02	0.00	0.00	0.01	6.58	0.00		Tree	Grass	Gravel	Background
GV_932	0.12	60.51	0.02	39.24	0.00	0.11	0.00	0.00	0.00	0.00	0.00	0.00	0.00		Grass	Tree	Background	Sky
GV_933	1.29	49.81	0.01	48.37	0.01	0.23	0.12	0.00	0.00	0.00	0.01	0.16	0.00		Grass	Tree	Background	Sky
GV_934	1.30	8.39	0.04	59.15	4.77	1.08	10.96	0.28	0.42	8.70	0.07	4.84	0.00		Tree	Soil	Sidewalk	Grass
GV_935	0.13	28.09	0.01	50.77	0.00	0.05	18.45	0.61	0.00	0.00	0.00	1.89	0.00		Tree	Grass	Soil	Gravel
GV_936	1.07	25.96	0.00	69.32	0.01	0.19	0.31	0.35	0.00	0.00	0.00	2.80	0.00		Tree	Grass	Gravel	Background
GV_937	0.09	27.16	0.00	57.05	0.02	0.11	8.80	0.00	0.00	0.00	0.01	6.76	0.00		Tree	Grass	Soil	Gravel
GV_938	0.19	16.13	0.00	78.49	0.00	0.20	4.18	0.01	0.00	0.00	0.00	0.82	0.00		Tree	Grass	Soil	Gravel
GV_939	0.32	38.10	0.00	60.57	0.00	0.00	0.54	0.00	0.00	0.00	0.00	0.47	0.00		Tree	Grass	Soil	Gravel
GV_940	1.68	25.74	0.06	56.77	1.41	2.41	1.96	0.03	0.01	2.76	0.00	7.17	0.00		Tree	Grass	Gravel	Sidewalk
GV_941	0.63	50.31	0.00	19.06	0.08	3.38	15.06	3.87	0.00	0.00	0.00	6.28	1.34		Grass	Tree	Soil	Gravel
GV_942	0.30	33.22	0.00	46.73	5.21	0.54	3.73	0.00	0.00	0.00	0.00	10.26	0.00		Tree	Grass	Gravel	Roads

GV_943	0.05	36.12	0.00	32.32	0.00	0.00	31.20	0.30	0.00	0.00	0.00	0.00	0.00		Grass	Tree	Soil	Bare Rock
GV_944	0.84	27.67	0.00	61.49	0.14	0.10	7.36	0.27	0.00	0.00	0.00	2.13	0.00		Tree	Grass	Soil	Gravel
GV_945	1.98	62.67	0.00	14.98	0.00	0.00	18.89	1.02	0.00	0.00	0.00	0.46	0.00		Grass	Soil	Tree	Background
GV_946	0.43	74.80	0.00	22.39	0.00	0.97	1.06	0.03	0.00	0.00	0.00	0.30	0.00		Grass	Tree	Soil	Sky
GV_947	0.31	21.26	0.03	77.70	0.00	0.07	0.22	0.02	0.00	0.00	0.00	0.38	0.00		Tree	Grass	Gravel	Background
GV_948	1.55	44.22	0.05	45.47	0.02	0.50	6.15	0.10	0.00	0.02	0.00	1.92	0.00		Tree	Grass	Soil	Gravel
GV_949	0.58	22.28	0.00	73.58	0.00	0.22	1.27	0.02	0.00	0.00	0.00	2.05	0.00		Tree	Grass	Gravel	Soil
GV_950	0.48	19.92	0.00	29.15	0.00	0.89	48.64	0.92	0.00	0.00	0.00	0.00	0.00		Soil	Tree	Grass	Bare Rock
GV_951	0.25	29.55	0.00	66.98	0.00	0.18	1.37	0.00	0.00	0.00	0.00	1.66	0.00		Tree	Grass	Gravel	Soil
GV_952	2.76	73.88	0.00	21.36	0.00	0.00	1.99	0.00	0.00	0.00	0.00	0.00	0.00		Grass	Tree	Background	Soil
GV_953	0.39	35.01	0.05	37.16	10.10	0.45	5.36	0.03	0.00	0.12	0.03	11.29	0.01		Tree	Grass	Gravel	Roads
GV_954	0.74	30.18	0.03	54.66	0.91	1.16	6.07	0.02	0.00	2.04	0.00	4.19	0.00		Tree	Grass	Soil	Gravel
GV_955	0.09	25.02	0.05	39.72	5.05	1.52	5.96	0.05	6.20	14.29	0.00	1.88	0.17		Tree	Grass	Sidewalk	Sand
GV_956	1.39	80.44	0.93	14.22	1.35	0.03	0.45	0.02	0.00	0.70	0.00	0.47	0.00		Grass	Tree	Background	Roads
GV_957	0.82	54.59	0.06	38.96	0.11	0.65	2.58	0.36	0.00	0.13	0.01	1.74	0.00		Grass	Tree	Soil	Gravel
GV_958	0.90	75.60	0.02	14.99	0.02	0.00	4.12	0.00	0.00	0.00	2.58	1.77	0.00		Grass	Tree	Soil	Water
GV_959	2.88	44.91	0.08	27.59	0.60	3.10	11.53	0.51	0.01	0.34	4.17	4.24	0.04		Grass	Tree	Soil	Gravel
GV_960	0.83	39.79	0.18	23.94	4.50	2.41	16.12	0.97	0.01	0.00	8.70	2.45	0.08		Grass	Tree	Soil	Water
GV_961	0.09	38.92	0.01	32.01	16.03	0.27	8.11	0.00	1.02	2.80	0.00	0.73	0.00		Grass	Tree	Roads	Soil
GV_962	1.85	84.94	0.77	11.78	0.22	0.10	0.01	0.00	0.00	0.33	0.00	0.00	0.00		Grass	Tree	Background	Building
GV_963	0.35	80.53	0.07	0.11	0.44	16.26	0.82	0.10	0.00	0.01	0.05	0.00	1.24		Grass	Sky	Blue Mountain	Soil
GV_964	4.39	4.65	0.68	74.41	10.88	1.06	0.25	0.07	0.01	0.10	0.00	3.50	0.00		Tree	Roads	Grass	Background
GV_965	3.99	11.65	0.00	38.66	0.02	9.26	3.76	23.28	0.35	0.54	4.41	4.08	0.01		Tree	Bare Rock	Grass	Sky
GV_966	2.69	47.64	0.61	16.01	23.72	4.32	0.47	1.30	0.00	2.91	0.10	0.24	0.00		Grass	Roads	Tree	Sky
GV_967	4.21	56.46	3.03	11.52	0.77	12.61	4.29	2.71	0.00	4.09	0.00	0.01	0.31		Grass	Sky	Tree	Soil
GV_968	1.42	70.00	0.56	4.06	2.17	12.90	2.00	1.20	0.00	0.16	5.30	0.11	0.13		Grass	Sky	Water	Tree
GV_969	1.37	72.61	0.05	1.17	11.68	10.00	1.35	1.37	0.00	0.29	0.06	0.02	0.03		Grass	Roads	Sky	Bare Rock
GV_970	1.51	68.70	0.03	1.79	1.97	4.86	15.40	3.74	0.00	0.00	0.53	0.09	1.38		Grass	Soil	Sky	Bare Rock
GV_971	2.18	63.80	0.00	0.07	0.04	5.24	25.97	0.47	0.00	0.10	0.10	0.01	2.02		Grass	Soil	Sky	Background
GV_972	5.37	32.16	8.73	25.09	5.55	4.56	0.56	0.03	0.00	17.89	0.00	0.06	0.00		Grass	Tree	Sidewalk	Building
GV_973	4.95	24.92	0.24	13.74	48.49	1.30	0.91	0.40	0.59	4.01	0.22	0.22	0.00		Roads	Grass	Tree	Background
GV_974	7.32	47.53	13.99	8.76	0.55	21.59	0.12	0.00	0.11	0.01	0.00	0.02	0.00		Grass	Sky	Building	Tree
GV_975	0.81	60.72	0.21	24.70	1.82	10.86	0.02	0.00	0.00	0.86	0.01	0.01	0.00		Grass	Tree	Sky	Roads
GV_976	2.18	13.22	0.64	15.13	10.35	39.00	0.19	4.10	0.01	3.34	11.47	0.30	0.05		Sky	Tree	Grass	Water
GV_977	0.15	48.44	0.01	17.39	0.01	28.23	5.69	0.00	0.00	0.00	0.00	0.07	0.03		Grass	Sky	Tree	Soil
GV_978	1.98	55.56	1.44	26.21	1.46	12.33	0.84	0.00	0.00	0.05	0.02	0.00	0.13		Grass	Tree	Sky	Background
GV_979	15.54	35.86	29.42	3.34	0.06	14.77	0.10	0.00	0.00	0.73	0.00	0.17	0.00		Grass	Building	Background	Sky
GV_980	0.64	76.56	7.20	9.71	0.00	5.60	0.04	0.00	0.00	0.25	0.00	0.00	0.00		Grass	Tree	Building	Sky
GV_981	1.46	7.25	0.08	6.07	30.40	20.44	8.17	12.22	11.32	0.00	1.02	1.57	0.01		Roads	Sky	Bare Rock	Sand
GV_982	2.51	31.33	41.61	9.91	1.90	11.89	0.18	0.00	0.00	0.59	0.00	0.07	0.00		Building	Grass	Sky	Tree
GV_983	2.21	22.88	0.01	22.62	8.54	11.03	3.09	0.07	22.88	0.02	0.14	6.49	0.02		Grass	Sand	Tree	Sky
GV_984	2.63	5.71	1.29	6.34	5.66	14.94	38.10	11.78	7.35	1.36	0.24	4.45	0.14		Soil	Sky	Bare Rock	Sand
GV_985	1.40	39.47	0.17	39.54	0.04	12.00	4.45	0.22	0.70	0.82	0.01	1.19	0.00		Tree	Grass	Sky	Soil
GV_986	8.79	4.85	18.71	15.25	0.84	30.25	2.54	14.86	0.00	3.84	0.00	0.06	0.00		Sky	Building	Tree	Bare Rock
GV_987	2.32	23.91	0.12	38.46	28.34	6.45	0.27	0.04	0.00	0.01	0.00	0.07	0.00		Tree	Roads	Grass	Sky
GV_988	1.91	16.42	4.31	23.60	32.37	5.43	0.32	0.00	0.00	15.41	0.00	0.24	0.00		Roads	Tree	Grass	Sidewalk
GV_989	1.56	30.01	5.53	24.96	20.06	9.26	0.05	0.99	0.00	7.58	0.00	0.00	0.00		Grass	Tree	Roads	Sky
GV_990	1.01	39.39	0.23	13.53	20.15	21.06	0.06	0.60	0.63	0.23	3.03	0.08	0.00		Grass	Sky	Roads	Tree
GV_991	1.61	30.54	0.14	30.57	30.67	3.66	1.59	0.39	0.00	0.51	0.01	0.30	0.00		Roads	Tree	Grass	Sky
GV_992	0.20	47.76	0.13	4.51	7.91	23.40	4.81	0.22	0.21	0.01	0.00	10.83	0.00		Grass	Sky	Gravel	Roads
GV_993	2.37	11.03	0.66	23.53	0.45	20.56	0.07	0.24	0.00	39.29	0.00	0.04	1.77		Sidewalk	Tree	Sky	Grass
GV_994	3.42	11.40	9.36	64.39	0.10	2.24	0.12	1.28	0.00	7.69	0.00	0.00	0.00		Tree	Grass	Building	Sidewalk
GV_995	1.32	67.00	0.00	3.86	0.57	0.89	7.47	6.67	1.67	0.00	4.04	6.52	0.00		Grass	Soil	Bare Rock	Gravel
GV_996	0.14	47.71	0.00	31.45	6.57	0.43	4.94	0.08	0.00	0.00	0.01	8.67	0.00		Grass	Tree	Gravel	Roads
GV_997	0.35	36.69	0.33	8.97	38.01	13.57	0.17	0.00	0.00	0.00	0.00	0.46	1.44		Roads	Grass	Sky	Tree
GV_998	0.62	33.44	0.59	10.33	41.50	9.90	0.26	0.36	0.00	0.00	0.00	2.26	0.74		Roads	Grass	Tree	Sky
GV_999	0.28	36.41	0.00	35.44	0.64	0.06	11.04	0.00	0.43	4.58	0.00	11.10	0.00		Grass	Tree	Gravel	Soil
GV_1000	0.13	43.91	0.00	38.16	3.80	0.61	0.93	0.00	5.30	0.65	0.00	6.52	0.00		Grass	Tree	Gravel	Sand
GV_1001	0.49	39.24	0.00	43.63	1.06	0.27	11.50	0.37	0.21	0.00	0.01	3.23	0.00		Tree	Grass	Soil	Gravel

GV_1002	0.08	56.23	0.00	24.24	0.16	0.00	13.60	0.03	0.00	0.00	0.00	5.66	0.00		Grass	Tree	Soil	Gravel
GV_1003	0.00	58.07	0.00	19.30	11.55	0.25	6.16	0.00	0.00	0.84	0.00	3.82	0.00		Grass	Tree	Roads	Soil
GV_1004	0.02	38.79	0.00	47.38	0.00	0.18	10.71	0.04	1.04	0.00	0.26	1.57	0.00		Tree	Grass	Soil	Gravel
GV_1005	0.04	49.24	0.00	34.88	0.09	0.59	13.46	0.00	0.01	0.20	0.00	1.50	0.00		Grass	Tree	Soil	Gravel
GV_1006	0.19	18.99	0.00	35.07	0.02	1.43	40.50	0.19	0.00	0.00	0.00	3.61	0.00		Soil	Tree	Grass	Gravel
GV_1007	0.10	38.96	0.00	34.96	2.38	0.88	9.50	0.07	0.00	3.89	0.00	9.27	0.00		Grass	Tree	Soil	Gravel
GV_1008	0.03	57.05	0.00	34.61	1.10	0.47	0.52	0.00	0.00	0.57	0.02	5.63	0.00		Grass	Tree	Gravel	Roads
GV_1009	0.59	36.97	0.00	60.22	0.02	0.02	0.72	0.10	0.00	0.00	0.01	1.37	0.00		Tree	Grass	Gravel	Soil
GV_1010	0.18	21.35	0.00	60.10	0.56	0.01	16.17	0.06	0.00	0.00	0.01	1.56	0.00		Tree	Grass	Soil	Gravel
GV_1011	0.00	14.88	0.00	63.08	3.90	2.63	6.92	0.00	0.00	0.02	0.00	8.58	0.00		Tree	Grass	Gravel	Soil
GV_1012	1.23	16.34	0.67	67.19	7.40	0.27	1.51	0.20	0.04	2.53	0.12	2.49	0.00		Tree	Grass	Roads	Sidewalk
GV_1013	0.71	10.31	0.00	71.56	0.03	0.09	2.91	7.56	0.00	0.00	0.00	6.83	0.00		Tree	Grass	Bare Rock	Gravel
GV_1014	0.84	11.22	0.00	55.65	0.08	0.00	26.99	0.33	0.00	0.00	0.00	4.89	0.00		Tree	Soil	Grass	Gravel
GV_1015	0.29	33.94	0.00	54.00	0.02	0.01	10.40	0.07	0.00	0.00	0.00	1.28	0.00		Tree	Grass	Soil	Gravel
GV_1016	0.61	9.28	0.00	49.82	5.43	0.05	26.45	0.26	0.00	2.24	0.00	5.85	0.00		Tree	Soil	Grass	Gravel
GV_1017	0.56	23.81	0.00	59.84	0.31	0.01	14.00	1.03	0.08	0.00	0.01	0.34	0.00		Tree	Grass	Soil	Bare Rock
GV_1018	0.13	33.60	0.00	62.46	0.00	0.04	2.99	0.01	0.00	0.00	0.00	0.76	0.00		Tree	Grass	Soil	Gravel
GV_1019	0.38	12.11	0.00	47.39	0.18	0.04	27.06	10.62	0.00	0.07	0.00	2.14	0.00		Tree	Soil	Grass	Bare Rock
GV_1020	0.33	37.70	0.00	54.64	0.01	0.87	6.05	0.01	0.00	0.01	0.00	0.38	0.00		Tree	Grass	Soil	Sky
GV_1021	1.50	36.99	0.00	41.09	0.00	0.00	17.62	0.34	0.00	0.00	0.00	2.45	0.00		Tree	Grass	Soil	Gravel
GV_1022	0.30	6.98	0.00	80.54	1.19	0.01	10.43	0.13	0.00	0.00	0.00	0.41	0.00		Tree	Soil	Grass	Roads
GV_1023	0.05	49.20	0.00	48.31	0.31	0.57	1.38	0.00	0.00	0.00	0.00	0.18	0.00		Grass	Tree	Soil	Sky
GV_1024	0.32	21.88	0.00	69.11	0.00	0.01	7.82	0.04	0.00	0.00	0.00	0.83	0.00		Tree	Grass	Soil	Gravel
GV_1025	0.63	19.72	0.01	50.12	0.68	0.22	26.95	0.06	0.00	0.00	0.00	1.55	0.06		Tree	Soil	Grass	Gravel
GV_1026	0.27	4.49	0.00	63.27	11.43	0.32	15.98	0.23	0.00	0.00	0.00	4.01	0.00		Tree	Soil	Roads	Grass
GV_1027	0.01	37.62	0.00	39.86	12.41	0.65	6.76	0.00	0.00	0.00	0.00	2.68	0.01		Tree	Grass	Roads	Soil
GV_1028	0.11	11.55	0.00	55.70	14.10	0.00	11.25	0.20	0.00	0.00	0.00	7.08	0.00		Tree	Roads	Grass	Soil
GV_1029	0.31	25.48	0.05	67.46	0.00	0.00	3.57	3.02	0.00	0.00	0.00	0.11	0.00		Tree	Grass	Soil	Bare Rock
GV_1030	0.05	28.63	0.04	41.30	1.32	3.67	14.19	0.00	0.01	3.32	0.38	6.88	0.21		Tree	Grass	Soil	Gravel
GV_1031	0.00	18.88	0.09	48.64	16.84	13.89	0.00	0.00	0.00	0.01	0.00	0.65	0.98		Tree	Grass	Roads	Sky
GV_1032	0.39	25.61	0.01	46.06	11.58	0.77	3.63	0.00	0.00	4.81	0.46	6.69	0.00		Tree	Grass	Roads	Gravel
GV_1033	1.58	10.16	0.06	73.38	0.33	0.12	10.12	0.00	0.00	0.00	0.09	4.17	0.00		Tree	Grass	Soil	Gravel
GV_1034	0.96	12.93	1.38	67.06	1.09	1.15	11.87	0.04	0.02	2.81	0.00	0.68	0.00		Tree	Grass	Soil	Sidewalk
GV_1035	0.01	4.22	0.00	86.36	0.00	0.00	8.82	0.41	0.00	0.00	0.00	0.17	0.00		Tree	Soil	Grass	Bare Rock
GV_1036	0.20	17.80	0.00	75.89	0.00	0.06	5.67	0.00	0.00	0.00	0.00	0.37	0.00		Tree	Grass	Soil	Gravel
GV_1037	0.08	15.39	0.04	75.01	0.07	0.93	4.28	0.00	0.00	0.00	0.00	4.21	0.00		Tree	Grass	Soil	Gravel
GV_1038	0.25	28.72	0.00	50.65	13.69	0.92	0.35	0.00	0.06	1.59	0.00	3.76	0.01		Tree	Grass	Roads	Gravel
GV_1039	1.57	8.40	0.00	85.86	0.06	0.00	3.78	0.25	0.00	0.00	0.00	0.08	0.00		Tree	Grass	Soil	Background
GV_1040	0.00	15.18	0.00	59.97	0.01	1.08	7.69	0.00	0.00	0.00	0.00	16.07	0.00		Tree	Gravel	Grass	Soil
GV_1041	0.07	36.78	0.00	56.79	0.51	3.36	0.12	0.02	0.00	0.08	0.00	2.28	0.00		Tree	Grass	Sky	Gravel
GV_1042	0.02	27.94	0.00	53.94	2.19	3.03	0.13	0.11	0.43	0.00	0.00	12.21	0.00		Tree	Grass	Gravel	Sky
GV_1043	1.59	11.54	0.16	55.75	8.59	0.47	0.95	2.66	0.00	0.01	0.14	18.13	0.00		Tree	Gravel	Grass	Roads
GV_1044	0.08	22.66	0.00	73.56	0.01	0.19	3.40	0.00	0.00	0.00	0.00	0.10	0.00		Tree	Grass	Soil	Sky
GV_1045	0.17	9.42	0.00	79.63	0.00	0.15	7.11	0.00	0.00	0.00	0.00	3.52	0.00		Tree	Grass	Soil	Gravel
GV_1046	0.11	23.93	0.00	52.48	0.13	0.92	10.01	0.00	0.00	0.00	0.00	12.41	0.00		Tree	Grass	Gravel	Soil
GV_1047	0.14	11.55	0.00	79.72	0.16	0.13	3.44	0.00	0.00	0.00	0.00	4.87	0.00		Tree	Grass	Gravel	Soil
GV_1048	0.26	22.32	0.00	50.19	3.48	7.56	11.55	0.00	0.17	0.63	0.01	3.82	0.01		Tree	Grass	Soil	Sky
GV_1049	0.02	27.62	0.22	56.06	0.22	2.73	9.64	0.00	0.01	0.00	0.00	3.46	0.00		Tree	Grass	Soil	Gravel
GV_1050	0.27	8.09	0.00	84.95	0.00	0.02	6.14	0.08	0.00	0.00	0.00	0.46	0.00		Tree	Grass	Soil	Gravel
GV_1051	0.25	21.38	0.21	53.58	6.38	0.76	14.01	0.01	0.15	0.05	0.00	3.21	0.00		Tree	Grass	Soil	Roads
GV_1052	0.34	18.12	0.03	76.24	0.02	1.94	1.08	0.05	0.00	0.00	0.00	2.19	0.00		Tree	Grass	Gravel	Sky
GV_1053	0.54	23.27	0.02	72.22	0.24	0.19	0.22	2.27	0.00	0.60	0.00	0.43	0.00		Tree	Grass	Bare Rock	Sidewalk
GV_1054	0.06	41.75	0.03	45.98	0.65	0.56	9.66	0.06	0.00	0.00	0.00	1.24	0.00		Tree	Grass	Soil	Gravel
GV_1055	0.17	40.44	0.21	52.85	0.05	0.94	3.99	0.02	0.00	0.00	0.00	1.34	0.00		Tree	Grass	Soil	Gravel
GV_1056	0.54	13.31	0.00	84.27	0.00	0.00	1.83	0.05	0.00	0.00	0.00	0.00	0.00		Tree	Grass	Soil	Background
GV_1057	6.03	17.63	0.01	54.19	2.42	1.61	10.55	1.97	0.00	0.00	0.23	5.32	0.03		Tree	Grass	Soil	Background
GV_1058	1.19	7.93	0.99	82.98	0.01	0.31	6.19	0.02	0.00	0.00	0.00	0.38	0.00		Tree	Grass	Soil	Background
GV_1059	0.17	16.28	0.01	77.07	1.21	1.21	3.49	0.00	0.00	0.00	0.00	0.56	0.00		Tree	Grass	Soil	Roads
GV_1060	0.48	8.09	0.06	70.69	0.00	0.03	19.86	0.20	0.00	0.00	0.00	0.59	0.00		Tree	Soil	Grass	Gravel

GV_1061	0.05	11.04	0.01	81.61	0.01	0.17	7.11	0.00	0.00	0.00	0.00	0.01	0.00		Tree	Grass	Soil	Sky
GV_1062	0.48	24.64	0.08	67.70	0.31	3.77	1.49	0.08	0.01	0.00	0.03	1.40	0.00		Tree	Grass	Sky	Soil
GV_1063	0.05	19.43	0.05	41.76	16.57	1.71	0.11	0.00	0.00	0.00	0.01	20.30	0.00		Tree	Gravel	Grass	Roads
GV_1064	1.94	18.75	0.03	34.27	8.88	0.76	12.36	0.46	1.51	0.42	0.00	20.62	0.00		Tree	Gravel	Grass	Soil
GV_1065	0.88	24.24	0.65	44.72	10.88	0.83	1.08	0.05	0.01	3.38	0.00	13.19	0.10		Tree	Grass	Gravel	Roads
GV_1066	9.78	23.70	1.17	40.93	2.64	4.38	9.46	6.23	0.11	0.21	0.14	1.24	0.00		Tree	Grass	Background	Soil
GV_1067	0.14	17.53	0.14	54.03	7.85	1.67	0.00	0.00	0.00	0.00	0.00	18.64	0.00		Tree	Gravel	Grass	Roads
GV_1068	0.72	18.99	0.14	48.49	15.18	3.11	1.26	0.01	0.11	0.00	0.02	11.98	0.00		Tree	Grass	Roads	Gravel
GV_1069	1.30	23.43	1.92	46.97	12.02	1.59	3.13	0.15	0.01	0.01	0.07	9.37	0.02		Tree	Grass	Roads	Gravel
GV_1070	0.20	18.48	0.75	58.31	0.91	0.12	14.56	0.02	0.00	1.76	0.00	4.89	0.00		Tree	Grass	Soil	Gravel
GV_1071	2.09	32.94	0.01	45.27	0.07	1.55	15.23	1.64	0.00	0.03	0.00	1.18	0.00		Tree	Grass	Soil	Background
GV_1072	0.09	6.84	0.16	79.36	7.82	0.01	5.72	0.00	0.00	0.00	0.00	0.00	0.00		Tree	Roads	Grass	Soil
GV_1073	0.34	9.72	0.00	69.01	9.86	0.00	10.49	0.00	0.00	0.00	0.00	0.56	0.00		Tree	Soil	Roads	Grass
GV_1074	0.81	5.11	0.03	81.60	0.03	0.05	11.41	0.44	0.00	0.00	0.00	0.54	0.00		Tree	Soil	Grass	Background
GV_1075	1.53	18.41	0.00	66.20	0.39	1.00	10.72	0.49	0.00	0.00	0.07	1.21	0.00		Tree	Grass	Soil	Background
GV_1076	1.35	57.00	0.06	26.74	8.62	2.20	2.70	0.01	0.12	0.00	0.20	1.02	0.00		Grass	Tree	Roads	Soil
GV_1077	0.23	22.29	0.01	69.51	0.04	0.64	4.21	0.28	0.00	0.06	0.00	2.74	0.00		Tree	Grass	Soil	Gravel
GV_1078	0.20	52.49	0.02	32.00	0.21	4.14	4.28	0.39	0.00	0.00	0.00	6.28	0.00		Grass	Tree	Gravel	Soil
GV_1079	0.94	54.62	0.03	29.07	0.04	1.23	9.63	0.01	0.00	0.00	0.00	4.44	0.00		Grass	Tree	Soil	Gravel
GV_1080	2.92	37.06	0.00	26.06	5.69	0.75	7.98	11.19	0.00	0.00	0.70	7.65	0.00		Grass	Tree	Bare Rock	Soil
GV_1081	0.55	23.19	0.01	46.43	0.18	7.79	12.13	0.48	0.00	0.00	0.40	8.82	0.00		Tree	Grass	Soil	Gravel
GV_1082	0.11	50.73	0.16	38.63	4.95	2.66	0.46	0.01	0.00	0.00	1.20	1.09	0.00		Grass	Tree	Roads	Sky
GV_1083	0.15	32.30	0.38	45.84	1.76	11.98	4.31	0.02	0.00	0.00	0.00	3.26	0.00		Tree	Grass	Sky	Soil
GV_1084	0.04	55.25	0.34	27.73	7.22	8.00	0.60	0.28	0.00	0.00	0.00	0.55	0.00		Grass	Tree	Sky	Roads
GV_1085	1.05	29.79	0.11	49.18	9.25	6.61	2.90	0.05	0.00	0.02	0.09	0.75	0.19		Tree	Grass	Roads	Sky
GV_1086	0.94	61.49	3.08	17.75	5.57	10.41	0.24	0.10	0.00	0.04	0.29	0.09	0.00		Grass	Tree	Sky	Roads
GV_1087	0.16	62.05	1.45	18.42	4.24	12.68	0.04	0.08	0.00	0.00	0.00	0.87	0.00		Grass	Tree	Sky	Roads
GV_1088	0.08	60.18	0.12	26.53	5.07	4.15	0.96	0.58	0.00	0.00	0.01	2.33	0.00		Grass	Tree	Roads	Sky
GV_1089	0.83	37.12	0.02	25.57	3.22	3.83	16.34	1.65	0.00	0.00	0.03	11.40	0.00		Grass	Tree	Soil	Gravel
GV_1090	0.36	47.46	0.04	37.79	7.72	4.32	0.20	1.07	0.00	0.00	0.00	1.04	0.00		Grass	Tree	Roads	Sky
GV_1091	1.28	45.03	0.04	28.41	1.70	9.68	11.79	0.14	0.00	0.00	0.01	1.91	0.00		Grass	Tree	Soil	Sky
GV_1092	1.11	44.90	0.09	28.29	4.70	11.35	6.68	1.39	0.00	0.00	0.00	1.43	0.05		Grass	Tree	Sky	Soil
GV_1093	10.16	5.01	0.02	11.75	23.17	7.20	29.92	8.38	0.00	0.00	0.00	4.39	0.00		Soil	Roads	Tree	Background
GV_1094	0.59	6.35	0.25	13.68	7.87	9.74	57.90	2.60	0.00	0.00	0.05	0.98	0.00		Soil	Tree	Sky	Roads
GV_1095	0.42	5.60	0.10	13.53	13.84	8.75	52.96	1.49	0.00	0.00	0.06	3.23	0.02		Soil	Roads	Tree	Sky
GV_1096	2.84	10.25	0.86	11.33	16.24	13.85	17.55	20.79	0.17	0.23	0.55	5.33	0.01		Bare Rock	Soil	Roads	Sky
GV_1097	0.53	5.10	0.32	16.77	9.33	21.39	9.62	29.15	0.00	1.04	0.02	6.40	0.32		Bare Rock	Sky	Tree	Soil
GV_1098	0.06	33.47	0.05	31.56	16.07	5.59	1.12	0.00	0.00	0.00	0.00	12.10	0.00		Grass	Tree	Roads	Gravel
GV_1099	0.97	11.78	0.04	33.95	7.35	7.73	14.41	20.49	0.00	0.00	0.21	3.07	0.00		Tree	Bare Rock	Soil	Grass
GV_1100	0.88	16.37	0.45	29.44	6.18	7.27	17.81	13.27	0.00	0.46	0.22	7.65	0.00		Tree	Soil	Grass	Bare Rock
GV_1101	1.26	39.01	0.20	9.19	4.48	18.97	20.57	4.48	0.08	0.07	0.10	1.58	0.00		Grass	Soil	Sky	Tree
GV_1102	0.78	32.03	0.28	24.00	2.95	16.20	9.75	11.58	0.02	0.02	0.14	2.24	0.00		Grass	Tree	Sky	Bare Rock
GV_1103	20.46	3.37	25.58	11.82	15.64	12.17	1.13	0.51	2.58	1.30	5.39	0.06	0.00		Building	Background	Roads	Sky
GV_1104	12.30	28.69	15.93	15.46	17.09	0.95	0.01	0.06	0.00	7.07	0.00	2.44	0.00		Grass	Roads	Building	Tree
GV_1105	11.71	9.74	21.99	14.88	32.04	8.27	0.21	0.03	0.00	1.14	0.00	0.01	0.00		Roads	Building	Tree	Background
GV_1106	3.95	3.60	26.86	20.03	19.74	2.54	5.45	2.00	0.00	15.08	0.00	0.76	0.00		Building	Tree	Roads	Sidewalk
GV_1107	0.53	47.33	0.00	33.98	3.24	0.64	10.82	0.40	0.00	0.00	0.98	2.08	0.00		Grass	Tree	Soil	Roads
GV_1108	1.12	48.12	4.18	37.21	0.26	3.60	0.33	0.11	0.00	3.66	0.00	1.38	0.01		Grass	Tree	Building	Sidewalk
GV_1109	2.31	30.60	14.11	25.62	9.43	1.91	0.24	3.29	0.00	12.29	0.03	0.15	0.00		Grass	Tree	Building	Sidewalk
GV_1110	3.31	10.39	23.71	12.92	7.92	20.54	1.74	9.47	1.19	0.77	1.85	6.20	0.00		Building	Sky	Tree	Grass
GV_1111	5.89	36.81	9.50	15.54	7.56	13.97	2.97	0.09	0.02	3.07	0.10	4.47	0.00		Grass	Tree	Sky	Building
GV_1112	6.11	20.06	16.26	7.62	26.99	16.22	2.97	0.66	0.00	0.61	1.04	1.45	0.00		Roads	Grass	Building	Sky
GV_1113	1.75	21.76	0.00	5.30	32.18	27.63	6.83	2.10	0.00	0.00	0.00	2.41	0.02		Roads	Sky	Grass	Soil
GV_1114	1.32	58.33	0.02	2.81	3.73	20.52	2.95	2.40	0.00	0.09	0.19	7.44	0.19		Grass	Sky	Gravel	Roads
GV_1115	1.22	74.57	0.27	0.12	0.20	15.17	1.66	2.64	0.70	0.16	2.48	0.00	0.79		Grass	Sky	Bare Rock	Water
GV_1116	0.19	53.11	0.00	0.00	5.76	25.18	2.94	1.60	0.00	0.00	0.00	10.84	0.37		Grass	Sky	Gravel	Roads
GV_1117	2.61	65.42	2.90	26.20	1.28	1.48	0.04	0.00	0.00	0.07	0.00	0.00	0.00		Grass	Tree	Building	Background
GV_1118	0.62	18.92	9.84	24.36	24.76	4.02	0.32	0.06	0.00	16.89	0.00	0.21	0.00		Roads	Tree	Grass	Sidewalk
GV_1119	3.63	21.31	12.63	22.71	14.61	1.87	0.07	0.02	0.01	23.13	0.00	0.00	0.00		Sidewalk	Tree	Grass	Roads

GV_1120	4.71	25.19	0.12	44.07	1.12	1.98	13.96	0.06	0.11	2.51	0.02	6.13	0.01		Tree	Grass	Soil	Gravel
GV_1121	0.08	50.78	0.24	24.17	11.47	6.63	0.12	0.00	0.00	6.51	0.00	0.00	0.00		Grass	Tree	Roads	Sky
GV_1122	3.95	77.56	0.00	0.39	6.36	0.00	7.33	0.08	0.04	0.74	0.01	3.54	0.00		Grass	Soil	Roads	Background
GV_1123	0.94	59.78	0.20	0.00	16.98	0.01	13.50	0.03	0.03	7.72	0.18	0.64	0.00		Grass	Roads	Soil	Sidewalk
GV_1124	1.51	85.43	0.46	7.58	0.00	5.02	0.00	0.00	0.00	0.00	0.00	0.00	0.00		Grass	Tree	Sky	Background
GV_1125	0.34	8.58	1.02	5.16	1.67	79.41	0.76	0.07	0.00	0.11	0.68	0.07	2.12		Sky	Grass	Tree	Blue Mountain
GV_1126	0.24	0.01	7.46	3.92	1.44	77.11	0.00	0.15	0.19	9.46	0.01	0.00	0.00		Sky	Sidewalk	Building	Tree
GV_1127	2.68	42.84	0.51	21.26	18.44	5.23	0.32	0.13	0.76	2.75	0.00	4.94	0.12		Grass	Tree	Roads	Sky
GV_1128	0.46	4.84	0.04	6.39	74.49	12.35	0.07	0.24	0.89	0.22	0.00	0.01	0.00		Roads	Sky	Tree	Grass
GV_1129	0.77	71.23	0.12	14.37	1.39	10.26	0.69	0.04	0.00	0.71	0.00	0.42	0.00		Grass	Tree	Sky	Roads
GV_1130	1.31	28.15	0.09	13.74	39.98	8.05	0.82	0.00	0.00	0.02	0.00	7.74	0.09		Roads	Grass	Tree	Sky
GV_1131	11.51	37.57	1.82	17.91	7.84	10.54	9.90	0.02	0.53	0.02	0.07	2.18	0.09		Grass	Tree	Background	Sky
GV_1132	1.20	47.69	0.13	8.96	27.34	8.12	4.79	0.00	0.00	0.21	1.36	0.15	0.07		Grass	Roads	Tree	Sky
GV_1133	0.42	11.80	0.08	5.48	61.68	8.11	9.30	2.07	0.01	0.10	0.55	0.41	0.00		Roads	Grass	Soil	Sky
GV_1134	4.06	6.07	13.17	16.23	23.24	19.95	8.66	0.59	0.68	5.05	2.07	0.06	0.17		Roads	Sky	Tree	Building
GV_1135	0.09	76.45	0.08	6.59	11.08	4.25	0.39	0.01	0.00	0.00	0.00	0.98	0.08		Grass	Roads	Tree	Sky
GV_1136	0.78	37.48	1.09	17.60	21.93	13.53	2.35	0.10	0.00	0.02	0.00	5.13	0.00		Grass	Roads	Tree	Sky
GV_1137	0.04	35.70	3.82	6.20	24.23	26.36	0.43	0.33	0.60	0.03	2.20	0.01	0.05		Grass	Sky	Roads	Tree
GV_1138	0.88	51.97	0.84	23.01	0.88	22.41	0.00	0.00	0.00	0.00	0.00	0.00	0.00		Grass	Tree	Sky	Background
GV_1139	0.75	33.19	12.09	11.41	0.00	42.13	0.01	0.05	0.00	0.34	0.00	0.04	0.00		Sky	Grass	Building	Tree
GV_1140	1.44	27.85	0.62	28.16	14.58	3.56	8.03	0.02	0.01	0.09	0.33	15.32	0.00		Tree	Grass	Gravel	Roads
GV_1141	0.00	93.00	0.00	4.96	0.56	1.46	0.00	0.00	0.00	0.00	0.00	0.00	0.01		Grass	Tree	Sky	Roads
GV_1142	0.00	87.54	0.01	9.89	1.84	0.56	0.11	0.00	0.00	0.00	0.00	0.04	0.00		Grass	Tree	Roads	Sky
GV_1143	4.06	26.47	3.17	17.25	27.39	17.79	1.39	0.38	0.29	0.05	0.14	1.60	0.02		Roads	Grass	Sky	Tree
GV_1144	0.40	60.81	2.87	7.02	15.22	12.97	0.51	0.04	0.00	0.09	0.03	0.02	0.01		Grass	Roads	Sky	Tree
GV_1145	0.60	40.84	0.21	5.43	18.01	30.73	1.78	0.58	0.00	0.00	0.03	1.41	0.37		Grass	Sky	Roads	Tree
GV_1146	12.01	1.82	4.40	27.53	44.20	7.55	0.00	0.04	0.00	2.45	0.00	0.00	0.00		Roads	Tree	Background	Sky
GV_1147	6.79	11.11	5.79	35.79	23.05	2.80	0.64	0.25	0.73	8.21	4.72	0.12	0.00		Tree	Roads	Grass	Sidewalk
GV_1148	3.94	53.73	11.82	24.26	2.81	3.22	0.04	0.00	0.00	0.16	0.02	0.02	0.00		Grass	Tree	Building	Background
GV_1149	1.74	54.55	4.08	37.11	0.88	0.55	0.10	0.01	0.00	0.37	0.00	0.60	0.00		Grass	Tree	Building	Background
GV_1150	0.16	53.05	0.21	37.81	0.31	8.40	0.01	0.01	0.00	0.01	0.00	0.01	0.00		Grass	Tree	Sky	Roads
GV_1151	3.98	20.58	1.07	19.70	11.17	31.25	0.78	1.17	1.37	1.62	4.20	0.01	3.10		Sky	Grass	Tree	Roads
GV_1152	1.17	10.97	0.10	74.99	2.83	2.08	7.50	0.00	0.00	0.00	0.00	0.37	0.00		Tree	Grass	Soil	Roads
GV_1153	1.80	58.12	1.44	1.89	30.99	0.02	4.51	0.03	0.00	1.10	0.03	0.06	0.00		Grass	Roads	Soil	Tree
GV_1154	2.16	80.92	0.08	1.28	14.12	0.00	0.60	0.00	0.25	0.08	0.45	0.04	0.00		Grass	Roads	Background	Tree
GV_1155	0.44	73.36	0.06	1.34	15.32	0.00	3.25	0.01	0.00	5.98	0.00	0.24	0.00		Grass	Roads	Sidewalk	Soil
GV_1156	1.25	91.86	0.05	2.67	4.06	0.01	0.00	0.01	0.03	0.03	0.01	0.00	0.00		Grass	Roads	Tree	Background
GV_1157	2.69	53.91	18.90	7.16	0.16	0.07	1.83	1.56	0.00	13.49	0.00	0.23	0.00		Grass	Building	Sidewalk	Tree
GV_1158	0.19	51.14	0.00	6.17	0.06	40.18	1.89	0.00	0.00	0.01	0.00	0.35	0.00		Grass	Sky	Tree	Soil
GV_1159	4.95	58.81	28.50	1.99	1.12	0.99	0.30	0.01	0.00	3.35	0.00	0.00	0.00		Grass	Building	Background	Sidewalk
GV_1160	2.13	14.04	0.23	63.62	10.05	2.09	0.36	0.03	0.00	5.88	0.00	1.56	0.00		Tree	Grass	Roads	Sidewalk
GV_1161	0.59	9.84	1.34	74.79	0.19	0.10	2.74	3.91	0.23	0.01	0.01	6.24	0.00		Tree	Grass	Gravel	Bare Rock
GV_1162	6.34	22.71	3.74	6.41	20.03	25.59	8.50	1.85	2.08	0.85	0.46	0.76	0.68		Sky	Grass	Roads	Soil
GV_1163	1.77	47.97	0.01	21.69	0.00	27.69	0.51	0.00	0.00	0.01	0.00	0.34	0.00		Grass	Sky	Tree	Background
GV_1164	6.98	7.22	13.41	6.35	7.73	17.98	0.00	0.00	0.95	0.39	38.72	0.26	0.00		Water	Sky	Building	Roads
GV_1165	0.10	39.81	0.01	5.14	0.03	42.31	7.88	0.00	0.00	0.00	0.00	4.72	0.00		Sky	Grass	Soil	Tree
GV_1166	3.95	39.75	8.78	17.59	19.41	5.28	1.55	0.00	0.17	2.03	0.09	1.41	0.00		Grass	Roads	Tree	Building
GV_1167	3.51	11.83	9.48	14.20	19.46	17.34	3.38	4.88	0.03	4.70	10.46	0.74	0.01		Roads	Sky	Tree	Grass
GV_1168	5.24	20.30	6.00	8.07	18.25	28.41	11.44	1.03	0.11	0.12	0.00	0.60	0.43		Sky	Grass	Roads	Soil
GV_1169	0.40	97.31	0.01	1.09	0.00	0.00	1.19	0.00	0.00	0.00	0.00	0.00	0.00		Grass	Soil	Tree	Background
GV_1170	4.46	93.67	0.00	0.00	0.16	0.00	1.70	0.02	0.00	0.00	0.00	0.00	0.00		Grass	Background	Soil	Roads
GV_1171	1.02	48.25	0.00	5.50	0.33	40.18	1.07	0.00	0.00	0.12	0.00	3.54	0.00		Grass	Sky	Tree	Gravel
GV_1172	6.10	40.58	9.84	25.91	2.10	12.75	0.04	0.01	0.03	2.63	0.00	0.00	0.01		Grass	Tree	Sky	Building
GV_1173	18.01	16.43	7.50	55.32	0.12	1.46	0.12	0.04	0.00	0.88	0.00	0.11	0.00		Tree	Background	Grass	Building
GV_1174	0.41	42.78	0.34	20.27	0.01	35.82	0.01	0.00	0.00	0.00	0.00	0.36	0.01		Grass	Sky	Tree	Background
GV_1175	0.32	49.85	0.00	5.07	0.09	40.96	0.07	0.00	0.00	0.00	0.00	3.64	0.00		Grass	Sky	Tree	Gravel
GV_1176	4.20	31.58	46.65	6.87	2.41	0.82	0.27	0.35	0.00	6.45	0.00	0.41	0.00		Building	Grass	Tree	Sidewalk
GV_1177	0.33	35.36	0.15	15.87	11.11	34.39	0.03	0.00	0.00	0.01	0.00	2.75	0.00		Grass	Sky	Tree	Roads
GV_1178	0.97	52.96	0.22	4.79	3.99	32.93	1.47	0.42	0.00	0.05	0.06	2.14	0.00		Grass	Sky	Tree	Roads

GV_1179	8.33	15.99	0.13	45.03	9.68	5.45	12.92	0.19	1.02	0.09	0.03	1.03	0.09		Tree	Grass	Soil	Roads
GV_1180	12.02	69.48	0.01	0.22	3.78	0.18	7.91	0.04	0.34	5.61	0.40	0.01	0.00		Grass	Background	Soil	Sidewalk
GV_1181	30.70	5.74	12.25	15.17	20.06	2.43	4.10	0.34	0.11	3.73	0.00	5.38	0.00		Background	Roads	Tree	Building
GV_1182	2.34	8.84	25.58	25.26	21.28	7.65	0.39	0.25	0.62	7.15	0.16	0.50	0.00		Building	Tree	Roads	Grass
GV_1183	4.41	8.21	22.13	19.34	26.36	4.86	2.50	0.03	0.00	11.55	0.00	0.59	0.02		Roads	Building	Tree	Sidewalk
GV_1184	2.37	4.20	14.57	25.23	34.48	4.30	0.13	0.03	0.03	14.37	0.00	0.28	0.00		Roads	Tree	Building	Sidewalk
GV_1185	1.88	16.75	11.02	30.88	18.59	1.52	0.05	0.05	0.00	18.99	0.00	0.28	0.00		Tree	Sidewalk	Roads	Grass
GV_1186	2.80	7.29	18.29	20.98	29.42	3.92	0.02	0.00	0.00	17.28	0.00	0.00	0.00		Roads	Tree	Building	Sidewalk
GV_1187	7.85	3.79	27.35	15.91	21.21	0.98	0.27	0.12	0.01	22.34	0.00	0.18	0.00		Building	Sidewalk	Roads	Tree
GV_1188	2.68	3.08	34.27	12.41	20.86	2.23	0.03	0.00	0.00	24.32	0.00	0.12	0.00		Building	Sidewalk	Roads	Tree
GV_1189	11.82	1.84	19.10	12.90	35.57	6.45	0.02	0.01	0.00	12.30	0.00	0.00	0.00		Roads	Building	Tree	Sidewalk
GV_1190	4.69	2.88	27.96	29.09	12.90	6.54	0.17	0.51	0.00	14.98	0.00	0.29	0.00		Tree	Building	Sidewalk	Roads
GV_1191	6.58	0.72	45.58	8.64	18.01	2.62	0.00	0.16	0.00	15.33	0.00	2.35	0.00		Building	Roads	Sidewalk	Tree
GV_1192	15.43	4.13	32.25	6.36	25.61	1.87	0.75	0.09	0.00	11.71	0.01	1.80	0.00		Building	Roads	Background	Sidewalk
GV_1193	4.67	1.65	18.63	5.91	1.14	36.60	0.17	26.39	0.05	2.81	0.38	0.00	1.60		Sky	Bare Rock	Building	Tree
GV_1194	7.77	7.14	22.29	35.65	4.57	5.63	11.92	0.05	0.09	3.16	1.63	0.10	0.00		Tree	Building	Soil	Background
GV_1195	2.61	17.32	7.65	21.64	23.50	11.15	0.26	0.01	0.00	15.43	0.00	0.43	0.00		Roads	Tree	Grass	Sidewalk
GV_1196	18.23	4.87	13.99	25.48	28.22	3.61	0.08	0.98	0.00	3.87	0.00	0.67	0.00		Roads	Tree	Background	Building
GV_1197	3.68	37.94	0.49	49.66	2.71	0.77	0.26	0.00	0.00	2.96	0.00	1.54	0.00		Tree	Grass	Background	Sidewalk
GV_1198	0.67	50.49	1.27	20.09	0.00	27.47	0.00	0.00	0.00	0.00	0.00	0.00	0.00		Grass	Sky	Tree	Building
GV_1199	0.25	31.65	0.51	30.96	8.75	25.04	1.07	0.00	0.00	1.21	0.00	0.56	0.00		Grass	Tree	Sky	Roads
GV_1200	0.56	53.54	0.08	5.71	0.49	36.64	0.62	0.00	0.01	0.02	0.00	2.33	0.00		Grass	Sky	Tree	Gravel
GV_1201	10.36	5.38	1.86	26.16	26.86	18.33	1.51	0.22	0.03	8.80	0.02	0.00	0.47		Roads	Tree	Sky	Background
GV_1202	3.65	23.14	0.70	48.75	6.13	12.19	0.30	0.01	0.00	0.38	0.00	4.74	0.00		Tree	Grass	Sky	Roads
GV_1203	5.02	39.41	1.59	38.42	2.61	5.00	1.00	0.02	0.00	0.51	0.74	5.69	0.00		Grass	Tree	Gravel	Background
GV_1204	0.17	57.86	0.42	34.88	0.00	6.65	0.00	0.02	0.00	0.00	0.00	0.00	0.00		Grass	Tree	Sky	Building
GV_1205	12.43	29.29	10.15	16.50	9.24	2.52	7.56	0.09	0.01	2.91	0.04	9.27	0.00		Grass	Tree	Background	Building
GV_1206	3.48	23.77	12.83	17.48	7.29	22.85	0.50	0.04	0.00	0.31	0.00	11.45	0.00		Grass	Sky	Tree	Building
GV_1207	3.69	21.48	0.20	33.64	4.21	0.50	3.75	0.01	0.00	28.57	0.00	3.93	0.00		Tree	Sidewalk	Grass	Roads
GV_1208	6.94	2.76	0.68	39.86	42.36	6.87	0.00	0.00	0.00	0.04	0.00	0.50	0.00		Roads	Tree	Background	Sky
GV_1209	2.75	44.71	0.14	24.87	0.49	9.26	7.72	0.00	0.78	8.63	0.00	0.64	0.01		Grass	Tree	Sky	Sidewalk
GV_1210	8.95	0.38	28.21	8.09	1.98	27.91	0.01	7.35	1.32	3.01	12.79	0.00	0.00		Building	Sky	Water	Background
GV_1211	12.06	3.50	22.89	1.13	38.04	11.84	0.71	1.58	7.33	0.91	0.00	0.01	0.00		Roads	Building	Background	Sky
GV_1212	1.06	42.96	0.09	17.51	0.11	37.33	0.20	0.02	0.00	0.36	0.00	0.37	0.00		Grass	Sky	Tree	Background
GV_1213	4.20	26.00	13.64	34.35	2.42	0.41	1.87	0.13	0.00	16.88	0.00	0.09	0.00		Tree	Grass	Sidewalk	Building
GV_1214	31.40	1.88	12.71	16.44	10.08	7.15	7.62	0.04	0.00	12.41	0.00	0.26	0.00		Background	Tree	Building	Sidewalk
GV_1215	3.34	55.85	5.74	26.07	0.39	5.89	0.36	0.28	0.00	1.31	0.00	0.76	0.00		Grass	Tree	Sky	Building
GV_1216	3.56	57.92	5.35	10.01	13.36	2.12	4.10	0.07	0.07	1.50	0.03	1.89	0.01		Grass	Roads	Tree	Building
GV_1217	0.19	77.49	0.01	8.90	0.00	7.92	3.21	0.00	0.00	0.00	0.00	0.05	2.22		Grass	Tree	Sky	Soil
GV_1218	7.13	6.09	24.00	10.67	8.75	38.69	0.31	2.40	0.01	0.38	0.12	0.89	0.56		Sky	Building	Tree	Roads
GV_1219	11.20	13.59	13.34	31.32	10.11	18.35	0.56	1.18	0.01	0.30	0.00	0.04	0.00		Tree	Sky	Grass	Building
GV_1220	8.28	44.90	9.25	14.74	0.62	12.17	7.37	0.00	0.09	2.48	0.00	0.10	0.00		Grass	Tree	Sky	Building
GV_1221	2.24	48.25	1.14	36.89	0.33	7.42	0.01	0.05	0.00	3.65	0.00	0.02	0.00		Grass	Tree	Sky	Sidewalk
GV_1222	4.04	32.21	0.82	25.32	15.83	1.76	8.92	0.00	2.45	8.63	0.00	0.02	0.00		Grass	Tree	Roads	Soil
GV_1223	1.47	57.02	9.43	19.37	0.18	6.62	0.04	0.13	0.00	3.48	0.00	2.26	0.00		Grass	Tree	Building	Sky
GV_1224	0.58	25.71	3.87	36.71	19.59	6.29	0.18	0.00	0.00	7.03	0.00	0.04	0.00		Tree	Grass	Roads	Sidewalk
GV_1225	19.23	1.69	38.04	0.45	16.39	17.36	0.18	0.06	0.00	6.45	0.12	0.00	0.02		Building	Background	Sky	Roads
GV_1226	18.82	16.33	16.99	3.62	34.52	0.44	0.36	0.10	0.00	8.02	0.00	0.80	0.00		Roads	Background	Building	Grass
GV_1227	2.77	25.53	0.88	1.65	53.25	0.01	0.46	0.13	0.00	15.33	0.00	0.00	0.00		Roads	Grass	Sidewalk	Background
GV_1228	8.86	18.80	17.03	6.87	33.27	0.22	2.98	0.67	0.04	10.92	0.02	0.32	0.00		Roads	Grass	Building	Sidewalk
GV_1229	7.98	4.90	64.74	12.30	1.59	5.45	0.06	0.70	0.00	2.28	0.00	0.00	0.00		Building	Tree	Background	Sky
GV_1230	13.00	39.76	16.36	6.83	4.31	1.79	0.09	0.00	0.13	17.60	0.12	0.00	0.00		Grass	Sidewalk	Building	Background
GV_1231	0.75	58.49	0.03	4.49	3.37	22.56	1.25	0.00	0.00	0.00	0.00	9.05	0.02		Grass	Sky	Gravel	Tree
GV_1232	10.53	7.07	11.62	9.81	49.25	9.72	0.88	0.08	0.00	0.46	0.00	0.59	0.00		Roads	Building	Background	Tree
GV_1233	17.70	35.30	10.32	16.99	3.18	4.78	0.01	0.03	0.00	11.68	0.01	0.01	0.00		Grass	Background	Tree	Sidewalk
GV_1234	0.11	45.02	9.23	27.34	9.44	8.18	0.33	0.12	0.00	0.03	0.00	0.20	0.00		Grass	Tree	Roads	Building
GV_1235	2.88	37.14	12.39	31.57	0.24	6.28	2.06	0.17	0.00	7.20	0.00	0.06	0.00		Grass	Tree	Building	Sidewalk
GV_1236	12.85	4.33	28.34	11.86	18.10	1.99	0.11	0.02	0.00	22.17	0.00	0.23	0.00		Building	Sidewalk	Roads	Background
GV_1237	1.10	25.23	22.16	19.95	5.00	5.49	5.83	0.02	0.66	13.22	0.00	0.00	1.34		Grass	Building	Tree	Sidewalk

GV_1238	5.52	15.85	23.28	12.14	8.08	10.07	1.06	0.52	0.90	22.53	0.02	0.01	0.02		Building	Sidewalk	Grass	Tree
GV_1239	12.30	31.66	0.04	48.60	0.20	0.15	0.99	0.08	5.36	0.01	0.02	0.58	0.00		Tree	Grass	Background	Sand
GV_1240	3.03	12.41	3.14	14.25	19.74	19.07	0.16	0.46	1.42	10.70	15.53	0.00	0.08		Roads	Sky	Water	Tree
GV_1241	0.49	85.85	5.39	5.02	0.70	1.71	0.31	0.00	0.00	0.53	0.00	0.00	0.00		Grass	Building	Tree	Sky
GV_1242	0.13	71.87	0.05	0.00	0.33	0.00	26.80	0.01	0.00	0.81	0.00	0.00	0.00		Grass	Soil	Sidewalk	Roads
GV_1243	3.39	48.60	4.29	3.32	2.09	2.54	0.59	0.00	0.00	35.06	0.00	0.12	0.00		Grass	Sidewalk	Building	Background
GV_1244	2.00	47.87	2.72	10.05	0.04	37.17	0.07	0.00	0.00	0.08	0.00	0.00	0.00		Grass	Sky	Tree	Building
GV_1245	5.56	58.10	0.40	10.04	0.00	25.90	0.00	0.00	0.00	0.01	0.00	0.00	0.00		Grass	Sky	Tree	Background
GV_1246	5.28	41.95	1.94	8.89	0.04	41.83	0.06	0.00	0.00	0.02	0.00	0.00	0.00		Grass	Sky	Tree	Background
GV_1247	4.45	50.81	1.64	5.44	0.00	37.65	0.01	0.00	0.00	0.00	0.00	0.00	0.00		Grass	Sky	Tree	Background
GV_1248	0.12	81.14	1.05	7.60	0.00	10.09	0.00	0.00	0.00	0.00	0.00	0.00	0.00		Grass	Sky	Tree	Building
GV_1249	1.72	49.24	4.42	9.28	18.08	11.22	1.00	0.00	0.00	1.58	0.00	3.48	0.00		Grass	Roads	Sky	Tree
GV_1250	0.64	58.94	3.22	9.56	0.01	27.42	0.22	0.00	0.00	0.00	0.00	0.00	0.00		Grass	Sky	Tree	Building
GV_1251	2.47	33.97	5.88	6.61	13.96	31.44	1.20	0.05	0.00	3.77	0.01	0.00	0.64		Grass	Sky	Roads	Tree
GV_1252	1.23	45.98	7.57	22.90	12.04	9.43	0.14	0.00	0.00	0.70	0.00	0.01	0.00		Grass	Tree	Roads	Sky
GV_1253	17.01	0.23	11.01	27.02	18.82	25.38	0.00	0.03	0.00	0.50	0.00	0.01	0.00		Tree	Sky	Roads	Background
GV_1254	1.53	31.73	3.40	12.14	8.50	33.48	0.26	0.01	0.35	7.85	0.00	0.76	0.00		Sky	Grass	Tree	Roads
GV_1255	0.56	51.11	1.15	8.98	0.00	38.19	0.00	0.00	0.00	0.00	0.00	0.00	0.00		Grass	Sky	Tree	Building
GV_1256	0.32	67.68	2.28	8.47	0.00	21.24	0.00	0.00	0.00	0.00	0.00	0.00	0.00		Grass	Sky	Tree	Building
GV_1257	0.59	64.56	5.22	24.55	0.17	4.81	0.00	0.02	0.00	0.08	0.00	0.00	0.00		Grass	Tree	Building	Sky
GV_1258	3.47	54.24	2.49	20.32	2.53	5.52	1.03	0.94	0.00	6.37	0.00	3.08	0.02		Grass	Tree	Sidewalk	Sky
GV_1259	0.56	67.00	3.34	18.12	0.01	10.96	0.00	0.00	0.00	0.01	0.00	0.00	0.00		Grass	Tree	Sky	Building
GV_1260	1.20	98.15	0.01	0.56	0.08	0.00	0.00	0.00	0.00	0.00	0.00	0.00	0.00		Grass	Background	Tree	Roads
GV_1261	1.86	45.88	0.17	0.67	33.27	0.05	0.00	0.11	0.00	18.00	0.00	0.00	0.00		Grass	Roads	Sidewalk	Background
GV_1262	0.61	63.57	3.83	28.50	0.01	1.86	0.71	0.00	0.00	0.10	0.00	0.80	0.00		Grass	Tree	Building	Sky
GV_1263	2.98	0.86	41.36	4.88	29.12	3.90	0.03	0.41	0.01	16.46	0.00	0.00	0.00		Building	Roads	Sidewalk	Tree
GV_1264	2.43	1.35	46.59	0.74	29.31	1.78	0.00	0.01	0.00	17.65	0.00	0.14	0.00		Building	Roads	Sidewalk	Background
GV_1265	2.27	17.18	25.95	0.78	41.55	0.19	0.52	0.14	0.01	11.41	0.00	0.00	0.00		Roads	Building	Grass	Sidewalk
GV_1266	0.96	13.66	28.43	8.54	24.03	0.99	0.12	0.00	0.13	23.14	0.00	0.00	0.00		Building	Roads	Sidewalk	Grass
GV_1267	3.58	19.98	29.97	4.58	14.24	1.17	0.00	0.00	0.00	26.46	0.00	0.00	0.00		Building	Sidewalk	Grass	Roads
GV_1268	0.93	54.61	25.74	2.00	6.19	0.30	0.00	0.00	0.00	10.23	0.00	0.00	0.00		Grass	Building	Sidewalk	Roads
GV_1269	3.49	5.06	28.50	8.28	25.77	2.00	0.06	0.01	0.00	26.79	0.00	0.05	0.00		Building	Sidewalk	Roads	Tree
GV_1270	4.57	1.20	40.63	2.91	26.77	9.19	0.03	0.00	0.00	14.67	0.00	0.01	0.00		Building	Roads	Sidewalk	Sky
GV_1271	7.89	1.16	44.38	1.12	6.79	3.91	0.12	0.01	2.81	31.71	0.00	0.10	0.00		Building	Sidewalk	Background	Roads
GV_1272	2.64	1.08	30.63	3.15	15.31	0.75	8.95	0.01	0.00	34.94	0.00	2.54	0.00		Sidewalk	Building	Roads	Soil
GV_1273	0.58	7.74	4.74	11.53	50.75	14.10	0.36	1.76	2.69	2.78	2.77	0.04	0.15		Roads	Sky	Tree	Grass
GV_1274	3.08	64.66	0.07	13.61	15.18	1.16	0.00	0.01	0.00	1.59	0.00	0.65	0.00		Grass	Roads	Tree	Background
GV_1275	0.26	60.71	0.06	24.94	7.96	0.52	0.29	0.00	0.00	5.26	0.00	0.00	0.00		Grass	Tree	Roads	Sidewalk
GV_1276	11.74	37.60	11.32	13.75	8.01	8.75	0.30	0.86	0.00	7.61	0.00	0.01	0.05		Grass	Tree	Background	Building
GV_1277	11.21	26.20	12.56	9.94	7.23	9.31	0.04	0.20	0.11	23.00	0.18	0.02	0.00		Grass	Sidewalk	Building	Background
GV_1278	2.66	42.20	31.61	3.09	0.07	12.56	7.69	0.00	0.00	0.04	0.00	0.07	0.00		Grass	Building	Sky	Soil
GV_1279	1.43	40.27	8.66	18.08	14.81	14.07	0.05	0.00	0.00	2.59	0.00	0.00	0.03		Grass	Tree	Roads	Sky
GV_1280	1.20	13.11	0.15	13.92	14.56	24.17	19.63	0.14	6.03	3.63	3.00	0.00	0.46		Sky	Soil	Roads	Tree
GV_1281	1.06	49.52	4.03	37.19	0.25	4.13	0.07	0.03	0.00	3.71	0.00	0.01	0.00		Grass	Tree	Sky	Building
GV_1282	7.38	55.05	0.80	23.70	4.04	0.78	0.20	0.00	2.68	5.37	0.00	0.00	0.00		Grass	Tree	Background	Sidewalk
GV_1283	1.33	24.48	11.60	23.22	23.41	6.58	0.03	0.00	0.00	9.34	0.00	0.02	0.00		Grass	Roads	Tree	Building
GV_1284	0.45	40.63	1.73	36.82	16.09	2.13	0.02	0.00	0.00	2.13	0.00	0.00	0.00		Grass	Tree	Roads	Sky
GV_1285	3.06	33.12	1.32	39.92	16.86	1.49	0.11	0.00	0.00	4.10	0.00	0.02	0.00		Tree	Grass	Roads	Sidewalk
GV_1286	6.97	2.31	42.42	1.13	25.07	0.18	0.02	1.44	0.06	20.34	0.00	0.07	0.00		Building	Roads	Sidewalk	Background
GV_1287	3.92	1.69	46.12	0.09	25.96	3.50	0.01	0.03	0.00	18.43	0.00	0.24	0.00		Building	Roads	Sidewalk	Background
GV_1288	3.97	30.29	1.10	31.69	5.05	13.63	11.77	0.62	0.00	0.11	0.00	1.78	0.01		Tree	Grass	Sky	Soil
GV_1289	0.42	82.76	0.00	12.18	1.06	2.27	0.26	0.00	0.00	0.88	0.00	0.16	0.00		Grass	Tree	Sky	Roads
GV_1290	0.91	19.50	0.07	37.05	29.64	1.21	1.92	0.00	0.00	3.92	0.01	5.77	0.00		Tree	Roads	Grass	Gravel
GV_1291	0.07	49.30	0.41	14.34	0.89	29.85	1.00	0.00	0.00	0.19	0.03	3.92	0.00		Grass	Sky	Tree	Gravel
GV_1292	3.21	71.75	1.25	5.00	3.85	10.05	0.17	0.33	0.00	4.27	0.05	0.00	0.06		Grass	Sky	Tree	Sidewalk
GV_1293	3.87	76.63	0.67	0.79	1.88	5.29	8.70	0.05	0.00	1.90	0.00	0.02	0.20		Grass	Soil	Sky	Background
GV_1294	0.89	84.07	2.01	11.83	0.54	0.29	0.03	0.00	0.00	0.35	0.00	0.00	0.00		Grass	Tree	Building	Background
GV_1295	0.00	83.11	0.00	0.00	0.19	0.00	16.65	0.05	0.00	0.00	0.00	0.00	0.00		Grass	Soil	Roads	Bare Rock
GV_1296	0.25	63.69	2.38	9.64	0.02	23.79	0.22	0.00	0.00	0.00	0.00	0.00	0.00		Grass	Sky	Tree	Building

GV_1297	9.93	1.02	54.40	4.98	2.75	6.62	2.40	0.06	0.07	17.78	0.00	0.00	0.00		Building	Sidewalk	Background	Sky
GV_1298	12.02	9.19	10.25	15.17	26.60	4.66	0.06	0.00	0.03	21.34	0.00	0.67	0.02		Roads	Sidewalk	Tree	Background
GV_1299	0.46	88.14	5.61	4.29	1.12	0.13	0.01	0.00	0.00	0.21	0.03	0.00	0.00		Grass	Building	Tree	Roads
GV_1300	9.51	34.61	11.64	5.27	10.55	11.01	0.00	0.03	0.29	17.08	0.00	0.01	0.00		Grass	Sidewalk	Building	Sky
GV_1301	2.87	45.82	1.01	2.43	3.68	40.81	0.49	0.00	0.00	2.85	0.02	0.03	0.00		Grass	Sky	Roads	Background
GV_1302	8.82	44.42	9.51	1.55	1.84	30.22	0.65	0.00	0.00	2.88	0.00	0.10	0.00		Grass	Sky	Building	Background
GV_1303	0.51	81.12	2.83	7.02	6.70	0.06	0.62	0.00	0.00	0.55	0.00	0.59	0.00		Grass	Tree	Roads	Building
GV_1304	0.23	62.85	0.01	17.73	0.00	19.16	0.01	0.00	0.00	0.00	0.00	0.00	0.01		Grass	Sky	Tree	Background
GV_1305	5.36	78.00	0.02	4.05	1.39	0.12	7.32	1.55	0.00	0.70	0.37	1.12	0.00		Grass	Soil	Background	Tree
GV_1306	2.75	5.59	0.45	17.35	20.03	4.89	3.01	4.90	9.73	0.24	28.81	0.01	2.24		Water	Roads	Tree	Sand
GV_1307	17.27	8.57	8.73	15.95	15.41	4.19	0.83	0.00	0.00	29.03	0.00	0.01	0.00		Sidewalk	Background	Tree	Roads
GV_1308	0.88	2.24	4.70	6.78	13.43	29.93	38.82	0.25	1.35	0.79	0.01	0.39	0.42		Soil	Sky	Roads	Tree
GV_1309	1.46	19.19	0.58	10.81	7.58	30.02	28.52	0.29	0.08	0.01	0.00	0.09	1.37		Sky	Soil	Grass	Tree
GV_1310	1.59	14.11	3.87	11.34	17.18	26.93	19.36	3.88	0.90	0.01	0.01	0.76	0.07		Sky	Soil	Roads	Grass
GV_1311	1.42	93.96	0.39	3.41	0.06	0.68	0.08	0.00	0.00	0.00	0.00	0.00	0.00		Grass	Tree	Background	Sky
GV_1312	5.42	55.68	3.08	24.16	0.43	10.02	0.57	0.06	0.00	0.12	0.08	0.38	0.00		Grass	Tree	Sky	Background
GV_1313	5.90	51.87	12.21	19.77	0.64	8.79	0.21	0.02	0.00	0.23	0.00	0.38	0.00		Grass	Tree	Building	Sky
GV_1314	3.16	6.25	1.46	9.44	17.95	36.43	17.37	4.39	0.18	2.86	0.15	0.37	0.00		Sky	Roads	Soil	Tree
GV_1315	0.62	22.05	4.23	5.69	1.29	48.07	14.17	0.03	0.00	2.28	0.00	1.32	0.25		Sky	Grass	Soil	Tree
GV_1316	20.18	8.17	9.09	17.03	7.15	8.28	0.83	0.00	0.00	28.84	0.00	0.43	0.00		Sidewalk	Background	Tree	Building
GV_1317	8.09	7.58	17.16	15.69	23.22	21.50	0.20	0.00	0.00	6.50	0.00	0.07	0.00		Roads	Sky	Building	Tree
GV_1318	1.43	66.04	6.36	22.75	0.56	2.63	0.00	0.01	0.00	0.22	0.00	0.00	0.00		Grass	Tree	Building	Sky
GV_1319	3.51	1.88	41.57	4.43	6.99	5.39	0.90	2.20	0.12	33.00	0.00	0.02	0.00		Building	Sidewalk	Roads	Sky
GV_1320	3.40	48.30	22.45	12.73	3.60	3.48	1.26	0.00	0.00	4.69	0.00	0.10	0.00		Grass	Building	Tree	Sidewalk
GV_1321	5.94	3.99	19.70	18.95	9.84	20.52	0.00	0.00	0.00	21.03	0.00	0.01	0.00		Sidewalk	Sky	Building	Tree
GV_1322	5.79	6.68	16.64	17.60	21.91	7.59	0.53	10.69	0.04	11.51	0.00	1.02	0.00		Roads	Tree	Building	Sidewalk
GV_1323	15.44	11.30	23.59	6.29	25.23	5.14	0.04	0.70	0.00	12.24	0.00	0.03	0.00		Roads	Building	Background	Sidewalk
GV_1324	11.05	22.97	42.85	5.82	3.97	8.39	0.14	0.01	0.00	2.90	0.00	1.91	0.00		Building	Grass	Background	Sky
GV_1325	10.69	27.09	30.71	3.19	8.22	1.85	0.11	0.03	0.06	18.04	0.00	0.00	0.00		Building	Grass	Sidewalk	Background
GV_1326	2.58	31.12	6.41	21.25	24.09	4.07	1.66	0.01	0.00	8.72	0.00	0.09	0.00		Grass	Roads	Tree	Sidewalk
GV_1327	1.23	63.19	4.50	21.32	0.26	9.34	0.07	0.00	0.00	0.09	0.00	0.00	0.00		Grass	Tree	Sky	Building
GV_1328	1.68	60.54	3.85	25.44	2.32	5.26	0.00	0.00	0.00	0.91	0.00	0.02	0.00		Grass	Tree	Sky	Building
GV_1329	1.70	45.30	0.51	19.45	21.00	10.76	0.50	0.61	0.00	0.14	0.00	0.03	0.00		Grass	Roads	Tree	Sky
GV_1330	1.18	45.63	0.08	10.11	6.32	2.87	23.10	0.02	0.21	0.00	0.00	10.48	0.00		Grass	Soil	Gravel	Tree
GV_1331	2.65	72.40	2.70	21.48	0.00	0.52	0.11	0.02	0.00	0.11	0.00	0.00	0.00		Grass	Tree	Building	Background
GV_1332	1.42	24.08	14.54	6.28	31.31	19.60	1.50	0.01	0.00	1.12	0.00	0.13	0.00		Roads	Grass	Sky	Building
GV_1333	1.65	72.40	1.92	23.67	0.00	0.27	0.00	0.01	0.00	0.07	0.00	0.00	0.00		Grass	Tree	Building	Background
GV_1334	4.25	53.82	0.14	33.60	0.00	8.03	0.12	0.00	0.00	0.04	0.00	0.00	0.00		Grass	Tree	Sky	Background
GV_1335	0.40	36.39	12.76	12.07	7.66	20.81	0.01	0.00	0.00	9.85	0.00	0.05	0.00		Grass	Sky	Building	Tree
GV_1336	0.13	80.85	0.79	17.74	0.00	0.49	0.00	0.00	0.00	0.00	0.00	0.00	0.00		Grass	Tree	Building	Sky
GV_1337	0.90	63.47	0.00	28.38	0.81	6.29	0.10	0.00	0.00	0.06	0.00	0.00	0.00		Grass	Tree	Sky	Background
GV_1338	18.25	5.59	8.48	9.68	25.34	0.67	4.64	0.47	0.00	26.74	0.00	0.14	0.00		Sidewalk	Roads	Background	Tree
GV_1339	7.16	20.98	10.80	7.81	35.77	1.51	0.32	3.75	0.00	11.75	0.09	0.06	0.00		Roads	Grass	Sidewalk	Building
GV_1340	20.47	6.49	7.85	6.59	38.16	1.60	0.03	0.43	0.00	18.38	0.00	0.00	0.00		Roads	Background	Sidewalk	Building
GV_1341	9.90	11.38	7.58	21.56	27.03	16.72	0.02	0.06	0.28	5.43	0.00	0.04	0.00		Roads	Tree	Sky	Grass
GV_1342	2.32	49.99	1.99	32.25	0.31	11.83	0.76	0.00	0.00	0.19	0.00	0.35	0.00		Grass	Tree	Sky	Background
GV_1343	11.26	28.65	16.13	24.84	2.32	8.03	0.00	0.00	0.00	8.74	0.00	0.00	0.02		Grass	Tree	Building	Background
GV_1344	12.09	0.60	8.81	1.90	58.67	16.39	0.02	0.84	0.00	0.63	0.00	0.04	0.01		Roads	Sky	Background	Building
GV_1345	1.26	55.74	2.97	30.76	1.92	5.37	0.00	0.00	0.00	1.98	0.00	0.00	0.00		Grass	Tree	Sky	Building
GV_1346	0.18	41.23	0.21	30.60	13.96	9.95	0.01	0.00	0.00	3.31	0.00	0.55	0.00		Grass	Tree	Roads	Sky
GV_1347	3.41	15.92	24.85	3.81	29.02	13.57	0.15	4.33	0.00	4.58	0.37	0.00	0.00		Roads	Building	Grass	Sky
GV_1348	0.98	52.79	7.63	5.86	0.00	31.95	0.00	0.00	0.00	0.00	0.00	0.00	0.78		Grass	Sky	Building	Tree
GV_1349	0.34	49.28	0.05	8.40	0.00	41.13	0.13	0.01	0.01	0.00	0.00	0.06	0.59		Grass	Sky	Tree	Blue Mountain
GV_1350	0.01	88.58	0.01	0.04	0.02	2.31	8.59	0.00	0.00	0.00	0.00	0.17	0.29		Grass	Soil	Sky	Blue Mountain
GV_1351	16.83	9.51	8.74	13.66	11.25	3.11	0.72	0.00	0.24	35.51	0.00	0.44	0.00		Sidewalk	Background	Tree	Roads
GV_1352	0.06	60.93	1.19	28.90	0.00	8.91	0.00	0.00	0.00	0.00	0.00	0.00	0.00		Grass	Tree	Sky	Building
GV_1353	0.09	59.61	0.27	13.53	0.00	26.49	0.00	0.00	0.00	0.00	0.00	0.00	0.00		Grass	Sky	Tree	Building
GV_1354	0.03	57.63	0.48	35.06	0.00	6.80	0.00	0.00	0.00	0.00	0.00	0.00	0.00		Grass	Tree	Sky	Building
GV_1355	6.23	26.09	11.43	16.67	12.14	5.96	8.47	0.00	0.00	11.80	0.00	1.22	0.00		Grass	Tree	Roads	Sidewalk

GV_1356	7.15	3.74	36.56	7.69	27.60	1.41	0.03	0.36	0.00	15.42	0.00	0.02	0.00		Building	Roads	Sidewalk	Tree
GV_1357	1.85	62.50	1.70	10.09	0.00	23.83	0.00	0.00	0.00	0.01	0.00	0.00	0.01		Grass	Sky	Tree	Background
GV_1358	0.67	73.58	0.70	11.62	0.00	13.12	0.00	0.00	0.00	0.06	0.00	0.00	0.26		Grass	Sky	Tree	Building
GV_1359	1.46	66.55	6.22	14.14	0.56	4.35	3.92	0.00	0.00	1.73	0.00	0.07	1.01		Grass	Tree	Building	Sky
GV_1360	0.31	55.35	0.02	33.77	0.00	10.55	0.00	0.00	0.00	0.00	0.00	0.00	0.00		Grass	Tree	Sky	Background
GV_1361	12.55	17.73	2.85	8.85	18.11	37.07	1.48	0.10	0.00	0.43	0.18	0.03	0.61		Sky	Roads	Grass	Background
GV_1362	5.45	50.39	0.08	5.10	0.12	35.74	1.86	0.04	1.18	0.00	0.03	0.01	0.00		Grass	Sky	Background	Tree
GV_1363	4.23	75.07	5.26	10.90	3.41	0.44	0.22	0.00	0.00	0.02	0.16	0.14	0.17		Grass	Tree	Building	Background
GV_1364	1.60	45.06	0.14	17.23	0.31	26.07	9.27	0.02	0.00	0.00	0.00	0.03	0.26		Grass	Sky	Tree	Soil
GV_1365	0.35	82.26	0.29	10.86	0.00	6.24	0.00	0.00	0.00	0.00	0.00	0.00	0.00		Grass	Tree	Sky	Background
GV_1366	1.99	77.64	2.18	13.04	0.02	4.71	0.31	0.12	0.00	0.00	0.00	0.00	0.00		Grass	Tree	Sky	Building
GV_1367	7.50	58.72	0.77	15.68	3.35	9.43	0.04	0.00	0.07	4.06	0.39	0.00	0.00		Grass	Tree	Sky	Background
GV_1368	0.34	40.90	3.04	14.47	25.97	12.44	0.02	0.00	0.00	2.65	0.00	0.15	0.01		Grass	Roads	Tree	Sky
GV_1369	1.24	66.06	1.68	20.90	0.02	10.02	0.00	0.00	0.00	0.09	0.00	0.00	0.00		Grass	Tree	Sky	Building
GV_1370	0.02	47.26	0.06	17.10	0.29	31.31	0.15	0.06	0.00	0.00	0.09	3.66	0.01		Grass	Sky	Tree	Gravel
GV_1371	4.41	20.11	11.72	35.23	0.57	5.26	1.20	0.00	0.47	19.68	0.00	1.35	0.00		Tree	Grass	Sidewalk	Building
GV_1372	1.71	48.82	0.01	4.66	1.88	38.69	0.65	0.00	0.00	0.00	0.00	3.58	0.00		Grass	Sky	Tree	Gravel
GV_1373	3.28	54.60	2.14	22.88	12.05	4.24	0.26	0.00	0.00	0.41	0.00	0.16	0.00		Grass	Tree	Roads	Sky
GV_1374	3.94	65.15	0.00	20.23	6.26	0.04	4.06	0.02	0.00	0.04	0.00	0.26	0.00		Grass	Tree	Roads	Soil
GV_1375	0.18	79.76	0.00	10.15	8.77	0.01	0.24	0.00	0.00	0.89	0.00	0.00	0.00		Grass	Tree	Roads	Sidewalk
GV_1376	0.39	89.40	0.00	3.18	6.60	0.02	0.41	0.00	0.00	0.00	0.00	0.00	0.00		Grass	Roads	Tree	Soil
GV_1377	1.74	83.74	2.33	0.00	8.37	0.25	0.12	0.01	0.00	3.44	0.00	0.00	0.00		Grass	Roads	Sidewalk	Building
GV_1378	0.19	86.67	0.00	2.06	4.01	0.02	6.40	0.00	0.00	0.64	0.00	0.00	0.00		Grass	Soil	Roads	Tree
GV_1379	4.85	15.38	5.58	13.48	5.93	35.28	3.15	6.59	0.00	8.58	0.04	0.84	0.29		Sky	Grass	Tree	Sidewalk
GV_1380	7.17	2.31	30.27	15.19	17.80	4.06	1.81	1.02	0.00	8.88	0.00	11.48	0.02		Building	Roads	Tree	Gravel
GV_1381	0.37	97.20	0.00	1.80	0.00	0.01	0.58	0.03	0.01	0.00	0.00	0.01	0.00		Grass	Tree	Soil	Background
GV_1382	0.27	98.76	0.00	0.00	0.00	0.00	0.96	0.00	0.00	0.00	0.00	0.00	0.00		Grass	Soil	Background	Bare Rock
GV_1383	11.71	5.76	24.19	11.86	28.43	4.95	0.00	0.03	0.08	12.07	0.56	0.36	0.00		Roads	Building	Sidewalk	Tree
GV_1384	0.76	4.50	43.28	4.79	23.04	5.50	0.14	0.06	0.00	17.85	0.00	0.07	0.00		Building	Roads	Sidewalk	Sky
GV_1385	1.18	69.93	0.79	1.37	0.00	26.71	0.01	0.00	0.00	0.00	0.00	0.00	0.00		Grass	Sky	Tree	Background
GV_1386	1.07	44.59	0.28	17.26	0.27	24.84	5.72	0.04	0.00	0.12	0.00	5.80	0.00		Grass	Sky	Tree	Gravel
GV_1387	2.40	69.95	2.63	1.29	1.40	18.99	0.61	0.18	0.00	1.49	0.00	1.04	0.00		Grass	Sky	Building	Background
GV_1388	1.15	58.65	2.98	15.93	0.03	20.13	0.37	0.65	0.00	0.00	0.00	0.12	0.00		Grass	Sky	Tree	Building
GV_1389	8.04	7.46	22.31	12.90	24.78	10.64	0.14	0.47	0.27	12.89	0.01	0.08	0.00		Roads	Building	Tree	Sidewalk
GV_1390	0.49	33.23	0.02	8.54	35.44	3.43	4.05	0.00	0.37	0.00	2.20	12.24	0.00		Roads	Grass	Gravel	Tree
GV_1391	7.44	6.21	5.41	4.34	0.92	0.03	0.67	0.00	0.00	74.97	0.00	0.00	0.00		Sidewalk	Background	Grass	Building
GV_1392	0.20	58.02	0.22	12.26	0.46	25.10	0.07	0.00	0.00	0.07	0.00	3.60	0.00		Grass	Sky	Tree	Gravel
GV_1393	9.05	0.12	23.92	1.75	40.26	10.95	0.00	0.53	0.00	13.42	0.00	0.00	0.00		Roads	Building	Sidewalk	Sky
GV_1394	2.37	41.14	11.44	21.98	1.43	5.93	0.10	0.00	0.00	15.30	0.00	0.32	0.00		Grass	Tree	Sidewalk	Building
GV_1395	0.76	61.45	1.41	10.49	14.27	10.11	0.05	0.01	0.00	0.32	0.46	0.01	0.66		Grass	Roads	Tree	Sky
GV_1396	2.93	66.43	6.64	5.12	1.01	13.46	3.60	0.01	0.00	0.69	0.00	0.00	0.12		Grass	Sky	Building	Tree
GV_1397	0.32	51.73	0.03	8.07	13.28	23.62	0.00	0.00	0.00	0.38	0.00	2.56	0.00		Grass	Sky	Roads	Tree
GV_1398	0.69	56.38	0.14	21.28	0.41	17.35	1.10	0.12	0.00	0.01	0.00	2.51	0.00		Grass	Tree	Sky	Gravel
GV_1399	3.36	12.96	6.03	55.09	0.40	18.97	2.42	0.44	0.00	0.06	0.06	0.21	0.00		Tree	Sky	Grass	Building
GV_1400	1.49	59.37	1.78	20.80	0.58	13.60	0.98	0.00	0.00	0.47	0.00	0.93	0.00		Grass	Tree	Sky	Building
GV_1401	5.43	25.74	5.12	35.54	11.23	10.19	2.15	0.10	0.00	0.67	0.00	3.85	0.00		Tree	Grass	Roads	Sky
GV_1402	1.53	40.85	1.50	42.51	0.60	10.31	0.60	0.06	0.00	1.24	0.00	0.78	0.02		Tree	Grass	Sky	Background
GV_1403	0.61	21.80	0.03	53.10	11.88	2.67	0.31	0.39	0.00	4.84	0.00	4.37	0.00		Tree	Grass	Roads	Sidewalk
GV_1404	0.32	50.88	0.05	36.62	2.39	3.23	0.40	0.00	0.05	5.70	0.00	0.38	0.00		Grass	Tree	Sidewalk	Sky
GV_1405	1.84	10.98	0.84	61.89	4.26	0.69	11.36	1.38	0.00	0.00	0.00	6.78	0.00		Tree	Soil	Grass	Gravel
GV_1406	0.48	50.43	0.06	20.81	14.24	4.65	0.21	0.00	0.02	7.42	0.00	1.68	0.00		Grass	Tree	Roads	Sidewalk
GV_1407	1.94	17.67	0.11	47.44	3.54	0.94	9.80	0.45	0.05	0.51	0.13	17.41	0.00		Tree	Grass	Gravel	Soil
GV_1408	23.20	20.40	5.35	15.79	13.12	13.52	0.52	0.07	0.04	0.18	2.59	5.22	0.00		Background	Grass	Tree	Sky
GV_1409	14.08	0.05	31.42	0.57	30.34	12.70	0.00	0.01	0.07	0.26	1.50	9.00	0.00		Building	Roads	Background	Sky
GV_1410	6.78	71.38	3.69	11.95	0.03	2.78	0.00	0.00	0.00	3.38	0.00	0.00	0.00		Grass	Tree	Background	Building
GV_1411	2.14	56.62	0.16	3.10	18.65	10.54	1.86	0.08	0.00	4.70	0.00	1.11	1.03		Grass	Roads	Sky	Sidewalk
GV_1412	1.16	60.93	0.06	2.62	25.84	0.91	4.61	0.00	0.04	1.20	0.00	2.61	0.02		Grass	Roads	Soil	Tree
GV_1413	1.29	51.86	0.15	9.73	29.70	4.91	1.12	0.00	0.06	0.27	0.00	0.81	0.09		Grass	Roads	Tree	Sky
GV_1414	0.03	65.30	0.89	7.28	1.14	18.11	0.31	0.01	0.00	6.23	0.00	0.57	0.13		Grass	Sky	Tree	Sidewalk

GV_1415	1.17	76.76	2.31	8.71	0.00	10.55	0.05	0.00	0.00	0.05	0.00	0.01	0.39		Grass	Sky	Tree	Building
GV_1416	1.97	42.67	0.19	11.78	0.87	7.83	22.50	2.01	0.00	0.00	0.00	0.04	10.14		Grass	Soil	Tree	Blue Mountain
GV_1417	1.23	69.10	0.19	20.42	0.71	6.15	0.85	0.34	0.00	0.11	0.00	0.92	0.00		Grass	Tree	Sky	Background
GV_1418	24.82	0.73	40.83	0.75	1.00	1.94	0.02	0.52	0.02	29.09	0.00	0.27	0.00		Building	Sidewalk	Background	Sky
GV_1419	0.16	46.71	0.05	14.69	0.02	35.49	1.80	0.00	0.00	0.31	0.00	0.77	0.00		Grass	Sky	Tree	Soil
GV_1420	1.14	60.05	0.26	7.32	3.12	26.94	0.45	0.21	0.05	0.00	0.02	0.41	0.04		Grass	Sky	Tree	Roads
GV_1421	0.09	44.38	0.41	12.86	0.33	33.69	6.53	0.00	0.04	0.48	0.00	1.19	0.00		Grass	Sky	Tree	Soil
GV_1422	2.16	32.05	2.08	3.40	3.46	48.59	4.64	0.06	0.05	2.72	0.00	0.50	0.30		Sky	Grass	Soil	Roads
GV_1423	1.15	18.96	4.80	4.14	6.05	48.59	7.98	6.86	0.68	0.27	0.00	0.47	0.05		Sky	Grass	Soil	Bare Rock
GV_1424	1.25	18.61	0.13	6.21	0.12	46.64	24.94	1.89	0.00	0.02	0.00	0.00	0.19		Sky	Soil	Grass	Tree
GV_1425	0.96	32.20	4.21	15.66	16.68	15.89	5.58	0.00	0.00	0.09	0.00	8.73	0.00		Grass	Roads	Sky	Tree
GV_1426	1.18	23.30	5.87	13.02	37.74	17.39	0.08	0.04	0.00	1.35	0.00	0.02	0.00		Roads	Grass	Sky	Tree
GV_1427	1.56	32.94	1.15	20.21	9.50	29.64	1.26	0.05	1.38	0.00	0.00	2.29	0.00		Grass	Sky	Tree	Roads
GV_1428	5.66	73.64	3.80	14.77	0.04	1.74	0.00	0.00	0.00	0.35	0.00	0.00	0.00		Grass	Tree	Background	Building
GV_1429	8.94	2.57	15.39	7.43	32.79	23.71	1.91	0.45	0.00	6.60	0.00	0.20	0.00		Roads	Sky	Building	Background
GV_1430	6.10	1.23	28.13	9.72	34.90	5.84	0.00	0.00	0.00	14.07	0.00	0.00	0.00		Roads	Building	Sidewalk	Tree
GV_1431	6.75	7.82	0.88	0.06	2.22	0.00	1.32	0.00	0.00	80.95	0.00	0.00	0.00		Sidewalk	Grass	Background	Roads
GV_1432	9.32	7.37	0.79	1.38	15.12	0.06	0.83	0.17	0.02	64.92	0.00	0.03	0.00		Sidewalk	Roads	Background	Grass
GV_1433	0.71	47.64	0.19	29.03	1.26	8.16	8.69	0.06	0.00	2.67	0.00	1.58	0.00		Grass	Tree	Soil	Sky
GV_1434	3.95	26.22	1.07	48.41	0.56	4.41	11.30	0.02	0.00	0.83	0.00	3.23	0.00		Tree	Grass	Soil	Sky
GV_1435	6.51	37.42	0.09	17.22	9.52	2.00	21.14	2.23	0.16	1.10	0.05	2.55	0.00		Grass	Soil	Tree	Roads
GV_1436	0.61	84.30	0.02	4.38	1.98	2.71	2.85	0.00	0.01	0.59	0.00	2.32	0.23		Grass	Tree	Soil	Sky
GV_1437	1.18	9.27	38.01	13.29	10.10	9.28	0.01	0.00	0.00	18.84	0.00	0.02	0.00		Building	Sidewalk	Tree	Roads
GV_1438	3.06	66.24	0.00	20.35	0.01	4.17	3.38	0.01	0.00	0.00	0.00	2.78	0.00		Grass	Tree	Sky	Soil
GV_1439	3.63	57.82	0.17	0.86	21.37	6.87	2.89	0.02	0.70	3.20	1.34	1.13	0.00		Grass	Roads	Sky	Background
GV_1440	3.20	60.25	1.62	7.29	6.87	13.44	4.97	0.74	0.00	0.78	0.03	0.83	0.00		Grass	Sky	Tree	Roads
GV_1441	6.83	8.71	55.07	8.72	3.81	9.47	0.06	2.24	0.00	5.09	0.00	0.00	0.00		Building	Sky	Tree	Grass
GV_1442	8.12	51.62	12.78	1.64	4.97	1.46	1.77	0.01	0.20	16.44	0.91	0.08	0.00		Grass	Sidewalk	Building	Background
GV_1443	8.53	11.22	23.77	3.47	47.17	0.89	0.41	0.24	1.06	3.18	0.00	0.05	0.00		Roads	Building	Grass	Background
GV_1444	2.02	48.96	0.50	27.06	0.55	5.81	4.32	0.03	0.02	2.45	0.30	7.92	0.06		Grass	Tree	Gravel	Sky
GV_1445	6.09	36.24	9.35	32.83	0.89	10.33	2.07	0.69	0.00	0.99	0.07	0.44	0.00		Grass	Tree	Sky	Building
GV_1446	13.29	4.74	14.88	27.43	27.44	3.57	1.87	0.01	0.00	5.22	0.00	1.54	0.00		Roads	Tree	Building	Background
GV_1447	4.00	18.05	8.88	40.08	12.34	4.74	0.00	0.00	0.00	10.01	0.00	1.88	0.00		Tree	Grass	Roads	Sidewalk
GV_1448	1.59	43.63	0.22	11.37	7.85	29.01	1.99	0.13	1.26	0.03	0.69	2.24	0.00		Grass	Sky	Tree	Roads
GV_1449	0.20	36.51	0.03	9.57	0.13	48.43	4.30	0.01	0.00	0.31	0.00	0.49	0.00		Sky	Grass	Tree	Soil
GV_1450	0.21	15.07	0.12	69.89	0.01	0.00	10.27	0.61	0.00	0.00	0.01	3.81	0.00		Tree	Grass	Soil	Gravel
GV_1451	2.07	88.27	0.00	7.17	0.04	0.08	1.66	0.00	0.12	0.03	0.00	0.57	0.00		Grass	Tree	Background	Soil
GV_1452	2.24	51.08	0.00	36.02	0.00	0.00	9.04	0.00	0.00	0.00	0.00	1.61	0.00		Grass	Tree	Soil	Background
GV_1453	1.07	86.58	0.00	10.02	0.00	2.28	0.01	0.00	0.00	0.00	0.00	0.00	0.04		Grass	Tree	Sky	Background
GV_1454	0.14	72.54	0.00	20.09	0.00	5.73	1.48	0.02	0.00	0.00	0.00	0.00	0.00		Grass	Tree	Sky	Soil
GV_1455	0.92	77.58	0.00	16.26	0.00	4.85	0.17	0.00	0.00	0.00	0.00	0.22	0.00		Grass	Tree	Sky	Background
GV_1456	0.84	7.33	0.07	77.25	0.03	1.01	8.98	0.04	0.00	0.00	0.00	4.45	0.00		Tree	Soil	Grass	Gravel
GV_1457	1.11	22.17	0.00	71.50	0.30	0.61	0.74	1.05	0.02	0.01	0.02	2.48	0.00		Tree	Grass	Gravel	Background
GV_1458	0.54	52.46	0.04	14.25	0.16	21.97	6.33	0.03	0.09	3.01	0.10	0.86	0.17		Grass	Sky	Tree	Soil
GV_1459	0.36	50.57	0.06	9.53	0.00	23.25	15.32	0.04	0.51	0.00	0.00	0.00	0.35		Grass	Sky	Soil	Tree
GV_1460	0.34	66.19	0.03	6.71	0.00	24.76	1.50	0.07	0.01	0.00	0.00	0.00	0.40		Grass	Sky	Tree	Soil
GV_1461	1.12	81.69	0.72	6.01	1.58	0.04	3.04	0.02	2.13	1.50	1.80	0.35	0.00		Grass	Tree	Soil	Sand
GV_1462	1.36	73.82	2.64	8.81	0.40	12.80	0.00	0.00	0.00	0.18	0.00	0.00	0.00		Grass	Sky	Tree	Building
GV_1463	3.39	43.81	1.88	49.70	0.00	0.11	1.10	0.01	0.00	0.00	0.00	0.01	0.00		Tree	Grass	Background	Building
GV_1464	6.80	0.37	44.79	2.71	29.57	4.15	0.00	0.00	0.00	11.61	0.00	0.00	0.00		Building	Roads	Sidewalk	Background
GV_1465	4.28	22.16	19.47	11.67	13.01	2.97	21.59	1.68	0.00	1.67	0.02	1.48	0.00		Grass	Soil	Building	Roads
GV_1466	4.69	28.53	4.49	19.52	5.40	4.82	12.73	0.04	4.78	4.66	0.00	9.66	0.67		Grass	Tree	Soil	Gravel
GV_1467	2.02	74.97	0.24	12.07	0.00	9.40	0.11	0.01	0.00	0.02	0.02	0.00	1.15		Grass	Tree	Sky	Background
GV_1468	6.32	12.70	0.19	51.37	5.48	1.53	3.56	0.68	0.82	13.28	0.00	4.05	0.00		Tree	Sidewalk	Grass	Background
GV_1469	1.05	32.83	4.09	54.34	0.09	0.60	6.74	0.00	0.00	0.10	0.00	0.14	0.00		Tree	Grass	Soil	Building
GV_1470	1.75	26.36	0.48	3.18	33.63	9.18	7.66	0.20	7.80	0.65	3.16	5.92	0.01		Roads	Grass	Sky	Sand
GV_1471	1.08	64.30	3.62	1.29	10.38	8.15	7.59	0.88	0.00	0.30	0.48	1.83	0.09		Grass	Roads	Sky	Soil
GV_1472	3.92	58.90	6.05	19.18	1.93	4.54	5.15	0.20	0.00	0.10	0.00	0.04	0.00		Grass	Tree	Building	Soil
GV_1473	19.38	7.16	14.63	10.35	25.01	13.81	0.15	1.09	0.00	7.74	0.00	0.68	0.00		Roads	Background	Building	Sky

GV_1474	1.46	14.18	0.38	17.59	38.83	3.96	13.07	0.10	0.07	6.52	0.00	2.69	1.16		Roads	Tree	Grass	Soil
GV_1475	5.79	3.73	11.01	16.65	29.35	6.30	0.02	0.07	1.29	24.45	0.00	0.51	0.83		Roads	Sidewalk	Tree	Building
GV_1476	1.64	23.15	4.26	38.40	20.32	9.46	0.83	0.07	0.00	0.41	0.01	1.45	0.00		Tree	Grass	Roads	Sky
GV_1477	0.02	75.70	0.00	2.39	12.61	6.76	0.81	0.20	0.06	0.69	0.39	0.34	0.01		Grass	Roads	Sky	Tree
GV_1478	0.88	20.15	0.00	3.48	14.19	9.75	3.89	14.24	16.17	0.19	11.34	5.66	0.05		Grass	Sand	Bare Rock	Roads
GV_1479	2.70	68.94	2.33	2.35	8.29	8.47	0.62	0.05	0.08	3.05	2.33	0.73	0.06		Grass	Sky	Roads	Sidewalk
GV_1480	1.03	68.80	1.81	15.81	2.93	4.85	0.17	0.00	0.00	3.69	0.00	0.89	0.01		Grass	Tree	Sky	Sidewalk
GV_1481	0.16	57.88	0.20	16.01	11.47	11.08	0.37	0.39	0.15	1.09	0.00	1.19	0.00		Grass	Tree	Roads	Sky
GV_1482	0.05	72.51	0.08	2.26	0.81	6.38	10.67	0.22	0.07	2.05	0.00	4.90	0.00		Grass	Soil	Sky	Gravel
GV_1483	2.10	40.53	0.73	10.24	12.87	10.64	4.48	0.96	10.65	0.14	2.12	4.35	0.19		Grass	Roads	Sand	Sky
GV_1484	3.32	21.76	23.55	11.22	13.29	9.96	0.02	0.25	0.00	15.34	0.09	1.18	0.01		Building	Grass	Sidewalk	Roads
GV_1485	0.48	42.20	0.18	44.53	1.05	8.01	0.32	0.00	0.00	0.13	0.02	3.08	0.00		Tree	Grass	Sky	Gravel
GV_1486	0.77	48.76	3.59	29.90	1.48	6.54	7.23	0.73	0.00	0.03	0.03	0.95	0.00		Grass	Tree	Soil	Sky
GV_1487	2.39	59.28	3.82	30.60	0.04	3.32	0.09	0.00	0.00	0.46	0.00	0.00	0.00		Grass	Tree	Building	Sky
GV_1488	3.75	1.82	18.05	36.50	1.15	22.06	2.17	0.01	0.00	10.17	0.00	4.32	0.00		Tree	Sky	Building	Sidewalk
GV_1489	0.39	49.20	0.08	12.09	0.31	29.75	6.63	0.00	0.26	0.11	0.00	1.17	0.00		Grass	Sky	Tree	Soil
GV_1490	2.23	17.45	3.85	6.13	7.58	35.97	22.08	0.12	0.00	2.01	0.01	1.69	0.87		Sky	Soil	Grass	Roads
GV_1491	0.23	16.30	1.93	4.53	16.83	33.91	23.27	0.81	0.00	0.51	0.00	1.45	0.25		Sky	Soil	Roads	Grass
GV_1492	1.17	25.94	0.21	5.08	0.77	30.07	35.49	0.35	0.51	0.15	0.00	0.08	0.19		Soil	Sky	Grass	Tree
GV_1493	1.36	13.13	0.00	55.97	2.96	0.07	20.97	0.65	4.43	0.00	0.00	0.46	0.00		Tree	Soil	Grass	Sand
GV_1494	2.58	17.42	0.00	73.67	0.00	0.35	4.80	0.40	0.17	0.00	0.00	0.61	0.00		Tree	Grass	Soil	Background
GV_1495	1.84	47.88	0.00	33.71	0.25	0.28	14.15	1.02	0.00	0.00	0.00	0.87	0.00		Grass	Tree	Soil	Background
GV_1496	1.94	43.75	4.66	24.06	0.11	20.30	0.64	0.02	0.00	3.88	0.00	0.52	0.12		Grass	Tree	Sky	Building
GV_1497	0.02	90.12	0.01	9.85	0.00	0.00	0.00	0.00	0.00	0.00	0.00	0.00	0.00		Grass	Tree	Background	Building
GV_1498	0.32	47.32	0.00	9.63	0.00	37.79	0.44	0.00	0.00	0.00	0.00	4.51	0.00		Grass	Sky	Tree	Gravel
GV_1499	4.20	57.35	3.16	6.47	0.76	24.93	1.24	0.04	0.00	0.55	0.02	1.24	0.04		Grass	Sky	Tree	Background
GV_1500	1.94	54.44	3.16	37.15	0.11	2.76	0.00	0.00	0.00	0.44	0.00	0.00	0.00		Grass	Tree	Building	Sky
GV_1501	3.29	4.83	0.01	18.99	3.05	3.73	7.92	19.92	12.82	17.58	0.32	0.02	7.52		Bare Rock	Tree	Sidewalk	Sand
GV_1502	9.91	35.09	2.62	6.65	0.18	34.22	8.07	0.02	0.00	0.50	0.00	2.73	0.01		Grass	Sky	Background	Soil
GV_1503	2.32	30.21	13.72	31.73	1.02	7.47	0.96	0.03	0.00	0.81	0.00	11.74	0.00		Tree	Grass	Building	Gravel
GV_1504	2.28	29.46	2.15	48.99	0.01	17.06	0.00	0.00	0.00	0.04	0.00	0.00	0.00		Tree	Grass	Sky	Background
GV_1505	1.68	20.32	9.27	17.56	29.66	6.10	0.05	0.14	0.00	15.22	0.00	0.00	0.00		Roads	Grass	Tree	Sidewalk
GV_1506	0.29	74.15	0.05	16.58	0.29	3.59	4.06	0.29	0.00	0.06	0.01	0.62	0.02		Grass	Tree	Soil	Sky
GV_1507	0.43	47.04	0.06	42.55	0.03	1.79	7.92	0.03	0.00	0.02	0.00	0.12	0.00		Grass	Tree	Soil	Sky
GV_1508	1.54	23.45	0.00	55.95	4.62	0.02	8.35	1.01	0.00	0.00	0.02	5.04	0.00		Tree	Grass	Soil	Gravel
GV_1509	6.44	2.36	8.17	3.29	29.79	0.55	19.06	0.05	11.51	17.22	0.00	1.57	0.00		Roads	Soil	Sidewalk	Sand
GV_1510	3.33	4.01	20.30	25.75	17.48	22.83	0.82	0.03	0.00	4.69	0.61	0.12	0.03		Tree	Sky	Building	Roads
GV_1511	4.44	20.41	8.49	17.29	18.50	25.40	0.05	0.42	1.12	2.64	0.92	0.29	0.05		Sky	Grass	Roads	Tree
GV_1512	6.86	33.13	4.54	15.48	8.41	24.35	0.83	4.01	0.00	1.98	0.16	0.00	0.25		Grass	Sky	Tree	Roads
GV_1513	0.63	46.86	0.00	0.89	12.98	9.47	0.74	8.44	1.80	0.00	17.21	0.97	0.00		Grass	Water	Roads	Sky
GV_1514	1.98	49.99	0.21	17.09	14.87	3.71	9.98	1.59	0.00	0.07	0.02	0.48	0.00		Grass	Tree	Roads	Soil
GV_1515	5.84	50.51	1.54	27.78	2.94	1.83	7.56	0.21	1.61	0.06	0.02	0.09	0.00		Grass	Tree	Soil	Background
GV_1516	1.31	55.99	0.01	12.09	1.07	1.00	27.50	0.16	0.23	0.00	0.04	0.59	0.00		Grass	Soil	Tree	Background
GV_1517	5.85	36.88	5.47	13.61	4.93	20.99	0.90	0.18	0.01	0.32	5.41	3.35	2.09		Grass	Sky	Tree	Background
GV_1518	1.27	67.07	3.63	0.00	2.21	22.11	2.97	0.07	0.00	0.15	0.03	0.00	0.48		Grass	Sky	Building	Soil
GV_1519	2.75	15.56	0.89	13.36	14.97	15.51	0.15	4.65	24.91	0.55	5.17	0.42	1.11		Sand	Grass	Sky	Roads
GV_1520	0.79	15.24	0.02	6.81	11.73	22.61	0.02	6.27	19.19	0.00	17.25	0.00	0.07		Sky	Sand	Water	Grass
GV_1521	0.29	17.74	0.09	3.87	17.91	17.86	0.33	5.59	15.85	0.00	20.46	0.02	0.00		Water	Roads	Sky	Grass
GV_1522	8.71	1.46	25.50	16.22	30.92	7.74	0.61	0.04	0.03	8.08	0.00	0.67	0.00		Roads	Building	Tree	Background
GV_1523	2.77	47.09	0.76	1.99	0.15	35.57	11.12	0.07	0.00	0.06	0.04	0.04	0.35		Grass	Sky	Soil	Background
GV_1524	4.76	4.00	13.89	0.95	33.04	4.14	4.13	0.16	26.44	6.21	0.00	2.28	0.00		Roads	Sand	Building	Sidewalk
GV_1525	2.37	62.97	0.47	13.91	6.32	9.30	1.10	0.81	0.00	1.65	0.00	0.67	0.44		Grass	Tree	Sky	Roads
GV_1526	8.70	9.58	23.43	0.56	2.48	16.96	12.34	16.12	1.92	5.87	0.39	1.42	0.24		Building	Sky	Bare Rock	Soil
GV_1527	0.69	34.23	0.69	13.87	11.11	33.83	0.77	0.02	0.00	0.00	0.01	4.75	0.03		Grass	Sky	Tree	Roads
GV_1528	0.50	34.04	0.03	4.53	3.85	55.42	1.54	0.07	0.00	0.00	0.00	0.00	0.02		Sky	Grass	Tree	Roads
GV_1529	0.87	70.08	3.70	17.58	0.08	7.36	0.00	0.00	0.00	0.33	0.00	0.00	0.00		Grass	Tree	Sky	Building
GV_1530	5.10	94.79	0.00	0.02	0.00	0.07	0.00	0.00	0.00	0.02	0.00	0.00	0.00		Grass	Background	Sky	Sidewalk
GV_1531	0.36	45.89	2.93	41.80	0.01	8.07	0.03	0.00	0.00	0.90	0.00	0.00	0.00		Grass	Tree	Sky	Building
GV_1532	1.63	42.18	0.20	54.82	0.05	0.80	0.30	0.00	0.00	0.01	0.00	0.00	0.00		Tree	Grass	Background	Sky

GV_1533	0.11	57.52	3.05	37.33	0.01	1.43	0.03	0.00	0.00	0.51	0.00	0.00	0.00		Grass	Tree	Building	Sky
GV_1534	1.87	3.59	0.43	13.44	0.20	16.25	64.23	0.00	0.00	0.00	0.00	0.00	0.00		Soil	Sky	Tree	Grass
GV_1535	6.87	10.03	10.02	17.93	22.75	23.40	0.00	4.05	0.18	0.21	1.06	0.07	3.43		Sky	Roads	Tree	Grass
GV_1536	3.86	3.14	9.04	15.26	59.70	3.97	1.52	0.19	0.62	1.56	1.10	0.03	0.02		Roads	Tree	Building	Sky
GV_1537	0.13	61.14	0.02	8.24	1.90	24.36	1.22	0.02	0.00	0.10	0.03	2.84	0.00		Grass	Sky	Tree	Gravel
GV_1538	2.91	40.79	0.12	27.97	0.02	25.86	0.11	1.69	0.20	0.00	0.27	0.02	0.03		Grass	Tree	Sky	Background
GV_1539	5.53	51.87	5.38	30.08	0.09	5.04	0.32	1.22	0.10	0.28	0.09	0.00	0.00		Grass	Tree	Background	Building
GV_1540	2.02	19.78	0.31	0.89	19.27	9.25	0.26	10.10	16.20	0.02	21.89	0.01	0.00		Water	Grass	Roads	Sand
GV_1541	2.37	14.10	1.55	13.28	22.32	10.62	25.03	0.27	1.14	0.26	8.27	0.78	0.01		Soil	Roads	Grass	Tree
GV_1542	1.47	49.30	16.12	9.36	17.56	4.40	1.42	0.03	0.00	0.28	0.00	0.06	0.00		Grass	Roads	Building	Tree
GV_1543	3.12	1.42	28.72	6.55	10.87	34.53	0.06	2.19	0.06	2.52	0.50	9.45	0.00		Sky	Building	Roads	Gravel
GV_1544	0.80	24.18	0.39	24.43	0.86	14.55	32.48	0.56	0.35	0.00	0.00	1.10	0.29		Soil	Tree	Grass	Sky
GV_1545	0.62	15.54	0.00	66.43	0.85	0.03	8.00	8.21	0.00	0.00	0.01	0.32	0.00		Tree	Grass	Bare Rock	Soil
GV_1546	0.51	55.40	0.00	7.30	0.00	29.96	6.46	0.00	0.00	0.00	0.00	0.00	0.37		Grass	Sky	Tree	Soil
GV_1547	12.45	5.76	10.40	10.14	40.76	14.13	2.91	0.22	0.00	2.88	0.00	0.11	0.23		Roads	Sky	Background	Building
GV_1548	2.99	37.65	18.68	12.38	8.90	10.05	1.71	0.05	0.00	4.79	0.01	2.77	0.03		Grass	Building	Tree	Sky
GV_1549	1.10	65.62	3.72	15.61	0.22	13.49	0.00	0.04	0.00	0.21	0.00	0.00	0.00		Grass	Tree	Sky	Building
GV_1550	0.14	42.22	0.14	34.84	0.42	1.92	2.85	8.39	0.02	0.00	0.93	8.13	0.01		Grass	Tree	Bare Rock	Gravel
GV_1551	3.38	31.46	0.24	49.83	3.44	0.74	8.34	0.10	0.00	1.17	0.00	1.29	0.00		Tree	Grass	Soil	Roads
GV_1552	6.30	37.08	0.07	11.49	12.16	18.26	1.21	0.01	0.02	0.00	0.03	13.28	0.10		Grass	Sky	Gravel	Roads
GV_1553	2.08	48.12	0.56	7.30	7.75	17.85	12.06	2.40	0.04	0.17	0.06	1.47	0.14		Grass	Sky	Soil	Roads
GV_1554	2.65	50.03	5.27	18.74	5.30	10.41	2.70	0.21	0.00	0.43	0.00	4.16	0.10		Grass	Tree	Sky	Roads
GV_1555	3.26	44.19	2.33	23.76	10.32	7.25	0.15	0.07	0.00	5.63	1.01	2.01	0.01		Grass	Tree	Roads	Sky
GV_1556	1.63	38.99	0.01	10.71	13.11	10.93	4.73	3.25	0.13	0.06	5.84	10.38	0.22		Grass	Roads	Sky	Tree
GV_1557	0.97	47.01	0.49	8.80	12.09	20.92	1.21	0.05	3.99	1.21	3.25	0.00	0.02		Grass	Sky	Roads	Tree
GV_1558	2.19	50.92	0.21	25.28	0.22	10.83	8.20	1.63	0.00	0.24	0.00	0.26	0.01		Grass	Tree	Sky	Soil
GV_1559	2.63	26.03	3.58	20.09	3.35	13.55	17.95	0.05	0.01	0.06	0.00	12.72	0.00		Grass	Tree	Soil	Sky
GV_1560	1.27	74.31	0.84	5.38	1.38	9.49	5.10	0.19	0.72	0.73	0.58	0.00	0.00		Grass	Sky	Tree	Soil
GV_1561	0.75	8.29	0.13	1.14	14.86	16.38	0.25	8.47	25.99	0.00	23.71	0.00	0.02		Sand	Water	Sky	Roads
GV_1562	1.81	4.96	0.18	6.86	14.41	23.08	0.07	7.52	24.33	0.07	16.68	0.02	0.00		Sand	Sky	Water	Roads
GV_1563	1.88	20.45	0.02	9.00	12.39	8.97	0.23	3.71	25.59	0.08	17.46	0.00	0.21		Sand	Grass	Water	Roads
GV_1564	0.32	30.75	1.98	1.66	0.81	44.73	0.06	6.62	0.53	0.00	7.73	3.59	1.23		Sky	Grass	Water	Bare Rock
GV_1565	0.09	75.68	0.12	10.64	1.49	9.94	0.09	0.00	1.28	0.00	0.23	0.42	0.02		Grass	Tree	Sky	Roads
GV_1566	0.15	87.42	0.01	7.09	0.00	3.50	0.75	0.52	0.00	0.00	0.00	0.37	0.20		Grass	Tree	Sky	Soil
GV_1567	0.29	19.09	0.02	2.66	20.35	12.45	0.07	3.76	21.86	0.00	18.75	0.02	0.68		Sand	Roads	Grass	Water
GV_1568	0.77	19.83	0.16	1.31	22.03	12.72	4.54	2.51	19.60	0.03	16.18	0.03	0.30		Roads	Grass	Sand	Water
GV_1569	1.08	64.31	0.68	0.09	0.93	12.57	11.33	6.54	0.00	0.16	0.01	2.01	0.29		Grass	Sky	Soil	Bare Rock
GV_1570	1.01	14.76	0.00	0.04	36.91	9.04	1.60	6.60	18.13	0.09	10.55	1.26	0.00		Roads	Sand	Grass	Water
GV_1571	1.30	26.32	0.00	0.51	17.25	13.28	0.63	18.05	11.21	0.00	11.27	0.17	0.00		Grass	Bare Rock	Roads	Sky
GV_1572	4.03	41.00	1.15	7.95	39.91	0.02	0.51	0.00	0.00	5.36	0.00	0.08	0.00		Grass	Roads	Tree	Sidewalk
GV_1573	6.98	41.72	16.53	3.45	2.52	0.38	6.64	1.57	0.00	20.07	0.02	0.12	0.00		Grass	Sidewalk	Building	Background
GV_1574	3.25	57.33	11.01	2.76	0.66	3.95	0.46	0.00	0.00	20.57	0.01	0.01	0.00		Grass	Sidewalk	Building	Sky
GV_1575	0.82	6.39	0.19	0.72	27.80	16.78	0.35	11.73	18.52	0.05	16.59	0.05	0.00		Roads	Sand	Sky	Water
GV_1576	1.01	15.44	0.04	0.34	21.65	9.66	2.30	16.49	16.30	0.00	16.41	0.28	0.07		Roads	Bare Rock	Water	Sand
GV_1577	3.07	51.08	0.81	34.93	0.95	6.06	0.17	1.96	0.01	0.84	0.00	0.12	0.00		Grass	Tree	Sky	Background
GV_1578	0.08	83.62	0.00	0.39	0.02	6.05	5.66	3.86	0.00	0.00	0.01	0.31	0.01		Grass	Sky	Soil	Bare Rock
GV_1579	0.73	32.66	0.12	0.79	10.58	12.60	1.24	10.02	15.05	0.00	16.19	0.00	0.00		Grass	Water	Sand	Sky
GV_1580	3.02	28.25	9.65	19.43	1.25	29.19	0.96	0.34	0.00	7.71	0.00	0.14	0.06		Sky	Grass	Tree	Building
GV_1581	3.08	21.39	5.55	6.31	11.58	16.95	26.53	1.56	0.08	0.86	0.28	5.79	0.03		Soil	Grass	Sky	Roads
GV_1582	2.19	52.30	1.57	2.72	0.46	26.11	14.47	0.02	0.00	0.08	0.04	0.01	0.02		Grass	Sky	Soil	Tree
GV_1583	1.32	56.53	7.82	9.71	0.77	22.92	0.65	0.04	0.00	0.18	0.00	0.06	0.01		Grass	Sky	Tree	Building
GV_1584	0.76	76.58	0.35	2.53	5.13	3.11	10.65	0.14	0.00	0.11	0.00	0.64	0.00		Grass	Soil	Roads	Sky
GV_1585	11.00	11.57	0.00	5.87	11.64	4.26	14.04	35.08	0.00	0.01	1.84	4.68	0.00		Bare Rock	Soil	Roads	Grass
GV_1586	0.49	39.42	0.01	1.58	19.76	6.41	6.56	20.07	0.13	0.03	4.77	0.77	0.00		Grass	Bare Rock	Roads	Soil
GV_1587	2.02	29.24	0.45	7.97	3.77	19.30	12.78	6.34	0.39	17.63	0.00	0.06	0.05		Grass	Sky	Sidewalk	Soil
GV_1588	0.47	60.08	0.24	0.52	0.11	23.07	15.23	0.00	0.20	0.02	0.00	0.00	0.07		Grass	Sky	Soil	Tree
GV_1589	0.09	45.15	0.25	15.23	1.57	33.97	1.52	0.00	0.07	0.25	0.00	1.90	0.00		Grass	Sky	Tree	Gravel
GV_1590	0.10	68.46	0.03	10.57	0.00	3.56	8.26	0.58	8.36	0.00	0.00	0.07	0.01		Grass	Tree	Sand	Soil
GV_1591	2.16	14.79	0.19	44.52	17.46	3.70	11.42	0.24	4.27	0.00	0.12	1.12	0.00		Tree	Roads	Grass	Soil

GV_1592	28.40	2.73	28.38	0.53	0.21	21.45	2.59	14.52	0.00	1.05	0.00	0.15	0.00		Background	Building	Sky	Bare Rock
GV_1593	1.68	20.31	0.12	32.10	1.63	43.32	0.08	0.03	0.00	0.36	0.00	0.08	0.29		Sky	Tree	Grass	Background
GV_1594	1.25	29.98	2.91	30.26	14.45	9.12	10.94	0.01	0.00	0.05	0.00	0.79	0.24		Tree	Grass	Roads	Soil
GV_1595	1.23	16.03	0.22	8.02	20.47	10.77	25.55	5.29	0.56	5.97	0.06	5.85	0.00		Soil	Roads	Grass	Sky
GV_1596	0.33	85.14	5.75	3.10	0.58	4.78	0.00	0.13	0.00	0.10	0.00	0.09	0.00		Grass	Building	Sky	Tree
GV_1597	2.27	63.07	4.79	7.73	0.45	4.24	12.17	0.67	0.12	3.13	0.03	0.12	1.21		Grass	Soil	Tree	Building
GV_1598	0.58	71.53	5.41	11.62	0.14	5.37	0.16	0.23	0.45	0.03	0.90	3.58	0.00		Grass	Tree	Building	Sky
GV_1599	0.73	11.85	0.00	84.88	0.00	0.14	2.29	0.01	0.00	0.00	0.03	0.07	0.00		Tree	Grass	Soil	Background
GV_1600	5.07	44.60	2.98	22.76	11.21	8.08	0.48	0.10	0.00	4.45	0.00	0.27	0.00		Grass	Tree	Roads	Sky
GV_1601	0.16	0.00	0.00	0.00	0.00	40.85	59.00	0.00	0.00	0.00	0.00	0.00	0.00		Soil	Sky	Background	Grass
GV_1602	0.63	67.79	0.24	10.00	0.03	0.75	19.56	0.06	0.00	0.66	0.00	0.28	0.00		Grass	Soil	Tree	Sky
GV_1603	0.43	95.54	0.04	0.24	0.05	0.00	3.56	0.15	0.00	0.00	0.00	0.00	0.00		Grass	Soil	Background	Tree
GV_1604	0.38	46.31	0.11	11.31	2.60	35.55	0.70	0.00	0.13	0.68	0.05	2.13	0.05		Grass	Sky	Tree	Roads
GV_1605	2.89	61.38	4.88	24.36	0.09	3.22	2.47	0.22	0.00	0.34	0.09	0.06	0.00		Grass	Tree	Building	Sky
GV_1606	1.31	23.43	20.40	9.37	34.13	8.93	0.00	0.00	0.00	2.43	0.00	0.00	0.00		Roads	Grass	Building	Tree
GV_1607	0.27	34.48	0.69	25.59	28.09	6.84	0.07	0.05	0.00	2.25	0.00	1.57	0.09		Grass	Roads	Tree	Sky
GV_1608	0.19	57.21	0.05	14.41	10.88	9.15	0.34	4.86	0.44	0.01	2.36	0.01	0.09		Grass	Tree	Roads	Sky
GV_1609	0.83	62.83	0.95	25.04	4.04	3.37	0.20	0.75	0.14	0.63	0.00	1.21	0.01		Grass	Tree	Roads	Sky
GV_1610	0.79	25.00	10.51	0.06	24.19	23.03	5.24	0.08	0.00	0.01	0.00	11.02	0.07		Grass	Roads	Sky	Gravel
GV_1611	9.85	23.83	18.53	13.26	4.44	6.06	0.32	0.00	0.00	21.72	0.01	1.98	0.00		Grass	Sidewalk	Building	Tree
GV_1612	0.95	24.74	0.02	17.42	23.73	11.49	6.98	1.37	3.04	0.32	0.04	6.74	3.16		Grass	Roads	Tree	Sky
GV_1613	1.45	66.78	0.26	6.90	5.82	6.91	0.44	6.83	1.26	3.05	0.04	0.27	0.00		Grass	Sky	Tree	Bare Rock
GV_1614	0.65	56.37	0.47	9.53	8.88	10.94	5.29	2.04	4.93	0.20	0.46	0.23	0.01		Grass	Sky	Tree	Roads
GV_1615	1.92	70.92	0.19	13.64	0.47	5.91	2.62	0.80	0.00	2.06	0.02	1.45	0.00		Grass	Tree	Sky	Soil
GV_1616	1.12	16.42	0.05	3.43	21.30	23.73	0.34	9.63	8.84	0.00	15.14	0.00	0.00		Sky	Roads	Grass	Water
GV_1617	1.81	59.12	0.25	32.22	0.23	1.28	1.34	1.28	0.00	1.32	0.06	1.08	0.00		Grass	Tree	Background	Soil
GV_1618	1.83	70.45	0.11	20.02	0.01	6.29	0.88	0.06	0.00	0.01	0.00	0.34	0.00		Grass	Tree	Sky	Background
GV_1619	2.24	16.90	13.37	6.10	38.54	21.92	0.00	0.02	0.00	0.56	0.00	0.01	0.33		Roads	Sky	Grass	Building
GV_1620	2.94	21.90	9.12	4.62	23.02	16.59	7.31	4.75	0.00	9.50	0.00	0.10	0.14		Roads	Grass	Sky	Sidewalk
GV_1621	0.03	42.10	0.13	15.80	29.99	10.28	0.05	0.16	0.00	0.01	0.00	1.38	0.06		Grass	Roads	Tree	Sky
GV_1622	2.12	64.37	0.39	20.54	0.64	2.83	7.56	0.00	0.00	0.53	0.00	1.03	0.00		Grass	Tree	Soil	Sky
GV_1623	2.42	41.54	0.30	21.43	11.06	8.36	7.48	0.22	0.09	6.45	0.00	0.67	0.00		Grass	Tree	Roads	Sky
GV_1624	3.39	17.85	7.13	4.86	37.94	11.16	11.14	1.08	0.00	1.16	0.00	3.84	0.47		Roads	Grass	Sky	Soil
GV_1625	3.03	9.69	18.94	5.06	30.55	12.36	0.23	0.62	0.00	19.08	0.00	0.24	0.20		Roads	Sidewalk	Building	Sky
GV_1626	0.01	74.09	0.71	13.93	0.00	11.26	0.00	0.00	0.00	0.00	0.00	0.00	0.00		Grass	Tree	Sky	Building
GV_1627	0.04	70.28	0.09	18.56	3.67	6.78	0.07	0.00	0.01	0.12	0.01	0.37	0.00		Grass	Tree	Sky	Roads
GV_1628	1.56	31.01	23.32	29.73	3.09	4.15	0.31	1.85	0.00	4.70	0.00	0.28	0.00		Grass	Tree	Building	Sidewalk
GV_1629	0.05	73.54	0.07	6.16	10.46	2.06	1.58	0.00	0.00	4.42	0.06	1.60	0.00		Grass	Roads	Tree	Sidewalk
GV_1630	0.58	36.83	9.34	23.24	20.33	6.75	0.00	0.00	0.00	2.78	0.02	0.12	0.00		Grass	Tree	Roads	Building
GV_1631	0.63	55.49	0.89	23.22	12.74	4.20	1.52	0.00	0.00	0.05	0.26	0.97	0.01		Grass	Tree	Roads	Sky
GV_1632	0.21	18.55	0.04	60.35	13.40	0.57	0.28	0.00	0.22	0.17	0.01	6.20	0.00		Tree	Grass	Roads	Gravel
GV_1633	1.58	58.08	2.60	20.78	1.07	9.28	5.16	0.00	0.01	0.64	0.00	0.82	0.00		Grass	Tree	Sky	Soil
GV_1634	0.10	46.57	0.05	2.20	21.61	29.15	0.04	0.00	0.00	0.00	0.00	0.27	0.00		Grass	Sky	Roads	Tree
GV_1635	1.94	17.03	11.60	4.87	18.06	21.45	11.86	0.41	10.82	1.28	0.15	0.54	0.00		Sky	Roads	Grass	Soil
GV_1636	2.41	10.36	28.45	34.67	2.70	15.59	0.20	0.22	0.00	5.30	0.04	0.06	0.00		Tree	Building	Sky	Grass
GV_1637	0.30	24.33	0.10	30.82	13.05	22.91	0.00	0.01	0.00	6.29	0.00	0.95	1.24		Tree	Grass	Sky	Roads
GV_1638	0.18	24.91	0.13	41.53	11.20	18.30	0.00	0.51	0.00	0.00	0.12	3.06	0.05		Tree	Grass	Sky	Roads
GV_1639	0.66	44.43	0.00	26.31	17.56	0.01	10.77	0.00	0.00	0.00	0.00	0.27	0.00		Grass	Tree	Roads	Soil
GV_1640	5.55	34.40	0.12	10.46	27.16	5.91	10.63	3.27	0.04	0.22	0.01	1.22	1.00		Grass	Roads	Soil	Tree
GV_1641	0.65	51.67	0.20	4.83	22.82	9.10	9.68	0.05	0.01	0.49	0.00	0.10	0.40		Grass	Roads	Soil	Sky
GV_1642	0.21	63.25	0.05	3.99	20.77	8.39	0.36	0.10	0.00	0.04	0.00	2.16	0.68		Grass	Roads	Sky	Tree
GV_1643	16.72	33.15	0.75	5.58	10.36	16.87	8.48	0.42	0.03	0.18	0.00	5.84	1.61		Grass	Sky	Background	Roads
GV_1644	0.07	44.34	0.03	5.36	31.27	13.04	0.76	0.25	0.00	0.01	0.00	3.51	1.35		Grass	Roads	Sky	Tree
GV_1645	0.49	39.39	1.17	23.58	22.40	4.38	6.00	0.20	0.00	0.63	0.01	1.74	0.01		Grass	Tree	Roads	Soil
GV_1646	0.63	5.85	0.07	0.13	0.15	40.73	52.32	0.12	0.00	0.00	0.00	0.00	0.00		Soil	Sky	Grass	Background
GV_1647	8.97	21.89	2.35	2.32	12.71	31.85	15.57	0.43	1.24	1.40	0.00	1.02	0.24		Sky	Grass	Soil	Roads
GV_1648	1.44	25.99	0.13	7.36	0.12	21.09	41.91	1.67	0.00	0.00	0.07	0.08	0.13		Soil	Grass	Sky	Tree
GV_1649	1.09	27.62	4.16	3.11	28.19	26.25	9.07	0.00	0.00	0.03	0.00	0.48	0.00		Roads	Grass	Sky	Soil
GV_1650	1.51	28.86	4.10	0.74	23.11	31.84	8.40	0.32	0.00	0.04	0.00	1.06	0.01		Sky	Grass	Roads	Soil

GV_1651	1.15	28.02	1.86	0.13	12.04	31.44	23.82	0.00	0.00	0.00	0.00	1.20	0.33		Sky	Grass	Soil	Roads
GV_1652	0.67	29.43	0.08	16.38	39.14	6.20	7.97	0.01	0.00	0.02	0.00	0.09	0.00		Roads	Grass	Tree	Soil
GV_1653	3.17	64.76	0.40	12.61	11.53	5.37	1.53	0.00	0.00	0.02	0.05	0.51	0.05		Grass	Tree	Roads	Sky
GV_1654	0.56	23.11	0.42	32.67	5.62	7.96	17.38	0.04	0.00	4.66	0.00	7.02	0.56		Tree	Grass	Soil	Sky
GV_1655	0.30	58.25	0.04	8.53	5.39	13.84	11.61	1.02	0.00	0.00	0.00	0.74	0.30		Grass	Sky	Soil	Tree
GV_1656	3.85	73.57	5.44	7.79	1.89	6.58	0.53	0.03	0.00	0.07	0.00	0.24	0.01		Grass	Tree	Sky	Building
GV_1657	3.48	36.93	8.94	7.31	7.69	26.82	5.63	0.11	0.02	1.93	0.00	0.26	0.86		Grass	Sky	Building	Roads
GV_1658	2.52	45.58	1.33	14.32	0.93	5.06	1.48	0.16	0.59	26.23	0.08	0.02	1.71		Grass	Sidewalk	Tree	Sky
GV_1659	0.99	30.13	0.54	42.88	9.23	4.11	7.65	0.13	0.01	0.36	0.01	3.96	0.00		Tree	Grass	Roads	Soil
GV_1660	2.47	28.67	16.07	23.16	1.11	2.49	1.45	0.84	2.24	20.54	0.00	0.96	0.00		Grass	Tree	Sidewalk	Building
GV_1661	3.40	61.17	10.95	15.67	0.07	3.24	0.50	0.86	0.04	1.23	0.25	2.51	0.10		Grass	Tree	Building	Background
GV_1662	2.06	44.47	0.06	26.43	3.53	18.94	1.62	0.00	0.00	0.61	0.19	2.08	0.00		Grass	Tree	Sky	Roads
GV_1663	0.23	48.60	0.06	12.62	1.97	28.90	4.84	0.00	0.02	0.37	0.00	2.39	0.00		Grass	Sky	Tree	Soil
GV_1664	30.79	2.91	25.39	4.23	19.23	5.23	1.71	0.70	0.00	4.35	0.00	5.45	0.00		Background	Building	Roads	Gravel
GV_1665	31.60	3.28	16.20	0.44	32.04	3.25	4.16	1.95	4.15	0.13	0.00	2.80	0.00		Roads	Background	Building	Soil
GV_1666	9.65	32.28	0.92	25.14	14.30	8.71	4.66	0.40	0.29	1.34	0.06	2.25	0.00		Grass	Tree	Roads	Background
GV_1667	41.41	0.38	16.85	1.88	19.29	7.11	0.44	0.45	0.00	9.15	0.00	3.05	0.00		Background	Roads	Building	Sidewalk
GV_1668	16.76	1.07	30.96	2.73	14.39	14.55	6.24	2.60	2.08	3.53	0.00	5.08	0.00		Building	Background	Sky	Roads
GV_1669	2.92	39.06	9.41	19.37	10.02	7.11	7.59	0.20	0.00	3.94	0.04	0.32	0.01		Grass	Tree	Roads	Building
GV_1670	0.21	0.05	0.04	0.00	0.00	40.07	48.29	11.32	0.00	0.00	0.03	0.00	0.00		Soil	Sky	Bare Rock	Background
GV_1671	0.50	64.37	10.88	7.65	0.39	15.56	0.03	0.00	0.00	0.53	0.04	0.00	0.04		Grass	Sky	Building	Tree
GV_1672	0.54	34.06	0.43	0.00	43.33	15.24	4.07	0.12	0.00	0.01	0.03	2.17	0.00		Roads	Grass	Sky	Soil
GV_1673	0.57	20.00	0.78	9.91	42.35	23.91	0.91	0.04	0.04	0.00	0.00	1.50	0.00		Roads	Sky	Grass	Tree
GV_1674	0.02	41.39	1.30	0.00	23.94	9.45	12.52	0.01	0.02	0.00	0.00	11.35	0.00		Grass	Roads	Soil	Gravel
GV_1675	0.00	24.31	0.00	0.00	51.83	12.43	3.62	0.00	0.00	0.00	0.00	7.82	0.00		Roads	Grass	Sky	Gravel
GV_1676	0.00	31.28	0.01	0.10	46.57	12.96	0.06	0.00	0.00	0.00	6.51	0.26	2.26		Roads	Grass	Sky	Water
GV_1677	1.95	21.07	16.87	7.00	21.15	13.21	0.19	1.78	0.00	13.01	0.00	3.77	0.00		Roads	Grass	Building	Sky
GV_1678	4.26	2.40	18.03	6.29	48.89	6.92	0.10	1.25	2.97	8.84	0.04	0.02	0.00		Roads	Building	Sidewalk	Sky
GV_1679	3.07	2.22	41.65	5.42	17.82	7.55	0.13	1.35	0.00	20.52	0.08	0.19	0.00		Building	Sidewalk	Roads	Sky
GV_1680	1.73	58.47	24.82	3.31	0.81	8.10	0.14	0.02	0.00	2.41	0.00	0.01	0.20		Grass	Building	Sky	Tree
GV_1681	1.59	52.02	2.38	9.31	1.45	9.36	11.35	0.35	3.22	0.00	0.45	8.45	0.08		Grass	Soil	Sky	Tree
GV_1682	0.17	41.18	0.10	9.83	9.80	5.29	7.92	13.63	0.00	0.00	4.56	7.41	0.11		Grass	Bare Rock	Tree	Roads
GV_1683	3.40	56.59	2.44	7.20	3.42	8.41	14.80	0.61	1.27	1.01	0.58	0.25	0.01		Grass	Soil	Sky	Tree
GV_1684	6.36	41.07	2.19	19.19	22.84	3.03	0.30	1.31	0.00	0.37	0.00	3.34	0.00		Grass	Roads	Tree	Background
GV_1685	1.18	72.31	0.12	3.54	0.03	12.05	0.27	3.85	1.33	0.00	0.23	5.06	0.03		Grass	Sky	Gravel	Bare Rock
GV_1686	5.20	52.87	1.80	9.22	3.62	13.05	9.64	0.00	0.18	0.16	0.09	4.01	0.15		Grass	Sky	Soil	Tree
GV_1687	0.43	37.91	3.04	11.32	25.12	13.73	1.10	1.50	2.76	0.00	0.49	2.59	0.00		Grass	Roads	Sky	Tree
GV_1688	0.41	26.60	0.11	5.55	16.35	6.28	3.15	17.16	0.04	0.06	16.93	7.35	0.00		Grass	Bare Rock	Water	Roads
GV_1689	0.76	19.37	0.03	13.84	27.61	9.91	9.68	7.13	0.97	0.00	9.14	1.51	0.05		Roads	Grass	Tree	Sky
GV_1690	0.21	70.43	0.08	16.29	1.92	10.10	0.01	0.24	0.00	0.05	0.09	0.58	0.00		Grass	Tree	Sky	Roads
GV_1691	1.15	39.60	0.35	15.29	19.49	15.86	4.24	0.02	0.00	0.24	0.00	3.61	0.16		Grass	Roads	Sky	Tree
GV_1692	0.90	49.21	4.90	7.78	13.22	19.07	0.76	0.05	0.00	0.59	0.00	3.40	0.12		Grass	Sky	Roads	Tree
GV_1693	3.03	11.36	9.77	20.64	23.21	8.77	11.20	0.10	0.03	11.53	0.00	0.04	0.32		Roads	Tree	Sidewalk	Grass
GV_1694	5.65	0.57	18.35	5.21	35.33	4.83	2.53	0.27	0.00	6.68	0.02	20.52	0.02		Roads	Gravel	Building	Sidewalk
GV_1695	0.32	83.47	5.16	5.50	0.00	5.53	0.00	0.00	0.00	0.01	0.00	0.00	0.02		Grass	Sky	Tree	Building
GV_1696	0.15	82.34	2.63	6.03	0.00	8.51	0.00	0.00	0.00	0.02	0.00	0.00	0.31		Grass	Sky	Tree	Building
GV_1697	5.17	10.63	20.65	8.85	28.18	8.46	0.08	0.02	0.00	17.46	0.00	0.50	0.00		Roads	Building	Sidewalk	Grass
GV_1698	0.96	20.91	15.79	10.22	22.21	7.18	0.25	0.18	0.00	22.28	0.00	0.01	0.00		Sidewalk	Roads	Grass	Building
GV_1699	15.11	45.13	4.98	5.06	2.09	3.25	19.96	0.00	1.10	3.25	0.00	0.07	0.00		Grass	Soil	Background	Tree
GV_1700	0.89	41.28	0.05	5.94	6.60	21.66	19.75	0.99	0.39	0.46	0.71	0.45	0.85		Grass	Sky	Soil	Roads
GV_1701	1.24	57.40	0.02	20.79	13.33	2.94	3.03	0.00	0.00	0.55	0.01	0.69	0.00		Grass	Tree	Roads	Soil
GV_1702	8.43	48.28	0.12	19.91	0.54	8.87	11.98	0.24	0.31	0.75	0.10	0.16	0.31		Grass	Tree	Soil	Sky
GV_1703	1.64	66.70	0.01	15.95	12.70	1.17	1.39	0.00	0.00	0.00	0.00	0.45	0.00		Grass	Tree	Roads	Background
GV_1704	1.77	40.18	0.02	14.40	19.78	10.96	0.40	0.17	0.00	1.46	7.41	1.69	1.77		Grass	Roads	Tree	Sky
GV_1705	1.00	41.72	2.83	7.01	11.81	31.68	2.33	0.12	0.00	0.36	0.08	0.00	1.04		Grass	Sky	Roads	Tree
GV_1706	4.45	60.56	0.25	4.66	2.78	4.57	12.33	3.01	0.00	0.77	0.00	6.25	0.34		Grass	Soil	Gravel	Tree
GV_1707	1.43	70.06	0.01	6.66	2.66	7.79	7.73	1.78	0.00	1.04	0.02	0.22	0.60		Grass	Sky	Soil	Tree
GV_1708	0.16	67.18	0.35	3.06	17.00	8.64	0.01	0.00	0.00	0.21	0.00	1.39	1.99		Grass	Roads	Sky	Tree
GV_1709	0.20	60.54	0.25	8.90	2.34	12.66	0.37	0.00	0.03	13.72	0.00	0.01	0.96		Grass	Sidewalk	Sky	Tree

GV_1710	0.16	75.36	0.01	1.07	12.06	2.03	7.16	0.02	0.00	0.00	0.01	0.92	1.21		Grass	Roads	Soil	Sky
GV_1711	0.06	68.03	0.06	11.42	2.98	12.12	0.21	0.04	0.09	2.68	0.00	0.00	2.31		Grass	Sky	Tree	Roads
GV_1712	2.38	55.22	0.40	11.16	3.93	14.94	0.28	0.03	0.00	10.91	0.02	0.00	0.75		Grass	Sky	Tree	Sidewalk
GV_1713	0.10	38.20	0.10	4.15	11.04	45.93	0.34	0.01	0.00	0.00	0.00	0.11	0.00		Sky	Grass	Roads	Tree
GV_1714	0.42	92.57	0.13	5.58	0.29	0.55	0.00	0.00	0.00	0.24	0.11	0.00	0.10		Grass	Tree	Sky	Background
GV_1715	3.24	48.96	3.09	17.78	14.42	4.81	1.58	0.16	0.06	2.28	0.02	3.57	0.04		Grass	Tree	Roads	Sky
GV_1716	3.90	22.74	8.44	2.99	31.85	13.00	0.18	0.85	0.00	15.57	0.00	0.28	0.20		Roads	Grass	Sidewalk	Sky
GV_1717	1.69	89.87	0.98	1.91	1.07	4.29	0.00	0.08	0.00	0.07	0.00	0.00	0.05		Grass	Sky	Tree	Background
GV_1718	1.27	60.31	1.98	16.29	7.12	8.57	2.45	0.03	0.00	0.62	0.08	1.28	0.00		Grass	Tree	Sky	Roads
GV_1719	2.03	34.99	0.24	13.14	0.08	3.25	1.23	9.34	1.72	0.00	29.74	4.23	0.00		Grass	Water	Tree	Bare Rock
GV_1720	0.74	39.52	0.57	24.99	17.47	3.45	10.17	0.06	0.24	0.01	0.03	2.76	0.00		Grass	Tree	Roads	Soil
GV_1721	9.72	33.26	1.61	5.34	36.88	7.76	2.37	0.19	0.00	0.86	0.00	1.47	0.53		Roads	Grass	Background	Sky
GV_1722	0.62	6.25	16.51	9.94	35.16	6.09	0.01	0.01	0.00	25.29	0.00	0.12	0.00		Roads	Sidewalk	Building	Tree
GV_1723	3.51	10.79	0.94	51.94	20.96	7.11	3.58	0.28	0.06	0.03	0.02	0.79	0.00		Tree	Roads	Grass	Sky
GV_1724	11.29	7.22	17.51	15.43	2.86	18.99	0.06	1.34	0.07	14.08	9.52	0.06	1.58		Sky	Building	Tree	Sidewalk
GV_1725	0.93	34.55	0.00	0.02	0.36	11.31	49.48	0.26	0.08	0.00	0.94	0.00	2.06		Soil	Grass	Sky	Blue Mountain
GV_1726	0.01	56.63	0.24	19.43	5.04	8.65	5.18	0.05	0.00	0.00	0.00	3.54	1.23		Grass	Tree	Sky	Soil
GV_1727	1.94	19.93	0.02	41.46	7.93	1.49	23.19	0.47	0.04	0.00	0.01	3.50	0.00		Tree	Soil	Grass	Roads
GV_1728	0.02	43.39	0.00	11.54	11.33	5.63	3.30	0.60	0.00	0.65	0.09	21.49	1.95		Grass	Gravel	Tree	Roads
GV_1729	0.00	74.17	0.11	18.95	0.02	4.20	0.17	0.00	0.00	0.01	0.00	2.00	0.36		Grass	Tree	Sky	Gravel
GV_1730	0.01	49.20	0.01	16.59	20.80	6.72	0.02	0.00	0.00	0.00	0.00	3.90	2.74		Grass	Roads	Tree	Sky
GV_1731	0.87	27.58	0.56	10.80	11.90	7.32	18.13	0.00	0.00	0.45	0.01	22.15	0.21		Grass	Gravel	Soil	Roads
GV_1732	0.26	17.97	0.04	13.54	18.49	11.33	16.57	0.05	0.00	1.14	0.00	20.27	0.34		Gravel	Roads	Grass	Soil
GV_1733	0.86	50.98	0.86	20.03	16.63	5.09	0.13	0.06	0.01	0.12	0.03	5.20	0.01		Grass	Tree	Roads	Gravel
GV_1734	0.43	51.97	0.31	8.14	8.16	5.98	15.24	0.29	0.00	0.12	0.32	9.01	0.03		Grass	Soil	Gravel	Roads
GV_1735	0.03	41.52	0.06	29.56	20.17	3.10	1.88	0.01	0.00	0.35	0.00	3.02	0.29		Grass	Tree	Roads	Sky
GV_1736	0.31	30.77	0.60	54.21	0.38	2.55	10.96	0.06	0.00	0.00	0.00	0.15	0.00		Tree	Grass	Soil	Sky
GV_1737	0.25	21.84	0.16	41.24	3.37	15.50	5.00	10.95	0.16	0.05	0.17	1.12	0.19		Tree	Grass	Sky	Bare Rock
GV_1738	0.25	37.34	0.27	48.39	9.46	1.35	1.46	0.17	0.00	1.00	0.00	0.30	0.00		Tree	Grass	Roads	Soil
GV_1739	0.16	31.96	0.01	49.95	3.39	2.88	11.43	0.05	0.00	0.00	0.00	0.18	0.00		Tree	Grass	Soil	Roads
GV_1740	0.04	40.84	0.03	46.54	0.40	5.59	5.80	0.00	0.00	0.00	0.00	0.76	0.00		Tree	Grass	Soil	Sky
GV_1741	1.91	26.06	0.15	41.84	3.87	6.73	15.99	1.55	0.00	0.00	0.00	0.60	1.32		Tree	Grass	Soil	Sky
GV_1742	1.70	40.05	14.67	16.07	1.53	7.24	0.01	0.00	0.00	18.70	0.00	0.03	0.00		Grass	Sidewalk	Tree	Building
GV_1743	6.62	6.52	23.91	6.08	13.66	2.32	10.61	0.08	4.05	25.06	0.03	1.05	0.00		Sidewalk	Building	Roads	Soil
GV_1744	5.09	2.51	20.83	3.42	34.20	8.99	6.23	0.91	3.79	11.49	0.00	2.40	0.16		Roads	Building	Sidewalk	Sky
GV_1745	0.89	76.50	2.29	11.24	0.03	8.84	0.00	0.00	0.00	0.13	0.00	0.00	0.07		Grass	Tree	Sky	Building
GV_1746	2.12	32.15	1.80	31.08	2.65	12.56	11.49	0.14	1.16	2.71	0.05	2.08	0.00		Grass	Tree	Sky	Soil
GV_1747	2.50	46.07	1.70	24.02	16.76	3.13	0.00	0.11	0.00	5.59	0.10	0.01	0.00		Grass	Tree	Roads	Sidewalk
GV_1748	1.55	53.52	1.52	26.85	11.58	2.20	0.00	0.00	0.00	2.40	0.25	0.01	0.12		Grass	Tree	Roads	Sidewalk
GV_1749	0.72	32.88	0.26	19.40	30.87	0.66	0.56	0.12	0.00	2.92	0.09	11.49	0.03		Grass	Roads	Tree	Gravel
GV_1750	4.51	47.39	1.68	18.99	8.86	5.63	3.11	0.06	0.01	7.02	0.03	1.95	0.77		Grass	Tree	Roads	Sidewalk
GV_1751	1.03	32.87	0.91	13.12	26.06	10.25	5.56	1.63	0.02	1.08	3.48	3.74	0.24		Grass	Roads	Tree	Sky
GV_1752	2.17	20.86	1.21	12.25	16.97	5.31	20.87	3.33	0.03	0.00	1.79	15.19	0.00		Soil	Grass	Roads	Gravel
GV_1753	11.05	42.05	1.39	19.25	16.04	4.78	0.09	0.00	0.00	0.26	0.00	4.92	0.17		Grass	Tree	Roads	Background
GV_1754	0.47	54.16	0.59	37.89	1.07	1.77	0.02	0.04	0.00	3.89	0.00	0.08	0.02		Grass	Tree	Sidewalk	Sky
GV_1755	0.67	53.50	0.01	4.24	7.23	3.23	0.93	4.01	0.76	0.20	22.79	1.93	0.51		Grass	Water	Roads	Tree
GV_1756	0.86	72.73	3.70	8.59	7.51	4.94	0.02	0.42	0.00	0.38	0.19	0.01	0.65		Grass	Tree	Roads	Sky
GV_1757	0.69	19.56	0.13	3.60	36.91	8.46	6.77	9.48	0.03	0.11	11.95	1.71	0.59		Roads	Grass	Water	Bare Rock
GV_1758	1.24	53.13	0.02	1.73	0.95	7.37	7.79	6.97	0.00	0.07	15.39	5.26	0.07		Grass	Water	Soil	Sky
GV_1759	0.20	60.45	0.20	2.93	0.86	9.38	3.01	0.21	0.02	0.09	17.50	4.92	0.23		Grass	Water	Sky	Gravel
GV_1760	0.19	61.74	0.03	7.63	7.15	7.71	0.50	2.41	0.00	0.00	9.84	2.71	0.07		Grass	Water	Sky	Tree
GV_1761	1.50	49.29	2.11	2.89	7.18	8.70	8.98	2.16	0.00	5.88	0.01	10.55	0.73		Grass	Gravel	Soil	Sky
GV_1762	0.15	35.75	0.00	14.05	25.70	5.77	4.56	1.95	0.00	0.00	3.41	8.27	0.38		Grass	Roads	Tree	Gravel
GV_1763	0.51	57.02	0.72	5.08	7.52	8.86	2.12	3.58	0.00	7.77	0.00	3.80	3.03		Grass	Sky	Sidewalk	Roads
GV_1764	7.18	7.62	15.16	10.94	45.24	9.50	0.08	0.14	0.00	3.80	0.00	0.33	0.00		Roads	Building	Tree	Sky
GV_1765	11.60	23.79	8.24	4.68	36.84	3.96	6.39	1.06	0.00	1.11	1.32	1.02	0.00		Roads	Grass	Background	Building
GV_1766	1.02	80.38	7.73	1.58	1.30	6.32	0.03	0.64	0.00	0.15	0.00	0.02	0.83		Grass	Building	Sky	Tree
GV_1767	0.75	43.36	0.01	0.26	26.65	7.39	11.13	3.40	0.00	0.09	0.00	6.12	0.83		Grass	Roads	Soil	Sky
GV_1768	3.24	53.30	0.04	0.54	2.86	5.11	29.38	2.40	0.52	0.09	0.00	1.41	1.10		Grass	Soil	Sky	Background

GV_1769	0.19	55.70	0.00	0.01	21.33	3.09	17.46	1.31	0.00	0.14	0.00	0.57	0.20		Grass	Roads	Soil	Sky
GV_1770	1.85	39.23	0.02	3.39	35.96	7.00	1.54	5.43	0.00	0.31	0.02	3.78	1.44		Grass	Roads	Sky	Bare Rock
GV_1771	0.23	41.08	0.13	0.50	32.74	6.61	7.83	6.63	0.00	0.10	0.00	2.37	1.79		Grass	Roads	Soil	Bare Rock
GV_1772	1.48	42.59	0.80	2.59	41.48	5.54	0.05	4.85	0.00	0.02	0.00	0.01	0.57		Grass	Roads	Sky	Bare Rock
GV_1773	1.07	53.10	0.08	0.57	5.78	4.78	12.76	2.07	0.02	0.03	0.00	18.34	1.40		Grass	Gravel	Soil	Roads
GV_1774	0.22	50.70	0.11	1.02	21.13	4.58	10.31	3.20	0.00	0.05	0.00	7.57	1.09		Grass	Roads	Soil	Gravel
GV_1775	0.17	58.96	0.01	0.01	29.88	2.38	0.38	0.62	0.00	0.03	0.00	6.69	0.87		Grass	Roads	Gravel	Sky
GV_1776	3.78	88.85	0.00	0.83	0.16	1.43	4.36	0.17	0.00	0.00	0.04	0.23	0.14		Grass	Soil	Background	Sky
GV_1777	1.93	54.12	0.04	6.28	0.95	3.71	29.44	2.12	0.00	0.00	0.04	0.45	0.93		Grass	Soil	Tree	Sky
GV_1778	0.16	51.72	0.00	0.63	7.92	2.76	22.51	4.44	0.20	0.03	0.13	8.19	1.31		Grass	Soil	Gravel	Roads
GV_1779	0.37	54.39	0.00	0.72	3.00	4.38	25.56	2.20	0.66	1.15	0.32	6.08	1.17		Grass	Soil	Gravel	Sky
GV_1780	3.71	25.37	0.00	6.99	9.38	2.31	43.91	0.55	0.00	0.01	6.82	0.70	0.26		Soil	Grass	Roads	Tree
GV_1781	0.70	53.01	0.01	2.23	5.86	5.07	22.76	1.22	1.90	3.58	0.00	3.00	0.67		Grass	Soil	Roads	Sky
GV_1782	1.45	29.87	0.99	12.18	23.53	12.71	11.47	0.00	0.00	0.96	0.00	5.47	1.37		Grass	Roads	Sky	Tree
GV_1783	1.87	9.96	18.34	6.12	56.96	5.45	0.12	0.00	0.00	0.19	0.00	0.93	0.07		Roads	Building	Grass	Tree
GV_1784	8.27	39.75	21.13	13.50	5.07	3.57	0.06	0.02	0.00	1.16	0.00	7.47	0.00		Grass	Building	Tree	Background
GV_1785	1.34	72.11	5.54	6.07	0.10	13.16	0.58	0.00	0.00	0.01	0.09	0.01	1.00		Grass	Sky	Tree	Building
GV_1786	0.95	67.84	0.11	16.21	0.03	11.75	2.40	0.00	0.00	0.04	0.00	0.11	0.57		Grass	Tree	Sky	Soil
GV_1787	1.28	54.57	8.65	7.17	0.66	15.54	10.63	0.00	0.00	0.46	0.01	0.00	1.03		Grass	Sky	Soil	Building
GV_1788	2.77	21.89	5.42	10.30	37.34	17.35	0.05	0.37	0.00	0.39	0.00	3.98	0.12		Roads	Grass	Sky	Tree
GV_1789	0.09	66.27	0.80	18.26	0.08	8.71	0.71	0.03	0.00	0.09	0.01	4.45	0.52		Grass	Tree	Sky	Gravel
GV_1790	2.93	2.96	7.46	5.12	74.13	7.04	0.01	0.29	0.00	0.05	0.00	0.00	0.00		Roads	Building	Sky	Tree
GV_1791	7.19	2.23	9.64	9.93	60.06	8.99	0.00	0.01	0.00	1.95	0.00	0.00	0.00		Roads	Tree	Building	Sky
GV_1792	3.16	66.42	4.92	21.05	0.68	2.18	0.95	0.00	0.00	0.10	0.01	0.54	0.00		Grass	Tree	Building	Background
GV_1793	2.99	9.50	20.17	11.05	35.91	7.10	0.01	0.06	0.00	13.17	0.00	0.00	0.04		Roads	Building	Sidewalk	Tree
GV_1794	3.46	1.06	7.92	12.07	68.95	6.46	0.00	0.00	0.00	0.08	0.00	0.00	0.00		Roads	Tree	Building	Sky
GV_1795	7.40	40.05	10.37	15.97	6.05	9.85	0.38	1.37	0.00	8.20	0.02	0.35	0.00		Grass	Tree	Building	Sky
GV_1796	1.54	63.65	2.46	27.58	0.44	3.58	0.23	0.02	0.00	0.51	0.00	0.00	0.00		Grass	Tree	Sky	Building
GV_1797	3.21	17.87	2.28	24.61	33.11	6.01	0.78	0.03	0.00	0.58	0.00	11.53	0.00		Roads	Tree	Grass	Gravel
GV_1798	5.78	25.90	5.71	26.60	27.58	4.13	0.06	0.02	0.00	4.17	0.00	0.04	0.00		Roads	Tree	Grass	Background
GV_1799	4.18	22.46	20.67	12.88	20.47	6.71	0.42	0.02	0.00	6.70	0.00	5.48	0.00		Grass	Building	Roads	Tree
GV_1800	5.23	39.88	14.04	7.51	20.78	12.11	0.06	0.00	0.00	0.06	0.01	0.34	0.01		Grass	Roads	Building	Sky
GV_1801	4.09	1.05	5.56	8.28	71.78	8.96	0.07	0.00	0.00	0.17	0.00	0.00	0.05		Roads	Sky	Tree	Building
GV_1802	3.91	3.83	17.75	13.99	55.15	5.32	0.01	0.00	0.00	0.02	0.00	0.01	0.00		Roads	Building	Tree	Sky
GV_1803	1.25	68.26	6.13	3.24	3.33	1.28	0.53	0.00	0.00	15.89	0.00	0.09	0.00		Grass	Sidewalk	Building	Roads
GV_1804	2.46	31.33	19.27	11.83	6.44	5.43	0.02	0.00	0.00	23.19	0.00	0.03	0.00		Grass	Sidewalk	Building	Tree
GV_1805	3.06	30.01	21.01	10.83	0.32	5.27	0.00	0.00	1.12	28.37	0.00	0.00	0.00		Grass	Sidewalk	Building	Tree
GV_1806	1.96	53.16	18.92	13.92	0.52	6.67	0.09	0.31	0.00	4.35	0.00	0.06	0.05		Grass	Building	Tree	Sky
GV_1807	8.61	27.20	16.06	19.20	12.77	5.08	0.07	0.01	0.00	10.85	0.00	0.15	0.00		Grass	Tree	Building	Roads
GV_1808	1.87	12.24	24.32	26.01	8.73	8.19	7.93	0.92	0.00	9.75	0.00	0.04	0.00		Tree	Building	Grass	Sidewalk
GV_1809	0.54	11.78	35.36	22.75	5.28	5.02	11.14	0.00	0.00	8.06	0.00	0.07	0.00		Building	Tree	Grass	Soil
GV_1810	0.82	42.27	9.73	26.43	1.70	2.68	2.17	1.66	0.83	11.59	0.00	0.11	0.00		Grass	Tree	Sidewalk	Building
GV_1811	1.16	32.70	3.04	41.77	10.84	5.44	0.02	0.00	0.00	4.63	0.00	0.40	0.00		Tree	Grass	Roads	Sky
GV_1812	9.86	3.79	25.10	15.66	15.80	0.54	6.44	0.02	0.46	21.85	0.00	0.46	0.00		Building	Sidewalk	Roads	Tree
GV_1813	0.30	54.43	10.35	32.25	0.01	1.59	0.07	0.00	0.00	1.01	0.00	0.00	0.00		Grass	Tree	Building	Sky
GV_1814	3.45	30.31	3.94	45.71	2.52	6.63	1.80	0.05	0.00	0.19	0.10	4.72	0.57		Tree	Grass	Sky	Gravel
GV_1815	12.36	12.82	4.28	31.88	2.00	4.84	4.46	0.31	0.00	24.65	0.00	2.41	0.00		Tree	Sidewalk	Grass	Background
GV_1816	3.56	14.47	13.60	18.58	12.97	6.71	3.95	0.01	0.00	25.71	0.06	0.39	0.00		Sidewalk	Tree	Grass	Building
GV_1817	2.55	18.14	22.17	8.03	31.94	4.37	0.01	0.05	0.15	7.58	3.18	1.84	0.00		Roads	Building	Grass	Tree
GV_1818	2.63	26.26	35.06	11.75	2.51	8.74	0.13	0.09	0.00	12.80	0.00	0.03	0.00		Building	Grass	Sidewalk	Tree
GV_1819	2.70	54.30	16.55	10.77	2.11	6.84	0.31	0.82	0.00	2.39	0.00	3.22	0.00		Grass	Building	Tree	Sky
GV_1820	2.41	4.66	52.36	4.51	13.86	5.78	0.00	0.00	0.00	16.38	0.00	0.04	0.00		Building	Sidewalk	Roads	Sky
GV_1821	0.62	4.96	19.76	13.94	35.51	3.60	0.00	0.06	0.00	21.54	0.00	0.01	0.00		Roads	Sidewalk	Building	Tree
GV_1822	4.77	1.89	31.33	8.92	23.03	7.81	0.00	0.00	0.00	22.12	0.00	0.00	0.14		Building	Roads	Sidewalk	Tree
GV_1823	8.05	19.75	8.13	14.70	29.95	5.03	0.05	0.00	0.00	14.01	0.00	0.33	0.00		Roads	Grass	Tree	Sidewalk
GV_1824	2.19	0.98	8.56	4.95	76.98	6.13	0.01	0.00	0.00	0.19	0.00	0.00	0.01		Roads	Building	Sky	Tree
GV_1825	2.31	60.79	10.12	5.96	0.06	1.94	1.27	0.00	0.00	17.50	0.00	0.04	0.00		Grass	Sidewalk	Building	Tree
GV_1826	8.86	43.99	11.35	15.77	1.56	4.65	0.91	0.17	0.00	0.23	0.00	12.52	0.00		Grass	Tree	Gravel	Building
GV_1827	3.66	64.16	16.30	9.94	0.01	5.46	0.25	0.01	0.00	0.05	0.00	0.15	0.00		Grass	Building	Tree	Sky

GV_1828	3.95	16.45	26.42	12.78	16.81	2.55	0.04	0.00	0.00	20.95	0.00	0.06	0.00		Building	Sidewalk	Roads	Grass
GV_1829	0.63	29.01	11.58	26.10	16.46	1.30	0.01	0.00	0.00	14.81	0.00	0.09	0.00		Grass	Tree	Roads	Sidewalk
GV_1830	0.96	35.70	10.20	22.24	2.14	6.00	0.32	7.21	0.00	15.13	0.00	0.11	0.00		Grass	Tree	Sidewalk	Building
GV_1831	1.28	32.16	16.03	25.49	10.49	1.72	1.51	0.13	0.10	11.09	0.01	0.02	0.00		Grass	Tree	Building	Sidewalk
GV_1832	7.12	3.68	32.40	8.93	18.45	11.88	0.01	0.00	0.07	17.01	0.00	0.45	0.00		Building	Roads	Sidewalk	Sky
GV_1833	7.48	7.54	24.60	4.26	28.97	9.82	4.56	0.88	0.00	11.90	0.00	0.00	0.00		Roads	Building	Sidewalk	Sky
GV_1834	2.71	29.33	14.52	7.70	23.80	5.27	4.90	0.00	0.00	4.68	0.00	7.09	0.00		Grass	Roads	Building	Tree
GV_1835	1.35	26.12	4.42	20.79	27.41	5.53	5.20	0.01	0.07	8.67	0.00	0.42	0.01		Roads	Grass	Tree	Sidewalk
GV_1836	2.03	36.15	11.38	9.55	12.00	4.40	0.00	0.00	0.00	24.49	0.00	0.00	0.00		Grass	Sidewalk	Roads	Building
GV_1837	3.41	3.53	24.73	11.70	24.69	6.19	0.00	0.02	0.00	25.70	0.00	0.02	0.00		Sidewalk	Building	Roads	Tree
GV_1838	2.52	27.17	4.92	19.54	29.92	4.01	5.61	0.69	0.04	4.36	1.19	0.01	0.02		Roads	Grass	Tree	Soil
GV_1839	1.26	22.82	9.48	15.44	33.02	3.95	0.86	0.00	0.00	9.32	0.00	3.85	0.00		Roads	Grass	Tree	Building
GV_1840	2.27	4.05	8.42	12.02	42.19	5.66	0.07	0.09	0.00	22.82	0.00	2.40	0.00		Roads	Sidewalk	Tree	Building
GV_1841	0.48	43.94	20.83	2.45	6.38	11.17	0.30	0.00	0.00	13.29	0.00	1.16	0.01		Grass	Building	Sidewalk	Sky
GV_1842	5.76	49.52	7.24	4.86	4.61	9.40	0.00	0.01	0.00	18.46	0.00	0.01	0.12		Grass	Sidewalk	Sky	Building
GV_1843	1.51	33.41	14.28	13.61	12.18	4.18	0.00	0.00	0.00	20.78	0.00	0.06	0.00		Grass	Sidewalk	Building	Tree
GV_1844	3.64	27.77	10.12	4.86	28.18	12.38	6.31	0.00	0.00	5.84	0.00	0.89	0.01		Roads	Grass	Sky	Building
GV_1845	8.55	15.80	17.13	11.45	28.41	9.40	0.70	0.14	0.00	7.11	0.00	0.99	0.31		Roads	Building	Grass	Tree
GV_1846	2.01	24.70	10.48	2.36	28.92	15.02	6.92	0.02	0.00	6.83	0.00	1.87	0.87		Roads	Grass	Sky	Building
GV_1847	3.54	3.88	27.26	13.26	28.56	3.73	0.12	0.01	0.00	19.58	0.00	0.05	0.00		Roads	Building	Sidewalk	Tree
GV_1848	1.05	33.94	1.24	6.26	37.89	16.34	0.39	0.00	0.00	0.02	0.00	2.27	0.60		Roads	Grass	Sky	Tree
GV_1849	0.47	43.84	0.18	11.56	1.16	36.59	3.99	0.01	0.26	0.91	0.00	1.02	0.03		Grass	Sky	Tree	Soil
GV_1850	9.03	25.16	1.68	45.44	0.72	0.01	7.06	0.45	0.00	9.87	0.00	0.56	0.00		Tree	Grass	Sidewalk	Background
GV_1851	1.11	58.61	0.47	14.45	1.32	7.62	4.24	0.08	0.01	0.00	0.02	11.85	0.20		Grass	Tree	Gravel	Sky
GV_1852	2.13	57.88	0.54	15.72	0.14	6.16	0.18	0.00	0.02	1.44	0.00	15.38	0.41		Grass	Tree	Gravel	Sky
GV_1853	0.72	49.67	0.59	16.64	2.34	3.50	0.12	0.00	0.00	0.00	0.04	26.26	0.10		Grass	Gravel	Tree	Sky
GV_1854	0.65	50.00	0.24	13.44	0.30	31.78	0.99	0.00	0.17	0.21	0.02	2.21	0.00		Grass	Sky	Tree	Gravel
GV_1855	1.88	45.05	0.03	2.22	27.10	0.08	13.76	0.39	5.95	1.82	1.25	0.45	0.00		Grass	Roads	Soil	Sand
GV_1856	0.62	62.09	0.02	0.24	4.03	0.10	32.23	0.59	0.00	0.03	0.04	0.01	0.00		Grass	Soil	Roads	Background
GV_1857	2.91	87.67	3.13	0.53	0.55	2.62	1.18	0.13	0.23	0.55	0.46	0.05	0.00		Grass	Building	Background	Sky
GV_1858	0.37	27.38	0.07	0.00	1.63	33.05	34.82	0.00	1.83	0.12	0.01	0.54	0.16		Soil	Sky	Grass	Sand
GV_1859	0.60	8.16	0.00	0.00	1.15	20.49	43.68	21.83	0.51	3.57	0.01	0.00	0.00		Soil	Bare Rock	Sky	Grass
GV_1860	4.45	64.61	0.06	5.29	0.01	25.27	0.11	0.01	0.19	0.00	0.00	0.00	0.00		Grass	Sky	Tree	Background
GV_1861	2.31	26.21	15.55	6.03	34.78	14.35	0.59	0.00	0.00	0.09	0.00	0.08	0.00		Roads	Grass	Building	Sky
GV_1862	3.78	8.07	15.60	18.44	28.34	7.20	3.53	0.06	0.00	14.50	0.05	0.43	0.00		Roads	Tree	Building	Sidewalk
GV_1863	5.25	9.89	33.68	12.74	13.74	7.19	0.23	0.01	0.09	12.86	0.00	4.33	0.00		Building	Roads	Sidewalk	Tree
GV_1864	3.71	61.84	0.39	17.35	0.01	8.34	7.13	0.03	0.00	0.94	0.01	0.22	0.03		Grass	Tree	Sky	Soil
GV_1865	1.21	16.89	0.25	36.56	3.20	1.53	18.04	9.01	3.02	5.94	1.02	3.34	0.01		Tree	Soil	Grass	Bare Rock
GV_1866	0.66	38.15	0.39	18.43	18.82	5.08	0.94	0.44	0.05	15.25	0.00	1.46	0.34		Grass	Roads	Tree	Sidewalk
GV_1867	6.85	10.57	17.08	21.11	25.72	9.86	1.55	1.18	0.67	4.27	0.10	0.81	0.22		Roads	Tree	Building	Grass
GV_1868	7.30	12.54	15.57	21.87	17.15	8.05	1.57	4.81	0.64	6.45	1.88	1.04	1.14		Tree	Roads	Building	Grass
GV_1869	1.46	42.65	1.72	26.29	4.62	10.16	7.18	0.18	0.00	0.55	0.28	4.60	0.32		Grass	Tree	Sky	Soil
GV_1870	1.96	17.46	1.50	16.23	3.97	5.57	20.66	19.73	0.26	0.02	0.62	11.98	0.03		Soil	Bare Rock	Grass	Tree
GV_1871	1.06	27.40	3.16	21.03	13.02	7.40	19.18	3.47	0.61	0.79	1.38	1.50	0.00		Grass	Tree	Soil	Roads
GV_1872	0.41	70.94	1.61	20.33	0.20	6.21	0.21	0.00	0.00	0.09	0.00	0.00	0.00		Grass	Tree	Sky	Building
GV_1873	3.15	45.73	0.67	29.05	0.00	3.87	12.14	5.39	0.00	0.00	0.00	0.01	0.00		Grass	Tree	Soil	Bare Rock
GV_1874	0.23	77.21	2.62	15.05	0.00	4.81	0.00	0.01	0.00	0.07	0.00	0.00	0.00		Grass	Tree	Sky	Building
GV_1875	4.37	1.02	14.40	14.76	52.74	7.90	0.05	0.12	0.00	4.62	0.02	0.00	0.01		Roads	Tree	Building	Sky
GV_1876	0.98	19.46	18.49	15.29	9.20	7.84	0.16	0.21	0.00	28.22	0.00	0.11	0.03		Sidewalk	Grass	Building	Tree
GV_1877	1.21	16.40	23.24	12.29	12.06	1.29	0.10	0.01	0.13	33.25	0.00	0.00	0.00		Sidewalk	Building	Grass	Tree
GV_1878	1.78	14.85	39.18	3.30	11.12	2.51	0.41	1.05	0.00	25.79	0.00	0.01	0.00		Building	Sidewalk	Grass	Roads
GV_1879	6.81	2.50	6.46	16.68	55.03	3.53	0.64	0.86	0.15	7.29	0.00	0.04	0.02		Roads	Tree	Sidewalk	Background
GV_1880	2.08	7.64	7.42	40.99	17.17	1.04	0.87	1.86	0.00	18.60	0.00	2.34	0.00		Tree	Sidewalk	Roads	Grass
GV_1881	4.60	29.45	26.43	2.68	2.87	0.28	19.54	0.92	0.00	11.57	0.00	1.66	0.00		Grass	Building	Soil	Sidewalk
GV_1882	3.78	10.81	16.27	4.93	28.37	14.13	0.52	1.82	0.59	18.70	0.00	0.07	0.00		Roads	Sidewalk	Building	Sky
GV_1883	1.73	52.32	2.33	16.63	2.53	13.94	0.96	0.13	0.00	0.10	0.00	9.31	0.02		Grass	Tree	Sky	Gravel
GV_1884	3.18	34.58	3.95	9.82	31.99	12.97	1.04	1.83	0.00	0.21	0.00	0.27	0.17		Grass	Roads	Sky	Tree
GV_1885	2.94	25.33	1.74	14.71	39.92	10.15	3.43	0.05	0.00	0.68	0.11	0.91	0.01		Roads	Grass	Tree	Sky
GV_1886	1.95	44.71	3.63	16.19	2.66	5.99	0.97	0.10	0.00	23.75	0.00	0.03	0.02		Grass	Sidewalk	Tree	Sky

GV_1887	0.23	24.03	0.02	32.20	19.10	4.68	1.36	0.00	0.00	0.26	6.10	12.01	0.01		Tree	Grass	Roads	Gravel
GV_1888	1.65	38.89	0.16	7.64	17.49	16.05	0.84	7.48	0.00	0.69	0.00	8.95	0.15		Grass	Roads	Sky	Gravel
GV_1889	0.51	28.34	0.11	27.06	27.30	7.79	0.43	0.00	0.07	2.02	0.30	6.06	0.00		Grass	Roads	Tree	Sky
GV_1890	2.44	43.10	2.90	35.24	3.34	6.46	5.64	0.31	0.00	0.30	0.00	0.14	0.13		Grass	Tree	Sky	Soil
GV_1891	0.19	49.26	0.02	21.47	17.81	2.50	0.80	0.02	0.00	6.30	0.00	1.61	0.02		Grass	Tree	Roads	Sidewalk
GV_1892	8.79	33.30	0.43	24.49	4.57	8.09	10.97	0.51	6.87	0.12	0.01	1.07	0.77		Grass	Tree	Soil	Background
GV_1893	0.08	35.34	0.15	28.52	14.41	3.66	2.59	0.00	0.00	1.27	0.00	13.80	0.17		Grass	Tree	Roads	Gravel
GV_1894	0.00	34.23	0.21	41.75	2.08	3.13	10.13	0.00	4.71	0.61	0.01	3.12	0.00		Tree	Grass	Soil	Sand
GV_1895	0.58	22.14	0.53	31.67	33.28	7.50	0.27	0.02	0.00	1.52	0.00	2.22	0.27		Roads	Tree	Grass	Sky
GV_1896	2.30	31.60	1.85	30.13	25.20	6.97	0.01	0.00	0.00	1.76	0.00	0.00	0.18		Grass	Tree	Roads	Sky
GV_1897	9.76	9.91	15.34	18.68	22.89	3.19	0.16	4.41	0.00	11.96	0.00	3.68	0.02		Roads	Tree	Building	Sidewalk
GV_1898	0.15	54.24	0.06	34.47	8.91	0.07	0.28	0.06	0.00	0.00	0.00	1.77	0.00		Grass	Tree	Roads	Gravel
GV_1899	1.13	29.39	0.51	39.34	8.60	3.41	2.38	0.05	0.00	0.07	0.04	15.08	0.00		Tree	Grass	Gravel	Roads
GV_1900	1.49	42.66	0.56	26.74	4.06	1.50	4.71	0.00	0.00	0.00	0.00	17.78	0.51		Grass	Tree	Gravel	Soil
GV_1901	0.36	41.28	0.09	46.48	6.87	0.28	4.27	0.03	0.00	0.00	0.00	0.35	0.00		Tree	Grass	Roads	Soil
GV_1902	0.98	30.92	0.57	29.43	13.62	5.36	4.69	0.00	0.19	0.16	0.02	14.05	0.00		Grass	Tree	Gravel	Roads
GV_1903	0.04	16.33	0.15	30.63	10.36	10.35	7.05	4.42	0.00	0.00	0.00	20.67	0.00		Tree	Gravel	Grass	Roads
GV_1904	1.18	21.73	2.01	46.25	13.24	4.38	7.54	0.01	0.00	0.30	0.00	3.36	0.00		Tree	Grass	Roads	Soil
GV_1905	0.17	37.32	0.07	36.13	9.57	5.18	8.73	0.10	0.00	0.04	0.00	2.22	0.47		Grass	Tree	Roads	Soil
GV_1906	1.48	35.82	0.00	46.30	6.91	0.01	3.63	4.90	0.00	0.00	0.05	0.89	0.00		Tree	Grass	Roads	Bare Rock
GV_1907	0.63	49.71	0.00	30.06	0.00	3.35	4.19	8.54	0.00	0.01	0.06	3.45	0.01		Grass	Tree	Bare Rock	Soil
GV_1908	0.04	44.96	0.00	45.34	8.49	0.00	0.07	0.00	0.00	0.00	0.00	1.09	0.00		Tree	Grass	Roads	Gravel
GV_1909	3.78	26.13	0.01	69.82	0.09	0.01	0.13	0.01	0.01	0.00	0.00	0.01	0.00		Tree	Grass	Background	Soil
GV_1910	2.91	36.60	10.79	28.23	15.49	4.92	0.14	0.00	0.00	0.87	0.00	0.00	0.04		Grass	Tree	Roads	Building
GV_1911	2.79	31.71	0.03	33.21	27.34	0.44	0.14	0.00	0.00	0.06	0.00	4.27	0.02		Tree	Grass	Roads	Gravel
GV_1912	1.51	16.55	0.17	51.03	8.51	2.66	12.26	1.20	0.00	0.86	0.00	4.70	0.54		Tree	Grass	Soil	Roads
GV_1913	0.44	46.89	0.42	41.11	1.70	6.34	0.52	0.06	0.00	2.37	0.00	0.16	0.00		Grass	Tree	Sky	Sidewalk
GV_1914	0.47	13.27	0.00	38.01	43.74	3.07	0.04	0.00	0.21	0.73	0.00	0.34	0.13		Roads	Tree	Grass	Sky
GV_1915	0.11	43.93	0.04	42.73	3.79	4.15	2.09	0.32	0.16	0.62	0.08	1.96	0.04		Grass	Tree	Sky	Roads
GV_1916	0.12	42.84	0.00	34.85	5.43	6.67	3.49	0.00	0.00	0.12	0.00	5.81	0.67		Grass	Tree	Sky	Gravel
GV_1917	2.34	32.09	0.03	42.55	0.36	3.39	4.95	2.19	3.32	0.39	0.68	7.64	0.06		Tree	Grass	Gravel	Soil
GV_1918	0.72	13.87	0.42	49.42	23.33	5.32	1.53	0.01	0.00	3.78	0.07	0.86	0.68		Tree	Roads	Grass	Sky
GV_1919	2.68	38.70	0.00	31.94	5.14	1.90	17.67	0.07	0.00	0.57	0.00	1.24	0.08		Grass	Tree	Soil	Roads
GV_1920	0.54	28.83	0.11	34.06	15.65	7.52	6.34	0.48	1.17	0.08	1.19	4.01	0.01		Tree	Grass	Roads	Sky
GV_1921	0.39	12.86	0.00	38.77	0.26	7.71	29.38	7.01	1.04	0.02	0.00	1.20	1.35		Tree	Soil	Grass	Sky
GV_1922	1.33	42.57	0.21	22.14	24.18	3.53	3.55	0.21	0.00	0.01	0.11	1.87	0.28		Grass	Roads	Tree	Soil
GV_1923	0.15	34.75	0.28	16.28	24.96	7.36	3.80	0.17	0.00	0.33	0.50	10.31	1.10		Grass	Roads	Tree	Gravel
GV_1924	1.33	31.69	1.51	16.26	38.62	6.85	1.92	0.04	0.00	0.91	0.00	0.51	0.35		Roads	Grass	Tree	Sky
GV_1925	0.36	34.42	0.83	19.91	33.02	8.77	0.06	0.16	0.00	1.65	0.00	0.25	0.57		Grass	Roads	Tree	Sky
GV_1926	0.29	38.67	0.10	21.59	27.38	3.09	1.54	0.01	2.08	0.26	0.09	4.88	0.00		Grass	Roads	Tree	Gravel
GV_1927	0.43	49.71	0.44	9.11	30.16	4.86	2.03	0.46	0.00	0.13	0.84	1.71	0.13		Grass	Roads	Tree	Sky
GV_1928	0.32	39.48	0.05	4.92	16.87	12.59	18.86	6.25	0.00	0.00	0.19	0.40	0.06		Grass	Soil	Roads	Sky
GV_1929	3.47	41.79	0.02	26.78	7.62	4.26	9.27	4.45	0.17	0.33	0.08	1.44	0.32		Grass	Tree	Soil	Roads
GV_1930	9.28	34.60	1.60	26.97	5.09	5.95	5.47	3.47	0.63	0.95	4.44	1.08	0.46		Grass	Tree	Background	Sky
GV_1931	5.25	21.90	0.54	4.75	36.27	18.48	1.53	1.78	0.00	0.02	0.19	8.64	0.66		Roads	Grass	Sky	Gravel
GV_1932	0.66	6.91	0.55	0.15	55.00	21.63	6.97	0.90	0.00	0.01	0.00	7.22	0.00		Roads	Sky	Gravel	Soil
GV_1933	0.60	17.56	0.04	0.83	40.53	12.46	19.56	5.45	0.00	0.01	0.00	2.94	0.02		Roads	Soil	Grass	Sky
GV_1934	0.02	40.39	0.00	0.01	32.70	20.45	1.33	2.64	0.00	0.30	1.36	0.02	0.79		Grass	Roads	Sky	Bare Rock
GV_1935	2.75	4.70	11.73	4.82	52.23	18.86	1.56	2.58	0.00	0.03	0.00	0.73	0.01		Roads	Sky	Building	Tree
GV_1936	1.29	23.10	0.87	7.80	0.12	32.20	29.13	4.72	0.06	0.00	0.08	0.42	0.21		Sky	Soil	Grass	Tree
GV_1937	1.61	88.68	0.00	3.60	0.30	1.29	4.09	0.00	0.00	0.00	0.00	0.44	0.00		Grass	Soil	Tree	Background
GV_1938	3.39	54.01	6.62	3.69	0.35	6.54	0.49	0.21	0.00	24.69	0.00	0.01	0.00		Grass	Sidewalk	Building	Sky
GV_1939	7.09	52.18	19.57	7.95	2.51	0.16	2.07	0.01	0.00	8.16	0.00	0.29	0.00		Grass	Building	Sidewalk	Tree
GV_1940	0.39	58.52	0.06	7.77	2.92	25.40	0.31	0.00	0.02	0.56	0.00	4.04	0.02		Grass	Sky	Tree	Gravel
GV_1941	0.44	40.42	0.27	16.05	1.92	38.97	0.02	0.00	0.00	0.00	0.00	1.87	0.05		Grass	Sky	Tree	Roads
GV_1942	2.75	77.70	0.13	8.75	0.01	6.76	2.24	0.08	0.01	0.00	0.00	1.34	0.23		Grass	Tree	Sky	Background
GV_1943	1.03	70.82	0.40	4.08	0.00	23.59	0.07	0.00	0.00	0.01	0.00	0.00	0.00		Grass	Sky	Tree	Background
GV_1944	0.20	52.16	0.15	12.50	0.41	28.27	3.20	0.00	0.00	0.06	0.00	3.05	0.00		Grass	Sky	Tree	Soil
GV_1945	7.24	53.34	7.92	23.97	1.02	5.06	0.21	0.00	0.00	1.17	0.00	0.07	0.00		Grass	Tree	Building	Background

GV_1946	3.90	21.82	3.90	24.07	6.69	8.07	9.57	2.43	0.10	9.96	0.23	8.97	0.30		Tree	Grass	Sidewalk	Soil
GV_1947	7.41	23.68	7.38	23.86	0.01	3.78	0.00	0.00	0.00	33.89	0.00	0.00	0.00		Sidewalk	Tree	Grass	Background
GV_1948	0.25	23.33	0.00	0.51	0.15	38.51	35.56	1.65	0.00	0.00	0.00	0.00	0.05		Sky	Soil	Grass	Bare Rock
GV_1949	1.04	67.56	5.20	17.67	0.06	8.15	0.00	0.00	0.00	0.30	0.00	0.00	0.01		Grass	Tree	Sky	Building
GV_1950	3.16	21.99	12.21	17.03	16.93	5.93	0.10	0.00	0.00	21.70	0.00	0.94	0.00		Grass	Sidewalk	Tree	Roads
GV_1951	3.01	31.67	3.80	31.03	18.14	1.94	0.19	0.00	0.00	9.68	0.00	0.53	0.00		Grass	Tree	Roads	Sidewalk
GV_1952	0.50	28.60	0.71	7.53	44.68	13.12	3.05	0.11	0.00	0.00	0.00	1.69	0.01		Roads	Grass	Sky	Tree
GV_1953	2.24	13.00	0.05	0.42	2.93	2.64	44.54	32.93	1.04	0.00	0.13	0.04	0.05		Soil	Bare Rock	Grass	Roads
GV_1954	0.33	81.49	0.07	13.20	0.00	4.90	0.00	0.00	0.00	0.00	0.00	0.00	0.00		Grass	Tree	Sky	Background
GV_1955	0.99	71.46	1.19	13.28	4.72	8.32	0.00	0.00	0.00	0.06	0.00	0.00	0.00		Grass	Tree	Sky	Roads
GV_1956	0.15	78.10	0.18	13.88	0.00	7.69	0.00	0.00	0.00	0.00	0.00	0.00	0.00		Grass	Tree	Sky	Building
GV_1957	0.77	49.75	2.04	17.32	3.74	25.63	0.05	0.03	0.04	0.48	0.14	0.00	0.00		Grass	Sky	Tree	Roads
GV_1958	1.61	58.00	1.92	16.91	4.62	16.01	0.00	0.03	0.07	0.79	0.02	0.00	0.01		Grass	Tree	Sky	Roads
GV_1959	8.94	4.49	0.00	0.11	0.00	41.89	42.69	1.88	0.00	0.00	0.00	0.00	0.00		Soil	Sky	Background	Grass
GV_1960	8.77	8.69	22.04	0.14	41.31	9.36	0.34	0.86	0.00	7.82	0.20	0.48	0.00		Roads	Building	Sky	Background
GV_1961	1.97	9.89	0.12	0.63	21.09	0.00	34.39	0.77	22.86	6.26	1.98	0.02	0.00		Soil	Sand	Roads	Grass
GV_1962	1.22	50.16	0.02	7.74	3.44	31.91	2.58	0.04	0.76	0.21	0.00	1.91	0.00		Grass	Sky	Tree	Roads
GV_1963	5.25	31.41	4.41	39.70	1.17	5.61	6.08	0.06	0.00	1.47	0.10	4.73	0.00		Tree	Grass	Soil	Sky
GV_1964	7.04	12.56	2.47	0.48	7.69	36.63	23.32	9.19	0.00	0.49	0.05	0.09	0.00		Sky	Soil	Grass	Bare Rock
GV_1965	0.90	8.37	0.00	0.14	0.01	46.67	35.21	8.10	0.59	0.00	0.00	0.00	0.00		Sky	Soil	Grass	Bare Rock
GV_1966	0.98	27.55	6.73	10.07	30.67	10.07	0.69	0.00	0.00	6.66	0.00	6.57	0.00		Roads	Grass	Tree	Sky
GV_1967	4.14	27.34	10.96	6.78	16.38	29.10	2.10	0.05	0.00	0.09	0.00	2.32	0.75		Sky	Grass	Roads	Building
GV_1968	5.20	0.49	47.03	0.93	17.78	9.26	0.05	4.80	0.00	14.29	0.00	0.17	0.00		Building	Roads	Sidewalk	Sky
GV_1969	3.58	13.37	13.42	31.52	20.04	4.05	1.83	0.01	0.01	4.48	0.92	6.76	0.00		Tree	Roads	Building	Grass
GV_1970	7.79	8.93	19.21	17.93	18.48	2.96	0.16	0.26	0.00	24.10	0.01	0.16	0.00		Sidewalk	Building	Roads	Tree
GV_1971	1.98	34.88	34.31	9.73	3.27	6.25	0.07	0.09	0.00	9.31	0.00	0.11	0.00		Grass	Building	Tree	Sidewalk
GV_1972	0.71	14.10	38.42	13.31	8.10	3.85	0.07	0.00	1.03	20.37	0.00	0.04	0.00		Building	Sidewalk	Grass	Tree
GV_1973	3.78	6.20	29.68	15.71	27.81	9.08	0.49	0.46	0.01	5.55	0.21	1.00	0.00		Building	Roads	Tree	Sky
GV_1974	4.63	13.90	19.72	2.06	32.56	13.91	1.18	1.92	0.03	8.15	1.11	0.65	0.18		Roads	Building	Sky	Grass
GV_1975	1.77	4.05	23.27	4.61	16.49	11.03	0.24	0.01	0.00	36.58	0.84	1.11	0.00		Sidewalk	Building	Roads	Sky
GV_1976	11.13	2.00	16.99	5.66	35.33	14.57	5.01	0.87	0.70	2.87	2.44	2.43	0.00		Roads	Building	Sky	Background
GV_1977	5.98	8.73	10.47	1.99	30.98	12.71	8.78	1.77	2.05	2.43	12.42	1.64	0.06		Roads	Sky	Water	Building
GV_1978	3.52	11.71	12.67	9.42	33.84	11.87	2.08	0.17	0.00	14.16	0.03	0.54	0.00		Roads	Sidewalk	Building	Sky
GV_1979	2.39	22.37	6.60	3.32	40.28	7.02	0.18	0.24	1.35	15.92	0.04	0.30	0.00		Roads	Grass	Sidewalk	Sky
GV_1980	3.31	9.79	20.24	1.80	49.98	6.45	0.00	1.35	0.77	5.65	0.00	0.63	0.03		Roads	Building	Grass	Sky
GV_1981	0.27	67.06	13.70	8.24	0.00	10.35	0.00	0.00	0.00	0.35	0.00	0.00	0.02		Grass	Building	Sky	Tree
GV_1982	2.18	1.07	11.99	17.84	29.04	7.79	1.11	0.01	3.53	25.44	0.00	0.01	0.00		Roads	Sidewalk	Tree	Building
GV_1983	5.87	2.05	41.53	2.00	18.75	2.06	1.91	0.70	3.03	22.01	0.00	0.09	0.00		Building	Sidewalk	Roads	Background
GV_1984	1.70	4.83	15.64	16.11	18.56	2.93	0.01	0.02	0.00	39.92	0.00	0.27	0.01		Sidewalk	Roads	Tree	Building
GV_1985	6.05	24.64	21.22	8.21	17.47	10.87	4.19	1.19	0.18	3.62	0.00	2.33	0.02		Grass	Building	Roads	Sky
GV_1986	8.93	3.10	24.55	14.20	15.86	10.86	0.21	0.25	0.00	17.83	3.93	0.22	0.07		Building	Sidewalk	Roads	Tree
GV_1987	3.51	6.63	31.15	6.30	20.17	14.62	0.73	0.26	0.48	15.87	0.00	0.08	0.20		Building	Roads	Sidewalk	Sky
GV_1988	3.01	6.50	43.75	9.08	13.44	2.81	0.17	0.04	0.00	18.76	0.00	2.44	0.00		Building	Sidewalk	Roads	Tree
GV_1989	17.82	1.77	40.71	3.09	10.27	1.32	12.62	0.13	0.00	12.25	0.00	0.03	0.00		Building	Background	Soil	Sidewalk
GV_1990	6.01	1.09	40.22	3.61	21.88	1.56	3.97	0.04	0.00	16.26	0.00	5.37	0.00		Building	Roads	Sidewalk	Background
GV_1991	4.69	5.49	19.34	27.32	21.45	6.16	0.27	9.22	0.07	5.68	0.02	0.30	0.00		Tree	Roads	Building	Bare Rock
GV_1992	4.79	5.37	7.02	23.39	26.38	3.66	0.12	6.23	0.00	22.93	0.01	0.10	0.00		Roads	Tree	Sidewalk	Building
GV_1993	2.51	1.98	41.93	3.90	21.60	19.52	0.70	0.01	0.00	4.15	0.00	3.71	0.00		Building	Roads	Sky	Sidewalk
GV_1994	3.99	7.47	35.76	18.56	7.60	6.04	2.20	0.33	0.00	16.49	0.00	1.57	0.00		Building	Tree	Sidewalk	Roads
GV_1995	3.07	6.83	28.28	20.84	2.79	14.27	0.30	6.40	0.01	16.05	0.00	1.15	0.01		Building	Tree	Sidewalk	Sky
GV_1996	6.25	0.48	44.07	0.55	20.16	2.90	0.01	12.65	0.00	12.62	0.01	0.30	0.00		Building	Roads	Bare Rock	Sidewalk
GV_1997	8.66	1.36	51.58	6.78	13.88	12.03	3.06	0.49	0.01	1.65	0.00	0.50	0.00		Building	Roads	Sky	Background
GV_1998	1.66	1.48	49.25	7.04	30.50	1.72	0.03	0.00	0.18	8.05	0.04	0.03	0.00		Building	Roads	Sidewalk	Tree
GV_1999	4.52	2.92	37.60	25.63	9.70	4.84	0.94	3.09	0.37	9.62	0.00	0.76	0.00		Building	Tree	Roads	Sidewalk
GV_2000	7.87	1.43	23.15	14.66	33.36	2.23	0.01	0.00	0.15	17.07	0.00	0.06	0.00		Roads	Building	Sidewalk	Tree
GV_2001	12.22	1.18	32.49	10.39	27.47	2.99	0.05	0.08	0.00	3.06	0.03	10.03	0.00		Building	Roads	Background	Tree
GV_2002	14.72	1.67	27.01	32.28	3.39	0.78	3.04	1.31	0.00	12.59	0.00	3.21	0.00		Tree	Building	Background	Sidewalk
GV_2003	3.27	0.31	52.72	0.00	27.37	5.04	0.12	0.01	0.15	11.00	0.00	0.00	0.00		Building	Roads	Sidewalk	Sky
GV_2004	6.23	1.71	52.98	0.99	31.93	1.88	0.00	0.00	0.00	4.28	0.00	0.00	0.00		Building	Roads	Background	Sidewalk

GV_2005	0.93	66.42	2.19	20.93	0.00	9.50	0.04	0.00	0.00	0.00	0.00	0.00	0.00		Grass	Tree	Sky	Building
GV_2006	0.42	51.59	0.03	5.57	12.54	2.82	5.10	11.49	0.36	0.02	3.97	5.64	0.45		Grass	Roads	Bare Rock	Gravel
GV_2007	3.86	34.57	0.22	22.61	6.35	5.70	11.17	4.17	0.16	0.01	1.85	7.52	1.81		Grass	Tree	Soil	Gravel
GV_2008	1.59	80.95	6.00	1.48	0.02	9.94	0.00	0.00	0.00	0.00	0.00	0.00	0.01		Grass	Sky	Building	Background
GV_2009	0.16	34.16	0.00	3.51	16.33	3.13	28.64	3.28	7.94	2.39	0.00	0.03	0.44		Grass	Soil	Roads	Sand
GV_2010	16.00	8.40	3.30	25.26	3.42	35.35	0.39	0.08	0.00	7.27	0.35	0.17	0.00		Sky	Tree	Background	Grass
GV_2011	0.60	29.40	0.01	1.41	7.47	50.61	1.87	0.17	0.40	0.00	0.00	8.06	0.00		Sky	Grass	Gravel	Roads
GV_2012	19.15	8.90	13.20	22.07	19.97	2.97	0.10	0.79	0.00	11.58	0.00	1.27	0.00		Tree	Roads	Background	Building
GV_2013	9.08	8.98	24.97	17.22	21.94	3.43	1.09	2.62	0.00	10.58	0.00	0.10	0.00		Building	Roads	Tree	Sidewalk
GV_2014	6.85	0.69	51.82	0.78	27.72	9.82	0.62	0.02	0.01	1.65	0.00	0.04	0.00		Building	Roads	Sky	Background
GV_2015	3.34	1.80	54.83	0.71	25.16	8.35	0.08	0.28	0.01	5.42	0.00	0.02	0.00		Building	Roads	Sky	Sidewalk
GV_2016	5.91	0.83	22.84	9.36	28.88	0.63	2.19	0.01	4.94	24.20	0.00	0.21	0.00		Roads	Sidewalk	Building	Tree
GV_2017	6.74	1.73	31.19	17.45	20.31	3.08	0.30	1.35	0.00	16.36	0.00	1.48	0.00		Building	Roads	Tree	Sidewalk
GV_2018	7.06	5.95	40.89	6.95	9.21	3.78	0.00	0.01	0.00	25.97	0.00	0.18	0.00		Building	Sidewalk	Roads	Background
GV_2019	5.87	4.29	30.46	7.60	35.66	3.87	1.60	0.03	0.00	2.92	0.00	7.69	0.00		Roads	Building	Gravel	Tree
GV_2020	3.96	7.54	35.06	16.87	5.34	3.62	0.74	21.04	0.00	4.97	0.00	0.85	0.00		Building	Bare Rock	Tree	Grass
GV_2021	13.60	20.16	9.85	32.90	3.83	1.96	4.54	0.70	0.00	12.26	0.00	0.20	0.00		Tree	Grass	Background	Sidewalk
GV_2022	6.76	0.54	52.98	3.46	10.83	1.19	0.03	4.81	0.00	19.04	0.00	0.37	0.00		Building	Sidewalk	Roads	Background
GV_2023	6.26	1.51	62.58	2.62	6.20	1.60	0.02	10.35	0.00	8.81	0.00	0.06	0.00		Building	Bare Rock	Sidewalk	Background
GV_2024	9.96	4.06	49.90	17.02	5.58	4.00	1.04	0.17	0.00	7.01	0.00	1.26	0.00		Building	Tree	Background	Sidewalk
GV_2025	9.50	0.86	44.42	3.17	34.84	1.49	0.03	1.62	0.00	3.99	0.00	0.09	0.00		Building	Roads	Background	Sidewalk
GV_2026	6.64	0.30	44.72	12.70	23.03	1.22	0.03	0.05	0.00	11.29	0.00	0.02	0.00		Building	Roads	Tree	Sidewalk
GV_2027	5.83	1.28	25.62	5.54	40.14	7.07	11.07	0.18	0.00	2.05	0.00	1.24	0.00		Roads	Building	Soil	Sky
GV_2028	15.76	33.20	13.59	13.36	3.12	5.30	1.37	1.21	0.00	11.01	1.56	0.50	0.00		Grass	Background	Building	Tree
GV_2029	6.71	5.99	35.07	3.80	18.62	7.00	0.27	0.17	2.66	9.02	6.31	4.38	0.00		Building	Roads	Sidewalk	Sky
GV_2030	10.24	5.19	27.52	9.11	22.76	2.21	0.09	0.51	0.14	14.81	6.30	1.10	0.00		Building	Roads	Sidewalk	Background
GV_2031	6.14	8.40	26.93	10.57	17.12	1.01	1.30	8.08	1.36	12.95	5.73	0.42	0.00		Building	Roads	Sidewalk	Tree
GV_2032	1.93	8.77	29.50	9.69	26.07	3.81	0.00	0.01	0.00	19.21	0.00	1.00	0.00		Building	Roads	Sidewalk	Tree
GV_2033	0.93	2.48	39.73	12.20	18.92	5.63	0.61	0.54	0.00	17.99	0.00	0.95	0.00		Building	Roads	Sidewalk	Tree
GV_2034	3.51	2.33	29.85	8.98	30.85	4.07	1.36	0.06	0.00	18.88	0.00	0.11	0.00		Roads	Building	Sidewalk	Tree
GV_2035	4.65	20.94	24.29	25.53	1.31	2.38	2.95	9.46	0.00	7.29	0.00	1.19	0.00		Tree	Building	Grass	Bare Rock
GV_2036	3.87	38.79	13.89	15.21	11.26	4.12	0.43	0.01	0.00	9.34	0.00	3.07	0.00		Grass	Tree	Building	Roads
GV_2037	4.04	0.66	31.22	9.87	31.05	3.39	0.01	0.00	0.00	19.29	0.00	0.47	0.00		Building	Roads	Sidewalk	Tree
GV_2038	10.71	10.86	13.83	17.68	25.19	6.42	0.20	4.83	2.15	7.80	0.00	0.32	0.00		Roads	Tree	Building	Grass
GV_2039	10.74	2.56	56.43	2.28	11.48	3.06	0.00	2.94	0.00	9.07	0.30	1.14	0.00		Building	Roads	Background	Sidewalk
GV_2040	11.52	3.12	55.49	2.13	6.84	3.59	0.00	0.01	0.00	17.26	0.00	0.03	0.00		Building	Sidewalk	Background	Roads
GV_2041	13.38	0.71	42.83	6.62	20.84	2.85	0.04	0.81	0.00	10.37	0.00	1.55	0.00		Building	Roads	Background	Sidewalk
GV_2042	13.11	11.82	30.91	13.57	21.55	3.52	0.41	0.28	0.00	3.84	0.29	0.69	0.00		Building	Roads	Tree	Background
GV_2043	2.40	29.73	18.20	4.31	32.11	11.02	0.05	0.00	0.00	2.06	0.00	0.06	0.07		Roads	Grass	Building	Sky
GV_2044	3.21	19.22	11.07	24.27	17.23	8.83	4.02	0.95	0.73	10.31	0.03	0.12	0.02		Tree	Grass	Roads	Building
GV_2045	3.11	13.61	9.76	10.65	1.80	11.60	33.12	0.03	14.52	1.45	0.00	0.35	0.00		Soil	Sand	Grass	Sky
GV_2046	2.43	9.69	9.62	19.05	1.93	5.58	34.75	0.16	16.70	0.08	0.00	0.00	0.00		Soil	Tree	Sand	Grass
GV_2047	3.79	11.10	11.56	14.65	3.31	16.51	20.95	0.35	17.09	0.65	0.02	0.01	0.00		Soil	Sand	Sky	Tree
GV_2048	4.36	14.31	16.81	11.83	2.30	22.16	21.10	0.71	5.59	0.22	0.00	0.58	0.01		Sky	Soil	Building	Grass
GV_2049	5.11	56.70	9.20	10.28	3.26	14.09	0.08	0.00	0.00	1.26	0.00	0.00	0.02		Grass	Sky	Tree	Building
GV_2050	7.78	17.94	7.82	19.53	9.65	17.93	6.11	0.41	0.00	11.65	0.00	1.13	0.05		Tree	Grass	Sky	Sidewalk
GV_2051	1.69	21.17	0.07	44.32	10.13	0.31	13.76	5.10	0.72	0.02	0.18	2.53	0.00		Tree	Grass	Soil	Roads
GV_2052	2.03	78.86	0.07	6.11	0.00	12.61	0.00	0.00	0.00	0.00	0.00	0.00	0.32		Grass	Sky	Tree	Background
GV_2053	9.67	0.68	64.19	5.55	0.53	3.91	0.05	12.02	0.00	2.81	0.00	0.59	0.00		Building	Bare Rock	Background	Tree
GV_2054	8.28	3.56	2.51	5.95	9.63	28.93	0.15	7.82	3.00	0.90	29.23	0.02	0.00		Water	Sky	Roads	Background
GV_2055	3.52	62.35	1.43	22.86	0.65	8.00	0.87	0.02	0.00	0.14	0.00	0.00	0.17		Grass	Tree	Sky	Background
GV_2056	3.12	3.91	33.72	14.40	17.17	9.64	0.00	0.50	0.00	17.54	0.00	0.00	0.00		Building	Sidewalk	Roads	Tree
GV_2057	0.21	46.90	0.07	11.52	0.31	35.21	3.83	0.00	0.00	1.05	0.02	0.89	0.01		Grass	Sky	Tree	Soil
GV_2058	0.92	11.99	2.84	7.23	5.83	39.33	27.97	0.08	0.00	0.72	0.01	2.09	0.98		Sky	Soil	Grass	Tree
GV_2059	6.04	14.68	2.54	4.34	8.32	39.92	20.68	2.64	0.23	0.12	0.00	0.28	0.20		Sky	Soil	Grass	Roads
GV_2060	0.88	33.20	0.62	6.69	0.11	29.41	24.63	2.45	0.82	0.00	0.60	0.22	0.36		Grass	Sky	Soil	Tree
GV_2061	1.33	37.25	0.11	13.85	5.05	36.91	2.33	0.00	0.07	0.24	0.00	2.83	0.02		Grass	Sky	Tree	Roads
GV_2062	2.84	52.60	1.11	22.94	13.22	1.23	0.69	0.03	0.00	3.54	0.00	1.79	0.00		Grass	Tree	Roads	Sidewalk
GV_2063	2.53	60.80	3.81	18.11	12.79	1.62	0.30	0.04	0.00	0.01	0.00	0.00	0.00		Grass	Tree	Roads	Building

GV_2064	13.89	41.94	0.18	3.82	22.37	2.95	1.51	0.02	0.01	12.98	0.03	0.29	0.02		Grass	Roads	Background	Sidewalk
GV_2065	0.18	95.15	1.46	2.73	0.00	0.41	0.01	0.00	0.00	0.05	0.00	0.00	0.00		Grass	Tree	Building	Sky
GV_2066	0.58	32.72	4.98	22.30	4.79	8.68	0.02	1.72	0.00	20.84	3.17	0.13	0.08		Grass	Tree	Sidewalk	Sky
GV_2067	4.02	90.83	1.92	1.71	0.12	0.14	1.19	0.06	0.00	0.00	0.00	0.00	0.00		Grass	Background	Building	Tree
GV_2068	9.68	13.15	0.46	0.83	0.05	38.97	35.49	1.37	0.01	0.00	0.00	0.00	0.00		Sky	Soil	Grass	Background
GV_2069	0.91	4.42	0.00	3.63	0.00	42.44	47.06	1.02	0.49	0.00	0.00	0.04	0.00		Soil	Sky	Grass	Tree
GV_2070	0.10	4.60	0.00	0.86	0.00	40.27	44.57	9.61	0.00	0.00	0.00	0.00	0.00		Soil	Sky	Bare Rock	Grass
GV_2071	4.27	0.92	0.34	0.01	0.06	30.74	56.95	5.49	0.42	0.00	0.74	0.05	0.00		Soil	Sky	Bare Rock	Background
GV_2072	0.85	0.28	3.61	0.00	0.11	38.56	55.55	0.09	0.94	0.00	0.00	0.00	0.00		Soil	Sky	Building	Sand
GV_2073	0.06	53.03	0.04	39.74	0.08	2.63	3.64	0.41	0.00	0.00	0.00	0.38	0.00		Grass	Tree	Soil	Sky
GV_2074	0.24	41.04	0.28	41.99	0.65	4.08	8.34	0.00	0.00	1.29	0.00	2.08	0.00		Tree	Grass	Soil	Sky
GV_2075	0.32	55.88	0.19	15.63	15.21	10.96	0.36	0.00	0.00	0.04	0.00	1.42	0.00		Grass	Tree	Roads	Sky
GV_2076	0.25	38.07	0.14	0.16	0.17	31.21	29.98	0.00	0.00	0.01	0.00	0.00	0.00		Grass	Sky	Soil	Background
GV_2077	5.85	1.95	35.91	14.36	16.70	10.05	0.07	1.45	0.40	12.77	0.39	0.00	0.09		Building	Roads	Tree	Sidewalk
GV_2078	1.29	6.65	4.18	1.47	26.75	21.41	33.45	0.43	0.68	0.00	3.66	0.05	0.00		Soil	Roads	Sky	Grass
GV_2079	4.61	53.91	0.00	0.02	6.12	30.39	0.64	1.61	2.38	0.02	0.06	0.00	0.24		Grass	Sky	Roads	Background
GV_2080	8.02	45.11	25.48	3.02	0.75	0.63	4.91	0.41	0.03	11.55	0.00	0.09	0.00		Grass	Building	Sidewalk	Background
GV_2081	4.81	44.64	14.88	15.33	3.15	5.07	0.09	0.00	0.00	11.92	0.02	0.09	0.00		Grass	Tree	Building	Sidewalk
GV_2082	0.40	49.43	0.00	10.52	0.12	29.23	10.16	0.00	0.00	0.00	0.00	0.13	0.00		Grass	Sky	Tree	Soil
GV_2083	3.79	43.90	0.00	11.23	6.68	26.00	8.06	0.00	0.01	0.00	0.00	0.32	0.00		Grass	Sky	Tree	Soil
GV_2084	0.81	48.28	2.39	9.15	1.45	37.01	0.04	0.00	0.00	0.03	0.00	0.84	0.00		Grass	Sky	Tree	Building
GV_2085	13.09	16.53	2.51	9.73	1.36	0.16	45.18	7.36	0.04	3.39	0.00	0.64	0.00		Soil	Grass	Background	Tree
GV_2086	1.87	30.84	3.08	49.69	0.68	1.56	0.21	11.13	0.00	0.63	0.00	0.32	0.00		Tree	Grass	Bare Rock	Building
GV_2087	1.16	80.76	0.02	7.92	0.05	0.01	7.33	0.03	0.00	0.00	0.00	2.72	0.00		Grass	Tree	Soil	Gravel
GV_2088	2.69	45.65	0.06	10.52	1.19	30.75	1.10	0.13	4.55	0.00	0.08	3.27	0.00		Grass	Sky	Tree	Sand
GV_2089	1.16	28.76	0.13	4.90	20.62	36.64	0.31	0.44	0.48	0.00	0.63	5.92	0.01		Sky	Grass	Roads	Gravel
GV_2090	1.69	30.40	6.37	16.40	33.96	0.25	2.03	0.39	0.00	8.37	0.00	0.13	0.00		Roads	Grass	Tree	Sidewalk
GV_2091	2.15	37.49	2.33	13.03	41.16	0.19	0.71	0.99	0.00	1.66	0.02	0.26	0.00		Roads	Grass	Tree	Building
GV_2092	1.96	43.23	1.17	11.09	27.19	0.25	6.09	1.41	0.06	6.18	0.35	1.03	0.00		Grass	Roads	Tree	Sidewalk
GV_2093	1.83	32.34	1.93	23.44	27.36	0.18	5.05	1.21	0.00	5.71	0.04	0.91	0.00		Grass	Roads	Tree	Sidewalk
GV_2094	4.21	38.30	0.52	8.94	32.87	0.19	7.68	0.45	0.01	6.48	0.15	0.19	0.00		Grass	Roads	Tree	Soil
GV_2095	11.58	36.75	2.06	9.88	23.24	0.62	1.21	1.32	0.37	9.08	0.76	3.11	0.00		Grass	Roads	Background	Tree
GV_2096	1.22	37.35	1.86	28.11	8.42	12.50	0.08	0.01	0.00	10.44	0.00	0.01	0.00		Grass	Tree	Sky	Sidewalk
GV_2097	0.81	25.37	0.23	10.09	0.00	28.83	33.41	1.17	0.01	0.00	0.06	0.01	0.02		Soil	Sky	Grass	Tree
GV_2098	1.85	78.71	1.35	13.34	0.68	1.40	0.00	0.00	0.00	2.66	0.00	0.00	0.02		Grass	Tree	Sidewalk	Background
GV_2099	1.44	43.86	0.09	10.67	0.34	35.79	3.03	0.06	0.00	0.00	0.00	4.72	0.00		Grass	Sky	Tree	Gravel
GV_2100	0.62	39.32	2.34	7.90	9.28	39.85	0.53	0.03	0.00	0.00	0.00	0.08	0.05		Sky	Grass	Roads	Tree
GV_2101	1.43	66.20	12.38	10.15	0.54	8.23	0.32	0.01	0.00	0.33	0.00	0.41	0.00		Grass	Building	Tree	Sky
GV_2102	4.69	10.13	25.68	8.10	12.24	30.05	5.76	0.89	0.00	0.43	0.31	1.65	0.07		Sky	Building	Roads	Grass
GV_2103	0.15	0.50	0.05	0.02	0.01	41.81	51.38	6.01	0.05	0.00	0.00	0.00	0.00		Soil	Sky	Bare Rock	Grass
GV_2104	0.07	2.16	0.00	1.03	0.00	39.64	49.31	7.68	0.11	0.00	0.00	0.00	0.00		Soil	Sky	Bare Rock	Grass
GV_2105	2.15	36.51	0.58	28.34	9.57	9.22	1.89	0.00	0.00	10.69	0.20	0.85	0.00		Grass	Tree	Sidewalk	Roads
GV_2106	1.37	30.18	0.04	50.16	9.74	1.00	4.24	0.06	0.00	0.03	0.00	3.18	0.00		Tree	Grass	Roads	Soil
GV_2107	0.58	25.65	0.88	4.81	11.35	45.33	2.74	0.15	0.00	0.00	0.00	8.50	0.00		Sky	Grass	Roads	Gravel
GV_2108	2.59	27.48	0.00	0.40	0.27	23.01	44.17	0.04	2.05	0.00	0.00	0.00	0.00		Soil	Grass	Sky	Background
GV_2109	1.13	75.13	0.01	3.87	0.01	19.14	0.29	0.00	0.00	0.05	0.00	0.37	0.00		Grass	Sky	Tree	Background
GV_2110	1.88	24.90	2.36	30.68	13.24	16.73	6.43	2.63	0.00	0.01	0.01	1.14	0.00		Tree	Grass	Sky	Roads
GV_2111	1.86	32.38	1.20	41.82	2.70	15.92	1.54	0.06	0.05	0.02	0.00	2.45	0.00		Tree	Grass	Sky	Roads
GV_2112	1.02	25.60	0.17	2.10	13.54	54.66	1.98	0.45	0.01	0.01	0.04	0.11	0.33		Sky	Grass	Roads	Tree
GV_2113	0.92	49.13	0.02	20.81	0.46	27.56	0.26	0.00	0.04	0.00	0.00	0.78	0.02		Grass	Sky	Tree	Background
GV_2114	1.96	45.70	0.03	11.01	0.40	37.18	2.24	0.29	0.46	0.00	0.00	0.71	0.02		Grass	Sky	Tree	Soil
GV_2115	0.97	33.43	0.07	21.75	1.16	5.69	21.18	1.12	10.35	0.35	2.42	1.38	0.12		Grass	Tree	Soil	Sand
GV_2116	1.64	28.01	2.34	13.64	19.09	10.20	6.27	3.81	0.00	13.59	0.00	1.36	0.06		Grass	Roads	Tree	Sidewalk
GV_2117	3.71	18.62	30.12	8.90	12.98	11.61	1.10	0.34	0.00	9.72	0.82	0.63	1.45		Building	Grass	Roads	Sky
GV_2118	3.31	29.81	9.32	12.67	2.90	7.70	1.32	0.81	0.00	27.52	2.77	0.36	1.51		Grass	Sidewalk	Tree	Building
GV_2119	2.69	22.62	5.95	15.35	8.32	10.83	0.98	5.63	0.00	25.39	0.76	0.53	0.95		Sidewalk	Grass	Tree	Sky
GV_2120	3.11	33.41	0.03	39.05	15.10	1.39	4.61	0.25	0.00	0.92	0.01	2.11	0.00		Tree	Grass	Roads	Soil
GV_2121	1.68	31.18	3.94	16.30	19.28	8.68	12.23	0.23	0.00	1.14	0.00	5.22	0.13		Grass	Roads	Tree	Soil
GV_2122	4.46	33.48	1.05	19.38	11.64	16.35	10.61	0.09	0.00	1.20	0.02	1.53	0.17		Grass	Tree	Sky	Roads

GV_2123	4.74	13.49	11.28	11.79	21.92	12.21	11.74	0.17	3.64	5.79	0.00	3.21	0.01		Roads	Grass	Sky	Tree
GV_2124	2.26	28.44	2.04	15.63	22.68	10.29	15.72	0.08	1.08	0.08	0.00	1.59	0.11		Grass	Roads	Soil	Tree
GV_2125	2.12	24.66	1.50	24.38	1.21	9.98	29.46	5.65	0.20	0.08	0.01	0.73	0.01		Soil	Grass	Tree	Sky
GV_2126	7.58	25.42	10.07	31.66	13.92	1.35	0.30	0.23	0.09	8.31	0.00	1.07	0.00		Tree	Grass	Roads	Building
GV_2127	0.96	61.98	1.46	19.68	0.10	11.66	0.78	0.00	0.01	3.34	0.01	0.03	0.00		Grass	Tree	Sky	Sidewalk
GV_2128	3.79	16.12	8.76	26.31	4.81	4.37	12.02	0.26	0.00	10.62	12.38	0.51	0.06		Tree	Grass	Water	Soil
GV_2129	3.69	33.95	2.60	10.11	6.12	8.24	4.41	0.95	0.56	7.89	19.48	1.89	0.10		Grass	Water	Tree	Sky
GV_2130	3.81	13.05	3.06	48.73	20.19	7.13	0.97	0.82	0.00	0.15	0.15	1.94	0.00		Tree	Roads	Grass	Sky
GV_2131	19.34	24.53	9.38	21.59	2.61	5.46	5.99	0.18	0.00	10.04	0.24	0.46	0.18		Grass	Tree	Background	Sidewalk
GV_2132	6.48	10.26	13.92	22.89	21.42	7.49	3.74	0.43	0.00	5.25	0.01	8.08	0.02		Tree	Roads	Building	Grass
GV_2133	8.77	6.83	15.91	2.05	10.74	12.56	1.37	0.75	0.04	13.77	23.62	3.11	0.49		Water	Building	Sidewalk	Sky
GV_2134	9.90	6.23	27.24	1.37	5.91	8.37	0.39	1.02	0.05	15.64	19.55	4.03	0.29		Building	Water	Sidewalk	Background
GV_2135	3.91	5.39	12.00	7.51	17.37	15.29	12.57	4.89	0.39	18.52	0.01	1.93	0.22		Sidewalk	Roads	Sky	Soil
GV_2136	3.42	18.43	12.02	31.30	23.84	2.14	0.35	0.36	0.01	6.83	0.00	1.30	0.00		Tree	Roads	Grass	Building
GV_2137	0.31	1.01	0.00	0.00	0.00	41.78	56.39	0.00	0.51	0.00	0.00	0.00	0.00		Soil	Sky	Grass	Sand
GV_2138	0.03	0.00	0.00	0.00	0.00	42.23	46.69	11.05	0.00	0.00	0.00	0.00	0.00		Soil	Sky	Bare Rock	Background
GV_2139	0.24	70.18	0.00	21.71	0.00	5.49	2.36	0.00	0.00	0.00	0.00	0.01	0.00		Grass	Tree	Sky	Soil
GV_2140	0.13	56.03	0.05	6.97	2.80	27.50	5.64	0.02	0.00	0.01	0.00	0.81	0.03		Grass	Sky	Tree	Soil
GV_2141	1.08	46.06	0.04	16.17	1.30	30.12	1.61	0.32	0.03	0.00	0.00	3.29	0.00		Grass	Sky	Tree	Gravel
GV_2142	6.52	48.32	9.31	24.65	4.36	5.35	1.49	0.00	0.00	0.00	0.00	0.00	0.01		Grass	Tree	Building	Background
GV_2143	5.21	19.93	0.05	33.43	9.94	0.08	16.52	6.31	0.30	0.00	0.36	7.87	0.00		Tree	Grass	Soil	Roads
GV_2144	2.41	2.78	0.08	71.56	12.84	0.47	2.98	0.77	0.09	0.00	0.01	6.02	0.00		Tree	Roads	Gravel	Soil
GV_2145	4.31	4.07	0.01	52.15	9.64	0.01	25.17	1.32	0.03	0.03	0.00	3.26	0.00		Tree	Soil	Roads	Background
GV_2146	4.37	22.11	0.00	51.36	0.11	0.02	7.15	13.54	0.00	0.07	0.00	1.27	0.00		Tree	Grass	Bare Rock	Soil
GV_2147	6.12	4.65	0.62	15.87	6.10	3.19	28.10	4.90	21.64	0.91	4.49	1.01	2.39		Soil	Sand	Tree	Background
GV_2148	5.96	8.30	0.15	26.67	8.32	0.78	35.93	10.60	0.00	0.93	0.40	1.71	0.24		Soil	Tree	Bare Rock	Roads
GV_2149	4.84	26.68	0.02	35.53	1.55	0.27	19.92	3.03	0.02	5.17	0.19	2.69	0.08		Tree	Grass	Soil	Sidewalk
GV_2150	6.00	24.55	0.05	18.68	0.65	0.74	32.66	8.88	0.27	1.40	0.30	5.38	0.45		Soil	Grass	Tree	Bare Rock
GV_2151	1.52	38.01	0.00	8.96	1.71	0.55	40.31	2.07	0.03	0.64	0.45	5.73	0.03		Soil	Grass	Tree	Gravel
GV_2152	6.34	28.63	0.46	37.92	3.15	3.21	10.11	2.67	0.00	3.67	0.01	3.71	0.11		Tree	Grass	Soil	Background
GV_2153	0.73	1.91	0.03	0.00	29.35	4.42	5.33	50.43	0.00	0.46	0.12	7.22	0.00		Bare Rock	Roads	Gravel	Soil
GV_2154	1.61	3.81	0.00	0.04	9.53	5.02	62.22	15.14	0.08	0.00	0.03	2.53	0.00		Soil	Bare Rock	Roads	Sky
GV_2155	10.35	16.06	0.86	30.28	15.16	1.57	8.10	10.63	0.08	0.39	1.25	5.23	0.05		Tree	Grass	Roads	Bare Rock
GV_2156	3.52	22.31	0.19	4.27	53.99	7.32	2.90	4.88	0.00	0.20	0.02	0.12	0.30		Roads	Grass	Sky	Bare Rock
GV_2157	2.50	58.02	0.05	2.79	4.98	7.29	2.36	4.15	6.13	0.00	7.90	3.64	0.17		Grass	Water	Sky	Sand
GV_2158	0.29	40.27	0.06	2.67	0.41	9.99	4.16	30.00	4.14	0.00	6.83	0.07	1.12		Grass	Bare Rock	Sky	Water
GV_2159	0.69	12.25	0.15	1.12	52.00	7.88	0.78	13.46	0.00	0.00	0.00	11.34	0.34		Roads	Bare Rock	Grass	Gravel
GV_2160	1.29	19.60	0.16	3.38	28.84	15.46	2.22	17.24	0.00	0.10	0.00	11.29	0.41		Roads	Grass	Bare Rock	Sky
GV_2161	11.03	26.65	0.26	4.67	6.55	8.33	1.87	35.57	0.09	0.00	1.75	2.23	1.00		Bare Rock	Grass	Background	Sky
GV_2162	0.54	1.37	8.77	0.17	0.03	42.14	40.47	0.05	2.31	0.02	0.38	0.01	3.74		Sky	Soil	Building	Blue Mountain
GV_2163	0.11	43.37	0.18	5.31	15.51	31.93	2.98	0.06	0.00	0.00	0.00	0.53	0.02		Grass	Sky	Roads	Tree
GV_2164	0.55	23.38	0.15	15.79	3.33	45.21	9.47	0.66	0.00	0.00	0.00	1.46	0.00		Sky	Grass	Tree	Soil
GV_2165	5.21	44.46	9.20	27.87	2.67	7.92	0.17	0.17	0.00	1.76	0.00	0.58	0.00		Grass	Tree	Building	Sky
GV_2166	12.52	0.06	8.62	0.07	23.89	18.59	2.27	12.06	16.72	4.49	0.61	0.00	0.09		Roads	Sky	Sand	Background
GV_2167	22.69	24.74	3.01	25.53	6.08	0.42	2.51	0.50	0.00	1.54	0.02	12.94	0.00		Tree	Grass	Background	Gravel
GV_2168	0.17	45.64	0.16	10.86	0.36	30.98	11.74	0.00	0.00	0.00	0.00	0.08	0.00		Grass	Sky	Soil	Tree
GV_2169	5.40	3.61	26.86	7.40	7.94	32.60	10.14	2.01	0.00	0.61	3.00	0.40	0.02		Sky	Building	Soil	Roads
GV_2170	11.15	0.56	6.18	0.35	0.29	40.02	0.53	1.56	0.08	0.88	38.40	0.00	0.00		Sky	Water	Background	Building
GV_2171	4.78	14.40	0.17	41.80	4.34	23.50	1.64	1.67	0.37	0.07	0.60	6.66	0.01		Tree	Sky	Grass	Gravel
GV_2172	2.71	18.49	0.27	8.10	9.44	48.01	1.34	0.05	0.00	0.49	0.00	11.02	0.08		Sky	Grass	Gravel	Roads
GV_2173	0.77	41.97	0.02	5.54	15.29	11.13	18.27	3.39	0.05	0.00	0.02	3.46	0.06		Grass	Soil	Roads	Sky
GV_2174	0.97	83.11	0.90	9.01	0.76	5.15	0.00	0.00	0.00	0.09	0.00	0.00	0.00		Grass	Tree	Sky	Background
GV_2175	0.53	36.27	0.00	30.22	0.46	0.00	31.23	1.27	0.01	0.00	0.00	0.00	0.00		Grass	Soil	Tree	Bare Rock
GV_2176	0.01	52.50	0.00	0.24	0.06	13.62	33.54	0.00	0.00	0.02	0.00	0.00	0.00		Grass	Soil	Sky	Tree
GV_2177	2.63	34.00	0.12	11.44	11.10	9.95	10.11	5.30	0.00	0.00	2.65	12.69	0.00		Grass	Gravel	Tree	Roads
GV_2178	2.98	46.71	5.59	32.89	1.48	5.92	0.17	0.06	0.00	3.54	0.00	0.67	0.00		Grass	Tree	Sky	Building
GV_2179	3.77	35.49	12.17	32.07	0.42	10.04	1.39	0.10	0.00	3.27	0.00	1.27	0.00		Grass	Tree	Building	Sky
GV_2180	0.29	10.84	6.86	0.00	0.00	81.96	0.00	0.00	0.01	0.00	0.03	0.00	0.00		Sky	Grass	Building	Background
GV_2181	0.18	10.79	10.49	2.22	0.22	76.06	0.00	0.00	0.00	0.00	0.03	0.02	0.00		Sky	Grass	Building	Tree

GV_2182	5.20	27.55	2.16	19.15	34.99	8.42	0.02	0.00	0.00	2.52	0.00	0.00	0.00		Roads	Grass	Tree	Sky
GV_2183	4.05	24.14	21.26	21.18	2.64	6.59	0.35	0.00	1.06	18.71	0.00	0.01	0.00		Grass	Building	Tree	Sidewalk
GV_2184	0.33	41.34	0.13	12.31	9.97	34.20	0.92	0.45	0.00	0.00	0.00	0.33	0.01		Grass	Sky	Tree	Roads
GV_2185	20.10	13.68	7.70	27.66	4.81	16.32	0.98	5.81	0.00	2.84	0.01	0.03	0.07		Tree	Background	Sky	Grass
GV_2186	8.00	20.88	6.59	25.98	16.45	10.59	0.12	1.17	0.00	8.43	0.04	1.14	0.60		Tree	Grass	Roads	Sky
GV_2187	1.13	75.60	0.08	0.63	0.22	20.05	1.83	0.00	0.00	0.09	0.30	0.02	0.04		Grass	Sky	Soil	Background
GV_2188	3.52	6.17	0.70	3.51	70.16	12.87	1.93	0.86	0.00	0.01	0.00	0.26	0.02		Roads	Sky	Grass	Background
GV_2189	0.56	51.50	0.06	2.69	0.24	13.78	17.18	0.00	1.20	0.00	0.03	12.63	0.14		Grass	Soil	Sky	Gravel
GV_2190	0.73	4.82	0.00	0.41	38.25	14.98	7.76	24.64	3.72	0.00	4.04	0.02	0.62		Roads	Bare Rock	Sky	Soil
GV_2191	9.62	56.60	0.04	19.00	7.11	0.62	2.56	2.38	0.26	0.19	0.06	1.56	0.00		Grass	Tree	Background	Roads
GV_2192	1.39	12.62	5.08	41.19	19.13	8.33	0.10	6.68	0.00	4.26	0.06	1.08	0.07		Tree	Roads	Grass	Sky
GV_2193	1.37	20.97	0.07	61.38	0.17	0.47	5.46	0.24	0.16	0.00	0.01	9.71	0.00		Tree	Grass	Gravel	Soil
GV_2194	1.25	39.99	0.07	4.20	0.71	31.03	19.67	0.13	0.00	0.00	0.03	2.52	0.40		Grass	Sky	Soil	Tree
GV_2195	1.81	6.53	0.03	0.52	28.99	12.58	7.35	25.93	3.71	1.14	11.22	0.15	0.03		Roads	Bare Rock	Sky	Water
GV_2196	0.24	2.05	0.02	1.56	10.05	15.24	8.13	6.42	25.55	8.41	21.63	0.41	0.29		Sand	Water	Sky	Roads
GV_2197	9.54	2.44	51.14	6.01	16.44	1.00	0.13	0.02	0.01	13.16	0.00	0.11	0.00		Building	Roads	Sidewalk	Background
GV_2198	9.38	1.63	52.57	10.09	14.38	2.07	0.01	0.00	0.04	3.84	0.00	6.00	0.00		Building	Roads	Tree	Background
GV_2199	0.41	38.77	0.20	23.63	28.37	3.19	0.82	0.00	0.00	0.17	0.02	4.41	0.01		Grass	Roads	Tree	Gravel
GV_2200	1.60	14.51	0.10	42.33	18.00	8.07	0.23	0.00	0.01	0.16	0.00	14.95	0.03		Tree	Roads	Gravel	Grass
GV_2201	0.96	38.30	1.29	18.88	25.13	12.70	0.15	0.00	0.00	0.22	0.03	2.15	0.17		Grass	Roads	Tree	Sky
GV_2202	1.19	12.91	7.68	16.77	42.19	14.60	0.54	0.03	0.00	0.21	0.00	3.89	0.00		Roads	Tree	Sky	Grass
GV_2203	4.47	25.36	0.03	31.25	33.84	0.50	0.30	0.00	0.00	0.26	0.00	3.99	0.00		Roads	Tree	Grass	Background
GV_2204	0.73	64.13	0.01	16.92	10.82	0.26	0.63	0.00	0.24	0.15	0.15	5.95	0.00		Grass	Tree	Roads	Gravel
GV_2205	2.28	40.66	2.45	23.77	7.60	16.39	0.89	1.70	0.00	0.89	0.00	3.37	0.01		Grass	Tree	Sky	Roads
GV_2206	4.75	9.21	9.50	20.81	30.24	11.19	1.15	6.34	0.14	5.56	0.47	0.56	0.06		Roads	Tree	Sky	Building
GV_2207	3.32	31.18	8.90	5.87	16.53	14.92	0.36	2.59	0.00	9.94	1.06	1.58	3.76		Grass	Roads	Sky	Sidewalk
GV_2208	0.41	36.94	0.02	12.36	0.43	38.31	10.98	0.00	0.00	0.01	0.00	0.53	0.02		Sky	Grass	Tree	Soil
GV_2209	1.27	32.71	0.32	20.25	1.66	39.81	3.29	0.03	0.10	0.03	0.00	0.35	0.17		Sky	Grass	Tree	Soil
GV_2210	2.29	56.76	5.58	12.16	6.81	13.83	0.01	0.00	0.00	2.55	0.00	0.00	0.01		Grass	Sky	Tree	Roads
GV_2211	1.10	0.90	0.01	0.00	28.17	6.85	16.64	10.74	0.06	34.12	1.38	0.02	0.00		Sidewalk	Roads	Soil	Bare Rock
GV_2212	10.65	0.16	0.00	0.00	29.08	0.02	22.67	1.51	0.00	35.90	0.00	0.02	0.00		Sidewalk	Roads	Soil	Background
GV_2213	0.33	0.05	61.65	0.01	0.00	26.35	0.00	0.00	0.00	11.60	0.00	0.00	0.00		Building	Sky	Sidewalk	Background
GV_2214	0.84	0.83	1.29	1.28	0.00	85.56	0.00	0.03	0.00	9.99	0.16	0.01	0.00		Sky	Sidewalk	Building	Tree
GV_2215	1.27	5.88	1.10	21.14	67.28	3.15	0.01	0.00	0.00	0.17	0.00	0.00	0.00		Roads	Tree	Grass	Sky
GV_2216	7.73	0.58	24.20	3.39	28.79	3.13	0.05	0.34	0.07	31.69	0.01	0.00	0.01		Sidewalk	Roads	Building	Background
GV_2217	2.45	62.84	3.27	19.74	4.30	7.09	0.00	0.00	0.00	0.26	0.00	0.00	0.05		Grass	Tree	Sky	Roads
GV_2218	0.81	75.86	8.45	3.39	4.50	2.57	0.31	0.01	0.00	2.10	0.01	1.99	0.00		Grass	Building	Roads	Tree
GV_2219	0.02	95.35	0.04	0.63	2.73	0.77	0.19	0.01	0.00	0.00	0.00	0.26	0.00		Grass	Roads	Sky	Tree
GV_2220	0.00	51.17	0.02	19.51	5.62	18.91	2.89	0.00	0.00	1.76	0.00	0.12	0.00		Grass	Tree	Sky	Roads
GV_2221	2.23	67.85	4.02	7.21	8.10	0.08	7.72	0.00	0.00	2.22	0.00	0.58	0.00		Grass	Roads	Soil	Tree
GV_2222	0.95	73.99	0.00	13.28	10.56	0.23	0.20	0.00	0.00	0.00	0.00	0.79	0.00		Grass	Tree	Roads	Background
GV_2223	2.65	35.78	0.03	0.01	0.24	36.66	17.32	3.46	1.62	1.19	0.21	0.00	0.83		Sky	Grass	Soil	Bare Rock
GV_2224	2.09	46.42	0.33	1.59	4.12	42.91	1.20	0.02	0.01	0.34	0.04	0.91	0.01		Grass	Sky	Roads	Background
GV_2225	1.18	50.89	2.02	1.00	1.72	0.05	38.90	0.02	0.00	3.98	0.00	0.23	0.00		Grass	Soil	Sidewalk	Building
GV_2226	3.16	42.08	0.71	3.89	12.36	4.76	30.06	0.88	0.30	0.20	0.50	1.11	0.00		Grass	Soil	Roads	Sky
GV_2227	8.21	21.39	3.17	12.03	10.06	1.67	22.76	0.09	1.94	13.34	0.01	5.33	0.01		Soil	Grass	Sidewalk	Tree
GV_2228	3.37	57.85	1.37	22.05	0.07	14.40	0.13	0.06	0.00	0.01	0.01	0.68	0.00		Grass	Tree	Sky	Background
GV_2229	1.41	45.78	0.00	27.00	1.76	0.74	18.36	1.10	0.00	0.00	0.00	3.84	0.00		Grass	Tree	Soil	Gravel
GV_2230	1.25	64.63	1.01	10.87	6.49	12.22	0.52	0.30	0.00	0.09	0.11	1.91	0.59		Grass	Sky	Tree	Roads
GV_2231	0.44	87.77	2.34	3.58	0.17	5.57	0.02	0.01	0.00	0.09	0.00	0.00	0.01		Grass	Sky	Tree	Building
GV_2232	4.20	58.20	1.07	15.92	0.82	4.31	14.05	0.06	0.03	0.45	0.71	0.15	0.00		Grass	Tree	Soil	Sky
GV_2233	2.15	42.75	0.09	30.58	13.76	2.17	3.79	0.11	0.00	0.00	0.01	4.59	0.00		Grass	Tree	Roads	Gravel
GV_2234	6.10	3.55	2.63	28.50	21.70	19.41	4.17	1.63	10.55	1.02	0.32	0.41	0.00		Tree	Roads	Sky	Sand
GV_2235	0.23	34.01	0.01	12.88	0.22	41.20	10.68	0.00	0.03	0.00	0.00	0.71	0.02		Sky	Grass	Tree	Soil
GV_2236	0.29	38.58	0.02	9.92	3.08	37.84	10.01	0.00	0.00	0.01	0.00	0.11	0.14		Grass	Sky	Soil	Tree
GV_2237	0.81	7.85	0.24	16.61	3.29	25.86	23.67	0.08	7.28	0.00	4.15	10.16	0.00		Sky	Soil	Tree	Gravel
GV_2238	1.90	16.97	1.69	17.35	0.06	14.00	46.13	0.18	0.40	0.91	0.00	0.04	0.37		Soil	Tree	Grass	Sky
GV_2239	5.73	63.96	1.43	12.05	0.22	7.04	7.80	0.69	0.00	0.01	0.00	1.07	0.00		Grass	Tree	Soil	Sky
GV_2240	0.08	83.53	0.08	9.66	0.10	6.05	0.46	0.05	0.00	0.00	0.00	0.00	0.00		Grass	Tree	Sky	Soil

GV_2241	2.23	49.55	0.01	26.46	0.24	0.25	13.13	2.92	0.86	0.00	0.04	4.31	0.00		Grass	Tree	Soil	Gravel
GV_2242	1.29	45.28	0.00	18.71	0.24	0.04	18.82	1.80	0.83	0.60	0.23	12.15	0.00		Grass	Soil	Tree	Gravel
GV_2243	3.99	35.67	0.00	7.68	0.04	0.11	22.82	5.33	0.36	0.00	0.22	23.79	0.00		Grass	Gravel	Soil	Tree
GV_2244	3.19	26.60	0.03	28.04	11.16	0.09	14.63	0.11	0.00	6.75	0.00	9.38	0.00		Tree	Grass	Soil	Roads
GV_2245	3.30	45.38	0.00	14.99	6.58	0.30	13.42	13.63	0.16	0.00	1.59	0.66	0.00		Grass	Tree	Bare Rock	Soil
GV_2246	0.96	31.24	0.10	39.37	19.16	1.77	0.66	0.02	0.00	0.00	0.00	6.72	0.00		Tree	Grass	Roads	Gravel
GV_2247	1.17	6.76	0.03	69.61	0.01	0.02	17.01	0.00	0.00	0.00	0.02	5.36	0.00		Tree	Soil	Grass	Gravel
GV_2248	1.68	5.02	0.00	79.67	0.77	0.06	10.86	0.07	0.00	0.00	0.03	1.86	0.00		Tree	Soil	Grass	Gravel
GV_2249	3.28	42.45	0.00	30.50	0.04	0.07	6.40	6.41	0.01	0.00	0.06	10.78	0.00		Grass	Tree	Gravel	Bare Rock
GV_2250	1.11	13.63	0.02	58.91	14.42	3.01	0.41	0.04	0.00	0.00	0.00	8.46	0.00		Tree	Roads	Grass	Gravel
GV_2251	1.12	36.41	0.06	42.92	8.39	0.22	0.84	0.00	0.00	0.00	0.02	10.03	0.00		Tree	Grass	Gravel	Roads
GV_2252	1.35	37.66	0.01	40.80	14.21	0.71	0.71	0.00	0.00	0.00	0.00	4.55	0.00		Tree	Grass	Roads	Gravel
GV_2253	0.49	20.52	0.00	40.57	0.00	0.08	25.15	0.24	0.00	0.08	0.00	12.88	0.00		Tree	Soil	Grass	Gravel
GV_2254	6.26	51.93	0.33	14.73	11.21	1.63	8.91	0.06	0.00	2.06	0.42	2.45	0.00		Grass	Tree	Roads	Soil
GV_2255	0.19	32.37	0.26	28.30	12.98	10.13	7.60	0.00	0.00	0.67	0.00	7.34	0.16		Grass	Tree	Roads	Sky
GV_2256	3.06	31.75	0.74	16.63	35.62	3.28	3.70	0.01	0.00	0.82	0.00	3.51	0.87		Roads	Grass	Tree	Soil
GV_2257	6.36	33.46	0.24	34.04	11.40	3.48	7.07	0.49	0.01	1.01	0.00	2.44	0.01		Tree	Grass	Roads	Soil
GV_2258	8.80	28.08	3.93	33.84	11.15	4.71	5.75	0.57	0.64	1.97	0.56	0.01	0.00		Tree	Grass	Roads	Background
GV_2259	0.80	42.45	0.00	30.45	7.82	0.20	15.51	1.64	0.00	0.00	0.09	1.03	0.00		Grass	Tree	Soil	Roads
GV_2260	0.04	45.88	0.00	27.19	6.92	1.39	8.12	0.00	4.57	0.15	0.00	5.68	0.05		Grass	Tree	Soil	Roads
GV_2261	0.76	77.87	0.14	18.27	0.09	1.48	0.54	0.03	0.66	0.00	0.16	0.01	0.00		Grass	Tree	Sky	Background
GV_2262	2.08	56.40	0.00	26.97	0.07	0.08	4.80	1.98	0.01	0.00	4.29	3.31	0.00		Grass	Tree	Soil	Water
GV_2263	4.90	37.82	0.03	29.87	3.41	3.18	13.49	3.84	0.00	0.00	3.02	0.43	0.00		Grass	Tree	Soil	Background
GV_2264	2.17	37.60	0.01	35.84	0.04	3.13	7.07	1.68	0.06	0.00	0.71	11.70	0.00		Grass	Tree	Gravel	Soil
GV_2265	0.01	24.08	0.00	66.78	0.00	0.03	7.33	0.00	0.00	0.00	0.00	1.78	0.00		Tree	Grass	Soil	Gravel
GV_2266	7.68	14.25	0.08	56.00	2.19	0.40	8.73	4.18	0.00	0.00	0.00	6.49	0.00		Tree	Grass	Soil	Background
GV_2267	1.82	30.63	0.00	44.81	4.76	0.06	11.20	1.05	0.00	0.00	0.00	5.67	0.00		Tree	Grass	Soil	Gravel
GV_2268	6.04	12.09	0.12	57.02	15.28	0.27	4.66	0.00	0.00	0.00	0.00	4.52	0.00		Tree	Roads	Grass	Background
GV_2269	0.37	2.82	0.02	0.59	0.09	40.94	15.28	13.72	1.50	0.03	24.16	0.44	0.03		Sky	Water	Soil	Bare Rock
GV_2270	3.74	19.70	0.19	43.01	0.34	22.44	8.85	0.05	0.01	1.11	0.00	0.56	0.00		Tree	Sky	Grass	Soil
GV_2271	3.93	8.34	3.18	24.85	9.02	46.63	0.04	0.77	0.05	0.98	0.48	0.23	1.51		Sky	Tree	Roads	Grass
GV_2272	0.27	56.72	0.00	10.84	0.05	31.04	0.79	0.00	0.00	0.00	0.00	0.29	0.00		Grass	Sky	Tree	Soil
GV_2273	0.41	39.68	0.01	12.83	0.33	29.89	16.68	0.06	0.00	0.01	0.00	0.09	0.00		Grass	Sky	Soil	Tree
GV_2274	2.25	2.83	0.90	3.56	1.84	40.18	4.51	18.00	6.48	5.68	11.76	0.06	1.95		Sky	Bare Rock	Water	Sand
GV_2275	2.51	58.55	0.59	28.22	0.10	7.22	0.20	0.92	0.16	0.01	0.68	0.85	0.00		Grass	Tree	Sky	Background
GV_2276	0.99	29.63	0.11	0.17	0.12	40.71	16.25	3.69	0.57	2.75	2.14	0.00	2.87		Sky	Grass	Soil	Bare Rock
GV_2277	1.21	5.81	0.49	19.80	0.00	69.46	0.00	0.23	0.00	2.97	0.00	0.00	0.02		Sky	Tree	Grass	Sidewalk
GV_2278	0.20	4.71	1.26	24.11	1.76	65.90	0.04	1.85	0.00	0.13	0.00	0.03	0.02		Sky	Tree	Grass	Bare Rock
GV_2279	2.46	16.64	17.35	18.27	13.41	22.83	0.42	0.07	0.73	7.14	0.06	0.00	0.63		Sky	Tree	Building	Grass
GV_2280	0.14	3.59	1.06	27.06	0.23	65.42	0.60	1.85	0.00	0.04	0.00	0.01	0.02		Sky	Tree	Grass	Bare Rock
GV_2281	12.91	59.30	2.48	9.20	0.69	12.66	1.82	0.13	0.00	0.00	0.00	0.79	0.04		Grass	Background	Sky	Tree
GV_2282	0.77	39.67	0.04	13.79	1.35	40.30	2.96	0.00	0.00	0.00	0.00	1.11	0.00		Sky	Grass	Tree	Soil
GV_2283	3.05	25.87	0.53	32.06	2.40	4.51	22.08	0.12	5.71	0.00	0.22	3.47	0.00		Tree	Grass	Soil	Sand
GV_2284	2.53	20.38	0.47	49.93	0.41	3.28	4.37	0.13	11.46	0.29	0.07	6.69	0.00		Tree	Grass	Sand	Gravel
GV_2285	5.37	39.53	12.23	24.81	1.43	8.29	0.37	0.04	0.00	7.93	0.00	0.00	0.00		Grass	Tree	Building	Sky
GV_2286	5.37	39.53	12.23	24.81	1.43	8.29	0.37	0.04	0.00	7.93	0.00	0.00	0.00		Grass	Tree	Building	Sky
GV_2287	3.54	66.74	0.16	18.52	0.93	8.94	0.36	0.03	0.00	0.79	0.00	0.00	0.00		Grass	Tree	Sky	Background
GV_2288	4.43	18.90	3.11	49.38	14.18	7.65	1.46	0.01	0.00	0.25	0.00	0.63	0.00		Tree	Grass	Roads	Sky
GV_2289	1.90	0.17	5.08	17.59	0.00	72.69	0.00	2.40	0.00	0.00	0.03	0.00	0.14		Sky	Tree	Building	Bare Rock
GV_2290	1.61	0.26	77.73	1.51	0.09	8.32	0.01	0.00	0.00	10.36	0.00	0.11	0.00		Building	Sidewalk	Sky	Background
GV_2291	1.13	34.14	5.09	35.22	0.81	6.26	1.31	1.90	0.00	13.85	0.00	0.30	0.00		Tree	Grass	Sidewalk	Sky
GV_2292	0.70	67.22	19.90	4.01	4.40	0.05	2.05	0.01	0.00	1.64	0.00	0.03	0.00		Grass	Building	Roads	Tree
GV_2293	0.34	49.45	0.22	33.03	10.46	4.72	0.03	0.00	0.00	1.22	0.52	0.00	0.00		Grass	Tree	Roads	Sky
GV_2294	0.62	32.72	0.00	12.85	1.28	44.34	7.67	0.12	0.02	0.00	0.00	0.15	0.24		Sky	Grass	Tree	Soil
GV_2295	0.62	37.65	0.03	15.11	5.59	36.96	2.98	0.01	0.00	0.04	0.00	0.99	0.02		Grass	Sky	Tree	Roads
GV_2296	12.68	53.91	14.67	13.71	0.55	1.71	2.67	0.01	0.00	0.00	0.00	0.09	0.00		Grass	Building	Tree	Background
GV_2297	0.24	50.43	0.02	14.15	0.06	33.61	1.02	0.00	0.00	0.00	0.03	0.44	0.00		Grass	Sky	Tree	Soil
GV_2298	0.13	40.58	0.38	30.01	20.04	0.80	2.33	0.09	0.18	0.33	0.02	5.10	0.00		Grass	Tree	Roads	Gravel
GV_2299	2.95	33.77	0.02	17.00	0.09	41.95	1.01	0.38	0.01	0.00	0.00	2.84	0.00		Sky	Grass	Tree	Background

GV_2300	2.94	29.28	18.84	24.64	11.88	6.35	0.12	0.43	0.00	5.45	0.00	0.07	0.00		Grass	Tree	Building	Roads
GV_2301	1.56	25.59	15.33	16.89	6.32	10.69	3.60	0.74	0.01	18.94	0.00	0.34	0.00		Grass	Sidewalk	Tree	Building
GV_2302	0.24	50.83	0.00	7.87	0.00	37.62	3.41	0.00	0.00	0.00	0.00	0.00	0.03		Grass	Sky	Tree	Soil
GV_2303	3.01	38.81	4.32	14.66	17.26	16.84	0.02	0.00	0.00	4.50	0.00	0.47	0.10		Grass	Roads	Sky	Tree
GV_2304	0.60	26.88	14.64	17.04	12.83	5.50	0.07	0.00	0.45	21.52	0.00	0.48	0.00		Grass	Sidewalk	Tree	Building
GV_2305	0.49	60.31	2.46	30.60	0.92	4.23	0.00	0.00	0.00	1.00	0.00	0.00	0.00		Grass	Tree	Sky	Building
GV_2306	1.19	4.82	8.79	24.41	32.40	9.85	0.46	0.02	2.04	15.95	0.00	0.07	0.00		Roads	Tree	Sidewalk	Sky
GV_2307	2.77	33.30	0.14	21.00	1.27	33.46	6.51	0.42	0.01	0.13	0.00	0.73	0.25		Sky	Grass	Tree	Soil
GV_2308	1.14	58.52	0.88	13.85	0.01	25.59	0.01	0.00	0.00	0.01	0.00	0.00	0.00		Grass	Sky	Tree	Background
GV_2309	3.70	60.45	10.69	23.12	0.13	1.62	0.03	0.00	0.00	0.18	0.00	0.09	0.00		Grass	Tree	Building	Background
GV_2310	0.52	0.95	0.02	0.02	5.09	19.83	72.96	0.59	0.00	0.00	0.00	0.00	0.00		Soil	Sky	Roads	Grass
GV_2311	0.02	0.36	0.00	0.00	0.00	41.23	47.28	11.10	0.00	0.00	0.00	0.00	0.00		Soil	Sky	Bare Rock	Grass
GV_2312	0.03	2.97	0.00	0.01	0.00	40.74	45.70	8.14	2.40	0.00	0.00	0.01	0.00		Soil	Sky	Bare Rock	Grass
GV_2313	0.65	36.05	0.03	0.22	0.99	43.79	9.20	7.24	0.27	0.00	0.17	0.00	1.39		Sky	Grass	Soil	Bare Rock
GV_2314	0.44	0.10	0.00	0.10	0.00	41.21	46.74	11.20	0.22	0.00	0.01	0.00	0.00		Soil	Sky	Bare Rock	Background
GV_2315	0.43	2.90	0.62	0.07	0.11	48.43	33.48	2.34	7.01	0.00	3.67	0.05	0.90		Sky	Soil	Sand	Water
GV_2316	3.63	53.74	7.03	16.97	1.82	14.45	0.58	0.18	0.00	1.22	0.00	0.20	0.17		Grass	Tree	Sky	Building
GV_2317	0.77	68.39	4.59	9.98	5.67	7.20	0.09	0.00	0.12	1.64	0.16	1.39	0.00		Grass	Tree	Sky	Roads
GV_2318	4.74	69.15	4.97	4.92	8.23	2.86	0.89	0.05	0.00	4.16	0.00	0.03	0.00		Grass	Roads	Building	Tree
GV_2319	0.60	44.03	0.19	39.85	7.82	6.72	0.10	0.00	0.20	0.04	0.00	0.45	0.00		Grass	Tree	Roads	Sky
GV_2320	3.10	17.36	10.44	17.76	24.43	8.71	2.99	7.44	0.00	6.24	0.00	1.54	0.00		Roads	Tree	Grass	Building
GV_2321	1.08	20.42	0.00	56.59	8.61	3.66	4.60	0.04	0.00	0.00	0.00	5.00	0.00		Tree	Grass	Roads	Gravel
GV_2322	1.15	1.49	0.82	1.52	60.77	30.17	0.18	2.01	0.27	0.10	1.47	0.05	0.00		Roads	Sky	Bare Rock	Tree
GV_2323	0.56	43.59	1.28	15.36	1.99	23.17	6.63	0.34	0.02	0.29	1.89	4.85	0.02		Grass	Sky	Tree	Soil
GV_2324	5.51	81.12	0.00	10.61	0.32	1.33	0.76	0.01	0.00	0.29	0.00	0.05	0.00		Grass	Tree	Background	Sky
GV_2325	3.20	0.86	0.00	50.63	0.00	0.00	45.31	0.00	0.00	0.00	0.00	0.00	0.00		Tree	Soil	Background	Grass
GV_2326	0.86	27.33	0.00	38.59	0.00	0.00	33.14	0.07	0.00	0.00	0.00	0.00	0.00		Tree	Soil	Grass	Background
GV_2327	4.78	47.01	0.02	18.12	5.44	0.04	20.45	0.21	0.00	0.00	0.00	3.95	0.00		Grass	Soil	Tree	Roads
GV_2328	6.07	63.81	0.00	2.88	0.15	0.72	16.29	9.90	0.00	0.00	0.12	0.07	0.00		Grass	Soil	Bare Rock	Background
GV_2329	1.13	63.41	0.00	24.01	0.00	0.18	10.78	0.32	0.00	0.00	0.00	0.18	0.00		Grass	Tree	Soil	Background
GV_2330	0.00	36.88	0.00	42.47	0.19	0.00	13.25	0.02	0.00	0.00	0.00	7.18	0.00		Tree	Grass	Soil	Gravel
GV_2331	1.15	34.62	0.02	43.13	8.16	0.00	10.01	0.14	0.00	0.00	0.00	2.77	0.00		Tree	Grass	Soil	Roads
GV_2332	0.48	65.74	0.02	15.65	5.56	6.42	1.66	0.01	0.02	0.14	4.13	0.19	0.00		Grass	Tree	Sky	Roads
GV_2333	0.35	18.89	0.00	45.82	0.00	0.39	34.38	0.09	0.00	0.00	0.00	0.08	0.00		Tree	Soil	Grass	Sky
GV_2334	3.34	57.01	0.02	24.59	0.91	4.90	3.71	0.89	0.73	3.40	0.10	0.40	0.00		Grass	Tree	Sky	Soil
GV_2335	2.04	67.01	0.00	15.20	2.16	7.31	3.17	0.01	0.06	2.85	0.00	0.19	0.00		Grass	Tree	Sky	Soil
GV_2336	4.31	38.76	3.78	25.87	5.62	6.36	2.11	0.15	0.00	12.97	0.00	0.07	0.00		Grass	Tree	Sidewalk	Sky
GV_2337	0.26	6.76	0.01	10.49	23.69	17.48	16.12	6.29	0.11	0.15	7.03	11.62	0.00		Roads	Sky	Soil	Gravel
GV_2338	0.85	56.09	0.57	25.49	0.03	3.11	13.58	0.10	0.00	0.01	0.00	0.15	0.00		Grass	Tree	Soil	Sky
GV_2339	0.69	7.04	0.73	29.38	56.69	5.17	0.13	0.04	0.00	0.12	0.00	0.00	0.00		Roads	Tree	Grass	Sky
GV_2340	0.16	39.45	0.05	14.81	27.51	15.09	1.40	0.36	0.69	0.27	0.04	0.11	0.05		Grass	Roads	Sky	Tree
GV_2341	1.23	35.04	0.17	41.84	20.95	0.31	0.08	0.00	0.00	0.17	0.00	0.21	0.00		Tree	Grass	Roads	Background
GV_2342	0.95	48.16	0.00	33.56	3.19	0.46	9.85	0.06	0.00	1.79	0.00	1.97	0.00		Grass	Tree	Soil	Roads
GV_2343	0.53	18.53	0.00	53.97	0.00	0.00	26.77	0.21	0.00	0.00	0.00	0.00	0.00		Tree	Soil	Grass	Background
GV_2344	0.97	30.42	0.00	47.97	0.01	0.00	19.86	0.07	0.00	0.00	0.00	0.69	0.00		Tree	Grass	Soil	Background
GV_2345	1.06	2.25	0.06	0.96	17.32	29.98	4.41	41.91	0.84	0.00	1.20	0.03	0.00		Bare Rock	Sky	Roads	Soil
GV_2346	0.08	14.80	0.26	10.81	22.61	27.62	5.23	2.17	0.03	0.56	0.03	15.80	0.00		Sky	Roads	Gravel	Grass
GV_2347	2.14	28.34	7.38	10.52	1.30	18.87	23.90	5.51	0.01	0.01	0.13	1.88	0.01		Grass	Soil	Sky	Tree
GV_2348	3.08	3.20	12.56	35.63	16.59	14.35	1.17	2.59	0.00	6.27	0.05	4.52	0.00		Tree	Roads	Sky	Building
GV_2349	1.45	16.52	4.30	27.71	22.03	10.12	13.15	0.46	0.68	0.04	0.39	3.14	0.00		Tree	Roads	Grass	Soil
GV_2350	4.40	24.09	27.80	9.13	1.22	20.86	0.55	1.30	0.00	9.02	0.00	1.63	0.00		Building	Grass	Sky	Tree
GV_2351	0.85	29.78	0.01	41.35	1.49	1.18	15.74	2.33	0.03	0.00	0.00	7.23	0.00		Tree	Grass	Soil	Gravel
GV_2352	1.26	30.25	0.05	33.33	5.30	8.12	5.81	0.38	1.47	0.11	0.00	13.92	0.00		Tree	Grass	Gravel	Sky
GV_2353	0.65	49.39	0.00	33.94	0.03	0.84	8.59	0.54	0.22	0.00	0.00	5.80	0.00		Grass	Tree	Soil	Gravel
GV_2354	0.42	8.71	0.30	43.82	13.84	2.40	20.39	0.02	0.00	0.57	0.00	9.52	0.00		Tree	Soil	Roads	Gravel
GV_2355	1.33	47.75	0.07	28.50	0.00	0.31	16.66	4.03	0.00	0.00	0.05	1.30	0.00		Grass	Tree	Soil	Bare Rock
GV_2356	0.61	7.13	0.40	33.40	25.07	11.34	14.22	1.31	0.05	6.09	0.00	0.37	0.00		Tree	Roads	Soil	Sky
GV_2357	0.37	13.48	0.31	35.57	42.66	2.04	0.02	0.18	0.00	4.96	0.35	0.06	0.00		Roads	Tree	Grass	Sidewalk
GV_2358	1.09	22.09	0.00	27.09	0.00	0.04	48.41	0.27	0.00	0.00	0.00	1.01	0.00		Soil	Tree	Grass	Background

GV_2359	0.26	21.76	0.06	46.30	22.95	1.93	3.93	0.00	0.00	0.00	0.00	2.81	0.00		Tree	Roads	Grass	Soil
GV_2360	0.28	32.31	0.08	28.48	11.26	4.21	9.82	0.26	0.76	5.86	0.04	6.63	0.00		Grass	Tree	Roads	Soil
GV_2361	0.26	18.87	0.07	42.17	15.15	0.29	9.92	0.15	0.00	0.00	1.11	12.03	0.00		Tree	Grass	Roads	Gravel
GV_2362	0.02	59.14	0.00	34.91	0.00	0.10	5.58	0.00	0.00	0.00	0.00	0.25	0.00		Grass	Tree	Soil	Gravel
GV_2363	0.73	56.79	0.06	25.29	6.76	9.36	0.44	0.22	0.00	0.26	0.07	0.01	0.00		Grass	Tree	Sky	Roads
GV_2364	1.67	69.91	0.01	20.33	5.15	2.15	0.45	0.00	0.00	0.00	0.00	0.35	0.00		Grass	Tree	Roads	Sky
GV_2365	6.52	47.65	7.74	28.78	0.46	7.15	0.20	0.00	0.00	1.16	0.00	0.33	0.00		Grass	Tree	Building	Sky
GV_2366	3.19	1.79	4.94	43.97	16.42	24.32	0.17	0.06	0.00	4.63	0.00	0.50	0.00		Tree	Sky	Roads	Building
GV_2367	1.88	62.10	0.88	17.16	0.96	16.97	0.00	0.00	0.00	0.01	0.05	0.00	0.00		Grass	Tree	Sky	Background
GV_2368	1.64	20.19	0.80	24.84	0.08	51.51	0.00	0.00	0.00	0.01	0.00	0.00	0.93		Sky	Tree	Grass	Background
GV_2369	0.08	73.05	0.00	10.40	0.85	0.00	11.15	0.11	0.00	0.00	0.00	4.35	0.00		Grass	Soil	Tree	Gravel
GV_2370	0.17	60.97	0.00	38.81	0.00	0.02	0.03	0.00	0.00	0.00	0.00	0.00	0.00		Grass	Tree	Background	Soil
GV_2371	0.10	73.04	0.01	21.66	0.00	0.01	1.74	0.93	0.00	0.00	0.00	2.52	0.00		Grass	Tree	Gravel	Soil
GV_2372	1.26	7.75	0.10	28.54	55.31	6.99	0.00	0.00	0.00	0.05	0.00	0.00	0.00		Roads	Tree	Grass	Sky
GV_2373	0.03	18.41	0.00	2.34	0.81	16.16	3.84	40.77	14.91	0.02	2.68	0.01	0.02		Bare Rock	Grass	Sky	Sand
GV_2374	0.17	63.40	0.01	1.29	10.92	14.64	2.06	1.91	0.00	2.41	1.97	0.00	1.22		Grass	Sky	Roads	Sidewalk
GV_2375	0.00	84.57	0.00	4.59	0.00	10.81	0.01	0.00	0.00	0.00	0.00	0.01	0.01		Grass	Sky	Tree	Gravel
GV_2376	0.65	12.69	0.00	0.01	15.09	21.99	11.07	30.98	1.55	0.08	5.52	0.20	0.16		Bare Rock	Sky	Roads	Grass
GV_2377	0.00	72.83	0.00	18.38	0.16	0.10	6.41	0.14	0.00	0.00	0.05	1.93	0.00		Grass	Tree	Soil	Gravel
GV_2378	0.40	68.70	0.00	16.97	0.00	0.25	3.62	7.54	0.00	0.00	0.00	2.50	0.00		Grass	Tree	Bare Rock	Soil
GV_2379	0.52	69.52	0.01	4.95	1.98	10.43	3.65	2.65	0.51	0.33	5.20	0.00	0.24		Grass	Sky	Water	Tree
GV_2380	0.01	4.67	0.01	18.67	58.89	16.05	0.04	0.01	0.00	1.60	0.00	0.03	0.03		Roads	Tree	Sky	Grass
GV_2381	0.24	14.27	0.00	80.49	0.00	0.06	3.46	0.03	0.00	0.00	0.00	1.46	0.00		Tree	Grass	Soil	Gravel
GV_2382	0.00	55.87	0.00	33.54	0.00	10.09	0.31	0.00	0.00	0.00	0.00	0.00	0.18		Grass	Tree	Sky	Soil
GV_2383	0.23	5.69	0.08	17.37	58.08	13.12	0.00	0.00	0.00	3.97	1.44	0.00	0.00		Roads	Tree	Sky	Grass
GV_2384	1.39	57.61	0.27	24.90	1.07	13.33	0.63	0.49	0.01	0.23	0.00	0.02	0.07		Grass	Tree	Sky	Background
GV_2385	1.46	85.23	0.15	4.54	0.00	8.08	0.00	0.00	0.00	0.00	0.00	0.46	0.07		Grass	Sky	Tree	Background
GV_2386	7.58	67.88	0.31	15.65	0.00	3.40	4.98	0.02	0.00	0.00	0.00	0.18	0.01		Grass	Tree	Background	Soil
GV_2387	0.74	73.20	0.13	15.81	2.94	6.18	0.76	0.14	0.00	0.00	0.10	0.01	0.00		Grass	Tree	Sky	Roads
GV_2388	2.12	13.52	0.02	44.74	29.45	0.03	0.87	2.71	2.08	0.01	0.65	3.81	0.00		Tree	Roads	Grass	Gravel
GV_2389	1.11	78.38	0.00	4.42	0.01	0.05	11.86	0.63	0.00	0.00	0.00	3.53	0.00		Grass	Soil	Tree	Gravel
GV_2390	0.44	36.67	0.00	50.10	0.02	0.00	12.28	0.04	0.00	0.00	0.00	0.46	0.00		Tree	Grass	Soil	Gravel
GV_2391	0.09	49.58	0.00	48.50	0.00	0.03	1.45	0.01	0.00	0.00	0.00	0.34	0.00		Grass	Tree	Soil	Gravel
GV_2392	1.43	57.93	0.45	19.15	0.00	3.49	12.42	3.33	0.00	0.06	0.00	1.74	0.00		Grass	Tree	Soil	Sky
GV_2393	1.09	76.09	0.00	9.29	0.00	3.59	9.04	0.01	0.00	0.06	0.00	0.84	0.00		Grass	Tree	Soil	Sky
GV_2394	0.16	87.67	0.00	1.39	0.02	0.01	10.63	0.04	0.00	0.00	0.00	0.09	0.00		Grass	Soil	Tree	Background
GV_2395	0.01	92.00	0.00	2.38	0.00	0.00	5.13	0.02	0.00	0.00	0.00	0.45	0.00		Grass	Soil	Tree	Gravel
GV_2396	0.77	67.12	0.00	17.69	0.00	0.05	9.09	0.01	0.00	0.00	0.00	5.27	0.00		Grass	Tree	Soil	Gravel
GV_2397	2.84	47.81	0.28	11.90	28.49	1.62	1.67	0.00	0.01	0.08	2.14	3.16	0.00		Grass	Roads	Tree	Gravel
GV_2398	5.61	43.82	8.51	30.10	1.47	6.85	1.40	0.02	0.00	1.56	0.00	0.66	0.00		Grass	Tree	Building	Sky
GV_2399	5.32	89.24	0.00	5.12	0.01	0.00	0.04	0.00	0.14	0.00	0.00	0.12	0.00		Grass	Background	Tree	Sand
GV_2400	3.53	32.13	2.88	30.50	27.62	2.63	0.32	0.01	0.00	0.26	0.00	0.12	0.00		Grass	Tree	Roads	Background
GV_2401	1.69	82.70	1.65	12.70	0.01	1.09	0.14	0.00	0.00	0.02	0.00	0.00	0.00		Grass	Tree	Background	Building
GV_2402	1.40	67.23	1.71	14.92	0.71	0.40	0.00	0.01	0.00	13.62	0.00	0.00	0.00		Grass	Tree	Sidewalk	Building
GV_2403	5.89	85.48	0.84	6.52	0.00	0.00	0.06	0.00	0.00	1.19	0.00	0.00	0.00		Grass	Tree	Background	Sidewalk
GV_2404	7.81	9.43	1.34	31.78	17.44	0.69	20.49	0.85	0.11	8.90	0.31	0.84	0.01		Tree	Soil	Roads	Grass
GV_2405	12.31	65.39	0.80	20.83	0.22	0.27	0.17	0.00	0.00	0.00	0.00	0.00	0.00		Grass	Tree	Background	Building
GV_2406	0.62	33.28	0.35	61.61	1.30	1.10	0.16	0.00	0.00	0.00	0.00	1.57	0.00		Tree	Grass	Gravel	Roads
GV_2407	5.73	78.46	0.33	14.62	0.08	0.40	0.01	0.00	0.00	0.08	0.00	0.30	0.00		Grass	Tree	Background	Sky
GV_2408	1.44	35.02	1.17	37.53	2.20	19.61	0.00	0.03	0.00	2.59	0.00	0.39	0.01		Tree	Grass	Sky	Sidewalk
GV_2409	0.23	81.83	0.17	1.48	1.79	0.36	2.50	0.00	0.00	1.11	10.40	0.12	0.00		Grass	Water	Soil	Roads
GV_2410	0.40	77.46	1.01	17.93	0.00	3.16	0.00	0.00	0.00	0.04	0.00	0.00	0.00		Grass	Tree	Sky	Building
GV_2411	0.90	34.15	0.04	54.85	8.52	0.05	0.72	0.02	0.00	0.00	0.00	0.75	0.00		Tree	Grass	Roads	Background
GV_2412	12.21	55.32	1.43	26.42	0.40	1.26	0.06	0.00	0.00	2.83	0.00	0.07	0.00		Grass	Tree	Background	Sidewalk
GV_2413	1.88	72.59	1.87	20.54	0.04	1.94	0.18	0.00	0.00	0.74	0.00	0.21	0.00		Grass	Tree	Sky	Background
GV_2414	3.15	9.24	0.06	6.31	0.00	63.55	1.89	1.04	0.00	0.00	14.77	0.00	0.00		Sky	Water	Grass	Tree
GV_2415	0.57	47.86	10.18	0.82	1.01	11.18	26.28	0.03	0.00	0.00	0.01	2.04	0.02		Grass	Soil	Sky	Building
GV_2416	2.24	15.92	20.83	8.85	8.70	17.63	4.70	0.21	0.77	17.76	0.00	2.39	0.00		Building	Sidewalk	Sky	Grass
GV_2417	3.56	39.57	16.71	14.57	5.57	12.17	0.04	0.06	0.00	7.75	0.00	0.00	0.00		Grass	Building	Tree	Sky

GV_2418	8.46	5.60	21.97	17.47	26.95	11.10	6.13	0.07	0.00	2.23	0.00	0.01	0.00		Roads	Building	Tree	Sky
GV_2419	3.56	7.41	36.45	6.52	30.80	9.06	1.19	0.01	0.01	2.84	0.00	2.15	0.00		Building	Roads	Sky	Grass
GV_2420	0.41	54.40	3.21	10.33	10.04	11.10	0.24	0.10	0.00	6.74	0.00	3.43	0.01		Grass	Sky	Tree	Roads
GV_2421	2.63	18.72	2.46	41.94	10.95	6.44	4.21	0.70	0.04	9.37	0.00	2.51	0.02		Tree	Grass	Roads	Sidewalk
GV_2422	1.73	19.35	20.91	28.89	8.74	6.30	4.08	0.49	0.00	5.79	0.00	3.72	0.02		Tree	Building	Grass	Roads
GV_2423	1.27	49.97	2.11	16.21	15.36	10.23	0.19	0.00	0.00	4.64	0.00	0.01	0.00		Grass	Tree	Roads	Sky
GV_2424	2.48	3.28	23.58	18.47	37.86	5.76	0.06	0.00	0.78	7.43	0.00	0.28	0.01		Roads	Building	Tree	Sidewalk
GV_2425	50.95	13.22	2.67	2.08	4.18	0.17	3.53	2.38	0.67	18.48	0.00	1.67	0.00		Background	Sidewalk	Grass	Roads
GV_2426	0.31	59.01	0.10	37.58	0.42	2.45	0.00	0.00	0.00	0.12	0.01	0.01	0.00		Grass	Tree	Sky	Roads
GV_2427	0.22	47.71	0.11	44.72	2.48	1.16	3.06	0.00	0.00	0.13	0.00	0.41	0.00		Grass	Tree	Soil	Roads
GV_2428	0.03	66.11	0.03	13.05	8.79	6.31	0.03	0.00	0.00	5.42	0.00	0.22	0.00		Grass	Tree	Roads	Sky
GV_2429	0.02	65.63	0.00	29.61	0.00	4.74	0.00	0.00	0.00	0.00	0.00	0.00	0.00		Grass	Tree	Sky	Background
GV_2430	1.69	44.12	0.01	39.83	4.02	0.01	7.81	0.34	0.00	0.00	0.52	1.65	0.00		Grass	Tree	Soil	Roads
GV_2431	0.11	27.53	0.03	57.13	2.03	0.01	10.28	0.00	0.00	0.00	0.00	2.87	0.00		Tree	Grass	Soil	Gravel
GV_2432	23.00	5.72	2.72	25.43	5.12	8.53	25.74	0.07	3.43	0.00	0.07	0.17	0.00		Soil	Tree	Background	Sky
GV_2433	0.09	49.65	0.20	15.44	11.53	20.77	0.06	0.00	0.00	0.03	0.05	2.01	0.17		Grass	Sky	Tree	Roads
GV_2434	0.87	36.08	1.16	19.60	20.93	10.10	3.04	0.00	0.00	0.11	0.00	7.99	0.13		Grass	Roads	Tree	Sky
GV_2435	0.74	58.88	2.35	10.94	12.04	12.24	0.01	0.00	0.00	2.36	0.00	0.44	0.00		Grass	Sky	Roads	Tree
GV_2436	0.46	52.20	0.15	11.33	25.94	7.26	1.49	0.00	0.00	0.73	0.01	0.42	0.00		Grass	Roads	Tree	Sky
GV_2437	0.33	25.86	3.57	18.22	30.80	10.90	1.34	7.28	0.00	1.57	0.00	0.12	0.00		Roads	Grass	Tree	Sky
GV_2438	0.20	60.31	0.10	7.20	9.54	8.92	3.71	0.03	0.00	0.00	0.00	9.99	0.00		Grass	Gravel	Roads	Sky
GV_2439	1.61	76.82	0.04	0.45	0.03	17.83	3.14	0.08	0.00	0.00	0.00	0.00	0.00		Grass	Sky	Soil	Background
GV_2440	2.06	1.33	0.00	0.00	0.09	41.48	44.25	10.79	0.00	0.00	0.00	0.00	0.00		Soil	Sky	Bare Rock	Background
GV_2441	0.90	15.61	0.12	0.04	0.15	43.26	33.05	6.82	0.00	0.00	0.05	0.00	0.00		Sky	Soil	Grass	Bare Rock
GV_2442	2.90	38.73	0.19	9.84	0.03	25.31	21.71	1.06	0.00	0.00	0.00	0.23	0.00		Grass	Sky	Soil	Tree
GV_2443	6.65	41.65	1.78	29.98	7.69	4.47	2.76	0.53	0.67	0.52	0.51	2.80	0.00		Grass	Tree	Roads	Background
GV_2444	18.03	34.92	0.82	0.66	3.70	30.19	11.64	0.00	0.00	0.00	0.00	0.03	0.01		Grass	Sky	Background	Soil
GV_2445	0.52	36.51	0.00	0.46	19.83	38.91	0.10	0.00	0.00	0.00	0.00	3.67	0.00		Sky	Grass	Roads	Gravel
GV_2446	8.44	17.43	0.01	0.02	0.00	41.32	32.02	0.76	0.00	0.00	0.00	0.00	0.00		Sky	Soil	Grass	Background
GV_2447	10.72	37.24	5.23	42.08	0.24	3.39	0.00	0.00	0.01	0.05	0.07	0.97	0.00		Tree	Grass	Background	Building
GV_2448	0.61	48.97	0.69	26.37	9.23	8.70	1.84	0.22	2.38	0.04	0.23	0.47	0.26		Grass	Tree	Roads	Sky
GV_2449	0.89	34.51	0.09	7.32	9.32	32.35	13.21	1.26	0.00	0.02	0.00	1.00	0.03		Grass	Sky	Soil	Roads
GV_2450	1.00	44.68	0.08	22.51	17.87	9.48	2.12	0.13	0.02	0.20	0.00	1.25	0.66		Grass	Tree	Roads	Sky
GV_2451	2.59	72.42	0.14	20.55	0.12	2.34	0.53	0.00	0.00	0.00	0.11	1.16	0.06		Grass	Tree	Background	Sky
GV_2452	2.03	46.18	0.58	31.63	9.23	4.21	2.23	0.14	0.00	0.17	0.01	3.59	0.00		Grass	Tree	Roads	Sky
GV_2453	0.18	63.85	0.29	6.66	0.01	20.88	7.89	0.05	0.00	0.04	0.00	0.15	0.00		Grass	Sky	Soil	Tree
GV_2454	0.01	3.68	0.00	0.00	0.12	40.67	44.46	11.02	0.05	0.00	0.00	0.00	0.00		Soil	Sky	Bare Rock	Grass
GV_2455	1.32	22.80	0.25	4.68	0.04	29.21	19.53	11.08	10.52	0.03	0.31	0.25	0.00		Sky	Grass	Soil	Bare Rock
GV_2456	0.05	97.55	0.05	0.05	0.82	0.00	1.43	0.00	0.00	0.00	0.00	0.05	0.00		Grass	Soil	Roads	Building
GV_2457	1.96	88.90	0.00	0.02	0.26	0.03	8.60	0.15	0.00	0.00	0.01	0.06	0.00		Grass	Soil	Background	Roads
GV_2458	2.17	96.78	0.13	0.02	0.46	0.34	0.07	0.00	0.00	0.00	0.02	0.00	0.00		Grass	Background	Roads	Sky
GV_2459	10.40	85.66	0.00	0.02	3.05	0.12	0.46	0.22	0.00	0.00	0.00	0.08	0.00		Grass	Background	Roads	Soil
GV_2460	0.44	66.35	0.00	30.24	0.05	1.02	1.04	0.66	0.00	0.00	0.00	0.20	0.00		Grass	Tree	Soil	Sky
GV_2461	0.81	53.46	1.40	29.60	7.69	5.37	0.65	0.00	0.54	0.41	0.01	0.04	0.00		Grass	Tree	Roads	Sky
GV_2462	1.09	39.49	19.05	9.30	5.18	9.07	0.00	0.00	0.00	16.83	0.00	0.00	0.00		Grass	Building	Sidewalk	Tree
GV_2463	1.61	75.00	0.10	7.13	0.07	0.81	14.57	0.15	0.00	0.00	0.23	0.32	0.01		Grass	Soil	Tree	Background
GV_2464	5.52	62.73	17.35	6.65	2.93	0.80	1.23	0.00	0.00	2.74	0.00	0.05	0.00		Grass	Building	Tree	Background
GV_2465	3.54	7.39	0.94	19.67	0.18	31.04	25.64	11.49	0.00	0.00	0.11	0.00	0.00		Sky	Soil	Tree	Bare Rock
GV_2466	3.90	57.33	0.11	34.78	0.00	3.82	0.02	0.00	0.00	0.00	0.00	0.03	0.00		Grass	Tree	Background	Sky
GV_2467	1.67	36.24	0.27	28.28	0.35	33.11	0.03	0.02	0.00	0.00	0.01	0.00	0.03		Grass	Sky	Tree	Background
GV_2468	0.08	58.20	0.24	22.98	0.00	18.49	0.00	0.01	0.00	0.00	0.00	0.00	0.00		Grass	Tree	Sky	Building
GV_2469	4.16	33.85	3.12	2.59	8.79	19.26	26.16	0.07	0.00	0.03	0.33	0.92	0.71		Grass	Soil	Sky	Roads
GV_2470	2.22	40.94	8.37	13.27	12.35	5.60	0.06	0.02	4.83	12.18	0.16	0.00	0.00		Grass	Tree	Roads	Sidewalk
GV_2471	1.52	38.44	8.82	4.30	11.58	12.43	3.58	0.96	0.14	1.36	16.68	0.17	0.04		Grass	Water	Sky	Roads
GV_2472	5.72	2.30	36.25	3.24	34.81	5.53	0.22	0.11	0.26	11.51	0.00	0.05	0.00		Building	Roads	Sidewalk	Background
GV_2473	10.79	3.86	24.80	8.12	35.63	7.13	1.42	2.82	0.00	2.60	0.00	2.84	0.00		Roads	Building	Background	Tree
GV_2474	0.27	53.78	0.17	22.10	0.00	23.66	0.03	0.00	0.00	0.00	0.00	0.00	0.00		Grass	Sky	Tree	Background
GV_2475	0.96	84.58	0.49	12.89	0.00	1.08	0.00	0.00	0.00	0.00	0.00	0.00	0.00		Grass	Tree	Sky	Background
GV_2476	11.13	5.43	40.73	3.12	26.68	5.75	0.05	0.13	0.09	6.79	0.00	0.10	0.00		Building	Roads	Background	Sidewalk

GV_2477	1.28	28.34	1.23	21.79	25.27	4.74	0.23	0.01	0.00	15.94	0.00	0.93	0.24		Grass	Roads	Tree	Sidewalk
GV_2478	1.38	45.46	2.21	32.06	9.62	2.10	2.40	0.00	0.03	4.43	0.01	0.29	0.00		Grass	Tree	Roads	Sidewalk
GV_2479	2.67	42.83	0.18	4.79	17.40	28.05	0.14	0.31	0.14	0.08	0.03	3.34	0.04		Grass	Sky	Roads	Tree
GV_2480	5.31	41.59	1.15	19.62	8.41	15.44	6.72	1.59	0.00	0.00	0.00	0.16	0.00		Grass	Tree	Sky	Roads
GV_2481	0.20	98.84	0.00	0.00	0.27	0.00	0.70	0.00	0.00	0.00	0.00	0.00	0.00		Grass	Soil	Roads	Background
GV_2482	0.00	98.39	0.00	0.00	1.58	0.00	0.02	0.00	0.00	0.00	0.00	0.00	0.00		Grass	Roads	Soil	Water
GV_2483	0.00	96.11	0.00	0.00	3.04	0.00	0.84	0.00	0.00	0.00	0.00	0.00	0.00		Grass	Roads	Soil	Water
GV_2484	0.01	94.57	0.00	0.00	5.13	0.00	0.00	0.00	0.00	0.29	0.00	0.00	0.00		Grass	Roads	Sidewalk	Background
GV_2485	0.04	99.41	0.00	0.00	0.07	0.00	0.48	0.00	0.00	0.00	0.00	0.00	0.00		Grass	Soil	Roads	Background
GV_2486	0.00	100.00	0.00	0.00	0.00	0.00	0.00	0.00	0.00	0.00	0.00	0.00	0.00		Grass	Soil	Background	Background
GV_2487	0.00	77.90	0.00	0.00	18.26	0.00	3.47	0.00	0.00	0.33	0.00	0.04	0.00		Grass	Roads	Soil	Sidewalk
GV_2488	0.08	76.52	0.00	0.00	22.20	0.00	1.06	0.00	0.00	0.00	0.00	0.14	0.00		Grass	Roads	Soil	Gravel
GV_2489	0.00	100.00	0.00	0.00	0.00	0.00	0.00	0.00	0.00	0.00	0.00	0.00	0.00		Grass	Roads	Background	Background
GV_2490	0.00	99.79	0.00	0.00	0.05	0.00	0.16	0.00	0.00	0.00	0.00	0.00	0.00		Grass	Soil	Roads	Background
GV_2491	0.00	43.99	0.00	0.00	25.51	0.00	26.68	3.66	0.00	0.00	0.16	0.01	0.00		Grass	Soil	Roads	Bare Rock
GV_2492	0.00	47.81	0.00	0.00	37.62	0.00	3.42	11.11	0.00	0.00	0.00	0.05	0.00		Grass	Roads	Bare Rock	Soil
GV_2493	0.58	96.66	0.00	0.00	2.75	0.00	0.01	0.00	0.00	0.00	0.01	0.00	0.00		Grass	Roads	Background	Water
GV_2494	0.00	55.95	0.00	0.00	34.92	0.00	9.13	0.00	0.00	0.00	0.00	0.00	0.00		Grass	Roads	Soil	Background
GV_2495	0.00	99.92	0.00	0.00	0.00	0.00	0.07	0.00	0.00	0.00	0.00	0.00	0.00		Grass	Soil	Background	Water
GV_2496	0.03	99.84	0.00	0.00	0.04	0.00	0.08	0.00	0.00	0.00	0.01	0.00	0.00		Grass	Soil	Roads	Background
GV_2497	0.00	97.36	0.00	0.00	1.74	0.00	0.89	0.00	0.00	0.00	0.00	0.00	0.00		Grass	Roads	Soil	Gravel
GV_2498	0.14	99.60	0.00	0.00	0.24	0.00	0.00	0.00	0.00	0.00	0.01	0.00	0.00		Grass	Roads	Background	Water
GV_2499	0.03	99.94	0.00	0.00	0.00	0.00	0.03	0.00	0.00	0.00	0.00	0.00	0.00		Grass	Background	Soil	Building
GV_2500	0.00	53.16	0.00	0.00	24.35	0.00	17.95	4.54	0.00	0.00	0.00	0.00	0.00		Grass	Roads	Soil	Bare Rock
GV_2501	0.11	90.53	0.00	0.00	5.80	0.06	3.22	0.00	0.00	0.00	0.00	0.28	0.00		Grass	Roads	Soil	Gravel
GV_2502	0.14	48.06	0.01	27.69	3.42	2.12	2.38	0.06	0.00	3.88	0.00	12.23	0.00		Grass	Tree	Gravel	Sidewalk
GV_2503	0.48	70.85	0.01	17.03	2.15	3.49	1.63	0.14	0.00	2.08	0.00	2.13	0.02		Grass	Tree	Sky	Roads
GV_2504	15.86	16.33	7.21	17.28	2.74	0.85	5.60	32.89	0.00	0.43	0.00	0.81	0.00		Bare Rock	Tree	Grass	Background
GV_2505	2.36	80.40	1.33	2.77	0.14	1.20	10.56	0.06	0.19	0.01	0.00	0.97	0.03		Grass	Soil	Tree	Background
GV_2506	0.95	63.04	0.55	0.49	1.05	0.29	21.62	0.81	2.97	0.08	0.01	8.08	0.04		Grass	Soil	Gravel	Sand
GV_2507	0.74	50.12	0.00	0.65	0.14	0.47	46.38	1.11	0.00	0.00	0.00	0.38	0.00		Grass	Soil	Bare Rock	Background
GV_2508	3.36	76.88	1.10	8.28	0.03	9.90	0.41	0.00	0.00	0.00	0.00	0.03	0.00		Grass	Sky	Tree	Background
GV_2509	1.31	89.65	1.81	3.76	0.10	1.33	1.23	0.00	0.00	0.52	0.00	0.28	0.00		Grass	Tree	Building	Sky
GV_2510	5.43	82.20	0.80	5.17	0.85	1.12	1.60	0.67	0.00	1.86	0.01	0.30	0.00		Grass	Background	Tree	Sidewalk
GV_2511	0.67	46.05	0.02	24.89	0.08	28.28	0.00	0.00	0.00	0.00	0.00	0.00	0.00		Grass	Sky	Tree	Background
GV_2512	7.92	43.98	10.98	6.83	0.34	15.09	6.41	6.52	0.05	0.61	0.03	0.00	1.25		Grass	Sky	Building	Background
GV_2513	1.32	9.63	0.26	78.95	0.85	4.50	3.32	0.40	0.00	0.00	0.20	0.56	0.00		Tree	Grass	Sky	Soil
GV_2514	2.45	62.55	3.25	14.31	0.20	1.55	12.17	1.48	0.01	0.00	0.47	1.58	0.00		Grass	Tree	Soil	Building
GV_2515	9.58	81.00	0.01	3.19	0.00	0.00	4.14	0.01	1.98	0.11	0.00	0.00	0.00		Grass	Background	Soil	Tree
GV_2516	5.42	22.52	0.23	16.31	0.00	5.77	43.85	2.03	1.74	0.00	0.71	1.41	0.00		Soil	Grass	Tree	Sky
GV_2517	6.03	77.21	0.00	0.02	0.66	9.00	2.83	3.35	0.00	0.83	0.00	0.09	0.01		Grass	Sky	Background	Bare Rock
GV_2518	1.18	68.26	0.76	18.19	0.46	4.87	4.86	0.00	0.00	0.01	0.00	0.89	0.53		Grass	Tree	Sky	Soil
GV_2519	2.16	49.10	0.05	28.84	0.00	19.85	0.00	0.00	0.00	0.00	0.00	0.00	0.00		Grass	Tree	Sky	Background
GV_2520	2.28	4.68	0.12	4.72	7.82	11.12	6.78	10.00	4.91	0.01	45.88	1.67	0.00		Water	Sky	Bare Rock	Roads
GV_2521	2.83	52.39	22.15	0.01	0.20	4.55	0.12	0.00	0.00	17.75	0.00	0.00	0.00		Grass	Building	Sidewalk	Sky
GV_2522	6.37	35.56	0.01	9.38	0.03	0.00	47.94	0.39	0.00	0.00	0.20	0.13	0.00		Soil	Grass	Tree	Background
GV_2523	5.55	33.05	5.11	25.10	2.35	20.67	4.22	0.81	0.00	2.36	0.03	0.41	0.35		Grass	Tree	Sky	Background
GV_2524	0.90	53.71	0.06	24.35	0.00	20.98	0.00	0.01	0.00	0.00	0.00	0.00	0.00		Grass	Tree	Sky	Background
GV_2525	11.69	22.86	0.11	6.59	1.57	27.06	6.85	20.52	0.02	0.00	2.36	0.36	0.00		Sky	Grass	Bare Rock	Background
GV_2526	0.48	34.39	0.01	5.42	0.29	33.62	24.72	0.89	0.16	0.00	0.01	0.01	0.00		Grass	Sky	Soil	Tree
GV_2527	4.04	31.77	15.49	27.95	2.55	9.03	2.23	1.08	0.00	3.62	0.00	2.24	0.01		Grass	Tree	Building	Sky
GV_2528	1.08	48.10	12.57	6.70	5.31	13.81	0.03	0.02	0.00	12.38	0.00	0.00	0.00		Grass	Sky	Building	Sidewalk
GV_2529	1.65	77.09	0.14	20.36	0.00	0.77	0.00	0.00	0.00	0.00	0.00	0.00	0.00		Grass	Tree	Background	Sky
GV_2530	0.36	63.18	0.02	22.69	0.00	13.64	0.03	0.00	0.00	0.00	0.00	0.00	0.08		Grass	Tree	Sky	Background
GV_2531	8.09	14.61	0.88	15.70	4.58	11.78	2.57	2.43	2.05	17.51	18.30	0.01	1.49		Water	Sidewalk	Tree	Grass
GV_2532	2.01	20.07	0.89	22.26	1.16	27.79	6.83	0.34	1.80	6.01	0.00	10.16	0.67		Sky	Tree	Grass	Gravel
GV_2533	0.25	16.06	0.00	1.04	0.46	40.01	34.70	2.50	4.82	0.00	0.08	0.08	0.00		Sky	Soil	Grass	Sand
GV_2534	0.85	10.23	0.04	0.16	0.00	34.90	40.04	12.64	1.14	0.00	0.00	0.00	0.00		Soil	Sky	Bare Rock	Grass
GV_2535	0.27	22.38	0.00	1.94	0.01	31.49	39.06	4.59	0.26	0.00	0.00	0.00	0.00		Soil	Sky	Grass	Bare Rock

GV_2536	0.61	0.01	0.00	0.00	0.00	36.25	61.93	1.18	0.01	0.00	0.00	0.00	0.00		Soil	Sky	Bare Rock	Background
GV_2537	0.09	1.51	0.03	0.31	0.00	35.26	32.62	30.17	0.00	0.00	0.00	0.00	0.00		Sky	Soil	Bare Rock	Grass
GV_2538	1.34	4.47	0.06	3.26	0.00	34.33	44.48	12.02	0.06	0.00	0.00	0.00	0.00		Soil	Sky	Bare Rock	Grass
GV_2539	5.59	8.43	3.79	22.88	29.25	16.91	2.59	2.58	0.87	2.88	4.08	0.15	0.00		Roads	Tree	Sky	Grass
GV_2540	1.89	23.52	0.00	42.25	14.60	0.01	14.57	0.04	0.00	0.00	0.00	3.12	0.00		Tree	Grass	Roads	Soil
GV_2541	2.54	45.51	0.02	28.46	15.40	0.09	5.35	0.12	0.00	0.00	0.03	2.47	0.00		Grass	Tree	Roads	Soil
GV_2542	3.56	17.32	10.45	32.97	10.11	2.90	20.78	0.09	0.00	0.57	0.00	1.26	0.00		Tree	Soil	Grass	Building
GV_2543	0.59	43.07	0.02	32.52	0.03	1.35	14.41	0.02	0.00	0.00	0.00	7.99	0.00		Grass	Tree	Soil	Gravel
GV_2544	0.65	69.15	0.18	10.20	0.00	13.93	5.80	0.00	0.00	0.00	0.00	0.10	0.00		Grass	Sky	Tree	Soil
GV_2545	0.42	85.51	0.10	4.71	0.00	6.12	2.30	0.00	0.00	0.00	0.00	0.82	0.00		Grass	Sky	Tree	Soil
GV_2546	0.11	71.32	0.11	12.24	0.00	15.27	0.46	0.00	0.00	0.00	0.00	0.48	0.00		Grass	Sky	Tree	Gravel
GV_2547	0.72	67.29	0.00	5.28	2.79	0.01	8.47	3.93	0.00	0.00	0.00	11.52	0.00		Grass	Gravel	Soil	Tree
GV_2548	2.70	52.14	0.00	37.61	1.65	4.71	0.88	0.17	0.00	0.00	0.00	0.13	0.00		Grass	Tree	Sky	Background
GV_2549	0.14	72.98	0.02	21.06	0.00	5.75	0.00	0.01	0.00	0.00	0.00	0.05	0.00		Grass	Tree	Sky	Background
GV_2550	4.16	27.75	0.00	57.89	0.00	0.02	9.35	0.04	0.00	0.00	0.00	0.78	0.00		Tree	Grass	Soil	Background
GV_2551	0.25	37.84	0.00	46.24	0.19	1.57	13.76	0.12	0.00	0.00	0.00	0.04	0.00		Tree	Grass	Soil	Sky
GV_2552	1.43	66.68	0.01	16.26	0.00	0.60	12.78	0.03	0.00	0.00	0.00	2.22	0.00		Grass	Tree	Soil	Gravel
GV_2553	0.43	70.24	0.00	12.27	0.00	1.99	9.94	0.00	0.00	0.00	0.01	5.12	0.00		Grass	Tree	Soil	Gravel
GV_2554	0.55	69.73	0.46	17.81	0.01	7.14	2.37	0.00	0.00	0.00	0.00	1.92	0.00		Grass	Tree	Sky	Soil
GV_2555	0.53	64.95	0.02	9.03	0.00	20.07	5.39	0.01	0.00	0.00	0.00	0.01	0.00		Grass	Sky	Tree	Soil
GV_2556	0.83	50.95	0.00	11.22	0.00	0.84	32.67	2.64	0.51	0.02	0.32	0.01	0.00		Grass	Soil	Tree	Bare Rock
GV_2557	2.15	18.14	0.00	46.31	0.00	0.00	29.86	1.96	0.00	0.00	0.00	1.58	0.00		Tree	Soil	Grass	Background
GV_2558	0.77	33.08	0.00	46.30	3.66	0.03	7.23	6.79	0.00	0.00	0.01	2.15	0.00		Tree	Grass	Soil	Bare Rock
GV_2559	2.14	18.85	0.01	65.98	0.00	0.15	8.18	3.19	0.00	0.00	0.01	1.50	0.00		Tree	Grass	Soil	Bare Rock
GV_2560	3.69	54.89	0.00	40.63	0.07	0.01	0.24	0.19	0.00	0.00	0.00	0.27	0.00		Grass	Tree	Background	Gravel
GV_2561	6.52	1.64	31.30	4.39	28.64	10.19	0.17	4.22	0.14	12.73	0.08	0.00	0.00		Building	Roads	Sidewalk	Sky
GV_2562	9.12	13.59	25.29	7.33	17.69	10.23	1.51	0.31	0.00	12.03	0.00	2.91	0.00		Building	Roads	Grass	Sidewalk
GV_2563	3.68	49.76	0.32	27.99	0.22	16.68	0.95	0.04	0.00	0.37	0.00	0.01	0.00		Grass	Tree	Sky	Background
GV_2564	4.18	45.76	1.88	27.96	1.76	14.49	0.65	0.02	0.00	2.24	0.00	1.05	0.00		Grass	Tree	Sky	Background
GV_2565	0.88	19.07	4.52	5.46	24.57	24.84	12.98	0.02	0.00	2.02	0.00	5.65	0.00		Sky	Roads	Grass	Soil
GV_2566	0.00	57.48	0.13	1.30	1.88	12.73	21.13	0.00	0.00	0.00	0.00	5.34	0.00		Grass	Soil	Sky	Gravel
GV_2567	0.04	42.44	0.11	10.82	0.21	16.64	29.49	0.01	0.00	0.21	0.00	0.03	0.00		Grass	Soil	Sky	Tree
GV_2568	0.05	45.25	0.08	6.35	0.83	23.57	8.55	0.00	0.00	0.10	0.00	15.22	0.00		Grass	Sky	Gravel	Soil
GV_2569	0.13	45.98	0.41	7.14	3.15	24.47	14.56	0.00	0.45	0.00	0.00	3.70	0.00		Grass	Sky	Soil	Tree
GV_2570	0.31	36.58	0.02	19.67	1.04	27.45	8.41	0.03	0.00	0.01	0.00	6.49	0.00		Grass	Sky	Tree	Soil
GV_2571	0.10	23.45	0.07	53.95	14.03	4.00	1.35	0.00	0.00	0.00	0.00	3.04	0.00		Tree	Grass	Roads	Sky
GV_2572	3.36	47.79	2.36	16.50	9.58	7.58	8.95	0.46	0.09	0.71	0.03	2.58	0.00		Grass	Tree	Roads	Soil
GV_2573	0.03	99.51	0.00	0.00	0.41	0.00	0.00	0.00	0.00	0.00	0.04	0.00	0.00		Grass	Roads	Water	Background
GV_2574	0.00	99.70	0.00	0.00	0.00	0.00	0.30	0.00	0.00	0.00	0.00	0.00	0.00		Grass	Soil	Background	Building
GV_2575	0.00	71.75	0.00	0.00	0.00	0.00	28.25	0.00	0.00	0.00	0.00	0.00	0.00		Grass	Soil	Background	Background
GV_2576	0.00	99.97	0.00	0.00	0.00	0.00	0.03	0.00	0.00	0.00	0.00	0.00	0.00		Grass	Soil	Water	Background
GV_2577	2.47	41.21	21.59	4.47	5.08	18.27	0.13	0.05	0.00	6.36	0.00	0.38	0.00		Grass	Building	Sky	Sidewalk
GV_2578	4.96	65.37	0.25	19.07	1.44	0.08	6.02	0.05	0.23	0.72	0.00	1.80	0.00		Grass	Tree	Soil	Background
GV_2579	2.36	27.19	8.74	18.65	5.39	14.44	0.04	0.17	0.00	22.79	0.00	0.06	0.17		Grass	Sidewalk	Tree	Sky
GV_2580	2.37	32.55	9.09	5.48	5.53	12.81	0.07	0.00	0.00	31.81	0.14	0.15	0.00		Grass	Sidewalk	Sky	Building
GV_2581	0.69	52.86	1.81	27.96	2.98	6.53	0.06	0.00	0.00	7.10	0.00	0.00	0.00		Grass	Tree	Sidewalk	Sky
GV_2582	4.50	46.84	0.13	0.17	12.36	33.70	1.51	0.12	0.00	0.02	0.30	0.07	0.28		Grass	Sky	Roads	Background
GV_2583	1.34	61.93	13.17	7.40	0.11	15.43	0.00	0.00	0.00	0.31	0.01	0.30	0.00		Grass	Sky	Building	Tree
GV_2584	1.76	88.64	0.05	0.28	7.52	0.18	0.26	0.01	0.00	1.23	0.06	0.00	0.00		Grass	Roads	Background	Sidewalk
GV_2585	0.49	46.05	0.00	0.11	45.24	0.04	5.68	0.03	0.00	2.31	0.05	0.00	0.00		Grass	Roads	Soil	Sidewalk
GV_2586	10.13	50.23	1.68	9.14	4.61	0.73	1.23	1.68	0.00	18.89	0.00	1.68	0.00		Grass	Sidewalk	Background	Tree
GV_2587	1.10	47.76	15.12	5.71	2.08	18.00	0.12	0.15	0.00	9.96	0.00	0.00	0.00		Grass	Sky	Building	Sidewalk
GV_2588	0.00	100.00	0.00	0.00	0.00	0.00	0.00	0.00	0.00	0.00	0.00	0.00	0.00		Grass	Background	Building	Building
GV_2589	0.07	99.36	0.00	0.00	0.03	0.00	0.51	0.00	0.00	0.00	0.03	0.00	0.00		Grass	Soil	Background	Water
GV_2590	0.38	58.57	0.01	0.00	35.09	0.02	3.90	0.00	0.00	0.10	0.00	1.93	0.00		Grass	Roads	Soil	Gravel
GV_2591	0.22	99.75	0.00	0.00	0.00	0.00	0.00	0.00	0.00	0.00	0.02	0.00	0.00		Grass	Background	Water	Soil
GV_2592	2.02	86.05	0.00	0.00	5.59	0.00	6.31	0.01	0.00	0.02	0.00	0.00	0.00		Grass	Soil	Roads	Background
GV_2593	0.00	99.97	0.00	0.00	0.00	0.00	0.00	0.00	0.00	0.00	0.03	0.00	0.00		Grass	Water	Soil	Sand
GV_2594	0.00	99.98	0.00	0.00	0.00	0.00	0.02	0.00	0.00	0.00	0.00	0.00	0.00		Grass	Soil	Background	Building

GV_2595	6.05	39.45	18.34	15.01	3.11	0.88	0.24	0.14	0.00	16.76	0.00	0.03	0.00		Grass	Building	Sidewalk	Tree
GV_2596	1.25	74.77	0.00	11.27	0.01	12.16	0.35	0.00	0.00	0.00	0.00	0.18	0.00		Grass	Sky	Tree	Background
GV_2597	1.64	36.70	0.26	11.03	5.91	24.39	2.60	0.98	0.06	1.98	7.31	5.54	1.60		Grass	Sky	Tree	Water
GV_2598	1.03	2.96	0.04	83.32	0.00	0.19	11.22	0.08	0.00	0.00	0.09	1.05	0.02		Tree	Soil	Grass	Gravel
GV_2599	0.46	2.85	0.00	1.64	35.90	25.28	0.48	14.89	17.89	0.01	0.03	0.56	0.01		Roads	Sky	Sand	Bare Rock
GV_2600	0.94	5.73	0.19	79.78	3.27	0.13	6.03	0.10	0.00	0.00	0.08	3.76	0.00		Tree	Soil	Grass	Gravel
GV_2601	1.24	53.67	17.62	9.20	0.36	17.37	0.30	0.00	0.00	0.22	0.01	0.00	0.01		Grass	Building	Sky	Tree
GV_2602	2.21	78.81	0.19	1.50	0.02	0.19	16.80	0.09	0.15	0.04	0.00	0.00	0.00		Grass	Soil	Background	Tree
GV_2603	6.47	27.36	16.18	8.89	2.35	37.94	0.21	0.05	0.00	0.24	0.00	0.31	0.00		Sky	Grass	Building	Tree
GV_2604	6.35	1.55	0.08	47.62	8.77	35.57	0.03	0.01	0.00	0.00	0.00	0.01	0.01		Tree	Sky	Roads	Background
GV_2605	0.42	54.08	0.00	33.33	0.00	0.74	11.29	0.12	0.00	0.00	0.01	0.00	0.00		Grass	Tree	Soil	Sky
GV_2606	1.37	86.29	0.00	0.34	0.53	5.51	5.80	0.00	0.01	0.00	0.00	0.04	0.11		Grass	Soil	Sky	Background
GV_2607	0.21	76.21	0.00	20.77	0.00	0.01	2.50	0.02	0.00	0.00	0.01	0.26	0.00		Grass	Tree	Soil	Gravel
GV_2608	0.43	77.82	0.00	18.62	0.06	0.93	1.68	0.15	0.00	0.00	0.00	0.31	0.00		Grass	Tree	Soil	Sky
GV_2609	0.08	83.00	0.00	5.99	0.04	0.00	8.14	0.01	0.00	0.00	0.29	2.45	0.00		Grass	Soil	Tree	Gravel
GV_2610	0.63	29.77	0.71	5.64	28.30	24.77	7.58	0.01	0.00	0.23	0.00	1.89	0.47		Grass	Roads	Sky	Soil
GV_2611	0.04	33.05	0.04	4.05	44.66	16.78	0.20	0.00	0.00	0.00	0.00	0.99	0.19		Roads	Grass	Sky	Tree
GV_2612	5.96	25.65	0.35	14.88	2.37	6.10	2.23	30.91	1.76	0.00	6.24	3.55	0.00		Bare Rock	Grass	Tree	Water
GV_2613	0.06	40.35	0.09	0.95	16.79	38.31	0.80	0.00	0.00	0.07	0.00	2.58	0.00		Grass	Sky	Roads	Gravel
GV_2614	0.28	84.82	0.00	0.00	5.00	0.00	3.07	0.01	0.09	6.71	0.01	0.00	0.00		Grass	Sidewalk	Roads	Soil
GV_2615	0.00	99.62	0.00	0.00	0.00	0.00	0.38	0.00	0.00	0.00	0.00	0.00	0.00		Grass	Soil	Background	Background
GV_2616	0.00	98.88	0.00	0.00	0.26	0.00	0.00	0.00	0.00	0.86	0.00	0.00	0.00		Grass	Sidewalk	Roads	Sky
GV_2617	0.62	57.78	8.16	11.33	0.01	22.07	0.00	0.00	0.00	0.02	0.00	0.00	0.01		Grass	Sky	Tree	Building
GV_2618	1.64	34.56	0.11	31.54	8.12	18.49	0.11	0.00	0.00	4.97	0.00	0.19	0.27		Grass	Tree	Sky	Roads
GV_2619	0.19	99.66	0.00	0.00	0.00	0.04	0.00	0.00	0.00	0.00	0.11	0.00	0.00		Grass	Background	Water	Sky
GV_2620	0.21	85.59	0.00	0.00	10.76	0.02	2.04	0.00	0.01	0.04	1.14	0.18	0.00		Grass	Roads	Soil	Water
GV_2621	0.85	94.77	0.00	0.00	0.00	0.00	4.23	0.15	0.00	0.00	0.00	0.00	0.00		Grass	Soil	Background	Bare Rock
GV_2622	0.27	90.53	0.03	2.67	0.00	0.03	6.14	0.09	0.00	0.00	0.02	0.20	0.00		Grass	Soil	Tree	Background
GV_2623	0.18	93.20	0.00	1.70	0.00	0.00	4.79	0.01	0.00	0.00	0.00	0.12	0.00		Grass	Soil	Tree	Background
GV_2624	0.00	100.00	0.00	0.00	0.00	0.00	0.00	0.00	0.00	0.00	0.00	0.00	0.00		Grass	Background	Background	Background
GV_2625	0.12	97.91	0.00	0.00	0.00	0.00	1.97	0.00	0.00	0.00	0.00	0.00	0.00		Grass	Soil	Background	Building
GV_2626	0.17	99.81	0.00	0.00	0.00	0.00	0.02	0.00	0.00	0.00	0.00	0.00	0.00		Grass	Background	Soil	Building
GV_2627	0.08	94.89	0.00	0.17	0.00	0.00	4.59	0.00	0.00	0.00	0.00	0.27	0.00		Grass	Soil	Gravel	Tree
GV_2628	8.19	46.58	0.88	6.09	26.78	11.31	0.00	0.00	0.02	0.15	0.00	0.00	0.00		Grass	Roads	Sky	Background
GV_2629	1.18	77.83	0.83	9.61	0.65	4.16	3.61	0.01	0.00	0.00	0.00	2.13	0.00		Grass	Tree	Sky	Soil
GV_2630	7.20	22.89	1.44	27.01	14.66	3.28	20.93	1.45	0.00	0.13	0.03	0.99	0.00		Tree	Grass	Soil	Roads
GV_2631	29.41	15.75	20.72	6.37	10.87	5.78	1.57	0.00	0.01	1.02	0.00	8.49	0.00		Background	Building	Grass	Roads
GV_2632	11.86	11.28	32.55	15.57	12.36	3.09	0.57	3.92	0.01	6.42	0.00	2.38	0.00		Building	Tree	Roads	Background
GV_2633	3.84	1.40	40.65	5.16	17.94	10.79	0.24	5.10	9.78	5.05	0.00	0.02	0.01		Building	Roads	Sky	Sand
GV_2634	9.49	3.52	56.74	6.23	2.03	13.37	0.04	1.77	0.00	6.74	0.00	0.07	0.00		Building	Sky	Background	Sidewalk
GV_2635	0.19	8.41	0.03	0.07	0.17	39.07	43.14	8.53	0.37	0.00	0.00	0.00	0.01		Soil	Sky	Bare Rock	Grass
GV_2636	0.13	0.09	0.02	0.00	0.39	38.92	56.47	0.11	3.85	0.00	0.01	0.00	0.00		Soil	Sky	Sand	Roads
GV_2637	0.06	0.02	0.07	0.01	0.01	36.52	57.18	6.12	0.02	0.00	0.00	0.00	0.00		Soil	Sky	Bare Rock	Building
GV_2638	8.70	75.29	0.31	7.22	1.05	0.53	3.55	0.96	0.00	2.34	0.00	0.04	0.00		Grass	Background	Tree	Soil
GV_2639	1.71	30.84	0.00	18.53	0.94	9.47	14.37	20.99	1.38	0.00	1.20	0.57	0.00		Grass	Bare Rock	Tree	Soil
GV_2640	1.04	8.31	3.46	25.15	2.89	15.49	42.73	0.84	0.02	0.00	0.03	0.04	0.00		Soil	Tree	Sky	Grass
GV_2641	0.00	0.00	0.00	0.00	0.00	45.10	54.89	0.00	0.00	0.00	0.00	0.00	0.00		Soil	Sky	Grass	Tree
GV_2642	0.77	90.41	0.20	0.00	0.21	0.07	8.24	0.00	0.00	0.06	0.00	0.00	0.03		Grass	Soil	Background	Roads
GV_2643	0.88	52.39	6.21	6.34	0.19	31.87	0.25	0.28	0.02	0.00	1.49	0.00	0.08		Grass	Sky	Tree	Building
GV_2644	7.74	45.87	22.16	7.11	0.67	12.55	1.68	0.02	0.00	1.02	0.85	0.12	0.22		Grass	Building	Sky	Background
GV_2645	2.40	1.85	37.75	7.78	20.42	8.04	0.22	4.51	5.04	11.27	0.08	0.64	0.00		Building	Roads	Sidewalk	Sky
GV_2646	20.49	4.51	32.93	7.96	14.90	2.59	0.03	0.03	0.00	14.03	0.00	2.53	0.00		Building	Background	Roads	Sidewalk
GV_2647	11.61	3.33	23.94	3.11	7.16	17.06	2.08	15.79	0.53	6.00	9.07	0.32	0.00		Building	Sky	Bare Rock	Background
GV_2648	5.56	3.88	33.52	9.21	24.94	7.63	5.97	6.04	0.00	1.40	0.01	1.83	0.00		Building	Roads	Tree	Sky
GV_2649	9.56	9.90	22.64	21.12	15.42	4.13	0.23	2.47	0.07	12.82	1.22	0.32	0.10		Building	Tree	Roads	Sidewalk
GV_2650	10.20	0.12	73.26	0.37	0.17	12.25	0.00	0.13	0.00	3.51	0.00	0.00	0.00		Building	Sky	Background	Sidewalk
GV_2651	3.96	67.98	0.03	1.55	24.26	0.41	0.75	0.00	0.00	0.04	0.00	1.03	0.00		Grass	Roads	Background	Tree
GV_2652	1.99	38.32	2.97	22.62	0.54	28.94	1.05	0.02	1.17	0.95	0.00	0.21	1.22		Grass	Sky	Tree	Building
GV_2653	1.16	83.75	0.23	11.94	0.06	1.63	1.13	0.01	0.00	0.08	0.00	0.00	0.01		Grass	Tree	Sky	Background

GV_2654	0.63	85.07	4.42	5.80	0.12	3.83	0.00	0.01	0.00	0.10	0.00	0.00	0.00		Grass	Tree	Building	Sky
GV_2655	0.00	0.00	0.00	0.00	0.00	35.74	64.26	0.00	0.00	0.00	0.00	0.00	0.00		Soil	Sky	Grass	Background
GV_2656	0.66	24.09	0.01	9.37	1.48	6.41	38.46	14.33	2.05	0.01	0.70	2.43	0.00		Soil	Grass	Bare Rock	Tree
GV_2657	0.12	22.32	0.00	0.02	0.15	18.33	58.53	0.09	0.35	0.00	0.08	0.00	0.00		Soil	Grass	Sky	Sand
GV_2658	0.01	0.13	0.07	0.05	0.00	22.53	73.63	3.58	0.00	0.00	0.00	0.00	0.00		Soil	Sky	Bare Rock	Grass
GV_2659	0.12	0.02	0.00	0.00	0.00	15.70	83.75	0.42	0.00	0.00	0.00	0.00	0.00		Soil	Sky	Bare Rock	Background
GV_2660	0.10	74.81	0.00	0.01	0.00	20.52	1.49	0.83	2.23	0.00	0.00	0.01	0.00		Grass	Sky	Sand	Soil
GV_2661	0.67	5.20	1.96	0.53	3.30	36.03	45.04	0.45	2.43	4.38	0.00	0.01	0.00		Soil	Sky	Grass	Sidewalk
GV_2662	0.08	19.73	0.01	8.25	0.06	26.08	43.75	1.87	0.03	0.00	0.00	0.15	0.00		Soil	Sky	Grass	Tree
GV_2663	12.39	43.31	24.52	2.76	1.88	11.00	0.26	0.00	0.00	2.38	1.23	0.24	0.03		Grass	Building	Background	Sky
GV_2664	0.03	85.46	0.00	0.00	1.64	0.00	0.77	0.16	0.83	0.42	8.99	1.70	0.00		Grass	Water	Gravel	Roads
GV_2665	0.00	99.72	0.00	0.00	0.05	0.00	0.00	0.00	0.00	0.00	0.24	0.00	0.00		Grass	Water	Roads	Background
GV_2666	1.10	58.49	0.81	18.69	10.16	6.75	1.02	0.00	1.03	1.01	0.78	0.16	0.01		Grass	Tree	Roads	Sky
GV_2667	0.64	80.66	0.06	11.76	0.03	2.54	4.13	0.01	0.00	0.13	0.00	0.00	0.03		Grass	Tree	Soil	Sky
GV_2668	0.88	3.02	4.61	2.18	0.04	5.75	70.70	4.35	6.99	0.85	0.62	0.01	0.00		Soil	Sand	Sky	Building
GV_2669	3.77	16.29	1.95	51.08	20.60	1.67	0.06	0.04	0.00	0.21	0.04	4.31	0.00		Tree	Roads	Grass	Gravel
GV_2670	0.01	0.00	0.00	0.00	0.00	18.31	78.04	3.61	0.03	0.00	0.00	0.00	0.00		Soil	Sky	Bare Rock	Sand
GV_2671	0.17	0.03	0.01	0.11	0.00	30.82	66.13	2.73	0.00	0.00	0.00	0.00	0.00		Soil	Sky	Bare Rock	Background
GV_2672	0.06	0.02	0.00	0.03	0.00	40.15	49.71	10.03	0.00	0.00	0.00	0.00	0.00		Soil	Sky	Bare Rock	Background
GV_2673	2.07	3.78	0.00	0.19	0.00	22.19	57.25	14.10	0.41	0.00	0.00	0.00	0.00		Soil	Sky	Bare Rock	Grass
GV_2674	0.38	2.26	0.00	0.00	0.00	40.89	54.78	0.00	1.68	0.00	0.00	0.00	0.00		Soil	Sky	Grass	Sand
GV_2675	0.18	0.02	0.01	0.02	0.00	38.46	59.85	1.47	0.00	0.00	0.00	0.00	0.00		Soil	Sky	Bare Rock	Background
GV_2676	0.02	99.97	0.00	0.00	0.00	0.00	0.00	0.00	0.00	0.00	0.00	0.00	0.00		Grass	Background	Soil	Building
GV_2677	0.03	99.97	0.00	0.00	0.00	0.00	0.00	0.00	0.00	0.00	0.00	0.00	0.00		Grass	Background	Building	Building
GV_2678	0.17	53.41	0.00	0.00	42.96	0.00	3.43	0.03	0.00	0.00	0.00	0.00	0.00		Grass	Roads	Soil	Background
GV_2679	0.00	99.99	0.00	0.00	0.00	0.00	0.01	0.00	0.00	0.00	0.00	0.00	0.00		Grass	Soil	Background	Background
GV_2680	0.00	94.48	0.00	0.00	0.37	0.00	4.07	0.00	0.00	0.00	0.01	1.08	0.00		Grass	Soil	Gravel	Roads
GV_2681	1.33	46.17	0.00	0.22	2.66	0.01	49.18	0.45	0.00	0.00	0.00	0.00	0.00		Soil	Grass	Roads	Background
GV_2682	1.18	82.65	0.00	0.00	0.69	0.00	15.46	0.02	0.00	0.00	0.00	0.00	0.00		Grass	Soil	Background	Roads
GV_2683	0.01	99.43	0.00	0.00	0.00	0.00	0.54	0.00	0.00	0.00	0.00	0.03	0.00		Grass	Soil	Gravel	Background
GV_2684	0.41	18.83	0.00	0.06	0.13	0.00	78.57	2.01	0.00	0.00	0.00	0.00	0.00		Soil	Grass	Bare Rock	Background
GV_2685	0.06	58.22	0.00	0.00	0.00	0.00	41.71	0.00	0.00	0.00	0.00	0.00	0.00		Grass	Soil	Background	Gravel
GV_2686	0.00	99.99	0.00	0.00	0.00	0.00	0.00	0.00	0.00	0.00	0.01	0.00	0.00		Grass	Water	Background	Background
GV_2687	0.15	99.16	0.00	0.00	0.00	0.00	0.67	0.00	0.00	0.00	0.02	0.00	0.00		Grass	Soil	Background	Water
GV_2688	0.00	57.28	0.00	0.00	0.18	0.00	42.37	0.09	0.00	0.00	0.00	0.08	0.00		Grass	Soil	Roads	Bare Rock
GV_2689	0.22	95.68	0.00	0.00	0.70	0.00	3.01	0.00	0.02	0.00	0.36	0.01	0.00		Grass	Soil	Roads	Water
GV_2690	2.51	57.58	0.00	0.19	0.00	0.00	39.53	0.00	0.00	0.00	0.00	0.19	0.00		Grass	Soil	Background	Tree
GV_2691	0.43	62.20	0.00	1.93	0.00	0.01	35.13	0.00	0.00	0.00	0.00	0.31	0.00		Grass	Soil	Tree	Background
GV_2692	0.82	81.99	0.00	0.73	0.00	0.00	10.50	0.00	0.00	0.00	0.02	5.94	0.00		Grass	Soil	Gravel	Background
GV_2693	0.54	86.86	0.00	0.05	1.07	0.00	11.38	0.00	0.00	0.00	0.00	0.10	0.00		Grass	Soil	Roads	Background
GV_2694	0.07	99.90	0.00	0.00	0.00	0.00	0.03	0.00	0.00	0.00	0.01	0.00	0.00		Grass	Background	Soil	Water
GV_2695	1.07	63.65	0.00	0.34	2.64	0.00	31.57	0.72	0.00	0.00	0.00	0.02	0.00		Grass	Soil	Roads	Background
GV_2696	1.12	94.40	0.00	0.00	0.00	0.00	4.47	0.00	0.00	0.00	0.00	0.00	0.00		Grass	Soil	Background	Gravel
GV_2697	0.13	74.87	0.00	0.00	0.50	0.00	23.27	0.08	0.00	0.82	0.00	0.33	0.00		Grass	Soil	Sidewalk	Roads
GV_2698	0.56	63.57	0.00	0.29	0.23	0.00	35.09	0.04	0.00	0.00	0.00	0.23	0.00		Grass	Soil	Background	Tree
GV_2699	0.07	99.10	0.00	0.03	0.00	0.00	0.80	0.00	0.00	0.00	0.00	0.00	0.00		Grass	Soil	Background	Tree
GV_2700	0.00	99.99	0.00	0.00	0.00	0.00	0.01	0.00	0.00	0.00	0.00	0.00	0.00		Grass	Soil	Background	Background
GV_2701	0.27	99.50	0.00	0.00	0.00	0.00	0.23	0.00	0.00	0.00	0.00	0.00	0.00		Grass	Background	Soil	Building
GV_2702	0.02	93.53	0.00	0.00	0.01	0.00	6.40	0.00	0.00	0.00	0.02	0.02	0.00		Grass	Soil	Water	Gravel
GV_2703	0.28	99.68	0.00	0.00	0.00	0.00	0.04	0.00	0.00	0.00	0.00	0.00	0.00		Grass	Background	Soil	Water
GV_2704	0.34	36.34	0.02	0.02	42.90	0.00	12.86	7.50	0.00	0.00	0.02	0.00	0.00		Roads	Grass	Soil	Bare Rock
GV_2705	2.25	51.26	0.00	0.05	2.19	0.00	44.16	0.01	0.00	0.00	0.00	0.08	0.00		Grass	Soil	Background	Roads
GV_2706	8.99	1.84	32.80	2.85	28.81	10.32	0.01	0.95	0.01	11.78	1.63	0.01	0.00		Building	Roads	Sidewalk	Sky
GV_2707	2.38	38.82	3.62	26.21	16.96	10.70	0.02	0.15	0.01	0.66	0.46	0.02	0.00		Grass	Tree	Roads	Sky
GV_2708	2.50	48.41	10.70	7.82	0.29	29.37	0.82	0.00	0.00	0.00	0.00	0.08	0.00		Grass	Sky	Building	Tree
GV_2709	0.73	61.38	0.00	0.40	2.41	18.33	10.53	0.01	4.87	0.00	1.09	0.00	0.24		Grass	Sky	Soil	Sand
GV_2710	0.74	2.97	0.00	0.00	33.47	0.00	58.27	0.00	0.04	0.00	0.00	4.50	0.00		Soil	Roads	Gravel	Grass
GV_2711	2.80	7.93	0.60	1.63	0.02	14.68	22.23	41.53	4.00	0.00	0.29	4.28	0.00		Bare Rock	Soil	Sky	Grass
GV_2712	0.14	1.43	0.00	0.00	0.07	40.98	55.18	0.14	2.05	0.00	0.00	0.00	0.00		Soil	Sky	Sand	Grass

GV_2713	3.49	2.90	2.37	4.94	0.13	26.54	39.25	20.00	0.00	0.00	0.27	0.11	0.00		Soil	Sky	Bare Rock	Tree
GV_2714	0.61	2.19	0.90	2.94	0.00	8.87	27.71	8.25	45.91	0.00	0.89	1.73	0.00		Sand	Soil	Sky	Bare Rock
GV_2715	0.11	5.57	9.13	0.93	0.06	6.54	60.37	1.17	15.89	0.23	0.00	0.00	0.01		Soil	Sand	Building	Sky
GV_2716	1.77	53.10	15.18	10.53	0.92	17.72	0.02	0.00	0.00	0.77	0.00	0.00	0.00		Grass	Sky	Building	Tree
GV_2717	0.50	10.98	0.08	1.06	0.00	0.00	29.17	58.22	0.00	0.00	0.00	0.00	0.00		Bare Rock	Soil	Grass	Tree
GV_2718	0.00	99.99	0.00	0.00	0.00	0.00	0.00	0.00	0.00	0.00	0.00	0.00	0.00		Grass	Water	Soil	Background
GV_2719	0.06	84.14	0.00	0.00	1.68	0.00	11.27	2.72	0.12	0.00	0.01	0.00	0.00		Grass	Soil	Bare Rock	Roads
GV_2720	0.00	100.00	0.00	0.00	0.00	0.00	0.00	0.00	0.00	0.00	0.00	0.00	0.00		Grass	Water	Background	Background
GV_2721	0.12	99.88	0.00	0.00	0.00	0.00	0.00	0.00	0.00	0.00	0.00	0.00	0.00		Grass	Background	Building	Building
GV_2722	0.36	63.20	0.00	0.00	35.82	0.00	0.07	0.55	0.00	0.00	0.00	0.00	0.00		Grass	Roads	Bare Rock	Background
GV_2723	0.00	99.94	0.00	0.00	0.00	0.00	0.06	0.00	0.00	0.00	0.00	0.00	0.00		Grass	Soil	Background	Background
GV_2724	0.18	99.82	0.00	0.00	0.01	0.00	0.00	0.00	0.00	0.00	0.00	0.00	0.00		Grass	Background	Roads	Building
GV_2725	0.00	2.29	0.00	0.00	0.06	0.00	35.74	61.91	0.00	0.00	0.00	0.00	0.00		Bare Rock	Soil	Grass	Roads
GV_2726	0.00	0.27	0.00	0.00	87.95	0.00	0.00	11.78	0.00	0.00	0.00	0.00	0.00		Roads	Bare Rock	Grass	Background
GV_2727	0.31	98.91	0.00	0.00	0.00	0.00	0.78	0.00	0.00	0.00	0.00	0.00	0.00		Grass	Soil	Background	Roads
GV_2728	0.01	3.16	0.00	0.00	53.25	0.00	1.50	42.04	0.00	0.05	0.00	0.00	0.00		Roads	Bare Rock	Grass	Soil
GV_2729	0.00	58.55	0.00	0.00	0.09	0.00	31.79	9.50	0.07	0.00	0.00	0.00	0.00		Grass	Soil	Bare Rock	Roads
GV_2730	0.20	75.23	0.00	0.00	0.00	0.00	24.41	0.03	0.00	0.00	0.00	0.13	0.00		Grass	Soil	Background	Gravel
GV_2731	0.70	72.02	0.00	0.00	0.00	0.00	25.97	1.30	0.00	0.00	0.00	0.01	0.00		Grass	Soil	Bare Rock	Background
GV_2732	1.83	31.17	0.00	0.01	0.00	0.00	66.62	0.26	0.00	0.00	0.00	0.11	0.00		Soil	Grass	Background	Bare Rock
GV_2733	0.54	87.82	0.00	0.00	0.00	0.00	11.44	0.00	0.00	0.00	0.00	0.19	0.00		Grass	Soil	Background	Gravel
GV_2734	0.32	91.23	0.00	0.00	0.58	0.01	7.26	0.00	0.00	0.56	0.03	0.00	0.00		Grass	Soil	Roads	Sidewalk
GV_2735	0.44	30.18	0.00	0.01	0.00	0.01	60.31	8.98	0.00	0.00	0.08	0.00	0.00		Soil	Grass	Bare Rock	Background
GV_2736	1.22	83.20	0.00	0.00	0.82	0.00	14.66	0.00	0.00	0.00	0.00	0.11	0.00		Grass	Soil	Background	Roads
GV_2737	0.12	93.70	0.00	0.00	0.01	0.00	6.16	0.00	0.00	0.00	0.00	0.00	0.00		Grass	Soil	Background	Roads
GV_2738	0.00	27.93	0.00	0.00	0.06	0.00	71.84	0.16	0.00	0.00	0.00	0.00	0.00		Soil	Grass	Bare Rock	Roads
GV_2739	0.02	99.98	0.00	0.00	0.00	0.00	0.01	0.00	0.00	0.00	0.00	0.00	0.00		Grass	Background	Soil	Building
GV_2740	0.00	99.94	0.00	0.00	0.00	0.00	0.06	0.00	0.00	0.00	0.00	0.00	0.00		Grass	Soil	Background	Background
GV_2741	0.00	84.09	0.00	0.00	0.61	0.00	15.28	0.01	0.00	0.00	0.00	0.01	0.00		Grass	Soil	Roads	Gravel
GV_2742	0.00	99.75	0.00	0.00	0.01	0.00	0.24	0.00	0.00	0.00	0.00	0.00	0.00		Grass	Soil	Roads	Background
GV_2743	2.99	29.61	0.00	0.07	0.00	0.00	59.24	8.00	0.00	0.00	0.00	0.09	0.00		Soil	Grass	Bare Rock	Background
GV_2744	0.61	60.84	0.00	0.05	0.02	0.00	38.11	0.34	0.00	0.00	0.00	0.03	0.00		Grass	Soil	Background	Bare Rock
GV_2745	0.96	79.95	0.02	1.20	0.00	0.19	17.52	0.11	0.00	0.00	0.02	0.02	0.00		Grass	Soil	Tree	Background
GV_2746	0.00	100.00	0.00	0.00	0.00	0.00	0.00	0.00	0.00	0.00	0.00	0.00	0.00		Grass	Background	Background	Background
GV_2747	0.14	70.26	0.00	0.18	0.00	0.00	28.55	0.43	0.00	0.00	0.00	0.44	0.00		Grass	Soil	Gravel	Bare Rock
GV_2748	0.72	80.18	0.00	0.00	0.00	0.00	19.03	0.06	0.00	0.00	0.00	0.00	0.00		Grass	Soil	Background	Bare Rock
GV_2749	1.40	45.28	0.23	38.98	1.96	10.66	0.03	0.00	0.00	0.01	1.37	0.09	0.00		Grass	Tree	Sky	Roads
GV_2750	5.13	23.03	4.02	34.31	16.19	2.80	10.18	0.53	0.04	3.02	0.00	0.74	0.00		Tree	Grass	Roads	Soil
GV_2751	4.63	22.18	1.21	52.46	9.55	0.90	4.90	0.35	0.01	0.85	0.01	2.95	0.00		Tree	Grass	Roads	Soil
GV_2752	19.35	17.12	12.55	29.30	10.44	2.16	4.94	0.01	0.00	3.62	0.01	0.49	0.00		Tree	Background	Grass	Building
GV_2753	1.03	98.44	0.00	0.52	0.00	0.01	0.00	0.00	0.00	0.00	0.00	0.00	0.00		Grass	Background	Tree	Sky
GV_2754	0.77	33.51	9.24	31.93	6.90	8.22	3.70	2.60	0.00	2.50	0.01	0.61	0.00		Grass	Tree	Building	Sky
GV_2755	8.44	20.21	7.77	24.96	0.00	11.06	0.08	0.00	0.00	27.48	0.00	0.00	0.00		Sidewalk	Tree	Grass	Sky
GV_2756	5.28	23.27	23.95	15.83	0.07	13.20	0.58	0.03	0.00	14.60	0.00	3.20	0.00		Building	Grass	Tree	Sidewalk
GV_2757	0.30	0.00	0.01	0.00	1.10	38.40	58.60	0.00	1.59	0.00	0.00	0.00	0.00		Soil	Sky	Sand	Roads
GV_2758	0.14	0.28	0.05	0.00	4.56	39.39	54.93	0.63	0.02	0.00	0.00	0.00	0.00		Soil	Sky	Roads	Bare Rock
GV_2759	0.91	0.01	0.00	0.00	0.83	16.07	82.14	0.00	0.04	0.00	0.00	0.00	0.00		Soil	Sky	Background	Roads
GV_2760	0.92	0.00	0.00	0.00	1.61	39.91	57.56	0.00	0.00	0.00	0.00	0.00	0.00		Soil	Sky	Roads	Background
GV_2761	1.05	39.90	16.36	5.36	0.69	23.18	0.01	0.02	0.00	13.42	0.00	0.00	0.00		Grass	Sky	Building	Sidewalk
GV_2762	0.97	54.77	1.23	16.47	0.54	20.49	3.45	0.02	0.01	0.00	0.00	1.81	0.24		Grass	Sky	Tree	Soil
GV_2763	16.46	10.20	25.77	17.74	14.27	10.23	0.80	0.00	0.00	2.33	0.00	2.20	0.00		Building	Tree	Background	Roads
GV_2764	13.94	12.33	7.27	21.75	22.19	6.09	7.39	0.41	0.02	2.87	0.00	5.73	0.00		Roads	Tree	Background	Grass
GV_2765	1.45	16.09	8.74	18.22	34.73	15.97	1.81	0.06	0.00	2.19	0.00	0.56	0.17		Roads	Tree	Grass	Sky
GV_2766	1.19	17.13	2.56	30.60	0.17	47.99	0.00	0.31	0.00	0.00	0.00	0.00	0.06		Sky	Tree	Grass	Building
GV_2767	0.94	72.02	3.14	15.50	5.00	1.18	0.44	0.02	0.00	1.75	0.00	0.00	0.00		Grass	Tree	Roads	Building
GV_2768	5.23	47.55	0.23	3.06	3.57	0.10	38.41	0.00	0.00	0.01	0.00	1.84	0.00		Grass	Soil	Background	Roads
GV_2769	0.28	95.97	0.70	2.98	0.02	0.04	0.00	0.00	0.00	0.00	0.00	0.01	0.00		Grass	Tree	Building	Background
GV_2770	0.80	52.73	0.01	5.58	0.00	40.85	0.00	0.00	0.00	0.00	0.00	0.03	0.00		Grass	Sky	Tree	Background
GV_2771	0.61	0.05	0.06	0.00	0.01	39.89	51.37	0.01	7.98	0.00	0.00	0.00	0.00		Soil	Sky	Sand	Background

GV_2772	0.56	51.21	10.22	9.60	0.00	28.38	0.00	0.00	0.00	0.03	0.00	0.00	0.00		Grass	Sky	Building	Tree
GV_2773	0.07	2.91	0.00	0.19	4.08	0.01	88.09	4.13	0.00	0.02	0.07	0.42	0.00		Soil	Bare Rock	Roads	Grass
GV_2774	0.00	100.00	0.00	0.00	0.00	0.00	0.00	0.00	0.00	0.00	0.00	0.00	0.00		Grass	Background	Building	Building
GV_2775	0.00	100.00	0.00	0.00	0.00	0.00	0.00	0.00	0.00	0.00	0.00	0.00	0.00		Grass	Background	Background	Background
GV_2776	0.00	100.00	0.00	0.00	0.00	0.00	0.00	0.00	0.00	0.00	0.00	0.00	0.00		Grass	Background	Building	Building
GV_2777	0.00	100.00	0.00	0.00	0.00	0.00	0.00	0.00	0.00	0.00	0.00	0.00	0.00		Grass	Background	Building	Building
GV_2778	11.81	39.65	2.27	11.77	0.97	2.12	30.59	0.05	0.13	0.57	0.03	0.03	0.01		Grass	Soil	Background	Tree
GV_2779	0.90	82.18	3.33	9.17	1.12	3.06	0.03	0.00	0.00	0.20	0.00	0.00	0.00		Grass	Tree	Building	Sky
GV_2780	4.63	2.86	0.30	47.25	0.17	26.04	1.51	4.45	0.33	0.44	12.01	0.00	0.00		Tree	Sky	Water	Background
GV_2781	4.23	0.32	5.24	71.14	0.00	13.32	0.25	5.19	0.00	0.00	0.01	0.04	0.25		Tree	Sky	Building	Bare Rock
GV_2782	4.13	2.04	0.59	80.44	0.06	8.20	0.18	0.66	0.00	0.00	3.61	0.10	0.00		Tree	Sky	Background	Water
GV_2783	7.27	11.11	7.86	49.96	0.07	14.12	0.22	0.49	0.00	0.05	8.82	0.03	0.00		Tree	Sky	Grass	Water
GV_2784	10.50	2.65	0.59	55.63	0.10	11.93	2.89	2.42	0.05	1.05	11.73	0.45	0.00		Tree	Sky	Water	Background
GV_2785	3.61	5.05	2.83	67.07	0.03	5.38	5.81	3.74	3.07	0.06	2.90	0.45	0.00		Tree	Soil	Sky	Grass
GV_2786	2.57	21.04	14.88	51.32	0.00	3.20	6.01	0.50	0.00	0.00	0.00	0.19	0.28		Tree	Grass	Building	Soil
GV_2787	4.04	55.77	10.10	6.77	0.22	7.70	14.85	0.18	0.00	0.21	0.00	0.01	0.15		Grass	Soil	Building	Sky
GV_2788	1.47	34.17	0.00	35.89	0.00	1.93	18.87	0.13	0.07	0.00	4.34	3.12	0.00		Tree	Grass	Soil	Water
GV_2789	0.13	26.82	0.11	11.40	37.23	20.38	0.94	0.50	0.00	0.03	0.00	2.04	0.42		Roads	Grass	Sky	Tree
GV_2790	1.33	24.39	5.59	10.54	34.18	20.61	1.17	0.48	0.00	0.01	0.00	0.52	1.19		Roads	Grass	Sky	Tree
GV_2791	1.45	31.30	0.09	32.81	10.56	0.75	5.57	0.00	0.01	0.01	0.16	17.31	0.00		Tree	Grass	Gravel	Roads
GV_2792	1.75	4.59	0.00	63.71	1.26	4.09	7.93	0.69	0.02	0.01	2.48	13.48	0.00		Tree	Gravel	Soil	Grass
GV_2793	1.21	28.72	0.00	39.67	11.23	1.94	2.53	3.95	0.14	0.04	4.94	5.63	0.00		Tree	Grass	Roads	Gravel
GV_2794	5.98	35.51	11.96	30.10	0.01	10.44	5.19	0.72	0.00	0.00	0.05	0.04	0.02		Grass	Tree	Building	Sky
GV_2795	2.99	32.39	2.37	38.53	1.23	5.88	11.60	0.13	0.00	0.44	0.01	4.43	0.00		Tree	Grass	Soil	Sky
GV_2796	5.38	9.64	13.78	45.56	0.00	11.58	13.50	0.51	0.00	0.00	0.04	0.00	0.00		Tree	Building	Soil	Sky
GV_2797	3.52	7.91	10.56	35.90	18.13	17.84	2.12	2.16	0.21	0.60	0.89	0.11	0.04		Tree	Roads	Sky	Building
GV_2798	0.15	11.43	0.00	50.16	0.02	14.82	1.36	1.06	1.41	2.37	16.79	0.43	0.00		Tree	Water	Sky	Grass
GV_2799	2.26	11.55	0.15	43.39	0.08	16.44	3.63	2.60	1.10	5.39	13.36	0.06	0.00		Tree	Sky	Water	Grass
GV_2800	2.66	22.76	21.74	25.27	0.04	1.89	13.82	4.55	7.13	0.10	0.00	0.04	0.00		Tree	Grass	Building	Soil
GV_2801	3.86	63.27	6.51	14.00	0.35	7.32	1.92	1.01	0.00	0.15	0.18	0.54	0.89		Grass	Tree	Sky	Building
GV_2802	2.03	21.98	1.14	64.85	0.21	5.48	1.06	0.47	0.00	2.09	0.55	0.14	0.00		Tree	Grass	Sky	Sidewalk
GV_2803	0.22	23.84	0.00	48.03	0.00	0.04	23.13	3.92	0.00	0.00	0.00	0.81	0.00		Tree	Grass	Soil	Bare Rock
GV_2804	3.34	26.85	0.36	27.31	18.50	16.88	2.36	0.37	0.00	1.49	0.32	2.18	0.03		Tree	Grass	Roads	Sky
GV_2805	4.24	52.22	14.49	13.25	1.41	8.56	3.43	0.04	0.00	0.97	0.00	1.38	0.00		Grass	Building	Tree	Sky
GV_2806	1.69	32.55	0.02	37.29	3.53	3.52	2.97	0.03	0.09	0.50	0.14	17.68	0.00		Tree	Grass	Gravel	Roads
GV_2807	0.52	41.65	0.00	29.46	15.52	0.39	6.63	0.11	0.01	0.04	0.02	5.64	0.00		Grass	Tree	Roads	Soil
GV_2808	0.02	34.39	0.00	43.63	11.25	1.44	0.83	0.00	0.00	0.00	0.00	8.45	0.00		Tree	Grass	Roads	Gravel
GV_2809	0.29	29.10	0.00	41.58	0.37	1.97	11.82	3.47	2.19	1.18	2.21	5.82	0.00		Tree	Grass	Soil	Gravel
GV_2810	0.87	12.98	0.00	30.14	15.32	1.59	31.73	0.53	0.00	0.41	0.00	6.38	0.05		Soil	Tree	Roads	Grass
GV_2811	2.97	21.26	0.01	43.47	8.97	2.03	3.91	0.49	0.06	0.00	0.01	16.82	0.00		Tree	Grass	Gravel	Roads
GV_2812	1.57	19.37	4.07	43.20	7.10	3.11	18.36	0.09	0.07	0.16	0.42	2.49	0.00		Tree	Grass	Soil	Roads
GV_2813	1.51	7.14	4.95	55.25	5.62	5.33	8.08	0.15	0.44	0.03	0.28	11.23	0.00		Tree	Gravel	Soil	Grass
GV_2814	9.34	32.37	15.80	28.89	0.04	11.95	1.00	0.31	0.00	0.04	0.15	0.10	0.00		Grass	Tree	Building	Sky
GV_2815	8.48	37.21	9.81	26.12	0.02	8.33	6.19	3.45	0.00	0.04	0.32	0.03	0.00		Grass	Tree	Building	Background
GV_2816	3.48	20.50	14.19	38.10	0.02	13.24	8.14	2.25	0.00	0.00	0.04	0.05	0.00		Tree	Grass	Building	Sky
GV_2817	5.07	14.45	8.72	26.94	5.95	11.55	23.35	0.86	0.00	0.64	1.54	0.92	0.03		Tree	Soil	Grass	Sky
GV_2818	3.75	17.27	14.50	22.96	8.68	13.74	18.34	0.22	0.00	0.16	0.23	0.11	0.04		Tree	Soil	Grass	Building
GV_2819	9.77	11.67	12.74	34.57	7.83	10.92	5.71	0.44	0.01	0.04	1.25	5.06	0.00		Tree	Building	Grass	Sky
GV_2820	2.31	10.74	7.24	54.64	1.28	5.63	7.45	0.35	0.00	0.04	0.05	10.27	0.00		Tree	Grass	Gravel	Soil
GV_2821	1.92	29.38	0.32	44.34	1.19	11.49	6.95	2.21	0.00	0.24	0.13	1.82	0.00		Tree	Grass	Sky	Soil
GV_2822	6.38	10.78	4.81	41.03	0.21	14.30	6.30	13.36	0.00	0.56	0.41	1.03	0.82		Tree	Sky	Bare Rock	Grass
GV_2823	3.90	8.29	0.38	39.84	4.12	8.96	7.00	4.62	1.34	0.64	2.57	17.00	1.32		Tree	Gravel	Sky	Grass
GV_2824	6.91	5.27	15.02	50.60	0.45	7.39	4.24	8.07	0.01	0.78	0.49	0.75	0.03		Tree	Building	Bare Rock	Sky
GV_2825	19.17	12.33	8.54	41.27	0.09	5.49	4.32	1.82	0.01	6.46	0.15	0.09	0.25		Tree	Background	Grass	Building
GV_2826	9.40	5.46	3.25	35.33	0.12	16.41	4.17	21.33	0.04	0.63	2.40	0.19	1.26		Tree	Bare Rock	Sky	Background
GV_2827	2.09	6.39	0.37	49.33	0.04	30.24	2.96	6.82	0.00	0.00	0.03	1.10	0.64		Tree	Sky	Bare Rock	Grass
GV_2828	3.66	7.30	0.39	71.10	0.53	10.22	1.82	4.42	0.00	0.36	0.15	0.06	0.00		Tree	Sky	Grass	Bare Rock
GV_2829	2.51	8.02	0.02	45.26	0.53	7.14	20.85	2.83	6.58	0.00	0.59	5.67	0.00		Tree	Soil	Grass	Sky
GV_2830	3.92	3.16	0.29	47.88	7.08	11.34	6.36	5.91	1.02	0.44	9.14	3.44	0.01		Tree	Sky	Water	Roads

GV_2831	2.39	1.73	0.04	71.39	0.12	2.37	6.49	4.68	0.10	0.23	10.39	0.07	0.00		Tree	Water	Soil	Bare Rock
GV_2832	3.49	19.56	12.63	46.60	0.02	7.07	7.03	0.95	0.03	0.33	2.17	0.12	0.01		Tree	Grass	Building	Sky
GV_2833	3.31	40.34	21.36	14.34	0.14	4.51	14.84	0.82	0.00	0.19	0.14	0.02	0.00		Grass	Building	Soil	Tree
GV_2834	4.28	7.43	7.66	63.13	0.00	7.38	5.07	2.90	0.00	1.22	0.55	0.38	0.00		Tree	Building	Grass	Sky
GV_2835	6.53	70.05	11.77	5.21	0.09	4.96	1.12	0.03	0.00	0.12	0.00	0.07	0.05		Grass	Building	Background	Tree
GV_2836	2.85	53.13	1.84	19.10	0.00	12.70	8.42	0.03	0.00	0.00	0.00	0.07	1.85		Grass	Tree	Sky	Soil
GV_2837	2.79	10.42	0.00	70.95	0.00	0.03	15.29	0.13	0.00	0.00	0.00	0.38	0.00		Tree	Soil	Grass	Background
GV_2838	1.40	36.50	0.00	51.06	0.00	0.79	7.47	1.05	0.01	0.00	1.29	0.42	0.00		Tree	Grass	Soil	Background
GV_2839	0.55	44.13	0.00	45.26	0.00	0.34	8.31	0.01	0.00	0.00	0.00	1.39	0.00		Tree	Grass	Soil	Gravel
GV_2840	0.97	21.75	0.40	33.99	29.02	4.45	7.48	0.40	0.00	0.71	0.13	0.36	0.34		Tree	Roads	Grass	Soil
GV_2841	2.33	8.52	0.47	30.23	44.33	7.05	2.05	0.18	0.03	0.00	0.00	4.79	0.01		Roads	Tree	Grass	Sky
GV_2842	0.81	33.28	0.03	33.87	4.20	17.58	0.03	1.59	0.00	8.16	0.09	0.19	0.18		Tree	Grass	Sky	Sidewalk
GV_2843	3.97	49.75	10.48	19.27	1.12	0.71	6.75	0.18	1.52	6.14	0.00	0.12	0.00		Grass	Tree	Building	Soil
GV_2844	4.76	27.70	2.12	30.64	11.96	1.46	7.80	2.34	0.20	5.70	1.35	3.55	0.42		Tree	Grass	Roads	Soil
GV_2845	0.73	50.13	0.99	27.61	9.63	6.35	2.57	0.01	0.61	1.04	0.23	0.11	0.00		Grass	Tree	Roads	Sky
GV_2846	0.46	89.00	0.06	7.35	0.86	1.63	0.17	0.00	0.22	0.25	0.00	0.00	0.00		Grass	Tree	Sky	Roads
GV_2847	5.29	38.51	19.59	8.39	7.43	16.97	0.23	0.01	0.00	3.46	0.00	0.12	0.00		Grass	Building	Sky	Tree
GV_2848	0.22	98.80	0.00	0.00	0.03	0.12	0.82	0.00	0.00	0.00	0.00	0.00	0.00		Grass	Soil	Background	Sky
GV_2849	0.19	99.72	0.00	0.00	0.01	0.01	0.04	0.00	0.00	0.00	0.03	0.00	0.00		Grass	Background	Soil	Water
GV_2850	0.01	99.96	0.00	0.00	0.00	0.00	0.00	0.00	0.00	0.00	0.03	0.00	0.00		Grass	Water	Background	Building
GV_2851	0.04	56.73	0.00	0.00	1.45	0.00	40.59	0.05	0.65	0.44	0.00	0.05	0.00		Grass	Soil	Roads	Sand
GV_2852	0.10	89.97	0.00	0.01	0.00	0.00	9.89	0.00	0.00	0.00	0.00	0.02	0.00		Grass	Soil	Background	Gravel
GV_2853	0.00	100.00	0.00	0.00	0.00	0.00	0.00	0.00	0.00	0.00	0.00	0.00	0.00		Grass	Background	Background	Background
GV_2854	1.25	84.32	0.00	13.75	0.00	0.01	0.60	0.00	0.00	0.00	0.00	0.08	0.00		Grass	Tree	Background	Soil
GV_2855	0.03	99.93	0.00	0.00	0.02	0.00	0.03	0.00	0.00	0.00	0.00	0.00	0.00		Grass	Soil	Background	Roads
GV_2856	0.00	100.00	0.00	0.00	0.00	0.00	0.00	0.00	0.00	0.00	0.00	0.00	0.00		Grass	Soil	Background	Background
GV_2857	0.05	37.57	0.00	0.00	0.00	0.00	62.37	0.00	0.00	0.00	0.00	0.00	0.00		Soil	Grass	Background	Building
GV_2858	0.00	99.81	0.00	0.00	0.19	0.00	0.00	0.00	0.00	0.00	0.00	0.00	0.00		Grass	Roads	Background	Background
GV_2859	0.00	99.96	0.00	0.00	0.02	0.00	0.02	0.00	0.00	0.00	0.00	0.00	0.00		Grass	Roads	Soil	Background
GV_2860	0.00	100.00	0.00	0.00	0.00	0.00	0.00	0.00	0.00	0.00	0.00	0.00	0.00		Grass	Background	Background	Background
GV_2861	0.20	52.25	0.00	0.00	0.00	0.13	46.67	0.05	0.00	0.00	0.02	0.68	0.00		Grass	Soil	Gravel	Background
GV_2862	0.81	42.89	17.96	6.56	1.40	16.85	0.73	0.15	0.00	12.66	0.00	0.00	0.00		Grass	Building	Sky	Sidewalk
GV_2863	0.74	48.71	0.88	32.30	9.05	5.63	1.89	0.04	0.34	0.05	0.36	0.00	0.01		Grass	Tree	Roads	Sky
GV_2864	2.11	40.62	0.51	15.54	17.81	10.25	0.92	0.05	0.00	0.13	9.52	2.35	0.18		Grass	Roads	Tree	Sky
GV_2865	4.60	37.92	0.01	40.27	0.03	0.12	16.85	0.11	0.00	0.00	0.00	0.08	0.00		Tree	Grass	Soil	Background
GV_2866	6.87	16.70	1.68	45.40	9.86	5.44	12.23	0.05	0.00	0.08	0.00	1.67	0.01		Tree	Grass	Soil	Roads
GV_2867	3.19	60.65	0.00	16.24	3.18	1.98	7.65	3.26	0.02	0.20	0.32	3.31	0.00		Grass	Tree	Soil	Gravel
GV_2868	7.11	73.22	0.04	9.93	0.03	0.04	6.21	2.27	0.15	0.00	0.01	1.00	0.00		Grass	Tree	Background	Soil
GV_2869	3.52	37.35	0.03	30.64	0.22	0.05	5.65	5.63	0.00	0.00	0.00	16.91	0.00		Grass	Tree	Gravel	Soil
GV_2870	3.19	8.87	0.40	44.82	20.91	4.80	5.25	0.24	0.03	0.87	0.13	10.48	0.00		Tree	Roads	Gravel	Grass
GV_2871	0.84	13.56	0.50	45.59	25.79	3.65	0.59	0.00	0.00	8.69	0.46	0.34	0.00		Tree	Roads	Grass	Sidewalk
GV_2872	1.36	38.14	0.51	27.65	10.90	2.95	14.15	0.50	0.05	2.33	0.67	0.79	0.00		Grass	Tree	Soil	Roads
GV_2873	0.32	23.15	0.19	14.21	0.31	10.69	34.67	11.94	0.00	0.00	0.00	4.51	0.01		Soil	Grass	Tree	Bare Rock
GV_2874	0.33	6.84	0.19	37.06	41.12	8.04	0.61	0.08	0.00	5.38	0.00	0.34	0.00		Roads	Tree	Sky	Grass
GV_2875	0.29	45.99	0.00	10.52	15.25	4.55	11.54	0.08	0.00	0.05	0.01	11.71	0.01		Grass	Roads	Gravel	Soil
GV_2876	1.93	67.07	0.00	8.28	3.40	0.08	12.17	1.81	0.14	0.14	0.03	4.96	0.00		Grass	Soil	Tree	Gravel
GV_2877	0.01	99.58	0.00	0.00	0.20	0.00	0.01	0.00	0.00	0.00	0.20	0.00	0.00		Grass	Roads	Water	Soil
GV_2878	0.04	91.76	0.00	0.00	0.00	0.00	7.69	0.00	0.00	0.00	0.11	0.40	0.00		Grass	Soil	Gravel	Water
GV_2879	0.15	99.85	0.00	0.00	0.00	0.00	0.00	0.00	0.00	0.00	0.00	0.00	0.00		Grass	Background	Building	Building
GV_2880	0.02	97.30	0.00	0.00	1.54	0.00	1.13	0.00	0.01	0.00	0.00	0.00	0.00		Grass	Roads	Soil	Background
GV_2881	0.80	33.10	29.61	5.96	0.60	26.94	0.00	0.02	0.00	2.95	0.00	0.01	0.00		Grass	Building	Sky	Tree
GV_2882	2.71	27.56	0.12	40.86	0.92	7.47	14.59	1.55	1.62	0.00	0.09	2.50	0.01		Tree	Grass	Soil	Sky
GV_2883	2.11	9.70	0.57	11.48	24.80	24.89	3.73	3.60	3.57	0.68	11.82	3.05	0.00		Sky	Roads	Water	Tree
GV_2884	5.61	74.43	3.32	3.01	0.18	0.22	7.19	3.65	0.32	1.84	0.20	0.02	0.00		Grass	Soil	Background	Bare Rock
GV_2885	0.17	97.33	0.00	0.01	1.82	0.00	0.28	0.00	0.00	0.00	0.00	0.38	0.00		Grass	Roads	Gravel	Soil
GV_2886	0.13	35.42	0.00	0.08	14.44	0.20	36.49	0.01	1.94	3.31	0.03	7.95	0.00		Soil	Grass	Roads	Gravel
GV_2887	1.34	64.04	0.00	0.00	11.33	0.01	4.96	0.22	1.27	11.60	0.11	5.11	0.00		Grass	Sidewalk	Roads	Gravel
GV_2888	0.32	66.34	0.00	0.00	7.53	0.07	21.85	0.05	1.96	0.01	0.23	1.63	0.00		Grass	Soil	Roads	Sand
GV_2889	0.01	54.43	0.00	0.00	37.34	0.02	1.39	0.00	0.00	0.04	0.00	6.78	0.00		Grass	Roads	Gravel	Soil

GV_2890	0.24	74.53	0.00	0.09	0.44	0.98	20.80	0.06	0.00	0.00	0.06	2.79	0.00		Grass	Soil	Gravel	Sky
GV_2891	0.00	56.99	0.00	0.00	1.23	0.00	40.83	0.05	0.00	0.00	0.00	0.90	0.00		Grass	Soil	Roads	Gravel
GV_2892	0.03	79.89	0.00	0.00	0.00	0.01	18.03	0.30	0.00	0.00	0.00	1.75	0.00		Grass	Soil	Gravel	Bare Rock
GV_2893	0.02	87.35	0.00	0.05	0.00	0.16	9.58	0.13	0.00	0.05	0.00	2.65	0.02		Grass	Soil	Gravel	Sky
GV_2894	0.25	89.06	0.00	0.01	0.02	0.08	10.56	0.00	0.00	0.00	0.00	0.00	0.00		Grass	Soil	Background	Sky
GV_2895	0.09	99.87	0.00	0.00	0.00	0.00	0.02	0.00	0.00	0.00	0.02	0.00	0.00		Grass	Background	Soil	Water
GV_2896	0.04	97.69	0.00	0.00	1.80	0.09	0.33	0.00	0.00	0.00	0.04	0.00	0.00		Grass	Roads	Soil	Sky
GV_2897	0.67	75.69	0.00	0.00	2.77	0.71	6.50	9.05	4.50	0.00	0.01	0.10	0.00		Grass	Bare Rock	Soil	Sand
GV_2898	0.22	58.57	0.00	0.00	16.28	0.00	8.04	2.26	14.51	0.00	0.00	0.12	0.00		Grass	Roads	Sand	Soil
GV_2899	0.00	98.69	0.00	0.00	0.27	0.89	0.02	0.00	0.00	0.00	0.11	0.02	0.00		Grass	Sky	Roads	Water
GV_2900	1.11	60.12	0.63	22.29	10.91	2.58	2.02	0.00	0.24	0.04	0.00	0.06	0.00		Grass	Tree	Roads	Sky
GV_2901	4.79	41.11	2.30	25.97	1.90	1.90	1.58	0.40	18.28	0.14	1.53	0.10	0.00		Grass	Tree	Sand	Background
GV_2902	1.11	3.31	0.04	44.06	0.08	0.17	48.12	1.72	0.25	0.00	0.01	1.14	0.00		Soil	Tree	Grass	Bare Rock
GV_2903	1.58	21.99	0.00	14.93	0.00	0.43	60.02	0.48	0.00	0.00	0.00	0.57	0.00		Soil	Grass	Tree	Background
GV_2904	3.06	3.15	0.00	49.31	0.00	0.27	43.87	0.27	0.00	0.00	0.04	0.03	0.00		Tree	Soil	Grass	Background
GV_2905	1.19	5.48	0.00	52.84	0.03	0.81	36.82	2.73	0.00	0.00	0.00	0.10	0.00		Tree	Soil	Grass	Bare Rock
GV_2906	2.53	17.31	0.04	67.50	1.26	6.04	2.09	0.18	0.02	0.11	0.01	2.91	0.00		Tree	Grass	Sky	Gravel
GV_2907	0.13	91.64	0.03	3.00	4.58	0.08	0.51	0.03	0.00	0.01	0.00	0.00	0.00		Grass	Roads	Tree	Soil
GV_2908	0.91	66.97	0.00	1.48	0.59	14.56	1.04	0.16	0.00	0.00	0.00	14.07	0.21		Grass	Sky	Gravel	Tree
GV_2909	2.15	27.39	0.28	39.48	0.66	1.91	18.43	0.03	0.00	0.00	0.01	9.53	0.12		Tree	Grass	Soil	Gravel
GV_2910	4.90	38.84	1.33	37.80	4.25	10.37	0.99	0.04	0.00	0.86	0.00	0.61	0.00		Grass	Tree	Sky	Background
GV_2911	7.65	44.69	1.53	11.11	0.00	34.43	0.55	0.03	0.00	0.00	0.00	0.01	0.00		Grass	Sky	Tree	Background
GV_2912	21.49	27.85	18.07	2.69	13.81	3.95	0.43	0.09	0.00	11.63	0.00	0.00	0.00		Grass	Background	Building	Roads
GV_2913	3.05	1.18	0.67	0.02	51.35	0.94	0.71	11.61	0.22	7.78	22.49	0.01	0.00		Roads	Water	Bare Rock	Sidewalk
GV_2914	2.44	47.20	7.68	20.77	0.00	17.69	3.87	0.33	0.00	0.00	0.01	0.00	0.00		Grass	Tree	Sky	Building
GV_2915	1.56	72.07	0.00	4.19	2.73	5.42	6.65	0.09	0.00	0.00	0.15	7.13	0.00		Grass	Gravel	Soil	Sky
