# Supplementary material for: Land Use Land Cover Labeling of GLOBE Images Using a Deep Learning Fusion Model
Source: Sensors (Basel). 2022 Sep 13;22(18):6895. doi: 10.3390/s22186895 (PMC9503776; doi:10.3390/s22186895)
Supplement: Supplementary file 1 [file sensors-22-06895-s001.zip › Table S1.pdf]

Supplement Table S1. 2915 GLOBE images with GPS positions and links for downloading the five directional images.

GLOBE IMAGE CODE	Latitude	Longitude	Elevation	Output Land Cover Label from Model	Google maps coordinates	North	South	East	West	Down
GV_1	45.516	-122.6822	926.9		https://www.google.com/maps/search/45.516,-122.6822	https://data.globe.gov/system/photos/2019/06/01/1087243/original.jpg	https://data.globe.gov/system/photos/2019/06/01/1087245/original.jpg	https://data.globe.gov/system/photos/2019/06/01/1087244/original.jpg	https://data.globe.gov/system/photos/2019/06/01/1087246/original.jpg	https://data.globe.gov/system/photos/2019/06/01/1087248/original.jpg
GV_2	54.3498	18.8662	141.8		https://www.google.com/maps/search/54.3498,18.8662	https://data.globe.gov/system/photos/2019/06/01/1087430/original.jpg	https://data.globe.gov/system/photos/2019/06/01/1087432/original.jpg	https://data.globe.gov/system/photos/2019/06/01/1087431/original.jpg	https://data.globe.gov/system/photos/2019/06/01/1087433/original.jpg	https://data.globe.gov/system/photos/2019/06/01/1087435/original.jpg
GV_3	41.3271	-72.9048	0		https://www.google.com/maps/search/41.3271,-72.9048	https://data.globe.gov/system/photos/2019/06/01/1087448/original.jpg	https://data.globe.gov/system/photos/2019/06/01/1087450/original.jpg	https://data.globe.gov/system/photos/2019/06/01/1087449/original.jpg	https://data.globe.gov/system/photos/2019/06/01/1087451/original.jpg	https://data.globe.gov/system/photos/2019/06/01/1087453/original.jpg
GV_4	45.5159	-122.6825	926.9		https://www.google.com/maps/search/45.5159,-122.6825	https://data.globe.gov/system/photos/2019/06/01/1087653/original.jpg	https://data.globe.gov/system/photos/2019/06/01/1087655/original.jpg	https://data.globe.gov/system/photos/2019/06/01/1087654/original.jpg	https://data.globe.gov/system/photos/2019/06/01/1087656/original.jpg	https://data.globe.gov/system/photos/2019/06/01/1087658/original.jpg
GV_5	45.5156	-122.6828	926.9		https://www.google.com/maps/search/45.5156,-122.6828	https://data.globe.gov/system/photos/2019/06/01/1087659/original.jpg	https://data.globe.gov/system/photos/2019/06/01/1087661/original.jpg	https://data.globe.gov/system/photos/2019/06/01/1087660/original.jpg	https://data.globe.gov/system/photos/2019/06/01/1087662/original.jpg	https://data.globe.gov/system/photos/2019/06/01/1087664/original.jpg
GV_6	45.516	-122.6829	926.9		https://www.google.com/maps/search/45.516,-122.6829	https://data.globe.gov/system/photos/2019/06/01/1087665/original.jpg	https://data.globe.gov/system/photos/2019/06/01/1087667/original.jpg	https://data.globe.gov/system/photos/2019/06/01/1087666/original.jpg	https://data.globe.gov/system/photos/2019/06/01/1087668/original.jpg	https://data.globe.gov/system/photos/2019/06/01/1087670/original.jpg
GV_7	54.4206	-0.523	-16		https://www.google.com/maps/search/54.4206,-0.523	https://data.globe.gov/system/photos/2019/06/01/1087955/original.jpg	https://data.globe.gov/system/photos/2019/06/01/1087957/original.jpg	https://data.globe.gov/system/photos/2019/06/01/1087956/original.jpg	https://data.globe.gov/system/photos/2019/06/01/1087958/original.jpg	https://data.globe.gov/system/photos/2019/06/01/1087960/original.jpg
GV_8	45.5012	-122.6717	926.9		https://www.google.com/maps/search/45.5012,-122.6717	https://data.globe.gov/system/photos/2019/06/02/1088426/original.jpg	https://data.globe.gov/system/photos/2019/06/02/1088428/original.jpg	https://data.globe.gov/system/photos/2019/06/02/1088427/original.jpg	https://data.globe.gov/system/photos/2019/06/02/1088429/original.jpg	https://data.globe.gov/system/photos/2019/06/02/1088431/original.jpg
GV_9	45.5019	-122.6722	926.9		https://www.google.com/maps/search/45.5019,-122.6722	https://data.globe.gov/system/photos/2019/06/02/1088432/original.jpg	https://data.globe.gov/system/photos/2019/06/02/1088434/original.jpg	https://data.globe.gov/system/photos/2019/06/02/1088433/original.jpg	https://data.globe.gov/system/photos/2019/06/02/1088435/original.jpg	https://data.globe.gov/system/photos/2019/06/02/1088437/original.jpg
GV_10	45.5037	-122.673	926.9		https://www.google.com/maps/search/45.5037,-122.673	https://data.globe.gov/system/photos/2019/06/02/1088439/original.jpg	https://data.globe.gov/system/photos/2019/06/02/1088441/original.jpg	https://data.globe.gov/system/photos/2019/06/02/1088440/original.jpg	https://data.globe.gov/system/photos/2019/06/02/1088442/original.jpg	https://data.globe.gov/system/photos/2019/06/02/1088444/original.jpg
GV_11	45.5059	-122.674	926.9		https://www.google.com/maps/search/45.5059,-122.674	https://data.globe.gov/system/photos/2019/06/02/1088445/original.jpg	https://data.globe.gov/system/photos/2019/06/02/1088447/original.jpg	https://data.globe.gov/system/photos/2019/06/02/1088446/original.jpg	https://data.globe.gov/system/photos/2019/06/02/1088448/original.jpg	https://data.globe.gov/system/photos/2019/06/02/1088450/original.jpg
GV_12	45.5079	-122.6719	926.9		https://www.google.com/maps/search/45.5079,-122.6719	https://data.globe.gov/system/photos/2019/06/03/1088457/original.jpg	https://data.globe.gov/system/photos/2019/06/03/1088459/original.jpg	https://data.globe.gov/system/photos/2019/06/03/1088458/original.jpg	https://data.globe.gov/system/photos/2019/06/03/1088460/original.jpg	https://data.globe.gov/system/photos/2019/06/03/1088462/original.jpg
GV_13	45.5088	-122.6728	926.9		https://www.google.com/maps/search/45.5088,-122.6728	https://data.globe.gov/system/photos/2019/06/03/1088463/original.jpg	https://data.globe.gov/system/photos/2019/06/03/1088465/original.jpg	https://data.globe.gov/system/photos/2019/06/03/1088464/original.jpg	https://data.globe.gov/system/photos/2019/06/03/1088466/original.jpg	https://data.globe.gov/system/photos/2019/06/03/1088468/original.jpg
GV_14	45.5098	-122.6756	926.9		https://www.google.com/maps/search/45.5098,-122.6756	https://data.globe.gov/system/photos/2019/06/03/1088469/original.jpg	https://data.globe.gov/system/photos/2019/06/03/1088471/original.jpg	https://data.globe.gov/system/photos/2019/06/03/1088470/original.jpg	https://data.globe.gov/system/photos/2019/06/03/1088472/original.jpg	https://data.globe.gov/system/photos/2019/06/03/1088474/original.jpg
GV_15	45.5108	-122.6786	926.9		https://www.google.com/maps/search/45.5108,-122.6786	https://data.globe.gov/system/photos/2019/06/03/1088475/original.jpg	https://data.globe.gov/system/photos/2019/06/03/1088477/original.jpg	https://data.globe.gov/system/photos/2019/06/03/1088476/original.jpg	https://data.globe.gov/system/photos/2019/06/03/1088478/original.jpg	https://data.globe.gov/system/photos/2019/06/03/1088480/original.jpg
GV_16	45.5112	-122.6798	926.9		https://www.google.com/maps/search/45.5112,-122.6798	https://data.globe.gov/system/photos/2019/06/03/1088481/original.jpg	https://data.globe.gov/system/photos/2019/06/03/1088483/original.jpg	https://data.globe.gov/system/photos/2019/06/03/1088482/original.jpg	https://data.globe.gov/system/photos/2019/06/03/1088484/original.jpg	https://data.globe.gov/system/photos/2019/06/03/1088486/original.jpg
GV_17	50.4932	21.6788	204.6		https://www.google.com/maps/search/50.4932,21.6788	https://data.globe.gov/system/photos/2019/06/03/1088631/original.jpg	https://data.globe.gov/system/photos/2019/06/03/1088633/original.jpg	https://data.globe.gov/system/photos/2019/06/03/1088632/original.jpg	https://data.globe.gov/system/photos/2019/06/03/1088634/original.jpg	https://data.globe.gov/system/photos/2019/06/03/1088636/original.jpg
GV_18	41.2654	-95.9243	321		https://www.google.com/maps/search/41.2654,-95.9243	https://data.globe.gov/system/photos/2019/06/03/1089028/original.jpg	https://data.globe.gov/system/photos/2019/06/03/1089030/original.jpg	https://data.globe.gov/system/photos/2019/06/03/1089029/original.jpg	https://data.globe.gov/system/photos/2019/06/03/1089031/original.jpg	https://data.globe.gov/system/photos/2019/06/03/1089033/original.jpg
GV_19	44.5556	-123.1369	141.6		https://www.google.com/maps/search/44.5556,-123.1369	https://data.globe.gov/system/photos/2019/06/03/1089193/original.jpg	https://data.globe.gov/system/photos/2019/06/03/1089195/original.jpg	https://data.globe.gov/system/photos/2019/06/03/1089194/original.jpg	https://data.globe.gov/system/photos/2019/06/03/1089196/original.jpg	https://data.globe.gov/system/photos/2019/06/03/1089198/original.jpg
GV_20	44.5517	-123.137	141.6		https://www.google.com/maps/search/44.5517,-123.137	https://data.globe.gov/system/photos/2019/06/03/1089199/original.jpg	https://data.globe.gov/system/photos/2019/06/03/1089201/original.jpg	https://data.globe.gov/system/photos/2019/06/03/1089200/original.jpg	https://data.globe.gov/system/photos/2019/06/03/1089202/original.jpg	https://data.globe.gov/system/photos/2019/06/03/1089204/original.jpg
GV_21	44.5668	-123.2958	141.6		https://www.google.com/maps/search/44.5668,-123.2958	https://data.globe.gov/system/photos/2019/06/03/1089220/original.jpg	https://data.globe.gov/system/photos/2019/06/03/1089222/original.jpg	https://data.globe.gov/system/photos/2019/06/03/1089221/original.jpg	https://data.globe.gov/system/photos/2019/06/03/1089223/original.jpg	https://data.globe.gov/system/photos/2019/06/03/1089225/original.jpg
GV_22	12.7703	79.4206	144.7		https://www.google.com/maps/search/12.7703,79.4206	https://data.globe.gov/system/photos/2019/06/04/1090918/original.jpg	https://data.globe.gov/system/photos/2019/06/04/1090920/original.jpg	https://data.globe.gov/system/photos/2019/06/04/1090919/original.jpg	https://data.globe.gov/system/photos/2019/06/04/1090921/original.jpg	https://data.globe.gov/system/photos/2019/06/04/1090923/original.jpg
GV_23	42.0856	-83.2096	166.9		https://www.google.com/maps/search/42.0856,-83.2096	https://data.globe.gov/system/photos/2019/06/04/1091614/original.jpg	https://data.globe.gov/system/photos/2019/06/04/1091616/original.jpg	https://data.globe.gov/system/photos/2019/06/04/1091615/original.jpg	https://data.globe.gov/system/photos/2019/06/04/1091617/original.jpg	https://data.globe.gov/system/photos/2019/06/04/1091619/original.jpg
GV_24	34.2545	-118.5913	296		https://www.google.com/maps/search/34.2545,-118.5913	https://data.globe.gov/system/photos/2019/06/04/1091710/original.jpg	https://data.globe.gov/system/photos/2019/06/04/1091712/original.jpg	https://data.globe.gov/system/photos/2019/06/04/1091711/original.jpg	https://data.globe.gov/system/photos/2019/06/04/1091713/original.jpg	https://data.globe.gov/system/photos/2019/06/04/1091715/original.jpg
GV_25	32.1376	-81.2386	0		https://www.google.com/maps/search/32.1376,-81.2386	https://data.globe.gov/system/photos/2019/06/04/1091919/original.jpg	https://data.globe.gov/system/photos/2019/06/04/1091921/original.jpg	https://data.globe.gov/system/photos/2019/06/04/1091920/original.jpg	https://data.globe.gov/system/photos/2019/06/04/1091922/original.jpg	https://data.globe.gov/system/photos/2019/06/04/1091924/original.jpg
GV_26	41.4134	-95.8588	321		https://www.google.com/maps/search/41.4134,-95.8588	https://data.globe.gov/system/photos/2019/06/04/1091991/original.jpg	https://data.globe.gov/system/photos/2019/06/04/1091993/original.jpg	https://data.globe.gov/system/photos/2019/06/04/1091992/original.jpg	https://data.globe.gov/system/photos/2019/06/04/1091994/original.jpg	https://data.globe.gov/system/photos/2019/06/04/1091997/original.jpg
GV_27	46.3687	-117.0578	1122.6		https://www.google.com/maps/search/46.3687,-117.0578	https://data.globe.gov/system/photos/2019/06/04/1092100/original.jpg	https://data.globe.gov/system/photos/2019/06/04/1092102/original.jpg	https://data.globe.gov/system/photos/2019/06/04/1092101/original.jpg	https://data.globe.gov/system/photos/2019/06/04/1092103/original.jpg	https://data.globe.gov/system/photos/2019/06/04/1092105/original.jpg
GV_28	-27.4685	153.1037	11.3		https://www.google.com/maps/search/-27.4685,153.1037	https://data.globe.gov/system/photos/2019/06/05/1092559/original.jpg	https://data.globe.gov/system/photos/2019/06/05/1092561/original.jpg	https://data.globe.gov/system/photos/2019/06/05/1092560/original.jpg	https://data.globe.gov/system/photos/2019/06/05/1092562/original.jpg	https://data.globe.gov/system/photos/2019/06/05/1092564/original.jpg
GV_29	44.5669	-123.2942	141.6		https://www.google.com/maps/search/44.5669,-123.2942	https://data.globe.gov/system/photos/2019/06/03/1092988/original.jpg	https://data.globe.gov/system/photos/2019/06/03/1092990/original.jpg	https://data.globe.gov/system/photos/2019/06/03/1092989/original.jpg	https://data.globe.gov/system/photos/2019/06/03/1092991/original.jpg	https://data.globe.gov/system/photos/2019/06/03/1092993/original.jpg
GV_30	44.5668	-123.2929	141.6		https://www.google.com/maps/search/44.5668,-123.2929	https://data.globe.gov/system/photos/2019/06/03/1093021/original.jpg	https://data.globe.gov/system/photos/2019/06/03/1093024/original.jpg	https://data.globe.gov/system/photos/2019/06/03/1093023/original.jpg	https://data.globe.gov/system/photos/2019/06/03/1093025/original.jpg	https://data.globe.gov/system/photos/2019/06/03/1093027/original.jpg
GV_31	44.5668	-123.2966	141.6		https://www.google.com/maps/search/44.5668,-123.2966	https://data.globe.gov/system/photos/2019/06/03/1093030/original.jpg	https://data.globe.gov/system/photos/2019/06/03/1093032/original.jpg	https://data.globe.gov/system/photos/2019/06/03/1093031/original.jpg	https://data.globe.gov/system/photos/2019/06/03/1093033/original.jpg	https://data.globe.gov/system/photos/2019/06/03/1093035/original.jpg
GV_32	44.5675	-123.2908	141.6		https://www.google.com/maps/search/44.5675,-123.2908	https://data.globe.gov/system/photos/2019/06/03/1093038/original.jpg	https://data.globe.gov/system/photos/2019/06/03/1093040/original.jpg	https://data.globe.gov/system/photos/2019/06/03/1093039/original.jpg	https://data.globe.gov/system/photos/2019/06/03/1093041/original.jpg	https://data.globe.gov/system/photos/2019/06/03/1093043/original.jpg
GV_33	44.5667	-123.2951	141.6		https://www.google.com/maps/search/44.5667,-123.2951	https://data.globe.gov/system/photos/2019/06/03/1093072/original.jpg	https://data.globe.gov/system/photos/2019/06/03/1093077/original.jpg	https://data.globe.gov/system/photos/2019/06/03/1093073/original.jpg	https://data.globe.gov/system/photos/2019/06/03/1093080/original.jpg	https://data.globe.gov/system/photos/2019/06/03/1093083/original.jpg
GV_34	42.0857	-83.2095	166.9		https://www.google.com/maps/search/42.0857,-83.2095	https://data.globe.gov/system/photos/2019/06/05/1093074/original.jpg	https://data.globe.gov/system/photos/2019/06/05/1093078/original.jpg	https://data.globe.gov/system/photos/2019/06/05/1093076/original.jpg	https://data.globe.gov/system/photos/2019/06/05/1093079/original.jpg	https://data.globe.gov/system/photos/2019/06/05/1093084/original.jpg
GV_35	50.5174	21.6704	204.6		https://www.google.com/maps/search/50.5174,21.6704	https://data.globe.gov/system/photos/2019/06/01/1093190/original.jpg	https://data.globe.gov/system/photos/2019/06/01/1093193/original.jpg	https://data.globe.gov/system/photos/2019/06/01/1093191/original.jpg	https://data.globe.gov/system/photos/2019/06/01/1093194/original.jpg	https://data.globe.gov/system/photos/2019/06/01/1093197/original.jpg
GV_36	50.4844	21.6871	204.6		https://www.google.com/maps/search/50.4844,21.6871	https://data.globe.gov/system/photos/2019/06/05/1093199/original.jpg	https://data.globe.gov/system/photos/2019/06/05/1093202/original.jpg	https://data.globe.gov/system/photos/2019/06/05/1093201/original.jpg	https://data.globe.gov/system/photos/2019/06/05/1093204/original.jpg	https://data.globe.gov/system/photos/2019/06/05/1093206/original.jpg
GV_37	50.5155	21.6542	204.6		https://www.google.com/maps/search/50.5155,21.6542	https://data.globe.gov/system/photos/2019/06/05/1093217/original.jpg	https://data.globe.gov/system/photos/2019/06/05/1093225/original.jpg	https://data.globe.gov/system/photos/2019/06/05/1093224/original.jpg	https://data.globe.gov/system/photos/2019/06/05/1093248/original.jpg	https://data.globe.gov/system/photos/2019/06/05/1093258/original.jpg
GV_38	41.1813	-95.9158	321		https://www.google.com/maps/search/41.1813,-95.9158	https://data.globe.gov/system/photos/2019/06/05/1093286/original.jpg	https://data.globe.gov/system/photos/2019/06/05/1093288/original.jpg	https://data.globe.gov/system/photos/2019/06/05/1093287/original.jpg	https://data.globe.gov/system/photos/2019/06/05/1093289/original.jpg	https://data.globe.gov/system/photos/2019/06/05/1093291/original.jpg
GV_39	47.3539	-101.3783	564.5		https://www.google.com/maps/search/47.3539,-101.3783	https://data.globe.gov/system/photos/2019/06/05/1093561/original.jpg	https://data.globe.gov/system/photos/2019/06/05/1093563/original.jpg	https://data.globe.gov/system/photos/2019/06/05/1093562/original.jpg	https://data.globe.gov/system/photos/2019/06/05/1093564/original.jpg	https://data.globe.gov/system/photos/2019/06/05/1093566/original.jpg
GV_40	47.3523	-101.3773	564.5		https://www.google.com/maps/search/47.3523,-101.3773	https://data.globe.gov/system/photos/2019/06/05/1093567/original.jpg	https://data.globe.gov/system/photos/2019/06/05/1093569/original.jpg	https://data.globe.gov/system/photos/2019/06/05/1093568/original.jpg	https://data.globe.gov/system/photos/2019/06/05/1093570/original.jpg	https://data.globe.gov/system/photos/2019/06/05/1093572/original.jpg
GV_41	-27.469	153.1059	3.7		https://www.google.com/maps/search/-27.469,153.1059	https://data.globe.gov/system/photos/2019/06/06/1093664/original.jpg	https://data.globe.gov/system/photos/2019/06/06/1093666/original.jpg	https://data.globe.gov/system/photos/2019/06/06/1093665/original.jpg	https://data.globe.gov/system/photos/2019/06/06/1093667/original.jpg	https://data.globe.gov/system/photos/2019/06/06/1093669/original.jpg
GV_42	24.434	118.3147	22.6		https://www.google.com/maps/search/24.434,118.3147	https://data.globe.gov/system/photos/2019/06/06/1093701/original.jpg	https://data.globe.gov/system/photos/2019/06/06/1093703/original.jpg	https://data.globe.gov/system/photos/2019/06/06/1093702/original.jpg	https://data.globe.gov/system/photos/2019/06/06/1093704/original.jpg	https://data.globe.gov/system/photos/2019/06/06/1093706/original.jpg
GV_43	24.4318	118.3107	8		https://www.google.com/maps/search/24.4318,118.3107	https://data.globe.gov/system/photos/2019/06/06/1093730/original.jpg	https://data.globe.gov/system/photos/2019/06/06/1093732/original.jpg	https://data.globe.gov/system/photos/2019/06/06/1093731/original.jpg	https://data.globe.gov/system/photos/2019/06/06/1093733/original.jpg	https://data.globe.gov/system/photos/2019/06/06/1093735/original.jpg
GV_44	45.4966	-122.6681	10.7		https://www.google.com/maps/search/45.4966,-122.6681	https://data.globe.gov/system/photos/2019/06/05/1093753/original.jpg	https://data.globe.gov/system/photos/2019/06/05/1093755/original.jpg	https://data.globe.gov/system/photos/2019/06/05/1093754/original.jpg	https://data.globe.gov/system/photos/2019/06/05/1093756/original.jpg	https://data.globe.gov/system/photos/2019/06/05/1093758/original.jpg
GV_45	15.3797	105.0233	127.2		https://www.google.com/maps/search/15.3797,105.0233	https://data.globe.gov/system/photos/2019/06/06/1093801/original.jpg	https://data.globe.gov/system/photos/2019/06/06/1093810/original.jpg	https://data.globe.gov/system/photos/2019/06/06/1093809/original.jpg	https://data.globe.gov/system/photos/2019/06/06/1093811/original.jpg	https://data.globe.gov/system/photos/2019/06/06/1093813/original.jpg
GV_46	15.3777	105.0234	126		https://www.google.com/maps/search/15.3777,105.0234	https://data.globe.gov/system/photos/2019/06/06/1093798/original.jpg	https://data.globe.gov/system/photos/2019/06/06/1093803/original.jpg	https://data.globe.gov/system/photos/2019/06/06/1093800/original.jpg	https://data.globe.gov/system/photos/2019/06/06/1093804/original.jpg	https://data.globe.gov/system/photos/2019/06/06/1093808/original.jpg
GV_47	53.4125	-0.647	22.7		https://www.google.com/maps/search/53.4125,-0.647	https://data.globe.gov/system/photos/2019/06/06/1094021/original.jpg	https://data.globe.gov/system/photos/2019/06/06/1094023/original.jpg	https://data.globe.gov/system/photos/2019/06/06/1094022/original.jpg	https://data.globe.gov/system/photos/2019/06/06/1094024/original.jpg	https://data.globe.gov/system/photos/2019/06/06/1094026/original.jpg
GV_48	45.6751	-111.215	1442.5		https://www.google.com/maps/search/45.6751,-111.215	https://data.globe.gov/system/photos/2019/06/06/1094087/original.jpg	https://data.globe.gov/system/photos/2019/06/06/1094089/original.jpg	https://data.globe.gov/system/photos/2019/06/06/1094088/original.jpg	https://data.globe.gov/system/photos/2019/06/06/1094090/original.jpg	https://data.globe.gov/system/photos/2019/06/06/1094092/original.jpg
GV_49	45.6702	-111.0424	1487		https://www.google.com/maps/search/45.6702,-111.0424	https://data.globe.gov/system/photos/2019/06/06/1094093/original.jpg	https://data.globe.gov/system/photos/2019/06/06/1094095/original.jpg	https://data.globe.gov/system/photos/2019/06/06/1094094/original.jpg	https://data.globe.gov/system/photos/2019/06/06/1094096/original.jpg	https://data.globe.gov/system/photos/2019/06/06/1094098/original.jpg
GV_50	45.6696	-111.0481	1489		https://www.google.com/maps/search/45.6696,-111.0481	https://data.globe.gov/system/photos/2019/06/06/1094099/original.jpg	https://data.globe.gov/system/photos/2019/06/06/1094101/original.jpg	https://data.globe.gov/system/photos/2019/06/06/1094100/original.jpg	https://data.globe.gov/system/photos/2019/06/06/1094102/original.jpg	https://data.globe.gov/system/photos/2019/06/06/1094104/original.jpg
GV_51	42.0859	-83.2099	177.7		https://www.google.com/maps/search/42.0859,-83.2099	https://data.globe.gov/system/photos/2019/06/06/1094170/original.jpg	https://data.globe.gov/system/photos/2019/06/06/1094172/original.jpg	https://data.globe.gov/system/photos/2019/06/06/1094171/original.jpg	https://data.globe.gov/system/photos/2019/06/06/1094173/original.jpg	https://data.globe.gov/system/photos/2019/06/06/1094175/original.jpg
GV_52	50.5043	21.6459	152.8		https://www.google.com/maps/search/50.5043,21.6459	https://data.globe.gov/system/photos/2019/06/06/1094307/original.jpg	https://data.globe.gov/system/photos/2019/06/06/1094309/original.jpg	https://data.globe.gov/system/photos/2019/06/06/1094308/original.jpg	https://data.globe.gov/system/photos/2019/06/06/1094310/original.jpg	https://data.globe.gov/system/photos/2019/06/06/1094312/original.jpg
GV_53	45.6628	-111.044	1500.5		https://www.google.com/maps/search/45.6628,-111.044	https://data.globe.gov/system/photos/2019/06/06/1094557/original.jpg	https://data.globe.gov/system/photos/2019/06/06/1094559/original.jpg	https://data.globe.gov/system/photos/2019/06/06/1094558/original.jpg	https://data.globe.gov/system/photos/2019/06/06/1094560/original.jpg	https://data.globe.gov/system/photos/2019/06/06/1094562/original.jpg
GV_54	24.4366	118.367	20.6		https://www.google.com/maps/search/24.4366,118.367	https://data.globe.gov/system/photos/2019/06/06/1094658/original.jpg	https://data.globe.gov/system/photos/2019/06/06/1094660/original.jpg	https://data.globe.gov/system/photos/2019/06/06/1094659/original.jpg	https://data.globe.gov/system/photos/2019/06/06/1094661/original.jpg	https://data.globe.gov/system/photos/2019/06/06/1094663/original.jpg
GV_55	13.7842	100.5341	9.2		https://www.google.com/maps/search/13.7842,100.5341	https://data.globe.gov/system/photos/2019/06/07/1094691/original.jpg	https://data.globe.gov/system/photos/2019/06/07/1094693/original.jpg	https://data.globe.gov/system/photos/2019/06/07/1094692/original.jpg	https://data.globe.gov/system/photos/2019/06/07/1094694/original.jpg	https://data.globe.gov/system/photos/2019/06/07/1094696/original.jpg
GV_56	-34.9341	138.6024	43.5		https://www.google.com/maps/search/-34.9341,138.6024	https://data.globe.gov/system/photos/2019/06/07/1094828/original.jpg	https://data.globe.gov/system/photos/2019/06/07/1094830/original.jpg	https://data.globe.gov/system/photos/2019/06/07/1094829/original.jpg	https://data.globe.gov/system/photos/2019/06/07/1094831/original.jpg	https://data.globe.gov/system/photos/2019/06/07/1094833/original.jpg
GV_57	50.7361	12.4031	332.5		https://www.google.com/maps/search/50.7361,12.4031	https://data.globe.gov/system/photos/2019/06/07/1094951/original.jpg	https://data.globe.gov/system/photos/2019/06/07/1094953/original.jpg	https://data.globe.gov/system/photos/2019/06/07/1094952/original.jpg	https://data.globe.gov/system/photos/2019/06/07/1094954/original.jpg	https://data.globe.gov/system/photos/2019/06/07/1094956/original.jpg
GV_58	41.6729	-88.5252	193.3		https://www.google.com/maps/search/41.6729,-88.5252	https://data.globe.gov/system/photos/2019/06/07/1095271/original.jpg	https://data.globe.gov/system/photos/2019/06/07/1095277/original.jpg	https://data.globe.gov/system/photos/2019/06/07/1095275/original.jpg	https://data.globe.gov/system/photos/2019/06/07/1095279/original.jpg	https://data.globe.gov/system/photos/2019/06/07/1095281/original.jpg
GV_59	46.313	-111.546	1211.1		https://www.google.com/maps/search/46.313,-111.546	https://data.globe.gov/system/photos/2019/06/07/1095288/original.jpg	https://data.globe.gov/system/photos/2019/06/07/1095290/original.jpg	https://data.globe.gov/system/photos/2019/06/07/1095289/original.jpg	https://data.globe.gov/system/photos/2019/06/07/1095291/original.jpg	https://data.globe.gov/system/photos/2019/06/07/1095293/original.jpg
GV_60	46.316	-111.5423	1196.7		https://www.google.com/maps/search/46.316,-111.5423	https://data.globe.gov/system/photos/2019/06/07/1095295/original.jpg	https://data.globe.gov/system/photos/2019/06/07/1095297/original.jpg	https://data.globe.gov/system/photos/2019/06/07/1095296/original.jpg	https://data.globe.gov/system/photos/2019/06/07/1095298/original.jpg	https://data.globe.gov/system/photos/2019/06/07/1095300/original.jpg
GV_61	46.3127	-111.5444	1200.8		https://www.google.com/maps/search/46.3127,-111.5444	https://data.globe.gov/system/photos/2019/06/07/1095307/original.jpg	https://data.globe.gov/system/photos/2019/06/07/1095309/original.jpg	https://data.globe.gov/system/photos/2019/06/07/1095308/original.jpg	https://data.globe.gov/system/photos/2019/06/07/1095310/original.jpg	https://data.globe.gov/system/photos/2019/06/07/1095312/original.jpg
GV_62	38.9936	-76.8404	63.3		https://www.google.com/maps/search/38.9936,-76.8404	https://data.globe.gov/system/photos/2019/06/07/1095314/original.jpg	https://data.globe.gov/system/photos/2019/06/07/1095316/original.jpg	https://data.globe.gov/system/photos/2019/06/07/1095315/original.jpg	https://data.globe.gov/system/photos/2019/06/07/1095317/original.jpg	https://data.globe.gov/system/photos/2019/06/07/1095319/original.jpg
GV_63	20.006	-105.4546	22.4		https://www.google.com/maps/search/20.006,-105.4546	https://data.globe.gov/system/photos/2019/06/07/1095320/original.jpg	https://data.globe.gov/system/photos/2019/06/07/1095322/original.jpg	https://data.globe.gov/system/photos/2019/06/07/1095321/original.jpg	https://data.globe.gov/system/photos/2019/06/07/1095323/original.jpg	https://data.globe.gov/system/photos/2019/06/07/1095325/original.jpg
GV_64	46.3005	-111.5413	1210.7		https://www.google.com/maps/search/46.3005,-111.5413	https://data.globe.gov/system/photos/2019/06/07/1095326/original.jpg	https://data.globe.gov/system/photos/2019/06/07/1095328/original.jpg	https://data.globe.gov/system/photos/2019/06/07/1095327/original.jpg	https://data.globe.gov/system/photos/2019/06/07/1095329/original.jpg	https://data.globe.gov/system/photos/2019/06/07/1095331/original.jpg
GV_65	46.2958	-111.5362	1206.2		https://www.google.com/maps/search/46.2958,-111.5362	https://data.globe.gov/system/photos/2019/06/07/1095361/original.jpg	https://data.globe.gov/system/photos/2019/06/07/1095363/original.jpg	https://data.globe.gov/system/photos/2019/06/07/1095362/original.jpg	https://data.globe.gov/system/photos/2019/06/07/1095364/original.jpg	https://data.globe.gov/system/photos/2019/06/07/1095366/original.jpg
GV_66	46.2946	-111.5326	1170.5		https://www.google.com/maps/search/46.2946,-111.5326	https://data.globe.gov/system/photos/2019/06/07/1095367/original.jpg	https://data.globe.gov/system/photos/2019/06/07/1095369/original.jpg	https://data.globe.gov/system/photos/2019/06/07/1095368/original.jpg	https://data.globe.gov/system/photos/2019/06/07/1095370/original.jpg	https://data.globe.gov/system/photos/2019/06/07/1095372/original.jpg
GV_67	46.2916	-111.5293	1172.6		https://www.google.com/maps/search/46.2916,-111.5293	https://data.globe.gov/system/photos/2019/06/07/1095378/original.jpg	https://data.globe.gov/system/photos/2019/06/07/1095380/original.jpg	https://data.globe.gov/system/photos/2019/06/07/1095379/original.jpg	https://data.globe.gov/system/photos/2019/06/07/1095381/original.jpg	https://data.globe.gov/system/photos/2019/06/07/1095383/original.jpg
GV_68	46.2865	-111.5312	1219.1		https://www.google.com/maps/search/46.2865,-111.5312	https://data.globe.gov/system/photos/2019/06/07/1095413/original.jpg	https://data.globe.gov/system/photos/2019/06/07/1095415/original.jpg	https://data.globe.gov/system/photos/2019/06/07/1095414/original.jpg	https://data.globe.gov/system/photos/2019/06/07/1095417/original.jpg	https://data.globe.gov/system/photos/2019/06/07/1095420/original.jpg
GV_69	46.2826	-111.524	1222.1		https://www.google.com/maps/search/46.2826,-111.524	https://data.globe.gov/system/photos/2019/06/07/1095449/original.jpg	https://data.globe.gov/system/photos/2019/06/07/1095451/original.jpg	https://data.globe.gov/system/photos/2019/06/07/1095450/original.jpg	https://data.globe.gov/system/photos/2019/06/07/1095452/original.jpg	https://data.globe.gov/system/photos/2019/06/07/1095454/original.jpg
GV_70	46.2801	-111.5212	1210.1		https://www.google.com/maps/search/46.2801,-111.5212	https://data.globe.gov/system/photos/2019/06/07/1095463/original.jpg	https://data.globe.gov/system/photos/2019/06/07/1095465/original.jpg	https://data.globe.gov/system/photos/2019/06/07/1095464/original.jpg	https://data.globe.gov/system/photos/2019/06/07/1095466/original.jpg	https://data.globe.gov/system/photos/2019/06/07/1095469/original.jpg
GV_71	44.6248	-123.0818	68.5		https://www.google.com/maps/search/44.6248,-123.0818	https://data.globe.gov/system/photos/2019/06/07/1095510/original.jpg	https://data.globe.gov/system/photos/2019/06/07/1095512/original.jpg	https://data.globe.gov/system/photos/2019/06/07/1095511/original.jpg	https://data.globe.gov/system/photos/2019/06/07/1095513/original.jpg	https://data.globe.gov/system/photos/2019/06/07/1095515/original.jpg
GV_72	-36.8946	149.915	5.3		https://www.google.com/maps/search/-36.8946,149.915	https://data.globe.gov/system/photos/2019/06/08/1095626/original.jpg	https://data.globe.gov/system/photos/2019/06/08/1095628/original.jpg	https://data.globe.gov/system/photos/2019/06/08/1095627/original.jpg	https://data.globe.gov/system/photos/2019/06/08/1095629/original.jpg	https://data.globe.gov/system/photos/2019/06/08/1095631/original.jpg
GV_73	25.0591	121.5248	14.2		https://www.google.com/maps/search/25.0591,121.5248	https://data.globe.gov/system/photos/2019/06/08/1095669/original.jpg	https://data.globe.gov/system/photos/2019/06/08/1095671/original.jpg	https://data.globe.gov/system/photos/2019/06/08/1095670/original.jpg	https://data.globe.gov/system/photos/2019/06/08/1095672/original.jpg	https://data.globe.gov/system/photos/2019/06/08/1095674/original.jpg
GV_74	25.0604	121.5295	9.3		https://www.google.com/maps/search/25.0604,121.5295	https://data.globe.gov/system/photos/2019/06/08/1095680/original.jpg	https://data.globe.gov/system/photos/2019/06/08/1095682/original.jpg	https://data.globe.gov/system/photos/2019/06/08/1095681/original.jpg	https://data.globe.gov/system/photos/2019/06/08/1095683/original.jpg	https://data.globe.gov/system/photos/2019/06/08/1095685/original.jpg
GV_75	25.0203	121.3068	81.6		https://www.google.com/maps/search/25.0203,121.3068	https://data.globe.gov/system/photos/2019/06/08/1095700/original.jpg	https://data.globe.gov/system/photos/2019/06/08/1095702/original.jpg	https://data.globe.gov/system/photos/2019/06/08/1095701/original.jpg	https://data.globe.gov/system/photos/2019/06/08/1095703/original.jpg	https://data.globe.gov/system/photos/2019/06/08/1095705/original.jpg
GV_76	54.408	18.6495	1.6		https://www.google.com/maps/search/54.408,18.6495	https://data.globe.gov/system/photos/2019/06/08/1095770/original.jpg	https://data.globe.gov/system/photos/2019/06/08/1095772/original.jpg	https://data.globe.gov/system/photos/2019/06/08/1095771/original.jpg	https://data.globe.gov/system/photos/2019/06/08/1095773/original.jpg	https://data.globe.gov/system/photos/2019/06/08/1095775/original.jpg
GV_77	44.5668	-123.2902	75.6		https://www.google.com/maps/search/44.5668,-123.2902	https://data.globe.gov/system/photos/2019/06/03/1095943/original.jpg	https://data.globe.gov/system/photos/2019/06/03/1095945/original.jpg	https://data.globe.gov/system/photos/2019/06/03/1095944/original.jpg	https://data.globe.gov/system/photos/2019/06/03/1095946/original.jpg	https://data.globe.gov/system/photos/2019/06/03/1095948/original.jpg
GV_78	44.5669	-123.291	77.1		https://www.google.com/maps/search/44.5669,-123.291	https://data.globe.gov/system/photos/2019/06/03/1095949/original.jpg	https://data.globe.gov/system/photos/2019/06/03/1095951/original.jpg	https://data.globe.gov/system/photos/2019/06/03/1095950/original.jpg	https://data.globe.gov/system/photos/2019/06/03/1095952/original.jpg	https://data.globe.gov/system/photos/2019/06/03/1095954/original.jpg
GV_79	45.4974	-122.6883	164.7		https://www.google.com/maps/search/45.4974,-122.6883	https://data.globe.gov/system/photos/2019/06/06/1095955/original.jpg	https://data.globe.gov/system/photos/2019/06/06/1095957/original.jpg	https://data.globe.gov/system/photos/2019/06/06/1095956/original.jpg	https://data.globe.gov/system/photos/2019/06/06/1095958/original.jpg	https://data.globe.gov/system/photos/2019/06/06/1095960/original.jpg
GV_80	45.4965	-122.6681	10.2		https://www.google.com/maps/search/45.4965,-122.6681	https://data.globe.gov/system/photos/2019/06/07/1095961/original.jpg	https://data.globe.gov/system/photos/2019/06/07/1095963/original.jpg	https://data.globe.gov/system/photos/2019/06/07/1095962/original.jpg	https://data.globe.gov/system/photos/2019/06/07/1095964/original.jpg	https://data.globe.gov/system/photos/2019/06/07/1095966/original.jpg
GV_81	45.6756	-111.0311	1471.2		https://www.google.com/maps/search/45.6756,-111.0311	https://data.globe.gov/system/photos/2019/06/08/1095998/original.jpg	https://data.globe.gov/system/photos/2019/06/08/1096000/original.jpg	https://data.globe.gov/system/photos/2019/06/08/1095999/original.jpg	https://data.globe.gov/system/photos/2019/06/08/1096001/original.jpg	https://data.globe.gov/system/photos/2019/06/08/1096003/original.jpg
GV_82	40.6666	-95.8311	336		https://www.google.com/maps/search/40.6666,-95.8311	https://data.globe.gov/system/photos/2019/06/08/1096074/original.jpg	https://data.globe.gov/system/photos/2019/06/08/1096076/original.jpg	https://data.globe.gov/system/photos/2019/06/08/1096075/original.jpg	https://data.globe.gov/system/photos/2019/06/08/1096077/original.jpg	https://data.globe.gov/system/photos/2019/06/08/1096079/original.jpg
GV_83	45.6721	-111.0248	1499.9		https://www.google.com/maps/search/45.6721,-111.0248	https://data.globe.gov/system/photos/2019/06/08/1096169/original.jpg	https://data.globe.gov/system/photos/2019/06/08/1096171/original.jpg	https://data.globe.gov/system/photos/2019/06/08/1096170/original.jpg	https://data.globe.gov/system/photos/2019/06/08/1096172/original.jpg	https://data.globe.gov/system/photos/2019/06/08/1096174/original.jpg

GV_84	-36.8935	149.915	1.2		https://www.google.com/maps/search/-36.8935,149.915	https://data.globe.gov/system/photos/2019/06/08/1096153/original.jpg	https://data.globe.gov/system/photos/2019/06/08/1096155/original.jpg	https://data.globe.gov/system/photos/2019/06/08/1096154/original.jpg	https://data.globe.gov/system/photos/2019/06/08/1096156/original.jpg	https://data.globe.gov/system/photos/2019/06/08/1096158/original.jpg
GV_85	64.9296	-147.6296	223.4		https://www.google.com/maps/search/64.9296,-147.6296	https://data.globe.gov/system/photos/2019/06/08/1096188/original.jpg	https://data.globe.gov/system/photos/2019/06/08/1096190/original.jpg	https://data.globe.gov/system/photos/2019/06/08/1096189/original.jpg	https://data.globe.gov/system/photos/2019/06/08/1096191/original.jpg	https://data.globe.gov/system/photos/2019/06/08/1096193/original.jpg
GV_86	25.0215	121.3066	76.1		https://www.google.com/maps/search/25.0215,121.3066	https://data.globe.gov/system/photos/2019/06/08/1096200/original.jpg	https://data.globe.gov/system/photos/2019/06/08/1096202/original.jpg	https://data.globe.gov/system/photos/2019/06/08/1096201/original.jpg	https://data.globe.gov/system/photos/2019/06/08/1096203/original.jpg	https://data.globe.gov/system/photos/2019/06/08/1096205/original.jpg
GV_87	24.6007	120.7523	14.4		https://www.google.com/maps/search/24.6007,120.7523	https://data.globe.gov/system/photos/2019/06/09/1096254/original.jpg	https://data.globe.gov/system/photos/2019/06/09/1096256/original.jpg	https://data.globe.gov/system/photos/2019/06/09/1096255/original.jpg	https://data.globe.gov/system/photos/2019/06/09/1096257/original.jpg	https://data.globe.gov/system/photos/2019/06/09/1096259/original.jpg
GV_88	-27.4684	153.1037	7.1		https://www.google.com/maps/search/-27.4684,153.1037	https://data.globe.gov/system/photos/2019/06/09/1096266/original.jpg	https://data.globe.gov/system/photos/2019/06/09/1096268/original.jpg	https://data.globe.gov/system/photos/2019/06/09/1096267/original.jpg	https://data.globe.gov/system/photos/2019/06/09/1096269/original.jpg	https://data.globe.gov/system/photos/2019/06/09/1096271/original.jpg
GV_89	24.6019	120.753	18.3		https://www.google.com/maps/search/24.6019,120.753	https://data.globe.gov/system/photos/2019/06/09/1096273/original.jpg	https://data.globe.gov/system/photos/2019/06/09/1096275/original.jpg	https://data.globe.gov/system/photos/2019/06/09/1096274/original.jpg	https://data.globe.gov/system/photos/2019/06/09/1096276/original.jpg	https://data.globe.gov/system/photos/2019/06/09/1096278/original.jpg
GV_90	65.0459	-147.4075	738.9		https://www.google.com/maps/search/65.0459,-147.4075	https://data.globe.gov/system/photos/2019/06/09/1096356/original.jpg	https://data.globe.gov/system/photos/2019/06/09/1096358/original.jpg	https://data.globe.gov/system/photos/2019/06/09/1096357/original.jpg	https://data.globe.gov/system/photos/2019/06/09/1096359/original.jpg	https://data.globe.gov/system/photos/2019/06/09/1096361/original.jpg
GV_91	54.3571	18.8282	0.7		https://www.google.com/maps/search/54.3571,18.8282	https://data.globe.gov/system/photos/2019/06/09/1096470/original.jpg	https://data.globe.gov/system/photos/2019/06/09/1096472/original.jpg	https://data.globe.gov/system/photos/2019/06/09/1096471/original.jpg	https://data.globe.gov/system/photos/2019/06/09/1096473/original.jpg	https://data.globe.gov/system/photos/2019/06/09/1096475/original.jpg
GV_92	-36.8946	149.9205	0		https://www.google.com/maps/search/-36.8946,149.9205	https://data.globe.gov/system/photos/2019/06/08/1096781/original.jpg	https://data.globe.gov/system/photos/2019/06/08/1096783/original.jpg	https://data.globe.gov/system/photos/2019/06/08/1096782/original.jpg	https://data.globe.gov/system/photos/2019/06/08/1096784/original.jpg	https://data.globe.gov/system/photos/2019/06/08/1096786/original.jpg
GV_93	-36.8972	149.917	1.8		https://www.google.com/maps/search/-36.8972,149.917	https://data.globe.gov/system/photos/2019/06/08/1096787/original.jpg	https://data.globe.gov/system/photos/2019/06/08/1096789/original.jpg	https://data.globe.gov/system/photos/2019/06/08/1096788/original.jpg	https://data.globe.gov/system/photos/2019/06/08/1096790/original.jpg	https://data.globe.gov/system/photos/2019/06/08/1096792/original.jpg
GV_94	13.7844	100.5342	8.1		https://www.google.com/maps/search/13.7844,100.5342	https://data.globe.gov/system/photos/2019/06/07/1096811/original.jpg	https://data.globe.gov/system/photos/2019/06/07/1096813/original.jpg	https://data.globe.gov/system/photos/2019/06/07/1096812/original.jpg	https://data.globe.gov/system/photos/2019/06/07/1096814/original.jpg	https://data.globe.gov/system/photos/2019/06/07/1096819/original.jpg
GV_95	25.0217	121.3057	75.5		https://www.google.com/maps/search/25.0217,121.3057	https://data.globe.gov/system/photos/2019/06/10/1096845/original.jpg	https://data.globe.gov/system/photos/2019/06/10/1096847/original.jpg	https://data.globe.gov/system/photos/2019/06/10/1096846/original.jpg	https://data.globe.gov/system/photos/2019/06/10/1096848/original.jpg	https://data.globe.gov/system/photos/2019/06/10/1096850/original.jpg
GV_96	13.7839	100.5351	10.7		https://www.google.com/maps/search/13.7839,100.5351	https://data.globe.gov/system/photos/2019/06/10/1096859/original.jpg	https://data.globe.gov/system/photos/2019/06/10/1096861/original.jpg	https://data.globe.gov/system/photos/2019/06/10/1096860/original.jpg	https://data.globe.gov/system/photos/2019/06/10/1096862/original.jpg	https://data.globe.gov/system/photos/2019/06/10/1096864/original.jpg
GV_97	13.786	100.5307	7.4		https://www.google.com/maps/search/13.786,100.5307	https://data.globe.gov/system/photos/2019/06/10/1096912/original.jpg	https://data.globe.gov/system/photos/2019/06/10/1096914/original.jpg	https://data.globe.gov/system/photos/2019/06/10/1096913/original.jpg	https://data.globe.gov/system/photos/2019/06/10/1096915/original.jpg	https://data.globe.gov/system/photos/2019/06/10/1096917/original.jpg
GV_98	13.7839	100.5337	10.5		https://www.google.com/maps/search/13.7839,100.5337	https://data.globe.gov/system/photos/2019/06/10/1096920/original.jpg	https://data.globe.gov/system/photos/2019/06/10/1096922/original.jpg	https://data.globe.gov/system/photos/2019/06/10/1096921/original.jpg	https://data.globe.gov/system/photos/2019/06/10/1096923/original.jpg	https://data.globe.gov/system/photos/2019/06/10/1096925/original.jpg
GV_99	13.7848	100.533	5.4		https://www.google.com/maps/search/13.7848,100.533	https://data.globe.gov/system/photos/2019/06/10/1097026/original.jpg	https://data.globe.gov/system/photos/2019/06/10/1097028/original.jpg	https://data.globe.gov/system/photos/2019/06/10/1097027/original.jpg	https://data.globe.gov/system/photos/2019/06/10/1097029/original.jpg	https://data.globe.gov/system/photos/2019/06/10/1097032/original.jpg
GV_100	13.784	100.5334	9.4		https://www.google.com/maps/search/13.784,100.5334	https://data.globe.gov/system/photos/2019/06/10/1097045/original.jpg	https://data.globe.gov/system/photos/2019/06/10/1097047/original.jpg	https://data.globe.gov/system/photos/2019/06/10/1097046/original.jpg	https://data.globe.gov/system/photos/2019/06/10/1097048/original.jpg	https://data.globe.gov/system/photos/2019/06/10/1097050/original.jpg
GV_101	63.8069	-148.9546	534.4		https://www.google.com/maps/search/63.8069,-148.9546	https://data.globe.gov/system/photos/2019/06/10/1097143/original.jpg	https://data.globe.gov/system/photos/2019/06/10/1097145/original.jpg	https://data.globe.gov/system/photos/2019/06/10/1097144/original.jpg	https://data.globe.gov/system/photos/2019/06/10/1097146/original.jpg	https://data.globe.gov/system/photos/2019/06/10/1097148/original.jpg
GV_102	63.807	-148.9541	534.2		https://www.google.com/maps/search/63.807,-148.9541	https://data.globe.gov/system/photos/2019/06/10/1097149/original.jpg	https://data.globe.gov/system/photos/2019/06/10/1097151/original.jpg	https://data.globe.gov/system/photos/2019/06/10/1097150/original.jpg	https://data.globe.gov/system/photos/2019/06/10/1097152/original.jpg	https://data.globe.gov/system/photos/2019/06/10/1097154/original.jpg
GV_103	63.8069	-148.9539	535		https://www.google.com/maps/search/63.8069,-148.9539	https://data.globe.gov/system/photos/2019/06/10/1097155/original.jpg	https://data.globe.gov/system/photos/2019/06/10/1097157/original.jpg	https://data.globe.gov/system/photos/2019/06/10/1097156/original.jpg	https://data.globe.gov/system/photos/2019/06/10/1097158/original.jpg	https://data.globe.gov/system/photos/2019/06/10/1097159/original.jpg
GV_104	63.807	-148.9511	534.1		https://www.google.com/maps/search/63.807,-148.9511	https://data.globe.gov/system/photos/2019/06/10/1097165/original.jpg	https://data.globe.gov/system/photos/2019/06/10/1097173/original.jpg	https://data.globe.gov/system/photos/2019/06/10/1097171/original.jpg	https://data.globe.gov/system/photos/2019/06/10/1097174/original.jpg	https://data.globe.gov/system/photos/2019/06/10/1097176/original.jpg
GV_105	64.7923	-148.1509	452.6		https://www.google.com/maps/search/64.7923,-148.1509	https://data.globe.gov/system/photos/2019/06/10/1097177/original.jpg	https://data.globe.gov/system/photos/2019/06/10/1097179/original.jpg	https://data.globe.gov/system/photos/2019/06/10/1097178/original.jpg	https://data.globe.gov/system/photos/2019/06/10/1097180/original.jpg	https://data.globe.gov/system/photos/2019/06/10/1097182/original.jpg
GV_106	13.8856	100.5869	3.4		https://www.google.com/maps/search/13.8856,100.5869	https://data.globe.gov/system/photos/2019/06/10/1097215/original.jpg	https://data.globe.gov/system/photos/2019/06/10/1097217/original.jpg	https://data.globe.gov/system/photos/2019/06/10/1097216/original.jpg	https://data.globe.gov/system/photos/2019/06/10/1097218/original.jpg	https://data.globe.gov/system/photos/2019/06/10/1097220/original.jpg
GV_107	43.131	-82.4923	177.9		https://www.google.com/maps/search/43.131,-82.4923	https://data.globe.gov/system/photos/2019/06/09/1097234/original.jpg	https://data.globe.gov/system/photos/2019/06/09/1097236/original.jpg	https://data.globe.gov/system/photos/2019/06/09/1097235/original.jpg	https://data.globe.gov/system/photos/2019/06/09/1097237/original.jpg	https://data.globe.gov/system/photos/2019/06/09/1097239/original.jpg
GV_108	13.784782	100.534474	6.7		https://www.google.com/maps/search/13.784782,100.53447	https://data.globe.gov/system/photos/2019/06/10/1097246/original.jpg	https://data.globe.gov/system/photos/2019/06/10/1097248/original.jpg	https://data.globe.gov/system/photos/2019/06/10/1097247/original.jpg	https://data.globe.gov/system/photos/2019/06/10/1097249/original.jpg	https://data.globe.gov/system/photos/2019/06/10/1097251/original.jpg
GV_109	65.1291	-147.4986	209.4		https://www.google.com/maps/search/65.1291,-147.4986	https://data.globe.gov/system/photos/2019/06/09/1097371/original.jpg	https://data.globe.gov/system/photos/2019/06/09/1097373/original.jpg	https://data.globe.gov/system/photos/2019/06/09/1097372/original.jpg	https://data.globe.gov/system/photos/2019/06/09/1097374/original.jpg	https://data.globe.gov/system/photos/2019/06/09/1097376/original.jpg
GV_110	65.1269	-147.5001	208.5		https://www.google.com/maps/search/65.1269,-147.5001	https://data.globe.gov/system/photos/2019/06/09/1097382/original.jpg	https://data.globe.gov/system/photos/2019/06/09/1097384/original.jpg	https://data.globe.gov/system/photos/2019/06/09/1097383/original.jpg	https://data.globe.gov/system/photos/2019/06/09/1097385/original.jpg	https://data.globe.gov/system/photos/2019/06/09/1097387/original.jpg
GV_111	65.1152	-147.4997	206.7		https://www.google.com/maps/search/65.1152,-147.4997	https://data.globe.gov/system/photos/2019/06/09/1097388/original.jpg	https://data.globe.gov/system/photos/2019/06/09/1097390/original.jpg	https://data.globe.gov/system/photos/2019/06/09/1097389/original.jpg	https://data.globe.gov/system/photos/2019/06/09/1097391/original.jpg	https://data.globe.gov/system/photos/2019/06/09/1097393/original.jpg
GV_112	64.8578	-147.8544	179.9		https://www.google.com/maps/search/64.8578,-147.8544	https://data.globe.gov/system/photos/2019/06/09/1097394/original.jpg	https://data.globe.gov/system/photos/2019/06/09/1097396/original.jpg	https://data.globe.gov/system/photos/2019/06/09/1097395/original.jpg	https://data.globe.gov/system/photos/2019/06/09/1097397/original.jpg	https://data.globe.gov/system/photos/2019/06/09/1097399/original.jpg
GV_113	64.8583	-147.8551	188.6		https://www.google.com/maps/search/64.8583,-147.8551	https://data.globe.gov/system/photos/2019/06/09/1097400/original.jpg	https://data.globe.gov/system/photos/2019/06/09/1097402/original.jpg	https://data.globe.gov/system/photos/2019/06/09/1097401/original.jpg	https://data.globe.gov/system/photos/2019/06/09/1097403/original.jpg	https://data.globe.gov/system/photos/2019/06/09/1097405/original.jpg
GV_114	64.8581	-147.8556	185.4		https://www.google.com/maps/search/64.8581,-147.8556	https://data.globe.gov/system/photos/2019/06/09/1097406/original.jpg	https://data.globe.gov/system/photos/2019/06/09/1097408/original.jpg	https://data.globe.gov/system/photos/2019/06/09/1097407/original.jpg	https://data.globe.gov/system/photos/2019/06/09/1097409/original.jpg	https://data.globe.gov/system/photos/2019/06/09/1097411/original.jpg
GV_115	63.7408	-148.9124	465.8		https://www.google.com/maps/search/63.7408,-148.9124	https://data.globe.gov/system/photos/2019/06/10/1097423/original.jpg	https://data.globe.gov/system/photos/2019/06/10/1097425/original.jpg	https://data.globe.gov/system/photos/2019/06/10/1097424/original.jpg	https://data.globe.gov/system/photos/2019/06/10/1097426/original.jpg	https://data.globe.gov/system/photos/2019/06/10/1097428/original.jpg
GV_116	63.8098	-148.9645	536.2		https://www.google.com/maps/search/63.8098,-148.9645	https://data.globe.gov/system/photos/2019/06/10/1097429/original.jpg	https://data.globe.gov/system/photos/2019/06/10/1097431/original.jpg	https://data.globe.gov/system/photos/2019/06/10/1097430/original.jpg	https://data.globe.gov/system/photos/2019/06/10/1097432/original.jpg	https://data.globe.gov/system/photos/2019/06/10/1097434/original.jpg
GV_117	63.749	-148.9	482.2		https://www.google.com/maps/search/63.749,-148.9	https://data.globe.gov/system/photos/2019/06/10/1097435/original.jpg	https://data.globe.gov/system/photos/2019/06/10/1097437/original.jpg	https://data.globe.gov/system/photos/2019/06/10/1097436/original.jpg	https://data.globe.gov/system/photos/2019/06/10/1097438/original.jpg	https://data.globe.gov/system/photos/2019/06/10/1097440/original.jpg
GV_118	63.8069	-148.9561	539.1		https://www.google.com/maps/search/63.8069,-148.9561	https://data.globe.gov/system/photos/2019/06/10/1097441/original.jpg	https://data.globe.gov/system/photos/2019/06/10/1097445/original.jpg	https://data.globe.gov/system/photos/2019/06/10/1097442/original.jpg	https://data.globe.gov/system/photos/2019/06/10/1097446/original.jpg	https://data.globe.gov/system/photos/2019/06/10/1097450/original.jpg
GV_119	64.5674	-149.1042	111.4		https://www.google.com/maps/search/64.5674,-149.1042	https://data.globe.gov/system/photos/2019/06/09/1097453/original.jpg	https://data.globe.gov/system/photos/2019/06/09/1097455/original.jpg	https://data.globe.gov/system/photos/2019/06/09/1097454/original.jpg	https://data.globe.gov/system/photos/2019/06/09/1097456/original.jpg	https://data.globe.gov/system/photos/2019/06/09/1097458/original.jpg
GV_120	64.325	-149.0364	159.2		https://www.google.com/maps/search/64.325,-149.0364	https://data.globe.gov/system/photos/2019/06/09/1097459/original.jpg	https://data.globe.gov/system/photos/2019/06/09/1097461/original.jpg	https://data.globe.gov/system/photos/2019/06/09/1097460/original.jpg	https://data.globe.gov/system/photos/2019/06/09/1097462/original.jpg	https://data.globe.gov/system/photos/2019/06/09/1097464/original.jpg
GV_121	64.7849	-148.2393	431.6		https://www.google.com/maps/search/64.7849,-148.2393	https://data.globe.gov/system/photos/2019/06/09/1097470/original.jpg	https://data.globe.gov/system/photos/2019/06/09/1097472/original.jpg	https://data.globe.gov/system/photos/2019/06/09/1097471/original.jpg	https://data.globe.gov/system/photos/2019/06/09/1097473/original.jpg	https://data.globe.gov/system/photos/2019/06/09/1097475/original.jpg
GV_122	64.7224	-148.468	227.5		https://www.google.com/maps/search/64.7224,-148.468	https://data.globe.gov/system/photos/2019/06/09/1097476/original.jpg	https://data.globe.gov/system/photos/2019/06/09/1097478/original.jpg	https://data.globe.gov/system/photos/2019/06/09/1097477/original.jpg	https://data.globe.gov/system/photos/2019/06/09/1097479/original.jpg	https://data.globe.gov/system/photos/2019/06/09/1097481/original.jpg
GV_123	64.7231	-148.4683	232.2		https://www.google.com/maps/search/64.7231,-148.4683	https://data.globe.gov/system/photos/2019/06/09/1097482/original.jpg	https://data.globe.gov/system/photos/2019/06/09/1097484/original.jpg	https://data.globe.gov/system/photos/2019/06/09/1097483/original.jpg	https://data.globe.gov/system/photos/2019/06/09/1097485/original.jpg	https://data.globe.gov/system/photos/2019/06/09/1097487/original.jpg
GV_124	64.606	-149.0896	118.9		https://www.google.com/maps/search/64.606,-149.0896	https://data.globe.gov/system/photos/2019/06/09/1097488/original.jpg	https://data.globe.gov/system/photos/2019/06/09/1097490/original.jpg	https://data.globe.gov/system/photos/2019/06/09/1097489/original.jpg	https://data.globe.gov/system/photos/2019/06/09/1097491/original.jpg	https://data.globe.gov/system/photos/2019/06/09/1097493/original.jpg
GV_125	64.5742	-149.1198	103.9		https://www.google.com/maps/search/64.5742,-149.1198	https://data.globe.gov/system/photos/2019/06/09/1097495/original.jpg	https://data.globe.gov/system/photos/2019/06/09/1097499/original.jpg	https://data.globe.gov/system/photos/2019/06/09/1097497/original.jpg	https://data.globe.gov/system/photos/2019/06/09/1097500/original.jpg	https://data.globe.gov/system/photos/2019/06/09/1097502/original.jpg
GV_126	64.5735	-149.1178	108.1		https://www.google.com/maps/search/64.5735,-149.1178	https://data.globe.gov/system/photos/2019/06/09/1097504/original.jpg	https://data.globe.gov/system/photos/2019/06/09/1097506/original.jpg	https://data.globe.gov/system/photos/2019/06/09/1097505/original.jpg	https://data.globe.gov/system/photos/2019/06/09/1097507/original.jpg	https://data.globe.gov/system/photos/2019/06/09/1097509/original.jpg
GV_127	65.0501	-147.4505	618.4		https://www.google.com/maps/search/65.0501,-147.4505	https://data.globe.gov/system/photos/2019/06/09/1097514/original.jpg	https://data.globe.gov/system/photos/2019/06/09/1097519/original.jpg	https://data.globe.gov/system/photos/2019/06/09/1097517/original.jpg	https://data.globe.gov/system/photos/2019/06/09/1097520/original.jpg	https://data.globe.gov/system/photos/2019/06/09/1097522/original.jpg
GV_128	65.1285	-147.4986	211.1		https://www.google.com/maps/search/65.1285,-147.4986	https://data.globe.gov/system/photos/2019/06/09/1097524/original.jpg	https://data.globe.gov/system/photos/2019/06/09/1097526/original.jpg	https://data.globe.gov/system/photos/2019/06/09/1097525/original.jpg	https://data.globe.gov/system/photos/2019/06/09/1097527/original.jpg	https://data.globe.gov/system/photos/2019/06/09/1097529/original.jpg
GV_129	64.8561	-147.8078	132.8		https://www.google.com/maps/search/64.8561,-147.8078	https://data.globe.gov/system/photos/2019/06/09/1097530/original.jpg	https://data.globe.gov/system/photos/2019/06/09/1097532/original.jpg	https://data.globe.gov/system/photos/2019/06/09/1097531/original.jpg	https://data.globe.gov/system/photos/2019/06/09/1097533/original.jpg	https://data.globe.gov/system/photos/2019/06/09/1097535/original.jpg
GV_130	64.8595	-147.8359	187.8		https://www.google.com/maps/search/64.8595,-147.8359	https://data.globe.gov/system/photos/2019/06/09/1097536/original.jpg	https://data.globe.gov/system/photos/2019/06/09/1097538/original.jpg	https://data.globe.gov/system/photos/2019/06/09/1097537/original.jpg	https://data.globe.gov/system/photos/2019/06/09/1097539/original.jpg	https://data.globe.gov/system/photos/2019/06/09/1097541/original.jpg
GV_131	64.8599	-147.8384	184.4		https://www.google.com/maps/search/64.8599,-147.8384	https://data.globe.gov/system/photos/2019/06/09/1097542/original.jpg	https://data.globe.gov/system/photos/2019/06/09/1097544/original.jpg	https://data.globe.gov/system/photos/2019/06/09/1097543/original.jpg	https://data.globe.gov/system/photos/2019/06/09/1097545/original.jpg	https://data.globe.gov/system/photos/2019/06/09/1097547/original.jpg
GV_132	64.8582	-147.8342	184.6		https://www.google.com/maps/search/64.8582,-147.8342	https://data.globe.gov/system/photos/2019/06/09/1097548/original.jpg	https://data.globe.gov/system/photos/2019/06/09/1097550/original.jpg	https://data.globe.gov/system/photos/2019/06/09/1097549/original.jpg	https://data.globe.gov/system/photos/2019/06/09/1097551/original.jpg	https://data.globe.gov/system/photos/2019/06/09/1097553/original.jpg
GV_133	64.8593	-147.8388	188.2		https://www.google.com/maps/search/64.8593,-147.8388	https://data.globe.gov/system/photos/2019/06/09/1097554/original.jpg	https://data.globe.gov/system/photos/2019/06/09/1097556/original.jpg	https://data.globe.gov/system/photos/2019/06/09/1097555/original.jpg	https://data.globe.gov/system/photos/2019/06/09/1097557/original.jpg	https://data.globe.gov/system/photos/2019/06/09/1097559/original.jpg
GV_134	64.8366	-147.8179	132.9		https://www.google.com/maps/search/64.8366,-147.8179	https://data.globe.gov/system/photos/2019/06/09/1097560/original.jpg	https://data.globe.gov/system/photos/2019/06/09/1097562/original.jpg	https://data.globe.gov/system/photos/2019/06/09/1097561/original.jpg	https://data.globe.gov/system/photos/2019/06/09/1097563/original.jpg	https://data.globe.gov/system/photos/2019/06/09/1097565/original.jpg
GV_135	65.0457	-147.4093	741.2		https://www.google.com/maps/search/65.0457,-147.4093	https://data.globe.gov/system/photos/2019/06/09/1097566/original.jpg	https://data.globe.gov/system/photos/2019/06/09/1097568/original.jpg	https://data.globe.gov/system/photos/2019/06/09/1097567/original.jpg	https://data.globe.gov/system/photos/2019/06/09/1097569/original.jpg	https://data.globe.gov/system/photos/2019/06/09/1097571/original.jpg
GV_136	65.0462	-147.4087	737		https://www.google.com/maps/search/65.0462,-147.4087	https://data.globe.gov/system/photos/2019/06/09/1097572/original.jpg	https://data.globe.gov/system/photos/2019/06/09/1097574/original.jpg	https://data.globe.gov/system/photos/2019/06/09/1097573/original.jpg	https://data.globe.gov/system/photos/2019/06/09/1097575/original.jpg	https://data.globe.gov/system/photos/2019/06/09/1097577/original.jpg
GV_137	65.0465	-147.4071	731.7		https://www.google.com/maps/search/65.0465,-147.4071	https://data.globe.gov/system/photos/2019/06/09/1097578/original.jpg	https://data.globe.gov/system/photos/2019/06/09/1097580/original.jpg	https://data.globe.gov/system/photos/2019/06/09/1097579/original.jpg	https://data.globe.gov/system/photos/2019/06/09/1097581/original.jpg	https://data.globe.gov/system/photos/2019/06/09/1097583/original.jpg
GV_138	65.1282	-147.4991	210.8		https://www.google.com/maps/search/65.1282,-147.4991	https://data.globe.gov/system/photos/2019/06/09/1097584/original.jpg	https://data.globe.gov/system/photos/2019/06/09/1097586/original.jpg	https://data.globe.gov/system/photos/2019/06/09/1097585/original.jpg	https://data.globe.gov/system/photos/2019/06/09/1097587/original.jpg	https://data.globe.gov/system/photos/2019/06/09/1097589/original.jpg
GV_139	65.0464	-147.406	727.6		https://www.google.com/maps/search/65.0464,-147.406	https://data.globe.gov/system/photos/2019/06/09/1097590/original.jpg	https://data.globe.gov/system/photos/2019/06/09/1097592/original.jpg	https://data.globe.gov/system/photos/2019/06/09/1097591/original.jpg	https://data.globe.gov/system/photos/2019/06/09/1097593/original.jpg	https://data.globe.gov/system/photos/2019/06/09/1097595/original.jpg
GV_140	65.0467	-147.404	714.3		https://www.google.com/maps/search/65.0467,-147.404	https://data.globe.gov/system/photos/2019/06/09/1097596/original.jpg	https://data.globe.gov/system/photos/2019/06/09/1097598/original.jpg	https://data.globe.gov/system/photos/2019/06/09/1097597/original.jpg	https://data.globe.gov/system/photos/2019/06/09/1097599/original.jpg	https://data.globe.gov/system/photos/2019/06/09/1097601/original.jpg
GV_141	65.0471	-147.4371	679.2		https://www.google.com/maps/search/65.0471,-147.4371	https://data.globe.gov/system/photos/2019/06/09/1097602/original.jpg	https://data.globe.gov/system/photos/2019/06/09/1097604/original.jpg	https://data.globe.gov/system/photos/2019/06/09/1097603/original.jpg	https://data.globe.gov/system/photos/2019/06/09/1097605/original.jpg	https://data.globe.gov/system/photos/2019/06/09/1097607/original.jpg
GV_142	64.8179	-147.8688	134.3		https://www.google.com/maps/search/64.8179,-147.8688	https://data.globe.gov/system/photos/2019/06/08/1097608/original.jpg	https://data.globe.gov/system/photos/2019/06/08/1097610/original.jpg	https://data.globe.gov/system/photos/2019/06/08/1097609/original.jpg	https://data.globe.gov/system/photos/2019/06/08/1097611/original.jpg	https://data.globe.gov/system/photos/2019/06/08/1097613/original.jpg
GV_143	64.8376	-147.8194	134.9		https://www.google.com/maps/search/64.8376,-147.8194	https://data.globe.gov/system/photos/2019/06/08/1097614/original.jpg	https://data.globe.gov/system/photos/2019/06/08/1097616/original.jpg	https://data.globe.gov/system/photos/2019/06/08/1097615/original.jpg	https://data.globe.gov/system/photos/2019/06/08/1097617/original.jpg	https://data.globe.gov/system/photos/2019/06/08/1097619/original.jpg
GV_144	64.9294	-147.6286	225.5		https://www.google.com/maps/search/64.9294,-147.6286	https://data.globe.gov/system/photos/2019/06/08/1097620/original.jpg	https://data.globe.gov/system/photos/2019/06/08/1097622/original.jpg	https://data.globe.gov/system/photos/2019/06/08/1097621/original.jpg	https://data.globe.gov/system/photos/2019/06/08/1097623/original.jpg	https://data.globe.gov/system/photos/2019/06/08/1097625/original.jpg
GV_145	65.0093	-147.4991	358.5		https://www.google.com/maps/search/65.0093,-147.4991	https://data.globe.gov/system/photos/2019/06/08/1097626/original.jpg	https://data.globe.gov/system/photos/2019/06/08/1097628/original.jpg	https://data.globe.gov/system/photos/2019/06/08/1097627/original.jpg	https://data.globe.gov/system/photos/2019/06/08/1097629/original.jpg	https://data.globe.gov/system/photos/2019/06/08/1097631/original.jpg
GV_146	65.0097	-147.4985	361.5		https://www.google.com/maps/search/65.0097,-147.4985	https://data.globe.gov/system/photos/2019/06/08/1097632/original.jpg	https://data.globe.gov/system/photos/2019/06/08/1097634/original.jpg	https://data.globe.gov/system/photos/2019/06/08/1097633/original.jpg	https://data.globe.gov/system/photos/2019/06/08/1097635/original.jpg	https://data.globe.gov/system/photos/2019/06/08/1097637/original.jpg
GV_147	65.0092	-147.5008	360		https://www.google.com/maps/search/65.0092,-147.5008	https://data.globe.gov/system/photos/2019/06/08/1097638/original.jpg	https://data.globe.gov/system/photos/2019/06/08/1097640/original.jpg	https://data.globe.gov/system/photos/2019/06/08/1097639/original.jpg	https://data.globe.gov/system/photos/2019/06/08/1097641/original.jpg	https://data.globe.gov/system/photos/2019/06/08/1097643/original.jpg
GV_148	65.0089	-147.5025	358.6		https://www.google.com/maps/search/65.0089,-147.5025	https://data.globe.gov/system/photos/2019/06/08/1097644/original.jpg	https://data.globe.gov/system/photos/2019/06/08/1097646/original.jpg	https://data.globe.gov/system/photos/2019/06/08/1097645/original.jpg	https://data.globe.gov/system/photos/2019/06/08/1097647/original.jpg	https://data.globe.gov/system/photos/2019/06/08/1097649/original.jpg
GV_149	65.0195	-147.4818	422.8		https://www.google.com/maps/search/65.0195,-147.4818	https://data.globe.gov/system/photos/2019/06/08/1097650/original.jpg	https://data.globe.gov/system/photos/2019/06/08/1097652/original.jpg	https://data.globe.gov/system/photos/2019/06/08/1097651/original.jpg	https://data.globe.gov/system/photos/2019/06/08/1097653/original.jpg	https://data.globe.gov/system/photos/2019/06/08/1097655/original.jpg
GV_150	41.1808	-95.9161	357		https://www.google.com/maps/search/41.1808,-95.9161	https://data.globe.gov/system/photos/2019/06/10/1097707/original.jpg	https://data.globe.gov/system/photos/2019/06/10/1097709/original.jpg	https://data.globe.gov/system/photos/2019/06/10/1097708/original.jpg	https://data.globe.gov/system/photos/2019/06/10/1097710/original.jpg	https://data.globe.gov/system/photos/2019/06/10/1097718/original.jpg
GV_151	64.8583	-147.8411	187.4		https://www.google.com/maps/search/64.8583,-147.8411	https://data.globe.gov/system/photos/2019/06/10/1097925/original.jpg	https://data.globe.gov/system/photos/2019/06/10/1097927/original.jpg	https://data.globe.gov/system/photos/2019/06/10/1097926/original.jpg	https://data.globe.gov/system/photos/2019/06/10/1097928/original.jpg	https://data.globe.gov/system/photos/2019/06/10/1097930/original.jpg
GV_152	64.8592	-147.8472	186.2		https://www.google.com/maps/search/64.8592,-147.8472	https://data.globe.gov/system/photos/2019/06/10/1097931/original.jpg	https://data.globe.gov/system/photos/2019/06/10/1097933/original.jpg	https://data.globe.gov/system/photos/2019/06/10/1097932/original.jpg	https://data.globe.gov/system/photos/2019/06/10/1097934/original.jpg	https://data.globe.gov/system/photos/2019/06/10/1097936/original.jpg
GV_153	64.859	-147.8501	187.8		https://www.google.com/maps/search/64.859,-147.8501	https://data.globe.gov/system/photos/2019/06/10/1097937/original.jpg	https://data.globe.gov/system/photos/2019/06/10/1097939/original.jpg	https://data.globe.gov/system/photos/2019/06/10/1097938/original.jpg	https://data.globe.gov/system/photos/2019/06/10/1097940/original.jpg	https://data.globe.gov/system/photos/2019/06/10/1097942/original.jpg
GV_154	64.8557	-147.8248	151.2		https://www.google.com/maps/search/64.8557,-147.8248	https://data.globe.gov/system/photos/2019/06/10/1097993/original.jpg	https://data.globe.gov/system/photos/2019/06/10/1097995/original.jpg	https://data.globe.gov/system/photos/2019/06/10/1097994/original.jpg	https://data.globe.gov/system/photos/2019/06/10/1097996/original.jpg	https://data.globe.gov/system/photos/2019/06/10/1097998/original.jpg
GV_155	64.8569	-147.8247	158.3		https://www.google.com/maps/search/64.8569,-147.8247	https://data.globe.gov/system/photos/2019/06/10/1097999/original.jpg	https://data.globe.gov/system/photos/2019/06/10/1098001/original.jpg	https://data.globe.gov/system/photos/2019/06/10/1098000/original.jpg	https://data.globe.gov/system/photos/2019/06/10/1098002/original.jpg	https://data.globe.gov/system/photos/2019/06/10/1098004/original.jpg
GV_156	64.858	-147.8315	181		https://www.google.com/maps/search/64.858,-147.8315	https://data.globe.gov/system/photos/2019/06/10/1098005/original.jpg	https://data.globe.gov/system/photos/2019/06/10/1098007/original.jpg	https://data.globe.gov/system/photos/2019/06/10/1098006/original.jpg	https://data.globe.gov/system/photos/2019/06/10/1098008/original.jpg	https://data.globe.gov/system/photos/2019/06/10/1098010/original.jpg
GV_157	64.8591	-147.8494	186		https://www.google.com/maps/search/64.8591,-147.8494	https://data.globe.gov/system/photos/2019/06/10/1098072/original.jpg	https://data.globe.gov/system/photos/2019/06/10/1098074/original.jpg	https://data.globe.gov/system/photos/2019/06/10/1098073/original.jpg	https://data.globe.gov/system/photos/2019/06/10/1098075/original.jpg	https://data.globe.gov/system/photos/2019/06/10/1098077/original.jpg
GV_158	64.8588	-147.8494	186.2		https://www.google.com/maps/search/64.8588,-147.8494	https://data.globe.gov/system/photos/2019/06/10/1098095/original.jpg	https://data.globe.gov/system/photos/2019/06/10/1098097/original.jpg	https://data.globe.gov/system/photos/2019/06/10/1098096/original.jpg	https://data.globe.gov/system/photos/2019/06/10/1098098/original.jpg	https://data.globe.gov/system/photos/2019/06/10/1098100/original.jpg
GV_159	13.7843	100.5339	8		https://www.google.com/maps/search/13.7843,100.5339	https://data.globe.gov/system/photos/2019/06/10/1098121/original.jpg	https://data.globe.gov/system/photos/2019/06/10/1098123/original.jpg	https://data.globe.gov/system/photos/2019/06/10/1098122/original.jpg	https://data.globe.gov/system/photos/2019/06/10/1098124/original.jpg	https://data.globe.gov/system/photos/2019/06/10/1098126/original.jpg
GV_160	64.861	-147.8314	179.2		https://www.google.com/maps/search/64.861,-147.8314	https://data.globe.gov/system/photos/2019/06/11/1098324/original.jpg	https://data.globe.gov/system/photos/2019/06/11/1098326/original.jpg	https://data.globe.gov/system/photos/2019/06/11/1098325/original.jpg	https://data.globe.gov/system/photos/2019/06/11/1098327/original.jpg	https://data.globe.gov/system/photos/2019/06/11/1098329/original.jpg
GV_161	64.8605	-147.8327	182.9		https://www.google.com/maps/search/64.8605,-147.8327	https://data.globe.gov/system/photos/2019/06/11/1098330/original.jpg	https://data.globe.gov/system/photos/2019/06/11/1098332/original.jpg	https://data.globe.gov/system/photos/2019/06/11/1098331/original.jpg	https://data.globe.gov/system/photos/2019/06/11/1098333/original.jpg	https://data.globe.gov/system/photos/2019/06/11/1098335/original.jpg
GV_162	50.4929	21.6786	147.5		https://www.google.com/maps/search/50.4929,21.6786	https://data.globe.gov/system/photos/2019/06/11/1098395/original.jpg	https://data.globe.gov/system/photos/2019/06/11/1098397/original.jpg	https://data.globe.gov/system/photos/2019/06/11/1098396/original.jpg	https://data.globe.gov/system/photos/2019/06/11/1098398/original.jpg	https://data.globe.gov/system/photos/2019/06/11/1098400/original.jpg
GV_163	50.4921	21.679	146.5		https://www.google.com/maps/search/50.4921,21.679	https://data.globe.gov/system/photos/2019/06/11/1098402/original.jpg	https://data.globe.gov/system/photos/2019/06/11/1098404/original.jpg	https://data.globe.gov/system/photos/2019/06/11/1098403/original.jpg	https://data.globe.gov/system/photos/2019/06/11/1098405/original.jpg	https://data.globe.gov/system/photos/2019/06/11/1098407/original.jpg
GV_164	50.4908	21.6784	146.5		https://www.google.com/maps/search/50.4908,21.6784	https://data.globe.gov/system/photos/2019/06/11/1098408/original.jpg	https://data.globe.gov/system/photos/2019/06/11/1098410/original.jpg	https://data.globe.gov/system/photos/2019/06/11/1098409/original.jpg	https://data.globe.gov/system/photos/2019/06/11/1098411/original.jpg	https://data.globe.gov/system/photos/2019/06/11/1098413/original.jpg
GV_165	46.5556	-112.317	1888.5		https://www.google.com/maps/search/46.5556,-112.317	https://data.globe.gov/system/photos/2019/06/11/1098600/original.jpg	https://data.globe.gov/system/photos/2019/06/11/1098602/original.jpg	https://data.globe.gov/system/photos/2019/06/11/1098601/original.jpg	https://data.globe.gov/system/photos/2019/06/11/1098603/original.jpg	https://data.globe.gov/system/photos/2019/06/11/1098605/original.jpg
GV_166	42.0842	-87.9781	207.6		https://www.google.com/maps/search/42.0842,-87.9781	https://data.globe.gov/system/photos/2019/06/11/1098712/original.jpg	https://data.globe.gov/system/photos/2019/06/11/1098714/original.jpg	https://data.globe.gov/system/photos/2019/06/11/1098713/original.jpg	https://data.globe.gov/system/photos/2019/06/11/1098715/original.jpg	https://data.globe.gov/system/photos/2019/06/11/1098717/original.jpg
GV_167	40.7566	-82.52	391.1		https://www.google.com/maps/search/40.7566,-82.52	https://data.globe.gov/system/photos/2019/06/11/1098757/original.jpg	https://data.globe.gov/system/photos/2019/06/11/1098759/original.jpg	https://data.globe.gov/system/photos/2019/06/11/1098758/original.jpg	https://data.globe.gov/system/photos/2019/06/11/1098760/original.jpg	https://data.globe.gov/system/photos/2019/06/11/1098762/original.jpg
GV_168	64.8589	-147.8496	186.2		https://www.google.com/maps/search/64.8589,-147.8496	https://data.globe.gov/system/photos/2019/06/11/1099044/original.jpg	https://data.globe.gov/system/photos/2019/06/11/1099046/original.jpg	https://data.globe.gov/system/photos/2019/06/11/1099045/original.jpg	https://data.globe.gov/system/photos/2019/06/11/1099047/original.jpg	https://data.globe.gov/system/photos/2019/06/11/1099049/original.jpg
GV_169	64.859	-147.8492	186		https://www.google.com/maps/search/64.859,-147.8492	https://data.globe.gov/system/photos/2019/06/11/1099052/original.jpg	https://data.globe.gov/system/photos/2019/06/11/1099054/original.jpg	https://data.globe.gov/system/photos/2019/06/11/1099053/original.jpg	https://data.globe.gov/system/photos/2019/06/11/1099055/original.jpg	https://data.globe.gov/system/photos/2019/06/11/1099057/original.jpg
GV_170	64.8584	-147.8494	185.6		https://www.google.com/maps/search/64.8584,-147.8494	https://data.globe.gov/system/photos/2019/06/11/1099070/original.jpg	https://data.globe.gov/system/photos/2019/06/11/1099072/original.jpg	https://data.globe.gov/system/photos/2019/06/11/1099071/original.jpg	https://data.globe.gov/system/photos/2019/06/11/1099073/original.jpg	https://data.globe.gov/system/photos/2019/06/11/1099075/original.jpg
GV_171	64.8594	-147.8505	185.7		https://www.google.com/maps/search/64.8594,-147.8505	https://data.globe.gov/system/photos/2019/06/11/1099104/original.jpg	https://data.globe.gov/system/photos/2019/06/11/1099106/original.jpg	https://data.globe.gov/system/photos/2019/06/11/1099105/original.jpg	https://data.globe.gov/system/photos/2019/06/11/1099107/original.jpg	https://data.globe.gov/system/photos/2019/06/11/1099109/original.jpg
GV_172	64.8542	-147.8557	139.4		https://www.google.com/maps/search/64.8542,-147.8557	https://data.globe.gov/system/photos/2019/06/11/1099115/original.jpg	https://data.globe.gov/system/photos/2019/06/11/1099117/original.jpg	https://data.globe.gov/system/photos/2019/06/11/1099116/original.jpg	https://data.globe.gov/system/photos/2019/06/11/1099118/original.jpg	https://data.globe.gov/system/photos/2019/06/11/1099120/original.jpg
GV_173	64.8591	-147.8497	186.5		https://www.google.com/maps/search/64.8591,-147.8497	https://data.globe.gov/system/photos/2019/06/11/1099137/original.jpg	https://data.globe.gov/system/photos/2019/06/11/1099149/original.jpg	https://data.globe.gov/system/photos/2019/06/11/1099148/original.jpg	https://data.globe.gov/system/photos/2019/06/11/1099150/original.jpg	https://data.globe.gov/system/photos/2019/06/11/1099152/original.jpg

GV_174	64.8595	-147.8487	185		https://www.google.com/maps/search/64.8595,-147.8487	https://data.globe.gov/system/photos/2019/06/11/1099181/original.jpg	https://data.globe.gov/system/photos/2019/06/11/1099183/original.jpg	https://data.globe.gov/system/photos/2019/06/11/1099182/original.jpg	https://data.globe.gov/system/photos/2019/06/11/1099184/original.jpg	https://data.globe.gov/system/photos/2019/06/11/1099186/original.jpg
GV_175	53.263	-0.6206	8		https://www.google.com/maps/search/53.263,-0.6206	https://data.globe.gov/system/photos/2019/06/11/1099257/original.jpg	https://data.globe.gov/system/photos/2019/06/11/1099259/original.jpg	https://data.globe.gov/system/photos/2019/06/11/1099258/original.jpg	https://data.globe.gov/system/photos/2019/06/11/1099260/original.jpg	https://data.globe.gov/system/photos/2019/06/11/1099262/original.jpg
GV_176	64.7061	-148.6437	407.1		https://www.google.com/maps/search/64.7061,-148.6437	https://data.globe.gov/system/photos/2019/06/12/1099471/original.jpg	https://data.globe.gov/system/photos/2019/06/12/1099473/original.jpg	https://data.globe.gov/system/photos/2019/06/12/1099472/original.jpg	https://data.globe.gov/system/photos/2019/06/12/1099474/original.jpg	https://data.globe.gov/system/photos/2019/06/12/1099476/original.jpg
GV_177	13.7844	100.5336	6.7		https://www.google.com/maps/search/13.7844,100.5336	https://data.globe.gov/system/photos/2019/06/12/1099488/original.jpg	https://data.globe.gov/system/photos/2019/06/12/1099490/original.jpg	https://data.globe.gov/system/photos/2019/06/12/1099489/original.jpg	https://data.globe.gov/system/photos/2019/06/12/1099491/original.jpg	https://data.globe.gov/system/photos/2019/06/12/1099493/original.jpg
GV_178	13.7845	100.5342	7.5		https://www.google.com/maps/search/13.7845,100.5342	https://data.globe.gov/system/photos/2019/06/12/1099622/original.jpg	https://data.globe.gov/system/photos/2019/06/12/1099624/original.jpg	https://data.globe.gov/system/photos/2019/06/12/1099623/original.jpg	https://data.globe.gov/system/photos/2019/06/12/1099625/original.jpg	https://data.globe.gov/system/photos/2019/06/12/1099627/original.jpg
GV_179	13.7844	100.5341	7.8		https://www.google.com/maps/search/13.7844,100.5341	https://data.globe.gov/system/photos/2019/06/12/1099628/original.jpg	https://data.globe.gov/system/photos/2019/06/12/1099630/original.jpg	https://data.globe.gov/system/photos/2019/06/12/1099629/original.jpg	https://data.globe.gov/system/photos/2019/06/12/1099631/original.jpg	https://data.globe.gov/system/photos/2019/06/12/1099633/original.jpg
GV_180	13.7843	100.5344	9.3		https://www.google.com/maps/search/13.7843,100.5344	https://data.globe.gov/system/photos/2019/06/12/1099669/original.jpg	https://data.globe.gov/system/photos/2019/06/12/1099671/original.jpg	https://data.globe.gov/system/photos/2019/06/12/1099670/original.jpg	https://data.globe.gov/system/photos/2019/06/12/1099672/original.jpg	https://data.globe.gov/system/photos/2019/06/12/1099674/original.jpg
GV_181	13.7843	100.534	8.2		https://www.google.com/maps/search/13.7843,100.534	https://data.globe.gov/system/photos/2019/06/12/1099675/original.jpg	https://data.globe.gov/system/photos/2019/06/12/1099683/original.jpg	https://data.globe.gov/system/photos/2019/06/12/1099682/original.jpg	https://data.globe.gov/system/photos/2019/06/12/1099684/original.jpg	https://data.globe.gov/system/photos/2019/06/12/1099686/original.jpg
GV_182	42.086	-83.2096	177.6		https://www.google.com/maps/search/42.086,-83.2096	https://data.globe.gov/system/photos/2019/06/12/1099800/original.jpg	https://data.globe.gov/system/photos/2019/06/12/1099802/original.jpg	https://data.globe.gov/system/photos/2019/06/12/1099801/original.jpg	https://data.globe.gov/system/photos/2019/06/12/1099803/original.jpg	https://data.globe.gov/system/photos/2019/06/12/1099805/original.jpg
GV_183	64.837	-147.7966	132.9		https://www.google.com/maps/search/64.837,-147.7966	https://data.globe.gov/system/photos/2019/06/12/1099819/original.jpg	https://data.globe.gov/system/photos/2019/06/12/1099821/original.jpg	https://data.globe.gov/system/photos/2019/06/12/1099820/original.jpg	https://data.globe.gov/system/photos/2019/06/12/1099822/original.jpg	https://data.globe.gov/system/photos/2019/06/12/1099824/original.jpg
GV_184	64.7063	-148.6418	408.7		https://www.google.com/maps/search/64.7063,-148.6418	https://data.globe.gov/system/photos/2019/06/12/1099831/original.jpg	https://data.globe.gov/system/photos/2019/06/12/1099833/original.jpg	https://data.globe.gov/system/photos/2019/06/12/1099832/original.jpg	https://data.globe.gov/system/photos/2019/06/12/1099834/original.jpg	https://data.globe.gov/system/photos/2019/06/12/1099836/original.jpg
GV_185	13.7847	100.5341	6.1		https://www.google.com/maps/search/13.7847,100.5341	https://data.globe.gov/system/photos/2019/06/10/1099844/original.jpg	https://data.globe.gov/system/photos/2019/06/10/1099851/original.jpg	https://data.globe.gov/system/photos/2019/06/10/1099847/original.jpg	https://data.globe.gov/system/photos/2019/06/10/1099854/original.jpg	https://data.globe.gov/system/photos/2019/06/10/1099857/original.jpg
GV_186	64.7064	-148.6432	407.1		https://www.google.com/maps/search/64.7064,-148.6432	https://data.globe.gov/system/photos/2019/06/12/1099880/original.jpg	https://data.globe.gov/system/photos/2019/06/12/1099882/original.jpg	https://data.globe.gov/system/photos/2019/06/12/1099881/original.jpg	https://data.globe.gov/system/photos/2019/06/12/1099883/original.jpg	https://data.globe.gov/system/photos/2019/06/12/1099885/original.jpg
GV_187	-39.9488	-71.0824	789.8		https://www.google.com/maps/search/-39.9488,-71.0824	https://data.globe.gov/system/photos/2019/06/12/1099987/original.jpg	https://data.globe.gov/system/photos/2019/06/12/1099989/original.jpg	https://data.globe.gov/system/photos/2019/06/12/1099988/original.jpg	https://data.globe.gov/system/photos/2019/06/12/1099990/original.jpg	https://data.globe.gov/system/photos/2019/06/12/1099992/original.jpg
GV_188	-39.9499	-71.0647	779.2		https://www.google.com/maps/search/-39.9499,-71.0647	https://data.globe.gov/system/photos/2019/06/12/1100019/original.jpg	https://data.globe.gov/system/photos/2019/06/12/1100021/original.jpg	https://data.globe.gov/system/photos/2019/06/12/1100020/original.jpg	https://data.globe.gov/system/photos/2019/06/12/1100022/original.jpg	https://data.globe.gov/system/photos/2019/06/12/1100024/original.jpg
GV_189	45.7382	-108.432	1059.9		https://www.google.com/maps/search/45.7382,-108.432	https://data.globe.gov/system/photos/2019/06/12/1100146/original.jpg	https://data.globe.gov/system/photos/2019/06/12/1100148/original.jpg	https://data.globe.gov/system/photos/2019/06/12/1100147/original.jpg	https://data.globe.gov/system/photos/2019/06/12/1100149/original.jpg	https://data.globe.gov/system/photos/2019/06/12/1100151/original.jpg
GV_190	64.8592	-147.8493	185.7		https://www.google.com/maps/search/64.8592,-147.8493	https://data.globe.gov/system/photos/2019/06/11/1100221/original.jpg	https://data.globe.gov/system/photos/2019/06/11/1100223/original.jpg	https://data.globe.gov/system/photos/2019/06/11/1100222/original.jpg	https://data.globe.gov/system/photos/2019/06/11/1100224/original.jpg	https://data.globe.gov/system/photos/2019/06/11/1100226/original.jpg
GV_191	46.6664	-117.0227	788.1		https://www.google.com/maps/search/46.6664,-117.0227	https://data.globe.gov/system/photos/2019/06/12/1100244/original.jpg	https://data.globe.gov/system/photos/2019/06/12/1100246/original.jpg	https://data.globe.gov/system/photos/2019/06/12/1100245/original.jpg	https://data.globe.gov/system/photos/2019/06/12/1100247/original.jpg	https://data.globe.gov/system/photos/2019/06/12/1100249/original.jpg
GV_192	64.8589	-147.8495	186.1		https://www.google.com/maps/search/64.8589,-147.8495	https://data.globe.gov/system/photos/2019/06/11/1100256/original.jpg	https://data.globe.gov/system/photos/2019/06/11/1100260/original.jpg	https://data.globe.gov/system/photos/2019/06/11/1100257/original.jpg	https://data.globe.gov/system/photos/2019/06/11/1100262/original.jpg	https://data.globe.gov/system/photos/2019/06/11/1100268/original.jpg
GV_193	64.8603	-147.8523	185.5		https://www.google.com/maps/search/64.8603,-147.8523	https://data.globe.gov/system/photos/2019/06/12/1100269/original.jpg	https://data.globe.gov/system/photos/2019/06/12/1100271/original.jpg	https://data.globe.gov/system/photos/2019/06/12/1100270/original.jpg	https://data.globe.gov/system/photos/2019/06/12/1100272/original.jpg	https://data.globe.gov/system/photos/2019/06/12/1100274/original.jpg
GV_194	64.8602	-147.8579	182.6		https://www.google.com/maps/search/64.8602,-147.8579	https://data.globe.gov/system/photos/2019/06/12/1100351/original.jpg	https://data.globe.gov/system/photos/2019/06/12/1100353/original.jpg	https://data.globe.gov/system/photos/2019/06/12/1100352/original.jpg	https://data.globe.gov/system/photos/2019/06/12/1100354/original.jpg	https://data.globe.gov/system/photos/2019/06/12/1100356/original.jpg
GV_195	64.8657	-147.8579	157.4		https://www.google.com/maps/search/64.8657,-147.8579	https://data.globe.gov/system/photos/2019/06/12/1100363/original.jpg	https://data.globe.gov/system/photos/2019/06/12/1100365/original.jpg	https://data.globe.gov/system/photos/2019/06/12/1100364/original.jpg	https://data.globe.gov/system/photos/2019/06/12/1100366/original.jpg	https://data.globe.gov/system/photos/2019/06/12/1100368/original.jpg
GV_196	64.8586	-147.8498	186.3		https://www.google.com/maps/search/64.8586,-147.8498	https://data.globe.gov/system/photos/2019/06/11/1100379/original.jpg	https://data.globe.gov/system/photos/2019/06/11/1100381/original.jpg	https://data.globe.gov/system/photos/2019/06/11/1100380/original.jpg	https://data.globe.gov/system/photos/2019/06/11/1100382/original.jpg	https://data.globe.gov/system/photos/2019/06/11/1100384/original.jpg
GV_197	64.8591	-147.8497	186.5		https://www.google.com/maps/search/64.8591,-147.8497	https://data.globe.gov/system/photos/2019/06/12/1100390/original.jpg	https://data.globe.gov/system/photos/2019/06/12/1100392/original.jpg	https://data.globe.gov/system/photos/2019/06/12/1100391/original.jpg	https://data.globe.gov/system/photos/2019/06/12/1100393/original.jpg	https://data.globe.gov/system/photos/2019/06/12/1100395/original.jpg
GV_198	13.784229	100.534393	9.7		https://www.google.com/maps/search/13.784229,100.53439	https://data.globe.gov/system/photos/2019/06/13/1100396/original.jpg	https://data.globe.gov/system/photos/2019/06/13/1100398/original.jpg	https://data.globe.gov/system/photos/2019/06/13/1100397/original.jpg	https://data.globe.gov/system/photos/2019/06/13/1100399/original.jpg	https://data.globe.gov/system/photos/2019/06/13/1100401/original.jpg
GV_199	13.7843	100.5336	7.5		https://www.google.com/maps/search/13.7843,100.5336	https://data.globe.gov/system/photos/2019/06/13/1100586/original.jpg	https://data.globe.gov/system/photos/2019/06/13/1100588/original.jpg	https://data.globe.gov/system/photos/2019/06/13/1100587/original.jpg	https://data.globe.gov/system/photos/2019/06/13/1100589/original.jpg	https://data.globe.gov/system/photos/2019/06/13/1100591/original.jpg
GV_200	13.7845	100.5338	6.4		https://www.google.com/maps/search/13.7845,100.5338	https://data.globe.gov/system/photos/2019/06/13/1100604/original.jpg	https://data.globe.gov/system/photos/2019/06/13/1100606/original.jpg	https://data.globe.gov/system/photos/2019/06/13/1100605/original.jpg	https://data.globe.gov/system/photos/2019/06/13/1100607/original.jpg	https://data.globe.gov/system/photos/2019/06/13/1100609/original.jpg
GV_201	13.7844	100.5339	7.3		https://www.google.com/maps/search/13.7844,100.5339	https://data.globe.gov/system/photos/2019/06/13/1100613/original.jpg	https://data.globe.gov/system/photos/2019/06/13/1100615/original.jpg	https://data.globe.gov/system/photos/2019/06/13/1100614/original.jpg	https://data.globe.gov/system/photos/2019/06/13/1100616/original.jpg	https://data.globe.gov/system/photos/2019/06/13/1100618/original.jpg
GV_202	13.7843	100.5338	7.7		https://www.google.com/maps/search/13.7843,100.5338	https://data.globe.gov/system/photos/2019/06/13/1100627/original.jpg	https://data.globe.gov/system/photos/2019/06/13/1100629/original.jpg	https://data.globe.gov/system/photos/2019/06/13/1100628/original.jpg	https://data.globe.gov/system/photos/2019/06/13/1100630/original.jpg	https://data.globe.gov/system/photos/2019/06/13/1100632/original.jpg
GV_203	13.7844	100.5337	6.9		https://www.google.com/maps/search/13.7844,100.5337	https://data.globe.gov/system/photos/2019/06/13/1100633/original.jpg	https://data.globe.gov/system/photos/2019/06/13/1100635/original.jpg	https://data.globe.gov/system/photos/2019/06/13/1100634/original.jpg	https://data.globe.gov/system/photos/2019/06/13/1100636/original.jpg	https://data.globe.gov/system/photos/2019/06/13/1100638/original.jpg
GV_204	13.7844	100.5339	7.3		https://www.google.com/maps/search/13.7844,100.5339	https://data.globe.gov/system/photos/2019/06/13/1100649/original.jpg	https://data.globe.gov/system/photos/2019/06/13/1100651/original.jpg	https://data.globe.gov/system/photos/2019/06/13/1100650/original.jpg	https://data.globe.gov/system/photos/2019/06/13/1100652/original.jpg	https://data.globe.gov/system/photos/2019/06/13/1100654/original.jpg
GV_205	13.7844	100.5338	7		https://www.google.com/maps/search/13.7844,100.5338	https://data.globe.gov/system/photos/2019/06/13/1100657/original.jpg	https://data.globe.gov/system/photos/2019/06/13/1100659/original.jpg	https://data.globe.gov/system/photos/2019/06/13/1100658/original.jpg	https://data.globe.gov/system/photos/2019/06/13/1100660/original.jpg	https://data.globe.gov/system/photos/2019/06/13/1100662/original.jpg
GV_206	24.4354	118.3148	21.7		https://www.google.com/maps/search/24.4354,118.3148	https://data.globe.gov/system/photos/2019/06/13/1100664/original.jpg	https://data.globe.gov/system/photos/2019/06/13/1100666/original.jpg	https://data.globe.gov/system/photos/2019/06/13/1100665/original.jpg	https://data.globe.gov/system/photos/2019/06/13/1100667/original.jpg	https://data.globe.gov/system/photos/2019/06/13/1100669/original.jpg
GV_207	50.5137	21.6615	153.1		https://www.google.com/maps/search/50.5137,21.6615	https://data.globe.gov/system/photos/2019/06/09/1100765/original.jpg	https://data.globe.gov/system/photos/2019/06/09/1100767/original.jpg	https://data.globe.gov/system/photos/2019/06/09/1100766/original.jpg	https://data.globe.gov/system/photos/2019/06/09/1100769/original.jpg	https://data.globe.gov/system/photos/2019/06/09/1100772/original.jpg
GV_208	50.5207	21.6812	148.5		https://www.google.com/maps/search/50.5207,21.6812	https://data.globe.gov/system/photos/2019/06/11/1100768/original.jpg	https://data.globe.gov/system/photos/2019/06/11/1100775/original.jpg	https://data.globe.gov/system/photos/2019/06/11/1100773/original.jpg	https://data.globe.gov/system/photos/2019/06/11/1100776/original.jpg	https://data.globe.gov/system/photos/2019/06/11/1100778/original.jpg
GV_209	13.7832	100.5336	13.4		https://www.google.com/maps/search/13.7832,100.5336	https://data.globe.gov/system/photos/2019/06/13/1100782/original.jpg	https://data.globe.gov/system/photos/2019/06/13/1100784/original.jpg	https://data.globe.gov/system/photos/2019/06/13/1100783/original.jpg	https://data.globe.gov/system/photos/2019/06/13/1100785/original.jpg	https://data.globe.gov/system/photos/2019/06/13/1100787/original.jpg
GV_210	13.7844	100.5337	6.9		https://www.google.com/maps/search/13.7844,100.5337	https://data.globe.gov/system/photos/2019/06/13/1100788/original.jpg	https://data.globe.gov/system/photos/2019/06/13/1100796/original.jpg	https://data.globe.gov/system/photos/2019/06/13/1100791/original.jpg	https://data.globe.gov/system/photos/2019/06/13/1100797/original.jpg	https://data.globe.gov/system/photos/2019/06/13/1100799/original.jpg
GV_211	13.7842	100.534	8.9		https://www.google.com/maps/search/13.7842,100.534	https://data.globe.gov/system/photos/2019/06/13/1100811/original.jpg	https://data.globe.gov/system/photos/2019/06/13/1100813/original.jpg	https://data.globe.gov/system/photos/2019/06/13/1100812/original.jpg	https://data.globe.gov/system/photos/2019/06/13/1100814/original.jpg	https://data.globe.gov/system/photos/2019/06/13/1100816/original.jpg
GV_212	13.7838	100.5339	11.5		https://www.google.com/maps/search/13.7838,100.5339	https://data.globe.gov/system/photos/2019/06/13/1100827/original.jpg	https://data.globe.gov/system/photos/2019/06/13/1100829/original.jpg	https://data.globe.gov/system/photos/2019/06/13/1100828/original.jpg	https://data.globe.gov/system/photos/2019/06/13/1100830/original.jpg	https://data.globe.gov/system/photos/2019/06/13/1100832/original.jpg
GV_213	46.0044	-122.8473	4.4		https://www.google.com/maps/search/46.0044,-122.8473	https://data.globe.gov/system/photos/2019/06/13/1101280/original.jpg	https://data.globe.gov/system/photos/2019/06/13/1101282/original.jpg	https://data.globe.gov/system/photos/2019/06/13/1101281/original.jpg	https://data.globe.gov/system/photos/2019/06/13/1101283/original.jpg	https://data.globe.gov/system/photos/2019/06/13/1101285/original.jpg
GV_214	64.859	-147.8504	187.9		https://www.google.com/maps/search/64.859,-147.8504	https://data.globe.gov/system/photos/2019/06/13/1101341/original.jpg	https://data.globe.gov/system/photos/2019/06/13/1101343/original.jpg	https://data.globe.gov/system/photos/2019/06/13/1101342/original.jpg	https://data.globe.gov/system/photos/2019/06/13/1101344/original.jpg	https://data.globe.gov/system/photos/2019/06/13/1101346/original.jpg
GV_215	50.734	12.4019	340.7		https://www.google.com/maps/search/50.734,12.4019	https://data.globe.gov/system/photos/2019/06/11/1101367/original.jpg	https://data.globe.gov/system/photos/2019/06/11/1101369/original.jpg	https://data.globe.gov/system/photos/2019/06/11/1101368/original.jpg	https://data.globe.gov/system/photos/2019/06/11/1101370/original.jpg	https://data.globe.gov/system/photos/2019/06/11/1101372/original.jpg
GV_216	64.8578	-147.8531	177.7		https://www.google.com/maps/search/64.8578,-147.8531	https://data.globe.gov/system/photos/2019/06/13/1101451/original.jpg	https://data.globe.gov/system/photos/2019/06/13/1101454/original.jpg	https://data.globe.gov/system/photos/2019/06/13/1101453/original.jpg	https://data.globe.gov/system/photos/2019/06/13/1101455/original.jpg	https://data.globe.gov/system/photos/2019/06/13/1101457/original.jpg
GV_217	39.166	-114.3794	1919.9		https://www.google.com/maps/search/39.166,-114.3794	https://data.globe.gov/system/photos/2019/06/10/1101482/original.jpg	https://data.globe.gov/system/photos/2019/06/10/1101484/original.jpg	https://data.globe.gov/system/photos/2019/06/10/1101483/original.jpg	https://data.globe.gov/system/photos/2019/06/10/1101485/original.jpg	https://data.globe.gov/system/photos/2019/06/10/1101487/original.jpg
GV_218	64.8593	-147.8501	186.1		https://www.google.com/maps/search/64.8593,-147.8501	https://data.globe.gov/system/photos/2019/06/13/1101512/original.jpg	https://data.globe.gov/system/photos/2019/06/13/1101514/original.jpg	https://data.globe.gov/system/photos/2019/06/13/1101513/original.jpg	https://data.globe.gov/system/photos/2019/06/13/1101515/original.jpg	https://data.globe.gov/system/photos/2019/06/13/1101517/original.jpg
GV_219	-27.4685	153.104	7.6		https://www.google.com/maps/search/-27.4685,153.104	https://data.globe.gov/system/photos/2019/06/14/1101542/original.jpg	https://data.globe.gov/system/photos/2019/06/14/1101544/original.jpg	https://data.globe.gov/system/photos/2019/06/14/1101543/original.jpg	https://data.globe.gov/system/photos/2019/06/14/1101545/original.jpg	https://data.globe.gov/system/photos/2019/06/14/1101547/original.jpg
GV_220	13.7831	100.5327	9.5		https://www.google.com/maps/search/13.7831,100.5327	https://data.globe.gov/system/photos/2019/06/12/1101556/original.jpg	https://data.globe.gov/system/photos/2019/06/12/1101558/original.jpg	https://data.globe.gov/system/photos/2019/06/12/1101557/original.jpg	https://data.globe.gov/system/photos/2019/06/12/1101559/original.jpg	https://data.globe.gov/system/photos/2019/06/12/1101569/original.jpg
GV_221	24.4332	118.3147	20.9		https://www.google.com/maps/search/24.4332,118.3147	https://data.globe.gov/system/photos/2019/06/14/1101619/original.jpg	https://data.globe.gov/system/photos/2019/06/14/1101621/original.jpg	https://data.globe.gov/system/photos/2019/06/14/1101620/original.jpg	https://data.globe.gov/system/photos/2019/06/14/1101622/original.jpg	https://data.globe.gov/system/photos/2019/06/14/1101624/original.jpg
GV_222	0.1393	34.5363	1462.7		https://www.google.com/maps/search/0.1393,34.5363	https://data.globe.gov/system/photos/2019/06/14/1101631/original.jpg	https://data.globe.gov/system/photos/2019/06/14/1101633/original.jpg	https://data.globe.gov/system/photos/2019/06/14/1101632/original.jpg	https://data.globe.gov/system/photos/2019/06/14/1101634/original.jpg	https://data.globe.gov/system/photos/2019/06/14/1101636/original.jpg
GV_223	13.7842	100.5339	8.7		https://www.google.com/maps/search/13.7842,100.5339	https://data.globe.gov/system/photos/2019/06/14/1101852/original.jpg	https://data.globe.gov/system/photos/2019/06/14/1101854/original.jpg	https://data.globe.gov/system/photos/2019/06/14/1101853/original.jpg	https://data.globe.gov/system/photos/2019/06/14/1101855/original.jpg	https://data.globe.gov/system/photos/2019/06/14/1101857/original.jpg
GV_224	64.8605	-147.8399	184.1		https://www.google.com/maps/search/64.8605,-147.8399	https://data.globe.gov/system/photos/2019/06/14/1101911/original.jpg	https://data.globe.gov/system/photos/2019/06/14/1101913/original.jpg	https://data.globe.gov/system/photos/2019/06/14/1101912/original.jpg	https://data.globe.gov/system/photos/2019/06/14/1101914/original.jpg	https://data.globe.gov/system/photos/2019/06/14/1101916/original.jpg
GV_225	64.8609	-147.8404	182.4		https://www.google.com/maps/search/64.8609,-147.8404	https://data.globe.gov/system/photos/2019/06/14/1101917/original.jpg	https://data.globe.gov/system/photos/2019/06/14/1101919/original.jpg	https://data.globe.gov/system/photos/2019/06/14/1101918/original.jpg	https://data.globe.gov/system/photos/2019/06/14/1101920/original.jpg	https://data.globe.gov/system/photos/2019/06/14/1101922/original.jpg
GV_226	64.8591	-147.8396	189.2		https://www.google.com/maps/search/64.8591,-147.8396	https://data.globe.gov/system/photos/2019/06/14/1101923/original.jpg	https://data.globe.gov/system/photos/2019/06/14/1101925/original.jpg	https://data.globe.gov/system/photos/2019/06/14/1101924/original.jpg	https://data.globe.gov/system/photos/2019/06/14/1101926/original.jpg	https://data.globe.gov/system/photos/2019/06/14/1101928/original.jpg
GV_227	64.8589	-147.8394	190.2		https://www.google.com/maps/search/64.8589,-147.8394	https://data.globe.gov/system/photos/2019/06/14/1101929/original.jpg	https://data.globe.gov/system/photos/2019/06/14/1101931/original.jpg	https://data.globe.gov/system/photos/2019/06/14/1101930/original.jpg	https://data.globe.gov/system/photos/2019/06/14/1101932/original.jpg	https://data.globe.gov/system/photos/2019/06/14/1101934/original.jpg
GV_228	64.8588	-147.8467	185.4		https://www.google.com/maps/search/64.8588,-147.8467	https://data.globe.gov/system/photos/2019/06/14/1101935/original.jpg	https://data.globe.gov/system/photos/2019/06/14/1101937/original.jpg	https://data.globe.gov/system/photos/2019/06/14/1101936/original.jpg	https://data.globe.gov/system/photos/2019/06/14/1101938/original.jpg	https://data.globe.gov/system/photos/2019/06/14/1101940/original.jpg
GV_229	64.8583	-147.8442	184.2		https://www.google.com/maps/search/64.8583,-147.8442	https://data.globe.gov/system/photos/2019/06/14/1101941/original.jpg	https://data.globe.gov/system/photos/2019/06/14/1101943/original.jpg	https://data.globe.gov/system/photos/2019/06/14/1101942/original.jpg	https://data.globe.gov/system/photos/2019/06/14/1101944/original.jpg	https://data.globe.gov/system/photos/2019/06/14/1101946/original.jpg
GV_230	64.8586	-147.8399	189.5		https://www.google.com/maps/search/64.8586,-147.8399	https://data.globe.gov/system/photos/2019/06/14/1101947/original.jpg	https://data.globe.gov/system/photos/2019/06/14/1101949/original.jpg	https://data.globe.gov/system/photos/2019/06/14/1101948/original.jpg	https://data.globe.gov/system/photos/2019/06/14/1101950/original.jpg	https://data.globe.gov/system/photos/2019/06/14/1101952/original.jpg
GV_231	64.8587	-147.8406	188		https://www.google.com/maps/search/64.8587,-147.8406	https://data.globe.gov/system/photos/2019/06/14/1101953/original.jpg	https://data.globe.gov/system/photos/2019/06/14/1101955/original.jpg	https://data.globe.gov/system/photos/2019/06/14/1101954/original.jpg	https://data.globe.gov/system/photos/2019/06/14/1101956/original.jpg	https://data.globe.gov/system/photos/2019/06/14/1101958/original.jpg
GV_232	64.8582	-147.8385	188.6		https://www.google.com/maps/search/64.8582,-147.8385	https://data.globe.gov/system/photos/2019/06/14/1101959/original.jpg	https://data.globe.gov/system/photos/2019/06/14/1101961/original.jpg	https://data.globe.gov/system/photos/2019/06/14/1101960/original.jpg	https://data.globe.gov/system/photos/2019/06/14/1101962/original.jpg	https://data.globe.gov/system/photos/2019/06/14/1101964/original.jpg
GV_233	64.859	-147.8437	186.5		https://www.google.com/maps/search/64.859,-147.8437	https://data.globe.gov/system/photos/2019/06/14/1101965/original.jpg	https://data.globe.gov/system/photos/2019/06/14/1101967/original.jpg	https://data.globe.gov/system/photos/2019/06/14/1101966/original.jpg	https://data.globe.gov/system/photos/2019/06/14/1101968/original.jpg	https://data.globe.gov/system/photos/2019/06/14/1101970/original.jpg
GV_234	64.8591	-147.8449	184.5		https://www.google.com/maps/search/64.8591,-147.8449	https://data.globe.gov/system/photos/2019/06/14/1101971/original.jpg	https://data.globe.gov/system/photos/2019/06/14/1101973/original.jpg	https://data.globe.gov/system/photos/2019/06/14/1101972/original.jpg	https://data.globe.gov/system/photos/2019/06/14/1101974/original.jpg	https://data.globe.gov/system/photos/2019/06/14/1101976/original.jpg
GV_235	64.8589	-147.8452	185.2		https://www.google.com/maps/search/64.8589,-147.8452	https://data.globe.gov/system/photos/2019/06/14/1101977/original.jpg	https://data.globe.gov/system/photos/2019/06/14/1101979/original.jpg	https://data.globe.gov/system/photos/2019/06/14/1101978/original.jpg	https://data.globe.gov/system/photos/2019/06/14/1101980/original.jpg	https://data.globe.gov/system/photos/2019/06/14/1101982/original.jpg
GV_236	64.8588	-147.8461	185.4		https://www.google.com/maps/search/64.8588,-147.8461	https://data.globe.gov/system/photos/2019/06/14/1101983/original.jpg	https://data.globe.gov/system/photos/2019/06/14/1101985/original.jpg	https://data.globe.gov/system/photos/2019/06/14/1101984/original.jpg	https://data.globe.gov/system/photos/2019/06/14/1101986/original.jpg	https://data.globe.gov/system/photos/2019/06/14/1101988/original.jpg
GV_237	64.8581	-147.8418	186.3		https://www.google.com/maps/search/64.8581,-147.8418	https://data.globe.gov/system/photos/2019/06/14/1101989/original.jpg	https://data.globe.gov/system/photos/2019/06/14/1101991/original.jpg	https://data.globe.gov/system/photos/2019/06/14/1101990/original.jpg	https://data.globe.gov/system/photos/2019/06/14/1101992/original.jpg	https://data.globe.gov/system/photos/2019/06/14/1101994/original.jpg
GV_238	64.8577	-147.841	180.5		https://www.google.com/maps/search/64.8577,-147.841	https://data.globe.gov/system/photos/2019/06/14/1101995/original.jpg	https://data.globe.gov/system/photos/2019/06/14/1101997/original.jpg	https://data.globe.gov/system/photos/2019/06/14/1101996/original.jpg	https://data.globe.gov/system/photos/2019/06/14/1101998/original.jpg	https://data.globe.gov/system/photos/2019/06/14/1102000/original.jpg
GV_239	64.8571	-147.8397	172.1		https://www.google.com/maps/search/64.8571,-147.8397	https://data.globe.gov/system/photos/2019/06/14/1102001/original.jpg	https://data.globe.gov/system/photos/2019/06/14/1102003/original.jpg	https://data.globe.gov/system/photos/2019/06/14/1102002/original.jpg	https://data.globe.gov/system/photos/2019/06/14/1102004/original.jpg	https://data.globe.gov/system/photos/2019/06/14/1102006/original.jpg
GV_240	13.7843	100.5344	9.3		https://www.google.com/maps/search/13.7843,100.5344	https://data.globe.gov/system/photos/2019/06/14/1102024/original.jpg	https://data.globe.gov/system/photos/2019/06/14/1102026/original.jpg	https://data.globe.gov/system/photos/2019/06/14/1102025/original.jpg	https://data.globe.gov/system/photos/2019/06/14/1102027/original.jpg	https://data.globe.gov/system/photos/2019/06/14/1102029/original.jpg
GV_241	13.7843	100.5341	8.5		https://www.google.com/maps/search/13.7843,100.5341	https://data.globe.gov/system/photos/2019/06/14/1102030/original.jpg	https://data.globe.gov/system/photos/2019/06/14/1102032/original.jpg	https://data.globe.gov/system/photos/2019/06/14/1102031/original.jpg	https://data.globe.gov/system/photos/2019/06/14/1102033/original.jpg	https://data.globe.gov/system/photos/2019/06/14/1102035/original.jpg
GV_242	13.7843	100.5344	9.3		https://www.google.com/maps/search/13.7843,100.5344	https://data.globe.gov/system/photos/2019/06/14/1102048/original.jpg	https://data.globe.gov/system/photos/2019/06/14/1102050/original.jpg	https://data.globe.gov/system/photos/2019/06/14/1102049/original.jpg	https://data.globe.gov/system/photos/2019/06/14/1102051/original.jpg	https://data.globe.gov/system/photos/2019/06/14/1102053/original.jpg
GV_243	50.5135	21.6607	154.6		https://www.google.com/maps/search/50.5135,21.6607	https://data.globe.gov/system/photos/2019/06/09/1102162/original.jpg	https://data.globe.gov/system/photos/2019/06/09/1102164/original.jpg	https://data.globe.gov/system/photos/2019/06/09/1102163/original.jpg	https://data.globe.gov/system/photos/2019/06/09/1102165/original.jpg	https://data.globe.gov/system/photos/2019/06/09/1102167/original.jpg
GV_244	53.265	-0.6244	7		https://www.google.com/maps/search/53.265,-0.6244	https://data.globe.gov/system/photos/2019/06/14/1102211/original.jpg	https://data.globe.gov/system/photos/2019/06/14/1102213/original.jpg	https://data.globe.gov/system/photos/2019/06/14/1102212/original.jpg	https://data.globe.gov/system/photos/2019/06/14/1102215/original.jpg	https://data.globe.gov/system/photos/2019/06/14/1102217/original.jpg
GV_245	64.8593	-147.8414	186.1		https://www.google.com/maps/search/64.8593,-147.8414	https://data.globe.gov/system/photos/2019/06/14/1102261/original.jpg	https://data.globe.gov/system/photos/2019/06/14/1102263/original.jpg	https://data.globe.gov/system/photos/2019/06/14/1102262/original.jpg	https://data.globe.gov/system/photos/2019/06/14/1102264/original.jpg	https://data.globe.gov/system/photos/2019/06/14/1102266/original.jpg
GV_246	41.9568	-83.4097	185.9		https://www.google.com/maps/search/41.9568,-83.4097	https://data.globe.gov/system/photos/2019/06/14/1102338/original.jpg	https://data.globe.gov/system/photos/2019/06/14/1102340/original.jpg	https://data.globe.gov/system/photos/2019/06/14/1102339/original.jpg	https://data.globe.gov/system/photos/2019/06/14/1102341/original.jpg	https://data.globe.gov/system/photos/2019/06/14/1102343/original.jpg
GV_247	45.6675	-111.0541	1486.2		https://www.google.com/maps/search/45.6675,-111.0541	https://data.globe.gov/system/photos/2019/06/14/1102515/original.jpg	https://data.globe.gov/system/photos/2019/06/14/1102517/original.jpg	https://data.globe.gov/system/photos/2019/06/14/1102516/original.jpg	https://data.globe.gov/system/photos/2019/06/14/1102518/original.jpg	https://data.globe.gov/system/photos/2019/06/14/1102520/original.jpg
GV_248	48.7139	-113.1191	1522.9		https://www.google.com/maps/search/48.7139,-113.1191	https://data.globe.gov/system/photos/2019/06/15/1102575/original.jpg	https://data.globe.gov/system/photos/2019/06/15/1102577/original.jpg	https://data.globe.gov/system/photos/2019/06/15/1102576/original.jpg	https://data.globe.gov/system/photos/2019/06/15/1102578/original.jpg	https://data.globe.gov/system/photos/2019/06/15/1102580/original.jpg
GV_249	-32.1469	115.9228	27.7		https://www.google.com/maps/search/-32.1469,115.9228	https://data.globe.gov/system/photos/2019/06/15/1102610/original.jpg	https://data.globe.gov/system/photos/2019/06/15/1102612/original.jpg	https://data.globe.gov/system/photos/2019/06/15/1102611/original.jpg	https://data.globe.gov/system/photos/2019/06/15/1102613/original.jpg	https://data.globe.gov/system/photos/2019/06/15/1102615/original.jpg
GV_250	-31.9793	115.8961	19.8		https://www.google.com/maps/search/-31.9793,115.8961	https://data.globe.gov/system/photos/2019/06/15/1102628/original.jpg	https://data.globe.gov/system/photos/2019/06/15/1102630/original.jpg	https://data.globe.gov/system/photos/2019/06/15/1102629/original.jpg	https://data.globe.gov/system/photos/2019/06/15/1102631/original.jpg	https://data.globe.gov/system/photos/2019/06/15/1102633/original.jpg
GV_251	53.4125	-0.647	22.7		https://www.google.com/maps/search/53.4125,-0.647	https://data.globe.gov/system/photos/2019/06/15/1102867/original.jpg	https://data.globe.gov/system/photos/2019/06/15/1102869/original.jpg	https://data.globe.gov/system/photos/2019/06/15/1102868/original.jpg	https://data.globe.gov/system/photos/2019/06/15/1102870/original.jpg	https://data.globe.gov/system/photos/2019/06/15/1102872/original.jpg
GV_252	64.8583	-147.831	181.5		https://www.google.com/maps/search/64.8583,-147.831	https://data.globe.gov/system/photos/2019/06/15/1102916/original.jpg	https://data.globe.gov/system/photos/2019/06/15/1102918/original.jpg	https://data.globe.gov/system/photos/2019/06/15/1102917/original.jpg	https://data.globe.gov/system/photos/2019/06/15/1102919/original.jpg	https://data.globe.gov/system/photos/2019/06/15/1102921/original.jpg
GV_253	38.8493	-77.1518	75		https://www.google.com/maps/search/38.8493,-77.1518	https://data.globe.gov/system/photos/2019/06/15/1103226/original.jpg	https://data.globe.gov/system/photos/2019/06/15/1103228/original.jpg	https://data.globe.gov/system/photos/2019/06/15/1103227/original.jpg	https://data.globe.gov/system/photos/2019/06/15/1103229/original.jpg	https://data.globe.gov/system/photos/2019/06/15/1103231/original.jpg
GV_254	63.7449	-148.9543	1107.9		https://www.google.com/maps/search/63.7449,-148.9543	https://data.globe.gov/system/photos/2019/06/15/1103248/original.jpg	https://data.globe.gov/system/photos/2019/06/15/1103250/original.jpg	https://data.globe.gov/system/photos/2019/06/15/1103249/original.jpg	https://data.globe.gov/system/photos/2019/06/15/1103251/original.jpg	https://data.globe.gov/system/photos/2019/06/15/1103253/original.jpg
GV_255	13.949	1.0037	238.2		https://www.google.com/maps/search/13.949,1.0037	https://data.globe.gov/system/photos/2019/06/16/1103450/original.jpg	https://data.globe.gov/system/photos/2019/06/16/1103452/original.jpg	https://data.globe.gov/system/photos/2019/06/16/1103451/original.jpg	https://data.globe.gov/system/photos/2019/06/16/1103453/original.jpg	https://data.globe.gov/system/photos/2019/06/16/1103455/original.jpg
GV_256	13.9428	100.4643	5.5		https://www.google.com/maps/search/13.9428,100.4643	https://data.globe.gov/system/photos/2019/06/16/1103459/original.jpg	https://data.globe.gov/system/photos/2019/06/16/1103461/original.jpg	https://data.globe.gov/system/photos/2019/06/16/1103460/original.jpg	https://data.globe.gov/system/photos/2019/06/16/1103462/original.jpg	https://data.globe.gov/system/photos/2019/06/16/1103464/original.jpg
GV_257	13.942845	100.464267	5.5		https://www.google.com/maps/search/13.942845,100.46426	https://data.globe.gov/system/photos/2019/06/16/1103472/original.jpg	https://data.globe.gov/system/photos/2019/06/16/1103474/original.jpg	https://data.globe.gov/system/photos/2019/06/16/1103473/original.jpg	https://data.globe.gov/system/photos/2019/06/16/1103475/original.jpg	https://data.globe.gov/system/photos/2019/06/16/1103477/original.jpg
GV_258	39.6823	-76.4206	149.4		https://www.google.com/maps/search/39.6823,-76.4206	https://data.globe.gov/system/photos/2019/06/17/1104019/original.jpg	https://data.globe.gov/system/photos/2019/06/17/1104021/original.jpg	https://data.globe.gov/system/photos/2019/06/17/1104020/original.jpg	https://data.globe.gov/system/photos/2019/06/17/1104022/original.jpg	https://data.globe.gov/system/photos/2019/06/17/1104030/original.jpg
GV_259	47.0784	-112.6181	1620.5		https://www.google.com/maps/search/47.0784,-112.6181	https://data.globe.gov/system/photos/2019/06/15/1104059/original.jpg	https://data.globe.gov/system/photos/2019/06/15/1104061/original.jpg	https://data.globe.gov/system/photos/2019/06/15/1104060/original.jpg	https://data.globe.gov/system/photos/2019/06/15/1104062/original.jpg	https://data.globe.gov/system/photos/2019/06/15/1104064/original.jpg
GV_260	46.9749	-112.3579	1870.1		https://www.google.com/maps/search/46.9749,-112.3579	https://data.globe.gov/system/photos/2019/06/16/1104071/original.jpg	https://data.globe.gov/system/photos/2019/06/16/1104073/original.jpg	https://data.globe.gov/system/photos/2019/06/16/1104072/original.jpg	https://data.globe.gov/system/photos/2019/06/16/1104074/original.jpg	https://data.globe.gov/system/photos/2019/06/16/1104076/original.jpg
GV_261	13.7848	100.5338	4.7		https://www.google.com/maps/search/13.7848,100.5338	https://data.globe.gov/system/photos/2019/06/17/1104094/original.jpg	https://data.globe.gov/system/photos/2019/06/17/1104096/original.jpg	https://data.globe.gov/system/photos/2019/06/17/1104095/original.jpg	https://data.globe.gov/system/photos/2019/06/17/1104097/original.jpg	https://data.globe.gov/system/photos/2019/06/17/1104099/original.jpg
GV_262	13.7845	100.5343	7.8		https://www.google.com/maps/search/13.7845,100.5343	https://data.globe.gov/system/photos/2019/06/17/1104142/original.jpg	https://data.globe.gov/system/photos/2019/06/17/1104144/original.jpg	https://data.globe.gov/system/photos/2019/06/17/1104143/original.jpg	https://data.globe.gov/system/photos/2019/06/17/1104145/original.jpg	https://data.globe.gov/system/photos/2019/06/17/1104147/original.jpg
GV_263	13.7843	100.5341	8.5		https://www.google.com/maps/search/13.7843,100.5341	https://data.globe.gov/system/photos/2019/06/17/1104182/original.jpg	https://data.globe.gov/system/photos/2019/06/17/1104184/original.jpg	https://data.globe.gov/system/photos/2019/06/17/1104183/original.jpg	https://data.globe.gov/system/photos/2019/06/17/1104185/original.jpg	https://data.globe.gov/system/photos/2019/06/17/1104187/original.jpg

GV_264	13.7861	100.5337	6.1		https://www.google.com/maps/search/13.7861,100.5337	https://data.globe.gov/system/photos/2019/06/17/1104216/original.jpg	https://data.globe.gov/system/photos/2019/06/17/1104218/original.jpg	https://data.globe.gov/system/photos/2019/06/17/1104217/original.jpg	https://data.globe.gov/system/photos/2019/06/17/1104219/original.jpg	https://data.globe.gov/system/photos/2019/06/17/1104221/original.jpg
GV_265	45.5128	-122.6835	42.6		https://www.google.com/maps/search/45.5128,-122.6835	https://data.globe.gov/system/photos/2019/06/15/1104234/original.jpg	https://data.globe.gov/system/photos/2019/06/15/1104236/original.jpg	https://data.globe.gov/system/photos/2019/06/15/1104235/original.jpg	https://data.globe.gov/system/photos/2019/06/15/1104237/original.jpg	https://data.globe.gov/system/photos/2019/06/15/1104239/original.jpg
GV_266	45.5214	-122.6729	9.9		https://www.google.com/maps/search/45.5214,-122.6729	https://data.globe.gov/system/photos/2019/06/15/1104240/original.jpg	https://data.globe.gov/system/photos/2019/06/15/1104242/original.jpg	https://data.globe.gov/system/photos/2019/06/15/1104241/original.jpg	https://data.globe.gov/system/photos/2019/06/15/1104243/original.jpg	https://data.globe.gov/system/photos/2019/06/15/1104245/original.jpg
GV_267	13.8861	100.7138	4.9		https://www.google.com/maps/search/13.8861,100.7138	https://data.globe.gov/system/photos/2019/06/17/1104373/original.jpg	https://data.globe.gov/system/photos/2019/06/17/1104375/original.jpg	https://data.globe.gov/system/photos/2019/06/17/1104374/original.jpg	https://data.globe.gov/system/photos/2019/06/17/1104376/original.jpg	https://data.globe.gov/system/photos/2019/06/17/1104378/original.jpg
GV_268	45.6668	-111.0595	1489.1		https://www.google.com/maps/search/45.6668,-111.0595	https://data.globe.gov/system/photos/2019/06/17/1104405/original.jpg	https://data.globe.gov/system/photos/2019/06/17/1104407/original.jpg	https://data.globe.gov/system/photos/2019/06/17/1104406/original.jpg	https://data.globe.gov/system/photos/2019/06/17/1104408/original.jpg	https://data.globe.gov/system/photos/2019/06/17/1104410/original.jpg
GV_269	39.2542	-77.1964	176.8		https://www.google.com/maps/search/39.2542,-77.1964	https://data.globe.gov/system/photos/2019/06/14/1104424/original.jpg	https://data.globe.gov/system/photos/2019/06/14/1104426/original.jpg	https://data.globe.gov/system/photos/2019/06/14/1104425/original.jpg	https://data.globe.gov/system/photos/2019/06/14/1104427/original.jpg	https://data.globe.gov/system/photos/2019/06/14/1104429/original.jpg
GV_270	35.3093	-106.577	1552.1		https://www.google.com/maps/search/35.3093,-106.577	https://data.globe.gov/system/photos/2019/06/17/1104533/original.jpg	https://data.globe.gov/system/photos/2019/06/17/1104538/original.jpg	https://data.globe.gov/system/photos/2019/06/17/1104535/original.jpg	https://data.globe.gov/system/photos/2019/06/17/1104539/original.jpg	https://data.globe.gov/system/photos/2019/06/17/1104541/original.jpg
GV_271	13.9652	100.4989	7.3		https://www.google.com/maps/search/13.9652,100.4989	https://data.globe.gov/system/photos/2019/06/17/1104791/original.jpg	https://data.globe.gov/system/photos/2019/06/17/1104799/original.jpg	https://data.globe.gov/system/photos/2019/06/17/1104798/original.jpg	https://data.globe.gov/system/photos/2019/06/17/1104800/original.jpg	https://data.globe.gov/system/photos/2019/06/17/1104802/original.jpg
GV_272	13.7847	100.5348	7.7		https://www.google.com/maps/search/13.7847,100.5348	https://data.globe.gov/system/photos/2019/06/18/1104844/original.jpg	https://data.globe.gov/system/photos/2019/06/18/1104846/original.jpg	https://data.globe.gov/system/photos/2019/06/18/1104845/original.jpg	https://data.globe.gov/system/photos/2019/06/18/1104847/original.jpg	https://data.globe.gov/system/photos/2019/06/18/1104849/original.jpg
GV_273	46.7462	-114.172	1019.1		https://www.google.com/maps/search/46.7462,-114.172	https://data.globe.gov/system/photos/2019/06/17/1104905/original.jpg	https://data.globe.gov/system/photos/2019/06/17/1104907/original.jpg	https://data.globe.gov/system/photos/2019/06/17/1104906/original.jpg	https://data.globe.gov/system/photos/2019/06/17/1104908/original.jpg	https://data.globe.gov/system/photos/2019/06/17/1104910/original.jpg
GV_274	46.7573	-114.3141	1102.1		https://www.google.com/maps/search/46.7573,-114.3141	https://data.globe.gov/system/photos/2019/06/17/1104928/original.jpg	https://data.globe.gov/system/photos/2019/06/17/1104931/original.jpg	https://data.globe.gov/system/photos/2019/06/17/1104930/original.jpg	https://data.globe.gov/system/photos/2019/06/17/1104932/original.jpg	https://data.globe.gov/system/photos/2019/06/17/1104934/original.jpg
GV_275	46.6348	-114.578	1593.9		https://www.google.com/maps/search/46.6348,-114.578	https://data.globe.gov/system/photos/2019/06/17/1104935/original.jpg	https://data.globe.gov/system/photos/2019/06/17/1104937/original.jpg	https://data.globe.gov/system/photos/2019/06/17/1104936/original.jpg	https://data.globe.gov/system/photos/2019/06/17/1104938/original.jpg	https://data.globe.gov/system/photos/2019/06/17/1104940/original.jpg
GV_276	46.634	-114.577	1596		https://www.google.com/maps/search/46.634,-114.577	https://data.globe.gov/system/photos/2019/06/17/1104941/original.jpg	https://data.globe.gov/system/photos/2019/06/17/1104943/original.jpg	https://data.globe.gov/system/photos/2019/06/17/1104942/original.jpg	https://data.globe.gov/system/photos/2019/06/17/1104944/original.jpg	https://data.globe.gov/system/photos/2019/06/17/1104946/original.jpg
GV_277	46.5394	-114.6753	1093.7		https://www.google.com/maps/search/46.5394,-114.6753	https://data.globe.gov/system/photos/2019/06/17/1104947/original.jpg	https://data.globe.gov/system/photos/2019/06/17/1104949/original.jpg	https://data.globe.gov/system/photos/2019/06/17/1104948/original.jpg	https://data.globe.gov/system/photos/2019/06/17/1104950/original.jpg	https://data.globe.gov/system/photos/2019/06/17/1104955/original.jpg
GV_278	46.5388	-114.6754	1093.1		https://www.google.com/maps/search/46.5388,-114.6754	https://data.globe.gov/system/photos/2019/06/17/1104960/original.jpg	https://data.globe.gov/system/photos/2019/06/17/1104962/original.jpg	https://data.globe.gov/system/photos/2019/06/17/1104961/original.jpg	https://data.globe.gov/system/photos/2019/06/17/1104963/original.jpg	https://data.globe.gov/system/photos/2019/06/17/1104965/original.jpg
GV_279	46.373	-115.2541	695.3		https://www.google.com/maps/search/46.373,-115.2541	https://data.globe.gov/system/photos/2019/06/18/1104966/original.jpg	https://data.globe.gov/system/photos/2019/06/18/1104968/original.jpg	https://data.globe.gov/system/photos/2019/06/18/1104967/original.jpg	https://data.globe.gov/system/photos/2019/06/18/1104969/original.jpg	https://data.globe.gov/system/photos/2019/06/18/1104971/original.jpg
GV_280	5.6367	-0.1879	87		https://www.google.com/maps/search/5.6367,-0.1879	https://data.globe.gov/system/photos/2019/06/18/1105297/original.jpg	https://data.globe.gov/system/photos/2019/06/18/1105299/original.jpg	https://data.globe.gov/system/photos/2019/06/18/1105298/original.jpg	https://data.globe.gov/system/photos/2019/06/18/1105300/original.jpg	https://data.globe.gov/system/photos/2019/06/18/1105302/original.jpg
GV_281	5.6501	-0.1967	144.3		https://www.google.com/maps/search/5.6501,-0.1967	https://data.globe.gov/system/photos/2019/06/18/1105308/original.jpg	https://data.globe.gov/system/photos/2019/06/18/1105310/original.jpg	https://data.globe.gov/system/photos/2019/06/18/1105309/original.jpg	https://data.globe.gov/system/photos/2019/06/18/1105311/original.jpg	https://data.globe.gov/system/photos/2019/06/18/1105313/original.jpg
GV_282	5.6488	-0.1963	130		https://www.google.com/maps/search/5.6488,-0.1963	https://data.globe.gov/system/photos/2019/06/18/1105318/original.jpg	https://data.globe.gov/system/photos/2019/06/18/1105320/original.jpg	https://data.globe.gov/system/photos/2019/06/18/1105319/original.jpg	https://data.globe.gov/system/photos/2019/06/18/1105321/original.jpg	https://data.globe.gov/system/photos/2019/06/18/1105323/original.jpg
GV_283	50.5174	21.6704	151.2		https://www.google.com/maps/search/50.5174,21.6704	https://data.globe.gov/system/photos/2019/06/05/1105771/original.jpg	https://data.globe.gov/system/photos/2019/06/05/1105773/original.jpg	https://data.globe.gov/system/photos/2019/06/05/1105772/original.jpg	https://data.globe.gov/system/photos/2019/06/05/1105774/original.jpg	https://data.globe.gov/system/photos/2019/06/05/1105776/original.jpg
GV_284	50.507	21.6944	146		https://www.google.com/maps/search/50.507,21.6944	https://data.globe.gov/system/photos/2019/06/05/1105777/original.jpg	https://data.globe.gov/system/photos/2019/06/05/1105779/original.jpg	https://data.globe.gov/system/photos/2019/06/05/1105778/original.jpg	https://data.globe.gov/system/photos/2019/06/05/1105780/original.jpg	https://data.globe.gov/system/photos/2019/06/05/1105782/original.jpg
GV_285	50.5235	21.693	155.4		https://www.google.com/maps/search/50.5235,21.693	https://data.globe.gov/system/photos/2019/06/05/1105783/original.jpg	https://data.globe.gov/system/photos/2019/06/05/1105785/original.jpg	https://data.globe.gov/system/photos/2019/06/05/1105784/original.jpg	https://data.globe.gov/system/photos/2019/06/05/1105786/original.jpg	https://data.globe.gov/system/photos/2019/06/05/1105788/original.jpg
GV_286	50.5252	21.6921	146.8		https://www.google.com/maps/search/50.5252,21.6921	https://data.globe.gov/system/photos/2019/06/05/1105789/original.jpg	https://data.globe.gov/system/photos/2019/06/05/1105791/original.jpg	https://data.globe.gov/system/photos/2019/06/05/1105790/original.jpg	https://data.globe.gov/system/photos/2019/06/05/1105792/original.jpg	https://data.globe.gov/system/photos/2019/06/05/1105794/original.jpg
GV_287	50.5237	21.6938	158.3		https://www.google.com/maps/search/50.5237,21.6938	https://data.globe.gov/system/photos/2019/06/05/1105795/original.jpg	https://data.globe.gov/system/photos/2019/06/05/1105797/original.jpg	https://data.globe.gov/system/photos/2019/06/05/1105796/original.jpg	https://data.globe.gov/system/photos/2019/06/05/1105798/original.jpg	https://data.globe.gov/system/photos/2019/06/05/1105800/original.jpg
GV_288	50.5268	21.6912	145.9		https://www.google.com/maps/search/50.5268,21.6912	https://data.globe.gov/system/photos/2019/06/05/1105801/original.jpg	https://data.globe.gov/system/photos/2019/06/05/1105804/original.jpg	https://data.globe.gov/system/photos/2019/06/05/1105802/original.jpg	https://data.globe.gov/system/photos/2019/06/05/1105806/original.jpg	https://data.globe.gov/system/photos/2019/06/05/1105809/original.jpg
GV_289	50.5271	21.6879	147.7		https://www.google.com/maps/search/50.5271,21.6879	https://data.globe.gov/system/photos/2019/06/05/1105811/original.jpg	https://data.globe.gov/system/photos/2019/06/05/1105813/original.jpg	https://data.globe.gov/system/photos/2019/06/05/1105812/original.jpg	https://data.globe.gov/system/photos/2019/06/05/1105815/original.jpg	https://data.globe.gov/system/photos/2019/06/05/1105817/original.jpg
GV_290	50.5236	21.6639	166.3		https://www.google.com/maps/search/50.5236,21.6639	https://data.globe.gov/system/photos/2019/06/05/1105819/original.jpg	https://data.globe.gov/system/photos/2019/06/05/1105821/original.jpg	https://data.globe.gov/system/photos/2019/06/05/1105820/original.jpg	https://data.globe.gov/system/photos/2019/06/05/1105822/original.jpg	https://data.globe.gov/system/photos/2019/06/05/1105824/original.jpg
GV_291	50.5269	21.6791	149		https://www.google.com/maps/search/50.5269,21.6791	https://data.globe.gov/system/photos/2019/06/05/1105825/original.jpg	https://data.globe.gov/system/photos/2019/06/05/1105827/original.jpg	https://data.globe.gov/system/photos/2019/06/05/1105826/original.jpg	https://data.globe.gov/system/photos/2019/06/05/1105828/original.jpg	https://data.globe.gov/system/photos/2019/06/05/1105830/original.jpg
GV_292	50.5261	21.6771	150		https://www.google.com/maps/search/50.5261,21.6771	https://data.globe.gov/system/photos/2019/06/05/1105831/original.jpg	https://data.globe.gov/system/photos/2019/06/05/1105835/original.jpg	https://data.globe.gov/system/photos/2019/06/05/1105833/original.jpg	https://data.globe.gov/system/photos/2019/06/05/1105837/original.jpg	https://data.globe.gov/system/photos/2019/06/05/1105839/original.jpg
GV_293	50.525	21.6723	158.1		https://www.google.com/maps/search/50.525,21.6723	https://data.globe.gov/system/photos/2019/06/05/1105840/original.jpg	https://data.globe.gov/system/photos/2019/06/05/1105843/original.jpg	https://data.globe.gov/system/photos/2019/06/05/1105841/original.jpg	https://data.globe.gov/system/photos/2019/06/05/1105844/original.jpg	https://data.globe.gov/system/photos/2019/06/05/1105847/original.jpg
GV_294	50.5249	21.6691	161.3		https://www.google.com/maps/search/50.5249,21.6691	https://data.globe.gov/system/photos/2019/06/05/1105848/original.jpg	https://data.globe.gov/system/photos/2019/06/05/1105850/original.jpg	https://data.globe.gov/system/photos/2019/06/05/1105849/original.jpg	https://data.globe.gov/system/photos/2019/06/05/1105851/original.jpg	https://data.globe.gov/system/photos/2019/06/05/1105853/original.jpg
GV_295	50.5242	21.6696	162.2		https://www.google.com/maps/search/50.5242,21.6696	https://data.globe.gov/system/photos/2019/06/05/1105854/original.jpg	https://data.globe.gov/system/photos/2019/06/05/1105857/original.jpg	https://data.globe.gov/system/photos/2019/06/05/1105855/original.jpg	https://data.globe.gov/system/photos/2019/06/05/1105858/original.jpg	https://data.globe.gov/system/photos/2019/06/05/1105860/original.jpg
GV_296	50.5236	21.6662	165.3		https://www.google.com/maps/search/50.5236,21.6662	https://data.globe.gov/system/photos/2019/06/05/1105861/original.jpg	https://data.globe.gov/system/photos/2019/06/05/1105864/original.jpg	https://data.globe.gov/system/photos/2019/06/05/1105862/original.jpg	https://data.globe.gov/system/photos/2019/06/05/1105865/original.jpg	https://data.globe.gov/system/photos/2019/06/05/1105867/original.jpg
GV_297	50.5232	21.661	166.3		https://www.google.com/maps/search/50.5232,21.661	https://data.globe.gov/system/photos/2019/06/05/1105868/original.jpg	https://data.globe.gov/system/photos/2019/06/05/1105870/original.jpg	https://data.globe.gov/system/photos/2019/06/05/1105869/original.jpg	https://data.globe.gov/system/photos/2019/06/05/1105872/original.jpg	https://data.globe.gov/system/photos/2019/06/05/1105874/original.jpg
GV_298	50.523	21.6583	167		https://www.google.com/maps/search/50.523,21.6583	https://data.globe.gov/system/photos/2019/06/05/1105875/original.jpg	https://data.globe.gov/system/photos/2019/06/05/1105877/original.jpg	https://data.globe.gov/system/photos/2019/06/05/1105876/original.jpg	https://data.globe.gov/system/photos/2019/06/05/1105878/original.jpg	https://data.globe.gov/system/photos/2019/06/05/1105880/original.jpg
GV_299	50.5196	21.6524	168.5		https://www.google.com/maps/search/50.5196,21.6524	https://data.globe.gov/system/photos/2019/06/05/1105881/original.jpg	https://data.globe.gov/system/photos/2019/06/05/1105883/original.jpg	https://data.globe.gov/system/photos/2019/06/05/1105882/original.jpg	https://data.globe.gov/system/photos/2019/06/05/1105884/original.jpg	https://data.globe.gov/system/photos/2019/06/05/1105886/original.jpg
GV_300	50.5225	21.6536	167.3		https://www.google.com/maps/search/50.5225,21.6536	https://data.globe.gov/system/photos/2019/06/05/1105887/original.jpg	https://data.globe.gov/system/photos/2019/06/05/1105889/original.jpg	https://data.globe.gov/system/photos/2019/06/05/1105888/original.jpg	https://data.globe.gov/system/photos/2019/06/05/1105890/original.jpg	https://data.globe.gov/system/photos/2019/06/05/1105892/original.jpg
GV_301	50.5217	21.6523	168		https://www.google.com/maps/search/50.5217,21.6523	https://data.globe.gov/system/photos/2019/06/05/1105893/original.jpg	https://data.globe.gov/system/photos/2019/06/05/1105895/original.jpg	https://data.globe.gov/system/photos/2019/06/05/1105894/original.jpg	https://data.globe.gov/system/photos/2019/06/05/1105896/original.jpg	https://data.globe.gov/system/photos/2019/06/05/1105898/original.jpg
GV_302	50.5142	21.6556	161.2		https://www.google.com/maps/search/50.5142,21.6556	https://data.globe.gov/system/photos/2019/06/05/1105899/original.jpg	https://data.globe.gov/system/photos/2019/06/05/1105903/original.jpg	https://data.globe.gov/system/photos/2019/06/05/1105901/original.jpg	https://data.globe.gov/system/photos/2019/06/05/1105904/original.jpg	https://data.globe.gov/system/photos/2019/06/05/1105907/original.jpg
GV_303	50.5198	21.6525	168.2		https://www.google.com/maps/search/50.5198,21.6525	https://data.globe.gov/system/photos/2019/06/05/1105908/original.jpg	https://data.globe.gov/system/photos/2019/06/05/1105911/original.jpg	https://data.globe.gov/system/photos/2019/06/05/1105910/original.jpg	https://data.globe.gov/system/photos/2019/06/05/1105912/original.jpg	https://data.globe.gov/system/photos/2019/06/05/1105914/original.jpg
GV_304	50.5169	21.6495	169.1		https://www.google.com/maps/search/50.5169,21.6495	https://data.globe.gov/system/photos/2019/06/05/1105915/original.jpg	https://data.globe.gov/system/photos/2019/06/05/1105917/original.jpg	https://data.globe.gov/system/photos/2019/06/05/1105916/original.jpg	https://data.globe.gov/system/photos/2019/06/05/1105918/original.jpg	https://data.globe.gov/system/photos/2019/06/05/1105920/original.jpg
GV_305	50.5176	21.6478	169.5		https://www.google.com/maps/search/50.5176,21.6478	https://data.globe.gov/system/photos/2019/06/05/1105921/original.jpg	https://data.globe.gov/system/photos/2019/06/05/1105923/original.jpg	https://data.globe.gov/system/photos/2019/06/05/1105922/original.jpg	https://data.globe.gov/system/photos/2019/06/05/1105924/original.jpg	https://data.globe.gov/system/photos/2019/06/05/1105926/original.jpg
GV_306	50.5154	21.6518	167.4		https://www.google.com/maps/search/50.5154,21.6518	https://data.globe.gov/system/photos/2019/06/05/1105927/original.jpg	https://data.globe.gov/system/photos/2019/06/05/1105929/original.jpg	https://data.globe.gov/system/photos/2019/06/05/1105928/original.jpg	https://data.globe.gov/system/photos/2019/06/05/1105930/original.jpg	https://data.globe.gov/system/photos/2019/06/05/1105932/original.jpg
GV_307	50.5143	21.6547	162.8		https://www.google.com/maps/search/50.5143,21.6547	https://data.globe.gov/system/photos/2019/06/05/1105933/original.jpg	https://data.globe.gov/system/photos/2019/06/05/1105935/original.jpg	https://data.globe.gov/system/photos/2019/06/05/1105934/original.jpg	https://data.globe.gov/system/photos/2019/06/05/1105936/original.jpg	https://data.globe.gov/system/photos/2019/06/05/1105938/original.jpg
GV_308	50.5142	21.6557	161		https://www.google.com/maps/search/50.5142,21.6557	https://data.globe.gov/system/photos/2019/06/05/1105939/original.jpg	https://data.globe.gov/system/photos/2019/06/05/1105941/original.jpg	https://data.globe.gov/system/photos/2019/06/05/1105940/original.jpg	https://data.globe.gov/system/photos/2019/06/05/1105942/original.jpg	https://data.globe.gov/system/photos/2019/06/05/1105944/original.jpg
GV_309	50.5142	21.6552	162		https://www.google.com/maps/search/50.5142,21.6552	https://data.globe.gov/system/photos/2019/06/05/1105945/original.jpg	https://data.globe.gov/system/photos/2019/06/05/1105947/original.jpg	https://data.globe.gov/system/photos/2019/06/05/1105946/original.jpg	https://data.globe.gov/system/photos/2019/06/05/1105948/original.jpg	https://data.globe.gov/system/photos/2019/06/05/1105950/original.jpg
GV_310	50.5077	21.6549	149.3		https://www.google.com/maps/search/50.5077,21.6549	https://data.globe.gov/system/photos/2019/06/06/1105951/original.jpg	https://data.globe.gov/system/photos/2019/06/06/1105953/original.jpg	https://data.globe.gov/system/photos/2019/06/06/1105952/original.jpg	https://data.globe.gov/system/photos/2019/06/06/1105954/original.jpg	https://data.globe.gov/system/photos/2019/06/06/1105956/original.jpg
GV_311	50.5077	21.6535	150		https://www.google.com/maps/search/50.5077,21.6535	https://data.globe.gov/system/photos/2019/06/06/1105957/original.jpg	https://data.globe.gov/system/photos/2019/06/06/1105961/original.jpg	https://data.globe.gov/system/photos/2019/06/06/1105959/original.jpg	https://data.globe.gov/system/photos/2019/06/06/1105962/original.jpg	https://data.globe.gov/system/photos/2019/06/06/1105967/original.jpg
GV_312	50.5071	21.6521	149.3		https://www.google.com/maps/search/50.5071,21.6521	https://data.globe.gov/system/photos/2019/06/06/1105968/original.jpg	https://data.globe.gov/system/photos/2019/06/06/1105974/original.jpg	https://data.globe.gov/system/photos/2019/06/06/1105971/original.jpg	https://data.globe.gov/system/photos/2019/06/06/1105976/original.jpg	https://data.globe.gov/system/photos/2019/06/06/1105981/original.jpg
GV_313	50.506	21.6505	150.1		https://www.google.com/maps/search/50.506,21.6505	https://data.globe.gov/system/photos/2019/06/06/1105984/original.jpg	https://data.globe.gov/system/photos/2019/06/06/1105988/original.jpg	https://data.globe.gov/system/photos/2019/06/06/1105987/original.jpg	https://data.globe.gov/system/photos/2019/06/06/1105989/original.jpg	https://data.globe.gov/system/photos/2019/06/06/1105991/original.jpg
GV_314	50.5054	21.6504	150.1		https://www.google.com/maps/search/50.5054,21.6504	https://data.globe.gov/system/photos/2019/06/06/1105992/original.jpg	https://data.globe.gov/system/photos/2019/06/06/1105994/original.jpg	https://data.globe.gov/system/photos/2019/06/06/1105993/original.jpg	https://data.globe.gov/system/photos/2019/06/06/1105995/original.jpg	https://data.globe.gov/system/photos/2019/06/06/1105999/original.jpg
GV_315	50.5045	21.652	147		https://www.google.com/maps/search/50.5045,21.652	https://data.globe.gov/system/photos/2019/06/06/1106002/original.jpg	https://data.globe.gov/system/photos/2019/06/06/1106007/original.jpg	https://data.globe.gov/system/photos/2019/06/06/1106004/original.jpg	https://data.globe.gov/system/photos/2019/06/06/1106009/original.jpg	https://data.globe.gov/system/photos/2019/06/06/1106013/original.jpg
GV_316	50.5027	21.6505	147.1		https://www.google.com/maps/search/50.5027,21.6505	https://data.globe.gov/system/photos/2019/06/06/1106015/original.jpg	https://data.globe.gov/system/photos/2019/06/06/1106019/original.jpg	https://data.globe.gov/system/photos/2019/06/06/1106018/original.jpg	https://data.globe.gov/system/photos/2019/06/06/1106020/original.jpg	https://data.globe.gov/system/photos/2019/06/06/1106022/original.jpg
GV_317	50.5005	21.6526	146.3		https://www.google.com/maps/search/50.5005,21.6526	https://data.globe.gov/system/photos/2019/06/06/1106023/original.jpg	https://data.globe.gov/system/photos/2019/06/06/1106025/original.jpg	https://data.globe.gov/system/photos/2019/06/06/1106024/original.jpg	https://data.globe.gov/system/photos/2019/06/06/1106026/original.jpg	https://data.globe.gov/system/photos/2019/06/06/1106028/original.jpg
GV_318	50.5067	21.6461	153		https://www.google.com/maps/search/50.5067,21.6461	https://data.globe.gov/system/photos/2019/06/06/1106029/original.jpg	https://data.globe.gov/system/photos/2019/06/06/1106031/original.jpg	https://data.globe.gov/system/photos/2019/06/06/1106030/original.jpg	https://data.globe.gov/system/photos/2019/06/06/1106032/original.jpg	https://data.globe.gov/system/photos/2019/06/06/1106034/original.jpg
GV_319	50.4987	21.6505	146.1		https://www.google.com/maps/search/50.4987,21.6505	https://data.globe.gov/system/photos/2019/06/06/1106035/original.jpg	https://data.globe.gov/system/photos/2019/06/06/1106037/original.jpg	https://data.globe.gov/system/photos/2019/06/06/1106036/original.jpg	https://data.globe.gov/system/photos/2019/06/06/1106038/original.jpg	https://data.globe.gov/system/photos/2019/06/06/1106040/original.jpg
GV_320	50.4983	21.6509	146		https://www.google.com/maps/search/50.4983,21.6509	https://data.globe.gov/system/photos/2019/06/06/1106041/original.jpg	https://data.globe.gov/system/photos/2019/06/06/1106043/original.jpg	https://data.globe.gov/system/photos/2019/06/06/1106042/original.jpg	https://data.globe.gov/system/photos/2019/06/06/1106044/original.jpg	https://data.globe.gov/system/photos/2019/06/06/1106046/original.jpg
GV_321	50.4991	21.649	146.4		https://www.google.com/maps/search/50.4991,21.649	https://data.globe.gov/system/photos/2019/06/06/1106047/original.jpg	https://data.globe.gov/system/photos/2019/06/06/1106049/original.jpg	https://data.globe.gov/system/photos/2019/06/06/1106048/original.jpg	https://data.globe.gov/system/photos/2019/06/06/1106051/original.jpg	https://data.globe.gov/system/photos/2019/06/06/1106056/original.jpg
GV_322	50.4982	21.6477	147.3		https://www.google.com/maps/search/50.4982,21.6477	https://data.globe.gov/system/photos/2019/06/06/1106058/original.jpg	https://data.globe.gov/system/photos/2019/06/06/1106061/original.jpg	https://data.globe.gov/system/photos/2019/06/06/1106060/original.jpg	https://data.globe.gov/system/photos/2019/06/06/1106062/original.jpg	https://data.globe.gov/system/photos/2019/06/06/1106064/original.jpg
GV_323	50.4966	21.6453	147		https://www.google.com/maps/search/50.4966,21.6453	https://data.globe.gov/system/photos/2019/06/06/1106065/original.jpg	https://data.globe.gov/system/photos/2019/06/06/1106067/original.jpg	https://data.globe.gov/system/photos/2019/06/06/1106066/original.jpg	https://data.globe.gov/system/photos/2019/06/06/1106068/original.jpg	https://data.globe.gov/system/photos/2019/06/06/1106070/original.jpg
GV_324	50.5038	21.6469	151.7		https://www.google.com/maps/search/50.5038,21.6469	https://data.globe.gov/system/photos/2019/06/06/1106071/original.jpg	https://data.globe.gov/system/photos/2019/06/06/1106073/original.jpg	https://data.globe.gov/system/photos/2019/06/06/1106072/original.jpg	https://data.globe.gov/system/photos/2019/06/06/1106074/original.jpg	https://data.globe.gov/system/photos/2019/06/06/1106076/original.jpg
GV_325	50.5033	21.6467	150.8		https://www.google.com/maps/search/50.5033,21.6467	https://data.globe.gov/system/photos/2019/06/06/1106077/original.jpg	https://data.globe.gov/system/photos/2019/06/06/1106079/original.jpg	https://data.globe.gov/system/photos/2019/06/06/1106078/original.jpg	https://data.globe.gov/system/photos/2019/06/06/1106080/original.jpg	https://data.globe.gov/system/photos/2019/06/06/1106082/original.jpg
GV_326	50.5039	21.6471	151.7		https://www.google.com/maps/search/50.5039,21.6471	https://data.globe.gov/system/photos/2019/06/06/1106083/original.jpg	https://data.globe.gov/system/photos/2019/06/06/1106085/original.jpg	https://data.globe.gov/system/photos/2019/06/06/1106084/original.jpg	https://data.globe.gov/system/photos/2019/06/06/1106086/original.jpg	https://data.globe.gov/system/photos/2019/06/06/1106088/original.jpg
GV_327	50.5038	21.647	151.6		https://www.google.com/maps/search/50.5038,21.647	https://data.globe.gov/system/photos/2019/06/06/1106089/original.jpg	https://data.globe.gov/system/photos/2019/06/06/1106091/original.jpg	https://data.globe.gov/system/photos/2019/06/06/1106090/original.jpg	https://data.globe.gov/system/photos/2019/06/06/1106092/original.jpg	https://data.globe.gov/system/photos/2019/06/06/1106094/original.jpg
GV_328	50.5067	21.646	153.2		https://www.google.com/maps/search/50.5067,21.646	https://data.globe.gov/system/photos/2019/06/06/1106095/original.jpg	https://data.globe.gov/system/photos/2019/06/06/1106097/original.jpg	https://data.globe.gov/system/photos/2019/06/06/1106096/original.jpg	https://data.globe.gov/system/photos/2019/06/06/1106098/original.jpg	https://data.globe.gov/system/photos/2019/06/06/1106100/original.jpg
GV_329	50.5073	21.6437	155.5		https://www.google.com/maps/search/50.5073,21.6437	https://data.globe.gov/system/photos/2019/06/06/1106101/original.jpg	https://data.globe.gov/system/photos/2019/06/06/1106103/original.jpg	https://data.globe.gov/system/photos/2019/06/06/1106102/original.jpg	https://data.globe.gov/system/photos/2019/06/06/1106104/original.jpg	https://data.globe.gov/system/photos/2019/06/06/1106106/original.jpg
GV_330	50.5106	21.6369	163.4		https://www.google.com/maps/search/50.5106,21.6369	https://data.globe.gov/system/photos/2019/06/06/1106107/original.jpg	https://data.globe.gov/system/photos/2019/06/06/1106109/original.jpg	https://data.globe.gov/system/photos/2019/06/06/1106108/original.jpg	https://data.globe.gov/system/photos/2019/06/06/1106110/original.jpg	https://data.globe.gov/system/photos/2019/06/06/1106112/original.jpg
GV_331	50.51	21.639	161.4		https://www.google.com/maps/search/50.51,21.639	https://data.globe.gov/system/photos/2019/06/06/1106113/original.jpg	https://data.globe.gov/system/photos/2019/06/06/1106115/original.jpg	https://data.globe.gov/system/photos/2019/06/06/1106114/original.jpg	https://data.globe.gov/system/photos/2019/06/06/1106116/original.jpg	https://data.globe.gov/system/photos/2019/06/06/1106118/original.jpg
GV_332	50.5112	21.6388	164.5		https://www.google.com/maps/search/50.5112,21.6388	https://data.globe.gov/system/photos/2019/06/06/1106119/original.jpg	https://data.globe.gov/system/photos/2019/06/06/1106121/original.jpg	https://data.globe.gov/system/photos/2019/06/06/1106120/original.jpg	https://data.globe.gov/system/photos/2019/06/06/1106122/original.jpg	https://data.globe.gov/system/photos/2019/06/06/1106124/original.jpg
GV_333	50.5115	21.6426	163.2		https://www.google.com/maps/search/50.5115,21.6426	https://data.globe.gov/system/photos/2019/06/06/1106125/original.jpg	https://data.globe.gov/system/photos/2019/06/06/1106127/original.jpg	https://data.globe.gov/system/photos/2019/06/06/1106126/original.jpg	https://data.globe.gov/system/photos/2019/06/06/1106128/original.jpg	https://data.globe.gov/system/photos/2019/06/06/1106130/original.jpg
GV_334	50.5124	21.6475	161		https://www.google.com/maps/search/50.5124,21.6475	https://data.globe.gov/system/photos/2019/06/06/1106131/original.jpg	https://data.globe.gov/system/photos/2019/06/06/1106133/original.jpg	https://data.globe.gov/system/photos/2019/06/06/1106132/original.jpg	https://data.globe.gov/system/photos/2019/06/06/1106134/original.jpg	https://data.globe.gov/system/photos/2019/06/06/1106136/original.jpg
GV_335	50.5135	21.6496	161.6		https://www.google.com/maps/search/50.5135,21.6496	https://data.globe.gov/system/photos/2019/06/06/1106137/original.jpg	https://data.globe.gov/system/photos/2019/06/06/1106142/original.jpg	https://data.globe.gov/system/photos/2019/06/06/1106140/original.jpg	https://data.globe.gov/system/photos/2019/06/06/1106144/original.jpg	https://data.globe.gov/system/photos/2019/06/06/1106147/original.jpg
GV_336	50.5124	21.6734	147		https://www.google.com/maps/search/50.5124,21.6734	https://data.globe.gov/system/photos/2019/06/06/1106149/original.jpg	https://data.globe.gov/system/photos/2019/06/06/1106151/original.jpg	https://data.globe.gov/system/photos/2019/06/06/1106150/original.jpg	https://data.globe.gov/system/photos/2019/06/06/1106152/original.jpg	https://data.globe.gov/system/photos/2019/06/06/1106154/original.jpg
GV_337	50.5141	21.6495	162.7		https://www.google.com/maps/search/50.5141,21.6495	https://data.globe.gov/system/photos/2019/06/06/1106155/original.jpg	https://data.globe.gov/system/photos/2019/06/06/1106157/original.jpg	https://data.globe.gov/system/photos/2019/06/06/1106156/original.jpg	https://data.globe.gov/system/photos/2019/06/06/1106158/original.jpg	https://data.globe.gov/system/photos/2019/06/06/1106160/original.jpg
GV_338	50.5145	21.646	166.1		https://www.google.com/maps/search/50.5145,21.646	https://data.globe.gov/system/photos/2019/06/06/1106161/original.jpg	https://data.globe.gov/system/photos/2019/06/06/1106163/original.jpg	https://data.globe.gov/system/photos/2019/06/06/1106162/original.jpg	https://data.globe.gov/system/photos/2019/06/06/1106164/original.jpg	https://data.globe.gov/system/photos/2019/06/06/1106166/original.jpg
GV_339	50.5141	21.6466	164.8		https://www.google.com/maps/search/50.5141,21.6466	https://data.globe.gov/system/photos/2019/06/06/1106167/original.jpg	https://data.globe.gov/system/photos/2019/06/06/1106169/original.jpg	https://data.globe.gov/system/photos/2019/06/06/1106168/original.jpg	https://data.globe.gov/system/photos/2019/06/06/1106170/original.jpg	https://data.globe.gov/system/photos/2019/06/06/1106172/original.jpg
GV_340	50.5135	21.6438	167.5		https://www.google.com/maps/search/50.5135,21.6438	https://data.globe.gov/system/photos/2019/06/06/1106173/original.jpg	https://data.globe.gov/system/photos/2019/06/06/1106175/original.jpg	https://data.globe.gov/system/photos/2019/06/06/1106174/original.jpg	https://data.globe.gov/system/photos/2019/06/06/1106176/original.jpg	https://data.globe.gov/system/photos/2019/06/06/1106178/original.jpg
GV_341	50.5155	21.6413	168.2		https://www.google.com/maps/search/50.5155,21.6413	https://data.globe.gov/system/photos/2019/06/06/1106179/original.jpg	https://data.globe.gov/system/photos/2019/06/06/1106181/original.jpg	https://data.globe.gov/system/photos/2019/06/06/1106180/original.jpg	https://data.globe.gov/system/photos/2019/06/06/1106182/original.jpg	https://data.globe.gov/system/photos/2019/06/06/1106184/original.jpg
GV_342	50.5201	21.6277	163.7		https://www.google.com/maps/search/50.5201,21.6277	https://data.globe.gov/system/photos/2019/06/06/1106185/original.jpg	https://data.globe.gov/system/photos/2019/06/06/1106187/original.jpg	https://data.globe.gov/system/photos/2019/06/06/1106186/original.jpg	https://data.globe.gov/system/photos/2019/06/06/1106188/original.jpg	https://data.globe.gov/system/photos/2019/06/06/1106190/original.jpg
GV_343	50.5171	21.6369	168.9		https://www.google.com/maps/search/50.5171,21.6369	https://data.globe.gov/system/photos/2019/06/06/1106191/original.jpg	https://data.globe.gov/system/photos/2019/06/06/1106193/original.jpg	https://data.globe.gov/system/photos/2019/06/06/1106192/original.jpg	https://data.globe.gov/system/photos/2019/06/06/1106194/original.jpg	https://data.globe.gov/system/photos/2019/06/06/1106196/original.jpg
GV_344	50.5189	21.6316	167.2		https://www.google.com/maps/search/50.5189,21.6316	https://data.globe.gov/system/photos/2019/06/06/1106197/original.jpg	https://data.globe.gov/system/photos/2019/06/06/1106199/original.jpg	https://data.globe.gov/system/photos/2019/06/06/1106198/original.jpg	https://data.globe.gov/system/photos/2019/06/06/1106200/original.jpg	https://data.globe.gov/system/photos/2019/06/06/1106202/original.jpg
GV_345	50.5202	21.6277	164.1		https://www.google.com/maps/search/50.5202,21.6277	https://data.globe.gov/system/photos/2019/06/06/1106203/original.jpg	https://data.globe.gov/system/photos/2019/06/06/1106205/original.jpg	https://data.globe.gov/system/photos/2019/06/06/1106204/original.jpg	https://data.globe.gov/system/photos/2019/06/06/1106206/original.jpg	https://data.globe.gov/system/photos/2019/06/06/1106208/original.jpg
GV_346	50.5193	21.6271	159.9		https://www.google.com/maps/search/50.5193,21.6271	https://data.globe.gov/system/photos/2019/06/06/1106209/original.jpg	https://data.globe.gov/system/photos/2019/06/06/1106211/original.jpg	https://data.globe.gov/system/photos/2019/06/06/1106210/original.jpg	https://data.globe.gov/system/photos/2019/06/06/1106212/original.jpg	https://data.globe.gov/system/photos/2019/06/06/1106214/original.jpg
GV_347	50.5345	21.6232	148.9		https://www.google.com/maps/search/50.5345,21.6232	https://data.globe.gov/system/photos/2019/06/06/1106215/original.jpg	https://data.globe.gov/system/photos/2019/06/06/1106217/original.jpg	https://data.globe.gov/system/photos/2019/06/06/1106216/original.jpg	https://data.globe.gov/system/photos/2019/06/06/1106218/original.jpg	https://data.globe.gov/system/photos/2019/06/06/1106220/original.jpg
GV_348	50.5223	21.6238	166.7		https://www.google.com/maps/search/50.5223,21.6238	https://data.globe.gov/system/photos/2019/06/06/1106221/original.jpg	https://data.globe.gov/system/photos/2019/06/06/1106223/original.jpg	https://data.globe.gov/system/photos/2019/06/06/1106222/original.jpg	https://data.globe.gov/system/photos/2019/06/06/1106224/original.jpg	https://data.globe.gov/system/photos/2019/06/06/1106226/original.jpg
GV_349	50.523	21.6228	164.1		https://www.google.com/maps/search/50.523,21.6228	https://data.globe.gov/system/photos/2019/06/06/1106227/original.jpg	https://data.globe.gov/system/photos/2019/06/06/1106229/original.jpg	https://data.globe.gov/system/photos/2019/06/06/1106228/original.jpg	https://data.globe.gov/system/photos/2019/06/06/1106230/original.jpg	https://data.globe.gov/system/photos/2019/06/06/1106232/original.jpg
GV_350	50.522	21.6217	158.8		https://www.google.com/maps/search/50.522,21.6217	https://data.globe.gov/system/photos/2019/06/06/1106233/original.jpg	https://data.globe.gov/system/photos/2019/06/06/1106235/original.jpg	https://data.globe.gov/system/photos/2019/06/06/1106234/original.jpg	https://data.globe.gov/system/photos/2019/06/06/1106236/original.jpg	https://data.globe.gov/system/photos/2019/06/06/1106238/original.jpg
GV_351	50.5222	21.6226	161.7		https://www.google.com/maps/search/50.5222,21.6226	https://data.globe.gov/system/photos/2019/06/06/1106239/original.jpg	https://data.globe.gov/system/photos/2019/06/06/1106241/original.jpg	https://data.globe.gov/system/photos/2019/06/06/1106240/original.jpg	https://data.globe.gov/system/photos/2019/06/06/1106242/original.jpg	https://data.globe.gov/system/photos/2019/06/06/1106244/original.jpg
GV_352	50.5224	21.6225	161.7		https://www.google.com/maps/search/50.5224,21.6225	https://data.globe.gov/system/photos/2019/06/06/1106245/original.jpg	https://data.globe.gov/system/photos/2019/06/06/1106247/original.jpg	https://data.globe.gov/system/photos/2019/06/06/1106246/original.jpg	https://data.globe.gov/system/photos/2019/06/06/1106248/original.jpg	https://data.globe.gov/system/photos/2019/06/06/1106250/original.jpg
GV_353	50.5259	21.6229	167.6		https://www.google.com/maps/search/50.5259,21.6229	https://data.globe.gov/system/photos/2019/06/06/1106251/original.jpg	https://data.globe.gov/system/photos/2019/06/06/1106253/original.jpg	https://data.globe.gov/system/photos/2019/06/06/1106252/original.jpg	https://data.globe.gov/system/photos/2019/06/06/1106254/original.jpg	https://data.globe.gov/system/photos/2019/06/06/1106256/original.jpg

GV_354	50.5272	21.6224	165.3		https://www.google.com/maps/search/50.5272,21.6224	https://data.globe.gov/system/photos/2019/06/06/1106257/original.jpg	https://data.globe.gov/system/photos/2019/06/06/1106259/original.jpg	https://data.globe.gov/system/photos/2019/06/06/1106258/original.jpg	https://data.globe.gov/system/photos/2019/06/06/1106260/original.jpg	https://data.globe.gov/system/photos/2019/06/06/1106262/original.jpg
GV_355	50.5288	21.6255	165.1		https://www.google.com/maps/search/50.5288,21.6255	https://data.globe.gov/system/photos/2019/06/06/1106263/original.jpg	https://data.globe.gov/system/photos/2019/06/06/1106265/original.jpg	https://data.globe.gov/system/photos/2019/06/06/1106264/original.jpg	https://data.globe.gov/system/photos/2019/06/06/1106266/original.jpg	https://data.globe.gov/system/photos/2019/06/06/1106268/original.jpg
GV_356	50.5303	21.6261	160.1		https://www.google.com/maps/search/50.5303,21.6261	https://data.globe.gov/system/photos/2019/06/06/1106269/original.jpg	https://data.globe.gov/system/photos/2019/06/06/1106271/original.jpg	https://data.globe.gov/system/photos/2019/06/06/1106270/original.jpg	https://data.globe.gov/system/photos/2019/06/06/1106272/original.jpg	https://data.globe.gov/system/photos/2019/06/06/1106274/original.jpg
GV_357	50.5308	21.6236	150.9		https://www.google.com/maps/search/50.5308,21.6236	https://data.globe.gov/system/photos/2019/06/06/1106275/original.jpg	https://data.globe.gov/system/photos/2019/06/06/1106277/original.jpg	https://data.globe.gov/system/photos/2019/06/06/1106276/original.jpg	https://data.globe.gov/system/photos/2019/06/06/1106278/original.jpg	https://data.globe.gov/system/photos/2019/06/06/1106280/original.jpg
GV_358	50.5326	21.6215	146.6		https://www.google.com/maps/search/50.5326,21.6215	https://data.globe.gov/system/photos/2019/06/06/1106281/original.jpg	https://data.globe.gov/system/photos/2019/06/06/1106283/original.jpg	https://data.globe.gov/system/photos/2019/06/06/1106282/original.jpg	https://data.globe.gov/system/photos/2019/06/06/1106284/original.jpg	https://data.globe.gov/system/photos/2019/06/06/1106286/original.jpg
GV_359	50.5358	21.6228	146.4		https://www.google.com/maps/search/50.5358,21.6228	https://data.globe.gov/system/photos/2019/06/06/1106287/original.jpg	https://data.globe.gov/system/photos/2019/06/06/1106289/original.jpg	https://data.globe.gov/system/photos/2019/06/06/1106288/original.jpg	https://data.globe.gov/system/photos/2019/06/06/1106290/original.jpg	https://data.globe.gov/system/photos/2019/06/06/1106292/original.jpg
GV_360	50.5345	21.623	148.6		https://www.google.com/maps/search/50.5345,21.623	https://data.globe.gov/system/photos/2019/06/06/1106293/original.jpg	https://data.globe.gov/system/photos/2019/06/06/1106295/original.jpg	https://data.globe.gov/system/photos/2019/06/06/1106294/original.jpg	https://data.globe.gov/system/photos/2019/06/06/1106296/original.jpg	https://data.globe.gov/system/photos/2019/06/06/1106298/original.jpg
GV_361	50.5345	21.6252	148.6		https://www.google.com/maps/search/50.5345,21.6252	https://data.globe.gov/system/photos/2019/06/06/1106299/original.jpg	https://data.globe.gov/system/photos/2019/06/06/1106301/original.jpg	https://data.globe.gov/system/photos/2019/06/06/1106300/original.jpg	https://data.globe.gov/system/photos/2019/06/06/1106302/original.jpg	https://data.globe.gov/system/photos/2019/06/06/1106304/original.jpg
GV_362	50.5156	21.6667	151		https://www.google.com/maps/search/50.5156,21.6667	https://data.globe.gov/system/photos/2019/06/07/1106305/original.jpg	https://data.globe.gov/system/photos/2019/06/07/1106307/original.jpg	https://data.globe.gov/system/photos/2019/06/07/1106306/original.jpg	https://data.globe.gov/system/photos/2019/06/07/1106308/original.jpg	https://data.globe.gov/system/photos/2019/06/07/1106310/original.jpg
GV_363	50.517	21.6664	153.5		https://www.google.com/maps/search/50.517,21.6664	https://data.globe.gov/system/photos/2019/06/07/1106311/original.jpg	https://data.globe.gov/system/photos/2019/06/07/1106313/original.jpg	https://data.globe.gov/system/photos/2019/06/07/1106312/original.jpg	https://data.globe.gov/system/photos/2019/06/07/1106314/original.jpg	https://data.globe.gov/system/photos/2019/06/07/1106316/original.jpg
GV_364	50.5175	21.6676	154		https://www.google.com/maps/search/50.5175,21.6676	https://data.globe.gov/system/photos/2019/06/07/1106317/original.jpg	https://data.globe.gov/system/photos/2019/06/07/1106319/original.jpg	https://data.globe.gov/system/photos/2019/06/07/1106318/original.jpg	https://data.globe.gov/system/photos/2019/06/07/1106320/original.jpg	https://data.globe.gov/system/photos/2019/06/07/1106322/original.jpg
GV_365	50.5153	21.6683	150.4		https://www.google.com/maps/search/50.5153,21.6683	https://data.globe.gov/system/photos/2019/06/07/1106323/original.jpg	https://data.globe.gov/system/photos/2019/06/07/1106325/original.jpg	https://data.globe.gov/system/photos/2019/06/07/1106324/original.jpg	https://data.globe.gov/system/photos/2019/06/07/1106326/original.jpg	https://data.globe.gov/system/photos/2019/06/07/1106328/original.jpg
GV_366	50.5201	21.6726	151.2		https://www.google.com/maps/search/50.5201,21.6726	https://data.globe.gov/system/photos/2019/06/07/1106329/original.jpg	https://data.globe.gov/system/photos/2019/06/07/1106337/original.jpg	https://data.globe.gov/system/photos/2019/06/07/1106330/original.jpg	https://data.globe.gov/system/photos/2019/06/07/1106338/original.jpg	https://data.globe.gov/system/photos/2019/06/07/1106340/original.jpg
GV_367	50.515	21.6618	155.7		https://www.google.com/maps/search/50.515,21.6618	https://data.globe.gov/system/photos/2019/06/07/1106341/original.jpg	https://data.globe.gov/system/photos/2019/06/07/1106343/original.jpg	https://data.globe.gov/system/photos/2019/06/07/1106342/original.jpg	https://data.globe.gov/system/photos/2019/06/07/1106344/original.jpg	https://data.globe.gov/system/photos/2019/06/07/1106346/original.jpg
GV_368	50.521	21.6746	151.1		https://www.google.com/maps/search/50.521,21.6746	https://data.globe.gov/system/photos/2019/06/07/1106347/original.jpg	https://data.globe.gov/system/photos/2019/06/07/1106349/original.jpg	https://data.globe.gov/system/photos/2019/06/07/1106348/original.jpg	https://data.globe.gov/system/photos/2019/06/07/1106350/original.jpg	https://data.globe.gov/system/photos/2019/06/07/1106352/original.jpg
GV_369	50.5218	21.677	150.3		https://www.google.com/maps/search/50.5218,21.677	https://data.globe.gov/system/photos/2019/06/07/1106353/original.jpg	https://data.globe.gov/system/photos/2019/06/07/1106355/original.jpg	https://data.globe.gov/system/photos/2019/06/07/1106354/original.jpg	https://data.globe.gov/system/photos/2019/06/07/1106356/original.jpg	https://data.globe.gov/system/photos/2019/06/07/1106358/original.jpg
GV_370	50.5211	21.6724	153		https://www.google.com/maps/search/50.5211,21.6724	https://data.globe.gov/system/photos/2019/06/07/1106359/original.jpg	https://data.globe.gov/system/photos/2019/06/07/1106361/original.jpg	https://data.globe.gov/system/photos/2019/06/07/1106360/original.jpg	https://data.globe.gov/system/photos/2019/06/07/1106362/original.jpg	https://data.globe.gov/system/photos/2019/06/07/1106364/original.jpg
GV_371	50.5227	21.6708	158.6		https://www.google.com/maps/search/50.5227,21.6708	https://data.globe.gov/system/photos/2019/06/07/1106365/original.jpg	https://data.globe.gov/system/photos/2019/06/07/1106367/original.jpg	https://data.globe.gov/system/photos/2019/06/07/1106366/original.jpg	https://data.globe.gov/system/photos/2019/06/07/1106368/original.jpg	https://data.globe.gov/system/photos/2019/06/07/1106370/original.jpg
GV_372	50.5238	21.6708	160.2		https://www.google.com/maps/search/50.5238,21.6708	https://data.globe.gov/system/photos/2019/06/07/1106371/original.jpg	https://data.globe.gov/system/photos/2019/06/07/1106373/original.jpg	https://data.globe.gov/system/photos/2019/06/07/1106372/original.jpg	https://data.globe.gov/system/photos/2019/06/07/1106374/original.jpg	https://data.globe.gov/system/photos/2019/06/07/1106376/original.jpg
GV_373	50.5184	21.6683	155.2		https://www.google.com/maps/search/50.5184,21.6683	https://data.globe.gov/system/photos/2019/06/07/1106377/original.jpg	https://data.globe.gov/system/photos/2019/06/07/1106379/original.jpg	https://data.globe.gov/system/photos/2019/06/07/1106378/original.jpg	https://data.globe.gov/system/photos/2019/06/07/1106380/original.jpg	https://data.globe.gov/system/photos/2019/06/07/1106382/original.jpg
GV_374	50.5184	21.6693	153.5		https://www.google.com/maps/search/50.5184,21.6693	https://data.globe.gov/system/photos/2019/06/07/1106383/original.jpg	https://data.globe.gov/system/photos/2019/06/07/1106385/original.jpg	https://data.globe.gov/system/photos/2019/06/07/1106384/original.jpg	https://data.globe.gov/system/photos/2019/06/07/1106386/original.jpg	https://data.globe.gov/system/photos/2019/06/07/1106388/original.jpg
GV_375	50.5185	21.6681	155.9		https://www.google.com/maps/search/50.5185,21.6681	https://data.globe.gov/system/photos/2019/06/07/1106389/original.jpg	https://data.globe.gov/system/photos/2019/06/07/1106391/original.jpg	https://data.globe.gov/system/photos/2019/06/07/1106390/original.jpg	https://data.globe.gov/system/photos/2019/06/07/1106392/original.jpg	https://data.globe.gov/system/photos/2019/06/07/1106394/original.jpg
GV_376	50.5178	21.6668	155.7		https://www.google.com/maps/search/50.5178,21.6668	https://data.globe.gov/system/photos/2019/06/07/1106395/original.jpg	https://data.globe.gov/system/photos/2019/06/07/1106397/original.jpg	https://data.globe.gov/system/photos/2019/06/07/1106396/original.jpg	https://data.globe.gov/system/photos/2019/06/07/1106398/original.jpg	https://data.globe.gov/system/photos/2019/06/07/1106400/original.jpg
GV_377	50.5182	21.6682	155		https://www.google.com/maps/search/50.5182,21.6682	https://data.globe.gov/system/photos/2019/06/07/1106401/original.jpg	https://data.globe.gov/system/photos/2019/06/07/1106403/original.jpg	https://data.globe.gov/system/photos/2019/06/07/1106402/original.jpg	https://data.globe.gov/system/photos/2019/06/07/1106404/original.jpg	https://data.globe.gov/system/photos/2019/06/07/1106406/original.jpg
GV_378	50.5148	21.6614	155.8		https://www.google.com/maps/search/50.5148,21.6614	https://data.globe.gov/system/photos/2019/06/07/1106407/original.jpg	https://data.globe.gov/system/photos/2019/06/07/1106409/original.jpg	https://data.globe.gov/system/photos/2019/06/07/1106408/original.jpg	https://data.globe.gov/system/photos/2019/06/07/1106410/original.jpg	https://data.globe.gov/system/photos/2019/06/07/1106412/original.jpg
GV_379	50.5142	21.6596	157		https://www.google.com/maps/search/50.5142,21.6596	https://data.globe.gov/system/photos/2019/06/07/1106413/original.jpg	https://data.globe.gov/system/photos/2019/06/07/1106415/original.jpg	https://data.globe.gov/system/photos/2019/06/07/1106414/original.jpg	https://data.globe.gov/system/photos/2019/06/07/1106416/original.jpg	https://data.globe.gov/system/photos/2019/06/07/1106418/original.jpg
GV_380	50.5148	21.6639	152.5		https://www.google.com/maps/search/50.5148,21.6639	https://data.globe.gov/system/photos/2019/06/01/1106419/original.jpg	https://data.globe.gov/system/photos/2019/06/01/1106421/original.jpg	https://data.globe.gov/system/photos/2019/06/01/1106420/original.jpg	https://data.globe.gov/system/photos/2019/06/01/1106422/original.jpg	https://data.globe.gov/system/photos/2019/06/01/1106424/original.jpg
GV_381	50.4858	21.6821	152		https://www.google.com/maps/search/50.4858,21.6821	https://data.globe.gov/system/photos/2019/06/01/1106425/original.jpg	https://data.globe.gov/system/photos/2019/06/01/1106427/original.jpg	https://data.globe.gov/system/photos/2019/06/01/1106426/original.jpg	https://data.globe.gov/system/photos/2019/06/01/1106428/original.jpg	https://data.globe.gov/system/photos/2019/06/01/1106430/original.jpg
GV_382	50.4867	21.6821	148.8		https://www.google.com/maps/search/50.4867,21.6821	https://data.globe.gov/system/photos/2019/06/05/1106431/original.jpg	https://data.globe.gov/system/photos/2019/06/05/1106434/original.jpg	https://data.globe.gov/system/photos/2019/06/05/1106433/original.jpg	https://data.globe.gov/system/photos/2019/06/05/1106436/original.jpg	https://data.globe.gov/system/photos/2019/06/05/1106439/original.jpg
GV_383	22.6785	120.3004	10.1		https://www.google.com/maps/search/22.6785,120.3004	https://data.globe.gov/system/photos/2019/06/19/1106496/original.jpg	https://data.globe.gov/system/photos/2019/06/19/1106498/original.jpg	https://data.globe.gov/system/photos/2019/06/19/1106497/original.jpg	https://data.globe.gov/system/photos/2019/06/19/1106499/original.jpg	https://data.globe.gov/system/photos/2019/06/19/1106501/original.jpg
GV_384	13.7846	100.5329	6.2		https://www.google.com/maps/search/13.7846,100.5329	https://data.globe.gov/system/photos/2019/06/19/1106684/original.jpg	https://data.globe.gov/system/photos/2019/06/19/1106686/original.jpg	https://data.globe.gov/system/photos/2019/06/19/1106685/original.jpg	https://data.globe.gov/system/photos/2019/06/19/1106687/original.jpg	https://data.globe.gov/system/photos/2019/06/19/1106689/original.jpg
GV_385	57.2179	-2.1844	40.4		https://www.google.com/maps/search/57.2179,-2.1844	https://data.globe.gov/system/photos/2019/06/19/1106746/original.jpg	https://data.globe.gov/system/photos/2019/06/19/1106748/original.jpg	https://data.globe.gov/system/photos/2019/06/19/1106747/original.jpg	https://data.globe.gov/system/photos/2019/06/19/1106749/original.jpg	https://data.globe.gov/system/photos/2019/06/19/1106751/original.jpg
GV_386	42.0857	-83.2504	179.3		https://www.google.com/maps/search/42.0857,-83.2504	https://data.globe.gov/system/photos/2019/06/19/1106777/original.jpg	https://data.globe.gov/system/photos/2019/06/19/1106779/original.jpg	https://data.globe.gov/system/photos/2019/06/19/1106778/original.jpg	https://data.globe.gov/system/photos/2019/06/19/1106780/original.jpg	https://data.globe.gov/system/photos/2019/06/19/1106782/original.jpg
GV_387	51.837	4.9968	1.1		https://www.google.com/maps/search/51.837,4.9968	https://data.globe.gov/system/photos/2019/06/19/1106827/original.jpg	https://data.globe.gov/system/photos/2019/06/19/1106829/original.jpg	https://data.globe.gov/system/photos/2019/06/19/1106828/original.jpg	https://data.globe.gov/system/photos/2019/06/19/1106830/original.jpg	https://data.globe.gov/system/photos/2019/06/19/1106832/original.jpg
GV_388	39.2529	-77.1974	183.3		https://www.google.com/maps/search/39.2529,-77.1974	https://data.globe.gov/system/photos/2019/06/19/1106877/original.jpg	https://data.globe.gov/system/photos/2019/06/19/1106879/original.jpg	https://data.globe.gov/system/photos/2019/06/19/1106878/original.jpg	https://data.globe.gov/system/photos/2019/06/19/1106880/original.jpg	https://data.globe.gov/system/photos/2019/06/19/1106882/original.jpg
GV_389	40.4115	-111.5037	1688		https://www.google.com/maps/search/40.4115,-111.5037	https://data.globe.gov/system/photos/2019/06/18/1106924/original.jpg	https://data.globe.gov/system/photos/2019/06/18/1106926/original.jpg	https://data.globe.gov/system/photos/2019/06/18/1106925/original.jpg	https://data.globe.gov/system/photos/2019/06/18/1106927/original.jpg	https://data.globe.gov/system/photos/2019/06/18/1106929/original.jpg
GV_390	37.226	-112.8878	1696.7		https://www.google.com/maps/search/37.226,-112.8878	https://data.globe.gov/system/photos/2019/06/18/1106930/original.jpg	https://data.globe.gov/system/photos/2019/06/18/1106932/original.jpg	https://data.globe.gov/system/photos/2019/06/18/1106931/original.jpg	https://data.globe.gov/system/photos/2019/06/18/1106933/original.jpg	https://data.globe.gov/system/photos/2019/06/18/1106935/original.jpg
GV_391	64.8659	-147.8595	154		https://www.google.com/maps/search/64.8659,-147.8595	https://data.globe.gov/system/photos/2019/06/12/1107044/original.jpg	https://data.globe.gov/system/photos/2019/06/12/1107046/original.jpg	https://data.globe.gov/system/photos/2019/06/12/1107045/original.jpg	https://data.globe.gov/system/photos/2019/06/12/1107047/original.jpg	https://data.globe.gov/system/photos/2019/06/12/1107049/original.jpg
GV_392	64.8658	-147.8576	157.2		https://www.google.com/maps/search/64.8658,-147.8576	https://data.globe.gov/system/photos/2019/06/12/1107050/original.jpg	https://data.globe.gov/system/photos/2019/06/12/1107052/original.jpg	https://data.globe.gov/system/photos/2019/06/12/1107051/original.jpg	https://data.globe.gov/system/photos/2019/06/12/1107053/original.jpg	https://data.globe.gov/system/photos/2019/06/12/1107055/original.jpg
GV_393	64.8704	-147.8228	151		https://www.google.com/maps/search/64.8704,-147.8228	https://data.globe.gov/system/photos/2019/06/14/1107056/original.jpg	https://data.globe.gov/system/photos/2019/06/14/1107058/original.jpg	https://data.globe.gov/system/photos/2019/06/14/1107057/original.jpg	https://data.globe.gov/system/photos/2019/06/14/1107059/original.jpg	https://data.globe.gov/system/photos/2019/06/14/1107061/original.jpg
GV_394	44.7454	-111.2636	2008.1		https://www.google.com/maps/search/44.7454,-111.2636	https://data.globe.gov/system/photos/2019/06/16/1107466/original.jpg	https://data.globe.gov/system/photos/2019/06/16/1107468/original.jpg	https://data.globe.gov/system/photos/2019/06/16/1107467/original.jpg	https://data.globe.gov/system/photos/2019/06/16/1107469/original.jpg	https://data.globe.gov/system/photos/2019/06/16/1107471/original.jpg
GV_395	44.7727	-111.2673	1995.7		https://www.google.com/maps/search/44.7727,-111.2673	https://data.globe.gov/system/photos/2019/06/16/1107472/original.jpg	https://data.globe.gov/system/photos/2019/06/16/1107474/original.jpg	https://data.globe.gov/system/photos/2019/06/16/1107473/original.jpg	https://data.globe.gov/system/photos/2019/06/16/1107475/original.jpg	https://data.globe.gov/system/photos/2019/06/16/1107477/original.jpg
GV_396	22.6807	120.2972	11.7		https://www.google.com/maps/search/22.6807,120.2972	https://data.globe.gov/system/photos/2019/06/19/1107616/original.jpg	https://data.globe.gov/system/photos/2019/06/19/1107618/original.jpg	https://data.globe.gov/system/photos/2019/06/19/1107617/original.jpg	https://data.globe.gov/system/photos/2019/06/19/1107619/original.jpg	https://data.globe.gov/system/photos/2019/06/19/1107621/original.jpg
GV_397	22.6806	120.2978	13.2		https://www.google.com/maps/search/22.6806,120.2978	https://data.globe.gov/system/photos/2019/06/19/1107628/original.jpg	https://data.globe.gov/system/photos/2019/06/19/1107630/original.jpg	https://data.globe.gov/system/photos/2019/06/19/1107629/original.jpg	https://data.globe.gov/system/photos/2019/06/19/1107631/original.jpg	https://data.globe.gov/system/photos/2019/06/19/1107633/original.jpg
GV_398	22.6786	120.2996	11.1		https://www.google.com/maps/search/22.6786,120.2996	https://data.globe.gov/system/photos/2019/06/19/1107634/original.jpg	https://data.globe.gov/system/photos/2019/06/19/1107636/original.jpg	https://data.globe.gov/system/photos/2019/06/19/1107635/original.jpg	https://data.globe.gov/system/photos/2019/06/19/1107637/original.jpg	https://data.globe.gov/system/photos/2019/06/19/1107639/original.jpg
GV_399	-35.5713	138.9251	2.4		https://www.google.com/maps/search/-35.5713,138.9251	https://data.globe.gov/system/photos/2019/06/20/1107880/original.jpg	https://data.globe.gov/system/photos/2019/06/20/1107882/original.jpg	https://data.globe.gov/system/photos/2019/06/20/1107881/original.jpg	https://data.globe.gov/system/photos/2019/06/20/1107883/original.jpg	https://data.globe.gov/system/photos/2019/06/20/1107885/original.jpg
GV_400	15.2773	104.8237	131.3		https://www.google.com/maps/search/15.2773,104.8237	https://data.globe.gov/system/photos/2019/06/20/1107944/original.jpg	https://data.globe.gov/system/photos/2019/06/20/1107952/original.jpg	https://data.globe.gov/system/photos/2019/06/20/1107949/original.jpg	https://data.globe.gov/system/photos/2019/06/20/1107954/original.jpg	https://data.globe.gov/system/photos/2019/06/20/1107958/original.jpg
GV_401	15.2773	1	271.4		https://www.google.com/maps/search/15.2773,1	https://data.globe.gov/system/photos/2019/06/20/1107945/original.jpg	https://data.globe.gov/system/photos/2019/06/20/1107951/original.jpg	https://data.globe.gov/system/photos/2019/06/20/1107948/original.jpg	https://data.globe.gov/system/photos/2019/06/20/1107953/original.jpg	https://data.globe.gov/system/photos/2019/06/20/1107956/original.jpg
GV_402	15.2774	104.8235	131.5		https://www.google.com/maps/search/15.2774,104.8235	https://data.globe.gov/system/photos/2019/06/20/1107959/original.jpg	https://data.globe.gov/system/photos/2019/06/20/1107962/original.jpg	https://data.globe.gov/system/photos/2019/06/20/1107960/original.jpg	https://data.globe.gov/system/photos/2019/06/20/1107964/original.jpg	https://data.globe.gov/system/photos/2019/06/20/1107966/original.jpg
GV_403	15.2774	10	453.7		https://www.google.com/maps/search/15.2774,10	https://data.globe.gov/system/photos/2019/06/20/1107967/original.jpg	https://data.globe.gov/system/photos/2019/06/20/1107969/original.jpg	https://data.globe.gov/system/photos/2019/06/20/1107968/original.jpg	https://data.globe.gov/system/photos/2019/06/20/1107970/original.jpg	https://data.globe.gov/system/photos/2019/06/20/1107972/original.jpg
GV_404	-35.5654	138.905	3.6		https://www.google.com/maps/search/-35.5654,138.905	https://data.globe.gov/system/photos/2019/06/20/1107996/original.jpg	https://data.globe.gov/system/photos/2019/06/20/1107998/original.jpg	https://data.globe.gov/system/photos/2019/06/20/1107997/original.jpg	https://data.globe.gov/system/photos/2019/06/20/1107999/original.jpg	https://data.globe.gov/system/photos/2019/06/20/1108001/original.jpg
GV_405	13.7844	100.5338	7		https://www.google.com/maps/search/13.7844,100.5338	https://data.globe.gov/system/photos/2019/06/20/1108005/original.jpg	https://data.globe.gov/system/photos/2019/06/20/1108007/original.jpg	https://data.globe.gov/system/photos/2019/06/20/1108006/original.jpg	https://data.globe.gov/system/photos/2019/06/20/1108008/original.jpg	https://data.globe.gov/system/photos/2019/06/20/1108010/original.jpg
GV_406	13.7845	100.5326	7.4		https://www.google.com/maps/search/13.7845,100.5326	https://data.globe.gov/system/photos/2019/06/20/1108011/original.jpg	https://data.globe.gov/system/photos/2019/06/20/1108013/original.jpg	https://data.globe.gov/system/photos/2019/06/20/1108012/original.jpg	https://data.globe.gov/system/photos/2019/06/20/1108014/original.jpg	https://data.globe.gov/system/photos/2019/06/20/1108016/original.jpg
GV_407	25.0126	121.2141	82.7		https://www.google.com/maps/search/25.0126,121.2141	https://data.globe.gov/system/photos/2019/06/20/1108017/original.jpg	https://data.globe.gov/system/photos/2019/06/20/1108019/original.jpg	https://data.globe.gov/system/photos/2019/06/20/1108018/original.jpg	https://data.globe.gov/system/photos/2019/06/20/1108020/original.jpg	https://data.globe.gov/system/photos/2019/06/20/1108022/original.jpg
GV_408	13.7911	100.544	10.8		https://www.google.com/maps/search/13.7911,100.544	https://data.globe.gov/system/photos/2019/06/20/1108085/original.jpg	https://data.globe.gov/system/photos/2019/06/20/1108087/original.jpg	https://data.globe.gov/system/photos/2019/06/20/1108086/original.jpg	https://data.globe.gov/system/photos/2019/06/20/1108088/original.jpg	https://data.globe.gov/system/photos/2019/06/20/1108090/original.jpg
GV_409	13.7925	100.5309	8.9		https://www.google.com/maps/search/13.7925,100.5309	https://data.globe.gov/system/photos/2019/06/11/1108119/original.jpg	https://data.globe.gov/system/photos/2019/06/11/1108121/original.jpg	https://data.globe.gov/system/photos/2019/06/11/1108120/original.jpg	https://data.globe.gov/system/photos/2019/06/11/1108122/original.jpg	https://data.globe.gov/system/photos/2019/06/11/1108124/original.jpg
GV_410	13.8039	100.5876	3.8		https://www.google.com/maps/search/13.8039,100.5876	https://data.globe.gov/system/photos/2019/06/20/1108131/original.jpg	https://data.globe.gov/system/photos/2019/06/20/1108133/original.jpg	https://data.globe.gov/system/photos/2019/06/20/1108132/original.jpg	https://data.globe.gov/system/photos/2019/06/20/1108134/original.jpg	https://data.globe.gov/system/photos/2019/06/20/1108136/original.jpg
GV_411	13.8039	100.5876	3.8		https://www.google.com/maps/search/13.8039,100.5876	https://data.globe.gov/system/photos/2019/06/20/1108138/original.jpg	https://data.globe.gov/system/photos/2019/06/20/1108140/original.jpg	https://data.globe.gov/system/photos/2019/06/20/1108139/original.jpg	https://data.globe.gov/system/photos/2019/06/20/1108141/original.jpg	https://data.globe.gov/system/photos/2019/06/20/1108144/original.jpg
GV_412	13.8039	100.5876	3.8		https://www.google.com/maps/search/13.8039,100.5876	https://data.globe.gov/system/photos/2019/06/20/1108185/original.jpg	https://data.globe.gov/system/photos/2019/06/20/1108187/original.jpg	https://data.globe.gov/system/photos/2019/06/20/1108186/original.jpg	https://data.globe.gov/system/photos/2019/06/20/1108188/original.jpg	https://data.globe.gov/system/photos/2019/06/20/1108190/original.jpg
GV_413	52.9048	-2.4771	91.7		https://www.google.com/maps/search/52.9048,-2.4771	https://data.globe.gov/system/photos/2019/06/20/1108271/original.jpg	https://data.globe.gov/system/photos/2019/06/20/1108273/original.jpg	https://data.globe.gov/system/photos/2019/06/20/1108272/original.jpg	https://data.globe.gov/system/photos/2019/06/20/1108274/original.jpg	https://data.globe.gov/system/photos/2019/06/20/1108277/original.jpg
GV_414	40.0085	-75.0419	3		https://www.google.com/maps/search/40.0085,-75.0419	https://data.globe.gov/system/photos/2019/06/20/1108278/original.jpg	https://data.globe.gov/system/photos/2019/06/20/1108280/original.jpg	https://data.globe.gov/system/photos/2019/06/20/1108279/original.jpg	https://data.globe.gov/system/photos/2019/06/20/1108281/original.jpg	https://data.globe.gov/system/photos/2019/06/20/1108283/original.jpg
GV_415	41.2609	-111.9425	1408.5		https://www.google.com/maps/search/41.2609,-111.9425	https://data.globe.gov/system/photos/2019/06/20/1108302/original.jpg	https://data.globe.gov/system/photos/2019/06/20/1108304/original.jpg	https://data.globe.gov/system/photos/2019/06/20/1108303/original.jpg	https://data.globe.gov/system/photos/2019/06/20/1108305/original.jpg	https://data.globe.gov/system/photos/2019/06/20/1108307/original.jpg
GV_416	-39.9513	-71.0682	779.2		https://www.google.com/maps/search/-39.9513,-71.0682	https://data.globe.gov/system/photos/2019/06/17/1108584/original.jpg	https://data.globe.gov/system/photos/2019/06/17/1108586/original.jpg	https://data.globe.gov/system/photos/2019/06/17/1108585/original.jpg	https://data.globe.gov/system/photos/2019/06/17/1108587/original.jpg	https://data.globe.gov/system/photos/2019/06/17/1108589/original.jpg
GV_417	42.2181	-83.2503	185.6		https://www.google.com/maps/search/42.2181,-83.2503	https://data.globe.gov/system/photos/2019/06/20/1108599/original.jpg	https://data.globe.gov/system/photos/2019/06/20/1108601/original.jpg	https://data.globe.gov/system/photos/2019/06/20/1108600/original.jpg	https://data.globe.gov/system/photos/2019/06/20/1108602/original.jpg	https://data.globe.gov/system/photos/2019/06/20/1108604/original.jpg
GV_418	42.216	-83.25	185.4		https://www.google.com/maps/search/42.216,-83.25	https://data.globe.gov/system/photos/2019/06/20/1108626/original.jpg	https://data.globe.gov/system/photos/2019/06/20/1108628/original.jpg	https://data.globe.gov/system/photos/2019/06/20/1108627/original.jpg	https://data.globe.gov/system/photos/2019/06/20/1108629/original.jpg	https://data.globe.gov/system/photos/2019/06/20/1108631/original.jpg
GV_419	64.859	-147.8502	188		https://www.google.com/maps/search/64.859,-147.8502	https://data.globe.gov/system/photos/2019/06/11/1108643/original.jpg	https://data.globe.gov/system/photos/2019/06/11/1108645/original.jpg	https://data.globe.gov/system/photos/2019/06/11/1108644/original.jpg	https://data.globe.gov/system/photos/2019/06/11/1108646/original.jpg	https://data.globe.gov/system/photos/2019/06/11/1108648/original.jpg
GV_420	25.0599	121.5165	5		https://www.google.com/maps/search/25.0599,121.5165	https://data.globe.gov/system/photos/2019/06/21/1108761/original.jpg	https://data.globe.gov/system/photos/2019/06/21/1108763/original.jpg	https://data.globe.gov/system/photos/2019/06/21/1108762/original.jpg	https://data.globe.gov/system/photos/2019/06/21/1108764/original.jpg	https://data.globe.gov/system/photos/2019/06/21/1108766/original.jpg
GV_421	25.0578	121.5227	12.8		https://www.google.com/maps/search/25.0578,121.5227	https://data.globe.gov/system/photos/2019/06/21/1108775/original.jpg	https://data.globe.gov/system/photos/2019/06/21/1108777/original.jpg	https://data.globe.gov/system/photos/2019/06/21/1108776/original.jpg	https://data.globe.gov/system/photos/2019/06/21/1108778/original.jpg	https://data.globe.gov/system/photos/2019/06/21/1108780/original.jpg
GV_422	25.0083	121.5358	16.4		https://www.google.com/maps/search/25.0083,121.5358	https://data.globe.gov/system/photos/2019/06/21/1108897/original.jpg	https://data.globe.gov/system/photos/2019/06/21/1108901/original.jpg	https://data.globe.gov/system/photos/2019/06/21/1108899/original.jpg	https://data.globe.gov/system/photos/2019/06/21/1108903/original.jpg	https://data.globe.gov/system/photos/2019/06/21/1108905/original.jpg
GV_423	15.2771	104.8237	131.6		https://www.google.com/maps/search/15.2771,104.8237	https://data.globe.gov/system/photos/2019/06/21/1108933/original.jpg	https://data.globe.gov/system/photos/2019/06/21/1108935/original.jpg	https://data.globe.gov/system/photos/2019/06/21/1108934/original.jpg	https://data.globe.gov/system/photos/2019/06/21/1108936/original.jpg	https://data.globe.gov/system/photos/2019/06/21/1108938/original.jpg
GV_424	-31.1553	116.2565	333.6		https://www.google.com/maps/search/-31.1553,116.2565	https://data.globe.gov/system/photos/2019/06/21/1108990/original.jpg	https://data.globe.gov/system/photos/2019/06/21/1108992/original.jpg	https://data.globe.gov/system/photos/2019/06/21/1108991/original.jpg	https://data.globe.gov/system/photos/2019/06/21/1108993/original.jpg	https://data.globe.gov/system/photos/2019/06/21/1108995/original.jpg
GV_425	38.9934	-76.84	62.8		https://www.google.com/maps/search/38.9934,-76.84	https://data.globe.gov/system/photos/2019/06/21/1109348/original.jpg	https://data.globe.gov/system/photos/2019/06/21/1109350/original.jpg	https://data.globe.gov/system/photos/2019/06/21/1109349/original.jpg	https://data.globe.gov/system/photos/2019/06/21/1109351/original.jpg	https://data.globe.gov/system/photos/2019/06/21/1109353/original.jpg
GV_426	42.0778	-83.2538	178.9		https://www.google.com/maps/search/42.0778,-83.2538	https://data.globe.gov/system/photos/2019/06/19/1109398/original.jpg	https://data.globe.gov/system/photos/2019/06/19/1109400/original.jpg	https://data.globe.gov/system/photos/2019/06/19/1109399/original.jpg	https://data.globe.gov/system/photos/2019/06/19/1109401/original.jpg	https://data.globe.gov/system/photos/2019/06/19/1109403/original.jpg
GV_427	42.0778	-83.2537	178.9		https://www.google.com/maps/search/42.0778,-83.2537	https://data.globe.gov/system/photos/2019/06/20/1109416/original.jpg	https://data.globe.gov/system/photos/2019/06/20/1109418/original.jpg	https://data.globe.gov/system/photos/2019/06/20/1109417/original.jpg	https://data.globe.gov/system/photos/2019/06/20/1109420/original.jpg	https://data.globe.gov/system/photos/2019/06/20/1109422/original.jpg
GV_428	42.0779	-83.254	179.4		https://www.google.com/maps/search/42.0779,-83.254	https://data.globe.gov/system/photos/2019/06/21/1109429/original.jpg	https://data.globe.gov/system/photos/2019/06/21/1109438/original.jpg	https://data.globe.gov/system/photos/2019/06/21/1109436/original.jpg	https://data.globe.gov/system/photos/2019/06/21/1109439/original.jpg	https://data.globe.gov/system/photos/2019/06/21/1109447/original.jpg
GV_429	38.9934	-76.8401	62.9		https://www.google.com/maps/search/38.9934,-76.8401	https://data.globe.gov/system/photos/2019/06/21/1109364/original.jpg	https://data.globe.gov/system/photos/2019/06/21/1109366/original.jpg	https://data.globe.gov/system/photos/2019/06/21/1109365/original.jpg	https://data.globe.gov/system/photos/2019/06/21/1109368/original.jpg	https://data.globe.gov/system/photos/2019/06/21/1109371/original.jpg
GV_430	42.0778	-83.2536	178.8		https://www.google.com/maps/search/42.0778,-83.2536	https://data.globe.gov/system/photos/2019/06/21/1109454/original.jpg	https://data.globe.gov/system/photos/2019/06/21/1109456/original.jpg	https://data.globe.gov/system/photos/2019/06/21/1109455/original.jpg	https://data.globe.gov/system/photos/2019/06/21/1109457/original.jpg	https://data.globe.gov/system/photos/2019/06/21/1109459/original.jpg
GV_431	-25.4982	-56.4575	139.3		https://www.google.com/maps/search/-25.4982,-56.4575	https://data.globe.gov/system/photos/2019/06/21/1109497/original.jpg	https://data.globe.gov/system/photos/2019/06/21/1109499/original.jpg	https://data.globe.gov/system/photos/2019/06/21/1109498/original.jpg	https://data.globe.gov/system/photos/2019/06/21/1109500/original.jpg	https://data.globe.gov/system/photos/2019/06/21/1109502/original.jpg
GV_432	61.716	-150.0181	82.7		https://www.google.com/maps/search/61.716,-150.0181	https://data.globe.gov/system/photos/2019/06/21/1109756/original.jpg	https://data.globe.gov/system/photos/2019/06/21/1109762/original.jpg	https://data.globe.gov/system/photos/2019/06/21/1109758/original.jpg	https://data.globe.gov/system/photos/2019/06/21/1109764/original.jpg	https://data.globe.gov/system/photos/2019/06/21/1109766/original.jpg
GV_433	46.5299	10.1292	1845.9		https://www.google.com/maps/search/46.5299,10.1292	https://data.globe.gov/system/photos/2019/06/14/1109767/original.jpg	https://data.globe.gov/system/photos/2019/06/14/1109769/original.jpg	https://data.globe.gov/system/photos/2019/06/14/1109768/original.jpg	https://data.globe.gov/system/photos/2019/06/14/1109770/original.jpg	https://data.globe.gov/system/photos/2019/06/14/1109772/original.jpg
GV_434	46.2929	10.2707	645.9		https://www.google.com/maps/search/46.2929,10.2707	https://data.globe.gov/system/photos/2019/06/15/1109773/original.jpg	https://data.globe.gov/system/photos/2019/06/15/1109775/original.jpg	https://data.globe.gov/system/photos/2019/06/15/1109774/original.jpg	https://data.globe.gov/system/photos/2019/06/15/1109776/original.jpg	https://data.globe.gov/system/photos/2019/06/15/1109780/original.jpg
GV_435	54.3002	18.0651	170.7		https://www.google.com/maps/search/54.3002,18.0651	https://data.globe.gov/system/photos/2019/06/21/1109781/original.jpg	https://data.globe.gov/system/photos/2019/06/21/1109784/original.jpg	https://data.globe.gov/system/photos/2019/06/21/1109782/original.jpg	https://data.globe.gov/system/photos/2019/06/21/1109785/original.jpg	https://data.globe.gov/system/photos/2019/06/21/1109823/original.jpg
GV_436	61.7165	-150.0162	83.8		https://www.google.com/maps/search/61.7165,-150.0162	https://data.globe.gov/system/photos/2019/06/21/1109777/original.jpg	https://data.globe.gov/system/photos/2019/06/21/1109783/original.jpg	https://data.globe.gov/system/photos/2019/06/21/1109779/original.jpg	https://data.globe.gov/system/photos/2019/06/21/1109787/original.jpg	https://data.globe.gov/system/photos/2019/06/21/1109789/original.jpg
GV_437	61.7163	-150.0213	83.8		https://www.google.com/maps/search/61.7163,-150.0213	https://data.globe.gov/system/photos/2019/06/21/1109851/original.jpg	https://data.globe.gov/system/photos/2019/06/21/1109853/original.jpg	https://data.globe.gov/system/photos/2019/06/21/1109852/original.jpg	https://data.globe.gov/system/photos/2019/06/21/1109854/original.jpg	https://data.globe.gov/system/photos/2019/06/21/1109856/original.jpg
GV_438	44.6245	-123.0829	68.4		https://www.google.com/maps/search/44.6245,-123.0829	https://data.globe.gov/system/photos/2019/06/21/1109867/original.jpg	https://data.globe.gov/system/photos/2019/06/21/1109869/original.jpg	https://data.globe.gov/system/photos/2019/06/21/1109868/original.jpg	https://data.globe.gov/system/photos/2019/06/21/1109870/original.jpg	https://data.globe.gov/system/photos/2019/06/21/1109872/original.jpg
GV_439	25.0216	121.3058	76.2		https://www.google.com/maps/search/25.0216,121.3058	https://data.globe.gov/system/photos/2019/06/22/1110011/original.jpg	https://data.globe.gov/system/photos/2019/06/22/1110013/original.jpg	https://data.globe.gov/system/photos/2019/06/22/1110012/original.jpg	https://data.globe.gov/system/photos/2019/06/22/1110014/original.jpg	https://data.globe.gov/system/photos/2019/06/22/1110016/original.jpg
GV_440	45.0216	-92.8689	277.2		https://www.google.com/maps/search/45.0216,-92.8689	https://data.globe.gov/system/photos/2019/06/21/1110104/original.jpg	https://data.globe.gov/system/photos/2019/06/21/1110106/original.jpg	https://data.globe.gov/system/photos/2019/06/21/1110105/original.jpg	https://data.globe.gov/system/photos/2019/06/21/1110107/original.jpg	https://data.globe.gov/system/photos/2019/06/21/1110109/original.jpg
GV_441	25.0265	121.3019	73.3		https://www.google.com/maps/search/25.0265,121.3019	https://data.globe.gov/system/photos/2019/06/22/1110165/original.jpg	https://data.globe.gov/system/photos/2019/06/22/1110167/original.jpg	https://data.globe.gov/system/photos/2019/06/22/1110166/original.jpg	https://data.globe.gov/system/photos/2019/06/22/1110168/original.jpg	https://data.globe.gov/system/photos/2019/06/22/1110170/original.jpg
GV_442	25.0299	121.3013	74.9		https://www.google.com/maps/search/25.0299,121.3013	https://data.globe.gov/system/photos/2019/06/22/1110171/original.jpg	https://data.globe.gov/system/photos/2019/06/22/1110173/original.jpg	https://data.globe.gov/system/photos/2019/06/22/1110172/original.jpg	https://data.globe.gov/system/photos/2019/06/22/1110174/original.jpg	https://data.globe.gov/system/photos/2019/06/22/1110176/original.jpg
GV_443	25.0628	121.5521	6.7		https://www.google.com/maps/search/25.0628,121.5521	https://data.globe.gov/system/photos/2019/06/22/1110177/original.jpg	https://data.globe.gov/system/photos/2019/06/22/1110179/original.jpg	https://data.globe.gov/system/photos/2019/06/22/1110178/original.jpg	https://data.globe.gov/system/photos/2019/06/22/1110180/original.jpg	https://data.globe.gov/system/photos/2019/06/22/1110182/original.jpg

GV_444	24.4354	118.3756	7		https://www.google.com/maps/search/24.4354,118.3756	https://data.globe.gov/system/photos/2019/06/22/1110267/original.jpg	https://data.globe.gov/system/photos/2019/06/22/1110269/original.jpg	https://data.globe.gov/system/photos/2019/06/22/1110268/original.jpg	https://data.globe.gov/system/photos/2019/06/22/1110270/original.jpg	https://data.globe.gov/system/photos/2019/06/22/1110272/original.jpg
GV_445	24.4355	118.3761	6.3		https://www.google.com/maps/search/24.4355,118.3761	https://data.globe.gov/system/photos/2019/06/22/1110285/original.jpg	https://data.globe.gov/system/photos/2019/06/22/1110287/original.jpg	https://data.globe.gov/system/photos/2019/06/22/1110286/original.jpg	https://data.globe.gov/system/photos/2019/06/22/1110288/original.jpg	https://data.globe.gov/system/photos/2019/06/22/1110290/original.jpg
GV_446	47.2973	-101.0915	508.8		https://www.google.com/maps/search/47.2973,-101.0915	https://data.globe.gov/system/photos/2019/06/22/1110634/original.jpg	https://data.globe.gov/system/photos/2019/06/22/1110636/original.jpg	https://data.globe.gov/system/photos/2019/06/22/1110635/original.jpg	https://data.globe.gov/system/photos/2019/06/22/1110637/original.jpg	https://data.globe.gov/system/photos/2019/06/22/1110639/original.jpg
GV_447	47.2973	-101.0914	509.1		https://www.google.com/maps/search/47.2973,-101.0914	https://data.globe.gov/system/photos/2019/06/22/1110640/original.jpg	https://data.globe.gov/system/photos/2019/06/22/1110642/original.jpg	https://data.globe.gov/system/photos/2019/06/22/1110641/original.jpg	https://data.globe.gov/system/photos/2019/06/22/1110643/original.jpg	https://data.globe.gov/system/photos/2019/06/22/1110645/original.jpg
GV_448	32.3049	-110.9148	800.7		https://www.google.com/maps/search/32.3049,-110.9148	https://data.globe.gov/system/photos/2019/06/22/1110764/original.jpg	https://data.globe.gov/system/photos/2019/06/22/1110766/original.jpg	https://data.globe.gov/system/photos/2019/06/22/1110765/original.jpg	https://data.globe.gov/system/photos/2019/06/22/1110767/original.jpg	https://data.globe.gov/system/photos/2019/06/22/1110769/original.jpg
GV_449	41.452	-84.751	255.6		https://www.google.com/maps/search/41.452,-84.751	https://data.globe.gov/system/photos/2019/06/22/1110777/original.jpg	https://data.globe.gov/system/photos/2019/06/22/1110779/original.jpg	https://data.globe.gov/system/photos/2019/06/22/1110778/original.jpg	https://data.globe.gov/system/photos/2019/06/22/1110780/original.jpg	https://data.globe.gov/system/photos/2019/06/22/1110782/original.jpg
GV_450	44.624	-123.0824	68.5		https://www.google.com/maps/search/44.624,-123.0824	https://data.globe.gov/system/photos/2019/06/22/1110846/original.jpg	https://data.globe.gov/system/photos/2019/06/22/1110848/original.jpg	https://data.globe.gov/system/photos/2019/06/22/1110847/original.jpg	https://data.globe.gov/system/photos/2019/06/22/1110849/original.jpg	https://data.globe.gov/system/photos/2019/06/22/1110851/original.jpg
GV_451	-25.4341	-56.2333	267.2		https://www.google.com/maps/search/-25.4341,-56.2333	https://data.globe.gov/system/photos/2019/06/22/1110888/original.jpg	https://data.globe.gov/system/photos/2019/06/22/1110890/original.jpg	https://data.globe.gov/system/photos/2019/06/22/1110889/original.jpg	https://data.globe.gov/system/photos/2019/06/22/1110891/original.jpg	https://data.globe.gov/system/photos/2019/06/22/1110893/original.jpg
GV_452	-25.4352	-56.2328	266.3		https://www.google.com/maps/search/-25.4352,-56.2328	https://data.globe.gov/system/photos/2019/06/22/1110894/original.jpg	https://data.globe.gov/system/photos/2019/06/22/1110896/original.jpg	https://data.globe.gov/system/photos/2019/06/22/1110895/original.jpg	https://data.globe.gov/system/photos/2019/06/22/1110897/original.jpg	https://data.globe.gov/system/photos/2019/06/22/1110899/original.jpg
GV_453	-25.4381	-56.2297	245.4		https://www.google.com/maps/search/-25.4381,-56.2297	https://data.globe.gov/system/photos/2019/06/22/1110900/original.jpg	https://data.globe.gov/system/photos/2019/06/22/1110902/original.jpg	https://data.globe.gov/system/photos/2019/06/22/1110901/original.jpg	https://data.globe.gov/system/photos/2019/06/22/1110903/original.jpg	https://data.globe.gov/system/photos/2019/06/22/1110905/original.jpg
GV_454	-25.4396	-56.2315	246.6		https://www.google.com/maps/search/-25.4396,-56.2315	https://data.globe.gov/system/photos/2019/06/22/1110906/original.jpg	https://data.globe.gov/system/photos/2019/06/22/1110908/original.jpg	https://data.globe.gov/system/photos/2019/06/22/1110907/original.jpg	https://data.globe.gov/system/photos/2019/06/22/1110910/original.jpg	https://data.globe.gov/system/photos/2019/06/22/1110913/original.jpg
GV_455	-25.4319	-56.2327	269.9		https://www.google.com/maps/search/-25.4319,-56.2327	https://data.globe.gov/system/photos/2019/06/22/1110926/original.jpg	https://data.globe.gov/system/photos/2019/06/22/1110930/original.jpg	https://data.globe.gov/system/photos/2019/06/22/1110927/original.jpg	https://data.globe.gov/system/photos/2019/06/22/1110932/original.jpg	https://data.globe.gov/system/photos/2019/06/22/1110934/original.jpg
GV_456	-25.4329	-56.226	279.6		https://www.google.com/maps/search/-25.4329,-56.226	https://data.globe.gov/system/photos/2019/06/22/1110935/original.jpg	https://data.globe.gov/system/photos/2019/06/22/1110937/original.jpg	https://data.globe.gov/system/photos/2019/06/22/1110936/original.jpg	https://data.globe.gov/system/photos/2019/06/22/1110938/original.jpg	https://data.globe.gov/system/photos/2019/06/22/1110940/original.jpg
GV_457	35.6886	-82.6437	635		https://www.google.com/maps/search/35.6886,-82.6437	https://data.globe.gov/system/photos/2019/06/22/1110982/original.jpg	https://data.globe.gov/system/photos/2019/06/22/1110984/original.jpg	https://data.globe.gov/system/photos/2019/06/22/1110983/original.jpg	https://data.globe.gov/system/photos/2019/06/22/1110985/original.jpg	https://data.globe.gov/system/photos/2019/06/22/1110987/original.jpg
GV_458	24.4359	118.3143	21.5		https://www.google.com/maps/search/24.4359,118.3143	https://data.globe.gov/system/photos/2019/06/23/1111027/original.jpg	https://data.globe.gov/system/photos/2019/06/23/1111029/original.jpg	https://data.globe.gov/system/photos/2019/06/23/1111028/original.jpg	https://data.globe.gov/system/photos/2019/06/23/1111030/original.jpg	https://data.globe.gov/system/photos/2019/06/23/1111032/original.jpg
GV_459	45.5323	-122.0937	1167		https://www.google.com/maps/search/45.5323,-122.0937	https://data.globe.gov/system/photos/2019/06/23/1111174/original.jpg	https://data.globe.gov/system/photos/2019/06/23/1111176/original.jpg	https://data.globe.gov/system/photos/2019/06/23/1111175/original.jpg	https://data.globe.gov/system/photos/2019/06/23/1111177/original.jpg	https://data.globe.gov/system/photos/2019/06/23/1111179/original.jpg
GV_460	45.5333	-122.0946	1156		https://www.google.com/maps/search/45.5333,-122.0946	https://data.globe.gov/system/photos/2019/06/23/1111180/original.jpg	https://data.globe.gov/system/photos/2019/06/23/1111182/original.jpg	https://data.globe.gov/system/photos/2019/06/23/1111181/original.jpg	https://data.globe.gov/system/photos/2019/06/23/1111183/original.jpg	https://data.globe.gov/system/photos/2019/06/23/1111185/original.jpg
GV_461	45.5354	-122.0952	1136.7		https://www.google.com/maps/search/45.5354,-122.0952	https://data.globe.gov/system/photos/2019/06/23/1111186/original.jpg	https://data.globe.gov/system/photos/2019/06/23/1111188/original.jpg	https://data.globe.gov/system/photos/2019/06/23/1111187/original.jpg	https://data.globe.gov/system/photos/2019/06/23/1111189/original.jpg	https://data.globe.gov/system/photos/2019/06/23/1111191/original.jpg
GV_462	45.5366	-122.0955	1125.9		https://www.google.com/maps/search/45.5366,-122.0955	https://data.globe.gov/system/photos/2019/06/23/1111192/original.jpg	https://data.globe.gov/system/photos/2019/06/23/1111194/original.jpg	https://data.globe.gov/system/photos/2019/06/23/1111193/original.jpg	https://data.globe.gov/system/photos/2019/06/23/1111195/original.jpg	https://data.globe.gov/system/photos/2019/06/23/1111197/original.jpg
GV_463	45.537	-122.0956	1121.6		https://www.google.com/maps/search/45.537,-122.0956	https://data.globe.gov/system/photos/2019/06/23/1111198/original.jpg	https://data.globe.gov/system/photos/2019/06/23/1111200/original.jpg	https://data.globe.gov/system/photos/2019/06/23/1111199/original.jpg	https://data.globe.gov/system/photos/2019/06/23/1111201/original.jpg	https://data.globe.gov/system/photos/2019/06/23/1111203/original.jpg
GV_464	45.5392	-122.0947	1100.9		https://www.google.com/maps/search/45.5392,-122.0947	https://data.globe.gov/system/photos/2019/06/23/1111204/original.jpg	https://data.globe.gov/system/photos/2019/06/23/1111206/original.jpg	https://data.globe.gov/system/photos/2019/06/23/1111205/original.jpg	https://data.globe.gov/system/photos/2019/06/23/1111207/original.jpg	https://data.globe.gov/system/photos/2019/06/23/1111209/original.jpg
GV_465	45.5394	-122.0957	1102.4		https://www.google.com/maps/search/45.5394,-122.0957	https://data.globe.gov/system/photos/2019/06/23/1111210/original.jpg	https://data.globe.gov/system/photos/2019/06/23/1111212/original.jpg	https://data.globe.gov/system/photos/2019/06/23/1111211/original.jpg	https://data.globe.gov/system/photos/2019/06/23/1111213/original.jpg	https://data.globe.gov/system/photos/2019/06/23/1111215/original.jpg
GV_466	45.5297	-122.0882	1186.4		https://www.google.com/maps/search/45.5297,-122.0882	https://data.globe.gov/system/photos/2019/06/23/1111216/original.jpg	https://data.globe.gov/system/photos/2019/06/23/1111218/original.jpg	https://data.globe.gov/system/photos/2019/06/23/1111217/original.jpg	https://data.globe.gov/system/photos/2019/06/23/1111219/original.jpg	https://data.globe.gov/system/photos/2019/06/23/1111221/original.jpg
GV_467	45.5349	-122.2607	253.2		https://www.google.com/maps/search/45.5349,-122.2607	https://data.globe.gov/system/photos/2019/06/23/1111222/original.jpg	https://data.globe.gov/system/photos/2019/06/23/1111224/original.jpg	https://data.globe.gov/system/photos/2019/06/23/1111223/original.jpg	https://data.globe.gov/system/photos/2019/06/23/1111225/original.jpg	https://data.globe.gov/system/photos/2019/06/23/1111227/original.jpg
GV_468	45.5288	-122.1253	691.6		https://www.google.com/maps/search/45.5288,-122.1253	https://data.globe.gov/system/photos/2019/06/23/1111240/original.jpg	https://data.globe.gov/system/photos/2019/06/23/1111242/original.jpg	https://data.globe.gov/system/photos/2019/06/23/1111241/original.jpg	https://data.globe.gov/system/photos/2019/06/23/1111243/original.jpg	https://data.globe.gov/system/photos/2019/06/23/1111245/original.jpg
GV_469	45.5484	-122.0937	937.4		https://www.google.com/maps/search/45.5484,-122.0937	https://data.globe.gov/system/photos/2019/06/23/1111246/original.jpg	https://data.globe.gov/system/photos/2019/06/23/1111248/original.jpg	https://data.globe.gov/system/photos/2019/06/23/1111247/original.jpg	https://data.globe.gov/system/photos/2019/06/23/1111249/original.jpg	https://data.globe.gov/system/photos/2019/06/23/1111251/original.jpg
GV_470	45.5266	-122.1229	701.8		https://www.google.com/maps/search/45.5266,-122.1229	https://data.globe.gov/system/photos/2019/06/23/1111252/original.jpg	https://data.globe.gov/system/photos/2019/06/23/1111254/original.jpg	https://data.globe.gov/system/photos/2019/06/23/1111253/original.jpg	https://data.globe.gov/system/photos/2019/06/23/1111255/original.jpg	https://data.globe.gov/system/photos/2019/06/23/1111257/original.jpg
GV_471	45.5328	-122.088	1212.1		https://www.google.com/maps/search/45.5328,-122.088	https://data.globe.gov/system/photos/2019/06/23/1111258/original.jpg	https://data.globe.gov/system/photos/2019/06/23/1111260/original.jpg	https://data.globe.gov/system/photos/2019/06/23/1111259/original.jpg	https://data.globe.gov/system/photos/2019/06/23/1111261/original.jpg	https://data.globe.gov/system/photos/2019/06/23/1111263/original.jpg
GV_472	45.5306	-122.0896	1210.2		https://www.google.com/maps/search/45.5306,-122.0896	https://data.globe.gov/system/photos/2019/06/23/1111264/original.jpg	https://data.globe.gov/system/photos/2019/06/23/1111266/original.jpg	https://data.globe.gov/system/photos/2019/06/23/1111265/original.jpg	https://data.globe.gov/system/photos/2019/06/23/1111267/original.jpg	https://data.globe.gov/system/photos/2019/06/23/1111269/original.jpg
GV_473	45.3357	-121.7106	1905.1		https://www.google.com/maps/search/45.3357,-121.7106	https://data.globe.gov/system/photos/2019/06/22/1111270/original.jpg	https://data.globe.gov/system/photos/2019/06/22/1111272/original.jpg	https://data.globe.gov/system/photos/2019/06/22/1111271/original.jpg	https://data.globe.gov/system/photos/2019/06/22/1111273/original.jpg	https://data.globe.gov/system/photos/2019/06/22/1111275/original.jpg
GV_474	45.3332	-121.7108	1838.3		https://www.google.com/maps/search/45.3332,-121.7108	https://data.globe.gov/system/photos/2019/06/22/1111276/original.jpg	https://data.globe.gov/system/photos/2019/06/22/1111278/original.jpg	https://data.globe.gov/system/photos/2019/06/22/1111277/original.jpg	https://data.globe.gov/system/photos/2019/06/22/1111279/original.jpg	https://data.globe.gov/system/photos/2019/06/22/1111281/original.jpg
GV_475	45.3329	-121.7113	1842.2		https://www.google.com/maps/search/45.3329,-121.7113	https://data.globe.gov/system/photos/2019/06/22/1111282/original.jpg	https://data.globe.gov/system/photos/2019/06/22/1111284/original.jpg	https://data.globe.gov/system/photos/2019/06/22/1111283/original.jpg	https://data.globe.gov/system/photos/2019/06/22/1111285/original.jpg	https://data.globe.gov/system/photos/2019/06/22/1111287/original.jpg
GV_476	45.3314	-121.7112	1815.9		https://www.google.com/maps/search/45.3314,-121.7112	https://data.globe.gov/system/photos/2019/06/22/1111293/original.jpg	https://data.globe.gov/system/photos/2019/06/22/1111295/original.jpg	https://data.globe.gov/system/photos/2019/06/22/1111294/original.jpg	https://data.globe.gov/system/photos/2019/06/22/1111296/original.jpg	https://data.globe.gov/system/photos/2019/06/22/1111298/original.jpg
GV_477	45.3298	-121.7084	1787.8		https://www.google.com/maps/search/45.3298,-121.7084	https://data.globe.gov/system/photos/2019/06/22/1111299/original.jpg	https://data.globe.gov/system/photos/2019/06/22/1111301/original.jpg	https://data.globe.gov/system/photos/2019/06/22/1111300/original.jpg	https://data.globe.gov/system/photos/2019/06/22/1111302/original.jpg	https://data.globe.gov/system/photos/2019/06/22/1111304/original.jpg
GV_478	45.5223	-122.157	557		https://www.google.com/maps/search/45.5223,-122.157	https://data.globe.gov/system/photos/2019/06/22/1111305/original.jpg	https://data.globe.gov/system/photos/2019/06/22/1111307/original.jpg	https://data.globe.gov/system/photos/2019/06/22/1111306/original.jpg	https://data.globe.gov/system/photos/2019/06/22/1111308/original.jpg	https://data.globe.gov/system/photos/2019/06/22/1111310/original.jpg
GV_479	45.52	-122.1483	603.6		https://www.google.com/maps/search/45.52,-122.1483	https://data.globe.gov/system/photos/2019/06/22/1111311/original.jpg	https://data.globe.gov/system/photos/2019/06/22/1111313/original.jpg	https://data.globe.gov/system/photos/2019/06/22/1111312/original.jpg	https://data.globe.gov/system/photos/2019/06/22/1111314/original.jpg	https://data.globe.gov/system/photos/2019/06/22/1111316/original.jpg
GV_480	45.5228	-122.1387	649		https://www.google.com/maps/search/45.5228,-122.1387	https://data.globe.gov/system/photos/2019/06/23/1111317/original.jpg	https://data.globe.gov/system/photos/2019/06/23/1111319/original.jpg	https://data.globe.gov/system/photos/2019/06/23/1111318/original.jpg	https://data.globe.gov/system/photos/2019/06/23/1111320/original.jpg	https://data.globe.gov/system/photos/2019/06/23/1111322/original.jpg
GV_481	45.5287	-122.1332	661		https://www.google.com/maps/search/45.5287,-122.1332	https://data.globe.gov/system/photos/2019/06/23/1111323/original.jpg	https://data.globe.gov/system/photos/2019/06/23/1111325/original.jpg	https://data.globe.gov/system/photos/2019/06/23/1111324/original.jpg	https://data.globe.gov/system/photos/2019/06/23/1111326/original.jpg	https://data.globe.gov/system/photos/2019/06/23/1111328/original.jpg
GV_482	45.5286	-122.1244	692.6		https://www.google.com/maps/search/45.5286,-122.1244	https://data.globe.gov/system/photos/2019/06/23/1111329/original.jpg	https://data.globe.gov/system/photos/2019/06/23/1111331/original.jpg	https://data.globe.gov/system/photos/2019/06/23/1111330/original.jpg	https://data.globe.gov/system/photos/2019/06/23/1111332/original.jpg	https://data.globe.gov/system/photos/2019/06/23/1111334/original.jpg
GV_483	45.5454	-122.1087	834.5		https://www.google.com/maps/search/45.5454,-122.1087	https://data.globe.gov/system/photos/2019/06/23/1111335/original.jpg	https://data.globe.gov/system/photos/2019/06/23/1111337/original.jpg	https://data.globe.gov/system/photos/2019/06/23/1111336/original.jpg	https://data.globe.gov/system/photos/2019/06/23/1111338/original.jpg	https://data.globe.gov/system/photos/2019/06/23/1111340/original.jpg
GV_484	45.5313	-122.0893	1199.9		https://www.google.com/maps/search/45.5313,-122.0893	https://data.globe.gov/system/photos/2019/06/23/1111341/original.jpg	https://data.globe.gov/system/photos/2019/06/23/1111343/original.jpg	https://data.globe.gov/system/photos/2019/06/23/1111342/original.jpg	https://data.globe.gov/system/photos/2019/06/23/1111344/original.jpg	https://data.globe.gov/system/photos/2019/06/23/1111346/original.jpg
GV_485	45.5319	-122.0887	1200.3		https://www.google.com/maps/search/45.5319,-122.0887	https://data.globe.gov/system/photos/2019/06/23/1111347/original.jpg	https://data.globe.gov/system/photos/2019/06/23/1111349/original.jpg	https://data.globe.gov/system/photos/2019/06/23/1111348/original.jpg	https://data.globe.gov/system/photos/2019/06/23/1111350/original.jpg	https://data.globe.gov/system/photos/2019/06/23/1111352/original.jpg
GV_486	45.5323	-122.0882	1214.3		https://www.google.com/maps/search/45.5323,-122.0882	https://data.globe.gov/system/photos/2019/06/23/1111353/original.jpg	https://data.globe.gov/system/photos/2019/06/23/1111355/original.jpg	https://data.globe.gov/system/photos/2019/06/23/1111354/original.jpg	https://data.globe.gov/system/photos/2019/06/23/1111356/original.jpg	https://data.globe.gov/system/photos/2019/06/23/1111358/original.jpg
GV_487	45.5327	-122.0876	1230.3		https://www.google.com/maps/search/45.5327,-122.0876	https://data.globe.gov/system/photos/2019/06/23/1111359/original.jpg	https://data.globe.gov/system/photos/2019/06/23/1111361/original.jpg	https://data.globe.gov/system/photos/2019/06/23/1111360/original.jpg	https://data.globe.gov/system/photos/2019/06/23/1111362/original.jpg	https://data.globe.gov/system/photos/2019/06/23/1111364/original.jpg
GV_488	45.5326	-122.0878	1222.8		https://www.google.com/maps/search/45.5326,-122.0878	https://data.globe.gov/system/photos/2019/06/23/1111365/original.jpg	https://data.globe.gov/system/photos/2019/06/23/1111367/original.jpg	https://data.globe.gov/system/photos/2019/06/23/1111366/original.jpg	https://data.globe.gov/system/photos/2019/06/23/1111368/original.jpg	https://data.globe.gov/system/photos/2019/06/23/1111370/original.jpg
GV_489	45.5305	-122.0911	1191		https://www.google.com/maps/search/45.5305,-122.0911	https://data.globe.gov/system/photos/2019/06/23/1111371/original.jpg	https://data.globe.gov/system/photos/2019/06/23/1111373/original.jpg	https://data.globe.gov/system/photos/2019/06/23/1111372/original.jpg	https://data.globe.gov/system/photos/2019/06/23/1111374/original.jpg	https://data.globe.gov/system/photos/2019/06/23/1111376/original.jpg
GV_490	45.3266	-121.6393	1389.6		https://www.google.com/maps/search/45.3266,-121.6393	https://data.globe.gov/system/photos/2019/06/22/1111377/original.jpg	https://data.globe.gov/system/photos/2019/06/22/1111379/original.jpg	https://data.globe.gov/system/photos/2019/06/22/1111378/original.jpg	https://data.globe.gov/system/photos/2019/06/22/1111380/original.jpg	https://data.globe.gov/system/photos/2019/06/22/1111382/original.jpg
GV_491	45.3266	-121.638	1386.1		https://www.google.com/maps/search/45.3266,-121.638	https://data.globe.gov/system/photos/2019/06/22/1111383/original.jpg	https://data.globe.gov/system/photos/2019/06/22/1111385/original.jpg	https://data.globe.gov/system/photos/2019/06/22/1111384/original.jpg	https://data.globe.gov/system/photos/2019/06/22/1111386/original.jpg	https://data.globe.gov/system/photos/2019/06/22/1111388/original.jpg
GV_492	45.3261	-121.6364	1383.1		https://www.google.com/maps/search/45.3261,-121.6364	https://data.globe.gov/system/photos/2019/06/22/1111389/original.jpg	https://data.globe.gov/system/photos/2019/06/22/1111391/original.jpg	https://data.globe.gov/system/photos/2019/06/22/1111390/original.jpg	https://data.globe.gov/system/photos/2019/06/22/1111392/original.jpg	https://data.globe.gov/system/photos/2019/06/22/1111394/original.jpg
GV_493	45.3228	-121.6364	1365.7		https://www.google.com/maps/search/45.3228,-121.6364	https://data.globe.gov/system/photos/2019/06/22/1111395/original.jpg	https://data.globe.gov/system/photos/2019/06/22/1111397/original.jpg	https://data.globe.gov/system/photos/2019/06/22/1111396/original.jpg	https://data.globe.gov/system/photos/2019/06/22/1111398/original.jpg	https://data.globe.gov/system/photos/2019/06/22/1111400/original.jpg
GV_494	45.3225	-121.6365	1364.8		https://www.google.com/maps/search/45.3225,-121.6365	https://data.globe.gov/system/photos/2019/06/22/1111401/original.jpg	https://data.globe.gov/system/photos/2019/06/22/1111403/original.jpg	https://data.globe.gov/system/photos/2019/06/22/1111402/original.jpg	https://data.globe.gov/system/photos/2019/06/22/1111404/original.jpg	https://data.globe.gov/system/photos/2019/06/22/1111406/original.jpg
GV_495	45.3223	-121.6359	1364		https://www.google.com/maps/search/45.3223,-121.6359	https://data.globe.gov/system/photos/2019/06/22/1111407/original.jpg	https://data.globe.gov/system/photos/2019/06/22/1111409/original.jpg	https://data.globe.gov/system/photos/2019/06/22/1111408/original.jpg	https://data.globe.gov/system/photos/2019/06/22/1111410/original.jpg	https://data.globe.gov/system/photos/2019/06/22/1111412/original.jpg
GV_496	45.3226	-121.6336	1359.8		https://www.google.com/maps/search/45.3226,-121.6336	https://data.globe.gov/system/photos/2019/06/22/1111433/original.jpg	https://data.globe.gov/system/photos/2019/06/22/1111435/original.jpg	https://data.globe.gov/system/photos/2019/06/22/1111434/original.jpg	https://data.globe.gov/system/photos/2019/06/22/1111436/original.jpg	https://data.globe.gov/system/photos/2019/06/22/1111438/original.jpg
GV_497	45.3314	-121.7075	1792.6		https://www.google.com/maps/search/45.3314,-121.7075	https://data.globe.gov/system/photos/2019/06/22/1111439/original.jpg	https://data.globe.gov/system/photos/2019/06/22/1111441/original.jpg	https://data.globe.gov/system/photos/2019/06/22/1111440/original.jpg	https://data.globe.gov/system/photos/2019/06/22/1111442/original.jpg	https://data.globe.gov/system/photos/2019/06/22/1111444/original.jpg
GV_498	45.3322	-121.7083	1813.8		https://www.google.com/maps/search/45.3322,-121.7083	https://data.globe.gov/system/photos/2019/06/22/1111445/original.jpg	https://data.globe.gov/system/photos/2019/06/22/1111447/original.jpg	https://data.globe.gov/system/photos/2019/06/22/1111446/original.jpg	https://data.globe.gov/system/photos/2019/06/22/1111448/original.jpg	https://data.globe.gov/system/photos/2019/06/22/1111450/original.jpg
GV_499	45.3333	-121.7084	1831.6		https://www.google.com/maps/search/45.3333,-121.7084	https://data.globe.gov/system/photos/2019/06/22/1111451/original.jpg	https://data.globe.gov/system/photos/2019/06/22/1111453/original.jpg	https://data.globe.gov/system/photos/2019/06/22/1111452/original.jpg	https://data.globe.gov/system/photos/2019/06/22/1111454/original.jpg	https://data.globe.gov/system/photos/2019/06/22/1111456/original.jpg
GV_500	45.3329	-121.709	1833.1		https://www.google.com/maps/search/45.3329,-121.709	https://data.globe.gov/system/photos/2019/06/22/1111457/original.jpg	https://data.globe.gov/system/photos/2019/06/22/1111459/original.jpg	https://data.globe.gov/system/photos/2019/06/22/1111458/original.jpg	https://data.globe.gov/system/photos/2019/06/22/1111460/original.jpg	https://data.globe.gov/system/photos/2019/06/22/1111462/original.jpg
GV_501	45.3329	-121.7098	1835.7		https://www.google.com/maps/search/45.3329,-121.7098	https://data.globe.gov/system/photos/2019/06/22/1111463/original.jpg	https://data.globe.gov/system/photos/2019/06/22/1111465/original.jpg	https://data.globe.gov/system/photos/2019/06/22/1111464/original.jpg	https://data.globe.gov/system/photos/2019/06/22/1111466/original.jpg	https://data.globe.gov/system/photos/2019/06/22/1111468/original.jpg
GV_502	45.3347	-121.7092	1870.2		https://www.google.com/maps/search/45.3347,-121.7092	https://data.globe.gov/system/photos/2019/06/22/1111469/original.jpg	https://data.globe.gov/system/photos/2019/06/22/1111471/original.jpg	https://data.globe.gov/system/photos/2019/06/22/1111470/original.jpg	https://data.globe.gov/system/photos/2019/06/22/1111472/original.jpg	https://data.globe.gov/system/photos/2019/06/22/1111474/original.jpg
GV_503	45.3362	-121.7089	1902.6		https://www.google.com/maps/search/45.3362,-121.7089	https://data.globe.gov/system/photos/2019/06/22/1111475/original.jpg	https://data.globe.gov/system/photos/2019/06/22/1111477/original.jpg	https://data.globe.gov/system/photos/2019/06/22/1111476/original.jpg	https://data.globe.gov/system/photos/2019/06/22/1111478/original.jpg	https://data.globe.gov/system/photos/2019/06/22/1111480/original.jpg
GV_504	45.3371	-121.7107	1920.9		https://www.google.com/maps/search/45.3371,-121.7107	https://data.globe.gov/system/photos/2019/06/22/1111481/original.jpg	https://data.globe.gov/system/photos/2019/06/22/1111483/original.jpg	https://data.globe.gov/system/photos/2019/06/22/1111482/original.jpg	https://data.globe.gov/system/photos/2019/06/22/1111484/original.jpg	https://data.globe.gov/system/photos/2019/06/22/1111486/original.jpg
GV_505	45.3365	-121.711	1909		https://www.google.com/maps/search/45.3365,-121.711	https://data.globe.gov/system/photos/2019/06/22/1111487/original.jpg	https://data.globe.gov/system/photos/2019/06/22/1111489/original.jpg	https://data.globe.gov/system/photos/2019/06/22/1111488/original.jpg	https://data.globe.gov/system/photos/2019/06/22/1111490/original.jpg	https://data.globe.gov/system/photos/2019/06/22/1111492/original.jpg
GV_506	45.495	-122.4043	117.9		https://www.google.com/maps/search/45.495,-122.4043	https://data.globe.gov/system/photos/2019/06/22/1111493/original.jpg	https://data.globe.gov/system/photos/2019/06/22/1111495/original.jpg	https://data.globe.gov/system/photos/2019/06/22/1111494/original.jpg	https://data.globe.gov/system/photos/2019/06/22/1111496/original.jpg	https://data.globe.gov/system/photos/2019/06/22/1111498/original.jpg
GV_507	45.2826	-121.685	1269.2		https://www.google.com/maps/search/45.2826,-121.685	https://data.globe.gov/system/photos/2019/06/22/1111499/original.jpg	https://data.globe.gov/system/photos/2019/06/22/1111501/original.jpg	https://data.globe.gov/system/photos/2019/06/22/1111500/original.jpg	https://data.globe.gov/system/photos/2019/06/22/1111502/original.jpg	https://data.globe.gov/system/photos/2019/06/22/1111504/original.jpg
GV_508	45.2813	-121.684	1250.5		https://www.google.com/maps/search/45.2813,-121.684	https://data.globe.gov/system/photos/2019/06/22/1111505/original.jpg	https://data.globe.gov/system/photos/2019/06/22/1111507/original.jpg	https://data.globe.gov/system/photos/2019/06/22/1111506/original.jpg	https://data.globe.gov/system/photos/2019/06/22/1111508/original.jpg	https://data.globe.gov/system/photos/2019/06/22/1111510/original.jpg
GV_509	45.2824	-121.6843	1265.5		https://www.google.com/maps/search/45.2824,-121.6843	https://data.globe.gov/system/photos/2019/06/22/1111511/original.jpg	https://data.globe.gov/system/photos/2019/06/22/1111513/original.jpg	https://data.globe.gov/system/photos/2019/06/22/1111512/original.jpg	https://data.globe.gov/system/photos/2019/06/22/1111514/original.jpg	https://data.globe.gov/system/photos/2019/06/22/1111516/original.jpg
GV_510	45.2824	-121.6843	1265.5		https://www.google.com/maps/search/45.2824,-121.6843	https://data.globe.gov/system/photos/2019/06/22/1111517/original.jpg	https://data.globe.gov/system/photos/2019/06/22/1111519/original.jpg	https://data.globe.gov/system/photos/2019/06/22/1111518/original.jpg	https://data.globe.gov/system/photos/2019/06/22/1111520/original.jpg	https://data.globe.gov/system/photos/2019/06/22/1111522/original.jpg
GV_511	45.2826	-121.6859	1261.7		https://www.google.com/maps/search/45.2826,-121.6859	https://data.globe.gov/system/photos/2019/06/22/1111523/original.jpg	https://data.globe.gov/system/photos/2019/06/22/1111525/original.jpg	https://data.globe.gov/system/photos/2019/06/22/1111524/original.jpg	https://data.globe.gov/system/photos/2019/06/22/1111526/original.jpg	https://data.globe.gov/system/photos/2019/06/22/1111528/original.jpg
GV_512	45.3036	-121.6742	1304.4		https://www.google.com/maps/search/45.3036,-121.6742	https://data.globe.gov/system/photos/2019/06/22/1111529/original.jpg	https://data.globe.gov/system/photos/2019/06/22/1111531/original.jpg	https://data.globe.gov/system/photos/2019/06/22/1111530/original.jpg	https://data.globe.gov/system/photos/2019/06/22/1111532/original.jpg	https://data.globe.gov/system/photos/2019/06/22/1111534/original.jpg
GV_513	45.3215	-121.6361	1381.9		https://www.google.com/maps/search/45.3215,-121.6361	https://data.globe.gov/system/photos/2019/06/22/1111535/original.jpg	https://data.globe.gov/system/photos/2019/06/22/1111537/original.jpg	https://data.globe.gov/system/photos/2019/06/22/1111536/original.jpg	https://data.globe.gov/system/photos/2019/06/22/1111538/original.jpg	https://data.globe.gov/system/photos/2019/06/22/1111540/original.jpg
GV_514	45.3045	-121.6742	1311.3		https://www.google.com/maps/search/45.3045,-121.6742	https://data.globe.gov/system/photos/2019/06/22/1111541/original.jpg	https://data.globe.gov/system/photos/2019/06/22/1111543/original.jpg	https://data.globe.gov/system/photos/2019/06/22/1111542/original.jpg	https://data.globe.gov/system/photos/2019/06/22/1111544/original.jpg	https://data.globe.gov/system/photos/2019/06/22/1111546/original.jpg
GV_515	45.3047	-121.6746	1313.4		https://www.google.com/maps/search/45.3047,-121.6746	https://data.globe.gov/system/photos/2019/06/22/1111547/original.jpg	https://data.globe.gov/system/photos/2019/06/22/1111549/original.jpg	https://data.globe.gov/system/photos/2019/06/22/1111548/original.jpg	https://data.globe.gov/system/photos/2019/06/22/1111550/original.jpg	https://data.globe.gov/system/photos/2019/06/22/1111552/original.jpg
GV_516	45.3056	-121.6766	1325.4		https://www.google.com/maps/search/45.3056,-121.6766	https://data.globe.gov/system/photos/2019/06/22/1111553/original.jpg	https://data.globe.gov/system/photos/2019/06/22/1111555/original.jpg	https://data.globe.gov/system/photos/2019/06/22/1111554/original.jpg	https://data.globe.gov/system/photos/2019/06/22/1111556/original.jpg	https://data.globe.gov/system/photos/2019/06/22/1111558/original.jpg
GV_517	45.3054	-121.6778	1328.5		https://www.google.com/maps/search/45.3054,-121.6778	https://data.globe.gov/system/photos/2019/06/22/1111559/original.jpg	https://data.globe.gov/system/photos/2019/06/22/1111561/original.jpg	https://data.globe.gov/system/photos/2019/06/22/1111560/original.jpg	https://data.globe.gov/system/photos/2019/06/22/1111562/original.jpg	https://data.globe.gov/system/photos/2019/06/22/1111564/original.jpg
GV_518	45.3059	-121.6785	1337.8		https://www.google.com/maps/search/45.3059,-121.6785	https://data.globe.gov/system/photos/2019/06/22/1111565/original.jpg	https://data.globe.gov/system/photos/2019/06/22/1111567/original.jpg	https://data.globe.gov/system/photos/2019/06/22/1111566/original.jpg	https://data.globe.gov/system/photos/2019/06/22/1111568/original.jpg	https://data.globe.gov/system/photos/2019/06/22/1111570/original.jpg
GV_519	45.3065	-121.6792	1351.3		https://www.google.com/maps/search/45.3065,-121.6792	https://data.globe.gov/system/photos/2019/06/22/1111571/original.jpg	https://data.globe.gov/system/photos/2019/06/22/1111573/original.jpg	https://data.globe.gov/system/photos/2019/06/22/1111572/original.jpg	https://data.globe.gov/system/photos/2019/06/22/1111574/original.jpg	https://data.globe.gov/system/photos/2019/06/22/1111576/original.jpg
GV_520	45.3069	-121.6792	1349.3		https://www.google.com/maps/search/45.3069,-121.6792	https://data.globe.gov/system/photos/2019/06/22/1111577/original.jpg	https://data.globe.gov/system/photos/2019/06/22/1111579/original.jpg	https://data.globe.gov/system/photos/2019/06/22/1111578/original.jpg	https://data.globe.gov/system/photos/2019/06/22/1111580/original.jpg	https://data.globe.gov/system/photos/2019/06/22/1111582/original.jpg
GV_521	45.3067	-121.6779	1342.4		https://www.google.com/maps/search/45.3067,-121.6779	https://data.globe.gov/system/photos/2019/06/22/1111583/original.jpg	https://data.globe.gov/system/photos/2019/06/22/1111585/original.jpg	https://data.globe.gov/system/photos/2019/06/22/1111584/original.jpg	https://data.globe.gov/system/photos/2019/06/22/1111586/original.jpg	https://data.globe.gov/system/photos/2019/06/22/1111588/original.jpg
GV_522	45.3057	-121.6755	1316.9		https://www.google.com/maps/search/45.3057,-121.6755	https://data.globe.gov/system/photos/2019/06/22/1111589/original.jpg	https://data.globe.gov/system/photos/2019/06/22/1111591/original.jpg	https://data.globe.gov/system/photos/2019/06/22/1111590/original.jpg	https://data.globe.gov/system/photos/2019/06/22/1111592/original.jpg	https://data.globe.gov/system/photos/2019/06/22/1111594/original.jpg
GV_523	45.3222	-121.6365	1367.1		https://www.google.com/maps/search/45.3222,-121.6365	https://data.globe.gov/system/photos/2019/06/22/1111595/original.jpg	https://data.globe.gov/system/photos/2019/06/22/1111597/original.jpg	https://data.globe.gov/system/photos/2019/06/22/1111596/original.jpg	https://data.globe.gov/system/photos/2019/06/22/1111598/original.jpg	https://data.globe.gov/system/photos/2019/06/22/1111600/original.jpg
GV_524	45.3211	-121.6362	1386.6		https://www.google.com/maps/search/45.3211,-121.6362	https://data.globe.gov/system/photos/2019/06/22/1111601/original.jpg	https://data.globe.gov/system/photos/2019/06/22/1111603/original.jpg	https://data.globe.gov/system/photos/2019/06/22/1111602/original.jpg	https://data.globe.gov/system/photos/2019/06/22/1111604/original.jpg	https://data.globe.gov/system/photos/2019/06/22/1111606/original.jpg
GV_525	45.3204	-121.6362	1393.6		https://www.google.com/maps/search/45.3204,-121.6362	https://data.globe.gov/system/photos/2019/06/22/1111607/original.jpg	https://data.globe.gov/system/photos/2019/06/22/1111609/original.jpg	https://data.globe.gov/system/photos/2019/06/22/1111608/original.jpg	https://data.globe.gov/system/photos/2019/06/22/1111610/original.jpg	https://data.globe.gov/system/photos/2019/06/22/1111612/original.jpg
GV_526	45.3202	-121.6368	1401.2		https://www.google.com/maps/search/45.3202,-121.6368	https://data.globe.gov/system/photos/2019/06/22/1111613/original.jpg	https://data.globe.gov/system/photos/2019/06/22/1111615/original.jpg	https://data.globe.gov/system/photos/2019/06/22/1111614/original.jpg	https://data.globe.gov/system/photos/2019/06/22/1111616/original.jpg	https://data.globe.gov/system/photos/2019/06/22/1111618/original.jpg
GV_527	45.3189	-121.6364	1404.2		https://www.google.com/maps/search/45.3189,-121.6364	https://data.globe.gov/system/photos/2019/06/22/1111619/original.jpg	https://data.globe.gov/system/photos/2019/06/22/1111621/original.jpg	https://data.globe.gov/system/photos/2019/06/22/1111620/original.jpg	https://data.globe.gov/system/photos/2019/06/22/1111622/original.jpg	https://data.globe.gov/system/photos/2019/06/22/1111624/original.jpg
GV_528	45.3188	-121.6364	1404.6		https://www.google.com/maps/search/45.3188,-121.6364	https://data.globe.gov/system/photos/2019/06/22/1111625/original.jpg	https://data.globe.gov/system/photos/2019/06/22/1111627/original.jpg	https://data.globe.gov/system/photos/2019/06/22/1111626/original.jpg	https://data.globe.gov/system/photos/2019/06/22/1111628/original.jpg	https://data.globe.gov/system/photos/2019/06/22/1111630/original.jpg
GV_529	45.3194	-121.6416	1438.8		https://www.google.com/maps/search/45.3194,-121.6416	https://data.globe.gov/system/photos/2019/06/22/1111631/original.jpg	https://data.globe.gov/system/photos/2019/06/22/1111633/original.jpg	https://data.globe.gov/system/photos/2019/06/22/1111632/original.jpg	https://data.globe.gov/system/photos/2019/06/22/1111634/original.jpg	https://data.globe.gov/system/photos/2019/06/22/1111636/original.jpg
GV_530	45.3186	-121.639	1416.4		https://www.google.com/maps/search/45.3186,-121.639	https://data.globe.gov/system/photos/2019/06/22/1111637/original.jpg	https://data.globe.gov/system/photos/2019/06/22/1111639/original.jpg	https://data.globe.gov/system/photos/2019/06/22/1111638/original.jpg	https://data.globe.gov/system/photos/2019/06/22/1111640/original.jpg	https://data.globe.gov/system/photos/2019/06/22/1111642/original.jpg
GV_531	45.3186	-121.6401	1407		https://www.google.com/maps/search/45.3186,-121.6401	https://data.globe.gov/system/photos/2019/06/22/1111643/original.jpg	https://data.globe.gov/system/photos/2019/06/22/1111645/original.jpg	https://data.globe.gov/system/photos/2019/06/22/1111644/original.jpg	https://data.globe.gov/system/photos/2019/06/22/1111646/original.jpg	https://data.globe.gov/system/photos/2019/06/22/1111648/original.jpg
GV_532	45.3186	-121.6403	1394.1		https://www.google.com/maps/search/45.3186,-121.6403	https://data.globe.gov/system/photos/2019/06/22/1111649/original.jpg	https://data.globe.gov/system/photos/2019/06/22/1111651/original.jpg	https://data.globe.gov/system/photos/2019/06/22/1111650/original.jpg	https://data.globe.gov/system/photos/2019/06/22/1111652/original.jpg	https://data.globe.gov/system/photos/2019/06/22/1111654/original.jpg
GV_533	45.3191	-121.6404	1432		https://www.google.com/maps/search/45.3191,-121.6404	https://data.globe.gov/system/photos/2019/06/22/1111655/original.jpg	https://data.globe.gov/system/photos/2019/06/22/1111657/original.jpg	https://data.globe.gov/system/photos/2019/06/22/1111656/original.jpg	https://data.globe.gov/system/photos/2019/06/22/1111658/original.jpg	https://data.globe.gov/system/photos/2019/06/22/1111660/original.jpg

GV_534	45.3194	-121.641	1437.3		https://www.google.com/maps/search/45.3194,-121.641	https://data.globe.gov/system/photos/2019/06/22/1111661/original.jpg	https://data.globe.gov/system/photos/2019/06/22/1111663/original.jpg	https://data.globe.gov/system/photos/2019/06/22/1111662/original.jpg	https://data.globe.gov/system/photos/2019/06/22/1111664/original.jpg	https://data.globe.gov/system/photos/2019/06/22/1111666/original.jpg
GV_535	45.3196	-121.6426	1452.2		https://www.google.com/maps/search/45.3196,-121.6426	https://data.globe.gov/system/photos/2019/06/22/1111667/original.jpg	https://data.globe.gov/system/photos/2019/06/22/1111669/original.jpg	https://data.globe.gov/system/photos/2019/06/22/1111668/original.jpg	https://data.globe.gov/system/photos/2019/06/22/1111670/original.jpg	https://data.globe.gov/system/photos/2019/06/22/1111672/original.jpg
GV_536	45.3197	-121.6452	1477.1		https://www.google.com/maps/search/45.3197,-121.6452	https://data.globe.gov/system/photos/2019/06/22/1111673/original.jpg	https://data.globe.gov/system/photos/2019/06/22/1111675/original.jpg	https://data.globe.gov/system/photos/2019/06/22/1111674/original.jpg	https://data.globe.gov/system/photos/2019/06/22/1111676/original.jpg	https://data.globe.gov/system/photos/2019/06/22/1111678/original.jpg
GV_537	45.3201	-121.6463	1489.1		https://www.google.com/maps/search/45.3201,-121.6463	https://data.globe.gov/system/photos/2019/06/22/1111679/original.jpg	https://data.globe.gov/system/photos/2019/06/22/1111681/original.jpg	https://data.globe.gov/system/photos/2019/06/22/1111680/original.jpg	https://data.globe.gov/system/photos/2019/06/22/1111682/original.jpg	https://data.globe.gov/system/photos/2019/06/22/1111684/original.jpg
GV_538	45.3208	-121.6487	1512.1		https://www.google.com/maps/search/45.3208,-121.6487	https://data.globe.gov/system/photos/2019/06/22/1111685/original.jpg	https://data.globe.gov/system/photos/2019/06/22/1111687/original.jpg	https://data.globe.gov/system/photos/2019/06/22/1111686/original.jpg	https://data.globe.gov/system/photos/2019/06/22/1111688/original.jpg	https://data.globe.gov/system/photos/2019/06/22/1111690/original.jpg
GV_539	45.3211	-121.649	1514.7		https://www.google.com/maps/search/45.3211,-121.649	https://data.globe.gov/system/photos/2019/06/22/1111691/original.jpg	https://data.globe.gov/system/photos/2019/06/22/1111693/original.jpg	https://data.globe.gov/system/photos/2019/06/22/1111692/original.jpg	https://data.globe.gov/system/photos/2019/06/22/1111694/original.jpg	https://data.globe.gov/system/photos/2019/06/22/1111696/original.jpg
GV_540	45.3221	-121.6499	1521.3		https://www.google.com/maps/search/45.3221,-121.6499	https://data.globe.gov/system/photos/2019/06/22/1111697/original.jpg	https://data.globe.gov/system/photos/2019/06/22/1111699/original.jpg	https://data.globe.gov/system/photos/2019/06/22/1111698/original.jpg	https://data.globe.gov/system/photos/2019/06/22/1111700/original.jpg	https://data.globe.gov/system/photos/2019/06/22/1111702/original.jpg
GV_541	45.3265	-121.6552	1572.8		https://www.google.com/maps/search/45.3265,-121.6552	https://data.globe.gov/system/photos/2019/06/22/1111703/original.jpg	https://data.globe.gov/system/photos/2019/06/22/1111705/original.jpg	https://data.globe.gov/system/photos/2019/06/22/1111704/original.jpg	https://data.globe.gov/system/photos/2019/06/22/1111706/original.jpg	https://data.globe.gov/system/photos/2019/06/22/1111708/original.jpg
GV_542	45.3242	-121.6515	1553.2		https://www.google.com/maps/search/45.3242,-121.6515	https://data.globe.gov/system/photos/2019/06/22/1111709/original.jpg	https://data.globe.gov/system/photos/2019/06/22/1111711/original.jpg	https://data.globe.gov/system/photos/2019/06/22/1111710/original.jpg	https://data.globe.gov/system/photos/2019/06/22/1111712/original.jpg	https://data.globe.gov/system/photos/2019/06/22/1111714/original.jpg
GV_543	45.3243	-121.6532	1549		https://www.google.com/maps/search/45.3243,-121.6532	https://data.globe.gov/system/photos/2019/06/22/1111715/original.jpg	https://data.globe.gov/system/photos/2019/06/22/1111717/original.jpg	https://data.globe.gov/system/photos/2019/06/22/1111716/original.jpg	https://data.globe.gov/system/photos/2019/06/22/1111718/original.jpg	https://data.globe.gov/system/photos/2019/06/22/1111720/original.jpg
GV_544	45.325	-121.6537	1554.2		https://www.google.com/maps/search/45.325,-121.6537	https://data.globe.gov/system/photos/2019/06/22/1111721/original.jpg	https://data.globe.gov/system/photos/2019/06/22/1111723/original.jpg	https://data.globe.gov/system/photos/2019/06/22/1111722/original.jpg	https://data.globe.gov/system/photos/2019/06/22/1111724/original.jpg	https://data.globe.gov/system/photos/2019/06/22/1111726/original.jpg
GV_545	45.3259	-121.6545	1568		https://www.google.com/maps/search/45.3259,-121.6545	https://data.globe.gov/system/photos/2019/06/22/1111727/original.jpg	https://data.globe.gov/system/photos/2019/06/22/1111729/original.jpg	https://data.globe.gov/system/photos/2019/06/22/1111728/original.jpg	https://data.globe.gov/system/photos/2019/06/22/1111730/original.jpg	https://data.globe.gov/system/photos/2019/06/22/1111732/original.jpg
GV_546	45.3265	-121.6549	1574.2		https://www.google.com/maps/search/45.3265,-121.6549	https://data.globe.gov/system/photos/2019/06/22/1111733/original.jpg	https://data.globe.gov/system/photos/2019/06/22/1111735/original.jpg	https://data.globe.gov/system/photos/2019/06/22/1111734/original.jpg	https://data.globe.gov/system/photos/2019/06/22/1111736/original.jpg	https://data.globe.gov/system/photos/2019/06/22/1111738/original.jpg
GV_547	45.3273	-121.6553	1580.9		https://www.google.com/maps/search/45.3273,-121.6553	https://data.globe.gov/system/photos/2019/06/22/1111739/original.jpg	https://data.globe.gov/system/photos/2019/06/22/1111741/original.jpg	https://data.globe.gov/system/photos/2019/06/22/1111740/original.jpg	https://data.globe.gov/system/photos/2019/06/22/1111742/original.jpg	https://data.globe.gov/system/photos/2019/06/22/1111744/original.jpg
GV_548	45.3277	-121.6541	1585.9		https://www.google.com/maps/search/45.3277,-121.6541	https://data.globe.gov/system/photos/2019/06/22/1111745/original.jpg	https://data.globe.gov/system/photos/2019/06/22/1111747/original.jpg	https://data.globe.gov/system/photos/2019/06/22/1111746/original.jpg	https://data.globe.gov/system/photos/2019/06/22/1111748/original.jpg	https://data.globe.gov/system/photos/2019/06/22/1111750/original.jpg
GV_549	45.3283	-121.6531	1601		https://www.google.com/maps/search/45.3283,-121.6531	https://data.globe.gov/system/photos/2019/06/22/1111751/original.jpg	https://data.globe.gov/system/photos/2019/06/22/1111753/original.jpg	https://data.globe.gov/system/photos/2019/06/22/1111752/original.jpg	https://data.globe.gov/system/photos/2019/06/22/1111754/original.jpg	https://data.globe.gov/system/photos/2019/06/22/1111756/original.jpg
GV_550	45.3283	-121.6517	1605.1		https://www.google.com/maps/search/45.3283,-121.6517	https://data.globe.gov/system/photos/2019/06/22/1111757/original.jpg	https://data.globe.gov/system/photos/2019/06/22/1111759/original.jpg	https://data.globe.gov/system/photos/2019/06/22/1111758/original.jpg	https://data.globe.gov/system/photos/2019/06/22/1111760/original.jpg	https://data.globe.gov/system/photos/2019/06/22/1111762/original.jpg
GV_551	45.3283	-121.6507	1594.3		https://www.google.com/maps/search/45.3283,-121.6507	https://data.globe.gov/system/photos/2019/06/22/1111763/original.jpg	https://data.globe.gov/system/photos/2019/06/22/1111765/original.jpg	https://data.globe.gov/system/photos/2019/06/22/1111764/original.jpg	https://data.globe.gov/system/photos/2019/06/22/1111766/original.jpg	https://data.globe.gov/system/photos/2019/06/22/1111768/original.jpg
GV_552	45.3287	-121.6506	1594		https://www.google.com/maps/search/45.3287,-121.6506	https://data.globe.gov/system/photos/2019/06/22/1111769/original.jpg	https://data.globe.gov/system/photos/2019/06/22/1111771/original.jpg	https://data.globe.gov/system/photos/2019/06/22/1111770/original.jpg	https://data.globe.gov/system/photos/2019/06/22/1111772/original.jpg	https://data.globe.gov/system/photos/2019/06/22/1111774/original.jpg
GV_553	45.3284	-121.6502	1584.1		https://www.google.com/maps/search/45.3284,-121.6502	https://data.globe.gov/system/photos/2019/06/22/1111775/original.jpg	https://data.globe.gov/system/photos/2019/06/22/1111777/original.jpg	https://data.globe.gov/system/photos/2019/06/22/1111776/original.jpg	https://data.globe.gov/system/photos/2019/06/22/1111778/original.jpg	https://data.globe.gov/system/photos/2019/06/22/1111780/original.jpg
GV_554	45.3285	-121.6491	1553.4		https://www.google.com/maps/search/45.3285,-121.6491	https://data.globe.gov/system/photos/2019/06/22/1111781/original.jpg	https://data.globe.gov/system/photos/2019/06/22/1111783/original.jpg	https://data.globe.gov/system/photos/2019/06/22/1111782/original.jpg	https://data.globe.gov/system/photos/2019/06/22/1111784/original.jpg	https://data.globe.gov/system/photos/2019/06/22/1111786/original.jpg
GV_555	45.3257	-121.6432	1384.4		https://www.google.com/maps/search/45.3257,-121.6432	https://data.globe.gov/system/photos/2019/06/22/1111787/original.jpg	https://data.globe.gov/system/photos/2019/06/22/1111789/original.jpg	https://data.globe.gov/system/photos/2019/06/22/1111788/original.jpg	https://data.globe.gov/system/photos/2019/06/22/1111790/original.jpg	https://data.globe.gov/system/photos/2019/06/22/1111792/original.jpg
GV_556	45.3287	-121.6467	1522.2		https://www.google.com/maps/search/45.3287,-121.6467	https://data.globe.gov/system/photos/2019/06/22/1111793/original.jpg	https://data.globe.gov/system/photos/2019/06/22/1111795/original.jpg	https://data.globe.gov/system/photos/2019/06/22/1111794/original.jpg	https://data.globe.gov/system/photos/2019/06/22/1111796/original.jpg	https://data.globe.gov/system/photos/2019/06/22/1111798/original.jpg
GV_557	45.3277	-121.6457	1484.8		https://www.google.com/maps/search/45.3277,-121.6457	https://data.globe.gov/system/photos/2019/06/22/1111799/original.jpg	https://data.globe.gov/system/photos/2019/06/22/1111801/original.jpg	https://data.globe.gov/system/photos/2019/06/22/1111800/original.jpg	https://data.globe.gov/system/photos/2019/06/22/1111802/original.jpg	https://data.globe.gov/system/photos/2019/06/22/1111804/original.jpg
GV_558	45.3274	-121.645	1461		https://www.google.com/maps/search/45.3274,-121.645	https://data.globe.gov/system/photos/2019/06/22/1111805/original.jpg	https://data.globe.gov/system/photos/2019/06/22/1111807/original.jpg	https://data.globe.gov/system/photos/2019/06/22/1111806/original.jpg	https://data.globe.gov/system/photos/2019/06/22/1111808/original.jpg	https://data.globe.gov/system/photos/2019/06/22/1111811/original.jpg
GV_559	45.327	-121.6447	1440.2		https://www.google.com/maps/search/45.327,-121.6447	https://data.globe.gov/system/photos/2019/06/22/1111813/original.jpg	https://data.globe.gov/system/photos/2019/06/22/1111816/original.jpg	https://data.globe.gov/system/photos/2019/06/22/1111814/original.jpg	https://data.globe.gov/system/photos/2019/06/22/1111817/original.jpg	https://data.globe.gov/system/photos/2019/06/22/1111820/original.jpg
GV_560	45.3264	-121.6444	1418.3		https://www.google.com/maps/search/45.3264,-121.6444	https://data.globe.gov/system/photos/2019/06/22/1111822/original.jpg	https://data.globe.gov/system/photos/2019/06/22/1111825/original.jpg	https://data.globe.gov/system/photos/2019/06/22/1111823/original.jpg	https://data.globe.gov/system/photos/2019/06/22/1111827/original.jpg	https://data.globe.gov/system/photos/2019/06/22/1111830/original.jpg
GV_561	45.3266	-121.6422	1400.4		https://www.google.com/maps/search/45.3266,-121.6422	https://data.globe.gov/system/photos/2019/06/22/1111831/original.jpg	https://data.globe.gov/system/photos/2019/06/22/1111834/original.jpg	https://data.globe.gov/system/photos/2019/06/22/1111832/original.jpg	https://data.globe.gov/system/photos/2019/06/22/1111835/original.jpg	https://data.globe.gov/system/photos/2019/06/22/1111837/original.jpg
GV_562	45.7739	-123.401	479.6		https://www.google.com/maps/search/45.7739,-123.401	https://data.globe.gov/system/photos/2019/06/23/1112441/original.jpg	https://data.globe.gov/system/photos/2019/06/23/1112443/original.jpg	https://data.globe.gov/system/photos/2019/06/23/1112442/original.jpg	https://data.globe.gov/system/photos/2019/06/23/1112444/original.jpg	https://data.globe.gov/system/photos/2019/06/23/1112446/original.jpg
GV_563	45.9849	-123.9331	7.1		https://www.google.com/maps/search/45.9849,-123.9331	https://data.globe.gov/system/photos/2019/06/23/1112447/original.jpg	https://data.globe.gov/system/photos/2019/06/23/1112449/original.jpg	https://data.globe.gov/system/photos/2019/06/23/1112448/original.jpg	https://data.globe.gov/system/photos/2019/06/23/1112450/original.jpg	https://data.globe.gov/system/photos/2019/06/23/1112452/original.jpg
GV_564	45.9975	-123.9243	4.6		https://www.google.com/maps/search/45.9975,-123.9243	https://data.globe.gov/system/photos/2019/06/23/1112458/original.jpg	https://data.globe.gov/system/photos/2019/06/23/1112460/original.jpg	https://data.globe.gov/system/photos/2019/06/23/1112459/original.jpg	https://data.globe.gov/system/photos/2019/06/23/1112461/original.jpg	https://data.globe.gov/system/photos/2019/06/23/1112463/original.jpg
GV_565	45.9973	-123.9242	5		https://www.google.com/maps/search/45.9973,-123.9242	https://data.globe.gov/system/photos/2019/06/23/1112464/original.jpg	https://data.globe.gov/system/photos/2019/06/23/1112466/original.jpg	https://data.globe.gov/system/photos/2019/06/23/1112465/original.jpg	https://data.globe.gov/system/photos/2019/06/23/1112467/original.jpg	https://data.globe.gov/system/photos/2019/06/23/1112469/original.jpg
GV_566	35.1445	-106.6805	1517		https://www.google.com/maps/search/35.1445,-106.6805	https://data.globe.gov/system/photos/2019/06/23/1112470/original.jpg	https://data.globe.gov/system/photos/2019/06/23/1112472/original.jpg	https://data.globe.gov/system/photos/2019/06/23/1112471/original.jpg	https://data.globe.gov/system/photos/2019/06/23/1112473/original.jpg	https://data.globe.gov/system/photos/2019/06/23/1112475/original.jpg
GV_567	29.9051	-95.6815	42.7		https://www.google.com/maps/search/29.9051,-95.6815	https://data.globe.gov/system/photos/2019/06/23/1112574/original.jpg	https://data.globe.gov/system/photos/2019/06/23/1112576/original.jpg	https://data.globe.gov/system/photos/2019/06/23/1112575/original.jpg	https://data.globe.gov/system/photos/2019/06/23/1112577/original.jpg	https://data.globe.gov/system/photos/2019/06/23/1112579/original.jpg
GV_568	45.9858	-123.9342	3.6		https://www.google.com/maps/search/45.9858,-123.9342	https://data.globe.gov/system/photos/2019/06/23/1112687/original.jpg	https://data.globe.gov/system/photos/2019/06/23/1112689/original.jpg	https://data.globe.gov/system/photos/2019/06/23/1112688/original.jpg	https://data.globe.gov/system/photos/2019/06/23/1112690/original.jpg	https://data.globe.gov/system/photos/2019/06/23/1112692/original.jpg
GV_569	45.9844	-123.9319	6		https://www.google.com/maps/search/45.9844,-123.9319	https://data.globe.gov/system/photos/2019/06/23/1112700/original.jpg	https://data.globe.gov/system/photos/2019/06/23/1112702/original.jpg	https://data.globe.gov/system/photos/2019/06/23/1112701/original.jpg	https://data.globe.gov/system/photos/2019/06/23/1112703/original.jpg	https://data.globe.gov/system/photos/2019/06/23/1112705/original.jpg
GV_570	45.9837	-123.9349	3.6		https://www.google.com/maps/search/45.9837,-123.9349	https://data.globe.gov/system/photos/2019/06/23/1112706/original.jpg	https://data.globe.gov/system/photos/2019/06/23/1112708/original.jpg	https://data.globe.gov/system/photos/2019/06/23/1112707/original.jpg	https://data.globe.gov/system/photos/2019/06/23/1112709/original.jpg	https://data.globe.gov/system/photos/2019/06/23/1112711/original.jpg
GV_571	45.9854	-123.9353	2		https://www.google.com/maps/search/45.9854,-123.9353	https://data.globe.gov/system/photos/2019/06/23/1112712/original.jpg	https://data.globe.gov/system/photos/2019/06/23/1112714/original.jpg	https://data.globe.gov/system/photos/2019/06/23/1112713/original.jpg	https://data.globe.gov/system/photos/2019/06/23/1112715/original.jpg	https://data.globe.gov/system/photos/2019/06/23/1112717/original.jpg
GV_572	45.985	-123.9339	5.9		https://www.google.com/maps/search/45.985,-123.9339	https://data.globe.gov/system/photos/2019/06/23/1112718/original.jpg	https://data.globe.gov/system/photos/2019/06/23/1112720/original.jpg	https://data.globe.gov/system/photos/2019/06/23/1112719/original.jpg	https://data.globe.gov/system/photos/2019/06/23/1112721/original.jpg	https://data.globe.gov/system/photos/2019/06/23/1112723/original.jpg
GV_573	45.9844	-123.932	6.4		https://www.google.com/maps/search/45.9844,-123.932	https://data.globe.gov/system/photos/2019/06/23/1112724/original.jpg	https://data.globe.gov/system/photos/2019/06/23/1112726/original.jpg	https://data.globe.gov/system/photos/2019/06/23/1112725/original.jpg	https://data.globe.gov/system/photos/2019/06/23/1112727/original.jpg	https://data.globe.gov/system/photos/2019/06/23/1112729/original.jpg
GV_574	45.9843	-123.9318	6.9		https://www.google.com/maps/search/45.9843,-123.9318	https://data.globe.gov/system/photos/2019/06/23/1112730/original.jpg	https://data.globe.gov/system/photos/2019/06/23/1112732/original.jpg	https://data.globe.gov/system/photos/2019/06/23/1112731/original.jpg	https://data.globe.gov/system/photos/2019/06/23/1112733/original.jpg	https://data.globe.gov/system/photos/2019/06/23/1112735/original.jpg
GV_575	45.9845	-123.9318	6.9		https://www.google.com/maps/search/45.9845,-123.9318	https://data.globe.gov/system/photos/2019/06/23/1112736/original.jpg	https://data.globe.gov/system/photos/2019/06/23/1112738/original.jpg	https://data.globe.gov/system/photos/2019/06/23/1112737/original.jpg	https://data.globe.gov/system/photos/2019/06/23/1112739/original.jpg	https://data.globe.gov/system/photos/2019/06/23/1112741/original.jpg
GV_576	46.1451	-123.9145	15.3		https://www.google.com/maps/search/46.1451,-123.9145	https://data.globe.gov/system/photos/2019/06/23/1112742/original.jpg	https://data.globe.gov/system/photos/2019/06/23/1112744/original.jpg	https://data.globe.gov/system/photos/2019/06/23/1112743/original.jpg	https://data.globe.gov/system/photos/2019/06/23/1112745/original.jpg	https://data.globe.gov/system/photos/2019/06/23/1112747/original.jpg
GV_577	46.1455	-123.9155	14.5		https://www.google.com/maps/search/46.1455,-123.9155	https://data.globe.gov/system/photos/2019/06/23/1112748/original.jpg	https://data.globe.gov/system/photos/2019/06/23/1112750/original.jpg	https://data.globe.gov/system/photos/2019/06/23/1112749/original.jpg	https://data.globe.gov/system/photos/2019/06/23/1112751/original.jpg	https://data.globe.gov/system/photos/2019/06/23/1112753/original.jpg
GV_578	46.1379	-123.8777	9.8		https://www.google.com/maps/search/46.1379,-123.8777	https://data.globe.gov/system/photos/2019/06/23/1112754/original.jpg	https://data.globe.gov/system/photos/2019/06/23/1112756/original.jpg	https://data.globe.gov/system/photos/2019/06/23/1112755/original.jpg	https://data.globe.gov/system/photos/2019/06/23/1112757/original.jpg	https://data.globe.gov/system/photos/2019/06/23/1112759/original.jpg
GV_579	46.1334	-123.8815	13.7		https://www.google.com/maps/search/46.1334,-123.8815	https://data.globe.gov/system/photos/2019/06/23/1112760/original.jpg	https://data.globe.gov/system/photos/2019/06/23/1112762/original.jpg	https://data.globe.gov/system/photos/2019/06/23/1112761/original.jpg	https://data.globe.gov/system/photos/2019/06/23/1112763/original.jpg	https://data.globe.gov/system/photos/2019/06/23/1112765/original.jpg
GV_580	46.1379	-123.8773	9.5		https://www.google.com/maps/search/46.1379,-123.8773	https://data.globe.gov/system/photos/2019/06/23/1112766/original.jpg	https://data.globe.gov/system/photos/2019/06/23/1112768/original.jpg	https://data.globe.gov/system/photos/2019/06/23/1112767/original.jpg	https://data.globe.gov/system/photos/2019/06/23/1112769/original.jpg	https://data.globe.gov/system/photos/2019/06/23/1112772/original.jpg
GV_581	46.1336	-123.881	15.6		https://www.google.com/maps/search/46.1336,-123.881	https://data.globe.gov/system/photos/2019/06/23/1112774/original.jpg	https://data.globe.gov/system/photos/2019/06/23/1112778/original.jpg	https://data.globe.gov/system/photos/2019/06/23/1112776/original.jpg	https://data.globe.gov/system/photos/2019/06/23/1112780/original.jpg	https://data.globe.gov/system/photos/2019/06/23/1112783/original.jpg
GV_582	46.1336	-123.8815	15.4		https://www.google.com/maps/search/46.1336,-123.8815	https://data.globe.gov/system/photos/2019/06/23/1112784/original.jpg	https://data.globe.gov/system/photos/2019/06/23/1112786/original.jpg	https://data.globe.gov/system/photos/2019/06/23/1112785/original.jpg	https://data.globe.gov/system/photos/2019/06/23/1112787/original.jpg	https://data.globe.gov/system/photos/2019/06/23/1112789/original.jpg
GV_583	46.1334	-123.8802	10.3		https://www.google.com/maps/search/46.1334,-123.8802	https://data.globe.gov/system/photos/2019/06/23/1112790/original.jpg	https://data.globe.gov/system/photos/2019/06/23/1112792/original.jpg	https://data.globe.gov/system/photos/2019/06/23/1112791/original.jpg	https://data.globe.gov/system/photos/2019/06/23/1112793/original.jpg	https://data.globe.gov/system/photos/2019/06/23/1112795/original.jpg
GV_584	46.1318	-123.8787	3.4		https://www.google.com/maps/search/46.1318,-123.8787	https://data.globe.gov/system/photos/2019/06/23/1112796/original.jpg	https://data.globe.gov/system/photos/2019/06/23/1112798/original.jpg	https://data.globe.gov/system/photos/2019/06/23/1112797/original.jpg	https://data.globe.gov/system/photos/2019/06/23/1112799/original.jpg	https://data.globe.gov/system/photos/2019/06/23/1112801/original.jpg
GV_585	46.1323	-123.8783	2.4		https://www.google.com/maps/search/46.1323,-123.8783	https://data.globe.gov/system/photos/2019/06/23/1112802/original.jpg	https://data.globe.gov/system/photos/2019/06/23/1112804/original.jpg	https://data.globe.gov/system/photos/2019/06/23/1112803/original.jpg	https://data.globe.gov/system/photos/2019/06/23/1112805/original.jpg	https://data.globe.gov/system/photos/2019/06/23/1112807/original.jpg
GV_586	46.1319	-123.8787	3.2		https://www.google.com/maps/search/46.1319,-123.8787	https://data.globe.gov/system/photos/2019/06/23/1112808/original.jpg	https://data.globe.gov/system/photos/2019/06/23/1112810/original.jpg	https://data.globe.gov/system/photos/2019/06/23/1112809/original.jpg	https://data.globe.gov/system/photos/2019/06/23/1112811/original.jpg	https://data.globe.gov/system/photos/2019/06/23/1112813/original.jpg
GV_587	46.1301	-123.8785	3.7		https://www.google.com/maps/search/46.1301,-123.8785	https://data.globe.gov/system/photos/2019/06/23/1112814/original.jpg	https://data.globe.gov/system/photos/2019/06/23/1112816/original.jpg	https://data.globe.gov/system/photos/2019/06/23/1112815/original.jpg	https://data.globe.gov/system/photos/2019/06/23/1112817/original.jpg	https://data.globe.gov/system/photos/2019/06/23/1112819/original.jpg
GV_588	46.1295	-123.8785	3.4		https://www.google.com/maps/search/46.1295,-123.8785	https://data.globe.gov/system/photos/2019/06/23/1112820/original.jpg	https://data.globe.gov/system/photos/2019/06/23/1112822/original.jpg	https://data.globe.gov/system/photos/2019/06/23/1112821/original.jpg	https://data.globe.gov/system/photos/2019/06/23/1112823/original.jpg	https://data.globe.gov/system/photos/2019/06/23/1112825/original.jpg
GV_589	46.1288	-123.8785	3.7		https://www.google.com/maps/search/46.1288,-123.8785	https://data.globe.gov/system/photos/2019/06/23/1112826/original.jpg	https://data.globe.gov/system/photos/2019/06/23/1112828/original.jpg	https://data.globe.gov/system/photos/2019/06/23/1112827/original.jpg	https://data.globe.gov/system/photos/2019/06/23/1112829/original.jpg	https://data.globe.gov/system/photos/2019/06/23/1112831/original.jpg
GV_590	46.1287	-123.8781	0.2		https://www.google.com/maps/search/46.1287,-123.8781	https://data.globe.gov/system/photos/2019/06/23/1112832/original.jpg	https://data.globe.gov/system/photos/2019/06/23/1112834/original.jpg	https://data.globe.gov/system/photos/2019/06/23/1112833/original.jpg	https://data.globe.gov/system/photos/2019/06/23/1112835/original.jpg	https://data.globe.gov/system/photos/2019/06/23/1112837/original.jpg
GV_591	46.1287	-123.8776	3.9		https://www.google.com/maps/search/46.1287,-123.8776	https://data.globe.gov/system/photos/2019/06/23/1112838/original.jpg	https://data.globe.gov/system/photos/2019/06/23/1112840/original.jpg	https://data.globe.gov/system/photos/2019/06/23/1112839/original.jpg	https://data.globe.gov/system/photos/2019/06/23/1112841/original.jpg	https://data.globe.gov/system/photos/2019/06/23/1112843/original.jpg
GV_592	46.1324	-123.8783	3.4		https://www.google.com/maps/search/46.1324,-123.8783	https://data.globe.gov/system/photos/2019/06/23/1112844/original.jpg	https://data.globe.gov/system/photos/2019/06/23/1112846/original.jpg	https://data.globe.gov/system/photos/2019/06/23/1112845/original.jpg	https://data.globe.gov/system/photos/2019/06/23/1112847/original.jpg	https://data.globe.gov/system/photos/2019/06/23/1112849/original.jpg
GV_593	46.133	-123.879	5.6		https://www.google.com/maps/search/46.133,-123.879	https://data.globe.gov/system/photos/2019/06/23/1112850/original.jpg	https://data.globe.gov/system/photos/2019/06/23/1112852/original.jpg	https://data.globe.gov/system/photos/2019/06/23/1112851/original.jpg	https://data.globe.gov/system/photos/2019/06/23/1112853/original.jpg	https://data.globe.gov/system/photos/2019/06/23/1112855/original.jpg
GV_594	46.1333	-123.8793	10.9		https://www.google.com/maps/search/46.1333,-123.8793	https://data.globe.gov/system/photos/2019/06/23/1112856/original.jpg	https://data.globe.gov/system/photos/2019/06/23/1112858/original.jpg	https://data.globe.gov/system/photos/2019/06/23/1112857/original.jpg	https://data.globe.gov/system/photos/2019/06/23/1112859/original.jpg	https://data.globe.gov/system/photos/2019/06/23/1112861/original.jpg
GV_595	46.1339	-123.8792	12.6		https://www.google.com/maps/search/46.1339,-123.8792	https://data.globe.gov/system/photos/2019/06/23/1112862/original.jpg	https://data.globe.gov/system/photos/2019/06/23/1112864/original.jpg	https://data.globe.gov/system/photos/2019/06/23/1112863/original.jpg	https://data.globe.gov/system/photos/2019/06/23/1112865/original.jpg	https://data.globe.gov/system/photos/2019/06/23/1112867/original.jpg
GV_596	46.1342	-123.8791	13		https://www.google.com/maps/search/46.1342,-123.8791	https://data.globe.gov/system/photos/2019/06/23/1112868/original.jpg	https://data.globe.gov/system/photos/2019/06/23/1112870/original.jpg	https://data.globe.gov/system/photos/2019/06/23/1112869/original.jpg	https://data.globe.gov/system/photos/2019/06/23/1112871/original.jpg	https://data.globe.gov/system/photos/2019/06/23/1112873/original.jpg
GV_597	46.1341	-123.8791	12.9		https://www.google.com/maps/search/46.1341,-123.8791	https://data.globe.gov/system/photos/2019/06/23/1112874/original.jpg	https://data.globe.gov/system/photos/2019/06/23/1112876/original.jpg	https://data.globe.gov/system/photos/2019/06/23/1112875/original.jpg	https://data.globe.gov/system/photos/2019/06/23/1112877/original.jpg	https://data.globe.gov/system/photos/2019/06/23/1112879/original.jpg
GV_598	46.1347	-123.8787	8.7		https://www.google.com/maps/search/46.1347,-123.8787	https://data.globe.gov/system/photos/2019/06/23/1112880/original.jpg	https://data.globe.gov/system/photos/2019/06/23/1112882/original.jpg	https://data.globe.gov/system/photos/2019/06/23/1112881/original.jpg	https://data.globe.gov/system/photos/2019/06/23/1112883/original.jpg	https://data.globe.gov/system/photos/2019/06/23/1112885/original.jpg
GV_599	46.1342	-123.879	13		https://www.google.com/maps/search/46.1342,-123.879	https://data.globe.gov/system/photos/2019/06/23/1112886/original.jpg	https://data.globe.gov/system/photos/2019/06/23/1112888/original.jpg	https://data.globe.gov/system/photos/2019/06/23/1112887/original.jpg	https://data.globe.gov/system/photos/2019/06/23/1112889/original.jpg	https://data.globe.gov/system/photos/2019/06/23/1112891/original.jpg
GV_600	46.1345	-123.8799	13.9		https://www.google.com/maps/search/46.1345,-123.8799	https://data.globe.gov/system/photos/2019/06/23/1112892/original.jpg	https://data.globe.gov/system/photos/2019/06/23/1112894/original.jpg	https://data.globe.gov/system/photos/2019/06/23/1112893/original.jpg	https://data.globe.gov/system/photos/2019/06/23/1112895/original.jpg	https://data.globe.gov/system/photos/2019/06/23/1112897/original.jpg
GV_601	46.1344	-123.8798	14		https://www.google.com/maps/search/46.1344,-123.8798	https://data.globe.gov/system/photos/2019/06/23/1112898/original.jpg	https://data.globe.gov/system/photos/2019/06/23/1112900/original.jpg	https://data.globe.gov/system/photos/2019/06/23/1112899/original.jpg	https://data.globe.gov/system/photos/2019/06/23/1112901/original.jpg	https://data.globe.gov/system/photos/2019/06/23/1112903/original.jpg
GV_602	46.1339	-123.8856	25.2		https://www.google.com/maps/search/46.1339,-123.8856	https://data.globe.gov/system/photos/2019/06/23/1112904/original.jpg	https://data.globe.gov/system/photos/2019/06/23/1112906/original.jpg	https://data.globe.gov/system/photos/2019/06/23/1112905/original.jpg	https://data.globe.gov/system/photos/2019/06/23/1112907/original.jpg	https://data.globe.gov/system/photos/2019/06/23/1112921/original.jpg
GV_603	46.1341	-123.8884	33.5		https://www.google.com/maps/search/46.1341,-123.8884	https://data.globe.gov/system/photos/2019/06/23/1112922/original.jpg	https://data.globe.gov/system/photos/2019/06/23/1112924/original.jpg	https://data.globe.gov/system/photos/2019/06/23/1112923/original.jpg	https://data.globe.gov/system/photos/2019/06/23/1112925/original.jpg	https://data.globe.gov/system/photos/2019/06/23/1112927/original.jpg
GV_604	46.1328	-123.8917	56.8		https://www.google.com/maps/search/46.1328,-123.8917	https://data.globe.gov/system/photos/2019/06/23/1112928/original.jpg	https://data.globe.gov/system/photos/2019/06/23/1112930/original.jpg	https://data.globe.gov/system/photos/2019/06/23/1112929/original.jpg	https://data.globe.gov/system/photos/2019/06/23/1112931/original.jpg	https://data.globe.gov/system/photos/2019/06/23/1112933/original.jpg
GV_605	46.1176	-123.8749	2.6		https://www.google.com/maps/search/46.1176,-123.8749	https://data.globe.gov/system/photos/2019/06/23/1112934/original.jpg	https://data.globe.gov/system/photos/2019/06/23/1112936/original.jpg	https://data.globe.gov/system/photos/2019/06/23/1112935/original.jpg	https://data.globe.gov/system/photos/2019/06/23/1112937/original.jpg	https://data.globe.gov/system/photos/2019/06/23/1112939/original.jpg
GV_606	46.1179	-123.8753	3.6		https://www.google.com/maps/search/46.1179,-123.8753	https://data.globe.gov/system/photos/2019/06/23/1112940/original.jpg	https://data.globe.gov/system/photos/2019/06/23/1112942/original.jpg	https://data.globe.gov/system/photos/2019/06/23/1112941/original.jpg	https://data.globe.gov/system/photos/2019/06/23/1112944/original.jpg	https://data.globe.gov/system/photos/2019/06/23/1112947/original.jpg
GV_607	46.1178	-123.876	4.4		https://www.google.com/maps/search/46.1178,-123.876	https://data.globe.gov/system/photos/2019/06/23/1112948/original.jpg	https://data.globe.gov/system/photos/2019/06/23/1112950/original.jpg	https://data.globe.gov/system/photos/2019/06/23/1112949/original.jpg	https://data.globe.gov/system/photos/2019/06/23/1112952/original.jpg	https://data.globe.gov/system/photos/2019/06/23/1112954/original.jpg
GV_608	46.1217	-123.8766	4.2		https://www.google.com/maps/search/46.1217,-123.8766	https://data.globe.gov/system/photos/2019/06/23/1112955/original.jpg	https://data.globe.gov/system/photos/2019/06/23/1112959/original.jpg	https://data.globe.gov/system/photos/2019/06/23/1112957/original.jpg	https://data.globe.gov/system/photos/2019/06/23/1112960/original.jpg	https://data.globe.gov/system/photos/2019/06/23/1112963/original.jpg
GV_609	46.1093	-123.8544	2.4		https://www.google.com/maps/search/46.1093,-123.8544	https://data.globe.gov/system/photos/2019/06/23/1112964/original.jpg	https://data.globe.gov/system/photos/2019/06/23/1112966/original.jpg	https://data.globe.gov/system/photos/2019/06/23/1112965/original.jpg	https://data.globe.gov/system/photos/2019/06/23/1112967/original.jpg	https://data.globe.gov/system/photos/2019/06/23/1112969/original.jpg
GV_610	46.1481	-123.8431	18.8		https://www.google.com/maps/search/46.1481,-123.8431	https://data.globe.gov/system/photos/2019/06/24/1112970/original.jpg	https://data.globe.gov/system/photos/2019/06/24/1112972/original.jpg	https://data.globe.gov/system/photos/2019/06/24/1112971/original.jpg	https://data.globe.gov/system/photos/2019/06/24/1112973/original.jpg	https://data.globe.gov/system/photos/2019/06/24/1112975/original.jpg
GV_611	46.152	-123.8393	3		https://www.google.com/maps/search/46.152,-123.8393	https://data.globe.gov/system/photos/2019/06/24/1112976/original.jpg	https://data.globe.gov/system/photos/2019/06/24/1112978/original.jpg	https://data.globe.gov/system/photos/2019/06/24/1112977/original.jpg	https://data.globe.gov/system/photos/2019/06/24/1112979/original.jpg	https://data.globe.gov/system/photos/2019/06/24/1112981/original.jpg
GV_612	46.1665	-123.8379	3.4		https://www.google.com/maps/search/46.1665,-123.8379	https://data.globe.gov/system/photos/2019/06/24/1112982/original.jpg	https://data.globe.gov/system/photos/2019/06/24/1112984/original.jpg	https://data.globe.gov/system/photos/2019/06/24/1112983/original.jpg	https://data.globe.gov/system/photos/2019/06/24/1112985/original.jpg	https://data.globe.gov/system/photos/2019/06/24/1112987/original.jpg
GV_613	46.1687	-123.8382	0.6		https://www.google.com/maps/search/46.1687,-123.8382	https://data.globe.gov/system/photos/2019/06/24/1112988/original.jpg	https://data.globe.gov/system/photos/2019/06/24/1112990/original.jpg	https://data.globe.gov/system/photos/2019/06/24/1112989/original.jpg	https://data.globe.gov/system/photos/2019/06/24/1112991/original.jpg	https://data.globe.gov/system/photos/2019/06/24/1112993/original.jpg
GV_614	46.1707	-123.8365	1.9		https://www.google.com/maps/search/46.1707,-123.8365	https://data.globe.gov/system/photos/2019/06/24/1112994/original.jpg	https://data.globe.gov/system/photos/2019/06/24/1112996/original.jpg	https://data.globe.gov/system/photos/2019/06/24/1112995/original.jpg	https://data.globe.gov/system/photos/2019/06/24/1112997/original.jpg	https://data.globe.gov/system/photos/2019/06/24/1112999/original.jpg
GV_615	46.1698	-123.8367	1		https://www.google.com/maps/search/46.1698,-123.8367	https://data.globe.gov/system/photos/2019/06/24/1113000/original.jpg	https://data.globe.gov/system/photos/2019/06/24/1113002/original.jpg	https://data.globe.gov/system/photos/2019/06/24/1113001/original.jpg	https://data.globe.gov/system/photos/2019/06/24/1113003/original.jpg	https://data.globe.gov/system/photos/2019/06/24/1113005/original.jpg
GV_616	46.1708	-123.8367	3.2		https://www.google.com/maps/search/46.1708,-123.8367	https://data.globe.gov/system/photos/2019/06/24/1113006/original.jpg	https://data.globe.gov/system/photos/2019/06/24/1113008/original.jpg	https://data.globe.gov/system/photos/2019/06/24/1113007/original.jpg	https://data.globe.gov/system/photos/2019/06/24/1113009/original.jpg	https://data.globe.gov/system/photos/2019/06/24/1113011/original.jpg
GV_617	46.1705	-123.8364	1		https://www.google.com/maps/search/46.1705,-123.8364	https://data.globe.gov/system/photos/2019/06/24/1113012/original.jpg	https://data.globe.gov/system/photos/2019/06/24/1113014/original.jpg	https://data.globe.gov/system/photos/2019/06/24/1113013/original.jpg	https://data.globe.gov/system/photos/2019/06/24/1113015/original.jpg	https://data.globe.gov/system/photos/2019/06/24/1113017/original.jpg
GV_618	46.1697	-123.8379	1		https://www.google.com/maps/search/46.1697,-123.8379	https://data.globe.gov/system/photos/2019/06/24/1113018/original.jpg	https://data.globe.gov/system/photos/2019/06/24/1113020/original.jpg	https://data.globe.gov/system/photos/2019/06/24/1113019/original.jpg	https://data.globe.gov/system/photos/2019/06/24/1113021/original.jpg	https://data.globe.gov/system/photos/2019/06/24/1113023/original.jpg
GV_619	46.1698	-123.8372	0.8		https://www.google.com/maps/search/46.1698,-123.8372	https://data.globe.gov/system/photos/2019/06/24/1113024/original.jpg	https://data.globe.gov/system/photos/2019/06/24/1113026/original.jpg	https://data.globe.gov/system/photos/2019/06/24/1113025/original.jpg	https://data.globe.gov/system/photos/2019/06/24/1113027/original.jpg	https://data.globe.gov/system/photos/2019/06/24/1113029/original.jpg
GV_620	46.1709	-123.838	4.3		https://www.google.com/maps/search/46.1709,-123.838	https://data.globe.gov/system/photos/2019/06/24/1113030/original.jpg	https://data.globe.gov/system/photos/2019/06/24/1113032/original.jpg	https://data.globe.gov/system/photos/2019/06/24/1113031/original.jpg	https://data.globe.gov/system/photos/2019/06/24/1113033/original.jpg	https://data.globe.gov/system/photos/2019/06/24/1113035/original.jpg
GV_621	46.1836	-123.8349	73		https://www.google.com/maps/search/46.1836,-123.8349	https://data.globe.gov/system/photos/2019/06/24/1113036/original.jpg	https://data.globe.gov/system/photos/2019/06/24/1113038/original.jpg	https://data.globe.gov/system/photos/2019/06/24/1113037/original.jpg	https://data.globe.gov/system/photos/2019/06/24/1113039/original.jpg	https://data.globe.gov/system/photos/2019/06/24/1113041/original.jpg
GV_622	46.1866	-123.8352	39.1		https://www.google.com/maps/search/46.1866,-123.8352	https://data.globe.gov/system/photos/2019/06/24/1113042/original.jpg	https://data.globe.gov/system/photos/2019/06/24/1113044/original.jpg	https://data.globe.gov/system/photos/2019/06/24/1113043/original.jpg	https://data.globe.gov/system/photos/2019/06/24/1113045/original.jpg	https://data.globe.gov/system/photos/2019/06/24/1113047/original.jpg
GV_623	46.1869	-123.8355	37.8		https://www.google.com/maps/search/46.1869,-123.8355	https://data.globe.gov/system/photos/2019/06/24/1113048/original.jpg	https://data.globe.gov/system/photos/2019/06/24/1113050/original.jpg	https://data.globe.gov/system/photos/2019/06/24/1113049/original.jpg	https://data.globe.gov/system/photos/2019/06/24/1113051/original.jpg	https://data.globe.gov/system/photos/2019/06/24/1113053/original.jpg

GV_624	46.1871	-123.8356	36.5		https://www.google.com/maps/search/46.1871,-123.8356	https://data.globe.gov/system/photos/2019/06/24/1113054/original.jpg	https://data.globe.gov/system/photos/2019/06/24/1113056/original.jpg	https://data.globe.gov/system/photos/2019/06/24/1113055/original.jpg	https://data.globe.gov/system/photos/2019/06/24/1113057/original.jpg	https://data.globe.gov/system/photos/2019/06/24/1113059/original.jpg
GV_625	46.1891	-123.8316	6.6		https://www.google.com/maps/search/46.1891,-123.8316	https://data.globe.gov/system/photos/2019/06/24/1113065/original.jpg	https://data.globe.gov/system/photos/2019/06/24/1113067/original.jpg	https://data.globe.gov/system/photos/2019/06/24/1113066/original.jpg	https://data.globe.gov/system/photos/2019/06/24/1113068/original.jpg	https://data.globe.gov/system/photos/2019/06/24/1113070/original.jpg
GV_626	46.19	-123.8241	5.2		https://www.google.com/maps/search/46.19,-123.8241	https://data.globe.gov/system/photos/2019/06/24/1113071/original.jpg	https://data.globe.gov/system/photos/2019/06/24/1113073/original.jpg	https://data.globe.gov/system/photos/2019/06/24/1113072/original.jpg	https://data.globe.gov/system/photos/2019/06/24/1113074/original.jpg	https://data.globe.gov/system/photos/2019/06/24/1113076/original.jpg
GV_627	46.1902	-123.8232	3.8		https://www.google.com/maps/search/46.1902,-123.8232	https://data.globe.gov/system/photos/2019/06/24/1113077/original.jpg	https://data.globe.gov/system/photos/2019/06/24/1113079/original.jpg	https://data.globe.gov/system/photos/2019/06/24/1113078/original.jpg	https://data.globe.gov/system/photos/2019/06/24/1113080/original.jpg	https://data.globe.gov/system/photos/2019/06/24/1113082/original.jpg
GV_628	46.1655	-123.5922	12.7		https://www.google.com/maps/search/46.1655,-123.5922	https://data.globe.gov/system/photos/2019/06/24/1113083/original.jpg	https://data.globe.gov/system/photos/2019/06/24/1113085/original.jpg	https://data.globe.gov/system/photos/2019/06/24/1113084/original.jpg	https://data.globe.gov/system/photos/2019/06/24/1113086/original.jpg	https://data.globe.gov/system/photos/2019/06/24/1113088/original.jpg
GV_629	46.1897	-123.8222	5.1		https://www.google.com/maps/search/46.1897,-123.8222	https://data.globe.gov/system/photos/2019/06/24/1113089/original.jpg	https://data.globe.gov/system/photos/2019/06/24/1113091/original.jpg	https://data.globe.gov/system/photos/2019/06/24/1113090/original.jpg	https://data.globe.gov/system/photos/2019/06/24/1113092/original.jpg	https://data.globe.gov/system/photos/2019/06/24/1113094/original.jpg
GV_630	46.1898	-123.82	5.3		https://www.google.com/maps/search/46.1898,-123.82	https://data.globe.gov/system/photos/2019/06/24/1113095/original.jpg	https://data.globe.gov/system/photos/2019/06/24/1113097/original.jpg	https://data.globe.gov/system/photos/2019/06/24/1113096/original.jpg	https://data.globe.gov/system/photos/2019/06/24/1113098/original.jpg	https://data.globe.gov/system/photos/2019/06/24/1113100/original.jpg
GV_631	46.1689	-123.741	7.1		https://www.google.com/maps/search/46.1689,-123.741	https://data.globe.gov/system/photos/2019/06/24/1113101/original.jpg	https://data.globe.gov/system/photos/2019/06/24/1113103/original.jpg	https://data.globe.gov/system/photos/2019/06/24/1113102/original.jpg	https://data.globe.gov/system/photos/2019/06/24/1113104/original.jpg	https://data.globe.gov/system/photos/2019/06/24/1113106/original.jpg
GV_632	46.1655	-123.5899	29		https://www.google.com/maps/search/46.1655,-123.5899	https://data.globe.gov/system/photos/2019/06/24/1113107/original.jpg	https://data.globe.gov/system/photos/2019/06/24/1113109/original.jpg	https://data.globe.gov/system/photos/2019/06/24/1113108/original.jpg	https://data.globe.gov/system/photos/2019/06/24/1113110/original.jpg	https://data.globe.gov/system/photos/2019/06/24/1113112/original.jpg
GV_633	46.1644	-123.5921	14.5		https://www.google.com/maps/search/46.1644,-123.5921	https://data.globe.gov/system/photos/2019/06/24/1113113/original.jpg	https://data.globe.gov/system/photos/2019/06/24/1113115/original.jpg	https://data.globe.gov/system/photos/2019/06/24/1113114/original.jpg	https://data.globe.gov/system/photos/2019/06/24/1113116/original.jpg	https://data.globe.gov/system/photos/2019/06/24/1113118/original.jpg
GV_634	46.1642	-123.5918	16.1		https://www.google.com/maps/search/46.1642,-123.5918	https://data.globe.gov/system/photos/2019/06/24/1113119/original.jpg	https://data.globe.gov/system/photos/2019/06/24/1113121/original.jpg	https://data.globe.gov/system/photos/2019/06/24/1113120/original.jpg	https://data.globe.gov/system/photos/2019/06/24/1113122/original.jpg	https://data.globe.gov/system/photos/2019/06/24/1113124/original.jpg
GV_635	46.1636	-123.5917	19.9		https://www.google.com/maps/search/46.1636,-123.5917	https://data.globe.gov/system/photos/2019/06/24/1113125/original.jpg	https://data.globe.gov/system/photos/2019/06/24/1113127/original.jpg	https://data.globe.gov/system/photos/2019/06/24/1113126/original.jpg	https://data.globe.gov/system/photos/2019/06/24/1113128/original.jpg	https://data.globe.gov/system/photos/2019/06/24/1113130/original.jpg
GV_636	46.165	-123.593	11.6		https://www.google.com/maps/search/46.165,-123.593	https://data.globe.gov/system/photos/2019/06/24/1113131/original.jpg	https://data.globe.gov/system/photos/2019/06/24/1113133/original.jpg	https://data.globe.gov/system/photos/2019/06/24/1113132/original.jpg	https://data.globe.gov/system/photos/2019/06/24/1113134/original.jpg	https://data.globe.gov/system/photos/2019/06/24/1113136/original.jpg
GV_637	46.1645	-123.5929	14.2		https://www.google.com/maps/search/46.1645,-123.5929	https://data.globe.gov/system/photos/2019/06/24/1113137/original.jpg	https://data.globe.gov/system/photos/2019/06/24/1113139/original.jpg	https://data.globe.gov/system/photos/2019/06/24/1113138/original.jpg	https://data.globe.gov/system/photos/2019/06/24/1113140/original.jpg	https://data.globe.gov/system/photos/2019/06/24/1113142/original.jpg
GV_638	46.1645	-123.5932	11		https://www.google.com/maps/search/46.1645,-123.5932	https://data.globe.gov/system/photos/2019/06/24/1113143/original.jpg	https://data.globe.gov/system/photos/2019/06/24/1113145/original.jpg	https://data.globe.gov/system/photos/2019/06/24/1113144/original.jpg	https://data.globe.gov/system/photos/2019/06/24/1113146/original.jpg	https://data.globe.gov/system/photos/2019/06/24/1113148/original.jpg
GV_639	46.1636	-123.5927	15.8		https://www.google.com/maps/search/46.1636,-123.5927	https://data.globe.gov/system/photos/2019/06/24/1113149/original.jpg	https://data.globe.gov/system/photos/2019/06/24/1113151/original.jpg	https://data.globe.gov/system/photos/2019/06/24/1113150/original.jpg	https://data.globe.gov/system/photos/2019/06/24/1113152/original.jpg	https://data.globe.gov/system/photos/2019/06/24/1113154/original.jpg
GV_640	46.163	-123.5926	16.9		https://www.google.com/maps/search/46.163,-123.5926	https://data.globe.gov/system/photos/2019/06/24/1113155/original.jpg	https://data.globe.gov/system/photos/2019/06/24/1113157/original.jpg	https://data.globe.gov/system/photos/2019/06/24/1113156/original.jpg	https://data.globe.gov/system/photos/2019/06/24/1113158/original.jpg	https://data.globe.gov/system/photos/2019/06/24/1113160/original.jpg
GV_641	46.165	-123.5919	13.3		https://www.google.com/maps/search/46.165,-123.5919	https://data.globe.gov/system/photos/2019/06/24/1113161/original.jpg	https://data.globe.gov/system/photos/2019/06/24/1113163/original.jpg	https://data.globe.gov/system/photos/2019/06/24/1113162/original.jpg	https://data.globe.gov/system/photos/2019/06/24/1113164/original.jpg	https://data.globe.gov/system/photos/2019/06/24/1113166/original.jpg
GV_642	15.178	104.8743	136.7		https://www.google.com/maps/search/15.178,104.8743	https://data.globe.gov/system/photos/2019/06/24/1113271/original.jpg	https://data.globe.gov/system/photos/2019/06/24/1113275/original.jpg	https://data.globe.gov/system/photos/2019/06/24/1113272/original.jpg	https://data.globe.gov/system/photos/2019/06/24/1113277/original.jpg	https://data.globe.gov/system/photos/2019/06/24/1113281/original.jpg
GV_643	15.1778	104.8737	136.8		https://www.google.com/maps/search/15.1778,104.8737	https://data.globe.gov/system/photos/2019/06/24/1113273/original.jpg	https://data.globe.gov/system/photos/2019/06/24/1113276/original.jpg	https://data.globe.gov/system/photos/2019/06/24/1113274/original.jpg	https://data.globe.gov/system/photos/2019/06/24/1113279/original.jpg	https://data.globe.gov/system/photos/2019/06/24/1113282/original.jpg
GV_644	15.1783	104.8735	137		https://www.google.com/maps/search/15.1783,104.8735	https://data.globe.gov/system/photos/2019/06/24/1113288/original.jpg	https://data.globe.gov/system/photos/2019/06/24/1113291/original.jpg	https://data.globe.gov/system/photos/2019/06/24/1113289/original.jpg	https://data.globe.gov/system/photos/2019/06/24/1113292/original.jpg	https://data.globe.gov/system/photos/2019/06/24/1113295/original.jpg
GV_645	53.4125	-0.6469	22.9		https://www.google.com/maps/search/53.4125,-0.6469	https://data.globe.gov/system/photos/2019/06/24/1113340/original.jpg	https://data.globe.gov/system/photos/2019/06/24/1113342/original.jpg	https://data.globe.gov/system/photos/2019/06/24/1113341/original.jpg	https://data.globe.gov/system/photos/2019/06/24/1113343/original.jpg	https://data.globe.gov/system/photos/2019/06/24/1113345/original.jpg
GV_646	44.3542	-100.3619	435.6		https://www.google.com/maps/search/44.3542,-100.3619	https://data.globe.gov/system/photos/2019/06/24/1113656/original.jpg	https://data.globe.gov/system/photos/2019/06/24/1113660/original.jpg	https://data.globe.gov/system/photos/2019/06/24/1113658/original.jpg	https://data.globe.gov/system/photos/2019/06/24/1113662/original.jpg	https://data.globe.gov/system/photos/2019/06/24/1113665/original.jpg
GV_647	35.334	-97.5504	369.6		https://www.google.com/maps/search/35.334,-97.5504	https://data.globe.gov/system/photos/2019/06/24/1113666/original.jpg	https://data.globe.gov/system/photos/2019/06/24/1113668/original.jpg	https://data.globe.gov/system/photos/2019/06/24/1113667/original.jpg	https://data.globe.gov/system/photos/2019/06/24/1113669/original.jpg	https://data.globe.gov/system/photos/2019/06/24/1113671/original.jpg
GV_648	39.2643	-120.6567	1461.6		https://www.google.com/maps/search/39.2643,-120.6567	https://data.globe.gov/system/photos/2019/06/23/1113792/original.jpg	https://data.globe.gov/system/photos/2019/06/23/1113794/original.jpg	https://data.globe.gov/system/photos/2019/06/23/1113793/original.jpg	https://data.globe.gov/system/photos/2019/06/23/1113795/original.jpg	https://data.globe.gov/system/photos/2019/06/23/1113797/original.jpg
GV_649	44.6245	-123.0826	68.6		https://www.google.com/maps/search/44.6245,-123.0826	https://data.globe.gov/system/photos/2019/06/24/1113814/original.jpg	https://data.globe.gov/system/photos/2019/06/24/1113817/original.jpg	https://data.globe.gov/system/photos/2019/06/24/1113816/original.jpg	https://data.globe.gov/system/photos/2019/06/24/1113818/original.jpg	https://data.globe.gov/system/photos/2019/06/24/1113820/original.jpg
GV_650	24.4359	118.3143	21.5		https://www.google.com/maps/search/24.4359,118.3143	https://data.globe.gov/system/photos/2019/06/25/1114150/original.jpg	https://data.globe.gov/system/photos/2019/06/25/1114152/original.jpg	https://data.globe.gov/system/photos/2019/06/25/1114151/original.jpg	https://data.globe.gov/system/photos/2019/06/25/1114153/original.jpg	https://data.globe.gov/system/photos/2019/06/25/1114155/original.jpg
GV_651	13.7839	100.534	11		https://www.google.com/maps/search/13.7839,100.534	https://data.globe.gov/system/photos/2019/06/25/1114887/original.jpg	https://data.globe.gov/system/photos/2019/06/25/1114889/original.jpg	https://data.globe.gov/system/photos/2019/06/25/1114888/original.jpg	https://data.globe.gov/system/photos/2019/06/25/1114890/original.jpg	https://data.globe.gov/system/photos/2019/06/25/1114892/original.jpg
GV_652	15.2782	104.8353	127		https://www.google.com/maps/search/15.2782,104.8353	https://data.globe.gov/system/photos/2019/06/25/1114941/original.jpg	https://data.globe.gov/system/photos/2019/06/25/1114943/original.jpg	https://data.globe.gov/system/photos/2019/06/25/1114942/original.jpg	https://data.globe.gov/system/photos/2019/06/25/1114944/original.jpg	https://data.globe.gov/system/photos/2019/06/25/1114946/original.jpg
GV_653	46.8675	-113.9878	972.2		https://www.google.com/maps/search/46.8675,-113.9878	https://data.globe.gov/system/photos/2019/06/22/1115016/original.jpg	https://data.globe.gov/system/photos/2019/06/22/1115018/original.jpg	https://data.globe.gov/system/photos/2019/06/22/1115017/original.jpg	https://data.globe.gov/system/photos/2019/06/22/1115019/original.jpg	https://data.globe.gov/system/photos/2019/06/22/1115021/original.jpg
GV_654	39.5655	-76.3728	137.8		https://www.google.com/maps/search/39.5655,-76.3728	https://data.globe.gov/system/photos/2019/06/25/1115102/original.jpg	https://data.globe.gov/system/photos/2019/06/25/1115104/original.jpg	https://data.globe.gov/system/photos/2019/06/25/1115103/original.jpg	https://data.globe.gov/system/photos/2019/06/25/1115105/original.jpg	https://data.globe.gov/system/photos/2019/06/25/1115107/original.jpg
GV_655	42.4907	-96.4158	332.9		https://www.google.com/maps/search/42.4907,-96.4158	https://data.globe.gov/system/photos/2019/06/25/1115114/original.jpg	https://data.globe.gov/system/photos/2019/06/25/1115116/original.jpg	https://data.globe.gov/system/photos/2019/06/25/1115115/original.jpg	https://data.globe.gov/system/photos/2019/06/25/1115117/original.jpg	https://data.globe.gov/system/photos/2019/06/25/1115119/original.jpg
GV_656	42.491	-96.4184	334		https://www.google.com/maps/search/42.491,-96.4184	https://data.globe.gov/system/photos/2019/06/25/1115126/original.jpg	https://data.globe.gov/system/photos/2019/06/25/1115128/original.jpg	https://data.globe.gov/system/photos/2019/06/25/1115127/original.jpg	https://data.globe.gov/system/photos/2019/06/25/1115129/original.jpg	https://data.globe.gov/system/photos/2019/06/25/1115131/original.jpg
GV_657	45.6698	-121.8942	28.8		https://www.google.com/maps/search/45.6698,-121.8942	https://data.globe.gov/system/photos/2019/06/25/1115184/original.jpg	https://data.globe.gov/system/photos/2019/06/25/1115186/original.jpg	https://data.globe.gov/system/photos/2019/06/25/1115185/original.jpg	https://data.globe.gov/system/photos/2019/06/25/1115188/original.jpg	https://data.globe.gov/system/photos/2019/06/25/1115194/original.jpg
GV_658	45.6695	-121.8942	28.8		https://www.google.com/maps/search/45.6695,-121.8942	https://data.globe.gov/system/photos/2019/06/25/1115203/original.jpg	https://data.globe.gov/system/photos/2019/06/25/1115205/original.jpg	https://data.globe.gov/system/photos/2019/06/25/1115204/original.jpg	https://data.globe.gov/system/photos/2019/06/25/1115206/original.jpg	https://data.globe.gov/system/photos/2019/06/25/1115208/original.jpg
GV_659	45.4872	15.5308	156.1		https://www.google.com/maps/search/45.4872,15.5308	https://data.globe.gov/system/photos/2019/06/25/1115227/original.jpg	https://data.globe.gov/system/photos/2019/06/25/1115229/original.jpg	https://data.globe.gov/system/photos/2019/06/25/1115228/original.jpg	https://data.globe.gov/system/photos/2019/06/25/1115230/original.jpg	https://data.globe.gov/system/photos/2019/06/25/1115232/original.jpg
GV_660	43.5841	-110.748	1942.1		https://www.google.com/maps/search/43.5841,-110.748	https://data.globe.gov/system/photos/2019/06/24/1115243/original.jpg	https://data.globe.gov/system/photos/2019/06/24/1115245/original.jpg	https://data.globe.gov/system/photos/2019/06/24/1115244/original.jpg	https://data.globe.gov/system/photos/2019/06/24/1115249/original.jpg	https://data.globe.gov/system/photos/2019/06/24/1115254/original.jpg
GV_661	62.6386	29.3757	100		https://www.google.com/maps/search/62.6386,29.3757	https://data.globe.gov/system/photos/2019/06/02/1115262/original.jpg	https://data.globe.gov/system/photos/2019/06/02/1115264/original.jpg	https://data.globe.gov/system/photos/2019/06/02/1115263/original.jpg	https://data.globe.gov/system/photos/2019/06/02/1115265/original.jpg	https://data.globe.gov/system/photos/2019/06/02/1115267/original.jpg
GV_662	-3.765	-73.2689	98		https://www.google.com/maps/search/-3.765,-73.2689	https://data.globe.gov/system/photos/2019/06/25/1115268/original.jpg	https://data.globe.gov/system/photos/2019/06/25/1115270/original.jpg	https://data.globe.gov/system/photos/2019/06/25/1115269/original.jpg	https://data.globe.gov/system/photos/2019/06/25/1115271/original.jpg	https://data.globe.gov/system/photos/2019/06/25/1115273/original.jpg
GV_663	40.0342	-105.2416	1608.8		https://www.google.com/maps/search/40.0342,-105.2416	https://data.globe.gov/system/photos/2019/06/25/1115292/original.jpg	https://data.globe.gov/system/photos/2019/06/25/1115300/original.jpg	https://data.globe.gov/system/photos/2019/06/25/1115299/original.jpg	https://data.globe.gov/system/photos/2019/06/25/1115306/original.jpg	https://data.globe.gov/system/photos/2019/06/25/1115308/original.jpg
GV_664	45.6702	-121.8941	28.4		https://www.google.com/maps/search/45.6702,-121.8941	https://data.globe.gov/system/photos/2019/06/25/1115428/original.jpg	https://data.globe.gov/system/photos/2019/06/25/1115430/original.jpg	https://data.globe.gov/system/photos/2019/06/25/1115429/original.jpg	https://data.globe.gov/system/photos/2019/06/25/1115431/original.jpg	https://data.globe.gov/system/photos/2019/06/25/1115433/original.jpg
GV_665	45.6703	-121.8936	28.7		https://www.google.com/maps/search/45.6703,-121.8936	https://data.globe.gov/system/photos/2019/06/25/1115435/original.jpg	https://data.globe.gov/system/photos/2019/06/25/1115437/original.jpg	https://data.globe.gov/system/photos/2019/06/25/1115436/original.jpg	https://data.globe.gov/system/photos/2019/06/25/1115438/original.jpg	https://data.globe.gov/system/photos/2019/06/25/1115440/original.jpg
GV_666	33.1586	-96.8108	217.2		https://www.google.com/maps/search/33.1586,-96.8108	https://data.globe.gov/system/photos/2019/06/25/1115572/original.jpg	https://data.globe.gov/system/photos/2019/06/25/1115574/original.jpg	https://data.globe.gov/system/photos/2019/06/25/1115573/original.jpg	https://data.globe.gov/system/photos/2019/06/25/1115575/original.jpg	https://data.globe.gov/system/photos/2019/06/25/1115577/original.jpg
GV_667	45.671	-121.8953	30.2		https://www.google.com/maps/search/45.671,-121.8953	https://data.globe.gov/system/photos/2019/06/25/1115791/original.jpg	https://data.globe.gov/system/photos/2019/06/25/1115793/original.jpg	https://data.globe.gov/system/photos/2019/06/25/1115792/original.jpg	https://data.globe.gov/system/photos/2019/06/25/1115794/original.jpg	https://data.globe.gov/system/photos/2019/06/25/1115796/original.jpg
GV_668	45.6691	-121.8949	28.9		https://www.google.com/maps/search/45.6691,-121.8949	https://data.globe.gov/system/photos/2019/06/26/1115803/original.jpg	https://data.globe.gov/system/photos/2019/06/26/1115805/original.jpg	https://data.globe.gov/system/photos/2019/06/26/1115804/original.jpg	https://data.globe.gov/system/photos/2019/06/26/1115806/original.jpg	https://data.globe.gov/system/photos/2019/06/26/1115808/original.jpg
GV_669	45.6705	-121.8956	30.3		https://www.google.com/maps/search/45.6705,-121.8956	https://data.globe.gov/system/photos/2019/06/26/1115809/original.jpg	https://data.globe.gov/system/photos/2019/06/26/1115811/original.jpg	https://data.globe.gov/system/photos/2019/06/26/1115810/original.jpg	https://data.globe.gov/system/photos/2019/06/26/1115812/original.jpg	https://data.globe.gov/system/photos/2019/06/26/1115814/original.jpg
GV_670	45.6705	-121.8957	30.3		https://www.google.com/maps/search/45.6705,-121.8957	https://data.globe.gov/system/photos/2019/06/26/1115815/original.jpg	https://data.globe.gov/system/photos/2019/06/26/1115817/original.jpg	https://data.globe.gov/system/photos/2019/06/26/1115816/original.jpg	https://data.globe.gov/system/photos/2019/06/26/1115818/original.jpg	https://data.globe.gov/system/photos/2019/06/26/1115820/original.jpg
GV_671	45.6702	-121.8961	30.2		https://www.google.com/maps/search/45.6702,-121.8961	https://data.globe.gov/system/photos/2019/06/26/1115821/original.jpg	https://data.globe.gov/system/photos/2019/06/26/1115823/original.jpg	https://data.globe.gov/system/photos/2019/06/26/1115822/original.jpg	https://data.globe.gov/system/photos/2019/06/26/1115824/original.jpg	https://data.globe.gov/system/photos/2019/06/26/1115826/original.jpg
GV_672	45.6696	-121.8961	30.1		https://www.google.com/maps/search/45.6696,-121.8961	https://data.globe.gov/system/photos/2019/06/26/1115828/original.jpg	https://data.globe.gov/system/photos/2019/06/26/1115830/original.jpg	https://data.globe.gov/system/photos/2019/06/26/1115829/original.jpg	https://data.globe.gov/system/photos/2019/06/26/1115831/original.jpg	https://data.globe.gov/system/photos/2019/06/26/1115833/original.jpg
GV_673	45.6695	-121.8966	30.8		https://www.google.com/maps/search/45.6695,-121.8966	https://data.globe.gov/system/photos/2019/06/26/1115834/original.jpg	https://data.globe.gov/system/photos/2019/06/26/1115836/original.jpg	https://data.globe.gov/system/photos/2019/06/26/1115835/original.jpg	https://data.globe.gov/system/photos/2019/06/26/1115837/original.jpg	https://data.globe.gov/system/photos/2019/06/26/1115839/original.jpg
GV_674	45.6685	-121.8966	26		https://www.google.com/maps/search/45.6685,-121.8966	https://data.globe.gov/system/photos/2019/06/26/1115840/original.jpg	https://data.globe.gov/system/photos/2019/06/26/1115842/original.jpg	https://data.globe.gov/system/photos/2019/06/26/1115841/original.jpg	https://data.globe.gov/system/photos/2019/06/26/1115843/original.jpg	https://data.globe.gov/system/photos/2019/06/26/1115845/original.jpg
GV_675	45.6688	-121.8977	27		https://www.google.com/maps/search/45.6688,-121.8977	https://data.globe.gov/system/photos/2019/06/26/1115846/original.jpg	https://data.globe.gov/system/photos/2019/06/26/1115848/original.jpg	https://data.globe.gov/system/photos/2019/06/26/1115847/original.jpg	https://data.globe.gov/system/photos/2019/06/26/1115849/original.jpg	https://data.globe.gov/system/photos/2019/06/26/1115851/original.jpg
GV_676	45.6681	-121.8981	27.2		https://www.google.com/maps/search/45.6681,-121.8981	https://data.globe.gov/system/photos/2019/06/26/1115852/original.jpg	https://data.globe.gov/system/photos/2019/06/26/1115854/original.jpg	https://data.globe.gov/system/photos/2019/06/26/1115853/original.jpg	https://data.globe.gov/system/photos/2019/06/26/1115855/original.jpg	https://data.globe.gov/system/photos/2019/06/26/1115857/original.jpg
GV_677	45.6676	-121.8982	27.9		https://www.google.com/maps/search/45.6676,-121.8982	https://data.globe.gov/system/photos/2019/06/26/1115858/original.jpg	https://data.globe.gov/system/photos/2019/06/26/1115860/original.jpg	https://data.globe.gov/system/photos/2019/06/26/1115859/original.jpg	https://data.globe.gov/system/photos/2019/06/26/1115861/original.jpg	https://data.globe.gov/system/photos/2019/06/26/1115863/original.jpg
GV_678	45.6677	-121.8977	29.8		https://www.google.com/maps/search/45.6677,-121.8977	https://data.globe.gov/system/photos/2019/06/26/1115864/original.jpg	https://data.globe.gov/system/photos/2019/06/26/1115866/original.jpg	https://data.globe.gov/system/photos/2019/06/26/1115865/original.jpg	https://data.globe.gov/system/photos/2019/06/26/1115867/original.jpg	https://data.globe.gov/system/photos/2019/06/26/1115869/original.jpg
GV_679	45.669	-121.8946	28.9		https://www.google.com/maps/search/45.669,-121.8946	https://data.globe.gov/system/photos/2019/06/26/1115871/original.jpg	https://data.globe.gov/system/photos/2019/06/26/1115873/original.jpg	https://data.globe.gov/system/photos/2019/06/26/1115872/original.jpg	https://data.globe.gov/system/photos/2019/06/26/1115874/original.jpg	https://data.globe.gov/system/photos/2019/06/26/1115876/original.jpg
GV_680	45.6684	-121.8971	29.9		https://www.google.com/maps/search/45.6684,-121.8971	https://data.globe.gov/system/photos/2019/06/26/1115877/original.jpg	https://data.globe.gov/system/photos/2019/06/26/1115879/original.jpg	https://data.globe.gov/system/photos/2019/06/26/1115878/original.jpg	https://data.globe.gov/system/photos/2019/06/26/1115880/original.jpg	https://data.globe.gov/system/photos/2019/06/26/1115882/original.jpg
GV_681	46.4076	-117.0805	280.9		https://www.google.com/maps/search/46.4076,-117.0805	https://data.globe.gov/system/photos/2019/06/24/1115908/original.jpg	https://data.globe.gov/system/photos/2019/06/24/1115910/original.jpg	https://data.globe.gov/system/photos/2019/06/24/1115909/original.jpg	https://data.globe.gov/system/photos/2019/06/24/1115911/original.jpg	https://data.globe.gov/system/photos/2019/06/24/1115913/original.jpg
GV_682	46.2008	-116.4152	1091.7		https://www.google.com/maps/search/46.2008,-116.4152	https://data.globe.gov/system/photos/2019/06/25/1115914/original.jpg	https://data.globe.gov/system/photos/2019/06/25/1115916/original.jpg	https://data.globe.gov/system/photos/2019/06/25/1115915/original.jpg	https://data.globe.gov/system/photos/2019/06/25/1115917/original.jpg	https://data.globe.gov/system/photos/2019/06/25/1115919/original.jpg
GV_683	46.3345	-116.1481	331.6		https://www.google.com/maps/search/46.3345,-116.1481	https://data.globe.gov/system/photos/2019/06/25/1115921/original.jpg	https://data.globe.gov/system/photos/2019/06/25/1115923/original.jpg	https://data.globe.gov/system/photos/2019/06/25/1115922/original.jpg	https://data.globe.gov/system/photos/2019/06/25/1115924/original.jpg	https://data.globe.gov/system/photos/2019/06/25/1115926/original.jpg
GV_684	46.3549	-116.1635	333.4		https://www.google.com/maps/search/46.3549,-116.1635	https://data.globe.gov/system/photos/2019/06/25/1115927/original.jpg	https://data.globe.gov/system/photos/2019/06/25/1115929/original.jpg	https://data.globe.gov/system/photos/2019/06/25/1115928/original.jpg	https://data.globe.gov/system/photos/2019/06/25/1115930/original.jpg	https://data.globe.gov/system/photos/2019/06/25/1115932/original.jpg
GV_685	46.3791	-116.1745	385.9		https://www.google.com/maps/search/46.3791,-116.1745	https://data.globe.gov/system/photos/2019/06/25/1115933/original.jpg	https://data.globe.gov/system/photos/2019/06/25/1115935/original.jpg	https://data.globe.gov/system/photos/2019/06/25/1115934/original.jpg	https://data.globe.gov/system/photos/2019/06/25/1115937/original.jpg	https://data.globe.gov/system/photos/2019/06/25/1115939/original.jpg
GV_686	46.387	-116.0471	944		https://www.google.com/maps/search/46.387,-116.0471	https://data.globe.gov/system/photos/2019/06/25/1115940/original.jpg	https://data.globe.gov/system/photos/2019/06/25/1115942/original.jpg	https://data.globe.gov/system/photos/2019/06/25/1115941/original.jpg	https://data.globe.gov/system/photos/2019/06/25/1115943/original.jpg	https://data.globe.gov/system/photos/2019/06/25/1115946/original.jpg
GV_687	46.3758	-115.9516	922.7		https://www.google.com/maps/search/46.3758,-115.9516	https://data.globe.gov/system/photos/2019/06/25/1115947/original.jpg	https://data.globe.gov/system/photos/2019/06/25/1115949/original.jpg	https://data.globe.gov/system/photos/2019/06/25/1115948/original.jpg	https://data.globe.gov/system/photos/2019/06/25/1115951/original.jpg	https://data.globe.gov/system/photos/2019/06/25/1115953/original.jpg
GV_688	46.3757	-115.9424	918.9		https://www.google.com/maps/search/46.3757,-115.9424	https://data.globe.gov/system/photos/2019/06/25/1115954/original.jpg	https://data.globe.gov/system/photos/2019/06/25/1115956/original.jpg	https://data.globe.gov/system/photos/2019/06/25/1115955/original.jpg	https://data.globe.gov/system/photos/2019/06/25/1115957/original.jpg	https://data.globe.gov/system/photos/2019/06/25/1115960/original.jpg
GV_689	46.3501	-115.9253	916.7		https://www.google.com/maps/search/46.3501,-115.9253	https://data.globe.gov/system/photos/2019/06/25/1115961/original.jpg	https://data.globe.gov/system/photos/2019/06/25/1115963/original.jpg	https://data.globe.gov/system/photos/2019/06/25/1115962/original.jpg	https://data.globe.gov/system/photos/2019/06/25/1115964/original.jpg	https://data.globe.gov/system/photos/2019/06/25/1115966/original.jpg
GV_690	24.4356	118.3145	22		https://www.google.com/maps/search/24.4356,118.3145	https://data.globe.gov/system/photos/2019/06/26/1116209/original.jpg	https://data.globe.gov/system/photos/2019/06/26/1116211/original.jpg	https://data.globe.gov/system/photos/2019/06/26/1116210/original.jpg	https://data.globe.gov/system/photos/2019/06/26/1116212/original.jpg	https://data.globe.gov/system/photos/2019/06/26/1116214/original.jpg
GV_691	24.4317	118.3137	11.2		https://www.google.com/maps/search/24.4317,118.3137	https://data.globe.gov/system/photos/2019/06/26/1116215/original.jpg	https://data.globe.gov/system/photos/2019/06/26/1116217/original.jpg	https://data.globe.gov/system/photos/2019/06/26/1116216/original.jpg	https://data.globe.gov/system/photos/2019/06/26/1116218/original.jpg	https://data.globe.gov/system/photos/2019/06/26/1116220/original.jpg
GV_692	24.4339	118.3113	19.2		https://www.google.com/maps/search/24.4339,118.3113	https://data.globe.gov/system/photos/2019/06/26/1116221/original.jpg	https://data.globe.gov/system/photos/2019/06/26/1116223/original.jpg	https://data.globe.gov/system/photos/2019/06/26/1116222/original.jpg	https://data.globe.gov/system/photos/2019/06/26/1116224/original.jpg	https://data.globe.gov/system/photos/2019/06/26/1116226/original.jpg
GV_693	45.6695	-121.8942	28.8		https://www.google.com/maps/search/45.6695,-121.8942	https://data.globe.gov/system/photos/2019/06/26/1116258/original.jpg	https://data.globe.gov/system/photos/2019/06/26/1116260/original.jpg	https://data.globe.gov/system/photos/2019/06/26/1116259/original.jpg	https://data.globe.gov/system/photos/2019/06/26/1116261/original.jpg	https://data.globe.gov/system/photos/2019/06/26/1116263/original.jpg
GV_694	45.6734	-121.8724	60.5		https://www.google.com/maps/search/45.6734,-121.8724	https://data.globe.gov/system/photos/2019/06/26/1116264/original.jpg	https://data.globe.gov/system/photos/2019/06/26/1116266/original.jpg	https://data.globe.gov/system/photos/2019/06/26/1116265/original.jpg	https://data.globe.gov/system/photos/2019/06/26/1116267/original.jpg	https://data.globe.gov/system/photos/2019/06/26/1116269/original.jpg
GV_695	45.6828	-121.8426	67.4		https://www.google.com/maps/search/45.6828,-121.8426	https://data.globe.gov/system/photos/2019/06/26/1116270/original.jpg	https://data.globe.gov/system/photos/2019/06/26/1116272/original.jpg	https://data.globe.gov/system/photos/2019/06/26/1116271/original.jpg	https://data.globe.gov/system/photos/2019/06/26/1116273/original.jpg	https://data.globe.gov/system/photos/2019/06/26/1116275/original.jpg
GV_696	45.6824	-121.8425	69.6		https://www.google.com/maps/search/45.6824,-121.8425	https://data.globe.gov/system/photos/2019/06/26/1116276/original.jpg	https://data.globe.gov/system/photos/2019/06/26/1116278/original.jpg	https://data.globe.gov/system/photos/2019/06/26/1116277/original.jpg	https://data.globe.gov/system/photos/2019/06/26/1116279/original.jpg	https://data.globe.gov/system/photos/2019/06/26/1116281/original.jpg
GV_697	45.6815	-121.8433	90.2		https://www.google.com/maps/search/45.6815,-121.8433	https://data.globe.gov/system/photos/2019/06/26/1116282/original.jpg	https://data.globe.gov/system/photos/2019/06/26/1116284/original.jpg	https://data.globe.gov/system/photos/2019/06/26/1116283/original.jpg	https://data.globe.gov/system/photos/2019/06/26/1116285/original.jpg	https://data.globe.gov/system/photos/2019/06/26/1116287/original.jpg
GV_698	45.6813	-121.8439	92.4		https://www.google.com/maps/search/45.6813,-121.8439	https://data.globe.gov/system/photos/2019/06/26/1116288/original.jpg	https://data.globe.gov/system/photos/2019/06/26/1116290/original.jpg	https://data.globe.gov/system/photos/2019/06/26/1116289/original.jpg	https://data.globe.gov/system/photos/2019/06/26/1116291/original.jpg	https://data.globe.gov/system/photos/2019/06/26/1116293/original.jpg
GV_699	45.681	-121.8426	127.5		https://www.google.com/maps/search/45.681,-121.8426	https://data.globe.gov/system/photos/2019/06/26/1116294/original.jpg	https://data.globe.gov/system/photos/2019/06/26/1116296/original.jpg	https://data.globe.gov/system/photos/2019/06/26/1116295/original.jpg	https://data.globe.gov/system/photos/2019/06/26/1116297/original.jpg	https://data.globe.gov/system/photos/2019/06/26/1116299/original.jpg
GV_700	45.6842	-121.8403	70.3		https://www.google.com/maps/search/45.6842,-121.8403	https://data.globe.gov/system/photos/2019/06/26/1116300/original.jpg	https://data.globe.gov/system/photos/2019/06/26/1116302/original.jpg	https://data.globe.gov/system/photos/2019/06/26/1116301/original.jpg	https://data.globe.gov/system/photos/2019/06/26/1116303/original.jpg	https://data.globe.gov/system/photos/2019/06/26/1116305/original.jpg
GV_701	45.893	-111.5525	1241.2		https://www.google.com/maps/search/45.893,-111.5525	https://data.globe.gov/system/photos/2019/06/25/1116373/original.jpg	https://data.globe.gov/system/photos/2019/06/25/1116375/original.jpg	https://data.globe.gov/system/photos/2019/06/25/1116374/original.jpg	https://data.globe.gov/system/photos/2019/06/25/1116376/original.jpg	https://data.globe.gov/system/photos/2019/06/25/1116378/original.jpg
GV_702	46.3564	-111.545	1170.9		https://www.google.com/maps/search/46.3564,-111.545	https://data.globe.gov/system/photos/2019/06/25/1116379/original.jpg	https://data.globe.gov/system/photos/2019/06/25/1116384/original.jpg	https://data.globe.gov/system/photos/2019/06/25/1116381/original.jpg	https://data.globe.gov/system/photos/2019/06/25/1116387/original.jpg	https://data.globe.gov/system/photos/2019/06/25/1116390/original.jpg
GV_703	46.6542	-111.7167	1159.9		https://www.google.com/maps/search/46.6542,-111.7167	https://data.globe.gov/system/photos/2019/06/25/1116392/original.jpg	https://data.globe.gov/system/photos/2019/06/25/1116394/original.jpg	https://data.globe.gov/system/photos/2019/06/25/1116393/original.jpg	https://data.globe.gov/system/photos/2019/06/25/1116395/original.jpg	https://data.globe.gov/system/photos/2019/06/25/1116397/original.jpg
GV_704	46.5863	-112.0164	1250.5		https://www.google.com/maps/search/46.5863,-112.0164	https://data.globe.gov/system/photos/2019/06/25/1116398/original.jpg	https://data.globe.gov/system/photos/2019/06/25/1116400/original.jpg	https://data.globe.gov/system/photos/2019/06/25/1116399/original.jpg	https://data.globe.gov/system/photos/2019/06/25/1116401/original.jpg	https://data.globe.gov/system/photos/2019/06/25/1116403/original.jpg
GV_705	46.5861	-112.0191	1257.1		https://www.google.com/maps/search/46.5861,-112.0191	https://data.globe.gov/system/photos/2019/06/25/1116404/original.jpg	https://data.globe.gov/system/photos/2019/06/25/1116406/original.jpg	https://data.globe.gov/system/photos/2019/06/25/1116405/original.jpg	https://data.globe.gov/system/photos/2019/06/25/1116407/original.jpg	https://data.globe.gov/system/photos/2019/06/25/1116409/original.jpg
GV_706	46.5919	-112.0425	1252.6		https://www.google.com/maps/search/46.5919,-112.0425	https://data.globe.gov/system/photos/2019/06/26/1116460/original.jpg	https://data.globe.gov/system/photos/2019/06/26/1116462/original.jpg	https://data.globe.gov/system/photos/2019/06/26/1116461/original.jpg	https://data.globe.gov/system/photos/2019/06/26/1116463/original.jpg	https://data.globe.gov/system/photos/2019/06/26/1116465/original.jpg
GV_707	46.2667	-111.4944	1171.5		https://www.google.com/maps/search/46.2667,-111.4944	https://data.globe.gov/system/photos/2019/06/26/1116742/original.jpg	https://data.globe.gov/system/photos/2019/06/26/1116744/original.jpg	https://data.globe.gov/system/photos/2019/06/26/1116743/original.jpg	https://data.globe.gov/system/photos/2019/06/26/1116745/original.jpg	https://data.globe.gov/system/photos/2019/06/26/1116747/original.jpg
GV_708	45.9202	-111.4987	1233.3		https://www.google.com/maps/search/45.9202,-111.4987	https://data.globe.gov/system/photos/2019/06/26/1116748/original.jpg	https://data.globe.gov/system/photos/2019/06/26/1116750/original.jpg	https://data.globe.gov/system/photos/2019/06/26/1116749/original.jpg	https://data.globe.gov/system/photos/2019/06/26/1116751/original.jpg	https://data.globe.gov/system/photos/2019/06/26/1116753/original.jpg
GV_709	45.926	-111.503	1233.4		https://www.google.com/maps/search/45.926,-111.503	https://data.globe.gov/system/photos/2019/06/26/1116754/original.jpg	https://data.globe.gov/system/photos/2019/06/26/1116756/original.jpg	https://data.globe.gov/system/photos/2019/06/26/1116755/original.jpg	https://data.globe.gov/system/photos/2019/06/26/1116757/original.jpg	https://data.globe.gov/system/photos/2019/06/26/1116759/original.jpg
GV_710	45.9284	-111.5005	1231.8		https://www.google.com/maps/search/45.9284,-111.5005	https://data.globe.gov/system/photos/2019/06/26/1116760/original.jpg	https://data.globe.gov/system/photos/2019/06/26/1116762/original.jpg	https://data.globe.gov/system/photos/2019/06/26/1116761/original.jpg	https://data.globe.gov/system/photos/2019/06/26/1116763/original.jpg	https://data.globe.gov/system/photos/2019/06/26/1116765/original.jpg
GV_711	35.3101	-106.5767	1550.6		https://www.google.com/maps/search/35.3101,-106.5767	https://data.globe.gov/system/photos/2019/06/26/1116858/original.jpg	https://data.globe.gov/system/photos/2019/06/26/1116862/original.jpg	https://data.globe.gov/system/photos/2019/06/26/1116860/original.jpg	https://data.globe.gov/system/photos/2019/06/26/1116863/original.jpg	https://data.globe.gov/system/photos/2019/06/26/1116867/original.jpg
GV_712	46.4356	-117.4239	848.6		https://www.google.com/maps/search/46.4356,-117.4239	https://data.globe.gov/system/photos/2019/06/26/1116910/original.jpg	https://data.globe.gov/system/photos/2019/06/26/1116912/original.jpg	https://data.globe.gov/system/photos/2019/06/26/1116911/original.jpg	https://data.globe.gov/system/photos/2019/06/26/1116913/original.jpg	https://data.globe.gov/system/photos/2019/06/26/1116915/original.jpg
GV_713	46.4247	-117.0336	224.8		https://www.google.com/maps/search/46.4247,-117.0336	https://data.globe.gov/system/photos/2019/06/26/1116918/original.jpg	https://data.globe.gov/system/photos/2019/06/26/1116920/original.jpg	https://data.globe.gov/system/photos/2019/06/26/1116919/original.jpg	https://data.globe.gov/system/photos/2019/06/26/1116921/original.jpg	https://data.globe.gov/system/photos/2019/06/26/1116923/original.jpg

GV_714	46.4243	-117.0337	227.6		https://www.google.com/maps/search/46.4243,-117.0337	https://data.globe.gov/system/photos/2019/06/26/1116924/original.jpg	https://data.globe.gov/system/photos/2019/06/26/1116926/original.jpg	https://data.globe.gov/system/photos/2019/06/26/1116925/original.jpg	https://data.globe.gov/system/photos/2019/06/26/1116927/original.jpg	https://data.globe.gov/system/photos/2019/06/26/1116929/original.jpg
GV_715	46.4243	-117.0336	227.6		https://www.google.com/maps/search/46.4243,-117.0336	https://data.globe.gov/system/photos/2019/06/26/1116930/original.jpg	https://data.globe.gov/system/photos/2019/06/26/1116932/original.jpg	https://data.globe.gov/system/photos/2019/06/26/1116931/original.jpg	https://data.globe.gov/system/photos/2019/06/26/1116933/original.jpg	https://data.globe.gov/system/photos/2019/06/26/1116935/original.jpg
GV_716	38.8539	-77.1527	66.9		https://www.google.com/maps/search/38.8539,-77.1527	https://data.globe.gov/system/photos/2019/06/26/1116949/original.jpg	https://data.globe.gov/system/photos/2019/06/26/1116951/original.jpg	https://data.globe.gov/system/photos/2019/06/26/1116950/original.jpg	https://data.globe.gov/system/photos/2019/06/26/1116952/original.jpg	https://data.globe.gov/system/photos/2019/06/26/1116954/original.jpg
GV_717	46.4239	-117.0337	224		https://www.google.com/maps/search/46.4239,-117.0337	https://data.globe.gov/system/photos/2019/06/26/1116973/original.jpg	https://data.globe.gov/system/photos/2019/06/26/1116975/original.jpg	https://data.globe.gov/system/photos/2019/06/26/1116974/original.jpg	https://data.globe.gov/system/photos/2019/06/26/1116976/original.jpg	https://data.globe.gov/system/photos/2019/06/26/1116978/original.jpg
GV_718	45.9949	-108.0029	877		https://www.google.com/maps/search/45.9949,-108.0029	https://data.globe.gov/system/photos/2019/06/26/1117102/original.jpg	https://data.globe.gov/system/photos/2019/06/26/1117104/original.jpg	https://data.globe.gov/system/photos/2019/06/26/1117103/original.jpg	https://data.globe.gov/system/photos/2019/06/26/1117105/original.jpg	https://data.globe.gov/system/photos/2019/06/26/1117107/original.jpg
GV_719	46.3684	-117.0584	228.6		https://www.google.com/maps/search/46.3684,-117.0584	https://data.globe.gov/system/photos/2019/06/27/1117166/original.jpg	https://data.globe.gov/system/photos/2019/06/27/1117168/original.jpg	https://data.globe.gov/system/photos/2019/06/27/1117167/original.jpg	https://data.globe.gov/system/photos/2019/06/27/1117169/original.jpg	https://data.globe.gov/system/photos/2019/06/27/1117171/original.jpg
GV_720	46.3688	-117.0588	225.1		https://www.google.com/maps/search/46.3688,-117.0588	https://data.globe.gov/system/photos/2019/06/27/1117172/original.jpg	https://data.globe.gov/system/photos/2019/06/27/1117174/original.jpg	https://data.globe.gov/system/photos/2019/06/27/1117173/original.jpg	https://data.globe.gov/system/photos/2019/06/27/1117175/original.jpg	https://data.globe.gov/system/photos/2019/06/27/1117177/original.jpg
GV_721	46.3626	-117.0605	226.6		https://www.google.com/maps/search/46.3626,-117.0605	https://data.globe.gov/system/photos/2019/06/27/1117178/original.jpg	https://data.globe.gov/system/photos/2019/06/27/1117180/original.jpg	https://data.globe.gov/system/photos/2019/06/27/1117179/original.jpg	https://data.globe.gov/system/photos/2019/06/27/1117181/original.jpg	https://data.globe.gov/system/photos/2019/06/27/1117183/original.jpg
GV_722	46.4215	-117.0383	224.8		https://www.google.com/maps/search/46.4215,-117.0383	https://data.globe.gov/system/photos/2019/06/27/1117184/original.jpg	https://data.globe.gov/system/photos/2019/06/27/1117186/original.jpg	https://data.globe.gov/system/photos/2019/06/27/1117185/original.jpg	https://data.globe.gov/system/photos/2019/06/27/1117187/original.jpg	https://data.globe.gov/system/photos/2019/06/27/1117191/original.jpg
GV_723	46.4211	-117.0378	226.1		https://www.google.com/maps/search/46.4211,-117.0378	https://data.globe.gov/system/photos/2019/06/27/1117194/original.jpg	https://data.globe.gov/system/photos/2019/06/27/1117198/original.jpg	https://data.globe.gov/system/photos/2019/06/27/1117196/original.jpg	https://data.globe.gov/system/photos/2019/06/27/1117199/original.jpg	https://data.globe.gov/system/photos/2019/06/27/1117201/original.jpg
GV_724	46.4214	-116.9977	221.4		https://www.google.com/maps/search/46.4214,-116.9977	https://data.globe.gov/system/photos/2019/06/27/1117202/original.jpg	https://data.globe.gov/system/photos/2019/06/27/1117204/original.jpg	https://data.globe.gov/system/photos/2019/06/27/1117203/original.jpg	https://data.globe.gov/system/photos/2019/06/27/1117205/original.jpg	https://data.globe.gov/system/photos/2019/06/27/1117207/original.jpg
GV_725	46.4211	-116.9985	221.2		https://www.google.com/maps/search/46.4211,-116.9985	https://data.globe.gov/system/photos/2019/06/27/1117208/original.jpg	https://data.globe.gov/system/photos/2019/06/27/1117210/original.jpg	https://data.globe.gov/system/photos/2019/06/27/1117209/original.jpg	https://data.globe.gov/system/photos/2019/06/27/1117211/original.jpg	https://data.globe.gov/system/photos/2019/06/27/1117213/original.jpg
GV_726	46.4211	-116.9991	221.2		https://www.google.com/maps/search/46.4211,-116.9991	https://data.globe.gov/system/photos/2019/06/27/1117214/original.jpg	https://data.globe.gov/system/photos/2019/06/27/1117216/original.jpg	https://data.globe.gov/system/photos/2019/06/27/1117215/original.jpg	https://data.globe.gov/system/photos/2019/06/27/1117217/original.jpg	https://data.globe.gov/system/photos/2019/06/27/1117219/original.jpg
GV_727	46.4207	-116.9993	219.8		https://www.google.com/maps/search/46.4207,-116.9993	https://data.globe.gov/system/photos/2019/06/27/1117220/original.jpg	https://data.globe.gov/system/photos/2019/06/27/1117222/original.jpg	https://data.globe.gov/system/photos/2019/06/27/1117221/original.jpg	https://data.globe.gov/system/photos/2019/06/27/1117223/original.jpg	https://data.globe.gov/system/photos/2019/06/27/1117225/original.jpg
GV_728	46.4249	-117.025	227.5		https://www.google.com/maps/search/46.4249,-117.025	https://data.globe.gov/system/photos/2019/06/27/1117228/original.jpg	https://data.globe.gov/system/photos/2019/06/27/1117233/original.jpg	https://data.globe.gov/system/photos/2019/06/27/1117230/original.jpg	https://data.globe.gov/system/photos/2019/06/27/1117235/original.jpg	https://data.globe.gov/system/photos/2019/06/27/1117237/original.jpg
GV_729	46.4249	-117.0239	229		https://www.google.com/maps/search/46.4249,-117.0239	https://data.globe.gov/system/photos/2019/06/27/1117238/original.jpg	https://data.globe.gov/system/photos/2019/06/27/1117240/original.jpg	https://data.globe.gov/system/photos/2019/06/27/1117239/original.jpg	https://data.globe.gov/system/photos/2019/06/27/1117241/original.jpg	https://data.globe.gov/system/photos/2019/06/27/1117243/original.jpg
GV_730	15.2276	104.8557	126.7		https://www.google.com/maps/search/15.2276,104.8557	https://data.globe.gov/system/photos/2019/06/27/1117296/original.jpg	https://data.globe.gov/system/photos/2019/06/27/1117298/original.jpg	https://data.globe.gov/system/photos/2019/06/27/1117297/original.jpg	https://data.globe.gov/system/photos/2019/06/27/1117299/original.jpg	https://data.globe.gov/system/photos/2019/06/27/1117301/original.jpg
GV_731	13.7847	100.5342	6.4		https://www.google.com/maps/search/13.7847,100.5342	https://data.globe.gov/system/photos/2019/06/27/1117354/original.jpg	https://data.globe.gov/system/photos/2019/06/27/1117356/original.jpg	https://data.globe.gov/system/photos/2019/06/27/1117355/original.jpg	https://data.globe.gov/system/photos/2019/06/27/1117357/original.jpg	https://data.globe.gov/system/photos/2019/06/27/1117359/original.jpg
GV_732	13.7844	100.534	7.6		https://www.google.com/maps/search/13.7844,100.534	https://data.globe.gov/system/photos/2019/06/27/1117369/original.jpg	https://data.globe.gov/system/photos/2019/06/27/1117371/original.jpg	https://data.globe.gov/system/photos/2019/06/27/1117370/original.jpg	https://data.globe.gov/system/photos/2019/06/27/1117372/original.jpg	https://data.globe.gov/system/photos/2019/06/27/1117374/original.jpg
GV_733	13.7844	100.5343	8.4		https://www.google.com/maps/search/13.7844,100.5343	https://data.globe.gov/system/photos/2019/06/27/1117375/original.jpg	https://data.globe.gov/system/photos/2019/06/27/1117377/original.jpg	https://data.globe.gov/system/photos/2019/06/27/1117376/original.jpg	https://data.globe.gov/system/photos/2019/06/27/1117378/original.jpg	https://data.globe.gov/system/photos/2019/06/27/1117380/original.jpg
GV_734	43.6121	11.1925	161.3		https://www.google.com/maps/search/43.6121,11.1925	https://data.globe.gov/system/photos/2019/06/18/1117499/original.jpg	https://data.globe.gov/system/photos/2019/06/18/1117501/original.jpg	https://data.globe.gov/system/photos/2019/06/18/1117500/original.jpg	https://data.globe.gov/system/photos/2019/06/18/1117502/original.jpg	https://data.globe.gov/system/photos/2019/06/18/1117505/original.jpg
GV_735	13.7819	100.5395	6.9		https://www.google.com/maps/search/13.7819,100.5395	https://data.globe.gov/system/photos/2019/06/19/1117586/original.jpg	https://data.globe.gov/system/photos/2019/06/19/1117588/original.jpg	https://data.globe.gov/system/photos/2019/06/19/1117587/original.jpg	https://data.globe.gov/system/photos/2019/06/19/1117589/original.jpg	https://data.globe.gov/system/photos/2019/06/19/1117590/original.jpg
GV_736	35.311	-106.5763	1549.7		https://www.google.com/maps/search/35.311,-106.5763	https://data.globe.gov/system/photos/2019/06/27/1117895/original.jpg	https://data.globe.gov/system/photos/2019/06/27/1117897/original.jpg	https://data.globe.gov/system/photos/2019/06/27/1117896/original.jpg	https://data.globe.gov/system/photos/2019/06/27/1117898/original.jpg	https://data.globe.gov/system/photos/2019/06/27/1117900/original.jpg
GV_737	46.3687	-117.0578	229.7		https://www.google.com/maps/search/46.3687,-117.0578	https://data.globe.gov/system/photos/2019/06/27/1117949/original.jpg	https://data.globe.gov/system/photos/2019/06/27/1117951/original.jpg	https://data.globe.gov/system/photos/2019/06/27/1117950/original.jpg	https://data.globe.gov/system/photos/2019/06/27/1117952/original.jpg	https://data.globe.gov/system/photos/2019/06/27/1117955/original.jpg
GV_738	42.0778	-83.2538	178.9		https://www.google.com/maps/search/42.0778,-83.2538	https://data.globe.gov/system/photos/2019/06/27/1117961/original.jpg	https://data.globe.gov/system/photos/2019/06/27/1117963/original.jpg	https://data.globe.gov/system/photos/2019/06/27/1117962/original.jpg	https://data.globe.gov/system/photos/2019/06/27/1117964/original.jpg	https://data.globe.gov/system/photos/2019/06/27/1117966/original.jpg
GV_739	23.83	90.4619	4.1		https://www.google.com/maps/search/23.83,90.4619	https://data.globe.gov/system/photos/2019/06/28/1118490/original.jpg	https://data.globe.gov/system/photos/2019/06/28/1118492/original.jpg	https://data.globe.gov/system/photos/2019/06/28/1118491/original.jpg	https://data.globe.gov/system/photos/2019/06/28/1118493/original.jpg	https://data.globe.gov/system/photos/2019/06/28/1118495/original.jpg
GV_740	38.9934	-76.8401	62.9		https://www.google.com/maps/search/38.9934,-76.8401	https://data.globe.gov/system/photos/2019/06/21/1118593/original.jpg	https://data.globe.gov/system/photos/2019/06/21/1118596/original.jpg	https://data.globe.gov/system/photos/2019/06/21/1118595/original.jpg	https://data.globe.gov/system/photos/2019/06/21/1118597/original.jpg	https://data.globe.gov/system/photos/2019/06/21/1118599/original.jpg
GV_741	38.9943	-76.8408	62.7		https://www.google.com/maps/search/38.9943,-76.8408	https://data.globe.gov/system/photos/2019/06/28/1118688/original.jpg	https://data.globe.gov/system/photos/2019/06/28/1118690/original.jpg	https://data.globe.gov/system/photos/2019/06/28/1118689/original.jpg	https://data.globe.gov/system/photos/2019/06/28/1118691/original.jpg	https://data.globe.gov/system/photos/2019/06/28/1118693/original.jpg
GV_742	47.3316	-101.3848	525.7		https://www.google.com/maps/search/47.3316,-101.3848	https://data.globe.gov/system/photos/2019/06/28/1118703/original.jpg	https://data.globe.gov/system/photos/2019/06/28/1118705/original.jpg	https://data.globe.gov/system/photos/2019/06/28/1118704/original.jpg	https://data.globe.gov/system/photos/2019/06/28/1118706/original.jpg	https://data.globe.gov/system/photos/2019/06/28/1118708/original.jpg
GV_743	42.5233	-83.9511	293.1		https://www.google.com/maps/search/42.5233,-83.9511	https://data.globe.gov/system/photos/2019/06/28/1118779/original.jpg	https://data.globe.gov/system/photos/2019/06/28/1118781/original.jpg	https://data.globe.gov/system/photos/2019/06/28/1118780/original.jpg	https://data.globe.gov/system/photos/2019/06/28/1118783/original.jpg	https://data.globe.gov/system/photos/2019/06/28/1118785/original.jpg
GV_744	46.3687	-117.0585	228.1		https://www.google.com/maps/search/46.3687,-117.0585	https://data.globe.gov/system/photos/2019/06/28/1119216/original.jpg	https://data.globe.gov/system/photos/2019/06/28/1119218/original.jpg	https://data.globe.gov/system/photos/2019/06/28/1119217/original.jpg	https://data.globe.gov/system/photos/2019/06/28/1119219/original.jpg	https://data.globe.gov/system/photos/2019/06/28/1119221/original.jpg
GV_745	46.3687	-117.0578	229.7		https://www.google.com/maps/search/46.3687,-117.0578	https://data.globe.gov/system/photos/2019/06/28/1119223/original.jpg	https://data.globe.gov/system/photos/2019/06/28/1119225/original.jpg	https://data.globe.gov/system/photos/2019/06/28/1119224/original.jpg	https://data.globe.gov/system/photos/2019/06/28/1119226/original.jpg	https://data.globe.gov/system/photos/2019/06/28/1119228/original.jpg
GV_746	30.4269	-97.8152	241.2		https://www.google.com/maps/search/30.4269,-97.8152	https://data.globe.gov/system/photos/2019/06/29/1119254/original.jpg	https://data.globe.gov/system/photos/2019/06/29/1119256/original.jpg	https://data.globe.gov/system/photos/2019/06/29/1119255/original.jpg	https://data.globe.gov/system/photos/2019/06/29/1119257/original.jpg	https://data.globe.gov/system/photos/2019/06/29/1119259/original.jpg
GV_747	46.3686	-117.0568	232.6		https://www.google.com/maps/search/46.3686,-117.0568	https://data.globe.gov/system/photos/2019/06/28/1119351/original.jpg	https://data.globe.gov/system/photos/2019/06/28/1119353/original.jpg	https://data.globe.gov/system/photos/2019/06/28/1119352/original.jpg	https://data.globe.gov/system/photos/2019/06/28/1119354/original.jpg	https://data.globe.gov/system/photos/2019/06/28/1119356/original.jpg
GV_748	46.3707	-117.054	233		https://www.google.com/maps/search/46.3707,-117.054	https://data.globe.gov/system/photos/2019/06/28/1119357/original.jpg	https://data.globe.gov/system/photos/2019/06/28/1119359/original.jpg	https://data.globe.gov/system/photos/2019/06/28/1119358/original.jpg	https://data.globe.gov/system/photos/2019/06/28/1119360/original.jpg	https://data.globe.gov/system/photos/2019/06/28/1119362/original.jpg
GV_749	46.3888	-117.0411	239.2		https://www.google.com/maps/search/46.3888,-117.0411	https://data.globe.gov/system/photos/2019/06/28/1119363/original.jpg	https://data.globe.gov/system/photos/2019/06/28/1119365/original.jpg	https://data.globe.gov/system/photos/2019/06/28/1119364/original.jpg	https://data.globe.gov/system/photos/2019/06/28/1119366/original.jpg	https://data.globe.gov/system/photos/2019/06/28/1119368/original.jpg
GV_750	46.404	-117.0353	226.9		https://www.google.com/maps/search/46.404,-117.0353	https://data.globe.gov/system/photos/2019/06/28/1119369/original.jpg	https://data.globe.gov/system/photos/2019/06/28/1119371/original.jpg	https://data.globe.gov/system/photos/2019/06/28/1119370/original.jpg	https://data.globe.gov/system/photos/2019/06/28/1119372/original.jpg	https://data.globe.gov/system/photos/2019/06/28/1119374/original.jpg
GV_751	46.4048	-117.0347	225.5		https://www.google.com/maps/search/46.4048,-117.0347	https://data.globe.gov/system/photos/2019/06/28/1119375/original.jpg	https://data.globe.gov/system/photos/2019/06/28/1119377/original.jpg	https://data.globe.gov/system/photos/2019/06/28/1119376/original.jpg	https://data.globe.gov/system/photos/2019/06/28/1119378/original.jpg	https://data.globe.gov/system/photos/2019/06/28/1119380/original.jpg
GV_752	46.4036	-117.0349	228.4		https://www.google.com/maps/search/46.4036,-117.0349	https://data.globe.gov/system/photos/2019/06/28/1119381/original.jpg	https://data.globe.gov/system/photos/2019/06/28/1119383/original.jpg	https://data.globe.gov/system/photos/2019/06/28/1119382/original.jpg	https://data.globe.gov/system/photos/2019/06/28/1119384/original.jpg	https://data.globe.gov/system/photos/2019/06/28/1119386/original.jpg
GV_753	46.4027	-117.0354	228.1		https://www.google.com/maps/search/46.4027,-117.0354	https://data.globe.gov/system/photos/2019/06/28/1119387/original.jpg	https://data.globe.gov/system/photos/2019/06/28/1119389/original.jpg	https://data.globe.gov/system/photos/2019/06/28/1119388/original.jpg	https://data.globe.gov/system/photos/2019/06/28/1119390/original.jpg	https://data.globe.gov/system/photos/2019/06/28/1119392/original.jpg
GV_754	46.4359	-116.9885	292.4		https://www.google.com/maps/search/46.4359,-116.9885	https://data.globe.gov/system/photos/2019/06/28/1119393/original.jpg	https://data.globe.gov/system/photos/2019/06/28/1119395/original.jpg	https://data.globe.gov/system/photos/2019/06/28/1119394/original.jpg	https://data.globe.gov/system/photos/2019/06/28/1119396/original.jpg	https://data.globe.gov/system/photos/2019/06/28/1119398/original.jpg
GV_755	46.4602	-117.0163	794.6		https://www.google.com/maps/search/46.4602,-117.0163	https://data.globe.gov/system/photos/2019/06/28/1119399/original.jpg	https://data.globe.gov/system/photos/2019/06/28/1119401/original.jpg	https://data.globe.gov/system/photos/2019/06/28/1119400/original.jpg	https://data.globe.gov/system/photos/2019/06/28/1119402/original.jpg	https://data.globe.gov/system/photos/2019/06/28/1119404/original.jpg
GV_756	46.4771	-117.0518	822.9		https://www.google.com/maps/search/46.4771,-117.0518	https://data.globe.gov/system/photos/2019/06/28/1119405/original.jpg	https://data.globe.gov/system/photos/2019/06/28/1119407/original.jpg	https://data.globe.gov/system/photos/2019/06/28/1119406/original.jpg	https://data.globe.gov/system/photos/2019/06/28/1119408/original.jpg	https://data.globe.gov/system/photos/2019/06/28/1119410/original.jpg
GV_757	46.4876	-117.0709	852.1		https://www.google.com/maps/search/46.4876,-117.0709	https://data.globe.gov/system/photos/2019/06/28/1119411/original.jpg	https://data.globe.gov/system/photos/2019/06/28/1119413/original.jpg	https://data.globe.gov/system/photos/2019/06/28/1119412/original.jpg	https://data.globe.gov/system/photos/2019/06/28/1119414/original.jpg	https://data.globe.gov/system/photos/2019/06/28/1119416/original.jpg
GV_758	45.6776	-120.864	234.2		https://www.google.com/maps/search/45.6776,-120.864	https://data.globe.gov/system/photos/2019/06/28/1119432/original.jpg	https://data.globe.gov/system/photos/2019/06/28/1119434/original.jpg	https://data.globe.gov/system/photos/2019/06/28/1119433/original.jpg	https://data.globe.gov/system/photos/2019/06/28/1119435/original.jpg	https://data.globe.gov/system/photos/2019/06/28/1119437/original.jpg
GV_759	45.6518	-121.2087	56.2		https://www.google.com/maps/search/45.6518,-121.2087	https://data.globe.gov/system/photos/2019/06/28/1119438/original.jpg	https://data.globe.gov/system/photos/2019/06/28/1119440/original.jpg	https://data.globe.gov/system/photos/2019/06/28/1119439/original.jpg	https://data.globe.gov/system/photos/2019/06/28/1119441/original.jpg	https://data.globe.gov/system/photos/2019/06/28/1119443/original.jpg
GV_760	45.651	-121.2081	43.4		https://www.google.com/maps/search/45.651,-121.2081	https://data.globe.gov/system/photos/2019/06/28/1119444/original.jpg	https://data.globe.gov/system/photos/2019/06/28/1119446/original.jpg	https://data.globe.gov/system/photos/2019/06/28/1119445/original.jpg	https://data.globe.gov/system/photos/2019/06/28/1119447/original.jpg	https://data.globe.gov/system/photos/2019/06/28/1119449/original.jpg
GV_761	45.6534	-121.2091	64.5		https://www.google.com/maps/search/45.6534,-121.2091	https://data.globe.gov/system/photos/2019/06/28/1119450/original.jpg	https://data.globe.gov/system/photos/2019/06/28/1119452/original.jpg	https://data.globe.gov/system/photos/2019/06/28/1119451/original.jpg	https://data.globe.gov/system/photos/2019/06/28/1119453/original.jpg	https://data.globe.gov/system/photos/2019/06/28/1119455/original.jpg
GV_762	45.654	-121.2112	63.9		https://www.google.com/maps/search/45.654,-121.2112	https://data.globe.gov/system/photos/2019/06/28/1119456/original.jpg	https://data.globe.gov/system/photos/2019/06/28/1119458/original.jpg	https://data.globe.gov/system/photos/2019/06/28/1119457/original.jpg	https://data.globe.gov/system/photos/2019/06/28/1119459/original.jpg	https://data.globe.gov/system/photos/2019/06/28/1119461/original.jpg
GV_763	45.5771	-122.121	24.3		https://www.google.com/maps/search/45.5771,-122.121	https://data.globe.gov/system/photos/2019/06/29/1119462/original.jpg	https://data.globe.gov/system/photos/2019/06/29/1119464/original.jpg	https://data.globe.gov/system/photos/2019/06/29/1119463/original.jpg	https://data.globe.gov/system/photos/2019/06/29/1119465/original.jpg	https://data.globe.gov/system/photos/2019/06/29/1119467/original.jpg
GV_764	45.5769	-122.1213	33.8		https://www.google.com/maps/search/45.5769,-122.1213	https://data.globe.gov/system/photos/2019/06/29/1119468/original.jpg	https://data.globe.gov/system/photos/2019/06/29/1119470/original.jpg	https://data.globe.gov/system/photos/2019/06/29/1119469/original.jpg	https://data.globe.gov/system/photos/2019/06/29/1119471/original.jpg	https://data.globe.gov/system/photos/2019/06/29/1119473/original.jpg
GV_765	45.5759	-122.1256	39.5		https://www.google.com/maps/search/45.5759,-122.1256	https://data.globe.gov/system/photos/2019/06/29/1119474/original.jpg	https://data.globe.gov/system/photos/2019/06/29/1119476/original.jpg	https://data.globe.gov/system/photos/2019/06/29/1119475/original.jpg	https://data.globe.gov/system/photos/2019/06/29/1119477/original.jpg	https://data.globe.gov/system/photos/2019/06/29/1119479/original.jpg
GV_766	45.5768	-122.1165	14.2		https://www.google.com/maps/search/45.5768,-122.1165	https://data.globe.gov/system/photos/2019/06/29/1119480/original.jpg	https://data.globe.gov/system/photos/2019/06/29/1119482/original.jpg	https://data.globe.gov/system/photos/2019/06/29/1119481/original.jpg	https://data.globe.gov/system/photos/2019/06/29/1119483/original.jpg	https://data.globe.gov/system/photos/2019/06/29/1119485/original.jpg
GV_767	45.5778	-122.1144	87		https://www.google.com/maps/search/45.5778,-122.1144	https://data.globe.gov/system/photos/2019/06/29/1119486/original.jpg	https://data.globe.gov/system/photos/2019/06/29/1119488/original.jpg	https://data.globe.gov/system/photos/2019/06/29/1119487/original.jpg	https://data.globe.gov/system/photos/2019/06/29/1119489/original.jpg	https://data.globe.gov/system/photos/2019/06/29/1119491/original.jpg
GV_768	45.5775	-122.1154	103.4		https://www.google.com/maps/search/45.5775,-122.1154	https://data.globe.gov/system/photos/2019/06/29/1119492/original.jpg	https://data.globe.gov/system/photos/2019/06/29/1119494/original.jpg	https://data.globe.gov/system/photos/2019/06/29/1119493/original.jpg	https://data.globe.gov/system/photos/2019/06/29/1119495/original.jpg	https://data.globe.gov/system/photos/2019/06/29/1119497/original.jpg
GV_769	45.5761	-122.1156	40.1		https://www.google.com/maps/search/45.5761,-122.1156	https://data.globe.gov/system/photos/2019/06/29/1119498/original.jpg	https://data.globe.gov/system/photos/2019/06/29/1119500/original.jpg	https://data.globe.gov/system/photos/2019/06/29/1119499/original.jpg	https://data.globe.gov/system/photos/2019/06/29/1119501/original.jpg	https://data.globe.gov/system/photos/2019/06/29/1119503/original.jpg
GV_770	45.5464	-122.1978	61.4		https://www.google.com/maps/search/45.5464,-122.1978	https://data.globe.gov/system/photos/2019/06/29/1119513/original.jpg	https://data.globe.gov/system/photos/2019/06/29/1119515/original.jpg	https://data.globe.gov/system/photos/2019/06/29/1119514/original.jpg	https://data.globe.gov/system/photos/2019/06/29/1119516/original.jpg	https://data.globe.gov/system/photos/2019/06/29/1119518/original.jpg
GV_771	45.534	-122.2319	148.4		https://www.google.com/maps/search/45.534,-122.2319	https://data.globe.gov/system/photos/2019/06/29/1119519/original.jpg	https://data.globe.gov/system/photos/2019/06/29/1119521/original.jpg	https://data.globe.gov/system/photos/2019/06/29/1119520/original.jpg	https://data.globe.gov/system/photos/2019/06/29/1119522/original.jpg	https://data.globe.gov/system/photos/2019/06/29/1119524/original.jpg
GV_772	45.5395	-122.2441	223.8		https://www.google.com/maps/search/45.5395,-122.2441	https://data.globe.gov/system/photos/2019/06/29/1119525/original.jpg	https://data.globe.gov/system/photos/2019/06/29/1119527/original.jpg	https://data.globe.gov/system/photos/2019/06/29/1119526/original.jpg	https://data.globe.gov/system/photos/2019/06/29/1119528/original.jpg	https://data.globe.gov/system/photos/2019/06/29/1119530/original.jpg
GV_773	45.5393	-122.2447	223.7		https://www.google.com/maps/search/45.5393,-122.2447	https://data.globe.gov/system/photos/2019/06/29/1119536/original.jpg	https://data.globe.gov/system/photos/2019/06/29/1119538/original.jpg	https://data.globe.gov/system/photos/2019/06/29/1119537/original.jpg	https://data.globe.gov/system/photos/2019/06/29/1119539/original.jpg	https://data.globe.gov/system/photos/2019/06/29/1119541/original.jpg
GV_774	45.5398	-122.2444	222.9		https://www.google.com/maps/search/45.5398,-122.2444	https://data.globe.gov/system/photos/2019/06/29/1119542/original.jpg	https://data.globe.gov/system/photos/2019/06/29/1119544/original.jpg	https://data.globe.gov/system/photos/2019/06/29/1119543/original.jpg	https://data.globe.gov/system/photos/2019/06/29/1119545/original.jpg	https://data.globe.gov/system/photos/2019/06/29/1119547/original.jpg
GV_775	45.5336	-122.2603	263.2		https://www.google.com/maps/search/45.5336,-122.2603	https://data.globe.gov/system/photos/2019/06/29/1119548/original.jpg	https://data.globe.gov/system/photos/2019/06/29/1119550/original.jpg	https://data.globe.gov/system/photos/2019/06/29/1119549/original.jpg	https://data.globe.gov/system/photos/2019/06/29/1119551/original.jpg	https://data.globe.gov/system/photos/2019/06/29/1119553/original.jpg
GV_776	45.5329	-122.2597	264.6		https://www.google.com/maps/search/45.5329,-122.2597	https://data.globe.gov/system/photos/2019/06/29/1119554/original.jpg	https://data.globe.gov/system/photos/2019/06/29/1119556/original.jpg	https://data.globe.gov/system/photos/2019/06/29/1119555/original.jpg	https://data.globe.gov/system/photos/2019/06/29/1119557/original.jpg	https://data.globe.gov/system/photos/2019/06/29/1119559/original.jpg
GV_777	46.2054	-119.0359	105.7		https://www.google.com/maps/search/46.2054,-119.0359	https://data.globe.gov/system/photos/2019/06/28/1119560/original.jpg	https://data.globe.gov/system/photos/2019/06/28/1119562/original.jpg	https://data.globe.gov/system/photos/2019/06/28/1119561/original.jpg	https://data.globe.gov/system/photos/2019/06/28/1119563/original.jpg	https://data.globe.gov/system/photos/2019/06/28/1119565/original.jpg
GV_778	46.2013	-119.0384	106.3		https://www.google.com/maps/search/46.2013,-119.0384	https://data.globe.gov/system/photos/2019/06/28/1119566/original.jpg	https://data.globe.gov/system/photos/2019/06/28/1119568/original.jpg	https://data.globe.gov/system/photos/2019/06/28/1119567/original.jpg	https://data.globe.gov/system/photos/2019/06/28/1119569/original.jpg	https://data.globe.gov/system/photos/2019/06/28/1119571/original.jpg
GV_779	46.192	-119.0516	104.3		https://www.google.com/maps/search/46.192,-119.0516	https://data.globe.gov/system/photos/2019/06/28/1119578/original.jpg	https://data.globe.gov/system/photos/2019/06/28/1119580/original.jpg	https://data.globe.gov/system/photos/2019/06/28/1119579/original.jpg	https://data.globe.gov/system/photos/2019/06/28/1119581/original.jpg	https://data.globe.gov/system/photos/2019/06/28/1119583/original.jpg
GV_780	46.1924	-119.0495	104.3		https://www.google.com/maps/search/46.1924,-119.0495	https://data.globe.gov/system/photos/2019/06/28/1119584/original.jpg	https://data.globe.gov/system/photos/2019/06/28/1119586/original.jpg	https://data.globe.gov/system/photos/2019/06/28/1119585/original.jpg	https://data.globe.gov/system/photos/2019/06/28/1119587/original.jpg	https://data.globe.gov/system/photos/2019/06/28/1119589/original.jpg
GV_781	46.1918	-119.0497	106.2		https://www.google.com/maps/search/46.1918,-119.0497	https://data.globe.gov/system/photos/2019/06/28/1119590/original.jpg	https://data.globe.gov/system/photos/2019/06/28/1119592/original.jpg	https://data.globe.gov/system/photos/2019/06/28/1119591/original.jpg	https://data.globe.gov/system/photos/2019/06/28/1119593/original.jpg	https://data.globe.gov/system/photos/2019/06/28/1119595/original.jpg
GV_782	46.1907	-119.0529	105.8		https://www.google.com/maps/search/46.1907,-119.0529	https://data.globe.gov/system/photos/2019/06/28/1119596/original.jpg	https://data.globe.gov/system/photos/2019/06/28/1119598/original.jpg	https://data.globe.gov/system/photos/2019/06/28/1119597/original.jpg	https://data.globe.gov/system/photos/2019/06/28/1119599/original.jpg	https://data.globe.gov/system/photos/2019/06/28/1119601/original.jpg
GV_783	46.198	-119.1299	120.3		https://www.google.com/maps/search/46.198,-119.1299	https://data.globe.gov/system/photos/2019/06/28/1119602/original.jpg	https://data.globe.gov/system/photos/2019/06/28/1119604/original.jpg	https://data.globe.gov/system/photos/2019/06/28/1119603/original.jpg	https://data.globe.gov/system/photos/2019/06/28/1119605/original.jpg	https://data.globe.gov/system/photos/2019/06/28/1119607/original.jpg
GV_784	46.1974	-119.1297	120.2		https://www.google.com/maps/search/46.1974,-119.1297	https://data.globe.gov/system/photos/2019/06/28/1119608/original.jpg	https://data.globe.gov/system/photos/2019/06/28/1119610/original.jpg	https://data.globe.gov/system/photos/2019/06/28/1119609/original.jpg	https://data.globe.gov/system/photos/2019/06/28/1119611/original.jpg	https://data.globe.gov/system/photos/2019/06/28/1119613/original.jpg
GV_785	45.9311	-119.3508	86.4		https://www.google.com/maps/search/45.9311,-119.3508	https://data.globe.gov/system/photos/2019/06/28/1119614/original.jpg	https://data.globe.gov/system/photos/2019/06/28/1119616/original.jpg	https://data.globe.gov/system/photos/2019/06/28/1119615/original.jpg	https://data.globe.gov/system/photos/2019/06/28/1119617/original.jpg	https://data.globe.gov/system/photos/2019/06/28/1119619/original.jpg
GV_786	45.9306	-119.3465	88.2		https://www.google.com/maps/search/45.9306,-119.3465	https://data.globe.gov/system/photos/2019/06/28/1119620/original.jpg	https://data.globe.gov/system/photos/2019/06/28/1119622/original.jpg	https://data.globe.gov/system/photos/2019/06/28/1119621/original.jpg	https://data.globe.gov/system/photos/2019/06/28/1119623/original.jpg	https://data.globe.gov/system/photos/2019/06/28/1119625/original.jpg
GV_787	45.9298	-119.3526	85.5		https://www.google.com/maps/search/45.9298,-119.3526	https://data.globe.gov/system/photos/2019/06/28/1119626/original.jpg	https://data.globe.gov/system/photos/2019/06/28/1119628/original.jpg	https://data.globe.gov/system/photos/2019/06/28/1119627/original.jpg	https://data.globe.gov/system/photos/2019/06/28/1119629/original.jpg	https://data.globe.gov/system/photos/2019/06/28/1119631/original.jpg
GV_788	45.9295	-119.3522	81.6		https://www.google.com/maps/search/45.9295,-119.3522	https://data.globe.gov/system/photos/2019/06/28/1119632/original.jpg	https://data.globe.gov/system/photos/2019/06/28/1119634/original.jpg	https://data.globe.gov/system/photos/2019/06/28/1119633/original.jpg	https://data.globe.gov/system/photos/2019/06/28/1119635/original.jpg	https://data.globe.gov/system/photos/2019/06/28/1119637/original.jpg
GV_789	45.9261	-119.413	86.5		https://www.google.com/maps/search/45.9261,-119.413	https://data.globe.gov/system/photos/2019/06/28/1119638/original.jpg	https://data.globe.gov/system/photos/2019/06/28/1119640/original.jpg	https://data.globe.gov/system/photos/2019/06/28/1119639/original.jpg	https://data.globe.gov/system/photos/2019/06/28/1119641/original.jpg	https://data.globe.gov/system/photos/2019/06/28/1119643/original.jpg
GV_790	45.9201	-119.6398	85.3		https://www.google.com/maps/search/45.9201,-119.6398	https://data.globe.gov/system/photos/2019/06/28/1119649/original.jpg	https://data.globe.gov/system/photos/2019/06/28/1119651/original.jpg	https://data.globe.gov/system/photos/2019/06/28/1119650/original.jpg	https://data.globe.gov/system/photos/2019/06/28/1119652/original.jpg	https://data.globe.gov/system/photos/2019/06/28/1119654/original.jpg
GV_791	45.8723	-119.7392	83.4		https://www.google.com/maps/search/45.8723,-119.7392	https://data.globe.gov/system/photos/2019/06/28/1119655/original.jpg	https://data.globe.gov/system/photos/2019/06/28/1119657/original.jpg	https://data.globe.gov/system/photos/2019/06/28/1119656/original.jpg	https://data.globe.gov/system/photos/2019/06/28/1119658/original.jpg	https://data.globe.gov/system/photos/2019/06/28/1119660/original.jpg
GV_792	45.8731	-119.7291	86.1		https://www.google.com/maps/search/45.8731,-119.7291	https://data.globe.gov/system/photos/2019/06/28/1119661/original.jpg	https://data.globe.gov/system/photos/2019/06/28/1119663/original.jpg	https://data.globe.gov/system/photos/2019/06/28/1119662/original.jpg	https://data.globe.gov/system/photos/2019/06/28/1119664/original.jpg	https://data.globe.gov/system/photos/2019/06/28/1119666/original.jpg
GV_793	45.8358	-119.9293	85.8		https://www.google.com/maps/search/45.8358,-119.9293	https://data.globe.gov/system/photos/2019/06/28/1119667/original.jpg	https://data.globe.gov/system/photos/2019/06/28/1119669/original.jpg	https://data.globe.gov/system/photos/2019/06/28/1119668/original.jpg	https://data.globe.gov/system/photos/2019/06/28/1119670/original.jpg	https://data.globe.gov/system/photos/2019/06/28/1119672/original.jpg
GV_794	45.7872	-120.1049	96.3		https://www.google.com/maps/search/45.7872,-120.1049	https://data.globe.gov/system/photos/2019/06/28/1119673/original.jpg	https://data.globe.gov/system/photos/2019/06/28/1119675/original.jpg	https://data.globe.gov/system/photos/2019/06/28/1119674/original.jpg	https://data.globe.gov/system/photos/2019/06/28/1119676/original.jpg	https://data.globe.gov/system/photos/2019/06/28/1119678/original.jpg
GV_795	45.7197	-120.318	102.1		https://www.google.com/maps/search/45.7197,-120.318	https://data.globe.gov/system/photos/2019/06/28/1119679/original.jpg	https://data.globe.gov/system/photos/2019/06/28/1119681/original.jpg	https://data.globe.gov/system/photos/2019/06/28/1119680/original.jpg	https://data.globe.gov/system/photos/2019/06/28/1119682/original.jpg	https://data.globe.gov/system/photos/2019/06/28/1119684/original.jpg
GV_796	45.7113	-120.3663	144.7		https://www.google.com/maps/search/45.7113,-120.3663	https://data.globe.gov/system/photos/2019/06/28/1119685/original.jpg	https://data.globe.gov/system/photos/2019/06/28/1119687/original.jpg	https://data.globe.gov/system/photos/2019/06/28/1119686/original.jpg	https://data.globe.gov/system/photos/2019/06/28/1119688/original.jpg	https://data.globe.gov/system/photos/2019/06/28/1119690/original.jpg
GV_797	45.7074	-120.3959	95.4		https://www.google.com/maps/search/45.7074,-120.3959	https://data.globe.gov/system/photos/2019/06/28/1119691/original.jpg	https://data.globe.gov/system/photos/2019/06/28/1119693/original.jpg	https://data.globe.gov/system/photos/2019/06/28/1119692/original.jpg	https://data.globe.gov/system/photos/2019/06/28/1119694/original.jpg	https://data.globe.gov/system/photos/2019/06/28/1119696/original.jpg
GV_798	45.6944	-120.8059	182.7		https://www.google.com/maps/search/45.6944,-120.8059	https://data.globe.gov/system/photos/2019/06/28/1119697/original.jpg	https://data.globe.gov/system/photos/2019/06/28/1119699/original.jpg	https://data.globe.gov/system/photos/2019/06/28/1119698/original.jpg	https://data.globe.gov/system/photos/2019/06/28/1119700/original.jpg	https://data.globe.gov/system/photos/2019/06/28/1119702/original.jpg
GV_799	45.6946	-120.8056	180.4		https://www.google.com/maps/search/45.6946,-120.8056	https://data.globe.gov/system/photos/2019/06/28/1119708/original.jpg	https://data.globe.gov/system/photos/2019/06/28/1119710/original.jpg	https://data.globe.gov/system/photos/2019/06/28/1119709/original.jpg	https://data.globe.gov/system/photos/2019/06/28/1119711/original.jpg	https://data.globe.gov/system/photos/2019/06/28/1119713/original.jpg
GV_800	45.6939	-120.8068	171.4		https://www.google.com/maps/search/45.6939,-120.8068	https://data.globe.gov/system/photos/2019/06/28/1119714/original.jpg	https://data.globe.gov/system/photos/2019/06/28/1119716/original.jpg	https://data.globe.gov/system/photos/2019/06/28/1119715/original.jpg	https://data.globe.gov/system/photos/2019/06/28/1119717/original.jpg	https://data.globe.gov/system/photos/2019/06/28/1119719/original.jpg
GV_801	45.6785	-120.8613	233.1		https://www.google.com/maps/search/45.6785,-120.8613	https://data.globe.gov/system/photos/2019/06/28/1119720/original.jpg	https://data.globe.gov/system/photos/2019/06/28/1119722/original.jpg	https://data.globe.gov/system/photos/2019/06/28/1119721/original.jpg	https://data.globe.gov/system/photos/2019/06/28/1119723/original.jpg	https://data.globe.gov/system/photos/2019/06/28/1119725/original.jpg
GV_802	45.6784	-120.8616	233		https://www.google.com/maps/search/45.6784,-120.8616	https://data.globe.gov/system/photos/2019/06/28/1119726/original.jpg	https://data.globe.gov/system/photos/2019/06/28/1119728/original.jpg	https://data.globe.gov/system/photos/2019/06/28/1119727/original.jpg	https://data.globe.gov/system/photos/2019/06/28/1119729/original.jpg	https://data.globe.gov/system/photos/2019/06/28/1119731/original.jpg
GV_803	45.6788	-120.862	235.8		https://www.google.com/maps/search/45.6788,-120.862	https://data.globe.gov/system/photos/2019/06/28/1119732/original.jpg	https://data.globe.gov/system/photos/2019/06/28/1119734/original.jpg	https://data.globe.gov/system/photos/2019/06/28/1119733/original.jpg	https://data.globe.gov/system/photos/2019/06/28/1119735/original.jpg	https://data.globe.gov/system/photos/2019/06/28/1119737/original.jpg

GV_804	45.6774	-120.8645	235.7		https://www.google.com/maps/search/45.6774,-120.8645	https://data.globe.gov/system/photos/2019/06/28/1119738/original.jpg	https://data.globe.gov/system/photos/2019/06/28/1119740/original.jpg	https://data.globe.gov/system/photos/2019/06/28/1119739/original.jpg	https://data.globe.gov/system/photos/2019/06/28/1119741/original.jpg	https://data.globe.gov/system/photos/2019/06/28/1119743/original.jpg
GV_805	45.6787	-120.863	235.4		https://www.google.com/maps/search/45.6787,-120.863	https://data.globe.gov/system/photos/2019/06/28/1119744/original.jpg	https://data.globe.gov/system/photos/2019/06/28/1119746/original.jpg	https://data.globe.gov/system/photos/2019/06/28/1119745/original.jpg	https://data.globe.gov/system/photos/2019/06/28/1119747/original.jpg	https://data.globe.gov/system/photos/2019/06/28/1119749/original.jpg
GV_806	45.6776	-120.8644	235.6		https://www.google.com/maps/search/45.6776,-120.8644	https://data.globe.gov/system/photos/2019/06/28/1119750/original.jpg	https://data.globe.gov/system/photos/2019/06/28/1119752/original.jpg	https://data.globe.gov/system/photos/2019/06/28/1119751/original.jpg	https://data.globe.gov/system/photos/2019/06/28/1119753/original.jpg	https://data.globe.gov/system/photos/2019/06/28/1119755/original.jpg
GV_807	45.6782	-120.8634	235.7		https://www.google.com/maps/search/45.6782,-120.8634	https://data.globe.gov/system/photos/2019/06/28/1119756/original.jpg	https://data.globe.gov/system/photos/2019/06/28/1119758/original.jpg	https://data.globe.gov/system/photos/2019/06/28/1119757/original.jpg	https://data.globe.gov/system/photos/2019/06/28/1119759/original.jpg	https://data.globe.gov/system/photos/2019/06/28/1119761/original.jpg
GV_808	45.6538	-121.2105	65.4		https://www.google.com/maps/search/45.6538,-121.2105	https://data.globe.gov/system/photos/2019/06/28/1119762/original.jpg	https://data.globe.gov/system/photos/2019/06/28/1119764/original.jpg	https://data.globe.gov/system/photos/2019/06/28/1119763/original.jpg	https://data.globe.gov/system/photos/2019/06/28/1119765/original.jpg	https://data.globe.gov/system/photos/2019/06/28/1119767/original.jpg
GV_809	45.6541	-121.2104	66.8		https://www.google.com/maps/search/45.6541,-121.2104	https://data.globe.gov/system/photos/2019/06/28/1119768/original.jpg	https://data.globe.gov/system/photos/2019/06/28/1119770/original.jpg	https://data.globe.gov/system/photos/2019/06/28/1119769/original.jpg	https://data.globe.gov/system/photos/2019/06/28/1119771/original.jpg	https://data.globe.gov/system/photos/2019/06/28/1119773/original.jpg
GV_810	45.5922	-122.0597	19.1		https://www.google.com/maps/search/45.5922,-122.0597	https://data.globe.gov/system/photos/2019/06/29/1119774/original.jpg	https://data.globe.gov/system/photos/2019/06/29/1119776/original.jpg	https://data.globe.gov/system/photos/2019/06/29/1119775/original.jpg	https://data.globe.gov/system/photos/2019/06/29/1119777/original.jpg	https://data.globe.gov/system/photos/2019/06/29/1119779/original.jpg
GV_811	45.5774	-122.1152	114.8		https://www.google.com/maps/search/45.5774,-122.1152	https://data.globe.gov/system/photos/2019/06/29/1119780/original.jpg	https://data.globe.gov/system/photos/2019/06/29/1119782/original.jpg	https://data.globe.gov/system/photos/2019/06/29/1119781/original.jpg	https://data.globe.gov/system/photos/2019/06/29/1119783/original.jpg	https://data.globe.gov/system/photos/2019/06/29/1119785/original.jpg
GV_812	45.5761	-122.1243	37.8		https://www.google.com/maps/search/45.5761,-122.1243	https://data.globe.gov/system/photos/2019/06/29/1119786/original.jpg	https://data.globe.gov/system/photos/2019/06/29/1119788/original.jpg	https://data.globe.gov/system/photos/2019/06/29/1119787/original.jpg	https://data.globe.gov/system/photos/2019/06/29/1119789/original.jpg	https://data.globe.gov/system/photos/2019/06/29/1119791/original.jpg
GV_813	45.5766	-122.122	74.7		https://www.google.com/maps/search/45.5766,-122.122	https://data.globe.gov/system/photos/2019/06/29/1119792/original.jpg	https://data.globe.gov/system/photos/2019/06/29/1119794/original.jpg	https://data.globe.gov/system/photos/2019/06/29/1119793/original.jpg	https://data.globe.gov/system/photos/2019/06/29/1119795/original.jpg	https://data.globe.gov/system/photos/2019/06/29/1119797/original.jpg
GV_814	45.5772	-122.1156	103.4		https://www.google.com/maps/search/45.5772,-122.1156	https://data.globe.gov/system/photos/2019/06/29/1119798/original.jpg	https://data.globe.gov/system/photos/2019/06/29/1119800/original.jpg	https://data.globe.gov/system/photos/2019/06/29/1119799/original.jpg	https://data.globe.gov/system/photos/2019/06/29/1119801/original.jpg	https://data.globe.gov/system/photos/2019/06/29/1119803/original.jpg
GV_815	45.5774	-122.1133	136.8		https://www.google.com/maps/search/45.5774,-122.1133	https://data.globe.gov/system/photos/2019/06/29/1119804/original.jpg	https://data.globe.gov/system/photos/2019/06/29/1119806/original.jpg	https://data.globe.gov/system/photos/2019/06/29/1119805/original.jpg	https://data.globe.gov/system/photos/2019/06/29/1119807/original.jpg	https://data.globe.gov/system/photos/2019/06/29/1119809/original.jpg
GV_816	45.5774	-122.1149	119.4		https://www.google.com/maps/search/45.5774,-122.1149	https://data.globe.gov/system/photos/2019/06/29/1119810/original.jpg	https://data.globe.gov/system/photos/2019/06/29/1119812/original.jpg	https://data.globe.gov/system/photos/2019/06/29/1119811/original.jpg	https://data.globe.gov/system/photos/2019/06/29/1119813/original.jpg	https://data.globe.gov/system/photos/2019/06/29/1119815/original.jpg
GV_817	45.5779	-122.1158	64.9		https://www.google.com/maps/search/45.5779,-122.1158	https://data.globe.gov/system/photos/2019/06/29/1119816/original.jpg	https://data.globe.gov/system/photos/2019/06/29/1119818/original.jpg	https://data.globe.gov/system/photos/2019/06/29/1119817/original.jpg	https://data.globe.gov/system/photos/2019/06/29/1119819/original.jpg	https://data.globe.gov/system/photos/2019/06/29/1119821/original.jpg
GV_818	45.5768	-122.116	50		https://www.google.com/maps/search/45.5768,-122.116	https://data.globe.gov/system/photos/2019/06/29/1119822/original.jpg	https://data.globe.gov/system/photos/2019/06/29/1119824/original.jpg	https://data.globe.gov/system/photos/2019/06/29/1119823/original.jpg	https://data.globe.gov/system/photos/2019/06/29/1119825/original.jpg	https://data.globe.gov/system/photos/2019/06/29/1119827/original.jpg
GV_819	45.5467	-122.1977	22.4		https://www.google.com/maps/search/45.5467,-122.1977	https://data.globe.gov/system/photos/2019/06/29/1119828/original.jpg	https://data.globe.gov/system/photos/2019/06/29/1119830/original.jpg	https://data.globe.gov/system/photos/2019/06/29/1119829/original.jpg	https://data.globe.gov/system/photos/2019/06/29/1119831/original.jpg	https://data.globe.gov/system/photos/2019/06/29/1119833/original.jpg
GV_820	46.1986	-119.0399	105.2		https://www.google.com/maps/search/46.1986,-119.0399	https://data.globe.gov/system/photos/2019/06/28/1119835/original.jpg	https://data.globe.gov/system/photos/2019/06/28/1119837/original.jpg	https://data.globe.gov/system/photos/2019/06/28/1119836/original.jpg	https://data.globe.gov/system/photos/2019/06/28/1119838/original.jpg	https://data.globe.gov/system/photos/2019/06/28/1119840/original.jpg
GV_821	46.199	-119.0394	106.8		https://www.google.com/maps/search/46.199,-119.0394	https://data.globe.gov/system/photos/2019/06/28/1119841/original.jpg	https://data.globe.gov/system/photos/2019/06/28/1119843/original.jpg	https://data.globe.gov/system/photos/2019/06/28/1119842/original.jpg	https://data.globe.gov/system/photos/2019/06/28/1119844/original.jpg	https://data.globe.gov/system/photos/2019/06/28/1119846/original.jpg
GV_822	46.1989	-119.0383	104.4		https://www.google.com/maps/search/46.1989,-119.0383	https://data.globe.gov/system/photos/2019/06/28/1119847/original.jpg	https://data.globe.gov/system/photos/2019/06/28/1119849/original.jpg	https://data.globe.gov/system/photos/2019/06/28/1119848/original.jpg	https://data.globe.gov/system/photos/2019/06/28/1119850/original.jpg	https://data.globe.gov/system/photos/2019/06/28/1119852/original.jpg
GV_823	46.1981	-119.0396	104.7		https://www.google.com/maps/search/46.1981,-119.0396	https://data.globe.gov/system/photos/2019/06/28/1119853/original.jpg	https://data.globe.gov/system/photos/2019/06/28/1119855/original.jpg	https://data.globe.gov/system/photos/2019/06/28/1119854/original.jpg	https://data.globe.gov/system/photos/2019/06/28/1119856/original.jpg	https://data.globe.gov/system/photos/2019/06/28/1119858/original.jpg
GV_824	46.1984	-119.0394	105.9		https://www.google.com/maps/search/46.1984,-119.0394	https://data.globe.gov/system/photos/2019/06/28/1119859/original.jpg	https://data.globe.gov/system/photos/2019/06/28/1119861/original.jpg	https://data.globe.gov/system/photos/2019/06/28/1119860/original.jpg	https://data.globe.gov/system/photos/2019/06/28/1119862/original.jpg	https://data.globe.gov/system/photos/2019/06/28/1119864/original.jpg
GV_825	46.1995	-119.0388	107.1		https://www.google.com/maps/search/46.1995,-119.0388	https://data.globe.gov/system/photos/2019/06/28/1119865/original.jpg	https://data.globe.gov/system/photos/2019/06/28/1119867/original.jpg	https://data.globe.gov/system/photos/2019/06/28/1119866/original.jpg	https://data.globe.gov/system/photos/2019/06/28/1119868/original.jpg	https://data.globe.gov/system/photos/2019/06/28/1119870/original.jpg
GV_826	46.2004	-119.0372	104.4		https://www.google.com/maps/search/46.2004,-119.0372	https://data.globe.gov/system/photos/2019/06/28/1119871/original.jpg	https://data.globe.gov/system/photos/2019/06/28/1119873/original.jpg	https://data.globe.gov/system/photos/2019/06/28/1119872/original.jpg	https://data.globe.gov/system/photos/2019/06/28/1119874/original.jpg	https://data.globe.gov/system/photos/2019/06/28/1119876/original.jpg
GV_827	46.2016	-119.0369	104.5		https://www.google.com/maps/search/46.2016,-119.0369	https://data.globe.gov/system/photos/2019/06/28/1119877/original.jpg	https://data.globe.gov/system/photos/2019/06/28/1119879/original.jpg	https://data.globe.gov/system/photos/2019/06/28/1119878/original.jpg	https://data.globe.gov/system/photos/2019/06/28/1119880/original.jpg	https://data.globe.gov/system/photos/2019/06/28/1119882/original.jpg
GV_828	46.2018	-119.0377	104.4		https://www.google.com/maps/search/46.2018,-119.0377	https://data.globe.gov/system/photos/2019/06/28/1119883/original.jpg	https://data.globe.gov/system/photos/2019/06/28/1119885/original.jpg	https://data.globe.gov/system/photos/2019/06/28/1119884/original.jpg	https://data.globe.gov/system/photos/2019/06/28/1119886/original.jpg	https://data.globe.gov/system/photos/2019/06/28/1119888/original.jpg
GV_829	46.2014	-119.0394	106.3		https://www.google.com/maps/search/46.2014,-119.0394	https://data.globe.gov/system/photos/2019/06/28/1119889/original.jpg	https://data.globe.gov/system/photos/2019/06/28/1119891/original.jpg	https://data.globe.gov/system/photos/2019/06/28/1119890/original.jpg	https://data.globe.gov/system/photos/2019/06/28/1119892/original.jpg	https://data.globe.gov/system/photos/2019/06/28/1119894/original.jpg
GV_830	46.2017	-119.0388	106		https://www.google.com/maps/search/46.2017,-119.0388	https://data.globe.gov/system/photos/2019/06/28/1119895/original.jpg	https://data.globe.gov/system/photos/2019/06/28/1119897/original.jpg	https://data.globe.gov/system/photos/2019/06/28/1119896/original.jpg	https://data.globe.gov/system/photos/2019/06/28/1119898/original.jpg	https://data.globe.gov/system/photos/2019/06/28/1119900/original.jpg
GV_831	46.2058	-119.0351	105.8		https://www.google.com/maps/search/46.2058,-119.0351	https://data.globe.gov/system/photos/2019/06/28/1119901/original.jpg	https://data.globe.gov/system/photos/2019/06/28/1119903/original.jpg	https://data.globe.gov/system/photos/2019/06/28/1119902/original.jpg	https://data.globe.gov/system/photos/2019/06/28/1119904/original.jpg	https://data.globe.gov/system/photos/2019/06/28/1119906/original.jpg
GV_832	46.1917	-119.0519	104.3		https://www.google.com/maps/search/46.1917,-119.0519	https://data.globe.gov/system/photos/2019/06/28/1119907/original.jpg	https://data.globe.gov/system/photos/2019/06/28/1119909/original.jpg	https://data.globe.gov/system/photos/2019/06/28/1119908/original.jpg	https://data.globe.gov/system/photos/2019/06/28/1119910/original.jpg	https://data.globe.gov/system/photos/2019/06/28/1119912/original.jpg
GV_833	46.1921	-119.0506	104.3		https://www.google.com/maps/search/46.1921,-119.0506	https://data.globe.gov/system/photos/2019/06/28/1119913/original.jpg	https://data.globe.gov/system/photos/2019/06/28/1119915/original.jpg	https://data.globe.gov/system/photos/2019/06/28/1119914/original.jpg	https://data.globe.gov/system/photos/2019/06/28/1119916/original.jpg	https://data.globe.gov/system/photos/2019/06/28/1119918/original.jpg
GV_834	46.1921	-119.0492	106.2		https://www.google.com/maps/search/46.1921,-119.0492	https://data.globe.gov/system/photos/2019/06/28/1119919/original.jpg	https://data.globe.gov/system/photos/2019/06/28/1119921/original.jpg	https://data.globe.gov/system/photos/2019/06/28/1119920/original.jpg	https://data.globe.gov/system/photos/2019/06/28/1119922/original.jpg	https://data.globe.gov/system/photos/2019/06/28/1119924/original.jpg
GV_835	46.1847	-119.033	104.9		https://www.google.com/maps/search/46.1847,-119.033	https://data.globe.gov/system/photos/2019/06/28/1119925/original.jpg	https://data.globe.gov/system/photos/2019/06/28/1119927/original.jpg	https://data.globe.gov/system/photos/2019/06/28/1119926/original.jpg	https://data.globe.gov/system/photos/2019/06/28/1119928/original.jpg	https://data.globe.gov/system/photos/2019/06/28/1119930/original.jpg
GV_836	46.1844	-119.032	104.3		https://www.google.com/maps/search/46.1844,-119.032	https://data.globe.gov/system/photos/2019/06/28/1119931/original.jpg	https://data.globe.gov/system/photos/2019/06/28/1119933/original.jpg	https://data.globe.gov/system/photos/2019/06/28/1119932/original.jpg	https://data.globe.gov/system/photos/2019/06/28/1119934/original.jpg	https://data.globe.gov/system/photos/2019/06/28/1119936/original.jpg
GV_837	46.1844	-119.0334	106.3		https://www.google.com/maps/search/46.1844,-119.0334	https://data.globe.gov/system/photos/2019/06/28/1119937/original.jpg	https://data.globe.gov/system/photos/2019/06/28/1119939/original.jpg	https://data.globe.gov/system/photos/2019/06/28/1119938/original.jpg	https://data.globe.gov/system/photos/2019/06/28/1119940/original.jpg	https://data.globe.gov/system/photos/2019/06/28/1119942/original.jpg
GV_838	46.1979	-119.1283	119.9		https://www.google.com/maps/search/46.1979,-119.1283	https://data.globe.gov/system/photos/2019/06/28/1119943/original.jpg	https://data.globe.gov/system/photos/2019/06/28/1119946/original.jpg	https://data.globe.gov/system/photos/2019/06/28/1119945/original.jpg	https://data.globe.gov/system/photos/2019/06/28/1119947/original.jpg	https://data.globe.gov/system/photos/2019/06/28/1119950/original.jpg
GV_839	46.1966	-119.1291	120.2		https://www.google.com/maps/search/46.1966,-119.1291	https://data.globe.gov/system/photos/2019/06/28/1119951/original.jpg	https://data.globe.gov/system/photos/2019/06/28/1119954/original.jpg	https://data.globe.gov/system/photos/2019/06/28/1119952/original.jpg	https://data.globe.gov/system/photos/2019/06/28/1119955/original.jpg	https://data.globe.gov/system/photos/2019/06/28/1119958/original.jpg
GV_840	45.9308	-119.348	81.6		https://www.google.com/maps/search/45.9308,-119.348	https://data.globe.gov/system/photos/2019/06/28/1119960/original.jpg	https://data.globe.gov/system/photos/2019/06/28/1119962/original.jpg	https://data.globe.gov/system/photos/2019/06/28/1119961/original.jpg	https://data.globe.gov/system/photos/2019/06/28/1119963/original.jpg	https://data.globe.gov/system/photos/2019/06/28/1119966/original.jpg
GV_841	45.93	-119.3477	84.1		https://www.google.com/maps/search/45.93,-119.3477	https://data.globe.gov/system/photos/2019/06/28/1119967/original.jpg	https://data.globe.gov/system/photos/2019/06/28/1119969/original.jpg	https://data.globe.gov/system/photos/2019/06/28/1119968/original.jpg	https://data.globe.gov/system/photos/2019/06/28/1119970/original.jpg	https://data.globe.gov/system/photos/2019/06/28/1119972/original.jpg
GV_842	45.9315	-119.4666	115.8		https://www.google.com/maps/search/45.9315,-119.4666	https://data.globe.gov/system/photos/2019/06/28/1119973/original.jpg	https://data.globe.gov/system/photos/2019/06/28/1119975/original.jpg	https://data.globe.gov/system/photos/2019/06/28/1119974/original.jpg	https://data.globe.gov/system/photos/2019/06/28/1119976/original.jpg	https://data.globe.gov/system/photos/2019/06/28/1119978/original.jpg
GV_843	45.9154	-119.6569	125.1		https://www.google.com/maps/search/45.9154,-119.6569	https://data.globe.gov/system/photos/2019/06/28/1119979/original.jpg	https://data.globe.gov/system/photos/2019/06/28/1119981/original.jpg	https://data.globe.gov/system/photos/2019/06/28/1119980/original.jpg	https://data.globe.gov/system/photos/2019/06/28/1119982/original.jpg	https://data.globe.gov/system/photos/2019/06/28/1119984/original.jpg
GV_844	45.9175	-119.6475	92.4		https://www.google.com/maps/search/45.9175,-119.6475	https://data.globe.gov/system/photos/2019/06/28/1119985/original.jpg	https://data.globe.gov/system/photos/2019/06/28/1119987/original.jpg	https://data.globe.gov/system/photos/2019/06/28/1119986/original.jpg	https://data.globe.gov/system/photos/2019/06/28/1119988/original.jpg	https://data.globe.gov/system/photos/2019/06/28/1119990/original.jpg
GV_845	45.9205	-119.6403	87.7		https://www.google.com/maps/search/45.9205,-119.6403	https://data.globe.gov/system/photos/2019/06/28/1119991/original.jpg	https://data.globe.gov/system/photos/2019/06/28/1119993/original.jpg	https://data.globe.gov/system/photos/2019/06/28/1119992/original.jpg	https://data.globe.gov/system/photos/2019/06/28/1119994/original.jpg	https://data.globe.gov/system/photos/2019/06/28/1119996/original.jpg
GV_846	45.9201	-119.6397	85.3		https://www.google.com/maps/search/45.9201,-119.6397	https://data.globe.gov/system/photos/2019/06/28/1119997/original.jpg	https://data.globe.gov/system/photos/2019/06/28/1119999/original.jpg	https://data.globe.gov/system/photos/2019/06/28/1119998/original.jpg	https://data.globe.gov/system/photos/2019/06/28/1120000/original.jpg	https://data.globe.gov/system/photos/2019/06/28/1120002/original.jpg
GV_847	45.873	-119.7438	84.7		https://www.google.com/maps/search/45.873,-119.7438	https://data.globe.gov/system/photos/2019/06/28/1120003/original.jpg	https://data.globe.gov/system/photos/2019/06/28/1120005/original.jpg	https://data.globe.gov/system/photos/2019/06/28/1120004/original.jpg	https://data.globe.gov/system/photos/2019/06/28/1120006/original.jpg	https://data.globe.gov/system/photos/2019/06/28/1120008/original.jpg
GV_848	45.8726	-119.7307	87.1		https://www.google.com/maps/search/45.8726,-119.7307	https://data.globe.gov/system/photos/2019/06/28/1120009/original.jpg	https://data.globe.gov/system/photos/2019/06/28/1120011/original.jpg	https://data.globe.gov/system/photos/2019/06/28/1120010/original.jpg	https://data.globe.gov/system/photos/2019/06/28/1120012/original.jpg	https://data.globe.gov/system/photos/2019/06/28/1120014/original.jpg
GV_849	45.8714	-119.7423	86.9		https://www.google.com/maps/search/45.8714,-119.7423	https://data.globe.gov/system/photos/2019/06/28/1120015/original.jpg	https://data.globe.gov/system/photos/2019/06/28/1120017/original.jpg	https://data.globe.gov/system/photos/2019/06/28/1120016/original.jpg	https://data.globe.gov/system/photos/2019/06/28/1120018/original.jpg	https://data.globe.gov/system/photos/2019/06/28/1120020/original.jpg
GV_850	45.8661	-119.7431	90.3		https://www.google.com/maps/search/45.8661,-119.7431	https://data.globe.gov/system/photos/2019/06/28/1120021/original.jpg	https://data.globe.gov/system/photos/2019/06/28/1120023/original.jpg	https://data.globe.gov/system/photos/2019/06/28/1120022/original.jpg	https://data.globe.gov/system/photos/2019/06/28/1120024/original.jpg	https://data.globe.gov/system/photos/2019/06/28/1120026/original.jpg
GV_851	45.8353	-119.9295	81.5		https://www.google.com/maps/search/45.8353,-119.9295	https://data.globe.gov/system/photos/2019/06/28/1120027/original.jpg	https://data.globe.gov/system/photos/2019/06/28/1120029/original.jpg	https://data.globe.gov/system/photos/2019/06/28/1120028/original.jpg	https://data.globe.gov/system/photos/2019/06/28/1120030/original.jpg	https://data.globe.gov/system/photos/2019/06/28/1120032/original.jpg
GV_852	45.8353	-119.931	84.5		https://www.google.com/maps/search/45.8353,-119.931	https://data.globe.gov/system/photos/2019/06/28/1120033/original.jpg	https://data.globe.gov/system/photos/2019/06/28/1120035/original.jpg	https://data.globe.gov/system/photos/2019/06/28/1120034/original.jpg	https://data.globe.gov/system/photos/2019/06/28/1120036/original.jpg	https://data.globe.gov/system/photos/2019/06/28/1120038/original.jpg
GV_853	45.8355	-119.9311	85.1		https://www.google.com/maps/search/45.8355,-119.9311	https://data.globe.gov/system/photos/2019/06/28/1120039/original.jpg	https://data.globe.gov/system/photos/2019/06/28/1120041/original.jpg	https://data.globe.gov/system/photos/2019/06/28/1120040/original.jpg	https://data.globe.gov/system/photos/2019/06/28/1120042/original.jpg	https://data.globe.gov/system/photos/2019/06/28/1120044/original.jpg
GV_854	45.8134	-120.0249	106.9		https://www.google.com/maps/search/45.8134,-120.0249	https://data.globe.gov/system/photos/2019/06/28/1120045/original.jpg	https://data.globe.gov/system/photos/2019/06/28/1120047/original.jpg	https://data.globe.gov/system/photos/2019/06/28/1120046/original.jpg	https://data.globe.gov/system/photos/2019/06/28/1120048/original.jpg	https://data.globe.gov/system/photos/2019/06/28/1120050/original.jpg
GV_855	45.7112	-120.3647	148.3		https://www.google.com/maps/search/45.7112,-120.3647	https://data.globe.gov/system/photos/2019/06/28/1120051/original.jpg	https://data.globe.gov/system/photos/2019/06/28/1120053/original.jpg	https://data.globe.gov/system/photos/2019/06/28/1120052/original.jpg	https://data.globe.gov/system/photos/2019/06/28/1120054/original.jpg	https://data.globe.gov/system/photos/2019/06/28/1120056/original.jpg
GV_856	45.7041	-120.4476	90.7		https://www.google.com/maps/search/45.7041,-120.4476	https://data.globe.gov/system/photos/2019/06/28/1120057/original.jpg	https://data.globe.gov/system/photos/2019/06/28/1120059/original.jpg	https://data.globe.gov/system/photos/2019/06/28/1120058/original.jpg	https://data.globe.gov/system/photos/2019/06/28/1120060/original.jpg	https://data.globe.gov/system/photos/2019/06/28/1120062/original.jpg
GV_857	45.7524	-120.63	114.7		https://www.google.com/maps/search/45.7524,-120.63	https://data.globe.gov/system/photos/2019/06/28/1120063/original.jpg	https://data.globe.gov/system/photos/2019/06/28/1120065/original.jpg	https://data.globe.gov/system/photos/2019/06/28/1120064/original.jpg	https://data.globe.gov/system/photos/2019/06/28/1120066/original.jpg	https://data.globe.gov/system/photos/2019/06/28/1120068/original.jpg
GV_858	45.6941	-120.8058	176.5		https://www.google.com/maps/search/45.6941,-120.8058	https://data.globe.gov/system/photos/2019/06/28/1120069/original.jpg	https://data.globe.gov/system/photos/2019/06/28/1120071/original.jpg	https://data.globe.gov/system/photos/2019/06/28/1120070/original.jpg	https://data.globe.gov/system/photos/2019/06/28/1120072/original.jpg	https://data.globe.gov/system/photos/2019/06/28/1120074/original.jpg
GV_859	45.6779	-120.8631	234.5		https://www.google.com/maps/search/45.6779,-120.8631	https://data.globe.gov/system/photos/2019/06/28/1120075/original.jpg	https://data.globe.gov/system/photos/2019/06/28/1120077/original.jpg	https://data.globe.gov/system/photos/2019/06/28/1120076/original.jpg	https://data.globe.gov/system/photos/2019/06/28/1120078/original.jpg	https://data.globe.gov/system/photos/2019/06/28/1120080/original.jpg
GV_860	45.677	-120.8646	233.5		https://www.google.com/maps/search/45.677,-120.8646	https://data.globe.gov/system/photos/2019/06/28/1120081/original.jpg	https://data.globe.gov/system/photos/2019/06/28/1120083/original.jpg	https://data.globe.gov/system/photos/2019/06/28/1120082/original.jpg	https://data.globe.gov/system/photos/2019/06/28/1120084/original.jpg	https://data.globe.gov/system/photos/2019/06/28/1120086/original.jpg
GV_861	46.5384	-117.0871	784.6		https://www.google.com/maps/search/46.5384,-117.0871	https://data.globe.gov/system/photos/2019/06/28/1120087/original.jpg	https://data.globe.gov/system/photos/2019/06/28/1120089/original.jpg	https://data.globe.gov/system/photos/2019/06/28/1120088/original.jpg	https://data.globe.gov/system/photos/2019/06/28/1120090/original.jpg	https://data.globe.gov/system/photos/2019/06/28/1120092/original.jpg
GV_862	46.5425	-117.0885	778.6		https://www.google.com/maps/search/46.5425,-117.0885	https://data.globe.gov/system/photos/2019/06/28/1120093/original.jpg	https://data.globe.gov/system/photos/2019/06/28/1120095/original.jpg	https://data.globe.gov/system/photos/2019/06/28/1120094/original.jpg	https://data.globe.gov/system/photos/2019/06/28/1120096/original.jpg	https://data.globe.gov/system/photos/2019/06/28/1120098/original.jpg
GV_863	46.5679	-117.1342	775.3		https://www.google.com/maps/search/46.5679,-117.1342	https://data.globe.gov/system/photos/2019/06/28/1120099/original.jpg	https://data.globe.gov/system/photos/2019/06/28/1120101/original.jpg	https://data.globe.gov/system/photos/2019/06/28/1120100/original.jpg	https://data.globe.gov/system/photos/2019/06/28/1120102/original.jpg	https://data.globe.gov/system/photos/2019/06/28/1120104/original.jpg
GV_864	46.6616	-117.1928	818.9		https://www.google.com/maps/search/46.6616,-117.1928	https://data.globe.gov/system/photos/2019/06/28/1120105/original.jpg	https://data.globe.gov/system/photos/2019/06/28/1120107/original.jpg	https://data.globe.gov/system/photos/2019/06/28/1120106/original.jpg	https://data.globe.gov/system/photos/2019/06/28/1120108/original.jpg	https://data.globe.gov/system/photos/2019/06/28/1120110/original.jpg
GV_865	46.6756	-117.1947	787.8		https://www.google.com/maps/search/46.6756,-117.1947	https://data.globe.gov/system/photos/2019/06/28/1120111/original.jpg	https://data.globe.gov/system/photos/2019/06/28/1120113/original.jpg	https://data.globe.gov/system/photos/2019/06/28/1120112/original.jpg	https://data.globe.gov/system/photos/2019/06/28/1120114/original.jpg	https://data.globe.gov/system/photos/2019/06/28/1120116/original.jpg
GV_866	46.7091	-117.2028	761.1		https://www.google.com/maps/search/46.7091,-117.2028	https://data.globe.gov/system/photos/2019/06/28/1120117/original.jpg	https://data.globe.gov/system/photos/2019/06/28/1120119/original.jpg	https://data.globe.gov/system/photos/2019/06/28/1120118/original.jpg	https://data.globe.gov/system/photos/2019/06/28/1120120/original.jpg	https://data.globe.gov/system/photos/2019/06/28/1120122/original.jpg
GV_867	46.3441	-118.9513	178.6		https://www.google.com/maps/search/46.3441,-118.9513	https://data.globe.gov/system/photos/2019/06/28/1120123/original.jpg	https://data.globe.gov/system/photos/2019/06/28/1120125/original.jpg	https://data.globe.gov/system/photos/2019/06/28/1120124/original.jpg	https://data.globe.gov/system/photos/2019/06/28/1120126/original.jpg	https://data.globe.gov/system/photos/2019/06/28/1120128/original.jpg
GV_868	46.3441	-118.9492	180.1		https://www.google.com/maps/search/46.3441,-118.9492	https://data.globe.gov/system/photos/2019/06/28/1120129/original.jpg	https://data.globe.gov/system/photos/2019/06/28/1120131/original.jpg	https://data.globe.gov/system/photos/2019/06/28/1120130/original.jpg	https://data.globe.gov/system/photos/2019/06/28/1120132/original.jpg	https://data.globe.gov/system/photos/2019/06/28/1120134/original.jpg
GV_869	46.3453	-118.9476	182.8		https://www.google.com/maps/search/46.3453,-118.9476	https://data.globe.gov/system/photos/2019/06/28/1120135/original.jpg	https://data.globe.gov/system/photos/2019/06/28/1120137/original.jpg	https://data.globe.gov/system/photos/2019/06/28/1120136/original.jpg	https://data.globe.gov/system/photos/2019/06/28/1120138/original.jpg	https://data.globe.gov/system/photos/2019/06/28/1120140/original.jpg
GV_870	46.3435	-118.9473	183		https://www.google.com/maps/search/46.3435,-118.9473	https://data.globe.gov/system/photos/2019/06/28/1120141/original.jpg	https://data.globe.gov/system/photos/2019/06/28/1120143/original.jpg	https://data.globe.gov/system/photos/2019/06/28/1120142/original.jpg	https://data.globe.gov/system/photos/2019/06/28/1120144/original.jpg	https://data.globe.gov/system/photos/2019/06/28/1120146/original.jpg
GV_871	46.3445	-118.9386	215.9		https://www.google.com/maps/search/46.3445,-118.9386	https://data.globe.gov/system/photos/2019/06/28/1120147/original.jpg	https://data.globe.gov/system/photos/2019/06/28/1120149/original.jpg	https://data.globe.gov/system/photos/2019/06/28/1120148/original.jpg	https://data.globe.gov/system/photos/2019/06/28/1120150/original.jpg	https://data.globe.gov/system/photos/2019/06/28/1120152/original.jpg
GV_872	46.3451	-118.9407	201.2		https://www.google.com/maps/search/46.3451,-118.9407	https://data.globe.gov/system/photos/2019/06/28/1120153/original.jpg	https://data.globe.gov/system/photos/2019/06/28/1120155/original.jpg	https://data.globe.gov/system/photos/2019/06/28/1120154/original.jpg	https://data.globe.gov/system/photos/2019/06/28/1120156/original.jpg	https://data.globe.gov/system/photos/2019/06/28/1120158/original.jpg
GV_873	46.3451	-118.9394	208.1		https://www.google.com/maps/search/46.3451,-118.9394	https://data.globe.gov/system/photos/2019/06/28/1120159/original.jpg	https://data.globe.gov/system/photos/2019/06/28/1120161/original.jpg	https://data.globe.gov/system/photos/2019/06/28/1120160/original.jpg	https://data.globe.gov/system/photos/2019/06/28/1120162/original.jpg	https://data.globe.gov/system/photos/2019/06/28/1120164/original.jpg
GV_874	46.3437	-118.9511	178		https://www.google.com/maps/search/46.3437,-118.9511	https://data.globe.gov/system/photos/2019/06/28/1120165/original.jpg	https://data.globe.gov/system/photos/2019/06/28/1120167/original.jpg	https://data.globe.gov/system/photos/2019/06/28/1120166/original.jpg	https://data.globe.gov/system/photos/2019/06/28/1120168/original.jpg	https://data.globe.gov/system/photos/2019/06/28/1120170/original.jpg
GV_875	46.3413	-118.9519	178.1		https://www.google.com/maps/search/46.3413,-118.9519	https://data.globe.gov/system/photos/2019/06/28/1120171/original.jpg	https://data.globe.gov/system/photos/2019/06/28/1120173/original.jpg	https://data.globe.gov/system/photos/2019/06/28/1120172/original.jpg	https://data.globe.gov/system/photos/2019/06/28/1120174/original.jpg	https://data.globe.gov/system/photos/2019/06/28/1120176/original.jpg
GV_876	46.3396	-118.9521	175.1		https://www.google.com/maps/search/46.3396,-118.9521	https://data.globe.gov/system/photos/2019/06/28/1120177/original.jpg	https://data.globe.gov/system/photos/2019/06/28/1120179/original.jpg	https://data.globe.gov/system/photos/2019/06/28/1120178/original.jpg	https://data.globe.gov/system/photos/2019/06/28/1120180/original.jpg	https://data.globe.gov/system/photos/2019/06/28/1120182/original.jpg
GV_877	46.3367	-118.9519	175		https://www.google.com/maps/search/46.3367,-118.9519	https://data.globe.gov/system/photos/2019/06/28/1120183/original.jpg	https://data.globe.gov/system/photos/2019/06/28/1120185/original.jpg	https://data.globe.gov/system/photos/2019/06/28/1120184/original.jpg	https://data.globe.gov/system/photos/2019/06/28/1120186/original.jpg	https://data.globe.gov/system/photos/2019/06/28/1120188/original.jpg
GV_878	46.3366	-118.9532	171.5		https://www.google.com/maps/search/46.3366,-118.9532	https://data.globe.gov/system/photos/2019/06/28/1120189/original.jpg	https://data.globe.gov/system/photos/2019/06/28/1120191/original.jpg	https://data.globe.gov/system/photos/2019/06/28/1120190/original.jpg	https://data.globe.gov/system/photos/2019/06/28/1120192/original.jpg	https://data.globe.gov/system/photos/2019/06/28/1120194/original.jpg
GV_879	46.3371	-118.9528	171.7		https://www.google.com/maps/search/46.3371,-118.9528	https://data.globe.gov/system/photos/2019/06/28/1120195/original.jpg	https://data.globe.gov/system/photos/2019/06/28/1120197/original.jpg	https://data.globe.gov/system/photos/2019/06/28/1120196/original.jpg	https://data.globe.gov/system/photos/2019/06/28/1120198/original.jpg	https://data.globe.gov/system/photos/2019/06/28/1120200/original.jpg
GV_880	46.3329	-118.9521	176.3		https://www.google.com/maps/search/46.3329,-118.9521	https://data.globe.gov/system/photos/2019/06/28/1120201/original.jpg	https://data.globe.gov/system/photos/2019/06/28/1120203/original.jpg	https://data.globe.gov/system/photos/2019/06/28/1120202/original.jpg	https://data.globe.gov/system/photos/2019/06/28/1120204/original.jpg	https://data.globe.gov/system/photos/2019/06/28/1120206/original.jpg
GV_881	46.3266	-118.952	183.2		https://www.google.com/maps/search/46.3266,-118.952	https://data.globe.gov/system/photos/2019/06/28/1120207/original.jpg	https://data.globe.gov/system/photos/2019/06/28/1120209/original.jpg	https://data.globe.gov/system/photos/2019/06/28/1120208/original.jpg	https://data.globe.gov/system/photos/2019/06/28/1120210/original.jpg	https://data.globe.gov/system/photos/2019/06/28/1120212/original.jpg
GV_882	46.3102	-118.952	190.2		https://www.google.com/maps/search/46.3102,-118.952	https://data.globe.gov/system/photos/2019/06/28/1120213/original.jpg	https://data.globe.gov/system/photos/2019/06/28/1120215/original.jpg	https://data.globe.gov/system/photos/2019/06/28/1120214/original.jpg	https://data.globe.gov/system/photos/2019/06/28/1120216/original.jpg	https://data.globe.gov/system/photos/2019/06/28/1120218/original.jpg
GV_883	46.3071	-118.952	190.5		https://www.google.com/maps/search/46.3071,-118.952	https://data.globe.gov/system/photos/2019/06/28/1120219/original.jpg	https://data.globe.gov/system/photos/2019/06/28/1120221/original.jpg	https://data.globe.gov/system/photos/2019/06/28/1120220/original.jpg	https://data.globe.gov/system/photos/2019/06/28/1120222/original.jpg	https://data.globe.gov/system/photos/2019/06/28/1120224/original.jpg
GV_884	46.2943	-118.952	196.5		https://www.google.com/maps/search/46.2943,-118.952	https://data.globe.gov/system/photos/2019/06/28/1120225/original.jpg	https://data.globe.gov/system/photos/2019/06/28/1120227/original.jpg	https://data.globe.gov/system/photos/2019/06/28/1120226/original.jpg	https://data.globe.gov/system/photos/2019/06/28/1120228/original.jpg	https://data.globe.gov/system/photos/2019/06/28/1120230/original.jpg
GV_885	46.2878	-118.9517	198.6		https://www.google.com/maps/search/46.2878,-118.9517	https://data.globe.gov/system/photos/2019/06/28/1120231/original.jpg	https://data.globe.gov/system/photos/2019/06/28/1120233/original.jpg	https://data.globe.gov/system/photos/2019/06/28/1120232/original.jpg	https://data.globe.gov/system/photos/2019/06/28/1120234/original.jpg	https://data.globe.gov/system/photos/2019/06/28/1120236/original.jpg
GV_886	46.2037	-119.0378	105.3		https://www.google.com/maps/search/46.2037,-119.0378	https://data.globe.gov/system/photos/2019/06/28/1120237/original.jpg	https://data.globe.gov/system/photos/2019/06/28/1120239/original.jpg	https://data.globe.gov/system/photos/2019/06/28/1120238/original.jpg	https://data.globe.gov/system/photos/2019/06/28/1120240/original.jpg	https://data.globe.gov/system/photos/2019/06/28/1120242/original.jpg
GV_887	46.1989	-119.0403	105.3		https://www.google.com/maps/search/46.1989,-119.0403	https://data.globe.gov/system/photos/2019/06/28/1120243/original.jpg	https://data.globe.gov/system/photos/2019/06/28/1120245/original.jpg	https://data.globe.gov/system/photos/2019/06/28/1120244/original.jpg	https://data.globe.gov/system/photos/2019/06/28/1120246/original.jpg	https://data.globe.gov/system/photos/2019/06/28/1120248/original.jpg
GV_888	46.1988	-119.0392	106.9		https://www.google.com/maps/search/46.1988,-119.0392	https://data.globe.gov/system/photos/2019/06/28/1120249/original.jpg	https://data.globe.gov/system/photos/2019/06/28/1120251/original.jpg	https://data.globe.gov/system/photos/2019/06/28/1120250/original.jpg	https://data.globe.gov/system/photos/2019/06/28/1120252/original.jpg	https://data.globe.gov/system/photos/2019/06/28/1120254/original.jpg
GV_889	46.2016	-119.0369	104.5		https://www.google.com/maps/search/46.2016,-119.0369	https://data.globe.gov/system/photos/2019/06/28/1120255/original.jpg	https://data.globe.gov/system/photos/2019/06/28/1120257/original.jpg	https://data.globe.gov/system/photos/2019/06/28/1120256/original.jpg	https://data.globe.gov/system/photos/2019/06/28/1120258/original.jpg	https://data.globe.gov/system/photos/2019/06/28/1120260/original.jpg
GV_890	46.2024	-119.0371	106.1		https://www.google.com/maps/search/46.2024,-119.0371	https://data.globe.gov/system/photos/2019/06/28/1120261/original.jpg	https://data.globe.gov/system/photos/2019/06/28/1120263/original.jpg	https://data.globe.gov/system/photos/2019/06/28/1120262/original.jpg	https://data.globe.gov/system/photos/2019/06/28/1120264/original.jpg	https://data.globe.gov/system/photos/2019/06/28/1120266/original.jpg
GV_891	46.2047	-119.0375	105.1		https://www.google.com/maps/search/46.2047,-119.0375	https://data.globe.gov/system/photos/2019/06/28/1120267/original.jpg	https://data.globe.gov/system/photos/2019/06/28/1120269/original.jpg	https://data.globe.gov/system/photos/2019/06/28/1120268/original.jpg	https://data.globe.gov/system/photos/2019/06/28/1120270/original.jpg	https://data.globe.gov/system/photos/2019/06/28/1120272/original.jpg
GV_892	46.1897	-119.0529	106.9		https://www.google.com/maps/search/46.1897,-119.0529	https://data.globe.gov/system/photos/2019/06/28/1120273/original.jpg	https://data.globe.gov/system/photos/2019/06/28/1120275/original.jpg	https://data.globe.gov/system/photos/2019/06/28/1120274/original.jpg	https://data.globe.gov/system/photos/2019/06/28/1120276/original.jpg	https://data.globe.gov/system/photos/2019/06/28/1120278/original.jpg
GV_893	46.1919	-119.0486	104.4		https://www.google.com/maps/search/46.1919,-119.0486	https://data.globe.gov/system/photos/2019/06/28/1120279/original.jpg	https://data.globe.gov/system/photos/2019/06/28/1120281/original.jpg	https://data.globe.gov/system/photos/2019/06/28/1120280/original.jpg	https://data.globe.gov/system/photos/2019/06/28/1120282/original.jpg	https://data.globe.gov/system/photos/2019/06/28/1120284/original.jpg

GV_894	46.191	-119.052	105.4		https://www.google.com/maps/search/46.191,-119.052	https://data.globe.gov/system/photos/2019/06/28/1120285/original.jpg	https://data.globe.gov/system/photos/2019/06/28/1120287/original.jpg	https://data.globe.gov/system/photos/2019/06/28/1120286/original.jpg	https://data.globe.gov/system/photos/2019/06/28/1120288/original.jpg	https://data.globe.gov/system/photos/2019/06/28/1120290/original.jpg
GV_895	46.1973	-119.1289	119.7		https://www.google.com/maps/search/46.1973,-119.1289	https://data.globe.gov/system/photos/2019/06/28/1120291/original.jpg	https://data.globe.gov/system/photos/2019/06/28/1120293/original.jpg	https://data.globe.gov/system/photos/2019/06/28/1120292/original.jpg	https://data.globe.gov/system/photos/2019/06/28/1120294/original.jpg	https://data.globe.gov/system/photos/2019/06/28/1120296/original.jpg
GV_896	46.1966	-119.1283	119.9		https://www.google.com/maps/search/46.1966,-119.1283	https://data.globe.gov/system/photos/2019/06/28/1120297/original.jpg	https://data.globe.gov/system/photos/2019/06/28/1120299/original.jpg	https://data.globe.gov/system/photos/2019/06/28/1120298/original.jpg	https://data.globe.gov/system/photos/2019/06/28/1120300/original.jpg	https://data.globe.gov/system/photos/2019/06/28/1120302/original.jpg
GV_897	45.9299	-119.3473	82.3		https://www.google.com/maps/search/45.9299,-119.3473	https://data.globe.gov/system/photos/2019/06/28/1120303/original.jpg	https://data.globe.gov/system/photos/2019/06/28/1120305/original.jpg	https://data.globe.gov/system/photos/2019/06/28/1120304/original.jpg	https://data.globe.gov/system/photos/2019/06/28/1120306/original.jpg	https://data.globe.gov/system/photos/2019/06/28/1120308/original.jpg
GV_898	45.9283	-119.3972	86.9		https://www.google.com/maps/search/45.9283,-119.3972	https://data.globe.gov/system/photos/2019/06/28/1120309/original.jpg	https://data.globe.gov/system/photos/2019/06/28/1120311/original.jpg	https://data.globe.gov/system/photos/2019/06/28/1120310/original.jpg	https://data.globe.gov/system/photos/2019/06/28/1120312/original.jpg	https://data.globe.gov/system/photos/2019/06/28/1120314/original.jpg
GV_899	24.4361	118.3674	17.9		https://www.google.com/maps/search/24.4361,118.3674	https://data.globe.gov/system/photos/2019/06/29/1120378/original.jpg	https://data.globe.gov/system/photos/2019/06/29/1120380/original.jpg	https://data.globe.gov/system/photos/2019/06/29/1120379/original.jpg	https://data.globe.gov/system/photos/2019/06/29/1120382/original.jpg	https://data.globe.gov/system/photos/2019/06/29/1120384/original.jpg
GV_900	30.0724	-99.2446	517.4		https://www.google.com/maps/search/30.0724,-99.2446	https://data.globe.gov/system/photos/2019/06/29/1120470/original.jpg	https://data.globe.gov/system/photos/2019/06/29/1120472/original.jpg	https://data.globe.gov/system/photos/2019/06/29/1120471/original.jpg	https://data.globe.gov/system/photos/2019/06/29/1120473/original.jpg	https://data.globe.gov/system/photos/2019/06/29/1120475/original.jpg
GV_901	-31.3627	116.1074	141.5		https://www.google.com/maps/search/-31.3627,116.1074	https://data.globe.gov/system/photos/2019/06/21/1120506/original.jpg	https://data.globe.gov/system/photos/2019/06/21/1120508/original.jpg	https://data.globe.gov/system/photos/2019/06/21/1120507/original.jpg	https://data.globe.gov/system/photos/2019/06/21/1120509/original.jpg	https://data.globe.gov/system/photos/2019/06/21/1120511/original.jpg
GV_902	-31.0866	116.4541	265.9		https://www.google.com/maps/search/-31.0866,116.4541	https://data.globe.gov/system/photos/2019/06/28/1120512/original.jpg	https://data.globe.gov/system/photos/2019/06/28/1120514/original.jpg	https://data.globe.gov/system/photos/2019/06/28/1120513/original.jpg	https://data.globe.gov/system/photos/2019/06/28/1120516/original.jpg	https://data.globe.gov/system/photos/2019/06/28/1120518/original.jpg
GV_903	-31.1081	116.3984	259.9		https://www.google.com/maps/search/-31.1081,116.3984	https://data.globe.gov/system/photos/2019/06/28/1120519/original.jpg	https://data.globe.gov/system/photos/2019/06/28/1120521/original.jpg	https://data.globe.gov/system/photos/2019/06/28/1120520/original.jpg	https://data.globe.gov/system/photos/2019/06/28/1120522/original.jpg	https://data.globe.gov/system/photos/2019/06/28/1120524/original.jpg
GV_904	-31.1432	116.3436	285.3		https://www.google.com/maps/search/-31.1432,116.3436	https://data.globe.gov/system/photos/2019/06/28/1120525/original.jpg	https://data.globe.gov/system/photos/2019/06/28/1120527/original.jpg	https://data.globe.gov/system/photos/2019/06/28/1120526/original.jpg	https://data.globe.gov/system/photos/2019/06/28/1120528/original.jpg	https://data.globe.gov/system/photos/2019/06/28/1120530/original.jpg
GV_905	-31.1598	116.1831	283.4		https://www.google.com/maps/search/-31.1598,116.1831	https://data.globe.gov/system/photos/2019/06/28/1120531/original.jpg	https://data.globe.gov/system/photos/2019/06/28/1120533/original.jpg	https://data.globe.gov/system/photos/2019/06/28/1120532/original.jpg	https://data.globe.gov/system/photos/2019/06/28/1120534/original.jpg	https://data.globe.gov/system/photos/2019/06/28/1120536/original.jpg
GV_906	-34.3789	-58.8918	7.6		https://www.google.com/maps/search/-34.3789,-58.8918	https://data.globe.gov/system/photos/2019/06/29/1121007/original.jpg	https://data.globe.gov/system/photos/2019/06/29/1121009/original.jpg	https://data.globe.gov/system/photos/2019/06/29/1121008/original.jpg	https://data.globe.gov/system/photos/2019/06/29/1121010/original.jpg	https://data.globe.gov/system/photos/2019/06/29/1121012/original.jpg
GV_907	24.441	118.3633	39.6		https://www.google.com/maps/search/24.441,118.3633	https://data.globe.gov/system/photos/2019/06/30/1121065/original.jpg	https://data.globe.gov/system/photos/2019/06/30/1121067/original.jpg	https://data.globe.gov/system/photos/2019/06/30/1121066/original.jpg	https://data.globe.gov/system/photos/2019/06/30/1121068/original.jpg	https://data.globe.gov/system/photos/2019/06/30/1121070/original.jpg
GV_908	37.3056	-122.0659	170.7		https://www.google.com/maps/search/37.3056,-122.0659	https://data.globe.gov/system/photos/2019/06/30/1121317/original.jpg	https://data.globe.gov/system/photos/2019/06/30/1121319/original.jpg	https://data.globe.gov/system/photos/2019/06/30/1121318/original.jpg	https://data.globe.gov/system/photos/2019/06/30/1121320/original.jpg	https://data.globe.gov/system/photos/2019/06/30/1121322/original.jpg
GV_909	45.551	-110.9629	1775.9		https://www.google.com/maps/search/45.551,-110.9629	https://data.globe.gov/system/photos/2019/06/30/1121447/original.jpg	https://data.globe.gov/system/photos/2019/06/30/1121449/original.jpg	https://data.globe.gov/system/photos/2019/06/30/1121448/original.jpg	https://data.globe.gov/system/photos/2019/06/30/1121450/original.jpg	https://data.globe.gov/system/photos/2019/06/30/1121453/original.jpg
GV_910	45.4868	-111.1288	2353.3		https://www.google.com/maps/search/45.4868,-111.1288	https://data.globe.gov/system/photos/2019/06/30/1121454/original.jpg	https://data.globe.gov/system/photos/2019/06/30/1121456/original.jpg	https://data.globe.gov/system/photos/2019/06/30/1121455/original.jpg	https://data.globe.gov/system/photos/2019/06/30/1121457/original.jpg	https://data.globe.gov/system/photos/2019/06/30/1121459/original.jpg
GV_911	45.1646	-122.123	288		https://www.google.com/maps/search/45.1646,-122.123	https://data.globe.gov/system/photos/2019/06/30/1121727/original.jpg	https://data.globe.gov/system/photos/2019/06/30/1121729/original.jpg	https://data.globe.gov/system/photos/2019/06/30/1121728/original.jpg	https://data.globe.gov/system/photos/2019/06/30/1121730/original.jpg	https://data.globe.gov/system/photos/2019/06/30/1121732/original.jpg
GV_912	45.1644	-122.1224	292.8		https://www.google.com/maps/search/45.1644,-122.1224	https://data.globe.gov/system/photos/2019/06/30/1121739/original.jpg	https://data.globe.gov/system/photos/2019/06/30/1121741/original.jpg	https://data.globe.gov/system/photos/2019/06/30/1121740/original.jpg	https://data.globe.gov/system/photos/2019/06/30/1121742/original.jpg	https://data.globe.gov/system/photos/2019/06/30/1121744/original.jpg
GV_913	45.1367	-122.082	333.3		https://www.google.com/maps/search/45.1367,-122.082	https://data.globe.gov/system/photos/2019/06/30/1121745/original.jpg	https://data.globe.gov/system/photos/2019/06/30/1121747/original.jpg	https://data.globe.gov/system/photos/2019/06/30/1121746/original.jpg	https://data.globe.gov/system/photos/2019/06/30/1121748/original.jpg	https://data.globe.gov/system/photos/2019/06/30/1121750/original.jpg
GV_914	45.1093	-122.076	424.2		https://www.google.com/maps/search/45.1093,-122.076	https://data.globe.gov/system/photos/2019/06/30/1121751/original.jpg	https://data.globe.gov/system/photos/2019/06/30/1121753/original.jpg	https://data.globe.gov/system/photos/2019/06/30/1121752/original.jpg	https://data.globe.gov/system/photos/2019/06/30/1121754/original.jpg	https://data.globe.gov/system/photos/2019/06/30/1121756/original.jpg
GV_915	45.1141	-122.0756	429.2		https://www.google.com/maps/search/45.1141,-122.0756	https://data.globe.gov/system/photos/2019/06/30/1121763/original.jpg	https://data.globe.gov/system/photos/2019/06/30/1121765/original.jpg	https://data.globe.gov/system/photos/2019/06/30/1121764/original.jpg	https://data.globe.gov/system/photos/2019/06/30/1121766/original.jpg	https://data.globe.gov/system/photos/2019/06/30/1121768/original.jpg
GV_916	45.109	-122.0765	417.4		https://www.google.com/maps/search/45.109,-122.0765	https://data.globe.gov/system/photos/2019/06/30/1121775/original.jpg	https://data.globe.gov/system/photos/2019/06/30/1121777/original.jpg	https://data.globe.gov/system/photos/2019/06/30/1121776/original.jpg	https://data.globe.gov/system/photos/2019/06/30/1121778/original.jpg	https://data.globe.gov/system/photos/2019/06/30/1121780/original.jpg
GV_917	45.1125	-122.0745	395.5		https://www.google.com/maps/search/45.1125,-122.0745	https://data.globe.gov/system/photos/2019/06/30/1121781/original.jpg	https://data.globe.gov/system/photos/2019/06/30/1121783/original.jpg	https://data.globe.gov/system/photos/2019/06/30/1121782/original.jpg	https://data.globe.gov/system/photos/2019/06/30/1121784/original.jpg	https://data.globe.gov/system/photos/2019/06/30/1121786/original.jpg
GV_918	45.1103	-122.0745	401.7		https://www.google.com/maps/search/45.1103,-122.0745	https://data.globe.gov/system/photos/2019/06/30/1121787/original.jpg	https://data.globe.gov/system/photos/2019/06/30/1121789/original.jpg	https://data.globe.gov/system/photos/2019/06/30/1121788/original.jpg	https://data.globe.gov/system/photos/2019/06/30/1121790/original.jpg	https://data.globe.gov/system/photos/2019/06/30/1121792/original.jpg
GV_919	45.0972	-122.0704	452.2		https://www.google.com/maps/search/45.0972,-122.0704	https://data.globe.gov/system/photos/2019/06/30/1121799/original.jpg	https://data.globe.gov/system/photos/2019/06/30/1121801/original.jpg	https://data.globe.gov/system/photos/2019/06/30/1121800/original.jpg	https://data.globe.gov/system/photos/2019/06/30/1121802/original.jpg	https://data.globe.gov/system/photos/2019/06/30/1121804/original.jpg
GV_920	45.0836	-122.0569	462.4		https://www.google.com/maps/search/45.0836,-122.0569	https://data.globe.gov/system/photos/2019/06/30/1121805/original.jpg	https://data.globe.gov/system/photos/2019/06/30/1121807/original.jpg	https://data.globe.gov/system/photos/2019/06/30/1121806/original.jpg	https://data.globe.gov/system/photos/2019/06/30/1121808/original.jpg	https://data.globe.gov/system/photos/2019/06/30/1121810/original.jpg
GV_921	45.0886	-122.0609	445		https://www.google.com/maps/search/45.0886,-122.0609	https://data.globe.gov/system/photos/2019/06/30/1121811/original.jpg	https://data.globe.gov/system/photos/2019/06/30/1121816/original.jpg	https://data.globe.gov/system/photos/2019/06/30/1121813/original.jpg	https://data.globe.gov/system/photos/2019/06/30/1121818/original.jpg	https://data.globe.gov/system/photos/2019/06/30/1121822/original.jpg
GV_922	45.0707	-122.0335	595.4		https://www.google.com/maps/search/45.0707,-122.0335	https://data.globe.gov/system/photos/2019/06/30/1121823/original.jpg	https://data.globe.gov/system/photos/2019/06/30/1121825/original.jpg	https://data.globe.gov/system/photos/2019/06/30/1121824/original.jpg	https://data.globe.gov/system/photos/2019/06/30/1121826/original.jpg	https://data.globe.gov/system/photos/2019/06/30/1121828/original.jpg
GV_923	45.0691	-122.0246	743.1		https://www.google.com/maps/search/45.0691,-122.0246	https://data.globe.gov/system/photos/2019/06/30/1121835/original.jpg	https://data.globe.gov/system/photos/2019/06/30/1121837/original.jpg	https://data.globe.gov/system/photos/2019/06/30/1121836/original.jpg	https://data.globe.gov/system/photos/2019/06/30/1121838/original.jpg	https://data.globe.gov/system/photos/2019/06/30/1121840/original.jpg
GV_924	45.0655	-122.0239	781.5		https://www.google.com/maps/search/45.0655,-122.0239	https://data.globe.gov/system/photos/2019/06/30/1121847/original.jpg	https://data.globe.gov/system/photos/2019/06/30/1121849/original.jpg	https://data.globe.gov/system/photos/2019/06/30/1121848/original.jpg	https://data.globe.gov/system/photos/2019/06/30/1121850/original.jpg	https://data.globe.gov/system/photos/2019/06/30/1121852/original.jpg
GV_925	45.0584	-122.0108	941.3		https://www.google.com/maps/search/45.0584,-122.0108	https://data.globe.gov/system/photos/2019/06/30/1121859/original.jpg	https://data.globe.gov/system/photos/2019/06/30/1121861/original.jpg	https://data.globe.gov/system/photos/2019/06/30/1121860/original.jpg	https://data.globe.gov/system/photos/2019/06/30/1121862/original.jpg	https://data.globe.gov/system/photos/2019/06/30/1121864/original.jpg
GV_926	45.0652	-122.0232	784.2		https://www.google.com/maps/search/45.0652,-122.0232	https://data.globe.gov/system/photos/2019/06/30/1121871/original.jpg	https://data.globe.gov/system/photos/2019/06/30/1121873/original.jpg	https://data.globe.gov/system/photos/2019/06/30/1121872/original.jpg	https://data.globe.gov/system/photos/2019/06/30/1121874/original.jpg	https://data.globe.gov/system/photos/2019/06/30/1121876/original.jpg
GV_927	45.0618	-122.0224	794.5		https://www.google.com/maps/search/45.0618,-122.0224	https://data.globe.gov/system/photos/2019/06/30/1121877/original.jpg	https://data.globe.gov/system/photos/2019/06/30/1121879/original.jpg	https://data.globe.gov/system/photos/2019/06/30/1121878/original.jpg	https://data.globe.gov/system/photos/2019/06/30/1121880/original.jpg	https://data.globe.gov/system/photos/2019/06/30/1121882/original.jpg
GV_928	45.0563	-122.0183	864.9		https://www.google.com/maps/search/45.0563,-122.0183	https://data.globe.gov/system/photos/2019/06/30/1121889/original.jpg	https://data.globe.gov/system/photos/2019/06/30/1121891/original.jpg	https://data.globe.gov/system/photos/2019/06/30/1121890/original.jpg	https://data.globe.gov/system/photos/2019/06/30/1121892/original.jpg	https://data.globe.gov/system/photos/2019/06/30/1121894/original.jpg
GV_929	45.0565	-122.0177	870.1		https://www.google.com/maps/search/45.0565,-122.0177	https://data.globe.gov/system/photos/2019/06/30/1121895/original.jpg	https://data.globe.gov/system/photos/2019/06/30/1121897/original.jpg	https://data.globe.gov/system/photos/2019/06/30/1121896/original.jpg	https://data.globe.gov/system/photos/2019/06/30/1121898/original.jpg	https://data.globe.gov/system/photos/2019/06/30/1121900/original.jpg
GV_930	45.0565	-122.0167	893.7		https://www.google.com/maps/search/45.0565,-122.0167	https://data.globe.gov/system/photos/2019/06/30/1121907/original.jpg	https://data.globe.gov/system/photos/2019/06/30/1121909/original.jpg	https://data.globe.gov/system/photos/2019/06/30/1121908/original.jpg	https://data.globe.gov/system/photos/2019/06/30/1121910/original.jpg	https://data.globe.gov/system/photos/2019/06/30/1121912/original.jpg
GV_931	45.0567	-122.0172	878.7		https://www.google.com/maps/search/45.0567,-122.0172	https://data.globe.gov/system/photos/2019/06/30/1121919/original.jpg	https://data.globe.gov/system/photos/2019/06/30/1121921/original.jpg	https://data.globe.gov/system/photos/2019/06/30/1121920/original.jpg	https://data.globe.gov/system/photos/2019/06/30/1121922/original.jpg	https://data.globe.gov/system/photos/2019/06/30/1121924/original.jpg
GV_932	45.0581	-122.0107	939.3		https://www.google.com/maps/search/45.0581,-122.0107	https://data.globe.gov/system/photos/2019/06/30/1121925/original.jpg	https://data.globe.gov/system/photos/2019/06/30/1121927/original.jpg	https://data.globe.gov/system/photos/2019/06/30/1121926/original.jpg	https://data.globe.gov/system/photos/2019/06/30/1121928/original.jpg	https://data.globe.gov/system/photos/2019/06/30/1121930/original.jpg
GV_933	45.059	-122.0111	943.7		https://www.google.com/maps/search/45.059,-122.0111	https://data.globe.gov/system/photos/2019/06/30/1121937/original.jpg	https://data.globe.gov/system/photos/2019/06/30/1121939/original.jpg	https://data.globe.gov/system/photos/2019/06/30/1121938/original.jpg	https://data.globe.gov/system/photos/2019/06/30/1121940/original.jpg	https://data.globe.gov/system/photos/2019/06/30/1121942/original.jpg
GV_934	45.0522	-121.9758	1183.2		https://www.google.com/maps/search/45.0522,-121.9758	https://data.globe.gov/system/photos/2019/06/30/1121943/original.jpg	https://data.globe.gov/system/photos/2019/06/30/1121945/original.jpg	https://data.globe.gov/system/photos/2019/06/30/1121944/original.jpg	https://data.globe.gov/system/photos/2019/06/30/1121946/original.jpg	https://data.globe.gov/system/photos/2019/06/30/1121948/original.jpg
GV_935	45.059	-122.0076	967.8		https://www.google.com/maps/search/45.059,-122.0076	https://data.globe.gov/system/photos/2019/06/30/1121949/original.jpg	https://data.globe.gov/system/photos/2019/06/30/1121951/original.jpg	https://data.globe.gov/system/photos/2019/06/30/1121950/original.jpg	https://data.globe.gov/system/photos/2019/06/30/1121952/original.jpg	https://data.globe.gov/system/photos/2019/06/30/1121954/original.jpg
GV_936	45.05	-121.9753	1214.5		https://www.google.com/maps/search/45.05,-121.9753	https://data.globe.gov/system/photos/2019/06/30/1121955/original.jpg	https://data.globe.gov/system/photos/2019/06/30/1121957/original.jpg	https://data.globe.gov/system/photos/2019/06/30/1121956/original.jpg	https://data.globe.gov/system/photos/2019/06/30/1121958/original.jpg	https://data.globe.gov/system/photos/2019/06/30/1121960/original.jpg
GV_937	45.0592	-122.0066	964.9		https://www.google.com/maps/search/45.0592,-122.0066	https://data.globe.gov/system/photos/2019/06/30/1121967/original.jpg	https://data.globe.gov/system/photos/2019/06/30/1121969/original.jpg	https://data.globe.gov/system/photos/2019/06/30/1121968/original.jpg	https://data.globe.gov/system/photos/2019/06/30/1121970/original.jpg	https://data.globe.gov/system/photos/2019/06/30/1121972/original.jpg
GV_938	45.0595	-122.007	958.7		https://www.google.com/maps/search/45.0595,-122.007	https://data.globe.gov/system/photos/2019/06/30/1121973/original.jpg	https://data.globe.gov/system/photos/2019/06/30/1121975/original.jpg	https://data.globe.gov/system/photos/2019/06/30/1121974/original.jpg	https://data.globe.gov/system/photos/2019/06/30/1121976/original.jpg	https://data.globe.gov/system/photos/2019/06/30/1121978/original.jpg
GV_939	45.0594	-122.0084	950		https://www.google.com/maps/search/45.0594,-122.0084	https://data.globe.gov/system/photos/2019/06/30/1121979/original.jpg	https://data.globe.gov/system/photos/2019/06/30/1121981/original.jpg	https://data.globe.gov/system/photos/2019/06/30/1121980/original.jpg	https://data.globe.gov/system/photos/2019/06/30/1121982/original.jpg	https://data.globe.gov/system/photos/2019/06/30/1121984/original.jpg
GV_940	45.059	-122.0083	951.5		https://www.google.com/maps/search/45.059,-122.0083	https://data.globe.gov/system/photos/2019/06/30/1121985/original.jpg	https://data.globe.gov/system/photos/2019/06/30/1121987/original.jpg	https://data.globe.gov/system/photos/2019/06/30/1121986/original.jpg	https://data.globe.gov/system/photos/2019/06/30/1121988/original.jpg	https://data.globe.gov/system/photos/2019/06/30/1121990/original.jpg
GV_941	45.0535	-121.9943	1053.6		https://www.google.com/maps/search/45.0535,-121.9943	https://data.globe.gov/system/photos/2019/06/30/1121992/original.jpg	https://data.globe.gov/system/photos/2019/06/30/1121994/original.jpg	https://data.globe.gov/system/photos/2019/06/30/1121993/original.jpg	https://data.globe.gov/system/photos/2019/06/30/1121995/original.jpg	https://data.globe.gov/system/photos/2019/06/30/1121997/original.jpg
GV_942	45.0544	-121.9831	1121.1		https://www.google.com/maps/search/45.0544,-121.9831	https://data.globe.gov/system/photos/2019/06/30/1122004/original.jpg	https://data.globe.gov/system/photos/2019/06/30/1122006/original.jpg	https://data.globe.gov/system/photos/2019/06/30/1122005/original.jpg	https://data.globe.gov/system/photos/2019/06/30/1122007/original.jpg	https://data.globe.gov/system/photos/2019/06/30/1122009/original.jpg
GV_943	45.0541	-121.9777	1165.5		https://www.google.com/maps/search/45.0541,-121.9777	https://data.globe.gov/system/photos/2019/06/30/1122010/original.jpg	https://data.globe.gov/system/photos/2019/06/30/1122018/original.jpg	https://data.globe.gov/system/photos/2019/06/30/1122017/original.jpg	https://data.globe.gov/system/photos/2019/06/30/1122019/original.jpg	https://data.globe.gov/system/photos/2019/06/30/1122021/original.jpg
GV_944	45.0508	-121.9754	1214.2		https://www.google.com/maps/search/45.0508,-121.9754	https://data.globe.gov/system/photos/2019/06/30/1122029/original.jpg	https://data.globe.gov/system/photos/2019/06/30/1122031/original.jpg	https://data.globe.gov/system/photos/2019/06/30/1122030/original.jpg	https://data.globe.gov/system/photos/2019/06/30/1122032/original.jpg	https://data.globe.gov/system/photos/2019/06/30/1122034/original.jpg
GV_945	45.0514	-121.9746	1188.2		https://www.google.com/maps/search/45.0514,-121.9746	https://data.globe.gov/system/photos/2019/06/30/1122035/original.jpg	https://data.globe.gov/system/photos/2019/06/30/1122037/original.jpg	https://data.globe.gov/system/photos/2019/06/30/1122036/original.jpg	https://data.globe.gov/system/photos/2019/06/30/1122038/original.jpg	https://data.globe.gov/system/photos/2019/06/30/1122040/original.jpg
GV_946	45.0486	-121.9738	1187.6		https://www.google.com/maps/search/45.0486,-121.9738	https://data.globe.gov/system/photos/2019/06/30/1122055/original.jpg	https://data.globe.gov/system/photos/2019/06/30/1122057/original.jpg	https://data.globe.gov/system/photos/2019/06/30/1122056/original.jpg	https://data.globe.gov/system/photos/2019/06/30/1122058/original.jpg	https://data.globe.gov/system/photos/2019/06/30/1122060/original.jpg
GV_947	45.0487	-121.9725	1180.8		https://www.google.com/maps/search/45.0487,-121.9725	https://data.globe.gov/system/photos/2019/06/30/1122067/original.jpg	https://data.globe.gov/system/photos/2019/06/30/1122069/original.jpg	https://data.globe.gov/system/photos/2019/06/30/1122068/original.jpg	https://data.globe.gov/system/photos/2019/06/30/1122070/original.jpg	https://data.globe.gov/system/photos/2019/06/30/1122072/original.jpg
GV_948	45.0487	-121.9747	1199.3		https://www.google.com/maps/search/45.0487,-121.9747	https://data.globe.gov/system/photos/2019/06/30/1122079/original.jpg	https://data.globe.gov/system/photos/2019/06/30/1122081/original.jpg	https://data.globe.gov/system/photos/2019/06/30/1122080/original.jpg	https://data.globe.gov/system/photos/2019/06/30/1122082/original.jpg	https://data.globe.gov/system/photos/2019/06/30/1122084/original.jpg
GV_949	45.0486	-121.9753	1210.7		https://www.google.com/maps/search/45.0486,-121.9753	https://data.globe.gov/system/photos/2019/06/30/1122091/original.jpg	https://data.globe.gov/system/photos/2019/06/30/1122093/original.jpg	https://data.globe.gov/system/photos/2019/06/30/1122092/original.jpg	https://data.globe.gov/system/photos/2019/06/30/1122094/original.jpg	https://data.globe.gov/system/photos/2019/06/30/1122096/original.jpg
GV_950	45.0553	-121.9793	1154.9		https://www.google.com/maps/search/45.0553,-121.9793	https://data.globe.gov/system/photos/2019/06/30/1122103/original.jpg	https://data.globe.gov/system/photos/2019/06/30/1122107/original.jpg	https://data.globe.gov/system/photos/2019/06/30/1122105/original.jpg	https://data.globe.gov/system/photos/2019/06/30/1122109/original.jpg	https://data.globe.gov/system/photos/2019/06/30/1122113/original.jpg
GV_951	45.0541	-121.9904	1087.4		https://www.google.com/maps/search/45.0541,-121.9904	https://data.globe.gov/system/photos/2019/06/30/1122114/original.jpg	https://data.globe.gov/system/photos/2019/06/30/1122117/original.jpg	https://data.globe.gov/system/photos/2019/06/30/1122116/original.jpg	https://data.globe.gov/system/photos/2019/06/30/1122118/original.jpg	https://data.globe.gov/system/photos/2019/06/30/1122120/original.jpg
GV_952	45.0539	-121.9908	1076.3		https://www.google.com/maps/search/45.0539,-121.9908	https://data.globe.gov/system/photos/2019/06/30/1122121/original.jpg	https://data.globe.gov/system/photos/2019/06/30/1122123/original.jpg	https://data.globe.gov/system/photos/2019/06/30/1122122/original.jpg	https://data.globe.gov/system/photos/2019/06/30/1122124/original.jpg	https://data.globe.gov/system/photos/2019/06/30/1122127/original.jpg
GV_953	45.0579	-122.0052	1005		https://www.google.com/maps/search/45.0579,-122.0052	https://data.globe.gov/system/photos/2019/06/30/1122134/original.jpg	https://data.globe.gov/system/photos/2019/06/30/1122137/original.jpg	https://data.globe.gov/system/photos/2019/06/30/1122136/original.jpg	https://data.globe.gov/system/photos/2019/06/30/1122138/original.jpg	https://data.globe.gov/system/photos/2019/06/30/1122140/original.jpg
GV_954	45.0668	-122.0244	788.9		https://www.google.com/maps/search/45.0668,-122.0244	https://data.globe.gov/system/photos/2019/06/30/1122141/original.jpg	https://data.globe.gov/system/photos/2019/06/30/1122144/original.jpg	https://data.globe.gov/system/photos/2019/06/30/1122142/original.jpg	https://data.globe.gov/system/photos/2019/06/30/1122145/original.jpg	https://data.globe.gov/system/photos/2019/06/30/1122147/original.jpg
GV_955	45.0888	-122.0644	440.3		https://www.google.com/maps/search/45.0888,-122.0644	https://data.globe.gov/system/photos/2019/06/30/1122156/original.jpg	https://data.globe.gov/system/photos/2019/06/30/1122158/original.jpg	https://data.globe.gov/system/photos/2019/06/30/1122157/original.jpg	https://data.globe.gov/system/photos/2019/06/30/1122159/original.jpg	https://data.globe.gov/system/photos/2019/06/30/1122161/original.jpg
GV_956	45.1562	-122.1126	302.9		https://www.google.com/maps/search/45.1562,-122.1126	https://data.globe.gov/system/photos/2019/06/30/1122162/original.jpg	https://data.globe.gov/system/photos/2019/06/30/1122164/original.jpg	https://data.globe.gov/system/photos/2019/06/30/1122163/original.jpg	https://data.globe.gov/system/photos/2019/06/30/1122165/original.jpg	https://data.globe.gov/system/photos/2019/06/30/1122167/original.jpg
GV_957	45.1562	-122.1134	307.9		https://www.google.com/maps/search/45.1562,-122.1134	https://data.globe.gov/system/photos/2019/06/30/1122168/original.jpg	https://data.globe.gov/system/photos/2019/06/30/1122170/original.jpg	https://data.globe.gov/system/photos/2019/06/30/1122169/original.jpg	https://data.globe.gov/system/photos/2019/06/30/1122172/original.jpg	https://data.globe.gov/system/photos/2019/06/30/1122174/original.jpg
GV_958	45.1972	-122.1816	232.7		https://www.google.com/maps/search/45.1972,-122.1816	https://data.globe.gov/system/photos/2019/06/30/1122181/original.jpg	https://data.globe.gov/system/photos/2019/06/30/1122183/original.jpg	https://data.globe.gov/system/photos/2019/06/30/1122182/original.jpg	https://data.globe.gov/system/photos/2019/06/30/1122184/original.jpg	https://data.globe.gov/system/photos/2019/06/30/1122186/original.jpg
GV_959	45.1975	-122.1825	230.9		https://www.google.com/maps/search/45.1975,-122.1825	https://data.globe.gov/system/photos/2019/06/30/1122187/original.jpg	https://data.globe.gov/system/photos/2019/06/30/1122189/original.jpg	https://data.globe.gov/system/photos/2019/06/30/1122188/original.jpg	https://data.globe.gov/system/photos/2019/06/30/1122190/original.jpg	https://data.globe.gov/system/photos/2019/06/30/1122192/original.jpg
GV_960	45.1974	-122.1824	227.5		https://www.google.com/maps/search/45.1974,-122.1824	https://data.globe.gov/system/photos/2019/06/30/1122193/original.jpg	https://data.globe.gov/system/photos/2019/06/30/1122195/original.jpg	https://data.globe.gov/system/photos/2019/06/30/1122194/original.jpg	https://data.globe.gov/system/photos/2019/06/30/1122196/original.jpg	https://data.globe.gov/system/photos/2019/06/30/1122198/original.jpg
GV_961	45.3864	-122.4412	55		https://www.google.com/maps/search/45.3864,-122.4412	https://data.globe.gov/system/photos/2019/06/30/1122205/original.jpg	https://data.globe.gov/system/photos/2019/06/30/1122207/original.jpg	https://data.globe.gov/system/photos/2019/06/30/1122206/original.jpg	https://data.globe.gov/system/photos/2019/06/30/1122208/original.jpg	https://data.globe.gov/system/photos/2019/06/30/1122210/original.jpg
GV_962	46.3643	-117.0597	229.8		https://www.google.com/maps/search/46.3643,-117.0597	https://data.globe.gov/system/photos/2019/06/30/1122211/original.jpg	https://data.globe.gov/system/photos/2019/06/30/1122213/original.jpg	https://data.globe.gov/system/photos/2019/06/30/1122212/original.jpg	https://data.globe.gov/system/photos/2019/06/30/1122214/original.jpg	https://data.globe.gov/system/photos/2019/06/30/1122216/original.jpg
GV_963	63.5174	-149.8182	1055.8		https://www.google.com/maps/search/63.5174,-149.8182	https://data.globe.gov/system/photos/2019/06/16/1122521/original.jpg	https://data.globe.gov/system/photos/2019/06/16/1122523/original.jpg	https://data.globe.gov/system/photos/2019/06/16/1122522/original.jpg	https://data.globe.gov/system/photos/2019/06/16/1122524/original.jpg	https://data.globe.gov/system/photos/2019/06/16/1122526/original.jpg
GV_964	38.8528	-77.1504	81.9		https://www.google.com/maps/search/38.8528,-77.1504	https://data.globe.gov/system/photos/2019/07/01/1122652/original.jpg	https://data.globe.gov/system/photos/2019/07/01/1122654/original.jpg	https://data.globe.gov/system/photos/2019/07/01/1122653/original.jpg	https://data.globe.gov/system/photos/2019/07/01/1122655/original.jpg	https://data.globe.gov/system/photos/2019/07/01/1122657/original.jpg
GV_965	43.7922	-114.1667	2671.3		https://www.google.com/maps/search/43.7922,-114.1667	https://data.globe.gov/system/photos/2019/06/26/1122730/original.jpg	https://data.globe.gov/system/photos/2019/06/26/1122732/original.jpg	https://data.globe.gov/system/photos/2019/06/26/1122731/original.jpg	https://data.globe.gov/system/photos/2019/06/26/1122733/original.jpg	https://data.globe.gov/system/photos/2019/06/26/1122736/original.jpg
GV_966	46.6941	-111.8144	1115.2		https://www.google.com/maps/search/46.6941,-111.8144	https://data.globe.gov/system/photos/2019/07/01/1122776/original.jpg	https://data.globe.gov/system/photos/2019/07/01/1122778/original.jpg	https://data.globe.gov/system/photos/2019/07/01/1122777/original.jpg	https://data.globe.gov/system/photos/2019/07/01/1122779/original.jpg	https://data.globe.gov/system/photos/2019/07/01/1122781/original.jpg
GV_967	46.6938	-111.8151	1115.7		https://www.google.com/maps/search/46.6938,-111.8151	https://data.globe.gov/system/photos/2019/07/01/1122788/original.jpg	https://data.globe.gov/system/photos/2019/07/01/1122790/original.jpg	https://data.globe.gov/system/photos/2019/07/01/1122789/original.jpg	https://data.globe.gov/system/photos/2019/07/01/1122791/original.jpg	https://data.globe.gov/system/photos/2019/07/01/1122793/original.jpg
GV_968	46.6934	-111.8155	1114		https://www.google.com/maps/search/46.6934,-111.8155	https://data.globe.gov/system/photos/2019/07/01/1122794/original.jpg	https://data.globe.gov/system/photos/2019/07/01/1122796/original.jpg	https://data.globe.gov/system/photos/2019/07/01/1122795/original.jpg	https://data.globe.gov/system/photos/2019/07/01/1122797/original.jpg	https://data.globe.gov/system/photos/2019/07/01/1122799/original.jpg
GV_969	46.6935	-111.8155	1114.1		https://www.google.com/maps/search/46.6935,-111.8155	https://data.globe.gov/system/photos/2019/07/01/1122801/original.jpg	https://data.globe.gov/system/photos/2019/07/01/1122803/original.jpg	https://data.globe.gov/system/photos/2019/07/01/1122802/original.jpg	https://data.globe.gov/system/photos/2019/07/01/1122804/original.jpg	https://data.globe.gov/system/photos/2019/07/01/1122806/original.jpg
GV_970	46.6957	-111.8125	1115.1		https://www.google.com/maps/search/46.6957,-111.8125	https://data.globe.gov/system/photos/2019/07/01/1122815/original.jpg	https://data.globe.gov/system/photos/2019/07/01/1122817/original.jpg	https://data.globe.gov/system/photos/2019/07/01/1122816/original.jpg	https://data.globe.gov/system/photos/2019/07/01/1122818/original.jpg	https://data.globe.gov/system/photos/2019/07/01/1122820/original.jpg
GV_971	46.6989	-111.8109	1164.1		https://www.google.com/maps/search/46.6989,-111.8109	https://data.globe.gov/system/photos/2019/07/01/1122822/original.jpg	https://data.globe.gov/system/photos/2019/07/01/1122824/original.jpg	https://data.globe.gov/system/photos/2019/07/01/1122823/original.jpg	https://data.globe.gov/system/photos/2019/07/01/1122825/original.jpg	https://data.globe.gov/system/photos/2019/07/01/1122827/original.jpg
GV_972	39.134	-77.1856	140.6		https://www.google.com/maps/search/39.134,-77.1856	https://data.globe.gov/system/photos/2019/06/29/1122839/original.jpg	https://data.globe.gov/system/photos/2019/06/29/1122841/original.jpg	https://data.globe.gov/system/photos/2019/06/29/1122840/original.jpg	https://data.globe.gov/system/photos/2019/06/29/1122842/original.jpg	https://data.globe.gov/system/photos/2019/06/29/1122844/original.jpg
GV_973	39.134	-77.1856	140.6		https://www.google.com/maps/search/39.134,-77.1856	https://data.globe.gov/system/photos/2019/06/29/1122845/original.jpg	https://data.globe.gov/system/photos/2019/06/29/1122847/original.jpg	https://data.globe.gov/system/photos/2019/06/29/1122846/original.jpg	https://data.globe.gov/system/photos/2019/06/29/1122848/original.jpg	https://data.globe.gov/system/photos/2019/06/29/1122850/original.jpg
GV_974	42.1014	-83.2736	181.4		https://www.google.com/maps/search/42.1014,-83.2736	https://data.globe.gov/system/photos/2019/07/01/1122944/original.jpg	https://data.globe.gov/system/photos/2019/07/01/1122946/original.jpg	https://data.globe.gov/system/photos/2019/07/01/1122945/original.jpg	https://data.globe.gov/system/photos/2019/07/01/1122947/original.jpg	https://data.globe.gov/system/photos/2019/07/01/1122949/original.jpg
GV_975	38.9115	-95.3037	256.3		https://www.google.com/maps/search/38.9115,-95.3037	https://data.globe.gov/system/photos/2019/07/01/1122998/original.jpg	https://data.globe.gov/system/photos/2019/07/01/1123003/original.jpg	https://data.globe.gov/system/photos/2019/07/01/1123001/original.jpg	https://data.globe.gov/system/photos/2019/07/01/1123006/original.jpg	https://data.globe.gov/system/photos/2019/07/01/1123009/original.jpg
GV_976	47.0719	-88.6411	341.6		https://www.google.com/maps/search/47.0719,-88.6411	https://data.globe.gov/system/photos/2019/07/01/1123070/original.jpg	https://data.globe.gov/system/photos/2019/07/01/1123072/original.jpg	https://data.globe.gov/system/photos/2019/07/01/1123071/original.jpg	https://data.globe.gov/system/photos/2019/07/01/1123073/original.jpg	https://data.globe.gov/system/photos/2019/07/01/1123075/original.jpg
GV_977	39.012	-76.8299	43.3		https://www.google.com/maps/search/39.012,-76.8299	https://data.globe.gov/system/photos/2019/07/02/1123567/original.jpg	https://data.globe.gov/system/photos/2019/07/02/1123569/original.jpg	https://data.globe.gov/system/photos/2019/07/02/1123568/original.jpg	https://data.globe.gov/system/photos/2019/07/02/1123570/original.jpg	https://data.globe.gov/system/photos/2019/07/02/1123572/original.jpg
GV_978	5.7964	-0.1224	86		https://www.google.com/maps/search/5.7964,-0.1224	https://data.globe.gov/system/photos/2019/07/02/1123691/original.jpg	https://data.globe.gov/system/photos/2019/07/02/1123693/original.jpg	https://data.globe.gov/system/photos/2019/07/02/1123692/original.jpg	https://data.globe.gov/system/photos/2019/07/02/1123695/original.jpg	https://data.globe.gov/system/photos/2019/07/02/1123698/original.jpg
GV_979	42.1013	-83.2736	181.2		https://www.google.com/maps/search/42.1013,-83.2736	https://data.globe.gov/system/photos/2019/07/02/1123743/original.jpg	https://data.globe.gov/system/photos/2019/07/02/1123745/original.jpg	https://data.globe.gov/system/photos/2019/07/02/1123744/original.jpg	https://data.globe.gov/system/photos/2019/07/02/1123746/original.jpg	https://data.globe.gov/system/photos/2019/07/02/1123748/original.jpg
GV_980	45.3489	-111.7226	1503.1		https://www.google.com/maps/search/45.3489,-111.7226	https://data.globe.gov/system/photos/2019/06/30/1123969/original.jpg	https://data.globe.gov/system/photos/2019/06/30/1123971/original.jpg	https://data.globe.gov/system/photos/2019/06/30/1123970/original.jpg	https://data.globe.gov/system/photos/2019/06/30/1123972/original.jpg	https://data.globe.gov/system/photos/2019/06/30/1123974/original.jpg
GV_981	40.1997	-75.4574	63.8		https://www.google.com/maps/search/40.1997,-75.4574	https://data.globe.gov/system/photos/2019/06/18/1124096/original.jpg	https://data.globe.gov/system/photos/2019/06/18/1124098/original.jpg	https://data.globe.gov/system/photos/2019/06/18/1124097/original.jpg	https://data.globe.gov/system/photos/2019/06/18/1124099/original.jpg	https://data.globe.gov/system/photos/2019/06/18/1124101/original.jpg
GV_982	42.1014	-83.2737	181.5		https://www.google.com/maps/search/42.1014,-83.2737	https://data.globe.gov/system/photos/2019/07/03/1124573/original.jpg	https://data.globe.gov/system/photos/2019/07/03/1124575/original.jpg	https://data.globe.gov/system/photos/2019/07/03/1124574/original.jpg	https://data.globe.gov/system/photos/2019/07/03/1124576/original.jpg	https://data.globe.gov/system/photos/2019/07/03/1124578/original.jpg
GV_983	45.5427	-122.3703	13.4		https://www.google.com/maps/search/45.5427,-122.3703	https://data.globe.gov/system/photos/2019/07/03/1124749/original.jpg	https://data.globe.gov/system/photos/2019/07/03/1124751/original.jpg	https://data.globe.gov/system/photos/2019/07/03/1124750/original.jpg	https://data.globe.gov/system/photos/2019/07/03/1124752/original.jpg	https://data.globe.gov/system/photos/2019/07/03/1124754/original.jpg

GV_984	-34.1981	140.3054	58.2		https://www.google.com/maps/search/-34.1981,140.3054	https://data.globe.gov/system/photos/2019/07/04/1125077/original.jpg	https://data.globe.gov/system/photos/2019/07/04/1125079/original.jpg	https://data.globe.gov/system/photos/2019/07/04/1125078/original.jpg	https://data.globe.gov/system/photos/2019/07/04/1125080/original.jpg	https://data.globe.gov/system/photos/2019/07/04/1125082/original.jpg
GV_985	6.762	80.0164	23		https://www.google.com/maps/search/6.762,80.0164	https://data.globe.gov/system/photos/2019/07/04/1125172/original.jpg	https://data.globe.gov/system/photos/2019/07/04/1125174/original.jpg	https://data.globe.gov/system/photos/2019/07/04/1125173/original.jpg	https://data.globe.gov/system/photos/2019/07/04/1125175/original.jpg	https://data.globe.gov/system/photos/2019/07/04/1125177/original.jpg
GV_986	24.402	118.3205	21.6		https://www.google.com/maps/search/24.402,118.3205	https://data.globe.gov/system/photos/2019/07/04/1125217/original.jpg	https://data.globe.gov/system/photos/2019/07/04/1125219/original.jpg	https://data.globe.gov/system/photos/2019/07/04/1125218/original.jpg	https://data.globe.gov/system/photos/2019/07/04/1125220/original.jpg	https://data.globe.gov/system/photos/2019/07/04/1125222/original.jpg
GV_987	24.4455	118.4072	40		https://www.google.com/maps/search/24.4455,118.4072	https://data.globe.gov/system/photos/2019/07/04/1125235/original.jpg	https://data.globe.gov/system/photos/2019/07/04/1125237/original.jpg	https://data.globe.gov/system/photos/2019/07/04/1125236/original.jpg	https://data.globe.gov/system/photos/2019/07/04/1125238/original.jpg	https://data.globe.gov/system/photos/2019/07/04/1125240/original.jpg
GV_988	32.8342	-79.9868	5		https://www.google.com/maps/search/32.8342,-79.9868	https://data.globe.gov/system/photos/2019/07/04/1125322/original.jpg	https://data.globe.gov/system/photos/2019/07/04/1125324/original.jpg	https://data.globe.gov/system/photos/2019/07/04/1125323/original.jpg	https://data.globe.gov/system/photos/2019/07/04/1125325/original.jpg	https://data.globe.gov/system/photos/2019/07/04/1125327/original.jpg
GV_989	32.8285	-79.9895	4.5		https://www.google.com/maps/search/32.8285,-79.9895	https://data.globe.gov/system/photos/2019/07/04/1125338/original.jpg	https://data.globe.gov/system/photos/2019/07/04/1125340/original.jpg	https://data.globe.gov/system/photos/2019/07/04/1125339/original.jpg	https://data.globe.gov/system/photos/2019/07/04/1125341/original.jpg	https://data.globe.gov/system/photos/2019/07/04/1125343/original.jpg
GV_990	41.4157	-76.5836	609.7		https://www.google.com/maps/search/41.4157,-76.5836	https://data.globe.gov/system/photos/2019/07/04/1125356/original.jpg	https://data.globe.gov/system/photos/2019/07/04/1125358/original.jpg	https://data.globe.gov/system/photos/2019/07/04/1125357/original.jpg	https://data.globe.gov/system/photos/2019/07/04/1125359/original.jpg	https://data.globe.gov/system/photos/2019/07/04/1125361/original.jpg
GV_991	41.3363	-72.9088	46.3		https://www.google.com/maps/search/41.3363,-72.9088	https://data.globe.gov/system/photos/2019/07/04/1125368/original.jpg	https://data.globe.gov/system/photos/2019/07/04/1125370/original.jpg	https://data.globe.gov/system/photos/2019/07/04/1125369/original.jpg	https://data.globe.gov/system/photos/2019/07/04/1125371/original.jpg	https://data.globe.gov/system/photos/2019/07/04/1125373/original.jpg
GV_992	-34.6643	144.1634	78.4		https://www.google.com/maps/search/-34.6643,144.1634	https://data.globe.gov/system/photos/2019/07/04/1125781/original.jpg	https://data.globe.gov/system/photos/2019/07/04/1125789/original.jpg	https://data.globe.gov/system/photos/2019/07/04/1125788/original.jpg	https://data.globe.gov/system/photos/2019/07/04/1125790/original.jpg	https://data.globe.gov/system/photos/2019/07/04/1125792/original.jpg
GV_993	38.0799	127.2181	113.5		https://www.google.com/maps/search/38.0799,127.2181	https://data.globe.gov/system/photos/2019/07/05/1126101/original.jpg	https://data.globe.gov/system/photos/2019/07/05/1126103/original.jpg	https://data.globe.gov/system/photos/2019/07/05/1126102/original.jpg	https://data.globe.gov/system/photos/2019/07/05/1126104/original.jpg	https://data.globe.gov/system/photos/2019/07/05/1126106/original.jpg
GV_994	38.0801	127.217	108.1		https://www.google.com/maps/search/38.0801,127.217	https://data.globe.gov/system/photos/2019/07/05/1126112/original.jpg	https://data.globe.gov/system/photos/2019/07/05/1126114/original.jpg	https://data.globe.gov/system/photos/2019/07/05/1126113/original.jpg	https://data.globe.gov/system/photos/2019/07/05/1126115/original.jpg	https://data.globe.gov/system/photos/2019/07/05/1126117/original.jpg
GV_995	45.8002	-121.486	128.4		https://www.google.com/maps/search/45.8002,-121.486	https://data.globe.gov/system/photos/2019/07/04/1126118/original.jpg	https://data.globe.gov/system/photos/2019/07/04/1126120/original.jpg	https://data.globe.gov/system/photos/2019/07/04/1126119/original.jpg	https://data.globe.gov/system/photos/2019/07/04/1126121/original.jpg	https://data.globe.gov/system/photos/2019/07/04/1126123/original.jpg
GV_996	46.0478	-121.5716	828.6		https://www.google.com/maps/search/46.0478,-121.5716	https://data.globe.gov/system/photos/2019/07/04/1126130/original.jpg	https://data.globe.gov/system/photos/2019/07/04/1126132/original.jpg	https://data.globe.gov/system/photos/2019/07/04/1126131/original.jpg	https://data.globe.gov/system/photos/2019/07/04/1126133/original.jpg	https://data.globe.gov/system/photos/2019/07/04/1126135/original.jpg
GV_997	45.9913	-121.52	573.4		https://www.google.com/maps/search/45.9913,-121.52	https://data.globe.gov/system/photos/2019/07/04/1126137/original.jpg	https://data.globe.gov/system/photos/2019/07/04/1126140/original.jpg	https://data.globe.gov/system/photos/2019/07/04/1126138/original.jpg	https://data.globe.gov/system/photos/2019/07/04/1126142/original.jpg	https://data.globe.gov/system/photos/2019/07/04/1126146/original.jpg
GV_998	45.9927	-121.5214	574		https://www.google.com/maps/search/45.9927,-121.5214	https://data.globe.gov/system/photos/2019/07/04/1126148/original.jpg	https://data.globe.gov/system/photos/2019/07/04/1126150/original.jpg	https://data.globe.gov/system/photos/2019/07/04/1126149/original.jpg	https://data.globe.gov/system/photos/2019/07/04/1126151/original.jpg	https://data.globe.gov/system/photos/2019/07/04/1126153/original.jpg
GV_999	46.0473	-121.5569	779.8		https://www.google.com/maps/search/46.0473,-121.5569	https://data.globe.gov/system/photos/2019/07/04/1126154/original.jpg	https://data.globe.gov/system/photos/2019/07/04/1126156/original.jpg	https://data.globe.gov/system/photos/2019/07/04/1126155/original.jpg	https://data.globe.gov/system/photos/2019/07/04/1126157/original.jpg	https://data.globe.gov/system/photos/2019/07/04/1126159/original.jpg
GV_1000	46.0467	-121.5609	787.7		https://www.google.com/maps/search/46.0467,-121.5609	https://data.globe.gov/system/photos/2019/07/04/1126160/original.jpg	https://data.globe.gov/system/photos/2019/07/04/1126162/original.jpg	https://data.globe.gov/system/photos/2019/07/04/1126161/original.jpg	https://data.globe.gov/system/photos/2019/07/04/1126163/original.jpg	https://data.globe.gov/system/photos/2019/07/04/1126165/original.jpg
GV_1001	46.0466	-121.5644	817.9		https://www.google.com/maps/search/46.0466,-121.5644	https://data.globe.gov/system/photos/2019/07/04/1126166/original.jpg	https://data.globe.gov/system/photos/2019/07/04/1126168/original.jpg	https://data.globe.gov/system/photos/2019/07/04/1126167/original.jpg	https://data.globe.gov/system/photos/2019/07/04/1126169/original.jpg	https://data.globe.gov/system/photos/2019/07/04/1126171/original.jpg
GV_1002	46.0473	-121.5685	826.7		https://www.google.com/maps/search/46.0473,-121.5685	https://data.globe.gov/system/photos/2019/07/04/1126172/original.jpg	https://data.globe.gov/system/photos/2019/07/04/1126176/original.jpg	https://data.globe.gov/system/photos/2019/07/04/1126173/original.jpg	https://data.globe.gov/system/photos/2019/07/04/1126177/original.jpg	https://data.globe.gov/system/photos/2019/07/04/1126180/original.jpg
GV_1003	46.0479	-121.5729	819.4		https://www.google.com/maps/search/46.0479,-121.5729	https://data.globe.gov/system/photos/2019/07/04/1126182/original.jpg	https://data.globe.gov/system/photos/2019/07/04/1126186/original.jpg	https://data.globe.gov/system/photos/2019/07/04/1126183/original.jpg	https://data.globe.gov/system/photos/2019/07/04/1126187/original.jpg	https://data.globe.gov/system/photos/2019/07/04/1126189/original.jpg
GV_1004	46.0493	-121.5734	823.4		https://www.google.com/maps/search/46.0493,-121.5734	https://data.globe.gov/system/photos/2019/07/04/1126190/original.jpg	https://data.globe.gov/system/photos/2019/07/04/1126192/original.jpg	https://data.globe.gov/system/photos/2019/07/04/1126191/original.jpg	https://data.globe.gov/system/photos/2019/07/04/1126193/original.jpg	https://data.globe.gov/system/photos/2019/07/04/1126195/original.jpg
GV_1005	46.0515	-121.573	822.8		https://www.google.com/maps/search/46.0515,-121.573	https://data.globe.gov/system/photos/2019/07/04/1126196/original.jpg	https://data.globe.gov/system/photos/2019/07/04/1126198/original.jpg	https://data.globe.gov/system/photos/2019/07/04/1126197/original.jpg	https://data.globe.gov/system/photos/2019/07/04/1126199/original.jpg	https://data.globe.gov/system/photos/2019/07/04/1126201/original.jpg
GV_1006	46.0545	-121.5696	819.6		https://www.google.com/maps/search/46.0545,-121.5696	https://data.globe.gov/system/photos/2019/07/04/1126208/original.jpg	https://data.globe.gov/system/photos/2019/07/04/1126210/original.jpg	https://data.globe.gov/system/photos/2019/07/04/1126209/original.jpg	https://data.globe.gov/system/photos/2019/07/04/1126211/original.jpg	https://data.globe.gov/system/photos/2019/07/04/1126213/original.jpg
GV_1007	46.0542	-121.5699	821.9		https://www.google.com/maps/search/46.0542,-121.5699	https://data.globe.gov/system/photos/2019/07/04/1126214/original.jpg	https://data.globe.gov/system/photos/2019/07/04/1126216/original.jpg	https://data.globe.gov/system/photos/2019/07/04/1126215/original.jpg	https://data.globe.gov/system/photos/2019/07/04/1126217/original.jpg	https://data.globe.gov/system/photos/2019/07/04/1126219/original.jpg
GV_1008	46.0538	-121.5705	821.8		https://www.google.com/maps/search/46.0538,-121.5705	https://data.globe.gov/system/photos/2019/07/04/1126220/original.jpg	https://data.globe.gov/system/photos/2019/07/04/1126222/original.jpg	https://data.globe.gov/system/photos/2019/07/04/1126221/original.jpg	https://data.globe.gov/system/photos/2019/07/04/1126223/original.jpg	https://data.globe.gov/system/photos/2019/07/04/1126225/original.jpg
GV_1009	46.0679	-121.5754	794.2		https://www.google.com/maps/search/46.0679,-121.5754	https://data.globe.gov/system/photos/2019/07/04/1126226/original.jpg	https://data.globe.gov/system/photos/2019/07/04/1126228/original.jpg	https://data.globe.gov/system/photos/2019/07/04/1126227/original.jpg	https://data.globe.gov/system/photos/2019/07/04/1126229/original.jpg	https://data.globe.gov/system/photos/2019/07/04/1126231/original.jpg
GV_1010	46.0681	-121.5751	794.5		https://www.google.com/maps/search/46.0681,-121.5751	https://data.globe.gov/system/photos/2019/07/04/1126232/original.jpg	https://data.globe.gov/system/photos/2019/07/04/1126234/original.jpg	https://data.globe.gov/system/photos/2019/07/04/1126233/original.jpg	https://data.globe.gov/system/photos/2019/07/04/1126235/original.jpg	https://data.globe.gov/system/photos/2019/07/04/1126237/original.jpg
GV_1011	46.0929	-121.6061	942.7		https://www.google.com/maps/search/46.0929,-121.6061	https://data.globe.gov/system/photos/2019/07/04/1126238/original.jpg	https://data.globe.gov/system/photos/2019/07/04/1126240/original.jpg	https://data.globe.gov/system/photos/2019/07/04/1126239/original.jpg	https://data.globe.gov/system/photos/2019/07/04/1126241/original.jpg	https://data.globe.gov/system/photos/2019/07/04/1126243/original.jpg
GV_1012	46.1045	-121.6085	951.9		https://www.google.com/maps/search/46.1045,-121.6085	https://data.globe.gov/system/photos/2019/07/04/1126244/original.jpg	https://data.globe.gov/system/photos/2019/07/04/1126246/original.jpg	https://data.globe.gov/system/photos/2019/07/04/1126245/original.jpg	https://data.globe.gov/system/photos/2019/07/04/1126247/original.jpg	https://data.globe.gov/system/photos/2019/07/04/1126249/original.jpg
GV_1013	46.1057	-121.6054	957.2		https://www.google.com/maps/search/46.1057,-121.6054	https://data.globe.gov/system/photos/2019/07/04/1126250/original.jpg	https://data.globe.gov/system/photos/2019/07/04/1126252/original.jpg	https://data.globe.gov/system/photos/2019/07/04/1126251/original.jpg	https://data.globe.gov/system/photos/2019/07/04/1126253/original.jpg	https://data.globe.gov/system/photos/2019/07/04/1126255/original.jpg
GV_1014	46.1065	-121.6055	962.7		https://www.google.com/maps/search/46.1065,-121.6055	https://data.globe.gov/system/photos/2019/07/04/1126256/original.jpg	https://data.globe.gov/system/photos/2019/07/04/1126258/original.jpg	https://data.globe.gov/system/photos/2019/07/04/1126257/original.jpg	https://data.globe.gov/system/photos/2019/07/04/1126259/original.jpg	https://data.globe.gov/system/photos/2019/07/04/1126261/original.jpg
GV_1015	46.1068	-121.6057	968.1		https://www.google.com/maps/search/46.1068,-121.6057	https://data.globe.gov/system/photos/2019/07/04/1126262/original.jpg	https://data.globe.gov/system/photos/2019/07/04/1126264/original.jpg	https://data.globe.gov/system/photos/2019/07/04/1126263/original.jpg	https://data.globe.gov/system/photos/2019/07/04/1126265/original.jpg	https://data.globe.gov/system/photos/2019/07/04/1126267/original.jpg
GV_1016	46.1037	-121.6061	958.9		https://www.google.com/maps/search/46.1037,-121.6061	https://data.globe.gov/system/photos/2019/07/04/1126268/original.jpg	https://data.globe.gov/system/photos/2019/07/04/1126270/original.jpg	https://data.globe.gov/system/photos/2019/07/04/1126269/original.jpg	https://data.globe.gov/system/photos/2019/07/04/1126271/original.jpg	https://data.globe.gov/system/photos/2019/07/04/1126273/original.jpg
GV_1017	46.1033	-121.6051	958.2		https://www.google.com/maps/search/46.1033,-121.6051	https://data.globe.gov/system/photos/2019/07/04/1126274/original.jpg	https://data.globe.gov/system/photos/2019/07/04/1126276/original.jpg	https://data.globe.gov/system/photos/2019/07/04/1126275/original.jpg	https://data.globe.gov/system/photos/2019/07/04/1126277/original.jpg	https://data.globe.gov/system/photos/2019/07/04/1126279/original.jpg
GV_1018	46.1042	-121.6052	962.2		https://www.google.com/maps/search/46.1042,-121.6052	https://data.globe.gov/system/photos/2019/07/04/1126280/original.jpg	https://data.globe.gov/system/photos/2019/07/04/1126282/original.jpg	https://data.globe.gov/system/photos/2019/07/04/1126281/original.jpg	https://data.globe.gov/system/photos/2019/07/04/1126283/original.jpg	https://data.globe.gov/system/photos/2019/07/04/1126285/original.jpg
GV_1019	46.103	-121.6063	957.8		https://www.google.com/maps/search/46.103,-121.6063	https://data.globe.gov/system/photos/2019/07/04/1126286/original.jpg	https://data.globe.gov/system/photos/2019/07/04/1126288/original.jpg	https://data.globe.gov/system/photos/2019/07/04/1126287/original.jpg	https://data.globe.gov/system/photos/2019/07/04/1126289/original.jpg	https://data.globe.gov/system/photos/2019/07/04/1126291/original.jpg
GV_1020	46.1023	-121.6065	956.8		https://www.google.com/maps/search/46.1023,-121.6065	https://data.globe.gov/system/photos/2019/07/04/1126292/original.jpg	https://data.globe.gov/system/photos/2019/07/04/1126294/original.jpg	https://data.globe.gov/system/photos/2019/07/04/1126293/original.jpg	https://data.globe.gov/system/photos/2019/07/04/1126295/original.jpg	https://data.globe.gov/system/photos/2019/07/04/1126297/original.jpg
GV_1021	46.103	-121.607	956.8		https://www.google.com/maps/search/46.103,-121.607	https://data.globe.gov/system/photos/2019/07/04/1126298/original.jpg	https://data.globe.gov/system/photos/2019/07/04/1126300/original.jpg	https://data.globe.gov/system/photos/2019/07/04/1126299/original.jpg	https://data.globe.gov/system/photos/2019/07/04/1126301/original.jpg	https://data.globe.gov/system/photos/2019/07/04/1126303/original.jpg
GV_1022	46.1029	-121.6081	949.1		https://www.google.com/maps/search/46.1029,-121.6081	https://data.globe.gov/system/photos/2019/07/04/1126304/original.jpg	https://data.globe.gov/system/photos/2019/07/04/1126306/original.jpg	https://data.globe.gov/system/photos/2019/07/04/1126305/original.jpg	https://data.globe.gov/system/photos/2019/07/04/1126307/original.jpg	https://data.globe.gov/system/photos/2019/07/04/1126309/original.jpg
GV_1023	46.1033	-121.6075	952.8		https://www.google.com/maps/search/46.1033,-121.6075	https://data.globe.gov/system/photos/2019/07/04/1126310/original.jpg	https://data.globe.gov/system/photos/2019/07/04/1126312/original.jpg	https://data.globe.gov/system/photos/2019/07/04/1126311/original.jpg	https://data.globe.gov/system/photos/2019/07/04/1126313/original.jpg	https://data.globe.gov/system/photos/2019/07/04/1126315/original.jpg
GV_1024	46.1036	-121.6067	958.7		https://www.google.com/maps/search/46.1036,-121.6067	https://data.globe.gov/system/photos/2019/07/04/1126316/original.jpg	https://data.globe.gov/system/photos/2019/07/04/1126318/original.jpg	https://data.globe.gov/system/photos/2019/07/04/1126317/original.jpg	https://data.globe.gov/system/photos/2019/07/04/1126319/original.jpg	https://data.globe.gov/system/photos/2019/07/04/1126321/original.jpg
GV_1025	46.1055	-121.604	967.5		https://www.google.com/maps/search/46.1055,-121.604	https://data.globe.gov/system/photos/2019/07/04/1126322/original.jpg	https://data.globe.gov/system/photos/2019/07/04/1126324/original.jpg	https://data.globe.gov/system/photos/2019/07/04/1126323/original.jpg	https://data.globe.gov/system/photos/2019/07/04/1126325/original.jpg	https://data.globe.gov/system/photos/2019/07/04/1126327/original.jpg
GV_1026	46.1067	-121.6035	971.9		https://www.google.com/maps/search/46.1067,-121.6035	https://data.globe.gov/system/photos/2019/07/04/1126328/original.jpg	https://data.globe.gov/system/photos/2019/07/04/1126330/original.jpg	https://data.globe.gov/system/photos/2019/07/04/1126329/original.jpg	https://data.globe.gov/system/photos/2019/07/04/1126331/original.jpg	https://data.globe.gov/system/photos/2019/07/04/1126333/original.jpg
GV_1027	46.1078	-121.603	975.2		https://www.google.com/maps/search/46.1078,-121.603	https://data.globe.gov/system/photos/2019/07/04/1126334/original.jpg	https://data.globe.gov/system/photos/2019/07/04/1126336/original.jpg	https://data.globe.gov/system/photos/2019/07/04/1126335/original.jpg	https://data.globe.gov/system/photos/2019/07/04/1126337/original.jpg	https://data.globe.gov/system/photos/2019/07/04/1126339/original.jpg
GV_1028	46.109	-121.6023	989.4		https://www.google.com/maps/search/46.109,-121.6023	https://data.globe.gov/system/photos/2019/07/04/1126340/original.jpg	https://data.globe.gov/system/photos/2019/07/04/1126342/original.jpg	https://data.globe.gov/system/photos/2019/07/04/1126341/original.jpg	https://data.globe.gov/system/photos/2019/07/04/1126343/original.jpg	https://data.globe.gov/system/photos/2019/07/04/1126345/original.jpg
GV_1029	46.1092	-121.6013	1002.5		https://www.google.com/maps/search/46.1092,-121.6013	https://data.globe.gov/system/photos/2019/07/04/1126346/original.jpg	https://data.globe.gov/system/photos/2019/07/04/1126348/original.jpg	https://data.globe.gov/system/photos/2019/07/04/1126347/original.jpg	https://data.globe.gov/system/photos/2019/07/04/1126349/original.jpg	https://data.globe.gov/system/photos/2019/07/04/1126351/original.jpg
GV_1030	46.1708	-121.6285	1212.6		https://www.google.com/maps/search/46.1708,-121.6285	https://data.globe.gov/system/photos/2019/07/04/1126352/original.jpg	https://data.globe.gov/system/photos/2019/07/04/1126354/original.jpg	https://data.globe.gov/system/photos/2019/07/04/1126353/original.jpg	https://data.globe.gov/system/photos/2019/07/04/1126355/original.jpg	https://data.globe.gov/system/photos/2019/07/04/1126357/original.jpg
GV_1031	46.1536	-121.6286	1142.7		https://www.google.com/maps/search/46.1536,-121.6286	https://data.globe.gov/system/photos/2019/07/04/1126358/original.jpg	https://data.globe.gov/system/photos/2019/07/04/1126360/original.jpg	https://data.globe.gov/system/photos/2019/07/04/1126359/original.jpg	https://data.globe.gov/system/photos/2019/07/04/1126361/original.jpg	https://data.globe.gov/system/photos/2019/07/04/1126363/original.jpg
GV_1032	46.168	-121.6298	1192.6		https://www.google.com/maps/search/46.168,-121.6298	https://data.globe.gov/system/photos/2019/07/04/1126364/original.jpg	https://data.globe.gov/system/photos/2019/07/04/1126366/original.jpg	https://data.globe.gov/system/photos/2019/07/04/1126365/original.jpg	https://data.globe.gov/system/photos/2019/07/04/1126367/original.jpg	https://data.globe.gov/system/photos/2019/07/04/1126369/original.jpg
GV_1033	46.1707	-121.6265	1226.3		https://www.google.com/maps/search/46.1707,-121.6265	https://data.globe.gov/system/photos/2019/07/04/1126370/original.jpg	https://data.globe.gov/system/photos/2019/07/04/1126372/original.jpg	https://data.globe.gov/system/photos/2019/07/04/1126371/original.jpg	https://data.globe.gov/system/photos/2019/07/04/1126373/original.jpg	https://data.globe.gov/system/photos/2019/07/04/1126375/original.jpg
GV_1034	46.1704	-121.6269	1227.2		https://www.google.com/maps/search/46.1704,-121.6269	https://data.globe.gov/system/photos/2019/07/04/1126376/original.jpg	https://data.globe.gov/system/photos/2019/07/04/1126378/original.jpg	https://data.globe.gov/system/photos/2019/07/04/1126377/original.jpg	https://data.globe.gov/system/photos/2019/07/04/1126379/original.jpg	https://data.globe.gov/system/photos/2019/07/04/1126381/original.jpg
GV_1035	46.1681	-121.6279	1243		https://www.google.com/maps/search/46.1681,-121.6279	https://data.globe.gov/system/photos/2019/07/04/1126382/original.jpg	https://data.globe.gov/system/photos/2019/07/04/1126384/original.jpg	https://data.globe.gov/system/photos/2019/07/04/1126383/original.jpg	https://data.globe.gov/system/photos/2019/07/04/1126385/original.jpg	https://data.globe.gov/system/photos/2019/07/04/1126387/original.jpg
GV_1036	46.1666	-121.6287	1217.2		https://www.google.com/maps/search/46.1666,-121.6287	https://data.globe.gov/system/photos/2019/07/04/1126388/original.jpg	https://data.globe.gov/system/photos/2019/07/04/1126390/original.jpg	https://data.globe.gov/system/photos/2019/07/04/1126389/original.jpg	https://data.globe.gov/system/photos/2019/07/04/1126391/original.jpg	https://data.globe.gov/system/photos/2019/07/04/1126393/original.jpg
GV_1037	46.1656	-121.6292	1196.6		https://www.google.com/maps/search/46.1656,-121.6292	https://data.globe.gov/system/photos/2019/07/04/1126394/original.jpg	https://data.globe.gov/system/photos/2019/07/04/1126396/original.jpg	https://data.globe.gov/system/photos/2019/07/04/1126395/original.jpg	https://data.globe.gov/system/photos/2019/07/04/1126397/original.jpg	https://data.globe.gov/system/photos/2019/07/04/1126399/original.jpg
GV_1038	46.1704	-121.6252	1231.3		https://www.google.com/maps/search/46.1704,-121.6252	https://data.globe.gov/system/photos/2019/07/04/1126400/original.jpg	https://data.globe.gov/system/photos/2019/07/04/1126402/original.jpg	https://data.globe.gov/system/photos/2019/07/04/1126401/original.jpg	https://data.globe.gov/system/photos/2019/07/04/1126403/original.jpg	https://data.globe.gov/system/photos/2019/07/04/1126405/original.jpg
GV_1039	46.1702	-121.6259	1233.1		https://www.google.com/maps/search/46.1702,-121.6259	https://data.globe.gov/system/photos/2019/07/04/1126406/original.jpg	https://data.globe.gov/system/photos/2019/07/04/1126408/original.jpg	https://data.globe.gov/system/photos/2019/07/04/1126407/original.jpg	https://data.globe.gov/system/photos/2019/07/04/1126409/original.jpg	https://data.globe.gov/system/photos/2019/07/04/1126411/original.jpg
GV_1040	46.1904	-121.6436	1210.1		https://www.google.com/maps/search/46.1904,-121.6436	https://data.globe.gov/system/photos/2019/07/04/1126412/original.jpg	https://data.globe.gov/system/photos/2019/07/04/1126414/original.jpg	https://data.globe.gov/system/photos/2019/07/04/1126413/original.jpg	https://data.globe.gov/system/photos/2019/07/04/1126415/original.jpg	https://data.globe.gov/system/photos/2019/07/04/1126417/original.jpg
GV_1041	46.191	-121.6425	1211.6		https://www.google.com/maps/search/46.191,-121.6425	https://data.globe.gov/system/photos/2019/07/04/1126418/original.jpg	https://data.globe.gov/system/photos/2019/07/04/1126420/original.jpg	https://data.globe.gov/system/photos/2019/07/04/1126419/original.jpg	https://data.globe.gov/system/photos/2019/07/04/1126421/original.jpg	https://data.globe.gov/system/photos/2019/07/04/1126423/original.jpg
GV_1042	46.1911	-121.6397	1214.3		https://www.google.com/maps/search/46.1911,-121.6397	https://data.globe.gov/system/photos/2019/07/04/1126424/original.jpg	https://data.globe.gov/system/photos/2019/07/04/1126426/original.jpg	https://data.globe.gov/system/photos/2019/07/04/1126425/original.jpg	https://data.globe.gov/system/photos/2019/07/04/1126427/original.jpg	https://data.globe.gov/system/photos/2019/07/04/1126429/original.jpg
GV_1043	46.1913	-121.6389	1214.8		https://www.google.com/maps/search/46.1913,-121.6389	https://data.globe.gov/system/photos/2019/07/04/1126430/original.jpg	https://data.globe.gov/system/photos/2019/07/04/1126432/original.jpg	https://data.globe.gov/system/photos/2019/07/04/1126431/original.jpg	https://data.globe.gov/system/photos/2019/07/04/1126433/original.jpg	https://data.globe.gov/system/photos/2019/07/04/1126435/original.jpg
GV_1044	46.1907	-121.6376	1223.6		https://www.google.com/maps/search/46.1907,-121.6376	https://data.globe.gov/system/photos/2019/07/04/1126436/original.jpg	https://data.globe.gov/system/photos/2019/07/04/1126438/original.jpg	https://data.globe.gov/system/photos/2019/07/04/1126437/original.jpg	https://data.globe.gov/system/photos/2019/07/04/1126439/original.jpg	https://data.globe.gov/system/photos/2019/07/04/1126441/original.jpg
GV_1045	46.1905	-121.6385	1221.2		https://www.google.com/maps/search/46.1905,-121.6385	https://data.globe.gov/system/photos/2019/07/04/1126442/original.jpg	https://data.globe.gov/system/photos/2019/07/04/1126444/original.jpg	https://data.globe.gov/system/photos/2019/07/04/1126443/original.jpg	https://data.globe.gov/system/photos/2019/07/04/1126445/original.jpg	https://data.globe.gov/system/photos/2019/07/04/1126447/original.jpg
GV_1046	46.1926	-121.6373	1217.2		https://www.google.com/maps/search/46.1926,-121.6373	https://data.globe.gov/system/photos/2019/07/04/1126448/original.jpg	https://data.globe.gov/system/photos/2019/07/04/1126450/original.jpg	https://data.globe.gov/system/photos/2019/07/04/1126449/original.jpg	https://data.globe.gov/system/photos/2019/07/04/1126451/original.jpg	https://data.globe.gov/system/photos/2019/07/04/1126453/original.jpg
GV_1047	46.1928	-121.6382	1215.4		https://www.google.com/maps/search/46.1928,-121.6382	https://data.globe.gov/system/photos/2019/07/04/1126454/original.jpg	https://data.globe.gov/system/photos/2019/07/04/1126456/original.jpg	https://data.globe.gov/system/photos/2019/07/04/1126455/original.jpg	https://data.globe.gov/system/photos/2019/07/04/1126457/original.jpg	https://data.globe.gov/system/photos/2019/07/04/1126459/original.jpg
GV_1048	46.1945	-121.636	1222.8		https://www.google.com/maps/search/46.1945,-121.636	https://data.globe.gov/system/photos/2019/07/04/1126460/original.jpg	https://data.globe.gov/system/photos/2019/07/04/1126462/original.jpg	https://data.globe.gov/system/photos/2019/07/04/1126461/original.jpg	https://data.globe.gov/system/photos/2019/07/04/1126463/original.jpg	https://data.globe.gov/system/photos/2019/07/04/1126465/original.jpg
GV_1049	46.1944	-121.6379	1221.3		https://www.google.com/maps/search/46.1944,-121.6379	https://data.globe.gov/system/photos/2019/07/04/1126466/original.jpg	https://data.globe.gov/system/photos/2019/07/04/1126468/original.jpg	https://data.globe.gov/system/photos/2019/07/04/1126467/original.jpg	https://data.globe.gov/system/photos/2019/07/04/1126469/original.jpg	https://data.globe.gov/system/photos/2019/07/04/1126471/original.jpg
GV_1050	46.2178	-121.6345	1106.8		https://www.google.com/maps/search/46.2178,-121.6345	https://data.globe.gov/system/photos/2019/07/04/1126472/original.jpg	https://data.globe.gov/system/photos/2019/07/04/1126474/original.jpg	https://data.globe.gov/system/photos/2019/07/04/1126473/original.jpg	https://data.globe.gov/system/photos/2019/07/04/1126475/original.jpg	https://data.globe.gov/system/photos/2019/07/04/1126477/original.jpg
GV_1051	46.195	-121.6394	1207.3		https://www.google.com/maps/search/46.195,-121.6394	https://data.globe.gov/system/photos/2019/07/04/1126478/original.jpg	https://data.globe.gov/system/photos/2019/07/04/1126480/original.jpg	https://data.globe.gov/system/photos/2019/07/04/1126479/original.jpg	https://data.globe.gov/system/photos/2019/07/04/1126481/original.jpg	https://data.globe.gov/system/photos/2019/07/04/1126483/original.jpg
GV_1052	46.1962	-121.6389	1204.8		https://www.google.com/maps/search/46.1962,-121.6389	https://data.globe.gov/system/photos/2019/07/04/1126484/original.jpg	https://data.globe.gov/system/photos/2019/07/04/1126486/original.jpg	https://data.globe.gov/system/photos/2019/07/04/1126485/original.jpg	https://data.globe.gov/system/photos/2019/07/04/1126487/original.jpg	https://data.globe.gov/system/photos/2019/07/04/1126489/original.jpg
GV_1053	46.1954	-121.6397	1202.2		https://www.google.com/maps/search/46.1954,-121.6397	https://data.globe.gov/system/photos/2019/07/04/1126490/original.jpg	https://data.globe.gov/system/photos/2019/07/04/1126492/original.jpg	https://data.globe.gov/system/photos/2019/07/04/1126491/original.jpg	https://data.globe.gov/system/photos/2019/07/04/1126493/original.jpg	https://data.globe.gov/system/photos/2019/07/04/1126495/original.jpg
GV_1054	46.2017	-121.6472	1174		https://www.google.com/maps/search/46.2017,-121.6472	https://data.globe.gov/system/photos/2019/07/04/1126502/original.jpg	https://data.globe.gov/system/photos/2019/07/04/1126504/original.jpg	https://data.globe.gov/system/photos/2019/07/04/1126503/original.jpg	https://data.globe.gov/system/photos/2019/07/04/1126505/original.jpg	https://data.globe.gov/system/photos/2019/07/04/1126507/original.jpg
GV_1055	46.2022	-121.647	1172.6		https://www.google.com/maps/search/46.2022,-121.647	https://data.globe.gov/system/photos/2019/07/04/1126508/original.jpg	https://data.globe.gov/system/photos/2019/07/04/1126510/original.jpg	https://data.globe.gov/system/photos/2019/07/04/1126509/original.jpg	https://data.globe.gov/system/photos/2019/07/04/1126511/original.jpg	https://data.globe.gov/system/photos/2019/07/04/1126513/original.jpg
GV_1056	46.2023	-121.6459	1182.9		https://www.google.com/maps/search/46.2023,-121.6459	https://data.globe.gov/system/photos/2019/07/04/1126514/original.jpg	https://data.globe.gov/system/photos/2019/07/04/1126516/original.jpg	https://data.globe.gov/system/photos/2019/07/04/1126515/original.jpg	https://data.globe.gov/system/photos/2019/07/04/1126517/original.jpg	https://data.globe.gov/system/photos/2019/07/04/1126519/original.jpg
GV_1057	46.2171	-121.6345	1117.3		https://www.google.com/maps/search/46.2171,-121.6345	https://data.globe.gov/system/photos/2019/07/04/1126520/original.jpg	https://data.globe.gov/system/photos/2019/07/04/1126522/original.jpg	https://data.globe.gov/system/photos/2019/07/04/1126521/original.jpg	https://data.globe.gov/system/photos/2019/07/04/1126523/original.jpg	https://data.globe.gov/system/photos/2019/07/04/1126525/original.jpg
GV_1058	46.2171	-121.634	1123.4		https://www.google.com/maps/search/46.2171,-121.634	https://data.globe.gov/system/photos/2019/07/04/1126526/original.jpg	https://data.globe.gov/system/photos/2019/07/04/1126528/original.jpg	https://data.globe.gov/system/photos/2019/07/04/1126527/original.jpg	https://data.globe.gov/system/photos/2019/07/04/1126529/original.jpg	https://data.globe.gov/system/photos/2019/07/04/1126531/original.jpg
GV_1059	46.2163	-121.632	1145		https://www.google.com/maps/search/46.2163,-121.632	https://data.globe.gov/system/photos/2019/07/04/1126532/original.jpg	https://data.globe.gov/system/photos/2019/07/04/1126534/original.jpg	https://data.globe.gov/system/photos/2019/07/04/1126533/original.jpg	https://data.globe.gov/system/photos/2019/07/04/1126535/original.jpg	https://data.globe.gov/system/photos/2019/07/04/1126537/original.jpg
GV_1060	46.2157	-121.6288	1177.4		https://www.google.com/maps/search/46.2157,-121.6288	https://data.globe.gov/system/photos/2019/07/04/1126538/original.jpg	https://data.globe.gov/system/photos/2019/07/04/1126540/original.jpg	https://data.globe.gov/system/photos/2019/07/04/1126539/original.jpg	https://data.globe.gov/system/photos/2019/07/04/1126541/original.jpg	https://data.globe.gov/system/photos/2019/07/04/1126543/original.jpg
GV_1061	46.2155	-121.6256	1203.2		https://www.google.com/maps/search/46.2155,-121.6256	https://data.globe.gov/system/photos/2019/07/04/1126544/original.jpg	https://data.globe.gov/system/photos/2019/07/04/1126546/original.jpg	https://data.globe.gov/system/photos/2019/07/04/1126545/original.jpg	https://data.globe.gov/system/photos/2019/07/04/1126547/original.jpg	https://data.globe.gov/system/photos/2019/07/04/1126549/original.jpg
GV_1062	46.2319	-121.6249	1104		https://www.google.com/maps/search/46.2319,-121.6249	https://data.globe.gov/system/photos/2019/07/05/1126550/original.jpg	https://data.globe.gov/system/photos/2019/07/05/1126552/original.jpg	https://data.globe.gov/system/photos/2019/07/05/1126551/original.jpg	https://data.globe.gov/system/photos/2019/07/05/1126553/original.jpg	https://data.globe.gov/system/photos/2019/07/05/1126555/original.jpg
GV_1063	46.2373	-121.629	1096.6		https://www.google.com/maps/search/46.2373,-121.629	https://data.globe.gov/system/photos/2019/07/05/1126556/original.jpg	https://data.globe.gov/system/photos/2019/07/05/1126558/original.jpg	https://data.globe.gov/system/photos/2019/07/05/1126557/original.jpg	https://data.globe.gov/system/photos/2019/07/05/1126559/original.jpg	https://data.globe.gov/system/photos/2019/07/05/1126561/original.jpg
GV_1064	46.2423	-121.6207	1121.9		https://www.google.com/maps/search/46.2423,-121.6207	https://data.globe.gov/system/photos/2019/07/05/1126562/original.jpg	https://data.globe.gov/system/photos/2019/07/05/1126564/original.jpg	https://data.globe.gov/system/photos/2019/07/05/1126563/original.jpg	https://data.globe.gov/system/photos/2019/07/05/1126565/original.jpg	https://data.globe.gov/system/photos/2019/07/05/1126567/original.jpg
GV_1065	46.2426	-121.6199	1119.5		https://www.google.com/maps/search/46.2426,-121.6199	https://data.globe.gov/system/photos/2019/07/05/1126568/original.jpg	https://data.globe.gov/system/photos/2019/07/05/1126570/original.jpg	https://data.globe.gov/system/photos/2019/07/05/1126569/original.jpg	https://data.globe.gov/system/photos/2019/07/05/1126571/original.jpg	https://data.globe.gov/system/photos/2019/07/05/1126574/original.jpg
GV_1066	46.2422	-121.6197	1137.5		https://www.google.com/maps/search/46.2422,-121.6197	https://data.globe.gov/system/photos/2019/07/05/1126577/original.jpg	https://data.globe.gov/system/photos/2019/07/05/1126580/original.jpg	https://data.globe.gov/system/photos/2019/07/05/1126579/original.jpg	https://data.globe.gov/system/photos/2019/07/05/1126581/original.jpg	https://data.globe.gov/system/photos/2019/07/05/1126584/original.jpg
GV_1067	46.2751	-121.6022	1316		https://www.google.com/maps/search/46.2751,-121.6022	https://data.globe.gov/system/photos/2019/07/05/1126586/original.jpg	https://data.globe.gov/system/photos/2019/07/05/1126588/original.jpg	https://data.globe.gov/system/photos/2019/07/05/1126587/original.jpg	https://data.globe.gov/system/photos/2019/07/05/1126589/original.jpg	https://data.globe.gov/system/photos/2019/07/05/1126591/original.jpg
GV_1068	46.2729	-121.5918	1381.2		https://www.google.com/maps/search/46.2729,-121.5918	https://data.globe.gov/system/photos/2019/07/05/1126592/original.jpg	https://data.globe.gov/system/photos/2019/07/05/1126594/original.jpg	https://data.globe.gov/system/photos/2019/07/05/1126593/original.jpg	https://data.globe.gov/system/photos/2019/07/05/1126595/original.jpg	https://data.globe.gov/system/photos/2019/07/05/1126597/original.jpg
GV_1069	46.2692	-121.5787	1439.9		https://www.google.com/maps/search/46.2692,-121.5787	https://data.globe.gov/system/photos/2019/07/05/1126598/original.jpg	https://data.globe.gov/system/photos/2019/07/05/1126600/original.jpg	https://data.globe.gov/system/photos/2019/07/05/1126599/original.jpg	https://data.globe.gov/system/photos/2019/07/05/1126601/original.jpg	https://data.globe.gov/system/photos/2019/07/05/1126603/original.jpg
GV_1070	46.2675	-121.5788	1444.5		https://www.google.com/maps/search/46.2675,-121.5788	https://data.globe.gov/system/photos/2019/07/05/1126604/original.jpg	https://data.globe.gov/system/photos/2019/07/05/1126606/original.jpg	https://data.globe.gov/system/photos/2019/07/05/1126605/original.jpg	https://data.globe.gov/system/photos/2019/07/05/1126607/original.jpg	https://data.globe.gov/system/photos/2019/07/05/1126609/original.jpg
GV_1071	46.2666	-121.5776	1444		https://www.google.com/maps/search/46.2666,-121.5776	https://data.globe.gov/system/photos/2019/07/05/1126610/original.jpg	https://data.globe.gov/system/photos/2019/07/05/1126612/original.jpg	https://data.globe.gov/system/photos/2019/07/05/1126611/original.jpg	https://data.globe.gov/system/photos/2019/07/05/1126613/original.jpg	https://data.globe.gov/system/photos/2019/07/05/1126615/original.jpg
GV_1072	46.2645	-121.5768	1452.9		https://www.google.com/maps/search/46.2645,-121.5768	https://data.globe.gov/system/photos/2019/07/05/1126616/original.jpg	https://data.globe.gov/system/photos/2019/07/05/1126618/original.jpg	https://data.globe.gov/system/photos/2019/07/05/1126617/original.jpg	https://data.globe.gov/system/photos/2019/07/05/1126619/original.jpg	https://data.globe.gov/system/photos/2019/07/05/1126621/original.jpg
GV_1073	46.262	-121.5739	1478.1		https://www.google.com/maps/search/46.262,-121.5739	https://data.globe.gov/system/photos/2019/07/05/1126622/original.jpg	https://data.globe.gov/system/photos/2019/07/05/1126624/original.jpg	https://data.globe.gov/system/photos/2019/07/05/1126623/original.jpg	https://data.globe.gov/system/photos/2019/07/05/1126625/original.jpg	https://data.globe.gov/system/photos/2019/07/05/1126627/original.jpg

GV_1074	46.2573	-121.5679	1556		https://www.google.com/maps/search/46.2573,-121.5679	https://data.globe.gov/system/photos/2019/07/05/1126628/original.jpg	https://data.globe.gov/system/photos/2019/07/05/1126630/original.jpg	https://data.globe.gov/system/photos/2019/07/05/1126629/original.jpg	https://data.globe.gov/system/photos/2019/07/05/1126631/original.jpg	https://data.globe.gov/system/photos/2019/07/05/1126633/original.jpg
GV_1075	46.2564	-121.5665	1583.2		https://www.google.com/maps/search/46.2564,-121.5665	https://data.globe.gov/system/photos/2019/07/05/1126634/original.jpg	https://data.globe.gov/system/photos/2019/07/05/1126636/original.jpg	https://data.globe.gov/system/photos/2019/07/05/1126635/original.jpg	https://data.globe.gov/system/photos/2019/07/05/1126637/original.jpg	https://data.globe.gov/system/photos/2019/07/05/1126639/original.jpg
GV_1076	46.2545	-121.5633	1609.3		https://www.google.com/maps/search/46.2545,-121.5633	https://data.globe.gov/system/photos/2019/07/05/1126640/original.jpg	https://data.globe.gov/system/photos/2019/07/05/1126642/original.jpg	https://data.globe.gov/system/photos/2019/07/05/1126641/original.jpg	https://data.globe.gov/system/photos/2019/07/05/1126643/original.jpg	https://data.globe.gov/system/photos/2019/07/05/1126645/original.jpg
GV_1077	46.2539	-121.5626	1620.6		https://www.google.com/maps/search/46.2539,-121.5626	https://data.globe.gov/system/photos/2019/07/05/1126646/original.jpg	https://data.globe.gov/system/photos/2019/07/05/1126648/original.jpg	https://data.globe.gov/system/photos/2019/07/05/1126647/original.jpg	https://data.globe.gov/system/photos/2019/07/05/1126649/original.jpg	https://data.globe.gov/system/photos/2019/07/05/1126651/original.jpg
GV_1078	46.2536	-121.5608	1643.3		https://www.google.com/maps/search/46.2536,-121.5608	https://data.globe.gov/system/photos/2019/07/05/1126652/original.jpg	https://data.globe.gov/system/photos/2019/07/05/1126654/original.jpg	https://data.globe.gov/system/photos/2019/07/05/1126653/original.jpg	https://data.globe.gov/system/photos/2019/07/05/1126655/original.jpg	https://data.globe.gov/system/photos/2019/07/05/1126657/original.jpg
GV_1079	46.2528	-121.5602	1649.7		https://www.google.com/maps/search/46.2528,-121.5602	https://data.globe.gov/system/photos/2019/07/05/1126658/original.jpg	https://data.globe.gov/system/photos/2019/07/05/1126660/original.jpg	https://data.globe.gov/system/photos/2019/07/05/1126659/original.jpg	https://data.globe.gov/system/photos/2019/07/05/1126661/original.jpg	https://data.globe.gov/system/photos/2019/07/05/1126663/original.jpg
GV_1080	46.2519	-121.5577	1669.7		https://www.google.com/maps/search/46.2519,-121.5577	https://data.globe.gov/system/photos/2019/07/05/1126664/original.jpg	https://data.globe.gov/system/photos/2019/07/05/1126666/original.jpg	https://data.globe.gov/system/photos/2019/07/05/1126665/original.jpg	https://data.globe.gov/system/photos/2019/07/05/1126667/original.jpg	https://data.globe.gov/system/photos/2019/07/05/1126669/original.jpg
GV_1081	46.2519	-121.5578	1671.9		https://www.google.com/maps/search/46.2519,-121.5578	https://data.globe.gov/system/photos/2019/07/05/1126670/original.jpg	https://data.globe.gov/system/photos/2019/07/05/1126672/original.jpg	https://data.globe.gov/system/photos/2019/07/05/1126671/original.jpg	https://data.globe.gov/system/photos/2019/07/05/1126673/original.jpg	https://data.globe.gov/system/photos/2019/07/05/1126675/original.jpg
GV_1082	46.251	-121.5575	1683.9		https://www.google.com/maps/search/46.251,-121.5575	https://data.globe.gov/system/photos/2019/07/05/1126676/original.jpg	https://data.globe.gov/system/photos/2019/07/05/1126678/original.jpg	https://data.globe.gov/system/photos/2019/07/05/1126677/original.jpg	https://data.globe.gov/system/photos/2019/07/05/1126679/original.jpg	https://data.globe.gov/system/photos/2019/07/05/1126681/original.jpg
GV_1083	46.2486	-121.5552	1725.8		https://www.google.com/maps/search/46.2486,-121.5552	https://data.globe.gov/system/photos/2019/07/05/1126682/original.jpg	https://data.globe.gov/system/photos/2019/07/05/1126684/original.jpg	https://data.globe.gov/system/photos/2019/07/05/1126683/original.jpg	https://data.globe.gov/system/photos/2019/07/05/1126685/original.jpg	https://data.globe.gov/system/photos/2019/07/05/1126687/original.jpg
GV_1084	46.2482	-121.5547	1728.8		https://www.google.com/maps/search/46.2482,-121.5547	https://data.globe.gov/system/photos/2019/07/05/1126688/original.jpg	https://data.globe.gov/system/photos/2019/07/05/1126690/original.jpg	https://data.globe.gov/system/photos/2019/07/05/1126689/original.jpg	https://data.globe.gov/system/photos/2019/07/05/1126691/original.jpg	https://data.globe.gov/system/photos/2019/07/05/1126693/original.jpg
GV_1085	46.2476	-121.5543	1732.8		https://www.google.com/maps/search/46.2476,-121.5543	https://data.globe.gov/system/photos/2019/07/05/1126694/original.jpg	https://data.globe.gov/system/photos/2019/07/05/1126696/original.jpg	https://data.globe.gov/system/photos/2019/07/05/1126695/original.jpg	https://data.globe.gov/system/photos/2019/07/05/1126697/original.jpg	https://data.globe.gov/system/photos/2019/07/05/1126699/original.jpg
GV_1086	46.2478	-121.5542	1731.2		https://www.google.com/maps/search/46.2478,-121.5542	https://data.globe.gov/system/photos/2019/07/05/1126700/original.jpg	https://data.globe.gov/system/photos/2019/07/05/1126702/original.jpg	https://data.globe.gov/system/photos/2019/07/05/1126701/original.jpg	https://data.globe.gov/system/photos/2019/07/05/1126703/original.jpg	https://data.globe.gov/system/photos/2019/07/05/1126705/original.jpg
GV_1087	46.2473	-121.5538	1733.6		https://www.google.com/maps/search/46.2473,-121.5538	https://data.globe.gov/system/photos/2019/07/05/1126706/original.jpg	https://data.globe.gov/system/photos/2019/07/05/1126708/original.jpg	https://data.globe.gov/system/photos/2019/07/05/1126707/original.jpg	https://data.globe.gov/system/photos/2019/07/05/1126709/original.jpg	https://data.globe.gov/system/photos/2019/07/05/1126711/original.jpg
GV_1088	46.2447	-121.5512	1766.3		https://www.google.com/maps/search/46.2447,-121.5512	https://data.globe.gov/system/photos/2019/07/05/1126712/original.jpg	https://data.globe.gov/system/photos/2019/07/05/1126714/original.jpg	https://data.globe.gov/system/photos/2019/07/05/1126713/original.jpg	https://data.globe.gov/system/photos/2019/07/05/1126715/original.jpg	https://data.globe.gov/system/photos/2019/07/05/1126717/original.jpg
GV_1089	46.2434	-121.5484	1789.9		https://www.google.com/maps/search/46.2434,-121.5484	https://data.globe.gov/system/photos/2019/07/05/1126718/original.jpg	https://data.globe.gov/system/photos/2019/07/05/1126720/original.jpg	https://data.globe.gov/system/photos/2019/07/05/1126719/original.jpg	https://data.globe.gov/system/photos/2019/07/05/1126721/original.jpg	https://data.globe.gov/system/photos/2019/07/05/1126723/original.jpg
GV_1090	46.2433	-121.5486	1788.9		https://www.google.com/maps/search/46.2433,-121.5486	https://data.globe.gov/system/photos/2019/07/05/1126724/original.jpg	https://data.globe.gov/system/photos/2019/07/05/1126726/original.jpg	https://data.globe.gov/system/photos/2019/07/05/1126725/original.jpg	https://data.globe.gov/system/photos/2019/07/05/1126727/original.jpg	https://data.globe.gov/system/photos/2019/07/05/1126729/original.jpg
GV_1091	46.2406	-121.5456	1831.9		https://www.google.com/maps/search/46.2406,-121.5456	https://data.globe.gov/system/photos/2019/07/05/1126730/original.jpg	https://data.globe.gov/system/photos/2019/07/05/1126732/original.jpg	https://data.globe.gov/system/photos/2019/07/05/1126731/original.jpg	https://data.globe.gov/system/photos/2019/07/05/1126733/original.jpg	https://data.globe.gov/system/photos/2019/07/05/1126735/original.jpg
GV_1092	46.2406	-121.545	1833.5		https://www.google.com/maps/search/46.2406,-121.545	https://data.globe.gov/system/photos/2019/07/05/1126736/original.jpg	https://data.globe.gov/system/photos/2019/07/05/1126738/original.jpg	https://data.globe.gov/system/photos/2019/07/05/1126737/original.jpg	https://data.globe.gov/system/photos/2019/07/05/1126739/original.jpg	https://data.globe.gov/system/photos/2019/07/05/1126741/original.jpg
GV_1093	46.2413	-121.5451	1825.8		https://www.google.com/maps/search/46.2413,-121.5451	https://data.globe.gov/system/photos/2019/07/05/1126742/original.jpg	https://data.globe.gov/system/photos/2019/07/05/1126744/original.jpg	https://data.globe.gov/system/photos/2019/07/05/1126743/original.jpg	https://data.globe.gov/system/photos/2019/07/05/1126745/original.jpg	https://data.globe.gov/system/photos/2019/07/05/1126747/original.jpg
GV_1094	46.2418	-121.5445	1828.7		https://www.google.com/maps/search/46.2418,-121.5445	https://data.globe.gov/system/photos/2019/07/05/1126748/original.jpg	https://data.globe.gov/system/photos/2019/07/05/1126752/original.jpg	https://data.globe.gov/system/photos/2019/07/05/1126749/original.jpg	https://data.globe.gov/system/photos/2019/07/05/1126756/original.jpg	https://data.globe.gov/system/photos/2019/07/05/1126759/original.jpg
GV_1095	46.2422	-121.5439	1831.6		https://www.google.com/maps/search/46.2422,-121.5439	https://data.globe.gov/system/photos/2019/07/05/1126760/original.jpg	https://data.globe.gov/system/photos/2019/07/05/1126762/original.jpg	https://data.globe.gov/system/photos/2019/07/05/1126761/original.jpg	https://data.globe.gov/system/photos/2019/07/05/1126763/original.jpg	https://data.globe.gov/system/photos/2019/07/05/1126765/original.jpg
GV_1096	46.2432	-121.5437	1821.8		https://www.google.com/maps/search/46.2432,-121.5437	https://data.globe.gov/system/photos/2019/07/05/1126766/original.jpg	https://data.globe.gov/system/photos/2019/07/05/1126768/original.jpg	https://data.globe.gov/system/photos/2019/07/05/1126767/original.jpg	https://data.globe.gov/system/photos/2019/07/05/1126769/original.jpg	https://data.globe.gov/system/photos/2019/07/05/1126771/original.jpg
GV_1097	46.2343	-121.5422	1934.8		https://www.google.com/maps/search/46.2343,-121.5422	https://data.globe.gov/system/photos/2019/07/05/1126778/original.jpg	https://data.globe.gov/system/photos/2019/07/05/1126780/original.jpg	https://data.globe.gov/system/photos/2019/07/05/1126779/original.jpg	https://data.globe.gov/system/photos/2019/07/05/1126781/original.jpg	https://data.globe.gov/system/photos/2019/07/05/1126783/original.jpg
GV_1098	46.2382	-121.5472	1855.7		https://www.google.com/maps/search/46.2382,-121.5472	https://data.globe.gov/system/photos/2019/07/05/1126784/original.jpg	https://data.globe.gov/system/photos/2019/07/05/1126786/original.jpg	https://data.globe.gov/system/photos/2019/07/05/1126785/original.jpg	https://data.globe.gov/system/photos/2019/07/05/1126787/original.jpg	https://data.globe.gov/system/photos/2019/07/05/1126789/original.jpg
GV_1099	46.2374	-121.5483	1850.8		https://www.google.com/maps/search/46.2374,-121.5483	https://data.globe.gov/system/photos/2019/07/05/1126790/original.jpg	https://data.globe.gov/system/photos/2019/07/05/1126792/original.jpg	https://data.globe.gov/system/photos/2019/07/05/1126791/original.jpg	https://data.globe.gov/system/photos/2019/07/05/1126793/original.jpg	https://data.globe.gov/system/photos/2019/07/05/1126795/original.jpg
GV_1100	46.2357	-121.5454	1891.6		https://www.google.com/maps/search/46.2357,-121.5454	https://data.globe.gov/system/photos/2019/07/05/1126796/original.jpg	https://data.globe.gov/system/photos/2019/07/05/1126798/original.jpg	https://data.globe.gov/system/photos/2019/07/05/1126797/original.jpg	https://data.globe.gov/system/photos/2019/07/05/1126799/original.jpg	https://data.globe.gov/system/photos/2019/07/05/1126801/original.jpg
GV_1101	46.2335	-121.5403	1973		https://www.google.com/maps/search/46.2335,-121.5403	https://data.globe.gov/system/photos/2019/07/05/1126802/original.jpg	https://data.globe.gov/system/photos/2019/07/05/1126804/original.jpg	https://data.globe.gov/system/photos/2019/07/05/1126803/original.jpg	https://data.globe.gov/system/photos/2019/07/05/1126805/original.jpg	https://data.globe.gov/system/photos/2019/07/05/1126807/original.jpg
GV_1102	46.2354	-121.5436	1909.9		https://www.google.com/maps/search/46.2354,-121.5436	https://data.globe.gov/system/photos/2019/07/05/1126808/original.jpg	https://data.globe.gov/system/photos/2019/07/05/1126810/original.jpg	https://data.globe.gov/system/photos/2019/07/05/1126809/original.jpg	https://data.globe.gov/system/photos/2019/07/05/1126811/original.jpg	https://data.globe.gov/system/photos/2019/07/05/1126813/original.jpg
GV_1103	24.1573	120.6593	94.8		https://www.google.com/maps/search/24.1573,120.6593	https://data.globe.gov/system/photos/2019/07/05/1126907/original.jpg	https://data.globe.gov/system/photos/2019/07/05/1126909/original.jpg	https://data.globe.gov/system/photos/2019/07/05/1126908/original.jpg	https://data.globe.gov/system/photos/2019/07/05/1126910/original.jpg	https://data.globe.gov/system/photos/2019/07/05/1126912/original.jpg
GV_1104	24.1545	120.6599	93		https://www.google.com/maps/search/24.1545,120.6599	https://data.globe.gov/system/photos/2019/07/05/1126928/original.jpg	https://data.globe.gov/system/photos/2019/07/05/1126930/original.jpg	https://data.globe.gov/system/photos/2019/07/05/1126929/original.jpg	https://data.globe.gov/system/photos/2019/07/05/1126931/original.jpg	https://data.globe.gov/system/photos/2019/07/05/1126933/original.jpg
GV_1105	24.1568	120.6596	94.2		https://www.google.com/maps/search/24.1568,120.6596	https://data.globe.gov/system/photos/2019/07/06/1127480/original.jpg	https://data.globe.gov/system/photos/2019/07/06/1127482/original.jpg	https://data.globe.gov/system/photos/2019/07/06/1127481/original.jpg	https://data.globe.gov/system/photos/2019/07/06/1127483/original.jpg	https://data.globe.gov/system/photos/2019/07/06/1127485/original.jpg
GV_1106	24.1562	120.6667	101.1		https://www.google.com/maps/search/24.1562,120.6667	https://data.globe.gov/system/photos/2019/07/06/1127486/original.jpg	https://data.globe.gov/system/photos/2019/07/06/1127488/original.jpg	https://data.globe.gov/system/photos/2019/07/06/1127487/original.jpg	https://data.globe.gov/system/photos/2019/07/06/1127489/original.jpg	https://data.globe.gov/system/photos/2019/07/06/1127491/original.jpg
GV_1107	41.4192	-76.5709	610.8		https://www.google.com/maps/search/41.4192,-76.5709	https://data.globe.gov/system/photos/2019/07/06/1128113/original.jpg	https://data.globe.gov/system/photos/2019/07/06/1128115/original.jpg	https://data.globe.gov/system/photos/2019/07/06/1128114/original.jpg	https://data.globe.gov/system/photos/2019/07/06/1128116/original.jpg	https://data.globe.gov/system/photos/2019/07/06/1128118/original.jpg
GV_1108	46.3684	-117.0578	230		https://www.google.com/maps/search/46.3684,-117.0578	https://data.globe.gov/system/photos/2019/07/06/1128139/original.jpg	https://data.globe.gov/system/photos/2019/07/06/1128141/original.jpg	https://data.globe.gov/system/photos/2019/07/06/1128140/original.jpg	https://data.globe.gov/system/photos/2019/07/06/1128142/original.jpg	https://data.globe.gov/system/photos/2019/07/06/1128144/original.jpg
GV_1109	24.1559	120.6666	102.4		https://www.google.com/maps/search/24.1559,120.6666	https://data.globe.gov/system/photos/2019/07/06/1128294/original.jpg	https://data.globe.gov/system/photos/2019/07/06/1128296/original.jpg	https://data.globe.gov/system/photos/2019/07/06/1128295/original.jpg	https://data.globe.gov/system/photos/2019/07/06/1128297/original.jpg	https://data.globe.gov/system/photos/2019/07/06/1128299/original.jpg
GV_1110	25.0224	121.3056	73.1		https://www.google.com/maps/search/25.0224,121.3056	https://data.globe.gov/system/photos/2019/07/07/1128452/original.jpg	https://data.globe.gov/system/photos/2019/07/07/1128454/original.jpg	https://data.globe.gov/system/photos/2019/07/07/1128453/original.jpg	https://data.globe.gov/system/photos/2019/07/07/1128455/original.jpg	https://data.globe.gov/system/photos/2019/07/07/1128457/original.jpg
GV_1111	25.0242	121.3038	71.9		https://www.google.com/maps/search/25.0242,121.3038	https://data.globe.gov/system/photos/2019/07/07/1128462/original.jpg	https://data.globe.gov/system/photos/2019/07/07/1128464/original.jpg	https://data.globe.gov/system/photos/2019/07/07/1128463/original.jpg	https://data.globe.gov/system/photos/2019/07/07/1128465/original.jpg	https://data.globe.gov/system/photos/2019/07/07/1128467/original.jpg
GV_1112	25.0245	121.3022	71.7		https://www.google.com/maps/search/25.0245,121.3022	https://data.globe.gov/system/photos/2019/07/07/1128469/original.jpg	https://data.globe.gov/system/photos/2019/07/07/1128471/original.jpg	https://data.globe.gov/system/photos/2019/07/07/1128470/original.jpg	https://data.globe.gov/system/photos/2019/07/07/1128472/original.jpg	https://data.globe.gov/system/photos/2019/07/07/1128474/original.jpg
GV_1113	46.4175	-117.1833	244.3		https://www.google.com/maps/search/46.4175,-117.1833	https://data.globe.gov/system/photos/2019/06/27/1128669/original.jpg	https://data.globe.gov/system/photos/2019/06/27/1128671/original.jpg	https://data.globe.gov/system/photos/2019/06/27/1128670/original.jpg	https://data.globe.gov/system/photos/2019/06/27/1128672/original.jpg	https://data.globe.gov/system/photos/2019/06/27/1128674/original.jpg
GV_1114	46.4135	-117.206	286.3		https://www.google.com/maps/search/46.4135,-117.206	https://data.globe.gov/system/photos/2019/06/27/1128675/original.jpg	https://data.globe.gov/system/photos/2019/06/27/1128677/original.jpg	https://data.globe.gov/system/photos/2019/06/27/1128676/original.jpg	https://data.globe.gov/system/photos/2019/06/27/1128678/original.jpg	https://data.globe.gov/system/photos/2019/06/27/1128680/original.jpg
GV_1115	46.4186	-117.1919	235.6		https://www.google.com/maps/search/46.4186,-117.1919	https://data.globe.gov/system/photos/2019/06/27/1128681/original.jpg	https://data.globe.gov/system/photos/2019/06/27/1128683/original.jpg	https://data.globe.gov/system/photos/2019/06/27/1128682/original.jpg	https://data.globe.gov/system/photos/2019/06/27/1128684/original.jpg	https://data.globe.gov/system/photos/2019/06/27/1128686/original.jpg
GV_1116	46.4184	-117.1888	244.2		https://www.google.com/maps/search/46.4184,-117.1888	https://data.globe.gov/system/photos/2019/06/27/1128687/original.jpg	https://data.globe.gov/system/photos/2019/06/27/1128689/original.jpg	https://data.globe.gov/system/photos/2019/06/27/1128688/original.jpg	https://data.globe.gov/system/photos/2019/06/27/1128690/original.jpg	https://data.globe.gov/system/photos/2019/06/27/1128692/original.jpg
GV_1117	46.4195	-117.0266	250.4		https://www.google.com/maps/search/46.4195,-117.0266	https://data.globe.gov/system/photos/2019/06/27/1128693/original.jpg	https://data.globe.gov/system/photos/2019/06/27/1128695/original.jpg	https://data.globe.gov/system/photos/2019/06/27/1128694/original.jpg	https://data.globe.gov/system/photos/2019/06/27/1128696/original.jpg	https://data.globe.gov/system/photos/2019/06/27/1128698/original.jpg
GV_1118	46.412	-117.0259	259.1		https://www.google.com/maps/search/46.412,-117.0259	https://data.globe.gov/system/photos/2019/06/27/1128699/original.jpg	https://data.globe.gov/system/photos/2019/06/27/1128701/original.jpg	https://data.globe.gov/system/photos/2019/06/27/1128700/original.jpg	https://data.globe.gov/system/photos/2019/06/27/1128702/original.jpg	https://data.globe.gov/system/photos/2019/06/27/1128704/original.jpg
GV_1119	46.4127	-117.0264	257.8		https://www.google.com/maps/search/46.4127,-117.0264	https://data.globe.gov/system/photos/2019/06/27/1128705/original.jpg	https://data.globe.gov/system/photos/2019/06/27/1128707/original.jpg	https://data.globe.gov/system/photos/2019/06/27/1128706/original.jpg	https://data.globe.gov/system/photos/2019/06/27/1128708/original.jpg	https://data.globe.gov/system/photos/2019/06/27/1128710/original.jpg
GV_1120	41.002	-96.248	318.3		https://www.google.com/maps/search/41.002,-96.248	https://data.globe.gov/system/photos/2019/07/04/1128711/original.jpg	https://data.globe.gov/system/photos/2019/07/04/1128713/original.jpg	https://data.globe.gov/system/photos/2019/07/04/1128712/original.jpg	https://data.globe.gov/system/photos/2019/07/04/1128714/original.jpg	https://data.globe.gov/system/photos/2019/07/04/1128716/original.jpg
GV_1121	41.0065	-96.2474	317.3		https://www.google.com/maps/search/41.0065,-96.2474	https://data.globe.gov/system/photos/2019/07/04/1128717/original.jpg	https://data.globe.gov/system/photos/2019/07/04/1128719/original.jpg	https://data.globe.gov/system/photos/2019/07/04/1128718/original.jpg	https://data.globe.gov/system/photos/2019/07/04/1128720/original.jpg	https://data.globe.gov/system/photos/2019/07/04/1128722/original.jpg
GV_1122	40.9872	-96.2139	354.9		https://www.google.com/maps/search/40.9872,-96.2139	https://data.globe.gov/system/photos/2019/07/06/1128723/original.jpg	https://data.globe.gov/system/photos/2019/07/06/1128725/original.jpg	https://data.globe.gov/system/photos/2019/07/06/1128724/original.jpg	https://data.globe.gov/system/photos/2019/07/06/1128726/original.jpg	https://data.globe.gov/system/photos/2019/07/06/1128728/original.jpg
GV_1123	40.9901	-96.2177	333.4		https://www.google.com/maps/search/40.9901,-96.2177	https://data.globe.gov/system/photos/2019/07/06/1128729/original.jpg	https://data.globe.gov/system/photos/2019/07/06/1128731/original.jpg	https://data.globe.gov/system/photos/2019/07/06/1128730/original.jpg	https://data.globe.gov/system/photos/2019/07/06/1128732/original.jpg	https://data.globe.gov/system/photos/2019/07/06/1128734/original.jpg
GV_1124	40.2155	-75.5105	76.6		https://www.google.com/maps/search/40.2155,-75.5105	https://data.globe.gov/system/photos/2019/07/07/1128933/original.jpg	https://data.globe.gov/system/photos/2019/07/07/1128936/original.jpg	https://data.globe.gov/system/photos/2019/07/07/1128935/original.jpg	https://data.globe.gov/system/photos/2019/07/07/1128937/original.jpg	https://data.globe.gov/system/photos/2019/07/07/1128939/original.jpg
GV_1125	47.467	-87.898	221.4		https://www.google.com/maps/search/47.467,-87.898	https://data.globe.gov/system/photos/2019/07/03/1128965/original.jpg	https://data.globe.gov/system/photos/2019/07/03/1128967/original.jpg	https://data.globe.gov/system/photos/2019/07/03/1128966/original.jpg	https://data.globe.gov/system/photos/2019/07/03/1128968/original.jpg	https://data.globe.gov/system/photos/2019/07/03/1128970/original.jpg
GV_1126	42.0784	-83.2241	177.7		https://www.google.com/maps/search/42.0784,-83.2241	https://data.globe.gov/system/photos/2019/07/07/1128977/original.jpg	https://data.globe.gov/system/photos/2019/07/07/1128979/original.jpg	https://data.globe.gov/system/photos/2019/07/07/1128978/original.jpg	https://data.globe.gov/system/photos/2019/07/07/1128980/original.jpg	https://data.globe.gov/system/photos/2019/07/07/1128982/original.jpg
GV_1127	45.8083	-122.798	7.2		https://www.google.com/maps/search/45.8083,-122.798	https://data.globe.gov/system/photos/2019/07/07/1129072/original.jpg	https://data.globe.gov/system/photos/2019/07/07/1129074/original.jpg	https://data.globe.gov/system/photos/2019/07/07/1129073/original.jpg	https://data.globe.gov/system/photos/2019/07/07/1129075/original.jpg	https://data.globe.gov/system/photos/2019/07/07/1129077/original.jpg
GV_1128	45.7727	-122.773	4.1		https://www.google.com/maps/search/45.7727,-122.773	https://data.globe.gov/system/photos/2019/07/07/1129078/original.jpg	https://data.globe.gov/system/photos/2019/07/07/1129080/original.jpg	https://data.globe.gov/system/photos/2019/07/07/1129079/original.jpg	https://data.globe.gov/system/photos/2019/07/07/1129081/original.jpg	https://data.globe.gov/system/photos/2019/07/07/1129083/original.jpg
GV_1129	45.7732	-122.774	8.1		https://www.google.com/maps/search/45.7732,-122.774	https://data.globe.gov/system/photos/2019/07/07/1129084/original.jpg	https://data.globe.gov/system/photos/2019/07/07/1129086/original.jpg	https://data.globe.gov/system/photos/2019/07/07/1129085/original.jpg	https://data.globe.gov/system/photos/2019/07/07/1129087/original.jpg	https://data.globe.gov/system/photos/2019/07/07/1129089/original.jpg
GV_1130	45.7571	-122.7713	8.1		https://www.google.com/maps/search/45.7571,-122.7713	https://data.globe.gov/system/photos/2019/07/07/1129090/original.jpg	https://data.globe.gov/system/photos/2019/07/07/1129092/original.jpg	https://data.globe.gov/system/photos/2019/07/07/1129091/original.jpg	https://data.globe.gov/system/photos/2019/07/07/1129093/original.jpg	https://data.globe.gov/system/photos/2019/07/07/1129095/original.jpg
GV_1131	45.8086	-122.7987	7.1		https://www.google.com/maps/search/45.8086,-122.7987	https://data.globe.gov/system/photos/2019/07/07/1129096/original.jpg	https://data.globe.gov/system/photos/2019/07/07/1129098/original.jpg	https://data.globe.gov/system/photos/2019/07/07/1129097/original.jpg	https://data.globe.gov/system/photos/2019/07/07/1129099/original.jpg	https://data.globe.gov/system/photos/2019/07/07/1129101/original.jpg
GV_1132	45.7785	-122.7789	9.1		https://www.google.com/maps/search/45.7785,-122.7789	https://data.globe.gov/system/photos/2019/07/07/1129102/original.jpg	https://data.globe.gov/system/photos/2019/07/07/1129104/original.jpg	https://data.globe.gov/system/photos/2019/07/07/1129103/original.jpg	https://data.globe.gov/system/photos/2019/07/07/1129105/original.jpg	https://data.globe.gov/system/photos/2019/07/07/1129107/original.jpg
GV_1133	45.7789	-122.7789	6.1		https://www.google.com/maps/search/45.7789,-122.7789	https://data.globe.gov/system/photos/2019/07/07/1129108/original.jpg	https://data.globe.gov/system/photos/2019/07/07/1129110/original.jpg	https://data.globe.gov/system/photos/2019/07/07/1129109/original.jpg	https://data.globe.gov/system/photos/2019/07/07/1129111/original.jpg	https://data.globe.gov/system/photos/2019/07/07/1129113/original.jpg
GV_1134	38.0495	-122.1606	5		https://www.google.com/maps/search/38.0495,-122.1606	https://data.globe.gov/system/photos/2019/07/07/1129146/original.jpg	https://data.globe.gov/system/photos/2019/07/07/1129148/original.jpg	https://data.globe.gov/system/photos/2019/07/07/1129147/original.jpg	https://data.globe.gov/system/photos/2019/07/07/1129149/original.jpg	https://data.globe.gov/system/photos/2019/07/07/1129151/original.jpg
GV_1135	45.7777	-122.7781	9.5		https://www.google.com/maps/search/45.7777,-122.7781	https://data.globe.gov/system/photos/2019/07/07/1129158/original.jpg	https://data.globe.gov/system/photos/2019/07/07/1129160/original.jpg	https://data.globe.gov/system/photos/2019/07/07/1129159/original.jpg	https://data.globe.gov/system/photos/2019/07/07/1129161/original.jpg	https://data.globe.gov/system/photos/2019/07/07/1129163/original.jpg
GV_1136	-34.8944	148.9753	552.8		https://www.google.com/maps/search/-34.8944,148.9753	https://data.globe.gov/system/photos/2019/07/07/1129176/original.jpg	https://data.globe.gov/system/photos/2019/07/07/1129178/original.jpg	https://data.globe.gov/system/photos/2019/07/07/1129177/original.jpg	https://data.globe.gov/system/photos/2019/07/07/1129179/original.jpg	https://data.globe.gov/system/photos/2019/07/07/1129181/original.jpg
GV_1137	-34.8475	148.2964	263.2		https://www.google.com/maps/search/-34.8475,148.2964	https://data.globe.gov/system/photos/2019/07/07/1129182/original.jpg	https://data.globe.gov/system/photos/2019/07/07/1129184/original.jpg	https://data.globe.gov/system/photos/2019/07/07/1129183/original.jpg	https://data.globe.gov/system/photos/2019/07/07/1129185/original.jpg	https://data.globe.gov/system/photos/2019/07/07/1129187/original.jpg
GV_1138	42.1204	-83.2194	178.4		https://www.google.com/maps/search/42.1204,-83.2194	https://data.globe.gov/system/photos/2019/07/08/1129194/original.jpg	https://data.globe.gov/system/photos/2019/07/08/1129196/original.jpg	https://data.globe.gov/system/photos/2019/07/08/1129195/original.jpg	https://data.globe.gov/system/photos/2019/07/08/1129197/original.jpg	https://data.globe.gov/system/photos/2019/07/08/1129199/original.jpg
GV_1139	42.1188	-83.2143	178.3		https://www.google.com/maps/search/42.1188,-83.2143	https://data.globe.gov/system/photos/2019/07/08/1129218/original.jpg	https://data.globe.gov/system/photos/2019/07/08/1129220/original.jpg	https://data.globe.gov/system/photos/2019/07/08/1129219/original.jpg	https://data.globe.gov/system/photos/2019/07/08/1129221/original.jpg	https://data.globe.gov/system/photos/2019/07/08/1129223/original.jpg
GV_1140	-35.162	147.5833	211		https://www.google.com/maps/search/-35.162,147.5833	https://data.globe.gov/system/photos/2019/07/08/1129235/original.jpg	https://data.globe.gov/system/photos/2019/07/08/1129237/original.jpg	https://data.globe.gov/system/photos/2019/07/08/1129236/original.jpg	https://data.globe.gov/system/photos/2019/07/08/1129238/original.jpg	https://data.globe.gov/system/photos/2019/07/08/1129240/original.jpg
GV_1141	46.3854	-112.4495	2018.5		https://www.google.com/maps/search/46.3854,-112.4495	https://data.globe.gov/system/photos/2019/07/06/1129241/original.jpg	https://data.globe.gov/system/photos/2019/07/06/1129243/original.jpg	https://data.globe.gov/system/photos/2019/07/06/1129242/original.jpg	https://data.globe.gov/system/photos/2019/07/06/1129244/original.jpg	https://data.globe.gov/system/photos/2019/07/06/1129246/original.jpg
GV_1142	46.3965	-112.4804	1970.1		https://www.google.com/maps/search/46.3965,-112.4804	https://data.globe.gov/system/photos/2019/07/07/1129247/original.jpg	https://data.globe.gov/system/photos/2019/07/07/1129249/original.jpg	https://data.globe.gov/system/photos/2019/07/07/1129248/original.jpg	https://data.globe.gov/system/photos/2019/07/07/1129250/original.jpg	https://data.globe.gov/system/photos/2019/07/07/1129252/original.jpg
GV_1143	-35.1059	147.2036	190.3		https://www.google.com/maps/search/-35.1059,147.2036	https://data.globe.gov/system/photos/2019/07/08/1129276/original.jpg	https://data.globe.gov/system/photos/2019/07/08/1129278/original.jpg	https://data.globe.gov/system/photos/2019/07/08/1129277/original.jpg	https://data.globe.gov/system/photos/2019/07/08/1129279/original.jpg	https://data.globe.gov/system/photos/2019/07/08/1129281/original.jpg
GV_1144	-35.0597	147.0421	172.8		https://www.google.com/maps/search/-35.0597,147.0421	https://data.globe.gov/system/photos/2019/07/08/1129288/original.jpg	https://data.globe.gov/system/photos/2019/07/08/1129290/original.jpg	https://data.globe.gov/system/photos/2019/07/08/1129289/original.jpg	https://data.globe.gov/system/photos/2019/07/08/1129291/original.jpg	https://data.globe.gov/system/photos/2019/07/08/1129293/original.jpg
GV_1145	-34.5089	144.9487	92.3		https://www.google.com/maps/search/-34.5089,144.9487	https://data.globe.gov/system/photos/2019/07/08/1129436/original.jpg	https://data.globe.gov/system/photos/2019/07/08/1129438/original.jpg	https://data.globe.gov/system/photos/2019/07/08/1129437/original.jpg	https://data.globe.gov/system/photos/2019/07/08/1129439/original.jpg	https://data.globe.gov/system/photos/2019/07/08/1129441/original.jpg
GV_1146	25.0905	121.5225	7.8		https://www.google.com/maps/search/25.0905,121.5225	https://data.globe.gov/system/photos/2019/07/08/1129446/original.jpg	https://data.globe.gov/system/photos/2019/07/08/1129448/original.jpg	https://data.globe.gov/system/photos/2019/07/08/1129447/original.jpg	https://data.globe.gov/system/photos/2019/07/08/1129449/original.jpg	https://data.globe.gov/system/photos/2019/07/08/1129451/original.jpg
GV_1147	25.0903	121.5222	9.6		https://www.google.com/maps/search/25.0903,121.5222	https://data.globe.gov/system/photos/2019/07/08/1129525/original.jpg	https://data.globe.gov/system/photos/2019/07/08/1129527/original.jpg	https://data.globe.gov/system/photos/2019/07/08/1129526/original.jpg	https://data.globe.gov/system/photos/2019/07/08/1129528/original.jpg	https://data.globe.gov/system/photos/2019/07/08/1129530/original.jpg
GV_1148	25.0166	121.2988	83.8		https://www.google.com/maps/search/25.0166,121.2988	https://data.globe.gov/system/photos/2019/07/08/1129532/original.jpg	https://data.globe.gov/system/photos/2019/07/08/1129534/original.jpg	https://data.globe.gov/system/photos/2019/07/08/1129533/original.jpg	https://data.globe.gov/system/photos/2019/07/08/1129535/original.jpg	https://data.globe.gov/system/photos/2019/07/08/1129537/original.jpg
GV_1149	39.1339	-77.186	140		https://www.google.com/maps/search/39.1339,-77.186	https://data.globe.gov/system/photos/2019/06/25/1129745/original.jpg	https://data.globe.gov/system/photos/2019/06/25/1129747/original.jpg	https://data.globe.gov/system/photos/2019/06/25/1129746/original.jpg	https://data.globe.gov/system/photos/2019/06/25/1129748/original.jpg	https://data.globe.gov/system/photos/2019/06/25/1129750/original.jpg
GV_1150	64	150	777.2		https://www.google.com/maps/search/64,150	https://data.globe.gov/system/photos/2019/06/16/1129831/original.jpg	https://data.globe.gov/system/photos/2019/06/16/1129833/original.jpg	https://data.globe.gov/system/photos/2019/06/16/1129832/original.jpg	https://data.globe.gov/system/photos/2019/06/16/1129834/original.jpg	https://data.globe.gov/system/photos/2019/06/16/1129836/original.jpg
GV_1151	65	150	256.7		https://www.google.com/maps/search/65,150	https://data.globe.gov/system/photos/2019/06/17/1129843/original.jpg	https://data.globe.gov/system/photos/2019/06/17/1129845/original.jpg	https://data.globe.gov/system/photos/2019/06/17/1129844/original.jpg	https://data.globe.gov/system/photos/2019/06/17/1129846/original.jpg	https://data.globe.gov/system/photos/2019/06/17/1129848/original.jpg
GV_1152	35.2354	-83.1868	811.6		https://www.google.com/maps/search/35.2354,-83.1868	https://data.globe.gov/system/photos/2019/07/06/1129849/original.jpg	https://data.globe.gov/system/photos/2019/07/06/1129851/original.jpg	https://data.globe.gov/system/photos/2019/07/06/1129850/original.jpg	https://data.globe.gov/system/photos/2019/07/06/1129852/original.jpg	https://data.globe.gov/system/photos/2019/07/06/1129854/original.jpg
GV_1153	40.8312	-74.212	93.5		https://www.google.com/maps/search/40.8312,-74.212	https://data.globe.gov/system/photos/2019/07/06/1130064/original.jpg	https://data.globe.gov/system/photos/2019/07/06/1130066/original.jpg	https://data.globe.gov/system/photos/2019/07/06/1130065/original.jpg	https://data.globe.gov/system/photos/2019/07/06/1130067/original.jpg	https://data.globe.gov/system/photos/2019/07/06/1130069/original.jpg
GV_1154	40.8317	-74.2126	93.8		https://www.google.com/maps/search/40.8317,-74.2126	https://data.globe.gov/system/photos/2019/07/06/1130071/original.jpg	https://data.globe.gov/system/photos/2019/07/06/1130073/original.jpg	https://data.globe.gov/system/photos/2019/07/06/1130072/original.jpg	https://data.globe.gov/system/photos/2019/07/06/1130074/original.jpg	https://data.globe.gov/system/photos/2019/07/06/1130076/original.jpg
GV_1155	40.84	-74.1892	59.3		https://www.google.com/maps/search/40.84,-74.1892	https://data.globe.gov/system/photos/2019/07/06/1130085/original.jpg	https://data.globe.gov/system/photos/2019/07/06/1130088/original.jpg	https://data.globe.gov/system/photos/2019/07/06/1130086/original.jpg	https://data.globe.gov/system/photos/2019/07/06/1130089/original.jpg	https://data.globe.gov/system/photos/2019/07/06/1130091/original.jpg
GV_1156	40.8403	-74.1906	55.5		https://www.google.com/maps/search/40.8403,-74.1906	https://data.globe.gov/system/photos/2019/07/06/1130092/original.jpg	https://data.globe.gov/system/photos/2019/07/06/1130094/original.jpg	https://data.globe.gov/system/photos/2019/07/06/1130093/original.jpg	https://data.globe.gov/system/photos/2019/07/06/1130095/original.jpg	https://data.globe.gov/system/photos/2019/07/06/1130097/original.jpg
GV_1157	40.8242	-74.2046	87		https://www.google.com/maps/search/40.8242,-74.2046	https://data.globe.gov/system/photos/2019/07/08/1130099/original.jpg	https://data.globe.gov/system/photos/2019/07/08/1130101/original.jpg	https://data.globe.gov/system/photos/2019/07/08/1130100/original.jpg	https://data.globe.gov/system/photos/2019/07/08/1130102/original.jpg	https://data.globe.gov/system/photos/2019/07/08/1130104/original.jpg
GV_1158	53.4286	-0.6173	31		https://www.google.com/maps/search/53.4286,-0.6173	https://data.globe.gov/system/photos/2019/07/08/1130158/original.jpg	https://data.globe.gov/system/photos/2019/07/08/1130160/original.jpg	https://data.globe.gov/system/photos/2019/07/08/1130159/original.jpg	https://data.globe.gov/system/photos/2019/07/08/1130161/original.jpg	https://data.globe.gov/system/photos/2019/07/08/1130163/original.jpg
GV_1159	35.2728	-97.4947	359.2		https://www.google.com/maps/search/35.2728,-97.4947	https://data.globe.gov/system/photos/2019/06/22/1130243/original.jpg	https://data.globe.gov/system/photos/2019/06/22/1130245/original.jpg	https://data.globe.gov/system/photos/2019/06/22/1130244/original.jpg	https://data.globe.gov/system/photos/2019/06/22/1130246/original.jpg	https://data.globe.gov/system/photos/2019/06/22/1130248/original.jpg
GV_1160	38.8541	-77.153	68.5		https://www.google.com/maps/search/38.8541,-77.153	https://data.globe.gov/system/photos/2019/07/09/1130391/original.jpg	https://data.globe.gov/system/photos/2019/07/09/1130393/original.jpg	https://data.globe.gov/system/photos/2019/07/09/1130392/original.jpg	https://data.globe.gov/system/photos/2019/07/09/1130394/original.jpg	https://data.globe.gov/system/photos/2019/07/09/1130396/original.jpg
GV_1161	38.9941	-76.8406	62		https://www.google.com/maps/search/38.9941,-76.8406	https://data.globe.gov/system/photos/2019/07/09/1130878/original.jpg	https://data.globe.gov/system/photos/2019/07/09/1130880/original.jpg	https://data.globe.gov/system/photos/2019/07/09/1130879/original.jpg	https://data.globe.gov/system/photos/2019/07/09/1130881/original.jpg	https://data.globe.gov/system/photos/2019/07/09/1130883/original.jpg
GV_1162	38.0455	-122.1609	3		https://www.google.com/maps/search/38.0455,-122.1609	https://data.globe.gov/system/photos/2019/07/09/1130914/original.jpg	https://data.globe.gov/system/photos/2019/07/09/1130916/original.jpg	https://data.globe.gov/system/photos/2019/07/09/1130915/original.jpg	https://data.globe.gov/system/photos/2019/07/09/1130917/original.jpg	https://data.globe.gov/system/photos/2019/07/09/1130919/original.jpg
GV_1163	53.411	-0.6006	44.9		https://www.google.com/maps/search/53.411,-0.6006	https://data.globe.gov/system/photos/2019/07/09/1131245/original.jpg	https://data.globe.gov/system/photos/2019/07/09/1131247/original.jpg	https://data.globe.gov/system/photos/2019/07/09/1131246/original.jpg	https://data.globe.gov/system/photos/2019/07/09/1131248/original.jpg	https://data.globe.gov/system/photos/2019/07/09/1131250/original.jpg

GV_1164	51.2123	6.275	61.6		https://www.google.com/maps/search/51.2123,6.275	https://data.globe.gov/system/photos/2019/07/09/1131542/original.jpg	https://data.globe.gov/system/photos/2019/07/09/1131544/original.jpg	https://data.globe.gov/system/photos/2019/07/09/1131543/original.jpg	https://data.globe.gov/system/photos/2019/07/09/1131545/original.jpg	https://data.globe.gov/system/photos/2019/07/09/1131547/original.jpg
GV_1165	53.4158	-0.6058	38		https://www.google.com/maps/search/53.4158,-0.6058	https://data.globe.gov/system/photos/2019/07/10/1132297/original.jpg	https://data.globe.gov/system/photos/2019/07/10/1132299/original.jpg	https://data.globe.gov/system/photos/2019/07/10/1132298/original.jpg	https://data.globe.gov/system/photos/2019/07/10/1132300/original.jpg	https://data.globe.gov/system/photos/2019/07/10/1132302/original.jpg
GV_1166	25.0216	121.3054	76.3		https://www.google.com/maps/search/25.0216,121.3054	https://data.globe.gov/system/photos/2019/07/11/1132314/original.jpg	https://data.globe.gov/system/photos/2019/07/11/1132316/original.jpg	https://data.globe.gov/system/photos/2019/07/11/1132315/original.jpg	https://data.globe.gov/system/photos/2019/07/11/1132317/original.jpg	https://data.globe.gov/system/photos/2019/07/11/1132319/original.jpg
GV_1167	38.0499	-122.1612	2.1		https://www.google.com/maps/search/38.0499,-122.1612	https://data.globe.gov/system/photos/2019/07/11/1132436/original.jpg	https://data.globe.gov/system/photos/2019/07/11/1132438/original.jpg	https://data.globe.gov/system/photos/2019/07/11/1132437/original.jpg	https://data.globe.gov/system/photos/2019/07/11/1132439/original.jpg	https://data.globe.gov/system/photos/2019/07/11/1132441/original.jpg
GV_1168	38.0448	-122.1598	1.8		https://www.google.com/maps/search/38.0448,-122.1598	https://data.globe.gov/system/photos/2019/07/11/1132631/original.jpg	https://data.globe.gov/system/photos/2019/07/11/1132634/original.jpg	https://data.globe.gov/system/photos/2019/07/11/1132633/original.jpg	https://data.globe.gov/system/photos/2019/07/11/1132635/original.jpg	https://data.globe.gov/system/photos/2019/07/11/1132637/original.jpg
GV_1169	50.5099	21.6364	163		https://www.google.com/maps/search/50.5099,21.6364	https://data.globe.gov/system/photos/2019/07/11/1132652/original.jpg	https://data.globe.gov/system/photos/2019/07/11/1132654/original.jpg	https://data.globe.gov/system/photos/2019/07/11/1132653/original.jpg	https://data.globe.gov/system/photos/2019/07/11/1132655/original.jpg	https://data.globe.gov/system/photos/2019/07/11/1132657/original.jpg
GV_1170	50.5093	21.6382	161		https://www.google.com/maps/search/50.5093,21.6382	https://data.globe.gov/system/photos/2019/07/11/1132659/original.jpg	https://data.globe.gov/system/photos/2019/07/11/1132661/original.jpg	https://data.globe.gov/system/photos/2019/07/11/1132660/original.jpg	https://data.globe.gov/system/photos/2019/07/11/1132662/original.jpg	https://data.globe.gov/system/photos/2019/07/11/1132664/original.jpg
GV_1171	53.4134	-0.605	37.8		https://www.google.com/maps/search/53.4134,-0.605	https://data.globe.gov/system/photos/2019/07/11/1132851/original.jpg	https://data.globe.gov/system/photos/2019/07/11/1132853/original.jpg	https://data.globe.gov/system/photos/2019/07/11/1132852/original.jpg	https://data.globe.gov/system/photos/2019/07/11/1132854/original.jpg	https://data.globe.gov/system/photos/2019/07/11/1132856/original.jpg
GV_1172	13.7844	100.5339	7.3		https://www.google.com/maps/search/13.7844,100.5339	https://data.globe.gov/system/photos/2019/07/12/1133406/original.jpg	https://data.globe.gov/system/photos/2019/07/12/1133408/original.jpg	https://data.globe.gov/system/photos/2019/07/12/1133407/original.jpg	https://data.globe.gov/system/photos/2019/07/12/1133409/original.jpg	https://data.globe.gov/system/photos/2019/07/12/1133411/original.jpg
GV_1173	13.7844	100.5342	8.1		https://www.google.com/maps/search/13.7844,100.5342	https://data.globe.gov/system/photos/2019/07/12/1133419/original.jpg	https://data.globe.gov/system/photos/2019/07/12/1133421/original.jpg	https://data.globe.gov/system/photos/2019/07/12/1133420/original.jpg	https://data.globe.gov/system/photos/2019/07/12/1133422/original.jpg	https://data.globe.gov/system/photos/2019/07/12/1133424/original.jpg
GV_1174	53.429	-0.6105	35.4		https://www.google.com/maps/search/53.429,-0.6105	https://data.globe.gov/system/photos/2019/07/12/1133688/original.jpg	https://data.globe.gov/system/photos/2019/07/12/1133690/original.jpg	https://data.globe.gov/system/photos/2019/07/12/1133689/original.jpg	https://data.globe.gov/system/photos/2019/07/12/1133691/original.jpg	https://data.globe.gov/system/photos/2019/07/12/1133693/original.jpg
GV_1175	53.414	-0.6051	37.9		https://www.google.com/maps/search/53.414,-0.6051	https://data.globe.gov/system/photos/2019/07/12/1133712/original.jpg	https://data.globe.gov/system/photos/2019/07/12/1133714/original.jpg	https://data.globe.gov/system/photos/2019/07/12/1133713/original.jpg	https://data.globe.gov/system/photos/2019/07/12/1133715/original.jpg	https://data.globe.gov/system/photos/2019/07/12/1133717/original.jpg
GV_1176	45.9948	-108.0024	878.5		https://www.google.com/maps/search/45.9948,-108.0024	https://data.globe.gov/system/photos/2019/07/12/1134030/original.jpg	https://data.globe.gov/system/photos/2019/07/12/1134033/original.jpg	https://data.globe.gov/system/photos/2019/07/12/1134032/original.jpg	https://data.globe.gov/system/photos/2019/07/12/1134035/original.jpg	https://data.globe.gov/system/photos/2019/07/12/1134038/original.jpg
GV_1177	53.429	-0.6107	35.4		https://www.google.com/maps/search/53.429,-0.6107	https://data.globe.gov/system/photos/2019/07/13/1134699/original.jpg	https://data.globe.gov/system/photos/2019/07/13/1134701/original.jpg	https://data.globe.gov/system/photos/2019/07/13/1134700/original.jpg	https://data.globe.gov/system/photos/2019/07/13/1134702/original.jpg	https://data.globe.gov/system/photos/2019/07/13/1134704/original.jpg
GV_1178	53.4141	-0.6051	38		https://www.google.com/maps/search/53.4141,-0.6051	https://data.globe.gov/system/photos/2019/07/13/1134711/original.jpg	https://data.globe.gov/system/photos/2019/07/13/1134713/original.jpg	https://data.globe.gov/system/photos/2019/07/13/1134712/original.jpg	https://data.globe.gov/system/photos/2019/07/13/1134714/original.jpg	https://data.globe.gov/system/photos/2019/07/13/1134716/original.jpg
GV_1179	2.884	-74.1949	393.1		https://www.google.com/maps/search/2.884,-74.1949	https://data.globe.gov/system/photos/2019/07/12/1134681/original.jpg	https://data.globe.gov/system/photos/2019/07/12/1134683/original.jpg	https://data.globe.gov/system/photos/2019/07/12/1134682/original.jpg	https://data.globe.gov/system/photos/2019/07/12/1134684/original.jpg	https://data.globe.gov/system/photos/2019/07/12/1134686/original.jpg
GV_1180	3.2483	-74.3616	770.6		https://www.google.com/maps/search/3.2483,-74.3616	https://data.globe.gov/system/photos/2019/07/13/1134692/original.jpg	https://data.globe.gov/system/photos/2019/07/13/1134694/original.jpg	https://data.globe.gov/system/photos/2019/07/13/1134693/original.jpg	https://data.globe.gov/system/photos/2019/07/13/1134695/original.jpg	https://data.globe.gov/system/photos/2019/07/13/1134697/original.jpg
GV_1181	45.5068	-122.682	49		https://www.google.com/maps/search/45.5068,-122.682	https://data.globe.gov/system/photos/2019/07/13/1134726/original.jpg	https://data.globe.gov/system/photos/2019/07/13/1134728/original.jpg	https://data.globe.gov/system/photos/2019/07/13/1134727/original.jpg	https://data.globe.gov/system/photos/2019/07/13/1134729/original.jpg	https://data.globe.gov/system/photos/2019/07/13/1134731/original.jpg
GV_1182	45.5073	-122.6828	51.2		https://www.google.com/maps/search/45.5073,-122.6828	https://data.globe.gov/system/photos/2019/07/13/1134732/original.jpg	https://data.globe.gov/system/photos/2019/07/13/1134734/original.jpg	https://data.globe.gov/system/photos/2019/07/13/1134733/original.jpg	https://data.globe.gov/system/photos/2019/07/13/1134735/original.jpg	https://data.globe.gov/system/photos/2019/07/13/1134737/original.jpg
GV_1183	45.5079	-122.6818	46.6		https://www.google.com/maps/search/45.5079,-122.6818	https://data.globe.gov/system/photos/2019/07/13/1134738/original.jpg	https://data.globe.gov/system/photos/2019/07/13/1134740/original.jpg	https://data.globe.gov/system/photos/2019/07/13/1134739/original.jpg	https://data.globe.gov/system/photos/2019/07/13/1134741/original.jpg	https://data.globe.gov/system/photos/2019/07/13/1134743/original.jpg
GV_1184	45.5078	-122.681	44.2		https://www.google.com/maps/search/45.5078,-122.681	https://data.globe.gov/system/photos/2019/07/13/1134744/original.jpg	https://data.globe.gov/system/photos/2019/07/13/1134746/original.jpg	https://data.globe.gov/system/photos/2019/07/13/1134745/original.jpg	https://data.globe.gov/system/photos/2019/07/13/1134747/original.jpg	https://data.globe.gov/system/photos/2019/07/13/1134749/original.jpg
GV_1185	45.5078	-122.6804	42.7		https://www.google.com/maps/search/45.5078,-122.6804	https://data.globe.gov/system/photos/2019/07/13/1134750/original.jpg	https://data.globe.gov/system/photos/2019/07/13/1134752/original.jpg	https://data.globe.gov/system/photos/2019/07/13/1134751/original.jpg	https://data.globe.gov/system/photos/2019/07/13/1134753/original.jpg	https://data.globe.gov/system/photos/2019/07/13/1134755/original.jpg
GV_1186	45.5079	-122.6801	41.4		https://www.google.com/maps/search/45.5079,-122.6801	https://data.globe.gov/system/photos/2019/07/13/1134756/original.jpg	https://data.globe.gov/system/photos/2019/07/13/1134758/original.jpg	https://data.globe.gov/system/photos/2019/07/13/1134757/original.jpg	https://data.globe.gov/system/photos/2019/07/13/1134759/original.jpg	https://data.globe.gov/system/photos/2019/07/13/1134761/original.jpg
GV_1187	45.508	-122.6805	42.8		https://www.google.com/maps/search/45.508,-122.6805	https://data.globe.gov/system/photos/2019/07/13/1134762/original.jpg	https://data.globe.gov/system/photos/2019/07/13/1134764/original.jpg	https://data.globe.gov/system/photos/2019/07/13/1134763/original.jpg	https://data.globe.gov/system/photos/2019/07/13/1134765/original.jpg	https://data.globe.gov/system/photos/2019/07/13/1134767/original.jpg
GV_1188	45.5081	-122.6813	45		https://www.google.com/maps/search/45.5081,-122.6813	https://data.globe.gov/system/photos/2019/07/13/1134768/original.jpg	https://data.globe.gov/system/photos/2019/07/13/1134770/original.jpg	https://data.globe.gov/system/photos/2019/07/13/1134769/original.jpg	https://data.globe.gov/system/photos/2019/07/13/1134771/original.jpg	https://data.globe.gov/system/photos/2019/07/13/1134773/original.jpg
GV_1189	45.5081	-122.6821	46.9		https://www.google.com/maps/search/45.5081,-122.6821	https://data.globe.gov/system/photos/2019/07/13/1134774/original.jpg	https://data.globe.gov/system/photos/2019/07/13/1134776/original.jpg	https://data.globe.gov/system/photos/2019/07/13/1134775/original.jpg	https://data.globe.gov/system/photos/2019/07/13/1134781/original.jpg	https://data.globe.gov/system/photos/2019/07/13/1134785/original.jpg
GV_1190	45.5118	-122.68	34.3		https://www.google.com/maps/search/45.5118,-122.68	https://data.globe.gov/system/photos/2019/07/13/1134790/original.jpg	https://data.globe.gov/system/photos/2019/07/13/1134792/original.jpg	https://data.globe.gov/system/photos/2019/07/13/1134791/original.jpg	https://data.globe.gov/system/photos/2019/07/13/1134793/original.jpg	https://data.globe.gov/system/photos/2019/07/13/1134795/original.jpg
GV_1191	45.5113	-122.6805	37		https://www.google.com/maps/search/45.5113,-122.6805	https://data.globe.gov/system/photos/2019/07/13/1134796/original.jpg	https://data.globe.gov/system/photos/2019/07/13/1134798/original.jpg	https://data.globe.gov/system/photos/2019/07/13/1134797/original.jpg	https://data.globe.gov/system/photos/2019/07/13/1134799/original.jpg	https://data.globe.gov/system/photos/2019/07/13/1134801/original.jpg
GV_1192	45.5113	-122.6868	53.6		https://www.google.com/maps/search/45.5113,-122.6868	https://data.globe.gov/system/photos/2019/07/13/1134802/original.jpg	https://data.globe.gov/system/photos/2019/07/13/1134804/original.jpg	https://data.globe.gov/system/photos/2019/07/13/1134803/original.jpg	https://data.globe.gov/system/photos/2019/07/13/1134805/original.jpg	https://data.globe.gov/system/photos/2019/07/13/1134814/original.jpg
GV_1193	51.5038	-0.1194	8.7		https://www.google.com/maps/search/51.5038,-0.1194	https://data.globe.gov/system/photos/2019/07/13/1134854/original.jpg	https://data.globe.gov/system/photos/2019/07/13/1134856/original.jpg	https://data.globe.gov/system/photos/2019/07/13/1134855/original.jpg	https://data.globe.gov/system/photos/2019/07/13/1134857/original.jpg	https://data.globe.gov/system/photos/2019/07/13/1134859/original.jpg
GV_1194	51.504	-0.0338	3.6		https://www.google.com/maps/search/51.504,-0.0338	https://data.globe.gov/system/photos/2019/07/13/1134860/original.jpg	https://data.globe.gov/system/photos/2019/07/13/1134862/original.jpg	https://data.globe.gov/system/photos/2019/07/13/1134861/original.jpg	https://data.globe.gov/system/photos/2019/07/13/1134863/original.jpg	https://data.globe.gov/system/photos/2019/07/13/1134865/original.jpg
GV_1195	45.5988	-122.6793	5.8		https://www.google.com/maps/search/45.5988,-122.6793	https://data.globe.gov/system/photos/2019/07/14/1135104/original.jpg	https://data.globe.gov/system/photos/2019/07/14/1135106/original.jpg	https://data.globe.gov/system/photos/2019/07/14/1135105/original.jpg	https://data.globe.gov/system/photos/2019/07/14/1135107/original.jpg	https://data.globe.gov/system/photos/2019/07/14/1135109/original.jpg
GV_1196	45.5988	-122.6805	5.9		https://www.google.com/maps/search/45.5988,-122.6805	https://data.globe.gov/system/photos/2019/07/14/1135110/original.jpg	https://data.globe.gov/system/photos/2019/07/14/1135112/original.jpg	https://data.globe.gov/system/photos/2019/07/14/1135111/original.jpg	https://data.globe.gov/system/photos/2019/07/14/1135113/original.jpg	https://data.globe.gov/system/photos/2019/07/14/1135115/original.jpg
GV_1197	45.5998	-122.6816	7.3		https://www.google.com/maps/search/45.5998,-122.6816	https://data.globe.gov/system/photos/2019/07/14/1135116/original.jpg	https://data.globe.gov/system/photos/2019/07/14/1135118/original.jpg	https://data.globe.gov/system/photos/2019/07/14/1135117/original.jpg	https://data.globe.gov/system/photos/2019/07/14/1135119/original.jpg	https://data.globe.gov/system/photos/2019/07/14/1135121/original.jpg
GV_1198	42.1226	-83.2073	178.3		https://www.google.com/maps/search/42.1226,-83.2073	https://data.globe.gov/system/photos/2019/07/14/1135407/original.jpg	https://data.globe.gov/system/photos/2019/07/14/1135409/original.jpg	https://data.globe.gov/system/photos/2019/07/14/1135408/original.jpg	https://data.globe.gov/system/photos/2019/07/14/1135410/original.jpg	https://data.globe.gov/system/photos/2019/07/14/1135412/original.jpg
GV_1199	53.4289	-0.6106	35.5		https://www.google.com/maps/search/53.4289,-0.6106	https://data.globe.gov/system/photos/2019/07/14/1135431/original.jpg	https://data.globe.gov/system/photos/2019/07/14/1135433/original.jpg	https://data.globe.gov/system/photos/2019/07/14/1135432/original.jpg	https://data.globe.gov/system/photos/2019/07/14/1135434/original.jpg	https://data.globe.gov/system/photos/2019/07/14/1135436/original.jpg
GV_1200	53.414	-0.6051	37.9		https://www.google.com/maps/search/53.414,-0.6051	https://data.globe.gov/system/photos/2019/07/14/1135449/original.jpg	https://data.globe.gov/system/photos/2019/07/14/1135451/original.jpg	https://data.globe.gov/system/photos/2019/07/14/1135450/original.jpg	https://data.globe.gov/system/photos/2019/07/14/1135452/original.jpg	https://data.globe.gov/system/photos/2019/07/14/1135454/original.jpg
GV_1201	51.4779	-9.00E-04	45.6		https://www.google.com/maps/search/51.4779,-0.0009	https://data.globe.gov/system/photos/2019/07/14/1135603/original.jpg	https://data.globe.gov/system/photos/2019/07/14/1135605/original.jpg	https://data.globe.gov/system/photos/2019/07/14/1135604/original.jpg	https://data.globe.gov/system/photos/2019/07/14/1135606/original.jpg	https://data.globe.gov/system/photos/2019/07/14/1135608/original.jpg
GV_1202	51.3318	0.0525	173.3		https://www.google.com/maps/search/51.3318,0.0525	https://data.globe.gov/system/photos/2019/07/14/1135613/original.jpg	https://data.globe.gov/system/photos/2019/07/14/1135615/original.jpg	https://data.globe.gov/system/photos/2019/07/14/1135614/original.jpg	https://data.globe.gov/system/photos/2019/07/14/1135616/original.jpg	https://data.globe.gov/system/photos/2019/07/14/1135618/original.jpg
GV_1203	51.3314	0.0531	174.2		https://www.google.com/maps/search/51.3314,0.0531	https://data.globe.gov/system/photos/2019/07/14/1135619/original.jpg	https://data.globe.gov/system/photos/2019/07/14/1135621/original.jpg	https://data.globe.gov/system/photos/2019/07/14/1135620/original.jpg	https://data.globe.gov/system/photos/2019/07/14/1135622/original.jpg	https://data.globe.gov/system/photos/2019/07/14/1135624/original.jpg
GV_1204	51.3311	0.053	174.5		https://www.google.com/maps/search/51.3311,0.053	https://data.globe.gov/system/photos/2019/07/14/1135625/original.jpg	https://data.globe.gov/system/photos/2019/07/14/1135627/original.jpg	https://data.globe.gov/system/photos/2019/07/14/1135626/original.jpg	https://data.globe.gov/system/photos/2019/07/14/1135628/original.jpg	https://data.globe.gov/system/photos/2019/07/14/1135630/original.jpg
GV_1205	51.4819	-0.006	7.2		https://www.google.com/maps/search/51.4819,-0.006	https://data.globe.gov/system/photos/2019/07/14/1135631/original.jpg	https://data.globe.gov/system/photos/2019/07/14/1135633/original.jpg	https://data.globe.gov/system/photos/2019/07/14/1135632/original.jpg	https://data.globe.gov/system/photos/2019/07/14/1135634/original.jpg	https://data.globe.gov/system/photos/2019/07/14/1135636/original.jpg
GV_1206	51.4805	-0.0045	10.5		https://www.google.com/maps/search/51.4805,-0.0045	https://data.globe.gov/system/photos/2019/07/14/1135637/original.jpg	https://data.globe.gov/system/photos/2019/07/14/1135639/original.jpg	https://data.globe.gov/system/photos/2019/07/14/1135638/original.jpg	https://data.globe.gov/system/photos/2019/07/14/1135640/original.jpg	https://data.globe.gov/system/photos/2019/07/14/1135642/original.jpg
GV_1207	51.4791	-0.0035	17.6		https://www.google.com/maps/search/51.4791,-0.0035	https://data.globe.gov/system/photos/2019/07/14/1135643/original.jpg	https://data.globe.gov/system/photos/2019/07/14/1135645/original.jpg	https://data.globe.gov/system/photos/2019/07/14/1135644/original.jpg	https://data.globe.gov/system/photos/2019/07/14/1135646/original.jpg	https://data.globe.gov/system/photos/2019/07/14/1135648/original.jpg
GV_1208	51.4777	-0.0015	39.7		https://www.google.com/maps/search/51.4777,-0.0015	https://data.globe.gov/system/photos/2019/07/14/1135649/original.jpg	https://data.globe.gov/system/photos/2019/07/14/1135651/original.jpg	https://data.globe.gov/system/photos/2019/07/14/1135650/original.jpg	https://data.globe.gov/system/photos/2019/07/14/1135652/original.jpg	https://data.globe.gov/system/photos/2019/07/14/1135654/original.jpg
GV_1209	51.4759	6.00E-04	45.3		https://www.google.com/maps/search/51.4759,0.0006	https://data.globe.gov/system/photos/2019/07/14/1135655/original.jpg	https://data.globe.gov/system/photos/2019/07/14/1135657/original.jpg	https://data.globe.gov/system/photos/2019/07/14/1135656/original.jpg	https://data.globe.gov/system/photos/2019/07/14/1135658/original.jpg	https://data.globe.gov/system/photos/2019/07/14/1135660/original.jpg
GV_1210	51.5015	-0.1232	0.1		https://www.google.com/maps/search/51.5015,-0.1232	https://data.globe.gov/system/photos/2019/07/14/1135661/original.jpg	https://data.globe.gov/system/photos/2019/07/14/1135663/original.jpg	https://data.globe.gov/system/photos/2019/07/14/1135662/original.jpg	https://data.globe.gov/system/photos/2019/07/14/1135664/original.jpg	https://data.globe.gov/system/photos/2019/07/14/1135666/original.jpg
GV_1211	51.5045	-0.0331	3.4		https://www.google.com/maps/search/51.5045,-0.0331	https://data.globe.gov/system/photos/2019/07/15/1135937/original.jpg	https://data.globe.gov/system/photos/2019/07/15/1135939/original.jpg	https://data.globe.gov/system/photos/2019/07/15/1135938/original.jpg	https://data.globe.gov/system/photos/2019/07/15/1135940/original.jpg	https://data.globe.gov/system/photos/2019/07/15/1135942/original.jpg
GV_1212	53.4291	-0.6103	35.4		https://www.google.com/maps/search/53.4291,-0.6103	https://data.globe.gov/system/photos/2019/07/15/1135962/original.jpg	https://data.globe.gov/system/photos/2019/07/15/1135964/original.jpg	https://data.globe.gov/system/photos/2019/07/15/1135963/original.jpg	https://data.globe.gov/system/photos/2019/07/15/1135965/original.jpg	https://data.globe.gov/system/photos/2019/07/15/1135967/original.jpg
GV_1213	39.4884	-77.805	120.6		https://www.google.com/maps/search/39.4884,-77.805	https://data.globe.gov/system/photos/2019/07/11/1136145/original.jpg	https://data.globe.gov/system/photos/2019/07/11/1136147/original.jpg	https://data.globe.gov/system/photos/2019/07/11/1136146/original.jpg	https://data.globe.gov/system/photos/2019/07/11/1136148/original.jpg	https://data.globe.gov/system/photos/2019/07/11/1136150/original.jpg
GV_1214	40.4468	-79.9926	224.1		https://www.google.com/maps/search/40.4468,-79.9926	https://data.globe.gov/system/photos/2019/07/15/1136159/original.jpg	https://data.globe.gov/system/photos/2019/07/15/1136161/original.jpg	https://data.globe.gov/system/photos/2019/07/15/1136160/original.jpg	https://data.globe.gov/system/photos/2019/07/15/1136162/original.jpg	https://data.globe.gov/system/photos/2019/07/15/1136164/original.jpg
GV_1215	46.3687	-117.0579	229.3		https://www.google.com/maps/search/46.3687,-117.0579	https://data.globe.gov/system/photos/2019/07/15/1136295/original.jpg	https://data.globe.gov/system/photos/2019/07/15/1136298/original.jpg	https://data.globe.gov/system/photos/2019/07/15/1136297/original.jpg	https://data.globe.gov/system/photos/2019/07/15/1136306/original.jpg	https://data.globe.gov/system/photos/2019/07/15/1136311/original.jpg
GV_1216	45.8705	-112.0979	1327.3		https://www.google.com/maps/search/45.8705,-112.0979	https://data.globe.gov/system/photos/2019/07/13/1136323/original.jpg	https://data.globe.gov/system/photos/2019/07/13/1136325/original.jpg	https://data.globe.gov/system/photos/2019/07/13/1136324/original.jpg	https://data.globe.gov/system/photos/2019/07/13/1136326/original.jpg	https://data.globe.gov/system/photos/2019/07/13/1136328/original.jpg
GV_1217	46.0088	-110.9105	1903.3		https://www.google.com/maps/search/46.0088,-110.9105	https://data.globe.gov/system/photos/2019/07/14/1136329/original.jpg	https://data.globe.gov/system/photos/2019/07/14/1136331/original.jpg	https://data.globe.gov/system/photos/2019/07/14/1136330/original.jpg	https://data.globe.gov/system/photos/2019/07/14/1136332/original.jpg	https://data.globe.gov/system/photos/2019/07/14/1136334/original.jpg
GV_1218	52.6503	-0.478	29.1		https://www.google.com/maps/search/52.6503,-0.478	https://data.globe.gov/system/photos/2019/07/15/1136368/original.jpg	https://data.globe.gov/system/photos/2019/07/15/1136370/original.jpg	https://data.globe.gov/system/photos/2019/07/15/1136369/original.jpg	https://data.globe.gov/system/photos/2019/07/15/1136371/original.jpg	https://data.globe.gov/system/photos/2019/07/15/1136373/original.jpg
GV_1219	52.2019	0.1151	12.7		https://www.google.com/maps/search/52.2019,0.1151	https://data.globe.gov/system/photos/2019/07/15/1136374/original.jpg	https://data.globe.gov/system/photos/2019/07/15/1136376/original.jpg	https://data.globe.gov/system/photos/2019/07/15/1136375/original.jpg	https://data.globe.gov/system/photos/2019/07/15/1136377/original.jpg	https://data.globe.gov/system/photos/2019/07/15/1136379/original.jpg
GV_1220	52.2042	0.1145	11.8		https://www.google.com/maps/search/52.2042,0.1145	https://data.globe.gov/system/photos/2019/07/15/1136386/original.jpg	https://data.globe.gov/system/photos/2019/07/15/1136388/original.jpg	https://data.globe.gov/system/photos/2019/07/15/1136387/original.jpg	https://data.globe.gov/system/photos/2019/07/15/1136389/original.jpg	https://data.globe.gov/system/photos/2019/07/15/1136391/original.jpg
GV_1221	52.2024	0.1126	14.7		https://www.google.com/maps/search/52.2024,0.1126	https://data.globe.gov/system/photos/2019/07/15/1136392/original.jpg	https://data.globe.gov/system/photos/2019/07/15/1136394/original.jpg	https://data.globe.gov/system/photos/2019/07/15/1136393/original.jpg	https://data.globe.gov/system/photos/2019/07/15/1136395/original.jpg	https://data.globe.gov/system/photos/2019/07/15/1136397/original.jpg
GV_1222	52.2036	0.1121	13		https://www.google.com/maps/search/52.2036,0.1121	https://data.globe.gov/system/photos/2019/07/15/1136398/original.jpg	https://data.globe.gov/system/photos/2019/07/15/1136400/original.jpg	https://data.globe.gov/system/photos/2019/07/15/1136399/original.jpg	https://data.globe.gov/system/photos/2019/07/15/1136401/original.jpg	https://data.globe.gov/system/photos/2019/07/15/1136403/original.jpg
GV_1223	52.2082	0.1151	11.9		https://www.google.com/maps/search/52.2082,0.1151	https://data.globe.gov/system/photos/2019/07/15/1136404/original.jpg	https://data.globe.gov/system/photos/2019/07/15/1136424/original.jpg	https://data.globe.gov/system/photos/2019/07/15/1136416/original.jpg	https://data.globe.gov/system/photos/2019/07/15/1136429/original.jpg	https://data.globe.gov/system/photos/2019/07/15/1136435/original.jpg
GV_1224	44.5661	-123.2767	78.1		https://www.google.com/maps/search/44.5661,-123.2767	https://data.globe.gov/system/photos/2019/07/15/1136603/original.jpg	https://data.globe.gov/system/photos/2019/07/15/1136605/original.jpg	https://data.globe.gov/system/photos/2019/07/15/1136604/original.jpg	https://data.globe.gov/system/photos/2019/07/15/1136606/original.jpg	https://data.globe.gov/system/photos/2019/07/15/1136608/original.jpg
GV_1225	42.3275	-83.0483	182.2		https://www.google.com/maps/search/42.3275,-83.0483	https://data.globe.gov/system/photos/2019/07/15/1136628/original.jpg	https://data.globe.gov/system/photos/2019/07/15/1136630/original.jpg	https://data.globe.gov/system/photos/2019/07/15/1136629/original.jpg	https://data.globe.gov/system/photos/2019/07/15/1136631/original.jpg	https://data.globe.gov/system/photos/2019/07/15/1136633/original.jpg
GV_1226	40.8243	-74.2045	87.1		https://www.google.com/maps/search/40.8243,-74.2045	https://data.globe.gov/system/photos/2019/07/08/1136637/original.jpg	https://data.globe.gov/system/photos/2019/07/08/1136639/original.jpg	https://data.globe.gov/system/photos/2019/07/08/1136638/original.jpg	https://data.globe.gov/system/photos/2019/07/08/1136640/original.jpg	https://data.globe.gov/system/photos/2019/07/08/1136642/original.jpg
GV_1227	40.8185	-74.1931	45.9		https://www.google.com/maps/search/40.8185,-74.1931	https://data.globe.gov/system/photos/2019/07/08/1136643/original.jpg	https://data.globe.gov/system/photos/2019/07/08/1136645/original.jpg	https://data.globe.gov/system/photos/2019/07/08/1136644/original.jpg	https://data.globe.gov/system/photos/2019/07/08/1136646/original.jpg	https://data.globe.gov/system/photos/2019/07/08/1136648/original.jpg
GV_1228	40.8242	-74.2046	87		https://www.google.com/maps/search/40.8242,-74.2046	https://data.globe.gov/system/photos/2019/07/15/1136649/original.jpg	https://data.globe.gov/system/photos/2019/07/15/1136651/original.jpg	https://data.globe.gov/system/photos/2019/07/15/1136650/original.jpg	https://data.globe.gov/system/photos/2019/07/15/1136652/original.jpg	https://data.globe.gov/system/photos/2019/07/15/1136654/original.jpg
GV_1229	40.824	-74.2045	87.7		https://www.google.com/maps/search/40.824,-74.2045	https://data.globe.gov/system/photos/2019/07/15/1136724/original.jpg	https://data.globe.gov/system/photos/2019/07/15/1136726/original.jpg	https://data.globe.gov/system/photos/2019/07/15/1136725/original.jpg	https://data.globe.gov/system/photos/2019/07/15/1136727/original.jpg	https://data.globe.gov/system/photos/2019/07/15/1136729/original.jpg
GV_1230	40.8241	-74.204	87.7		https://www.google.com/maps/search/40.8241,-74.204	https://data.globe.gov/system/photos/2019/07/15/1136732/original.jpg	https://data.globe.gov/system/photos/2019/07/15/1136734/original.jpg	https://data.globe.gov/system/photos/2019/07/15/1136733/original.jpg	https://data.globe.gov/system/photos/2019/07/15/1136735/original.jpg	https://data.globe.gov/system/photos/2019/07/15/1136737/original.jpg
GV_1231	53.4139	-0.6051	37.9		https://www.google.com/maps/search/53.4139,-0.6051	https://data.globe.gov/system/photos/2019/07/15/1136782/original.jpg	https://data.globe.gov/system/photos/2019/07/15/1136784/original.jpg	https://data.globe.gov/system/photos/2019/07/15/1136783/original.jpg	https://data.globe.gov/system/photos/2019/07/15/1136786/original.jpg	https://data.globe.gov/system/photos/2019/07/15/1136789/original.jpg
GV_1232	37.8176	-85.447	205.3		https://www.google.com/maps/search/37.8176,-85.447	https://data.globe.gov/system/photos/2019/07/15/1136836/original.jpg	https://data.globe.gov/system/photos/2019/07/15/1136838/original.jpg	https://data.globe.gov/system/photos/2019/07/15/1136837/original.jpg	https://data.globe.gov/system/photos/2019/07/15/1136839/original.jpg	https://data.globe.gov/system/photos/2019/07/15/1136841/original.jpg
GV_1233	36.9906	-121.5648	60.1		https://www.google.com/maps/search/36.9906,-121.5648	https://data.globe.gov/system/photos/2019/07/16/1136895/original.jpg	https://data.globe.gov/system/photos/2019/07/16/1136897/original.jpg	https://data.globe.gov/system/photos/2019/07/16/1136896/original.jpg	https://data.globe.gov/system/photos/2019/07/16/1136898/original.jpg	https://data.globe.gov/system/photos/2019/07/16/1136900/original.jpg
GV_1234	52.5648	-0.2867	12.5		https://www.google.com/maps/search/52.5648,-0.2867	https://data.globe.gov/system/photos/2019/07/16/1137040/original.jpg	https://data.globe.gov/system/photos/2019/07/16/1137042/original.jpg	https://data.globe.gov/system/photos/2019/07/16/1137041/original.jpg	https://data.globe.gov/system/photos/2019/07/16/1137043/original.jpg	https://data.globe.gov/system/photos/2019/07/16/1137045/original.jpg
GV_1235	40.4459	-79.9935	225.2		https://www.google.com/maps/search/40.4459,-79.9935	https://data.globe.gov/system/photos/2019/07/15/1137068/original.jpg	https://data.globe.gov/system/photos/2019/07/15/1137070/original.jpg	https://data.globe.gov/system/photos/2019/07/15/1137069/original.jpg	https://data.globe.gov/system/photos/2019/07/15/1137071/original.jpg	https://data.globe.gov/system/photos/2019/07/15/1137072/original.jpg
GV_1236	40.4444	-79.9955	224.6		https://www.google.com/maps/search/40.4444,-79.9955	https://data.globe.gov/system/photos/2019/07/15/1137073/original.jpg	https://data.globe.gov/system/photos/2019/07/15/1137075/original.jpg	https://data.globe.gov/system/photos/2019/07/15/1137074/original.jpg	https://data.globe.gov/system/photos/2019/07/15/1137076/original.jpg	https://data.globe.gov/system/photos/2019/07/15/1137078/original.jpg
GV_1237	40.4406	-80.0066	222.7		https://www.google.com/maps/search/40.4406,-80.0066	https://data.globe.gov/system/photos/2019/07/15/1137079/original.jpg	https://data.globe.gov/system/photos/2019/07/15/1137081/original.jpg	https://data.globe.gov/system/photos/2019/07/15/1137080/original.jpg	https://data.globe.gov/system/photos/2019/07/15/1137082/original.jpg	https://data.globe.gov/system/photos/2019/07/15/1137084/original.jpg
GV_1238	40.4411	-80.0069	222.2		https://www.google.com/maps/search/40.4411,-80.0069	https://data.globe.gov/system/photos/2019/07/15/1137085/original.jpg	https://data.globe.gov/system/photos/2019/07/15/1137087/original.jpg	https://data.globe.gov/system/photos/2019/07/15/1137086/original.jpg	https://data.globe.gov/system/photos/2019/07/15/1137088/original.jpg	https://data.globe.gov/system/photos/2019/07/15/1137090/original.jpg
GV_1239	40.4408	-80.0105	220.3		https://www.google.com/maps/search/40.4408,-80.0105	https://data.globe.gov/system/photos/2019/07/15/1137097/original.jpg	https://data.globe.gov/system/photos/2019/07/15/1137099/original.jpg	https://data.globe.gov/system/photos/2019/07/15/1137098/original.jpg	https://data.globe.gov/system/photos/2019/07/15/1137100/original.jpg	https://data.globe.gov/system/photos/2019/07/15/1137102/original.jpg
GV_1240	40.442	-80.0132	217.2		https://www.google.com/maps/search/40.442,-80.0132	https://data.globe.gov/system/photos/2019/07/15/1137103/original.jpg	https://data.globe.gov/system/photos/2019/07/15/1137105/original.jpg	https://data.globe.gov/system/photos/2019/07/15/1137104/original.jpg	https://data.globe.gov/system/photos/2019/07/15/1137106/original.jpg	https://data.globe.gov/system/photos/2019/07/15/1137108/original.jpg
GV_1241	42.3389	-82.979	176.3		https://www.google.com/maps/search/42.3389,-82.979	https://data.globe.gov/system/photos/2019/07/16/1137148/original.jpg	https://data.globe.gov/system/photos/2019/07/16/1137150/original.jpg	https://data.globe.gov/system/photos/2019/07/16/1137149/original.jpg	https://data.globe.gov/system/photos/2019/07/16/1137151/original.jpg	https://data.globe.gov/system/photos/2019/07/16/1137153/original.jpg
GV_1242	42.3388	-82.9794	176		https://www.google.com/maps/search/42.3388,-82.9794	https://data.globe.gov/system/photos/2019/07/16/1137154/original.jpg	https://data.globe.gov/system/photos/2019/07/16/1137156/original.jpg	https://data.globe.gov/system/photos/2019/07/16/1137155/original.jpg	https://data.globe.gov/system/photos/2019/07/16/1137157/original.jpg	https://data.globe.gov/system/photos/2019/07/16/1137159/original.jpg
GV_1243	42.3387	-82.9794	176.3		https://www.google.com/maps/search/42.3387,-82.9794	https://data.globe.gov/system/photos/2019/07/16/1137163/original.jpg	https://data.globe.gov/system/photos/2019/07/16/1137165/original.jpg	https://data.globe.gov/system/photos/2019/07/16/1137164/original.jpg	https://data.globe.gov/system/photos/2019/07/16/1137167/original.jpg	https://data.globe.gov/system/photos/2019/07/16/1137171/original.jpg
GV_1244	42.3384	-82.979	176.1		https://www.google.com/maps/search/42.3384,-82.979	https://data.globe.gov/system/photos/2019/07/16/1137202/original.jpg	https://data.globe.gov/system/photos/2019/07/16/1137206/original.jpg	https://data.globe.gov/system/photos/2019/07/16/1137205/original.jpg	https://data.globe.gov/system/photos/2019/07/16/1137207/original.jpg	https://data.globe.gov/system/photos/2019/07/16/1137209/original.jpg
GV_1245	42.3384	-82.979	176.1		https://www.google.com/maps/search/42.3384,-82.979	https://data.globe.gov/system/photos/2019/07/16/1137198/original.jpg	https://data.globe.gov/system/photos/2019/07/16/1137200/original.jpg	https://data.globe.gov/system/photos/2019/07/16/1137199/original.jpg	https://data.globe.gov/system/photos/2019/07/16/1137201/original.jpg	https://data.globe.gov/system/photos/2019/07/16/1137204/original.jpg
GV_1246	42.3385	-82.9791	176.1		https://www.google.com/maps/search/42.3385,-82.9791	https://data.globe.gov/system/photos/2019/07/16/1137210/original.jpg	https://data.globe.gov/system/photos/2019/07/16/1137214/original.jpg	https://data.globe.gov/system/photos/2019/07/16/1137212/original.jpg	https://data.globe.gov/system/photos/2019/07/16/1137217/original.jpg	https://data.globe.gov/system/photos/2019/07/16/1137221/original.jpg
GV_1247	42.3382	-82.9793	176.2		https://www.google.com/maps/search/42.3382,-82.9793	https://data.globe.gov/system/photos/2019/07/16/1137306/original.jpg	https://data.globe.gov/system/photos/2019/07/16/1137308/original.jpg	https://data.globe.gov/system/photos/2019/07/16/1137307/original.jpg	https://data.globe.gov/system/photos/2019/07/16/1137309/original.jpg	https://data.globe.gov/system/photos/2019/07/16/1137311/original.jpg
GV_1248	42.3381	-82.9787	176.1		https://www.google.com/maps/search/42.3381,-82.9787	https://data.globe.gov/system/photos/2019/07/16/1137332/original.jpg	https://data.globe.gov/system/photos/2019/07/16/1137334/original.jpg	https://data.globe.gov/system/photos/2019/07/16/1137333/original.jpg	https://data.globe.gov/system/photos/2019/07/16/1137335/original.jpg	https://data.globe.gov/system/photos/2019/07/16/1137337/original.jpg
GV_1249	42.3382	-82.9793	176.2		https://www.google.com/maps/search/42.3382,-82.9793	https://data.globe.gov/system/photos/2019/07/16/1137338/original.jpg	https://data.globe.gov/system/photos/2019/07/16/1137340/original.jpg	https://data.globe.gov/system/photos/2019/07/16/1137339/original.jpg	https://data.globe.gov/system/photos/2019/07/16/1137341/original.jpg	https://data.globe.gov/system/photos/2019/07/16/1137343/original.jpg
GV_1250	42.3384	-82.9791	176.2		https://www.google.com/maps/search/42.3384,-82.9791	https://data.globe.gov/system/photos/2019/07/16/1137437/original.jpg	https://data.globe.gov/system/photos/2019/07/16/1137439/original.jpg	https://data.globe.gov/system/photos/2019/07/16/1137438/original.jpg	https://data.globe.gov/system/photos/2019/07/16/1137440/original.jpg	https://data.globe.gov/system/photos/2019/07/16/1137442/original.jpg
GV_1251	43.0848	-79.0483	174.6		https://www.google.com/maps/search/43.0848,-79.0483	https://data.globe.gov/system/photos/2019/07/16/1137506/original.jpg	https://data.globe.gov/system/photos/2019/07/16/1137508/original.jpg	https://data.globe.gov/system/photos/2019/07/16/1137507/original.jpg	https://data.globe.gov/system/photos/2019/07/16/1137509/original.jpg	https://data.globe.gov/system/photos/2019/07/16/1137511/original.jpg
GV_1252	52.1314	-1.6021	114.8		https://www.google.com/maps/search/52.1314,-1.6021	https://data.globe.gov/system/photos/2019/07/16/1137547/original.jpg	https://data.globe.gov/system/photos/2019/07/16/1137549/original.jpg	https://data.globe.gov/system/photos/2019/07/16/1137548/original.jpg	https://data.globe.gov/system/photos/2019/07/16/1137550/original.jpg	https://data.globe.gov/system/photos/2019/07/16/1137552/original.jpg
GV_1253	52.201	-1.7119	46.5		https://www.google.com/maps/search/52.201,-1.7119	https://data.globe.gov/system/photos/2019/07/16/1137604/original.jpg	https://data.globe.gov/system/photos/2019/07/16/1137606/original.jpg	https://data.globe.gov/system/photos/2019/07/16/1137605/original.jpg	https://data.globe.gov/system/photos/2019/07/16/1137609/original.jpg	https://data.globe.gov/system/photos/2019/07/16/1137613/original.jpg

GV_1254	42.3387	-82.9793	176.2		https://www.google.com/maps/search/42.3387,-82.9793	https://data.globe.gov/system/photos/2019/07/16/1137607/original.jpg	https://data.globe.gov/system/photos/2019/07/16/1137611/original.jpg	https://data.globe.gov/system/photos/2019/07/16/1137608/original.jpg	https://data.globe.gov/system/photos/2019/07/16/1137612/original.jpg	https://data.globe.gov/system/photos/2019/07/16/1137615/original.jpg
GV_1255	42.3381	-82.9788	176.2		https://www.google.com/maps/search/42.3381,-82.9788	https://data.globe.gov/system/photos/2019/07/16/1137617/original.jpg	https://data.globe.gov/system/photos/2019/07/16/1137622/original.jpg	https://data.globe.gov/system/photos/2019/07/16/1137619/original.jpg	https://data.globe.gov/system/photos/2019/07/16/1137624/original.jpg	https://data.globe.gov/system/photos/2019/07/16/1137630/original.jpg
GV_1256	42.3382	-82.9791	176.2		https://www.google.com/maps/search/42.3382,-82.9791	https://data.globe.gov/system/photos/2019/07/16/1137633/original.jpg	https://data.globe.gov/system/photos/2019/07/16/1137638/original.jpg	https://data.globe.gov/system/photos/2019/07/16/1137635/original.jpg	https://data.globe.gov/system/photos/2019/07/16/1137641/original.jpg	https://data.globe.gov/system/photos/2019/07/16/1137645/original.jpg
GV_1257	52.8091	-0.6308	104.3		https://www.google.com/maps/search/52.8091,-0.6308	https://data.globe.gov/system/photos/2019/07/16/1137616/original.jpg	https://data.globe.gov/system/photos/2019/07/16/1137620/original.jpg	https://data.globe.gov/system/photos/2019/07/16/1137618/original.jpg	https://data.globe.gov/system/photos/2019/07/16/1137621/original.jpg	https://data.globe.gov/system/photos/2019/07/16/1137625/original.jpg
GV_1258	52.8089	-0.6303	103.4		https://www.google.com/maps/search/52.8089,-0.6303	https://data.globe.gov/system/photos/2019/07/16/1137627/original.jpg	https://data.globe.gov/system/photos/2019/07/16/1137629/original.jpg	https://data.globe.gov/system/photos/2019/07/16/1137628/original.jpg	https://data.globe.gov/system/photos/2019/07/16/1137631/original.jpg	https://data.globe.gov/system/photos/2019/07/16/1137634/original.jpg
GV_1259	52.8084	-0.6274	99.8		https://www.google.com/maps/search/52.8084,-0.6274	https://data.globe.gov/system/photos/2019/07/16/1137636/original.jpg	https://data.globe.gov/system/photos/2019/07/16/1137639/original.jpg	https://data.globe.gov/system/photos/2019/07/16/1137637/original.jpg	https://data.globe.gov/system/photos/2019/07/16/1137640/original.jpg	https://data.globe.gov/system/photos/2019/07/16/1137644/original.jpg
GV_1260	38.1583	-90.3673	174.3		https://www.google.com/maps/search/38.1583,-90.3673	https://data.globe.gov/system/photos/2019/07/16/1137718/original.jpg	https://data.globe.gov/system/photos/2019/07/16/1137730/original.jpg	https://data.globe.gov/system/photos/2019/07/16/1137729/original.jpg	https://data.globe.gov/system/photos/2019/07/16/1137731/original.jpg	https://data.globe.gov/system/photos/2019/07/16/1137733/original.jpg
GV_1261	44.1056	-70.2051	75.9		https://www.google.com/maps/search/44.1056,-70.2051	https://data.globe.gov/system/photos/2019/07/14/1137830/original.jpg	https://data.globe.gov/system/photos/2019/07/14/1137834/original.jpg	https://data.globe.gov/system/photos/2019/07/14/1137831/original.jpg	https://data.globe.gov/system/photos/2019/07/14/1137836/original.jpg	https://data.globe.gov/system/photos/2019/07/14/1137839/original.jpg
GV_1262	52.1311	-1.6019	114.7		https://www.google.com/maps/search/52.1311,-1.6019	https://data.globe.gov/system/photos/2019/07/16/1137860/original.jpg	https://data.globe.gov/system/photos/2019/07/16/1137862/original.jpg	https://data.globe.gov/system/photos/2019/07/16/1137861/original.jpg	https://data.globe.gov/system/photos/2019/07/16/1137863/original.jpg	https://data.globe.gov/system/photos/2019/07/16/1137865/original.jpg
GV_1263	42.3276	-83.0481	182.4		https://www.google.com/maps/search/42.3276,-83.0481	https://data.globe.gov/system/photos/2019/07/16/1137878/original.jpg	https://data.globe.gov/system/photos/2019/07/16/1137880/original.jpg	https://data.globe.gov/system/photos/2019/07/16/1137879/original.jpg	https://data.globe.gov/system/photos/2019/07/16/1137881/original.jpg	https://data.globe.gov/system/photos/2019/07/16/1137883/original.jpg
GV_1264	42.3272	-83.0479	182.9		https://www.google.com/maps/search/42.3272,-83.0479	https://data.globe.gov/system/photos/2019/07/16/1137884/original.jpg	https://data.globe.gov/system/photos/2019/07/16/1137886/original.jpg	https://data.globe.gov/system/photos/2019/07/16/1137885/original.jpg	https://data.globe.gov/system/photos/2019/07/16/1137887/original.jpg	https://data.globe.gov/system/photos/2019/07/16/1137889/original.jpg
GV_1265	42.3268	-83.0478	183.4		https://www.google.com/maps/search/42.3268,-83.0478	https://data.globe.gov/system/photos/2019/07/16/1137890/original.jpg	https://data.globe.gov/system/photos/2019/07/16/1137892/original.jpg	https://data.globe.gov/system/photos/2019/07/16/1137891/original.jpg	https://data.globe.gov/system/photos/2019/07/16/1137893/original.jpg	https://data.globe.gov/system/photos/2019/07/16/1137895/original.jpg
GV_1266	42.3271	-83.0468	179.9		https://www.google.com/maps/search/42.3271,-83.0468	https://data.globe.gov/system/photos/2019/07/16/1137896/original.jpg	https://data.globe.gov/system/photos/2019/07/16/1137898/original.jpg	https://data.globe.gov/system/photos/2019/07/16/1137897/original.jpg	https://data.globe.gov/system/photos/2019/07/16/1137899/original.jpg	https://data.globe.gov/system/photos/2019/07/16/1137901/original.jpg
GV_1267	42.3264	-83.0464	176.5		https://www.google.com/maps/search/42.3264,-83.0464	https://data.globe.gov/system/photos/2019/07/16/1137902/original.jpg	https://data.globe.gov/system/photos/2019/07/16/1137904/original.jpg	https://data.globe.gov/system/photos/2019/07/16/1137903/original.jpg	https://data.globe.gov/system/photos/2019/07/16/1137905/original.jpg	https://data.globe.gov/system/photos/2019/07/16/1137907/original.jpg
GV_1268	42.3271	-83.0473	180.1		https://www.google.com/maps/search/42.3271,-83.0473	https://data.globe.gov/system/photos/2019/07/16/1137908/original.jpg	https://data.globe.gov/system/photos/2019/07/16/1137910/original.jpg	https://data.globe.gov/system/photos/2019/07/16/1137909/original.jpg	https://data.globe.gov/system/photos/2019/07/16/1137911/original.jpg	https://data.globe.gov/system/photos/2019/07/16/1137913/original.jpg
GV_1269	42.3288	-83.0476	180.5		https://www.google.com/maps/search/42.3288,-83.0476	https://data.globe.gov/system/photos/2019/07/16/1137914/original.jpg	https://data.globe.gov/system/photos/2019/07/16/1137916/original.jpg	https://data.globe.gov/system/photos/2019/07/16/1137915/original.jpg	https://data.globe.gov/system/photos/2019/07/16/1137917/original.jpg	https://data.globe.gov/system/photos/2019/07/16/1137919/original.jpg
GV_1270	42.3287	-83.0492	181.3		https://www.google.com/maps/search/42.3287,-83.0492	https://data.globe.gov/system/photos/2019/07/16/1137920/original.jpg	https://data.globe.gov/system/photos/2019/07/16/1137922/original.jpg	https://data.globe.gov/system/photos/2019/07/16/1137921/original.jpg	https://data.globe.gov/system/photos/2019/07/16/1137923/original.jpg	https://data.globe.gov/system/photos/2019/07/16/1137925/original.jpg
GV_1271	42.3283	-83.0511	182.3		https://www.google.com/maps/search/42.3283,-83.0511	https://data.globe.gov/system/photos/2019/07/16/1137926/original.jpg	https://data.globe.gov/system/photos/2019/07/16/1137928/original.jpg	https://data.globe.gov/system/photos/2019/07/16/1137927/original.jpg	https://data.globe.gov/system/photos/2019/07/16/1137929/original.jpg	https://data.globe.gov/system/photos/2019/07/16/1137931/original.jpg
GV_1272	42.3275	-83.0519	179.4		https://www.google.com/maps/search/42.3275,-83.0519	https://data.globe.gov/system/photos/2019/07/16/1137932/original.jpg	https://data.globe.gov/system/photos/2019/07/16/1137934/original.jpg	https://data.globe.gov/system/photos/2019/07/16/1137933/original.jpg	https://data.globe.gov/system/photos/2019/07/16/1137935/original.jpg	https://data.globe.gov/system/photos/2019/07/16/1137937/original.jpg
GV_1273	40.0672	-80.7254	193.2		https://www.google.com/maps/search/40.0672,-80.7254	https://data.globe.gov/system/photos/2019/07/16/1137981/original.jpg	https://data.globe.gov/system/photos/2019/07/16/1137983/original.jpg	https://data.globe.gov/system/photos/2019/07/16/1137982/original.jpg	https://data.globe.gov/system/photos/2019/07/16/1137984/original.jpg	https://data.globe.gov/system/photos/2019/07/16/1137986/original.jpg
GV_1274	40.0739	-80.7251	211.2		https://www.google.com/maps/search/40.0739,-80.7251	https://data.globe.gov/system/photos/2019/07/16/1137987/original.jpg	https://data.globe.gov/system/photos/2019/07/16/1137989/original.jpg	https://data.globe.gov/system/photos/2019/07/16/1137988/original.jpg	https://data.globe.gov/system/photos/2019/07/16/1137990/original.jpg	https://data.globe.gov/system/photos/2019/07/16/1137992/original.jpg
GV_1275	40.0732	-80.7248	212.1		https://www.google.com/maps/search/40.0732,-80.7248	https://data.globe.gov/system/photos/2019/07/16/1137993/original.jpg	https://data.globe.gov/system/photos/2019/07/16/1137995/original.jpg	https://data.globe.gov/system/photos/2019/07/16/1137994/original.jpg	https://data.globe.gov/system/photos/2019/07/16/1137996/original.jpg	https://data.globe.gov/system/photos/2019/07/16/1137998/original.jpg
GV_1276	40.2691	-80.6139	200.6		https://www.google.com/maps/search/40.2691,-80.6139	https://data.globe.gov/system/photos/2019/07/16/1137999/original.jpg	https://data.globe.gov/system/photos/2019/07/16/1138001/original.jpg	https://data.globe.gov/system/photos/2019/07/16/1138000/original.jpg	https://data.globe.gov/system/photos/2019/07/16/1138002/original.jpg	https://data.globe.gov/system/photos/2019/07/16/1138004/original.jpg
GV_1277	40.3589	-80.614	216.6		https://www.google.com/maps/search/40.3589,-80.614	https://data.globe.gov/system/photos/2019/07/16/1138005/original.jpg	https://data.globe.gov/system/photos/2019/07/16/1138007/original.jpg	https://data.globe.gov/system/photos/2019/07/16/1138006/original.jpg	https://data.globe.gov/system/photos/2019/07/16/1138008/original.jpg	https://data.globe.gov/system/photos/2019/07/16/1138010/original.jpg
GV_1278	40.3587	-80.6133	214.7		https://www.google.com/maps/search/40.3587,-80.6133	https://data.globe.gov/system/photos/2019/07/16/1138011/original.jpg	https://data.globe.gov/system/photos/2019/07/16/1138013/original.jpg	https://data.globe.gov/system/photos/2019/07/16/1138012/original.jpg	https://data.globe.gov/system/photos/2019/07/16/1138014/original.jpg	https://data.globe.gov/system/photos/2019/07/16/1138016/original.jpg
GV_1279	39.917	-80.745	217.4		https://www.google.com/maps/search/39.917,-80.745	https://data.globe.gov/system/photos/2019/07/16/1138017/original.jpg	https://data.globe.gov/system/photos/2019/07/16/1138019/original.jpg	https://data.globe.gov/system/photos/2019/07/16/1138018/original.jpg	https://data.globe.gov/system/photos/2019/07/16/1138020/original.jpg	https://data.globe.gov/system/photos/2019/07/16/1138022/original.jpg
GV_1280	39.6295	-80.8749	184.3		https://www.google.com/maps/search/39.6295,-80.8749	https://data.globe.gov/system/photos/2019/07/16/1138023/original.jpg	https://data.globe.gov/system/photos/2019/07/16/1138025/original.jpg	https://data.globe.gov/system/photos/2019/07/16/1138024/original.jpg	https://data.globe.gov/system/photos/2019/07/16/1138026/original.jpg	https://data.globe.gov/system/photos/2019/07/16/1138028/original.jpg
GV_1281	39.5612	-81.0122	190.9		https://www.google.com/maps/search/39.5612,-81.0122	https://data.globe.gov/system/photos/2019/07/16/1138029/original.jpg	https://data.globe.gov/system/photos/2019/07/16/1138031/original.jpg	https://data.globe.gov/system/photos/2019/07/16/1138030/original.jpg	https://data.globe.gov/system/photos/2019/07/16/1138032/original.jpg	https://data.globe.gov/system/photos/2019/07/16/1138034/original.jpg
GV_1282	39.4442	-81.1507	186.2		https://www.google.com/maps/search/39.4442,-81.1507	https://data.globe.gov/system/photos/2019/07/16/1138035/original.jpg	https://data.globe.gov/system/photos/2019/07/16/1138037/original.jpg	https://data.globe.gov/system/photos/2019/07/16/1138036/original.jpg	https://data.globe.gov/system/photos/2019/07/16/1138038/original.jpg	https://data.globe.gov/system/photos/2019/07/16/1138040/original.jpg
GV_1283	39.4151	-81.4564	186.2		https://www.google.com/maps/search/39.4151,-81.4564	https://data.globe.gov/system/photos/2019/07/16/1138041/original.jpg	https://data.globe.gov/system/photos/2019/07/16/1138043/original.jpg	https://data.globe.gov/system/photos/2019/07/16/1138042/original.jpg	https://data.globe.gov/system/photos/2019/07/16/1138044/original.jpg	https://data.globe.gov/system/photos/2019/07/16/1138046/original.jpg
GV_1284	39.4155	-81.458	183.7		https://www.google.com/maps/search/39.4155,-81.458	https://data.globe.gov/system/photos/2019/07/16/1138047/original.jpg	https://data.globe.gov/system/photos/2019/07/16/1138049/original.jpg	https://data.globe.gov/system/photos/2019/07/16/1138048/original.jpg	https://data.globe.gov/system/photos/2019/07/16/1138050/original.jpg	https://data.globe.gov/system/photos/2019/07/16/1138052/original.jpg
GV_1285	39.4232	-81.4629	191.9		https://www.google.com/maps/search/39.4232,-81.4629	https://data.globe.gov/system/photos/2019/07/16/1138053/original.jpg	https://data.globe.gov/system/photos/2019/07/16/1138055/original.jpg	https://data.globe.gov/system/photos/2019/07/16/1138054/original.jpg	https://data.globe.gov/system/photos/2019/07/16/1138056/original.jpg	https://data.globe.gov/system/photos/2019/07/16/1138058/original.jpg
GV_1286	42.3286	-83.0477	180.8		https://www.google.com/maps/search/42.3286,-83.0477	https://data.globe.gov/system/photos/2019/07/16/1138108/original.jpg	https://data.globe.gov/system/photos/2019/07/16/1138110/original.jpg	https://data.globe.gov/system/photos/2019/07/16/1138109/original.jpg	https://data.globe.gov/system/photos/2019/07/16/1138111/original.jpg	https://data.globe.gov/system/photos/2019/07/16/1138113/original.jpg
GV_1287	42.3334	-83.0485	182.9		https://www.google.com/maps/search/42.3334,-83.0485	https://data.globe.gov/system/photos/2019/07/16/1138114/original.jpg	https://data.globe.gov/system/photos/2019/07/16/1138116/original.jpg	https://data.globe.gov/system/photos/2019/07/16/1138115/original.jpg	https://data.globe.gov/system/photos/2019/07/16/1138117/original.jpg	https://data.globe.gov/system/photos/2019/07/16/1138119/original.jpg
GV_1288	44.5495	-123.3	75.5		https://www.google.com/maps/search/44.5495,-123.3	https://data.globe.gov/system/photos/2019/07/16/1138120/original.jpg	https://data.globe.gov/system/photos/2019/07/16/1138122/original.jpg	https://data.globe.gov/system/photos/2019/07/16/1138121/original.jpg	https://data.globe.gov/system/photos/2019/07/16/1138123/original.jpg	https://data.globe.gov/system/photos/2019/07/16/1138125/original.jpg
GV_1289	44.5999	-123.2429	66.4		https://www.google.com/maps/search/44.5999,-123.2429	https://data.globe.gov/system/photos/2019/07/16/1138132/original.jpg	https://data.globe.gov/system/photos/2019/07/16/1138138/original.jpg	https://data.globe.gov/system/photos/2019/07/16/1138136/original.jpg	https://data.globe.gov/system/photos/2019/07/16/1138140/original.jpg	https://data.globe.gov/system/photos/2019/07/16/1138142/original.jpg
GV_1290	44.2962	-124.0349	29.2		https://www.google.com/maps/search/44.2962,-124.0349	https://data.globe.gov/system/photos/2019/07/16/1138149/original.jpg	https://data.globe.gov/system/photos/2019/07/16/1138151/original.jpg	https://data.globe.gov/system/photos/2019/07/16/1138150/original.jpg	https://data.globe.gov/system/photos/2019/07/16/1138152/original.jpg	https://data.globe.gov/system/photos/2019/07/16/1138154/original.jpg
GV_1291	53.4291	-0.6104	35.3		https://www.google.com/maps/search/53.4291,-0.6104	https://data.globe.gov/system/photos/2019/07/17/1138424/original.jpg	https://data.globe.gov/system/photos/2019/07/17/1138426/original.jpg	https://data.globe.gov/system/photos/2019/07/17/1138425/original.jpg	https://data.globe.gov/system/photos/2019/07/17/1138427/original.jpg	https://data.globe.gov/system/photos/2019/07/17/1138429/original.jpg
GV_1292	5.9941	116.092	6.1		https://www.google.com/maps/search/5.9941,116.092	https://data.globe.gov/system/photos/2019/07/17/1138462/original.jpg	https://data.globe.gov/system/photos/2019/07/17/1138464/original.jpg	https://data.globe.gov/system/photos/2019/07/17/1138463/original.jpg	https://data.globe.gov/system/photos/2019/07/17/1138465/original.jpg	https://data.globe.gov/system/photos/2019/07/17/1138467/original.jpg
GV_1293	5.9918	116.0988	2		https://www.google.com/maps/search/5.9918,116.0988	https://data.globe.gov/system/photos/2019/07/17/1138468/original.jpg	https://data.globe.gov/system/photos/2019/07/17/1138470/original.jpg	https://data.globe.gov/system/photos/2019/07/17/1138469/original.jpg	https://data.globe.gov/system/photos/2019/07/17/1138471/original.jpg	https://data.globe.gov/system/photos/2019/07/17/1138473/original.jpg
GV_1294	5.9626	116.0624	3.5		https://www.google.com/maps/search/5.9626,116.0624	https://data.globe.gov/system/photos/2019/07/17/1138474/original.jpg	https://data.globe.gov/system/photos/2019/07/17/1138476/original.jpg	https://data.globe.gov/system/photos/2019/07/17/1138475/original.jpg	https://data.globe.gov/system/photos/2019/07/17/1138477/original.jpg	https://data.globe.gov/system/photos/2019/07/17/1138479/original.jpg
GV_1295	46.1798	-123.8457	46.5		https://www.google.com/maps/search/46.1798,-123.8457	https://data.globe.gov/system/photos/2019/07/16/1138522/original.jpg	https://data.globe.gov/system/photos/2019/07/16/1138524/original.jpg	https://data.globe.gov/system/photos/2019/07/16/1138523/original.jpg	https://data.globe.gov/system/photos/2019/07/16/1138525/original.jpg	https://data.globe.gov/system/photos/2019/07/16/1138527/original.jpg
GV_1296	42.3384	-82.9791	176.2		https://www.google.com/maps/search/42.3384,-82.9791	https://data.globe.gov/system/photos/2019/07/16/1138538/original.jpg	https://data.globe.gov/system/photos/2019/07/16/1138540/original.jpg	https://data.globe.gov/system/photos/2019/07/16/1138539/original.jpg	https://data.globe.gov/system/photos/2019/07/16/1138541/original.jpg	https://data.globe.gov/system/photos/2019/07/16/1138543/original.jpg
GV_1297	42.3277	-83.0475	183.2		https://www.google.com/maps/search/42.3277,-83.0475	https://data.globe.gov/system/photos/2019/07/17/1138585/original.jpg	https://data.globe.gov/system/photos/2019/07/17/1138587/original.jpg	https://data.globe.gov/system/photos/2019/07/17/1138586/original.jpg	https://data.globe.gov/system/photos/2019/07/17/1138588/original.jpg	https://data.globe.gov/system/photos/2019/07/17/1138590/original.jpg
GV_1298	42.3585	-83.0623	194		https://www.google.com/maps/search/42.3585,-83.0623	https://data.globe.gov/system/photos/2019/07/17/1138728/original.jpg	https://data.globe.gov/system/photos/2019/07/17/1138731/original.jpg	https://data.globe.gov/system/photos/2019/07/17/1138730/original.jpg	https://data.globe.gov/system/photos/2019/07/17/1138733/original.jpg	https://data.globe.gov/system/photos/2019/07/17/1138736/original.jpg
GV_1299	46.182	-123.8367	77.3		https://www.google.com/maps/search/46.182,-123.8367	https://data.globe.gov/system/photos/2019/07/17/1138746/original.jpg	https://data.globe.gov/system/photos/2019/07/17/1138748/original.jpg	https://data.globe.gov/system/photos/2019/07/17/1138747/original.jpg	https://data.globe.gov/system/photos/2019/07/17/1138749/original.jpg	https://data.globe.gov/system/photos/2019/07/17/1138751/original.jpg
GV_1300	38.901	-90.0598	161.1		https://www.google.com/maps/search/38.901,-90.0598	https://data.globe.gov/system/photos/2019/07/17/1138784/original.jpg	https://data.globe.gov/system/photos/2019/07/17/1138786/original.jpg	https://data.globe.gov/system/photos/2019/07/17/1138785/original.jpg	https://data.globe.gov/system/photos/2019/07/17/1138787/original.jpg	https://data.globe.gov/system/photos/2019/07/17/1138789/original.jpg
GV_1301	42.3368	-82.9793	176		https://www.google.com/maps/search/42.3368,-82.9793	https://data.globe.gov/system/photos/2019/07/17/1138796/original.jpg	https://data.globe.gov/system/photos/2019/07/17/1138798/original.jpg	https://data.globe.gov/system/photos/2019/07/17/1138797/original.jpg	https://data.globe.gov/system/photos/2019/07/17/1138799/original.jpg	https://data.globe.gov/system/photos/2019/07/17/1138801/original.jpg
GV_1302	38.8587	-90.0683	133		https://www.google.com/maps/search/38.8587,-90.0683	https://data.globe.gov/system/photos/2019/07/17/1138851/original.jpg	https://data.globe.gov/system/photos/2019/07/17/1138853/original.jpg	https://data.globe.gov/system/photos/2019/07/17/1138852/original.jpg	https://data.globe.gov/system/photos/2019/07/17/1138854/original.jpg	https://data.globe.gov/system/photos/2019/07/17/1138856/original.jpg
GV_1303	38.2702	-90.4101	127.5		https://www.google.com/maps/search/38.2702,-90.4101	https://data.globe.gov/system/photos/2019/07/17/1138877/original.jpg	https://data.globe.gov/system/photos/2019/07/17/1138879/original.jpg	https://data.globe.gov/system/photos/2019/07/17/1138878/original.jpg	https://data.globe.gov/system/photos/2019/07/17/1138880/original.jpg	https://data.globe.gov/system/photos/2019/07/17/1138882/original.jpg
GV_1304	53.4121	-0.6047	37		https://www.google.com/maps/search/53.4121,-0.6047	https://data.globe.gov/system/photos/2019/07/17/1138937/original.jpg	https://data.globe.gov/system/photos/2019/07/17/1138939/original.jpg	https://data.globe.gov/system/photos/2019/07/17/1138938/original.jpg	https://data.globe.gov/system/photos/2019/07/17/1138940/original.jpg	https://data.globe.gov/system/photos/2019/07/17/1138942/original.jpg
GV_1305	44.1157	-70.2123	57.4		https://www.google.com/maps/search/44.1157,-70.2123	https://data.globe.gov/system/photos/2019/07/17/1138949/original.jpg	https://data.globe.gov/system/photos/2019/07/17/1138951/original.jpg	https://data.globe.gov/system/photos/2019/07/17/1138950/original.jpg	https://data.globe.gov/system/photos/2019/07/17/1138952/original.jpg	https://data.globe.gov/system/photos/2019/07/17/1138954/original.jpg
GV_1306	44.1196	-70.2118	51.4		https://www.google.com/maps/search/44.1196,-70.2118	https://data.globe.gov/system/photos/2019/07/17/1138961/original.jpg	https://data.globe.gov/system/photos/2019/07/17/1138963/original.jpg	https://data.globe.gov/system/photos/2019/07/17/1138962/original.jpg	https://data.globe.gov/system/photos/2019/07/17/1138964/original.jpg	https://data.globe.gov/system/photos/2019/07/17/1138966/original.jpg
GV_1307	42.3584	-83.0625	192.3		https://www.google.com/maps/search/42.3584,-83.0625	https://data.globe.gov/system/photos/2019/07/17/1138976/original.jpg	https://data.globe.gov/system/photos/2019/07/17/1138978/original.jpg	https://data.globe.gov/system/photos/2019/07/17/1138977/original.jpg	https://data.globe.gov/system/photos/2019/07/17/1138979/original.jpg	https://data.globe.gov/system/photos/2019/07/17/1138981/original.jpg
GV_1308	36.9855	-121.5785	78.9		https://www.google.com/maps/search/36.9855,-121.5785	https://data.globe.gov/system/photos/2019/07/17/1138988/original.jpg	https://data.globe.gov/system/photos/2019/07/17/1138990/original.jpg	https://data.globe.gov/system/photos/2019/07/17/1138989/original.jpg	https://data.globe.gov/system/photos/2019/07/17/1138991/original.jpg	https://data.globe.gov/system/photos/2019/07/17/1138993/original.jpg
GV_1309	36.9904	-121.5929	109.7		https://www.google.com/maps/search/36.9904,-121.5929	https://data.globe.gov/system/photos/2019/07/17/1138994/original.jpg	https://data.globe.gov/system/photos/2019/07/17/1138996/original.jpg	https://data.globe.gov/system/photos/2019/07/17/1138995/original.jpg	https://data.globe.gov/system/photos/2019/07/17/1138997/original.jpg	https://data.globe.gov/system/photos/2019/07/17/1139005/original.jpg
GV_1310	36.9933	-121.5957	102.6		https://www.google.com/maps/search/36.9933,-121.5957	https://data.globe.gov/system/photos/2019/07/17/1139006/original.jpg	https://data.globe.gov/system/photos/2019/07/17/1139008/original.jpg	https://data.globe.gov/system/photos/2019/07/17/1139007/original.jpg	https://data.globe.gov/system/photos/2019/07/17/1139009/original.jpg	https://data.globe.gov/system/photos/2019/07/17/1139011/original.jpg
GV_1311	44.6244	-123.0825	69		https://www.google.com/maps/search/44.6244,-123.0825	https://data.globe.gov/system/photos/2019/07/17/1139021/original.jpg	https://data.globe.gov/system/photos/2019/07/17/1139029/original.jpg	https://data.globe.gov/system/photos/2019/07/17/1139024/original.jpg	https://data.globe.gov/system/photos/2019/07/17/1139030/original.jpg	https://data.globe.gov/system/photos/2019/07/17/1139032/original.jpg
GV_1312	51.386	-2.3674	48.7		https://www.google.com/maps/search/51.386,-2.3674	https://data.globe.gov/system/photos/2019/07/17/1139092/original.jpg	https://data.globe.gov/system/photos/2019/07/17/1139098/original.jpg	https://data.globe.gov/system/photos/2019/07/17/1139094/original.jpg	https://data.globe.gov/system/photos/2019/07/17/1139100/original.jpg	https://data.globe.gov/system/photos/2019/07/17/1139106/original.jpg
GV_1313	51.5123	-1.7735	181.2		https://www.google.com/maps/search/51.5123,-1.7735	https://data.globe.gov/system/photos/2019/07/17/1139116/original.jpg	https://data.globe.gov/system/photos/2019/07/17/1139118/original.jpg	https://data.globe.gov/system/photos/2019/07/17/1139117/original.jpg	https://data.globe.gov/system/photos/2019/07/17/1139120/original.jpg	https://data.globe.gov/system/photos/2019/07/17/1139122/original.jpg
GV_1314	31.8728	-106.5733	1202.4		https://www.google.com/maps/search/31.8728,-106.5733	https://data.globe.gov/system/photos/2019/07/17/1139093/original.jpg	https://data.globe.gov/system/photos/2019/07/17/1139099/original.jpg	https://data.globe.gov/system/photos/2019/07/17/1139097/original.jpg	https://data.globe.gov/system/photos/2019/07/17/1139101/original.jpg	https://data.globe.gov/system/photos/2019/07/17/1139105/original.jpg
GV_1315	31.879	-106.603	1148.4		https://www.google.com/maps/search/31.879,-106.603	https://data.globe.gov/system/photos/2019/07/17/1139238/original.jpg	https://data.globe.gov/system/photos/2019/07/17/1139240/original.jpg	https://data.globe.gov/system/photos/2019/07/17/1139239/original.jpg	https://data.globe.gov/system/photos/2019/07/17/1139245/original.jpg	https://data.globe.gov/system/photos/2019/07/17/1139254/original.jpg
GV_1316	42.3561	-83.0615	191.1		https://www.google.com/maps/search/42.3561,-83.0615	https://data.globe.gov/system/photos/2019/07/17/1139253/original.jpg	https://data.globe.gov/system/photos/2019/07/17/1139257/original.jpg	https://data.globe.gov/system/photos/2019/07/17/1139255/original.jpg	https://data.globe.gov/system/photos/2019/07/17/1139259/original.jpg	https://data.globe.gov/system/photos/2019/07/17/1139264/original.jpg
GV_1317	44.5681	-123.2788	79		https://www.google.com/maps/search/44.5681,-123.2788	https://data.globe.gov/system/photos/2019/07/17/1139290/original.jpg	https://data.globe.gov/system/photos/2019/07/17/1139293/original.jpg	https://data.globe.gov/system/photos/2019/07/17/1139292/original.jpg	https://data.globe.gov/system/photos/2019/07/17/1139294/original.jpg	https://data.globe.gov/system/photos/2019/07/17/1139296/original.jpg
GV_1318	45.4939	-122.7559	108.5		https://www.google.com/maps/search/45.4939,-122.7559	https://data.globe.gov/system/photos/2019/07/17/1139316/original.jpg	https://data.globe.gov/system/photos/2019/07/17/1139319/original.jpg	https://data.globe.gov/system/photos/2019/07/17/1139317/original.jpg	https://data.globe.gov/system/photos/2019/07/17/1139321/original.jpg	https://data.globe.gov/system/photos/2019/07/17/1139325/original.jpg
GV_1319	42.3278	-83.0482	182.4		https://www.google.com/maps/search/42.3278,-83.0482	https://data.globe.gov/system/photos/2019/07/17/1139318/original.jpg	https://data.globe.gov/system/photos/2019/07/17/1139322/original.jpg	https://data.globe.gov/system/photos/2019/07/17/1139320/original.jpg	https://data.globe.gov/system/photos/2019/07/17/1139324/original.jpg	https://data.globe.gov/system/photos/2019/07/17/1139327/original.jpg
GV_1320	42.329	-83.0443	183		https://www.google.com/maps/search/42.329,-83.0443	https://data.globe.gov/system/photos/2019/07/17/1139328/original.jpg	https://data.globe.gov/system/photos/2019/07/17/1139330/original.jpg	https://data.globe.gov/system/photos/2019/07/17/1139329/original.jpg	https://data.globe.gov/system/photos/2019/07/17/1139331/original.jpg	https://data.globe.gov/system/photos/2019/07/17/1139333/original.jpg
GV_1321	42.3287	-83.0448	183.2		https://www.google.com/maps/search/42.3287,-83.0448	https://data.globe.gov/system/photos/2019/07/17/1139334/original.jpg	https://data.globe.gov/system/photos/2019/07/17/1139336/original.jpg	https://data.globe.gov/system/photos/2019/07/17/1139335/original.jpg	https://data.globe.gov/system/photos/2019/07/17/1139337/original.jpg	https://data.globe.gov/system/photos/2019/07/17/1139339/original.jpg
GV_1322	42.3281	-83.0445	183.3		https://www.google.com/maps/search/42.3281,-83.0445	https://data.globe.gov/system/photos/2019/07/17/1139340/original.jpg	https://data.globe.gov/system/photos/2019/07/17/1139342/original.jpg	https://data.globe.gov/system/photos/2019/07/17/1139341/original.jpg	https://data.globe.gov/system/photos/2019/07/17/1139343/original.jpg	https://data.globe.gov/system/photos/2019/07/17/1139345/original.jpg
GV_1323	42.329	-83.0441	183.3		https://www.google.com/maps/search/42.329,-83.0441	https://data.globe.gov/system/photos/2019/07/17/1139346/original.jpg	https://data.globe.gov/system/photos/2019/07/17/1139351/original.jpg	https://data.globe.gov/system/photos/2019/07/17/1139348/original.jpg	https://data.globe.gov/system/photos/2019/07/17/1139354/original.jpg	https://data.globe.gov/system/photos/2019/07/17/1139357/original.jpg
GV_1324	42.3292	-83.0451	182.1		https://www.google.com/maps/search/42.3292,-83.0451	https://data.globe.gov/system/photos/2019/07/17/1139358/original.jpg	https://data.globe.gov/system/photos/2019/07/17/1139360/original.jpg	https://data.globe.gov/system/photos/2019/07/17/1139359/original.jpg	https://data.globe.gov/system/photos/2019/07/17/1139361/original.jpg	https://data.globe.gov/system/photos/2019/07/17/1139363/original.jpg
GV_1325	42.3298	-83.0458	181.6		https://www.google.com/maps/search/42.3298,-83.0458	https://data.globe.gov/system/photos/2019/07/17/1139364/original.jpg	https://data.globe.gov/system/photos/2019/07/17/1139366/original.jpg	https://data.globe.gov/system/photos/2019/07/17/1139365/original.jpg	https://data.globe.gov/system/photos/2019/07/17/1139367/original.jpg	https://data.globe.gov/system/photos/2019/07/17/1139369/original.jpg
GV_1326	44.5755	-123.2843	74.9		https://www.google.com/maps/search/44.5755,-123.2843	https://data.globe.gov/system/photos/2019/07/17/1139347/original.jpg	https://data.globe.gov/system/photos/2019/07/17/1139350/original.jpg	https://data.globe.gov/system/photos/2019/07/17/1139349/original.jpg	https://data.globe.gov/system/photos/2019/07/17/1139352/original.jpg	https://data.globe.gov/system/photos/2019/07/17/1139356/original.jpg
GV_1327	45.4829	-122.7589	91.8		https://www.google.com/maps/search/45.4829,-122.7589	https://data.globe.gov/system/photos/2019/07/17/1139370/original.jpg	https://data.globe.gov/system/photos/2019/07/17/1139372/original.jpg	https://data.globe.gov/system/photos/2019/07/17/1139371/original.jpg	https://data.globe.gov/system/photos/2019/07/17/1139373/original.jpg	https://data.globe.gov/system/photos/2019/07/17/1139375/original.jpg
GV_1328	45.4765	-122.7547	69.4		https://www.google.com/maps/search/45.4765,-122.7547	https://data.globe.gov/system/photos/2019/07/17/1139376/original.jpg	https://data.globe.gov/system/photos/2019/07/17/1139378/original.jpg	https://data.globe.gov/system/photos/2019/07/17/1139377/original.jpg	https://data.globe.gov/system/photos/2019/07/17/1139379/original.jpg	https://data.globe.gov/system/photos/2019/07/17/1139381/original.jpg
GV_1329	47.6805	-117.2252	607.2		https://www.google.com/maps/search/47.6805,-117.2252	https://data.globe.gov/system/photos/2019/07/17/1139382/original.jpg	https://data.globe.gov/system/photos/2019/07/17/1139384/original.jpg	https://data.globe.gov/system/photos/2019/07/17/1139383/original.jpg	https://data.globe.gov/system/photos/2019/07/17/1139385/original.jpg	https://data.globe.gov/system/photos/2019/07/17/1139387/original.jpg
GV_1330	47.6544	-117.2905	609.8		https://www.google.com/maps/search/47.6544,-117.2905	https://data.globe.gov/system/photos/2019/07/17/1139388/original.jpg	https://data.globe.gov/system/photos/2019/07/17/1139390/original.jpg	https://data.globe.gov/system/photos/2019/07/17/1139389/original.jpg	https://data.globe.gov/system/photos/2019/07/17/1139391/original.jpg	https://data.globe.gov/system/photos/2019/07/17/1139393/original.jpg
GV_1331	47.6356	-117.222	635.6		https://www.google.com/maps/search/47.6356,-117.222	https://data.globe.gov/system/photos/2019/07/17/1139394/original.jpg	https://data.globe.gov/system/photos/2019/07/17/1139396/original.jpg	https://data.globe.gov/system/photos/2019/07/17/1139395/original.jpg	https://data.globe.gov/system/photos/2019/07/17/1139397/original.jpg	https://data.globe.gov/system/photos/2019/07/17/1139399/original.jpg
GV_1332	44.5735	-123.2832	76.6		https://www.google.com/maps/search/44.5735,-123.2832	https://data.globe.gov/system/photos/2019/07/17/1139400/original.jpg	https://data.globe.gov/system/photos/2019/07/17/1139402/original.jpg	https://data.globe.gov/system/photos/2019/07/17/1139401/original.jpg	https://data.globe.gov/system/photos/2019/07/17/1139403/original.jpg	https://data.globe.gov/system/photos/2019/07/17/1139405/original.jpg
GV_1333	44.5909	-72.6143	189.3		https://www.google.com/maps/search/44.5909,-72.6143	https://data.globe.gov/system/photos/2019/07/17/1139414/original.jpg	https://data.globe.gov/system/photos/2019/07/17/1139416/original.jpg	https://data.globe.gov/system/photos/2019/07/17/1139415/original.jpg	https://data.globe.gov/system/photos/2019/07/17/1139418/original.jpg	https://data.globe.gov/system/photos/2019/07/17/1139420/original.jpg
GV_1334	45.3432	-118.0863	849.4		https://www.google.com/maps/search/45.3432,-118.0863	https://data.globe.gov/system/photos/2019/07/15/1139422/original.jpg	https://data.globe.gov/system/photos/2019/07/15/1139424/original.jpg	https://data.globe.gov/system/photos/2019/07/15/1139423/original.jpg	https://data.globe.gov/system/photos/2019/07/15/1139425/original.jpg	https://data.globe.gov/system/photos/2019/07/15/1139427/original.jpg
GV_1335	44.5645	-123.2746	70.7		https://www.google.com/maps/search/44.5645,-123.2746	https://data.globe.gov/system/photos/2019/07/17/1139429/original.jpg	https://data.globe.gov/system/photos/2019/07/17/1139431/original.jpg	https://data.globe.gov/system/photos/2019/07/17/1139430/original.jpg	https://data.globe.gov/system/photos/2019/07/17/1139432/original.jpg	https://data.globe.gov/system/photos/2019/07/17/1139434/original.jpg
GV_1336	44.6018	-72.6267	197.5		https://www.google.com/maps/search/44.6018,-72.6267	https://data.globe.gov/system/photos/2019/07/17/1139444/original.jpg	https://data.globe.gov/system/photos/2019/07/17/1139446/original.jpg	https://data.globe.gov/system/photos/2019/07/17/1139445/original.jpg	https://data.globe.gov/system/photos/2019/07/17/1139447/original.jpg	https://data.globe.gov/system/photos/2019/07/17/1139449/original.jpg
GV_1337	44.6246	-72.6026	270.4		https://www.google.com/maps/search/44.6246,-72.6026	https://data.globe.gov/system/photos/2019/07/17/1139450/original.jpg	https://data.globe.gov/system/photos/2019/07/17/1139452/original.jpg	https://data.globe.gov/system/photos/2019/07/17/1139451/original.jpg	https://data.globe.gov/system/photos/2019/07/17/1139453/original.jpg	https://data.globe.gov/system/photos/2019/07/17/1139455/original.jpg
GV_1338	5.9691	116.0657	0.9		https://www.google.com/maps/search/5.9691,116.0657	https://data.globe.gov/system/photos/2019/07/17/1139456/original.jpg	https://data.globe.gov/system/photos/2019/07/17/1139458/original.jpg	https://data.globe.gov/system/photos/2019/07/17/1139457/original.jpg	https://data.globe.gov/system/photos/2019/07/17/1139459/original.jpg	https://data.globe.gov/system/photos/2019/07/17/1139461/original.jpg
GV_1339	5.9692	116.0664	2.9		https://www.google.com/maps/search/5.9692,116.0664	https://data.globe.gov/system/photos/2019/07/17/1139462/original.jpg	https://data.globe.gov/system/photos/2019/07/17/1139464/original.jpg	https://data.globe.gov/system/photos/2019/07/17/1139463/original.jpg	https://data.globe.gov/system/photos/2019/07/17/1139465/original.jpg	https://data.globe.gov/system/photos/2019/07/17/1139467/original.jpg
GV_1340	5.9705	116.0648	1.3		https://www.google.com/maps/search/5.9705,116.0648	https://data.globe.gov/system/photos/2019/07/17/1139468/original.jpg	https://data.globe.gov/system/photos/2019/07/17/1139470/original.jpg	https://data.globe.gov/system/photos/2019/07/17/1139469/original.jpg	https://data.globe.gov/system/photos/2019/07/17/1139471/original.jpg	https://data.globe.gov/system/photos/2019/07/17/1139473/original.jpg
GV_1341	44.5684	-123.2786	79.5		https://www.google.com/maps/search/44.5684,-123.2786	https://data.globe.gov/system/photos/2019/07/17/1139474/original.jpg	https://data.globe.gov/system/photos/2019/07/17/1139476/original.jpg	https://data.globe.gov/system/photos/2019/07/17/1139475/original.jpg	https://data.globe.gov/system/photos/2019/07/17/1139477/original.jpg	https://data.globe.gov/system/photos/2019/07/17/1139479/original.jpg
GV_1342	44.565	-123.273	70.2		https://www.google.com/maps/search/44.565,-123.273	https://data.globe.gov/system/photos/2019/07/17/1139480/original.jpg	https://data.globe.gov/system/photos/2019/07/17/1139482/original.jpg	https://data.globe.gov/system/photos/2019/07/17/1139481/original.jpg	https://data.globe.gov/system/photos/2019/07/17/1139483/original.jpg	https://data.globe.gov/system/photos/2019/07/17/1139485/original.jpg
GV_1343	13.7843	100.5339	8		https://www.google.com/maps/search/13.7843,100.5339	https://data.globe.gov/system/photos/2019/07/12/1139497/original.jpg	https://data.globe.gov/system/photos/2019/07/12/1139500/original.jpg	https://data.globe.gov/system/photos/2019/07/12/1139499/original.jpg	https://data.globe.gov/system/photos/2019/07/12/1139501/original.jpg	https://data.globe.gov/system/photos/2019/07/12/1139504/original.jpg

GV_1344	36.315	-115.3076	846.5		https://www.google.com/maps/search/36.315,-115.3076	https://data.globe.gov/system/photos/2019/07/17/1139519/original.jpg	https://data.globe.gov/system/photos/2019/07/17/1139521/original.jpg	https://data.globe.gov/system/photos/2019/07/17/1139520/original.jpg	https://data.globe.gov/system/photos/2019/07/17/1139522/original.jpg	https://data.globe.gov/system/photos/2019/07/17/1139524/original.jpg
GV_1345	39.391	-81.4205	194.9		https://www.google.com/maps/search/39.391,-81.4205	https://data.globe.gov/system/photos/2019/07/17/1139534/original.jpg	https://data.globe.gov/system/photos/2019/07/17/1139536/original.jpg	https://data.globe.gov/system/photos/2019/07/17/1139535/original.jpg	https://data.globe.gov/system/photos/2019/07/17/1139537/original.jpg	https://data.globe.gov/system/photos/2019/07/17/1139539/original.jpg
GV_1346	39.0874	-81.7703	180.1		https://www.google.com/maps/search/39.0874,-81.7703	https://data.globe.gov/system/photos/2019/07/17/1139540/original.jpg	https://data.globe.gov/system/photos/2019/07/17/1139542/original.jpg	https://data.globe.gov/system/photos/2019/07/17/1139541/original.jpg	https://data.globe.gov/system/photos/2019/07/17/1139543/original.jpg	https://data.globe.gov/system/photos/2019/07/17/1139545/original.jpg
GV_1347	39.0915	-84.8475	148.4		https://www.google.com/maps/search/39.0915,-84.8475	https://data.globe.gov/system/photos/2019/07/17/1139546/original.jpg	https://data.globe.gov/system/photos/2019/07/17/1139548/original.jpg	https://data.globe.gov/system/photos/2019/07/17/1139547/original.jpg	https://data.globe.gov/system/photos/2019/07/17/1139549/original.jpg	https://data.globe.gov/system/photos/2019/07/17/1139551/original.jpg
GV_1348	45.4641	-117.9655	834.1		https://www.google.com/maps/search/45.4641,-117.9655	https://data.globe.gov/system/photos/2019/07/16/1139552/original.jpg	https://data.globe.gov/system/photos/2019/07/16/1139554/original.jpg	https://data.globe.gov/system/photos/2019/07/16/1139553/original.jpg	https://data.globe.gov/system/photos/2019/07/16/1139555/original.jpg	https://data.globe.gov/system/photos/2019/07/16/1139557/original.jpg
GV_1349	45.5579	-117.9421	846.9		https://www.google.com/maps/search/45.5579,-117.9421	https://data.globe.gov/system/photos/2019/07/17/1139570/original.jpg	https://data.globe.gov/system/photos/2019/07/17/1139572/original.jpg	https://data.globe.gov/system/photos/2019/07/17/1139571/original.jpg	https://data.globe.gov/system/photos/2019/07/17/1139573/original.jpg	https://data.globe.gov/system/photos/2019/07/17/1139575/original.jpg
GV_1350	46.1533	-123.8649	4.7		https://www.google.com/maps/search/46.1533,-123.8649	https://data.globe.gov/system/photos/2019/07/18/1139595/original.jpg	https://data.globe.gov/system/photos/2019/07/18/1139597/original.jpg	https://data.globe.gov/system/photos/2019/07/18/1139596/original.jpg	https://data.globe.gov/system/photos/2019/07/18/1139598/original.jpg	https://data.globe.gov/system/photos/2019/07/18/1139600/original.jpg
GV_1351	42.3586	-83.0625	192.4		https://www.google.com/maps/search/42.3586,-83.0625	https://data.globe.gov/system/photos/2019/07/17/1139631/original.jpg	https://data.globe.gov/system/photos/2019/07/17/1139633/original.jpg	https://data.globe.gov/system/photos/2019/07/17/1139632/original.jpg	https://data.globe.gov/system/photos/2019/07/17/1139634/original.jpg	https://data.globe.gov/system/photos/2019/07/17/1139636/original.jpg
GV_1352	44.5663	-123.273	72		https://www.google.com/maps/search/44.5663,-123.273	https://data.globe.gov/system/photos/2019/07/17/1139646/original.jpg	https://data.globe.gov/system/photos/2019/07/17/1139649/original.jpg	https://data.globe.gov/system/photos/2019/07/17/1139648/original.jpg	https://data.globe.gov/system/photos/2019/07/17/1139650/original.jpg	https://data.globe.gov/system/photos/2019/07/17/1139652/original.jpg
GV_1353	44.5989	-123.2862	97.7		https://www.google.com/maps/search/44.5989,-123.2862	https://data.globe.gov/system/photos/2019/07/18/1139653/original.jpg	https://data.globe.gov/system/photos/2019/07/18/1139655/original.jpg	https://data.globe.gov/system/photos/2019/07/18/1139654/original.jpg	https://data.globe.gov/system/photos/2019/07/18/1139656/original.jpg	https://data.globe.gov/system/photos/2019/07/18/1139658/original.jpg
GV_1354	44.554	-123.2721	67.3		https://www.google.com/maps/search/44.554,-123.2721	https://data.globe.gov/system/photos/2019/07/18/1139659/original.jpg	https://data.globe.gov/system/photos/2019/07/18/1139661/original.jpg	https://data.globe.gov/system/photos/2019/07/18/1139660/original.jpg	https://data.globe.gov/system/photos/2019/07/18/1139662/original.jpg	https://data.globe.gov/system/photos/2019/07/18/1139664/original.jpg
GV_1355	42.3587	-83.0627	192.2		https://www.google.com/maps/search/42.3587,-83.0627	https://data.globe.gov/system/photos/2019/07/17/1139666/original.jpg	https://data.globe.gov/system/photos/2019/07/17/1139668/original.jpg	https://data.globe.gov/system/photos/2019/07/17/1139667/original.jpg	https://data.globe.gov/system/photos/2019/07/17/1139669/original.jpg	https://data.globe.gov/system/photos/2019/07/17/1139671/original.jpg
GV_1356	42.3337	-83.0434	182.5		https://www.google.com/maps/search/42.3337,-83.0434	https://data.globe.gov/system/photos/2019/07/17/1139672/original.jpg	https://data.globe.gov/system/photos/2019/07/17/1139674/original.jpg	https://data.globe.gov/system/photos/2019/07/17/1139673/original.jpg	https://data.globe.gov/system/photos/2019/07/17/1139675/original.jpg	https://data.globe.gov/system/photos/2019/07/17/1139677/original.jpg
GV_1357	44.5651	-123.2869	75.1		https://www.google.com/maps/search/44.5651,-123.2869	https://data.globe.gov/system/photos/2019/07/18/1139689/original.jpg	https://data.globe.gov/system/photos/2019/07/18/1139691/original.jpg	https://data.globe.gov/system/photos/2019/07/18/1139690/original.jpg	https://data.globe.gov/system/photos/2019/07/18/1139694/original.jpg	https://data.globe.gov/system/photos/2019/07/18/1139699/original.jpg
GV_1358	44.5864	-123.2462	68.9		https://www.google.com/maps/search/44.5864,-123.2462	https://data.globe.gov/system/photos/2019/07/18/1139713/original.jpg	https://data.globe.gov/system/photos/2019/07/18/1139715/original.jpg	https://data.globe.gov/system/photos/2019/07/18/1139714/original.jpg	https://data.globe.gov/system/photos/2019/07/18/1139716/original.jpg	https://data.globe.gov/system/photos/2019/07/18/1139718/original.jpg
GV_1359	44.5759	-123.251	70.4		https://www.google.com/maps/search/44.5759,-123.251	https://data.globe.gov/system/photos/2019/07/18/1139725/original.jpg	https://data.globe.gov/system/photos/2019/07/18/1139727/original.jpg	https://data.globe.gov/system/photos/2019/07/18/1139726/original.jpg	https://data.globe.gov/system/photos/2019/07/18/1139728/original.jpg	https://data.globe.gov/system/photos/2019/07/18/1139730/original.jpg
GV_1360	44.554	-123.2726	67		https://www.google.com/maps/search/44.554,-123.2726	https://data.globe.gov/system/photos/2019/07/18/1139731/original.jpg	https://data.globe.gov/system/photos/2019/07/18/1139733/original.jpg	https://data.globe.gov/system/photos/2019/07/18/1139732/original.jpg	https://data.globe.gov/system/photos/2019/07/18/1139734/original.jpg	https://data.globe.gov/system/photos/2019/07/18/1139736/original.jpg
GV_1361	47.5219	-111.2712	1030.7		https://www.google.com/maps/search/47.5219,-111.2712	https://data.globe.gov/system/photos/2019/07/18/1139784/original.jpg	https://data.globe.gov/system/photos/2019/07/18/1139786/original.jpg	https://data.globe.gov/system/photos/2019/07/18/1139785/original.jpg	https://data.globe.gov/system/photos/2019/07/18/1139787/original.jpg	https://data.globe.gov/system/photos/2019/07/18/1139789/original.jpg
GV_1362	44.5663	-123.2531	65.3		https://www.google.com/maps/search/44.5663,-123.2531	https://data.globe.gov/system/photos/2019/07/18/1139790/original.jpg	https://data.globe.gov/system/photos/2019/07/18/1139792/original.jpg	https://data.globe.gov/system/photos/2019/07/18/1139791/original.jpg	https://data.globe.gov/system/photos/2019/07/18/1139793/original.jpg	https://data.globe.gov/system/photos/2019/07/18/1139795/original.jpg
GV_1363	45.4151	-122.6731	31.6		https://www.google.com/maps/search/45.4151,-122.6731	https://data.globe.gov/system/photos/2019/07/18/1139832/original.jpg	https://data.globe.gov/system/photos/2019/07/18/1139834/original.jpg	https://data.globe.gov/system/photos/2019/07/18/1139833/original.jpg	https://data.globe.gov/system/photos/2019/07/18/1139835/original.jpg	https://data.globe.gov/system/photos/2019/07/18/1139837/original.jpg
GV_1364	44.582	-123.2925	110.6		https://www.google.com/maps/search/44.582,-123.2925	https://data.globe.gov/system/photos/2019/07/18/1139838/original.jpg	https://data.globe.gov/system/photos/2019/07/18/1139840/original.jpg	https://data.globe.gov/system/photos/2019/07/18/1139839/original.jpg	https://data.globe.gov/system/photos/2019/07/18/1139841/original.jpg	https://data.globe.gov/system/photos/2019/07/18/1139843/original.jpg
GV_1365	44.5977	-123.2862	92.7		https://www.google.com/maps/search/44.5977,-123.2862	https://data.globe.gov/system/photos/2019/07/18/1139844/original.jpg	https://data.globe.gov/system/photos/2019/07/18/1139846/original.jpg	https://data.globe.gov/system/photos/2019/07/18/1139845/original.jpg	https://data.globe.gov/system/photos/2019/07/18/1139847/original.jpg	https://data.globe.gov/system/photos/2019/07/18/1139849/original.jpg
GV_1366	44.5547	-123.2748	68		https://www.google.com/maps/search/44.5547,-123.2748	https://data.globe.gov/system/photos/2019/07/18/1139850/original.jpg	https://data.globe.gov/system/photos/2019/07/18/1139852/original.jpg	https://data.globe.gov/system/photos/2019/07/18/1139851/original.jpg	https://data.globe.gov/system/photos/2019/07/18/1139853/original.jpg	https://data.globe.gov/system/photos/2019/07/18/1139855/original.jpg
GV_1367	45.412	-122.6634	33.3		https://www.google.com/maps/search/45.412,-122.6634	https://data.globe.gov/system/photos/2019/07/18/1139866/original.jpg	https://data.globe.gov/system/photos/2019/07/18/1139868/original.jpg	https://data.globe.gov/system/photos/2019/07/18/1139867/original.jpg	https://data.globe.gov/system/photos/2019/07/18/1139869/original.jpg	https://data.globe.gov/system/photos/2019/07/18/1139871/original.jpg
GV_1368	41.4332	-81.6617	194.8		https://www.google.com/maps/search/41.4332,-81.6617	https://data.globe.gov/system/photos/2019/07/18/1139926/original.jpg	https://data.globe.gov/system/photos/2019/07/18/1139928/original.jpg	https://data.globe.gov/system/photos/2019/07/18/1139927/original.jpg	https://data.globe.gov/system/photos/2019/07/18/1139929/original.jpg	https://data.globe.gov/system/photos/2019/07/18/1139931/original.jpg
GV_1369	51.5122	-1.7719	181.4		https://www.google.com/maps/search/51.5122,-1.7719	https://data.globe.gov/system/photos/2019/07/18/1139944/original.jpg	https://data.globe.gov/system/photos/2019/07/18/1139946/original.jpg	https://data.globe.gov/system/photos/2019/07/18/1139945/original.jpg	https://data.globe.gov/system/photos/2019/07/18/1139947/original.jpg	https://data.globe.gov/system/photos/2019/07/18/1139949/original.jpg
GV_1370	53.4291	-0.6103	35.4		https://www.google.com/maps/search/53.4291,-0.6103	https://data.globe.gov/system/photos/2019/07/18/1140018/original.jpg	https://data.globe.gov/system/photos/2019/07/18/1140020/original.jpg	https://data.globe.gov/system/photos/2019/07/18/1140019/original.jpg	https://data.globe.gov/system/photos/2019/07/18/1140021/original.jpg	https://data.globe.gov/system/photos/2019/07/18/1140023/original.jpg
GV_1371	42.3017	-83.6889	275.4		https://www.google.com/maps/search/42.3017,-83.6889	https://data.globe.gov/system/photos/2019/07/18/1140098/original.jpg	https://data.globe.gov/system/photos/2019/07/18/1140100/original.jpg	https://data.globe.gov/system/photos/2019/07/18/1140099/original.jpg	https://data.globe.gov/system/photos/2019/07/18/1140101/original.jpg	https://data.globe.gov/system/photos/2019/07/18/1140103/original.jpg
GV_1372	53.414	-0.6052	37.9		https://www.google.com/maps/search/53.414,-0.6052	https://data.globe.gov/system/photos/2019/07/18/1140217/original.jpg	https://data.globe.gov/system/photos/2019/07/18/1140219/original.jpg	https://data.globe.gov/system/photos/2019/07/18/1140218/original.jpg	https://data.globe.gov/system/photos/2019/07/18/1140220/original.jpg	https://data.globe.gov/system/photos/2019/07/18/1140222/original.jpg
GV_1373	38.8843	-84.7526	154.2		https://www.google.com/maps/search/38.8843,-84.7526	https://data.globe.gov/system/photos/2019/07/18/1140364/original.jpg	https://data.globe.gov/system/photos/2019/07/18/1140366/original.jpg	https://data.globe.gov/system/photos/2019/07/18/1140365/original.jpg	https://data.globe.gov/system/photos/2019/07/18/1140367/original.jpg	https://data.globe.gov/system/photos/2019/07/18/1140369/original.jpg
GV_1374	38.8851	-84.7527	146.8		https://www.google.com/maps/search/38.8851,-84.7527	https://data.globe.gov/system/photos/2019/07/18/1140370/original.jpg	https://data.globe.gov/system/photos/2019/07/18/1140372/original.jpg	https://data.globe.gov/system/photos/2019/07/18/1140371/original.jpg	https://data.globe.gov/system/photos/2019/07/18/1140373/original.jpg	https://data.globe.gov/system/photos/2019/07/18/1140375/original.jpg
GV_1375	38.8861	-84.7522	146.1		https://www.google.com/maps/search/38.8861,-84.7522	https://data.globe.gov/system/photos/2019/07/18/1140376/original.jpg	https://data.globe.gov/system/photos/2019/07/18/1140378/original.jpg	https://data.globe.gov/system/photos/2019/07/18/1140377/original.jpg	https://data.globe.gov/system/photos/2019/07/18/1140379/original.jpg	https://data.globe.gov/system/photos/2019/07/18/1140381/original.jpg
GV_1376	38.8861	-84.7525	146.6		https://www.google.com/maps/search/38.8861,-84.7525	https://data.globe.gov/system/photos/2019/07/18/1140382/original.jpg	https://data.globe.gov/system/photos/2019/07/18/1140384/original.jpg	https://data.globe.gov/system/photos/2019/07/18/1140383/original.jpg	https://data.globe.gov/system/photos/2019/07/18/1140385/original.jpg	https://data.globe.gov/system/photos/2019/07/18/1140387/original.jpg
GV_1377	38.8846	-84.7538	144.5		https://www.google.com/maps/search/38.8846,-84.7538	https://data.globe.gov/system/photos/2019/07/18/1140388/original.jpg	https://data.globe.gov/system/photos/2019/07/18/1140390/original.jpg	https://data.globe.gov/system/photos/2019/07/18/1140389/original.jpg	https://data.globe.gov/system/photos/2019/07/18/1140391/original.jpg	https://data.globe.gov/system/photos/2019/07/18/1140393/original.jpg
GV_1378	38.8829	-84.7533	172.8		https://www.google.com/maps/search/38.8829,-84.7533	https://data.globe.gov/system/photos/2019/07/18/1140394/original.jpg	https://data.globe.gov/system/photos/2019/07/18/1140396/original.jpg	https://data.globe.gov/system/photos/2019/07/18/1140395/original.jpg	https://data.globe.gov/system/photos/2019/07/18/1140397/original.jpg	https://data.globe.gov/system/photos/2019/07/18/1140399/original.jpg
GV_1379	38.0446	-122.1568	0		https://www.google.com/maps/search/38.0446,-122.1568	https://data.globe.gov/system/photos/2019/07/18/1140472/original.jpg	https://data.globe.gov/system/photos/2019/07/18/1140474/original.jpg	https://data.globe.gov/system/photos/2019/07/18/1140473/original.jpg	https://data.globe.gov/system/photos/2019/07/18/1140475/original.jpg	https://data.globe.gov/system/photos/2019/07/18/1140478/original.jpg
GV_1380	42.3278	-83.0478	184		https://www.google.com/maps/search/42.3278,-83.0478	https://data.globe.gov/system/photos/2019/07/18/1140492/original.jpg	https://data.globe.gov/system/photos/2019/07/18/1140494/original.jpg	https://data.globe.gov/system/photos/2019/07/18/1140493/original.jpg	https://data.globe.gov/system/photos/2019/07/18/1140495/original.jpg	https://data.globe.gov/system/photos/2019/07/18/1140497/original.jpg
GV_1381	47.1155	-122.387	127.6		https://www.google.com/maps/search/47.1155,-122.387	https://data.globe.gov/system/photos/2019/07/18/1140555/original.jpg	https://data.globe.gov/system/photos/2019/07/18/1140560/original.jpg	https://data.globe.gov/system/photos/2019/07/18/1140557/original.jpg	https://data.globe.gov/system/photos/2019/07/18/1140570/original.jpg	https://data.globe.gov/system/photos/2019/07/18/1140573/original.jpg
GV_1382	47.0994	-122.3592	105.8		https://www.google.com/maps/search/47.0994,-122.3592	https://data.globe.gov/system/photos/2019/07/18/1140574/original.jpg	https://data.globe.gov/system/photos/2019/07/18/1140577/original.jpg	https://data.globe.gov/system/photos/2019/07/18/1140575/original.jpg	https://data.globe.gov/system/photos/2019/07/18/1140580/original.jpg	https://data.globe.gov/system/photos/2019/07/18/1140583/original.jpg
GV_1383	42.3282	-83.045	183		https://www.google.com/maps/search/42.3282,-83.045	https://data.globe.gov/system/photos/2019/07/18/1140600/original.jpg	https://data.globe.gov/system/photos/2019/07/18/1140602/original.jpg	https://data.globe.gov/system/photos/2019/07/18/1140601/original.jpg	https://data.globe.gov/system/photos/2019/07/18/1140603/original.jpg	https://data.globe.gov/system/photos/2019/07/18/1140605/original.jpg
GV_1384	42.3274	-83.047	179.5		https://www.google.com/maps/search/42.3274,-83.047	https://data.globe.gov/system/photos/2019/07/18/1140606/original.jpg	https://data.globe.gov/system/photos/2019/07/18/1140608/original.jpg	https://data.globe.gov/system/photos/2019/07/18/1140607/original.jpg	https://data.globe.gov/system/photos/2019/07/18/1140609/original.jpg	https://data.globe.gov/system/photos/2019/07/18/1140611/original.jpg
GV_1385	51.1791	-1.825	99.8		https://www.google.com/maps/search/51.1791,-1.825	https://data.globe.gov/system/photos/2019/07/18/1140613/original.jpg	https://data.globe.gov/system/photos/2019/07/18/1140615/original.jpg	https://data.globe.gov/system/photos/2019/07/18/1140614/original.jpg	https://data.globe.gov/system/photos/2019/07/18/1140616/original.jpg	https://data.globe.gov/system/photos/2019/07/18/1140620/original.jpg
GV_1386	51.1855	-1.8582	100		https://www.google.com/maps/search/51.1855,-1.8582	https://data.globe.gov/system/photos/2019/07/18/1140624/original.jpg	https://data.globe.gov/system/photos/2019/07/18/1140627/original.jpg	https://data.globe.gov/system/photos/2019/07/18/1140626/original.jpg	https://data.globe.gov/system/photos/2019/07/18/1140628/original.jpg	https://data.globe.gov/system/photos/2019/07/18/1140630/original.jpg
GV_1387	51.1788	-1.8265	102		https://www.google.com/maps/search/51.1788,-1.8265	https://data.globe.gov/system/photos/2019/07/18/1140631/original.jpg	https://data.globe.gov/system/photos/2019/07/18/1140633/original.jpg	https://data.globe.gov/system/photos/2019/07/18/1140632/original.jpg	https://data.globe.gov/system/photos/2019/07/18/1140634/original.jpg	https://data.globe.gov/system/photos/2019/07/18/1140636/original.jpg
GV_1388	51.4289	-1.8564	157.4		https://www.google.com/maps/search/51.4289,-1.8564	https://data.globe.gov/system/photos/2019/07/18/1140637/original.jpg	https://data.globe.gov/system/photos/2019/07/18/1140639/original.jpg	https://data.globe.gov/system/photos/2019/07/18/1140638/original.jpg	https://data.globe.gov/system/photos/2019/07/18/1140640/original.jpg	https://data.globe.gov/system/photos/2019/07/18/1140642/original.jpg
GV_1389	40.6331	-74.024	26.1		https://www.google.com/maps/search/40.6331,-74.024	https://data.globe.gov/system/photos/2019/07/18/1140676/original.jpg	https://data.globe.gov/system/photos/2019/07/18/1140678/original.jpg	https://data.globe.gov/system/photos/2019/07/18/1140677/original.jpg	https://data.globe.gov/system/photos/2019/07/18/1140680/original.jpg	https://data.globe.gov/system/photos/2019/07/18/1140682/original.jpg
GV_1390	47.7033	-117.6393	712.5		https://www.google.com/maps/search/47.7033,-117.6393	https://data.globe.gov/system/photos/2019/07/19/1140735/original.jpg	https://data.globe.gov/system/photos/2019/07/19/1140737/original.jpg	https://data.globe.gov/system/photos/2019/07/19/1140736/original.jpg	https://data.globe.gov/system/photos/2019/07/19/1140738/original.jpg	https://data.globe.gov/system/photos/2019/07/19/1140740/original.jpg
GV_1391	42.3592	-83.0625	192.2		https://www.google.com/maps/search/42.3592,-83.0625	https://data.globe.gov/system/photos/2019/07/17/1140755/original.jpg	https://data.globe.gov/system/photos/2019/07/17/1140757/original.jpg	https://data.globe.gov/system/photos/2019/07/17/1140756/original.jpg	https://data.globe.gov/system/photos/2019/07/17/1140758/original.jpg	https://data.globe.gov/system/photos/2019/07/17/1140760/original.jpg
GV_1392	53.4293	-0.6102	35.3		https://www.google.com/maps/search/53.4293,-0.6102	https://data.globe.gov/system/photos/2019/07/19/1140967/original.jpg	https://data.globe.gov/system/photos/2019/07/19/1140969/original.jpg	https://data.globe.gov/system/photos/2019/07/19/1140968/original.jpg	https://data.globe.gov/system/photos/2019/07/19/1140970/original.jpg	https://data.globe.gov/system/photos/2019/07/19/1140972/original.jpg
GV_1393	51.5607	-0.2775	32.5		https://www.google.com/maps/search/51.5607,-0.2775	https://data.globe.gov/system/photos/2019/07/19/1141038/original.jpg	https://data.globe.gov/system/photos/2019/07/19/1141040/original.jpg	https://data.globe.gov/system/photos/2019/07/19/1141039/original.jpg	https://data.globe.gov/system/photos/2019/07/19/1141041/original.jpg	https://data.globe.gov/system/photos/2019/07/19/1141043/original.jpg
GV_1394	42.1047	-83.2367	180.9		https://www.google.com/maps/search/42.1047,-83.2367	https://data.globe.gov/system/photos/2019/07/19/1141139/original.jpg	https://data.globe.gov/system/photos/2019/07/19/1141141/original.jpg	https://data.globe.gov/system/photos/2019/07/19/1141140/original.jpg	https://data.globe.gov/system/photos/2019/07/19/1141142/original.jpg	https://data.globe.gov/system/photos/2019/07/19/1141144/original.jpg
GV_1395	45.6693	-111.0647	1483.9		https://www.google.com/maps/search/45.6693,-111.0647	https://data.globe.gov/system/photos/2019/07/19/1141477/original.jpg	https://data.globe.gov/system/photos/2019/07/19/1141479/original.jpg	https://data.globe.gov/system/photos/2019/07/19/1141478/original.jpg	https://data.globe.gov/system/photos/2019/07/19/1141480/original.jpg	https://data.globe.gov/system/photos/2019/07/19/1141482/original.jpg
GV_1396	45.6652	-111.0579	1491.6		https://www.google.com/maps/search/45.6652,-111.0579	https://data.globe.gov/system/photos/2019/07/19/1141483/original.jpg	https://data.globe.gov/system/photos/2019/07/19/1141485/original.jpg	https://data.globe.gov/system/photos/2019/07/19/1141484/original.jpg	https://data.globe.gov/system/photos/2019/07/19/1141486/original.jpg	https://data.globe.gov/system/photos/2019/07/19/1141488/original.jpg
GV_1397	53.429	-0.6102	35.6		https://www.google.com/maps/search/53.429,-0.6102	https://data.globe.gov/system/photos/2019/07/20/1141972/original.jpg	https://data.globe.gov/system/photos/2019/07/20/1141974/original.jpg	https://data.globe.gov/system/photos/2019/07/20/1141973/original.jpg	https://data.globe.gov/system/photos/2019/07/20/1141975/original.jpg	https://data.globe.gov/system/photos/2019/07/20/1141977/original.jpg
GV_1398	53.4166	-0.6018	41.1		https://www.google.com/maps/search/53.4166,-0.6018	https://data.globe.gov/system/photos/2019/07/20/1141978/original.jpg	https://data.globe.gov/system/photos/2019/07/20/1141980/original.jpg	https://data.globe.gov/system/photos/2019/07/20/1141979/original.jpg	https://data.globe.gov/system/photos/2019/07/20/1141981/original.jpg	https://data.globe.gov/system/photos/2019/07/20/1141983/original.jpg
GV_1399	51.4755	-0.2977	8.9		https://www.google.com/maps/search/51.4755,-0.2977	https://data.globe.gov/system/photos/2019/07/20/1142135/original.jpg	https://data.globe.gov/system/photos/2019/07/20/1142137/original.jpg	https://data.globe.gov/system/photos/2019/07/20/1142136/original.jpg	https://data.globe.gov/system/photos/2019/07/20/1142138/original.jpg	https://data.globe.gov/system/photos/2019/07/20/1142140/original.jpg
GV_1400	51.4823	-0.2894	6.1		https://www.google.com/maps/search/51.4823,-0.2894	https://data.globe.gov/system/photos/2019/07/20/1142144/original.jpg	https://data.globe.gov/system/photos/2019/07/20/1142146/original.jpg	https://data.globe.gov/system/photos/2019/07/20/1142145/original.jpg	https://data.globe.gov/system/photos/2019/07/20/1142147/original.jpg	https://data.globe.gov/system/photos/2019/07/20/1142149/original.jpg
GV_1401	51.4844	-0.2911	5.6		https://www.google.com/maps/search/51.4844,-0.2911	https://data.globe.gov/system/photos/2019/07/20/1142150/original.jpg	https://data.globe.gov/system/photos/2019/07/20/1142152/original.jpg	https://data.globe.gov/system/photos/2019/07/20/1142151/original.jpg	https://data.globe.gov/system/photos/2019/07/20/1142153/original.jpg	https://data.globe.gov/system/photos/2019/07/20/1142155/original.jpg
GV_1402	51.4816	-0.2883	5.8		https://www.google.com/maps/search/51.4816,-0.2883	https://data.globe.gov/system/photos/2019/07/20/1142156/original.jpg	https://data.globe.gov/system/photos/2019/07/20/1142158/original.jpg	https://data.globe.gov/system/photos/2019/07/20/1142157/original.jpg	https://data.globe.gov/system/photos/2019/07/20/1142159/original.jpg	https://data.globe.gov/system/photos/2019/07/20/1142161/original.jpg
GV_1403	51.4831	-0.2904	6.3		https://www.google.com/maps/search/51.4831,-0.2904	https://data.globe.gov/system/photos/2019/07/20/1142167/original.jpg	https://data.globe.gov/system/photos/2019/07/20/1142169/original.jpg	https://data.globe.gov/system/photos/2019/07/20/1142168/original.jpg	https://data.globe.gov/system/photos/2019/07/20/1142170/original.jpg	https://data.globe.gov/system/photos/2019/07/20/1142172/original.jpg
GV_1404	51.4794	-0.2958	6		https://www.google.com/maps/search/51.4794,-0.2958	https://data.globe.gov/system/photos/2019/07/20/1142178/original.jpg	https://data.globe.gov/system/photos/2019/07/20/1142180/original.jpg	https://data.globe.gov/system/photos/2019/07/20/1142179/original.jpg	https://data.globe.gov/system/photos/2019/07/20/1142181/original.jpg	https://data.globe.gov/system/photos/2019/07/20/1142183/original.jpg
GV_1405	51.4754	-0.2973	9.3		https://www.google.com/maps/search/51.4754,-0.2973	https://data.globe.gov/system/photos/2019/07/20/1142184/original.jpg	https://data.globe.gov/system/photos/2019/07/20/1142186/original.jpg	https://data.globe.gov/system/photos/2019/07/20/1142185/original.jpg	https://data.globe.gov/system/photos/2019/07/20/1142187/original.jpg	https://data.globe.gov/system/photos/2019/07/20/1142189/original.jpg
GV_1406	51.4772	-0.2946	7		https://www.google.com/maps/search/51.4772,-0.2946	https://data.globe.gov/system/photos/2019/07/20/1142190/original.jpg	https://data.globe.gov/system/photos/2019/07/20/1142192/original.jpg	https://data.globe.gov/system/photos/2019/07/20/1142191/original.jpg	https://data.globe.gov/system/photos/2019/07/20/1142193/original.jpg	https://data.globe.gov/system/photos/2019/07/20/1142195/original.jpg
GV_1407	51.4792	-0.2905	6.5		https://www.google.com/maps/search/51.4792,-0.2905	https://data.globe.gov/system/photos/2019/07/20/1142196/original.jpg	https://data.globe.gov/system/photos/2019/07/20/1142198/original.jpg	https://data.globe.gov/system/photos/2019/07/20/1142197/original.jpg	https://data.globe.gov/system/photos/2019/07/20/1142199/original.jpg	https://data.globe.gov/system/photos/2019/07/20/1142201/original.jpg
GV_1408	51.4794	-0.2921	5.5		https://www.google.com/maps/search/51.4794,-0.2921	https://data.globe.gov/system/photos/2019/07/20/1142202/original.jpg	https://data.globe.gov/system/photos/2019/07/20/1142204/original.jpg	https://data.globe.gov/system/photos/2019/07/20/1142203/original.jpg	https://data.globe.gov/system/photos/2019/07/20/1142205/original.jpg	https://data.globe.gov/system/photos/2019/07/20/1142207/original.jpg
GV_1409	51.5604	-0.277	33.1		https://www.google.com/maps/search/51.5604,-0.277	https://data.globe.gov/system/photos/2019/07/20/1142208/original.jpg	https://data.globe.gov/system/photos/2019/07/20/1142210/original.jpg	https://data.globe.gov/system/photos/2019/07/20/1142209/original.jpg	https://data.globe.gov/system/photos/2019/07/20/1142211/original.jpg	https://data.globe.gov/system/photos/2019/07/20/1142213/original.jpg
GV_1410	38.0157	-122.2778	7.5		https://www.google.com/maps/search/38.0157,-122.2778	https://data.globe.gov/system/photos/2019/07/21/1142503/original.jpg	https://data.globe.gov/system/photos/2019/07/21/1142505/original.jpg	https://data.globe.gov/system/photos/2019/07/21/1142504/original.jpg	https://data.globe.gov/system/photos/2019/07/21/1142506/original.jpg	https://data.globe.gov/system/photos/2019/07/21/1142508/original.jpg
GV_1411	45.2643	-118.0429	841.5		https://www.google.com/maps/search/45.2643,-118.0429	https://data.globe.gov/system/photos/2019/07/16/1142873/original.jpg	https://data.globe.gov/system/photos/2019/07/16/1142875/original.jpg	https://data.globe.gov/system/photos/2019/07/16/1142874/original.jpg	https://data.globe.gov/system/photos/2019/07/16/1142876/original.jpg	https://data.globe.gov/system/photos/2019/07/16/1142878/original.jpg
GV_1412	45.2968	-118.1334	1289.4		https://www.google.com/maps/search/45.2968,-118.1334	https://data.globe.gov/system/photos/2019/07/16/1142879/original.jpg	https://data.globe.gov/system/photos/2019/07/16/1142881/original.jpg	https://data.globe.gov/system/photos/2019/07/16/1142880/original.jpg	https://data.globe.gov/system/photos/2019/07/16/1142882/original.jpg	https://data.globe.gov/system/photos/2019/07/16/1142884/original.jpg
GV_1413	45.302	-118.1388	1273		https://www.google.com/maps/search/45.302,-118.1388	https://data.globe.gov/system/photos/2019/07/16/1142885/original.jpg	https://data.globe.gov/system/photos/2019/07/16/1142887/original.jpg	https://data.globe.gov/system/photos/2019/07/16/1142886/original.jpg	https://data.globe.gov/system/photos/2019/07/16/1142888/original.jpg	https://data.globe.gov/system/photos/2019/07/16/1142890/original.jpg
GV_1414	45.6964	-111.0245	1441.8		https://www.google.com/maps/search/45.6964,-111.0245	https://data.globe.gov/system/photos/2019/07/21/1143089/original.jpg	https://data.globe.gov/system/photos/2019/07/21/1143091/original.jpg	https://data.globe.gov/system/photos/2019/07/21/1143090/original.jpg	https://data.globe.gov/system/photos/2019/07/21/1143092/original.jpg	https://data.globe.gov/system/photos/2019/07/21/1143094/original.jpg
GV_1415	45.6818	-111.0222	1465.3		https://www.google.com/maps/search/45.6818,-111.0222	https://data.globe.gov/system/photos/2019/07/21/1143113/original.jpg	https://data.globe.gov/system/photos/2019/07/21/1143116/original.jpg	https://data.globe.gov/system/photos/2019/07/21/1143115/original.jpg	https://data.globe.gov/system/photos/2019/07/21/1143117/original.jpg	https://data.globe.gov/system/photos/2019/07/21/1143119/original.jpg
GV_1416	45.4446	-111.0037	3090.7		https://www.google.com/maps/search/45.4446,-111.0037	https://data.globe.gov/system/photos/2019/07/20/1143120/original.jpg	https://data.globe.gov/system/photos/2019/07/20/1143122/original.jpg	https://data.globe.gov/system/photos/2019/07/20/1143121/original.jpg	https://data.globe.gov/system/photos/2019/07/20/1143123/original.jpg	https://data.globe.gov/system/photos/2019/07/20/1143125/original.jpg
GV_1417	45.4465	-110.9893	2649.2		https://www.google.com/maps/search/45.4465,-110.9893	https://data.globe.gov/system/photos/2019/07/20/1143138/original.jpg	https://data.globe.gov/system/photos/2019/07/20/1143140/original.jpg	https://data.globe.gov/system/photos/2019/07/20/1143139/original.jpg	https://data.globe.gov/system/photos/2019/07/20/1143141/original.jpg	https://data.globe.gov/system/photos/2019/07/20/1143143/original.jpg
GV_1418	42.328	-83.0478	182.4		https://www.google.com/maps/search/42.328,-83.0478	https://data.globe.gov/system/photos/2019/07/17/1143236/original.jpg	https://data.globe.gov/system/photos/2019/07/17/1143238/original.jpg	https://data.globe.gov/system/photos/2019/07/17/1143237/original.jpg	https://data.globe.gov/system/photos/2019/07/17/1143239/original.jpg	https://data.globe.gov/system/photos/2019/07/17/1143241/original.jpg
GV_1419	53.4291	-0.6103	35.4		https://www.google.com/maps/search/53.4291,-0.6103	https://data.globe.gov/system/photos/2019/07/21/1143427/original.jpg	https://data.globe.gov/system/photos/2019/07/21/1143429/original.jpg	https://data.globe.gov/system/photos/2019/07/21/1143428/original.jpg	https://data.globe.gov/system/photos/2019/07/21/1143430/original.jpg	https://data.globe.gov/system/photos/2019/07/21/1143432/original.jpg
GV_1420	53.4266	-0.6297	24.9		https://www.google.com/maps/search/53.4266,-0.6297	https://data.globe.gov/system/photos/2019/07/21/1143439/original.jpg	https://data.globe.gov/system/photos/2019/07/21/1143441/original.jpg	https://data.globe.gov/system/photos/2019/07/21/1143440/original.jpg	https://data.globe.gov/system/photos/2019/07/21/1143442/original.jpg	https://data.globe.gov/system/photos/2019/07/21/1143444/original.jpg
GV_1421	53.429	-0.6101	35.7		https://www.google.com/maps/search/53.429,-0.6101	https://data.globe.gov/system/photos/2019/07/22/1143445/original.jpg	https://data.globe.gov/system/photos/2019/07/22/1143447/original.jpg	https://data.globe.gov/system/photos/2019/07/22/1143446/original.jpg	https://data.globe.gov/system/photos/2019/07/22/1143448/original.jpg	https://data.globe.gov/system/photos/2019/07/22/1143450/original.jpg
GV_1422	31.8793	-106.6028	1147.6		https://www.google.com/maps/search/31.8793,-106.6028	https://data.globe.gov/system/photos/2019/07/22/1143536/original.jpg	https://data.globe.gov/system/photos/2019/07/22/1143538/original.jpg	https://data.globe.gov/system/photos/2019/07/22/1143537/original.jpg	https://data.globe.gov/system/photos/2019/07/22/1143539/original.jpg	https://data.globe.gov/system/photos/2019/07/22/1143541/original.jpg
GV_1423	31.8724	-106.5734	1199.9		https://www.google.com/maps/search/31.8724,-106.5734	https://data.globe.gov/system/photos/2019/07/22/1143556/original.jpg	https://data.globe.gov/system/photos/2019/07/22/1143558/original.jpg	https://data.globe.gov/system/photos/2019/07/22/1143557/original.jpg	https://data.globe.gov/system/photos/2019/07/22/1143559/original.jpg	https://data.globe.gov/system/photos/2019/07/22/1143561/original.jpg
GV_1424	31.8674	-106.5616	1222		https://www.google.com/maps/search/31.8674,-106.5616	https://data.globe.gov/system/photos/2019/07/22/1143568/original.jpg	https://data.globe.gov/system/photos/2019/07/22/1143570/original.jpg	https://data.globe.gov/system/photos/2019/07/22/1143569/original.jpg	https://data.globe.gov/system/photos/2019/07/22/1143571/original.jpg	https://data.globe.gov/system/photos/2019/07/22/1143573/original.jpg
GV_1425	42.3852	-83.4842	247.4		https://www.google.com/maps/search/42.3852,-83.4842	https://data.globe.gov/system/photos/2019/07/22/1143582/original.jpg	https://data.globe.gov/system/photos/2019/07/22/1143584/original.jpg	https://data.globe.gov/system/photos/2019/07/22/1143583/original.jpg	https://data.globe.gov/system/photos/2019/07/22/1143585/original.jpg	https://data.globe.gov/system/photos/2019/07/22/1143588/original.jpg
GV_1426	39.2946	-84.3994	239.9		https://www.google.com/maps/search/39.2946,-84.3994	https://data.globe.gov/system/photos/2019/07/22/1143705/original.jpg	https://data.globe.gov/system/photos/2019/07/22/1143707/original.jpg	https://data.globe.gov/system/photos/2019/07/22/1143706/original.jpg	https://data.globe.gov/system/photos/2019/07/22/1143708/original.jpg	https://data.globe.gov/system/photos/2019/07/22/1143710/original.jpg
GV_1427	53.4284	-0.6241	24.3		https://www.google.com/maps/search/53.4284,-0.6241	https://data.globe.gov/system/photos/2019/07/22/1143915/original.jpg	https://data.globe.gov/system/photos/2019/07/22/1143917/original.jpg	https://data.globe.gov/system/photos/2019/07/22/1143916/original.jpg	https://data.globe.gov/system/photos/2019/07/22/1143918/original.jpg	https://data.globe.gov/system/photos/2019/07/22/1143920/original.jpg
GV_1428	38.0155	-122.2773	8.2		https://www.google.com/maps/search/38.0155,-122.2773	https://data.globe.gov/system/photos/2019/07/23/1144094/original.jpg	https://data.globe.gov/system/photos/2019/07/23/1144096/original.jpg	https://data.globe.gov/system/photos/2019/07/23/1144095/original.jpg	https://data.globe.gov/system/photos/2019/07/23/1144097/original.jpg	https://data.globe.gov/system/photos/2019/07/23/1144099/original.jpg
GV_1429	22.6186	120.2782	0.6		https://www.google.com/maps/search/22.6186,120.2782	https://data.globe.gov/system/photos/2019/07/23/1144233/original.jpg	https://data.globe.gov/system/photos/2019/07/23/1144235/original.jpg	https://data.globe.gov/system/photos/2019/07/23/1144234/original.jpg	https://data.globe.gov/system/photos/2019/07/23/1144236/original.jpg	https://data.globe.gov/system/photos/2019/07/23/1144238/original.jpg
GV_1430	45.4977	-122.6696	12.6		https://www.google.com/maps/search/45.4977,-122.6696	https://data.globe.gov/system/photos/2019/07/23/1144598/original.jpg	https://data.globe.gov/system/photos/2019/07/23/1144600/original.jpg	https://data.globe.gov/system/photos/2019/07/23/1144599/original.jpg	https://data.globe.gov/system/photos/2019/07/23/1144601/original.jpg	https://data.globe.gov/system/photos/2019/07/23/1144603/original.jpg
GV_1431	42.3588	-83.0626	192.4		https://www.google.com/maps/search/42.3588,-83.0626	https://data.globe.gov/system/photos/2019/07/17/1144646/original.jpg	https://data.globe.gov/system/photos/2019/07/17/1144648/original.jpg	https://data.globe.gov/system/photos/2019/07/17/1144647/original.jpg	https://data.globe.gov/system/photos/2019/07/17/1144649/original.jpg	https://data.globe.gov/system/photos/2019/07/17/1144651/original.jpg
GV_1432	42.3586	-83.0627	192.1		https://www.google.com/maps/search/42.3586,-83.0627	https://data.globe.gov/system/photos/2019/07/17/1144655/original.jpg	https://data.globe.gov/system/photos/2019/07/17/1144657/original.jpg	https://data.globe.gov/system/photos/2019/07/17/1144656/original.jpg	https://data.globe.gov/system/photos/2019/07/17/1144658/original.jpg	https://data.globe.gov/system/photos/2019/07/17/1144660/original.jpg
GV_1433	35.3032	-106.5786	1538.1		https://www.google.com/maps/search/35.3032,-106.5786	https://data.globe.gov/system/photos/2019/07/23/1145043/original.jpg	https://data.globe.gov/system/photos/2019/07/23/1145046/original.jpg	https://data.globe.gov/system/photos/2019/07/23/1145045/original.jpg	https://data.globe.gov/system/photos/2019/07/23/1145047/original.jpg	https://data.globe.gov/system/photos/2019/07/23/1145049/original.jpg

GV_1434	35.3029	-106.578	1537.9		https://www.google.com/maps/search/35.3029,-106.578	https://data.globe.gov/system/photos/2019/07/23/1145051/original.jpg	https://data.globe.gov/system/photos/2019/07/23/1145053/original.jpg	https://data.globe.gov/system/photos/2019/07/23/1145052/original.jpg	https://data.globe.gov/system/photos/2019/07/23/1145054/original.jpg	https://data.globe.gov/system/photos/2019/07/23/1145056/original.jpg
GV_1435	35.3035	-106.5746	1538.2		https://www.google.com/maps/search/35.3035,-106.5746	https://data.globe.gov/system/photos/2019/07/23/1145057/original.jpg	https://data.globe.gov/system/photos/2019/07/23/1145059/original.jpg	https://data.globe.gov/system/photos/2019/07/23/1145058/original.jpg	https://data.globe.gov/system/photos/2019/07/23/1145060/original.jpg	https://data.globe.gov/system/photos/2019/07/23/1145062/original.jpg
GV_1436	35.3047	-106.5724	1538.4		https://www.google.com/maps/search/35.3047,-106.5724	https://data.globe.gov/system/photos/2019/07/23/1145063/original.jpg	https://data.globe.gov/system/photos/2019/07/23/1145065/original.jpg	https://data.globe.gov/system/photos/2019/07/23/1145064/original.jpg	https://data.globe.gov/system/photos/2019/07/23/1145067/original.jpg	https://data.globe.gov/system/photos/2019/07/23/1145072/original.jpg
GV_1437	22.6074	120.297	1.7		https://www.google.com/maps/search/22.6074,120.297	https://data.globe.gov/system/photos/2019/07/24/1145145/original.jpg	https://data.globe.gov/system/photos/2019/07/24/1145147/original.jpg	https://data.globe.gov/system/photos/2019/07/24/1145146/original.jpg	https://data.globe.gov/system/photos/2019/07/24/1145148/original.jpg	https://data.globe.gov/system/photos/2019/07/24/1145150/original.jpg
GV_1438	36.8181	-121.7309	26.3		https://www.google.com/maps/search/36.8181,-121.7309	https://data.globe.gov/system/photos/2019/07/24/1145577/original.jpg	https://data.globe.gov/system/photos/2019/07/24/1145582/original.jpg	https://data.globe.gov/system/photos/2019/07/24/1145581/original.jpg	https://data.globe.gov/system/photos/2019/07/24/1145583/original.jpg	https://data.globe.gov/system/photos/2019/07/24/1145585/original.jpg
GV_1439	40.8021	-74.2627	113.6		https://www.google.com/maps/search/40.8021,-74.2627	https://data.globe.gov/system/photos/2019/07/15/1145993/original.jpg	https://data.globe.gov/system/photos/2019/07/15/1145995/original.jpg	https://data.globe.gov/system/photos/2019/07/15/1145994/original.jpg	https://data.globe.gov/system/photos/2019/07/15/1145996/original.jpg	https://data.globe.gov/system/photos/2019/07/15/1145998/original.jpg
GV_1440	40.7976	-74.2538	167.4		https://www.google.com/maps/search/40.7976,-74.2538	https://data.globe.gov/system/photos/2019/07/15/1146001/original.jpg	https://data.globe.gov/system/photos/2019/07/15/1146003/original.jpg	https://data.globe.gov/system/photos/2019/07/15/1146002/original.jpg	https://data.globe.gov/system/photos/2019/07/15/1146004/original.jpg	https://data.globe.gov/system/photos/2019/07/15/1146006/original.jpg
GV_1441	40.8242	-74.2045	87.2		https://www.google.com/maps/search/40.8242,-74.2045	https://data.globe.gov/system/photos/2019/07/24/1146008/original.jpg	https://data.globe.gov/system/photos/2019/07/24/1146010/original.jpg	https://data.globe.gov/system/photos/2019/07/24/1146009/original.jpg	https://data.globe.gov/system/photos/2019/07/24/1146011/original.jpg	https://data.globe.gov/system/photos/2019/07/24/1146013/original.jpg
GV_1442	40.824	-74.2043	87.9		https://www.google.com/maps/search/40.824,-74.2043	https://data.globe.gov/system/photos/2019/07/24/1146033/original.jpg	https://data.globe.gov/system/photos/2019/07/24/1146035/original.jpg	https://data.globe.gov/system/photos/2019/07/24/1146034/original.jpg	https://data.globe.gov/system/photos/2019/07/24/1146036/original.jpg	https://data.globe.gov/system/photos/2019/07/24/1146038/original.jpg
GV_1443	22.6859	120.3112	16.6		https://www.google.com/maps/search/22.6859,120.3112	https://data.globe.gov/system/photos/2019/07/25/1146090/original.jpg	https://data.globe.gov/system/photos/2019/07/25/1146092/original.jpg	https://data.globe.gov/system/photos/2019/07/25/1146091/original.jpg	https://data.globe.gov/system/photos/2019/07/25/1146093/original.jpg	https://data.globe.gov/system/photos/2019/07/25/1146095/original.jpg
GV_1444	45.3841	-123.7866	50.2		https://www.google.com/maps/search/45.3841,-123.7866	https://data.globe.gov/system/photos/2019/07/25/1146155/original.jpg	https://data.globe.gov/system/photos/2019/07/25/1146157/original.jpg	https://data.globe.gov/system/photos/2019/07/25/1146156/original.jpg	https://data.globe.gov/system/photos/2019/07/25/1146158/original.jpg	https://data.globe.gov/system/photos/2019/07/25/1146160/original.jpg
GV_1445	45.509	-123.8647	12.3		https://www.google.com/maps/search/45.509,-123.8647	https://data.globe.gov/system/photos/2019/07/25/1146161/original.jpg	https://data.globe.gov/system/photos/2019/07/25/1146163/original.jpg	https://data.globe.gov/system/photos/2019/07/25/1146162/original.jpg	https://data.globe.gov/system/photos/2019/07/25/1146164/original.jpg	https://data.globe.gov/system/photos/2019/07/25/1146166/original.jpg
GV_1446	22.6115	120.3043	17.1		https://www.google.com/maps/search/22.6115,120.3043	https://data.globe.gov/system/photos/2019/07/25/1146191/original.jpg	https://data.globe.gov/system/photos/2019/07/25/1146193/original.jpg	https://data.globe.gov/system/photos/2019/07/25/1146192/original.jpg	https://data.globe.gov/system/photos/2019/07/25/1146194/original.jpg	https://data.globe.gov/system/photos/2019/07/25/1146196/original.jpg
GV_1447	22.6105	120.3012	7.2		https://www.google.com/maps/search/22.6105,120.3012	https://data.globe.gov/system/photos/2019/07/25/1146197/original.jpg	https://data.globe.gov/system/photos/2019/07/25/1146199/original.jpg	https://data.globe.gov/system/photos/2019/07/25/1146198/original.jpg	https://data.globe.gov/system/photos/2019/07/25/1146200/original.jpg	https://data.globe.gov/system/photos/2019/07/25/1146202/original.jpg
GV_1448	53.4291	-0.6103	35.4		https://www.google.com/maps/search/53.4291,-0.6103	https://data.globe.gov/system/photos/2019/07/23/1146217/original.jpg	https://data.globe.gov/system/photos/2019/07/23/1146219/original.jpg	https://data.globe.gov/system/photos/2019/07/23/1146218/original.jpg	https://data.globe.gov/system/photos/2019/07/23/1146220/original.jpg	https://data.globe.gov/system/photos/2019/07/23/1146222/original.jpg
GV_1449	53.4283	-0.6227	26		https://www.google.com/maps/search/53.4283,-0.6227	https://data.globe.gov/system/photos/2019/07/24/1146229/original.jpg	https://data.globe.gov/system/photos/2019/07/24/1146231/original.jpg	https://data.globe.gov/system/photos/2019/07/24/1146230/original.jpg	https://data.globe.gov/system/photos/2019/07/24/1146232/original.jpg	https://data.globe.gov/system/photos/2019/07/24/1146234/original.jpg
GV_1450	39.0774	-76.6068	11.4		https://www.google.com/maps/search/39.0774,-76.6068	https://data.globe.gov/system/photos/2019/07/25/1146363/original.jpg	https://data.globe.gov/system/photos/2019/07/25/1146365/original.jpg	https://data.globe.gov/system/photos/2019/07/25/1146364/original.jpg	https://data.globe.gov/system/photos/2019/07/25/1146366/original.jpg	https://data.globe.gov/system/photos/2019/07/25/1146368/original.jpg
GV_1451	36.8164	-121.7329	33.1		https://www.google.com/maps/search/36.8164,-121.7329	https://data.globe.gov/system/photos/2019/07/24/1146556/original.jpg	https://data.globe.gov/system/photos/2019/07/24/1146563/original.jpg	https://data.globe.gov/system/photos/2019/07/24/1146560/original.jpg	https://data.globe.gov/system/photos/2019/07/24/1146566/original.jpg	https://data.globe.gov/system/photos/2019/07/24/1146569/original.jpg
GV_1452	36.8139	-121.7291	5.6		https://www.google.com/maps/search/36.8139,-121.7291	https://data.globe.gov/system/photos/2019/07/24/1146570/original.jpg	https://data.globe.gov/system/photos/2019/07/24/1146572/original.jpg	https://data.globe.gov/system/photos/2019/07/24/1146571/original.jpg	https://data.globe.gov/system/photos/2019/07/24/1146573/original.jpg	https://data.globe.gov/system/photos/2019/07/24/1146578/original.jpg
GV_1453	36.8138	-121.7289	5.4		https://www.google.com/maps/search/36.8138,-121.7289	https://data.globe.gov/system/photos/2019/07/24/1146580/original.jpg	https://data.globe.gov/system/photos/2019/07/24/1146586/original.jpg	https://data.globe.gov/system/photos/2019/07/24/1146581/original.jpg	https://data.globe.gov/system/photos/2019/07/24/1146587/original.jpg	https://data.globe.gov/system/photos/2019/07/24/1146589/original.jpg
GV_1454	36.8138	-121.7287	5.7		https://www.google.com/maps/search/36.8138,-121.7287	https://data.globe.gov/system/photos/2019/07/24/1146590/original.jpg	https://data.globe.gov/system/photos/2019/07/24/1146592/original.jpg	https://data.globe.gov/system/photos/2019/07/24/1146591/original.jpg	https://data.globe.gov/system/photos/2019/07/24/1146593/original.jpg	https://data.globe.gov/system/photos/2019/07/24/1146595/original.jpg
GV_1455	36.8138	-121.7289	5.4		https://www.google.com/maps/search/36.8138,-121.7289	https://data.globe.gov/system/photos/2019/07/24/1146608/original.jpg	https://data.globe.gov/system/photos/2019/07/24/1146610/original.jpg	https://data.globe.gov/system/photos/2019/07/24/1146609/original.jpg	https://data.globe.gov/system/photos/2019/07/24/1146611/original.jpg	https://data.globe.gov/system/photos/2019/07/24/1146613/original.jpg
GV_1456	36.8164	-121.7332	30.5		https://www.google.com/maps/search/36.8164,-121.7332	https://data.globe.gov/system/photos/2019/07/24/1146614/original.jpg	https://data.globe.gov/system/photos/2019/07/24/1146616/original.jpg	https://data.globe.gov/system/photos/2019/07/24/1146615/original.jpg	https://data.globe.gov/system/photos/2019/07/24/1146617/original.jpg	https://data.globe.gov/system/photos/2019/07/24/1146619/original.jpg
GV_1457	36.8163	-121.7331	31.8		https://www.google.com/maps/search/36.8163,-121.7331	https://data.globe.gov/system/photos/2019/07/24/1146620/original.jpg	https://data.globe.gov/system/photos/2019/07/24/1146622/original.jpg	https://data.globe.gov/system/photos/2019/07/24/1146621/original.jpg	https://data.globe.gov/system/photos/2019/07/24/1146623/original.jpg	https://data.globe.gov/system/photos/2019/07/24/1146625/original.jpg
GV_1458	36.8166	-121.733	33.1		https://www.google.com/maps/search/36.8166,-121.733	https://data.globe.gov/system/photos/2019/07/24/1146626/original.jpg	https://data.globe.gov/system/photos/2019/07/24/1146628/original.jpg	https://data.globe.gov/system/photos/2019/07/24/1146627/original.jpg	https://data.globe.gov/system/photos/2019/07/24/1146629/original.jpg	https://data.globe.gov/system/photos/2019/07/24/1146631/original.jpg
GV_1459	36.8167	-121.7329	33.3		https://www.google.com/maps/search/36.8167,-121.7329	https://data.globe.gov/system/photos/2019/07/24/1146632/original.jpg	https://data.globe.gov/system/photos/2019/07/24/1146634/original.jpg	https://data.globe.gov/system/photos/2019/07/24/1146633/original.jpg	https://data.globe.gov/system/photos/2019/07/24/1146635/original.jpg	https://data.globe.gov/system/photos/2019/07/24/1146637/original.jpg
GV_1460	36.8166	-121.7327	33.9		https://www.google.com/maps/search/36.8166,-121.7327	https://data.globe.gov/system/photos/2019/07/24/1146638/original.jpg	https://data.globe.gov/system/photos/2019/07/24/1146640/original.jpg	https://data.globe.gov/system/photos/2019/07/24/1146639/original.jpg	https://data.globe.gov/system/photos/2019/07/24/1146641/original.jpg	https://data.globe.gov/system/photos/2019/07/24/1146643/original.jpg
GV_1461	36.8207	-121.7298	4.6		https://www.google.com/maps/search/36.8207,-121.7298	https://data.globe.gov/system/photos/2019/07/24/1146644/original.jpg	https://data.globe.gov/system/photos/2019/07/24/1146646/original.jpg	https://data.globe.gov/system/photos/2019/07/24/1146645/original.jpg	https://data.globe.gov/system/photos/2019/07/24/1146647/original.jpg	https://data.globe.gov/system/photos/2019/07/24/1146650/original.jpg
GV_1462	42.3385	-82.9792	176.1		https://www.google.com/maps/search/42.3385,-82.9792	https://data.globe.gov/system/photos/2019/07/16/1146848/original.jpg	https://data.globe.gov/system/photos/2019/07/16/1146857/original.jpg	https://data.globe.gov/system/photos/2019/07/16/1146849/original.jpg	https://data.globe.gov/system/photos/2019/07/16/1146858/original.jpg	https://data.globe.gov/system/photos/2019/07/16/1146860/original.jpg
GV_1463	47.0754	-114.7674	872.2		https://www.google.com/maps/search/47.0754,-114.7674	https://data.globe.gov/system/photos/2019/07/25/1146867/original.jpg	https://data.globe.gov/system/photos/2019/07/25/1146869/original.jpg	https://data.globe.gov/system/photos/2019/07/25/1146868/original.jpg	https://data.globe.gov/system/photos/2019/07/25/1146870/original.jpg	https://data.globe.gov/system/photos/2019/07/25/1146872/original.jpg
GV_1464	46.8721	-113.9939	977		https://www.google.com/maps/search/46.8721,-113.9939	https://data.globe.gov/system/photos/2019/07/25/1146873/original.jpg	https://data.globe.gov/system/photos/2019/07/25/1146875/original.jpg	https://data.globe.gov/system/photos/2019/07/25/1146874/original.jpg	https://data.globe.gov/system/photos/2019/07/25/1146876/original.jpg	https://data.globe.gov/system/photos/2019/07/25/1146878/original.jpg
GV_1465	46.8718	-113.9924	977.2		https://www.google.com/maps/search/46.8718,-113.9924	https://data.globe.gov/system/photos/2019/07/25/1146879/original.jpg	https://data.globe.gov/system/photos/2019/07/25/1146881/original.jpg	https://data.globe.gov/system/photos/2019/07/25/1146880/original.jpg	https://data.globe.gov/system/photos/2019/07/25/1146882/original.jpg	https://data.globe.gov/system/photos/2019/07/25/1146884/original.jpg
GV_1466	47.4552	-115.6973	1453.9		https://www.google.com/maps/search/47.4552,-115.6973	https://data.globe.gov/system/photos/2019/07/25/1146927/original.jpg	https://data.globe.gov/system/photos/2019/07/25/1146929/original.jpg	https://data.globe.gov/system/photos/2019/07/25/1146928/original.jpg	https://data.globe.gov/system/photos/2019/07/25/1146930/original.jpg	https://data.globe.gov/system/photos/2019/07/25/1146932/original.jpg
GV_1467	47.4559	-115.6984	1466.4		https://www.google.com/maps/search/47.4559,-115.6984	https://data.globe.gov/system/photos/2019/07/25/1146933/original.jpg	https://data.globe.gov/system/photos/2019/07/25/1146935/original.jpg	https://data.globe.gov/system/photos/2019/07/25/1146934/original.jpg	https://data.globe.gov/system/photos/2019/07/25/1146936/original.jpg	https://data.globe.gov/system/photos/2019/07/25/1146938/original.jpg
GV_1468	47.2955	-115.1697	826.1		https://www.google.com/maps/search/47.2955,-115.1697	https://data.globe.gov/system/photos/2019/07/25/1146939/original.jpg	https://data.globe.gov/system/photos/2019/07/25/1146941/original.jpg	https://data.globe.gov/system/photos/2019/07/25/1146940/original.jpg	https://data.globe.gov/system/photos/2019/07/25/1146942/original.jpg	https://data.globe.gov/system/photos/2019/07/25/1146944/original.jpg
GV_1469	47.0754	-114.7672	872.9		https://www.google.com/maps/search/47.0754,-114.7672	https://data.globe.gov/system/photos/2019/07/25/1146945/original.jpg	https://data.globe.gov/system/photos/2019/07/25/1146947/original.jpg	https://data.globe.gov/system/photos/2019/07/25/1146946/original.jpg	https://data.globe.gov/system/photos/2019/07/25/1146948/original.jpg	https://data.globe.gov/system/photos/2019/07/25/1146950/original.jpg
GV_1470	47.8048	-110.6796	800.4		https://www.google.com/maps/search/47.8048,-110.6796	https://data.globe.gov/system/photos/2019/07/26/1147157/original.jpg	https://data.globe.gov/system/photos/2019/07/26/1147159/original.jpg	https://data.globe.gov/system/photos/2019/07/26/1147158/original.jpg	https://data.globe.gov/system/photos/2019/07/26/1147160/original.jpg	https://data.globe.gov/system/photos/2019/07/26/1147162/original.jpg
GV_1471	47.289	-118.0341	609.5		https://www.google.com/maps/search/47.289,-118.0341	https://data.globe.gov/system/photos/2019/07/25/1147205/original.jpg	https://data.globe.gov/system/photos/2019/07/25/1147207/original.jpg	https://data.globe.gov/system/photos/2019/07/25/1147206/original.jpg	https://data.globe.gov/system/photos/2019/07/25/1147208/original.jpg	https://data.globe.gov/system/photos/2019/07/25/1147210/original.jpg
GV_1472	47.289	-118.0343	610		https://www.google.com/maps/search/47.289,-118.0343	https://data.globe.gov/system/photos/2019/07/25/1147211/original.jpg	https://data.globe.gov/system/photos/2019/07/25/1147213/original.jpg	https://data.globe.gov/system/photos/2019/07/25/1147212/original.jpg	https://data.globe.gov/system/photos/2019/07/25/1147214/original.jpg	https://data.globe.gov/system/photos/2019/07/25/1147216/original.jpg
GV_1473	47.6534	-117.4345	579.2		https://www.google.com/maps/search/47.6534,-117.4345	https://data.globe.gov/system/photos/2019/07/25/1147217/original.jpg	https://data.globe.gov/system/photos/2019/07/25/1147219/original.jpg	https://data.globe.gov/system/photos/2019/07/25/1147218/original.jpg	https://data.globe.gov/system/photos/2019/07/25/1147220/original.jpg	https://data.globe.gov/system/photos/2019/07/25/1147222/original.jpg
GV_1474	47.4535	-115.6955	1429.1		https://www.google.com/maps/search/47.4535,-115.6955	https://data.globe.gov/system/photos/2019/07/25/1147223/original.jpg	https://data.globe.gov/system/photos/2019/07/25/1147225/original.jpg	https://data.globe.gov/system/photos/2019/07/25/1147224/original.jpg	https://data.globe.gov/system/photos/2019/07/25/1147226/original.jpg	https://data.globe.gov/system/photos/2019/07/25/1147234/original.jpg
GV_1475	47.456	-115.6966	1444.1		https://www.google.com/maps/search/47.456,-115.6966	https://data.globe.gov/system/photos/2019/07/25/1147235/original.jpg	https://data.globe.gov/system/photos/2019/07/25/1147237/original.jpg	https://data.globe.gov/system/photos/2019/07/25/1147236/original.jpg	https://data.globe.gov/system/photos/2019/07/25/1147238/original.jpg	https://data.globe.gov/system/photos/2019/07/25/1147240/original.jpg
GV_1476	47.8056	-110.6818	803		https://www.google.com/maps/search/47.8056,-110.6818	https://data.globe.gov/system/photos/2019/07/26/1147241/original.jpg	https://data.globe.gov/system/photos/2019/07/26/1147249/original.jpg	https://data.globe.gov/system/photos/2019/07/26/1147248/original.jpg	https://data.globe.gov/system/photos/2019/07/26/1147250/original.jpg	https://data.globe.gov/system/photos/2019/07/26/1147252/original.jpg
GV_1477	47.805	-110.6792	803.2		https://www.google.com/maps/search/47.805,-110.6792	https://data.globe.gov/system/photos/2019/07/26/1147257/original.jpg	https://data.globe.gov/system/photos/2019/07/26/1147272/original.jpg	https://data.globe.gov/system/photos/2019/07/26/1147270/original.jpg	https://data.globe.gov/system/photos/2019/07/26/1147278/original.jpg	https://data.globe.gov/system/photos/2019/07/26/1147281/original.jpg
GV_1478	47.8048	-110.6799	802.5		https://www.google.com/maps/search/47.8048,-110.6799	https://data.globe.gov/system/photos/2019/07/26/1147282/original.jpg	https://data.globe.gov/system/photos/2019/07/26/1147284/original.jpg	https://data.globe.gov/system/photos/2019/07/26/1147283/original.jpg	https://data.globe.gov/system/photos/2019/07/26/1147285/original.jpg	https://data.globe.gov/system/photos/2019/07/26/1147288/original.jpg
GV_1479	47.8049	-110.6796	802.6		https://www.google.com/maps/search/47.8049,-110.6796	https://data.globe.gov/system/photos/2019/07/26/1147292/original.jpg	https://data.globe.gov/system/photos/2019/07/26/1147294/original.jpg	https://data.globe.gov/system/photos/2019/07/26/1147293/original.jpg	https://data.globe.gov/system/photos/2019/07/26/1147295/original.jpg	https://data.globe.gov/system/photos/2019/07/26/1147303/original.jpg
GV_1480	47.8051	-110.6806	803.8		https://www.google.com/maps/search/47.8051,-110.6806	https://data.globe.gov/system/photos/2019/07/26/1147304/original.jpg	https://data.globe.gov/system/photos/2019/07/26/1147306/original.jpg	https://data.globe.gov/system/photos/2019/07/26/1147305/original.jpg	https://data.globe.gov/system/photos/2019/07/26/1147307/original.jpg	https://data.globe.gov/system/photos/2019/07/26/1147309/original.jpg
GV_1481	47.8052	-110.6783	801.3		https://www.google.com/maps/search/47.8052,-110.6783	https://data.globe.gov/system/photos/2019/07/26/1147310/original.jpg	https://data.globe.gov/system/photos/2019/07/26/1147312/original.jpg	https://data.globe.gov/system/photos/2019/07/26/1147311/original.jpg	https://data.globe.gov/system/photos/2019/07/26/1147313/original.jpg	https://data.globe.gov/system/photos/2019/07/26/1147315/original.jpg
GV_1482	47.8056	-110.6779	804.6		https://www.google.com/maps/search/47.8056,-110.6779	https://data.globe.gov/system/photos/2019/07/26/1147334/original.jpg	https://data.globe.gov/system/photos/2019/07/26/1147336/original.jpg	https://data.globe.gov/system/photos/2019/07/26/1147335/original.jpg	https://data.globe.gov/system/photos/2019/07/26/1147337/original.jpg	https://data.globe.gov/system/photos/2019/07/26/1147339/original.jpg
GV_1483	47.8055	-110.6777	802.2		https://www.google.com/maps/search/47.8055,-110.6777	https://data.globe.gov/system/photos/2019/07/26/1147341/original.jpg	https://data.globe.gov/system/photos/2019/07/26/1147348/original.jpg	https://data.globe.gov/system/photos/2019/07/26/1147346/original.jpg	https://data.globe.gov/system/photos/2019/07/26/1147349/original.jpg	https://data.globe.gov/system/photos/2019/07/26/1147351/original.jpg
GV_1484	47.8102	-110.6732	802.2		https://www.google.com/maps/search/47.8102,-110.6732	https://data.globe.gov/system/photos/2019/07/26/1147352/original.jpg	https://data.globe.gov/system/photos/2019/07/26/1147354/original.jpg	https://data.globe.gov/system/photos/2019/07/26/1147353/original.jpg	https://data.globe.gov/system/photos/2019/07/26/1147355/original.jpg	https://data.globe.gov/system/photos/2019/07/26/1147357/original.jpg
GV_1485	47.8107	-110.6719	802		https://www.google.com/maps/search/47.8107,-110.6719	https://data.globe.gov/system/photos/2019/07/26/1147364/original.jpg	https://data.globe.gov/system/photos/2019/07/26/1147366/original.jpg	https://data.globe.gov/system/photos/2019/07/26/1147365/original.jpg	https://data.globe.gov/system/photos/2019/07/26/1147367/original.jpg	https://data.globe.gov/system/photos/2019/07/26/1147369/original.jpg
GV_1486	47.8108	-110.6723	802		https://www.google.com/maps/search/47.8108,-110.6723	https://data.globe.gov/system/photos/2019/07/26/1147370/original.jpg	https://data.globe.gov/system/photos/2019/07/26/1147372/original.jpg	https://data.globe.gov/system/photos/2019/07/26/1147371/original.jpg	https://data.globe.gov/system/photos/2019/07/26/1147373/original.jpg	https://data.globe.gov/system/photos/2019/07/26/1147375/original.jpg
GV_1487	22.6408	120.3213	9		https://www.google.com/maps/search/22.6408,120.3213	https://data.globe.gov/system/photos/2019/07/26/1147557/original.jpg	https://data.globe.gov/system/photos/2019/07/26/1147559/original.jpg	https://data.globe.gov/system/photos/2019/07/26/1147558/original.jpg	https://data.globe.gov/system/photos/2019/07/26/1147560/original.jpg	https://data.globe.gov/system/photos/2019/07/26/1147562/original.jpg
GV_1488	22.6406	120.3225	9.5		https://www.google.com/maps/search/22.6406,120.3225	https://data.globe.gov/system/photos/2019/07/26/1147648/original.jpg	https://data.globe.gov/system/photos/2019/07/26/1147650/original.jpg	https://data.globe.gov/system/photos/2019/07/26/1147649/original.jpg	https://data.globe.gov/system/photos/2019/07/26/1147651/original.jpg	https://data.globe.gov/system/photos/2019/07/26/1147653/original.jpg
GV_1489	53.4284	-0.6226	26		https://www.google.com/maps/search/53.4284,-0.6226	https://data.globe.gov/system/photos/2019/07/25/1147921/original.jpg	https://data.globe.gov/system/photos/2019/07/25/1147923/original.jpg	https://data.globe.gov/system/photos/2019/07/25/1147922/original.jpg	https://data.globe.gov/system/photos/2019/07/25/1147924/original.jpg	https://data.globe.gov/system/photos/2019/07/25/1147926/original.jpg
GV_1490	31.8804	-106.6023	1146.4		https://www.google.com/maps/search/31.8804,-106.6023	https://data.globe.gov/system/photos/2019/07/26/1148175/original.jpg	https://data.globe.gov/system/photos/2019/07/26/1148177/original.jpg	https://data.globe.gov/system/photos/2019/07/26/1148176/original.jpg	https://data.globe.gov/system/photos/2019/07/26/1148179/original.jpg	https://data.globe.gov/system/photos/2019/07/26/1148182/original.jpg
GV_1491	31.8731	-106.5725	1203.4		https://www.google.com/maps/search/31.8731,-106.5725	https://data.globe.gov/system/photos/2019/07/26/1148237/original.jpg	https://data.globe.gov/system/photos/2019/07/26/1148239/original.jpg	https://data.globe.gov/system/photos/2019/07/26/1148238/original.jpg	https://data.globe.gov/system/photos/2019/07/26/1148240/original.jpg	https://data.globe.gov/system/photos/2019/07/26/1148253/original.jpg
GV_1492	31.8682	-106.5624	1217.9		https://www.google.com/maps/search/31.8682,-106.5624	https://data.globe.gov/system/photos/2019/07/26/1148269/original.jpg	https://data.globe.gov/system/photos/2019/07/26/1148271/original.jpg	https://data.globe.gov/system/photos/2019/07/26/1148270/original.jpg	https://data.globe.gov/system/photos/2019/07/26/1148272/original.jpg	https://data.globe.gov/system/photos/2019/07/26/1148274/original.jpg
GV_1493	36.8179	-121.7311	28		https://www.google.com/maps/search/36.8179,-121.7311	https://data.globe.gov/system/photos/2019/07/24/1148381/original.jpg	https://data.globe.gov/system/photos/2019/07/24/1148383/original.jpg	https://data.globe.gov/system/photos/2019/07/24/1148382/original.jpg	https://data.globe.gov/system/photos/2019/07/24/1148384/original.jpg	https://data.globe.gov/system/photos/2019/07/24/1148386/original.jpg
GV_1494	36.8179	-121.731	28.6		https://www.google.com/maps/search/36.8179,-121.731	https://data.globe.gov/system/photos/2019/07/24/1148387/original.jpg	https://data.globe.gov/system/photos/2019/07/24/1148389/original.jpg	https://data.globe.gov/system/photos/2019/07/24/1148388/original.jpg	https://data.globe.gov/system/photos/2019/07/24/1148390/original.jpg	https://data.globe.gov/system/photos/2019/07/24/1148392/original.jpg
GV_1495	36.8181	-121.731	26.4		https://www.google.com/maps/search/36.8181,-121.731	https://data.globe.gov/system/photos/2019/07/24/1148399/original.jpg	https://data.globe.gov/system/photos/2019/07/24/1148401/original.jpg	https://data.globe.gov/system/photos/2019/07/24/1148400/original.jpg	https://data.globe.gov/system/photos/2019/07/24/1148402/original.jpg	https://data.globe.gov/system/photos/2019/07/24/1148404/original.jpg
GV_1496	38.0788	127.2184	112		https://www.google.com/maps/search/38.0788,127.2184	https://data.globe.gov/system/photos/2019/07/27/1148610/original.jpg	https://data.globe.gov/system/photos/2019/07/27/1148612/original.jpg	https://data.globe.gov/system/photos/2019/07/27/1148611/original.jpg	https://data.globe.gov/system/photos/2019/07/27/1148613/original.jpg	https://data.globe.gov/system/photos/2019/07/27/1148615/original.jpg
GV_1497	47.4578	8.9692	573		https://www.google.com/maps/search/47.4578,8.9692	https://data.globe.gov/system/photos/2019/07/27/1148707/original.jpg	https://data.globe.gov/system/photos/2019/07/27/1148709/original.jpg	https://data.globe.gov/system/photos/2019/07/27/1148708/original.jpg	https://data.globe.gov/system/photos/2019/07/27/1148710/original.jpg	https://data.globe.gov/system/photos/2019/07/27/1148712/original.jpg
GV_1498	53.4141	-0.6052	38		https://www.google.com/maps/search/53.4141,-0.6052	https://data.globe.gov/system/photos/2019/07/26/1148742/original.jpg	https://data.globe.gov/system/photos/2019/07/26/1148744/original.jpg	https://data.globe.gov/system/photos/2019/07/26/1148743/original.jpg	https://data.globe.gov/system/photos/2019/07/26/1148745/original.jpg	https://data.globe.gov/system/photos/2019/07/26/1148747/original.jpg
GV_1499	53.4265	-0.6297	25		https://www.google.com/maps/search/53.4265,-0.6297	https://data.globe.gov/system/photos/2019/07/27/1148772/original.jpg	https://data.globe.gov/system/photos/2019/07/27/1148774/original.jpg	https://data.globe.gov/system/photos/2019/07/27/1148773/original.jpg	https://data.globe.gov/system/photos/2019/07/27/1148775/original.jpg	https://data.globe.gov/system/photos/2019/07/27/1148777/original.jpg
GV_1500	40.3115	-75.9263	64.9		https://www.google.com/maps/search/40.3115,-75.9263	https://data.globe.gov/system/photos/2019/07/27/1148848/original.jpg	https://data.globe.gov/system/photos/2019/07/27/1148850/original.jpg	https://data.globe.gov/system/photos/2019/07/27/1148849/original.jpg	https://data.globe.gov/system/photos/2019/07/27/1148851/original.jpg	https://data.globe.gov/system/photos/2019/07/27/1148853/original.jpg
GV_1501	44.0015	-71.5356	1317.7		https://www.google.com/maps/search/44.0015,-71.5356	https://data.globe.gov/system/photos/2019/07/27/1149136/original.jpg	https://data.globe.gov/system/photos/2019/07/27/1149138/original.jpg	https://data.globe.gov/system/photos/2019/07/27/1149137/original.jpg	https://data.globe.gov/system/photos/2019/07/27/1149139/original.jpg	https://data.globe.gov/system/photos/2019/07/27/1149147/original.jpg
GV_1502	53.4266	-0.6296	24.9		https://www.google.com/maps/search/53.4266,-0.6296	https://data.globe.gov/system/photos/2019/07/28/1149329/original.jpg	https://data.globe.gov/system/photos/2019/07/28/1149331/original.jpg	https://data.globe.gov/system/photos/2019/07/28/1149330/original.jpg	https://data.globe.gov/system/photos/2019/07/28/1149332/original.jpg	https://data.globe.gov/system/photos/2019/07/28/1149334/original.jpg
GV_1503	24.4355	118.3139	22		https://www.google.com/maps/search/24.4355,118.3139	https://data.globe.gov/system/photos/2019/07/29/1149975/original.jpg	https://data.globe.gov/system/photos/2019/07/29/1149977/original.jpg	https://data.globe.gov/system/photos/2019/07/29/1149976/original.jpg	https://data.globe.gov/system/photos/2019/07/29/1149978/original.jpg	https://data.globe.gov/system/photos/2019/07/29/1149980/original.jpg
GV_1504	38.8933	-77.034	3.1		https://www.google.com/maps/search/38.8933,-77.034	https://data.globe.gov/system/photos/2019/07/29/1150140/original.jpg	https://data.globe.gov/system/photos/2019/07/29/1150142/original.jpg	https://data.globe.gov/system/photos/2019/07/29/1150141/original.jpg	https://data.globe.gov/system/photos/2019/07/29/1150143/original.jpg	https://data.globe.gov/system/photos/2019/07/29/1150145/original.jpg
GV_1505	39.8309	-77.239	158.7		https://www.google.com/maps/search/39.8309,-77.239	https://data.globe.gov/system/photos/2019/07/29/1150252/original.jpg	https://data.globe.gov/system/photos/2019/07/29/1150254/original.jpg	https://data.globe.gov/system/photos/2019/07/29/1150253/original.jpg	https://data.globe.gov/system/photos/2019/07/29/1150255/original.jpg	https://data.globe.gov/system/photos/2019/07/29/1150257/original.jpg
GV_1506	35.8203	-106.5348	2558.2		https://www.google.com/maps/search/35.8203,-106.5348	https://data.globe.gov/system/photos/2019/07/28/1150565/original.jpg	https://data.globe.gov/system/photos/2019/07/28/1150567/original.jpg	https://data.globe.gov/system/photos/2019/07/28/1150566/original.jpg	https://data.globe.gov/system/photos/2019/07/28/1150568/original.jpg	https://data.globe.gov/system/photos/2019/07/28/1150570/original.jpg
GV_1507	35.8218	-106.5404	2545.2		https://www.google.com/maps/search/35.8218,-106.5404	https://data.globe.gov/system/photos/2019/07/28/1150571/original.jpg	https://data.globe.gov/system/photos/2019/07/28/1150573/original.jpg	https://data.globe.gov/system/photos/2019/07/28/1150572/original.jpg	https://data.globe.gov/system/photos/2019/07/28/1150574/original.jpg	https://data.globe.gov/system/photos/2019/07/28/1150576/original.jpg
GV_1508	35.822	-106.5477	2539.7		https://www.google.com/maps/search/35.822,-106.5477	https://data.globe.gov/system/photos/2019/07/28/1150577/original.jpg	https://data.globe.gov/system/photos/2019/07/28/1150579/original.jpg	https://data.globe.gov/system/photos/2019/07/28/1150578/original.jpg	https://data.globe.gov/system/photos/2019/07/28/1150580/original.jpg	https://data.globe.gov/system/photos/2019/07/28/1150582/original.jpg
GV_1509	35.3087	-106.5771	1552.2		https://www.google.com/maps/search/35.3087,-106.5771	https://data.globe.gov/system/photos/2019/07/29/1150588/original.jpg	https://data.globe.gov/system/photos/2019/07/29/1150590/original.jpg	https://data.globe.gov/system/photos/2019/07/29/1150589/original.jpg	https://data.globe.gov/system/photos/2019/07/29/1150591/original.jpg	https://data.globe.gov/system/photos/2019/07/29/1150593/original.jpg
GV_1510	34.2367	-118.528	260.2		https://www.google.com/maps/search/34.2367,-118.528	https://data.globe.gov/system/photos/2019/07/25/1150698/original.jpg	https://data.globe.gov/system/photos/2019/07/25/1150700/original.jpg	https://data.globe.gov/system/photos/2019/07/25/1150699/original.jpg	https://data.globe.gov/system/photos/2019/07/25/1150701/original.jpg	https://data.globe.gov/system/photos/2019/07/25/1150703/original.jpg
GV_1511	34.2379	-118.5281	261.4		https://www.google.com/maps/search/34.2379,-118.5281	https://data.globe.gov/system/photos/2019/07/29/1150704/original.jpg	https://data.globe.gov/system/photos/2019/07/29/1150706/original.jpg	https://data.globe.gov/system/photos/2019/07/29/1150705/original.jpg	https://data.globe.gov/system/photos/2019/07/29/1150707/original.jpg	https://data.globe.gov/system/photos/2019/07/29/1150709/original.jpg
GV_1512	34.237	-118.5274	260.1		https://www.google.com/maps/search/34.237,-118.5274	https://data.globe.gov/system/photos/2019/07/29/1150716/original.jpg	https://data.globe.gov/system/photos/2019/07/29/1150718/original.jpg	https://data.globe.gov/system/photos/2019/07/29/1150717/original.jpg	https://data.globe.gov/system/photos/2019/07/29/1150719/original.jpg	https://data.globe.gov/system/photos/2019/07/29/1150721/original.jpg
GV_1513	47.5293	-111.2357	1012.1		https://www.google.com/maps/search/47.5293,-111.2357	https://data.globe.gov/system/photos/2019/07/25/1150729/original.jpg	https://data.globe.gov/system/photos/2019/07/25/1150731/original.jpg	https://data.globe.gov/system/photos/2019/07/25/1150730/original.jpg	https://data.globe.gov/system/photos/2019/07/25/1150732/original.jpg	https://data.globe.gov/system/photos/2019/07/25/1150734/original.jpg
GV_1514	47.5295	-111.2355	1011.2		https://www.google.com/maps/search/47.5295,-111.2355	https://data.globe.gov/system/photos/2019/07/25/1150735/original.jpg	https://data.globe.gov/system/photos/2019/07/25/1150737/original.jpg	https://data.globe.gov/system/photos/2019/07/25/1150736/original.jpg	https://data.globe.gov/system/photos/2019/07/25/1150738/original.jpg	https://data.globe.gov/system/photos/2019/07/25/1150740/original.jpg
GV_1515	47.5105	-111.3029	1012.9		https://www.google.com/maps/search/47.5105,-111.3029	https://data.globe.gov/system/photos/2019/07/25/1150760/original.jpg	https://data.globe.gov/system/photos/2019/07/25/1150762/original.jpg	https://data.globe.gov/system/photos/2019/07/25/1150761/original.jpg	https://data.globe.gov/system/photos/2019/07/25/1150763/original.jpg	https://data.globe.gov/system/photos/2019/07/25/1150765/original.jpg
GV_1516	47.5119	-111.3018	1012.4		https://www.google.com/maps/search/47.5119,-111.3018	https://data.globe.gov/system/photos/2019/07/25/1150766/original.jpg	https://data.globe.gov/system/photos/2019/07/25/1150768/original.jpg	https://data.globe.gov/system/photos/2019/07/25/1150767/original.jpg	https://data.globe.gov/system/photos/2019/07/25/1150769/original.jpg	https://data.globe.gov/system/photos/2019/07/25/1150771/original.jpg
GV_1517	47.8186	-110.6664	802.3		https://www.google.com/maps/search/47.8186,-110.6664	https://data.globe.gov/system/photos/2019/07/26/1150772/original.jpg	https://data.globe.gov/system/photos/2019/07/26/1150774/original.jpg	https://data.globe.gov/system/photos/2019/07/26/1150773/original.jpg	https://data.globe.gov/system/photos/2019/07/26/1150775/original.jpg	https://data.globe.gov/system/photos/2019/07/26/1150777/original.jpg
GV_1518	47.6802	-110.3448	976.6		https://www.google.com/maps/search/47.6802,-110.3448	https://data.globe.gov/system/photos/2019/07/29/1150778/original.jpg	https://data.globe.gov/system/photos/2019/07/29/1150780/original.jpg	https://data.globe.gov/system/photos/2019/07/29/1150779/original.jpg	https://data.globe.gov/system/photos/2019/07/29/1150781/original.jpg	https://data.globe.gov/system/photos/2019/07/29/1150783/original.jpg
GV_1519	47.9139	-110.0584	757		https://www.google.com/maps/search/47.9139,-110.0584	https://data.globe.gov/system/photos/2019/07/26/1150839/original.jpg	https://data.globe.gov/system/photos/2019/07/26/1150841/original.jpg	https://data.globe.gov/system/photos/2019/07/26/1150840/original.jpg	https://data.globe.gov/system/photos/2019/07/26/1150842/original.jpg	https://data.globe.gov/system/photos/2019/07/26/1150844/original.jpg
GV_1520	48.03	-110.2164	764		https://www.google.com/maps/search/48.03,-110.2164	https://data.globe.gov/system/photos/2019/07/26/1150848/original.jpg	https://data.globe.gov/system/photos/2019/07/26/1150850/original.jpg	https://data.globe.gov/system/photos/2019/07/26/1150849/original.jpg	https://data.globe.gov/system/photos/2019/07/26/1150851/original.jpg	https://data.globe.gov/system/photos/2019/07/26/1150853/original.jpg
GV_1521	48.0269	-110.1335	762		https://www.google.com/maps/search/48.0269,-110.1335	https://data.globe.gov/system/photos/2019/07/26/1150854/original.jpg	https://data.globe.gov/system/photos/2019/07/26/1150856/original.jpg	https://data.globe.gov/system/photos/2019/07/26/1150855/original.jpg	https://data.globe.gov/system/photos/2019/07/26/1150857/original.jpg	https://data.globe.gov/system/photos/2019/07/26/1150859/original.jpg
GV_1522	38.0485	-122.1596	6.2		https://www.google.com/maps/search/38.0485,-122.1596	https://data.globe.gov/system/photos/2019/07/30/1151147/original.jpg	https://data.globe.gov/system/photos/2019/07/30/1151149/original.jpg	https://data.globe.gov/system/photos/2019/07/30/1151148/original.jpg	https://data.globe.gov/system/photos/2019/07/30/1151150/original.jpg	https://data.globe.gov/system/photos/2019/07/30/1151152/original.jpg
GV_1523	60.5078	-150.5057	124.4		https://www.google.com/maps/search/60.5078,-150.5057	https://data.globe.gov/system/photos/2019/07/28/1151434/original.jpg	https://data.globe.gov/system/photos/2019/07/28/1151436/original.jpg	https://data.globe.gov/system/photos/2019/07/28/1151435/original.jpg	https://data.globe.gov/system/photos/2019/07/28/1151437/original.jpg	https://data.globe.gov/system/photos/2019/07/28/1151440/original.jpg

GV_1524	35.308	-106.5772	1551.8		https://www.google.com/maps/search/35.308,-106.5772	https://data.globe.gov/system/photos/2019/07/30/1151650/original.jpg	https://data.globe.gov/system/photos/2019/07/30/1151652/original.jpg	https://data.globe.gov/system/photos/2019/07/30/1151651/original.jpg	https://data.globe.gov/system/photos/2019/07/30/1151653/original.jpg	https://data.globe.gov/system/photos/2019/07/30/1151656/original.jpg
GV_1525	46.1337	-112.924	1583.4		https://www.google.com/maps/search/46.1337,-112.924	https://data.globe.gov/system/photos/2019/07/28/1151719/original.jpg	https://data.globe.gov/system/photos/2019/07/28/1151721/original.jpg	https://data.globe.gov/system/photos/2019/07/28/1151720/original.jpg	https://data.globe.gov/system/photos/2019/07/28/1151722/original.jpg	https://data.globe.gov/system/photos/2019/07/28/1151724/original.jpg
GV_1526	46.0118	-112.518	1701		https://www.google.com/maps/search/46.0118,-112.518	https://data.globe.gov/system/photos/2019/07/28/1151725/original.jpg	https://data.globe.gov/system/photos/2019/07/28/1151727/original.jpg	https://data.globe.gov/system/photos/2019/07/28/1151726/original.jpg	https://data.globe.gov/system/photos/2019/07/28/1151728/original.jpg	https://data.globe.gov/system/photos/2019/07/28/1151730/original.jpg
GV_1527	-34.4196	138.9258	310.9		https://www.google.com/maps/search/-34.4196,138.9258	https://data.globe.gov/system/photos/2019/07/30/1151752/original.jpg	https://data.globe.gov/system/photos/2019/07/30/1151754/original.jpg	https://data.globe.gov/system/photos/2019/07/30/1151753/original.jpg	https://data.globe.gov/system/photos/2019/07/30/1151755/original.jpg	https://data.globe.gov/system/photos/2019/07/30/1151757/original.jpg
GV_1528	-34.3864	138.9114	252.2		https://www.google.com/maps/search/-34.3864,138.9114	https://data.globe.gov/system/photos/2019/07/30/1151758/original.jpg	https://data.globe.gov/system/photos/2019/07/30/1151760/original.jpg	https://data.globe.gov/system/photos/2019/07/30/1151759/original.jpg	https://data.globe.gov/system/photos/2019/07/30/1151761/original.jpg	https://data.globe.gov/system/photos/2019/07/30/1151763/original.jpg
GV_1529	39.3532	-76.6214	107.8		https://www.google.com/maps/search/39.3532,-76.6214	https://data.globe.gov/system/photos/2019/07/31/1152433/original.jpg	https://data.globe.gov/system/photos/2019/07/31/1152435/original.jpg	https://data.globe.gov/system/photos/2019/07/31/1152434/original.jpg	https://data.globe.gov/system/photos/2019/07/31/1152436/original.jpg	https://data.globe.gov/system/photos/2019/07/31/1152438/original.jpg
GV_1530	39.3535	-76.6206	107.4		https://www.google.com/maps/search/39.3535,-76.6206	https://data.globe.gov/system/photos/2019/07/31/1152439/original.jpg	https://data.globe.gov/system/photos/2019/07/31/1152442/original.jpg	https://data.globe.gov/system/photos/2019/07/31/1152441/original.jpg	https://data.globe.gov/system/photos/2019/07/31/1152443/original.jpg	https://data.globe.gov/system/photos/2019/07/31/1152445/original.jpg
GV_1531	39.3507	-76.6219	107.3		https://www.google.com/maps/search/39.3507,-76.6219	https://data.globe.gov/system/photos/2019/07/31/1152446/original.jpg	https://data.globe.gov/system/photos/2019/07/31/1152448/original.jpg	https://data.globe.gov/system/photos/2019/07/31/1152447/original.jpg	https://data.globe.gov/system/photos/2019/07/31/1152449/original.jpg	https://data.globe.gov/system/photos/2019/07/31/1152451/original.jpg
GV_1532	39.3514	-76.6224	108.5		https://www.google.com/maps/search/39.3514,-76.6224	https://data.globe.gov/system/photos/2019/07/31/1152476/original.jpg	https://data.globe.gov/system/photos/2019/07/31/1152478/original.jpg	https://data.globe.gov/system/photos/2019/07/31/1152477/original.jpg	https://data.globe.gov/system/photos/2019/07/31/1152479/original.jpg	https://data.globe.gov/system/photos/2019/07/31/1152481/original.jpg
GV_1533	39.3511	-76.6222	108.5		https://www.google.com/maps/search/39.3511,-76.6222	https://data.globe.gov/system/photos/2019/07/31/1152496/original.jpg	https://data.globe.gov/system/photos/2019/07/31/1152498/original.jpg	https://data.globe.gov/system/photos/2019/07/31/1152497/original.jpg	https://data.globe.gov/system/photos/2019/07/31/1152499/original.jpg	https://data.globe.gov/system/photos/2019/07/31/1152501/original.jpg
GV_1534	52.1341	-109.79	693.9		https://www.google.com/maps/search/52.1341,-109.79	https://data.globe.gov/system/photos/2019/07/31/1152544/original.jpg	https://data.globe.gov/system/photos/2019/07/31/1152546/original.jpg	https://data.globe.gov/system/photos/2019/07/31/1152545/original.jpg	https://data.globe.gov/system/photos/2019/07/31/1152547/original.jpg	https://data.globe.gov/system/photos/2019/07/31/1152555/original.jpg
GV_1535	45.1499	-122.5671	121.1		https://www.google.com/maps/search/45.1499,-122.5671	https://data.globe.gov/system/photos/2019/07/31/1152619/original.jpg	https://data.globe.gov/system/photos/2019/07/31/1152622/original.jpg	https://data.globe.gov/system/photos/2019/07/31/1152621/original.jpg	https://data.globe.gov/system/photos/2019/07/31/1152624/original.jpg	https://data.globe.gov/system/photos/2019/07/31/1152626/original.jpg
GV_1536	45.1534	-122.5851	107.9		https://www.google.com/maps/search/45.1534,-122.5851	https://data.globe.gov/system/photos/2019/07/31/1152627/original.jpg	https://data.globe.gov/system/photos/2019/07/31/1152629/original.jpg	https://data.globe.gov/system/photos/2019/07/31/1152628/original.jpg	https://data.globe.gov/system/photos/2019/07/31/1152630/original.jpg	https://data.globe.gov/system/photos/2019/07/31/1152632/original.jpg
GV_1537	53.4283	-0.6222	26		https://www.google.com/maps/search/53.4283,-0.6222	https://data.globe.gov/system/photos/2019/07/31/1152657/original.jpg	https://data.globe.gov/system/photos/2019/07/31/1152659/original.jpg	https://data.globe.gov/system/photos/2019/07/31/1152658/original.jpg	https://data.globe.gov/system/photos/2019/07/31/1152660/original.jpg	https://data.globe.gov/system/photos/2019/07/31/1152662/original.jpg
GV_1538	45.0908	-122.6372	117.9		https://www.google.com/maps/search/45.0908,-122.6372	https://data.globe.gov/system/photos/2019/07/19/1152680/original.jpg	https://data.globe.gov/system/photos/2019/07/19/1152682/original.jpg	https://data.globe.gov/system/photos/2019/07/19/1152681/original.jpg	https://data.globe.gov/system/photos/2019/07/19/1152683/original.jpg	https://data.globe.gov/system/photos/2019/07/19/1152685/original.jpg
GV_1539	45.0915	-122.6373	114.9		https://www.google.com/maps/search/45.0915,-122.6373	https://data.globe.gov/system/photos/2019/07/19/1152686/original.jpg	https://data.globe.gov/system/photos/2019/07/19/1152688/original.jpg	https://data.globe.gov/system/photos/2019/07/19/1152687/original.jpg	https://data.globe.gov/system/photos/2019/07/19/1152689/original.jpg	https://data.globe.gov/system/photos/2019/07/19/1152709/original.jpg
GV_1540	47.7368	-109.8724	744.3		https://www.google.com/maps/search/47.7368,-109.8724	https://data.globe.gov/system/photos/2019/07/28/1152733/original.jpg	https://data.globe.gov/system/photos/2019/07/28/1152735/original.jpg	https://data.globe.gov/system/photos/2019/07/28/1152734/original.jpg	https://data.globe.gov/system/photos/2019/07/28/1152736/original.jpg	https://data.globe.gov/system/photos/2019/07/28/1152738/original.jpg
GV_1541	47.7392	-109.6235	735.8		https://www.google.com/maps/search/47.7392,-109.6235	https://data.globe.gov/system/photos/2019/07/29/1152739/original.jpg	https://data.globe.gov/system/photos/2019/07/29/1152741/original.jpg	https://data.globe.gov/system/photos/2019/07/29/1152740/original.jpg	https://data.globe.gov/system/photos/2019/07/29/1152742/original.jpg	https://data.globe.gov/system/photos/2019/07/29/1152744/original.jpg
GV_1542	-34.543	138.9843	275.7		https://www.google.com/maps/search/-34.543,138.9843	https://data.globe.gov/system/photos/2019/08/01/1152894/original.jpg	https://data.globe.gov/system/photos/2019/08/01/1152896/original.jpg	https://data.globe.gov/system/photos/2019/08/01/1152895/original.jpg	https://data.globe.gov/system/photos/2019/08/01/1152897/original.jpg	https://data.globe.gov/system/photos/2019/08/01/1152899/original.jpg
GV_1543	36.3329	-119.6727	68.9		https://www.google.com/maps/search/36.3329,-119.6727	https://data.globe.gov/system/photos/2019/08/01/1153180/original.jpg	https://data.globe.gov/system/photos/2019/08/01/1153182/original.jpg	https://data.globe.gov/system/photos/2019/08/01/1153181/original.jpg	https://data.globe.gov/system/photos/2019/08/01/1153183/original.jpg	https://data.globe.gov/system/photos/2019/08/01/1153185/original.jpg
GV_1544	34.8472	-108.0129	2195.2		https://www.google.com/maps/search/34.8472,-108.0129	https://data.globe.gov/system/photos/2019/08/01/1153306/original.jpg	https://data.globe.gov/system/photos/2019/08/01/1153308/original.jpg	https://data.globe.gov/system/photos/2019/08/01/1153307/original.jpg	https://data.globe.gov/system/photos/2019/08/01/1153309/original.jpg	https://data.globe.gov/system/photos/2019/08/01/1153311/original.jpg
GV_1545	35.1137	-107.7955	1950.3		https://www.google.com/maps/search/35.1137,-107.7955	https://data.globe.gov/system/photos/2019/07/29/1153312/original.jpg	https://data.globe.gov/system/photos/2019/07/29/1153314/original.jpg	https://data.globe.gov/system/photos/2019/07/29/1153313/original.jpg	https://data.globe.gov/system/photos/2019/07/29/1153315/original.jpg	https://data.globe.gov/system/photos/2019/07/29/1153317/original.jpg
GV_1546	35.1677	-107.862	2127.1		https://www.google.com/maps/search/35.1677,-107.862	https://data.globe.gov/system/photos/2019/07/28/1153318/original.jpg	https://data.globe.gov/system/photos/2019/07/28/1153320/original.jpg	https://data.globe.gov/system/photos/2019/07/28/1153319/original.jpg	https://data.globe.gov/system/photos/2019/07/28/1153321/original.jpg	https://data.globe.gov/system/photos/2019/07/28/1153323/original.jpg
GV_1547	48.442	-113.2193	1466.5		https://www.google.com/maps/search/48.442,-113.2193	https://data.globe.gov/system/photos/2019/08/01/1153334/original.jpg	https://data.globe.gov/system/photos/2019/08/01/1153336/original.jpg	https://data.globe.gov/system/photos/2019/08/01/1153335/original.jpg	https://data.globe.gov/system/photos/2019/08/01/1153337/original.jpg	https://data.globe.gov/system/photos/2019/08/01/1153339/original.jpg
GV_1548	48.4423	-113.2196	1465.3		https://www.google.com/maps/search/48.4423,-113.2196	https://data.globe.gov/system/photos/2019/08/01/1153346/original.jpg	https://data.globe.gov/system/photos/2019/08/01/1153348/original.jpg	https://data.globe.gov/system/photos/2019/08/01/1153347/original.jpg	https://data.globe.gov/system/photos/2019/08/01/1153349/original.jpg	https://data.globe.gov/system/photos/2019/08/01/1153351/original.jpg
GV_1549	48.4423	-113.2202	1464.1		https://www.google.com/maps/search/48.4423,-113.2202	https://data.globe.gov/system/photos/2019/08/01/1153352/original.jpg	https://data.globe.gov/system/photos/2019/08/01/1153354/original.jpg	https://data.globe.gov/system/photos/2019/08/01/1153353/original.jpg	https://data.globe.gov/system/photos/2019/08/01/1153355/original.jpg	https://data.globe.gov/system/photos/2019/08/01/1153357/original.jpg
GV_1550	46.9757	-112.5327	1455.5		https://www.google.com/maps/search/46.9757,-112.5327	https://data.globe.gov/system/photos/2019/08/01/1153364/original.jpg	https://data.globe.gov/system/photos/2019/08/01/1153366/original.jpg	https://data.globe.gov/system/photos/2019/08/01/1153365/original.jpg	https://data.globe.gov/system/photos/2019/08/01/1153367/original.jpg	https://data.globe.gov/system/photos/2019/08/01/1153369/original.jpg
GV_1551	46.978	-112.5317	1458.5		https://www.google.com/maps/search/46.978,-112.5317	https://data.globe.gov/system/photos/2019/08/01/1153370/original.jpg	https://data.globe.gov/system/photos/2019/08/01/1153372/original.jpg	https://data.globe.gov/system/photos/2019/08/01/1153371/original.jpg	https://data.globe.gov/system/photos/2019/08/01/1153373/original.jpg	https://data.globe.gov/system/photos/2019/08/01/1153375/original.jpg
GV_1552	46.9782	-112.5334	1456		https://www.google.com/maps/search/46.9782,-112.5334	https://data.globe.gov/system/photos/2019/08/01/1153376/original.jpg	https://data.globe.gov/system/photos/2019/08/01/1153378/original.jpg	https://data.globe.gov/system/photos/2019/08/01/1153377/original.jpg	https://data.globe.gov/system/photos/2019/08/01/1153379/original.jpg	https://data.globe.gov/system/photos/2019/08/01/1153381/original.jpg
GV_1553	46.9756	-112.3578	1870.1		https://www.google.com/maps/search/46.9756,-112.3578	https://data.globe.gov/system/photos/2019/08/01/1153382/original.jpg	https://data.globe.gov/system/photos/2019/08/01/1153384/original.jpg	https://data.globe.gov/system/photos/2019/08/01/1153383/original.jpg	https://data.globe.gov/system/photos/2019/08/01/1153385/original.jpg	https://data.globe.gov/system/photos/2019/08/01/1153387/original.jpg
GV_1554	46.975	-112.3581	1873.4		https://www.google.com/maps/search/46.975,-112.3581	https://data.globe.gov/system/photos/2019/08/01/1153388/original.jpg	https://data.globe.gov/system/photos/2019/08/01/1153390/original.jpg	https://data.globe.gov/system/photos/2019/08/01/1153389/original.jpg	https://data.globe.gov/system/photos/2019/08/01/1153391/original.jpg	https://data.globe.gov/system/photos/2019/08/01/1153393/original.jpg
GV_1555	46.2661	-111.4928	1173.1		https://www.google.com/maps/search/46.2661,-111.4928	https://data.globe.gov/system/photos/2019/07/31/1153442/original.jpg	https://data.globe.gov/system/photos/2019/07/31/1153444/original.jpg	https://data.globe.gov/system/photos/2019/07/31/1153443/original.jpg	https://data.globe.gov/system/photos/2019/07/31/1153445/original.jpg	https://data.globe.gov/system/photos/2019/07/31/1153447/original.jpg
GV_1556	44.6465	-110.9307	2062.2		https://www.google.com/maps/search/44.6465,-110.9307	https://data.globe.gov/system/photos/2019/07/31/1153496/original.jpg	https://data.globe.gov/system/photos/2019/07/31/1153498/original.jpg	https://data.globe.gov/system/photos/2019/07/31/1153497/original.jpg	https://data.globe.gov/system/photos/2019/07/31/1153499/original.jpg	https://data.globe.gov/system/photos/2019/07/31/1153501/original.jpg
GV_1557	48.0311	-110.2362	765.3		https://www.google.com/maps/search/48.0311,-110.2362	https://data.globe.gov/system/photos/2019/07/29/1153556/original.jpg	https://data.globe.gov/system/photos/2019/07/29/1153558/original.jpg	https://data.globe.gov/system/photos/2019/07/29/1153557/original.jpg	https://data.globe.gov/system/photos/2019/07/29/1153559/original.jpg	https://data.globe.gov/system/photos/2019/07/29/1153561/original.jpg
GV_1558	48.0316	-110.236	768.7		https://www.google.com/maps/search/48.0316,-110.236	https://data.globe.gov/system/photos/2019/07/29/1153562/original.jpg	https://data.globe.gov/system/photos/2019/07/29/1153564/original.jpg	https://data.globe.gov/system/photos/2019/07/29/1153563/original.jpg	https://data.globe.gov/system/photos/2019/07/29/1153565/original.jpg	https://data.globe.gov/system/photos/2019/07/29/1153567/original.jpg
GV_1559	48.0318	-110.236	768.6		https://www.google.com/maps/search/48.0318,-110.236	https://data.globe.gov/system/photos/2019/07/26/1153753/original.jpg	https://data.globe.gov/system/photos/2019/07/26/1153755/original.jpg	https://data.globe.gov/system/photos/2019/07/26/1153754/original.jpg	https://data.globe.gov/system/photos/2019/07/26/1153756/original.jpg	https://data.globe.gov/system/photos/2019/07/26/1153758/original.jpg
GV_1560	48.0318	-110.2351	765		https://www.google.com/maps/search/48.0318,-110.2351	https://data.globe.gov/system/photos/2019/07/26/1153759/original.jpg	https://data.globe.gov/system/photos/2019/07/26/1153761/original.jpg	https://data.globe.gov/system/photos/2019/07/26/1153760/original.jpg	https://data.globe.gov/system/photos/2019/07/26/1153762/original.jpg	https://data.globe.gov/system/photos/2019/07/26/1153764/original.jpg
GV_1561	48.0033	-110.1222	761		https://www.google.com/maps/search/48.0033,-110.1222	https://data.globe.gov/system/photos/2019/07/26/1153766/original.jpg	https://data.globe.gov/system/photos/2019/07/26/1153768/original.jpg	https://data.globe.gov/system/photos/2019/07/26/1153767/original.jpg	https://data.globe.gov/system/photos/2019/07/26/1153769/original.jpg	https://data.globe.gov/system/photos/2019/07/26/1153771/original.jpg
GV_1562	47.9851	-110.1119	760		https://www.google.com/maps/search/47.9851,-110.1119	https://data.globe.gov/system/photos/2019/07/26/1153772/original.jpg	https://data.globe.gov/system/photos/2019/07/26/1153775/original.jpg	https://data.globe.gov/system/photos/2019/07/26/1153774/original.jpg	https://data.globe.gov/system/photos/2019/07/26/1153776/original.jpg	https://data.globe.gov/system/photos/2019/07/26/1153778/original.jpg
GV_1563	47.9556	-110.087	758		https://www.google.com/maps/search/47.9556,-110.087	https://data.globe.gov/system/photos/2019/07/26/1153780/original.jpg	https://data.globe.gov/system/photos/2019/07/26/1153782/original.jpg	https://data.globe.gov/system/photos/2019/07/26/1153781/original.jpg	https://data.globe.gov/system/photos/2019/07/26/1153783/original.jpg	https://data.globe.gov/system/photos/2019/07/26/1153785/original.jpg
GV_1564	47.9299	-110.0642	768.2		https://www.google.com/maps/search/47.9299,-110.0642	https://data.globe.gov/system/photos/2019/07/26/1153786/original.jpg	https://data.globe.gov/system/photos/2019/07/26/1153788/original.jpg	https://data.globe.gov/system/photos/2019/07/26/1153787/original.jpg	https://data.globe.gov/system/photos/2019/07/26/1153789/original.jpg	https://data.globe.gov/system/photos/2019/07/26/1153791/original.jpg
GV_1565	47.9139	-110.0574	760.9		https://www.google.com/maps/search/47.9139,-110.0574	https://data.globe.gov/system/photos/2019/07/26/1153792/original.jpg	https://data.globe.gov/system/photos/2019/07/26/1153794/original.jpg	https://data.globe.gov/system/photos/2019/07/26/1153793/original.jpg	https://data.globe.gov/system/photos/2019/07/26/1153795/original.jpg	https://data.globe.gov/system/photos/2019/07/26/1153797/original.jpg
GV_1566	47.9127	-110.0575	761.3		https://www.google.com/maps/search/47.9127,-110.0575	https://data.globe.gov/system/photos/2019/07/27/1153850/original.jpg	https://data.globe.gov/system/photos/2019/07/27/1153853/original.jpg	https://data.globe.gov/system/photos/2019/07/27/1153852/original.jpg	https://data.globe.gov/system/photos/2019/07/27/1153854/original.jpg	https://data.globe.gov/system/photos/2019/07/27/1153856/original.jpg
GV_1567	47.9032	-110.0598	756		https://www.google.com/maps/search/47.9032,-110.0598	https://data.globe.gov/system/photos/2019/07/27/1153857/original.jpg	https://data.globe.gov/system/photos/2019/07/27/1153859/original.jpg	https://data.globe.gov/system/photos/2019/07/27/1153858/original.jpg	https://data.globe.gov/system/photos/2019/07/27/1153860/original.jpg	https://data.globe.gov/system/photos/2019/07/27/1153862/original.jpg
GV_1568	47.8789	-110.0649	756		https://www.google.com/maps/search/47.8789,-110.0649	https://data.globe.gov/system/photos/2019/07/27/1153863/original.jpg	https://data.globe.gov/system/photos/2019/07/27/1153865/original.jpg	https://data.globe.gov/system/photos/2019/07/27/1153864/original.jpg	https://data.globe.gov/system/photos/2019/07/27/1153866/original.jpg	https://data.globe.gov/system/photos/2019/07/27/1153868/original.jpg
GV_1569	47.832	-110.0743	773.8		https://www.google.com/maps/search/47.832,-110.0743	https://data.globe.gov/system/photos/2019/07/27/1153900/original.jpg	https://data.globe.gov/system/photos/2019/07/27/1153902/original.jpg	https://data.globe.gov/system/photos/2019/07/27/1153901/original.jpg	https://data.globe.gov/system/photos/2019/07/27/1153903/original.jpg	https://data.globe.gov/system/photos/2019/07/27/1153905/original.jpg
GV_1570	47.8035	-109.9997	750.3		https://www.google.com/maps/search/47.8035,-109.9997	https://data.globe.gov/system/photos/2019/07/27/1153913/original.jpg	https://data.globe.gov/system/photos/2019/07/27/1153916/original.jpg	https://data.globe.gov/system/photos/2019/07/27/1153914/original.jpg	https://data.globe.gov/system/photos/2019/07/27/1153917/original.jpg	https://data.globe.gov/system/photos/2019/07/27/1153919/original.jpg
GV_1571	47.7883	-109.9812	749.5		https://www.google.com/maps/search/47.7883,-109.9812	https://data.globe.gov/system/photos/2019/07/27/1153920/original.jpg	https://data.globe.gov/system/photos/2019/07/27/1153923/original.jpg	https://data.globe.gov/system/photos/2019/07/27/1153922/original.jpg	https://data.globe.gov/system/photos/2019/07/27/1153925/original.jpg	https://data.globe.gov/system/photos/2019/07/27/1153929/original.jpg
GV_1572	40.8211	-74.2058	86.3		https://www.google.com/maps/search/40.8211,-74.2058	https://data.globe.gov/system/photos/2019/07/30/1153935/original.jpg	https://data.globe.gov/system/photos/2019/07/30/1153940/original.jpg	https://data.globe.gov/system/photos/2019/07/30/1153938/original.jpg	https://data.globe.gov/system/photos/2019/07/30/1153942/original.jpg	https://data.globe.gov/system/photos/2019/07/30/1153945/original.jpg
GV_1573	40.8241	-74.2038	88.8		https://www.google.com/maps/search/40.8241,-74.2038	https://data.globe.gov/system/photos/2019/08/01/1153948/original.jpg	https://data.globe.gov/system/photos/2019/08/01/1153951/original.jpg	https://data.globe.gov/system/photos/2019/08/01/1153950/original.jpg	https://data.globe.gov/system/photos/2019/08/01/1153952/original.jpg	https://data.globe.gov/system/photos/2019/08/01/1153955/original.jpg
GV_1574	40.8241	-74.2045	87.4		https://www.google.com/maps/search/40.8241,-74.2045	https://data.globe.gov/system/photos/2019/08/01/1153956/original.jpg	https://data.globe.gov/system/photos/2019/08/01/1153958/original.jpg	https://data.globe.gov/system/photos/2019/08/01/1153957/original.jpg	https://data.globe.gov/system/photos/2019/08/01/1153960/original.jpg	https://data.globe.gov/system/photos/2019/08/01/1153963/original.jpg
GV_1575	47.8234	-110.0678	752.7		https://www.google.com/maps/search/47.8234,-110.0678	https://data.globe.gov/system/photos/2019/07/27/1153931/original.jpg	https://data.globe.gov/system/photos/2019/07/27/1153933/original.jpg	https://data.globe.gov/system/photos/2019/07/27/1153932/original.jpg	https://data.globe.gov/system/photos/2019/07/27/1153934/original.jpg	https://data.globe.gov/system/photos/2019/07/27/1153937/original.jpg
GV_1576	47.819	-110.0462	752.3		https://www.google.com/maps/search/47.819,-110.0462	https://data.globe.gov/system/photos/2019/07/27/1153939/original.jpg	https://data.globe.gov/system/photos/2019/07/27/1153944/original.jpg	https://data.globe.gov/system/photos/2019/07/27/1153941/original.jpg	https://data.globe.gov/system/photos/2019/07/27/1153946/original.jpg	https://data.globe.gov/system/photos/2019/07/27/1153949/original.jpg
GV_1577	47.7837	-109.9568	753.4		https://www.google.com/maps/search/47.7837,-109.9568	https://data.globe.gov/system/photos/2019/07/27/1153964/original.jpg	https://data.globe.gov/system/photos/2019/07/27/1153966/original.jpg	https://data.globe.gov/system/photos/2019/07/27/1153965/original.jpg	https://data.globe.gov/system/photos/2019/07/27/1153967/original.jpg	https://data.globe.gov/system/photos/2019/07/27/1153969/original.jpg
GV_1578	47.7625	-109.9189	750		https://www.google.com/maps/search/47.7625,-109.9189	https://data.globe.gov/system/photos/2019/07/28/1153987/original.jpg	https://data.globe.gov/system/photos/2019/07/28/1153989/original.jpg	https://data.globe.gov/system/photos/2019/07/28/1153988/original.jpg	https://data.globe.gov/system/photos/2019/07/28/1153990/original.jpg	https://data.globe.gov/system/photos/2019/07/28/1153992/original.jpg
GV_1579	47.7633	-109.9003	746.6		https://www.google.com/maps/search/47.7633,-109.9003	https://data.globe.gov/system/photos/2019/07/28/1153998/original.jpg	https://data.globe.gov/system/photos/2019/07/28/1154000/original.jpg	https://data.globe.gov/system/photos/2019/07/28/1153999/original.jpg	https://data.globe.gov/system/photos/2019/07/28/1154001/original.jpg	https://data.globe.gov/system/photos/2019/07/28/1154003/original.jpg
GV_1580	47.5304	-111.2355	987.9		https://www.google.com/maps/search/47.5304,-111.2355	https://data.globe.gov/system/photos/2019/08/01/1154014/original.jpg	https://data.globe.gov/system/photos/2019/08/01/1154016/original.jpg	https://data.globe.gov/system/photos/2019/08/01/1154015/original.jpg	https://data.globe.gov/system/photos/2019/08/01/1154017/original.jpg	https://data.globe.gov/system/photos/2019/08/01/1154019/original.jpg
GV_1581	46.9897	-107.8599	808.2		https://www.google.com/maps/search/46.9897,-107.8599	https://data.globe.gov/system/photos/2019/07/30/1154020/original.jpg	https://data.globe.gov/system/photos/2019/07/30/1154022/original.jpg	https://data.globe.gov/system/photos/2019/07/30/1154021/original.jpg	https://data.globe.gov/system/photos/2019/07/30/1154023/original.jpg	https://data.globe.gov/system/photos/2019/07/30/1154025/original.jpg
GV_1582	46.9898	-107.859	807.8		https://www.google.com/maps/search/46.9898,-107.859	https://data.globe.gov/system/photos/2019/07/30/1154026/original.jpg	https://data.globe.gov/system/photos/2019/07/30/1154028/original.jpg	https://data.globe.gov/system/photos/2019/07/30/1154027/original.jpg	https://data.globe.gov/system/photos/2019/07/30/1154029/original.jpg	https://data.globe.gov/system/photos/2019/07/30/1154031/original.jpg
GV_1583	47.3253	-106.1715	753.4		https://www.google.com/maps/search/47.3253,-106.1715	https://data.globe.gov/system/photos/2019/07/30/1154032/original.jpg	https://data.globe.gov/system/photos/2019/07/30/1154034/original.jpg	https://data.globe.gov/system/photos/2019/07/30/1154033/original.jpg	https://data.globe.gov/system/photos/2019/07/30/1154035/original.jpg	https://data.globe.gov/system/photos/2019/07/30/1154037/original.jpg
GV_1584	46.9262	-103.5291	721.1		https://www.google.com/maps/search/46.9262,-103.5291	https://data.globe.gov/system/photos/2019/07/31/1154038/original.jpg	https://data.globe.gov/system/photos/2019/07/31/1154040/original.jpg	https://data.globe.gov/system/photos/2019/07/31/1154039/original.jpg	https://data.globe.gov/system/photos/2019/07/31/1154041/original.jpg	https://data.globe.gov/system/photos/2019/07/31/1154043/original.jpg
GV_1585	46.9209	-103.5293	686.1		https://www.google.com/maps/search/46.9209,-103.5293	https://data.globe.gov/system/photos/2019/07/31/1154044/original.jpg	https://data.globe.gov/system/photos/2019/07/31/1154046/original.jpg	https://data.globe.gov/system/photos/2019/07/31/1154045/original.jpg	https://data.globe.gov/system/photos/2019/07/31/1154047/original.jpg	https://data.globe.gov/system/photos/2019/07/31/1154049/original.jpg
GV_1586	46.9218	-103.529	685.7		https://www.google.com/maps/search/46.9218,-103.529	https://data.globe.gov/system/photos/2019/07/31/1154050/original.jpg	https://data.globe.gov/system/photos/2019/07/31/1154052/original.jpg	https://data.globe.gov/system/photos/2019/07/31/1154051/original.jpg	https://data.globe.gov/system/photos/2019/07/31/1154053/original.jpg	https://data.globe.gov/system/photos/2019/07/31/1154055/original.jpg
GV_1587	46.8953	-103.3809	846.3		https://www.google.com/maps/search/46.8953,-103.3809	https://data.globe.gov/system/photos/2019/07/31/1154056/original.jpg	https://data.globe.gov/system/photos/2019/07/31/1154058/original.jpg	https://data.globe.gov/system/photos/2019/07/31/1154057/original.jpg	https://data.globe.gov/system/photos/2019/07/31/1154059/original.jpg	https://data.globe.gov/system/photos/2019/07/31/1154061/original.jpg
GV_1588	46.8951	-103.3797	844.3		https://www.google.com/maps/search/46.8951,-103.3797	https://data.globe.gov/system/photos/2019/07/31/1154062/original.jpg	https://data.globe.gov/system/photos/2019/07/31/1154064/original.jpg	https://data.globe.gov/system/photos/2019/07/31/1154063/original.jpg	https://data.globe.gov/system/photos/2019/07/31/1154065/original.jpg	https://data.globe.gov/system/photos/2019/07/31/1154067/original.jpg
GV_1589	53.4283	-0.6226	26		https://www.google.com/maps/search/53.4283,-0.6226	https://data.globe.gov/system/photos/2019/08/01/1154074/original.jpg	https://data.globe.gov/system/photos/2019/08/01/1154076/original.jpg	https://data.globe.gov/system/photos/2019/08/01/1154075/original.jpg	https://data.globe.gov/system/photos/2019/08/01/1154077/original.jpg	https://data.globe.gov/system/photos/2019/08/01/1154079/original.jpg
GV_1590	47.7048	-109.7545	743.9		https://www.google.com/maps/search/47.7048,-109.7545	https://data.globe.gov/system/photos/2019/07/28/1154086/original.jpg	https://data.globe.gov/system/photos/2019/07/28/1154088/original.jpg	https://data.globe.gov/system/photos/2019/07/28/1154087/original.jpg	https://data.globe.gov/system/photos/2019/07/28/1154089/original.jpg	https://data.globe.gov/system/photos/2019/07/28/1154091/original.jpg
GV_1591	44.7145	-110.6584	2334.5		https://www.google.com/maps/search/44.7145,-110.6584	https://data.globe.gov/system/photos/2019/07/31/1154161/original.jpg	https://data.globe.gov/system/photos/2019/07/31/1154163/original.jpg	https://data.globe.gov/system/photos/2019/07/31/1154162/original.jpg	https://data.globe.gov/system/photos/2019/07/31/1154164/original.jpg	https://data.globe.gov/system/photos/2019/07/31/1154166/original.jpg
GV_1592	48.1428	-103.6218	566.8		https://www.google.com/maps/search/48.1428,-103.6218	https://data.globe.gov/system/photos/2019/08/02/1154178/original.jpg	https://data.globe.gov/system/photos/2019/08/02/1154180/original.jpg	https://data.globe.gov/system/photos/2019/08/02/1154179/original.jpg	https://data.globe.gov/system/photos/2019/08/02/1154181/original.jpg	https://data.globe.gov/system/photos/2019/08/02/1154183/original.jpg
GV_1593	38.0784	127.2182	113		https://www.google.com/maps/search/38.0784,127.2182	https://data.globe.gov/system/photos/2019/08/02/1154400/original.jpg	https://data.globe.gov/system/photos/2019/08/02/1154402/original.jpg	https://data.globe.gov/system/photos/2019/08/02/1154401/original.jpg	https://data.globe.gov/system/photos/2019/08/02/1154403/original.jpg	https://data.globe.gov/system/photos/2019/08/02/1154405/original.jpg
GV_1594	44.8061	-110.4454	2699.1		https://www.google.com/maps/search/44.8061,-110.4454	https://data.globe.gov/system/photos/2019/07/31/1154497/original.jpg	https://data.globe.gov/system/photos/2019/07/31/1154499/original.jpg	https://data.globe.gov/system/photos/2019/07/31/1154498/original.jpg	https://data.globe.gov/system/photos/2019/07/31/1154500/original.jpg	https://data.globe.gov/system/photos/2019/07/31/1154502/original.jpg
GV_1595	44.7287	-110.7034	2283.9		https://www.google.com/maps/search/44.7287,-110.7034	https://data.globe.gov/system/photos/2019/07/31/1154513/original.jpg	https://data.globe.gov/system/photos/2019/07/31/1154515/original.jpg	https://data.globe.gov/system/photos/2019/07/31/1154514/original.jpg	https://data.globe.gov/system/photos/2019/07/31/1154516/original.jpg	https://data.globe.gov/system/photos/2019/07/31/1154518/original.jpg
GV_1596	47.7177	-109.8501	746.9		https://www.google.com/maps/search/47.7177,-109.8501	https://data.globe.gov/system/photos/2019/07/28/1154535/original.jpg	https://data.globe.gov/system/photos/2019/07/28/1154537/original.jpg	https://data.globe.gov/system/photos/2019/07/28/1154536/original.jpg	https://data.globe.gov/system/photos/2019/07/28/1154538/original.jpg	https://data.globe.gov/system/photos/2019/07/28/1154540/original.jpg
GV_1597	47.7173	-109.8487	745.6		https://www.google.com/maps/search/47.7173,-109.8487	https://data.globe.gov/system/photos/2019/07/28/1154541/original.jpg	https://data.globe.gov/system/photos/2019/07/28/1154543/original.jpg	https://data.globe.gov/system/photos/2019/07/28/1154542/original.jpg	https://data.globe.gov/system/photos/2019/07/28/1154544/original.jpg	https://data.globe.gov/system/photos/2019/07/28/1154546/original.jpg
GV_1598	47.7174	-109.848	746.2		https://www.google.com/maps/search/47.7174,-109.848	https://data.globe.gov/system/photos/2019/07/28/1154547/original.jpg	https://data.globe.gov/system/photos/2019/07/28/1154549/original.jpg	https://data.globe.gov/system/photos/2019/07/28/1154548/original.jpg	https://data.globe.gov/system/photos/2019/07/28/1154550/original.jpg	https://data.globe.gov/system/photos/2019/07/28/1154552/original.jpg
GV_1599	38.9942	-76.8402	63.3		https://www.google.com/maps/search/38.9942,-76.8402	https://data.globe.gov/system/photos/2019/08/02/1154831/original.jpg	https://data.globe.gov/system/photos/2019/08/02/1154833/original.jpg	https://data.globe.gov/system/photos/2019/08/02/1154832/original.jpg	https://data.globe.gov/system/photos/2019/08/02/1154834/original.jpg	https://data.globe.gov/system/photos/2019/08/02/1154836/original.jpg
GV_1600	26.3345	-80.1144	3.2		https://www.google.com/maps/search/26.3345,-80.1144	https://data.globe.gov/system/photos/2019/07/27/1155013/original.jpg	https://data.globe.gov/system/photos/2019/07/27/1155016/original.jpg	https://data.globe.gov/system/photos/2019/07/27/1155014/original.jpg	https://data.globe.gov/system/photos/2019/07/27/1155018/original.jpg	https://data.globe.gov/system/photos/2019/07/27/1155021/original.jpg
GV_1601	-27.4892	135.4173	131.4		https://www.google.com/maps/search/-27.4892,135.4173	https://data.globe.gov/system/photos/2019/08/02/1155060/original.jpg	https://data.globe.gov/system/photos/2019/08/02/1155065/original.jpg	https://data.globe.gov/system/photos/2019/08/02/1155063/original.jpg	https://data.globe.gov/system/photos/2019/08/02/1155066/original.jpg	https://data.globe.gov/system/photos/2019/08/02/1155068/original.jpg
GV_1602	-34.6652	138.8233	241.2		https://www.google.com/maps/search/-34.6652,138.8233	https://data.globe.gov/system/photos/2019/08/03/1155087/original.jpg	https://data.globe.gov/system/photos/2019/08/03/1155089/original.jpg	https://data.globe.gov/system/photos/2019/08/03/1155088/original.jpg	https://data.globe.gov/system/photos/2019/08/03/1155090/original.jpg	https://data.globe.gov/system/photos/2019/08/03/1155092/original.jpg
GV_1603	-34.6648	138.8214	207.6		https://www.google.com/maps/search/-34.6648,138.8214	https://data.globe.gov/system/photos/2019/08/03/1155163/original.jpg	https://data.globe.gov/system/photos/2019/08/03/1155165/original.jpg	https://data.globe.gov/system/photos/2019/08/03/1155164/original.jpg	https://data.globe.gov/system/photos/2019/08/03/1155166/original.jpg	https://data.globe.gov/system/photos/2019/08/03/1155168/original.jpg
GV_1604	53.4283	-0.6226	26		https://www.google.com/maps/search/53.4283,-0.6226	https://data.globe.gov/system/photos/2019/08/02/1155229/original.jpg	https://data.globe.gov/system/photos/2019/08/02/1155231/original.jpg	https://data.globe.gov/system/photos/2019/08/02/1155230/original.jpg	https://data.globe.gov/system/photos/2019/08/02/1155232/original.jpg	https://data.globe.gov/system/photos/2019/08/02/1155234/original.jpg
GV_1605	26.333	-80.1199	4.1		https://www.google.com/maps/search/26.333,-80.1199	https://data.globe.gov/system/photos/2019/08/02/1155299/original.jpg	https://data.globe.gov/system/photos/2019/08/02/1155301/original.jpg	https://data.globe.gov/system/photos/2019/08/02/1155300/original.jpg	https://data.globe.gov/system/photos/2019/08/02/1155302/original.jpg	https://data.globe.gov/system/photos/2019/08/02/1155304/original.jpg
GV_1606	45.0328	-92.8266	289.8		https://www.google.com/maps/search/45.0328,-92.8266	https://data.globe.gov/system/photos/2019/08/02/1155361/original.jpg	https://data.globe.gov/system/photos/2019/08/02/1155363/original.jpg	https://data.globe.gov/system/photos/2019/08/02/1155362/original.jpg	https://data.globe.gov/system/photos/2019/08/02/1155364/original.jpg	https://data.globe.gov/system/photos/2019/08/02/1155366/original.jpg
GV_1607	45.008	-109.9864	2255.5		https://www.google.com/maps/search/45.008,-109.9864	https://data.globe.gov/system/photos/2019/07/30/1155367/original.jpg	https://data.globe.gov/system/photos/2019/07/30/1155369/original.jpg	https://data.globe.gov/system/photos/2019/07/30/1155368/original.jpg	https://data.globe.gov/system/photos/2019/07/30/1155370/original.jpg	https://data.globe.gov/system/photos/2019/07/30/1155372/original.jpg
GV_1608	47.7042	-109.7548	739.4		https://www.google.com/maps/search/47.7042,-109.7548	https://data.globe.gov/system/photos/2019/07/29/1155466/original.jpg	https://data.globe.gov/system/photos/2019/07/29/1155468/original.jpg	https://data.globe.gov/system/photos/2019/07/29/1155467/original.jpg	https://data.globe.gov/system/photos/2019/07/29/1155470/original.jpg	https://data.globe.gov/system/photos/2019/07/29/1155472/original.jpg
GV_1609	47.7043	-109.7545	742.3		https://www.google.com/maps/search/47.7043,-109.7545	https://data.globe.gov/system/photos/2019/07/29/1155474/original.jpg	https://data.globe.gov/system/photos/2019/07/29/1155477/original.jpg	https://data.globe.gov/system/photos/2019/07/29/1155475/original.jpg	https://data.globe.gov/system/photos/2019/07/29/1155480/original.jpg	https://data.globe.gov/system/photos/2019/07/29/1155486/original.jpg
GV_1610	47.8396	-110.6853	879.3		https://www.google.com/maps/search/47.8396,-110.6853	https://data.globe.gov/system/photos/2019/07/29/1155499/original.jpg	https://data.globe.gov/system/photos/2019/07/29/1155501/original.jpg	https://data.globe.gov/system/photos/2019/07/29/1155500/original.jpg	https://data.globe.gov/system/photos/2019/07/29/1155502/original.jpg	https://data.globe.gov/system/photos/2019/07/29/1155504/original.jpg
GV_1611	47.8103	-110.6731	802.3		https://www.google.com/maps/search/47.8103,-110.6731	https://data.globe.gov/system/photos/2019/07/29/1155505/original.jpg	https://data.globe.gov/system/photos/2019/07/29/1155507/original.jpg	https://data.globe.gov/system/photos/2019/07/29/1155506/original.jpg	https://data.globe.gov/system/photos/2019/07/29/1155508/original.jpg	https://data.globe.gov/system/photos/2019/07/29/1155510/original.jpg
GV_1612	46.8243	-110.698	2134.1		https://www.google.com/maps/search/46.8243,-110.698	https://data.globe.gov/system/photos/2019/07/30/1155511/original.jpg	https://data.globe.gov/system/photos/2019/07/30/1155513/original.jpg	https://data.globe.gov/system/photos/2019/07/30/1155512/original.jpg	https://data.globe.gov/system/photos/2019/07/30/1155514/original.jpg	https://data.globe.gov/system/photos/2019/07/30/1155516/original.jpg
GV_1613	47.7171	-109.8476	744.3		https://www.google.com/maps/search/47.7171,-109.8476	https://data.globe.gov/system/photos/2019/07/28/1155611/original.jpg	https://data.globe.gov/system/photos/2019/07/28/1155613/original.jpg	https://data.globe.gov/system/photos/2019/07/28/1155612/original.jpg	https://data.globe.gov/system/photos/2019/07/28/1155614/original.jpg	https://data.globe.gov/system/photos/2019/07/28/1155616/original.jpg

GV_1614	47.7169	-109.8487	743		https://www.google.com/maps/search/47.7169,-109.8487	https://data.globe.gov/system/photos/2019/07/28/1155620/original.jpg	https://data.globe.gov/system/photos/2019/07/28/1155623/original.jpg	https://data.globe.gov/system/photos/2019/07/28/1155622/original.jpg	https://data.globe.gov/system/photos/2019/07/28/1155626/original.jpg	https://data.globe.gov/system/photos/2019/07/28/1155628/original.jpg
GV_1615	47.7174	-109.8506	747		https://www.google.com/maps/search/47.7174,-109.8506	https://data.globe.gov/system/photos/2019/07/28/1155654/original.jpg	https://data.globe.gov/system/photos/2019/07/28/1155656/original.jpg	https://data.globe.gov/system/photos/2019/07/28/1155655/original.jpg	https://data.globe.gov/system/photos/2019/07/28/1155657/original.jpg	https://data.globe.gov/system/photos/2019/07/28/1155659/original.jpg
GV_1616	47.7084	-109.7654	738.4		https://www.google.com/maps/search/47.7084,-109.7654	https://data.globe.gov/system/photos/2019/07/28/1155660/original.jpg	https://data.globe.gov/system/photos/2019/07/28/1155662/original.jpg	https://data.globe.gov/system/photos/2019/07/28/1155661/original.jpg	https://data.globe.gov/system/photos/2019/07/28/1155663/original.jpg	https://data.globe.gov/system/photos/2019/07/28/1155665/original.jpg
GV_1617	47.7174	-109.8504	747.2		https://www.google.com/maps/search/47.7174,-109.8504	https://data.globe.gov/system/photos/2019/07/28/1155666/original.jpg	https://data.globe.gov/system/photos/2019/07/28/1155668/original.jpg	https://data.globe.gov/system/photos/2019/07/28/1155667/original.jpg	https://data.globe.gov/system/photos/2019/07/28/1155669/original.jpg	https://data.globe.gov/system/photos/2019/07/28/1155671/original.jpg
GV_1618	47.7043	-109.7537	741.8		https://www.google.com/maps/search/47.7043,-109.7537	https://data.globe.gov/system/photos/2019/07/28/1155672/original.jpg	https://data.globe.gov/system/photos/2019/07/28/1155674/original.jpg	https://data.globe.gov/system/photos/2019/07/28/1155673/original.jpg	https://data.globe.gov/system/photos/2019/07/28/1155676/original.jpg	https://data.globe.gov/system/photos/2019/07/28/1155683/original.jpg
GV_1619	45.0298	-110.7087	1618.2		https://www.google.com/maps/search/45.0298,-110.7087	https://data.globe.gov/system/photos/2019/07/30/1155684/original.jpg	https://data.globe.gov/system/photos/2019/07/30/1155686/original.jpg	https://data.globe.gov/system/photos/2019/07/30/1155685/original.jpg	https://data.globe.gov/system/photos/2019/07/30/1155687/original.jpg	https://data.globe.gov/system/photos/2019/07/30/1155689/original.jpg
GV_1620	45.0296	-110.7089	1618		https://www.google.com/maps/search/45.0296,-110.7089	https://data.globe.gov/system/photos/2019/07/30/1155690/original.jpg	https://data.globe.gov/system/photos/2019/07/30/1155692/original.jpg	https://data.globe.gov/system/photos/2019/07/30/1155691/original.jpg	https://data.globe.gov/system/photos/2019/07/30/1155696/original.jpg	https://data.globe.gov/system/photos/2019/07/30/1155701/original.jpg
GV_1621	44.9415	-110.6279	2007.5		https://www.google.com/maps/search/44.9415,-110.6279	https://data.globe.gov/system/photos/2019/07/30/1155702/original.jpg	https://data.globe.gov/system/photos/2019/07/30/1155704/original.jpg	https://data.globe.gov/system/photos/2019/07/30/1155703/original.jpg	https://data.globe.gov/system/photos/2019/07/30/1155705/original.jpg	https://data.globe.gov/system/photos/2019/07/30/1155707/original.jpg
GV_1622	44.9152	-110.4384	2045.7		https://www.google.com/maps/search/44.9152,-110.4384	https://data.globe.gov/system/photos/2019/07/30/1155708/original.jpg	https://data.globe.gov/system/photos/2019/07/30/1155710/original.jpg	https://data.globe.gov/system/photos/2019/07/30/1155709/original.jpg	https://data.globe.gov/system/photos/2019/07/30/1155711/original.jpg	https://data.globe.gov/system/photos/2019/07/30/1155713/original.jpg
GV_1623	44.9163	-110.4394	2054.9		https://www.google.com/maps/search/44.9163,-110.4394	https://data.globe.gov/system/photos/2019/07/30/1155714/original.jpg	https://data.globe.gov/system/photos/2019/07/30/1155716/original.jpg	https://data.globe.gov/system/photos/2019/07/30/1155715/original.jpg	https://data.globe.gov/system/photos/2019/07/30/1155717/original.jpg	https://data.globe.gov/system/photos/2019/07/30/1155719/original.jpg
GV_1624	45.6546	-110.5714	1379.7		https://www.google.com/maps/search/45.6546,-110.5714	https://data.globe.gov/system/photos/2019/07/30/1155720/original.jpg	https://data.globe.gov/system/photos/2019/07/30/1155722/original.jpg	https://data.globe.gov/system/photos/2019/07/30/1155721/original.jpg	https://data.globe.gov/system/photos/2019/07/30/1155723/original.jpg	https://data.globe.gov/system/photos/2019/07/30/1155725/original.jpg
GV_1625	45.0296	-110.7055	1622.8		https://www.google.com/maps/search/45.0296,-110.7055	https://data.globe.gov/system/photos/2019/07/30/1155726/original.jpg	https://data.globe.gov/system/photos/2019/07/30/1155728/original.jpg	https://data.globe.gov/system/photos/2019/07/30/1155727/original.jpg	https://data.globe.gov/system/photos/2019/07/30/1155729/original.jpg	https://data.globe.gov/system/photos/2019/07/30/1155731/original.jpg
GV_1626	45.0311	-92.8347	284.8		https://www.google.com/maps/search/45.0311,-92.8347	https://data.globe.gov/system/photos/2019/08/03/1155830/original.jpg	https://data.globe.gov/system/photos/2019/08/03/1155832/original.jpg	https://data.globe.gov/system/photos/2019/08/03/1155831/original.jpg	https://data.globe.gov/system/photos/2019/08/03/1155833/original.jpg	https://data.globe.gov/system/photos/2019/08/03/1155835/original.jpg
GV_1627	45.0314	-92.8341	284.4		https://www.google.com/maps/search/45.0314,-92.8341	https://data.globe.gov/system/photos/2019/08/03/1155836/original.jpg	https://data.globe.gov/system/photos/2019/08/03/1155838/original.jpg	https://data.globe.gov/system/photos/2019/08/03/1155837/original.jpg	https://data.globe.gov/system/photos/2019/08/03/1155839/original.jpg	https://data.globe.gov/system/photos/2019/08/03/1155841/original.jpg
GV_1628	45.0304	-92.8338	283.4		https://www.google.com/maps/search/45.0304,-92.8338	https://data.globe.gov/system/photos/2019/08/03/1155842/original.jpg	https://data.globe.gov/system/photos/2019/08/03/1155844/original.jpg	https://data.globe.gov/system/photos/2019/08/03/1155843/original.jpg	https://data.globe.gov/system/photos/2019/08/03/1155845/original.jpg	https://data.globe.gov/system/photos/2019/08/03/1155847/original.jpg
GV_1629	45.0296	-92.8348	284.2		https://www.google.com/maps/search/45.0296,-92.8348	https://data.globe.gov/system/photos/2019/08/03/1155848/original.jpg	https://data.globe.gov/system/photos/2019/08/03/1155850/original.jpg	https://data.globe.gov/system/photos/2019/08/03/1155849/original.jpg	https://data.globe.gov/system/photos/2019/08/03/1155851/original.jpg	https://data.globe.gov/system/photos/2019/08/03/1155853/original.jpg
GV_1630	45.0302	-92.835	286.5		https://www.google.com/maps/search/45.0302,-92.835	https://data.globe.gov/system/photos/2019/08/03/1155854/original.jpg	https://data.globe.gov/system/photos/2019/08/03/1155856/original.jpg	https://data.globe.gov/system/photos/2019/08/03/1155855/original.jpg	https://data.globe.gov/system/photos/2019/08/03/1155857/original.jpg	https://data.globe.gov/system/photos/2019/08/03/1155860/original.jpg
GV_1631	45.0303	-92.8366	285.6		https://www.google.com/maps/search/45.0303,-92.8366	https://data.globe.gov/system/photos/2019/08/03/1155862/original.jpg	https://data.globe.gov/system/photos/2019/08/03/1155866/original.jpg	https://data.globe.gov/system/photos/2019/08/03/1155865/original.jpg	https://data.globe.gov/system/photos/2019/08/03/1155867/original.jpg	https://data.globe.gov/system/photos/2019/08/03/1155870/original.jpg
GV_1632	45.0302	-92.8375	285.3		https://www.google.com/maps/search/45.0302,-92.8375	https://data.globe.gov/system/photos/2019/08/03/1155871/original.jpg	https://data.globe.gov/system/photos/2019/08/03/1155873/original.jpg	https://data.globe.gov/system/photos/2019/08/03/1155872/original.jpg	https://data.globe.gov/system/photos/2019/08/03/1155874/original.jpg	https://data.globe.gov/system/photos/2019/08/03/1155876/original.jpg
GV_1633	45.0307	-92.8346	286.5		https://www.google.com/maps/search/45.0307,-92.8346	https://data.globe.gov/system/photos/2019/08/03/1155877/original.jpg	https://data.globe.gov/system/photos/2019/08/03/1155885/original.jpg	https://data.globe.gov/system/photos/2019/08/03/1155882/original.jpg	https://data.globe.gov/system/photos/2019/08/03/1155886/original.jpg	https://data.globe.gov/system/photos/2019/08/03/1155888/original.jpg
GV_1634	-34.4169	138.863	231.9		https://www.google.com/maps/search/-34.4169,138.863	https://data.globe.gov/system/photos/2019/08/04/1155995/original.jpg	https://data.globe.gov/system/photos/2019/08/04/1155997/original.jpg	https://data.globe.gov/system/photos/2019/08/04/1155996/original.jpg	https://data.globe.gov/system/photos/2019/08/04/1155998/original.jpg	https://data.globe.gov/system/photos/2019/08/04/1156000/original.jpg
GV_1635	24.4496	118.3277	26		https://www.google.com/maps/search/24.4496,118.3277	https://data.globe.gov/system/photos/2019/08/04/1156014/original.jpg	https://data.globe.gov/system/photos/2019/08/04/1156016/original.jpg	https://data.globe.gov/system/photos/2019/08/04/1156015/original.jpg	https://data.globe.gov/system/photos/2019/08/04/1156017/original.jpg	https://data.globe.gov/system/photos/2019/08/04/1156019/original.jpg
GV_1636	24.4631	118.4173	202.7		https://www.google.com/maps/search/24.4631,118.4173	https://data.globe.gov/system/photos/2019/08/04/1156114/original.jpg	https://data.globe.gov/system/photos/2019/08/04/1156116/original.jpg	https://data.globe.gov/system/photos/2019/08/04/1156115/original.jpg	https://data.globe.gov/system/photos/2019/08/04/1156117/original.jpg	https://data.globe.gov/system/photos/2019/08/04/1156119/original.jpg
GV_1637	24.4618	118.4134	188.1		https://www.google.com/maps/search/24.4618,118.4134	https://data.globe.gov/system/photos/2019/08/04/1156120/original.jpg	https://data.globe.gov/system/photos/2019/08/04/1156122/original.jpg	https://data.globe.gov/system/photos/2019/08/04/1156121/original.jpg	https://data.globe.gov/system/photos/2019/08/04/1156123/original.jpg	https://data.globe.gov/system/photos/2019/08/04/1156125/original.jpg
GV_1638	24.46	118.4105	177.7		https://www.google.com/maps/search/24.46,118.4105	https://data.globe.gov/system/photos/2019/08/04/1156126/original.jpg	https://data.globe.gov/system/photos/2019/08/04/1156128/original.jpg	https://data.globe.gov/system/photos/2019/08/04/1156127/original.jpg	https://data.globe.gov/system/photos/2019/08/04/1156129/original.jpg	https://data.globe.gov/system/photos/2019/08/04/1156131/original.jpg
GV_1639	5.667	-0.1815	67.3		https://www.google.com/maps/search/5.667,-0.1815	https://data.globe.gov/system/photos/2019/08/04/1156222/original.jpg	https://data.globe.gov/system/photos/2019/08/04/1156224/original.jpg	https://data.globe.gov/system/photos/2019/08/04/1156223/original.jpg	https://data.globe.gov/system/photos/2019/08/04/1156225/original.jpg	https://data.globe.gov/system/photos/2019/08/04/1156227/original.jpg
GV_1640	45.1708	-110.8691	1539.4		https://www.google.com/maps/search/45.1708,-110.8691	https://data.globe.gov/system/photos/2019/07/30/1156282/original.jpg	https://data.globe.gov/system/photos/2019/07/30/1156284/original.jpg	https://data.globe.gov/system/photos/2019/07/30/1156283/original.jpg	https://data.globe.gov/system/photos/2019/07/30/1156285/original.jpg	https://data.globe.gov/system/photos/2019/07/30/1156287/original.jpg
GV_1641	44.915	-110.2752	1991.6		https://www.google.com/maps/search/44.915,-110.2752	https://data.globe.gov/system/photos/2019/07/30/1156288/original.jpg	https://data.globe.gov/system/photos/2019/07/30/1156290/original.jpg	https://data.globe.gov/system/photos/2019/07/30/1156289/original.jpg	https://data.globe.gov/system/photos/2019/07/30/1156291/original.jpg	https://data.globe.gov/system/photos/2019/07/30/1156293/original.jpg
GV_1642	44.8861	-110.2223	2004.4		https://www.google.com/maps/search/44.8861,-110.2223	https://data.globe.gov/system/photos/2019/07/30/1156294/original.jpg	https://data.globe.gov/system/photos/2019/07/30/1156296/original.jpg	https://data.globe.gov/system/photos/2019/07/30/1156295/original.jpg	https://data.globe.gov/system/photos/2019/07/30/1156297/original.jpg	https://data.globe.gov/system/photos/2019/07/30/1156299/original.jpg
GV_1643	44.8693	-110.1663	2029.6		https://www.google.com/maps/search/44.8693,-110.1663	https://data.globe.gov/system/photos/2019/07/30/1156300/original.jpg	https://data.globe.gov/system/photos/2019/07/30/1156302/original.jpg	https://data.globe.gov/system/photos/2019/07/30/1156301/original.jpg	https://data.globe.gov/system/photos/2019/07/30/1156303/original.jpg	https://data.globe.gov/system/photos/2019/07/30/1156305/original.jpg
GV_1644	45.3354	-110.7701	1492.7		https://www.google.com/maps/search/45.3354,-110.7701	https://data.globe.gov/system/photos/2019/07/30/1156306/original.jpg	https://data.globe.gov/system/photos/2019/07/30/1156308/original.jpg	https://data.globe.gov/system/photos/2019/07/30/1156307/original.jpg	https://data.globe.gov/system/photos/2019/07/30/1156309/original.jpg	https://data.globe.gov/system/photos/2019/07/30/1156313/original.jpg
GV_1645	5.6686	-0.1892	60.4		https://www.google.com/maps/search/5.6686,-0.1892	https://data.globe.gov/system/photos/2019/08/04/1156322/original.jpg	https://data.globe.gov/system/photos/2019/08/04/1156324/original.jpg	https://data.globe.gov/system/photos/2019/08/04/1156323/original.jpg	https://data.globe.gov/system/photos/2019/08/04/1156325/original.jpg	https://data.globe.gov/system/photos/2019/08/04/1156327/original.jpg
GV_1646	52.1289	-109.7742	697		https://www.google.com/maps/search/52.1289,-109.7742	https://data.globe.gov/system/photos/2019/08/02/1156364/original.jpg	https://data.globe.gov/system/photos/2019/08/02/1156373/original.jpg	https://data.globe.gov/system/photos/2019/08/02/1156366/original.jpg	https://data.globe.gov/system/photos/2019/08/02/1156376/original.jpg	https://data.globe.gov/system/photos/2019/08/02/1156380/original.jpg
GV_1647	31.8724	-106.5725	1202.4		https://www.google.com/maps/search/31.8724,-106.5725	https://data.globe.gov/system/photos/2019/08/05/1157367/original.jpg	https://data.globe.gov/system/photos/2019/08/05/1157369/original.jpg	https://data.globe.gov/system/photos/2019/08/05/1157368/original.jpg	https://data.globe.gov/system/photos/2019/08/05/1157370/original.jpg	https://data.globe.gov/system/photos/2019/08/05/1157372/original.jpg
GV_1648	31.8674	-106.5617	1221.3		https://www.google.com/maps/search/31.8674,-106.5617	https://data.globe.gov/system/photos/2019/08/05/1157422/original.jpg	https://data.globe.gov/system/photos/2019/08/05/1157424/original.jpg	https://data.globe.gov/system/photos/2019/08/05/1157423/original.jpg	https://data.globe.gov/system/photos/2019/08/05/1157425/original.jpg	https://data.globe.gov/system/photos/2019/08/05/1157427/original.jpg
GV_1649	47.5402	-111.7095	1040.5		https://www.google.com/maps/search/47.5402,-111.7095	https://data.globe.gov/system/photos/2019/08/05/1157436/original.jpg	https://data.globe.gov/system/photos/2019/08/05/1157439/original.jpg	https://data.globe.gov/system/photos/2019/08/05/1157437/original.jpg	https://data.globe.gov/system/photos/2019/08/05/1157440/original.jpg	https://data.globe.gov/system/photos/2019/08/05/1157442/original.jpg
GV_1650	47.5	-111.8191	1073.4		https://www.google.com/maps/search/47.5,-111.8191	https://data.globe.gov/system/photos/2019/08/05/1157443/original.jpg	https://data.globe.gov/system/photos/2019/08/05/1157445/original.jpg	https://data.globe.gov/system/photos/2019/08/05/1157444/original.jpg	https://data.globe.gov/system/photos/2019/08/05/1157446/original.jpg	https://data.globe.gov/system/photos/2019/08/05/1157448/original.jpg
GV_1651	47.3356	-112.077	1247.3		https://www.google.com/maps/search/47.3356,-112.077	https://data.globe.gov/system/photos/2019/08/05/1157449/original.jpg	https://data.globe.gov/system/photos/2019/08/05/1157451/original.jpg	https://data.globe.gov/system/photos/2019/08/05/1157450/original.jpg	https://data.globe.gov/system/photos/2019/08/05/1157452/original.jpg	https://data.globe.gov/system/photos/2019/08/05/1157454/original.jpg
GV_1652	47.0762	-112.3711	1714.7		https://www.google.com/maps/search/47.0762,-112.3711	https://data.globe.gov/system/photos/2019/08/05/1157455/original.jpg	https://data.globe.gov/system/photos/2019/08/05/1157457/original.jpg	https://data.globe.gov/system/photos/2019/08/05/1157456/original.jpg	https://data.globe.gov/system/photos/2019/08/05/1157458/original.jpg	https://data.globe.gov/system/photos/2019/08/05/1157460/original.jpg
GV_1653	47.0762	-112.3709	1710.9		https://www.google.com/maps/search/47.0762,-112.3709	https://data.globe.gov/system/photos/2019/08/05/1157461/original.jpg	https://data.globe.gov/system/photos/2019/08/05/1157463/original.jpg	https://data.globe.gov/system/photos/2019/08/05/1157462/original.jpg	https://data.globe.gov/system/photos/2019/08/05/1157464/original.jpg	https://data.globe.gov/system/photos/2019/08/05/1157466/original.jpg
GV_1654	47.102	-112.4795	1645.8		https://www.google.com/maps/search/47.102,-112.4795	https://data.globe.gov/system/photos/2019/08/05/1157467/original.jpg	https://data.globe.gov/system/photos/2019/08/05/1157470/original.jpg	https://data.globe.gov/system/photos/2019/08/05/1157468/original.jpg	https://data.globe.gov/system/photos/2019/08/05/1157471/original.jpg	https://data.globe.gov/system/photos/2019/08/05/1157473/original.jpg
GV_1655	47.1379	-112.4579	1698		https://www.google.com/maps/search/47.1379,-112.4579	https://data.globe.gov/system/photos/2019/08/05/1157474/original.jpg	https://data.globe.gov/system/photos/2019/08/05/1157476/original.jpg	https://data.globe.gov/system/photos/2019/08/05/1157475/original.jpg	https://data.globe.gov/system/photos/2019/08/05/1157477/original.jpg	https://data.globe.gov/system/photos/2019/08/05/1157479/original.jpg
GV_1656	47.1393	-112.456	1706.4		https://www.google.com/maps/search/47.1393,-112.456	https://data.globe.gov/system/photos/2019/08/05/1157480/original.jpg	https://data.globe.gov/system/photos/2019/08/05/1157482/original.jpg	https://data.globe.gov/system/photos/2019/08/05/1157481/original.jpg	https://data.globe.gov/system/photos/2019/08/05/1157483/original.jpg	https://data.globe.gov/system/photos/2019/08/05/1157485/original.jpg
GV_1657	47.0019	-113.3701	1167.8		https://www.google.com/maps/search/47.0019,-113.3701	https://data.globe.gov/system/photos/2019/08/05/1157486/original.jpg	https://data.globe.gov/system/photos/2019/08/05/1157488/original.jpg	https://data.globe.gov/system/photos/2019/08/05/1157487/original.jpg	https://data.globe.gov/system/photos/2019/08/05/1157489/original.jpg	https://data.globe.gov/system/photos/2019/08/05/1157491/original.jpg
GV_1658	46.7553	-114.0949	976.8		https://www.google.com/maps/search/46.7553,-114.0949	https://data.globe.gov/system/photos/2019/08/05/1157492/original.jpg	https://data.globe.gov/system/photos/2019/08/05/1157494/original.jpg	https://data.globe.gov/system/photos/2019/08/05/1157493/original.jpg	https://data.globe.gov/system/photos/2019/08/05/1157495/original.jpg	https://data.globe.gov/system/photos/2019/08/05/1157497/original.jpg
GV_1659	46.7519	-114.0891	970.2		https://www.google.com/maps/search/46.7519,-114.0891	https://data.globe.gov/system/photos/2019/08/05/1157498/original.jpg	https://data.globe.gov/system/photos/2019/08/05/1157500/original.jpg	https://data.globe.gov/system/photos/2019/08/05/1157499/original.jpg	https://data.globe.gov/system/photos/2019/08/05/1157501/original.jpg	https://data.globe.gov/system/photos/2019/08/05/1157503/original.jpg
GV_1660	46.6346	-114.5774	1594.2		https://www.google.com/maps/search/46.6346,-114.5774	https://data.globe.gov/system/photos/2019/08/05/1157511/original.jpg	https://data.globe.gov/system/photos/2019/08/05/1157513/original.jpg	https://data.globe.gov/system/photos/2019/08/05/1157512/original.jpg	https://data.globe.gov/system/photos/2019/08/05/1157514/original.jpg	https://data.globe.gov/system/photos/2019/08/05/1157516/original.jpg
GV_1661	46.6342	-114.5768	1592.4		https://www.google.com/maps/search/46.6342,-114.5768	https://data.globe.gov/system/photos/2019/08/05/1157517/original.jpg	https://data.globe.gov/system/photos/2019/08/05/1157519/original.jpg	https://data.globe.gov/system/photos/2019/08/05/1157518/original.jpg	https://data.globe.gov/system/photos/2019/08/05/1157520/original.jpg	https://data.globe.gov/system/photos/2019/08/05/1157522/original.jpg
GV_1662	53.4285	-0.6158	33.1		https://www.google.com/maps/search/53.4285,-0.6158	https://data.globe.gov/system/photos/2019/08/03/1157537/original.jpg	https://data.globe.gov/system/photos/2019/08/03/1157539/original.jpg	https://data.globe.gov/system/photos/2019/08/03/1157538/original.jpg	https://data.globe.gov/system/photos/2019/08/03/1157540/original.jpg	https://data.globe.gov/system/photos/2019/08/03/1157542/original.jpg
GV_1663	53.4283	-0.6226	26		https://www.google.com/maps/search/53.4283,-0.6226	https://data.globe.gov/system/photos/2019/08/05/1157552/original.jpg	https://data.globe.gov/system/photos/2019/08/05/1157554/original.jpg	https://data.globe.gov/system/photos/2019/08/05/1157553/original.jpg	https://data.globe.gov/system/photos/2019/08/05/1157555/original.jpg	https://data.globe.gov/system/photos/2019/08/05/1157558/original.jpg
GV_1664	48.2361	-101.2992	474.3		https://www.google.com/maps/search/48.2361,-101.2992	https://data.globe.gov/system/photos/2019/08/05/1157604/original.jpg	https://data.globe.gov/system/photos/2019/08/05/1157606/original.jpg	https://data.globe.gov/system/photos/2019/08/05/1157605/original.jpg	https://data.globe.gov/system/photos/2019/08/05/1157607/original.jpg	https://data.globe.gov/system/photos/2019/08/05/1157609/original.jpg
GV_1665	48.2369	-101.297	473.7		https://www.google.com/maps/search/48.2369,-101.297	https://data.globe.gov/system/photos/2019/08/05/1157610/original.jpg	https://data.globe.gov/system/photos/2019/08/05/1157612/original.jpg	https://data.globe.gov/system/photos/2019/08/05/1157611/original.jpg	https://data.globe.gov/system/photos/2019/08/05/1157613/original.jpg	https://data.globe.gov/system/photos/2019/08/05/1157615/original.jpg
GV_1666	48.2358	-101.2993	474.7		https://www.google.com/maps/search/48.2358,-101.2993	https://data.globe.gov/system/photos/2019/08/05/1157616/original.jpg	https://data.globe.gov/system/photos/2019/08/05/1157618/original.jpg	https://data.globe.gov/system/photos/2019/08/05/1157617/original.jpg	https://data.globe.gov/system/photos/2019/08/05/1157619/original.jpg	https://data.globe.gov/system/photos/2019/08/05/1157621/original.jpg
GV_1667	48.5066	-111.8568	1000.1		https://www.google.com/maps/search/48.5066,-111.8568	https://data.globe.gov/system/photos/2019/08/05/1157622/original.jpg	https://data.globe.gov/system/photos/2019/08/05/1157624/original.jpg	https://data.globe.gov/system/photos/2019/08/05/1157623/original.jpg	https://data.globe.gov/system/photos/2019/08/05/1157625/original.jpg	https://data.globe.gov/system/photos/2019/08/05/1157627/original.jpg
GV_1668	48.5068	-111.8567	998.8		https://www.google.com/maps/search/48.5068,-111.8567	https://data.globe.gov/system/photos/2019/08/05/1157628/original.jpg	https://data.globe.gov/system/photos/2019/08/05/1157630/original.jpg	https://data.globe.gov/system/photos/2019/08/05/1157629/original.jpg	https://data.globe.gov/system/photos/2019/08/05/1157631/original.jpg	https://data.globe.gov/system/photos/2019/08/05/1157633/original.jpg
GV_1669	-34.4561	138.9274	270		https://www.google.com/maps/search/-34.4561,138.9274	https://data.globe.gov/system/photos/2019/08/06/1157825/original.jpg	https://data.globe.gov/system/photos/2019/08/06/1157827/original.jpg	https://data.globe.gov/system/photos/2019/08/06/1157826/original.jpg	https://data.globe.gov/system/photos/2019/08/06/1157828/original.jpg	https://data.globe.gov/system/photos/2019/08/06/1157830/original.jpg
GV_1670	-27.2917	133.7028	320.7		https://www.google.com/maps/search/-27.2917,133.7028	https://data.globe.gov/system/photos/2019/08/05/1157876/original.jpg	https://data.globe.gov/system/photos/2019/08/05/1157878/original.jpg	https://data.globe.gov/system/photos/2019/08/05/1157877/original.jpg	https://data.globe.gov/system/photos/2019/08/05/1157880/original.jpg	https://data.globe.gov/system/photos/2019/08/05/1157882/original.jpg
GV_1671	48.4423	-113.2198	1464.2		https://www.google.com/maps/search/48.4423,-113.2198	https://data.globe.gov/system/photos/2019/08/06/1158226/original.jpg	https://data.globe.gov/system/photos/2019/08/06/1158228/original.jpg	https://data.globe.gov/system/photos/2019/08/06/1158227/original.jpg	https://data.globe.gov/system/photos/2019/08/06/1158230/original.jpg	https://data.globe.gov/system/photos/2019/08/06/1158232/original.jpg
GV_1672	48.4649	-112.781	1263.8		https://www.google.com/maps/search/48.4649,-112.781	https://data.globe.gov/system/photos/2019/08/06/1158233/original.jpg	https://data.globe.gov/system/photos/2019/08/06/1158235/original.jpg	https://data.globe.gov/system/photos/2019/08/06/1158234/original.jpg	https://data.globe.gov/system/photos/2019/08/06/1158236/original.jpg	https://data.globe.gov/system/photos/2019/08/06/1158238/original.jpg
GV_1673	48.1984	-112.5073	1253.6		https://www.google.com/maps/search/48.1984,-112.5073	https://data.globe.gov/system/photos/2019/08/06/1158262/original.jpg	https://data.globe.gov/system/photos/2019/08/06/1158264/original.jpg	https://data.globe.gov/system/photos/2019/08/06/1158263/original.jpg	https://data.globe.gov/system/photos/2019/08/06/1158265/original.jpg	https://data.globe.gov/system/photos/2019/08/06/1158267/original.jpg
GV_1674	48.1446	-112.4122	1273.7		https://www.google.com/maps/search/48.1446,-112.4122	https://data.globe.gov/system/photos/2019/08/06/1158268/original.jpg	https://data.globe.gov/system/photos/2019/08/06/1158270/original.jpg	https://data.globe.gov/system/photos/2019/08/06/1158269/original.jpg	https://data.globe.gov/system/photos/2019/08/06/1158271/original.jpg	https://data.globe.gov/system/photos/2019/08/06/1158273/original.jpg
GV_1675	48.1443	-112.4115	1275.6		https://www.google.com/maps/search/48.1443,-112.4115	https://data.globe.gov/system/photos/2019/08/06/1158274/original.jpg	https://data.globe.gov/system/photos/2019/08/06/1158276/original.jpg	https://data.globe.gov/system/photos/2019/08/06/1158275/original.jpg	https://data.globe.gov/system/photos/2019/08/06/1158277/original.jpg	https://data.globe.gov/system/photos/2019/08/06/1158279/original.jpg
GV_1676	48.0965	-112.3516	1342.4		https://www.google.com/maps/search/48.0965,-112.3516	https://data.globe.gov/system/photos/2019/08/06/1158286/original.jpg	https://data.globe.gov/system/photos/2019/08/06/1158288/original.jpg	https://data.globe.gov/system/photos/2019/08/06/1158287/original.jpg	https://data.globe.gov/system/photos/2019/08/06/1158289/original.jpg	https://data.globe.gov/system/photos/2019/08/06/1158291/original.jpg
GV_1677	45.8952	-111.5527	1239.4		https://www.google.com/maps/search/45.8952,-111.5527	https://data.globe.gov/system/photos/2019/08/06/1158298/original.jpg	https://data.globe.gov/system/photos/2019/08/06/1158300/original.jpg	https://data.globe.gov/system/photos/2019/08/06/1158299/original.jpg	https://data.globe.gov/system/photos/2019/08/06/1158301/original.jpg	https://data.globe.gov/system/photos/2019/08/06/1158303/original.jpg
GV_1678	45.8953	-111.5525	1240.1		https://www.google.com/maps/search/45.8953,-111.5525	https://data.globe.gov/system/photos/2019/08/06/1158304/original.jpg	https://data.globe.gov/system/photos/2019/08/06/1158306/original.jpg	https://data.globe.gov/system/photos/2019/08/06/1158305/original.jpg	https://data.globe.gov/system/photos/2019/08/06/1158307/original.jpg	https://data.globe.gov/system/photos/2019/08/06/1158309/original.jpg
GV_1679	45.8931	-111.5525	1241.1		https://www.google.com/maps/search/45.8931,-111.5525	https://data.globe.gov/system/photos/2019/08/06/1158310/original.jpg	https://data.globe.gov/system/photos/2019/08/06/1158312/original.jpg	https://data.globe.gov/system/photos/2019/08/06/1158311/original.jpg	https://data.globe.gov/system/photos/2019/08/06/1158313/original.jpg	https://data.globe.gov/system/photos/2019/08/06/1158315/original.jpg
GV_1680	45.9205	-111.4988	1233.6		https://www.google.com/maps/search/45.9205,-111.4988	https://data.globe.gov/system/photos/2019/08/06/1158316/original.jpg	https://data.globe.gov/system/photos/2019/08/06/1158318/original.jpg	https://data.globe.gov/system/photos/2019/08/06/1158317/original.jpg	https://data.globe.gov/system/photos/2019/08/06/1158319/original.jpg	https://data.globe.gov/system/photos/2019/08/06/1158321/original.jpg
GV_1681	45.9261	-111.5033	1232.8		https://www.google.com/maps/search/45.9261,-111.5033	https://data.globe.gov/system/photos/2019/08/06/1158322/original.jpg	https://data.globe.gov/system/photos/2019/08/06/1158324/original.jpg	https://data.globe.gov/system/photos/2019/08/06/1158323/original.jpg	https://data.globe.gov/system/photos/2019/08/06/1158325/original.jpg	https://data.globe.gov/system/photos/2019/08/06/1158327/original.jpg
GV_1682	45.9258	-111.5035	1232		https://www.google.com/maps/search/45.9258,-111.5035	https://data.globe.gov/system/photos/2019/08/06/1158328/original.jpg	https://data.globe.gov/system/photos/2019/08/06/1158330/original.jpg	https://data.globe.gov/system/photos/2019/08/06/1158329/original.jpg	https://data.globe.gov/system/photos/2019/08/06/1158331/original.jpg	https://data.globe.gov/system/photos/2019/08/06/1158335/original.jpg
GV_1683	45.9264	-111.5039	1232.1		https://www.google.com/maps/search/45.9264,-111.5039	https://data.globe.gov/system/photos/2019/08/06/1158336/original.jpg	https://data.globe.gov/system/photos/2019/08/06/1158339/original.jpg	https://data.globe.gov/system/photos/2019/08/06/1158337/original.jpg	https://data.globe.gov/system/photos/2019/08/06/1158341/original.jpg	https://data.globe.gov/system/photos/2019/08/06/1158344/original.jpg
GV_1684	45.9334	-111.4943	1239.1		https://www.google.com/maps/search/45.9334,-111.4943	https://data.globe.gov/system/photos/2019/08/06/1158376/original.jpg	https://data.globe.gov/system/photos/2019/08/06/1158384/original.jpg	https://data.globe.gov/system/photos/2019/08/06/1158382/original.jpg	https://data.globe.gov/system/photos/2019/08/06/1158385/original.jpg	https://data.globe.gov/system/photos/2019/08/06/1158387/original.jpg
GV_1685	45.9334	-111.4943	1239.1		https://www.google.com/maps/search/45.9334,-111.4943	https://data.globe.gov/system/photos/2019/08/06/1158388/original.jpg	https://data.globe.gov/system/photos/2019/08/06/1158390/original.jpg	https://data.globe.gov/system/photos/2019/08/06/1158389/original.jpg	https://data.globe.gov/system/photos/2019/08/06/1158391/original.jpg	https://data.globe.gov/system/photos/2019/08/06/1158393/original.jpg
GV_1686	45.9336	-111.4934	1242.8		https://www.google.com/maps/search/45.9336,-111.4934	https://data.globe.gov/system/photos/2019/08/06/1158412/original.jpg	https://data.globe.gov/system/photos/2019/08/06/1158414/original.jpg	https://data.globe.gov/system/photos/2019/08/06/1158413/original.jpg	https://data.globe.gov/system/photos/2019/08/06/1158415/original.jpg	https://data.globe.gov/system/photos/2019/08/06/1158417/original.jpg
GV_1687	45.9401	-111.4907	1229.5		https://www.google.com/maps/search/45.9401,-111.4907	https://data.globe.gov/system/photos/2019/08/06/1158418/original.jpg	https://data.globe.gov/system/photos/2019/08/06/1158420/original.jpg	https://data.globe.gov/system/photos/2019/08/06/1158419/original.jpg	https://data.globe.gov/system/photos/2019/08/06/1158422/original.jpg	https://data.globe.gov/system/photos/2019/08/06/1158425/original.jpg
GV_1688	45.9355	-111.495	1231.9		https://www.google.com/maps/search/45.9355,-111.495	https://data.globe.gov/system/photos/2019/08/06/1158429/original.jpg	https://data.globe.gov/system/photos/2019/08/06/1158432/original.jpg	https://data.globe.gov/system/photos/2019/08/06/1158431/original.jpg	https://data.globe.gov/system/photos/2019/08/06/1158433/original.jpg	https://data.globe.gov/system/photos/2019/08/06/1158435/original.jpg
GV_1689	45.9384	-111.4935	1229.8		https://www.google.com/maps/search/45.9384,-111.4935	https://data.globe.gov/system/photos/2019/08/06/1158442/original.jpg	https://data.globe.gov/system/photos/2019/08/06/1158444/original.jpg	https://data.globe.gov/system/photos/2019/08/06/1158443/original.jpg	https://data.globe.gov/system/photos/2019/08/06/1158445/original.jpg	https://data.globe.gov/system/photos/2019/08/06/1158447/original.jpg
GV_1690	45.9376	-111.4939	1232		https://www.google.com/maps/search/45.9376,-111.4939	https://data.globe.gov/system/photos/2019/08/06/1158448/original.jpg	https://data.globe.gov/system/photos/2019/08/06/1158450/original.jpg	https://data.globe.gov/system/photos/2019/08/06/1158449/original.jpg	https://data.globe.gov/system/photos/2019/08/06/1158451/original.jpg	https://data.globe.gov/system/photos/2019/08/06/1158458/original.jpg
GV_1691	45.9198	-111.4992	1233		https://www.google.com/maps/search/45.9198,-111.4992	https://data.globe.gov/system/photos/2019/08/06/1158464/original.jpg	https://data.globe.gov/system/photos/2019/08/06/1158472/original.jpg	https://data.globe.gov/system/photos/2019/08/06/1158467/original.jpg	https://data.globe.gov/system/photos/2019/08/06/1158474/original.jpg	https://data.globe.gov/system/photos/2019/08/06/1158476/original.jpg
GV_1692	45.9204	-111.5001	1232.9		https://www.google.com/maps/search/45.9204,-111.5001	https://data.globe.gov/system/photos/2019/08/06/1158477/original.jpg	https://data.globe.gov/system/photos/2019/08/06/1158479/original.jpg	https://data.globe.gov/system/photos/2019/08/06/1158478/original.jpg	https://data.globe.gov/system/photos/2019/08/06/1158480/original.jpg	https://data.globe.gov/system/photos/2019/08/06/1158482/original.jpg
GV_1693	45.6586	-111.0449	1506.3		https://www.google.com/maps/search/45.6586,-111.0449	https://data.globe.gov/system/photos/2019/08/06/1158483/original.jpg	https://data.globe.gov/system/photos/2019/08/06/1158485/original.jpg	https://data.globe.gov/system/photos/2019/08/06/1158484/original.jpg	https://data.globe.gov/system/photos/2019/08/06/1158486/original.jpg	https://data.globe.gov/system/photos/2019/08/06/1158488/original.jpg
GV_1694	45.6792	-111.0356	1467.7		https://www.google.com/maps/search/45.6792,-111.0356	https://data.globe.gov/system/photos/2019/08/06/1158489/original.jpg	https://data.globe.gov/system/photos/2019/08/06/1158491/original.jpg	https://data.globe.gov/system/photos/2019/08/06/1158490/original.jpg	https://data.globe.gov/system/photos/2019/08/06/1158492/original.jpg	https://data.globe.gov/system/photos/2019/08/06/1158494/original.jpg
GV_1695	45.6661	-111.0587	1490.3		https://www.google.com/maps/search/45.6661,-111.0587	https://data.globe.gov/system/photos/2019/08/06/1158723/original.jpg	https://data.globe.gov/system/photos/2019/08/06/1158725/original.jpg	https://data.globe.gov/system/photos/2019/08/06/1158724/original.jpg	https://data.globe.gov/system/photos/2019/08/06/1158726/original.jpg	https://data.globe.gov/system/photos/2019/08/06/1158728/original.jpg
GV_1696	45.6652	-111.0601	1491		https://www.google.com/maps/search/45.6652,-111.0601	https://data.globe.gov/system/photos/2019/08/06/1158729/original.jpg	https://data.globe.gov/system/photos/2019/08/06/1158731/original.jpg	https://data.globe.gov/system/photos/2019/08/06/1158730/original.jpg	https://data.globe.gov/system/photos/2019/08/06/1158732/original.jpg	https://data.globe.gov/system/photos/2019/08/06/1158734/original.jpg
GV_1697	45.6656	-111.0561	1490		https://www.google.com/maps/search/45.6656,-111.0561	https://data.globe.gov/system/photos/2019/08/06/1158735/original.jpg	https://data.globe.gov/system/photos/2019/08/06/1158737/original.jpg	https://data.globe.gov/system/photos/2019/08/06/1158736/original.jpg	https://data.globe.gov/system/photos/2019/08/06/1158738/original.jpg	https://data.globe.gov/system/photos/2019/08/06/1158740/original.jpg
GV_1698	45.666	-111.0557	1489.4		https://www.google.com/maps/search/45.666,-111.0557	https://data.globe.gov/system/photos/2019/08/06/1158747/original.jpg	https://data.globe.gov/system/photos/2019/08/06/1158749/original.jpg	https://data.globe.gov/system/photos/2019/08/06/1158748/original.jpg	https://data.globe.gov/system/photos/2019/08/06/1158750/original.jpg	https://data.globe.gov/system/photos/2019/08/06/1158752/original.jpg
GV_1699	45.6649	-111.0583	1491.9		https://www.google.com/maps/search/45.6649,-111.0583	https://data.globe.gov/system/photos/2019/08/06/1158753/original.jpg	https://data.globe.gov/system/photos/2019/08/06/1158755/original.jpg	https://data.globe.gov/system/photos/2019/08/06/1158754/original.jpg	https://data.globe.gov/system/photos/2019/08/06/1158756/original.jpg	https://data.globe.gov/system/photos/2019/08/06/1158758/original.jpg
GV_1700	46.5353	-114.0935	994.9		https://www.google.com/maps/search/46.5353,-114.0935	https://data.globe.gov/system/photos/2019/08/06/1158983/original.jpg	https://data.globe.gov/system/photos/2019/08/06/1158985/original.jpg	https://data.globe.gov/system/photos/2019/08/06/1158984/original.jpg	https://data.globe.gov/system/photos/2019/08/06/1158986/original.jpg	https://data.globe.gov/system/photos/2019/08/06/1158988/original.jpg
GV_1701	46.5405	-114.0938	993.9		https://www.google.com/maps/search/46.5405,-114.0938	https://data.globe.gov/system/photos/2019/08/06/1158989/original.jpg	https://data.globe.gov/system/photos/2019/08/06/1158991/original.jpg	https://data.globe.gov/system/photos/2019/08/06/1158990/original.jpg	https://data.globe.gov/system/photos/2019/08/06/1158992/original.jpg	https://data.globe.gov/system/photos/2019/08/06/1158994/original.jpg
GV_1702	46.5398	-114.0949	993.8		https://www.google.com/maps/search/46.5398,-114.0949	https://data.globe.gov/system/photos/2019/08/06/1158995/original.jpg	https://data.globe.gov/system/photos/2019/08/06/1158997/original.jpg	https://data.globe.gov/system/photos/2019/08/06/1158996/original.jpg	https://data.globe.gov/system/photos/2019/08/06/1159002/original.jpg	https://data.globe.gov/system/photos/2019/08/06/1159006/original.jpg
GV_1703	46.5392	-114.0954	994.1		https://www.google.com/maps/search/46.5392,-114.0954	https://data.globe.gov/system/photos/2019/08/06/1159007/original.jpg	https://data.globe.gov/system/photos/2019/08/06/1159009/original.jpg	https://data.globe.gov/system/photos/2019/08/06/1159008/original.jpg	https://data.globe.gov/system/photos/2019/08/06/1159010/original.jpg	https://data.globe.gov/system/photos/2019/08/06/1159012/original.jpg

GV_1704	46.5383	-114.0963	993.9		https://www.google.com/maps/search/46.5383,-114.0963	https://data.globe.gov/system/photos/2019/08/06/1159013/original.jpg	https://data.globe.gov/system/photos/2019/08/06/1159015/original.jpg	https://data.globe.gov/system/photos/2019/08/06/1159014/original.jpg	https://data.globe.gov/system/photos/2019/08/06/1159016/original.jpg	https://data.globe.gov/system/photos/2019/08/06/1159018/original.jpg
GV_1705	45.9814	-114.1596	1206.5		https://www.google.com/maps/search/45.9814,-114.1596	https://data.globe.gov/system/photos/2019/08/06/1159019/original.jpg	https://data.globe.gov/system/photos/2019/08/06/1159021/original.jpg	https://data.globe.gov/system/photos/2019/08/06/1159020/original.jpg	https://data.globe.gov/system/photos/2019/08/06/1159022/original.jpg	https://data.globe.gov/system/photos/2019/08/06/1159125/original.jpg
GV_1706	45.8363	-113.9813	1348.9		https://www.google.com/maps/search/45.8363,-113.9813	https://data.globe.gov/system/photos/2019/08/06/1159126/original.jpg	https://data.globe.gov/system/photos/2019/08/06/1159128/original.jpg	https://data.globe.gov/system/photos/2019/08/06/1159127/original.jpg	https://data.globe.gov/system/photos/2019/08/06/1159129/original.jpg	https://data.globe.gov/system/photos/2019/08/06/1159131/original.jpg
GV_1707	45.8367	-113.9729	1355		https://www.google.com/maps/search/45.8367,-113.9729	https://data.globe.gov/system/photos/2019/08/06/1159132/original.jpg	https://data.globe.gov/system/photos/2019/08/06/1159134/original.jpg	https://data.globe.gov/system/photos/2019/08/06/1159133/original.jpg	https://data.globe.gov/system/photos/2019/08/06/1159135/original.jpg	https://data.globe.gov/system/photos/2019/08/06/1159137/original.jpg
GV_1708	45.167	-113.8626	1221.6		https://www.google.com/maps/search/45.167,-113.8626	https://data.globe.gov/system/photos/2019/08/06/1159138/original.jpg	https://data.globe.gov/system/photos/2019/08/06/1159140/original.jpg	https://data.globe.gov/system/photos/2019/08/06/1159139/original.jpg	https://data.globe.gov/system/photos/2019/08/06/1159141/original.jpg	https://data.globe.gov/system/photos/2019/08/06/1159143/original.jpg
GV_1709	45.1659	-113.8644	1219.7		https://www.google.com/maps/search/45.1659,-113.8644	https://data.globe.gov/system/photos/2019/08/06/1159144/original.jpg	https://data.globe.gov/system/photos/2019/08/06/1159146/original.jpg	https://data.globe.gov/system/photos/2019/08/06/1159145/original.jpg	https://data.globe.gov/system/photos/2019/08/06/1159147/original.jpg	https://data.globe.gov/system/photos/2019/08/06/1159149/original.jpg
GV_1710	45.168	-113.8621	1219.6		https://www.google.com/maps/search/45.168,-113.8621	https://data.globe.gov/system/photos/2019/08/06/1159150/original.jpg	https://data.globe.gov/system/photos/2019/08/06/1159152/original.jpg	https://data.globe.gov/system/photos/2019/08/06/1159151/original.jpg	https://data.globe.gov/system/photos/2019/08/06/1159153/original.jpg	https://data.globe.gov/system/photos/2019/08/06/1159155/original.jpg
GV_1711	45.1677	-113.8639	1217.4		https://www.google.com/maps/search/45.1677,-113.8639	https://data.globe.gov/system/photos/2019/08/06/1159156/original.jpg	https://data.globe.gov/system/photos/2019/08/06/1159158/original.jpg	https://data.globe.gov/system/photos/2019/08/06/1159157/original.jpg	https://data.globe.gov/system/photos/2019/08/06/1159159/original.jpg	https://data.globe.gov/system/photos/2019/08/06/1159161/original.jpg
GV_1712	45.1673	-113.8666	1216.1		https://www.google.com/maps/search/45.1673,-113.8666	https://data.globe.gov/system/photos/2019/08/06/1159162/original.jpg	https://data.globe.gov/system/photos/2019/08/06/1159164/original.jpg	https://data.globe.gov/system/photos/2019/08/06/1159163/original.jpg	https://data.globe.gov/system/photos/2019/08/06/1159165/original.jpg	https://data.globe.gov/system/photos/2019/08/06/1159167/original.jpg
GV_1713	-34.3464	138.9457	234.5		https://www.google.com/maps/search/-34.3464,138.9457	https://data.globe.gov/system/photos/2019/08/07/1159085/original.jpg	https://data.globe.gov/system/photos/2019/08/07/1159087/original.jpg	https://data.globe.gov/system/photos/2019/08/07/1159086/original.jpg	https://data.globe.gov/system/photos/2019/08/07/1159088/original.jpg	https://data.globe.gov/system/photos/2019/08/07/1159090/original.jpg
GV_1714	64.7584	26.4272	74		https://www.google.com/maps/search/64.7584,26.4272	https://data.globe.gov/system/photos/2019/08/07/1159277/original.jpg	https://data.globe.gov/system/photos/2019/08/07/1159279/original.jpg	https://data.globe.gov/system/photos/2019/08/07/1159278/original.jpg	https://data.globe.gov/system/photos/2019/08/07/1159280/original.jpg	https://data.globe.gov/system/photos/2019/08/07/1159282/original.jpg
GV_1715	45.8246	-109.9652	1250.9		https://www.google.com/maps/search/45.8246,-109.9652	https://data.globe.gov/system/photos/2019/08/07/1159331/original.jpg	https://data.globe.gov/system/photos/2019/08/07/1159333/original.jpg	https://data.globe.gov/system/photos/2019/08/07/1159332/original.jpg	https://data.globe.gov/system/photos/2019/08/07/1159334/original.jpg	https://data.globe.gov/system/photos/2019/08/07/1159336/original.jpg
GV_1716	45.6615	-110.7004	1527.4		https://www.google.com/maps/search/45.6615,-110.7004	https://data.globe.gov/system/photos/2019/08/07/1159540/original.jpg	https://data.globe.gov/system/photos/2019/08/07/1159542/original.jpg	https://data.globe.gov/system/photos/2019/08/07/1159541/original.jpg	https://data.globe.gov/system/photos/2019/08/07/1159543/original.jpg	https://data.globe.gov/system/photos/2019/08/07/1159546/original.jpg
GV_1717	45.6614	-110.7011	1527.4		https://www.google.com/maps/search/45.6614,-110.7011	https://data.globe.gov/system/photos/2019/08/07/1159547/original.jpg	https://data.globe.gov/system/photos/2019/08/07/1159549/original.jpg	https://data.globe.gov/system/photos/2019/08/07/1159548/original.jpg	https://data.globe.gov/system/photos/2019/08/07/1159551/original.jpg	https://data.globe.gov/system/photos/2019/08/07/1159553/original.jpg
GV_1718	45.8245	-109.9656	1251		https://www.google.com/maps/search/45.8245,-109.9656	https://data.globe.gov/system/photos/2019/08/07/1159554/original.jpg	https://data.globe.gov/system/photos/2019/08/07/1159557/original.jpg	https://data.globe.gov/system/photos/2019/08/07/1159556/original.jpg	https://data.globe.gov/system/photos/2019/08/07/1159558/original.jpg	https://data.globe.gov/system/photos/2019/08/07/1159560/original.jpg
GV_1719	45.788	-110.0663	1250.1		https://www.google.com/maps/search/45.788,-110.0663	https://data.globe.gov/system/photos/2019/08/07/1159561/original.jpg	https://data.globe.gov/system/photos/2019/08/07/1159564/original.jpg	https://data.globe.gov/system/photos/2019/08/07/1159562/original.jpg	https://data.globe.gov/system/photos/2019/08/07/1159565/original.jpg	https://data.globe.gov/system/photos/2019/08/07/1159567/original.jpg
GV_1720	45.7877	-110.0661	1250.4		https://www.google.com/maps/search/45.7877,-110.0661	https://data.globe.gov/system/photos/2019/08/07/1159568/original.jpg	https://data.globe.gov/system/photos/2019/08/07/1159570/original.jpg	https://data.globe.gov/system/photos/2019/08/07/1159569/original.jpg	https://data.globe.gov/system/photos/2019/08/07/1159572/original.jpg	https://data.globe.gov/system/photos/2019/08/07/1159574/original.jpg
GV_1721	45.6677	-110.8073	1743.2		https://www.google.com/maps/search/45.6677,-110.8073	https://data.globe.gov/system/photos/2019/08/07/1159577/original.jpg	https://data.globe.gov/system/photos/2019/08/07/1159579/original.jpg	https://data.globe.gov/system/photos/2019/08/07/1159578/original.jpg	https://data.globe.gov/system/photos/2019/08/07/1159580/original.jpg	https://data.globe.gov/system/photos/2019/08/07/1159583/original.jpg
GV_1722	45.6651	-111.047	1498.9		https://www.google.com/maps/search/45.6651,-111.047	https://data.globe.gov/system/photos/2019/08/07/1159584/original.jpg	https://data.globe.gov/system/photos/2019/08/07/1159586/original.jpg	https://data.globe.gov/system/photos/2019/08/07/1159585/original.jpg	https://data.globe.gov/system/photos/2019/08/07/1159587/original.jpg	https://data.globe.gov/system/photos/2019/08/07/1159590/original.jpg
GV_1723	38.854	-77.1526	67.2		https://www.google.com/maps/search/38.854,-77.1526	https://data.globe.gov/system/photos/2019/08/07/1159746/original.jpg	https://data.globe.gov/system/photos/2019/08/07/1159748/original.jpg	https://data.globe.gov/system/photos/2019/08/07/1159747/original.jpg	https://data.globe.gov/system/photos/2019/08/07/1159749/original.jpg	https://data.globe.gov/system/photos/2019/08/07/1159751/original.jpg
GV_1724	46.8315	-111.9517	1092		https://www.google.com/maps/search/46.8315,-111.9517	https://data.globe.gov/system/photos/2019/08/07/1159778/original.jpg	https://data.globe.gov/system/photos/2019/08/07/1159780/original.jpg	https://data.globe.gov/system/photos/2019/08/07/1159779/original.jpg	https://data.globe.gov/system/photos/2019/08/07/1159781/original.jpg	https://data.globe.gov/system/photos/2019/08/07/1159783/original.jpg
GV_1725	45.4446	-111.0038	3088.6		https://www.google.com/maps/search/45.4446,-111.0038	https://data.globe.gov/system/photos/2019/08/07/1159833/original.jpg	https://data.globe.gov/system/photos/2019/08/07/1159835/original.jpg	https://data.globe.gov/system/photos/2019/08/07/1159834/original.jpg	https://data.globe.gov/system/photos/2019/08/07/1159836/original.jpg	https://data.globe.gov/system/photos/2019/08/07/1159838/original.jpg
GV_1726	45.9112	-110.9552	2299.1		https://www.google.com/maps/search/45.9112,-110.9552	https://data.globe.gov/system/photos/2019/08/08/1159903/original.jpg	https://data.globe.gov/system/photos/2019/08/08/1159905/original.jpg	https://data.globe.gov/system/photos/2019/08/08/1159904/original.jpg	https://data.globe.gov/system/photos/2019/08/08/1159906/original.jpg	https://data.globe.gov/system/photos/2019/08/08/1159908/original.jpg
GV_1727	45.9058	-110.9579	2305.2		https://www.google.com/maps/search/45.9058,-110.9579	https://data.globe.gov/system/photos/2019/08/08/1159909/original.jpg	https://data.globe.gov/system/photos/2019/08/08/1159911/original.jpg	https://data.globe.gov/system/photos/2019/08/08/1159910/original.jpg	https://data.globe.gov/system/photos/2019/08/08/1159912/original.jpg	https://data.globe.gov/system/photos/2019/08/08/1159914/original.jpg
GV_1728	45.9029	-110.967	2502		https://www.google.com/maps/search/45.9029,-110.967	https://data.globe.gov/system/photos/2019/08/08/1159921/original.jpg	https://data.globe.gov/system/photos/2019/08/08/1159923/original.jpg	https://data.globe.gov/system/photos/2019/08/08/1159922/original.jpg	https://data.globe.gov/system/photos/2019/08/08/1159924/original.jpg	https://data.globe.gov/system/photos/2019/08/08/1159926/original.jpg
GV_1729	45.8181	-110.9078	1978.8		https://www.google.com/maps/search/45.8181,-110.9078	https://data.globe.gov/system/photos/2019/08/08/1159940/original.jpg	https://data.globe.gov/system/photos/2019/08/08/1159942/original.jpg	https://data.globe.gov/system/photos/2019/08/08/1159941/original.jpg	https://data.globe.gov/system/photos/2019/08/08/1159943/original.jpg	https://data.globe.gov/system/photos/2019/08/08/1159945/original.jpg
GV_1730	45.8181	-110.9061	1957.6		https://www.google.com/maps/search/45.8181,-110.9061	https://data.globe.gov/system/photos/2019/08/08/1159946/original.jpg	https://data.globe.gov/system/photos/2019/08/08/1159948/original.jpg	https://data.globe.gov/system/photos/2019/08/08/1159947/original.jpg	https://data.globe.gov/system/photos/2019/08/08/1159949/original.jpg	https://data.globe.gov/system/photos/2019/08/08/1159951/original.jpg
GV_1731	45.8172	-110.8938	1846.9		https://www.google.com/maps/search/45.8172,-110.8938	https://data.globe.gov/system/photos/2019/08/08/1159952/original.jpg	https://data.globe.gov/system/photos/2019/08/08/1159954/original.jpg	https://data.globe.gov/system/photos/2019/08/08/1159953/original.jpg	https://data.globe.gov/system/photos/2019/08/08/1159955/original.jpg	https://data.globe.gov/system/photos/2019/08/08/1159957/original.jpg
GV_1732	45.8169	-110.891	1834.2		https://www.google.com/maps/search/45.8169,-110.891	https://data.globe.gov/system/photos/2019/08/08/1159958/original.jpg	https://data.globe.gov/system/photos/2019/08/08/1159960/original.jpg	https://data.globe.gov/system/photos/2019/08/08/1159959/original.jpg	https://data.globe.gov/system/photos/2019/08/08/1159961/original.jpg	https://data.globe.gov/system/photos/2019/08/08/1159963/original.jpg
GV_1733	45.8804	-110.8831	1932.8		https://www.google.com/maps/search/45.8804,-110.8831	https://data.globe.gov/system/photos/2019/08/08/1159964/original.jpg	https://data.globe.gov/system/photos/2019/08/08/1159966/original.jpg	https://data.globe.gov/system/photos/2019/08/08/1159965/original.jpg	https://data.globe.gov/system/photos/2019/08/08/1159967/original.jpg	https://data.globe.gov/system/photos/2019/08/08/1159969/original.jpg
GV_1734	45.9089	-110.9277	2088.3		https://www.google.com/maps/search/45.9089,-110.9277	https://data.globe.gov/system/photos/2019/08/08/1159970/original.jpg	https://data.globe.gov/system/photos/2019/08/08/1159972/original.jpg	https://data.globe.gov/system/photos/2019/08/08/1159971/original.jpg	https://data.globe.gov/system/photos/2019/08/08/1159973/original.jpg	https://data.globe.gov/system/photos/2019/08/08/1159975/original.jpg
GV_1735	45.9072	-110.9412	2185.5		https://www.google.com/maps/search/45.9072,-110.9412	https://data.globe.gov/system/photos/2019/08/08/1159976/original.jpg	https://data.globe.gov/system/photos/2019/08/08/1159978/original.jpg	https://data.globe.gov/system/photos/2019/08/08/1159977/original.jpg	https://data.globe.gov/system/photos/2019/08/08/1159979/original.jpg	https://data.globe.gov/system/photos/2019/08/08/1159981/original.jpg
GV_1736	45.906	-110.9595	2315.4		https://www.google.com/maps/search/45.906,-110.9595	https://data.globe.gov/system/photos/2019/08/08/1159982/original.jpg	https://data.globe.gov/system/photos/2019/08/08/1159984/original.jpg	https://data.globe.gov/system/photos/2019/08/08/1159983/original.jpg	https://data.globe.gov/system/photos/2019/08/08/1159985/original.jpg	https://data.globe.gov/system/photos/2019/08/08/1159987/original.jpg
GV_1737	45.9054	-110.9594	2308.2		https://www.google.com/maps/search/45.9054,-110.9594	https://data.globe.gov/system/photos/2019/08/08/1159988/original.jpg	https://data.globe.gov/system/photos/2019/08/08/1159990/original.jpg	https://data.globe.gov/system/photos/2019/08/08/1159989/original.jpg	https://data.globe.gov/system/photos/2019/08/08/1159991/original.jpg	https://data.globe.gov/system/photos/2019/08/08/1159993/original.jpg
GV_1738	45.9063	-110.9597	2320.9		https://www.google.com/maps/search/45.9063,-110.9597	https://data.globe.gov/system/photos/2019/08/08/1159994/original.jpg	https://data.globe.gov/system/photos/2019/08/08/1159996/original.jpg	https://data.globe.gov/system/photos/2019/08/08/1159995/original.jpg	https://data.globe.gov/system/photos/2019/08/08/1159997/original.jpg	https://data.globe.gov/system/photos/2019/08/08/1159999/original.jpg
GV_1739	45.9059	-110.9619	2355.2		https://www.google.com/maps/search/45.9059,-110.9619	https://data.globe.gov/system/photos/2019/08/08/1160000/original.jpg	https://data.globe.gov/system/photos/2019/08/08/1160002/original.jpg	https://data.globe.gov/system/photos/2019/08/08/1160001/original.jpg	https://data.globe.gov/system/photos/2019/08/08/1160003/original.jpg	https://data.globe.gov/system/photos/2019/08/08/1160005/original.jpg
GV_1740	45.9053	-110.9656	2405		https://www.google.com/maps/search/45.9053,-110.9656	https://data.globe.gov/system/photos/2019/08/08/1160006/original.jpg	https://data.globe.gov/system/photos/2019/08/08/1160008/original.jpg	https://data.globe.gov/system/photos/2019/08/08/1160007/original.jpg	https://data.globe.gov/system/photos/2019/08/08/1160009/original.jpg	https://data.globe.gov/system/photos/2019/08/08/1160011/original.jpg
GV_1741	45.9041	-110.9647	2444.7		https://www.google.com/maps/search/45.9041,-110.9647	https://data.globe.gov/system/photos/2019/08/08/1160012/original.jpg	https://data.globe.gov/system/photos/2019/08/08/1160014/original.jpg	https://data.globe.gov/system/photos/2019/08/08/1160013/original.jpg	https://data.globe.gov/system/photos/2019/08/08/1160015/original.jpg	https://data.globe.gov/system/photos/2019/08/08/1160017/original.jpg
GV_1742	45.6656	-111.0466	1497.3		https://www.google.com/maps/search/45.6656,-111.0466	https://data.globe.gov/system/photos/2019/08/07/1160018/original.jpg	https://data.globe.gov/system/photos/2019/08/07/1160020/original.jpg	https://data.globe.gov/system/photos/2019/08/07/1160019/original.jpg	https://data.globe.gov/system/photos/2019/08/07/1160021/original.jpg	https://data.globe.gov/system/photos/2019/08/07/1160023/original.jpg
GV_1743	45.6646	-111.0474	1499.9		https://www.google.com/maps/search/45.6646,-111.0474	https://data.globe.gov/system/photos/2019/08/07/1160024/original.jpg	https://data.globe.gov/system/photos/2019/08/07/1160026/original.jpg	https://data.globe.gov/system/photos/2019/08/07/1160025/original.jpg	https://data.globe.gov/system/photos/2019/08/07/1160027/original.jpg	https://data.globe.gov/system/photos/2019/08/07/1160029/original.jpg
GV_1744	45.6639	-111.0479	1500.5		https://www.google.com/maps/search/45.6639,-111.0479	https://data.globe.gov/system/photos/2019/08/07/1160030/original.jpg	https://data.globe.gov/system/photos/2019/08/07/1160032/original.jpg	https://data.globe.gov/system/photos/2019/08/07/1160031/original.jpg	https://data.globe.gov/system/photos/2019/08/07/1160033/original.jpg	https://data.globe.gov/system/photos/2019/08/07/1160035/original.jpg
GV_1745	45.6623	-111.0474	1501.8		https://www.google.com/maps/search/45.6623,-111.0474	https://data.globe.gov/system/photos/2019/08/07/1160036/original.jpg	https://data.globe.gov/system/photos/2019/08/07/1160038/original.jpg	https://data.globe.gov/system/photos/2019/08/07/1160037/original.jpg	https://data.globe.gov/system/photos/2019/08/07/1160039/original.jpg	https://data.globe.gov/system/photos/2019/08/07/1160041/original.jpg
GV_1746	45.6536	-110.5581	1370.3		https://www.google.com/maps/search/45.6536,-110.5581	https://data.globe.gov/system/photos/2019/08/07/1160042/original.jpg	https://data.globe.gov/system/photos/2019/08/07/1160044/original.jpg	https://data.globe.gov/system/photos/2019/08/07/1160043/original.jpg	https://data.globe.gov/system/photos/2019/08/07/1160045/original.jpg	https://data.globe.gov/system/photos/2019/08/07/1160047/original.jpg
GV_1747	45.6546	-110.5587	1369.3		https://www.google.com/maps/search/45.6546,-110.5587	https://data.globe.gov/system/photos/2019/08/07/1160048/original.jpg	https://data.globe.gov/system/photos/2019/08/07/1160050/original.jpg	https://data.globe.gov/system/photos/2019/08/07/1160049/original.jpg	https://data.globe.gov/system/photos/2019/08/07/1160051/original.jpg	https://data.globe.gov/system/photos/2019/08/07/1160053/original.jpg
GV_1748	45.6548	-110.5585	1369.7		https://www.google.com/maps/search/45.6548,-110.5585	https://data.globe.gov/system/photos/2019/08/07/1160054/original.jpg	https://data.globe.gov/system/photos/2019/08/07/1160056/original.jpg	https://data.globe.gov/system/photos/2019/08/07/1160055/original.jpg	https://data.globe.gov/system/photos/2019/08/07/1160057/original.jpg	https://data.globe.gov/system/photos/2019/08/07/1160059/original.jpg
GV_1749	45.6533	-110.5577	1368.9		https://www.google.com/maps/search/45.6533,-110.5577	https://data.globe.gov/system/photos/2019/08/07/1160060/original.jpg	https://data.globe.gov/system/photos/2019/08/07/1160062/original.jpg	https://data.globe.gov/system/photos/2019/08/07/1160061/original.jpg	https://data.globe.gov/system/photos/2019/08/07/1160063/original.jpg	https://data.globe.gov/system/photos/2019/08/07/1160065/original.jpg
GV_1750	45.6537	-110.5572	1368.8		https://www.google.com/maps/search/45.6537,-110.5572	https://data.globe.gov/system/photos/2019/08/07/1160066/original.jpg	https://data.globe.gov/system/photos/2019/08/07/1160068/original.jpg	https://data.globe.gov/system/photos/2019/08/07/1160067/original.jpg	https://data.globe.gov/system/photos/2019/08/07/1160069/original.jpg	https://data.globe.gov/system/photos/2019/08/07/1160071/original.jpg
GV_1751	45.6538	-110.556	1368.4		https://www.google.com/maps/search/45.6538,-110.556	https://data.globe.gov/system/photos/2019/08/07/1160072/original.jpg	https://data.globe.gov/system/photos/2019/08/07/1160074/original.jpg	https://data.globe.gov/system/photos/2019/08/07/1160073/original.jpg	https://data.globe.gov/system/photos/2019/08/07/1160075/original.jpg	https://data.globe.gov/system/photos/2019/08/07/1160077/original.jpg
GV_1752	45.6497	-110.5623	1369		https://www.google.com/maps/search/45.6497,-110.5623	https://data.globe.gov/system/photos/2019/08/07/1160078/original.jpg	https://data.globe.gov/system/photos/2019/08/07/1160080/original.jpg	https://data.globe.gov/system/photos/2019/08/07/1160079/original.jpg	https://data.globe.gov/system/photos/2019/08/07/1160081/original.jpg	https://data.globe.gov/system/photos/2019/08/07/1160083/original.jpg
GV_1753	45.6501	-110.5626	1371.6		https://www.google.com/maps/search/45.6501,-110.5626	https://data.globe.gov/system/photos/2019/08/07/1160084/original.jpg	https://data.globe.gov/system/photos/2019/08/07/1160086/original.jpg	https://data.globe.gov/system/photos/2019/08/07/1160085/original.jpg	https://data.globe.gov/system/photos/2019/08/07/1160087/original.jpg	https://data.globe.gov/system/photos/2019/08/07/1160089/original.jpg
GV_1754	45.2953	-110.8343	1503.3		https://www.google.com/maps/search/45.2953,-110.8343	https://data.globe.gov/system/photos/2019/08/07/1160090/original.jpg	https://data.globe.gov/system/photos/2019/08/07/1160092/original.jpg	https://data.globe.gov/system/photos/2019/08/07/1160091/original.jpg	https://data.globe.gov/system/photos/2019/08/07/1160093/original.jpg	https://data.globe.gov/system/photos/2019/08/07/1160095/original.jpg
GV_1755	45.2949	-110.8346	1502.5		https://www.google.com/maps/search/45.2949,-110.8346	https://data.globe.gov/system/photos/2019/08/07/1160096/original.jpg	https://data.globe.gov/system/photos/2019/08/07/1160098/original.jpg	https://data.globe.gov/system/photos/2019/08/07/1160097/original.jpg	https://data.globe.gov/system/photos/2019/08/07/1160099/original.jpg	https://data.globe.gov/system/photos/2019/08/07/1160101/original.jpg
GV_1756	45.2948	-110.8352	1502		https://www.google.com/maps/search/45.2948,-110.8352	https://data.globe.gov/system/photos/2019/08/07/1160102/original.jpg	https://data.globe.gov/system/photos/2019/08/07/1160104/original.jpg	https://data.globe.gov/system/photos/2019/08/07/1160103/original.jpg	https://data.globe.gov/system/photos/2019/08/07/1160105/original.jpg	https://data.globe.gov/system/photos/2019/08/07/1160107/original.jpg
GV_1757	45.2534	-110.871	1508.5		https://www.google.com/maps/search/45.2534,-110.871	https://data.globe.gov/system/photos/2019/08/07/1160108/original.jpg	https://data.globe.gov/system/photos/2019/08/07/1160110/original.jpg	https://data.globe.gov/system/photos/2019/08/07/1160109/original.jpg	https://data.globe.gov/system/photos/2019/08/07/1160111/original.jpg	https://data.globe.gov/system/photos/2019/08/07/1160113/original.jpg
GV_1758	45.2531	-110.8715	1508.5		https://www.google.com/maps/search/45.2531,-110.8715	https://data.globe.gov/system/photos/2019/08/07/1160114/original.jpg	https://data.globe.gov/system/photos/2019/08/07/1160116/original.jpg	https://data.globe.gov/system/photos/2019/08/07/1160115/original.jpg	https://data.globe.gov/system/photos/2019/08/07/1160117/original.jpg	https://data.globe.gov/system/photos/2019/08/07/1160119/original.jpg
GV_1759	45.2521	-110.8734	1510.8		https://www.google.com/maps/search/45.2521,-110.8734	https://data.globe.gov/system/photos/2019/08/07/1160120/original.jpg	https://data.globe.gov/system/photos/2019/08/07/1160122/original.jpg	https://data.globe.gov/system/photos/2019/08/07/1160121/original.jpg	https://data.globe.gov/system/photos/2019/08/07/1160123/original.jpg	https://data.globe.gov/system/photos/2019/08/07/1160125/original.jpg
GV_1760	45.2527	-110.8727	1510.7		https://www.google.com/maps/search/45.2527,-110.8727	https://data.globe.gov/system/photos/2019/08/07/1160126/original.jpg	https://data.globe.gov/system/photos/2019/08/07/1160128/original.jpg	https://data.globe.gov/system/photos/2019/08/07/1160127/original.jpg	https://data.globe.gov/system/photos/2019/08/07/1160129/original.jpg	https://data.globe.gov/system/photos/2019/08/07/1160131/original.jpg
GV_1761	45.254	-110.8706	1511.2		https://www.google.com/maps/search/45.254,-110.8706	https://data.globe.gov/system/photos/2019/08/07/1160132/original.jpg	https://data.globe.gov/system/photos/2019/08/07/1160134/original.jpg	https://data.globe.gov/system/photos/2019/08/07/1160133/original.jpg	https://data.globe.gov/system/photos/2019/08/07/1160135/original.jpg	https://data.globe.gov/system/photos/2019/08/07/1160137/original.jpg
GV_1762	45.1885	-110.8973	1535.9		https://www.google.com/maps/search/45.1885,-110.8973	https://data.globe.gov/system/photos/2019/08/07/1160138/original.jpg	https://data.globe.gov/system/photos/2019/08/07/1160140/original.jpg	https://data.globe.gov/system/photos/2019/08/07/1160139/original.jpg	https://data.globe.gov/system/photos/2019/08/07/1160141/original.jpg	https://data.globe.gov/system/photos/2019/08/07/1160143/original.jpg
GV_1763	45.0861	-110.7759	1572.5		https://www.google.com/maps/search/45.0861,-110.7759	https://data.globe.gov/system/photos/2019/08/07/1160144/original.jpg	https://data.globe.gov/system/photos/2019/08/07/1160146/original.jpg	https://data.globe.gov/system/photos/2019/08/07/1160145/original.jpg	https://data.globe.gov/system/photos/2019/08/07/1160147/original.jpg	https://data.globe.gov/system/photos/2019/08/07/1160149/original.jpg
GV_1764	45.0356	-110.7136	1610.3		https://www.google.com/maps/search/45.0356,-110.7136	https://data.globe.gov/system/photos/2019/08/07/1160150/original.jpg	https://data.globe.gov/system/photos/2019/08/07/1160152/original.jpg	https://data.globe.gov/system/photos/2019/08/07/1160151/original.jpg	https://data.globe.gov/system/photos/2019/08/07/1160153/original.jpg	https://data.globe.gov/system/photos/2019/08/07/1160155/original.jpg
GV_1765	45.0323	-110.7056	1597.8		https://www.google.com/maps/search/45.0323,-110.7056	https://data.globe.gov/system/photos/2019/08/07/1160156/original.jpg	https://data.globe.gov/system/photos/2019/08/07/1160158/original.jpg	https://data.globe.gov/system/photos/2019/08/07/1160157/original.jpg	https://data.globe.gov/system/photos/2019/08/07/1160159/original.jpg	https://data.globe.gov/system/photos/2019/08/07/1160161/original.jpg
GV_1766	45.0307	-110.7114	1607.2		https://www.google.com/maps/search/45.0307,-110.7114	https://data.globe.gov/system/photos/2019/08/07/1160162/original.jpg	https://data.globe.gov/system/photos/2019/08/07/1160164/original.jpg	https://data.globe.gov/system/photos/2019/08/07/1160163/original.jpg	https://data.globe.gov/system/photos/2019/08/07/1160165/original.jpg	https://data.globe.gov/system/photos/2019/08/07/1160167/original.jpg
GV_1767	45.0332	-110.7214	1617.8		https://www.google.com/maps/search/45.0332,-110.7214	https://data.globe.gov/system/photos/2019/08/07/1160168/original.jpg	https://data.globe.gov/system/photos/2019/08/07/1160170/original.jpg	https://data.globe.gov/system/photos/2019/08/07/1160169/original.jpg	https://data.globe.gov/system/photos/2019/08/07/1160171/original.jpg	https://data.globe.gov/system/photos/2019/08/07/1160173/original.jpg
GV_1768	45.0356	-110.7285	1643.4		https://www.google.com/maps/search/45.0356,-110.7285	https://data.globe.gov/system/photos/2019/08/07/1160174/original.jpg	https://data.globe.gov/system/photos/2019/08/07/1160176/original.jpg	https://data.globe.gov/system/photos/2019/08/07/1160175/original.jpg	https://data.globe.gov/system/photos/2019/08/07/1160177/original.jpg	https://data.globe.gov/system/photos/2019/08/07/1160179/original.jpg
GV_1769	45.0426	-110.7443	1606.1		https://www.google.com/maps/search/45.0426,-110.7443	https://data.globe.gov/system/photos/2019/08/07/1160180/original.jpg	https://data.globe.gov/system/photos/2019/08/07/1160182/original.jpg	https://data.globe.gov/system/photos/2019/08/07/1160181/original.jpg	https://data.globe.gov/system/photos/2019/08/07/1160183/original.jpg	https://data.globe.gov/system/photos/2019/08/07/1160185/original.jpg
GV_1770	45.0442	-110.7474	1599.4		https://www.google.com/maps/search/45.0442,-110.7474	https://data.globe.gov/system/photos/2019/08/07/1160186/original.jpg	https://data.globe.gov/system/photos/2019/08/07/1160188/original.jpg	https://data.globe.gov/system/photos/2019/08/07/1160187/original.jpg	https://data.globe.gov/system/photos/2019/08/07/1160189/original.jpg	https://data.globe.gov/system/photos/2019/08/07/1160191/original.jpg
GV_1771	45.0448	-110.7495	1596.7		https://www.google.com/maps/search/45.0448,-110.7495	https://data.globe.gov/system/photos/2019/08/07/1160192/original.jpg	https://data.globe.gov/system/photos/2019/08/07/1160194/original.jpg	https://data.globe.gov/system/photos/2019/08/07/1160193/original.jpg	https://data.globe.gov/system/photos/2019/08/07/1160195/original.jpg	https://data.globe.gov/system/photos/2019/08/07/1160197/original.jpg
GV_1772	45.0478	-110.7543	1589.5		https://www.google.com/maps/search/45.0478,-110.7543	https://data.globe.gov/system/photos/2019/08/07/1160198/original.jpg	https://data.globe.gov/system/photos/2019/08/07/1160200/original.jpg	https://data.globe.gov/system/photos/2019/08/07/1160199/original.jpg	https://data.globe.gov/system/photos/2019/08/07/1160201/original.jpg	https://data.globe.gov/system/photos/2019/08/07/1160203/original.jpg
GV_1773	45.0502	-110.7557	1584.2		https://www.google.com/maps/search/45.0502,-110.7557	https://data.globe.gov/system/photos/2019/08/07/1160204/original.jpg	https://data.globe.gov/system/photos/2019/08/07/1160206/original.jpg	https://data.globe.gov/system/photos/2019/08/07/1160205/original.jpg	https://data.globe.gov/system/photos/2019/08/07/1160207/original.jpg	https://data.globe.gov/system/photos/2019/08/07/1160209/original.jpg
GV_1774	45.0595	-110.7722	1581.1		https://www.google.com/maps/search/45.0595,-110.7722	https://data.globe.gov/system/photos/2019/08/07/1160210/original.jpg	https://data.globe.gov/system/photos/2019/08/07/1160212/original.jpg	https://data.globe.gov/system/photos/2019/08/07/1160211/original.jpg	https://data.globe.gov/system/photos/2019/08/07/1160213/original.jpg	https://data.globe.gov/system/photos/2019/08/07/1160215/original.jpg
GV_1775	45.0558	-110.7685	1579.6		https://www.google.com/maps/search/45.0558,-110.7685	https://data.globe.gov/system/photos/2019/08/07/1160216/original.jpg	https://data.globe.gov/system/photos/2019/08/07/1160218/original.jpg	https://data.globe.gov/system/photos/2019/08/07/1160217/original.jpg	https://data.globe.gov/system/photos/2019/08/07/1160219/original.jpg	https://data.globe.gov/system/photos/2019/08/07/1160221/original.jpg
GV_1776	45.0651	-110.7774	1605.2		https://www.google.com/maps/search/45.0651,-110.7774	https://data.globe.gov/system/photos/2019/08/07/1160222/original.jpg	https://data.globe.gov/system/photos/2019/08/07/1160224/original.jpg	https://data.globe.gov/system/photos/2019/08/07/1160223/original.jpg	https://data.globe.gov/system/photos/2019/08/07/1160225/original.jpg	https://data.globe.gov/system/photos/2019/08/07/1160227/original.jpg
GV_1777	45.0651	-110.776	1587.9		https://www.google.com/maps/search/45.0651,-110.776	https://data.globe.gov/system/photos/2019/08/07/1160228/original.jpg	https://data.globe.gov/system/photos/2019/08/07/1160230/original.jpg	https://data.globe.gov/system/photos/2019/08/07/1160229/original.jpg	https://data.globe.gov/system/photos/2019/08/07/1160231/original.jpg	https://data.globe.gov/system/photos/2019/08/07/1160233/original.jpg
GV_1778	45.0923	-110.7839	1585.2		https://www.google.com/maps/search/45.0923,-110.7839	https://data.globe.gov/system/photos/2019/08/07/1160234/original.jpg	https://data.globe.gov/system/photos/2019/08/07/1160236/original.jpg	https://data.globe.gov/system/photos/2019/08/07/1160235/original.jpg	https://data.globe.gov/system/photos/2019/08/07/1160237/original.jpg	https://data.globe.gov/system/photos/2019/08/07/1160240/original.jpg
GV_1779	45.1056	-110.7898	1564.4		https://www.google.com/maps/search/45.1056,-110.7898	https://data.globe.gov/system/photos/2019/08/07/1160241/original.jpg	https://data.globe.gov/system/photos/2019/08/07/1160243/original.jpg	https://data.globe.gov/system/photos/2019/08/07/1160242/original.jpg	https://data.globe.gov/system/photos/2019/08/07/1160245/original.jpg	https://data.globe.gov/system/photos/2019/08/07/1160248/original.jpg
GV_1780	45.1078	-110.7903	1553.3		https://www.google.com/maps/search/45.1078,-110.7903	https://data.globe.gov/system/photos/2019/08/07/1160249/original.jpg	https://data.globe.gov/system/photos/2019/08/07/1160253/original.jpg	https://data.globe.gov/system/photos/2019/08/07/1160251/original.jpg	https://data.globe.gov/system/photos/2019/08/07/1160254/original.jpg	https://data.globe.gov/system/photos/2019/08/07/1160256/original.jpg
GV_1781	45.108	-110.7909	1555		https://www.google.com/maps/search/45.108,-110.7909	https://data.globe.gov/system/photos/2019/08/07/1160258/original.jpg	https://data.globe.gov/system/photos/2019/08/07/1160260/original.jpg	https://data.globe.gov/system/photos/2019/08/07/1160259/original.jpg	https://data.globe.gov/system/photos/2019/08/07/1160261/original.jpg	https://data.globe.gov/system/photos/2019/08/07/1160264/original.jpg
GV_1782	45.8167	-110.8836	1806.7		https://www.google.com/maps/search/45.8167,-110.8836	https://data.globe.gov/system/photos/2019/08/08/1160266/original.jpg	https://data.globe.gov/system/photos/2019/08/08/1160270/original.jpg	https://data.globe.gov/system/photos/2019/08/08/1160267/original.jpg	https://data.globe.gov/system/photos/2019/08/08/1160272/original.jpg	https://data.globe.gov/system/photos/2019/08/08/1160275/original.jpg
GV_1783	45.8174	-110.8963	1859		https://www.google.com/maps/search/45.8174,-110.8963	https://data.globe.gov/system/photos/2019/08/08/1160276/original.jpg	https://data.globe.gov/system/photos/2019/08/08/1160278/original.jpg	https://data.globe.gov/system/photos/2019/08/08/1160277/original.jpg	https://data.globe.gov/system/photos/2019/08/08/1160279/original.jpg	https://data.globe.gov/system/photos/2019/08/08/1160281/original.jpg
GV_1784	45.8178	-110.8974	1864.7		https://www.google.com/maps/search/45.8178,-110.8974	https://data.globe.gov/system/photos/2019/08/08/1160282/original.jpg	https://data.globe.gov/system/photos/2019/08/08/1160284/original.jpg	https://data.globe.gov/system/photos/2019/08/08/1160283/original.jpg	https://data.globe.gov/system/photos/2019/08/08/1160285/original.jpg	https://data.globe.gov/system/photos/2019/08/08/1160287/original.jpg
GV_1785	45.8172	-110.8975	1868.1		https://www.google.com/maps/search/45.8172,-110.8975	https://data.globe.gov/system/photos/2019/08/08/1160288/original.jpg	https://data.globe.gov/system/photos/2019/08/08/1160290/original.jpg	https://data.globe.gov/system/photos/2019/08/08/1160289/original.jpg	https://data.globe.gov/system/photos/2019/08/08/1160291/original.jpg	https://data.globe.gov/system/photos/2019/08/08/1160293/original.jpg
GV_1786	45.8171	-110.8993	1890.3		https://www.google.com/maps/search/45.8171,-110.8993	https://data.globe.gov/system/photos/2019/08/08/1160294/original.jpg	https://data.globe.gov/system/photos/2019/08/08/1160296/original.jpg	https://data.globe.gov/system/photos/2019/08/08/1160295/original.jpg	https://data.globe.gov/system/photos/2019/08/08/1160297/original.jpg	https://data.globe.gov/system/photos/2019/08/08/1160299/original.jpg
GV_1787	45.8168	-110.8967	1868.3		https://www.google.com/maps/search/45.8168,-110.8967	https://data.globe.gov/system/photos/2019/08/08/1160300/original.jpg	https://data.globe.gov/system/photos/2019/08/08/1160302/original.jpg	https://data.globe.gov/system/photos/2019/08/08/1160301/original.jpg	https://data.globe.gov/system/photos/2019/08/08/1160303/original.jpg	https://data.globe.gov/system/photos/2019/08/08/1160305/original.jpg
GV_1788	45.8166	-110.8958	1866.7		https://www.google.com/maps/search/45.8166,-110.8958	https://data.globe.gov/system/photos/2019/08/08/1160306/original.jpg	https://data.globe.gov/system/photos/2019/08/08/1160308/original.jpg	https://data.globe.gov/system/photos/2019/08/08/1160307/original.jpg	https://data.globe.gov/system/photos/2019/08/08/1160309/original.jpg	https://data.globe.gov/system/photos/2019/08/08/1160311/original.jpg
GV_1789	45.8187	-110.9073	1962.1		https://www.google.com/maps/search/45.8187,-110.9073	https://data.globe.gov/system/photos/2019/08/08/1160312/original.jpg	https://data.globe.gov/system/photos/2019/08/08/1160314/original.jpg	https://data.globe.gov/system/photos/2019/08/08/1160313/original.jpg	https://data.globe.gov/system/photos/2019/08/08/1160315/original.jpg	https://data.globe.gov/system/photos/2019/08/08/1160317/original.jpg
GV_1790	45.6687	-111.0546	1484		https://www.google.com/maps/search/45.6687,-111.0546	https://data.globe.gov/system/photos/2019/08/08/1160444/original.jpg	https://data.globe.gov/system/photos/2019/08/08/1160446/original.jpg	https://data.globe.gov/system/photos/2019/08/08/1160445/original.jpg	https://data.globe.gov/system/photos/2019/08/08/1160447/original.jpg	https://data.globe.gov/system/photos/2019/08/08/1160449/original.jpg
GV_1791	45.6697	-111.0542	1483.5		https://www.google.com/maps/search/45.6697,-111.0542	https://data.globe.gov/system/photos/2019/08/08/1160450/original.jpg	https://data.globe.gov/system/photos/2019/08/08/1160452/original.jpg	https://data.globe.gov/system/photos/2019/08/08/1160451/original.jpg	https://data.globe.gov/system/photos/2019/08/08/1160453/original.jpg	https://data.globe.gov/system/photos/2019/08/08/1160455/original.jpg
GV_1792	45.6697	-111.0534	1484		https://www.google.com/maps/search/45.6697,-111.0534	https://data.globe.gov/system/photos/2019/08/08/1160458/original.jpg	https://data.globe.gov/system/photos/2019/08/08/1160467/original.jpg	https://data.globe.gov/system/photos/2019/08/08/1160463/original.jpg	https://data.globe.gov/system/photos/2019/08/08/1160469/original.jpg	https://data.globe.gov/system/photos/2019/08/08/1160472/original.jpg
GV_1793	45.6693	-111.0528	1484.9		https://www.google.com/maps/search/45.6693,-111.0528	https://data.globe.gov/system/photos/2019/08/08/1160474/original.jpg	https://data.globe.gov/system/photos/2019/08/08/1160476/original.jpg	https://data.globe.gov/system/photos/2019/08/08/1160475/original.jpg	https://data.globe.gov/system/photos/2019/08/08/1160477/original.jpg	https://data.globe.gov/system/photos/2019/08/08/1160479/original.jpg

GV_1794	45.6698	-111.0527	1483.5		https://www.google.com/maps/search/45.6698,-111.0527	https://data.globe.gov/system/photos/2019/08/08/1160480/original.jpg	https://data.globe.gov/system/photos/2019/08/08/1160482/original.jpg	https://data.globe.gov/system/photos/2019/08/08/1160481/original.jpg	https://data.globe.gov/system/photos/2019/08/08/1160483/original.jpg	https://data.globe.gov/system/photos/2019/08/08/1160485/original.jpg
GV_1795	45.6705	-111.0534	1481.2		https://www.google.com/maps/search/45.6705,-111.0534	https://data.globe.gov/system/photos/2019/08/08/1160486/original.jpg	https://data.globe.gov/system/photos/2019/08/08/1160488/original.jpg	https://data.globe.gov/system/photos/2019/08/08/1160487/original.jpg	https://data.globe.gov/system/photos/2019/08/08/1160489/original.jpg	https://data.globe.gov/system/photos/2019/08/08/1160491/original.jpg
GV_1796	45.6708	-111.0524	1481.9		https://www.google.com/maps/search/45.6708,-111.0524	https://data.globe.gov/system/photos/2019/08/08/1160492/original.jpg	https://data.globe.gov/system/photos/2019/08/08/1160501/original.jpg	https://data.globe.gov/system/photos/2019/08/08/1160493/original.jpg	https://data.globe.gov/system/photos/2019/08/08/1160506/original.jpg	https://data.globe.gov/system/photos/2019/08/08/1160508/original.jpg
GV_1797	45.671	-111.052	1481		https://www.google.com/maps/search/45.671,-111.052	https://data.globe.gov/system/photos/2019/08/08/1160509/original.jpg	https://data.globe.gov/system/photos/2019/08/08/1160511/original.jpg	https://data.globe.gov/system/photos/2019/08/08/1160510/original.jpg	https://data.globe.gov/system/photos/2019/08/08/1160512/original.jpg	https://data.globe.gov/system/photos/2019/08/08/1160514/original.jpg
GV_1798	45.6712	-111.0529	1481.2		https://www.google.com/maps/search/45.6712,-111.0529	https://data.globe.gov/system/photos/2019/08/08/1160520/original.jpg	https://data.globe.gov/system/photos/2019/08/08/1160522/original.jpg	https://data.globe.gov/system/photos/2019/08/08/1160521/original.jpg	https://data.globe.gov/system/photos/2019/08/08/1160523/original.jpg	https://data.globe.gov/system/photos/2019/08/08/1160525/original.jpg
GV_1799	45.6711	-111.0542	1482.7		https://www.google.com/maps/search/45.6711,-111.0542	https://data.globe.gov/system/photos/2019/08/08/1160526/original.jpg	https://data.globe.gov/system/photos/2019/08/08/1160534/original.jpg	https://data.globe.gov/system/photos/2019/08/08/1160527/original.jpg	https://data.globe.gov/system/photos/2019/08/08/1160535/original.jpg	https://data.globe.gov/system/photos/2019/08/08/1160537/original.jpg
GV_1800	45.6707	-111.055	1482.9		https://www.google.com/maps/search/45.6707,-111.055	https://data.globe.gov/system/photos/2019/08/08/1160538/original.jpg	https://data.globe.gov/system/photos/2019/08/08/1160540/original.jpg	https://data.globe.gov/system/photos/2019/08/08/1160539/original.jpg	https://data.globe.gov/system/photos/2019/08/08/1160541/original.jpg	https://data.globe.gov/system/photos/2019/08/08/1160543/original.jpg
GV_1801	45.6706	-111.056	1483.1		https://www.google.com/maps/search/45.6706,-111.056	https://data.globe.gov/system/photos/2019/08/08/1160544/original.jpg	https://data.globe.gov/system/photos/2019/08/08/1160546/original.jpg	https://data.globe.gov/system/photos/2019/08/08/1160545/original.jpg	https://data.globe.gov/system/photos/2019/08/08/1160547/original.jpg	https://data.globe.gov/system/photos/2019/08/08/1160549/original.jpg
GV_1802	45.6707	-111.051	1484.1		https://www.google.com/maps/search/45.6707,-111.051	https://data.globe.gov/system/photos/2019/08/08/1160550/original.jpg	https://data.globe.gov/system/photos/2019/08/08/1160552/original.jpg	https://data.globe.gov/system/photos/2019/08/08/1160551/original.jpg	https://data.globe.gov/system/photos/2019/08/08/1160553/original.jpg	https://data.globe.gov/system/photos/2019/08/08/1160555/original.jpg
GV_1803	38.9929	-76.8408	62.9		https://www.google.com/maps/search/38.9929,-76.8408	https://data.globe.gov/system/photos/2019/08/08/1160581/original.jpg	https://data.globe.gov/system/photos/2019/08/08/1160583/original.jpg	https://data.globe.gov/system/photos/2019/08/08/1160582/original.jpg	https://data.globe.gov/system/photos/2019/08/08/1160585/original.jpg	https://data.globe.gov/system/photos/2019/08/08/1160589/original.jpg
GV_1804	45.6704	-111.05	1486.5		https://www.google.com/maps/search/45.6704,-111.05	https://data.globe.gov/system/photos/2019/08/08/1160690/original.jpg	https://data.globe.gov/system/photos/2019/08/08/1160692/original.jpg	https://data.globe.gov/system/photos/2019/08/08/1160691/original.jpg	https://data.globe.gov/system/photos/2019/08/08/1160693/original.jpg	https://data.globe.gov/system/photos/2019/08/08/1160695/original.jpg
GV_1805	45.6697	-111.0493	1486.6		https://www.google.com/maps/search/45.6697,-111.0493	https://data.globe.gov/system/photos/2019/08/08/1160696/original.jpg	https://data.globe.gov/system/photos/2019/08/08/1160698/original.jpg	https://data.globe.gov/system/photos/2019/08/08/1160697/original.jpg	https://data.globe.gov/system/photos/2019/08/08/1160699/original.jpg	https://data.globe.gov/system/photos/2019/08/08/1160701/original.jpg
GV_1806	45.6688	-111.0495	1491.5		https://www.google.com/maps/search/45.6688,-111.0495	https://data.globe.gov/system/photos/2019/08/08/1160702/original.jpg	https://data.globe.gov/system/photos/2019/08/08/1160704/original.jpg	https://data.globe.gov/system/photos/2019/08/08/1160703/original.jpg	https://data.globe.gov/system/photos/2019/08/08/1160705/original.jpg	https://data.globe.gov/system/photos/2019/08/08/1160707/original.jpg
GV_1807	45.6665	-111.0502	1496.8		https://www.google.com/maps/search/45.6665,-111.0502	https://data.globe.gov/system/photos/2019/08/08/1160708/original.jpg	https://data.globe.gov/system/photos/2019/08/08/1160710/original.jpg	https://data.globe.gov/system/photos/2019/08/08/1160709/original.jpg	https://data.globe.gov/system/photos/2019/08/08/1160711/original.jpg	https://data.globe.gov/system/photos/2019/08/08/1160713/original.jpg
GV_1808	45.6671	-111.0492	1498.6		https://www.google.com/maps/search/45.6671,-111.0492	https://data.globe.gov/system/photos/2019/08/08/1160714/original.jpg	https://data.globe.gov/system/photos/2019/08/08/1160716/original.jpg	https://data.globe.gov/system/photos/2019/08/08/1160715/original.jpg	https://data.globe.gov/system/photos/2019/08/08/1160717/original.jpg	https://data.globe.gov/system/photos/2019/08/08/1160719/original.jpg
GV_1809	45.6671	-111.0497	1498.4		https://www.google.com/maps/search/45.6671,-111.0497	https://data.globe.gov/system/photos/2019/08/08/1160720/original.jpg	https://data.globe.gov/system/photos/2019/08/08/1160722/original.jpg	https://data.globe.gov/system/photos/2019/08/08/1160721/original.jpg	https://data.globe.gov/system/photos/2019/08/08/1160723/original.jpg	https://data.globe.gov/system/photos/2019/08/08/1160725/original.jpg
GV_1810	45.6661	-111.0494	1497.8		https://www.google.com/maps/search/45.6661,-111.0494	https://data.globe.gov/system/photos/2019/08/08/1160726/original.jpg	https://data.globe.gov/system/photos/2019/08/08/1160728/original.jpg	https://data.globe.gov/system/photos/2019/08/08/1160727/original.jpg	https://data.globe.gov/system/photos/2019/08/08/1160729/original.jpg	https://data.globe.gov/system/photos/2019/08/08/1160731/original.jpg
GV_1811	45.6656	-111.0488	1499.2		https://www.google.com/maps/search/45.6656,-111.0488	https://data.globe.gov/system/photos/2019/08/08/1160732/original.jpg	https://data.globe.gov/system/photos/2019/08/08/1160734/original.jpg	https://data.globe.gov/system/photos/2019/08/08/1160733/original.jpg	https://data.globe.gov/system/photos/2019/08/08/1160735/original.jpg	https://data.globe.gov/system/photos/2019/08/08/1160737/original.jpg
GV_1812	45.6664	-111.0484	1499.3		https://www.google.com/maps/search/45.6664,-111.0484	https://data.globe.gov/system/photos/2019/08/08/1160738/original.jpg	https://data.globe.gov/system/photos/2019/08/08/1160740/original.jpg	https://data.globe.gov/system/photos/2019/08/08/1160739/original.jpg	https://data.globe.gov/system/photos/2019/08/08/1160741/original.jpg	https://data.globe.gov/system/photos/2019/08/08/1160743/original.jpg
GV_1813	45.6664	-111.0511	1493.9		https://www.google.com/maps/search/45.6664,-111.0511	https://data.globe.gov/system/photos/2019/08/08/1160861/original.jpg	https://data.globe.gov/system/photos/2019/08/08/1160863/original.jpg	https://data.globe.gov/system/photos/2019/08/08/1160862/original.jpg	https://data.globe.gov/system/photos/2019/08/08/1160864/original.jpg	https://data.globe.gov/system/photos/2019/08/08/1160866/original.jpg
GV_1814	45.6664	-111.0517	1492.4		https://www.google.com/maps/search/45.6664,-111.0517	https://data.globe.gov/system/photos/2019/08/08/1160867/original.jpg	https://data.globe.gov/system/photos/2019/08/08/1160869/original.jpg	https://data.globe.gov/system/photos/2019/08/08/1160868/original.jpg	https://data.globe.gov/system/photos/2019/08/08/1160870/original.jpg	https://data.globe.gov/system/photos/2019/08/08/1160872/original.jpg
GV_1815	45.6655	-111.0488	1499.4		https://www.google.com/maps/search/45.6655,-111.0488	https://data.globe.gov/system/photos/2019/08/08/1160873/original.jpg	https://data.globe.gov/system/photos/2019/08/08/1160881/original.jpg	https://data.globe.gov/system/photos/2019/08/08/1160877/original.jpg	https://data.globe.gov/system/photos/2019/08/08/1160882/original.jpg	https://data.globe.gov/system/photos/2019/08/08/1160884/original.jpg
GV_1816	45.665	-111.0485	1498.9		https://www.google.com/maps/search/45.665,-111.0485	https://data.globe.gov/system/photos/2019/08/08/1160885/original.jpg	https://data.globe.gov/system/photos/2019/08/08/1160892/original.jpg	https://data.globe.gov/system/photos/2019/08/08/1160888/original.jpg	https://data.globe.gov/system/photos/2019/08/08/1160894/original.jpg	https://data.globe.gov/system/photos/2019/08/08/1160897/original.jpg
GV_1817	45.6651	-111.048	1499.2		https://www.google.com/maps/search/45.6651,-111.048	https://data.globe.gov/system/photos/2019/08/08/1160898/original.jpg	https://data.globe.gov/system/photos/2019/08/08/1160900/original.jpg	https://data.globe.gov/system/photos/2019/08/08/1160899/original.jpg	https://data.globe.gov/system/photos/2019/08/08/1160901/original.jpg	https://data.globe.gov/system/photos/2019/08/08/1160903/original.jpg
GV_1818	45.6651	-111.0497	1498.8		https://www.google.com/maps/search/45.6651,-111.0497	https://data.globe.gov/system/photos/2019/08/08/1160984/original.jpg	https://data.globe.gov/system/photos/2019/08/08/1160986/original.jpg	https://data.globe.gov/system/photos/2019/08/08/1160985/original.jpg	https://data.globe.gov/system/photos/2019/08/08/1160987/original.jpg	https://data.globe.gov/system/photos/2019/08/08/1160989/original.jpg
GV_1819	45.6649	-111.0507	1498.4		https://www.google.com/maps/search/45.6649,-111.0507	https://data.globe.gov/system/photos/2019/08/08/1160990/original.jpg	https://data.globe.gov/system/photos/2019/08/08/1160992/original.jpg	https://data.globe.gov/system/photos/2019/08/08/1160991/original.jpg	https://data.globe.gov/system/photos/2019/08/08/1160993/original.jpg	https://data.globe.gov/system/photos/2019/08/08/1160995/original.jpg
GV_1820	45.664	-111.0509	1498.8		https://www.google.com/maps/search/45.664,-111.0509	https://data.globe.gov/system/photos/2019/08/08/1160996/original.jpg	https://data.globe.gov/system/photos/2019/08/08/1160998/original.jpg	https://data.globe.gov/system/photos/2019/08/08/1160997/original.jpg	https://data.globe.gov/system/photos/2019/08/08/1160999/original.jpg	https://data.globe.gov/system/photos/2019/08/08/1161001/original.jpg
GV_1821	45.6634	-111.0514	1498.7		https://www.google.com/maps/search/45.6634,-111.0514	https://data.globe.gov/system/photos/2019/08/08/1161002/original.jpg	https://data.globe.gov/system/photos/2019/08/08/1161004/original.jpg	https://data.globe.gov/system/photos/2019/08/08/1161003/original.jpg	https://data.globe.gov/system/photos/2019/08/08/1161005/original.jpg	https://data.globe.gov/system/photos/2019/08/08/1161007/original.jpg
GV_1822	45.6627	-111.0507	1501.7		https://www.google.com/maps/search/45.6627,-111.0507	https://data.globe.gov/system/photos/2019/08/08/1161008/original.jpg	https://data.globe.gov/system/photos/2019/08/08/1161010/original.jpg	https://data.globe.gov/system/photos/2019/08/08/1161009/original.jpg	https://data.globe.gov/system/photos/2019/08/08/1161011/original.jpg	https://data.globe.gov/system/photos/2019/08/08/1161013/original.jpg
GV_1823	45.6639	-111.053	1494.2		https://www.google.com/maps/search/45.6639,-111.053	https://data.globe.gov/system/photos/2019/08/08/1161014/original.jpg	https://data.globe.gov/system/photos/2019/08/08/1161016/original.jpg	https://data.globe.gov/system/photos/2019/08/08/1161015/original.jpg	https://data.globe.gov/system/photos/2019/08/08/1161017/original.jpg	https://data.globe.gov/system/photos/2019/08/08/1161019/original.jpg
GV_1824	45.6645	-111.0541	1492.8		https://www.google.com/maps/search/45.6645,-111.0541	https://data.globe.gov/system/photos/2019/08/08/1161020/original.jpg	https://data.globe.gov/system/photos/2019/08/08/1161022/original.jpg	https://data.globe.gov/system/photos/2019/08/08/1161021/original.jpg	https://data.globe.gov/system/photos/2019/08/08/1161023/original.jpg	https://data.globe.gov/system/photos/2019/08/08/1161025/original.jpg
GV_1825	45.665	-111.0558	1491.4		https://www.google.com/maps/search/45.665,-111.0558	https://data.globe.gov/system/photos/2019/08/08/1161026/original.jpg	https://data.globe.gov/system/photos/2019/08/08/1161028/original.jpg	https://data.globe.gov/system/photos/2019/08/08/1161027/original.jpg	https://data.globe.gov/system/photos/2019/08/08/1161029/original.jpg	https://data.globe.gov/system/photos/2019/08/08/1161031/original.jpg
GV_1826	45.6655	-111.055	1488.4		https://www.google.com/maps/search/45.6655,-111.055	https://data.globe.gov/system/photos/2019/08/08/1161032/original.jpg	https://data.globe.gov/system/photos/2019/08/08/1161034/original.jpg	https://data.globe.gov/system/photos/2019/08/08/1161033/original.jpg	https://data.globe.gov/system/photos/2019/08/08/1161035/original.jpg	https://data.globe.gov/system/photos/2019/08/08/1161037/original.jpg
GV_1827	45.6654	-111.0553	1488.1		https://www.google.com/maps/search/45.6654,-111.0553	https://data.globe.gov/system/photos/2019/08/08/1161038/original.jpg	https://data.globe.gov/system/photos/2019/08/08/1161040/original.jpg	https://data.globe.gov/system/photos/2019/08/08/1161039/original.jpg	https://data.globe.gov/system/photos/2019/08/08/1161041/original.jpg	https://data.globe.gov/system/photos/2019/08/08/1161043/original.jpg
GV_1828	45.6693	-111.0487	1489.1		https://www.google.com/maps/search/45.6693,-111.0487	https://data.globe.gov/system/photos/2019/08/08/1161044/original.jpg	https://data.globe.gov/system/photos/2019/08/08/1161046/original.jpg	https://data.globe.gov/system/photos/2019/08/08/1161045/original.jpg	https://data.globe.gov/system/photos/2019/08/08/1161047/original.jpg	https://data.globe.gov/system/photos/2019/08/08/1161049/original.jpg
GV_1829	45.6693	-111.0475	1490.7		https://www.google.com/maps/search/45.6693,-111.0475	https://data.globe.gov/system/photos/2019/08/08/1161050/original.jpg	https://data.globe.gov/system/photos/2019/08/08/1161052/original.jpg	https://data.globe.gov/system/photos/2019/08/08/1161051/original.jpg	https://data.globe.gov/system/photos/2019/08/08/1161053/original.jpg	https://data.globe.gov/system/photos/2019/08/08/1161055/original.jpg
GV_1830	45.6687	-111.0494	1491.8		https://www.google.com/maps/search/45.6687,-111.0494	https://data.globe.gov/system/photos/2019/08/08/1161056/original.jpg	https://data.globe.gov/system/photos/2019/08/08/1161058/original.jpg	https://data.globe.gov/system/photos/2019/08/08/1161057/original.jpg	https://data.globe.gov/system/photos/2019/08/08/1161059/original.jpg	https://data.globe.gov/system/photos/2019/08/08/1161061/original.jpg
GV_1831	45.6687	-111.0483	1494.9		https://www.google.com/maps/search/45.6687,-111.0483	https://data.globe.gov/system/photos/2019/08/08/1161062/original.jpg	https://data.globe.gov/system/photos/2019/08/08/1161064/original.jpg	https://data.globe.gov/system/photos/2019/08/08/1161063/original.jpg	https://data.globe.gov/system/photos/2019/08/08/1161065/original.jpg	https://data.globe.gov/system/photos/2019/08/08/1161067/original.jpg
GV_1832	45.6694	-111.0498	1487.8		https://www.google.com/maps/search/45.6694,-111.0498	https://data.globe.gov/system/photos/2019/08/08/1161068/original.jpg	https://data.globe.gov/system/photos/2019/08/08/1161070/original.jpg	https://data.globe.gov/system/photos/2019/08/08/1161069/original.jpg	https://data.globe.gov/system/photos/2019/08/08/1161071/original.jpg	https://data.globe.gov/system/photos/2019/08/08/1161073/original.jpg
GV_1833	45.6792	-111.0487	1466.9		https://www.google.com/maps/search/45.6792,-111.0487	https://data.globe.gov/system/photos/2019/08/08/1161109/original.jpg	https://data.globe.gov/system/photos/2019/08/08/1161111/original.jpg	https://data.globe.gov/system/photos/2019/08/08/1161110/original.jpg	https://data.globe.gov/system/photos/2019/08/08/1161112/original.jpg	https://data.globe.gov/system/photos/2019/08/08/1161114/original.jpg
GV_1834	45.6795	-111.0495	1466.4		https://www.google.com/maps/search/45.6795,-111.0495	https://data.globe.gov/system/photos/2019/08/08/1161115/original.jpg	https://data.globe.gov/system/photos/2019/08/08/1161117/original.jpg	https://data.globe.gov/system/photos/2019/08/08/1161116/original.jpg	https://data.globe.gov/system/photos/2019/08/08/1161118/original.jpg	https://data.globe.gov/system/photos/2019/08/08/1161120/original.jpg
GV_1835	45.6804	-111.0517	1466.1		https://www.google.com/maps/search/45.6804,-111.0517	https://data.globe.gov/system/photos/2019/08/08/1161121/original.jpg	https://data.globe.gov/system/photos/2019/08/08/1161123/original.jpg	https://data.globe.gov/system/photos/2019/08/08/1161122/original.jpg	https://data.globe.gov/system/photos/2019/08/08/1161124/original.jpg	https://data.globe.gov/system/photos/2019/08/08/1161126/original.jpg
GV_1836	45.6836	-111.0516	1461.7		https://www.google.com/maps/search/45.6836,-111.0516	https://data.globe.gov/system/photos/2019/08/08/1161127/original.jpg	https://data.globe.gov/system/photos/2019/08/08/1161129/original.jpg	https://data.globe.gov/system/photos/2019/08/08/1161128/original.jpg	https://data.globe.gov/system/photos/2019/08/08/1161130/original.jpg	https://data.globe.gov/system/photos/2019/08/08/1161132/original.jpg
GV_1837	45.6827	-111.0517	1463.3		https://www.google.com/maps/search/45.6827,-111.0517	https://data.globe.gov/system/photos/2019/08/08/1161133/original.jpg	https://data.globe.gov/system/photos/2019/08/08/1161135/original.jpg	https://data.globe.gov/system/photos/2019/08/08/1161134/original.jpg	https://data.globe.gov/system/photos/2019/08/08/1161136/original.jpg	https://data.globe.gov/system/photos/2019/08/08/1161138/original.jpg
GV_1838	45.6812	-111.0502	1464.5		https://www.google.com/maps/search/45.6812,-111.0502	https://data.globe.gov/system/photos/2019/08/08/1161139/original.jpg	https://data.globe.gov/system/photos/2019/08/08/1161141/original.jpg	https://data.globe.gov/system/photos/2019/08/08/1161140/original.jpg	https://data.globe.gov/system/photos/2019/08/08/1161142/original.jpg	https://data.globe.gov/system/photos/2019/08/08/1161144/original.jpg
GV_1839	45.6804	-111.0497	1465.3		https://www.google.com/maps/search/45.6804,-111.0497	https://data.globe.gov/system/photos/2019/08/08/1161145/original.jpg	https://data.globe.gov/system/photos/2019/08/08/1161147/original.jpg	https://data.globe.gov/system/photos/2019/08/08/1161146/original.jpg	https://data.globe.gov/system/photos/2019/08/08/1161148/original.jpg	https://data.globe.gov/system/photos/2019/08/08/1161150/original.jpg
GV_1840	45.6797	-111.0489	1466.6		https://www.google.com/maps/search/45.6797,-111.0489	https://data.globe.gov/system/photos/2019/08/08/1161151/original.jpg	https://data.globe.gov/system/photos/2019/08/08/1161153/original.jpg	https://data.globe.gov/system/photos/2019/08/08/1161152/original.jpg	https://data.globe.gov/system/photos/2019/08/08/1161154/original.jpg	https://data.globe.gov/system/photos/2019/08/08/1161156/original.jpg
GV_1841	45.6929	-111.0359	1448		https://www.google.com/maps/search/45.6929,-111.0359	https://data.globe.gov/system/photos/2019/08/09/1161158/original.jpg	https://data.globe.gov/system/photos/2019/08/09/1161160/original.jpg	https://data.globe.gov/system/photos/2019/08/09/1161159/original.jpg	https://data.globe.gov/system/photos/2019/08/09/1161161/original.jpg	https://data.globe.gov/system/photos/2019/08/09/1161163/original.jpg
GV_1842	45.692	-111.0334	1447.9		https://www.google.com/maps/search/45.692,-111.0334	https://data.globe.gov/system/photos/2019/08/09/1161164/original.jpg	https://data.globe.gov/system/photos/2019/08/09/1161166/original.jpg	https://data.globe.gov/system/photos/2019/08/09/1161165/original.jpg	https://data.globe.gov/system/photos/2019/08/09/1161167/original.jpg	https://data.globe.gov/system/photos/2019/08/09/1161169/original.jpg
GV_1843	45.6916	-111.0323	1449.3		https://www.google.com/maps/search/45.6916,-111.0323	https://data.globe.gov/system/photos/2019/08/09/1161170/original.jpg	https://data.globe.gov/system/photos/2019/08/09/1161172/original.jpg	https://data.globe.gov/system/photos/2019/08/09/1161171/original.jpg	https://data.globe.gov/system/photos/2019/08/09/1161173/original.jpg	https://data.globe.gov/system/photos/2019/08/09/1161175/original.jpg
GV_1844	45.6911	-111.0317	1450.2		https://www.google.com/maps/search/45.6911,-111.0317	https://data.globe.gov/system/photos/2019/08/09/1161176/original.jpg	https://data.globe.gov/system/photos/2019/08/09/1161178/original.jpg	https://data.globe.gov/system/photos/2019/08/09/1161177/original.jpg	https://data.globe.gov/system/photos/2019/08/09/1161179/original.jpg	https://data.globe.gov/system/photos/2019/08/09/1161181/original.jpg
GV_1845	45.693	-111.0427	1447.6		https://www.google.com/maps/search/45.693,-111.0427	https://data.globe.gov/system/photos/2019/08/09/1161182/original.jpg	https://data.globe.gov/system/photos/2019/08/09/1161184/original.jpg	https://data.globe.gov/system/photos/2019/08/09/1161183/original.jpg	https://data.globe.gov/system/photos/2019/08/09/1161185/original.jpg	https://data.globe.gov/system/photos/2019/08/09/1161187/original.jpg
GV_1846	45.6952	-111.0558	1446.1		https://www.google.com/maps/search/45.6952,-111.0558	https://data.globe.gov/system/photos/2019/08/09/1161188/original.jpg	https://data.globe.gov/system/photos/2019/08/09/1161190/original.jpg	https://data.globe.gov/system/photos/2019/08/09/1161189/original.jpg	https://data.globe.gov/system/photos/2019/08/09/1161191/original.jpg	https://data.globe.gov/system/photos/2019/08/09/1161193/original.jpg
GV_1847	45.6784	-111.0471	1470.7		https://www.google.com/maps/search/45.6784,-111.0471	https://data.globe.gov/system/photos/2019/08/09/1161194/original.jpg	https://data.globe.gov/system/photos/2019/08/09/1161196/original.jpg	https://data.globe.gov/system/photos/2019/08/09/1161195/original.jpg	https://data.globe.gov/system/photos/2019/08/09/1161197/original.jpg	https://data.globe.gov/system/photos/2019/08/09/1161199/original.jpg
GV_1848	45.6676	-111.0732	1487.3		https://www.google.com/maps/search/45.6676,-111.0732	https://data.globe.gov/system/photos/2019/08/09/1161200/original.jpg	https://data.globe.gov/system/photos/2019/08/09/1161202/original.jpg	https://data.globe.gov/system/photos/2019/08/09/1161201/original.jpg	https://data.globe.gov/system/photos/2019/08/09/1161203/original.jpg	https://data.globe.gov/system/photos/2019/08/09/1161205/original.jpg
GV_1849	53.4283	-0.6226	26		https://www.google.com/maps/search/53.4283,-0.6226	https://data.globe.gov/system/photos/2019/08/08/1161344/original.jpg	https://data.globe.gov/system/photos/2019/08/08/1161346/original.jpg	https://data.globe.gov/system/photos/2019/08/08/1161345/original.jpg	https://data.globe.gov/system/photos/2019/08/08/1161347/original.jpg	https://data.globe.gov/system/photos/2019/08/08/1161349/original.jpg
GV_1850	46.7513	-114.0889	973.5		https://www.google.com/maps/search/46.7513,-114.0889	https://data.globe.gov/system/photos/2019/08/09/1161637/original.jpg	https://data.globe.gov/system/photos/2019/08/09/1161639/original.jpg	https://data.globe.gov/system/photos/2019/08/09/1161638/original.jpg	https://data.globe.gov/system/photos/2019/08/09/1161640/original.jpg	https://data.globe.gov/system/photos/2019/08/09/1161642/original.jpg
GV_1851	46.7497	-114.0891	972.1		https://www.google.com/maps/search/46.7497,-114.0891	https://data.globe.gov/system/photos/2019/08/09/1161643/original.jpg	https://data.globe.gov/system/photos/2019/08/09/1161645/original.jpg	https://data.globe.gov/system/photos/2019/08/09/1161644/original.jpg	https://data.globe.gov/system/photos/2019/08/09/1161646/original.jpg	https://data.globe.gov/system/photos/2019/08/09/1161648/original.jpg
GV_1852	46.7498	-114.0872	972.1		https://www.google.com/maps/search/46.7498,-114.0872	https://data.globe.gov/system/photos/2019/08/09/1161649/original.jpg	https://data.globe.gov/system/photos/2019/08/09/1161651/original.jpg	https://data.globe.gov/system/photos/2019/08/09/1161650/original.jpg	https://data.globe.gov/system/photos/2019/08/09/1161652/original.jpg	https://data.globe.gov/system/photos/2019/08/09/1161654/original.jpg
GV_1853	46.7504	-114.0875	971.3		https://www.google.com/maps/search/46.7504,-114.0875	https://data.globe.gov/system/photos/2019/08/09/1161655/original.jpg	https://data.globe.gov/system/photos/2019/08/09/1161657/original.jpg	https://data.globe.gov/system/photos/2019/08/09/1161656/original.jpg	https://data.globe.gov/system/photos/2019/08/09/1161658/original.jpg	https://data.globe.gov/system/photos/2019/08/09/1161660/original.jpg
GV_1854	53.4284	-0.6226	26		https://www.google.com/maps/search/53.4284,-0.6226	https://data.globe.gov/system/photos/2019/08/09/1161825/original.jpg	https://data.globe.gov/system/photos/2019/08/09/1161832/original.jpg	https://data.globe.gov/system/photos/2019/08/09/1161829/original.jpg	https://data.globe.gov/system/photos/2019/08/09/1161835/original.jpg	https://data.globe.gov/system/photos/2019/08/09/1161841/original.jpg
GV_1855	45.5916	-111.2058	1498.4		https://www.google.com/maps/search/45.5916,-111.2058	https://data.globe.gov/system/photos/2019/08/09/1161940/original.jpg	https://data.globe.gov/system/photos/2019/08/09/1161942/original.jpg	https://data.globe.gov/system/photos/2019/08/09/1161941/original.jpg	https://data.globe.gov/system/photos/2019/08/09/1161943/original.jpg	https://data.globe.gov/system/photos/2019/08/09/1161945/original.jpg
GV_1856	45.5981	-111.2067	1492.5		https://www.google.com/maps/search/45.5981,-111.2067	https://data.globe.gov/system/photos/2019/08/09/1161952/original.jpg	https://data.globe.gov/system/photos/2019/08/09/1161954/original.jpg	https://data.globe.gov/system/photos/2019/08/09/1161953/original.jpg	https://data.globe.gov/system/photos/2019/08/09/1161955/original.jpg	https://data.globe.gov/system/photos/2019/08/09/1161957/original.jpg
GV_1857	48.6336	-112.3466	1088.7		https://www.google.com/maps/search/48.6336,-112.3466	https://data.globe.gov/system/photos/2019/08/08/1161991/original.jpg	https://data.globe.gov/system/photos/2019/08/08/1161993/original.jpg	https://data.globe.gov/system/photos/2019/08/08/1161992/original.jpg	https://data.globe.gov/system/photos/2019/08/08/1161994/original.jpg	https://data.globe.gov/system/photos/2019/08/08/1161996/original.jpg
GV_1858	48.5991	-112.798	1270.3		https://www.google.com/maps/search/48.5991,-112.798	https://data.globe.gov/system/photos/2019/08/08/1161997/original.jpg	https://data.globe.gov/system/photos/2019/08/08/1161999/original.jpg	https://data.globe.gov/system/photos/2019/08/08/1161998/original.jpg	https://data.globe.gov/system/photos/2019/08/08/1162000/original.jpg	https://data.globe.gov/system/photos/2019/08/08/1162002/original.jpg
GV_1859	48.4593	-112.4546	1126.6		https://www.google.com/maps/search/48.4593,-112.4546	https://data.globe.gov/system/photos/2019/08/09/1162003/original.jpg	https://data.globe.gov/system/photos/2019/08/09/1162005/original.jpg	https://data.globe.gov/system/photos/2019/08/09/1162004/original.jpg	https://data.globe.gov/system/photos/2019/08/09/1162006/original.jpg	https://data.globe.gov/system/photos/2019/08/09/1162008/original.jpg
GV_1860	48.4614	-112.4594	1105		https://www.google.com/maps/search/48.4614,-112.4594	https://data.globe.gov/system/photos/2019/08/09/1162015/original.jpg	https://data.globe.gov/system/photos/2019/08/09/1162017/original.jpg	https://data.globe.gov/system/photos/2019/08/09/1162016/original.jpg	https://data.globe.gov/system/photos/2019/08/09/1162018/original.jpg	https://data.globe.gov/system/photos/2019/08/09/1162020/original.jpg
GV_1861	46.4048	-112.7348	1379.2		https://www.google.com/maps/search/46.4048,-112.7348	https://data.globe.gov/system/photos/2019/08/09/1162157/original.jpg	https://data.globe.gov/system/photos/2019/08/09/1162159/original.jpg	https://data.globe.gov/system/photos/2019/08/09/1162158/original.jpg	https://data.globe.gov/system/photos/2019/08/09/1162160/original.jpg	https://data.globe.gov/system/photos/2019/08/09/1162162/original.jpg
GV_1862	46.3973	-112.7355	1381		https://www.google.com/maps/search/46.3973,-112.7355	https://data.globe.gov/system/photos/2019/08/09/1162163/original.jpg	https://data.globe.gov/system/photos/2019/08/09/1162165/original.jpg	https://data.globe.gov/system/photos/2019/08/09/1162164/original.jpg	https://data.globe.gov/system/photos/2019/08/09/1162166/original.jpg	https://data.globe.gov/system/photos/2019/08/09/1162168/original.jpg
GV_1863	46.703	-113.3456	1154.3		https://www.google.com/maps/search/46.703,-113.3456	https://data.globe.gov/system/photos/2019/08/09/1162170/original.jpg	https://data.globe.gov/system/photos/2019/08/09/1162172/original.jpg	https://data.globe.gov/system/photos/2019/08/09/1162171/original.jpg	https://data.globe.gov/system/photos/2019/08/09/1162173/original.jpg	https://data.globe.gov/system/photos/2019/08/09/1162175/original.jpg
GV_1864	46.7027	-113.3455	1155.5		https://www.google.com/maps/search/46.7027,-113.3455	https://data.globe.gov/system/photos/2019/08/09/1162176/original.jpg	https://data.globe.gov/system/photos/2019/08/09/1162178/original.jpg	https://data.globe.gov/system/photos/2019/08/09/1162177/original.jpg	https://data.globe.gov/system/photos/2019/08/09/1162179/original.jpg	https://data.globe.gov/system/photos/2019/08/09/1162181/original.jpg
GV_1865	46.8642	-113.9517	973		https://www.google.com/maps/search/46.8642,-113.9517	https://data.globe.gov/system/photos/2019/08/09/1162182/original.jpg	https://data.globe.gov/system/photos/2019/08/09/1162184/original.jpg	https://data.globe.gov/system/photos/2019/08/09/1162183/original.jpg	https://data.globe.gov/system/photos/2019/08/09/1162185/original.jpg	https://data.globe.gov/system/photos/2019/08/09/1162187/original.jpg
GV_1866	46.8647	-113.9518	980.8		https://www.google.com/maps/search/46.8647,-113.9518	https://data.globe.gov/system/photos/2019/08/09/1162189/original.jpg	https://data.globe.gov/system/photos/2019/08/09/1162191/original.jpg	https://data.globe.gov/system/photos/2019/08/09/1162190/original.jpg	https://data.globe.gov/system/photos/2019/08/09/1162192/original.jpg	https://data.globe.gov/system/photos/2019/08/09/1162194/original.jpg
GV_1867	46.8674	-113.9836	974.1		https://www.google.com/maps/search/46.8674,-113.9836	https://data.globe.gov/system/photos/2019/08/09/1162195/original.jpg	https://data.globe.gov/system/photos/2019/08/09/1162197/original.jpg	https://data.globe.gov/system/photos/2019/08/09/1162196/original.jpg	https://data.globe.gov/system/photos/2019/08/09/1162198/original.jpg	https://data.globe.gov/system/photos/2019/08/09/1162200/original.jpg
GV_1868	46.8674	-113.9835	974		https://www.google.com/maps/search/46.8674,-113.9835	https://data.globe.gov/system/photos/2019/08/09/1162201/original.jpg	https://data.globe.gov/system/photos/2019/08/09/1162203/original.jpg	https://data.globe.gov/system/photos/2019/08/09/1162202/original.jpg	https://data.globe.gov/system/photos/2019/08/09/1162204/original.jpg	https://data.globe.gov/system/photos/2019/08/09/1162206/original.jpg
GV_1869	46.8666	-113.9843	972.5		https://www.google.com/maps/search/46.8666,-113.9843	https://data.globe.gov/system/photos/2019/08/09/1162207/original.jpg	https://data.globe.gov/system/photos/2019/08/09/1162209/original.jpg	https://data.globe.gov/system/photos/2019/08/09/1162208/original.jpg	https://data.globe.gov/system/photos/2019/08/09/1162210/original.jpg	https://data.globe.gov/system/photos/2019/08/09/1162212/original.jpg
GV_1870	46.8671	-113.9848	972.6		https://www.google.com/maps/search/46.8671,-113.9848	https://data.globe.gov/system/photos/2019/08/09/1162213/original.jpg	https://data.globe.gov/system/photos/2019/08/09/1162215/original.jpg	https://data.globe.gov/system/photos/2019/08/09/1162214/original.jpg	https://data.globe.gov/system/photos/2019/08/09/1162216/original.jpg	https://data.globe.gov/system/photos/2019/08/09/1162218/original.jpg
GV_1871	46.8671	-113.9841	970.3		https://www.google.com/maps/search/46.8671,-113.9841	https://data.globe.gov/system/photos/2019/08/09/1162219/original.jpg	https://data.globe.gov/system/photos/2019/08/09/1162221/original.jpg	https://data.globe.gov/system/photos/2019/08/09/1162220/original.jpg	https://data.globe.gov/system/photos/2019/08/09/1162222/original.jpg	https://data.globe.gov/system/photos/2019/08/09/1162224/original.jpg
GV_1872	46.8659	-113.9835	974.9		https://www.google.com/maps/search/46.8659,-113.9835	https://data.globe.gov/system/photos/2019/08/09/1162225/original.jpg	https://data.globe.gov/system/photos/2019/08/09/1162227/original.jpg	https://data.globe.gov/system/photos/2019/08/09/1162226/original.jpg	https://data.globe.gov/system/photos/2019/08/09/1162228/original.jpg	https://data.globe.gov/system/photos/2019/08/09/1162230/original.jpg
GV_1873	46.8655	-113.9835	975.7		https://www.google.com/maps/search/46.8655,-113.9835	https://data.globe.gov/system/photos/2019/08/09/1162232/original.jpg	https://data.globe.gov/system/photos/2019/08/09/1162234/original.jpg	https://data.globe.gov/system/photos/2019/08/09/1162233/original.jpg	https://data.globe.gov/system/photos/2019/08/09/1162235/original.jpg	https://data.globe.gov/system/photos/2019/08/09/1162237/original.jpg
GV_1874	46.8656	-113.9853	974.8		https://www.google.com/maps/search/46.8656,-113.9853	https://data.globe.gov/system/photos/2019/08/09/1162238/original.jpg	https://data.globe.gov/system/photos/2019/08/09/1162240/original.jpg	https://data.globe.gov/system/photos/2019/08/09/1162239/original.jpg	https://data.globe.gov/system/photos/2019/08/09/1162241/original.jpg	https://data.globe.gov/system/photos/2019/08/09/1162243/original.jpg
GV_1875	46.8641	-113.9845	976.2		https://www.google.com/maps/search/46.8641,-113.9845	https://data.globe.gov/system/photos/2019/08/09/1162245/original.jpg	https://data.globe.gov/system/photos/2019/08/09/1162247/original.jpg	https://data.globe.gov/system/photos/2019/08/09/1162246/original.jpg	https://data.globe.gov/system/photos/2019/08/09/1162248/original.jpg	https://data.globe.gov/system/photos/2019/08/09/1162250/original.jpg
GV_1876	46.8636	-113.9844	977.7		https://www.google.com/maps/search/46.8636,-113.9844	https://data.globe.gov/system/photos/2019/08/09/1162251/original.jpg	https://data.globe.gov/system/photos/2019/08/09/1162253/original.jpg	https://data.globe.gov/system/photos/2019/08/09/1162252/original.jpg	https://data.globe.gov/system/photos/2019/08/09/1162254/original.jpg	https://data.globe.gov/system/photos/2019/08/09/1162256/original.jpg
GV_1877	46.8631	-113.9849	978.2		https://www.google.com/maps/search/46.8631,-113.9849	https://data.globe.gov/system/photos/2019/08/09/1162257/original.jpg	https://data.globe.gov/system/photos/2019/08/09/1162259/original.jpg	https://data.globe.gov/system/photos/2019/08/09/1162258/original.jpg	https://data.globe.gov/system/photos/2019/08/09/1162260/original.jpg	https://data.globe.gov/system/photos/2019/08/09/1162262/original.jpg
GV_1878	46.8634	-113.9859	978.9		https://www.google.com/maps/search/46.8634,-113.9859	https://data.globe.gov/system/photos/2019/08/09/1162263/original.jpg	https://data.globe.gov/system/photos/2019/08/09/1162265/original.jpg	https://data.globe.gov/system/photos/2019/08/09/1162264/original.jpg	https://data.globe.gov/system/photos/2019/08/09/1162266/original.jpg	https://data.globe.gov/system/photos/2019/08/09/1162268/original.jpg
GV_1879	46.8645	-113.9856	975.9		https://www.google.com/maps/search/46.8645,-113.9856	https://data.globe.gov/system/photos/2019/08/09/1162270/original.jpg	https://data.globe.gov/system/photos/2019/08/09/1162272/original.jpg	https://data.globe.gov/system/photos/2019/08/09/1162271/original.jpg	https://data.globe.gov/system/photos/2019/08/09/1162273/original.jpg	https://data.globe.gov/system/photos/2019/08/09/1162275/original.jpg
GV_1880	46.8601	-113.9894	980.9		https://www.google.com/maps/search/46.8601,-113.9894	https://data.globe.gov/system/photos/2019/08/09/1162276/original.jpg	https://data.globe.gov/system/photos/2019/08/09/1162278/original.jpg	https://data.globe.gov/system/photos/2019/08/09/1162277/original.jpg	https://data.globe.gov/system/photos/2019/08/09/1162279/original.jpg	https://data.globe.gov/system/photos/2019/08/09/1162281/original.jpg
GV_1881	46.8732	-113.9956	977.1		https://www.google.com/maps/search/46.8732,-113.9956	https://data.globe.gov/system/photos/2019/08/09/1162282/original.jpg	https://data.globe.gov/system/photos/2019/08/09/1162284/original.jpg	https://data.globe.gov/system/photos/2019/08/09/1162283/original.jpg	https://data.globe.gov/system/photos/2019/08/09/1162285/original.jpg	https://data.globe.gov/system/photos/2019/08/09/1162287/original.jpg
GV_1882	46.8522	-114.0135	971.8		https://www.google.com/maps/search/46.8522,-114.0135	https://data.globe.gov/system/photos/2019/08/09/1162288/original.jpg	https://data.globe.gov/system/photos/2019/08/09/1162290/original.jpg	https://data.globe.gov/system/photos/2019/08/09/1162289/original.jpg	https://data.globe.gov/system/photos/2019/08/09/1162291/original.jpg	https://data.globe.gov/system/photos/2019/08/09/1162293/original.jpg
GV_1883	46.7475	-114.0897	975.9		https://www.google.com/maps/search/46.7475,-114.0897	https://data.globe.gov/system/photos/2019/08/09/1162294/original.jpg	https://data.globe.gov/system/photos/2019/08/09/1162296/original.jpg	https://data.globe.gov/system/photos/2019/08/09/1162295/original.jpg	https://data.globe.gov/system/photos/2019/08/09/1162297/original.jpg	https://data.globe.gov/system/photos/2019/08/09/1162299/original.jpg

GV_1884	46.7564	-114.0894	972.6		https://www.google.com/maps/search/46.7564,-114.0894	https://data.globe.gov/system/photos/2019/08/09/1162300/original.jpg	https://data.globe.gov/system/photos/2019/08/09/1162302/original.jpg	https://data.globe.gov/system/photos/2019/08/09/1162301/original.jpg	https://data.globe.gov/system/photos/2019/08/09/1162303/original.jpg	https://data.globe.gov/system/photos/2019/08/09/1162305/original.jpg
GV_1885	46.7535	-114.0895	973		https://www.google.com/maps/search/46.7535,-114.0895	https://data.globe.gov/system/photos/2019/08/09/1162306/original.jpg	https://data.globe.gov/system/photos/2019/08/09/1162308/original.jpg	https://data.globe.gov/system/photos/2019/08/09/1162307/original.jpg	https://data.globe.gov/system/photos/2019/08/09/1162309/original.jpg	https://data.globe.gov/system/photos/2019/08/09/1162311/original.jpg
GV_1886	46.7527	-114.0895	973.3		https://www.google.com/maps/search/46.7527,-114.0895	https://data.globe.gov/system/photos/2019/08/09/1162312/original.jpg	https://data.globe.gov/system/photos/2019/08/09/1162314/original.jpg	https://data.globe.gov/system/photos/2019/08/09/1162313/original.jpg	https://data.globe.gov/system/photos/2019/08/09/1162315/original.jpg	https://data.globe.gov/system/photos/2019/08/09/1162317/original.jpg
GV_1887	46.7432	-114.1519	1009		https://www.google.com/maps/search/46.7432,-114.1519	https://data.globe.gov/system/photos/2019/08/09/1162318/original.jpg	https://data.globe.gov/system/photos/2019/08/09/1162320/original.jpg	https://data.globe.gov/system/photos/2019/08/09/1162319/original.jpg	https://data.globe.gov/system/photos/2019/08/09/1162321/original.jpg	https://data.globe.gov/system/photos/2019/08/09/1162323/original.jpg
GV_1888	46.7597	-114.3219	1108.4		https://www.google.com/maps/search/46.7597,-114.3219	https://data.globe.gov/system/photos/2019/08/09/1162324/original.jpg	https://data.globe.gov/system/photos/2019/08/09/1162326/original.jpg	https://data.globe.gov/system/photos/2019/08/09/1162325/original.jpg	https://data.globe.gov/system/photos/2019/08/09/1162327/original.jpg	https://data.globe.gov/system/photos/2019/08/09/1162329/original.jpg
GV_1889	46.7667	-114.3432	1122.6		https://www.google.com/maps/search/46.7667,-114.3432	https://data.globe.gov/system/photos/2019/08/09/1162330/original.jpg	https://data.globe.gov/system/photos/2019/08/09/1162332/original.jpg	https://data.globe.gov/system/photos/2019/08/09/1162331/original.jpg	https://data.globe.gov/system/photos/2019/08/09/1162333/original.jpg	https://data.globe.gov/system/photos/2019/08/09/1162335/original.jpg
GV_1890	46.6347	-114.5784	1593.1		https://www.google.com/maps/search/46.6347,-114.5784	https://data.globe.gov/system/photos/2019/08/09/1162337/original.jpg	https://data.globe.gov/system/photos/2019/08/09/1162339/original.jpg	https://data.globe.gov/system/photos/2019/08/09/1162338/original.jpg	https://data.globe.gov/system/photos/2019/08/09/1162340/original.jpg	https://data.globe.gov/system/photos/2019/08/09/1162342/original.jpg
GV_1891	46.7688	-114.4644	1198.8		https://www.google.com/maps/search/46.7688,-114.4644	https://data.globe.gov/system/photos/2019/08/09/1162343/original.jpg	https://data.globe.gov/system/photos/2019/08/09/1162346/original.jpg	https://data.globe.gov/system/photos/2019/08/09/1162345/original.jpg	https://data.globe.gov/system/photos/2019/08/09/1162347/original.jpg	https://data.globe.gov/system/photos/2019/08/09/1162349/original.jpg
GV_1892	46.7257	-114.5328	1261.6		https://www.google.com/maps/search/46.7257,-114.5328	https://data.globe.gov/system/photos/2019/08/09/1162351/original.jpg	https://data.globe.gov/system/photos/2019/08/09/1162353/original.jpg	https://data.globe.gov/system/photos/2019/08/09/1162352/original.jpg	https://data.globe.gov/system/photos/2019/08/09/1162355/original.jpg	https://data.globe.gov/system/photos/2019/08/09/1162357/original.jpg
GV_1893	46.66	-114.5825	1447.7		https://www.google.com/maps/search/46.66,-114.5825	https://data.globe.gov/system/photos/2019/08/09/1162358/original.jpg	https://data.globe.gov/system/photos/2019/08/09/1162361/original.jpg	https://data.globe.gov/system/photos/2019/08/09/1162360/original.jpg	https://data.globe.gov/system/photos/2019/08/09/1162362/original.jpg	https://data.globe.gov/system/photos/2019/08/09/1162364/original.jpg
GV_1894	46.6363	-114.5824	1590.9		https://www.google.com/maps/search/46.6363,-114.5824	https://data.globe.gov/system/photos/2019/08/09/1162365/original.jpg	https://data.globe.gov/system/photos/2019/08/09/1162368/original.jpg	https://data.globe.gov/system/photos/2019/08/09/1162366/original.jpg	https://data.globe.gov/system/photos/2019/08/09/1162369/original.jpg	https://data.globe.gov/system/photos/2019/08/09/1162371/original.jpg
GV_1895	46.6356	-114.58	1597.3		https://www.google.com/maps/search/46.6356,-114.58	https://data.globe.gov/system/photos/2019/08/09/1162372/original.jpg	https://data.globe.gov/system/photos/2019/08/09/1162374/original.jpg	https://data.globe.gov/system/photos/2019/08/09/1162373/original.jpg	https://data.globe.gov/system/photos/2019/08/09/1162375/original.jpg	https://data.globe.gov/system/photos/2019/08/09/1162377/original.jpg
GV_1896	46.6351	-114.5783	1595		https://www.google.com/maps/search/46.6351,-114.5783	https://data.globe.gov/system/photos/2019/08/09/1162378/original.jpg	https://data.globe.gov/system/photos/2019/08/09/1162380/original.jpg	https://data.globe.gov/system/photos/2019/08/09/1162379/original.jpg	https://data.globe.gov/system/photos/2019/08/09/1162381/original.jpg	https://data.globe.gov/system/photos/2019/08/09/1162383/original.jpg
GV_1897	46.6347	-114.5777	1594.3		https://www.google.com/maps/search/46.6347,-114.5777	https://data.globe.gov/system/photos/2019/08/09/1162385/original.jpg	https://data.globe.gov/system/photos/2019/08/09/1162387/original.jpg	https://data.globe.gov/system/photos/2019/08/09/1162386/original.jpg	https://data.globe.gov/system/photos/2019/08/09/1162388/original.jpg	https://data.globe.gov/system/photos/2019/08/09/1162390/original.jpg
GV_1898	46.5391	-114.6748	1092.5		https://www.google.com/maps/search/46.5391,-114.6748	https://data.globe.gov/system/photos/2019/08/09/1162391/original.jpg	https://data.globe.gov/system/photos/2019/08/09/1162393/original.jpg	https://data.globe.gov/system/photos/2019/08/09/1162392/original.jpg	https://data.globe.gov/system/photos/2019/08/09/1162394/original.jpg	https://data.globe.gov/system/photos/2019/08/09/1162396/original.jpg
GV_1899	46.6333	-114.5749	1596.2		https://www.google.com/maps/search/46.6333,-114.5749	https://data.globe.gov/system/photos/2019/08/09/1162397/original.jpg	https://data.globe.gov/system/photos/2019/08/09/1162399/original.jpg	https://data.globe.gov/system/photos/2019/08/09/1162398/original.jpg	https://data.globe.gov/system/photos/2019/08/09/1162400/original.jpg	https://data.globe.gov/system/photos/2019/08/09/1162402/original.jpg
GV_1900	46.6333	-114.5746	1595.2		https://www.google.com/maps/search/46.6333,-114.5746	https://data.globe.gov/system/photos/2019/08/09/1162403/original.jpg	https://data.globe.gov/system/photos/2019/08/09/1162405/original.jpg	https://data.globe.gov/system/photos/2019/08/09/1162404/original.jpg	https://data.globe.gov/system/photos/2019/08/09/1162406/original.jpg	https://data.globe.gov/system/photos/2019/08/09/1162408/original.jpg
GV_1901	46.5391	-114.6764	1114.1		https://www.google.com/maps/search/46.5391,-114.6764	https://data.globe.gov/system/photos/2019/08/09/1162409/original.jpg	https://data.globe.gov/system/photos/2019/08/09/1162411/original.jpg	https://data.globe.gov/system/photos/2019/08/09/1162410/original.jpg	https://data.globe.gov/system/photos/2019/08/09/1162412/original.jpg	https://data.globe.gov/system/photos/2019/08/09/1162414/original.jpg
GV_1902	46.6358	-114.5803	1595.5		https://www.google.com/maps/search/46.6358,-114.5803	https://data.globe.gov/system/photos/2019/08/09/1162415/original.jpg	https://data.globe.gov/system/photos/2019/08/09/1162417/original.jpg	https://data.globe.gov/system/photos/2019/08/09/1162416/original.jpg	https://data.globe.gov/system/photos/2019/08/09/1162418/original.jpg	https://data.globe.gov/system/photos/2019/08/09/1162420/original.jpg
GV_1903	46.616	-114.5906	1478.6		https://www.google.com/maps/search/46.616,-114.5906	https://data.globe.gov/system/photos/2019/08/09/1162421/original.jpg	https://data.globe.gov/system/photos/2019/08/09/1162423/original.jpg	https://data.globe.gov/system/photos/2019/08/09/1162422/original.jpg	https://data.globe.gov/system/photos/2019/08/09/1162424/original.jpg	https://data.globe.gov/system/photos/2019/08/09/1162426/original.jpg
GV_1904	46.5956	-114.6002	1337.2		https://www.google.com/maps/search/46.5956,-114.6002	https://data.globe.gov/system/photos/2019/08/09/1162427/original.jpg	https://data.globe.gov/system/photos/2019/08/09/1162429/original.jpg	https://data.globe.gov/system/photos/2019/08/09/1162428/original.jpg	https://data.globe.gov/system/photos/2019/08/09/1162430/original.jpg	https://data.globe.gov/system/photos/2019/08/09/1162432/original.jpg
GV_1905	46.5638	-114.6465	1137.5		https://www.google.com/maps/search/46.5638,-114.6465	https://data.globe.gov/system/photos/2019/08/09/1162433/original.jpg	https://data.globe.gov/system/photos/2019/08/09/1162435/original.jpg	https://data.globe.gov/system/photos/2019/08/09/1162434/original.jpg	https://data.globe.gov/system/photos/2019/08/09/1162436/original.jpg	https://data.globe.gov/system/photos/2019/08/09/1162438/original.jpg
GV_1906	46.539	-114.6744	1086.8		https://www.google.com/maps/search/46.539,-114.6744	https://data.globe.gov/system/photos/2019/08/09/1162440/original.jpg	https://data.globe.gov/system/photos/2019/08/09/1162442/original.jpg	https://data.globe.gov/system/photos/2019/08/09/1162441/original.jpg	https://data.globe.gov/system/photos/2019/08/09/1162443/original.jpg	https://data.globe.gov/system/photos/2019/08/09/1162445/original.jpg
GV_1907	46.5384	-114.6746	1112.8		https://www.google.com/maps/search/46.5384,-114.6746	https://data.globe.gov/system/photos/2019/08/09/1162446/original.jpg	https://data.globe.gov/system/photos/2019/08/09/1162448/original.jpg	https://data.globe.gov/system/photos/2019/08/09/1162447/original.jpg	https://data.globe.gov/system/photos/2019/08/09/1162449/original.jpg	https://data.globe.gov/system/photos/2019/08/09/1162451/original.jpg
GV_1908	46.5385	-114.6752	1092.3		https://www.google.com/maps/search/46.5385,-114.6752	https://data.globe.gov/system/photos/2019/08/09/1162452/original.jpg	https://data.globe.gov/system/photos/2019/08/09/1162454/original.jpg	https://data.globe.gov/system/photos/2019/08/09/1162453/original.jpg	https://data.globe.gov/system/photos/2019/08/09/1162455/original.jpg	https://data.globe.gov/system/photos/2019/08/09/1162457/original.jpg
GV_1909	46.5385	-114.6752	1092.3		https://www.google.com/maps/search/46.5385,-114.6752	https://data.globe.gov/system/photos/2019/08/09/1162458/original.jpg	https://data.globe.gov/system/photos/2019/08/09/1162460/original.jpg	https://data.globe.gov/system/photos/2019/08/09/1162459/original.jpg	https://data.globe.gov/system/photos/2019/08/09/1162461/original.jpg	https://data.globe.gov/system/photos/2019/08/09/1162463/original.jpg
GV_1910	46.5102	-114.711	1040		https://www.google.com/maps/search/46.5102,-114.711	https://data.globe.gov/system/photos/2019/08/09/1162464/original.jpg	https://data.globe.gov/system/photos/2019/08/09/1162466/original.jpg	https://data.globe.gov/system/photos/2019/08/09/1162465/original.jpg	https://data.globe.gov/system/photos/2019/08/09/1162467/original.jpg	https://data.globe.gov/system/photos/2019/08/09/1162469/original.jpg
GV_1911	46.5095	-114.7796	1006.2		https://www.google.com/maps/search/46.5095,-114.7796	https://data.globe.gov/system/photos/2019/08/09/1162470/original.jpg	https://data.globe.gov/system/photos/2019/08/09/1162472/original.jpg	https://data.globe.gov/system/photos/2019/08/09/1162471/original.jpg	https://data.globe.gov/system/photos/2019/08/09/1162473/original.jpg	https://data.globe.gov/system/photos/2019/08/09/1162475/original.jpg
GV_1912	46.5045	-114.8252	972.5		https://www.google.com/maps/search/46.5045,-114.8252	https://data.globe.gov/system/photos/2019/08/09/1162476/original.jpg	https://data.globe.gov/system/photos/2019/08/09/1162478/original.jpg	https://data.globe.gov/system/photos/2019/08/09/1162477/original.jpg	https://data.globe.gov/system/photos/2019/08/09/1162479/original.jpg	https://data.globe.gov/system/photos/2019/08/09/1162481/original.jpg
GV_1913	46.4647	-114.9405	916.7		https://www.google.com/maps/search/46.4647,-114.9405	https://data.globe.gov/system/photos/2019/08/09/1162482/original.jpg	https://data.globe.gov/system/photos/2019/08/09/1162484/original.jpg	https://data.globe.gov/system/photos/2019/08/09/1162483/original.jpg	https://data.globe.gov/system/photos/2019/08/09/1162485/original.jpg	https://data.globe.gov/system/photos/2019/08/09/1162487/original.jpg
GV_1914	46.4502	-115.0838	841.2		https://www.google.com/maps/search/46.4502,-115.0838	https://data.globe.gov/system/photos/2019/08/09/1162488/original.jpg	https://data.globe.gov/system/photos/2019/08/09/1162490/original.jpg	https://data.globe.gov/system/photos/2019/08/09/1162489/original.jpg	https://data.globe.gov/system/photos/2019/08/09/1162491/original.jpg	https://data.globe.gov/system/photos/2019/08/09/1162493/original.jpg
GV_1915	46.4248	-115.1565	782		https://www.google.com/maps/search/46.4248,-115.1565	https://data.globe.gov/system/photos/2019/08/09/1162494/original.jpg	https://data.globe.gov/system/photos/2019/08/09/1162496/original.jpg	https://data.globe.gov/system/photos/2019/08/09/1162495/original.jpg	https://data.globe.gov/system/photos/2019/08/09/1162497/original.jpg	https://data.globe.gov/system/photos/2019/08/09/1162499/original.jpg
GV_1916	46.3589	-115.281	661.8		https://www.google.com/maps/search/46.3589,-115.281	https://data.globe.gov/system/photos/2019/08/09/1162500/original.jpg	https://data.globe.gov/system/photos/2019/08/09/1162502/original.jpg	https://data.globe.gov/system/photos/2019/08/09/1162501/original.jpg	https://data.globe.gov/system/photos/2019/08/09/1162503/original.jpg	https://data.globe.gov/system/photos/2019/08/09/1162505/original.jpg
GV_1917	46.3344	-115.3448	605.1		https://www.google.com/maps/search/46.3344,-115.3448	https://data.globe.gov/system/photos/2019/08/09/1162506/original.jpg	https://data.globe.gov/system/photos/2019/08/09/1162508/original.jpg	https://data.globe.gov/system/photos/2019/08/09/1162507/original.jpg	https://data.globe.gov/system/photos/2019/08/09/1162509/original.jpg	https://data.globe.gov/system/photos/2019/08/09/1162511/original.jpg
GV_1918	46.3344	-115.3448	605.1		https://www.google.com/maps/search/46.3344,-115.3448	https://data.globe.gov/system/photos/2019/08/09/1162512/original.jpg	https://data.globe.gov/system/photos/2019/08/09/1162514/original.jpg	https://data.globe.gov/system/photos/2019/08/09/1162513/original.jpg	https://data.globe.gov/system/photos/2019/08/09/1162515/original.jpg	https://data.globe.gov/system/photos/2019/08/09/1162517/original.jpg
GV_1919	46.2671	-115.3939	552		https://www.google.com/maps/search/46.2671,-115.3939	https://data.globe.gov/system/photos/2019/08/09/1162518/original.jpg	https://data.globe.gov/system/photos/2019/08/09/1162520/original.jpg	https://data.globe.gov/system/photos/2019/08/09/1162519/original.jpg	https://data.globe.gov/system/photos/2019/08/09/1162521/original.jpg	https://data.globe.gov/system/photos/2019/08/09/1162523/original.jpg
GV_1920	46.1391	-115.602	441.3		https://www.google.com/maps/search/46.1391,-115.602	https://data.globe.gov/system/photos/2019/08/09/1162524/original.jpg	https://data.globe.gov/system/photos/2019/08/09/1162526/original.jpg	https://data.globe.gov/system/photos/2019/08/09/1162525/original.jpg	https://data.globe.gov/system/photos/2019/08/09/1162527/original.jpg	https://data.globe.gov/system/photos/2019/08/09/1162529/original.jpg
GV_1921	46.1372	-115.6742	430		https://www.google.com/maps/search/46.1372,-115.6742	https://data.globe.gov/system/photos/2019/08/09/1162530/original.jpg	https://data.globe.gov/system/photos/2019/08/09/1162532/original.jpg	https://data.globe.gov/system/photos/2019/08/09/1162531/original.jpg	https://data.globe.gov/system/photos/2019/08/09/1162533/original.jpg	https://data.globe.gov/system/photos/2019/08/09/1162535/original.jpg
GV_1922	46.1357	-115.9544	384.8		https://www.google.com/maps/search/46.1357,-115.9544	https://data.globe.gov/system/photos/2019/08/09/1162536/original.jpg	https://data.globe.gov/system/photos/2019/08/09/1162538/original.jpg	https://data.globe.gov/system/photos/2019/08/09/1162537/original.jpg	https://data.globe.gov/system/photos/2019/08/09/1162539/original.jpg	https://data.globe.gov/system/photos/2019/08/09/1162541/original.jpg
GV_1923	46.1798	-116.0004	370.4		https://www.google.com/maps/search/46.1798,-116.0004	https://data.globe.gov/system/photos/2019/08/09/1162542/original.jpg	https://data.globe.gov/system/photos/2019/08/09/1162544/original.jpg	https://data.globe.gov/system/photos/2019/08/09/1162543/original.jpg	https://data.globe.gov/system/photos/2019/08/09/1162545/original.jpg	https://data.globe.gov/system/photos/2019/08/09/1162547/original.jpg
GV_1924	46.2106	-116.0055	369.1		https://www.google.com/maps/search/46.2106,-116.0055	https://data.globe.gov/system/photos/2019/08/09/1162548/original.jpg	https://data.globe.gov/system/photos/2019/08/09/1162550/original.jpg	https://data.globe.gov/system/photos/2019/08/09/1162549/original.jpg	https://data.globe.gov/system/photos/2019/08/09/1162551/original.jpg	https://data.globe.gov/system/photos/2019/08/09/1162553/original.jpg
GV_1925	46.2106	-116.0062	367.1		https://www.google.com/maps/search/46.2106,-116.0062	https://data.globe.gov/system/photos/2019/08/09/1162554/original.jpg	https://data.globe.gov/system/photos/2019/08/09/1162556/original.jpg	https://data.globe.gov/system/photos/2019/08/09/1162555/original.jpg	https://data.globe.gov/system/photos/2019/08/09/1162557/original.jpg	https://data.globe.gov/system/photos/2019/08/09/1162559/original.jpg
GV_1926	46.2224	-116.0097	366.5		https://www.google.com/maps/search/46.2224,-116.0097	https://data.globe.gov/system/photos/2019/08/09/1162560/original.jpg	https://data.globe.gov/system/photos/2019/08/09/1162562/original.jpg	https://data.globe.gov/system/photos/2019/08/09/1162561/original.jpg	https://data.globe.gov/system/photos/2019/08/09/1162563/original.jpg	https://data.globe.gov/system/photos/2019/08/09/1162565/original.jpg
GV_1927	46.2543	-116.0611	355.3		https://www.google.com/maps/search/46.2543,-116.0611	https://data.globe.gov/system/photos/2019/08/09/1162566/original.jpg	https://data.globe.gov/system/photos/2019/08/09/1162568/original.jpg	https://data.globe.gov/system/photos/2019/08/09/1162567/original.jpg	https://data.globe.gov/system/photos/2019/08/09/1162569/original.jpg	https://data.globe.gov/system/photos/2019/08/09/1162571/original.jpg
GV_1928	46.3236	-116.1404	339.2		https://www.google.com/maps/search/46.3236,-116.1404	https://data.globe.gov/system/photos/2019/08/09/1162572/original.jpg	https://data.globe.gov/system/photos/2019/08/09/1162574/original.jpg	https://data.globe.gov/system/photos/2019/08/09/1162573/original.jpg	https://data.globe.gov/system/photos/2019/08/09/1162575/original.jpg	https://data.globe.gov/system/photos/2019/08/09/1162577/original.jpg
GV_1929	46.501	-116.3296	299.8		https://www.google.com/maps/search/46.501,-116.3296	https://data.globe.gov/system/photos/2019/08/09/1162578/original.jpg	https://data.globe.gov/system/photos/2019/08/09/1162580/original.jpg	https://data.globe.gov/system/photos/2019/08/09/1162579/original.jpg	https://data.globe.gov/system/photos/2019/08/09/1162581/original.jpg	https://data.globe.gov/system/photos/2019/08/09/1162583/original.jpg
GV_1930	46.5012	-116.3295	295.6		https://www.google.com/maps/search/46.5012,-116.3295	https://data.globe.gov/system/photos/2019/08/09/1162584/original.jpg	https://data.globe.gov/system/photos/2019/08/09/1162586/original.jpg	https://data.globe.gov/system/photos/2019/08/09/1162585/original.jpg	https://data.globe.gov/system/photos/2019/08/09/1162587/original.jpg	https://data.globe.gov/system/photos/2019/08/09/1162589/original.jpg
GV_1931	46.4297	-117.034	228.9		https://www.google.com/maps/search/46.4297,-117.034	https://data.globe.gov/system/photos/2019/08/10/1162590/original.jpg	https://data.globe.gov/system/photos/2019/08/10/1162592/original.jpg	https://data.globe.gov/system/photos/2019/08/10/1162591/original.jpg	https://data.globe.gov/system/photos/2019/08/10/1162593/original.jpg	https://data.globe.gov/system/photos/2019/08/10/1162595/original.jpg
GV_1932	46.4361	-117.4253	848.6		https://www.google.com/maps/search/46.4361,-117.4253	https://data.globe.gov/system/photos/2019/08/10/1162596/original.jpg	https://data.globe.gov/system/photos/2019/08/10/1162598/original.jpg	https://data.globe.gov/system/photos/2019/08/10/1162597/original.jpg	https://data.globe.gov/system/photos/2019/08/10/1162599/original.jpg	https://data.globe.gov/system/photos/2019/08/10/1162601/original.jpg
GV_1933	46.4657	-117.4777	692.8		https://www.google.com/maps/search/46.4657,-117.4777	https://data.globe.gov/system/photos/2019/08/10/1162602/original.jpg	https://data.globe.gov/system/photos/2019/08/10/1162604/original.jpg	https://data.globe.gov/system/photos/2019/08/10/1162603/original.jpg	https://data.globe.gov/system/photos/2019/08/10/1162605/original.jpg	https://data.globe.gov/system/photos/2019/08/10/1162607/original.jpg
GV_1934	45.9303	-119.0972	108.4		https://www.google.com/maps/search/45.9303,-119.0972	https://data.globe.gov/system/photos/2019/08/10/1162608/original.jpg	https://data.globe.gov/system/photos/2019/08/10/1162610/original.jpg	https://data.globe.gov/system/photos/2019/08/10/1162609/original.jpg	https://data.globe.gov/system/photos/2019/08/10/1162611/original.jpg	https://data.globe.gov/system/photos/2019/08/10/1162613/original.jpg
GV_1935	44.2682	-79.5637	246.9		https://www.google.com/maps/search/44.2682,-79.5637	https://data.globe.gov/system/photos/2019/08/09/1162837/original.jpg	https://data.globe.gov/system/photos/2019/08/09/1162839/original.jpg	https://data.globe.gov/system/photos/2019/08/09/1162838/original.jpg	https://data.globe.gov/system/photos/2019/08/09/1162840/original.jpg	https://data.globe.gov/system/photos/2019/08/09/1162842/original.jpg
GV_1936	31.8677	-106.5614	1222.5		https://www.google.com/maps/search/31.8677,-106.5614	https://data.globe.gov/system/photos/2019/08/10/1163030/original.jpg	https://data.globe.gov/system/photos/2019/08/10/1163032/original.jpg	https://data.globe.gov/system/photos/2019/08/10/1163031/original.jpg	https://data.globe.gov/system/photos/2019/08/10/1163033/original.jpg	https://data.globe.gov/system/photos/2019/08/10/1163035/original.jpg
GV_1937	46.9388	-100.9825	529.5		https://www.google.com/maps/search/46.9388,-100.9825	https://data.globe.gov/system/photos/2019/08/11/1163707/original.jpg	https://data.globe.gov/system/photos/2019/08/11/1163709/original.jpg	https://data.globe.gov/system/photos/2019/08/11/1163708/original.jpg	https://data.globe.gov/system/photos/2019/08/11/1163710/original.jpg	https://data.globe.gov/system/photos/2019/08/11/1163711/original.jpg
GV_1938	40.8241	-74.2044	87.7		https://www.google.com/maps/search/40.8241,-74.2044	https://data.globe.gov/system/photos/2019/08/11/1164074/original.jpg	https://data.globe.gov/system/photos/2019/08/11/1164076/original.jpg	https://data.globe.gov/system/photos/2019/08/11/1164075/original.jpg	https://data.globe.gov/system/photos/2019/08/11/1164077/original.jpg	https://data.globe.gov/system/photos/2019/08/11/1164079/original.jpg
GV_1939	40.8242	-74.2045	87.2		https://www.google.com/maps/search/40.8242,-74.2045	https://data.globe.gov/system/photos/2019/08/11/1164081/original.jpg	https://data.globe.gov/system/photos/2019/08/11/1164083/original.jpg	https://data.globe.gov/system/photos/2019/08/11/1164082/original.jpg	https://data.globe.gov/system/photos/2019/08/11/1164084/original.jpg	https://data.globe.gov/system/photos/2019/08/11/1164086/original.jpg
GV_1940	53.4284	-0.6224	26		https://www.google.com/maps/search/53.4284,-0.6224	https://data.globe.gov/system/photos/2019/08/10/1164143/original.jpg	https://data.globe.gov/system/photos/2019/08/10/1164145/original.jpg	https://data.globe.gov/system/photos/2019/08/10/1164144/original.jpg	https://data.globe.gov/system/photos/2019/08/10/1164146/original.jpg	https://data.globe.gov/system/photos/2019/08/10/1164148/original.jpg
GV_1941	53.414	-0.6052	37.9		https://www.google.com/maps/search/53.414,-0.6052	https://data.globe.gov/system/photos/2019/08/11/1164155/original.jpg	https://data.globe.gov/system/photos/2019/08/11/1164157/original.jpg	https://data.globe.gov/system/photos/2019/08/11/1164156/original.jpg	https://data.globe.gov/system/photos/2019/08/11/1164158/original.jpg	https://data.globe.gov/system/photos/2019/08/11/1164160/original.jpg
GV_1942	40.2366	-75.4606	62.1		https://www.google.com/maps/search/40.2366,-75.4606	https://data.globe.gov/system/photos/2019/08/11/1164184/original.jpg	https://data.globe.gov/system/photos/2019/08/11/1164186/original.jpg	https://data.globe.gov/system/photos/2019/08/11/1164185/original.jpg	https://data.globe.gov/system/photos/2019/08/11/1164187/original.jpg	https://data.globe.gov/system/photos/2019/08/11/1164189/original.jpg
GV_1943	42.3382	-82.9793	176.2		https://www.google.com/maps/search/42.3382,-82.9793	https://data.globe.gov/system/photos/2019/07/16/1164377/original.jpg	https://data.globe.gov/system/photos/2019/07/16/1164379/original.jpg	https://data.globe.gov/system/photos/2019/07/16/1164378/original.jpg	https://data.globe.gov/system/photos/2019/07/16/1164380/original.jpg	https://data.globe.gov/system/photos/2019/07/16/1164384/original.jpg
GV_1944	53.4284	-0.6223	26		https://www.google.com/maps/search/53.4284,-0.6223	https://data.globe.gov/system/photos/2019/08/12/1164972/original.jpg	https://data.globe.gov/system/photos/2019/08/12/1164974/original.jpg	https://data.globe.gov/system/photos/2019/08/12/1164973/original.jpg	https://data.globe.gov/system/photos/2019/08/12/1164975/original.jpg	https://data.globe.gov/system/photos/2019/08/12/1164977/original.jpg
GV_1945	43.2889	-123.3307	169.7		https://www.google.com/maps/search/43.2889,-123.3307	https://data.globe.gov/system/photos/2019/08/12/1165126/original.jpg	https://data.globe.gov/system/photos/2019/08/12/1165128/original.jpg	https://data.globe.gov/system/photos/2019/08/12/1165127/original.jpg	https://data.globe.gov/system/photos/2019/08/12/1165129/original.jpg	https://data.globe.gov/system/photos/2019/08/12/1165131/original.jpg
GV_1946	44.7383	-123.0528	61.2		https://www.google.com/maps/search/44.7383,-123.0528	https://data.globe.gov/system/photos/2019/08/12/1165138/original.jpg	https://data.globe.gov/system/photos/2019/08/12/1165140/original.jpg	https://data.globe.gov/system/photos/2019/08/12/1165139/original.jpg	https://data.globe.gov/system/photos/2019/08/12/1165141/original.jpg	https://data.globe.gov/system/photos/2019/08/12/1165143/original.jpg
GV_1947	44.5657	-123.279	77.9		https://www.google.com/maps/search/44.5657,-123.279	https://data.globe.gov/system/photos/2019/08/12/1165150/original.jpg	https://data.globe.gov/system/photos/2019/08/12/1165152/original.jpg	https://data.globe.gov/system/photos/2019/08/12/1165151/original.jpg	https://data.globe.gov/system/photos/2019/08/12/1165153/original.jpg	https://data.globe.gov/system/photos/2019/08/12/1165156/original.jpg
GV_1948	-24.5725	133.148	454.9		https://www.google.com/maps/search/-24.5725,133.148	https://data.globe.gov/system/photos/2019/08/09/1165267/original.jpg	https://data.globe.gov/system/photos/2019/08/09/1165269/original.jpg	https://data.globe.gov/system/photos/2019/08/09/1165268/original.jpg	https://data.globe.gov/system/photos/2019/08/09/1165270/original.jpg	https://data.globe.gov/system/photos/2019/08/09/1165272/original.jpg
GV_1949	44.5651	-123.2856	74.7		https://www.google.com/maps/search/44.5651,-123.2856	https://data.globe.gov/system/photos/2019/08/13/1165330/original.jpg	https://data.globe.gov/system/photos/2019/08/13/1165332/original.jpg	https://data.globe.gov/system/photos/2019/08/13/1165331/original.jpg	https://data.globe.gov/system/photos/2019/08/13/1165333/original.jpg	https://data.globe.gov/system/photos/2019/08/13/1165335/original.jpg
GV_1950	44.5646	-123.2857	73.8		https://www.google.com/maps/search/44.5646,-123.2857	https://data.globe.gov/system/photos/2019/08/13/1165336/original.jpg	https://data.globe.gov/system/photos/2019/08/13/1165338/original.jpg	https://data.globe.gov/system/photos/2019/08/13/1165337/original.jpg	https://data.globe.gov/system/photos/2019/08/13/1165339/original.jpg	https://data.globe.gov/system/photos/2019/08/13/1165341/original.jpg
GV_1951	44.5642	-123.2848	73.1		https://www.google.com/maps/search/44.5642,-123.2848	https://data.globe.gov/system/photos/2019/08/13/1165342/original.jpg	https://data.globe.gov/system/photos/2019/08/13/1165344/original.jpg	https://data.globe.gov/system/photos/2019/08/13/1165343/original.jpg	https://data.globe.gov/system/photos/2019/08/13/1165345/original.jpg	https://data.globe.gov/system/photos/2019/08/13/1165347/original.jpg
GV_1952	44.5608	-123.1369	70.5		https://www.google.com/maps/search/44.5608,-123.1369	https://data.globe.gov/system/photos/2019/08/13/1165348/original.jpg	https://data.globe.gov/system/photos/2019/08/13/1165350/original.jpg	https://data.globe.gov/system/photos/2019/08/13/1165349/original.jpg	https://data.globe.gov/system/photos/2019/08/13/1165351/original.jpg	https://data.globe.gov/system/photos/2019/08/13/1165353/original.jpg
GV_1953	-37.9441	144.9977	0		https://www.google.com/maps/search/-37.9441,144.9977	https://data.globe.gov/system/photos/2019/08/13/1165447/original.jpg	https://data.globe.gov/system/photos/2019/08/13/1165449/original.jpg	https://data.globe.gov/system/photos/2019/08/13/1165448/original.jpg	https://data.globe.gov/system/photos/2019/08/13/1165450/original.jpg	https://data.globe.gov/system/photos/2019/08/13/1165452/original.jpg
GV_1954	45.0535	-92.8302	269.6		https://www.google.com/maps/search/45.0535,-92.8302	https://data.globe.gov/system/photos/2019/08/11/1165944/original.jpg	https://data.globe.gov/system/photos/2019/08/11/1165947/original.jpg	https://data.globe.gov/system/photos/2019/08/11/1165945/original.jpg	https://data.globe.gov/system/photos/2019/08/11/1165948/original.jpg	https://data.globe.gov/system/photos/2019/08/11/1165950/original.jpg
GV_1955	45.0543	-92.8293	268.8		https://www.google.com/maps/search/45.0543,-92.8293	https://data.globe.gov/system/photos/2019/08/11/1165952/original.jpg	https://data.globe.gov/system/photos/2019/08/11/1165954/original.jpg	https://data.globe.gov/system/photos/2019/08/11/1165953/original.jpg	https://data.globe.gov/system/photos/2019/08/11/1165955/original.jpg	https://data.globe.gov/system/photos/2019/08/11/1165958/original.jpg
GV_1956	45.0538	-92.8288	267.8		https://www.google.com/maps/search/45.0538,-92.8288	https://data.globe.gov/system/photos/2019/08/11/1165959/original.jpg	https://data.globe.gov/system/photos/2019/08/11/1165961/original.jpg	https://data.globe.gov/system/photos/2019/08/11/1165960/original.jpg	https://data.globe.gov/system/photos/2019/08/11/1165963/original.jpg	https://data.globe.gov/system/photos/2019/08/11/1165965/original.jpg
GV_1957	42.1357	-83.2652	181.7		https://www.google.com/maps/search/42.1357,-83.2652	https://data.globe.gov/system/photos/2019/08/13/1166244/original.jpg	https://data.globe.gov/system/photos/2019/08/13/1166248/original.jpg	https://data.globe.gov/system/photos/2019/08/13/1166246/original.jpg	https://data.globe.gov/system/photos/2019/08/13/1166250/original.jpg	https://data.globe.gov/system/photos/2019/08/13/1166254/original.jpg
GV_1958	42.1353	-83.2641	184.1		https://www.google.com/maps/search/42.1353,-83.2641	https://data.globe.gov/system/photos/2019/08/13/1166245/original.jpg	https://data.globe.gov/system/photos/2019/08/13/1166249/original.jpg	https://data.globe.gov/system/photos/2019/08/13/1166247/original.jpg	https://data.globe.gov/system/photos/2019/08/13/1166252/original.jpg	https://data.globe.gov/system/photos/2019/08/13/1166255/original.jpg
GV_1959	-25.3509	130.7868	568.4		https://www.google.com/maps/search/-25.3509,130.7868	https://data.globe.gov/system/photos/2019/08/13/1166404/original.jpg	https://data.globe.gov/system/photos/2019/08/13/1166406/original.jpg	https://data.globe.gov/system/photos/2019/08/13/1166405/original.jpg	https://data.globe.gov/system/photos/2019/08/13/1166407/original.jpg	https://data.globe.gov/system/photos/2019/08/13/1166409/original.jpg
GV_1960	38.1865	-85.7411	144.9		https://www.google.com/maps/search/38.1865,-85.7411	https://data.globe.gov/system/photos/2019/08/13/1166428/original.jpg	https://data.globe.gov/system/photos/2019/08/13/1166430/original.jpg	https://data.globe.gov/system/photos/2019/08/13/1166429/original.jpg	https://data.globe.gov/system/photos/2019/08/13/1166431/original.jpg	https://data.globe.gov/system/photos/2019/08/13/1166433/original.jpg
GV_1961	-37.9432	144.9989	0		https://www.google.com/maps/search/-37.9432,144.9989	https://data.globe.gov/system/photos/2019/08/14/1166642/original.jpg	https://data.globe.gov/system/photos/2019/08/14/1166644/original.jpg	https://data.globe.gov/system/photos/2019/08/14/1166643/original.jpg	https://data.globe.gov/system/photos/2019/08/14/1166645/original.jpg	https://data.globe.gov/system/photos/2019/08/14/1166647/original.jpg
GV_1962	53.4283	-0.6229	25.9		https://www.google.com/maps/search/53.4283,-0.6229	https://data.globe.gov/system/photos/2019/08/13/1166733/original.jpg	https://data.globe.gov/system/photos/2019/08/13/1166735/original.jpg	https://data.globe.gov/system/photos/2019/08/13/1166734/original.jpg	https://data.globe.gov/system/photos/2019/08/13/1166736/original.jpg	https://data.globe.gov/system/photos/2019/08/13/1166738/original.jpg
GV_1963	24.4358	118.3143	21.7		https://www.google.com/maps/search/24.4358,118.3143	https://data.globe.gov/system/photos/2019/08/14/1166766/original.jpg	https://data.globe.gov/system/photos/2019/08/14/1166768/original.jpg	https://data.globe.gov/system/photos/2019/08/14/1166767/original.jpg	https://data.globe.gov/system/photos/2019/08/14/1166769/original.jpg	https://data.globe.gov/system/photos/2019/08/14/1166771/original.jpg
GV_1964	-24.8628	129.0576	602.6		https://www.google.com/maps/search/-24.8628,129.0576	https://data.globe.gov/system/photos/2019/08/14/1166772/original.jpg	https://data.globe.gov/system/photos/2019/08/14/1166774/original.jpg	https://data.globe.gov/system/photos/2019/08/14/1166773/original.jpg	https://data.globe.gov/system/photos/2019/08/14/1166775/original.jpg	https://data.globe.gov/system/photos/2019/08/14/1166777/original.jpg
GV_1965	-25.1601	128.5265	602.9		https://www.google.com/maps/search/-25.1601,128.5265	https://data.globe.gov/system/photos/2019/08/14/1166778/original.jpg	https://data.globe.gov/system/photos/2019/08/14/1166780/original.jpg	https://data.globe.gov/system/photos/2019/08/14/1166779/original.jpg	https://data.globe.gov/system/photos/2019/08/14/1166781/original.jpg	https://data.globe.gov/system/photos/2019/08/14/1166783/original.jpg
GV_1966	44.0213	-90.4939	296.5		https://www.google.com/maps/search/44.0213,-90.4939	https://data.globe.gov/system/photos/2019/08/11/1166835/original.jpg	https://data.globe.gov/system/photos/2019/08/11/1166837/original.jpg	https://data.globe.gov/system/photos/2019/08/11/1166836/original.jpg	https://data.globe.gov/system/photos/2019/08/11/1166838/original.jpg	https://data.globe.gov/system/photos/2019/08/11/1166840/original.jpg
GV_1967	38.0455	-122.1611	2.8		https://www.google.com/maps/search/38.0455,-122.1611	https://data.globe.gov/system/photos/2019/08/14/1166868/original.jpg	https://data.globe.gov/system/photos/2019/08/14/1166870/original.jpg	https://data.globe.gov/system/photos/2019/08/14/1166869/original.jpg	https://data.globe.gov/system/photos/2019/08/14/1166871/original.jpg	https://data.globe.gov/system/photos/2019/08/14/1166873/original.jpg
GV_1968	38.256	-85.75	140.1		https://www.google.com/maps/search/38.256,-85.75	https://data.globe.gov/system/photos/2019/08/14/1166927/original.jpg	https://data.globe.gov/system/photos/2019/08/14/1166929/original.jpg	https://data.globe.gov/system/photos/2019/08/14/1166928/original.jpg	https://data.globe.gov/system/photos/2019/08/14/1166930/original.jpg	https://data.globe.gov/system/photos/2019/08/14/1166932/original.jpg
GV_1969	38.257	-85.7509	137.3		https://www.google.com/maps/search/38.257,-85.7509	https://data.globe.gov/system/photos/2019/08/14/1166933/original.jpg	https://data.globe.gov/system/photos/2019/08/14/1166935/original.jpg	https://data.globe.gov/system/photos/2019/08/14/1166934/original.jpg	https://data.globe.gov/system/photos/2019/08/14/1166936/original.jpg	https://data.globe.gov/system/photos/2019/08/14/1166938/original.jpg
GV_1970	38.2577	-85.7514	134.5		https://www.google.com/maps/search/38.2577,-85.7514	https://data.globe.gov/system/photos/2019/08/14/1166939/original.jpg	https://data.globe.gov/system/photos/2019/08/14/1166941/original.jpg	https://data.globe.gov/system/photos/2019/08/14/1166940/original.jpg	https://data.globe.gov/system/photos/2019/08/14/1166942/original.jpg	https://data.globe.gov/system/photos/2019/08/14/1166944/original.jpg
GV_1971	38.2576	-85.753	134.8		https://www.google.com/maps/search/38.2576,-85.753	https://data.globe.gov/system/photos/2019/08/14/1166945/original.jpg	https://data.globe.gov/system/photos/2019/08/14/1166947/original.jpg	https://data.globe.gov/system/photos/2019/08/14/1166946/original.jpg	https://data.globe.gov/system/photos/2019/08/14/1166948/original.jpg	https://data.globe.gov/system/photos/2019/08/14/1166950/original.jpg
GV_1972	38.2582	-85.7532	134.2		https://www.google.com/maps/search/38.2582,-85.7532	https://data.globe.gov/system/photos/2019/08/14/1166951/original.jpg	https://data.globe.gov/system/photos/2019/08/14/1166953/original.jpg	https://data.globe.gov/system/photos/2019/08/14/1166952/original.jpg	https://data.globe.gov/system/photos/2019/08/14/1166954/original.jpg	https://data.globe.gov/system/photos/2019/08/14/1166956/original.jpg
GV_1973	38.2596	-85.749	133.4		https://www.google.com/maps/search/38.2596,-85.749	https://data.globe.gov/system/photos/2019/08/14/1166957/original.jpg	https://data.globe.gov/system/photos/2019/08/14/1166959/original.jpg	https://data.globe.gov/system/photos/2019/08/14/1166958/original.jpg	https://data.globe.gov/system/photos/2019/08/14/1166960/original.jpg	https://data.globe.gov/system/photos/2019/08/14/1166962/original.jpg

GV_1974	38.2595	-85.7522	128.9		https://www.google.com/maps/search/38.2595,-85.7522	https://data.globe.gov/system/photos/2019/08/14/1166963/original.jpg	https://data.globe.gov/system/photos/2019/08/14/1166965/original.jpg	https://data.globe.gov/system/photos/2019/08/14/1166964/original.jpg	https://data.globe.gov/system/photos/2019/08/14/1166966/original.jpg	https://data.globe.gov/system/photos/2019/08/14/1166968/original.jpg
GV_1975	38.2596	-85.7517	129		https://www.google.com/maps/search/38.2596,-85.7517	https://data.globe.gov/system/photos/2019/08/14/1166969/original.jpg	https://data.globe.gov/system/photos/2019/08/14/1166971/original.jpg	https://data.globe.gov/system/photos/2019/08/14/1166970/original.jpg	https://data.globe.gov/system/photos/2019/08/14/1166972/original.jpg	https://data.globe.gov/system/photos/2019/08/14/1166974/original.jpg
GV_1976	38.2598	-85.7506	129.2		https://www.google.com/maps/search/38.2598,-85.7506	https://data.globe.gov/system/photos/2019/08/14/1166975/original.jpg	https://data.globe.gov/system/photos/2019/08/14/1166977/original.jpg	https://data.globe.gov/system/photos/2019/08/14/1166976/original.jpg	https://data.globe.gov/system/photos/2019/08/14/1166978/original.jpg	https://data.globe.gov/system/photos/2019/08/14/1166980/original.jpg
GV_1977	38.2601	-85.7497	129.2		https://www.google.com/maps/search/38.2601,-85.7497	https://data.globe.gov/system/photos/2019/08/14/1166987/original.jpg	https://data.globe.gov/system/photos/2019/08/14/1166989/original.jpg	https://data.globe.gov/system/photos/2019/08/14/1166988/original.jpg	https://data.globe.gov/system/photos/2019/08/14/1166990/original.jpg	https://data.globe.gov/system/photos/2019/08/14/1166992/original.jpg
GV_1978	38.2604	-85.7488	133.4		https://www.google.com/maps/search/38.2604,-85.7488	https://data.globe.gov/system/photos/2019/08/14/1166993/original.jpg	https://data.globe.gov/system/photos/2019/08/14/1166995/original.jpg	https://data.globe.gov/system/photos/2019/08/14/1166994/original.jpg	https://data.globe.gov/system/photos/2019/08/14/1166996/original.jpg	https://data.globe.gov/system/photos/2019/08/14/1166998/original.jpg
GV_1979	38.2606	-85.7478	130.1		https://www.google.com/maps/search/38.2606,-85.7478	https://data.globe.gov/system/photos/2019/08/14/1166999/original.jpg	https://data.globe.gov/system/photos/2019/08/14/1167001/original.jpg	https://data.globe.gov/system/photos/2019/08/14/1167000/original.jpg	https://data.globe.gov/system/photos/2019/08/14/1167002/original.jpg	https://data.globe.gov/system/photos/2019/08/14/1167004/original.jpg
GV_1980	38.2588	-85.7478	145		https://www.google.com/maps/search/38.2588,-85.7478	https://data.globe.gov/system/photos/2019/08/14/1167005/original.jpg	https://data.globe.gov/system/photos/2019/08/14/1167007/original.jpg	https://data.globe.gov/system/photos/2019/08/14/1167006/original.jpg	https://data.globe.gov/system/photos/2019/08/14/1167008/original.jpg	https://data.globe.gov/system/photos/2019/08/14/1167010/original.jpg
GV_1981	38.2584	-85.748	138.6		https://www.google.com/maps/search/38.2584,-85.748	https://data.globe.gov/system/photos/2019/08/14/1167011/original.jpg	https://data.globe.gov/system/photos/2019/08/14/1167013/original.jpg	https://data.globe.gov/system/photos/2019/08/14/1167012/original.jpg	https://data.globe.gov/system/photos/2019/08/14/1167014/original.jpg	https://data.globe.gov/system/photos/2019/08/14/1167016/original.jpg
GV_1982	38.2577	-85.7492	136.4		https://www.google.com/maps/search/38.2577,-85.7492	https://data.globe.gov/system/photos/2019/08/14/1167017/original.jpg	https://data.globe.gov/system/photos/2019/08/14/1167019/original.jpg	https://data.globe.gov/system/photos/2019/08/14/1167018/original.jpg	https://data.globe.gov/system/photos/2019/08/14/1167020/original.jpg	https://data.globe.gov/system/photos/2019/08/14/1167022/original.jpg
GV_1983	38.2586	-85.7555	140.3		https://www.google.com/maps/search/38.2586,-85.7555	https://data.globe.gov/system/photos/2019/08/14/1167023/original.jpg	https://data.globe.gov/system/photos/2019/08/14/1167025/original.jpg	https://data.globe.gov/system/photos/2019/08/14/1167024/original.jpg	https://data.globe.gov/system/photos/2019/08/14/1167026/original.jpg	https://data.globe.gov/system/photos/2019/08/14/1167028/original.jpg
GV_1984	38.2585	-85.7537	133.9		https://www.google.com/maps/search/38.2585,-85.7537	https://data.globe.gov/system/photos/2019/08/14/1167029/original.jpg	https://data.globe.gov/system/photos/2019/08/14/1167031/original.jpg	https://data.globe.gov/system/photos/2019/08/14/1167030/original.jpg	https://data.globe.gov/system/photos/2019/08/14/1167032/original.jpg	https://data.globe.gov/system/photos/2019/08/14/1167034/original.jpg
GV_1985	38.2591	-85.7563	129.7		https://www.google.com/maps/search/38.2591,-85.7563	https://data.globe.gov/system/photos/2019/08/14/1167035/original.jpg	https://data.globe.gov/system/photos/2019/08/14/1167037/original.jpg	https://data.globe.gov/system/photos/2019/08/14/1167036/original.jpg	https://data.globe.gov/system/photos/2019/08/14/1167038/original.jpg	https://data.globe.gov/system/photos/2019/08/14/1167040/original.jpg
GV_1986	38.259	-85.7596	129.8		https://www.google.com/maps/search/38.259,-85.7596	https://data.globe.gov/system/photos/2019/08/14/1167041/original.jpg	https://data.globe.gov/system/photos/2019/08/14/1167043/original.jpg	https://data.globe.gov/system/photos/2019/08/14/1167042/original.jpg	https://data.globe.gov/system/photos/2019/08/14/1167044/original.jpg	https://data.globe.gov/system/photos/2019/08/14/1167046/original.jpg
GV_1987	38.259	-85.7583	130.9		https://www.google.com/maps/search/38.259,-85.7583	https://data.globe.gov/system/photos/2019/08/14/1167047/original.jpg	https://data.globe.gov/system/photos/2019/08/14/1167049/original.jpg	https://data.globe.gov/system/photos/2019/08/14/1167048/original.jpg	https://data.globe.gov/system/photos/2019/08/14/1167050/original.jpg	https://data.globe.gov/system/photos/2019/08/14/1167052/original.jpg
GV_1988	38.2583	-85.7598	133.6		https://www.google.com/maps/search/38.2583,-85.7598	https://data.globe.gov/system/photos/2019/08/14/1167053/original.jpg	https://data.globe.gov/system/photos/2019/08/14/1167055/original.jpg	https://data.globe.gov/system/photos/2019/08/14/1167054/original.jpg	https://data.globe.gov/system/photos/2019/08/14/1167056/original.jpg	https://data.globe.gov/system/photos/2019/08/14/1167058/original.jpg
GV_1989	38.2563	-85.7579	140.7		https://www.google.com/maps/search/38.2563,-85.7579	https://data.globe.gov/system/photos/2019/08/14/1167059/original.jpg	https://data.globe.gov/system/photos/2019/08/14/1167061/original.jpg	https://data.globe.gov/system/photos/2019/08/14/1167060/original.jpg	https://data.globe.gov/system/photos/2019/08/14/1167062/original.jpg	https://data.globe.gov/system/photos/2019/08/14/1167064/original.jpg
GV_1990	38.2569	-85.7587	140.4		https://www.google.com/maps/search/38.2569,-85.7587	https://data.globe.gov/system/photos/2019/08/14/1167065/original.jpg	https://data.globe.gov/system/photos/2019/08/14/1167067/original.jpg	https://data.globe.gov/system/photos/2019/08/14/1167066/original.jpg	https://data.globe.gov/system/photos/2019/08/14/1167068/original.jpg	https://data.globe.gov/system/photos/2019/08/14/1167070/original.jpg
GV_1991	38.2574	-85.7583	137.4		https://www.google.com/maps/search/38.2574,-85.7583	https://data.globe.gov/system/photos/2019/08/14/1167071/original.jpg	https://data.globe.gov/system/photos/2019/08/14/1167073/original.jpg	https://data.globe.gov/system/photos/2019/08/14/1167072/original.jpg	https://data.globe.gov/system/photos/2019/08/14/1167074/original.jpg	https://data.globe.gov/system/photos/2019/08/14/1167076/original.jpg
GV_1992	38.2581	-85.758	138.1		https://www.google.com/maps/search/38.2581,-85.758	https://data.globe.gov/system/photos/2019/08/14/1167077/original.jpg	https://data.globe.gov/system/photos/2019/08/14/1167079/original.jpg	https://data.globe.gov/system/photos/2019/08/14/1167078/original.jpg	https://data.globe.gov/system/photos/2019/08/14/1167080/original.jpg	https://data.globe.gov/system/photos/2019/08/14/1167082/original.jpg
GV_1993	38.2589	-85.7578	140		https://www.google.com/maps/search/38.2589,-85.7578	https://data.globe.gov/system/photos/2019/08/14/1167083/original.jpg	https://data.globe.gov/system/photos/2019/08/14/1167085/original.jpg	https://data.globe.gov/system/photos/2019/08/14/1167084/original.jpg	https://data.globe.gov/system/photos/2019/08/14/1167086/original.jpg	https://data.globe.gov/system/photos/2019/08/14/1167088/original.jpg
GV_1994	38.2582	-85.7575	140.1		https://www.google.com/maps/search/38.2582,-85.7575	https://data.globe.gov/system/photos/2019/08/14/1167089/original.jpg	https://data.globe.gov/system/photos/2019/08/14/1167091/original.jpg	https://data.globe.gov/system/photos/2019/08/14/1167090/original.jpg	https://data.globe.gov/system/photos/2019/08/14/1167092/original.jpg	https://data.globe.gov/system/photos/2019/08/14/1167094/original.jpg
GV_1995	38.2585	-85.7576	145.5		https://www.google.com/maps/search/38.2585,-85.7576	https://data.globe.gov/system/photos/2019/08/14/1167095/original.jpg	https://data.globe.gov/system/photos/2019/08/14/1167097/original.jpg	https://data.globe.gov/system/photos/2019/08/14/1167096/original.jpg	https://data.globe.gov/system/photos/2019/08/14/1167098/original.jpg	https://data.globe.gov/system/photos/2019/08/14/1167100/original.jpg
GV_1996	38.2532	-85.7563	140.8		https://www.google.com/maps/search/38.2532,-85.7563	https://data.globe.gov/system/photos/2019/08/14/1167101/original.jpg	https://data.globe.gov/system/photos/2019/08/14/1167103/original.jpg	https://data.globe.gov/system/photos/2019/08/14/1167102/original.jpg	https://data.globe.gov/system/photos/2019/08/14/1167104/original.jpg	https://data.globe.gov/system/photos/2019/08/14/1167106/original.jpg
GV_1997	38.2556	-85.7568	141.6		https://www.google.com/maps/search/38.2556,-85.7568	https://data.globe.gov/system/photos/2019/08/14/1167119/original.jpg	https://data.globe.gov/system/photos/2019/08/14/1167121/original.jpg	https://data.globe.gov/system/photos/2019/08/14/1167120/original.jpg	https://data.globe.gov/system/photos/2019/08/14/1167122/original.jpg	https://data.globe.gov/system/photos/2019/08/14/1167124/original.jpg
GV_1998	38.2552	-85.7569	141.5		https://www.google.com/maps/search/38.2552,-85.7569	https://data.globe.gov/system/photos/2019/08/14/1167125/original.jpg	https://data.globe.gov/system/photos/2019/08/14/1167127/original.jpg	https://data.globe.gov/system/photos/2019/08/14/1167126/original.jpg	https://data.globe.gov/system/photos/2019/08/14/1167128/original.jpg	https://data.globe.gov/system/photos/2019/08/14/1167130/original.jpg
GV_1999	38.2533	-85.7546	141.3		https://www.google.com/maps/search/38.2533,-85.7546	https://data.globe.gov/system/photos/2019/08/14/1167131/original.jpg	https://data.globe.gov/system/photos/2019/08/14/1167133/original.jpg	https://data.globe.gov/system/photos/2019/08/14/1167132/original.jpg	https://data.globe.gov/system/photos/2019/08/14/1167134/original.jpg	https://data.globe.gov/system/photos/2019/08/14/1167136/original.jpg
GV_2000	38.2534	-85.7536	140.3		https://www.google.com/maps/search/38.2534,-85.7536	https://data.globe.gov/system/photos/2019/08/14/1167137/original.jpg	https://data.globe.gov/system/photos/2019/08/14/1167139/original.jpg	https://data.globe.gov/system/photos/2019/08/14/1167138/original.jpg	https://data.globe.gov/system/photos/2019/08/14/1167140/original.jpg	https://data.globe.gov/system/photos/2019/08/14/1167142/original.jpg
GV_2001	38.2549	-85.7536	141.6		https://www.google.com/maps/search/38.2549,-85.7536	https://data.globe.gov/system/photos/2019/08/14/1167589/original.jpg	https://data.globe.gov/system/photos/2019/08/14/1167591/original.jpg	https://data.globe.gov/system/photos/2019/08/14/1167590/original.jpg	https://data.globe.gov/system/photos/2019/08/14/1167592/original.jpg	https://data.globe.gov/system/photos/2019/08/14/1167594/original.jpg
GV_2002	38.2536	-85.7546	141		https://www.google.com/maps/search/38.2536,-85.7546	https://data.globe.gov/system/photos/2019/08/14/1167595/original.jpg	https://data.globe.gov/system/photos/2019/08/14/1167597/original.jpg	https://data.globe.gov/system/photos/2019/08/14/1167596/original.jpg	https://data.globe.gov/system/photos/2019/08/14/1167598/original.jpg	https://data.globe.gov/system/photos/2019/08/14/1167600/original.jpg
GV_2003	38.2547	-85.7553	141.1		https://www.google.com/maps/search/38.2547,-85.7553	https://data.globe.gov/system/photos/2019/08/14/1167601/original.jpg	https://data.globe.gov/system/photos/2019/08/14/1167603/original.jpg	https://data.globe.gov/system/photos/2019/08/14/1167602/original.jpg	https://data.globe.gov/system/photos/2019/08/14/1167604/original.jpg	https://data.globe.gov/system/photos/2019/08/14/1167606/original.jpg
GV_2004	38.2553	-85.7562	141.6		https://www.google.com/maps/search/38.2553,-85.7562	https://data.globe.gov/system/photos/2019/08/14/1167607/original.jpg	https://data.globe.gov/system/photos/2019/08/14/1167609/original.jpg	https://data.globe.gov/system/photos/2019/08/14/1167608/original.jpg	https://data.globe.gov/system/photos/2019/08/14/1167610/original.jpg	https://data.globe.gov/system/photos/2019/08/14/1167612/original.jpg
GV_2005	41.7818	-87.9801	225.6		https://www.google.com/maps/search/41.7818,-87.9801	https://data.globe.gov/system/photos/2019/08/13/1167811/original.jpg	https://data.globe.gov/system/photos/2019/08/13/1167813/original.jpg	https://data.globe.gov/system/photos/2019/08/13/1167812/original.jpg	https://data.globe.gov/system/photos/2019/08/13/1167814/original.jpg	https://data.globe.gov/system/photos/2019/08/13/1167816/original.jpg
GV_2006	46.721	-113.5732	1102.1		https://www.google.com/maps/search/46.721,-113.5732	https://data.globe.gov/system/photos/2019/08/08/1167819/original.jpg	https://data.globe.gov/system/photos/2019/08/08/1167821/original.jpg	https://data.globe.gov/system/photos/2019/08/08/1167820/original.jpg	https://data.globe.gov/system/photos/2019/08/08/1167822/original.jpg	https://data.globe.gov/system/photos/2019/08/08/1167824/original.jpg
GV_2007	46.7216	-113.573	1101.7		https://www.google.com/maps/search/46.7216,-113.573	https://data.globe.gov/system/photos/2019/08/08/1167825/original.jpg	https://data.globe.gov/system/photos/2019/08/08/1167827/original.jpg	https://data.globe.gov/system/photos/2019/08/08/1167826/original.jpg	https://data.globe.gov/system/photos/2019/08/08/1167828/original.jpg	https://data.globe.gov/system/photos/2019/08/08/1167830/original.jpg
GV_2008	47.1268	-104.6913	643.1		https://www.google.com/maps/search/47.1268,-104.6913	https://data.globe.gov/system/photos/2019/08/10/1167836/original.jpg	https://data.globe.gov/system/photos/2019/08/10/1167838/original.jpg	https://data.globe.gov/system/photos/2019/08/10/1167837/original.jpg	https://data.globe.gov/system/photos/2019/08/10/1167839/original.jpg	https://data.globe.gov/system/photos/2019/08/10/1167841/original.jpg
GV_2009	47.0621	-104.6686	774.5		https://www.google.com/maps/search/47.0621,-104.6686	https://data.globe.gov/system/photos/2019/08/10/1167842/original.jpg	https://data.globe.gov/system/photos/2019/08/10/1167844/original.jpg	https://data.globe.gov/system/photos/2019/08/10/1167843/original.jpg	https://data.globe.gov/system/photos/2019/08/10/1167845/original.jpg	https://data.globe.gov/system/photos/2019/08/10/1167847/original.jpg
GV_2010	24.4364	118.3688	17.1		https://www.google.com/maps/search/24.4364,118.3688	https://data.globe.gov/system/photos/2019/08/16/1168690/original.jpg	https://data.globe.gov/system/photos/2019/08/16/1168692/original.jpg	https://data.globe.gov/system/photos/2019/08/16/1168691/original.jpg	https://data.globe.gov/system/photos/2019/08/16/1168693/original.jpg	https://data.globe.gov/system/photos/2019/08/16/1168695/original.jpg
GV_2011	-34.3962	138.9086	277.8		https://www.google.com/maps/search/-34.3962,138.9086	https://data.globe.gov/system/photos/2019/08/16/1168882/original.jpg	https://data.globe.gov/system/photos/2019/08/16/1168884/original.jpg	https://data.globe.gov/system/photos/2019/08/16/1168883/original.jpg	https://data.globe.gov/system/photos/2019/08/16/1168885/original.jpg	https://data.globe.gov/system/photos/2019/08/16/1168887/original.jpg
GV_2012	38.2557	-85.7535	140.9		https://www.google.com/maps/search/38.2557,-85.7535	https://data.globe.gov/system/photos/2019/08/15/1169008/original.jpg	https://data.globe.gov/system/photos/2019/08/15/1169010/original.jpg	https://data.globe.gov/system/photos/2019/08/15/1169009/original.jpg	https://data.globe.gov/system/photos/2019/08/15/1169011/original.jpg	https://data.globe.gov/system/photos/2019/08/15/1169013/original.jpg
GV_2013	38.2566	-85.7536	130.4		https://www.google.com/maps/search/38.2566,-85.7536	https://data.globe.gov/system/photos/2019/08/15/1169014/original.jpg	https://data.globe.gov/system/photos/2019/08/15/1169016/original.jpg	https://data.globe.gov/system/photos/2019/08/15/1169015/original.jpg	https://data.globe.gov/system/photos/2019/08/15/1169017/original.jpg	https://data.globe.gov/system/photos/2019/08/15/1169019/original.jpg
GV_2014	38.2577	-85.7532	133.6		https://www.google.com/maps/search/38.2577,-85.7532	https://data.globe.gov/system/photos/2019/08/15/1169020/original.jpg	https://data.globe.gov/system/photos/2019/08/15/1169022/original.jpg	https://data.globe.gov/system/photos/2019/08/15/1169021/original.jpg	https://data.globe.gov/system/photos/2019/08/15/1169023/original.jpg	https://data.globe.gov/system/photos/2019/08/15/1169025/original.jpg
GV_2015	38.2582	-85.7532	134.2		https://www.google.com/maps/search/38.2582,-85.7532	https://data.globe.gov/system/photos/2019/08/15/1169026/original.jpg	https://data.globe.gov/system/photos/2019/08/15/1169028/original.jpg	https://data.globe.gov/system/photos/2019/08/15/1169027/original.jpg	https://data.globe.gov/system/photos/2019/08/15/1169029/original.jpg	https://data.globe.gov/system/photos/2019/08/15/1169031/original.jpg
GV_2016	38.2565	-85.7542	130.4		https://www.google.com/maps/search/38.2565,-85.7542	https://data.globe.gov/system/photos/2019/08/15/1169032/original.jpg	https://data.globe.gov/system/photos/2019/08/15/1169034/original.jpg	https://data.globe.gov/system/photos/2019/08/15/1169033/original.jpg	https://data.globe.gov/system/photos/2019/08/15/1169035/original.jpg	https://data.globe.gov/system/photos/2019/08/15/1169037/original.jpg
GV_2017	38.2567	-85.7566	141		https://www.google.com/maps/search/38.2567,-85.7566	https://data.globe.gov/system/photos/2019/08/15/1169038/original.jpg	https://data.globe.gov/system/photos/2019/08/15/1169040/original.jpg	https://data.globe.gov/system/photos/2019/08/15/1169039/original.jpg	https://data.globe.gov/system/photos/2019/08/15/1169041/original.jpg	https://data.globe.gov/system/photos/2019/08/15/1169043/original.jpg
GV_2018	38.2568	-85.758	140.7		https://www.google.com/maps/search/38.2568,-85.758	https://data.globe.gov/system/photos/2019/08/15/1169044/original.jpg	https://data.globe.gov/system/photos/2019/08/15/1169046/original.jpg	https://data.globe.gov/system/photos/2019/08/15/1169045/original.jpg	https://data.globe.gov/system/photos/2019/08/15/1169047/original.jpg	https://data.globe.gov/system/photos/2019/08/15/1169049/original.jpg
GV_2019	38.2567	-85.7583	140.1		https://www.google.com/maps/search/38.2567,-85.7583	https://data.globe.gov/system/photos/2019/08/15/1169050/original.jpg	https://data.globe.gov/system/photos/2019/08/15/1169052/original.jpg	https://data.globe.gov/system/photos/2019/08/15/1169051/original.jpg	https://data.globe.gov/system/photos/2019/08/15/1169053/original.jpg	https://data.globe.gov/system/photos/2019/08/15/1169055/original.jpg
GV_2020	38.2571	-85.7605	139.8		https://www.google.com/maps/search/38.2571,-85.7605	https://data.globe.gov/system/photos/2019/08/15/1169056/original.jpg	https://data.globe.gov/system/photos/2019/08/15/1169058/original.jpg	https://data.globe.gov/system/photos/2019/08/15/1169057/original.jpg	https://data.globe.gov/system/photos/2019/08/15/1169059/original.jpg	https://data.globe.gov/system/photos/2019/08/15/1169061/original.jpg
GV_2021	38.2574	-85.7615	138.5		https://www.google.com/maps/search/38.2574,-85.7615	https://data.globe.gov/system/photos/2019/08/15/1169062/original.jpg	https://data.globe.gov/system/photos/2019/08/15/1169064/original.jpg	https://data.globe.gov/system/photos/2019/08/15/1169063/original.jpg	https://data.globe.gov/system/photos/2019/08/15/1169065/original.jpg	https://data.globe.gov/system/photos/2019/08/15/1169067/original.jpg
GV_2022	38.2575	-85.7645	139.4		https://www.google.com/maps/search/38.2575,-85.7645	https://data.globe.gov/system/photos/2019/08/15/1169068/original.jpg	https://data.globe.gov/system/photos/2019/08/15/1169070/original.jpg	https://data.globe.gov/system/photos/2019/08/15/1169069/original.jpg	https://data.globe.gov/system/photos/2019/08/15/1169071/original.jpg	https://data.globe.gov/system/photos/2019/08/15/1169073/original.jpg
GV_2023	38.2573	-85.7626	140.4		https://www.google.com/maps/search/38.2573,-85.7626	https://data.globe.gov/system/photos/2019/08/15/1169074/original.jpg	https://data.globe.gov/system/photos/2019/08/15/1169076/original.jpg	https://data.globe.gov/system/photos/2019/08/15/1169075/original.jpg	https://data.globe.gov/system/photos/2019/08/15/1169077/original.jpg	https://data.globe.gov/system/photos/2019/08/15/1169079/original.jpg
GV_2024	38.2575	-85.7638	139.8		https://www.google.com/maps/search/38.2575,-85.7638	https://data.globe.gov/system/photos/2019/08/15/1169080/original.jpg	https://data.globe.gov/system/photos/2019/08/15/1169082/original.jpg	https://data.globe.gov/system/photos/2019/08/15/1169081/original.jpg	https://data.globe.gov/system/photos/2019/08/15/1169083/original.jpg	https://data.globe.gov/system/photos/2019/08/15/1169085/original.jpg
GV_2025	38.2575	-85.7655	139.5		https://www.google.com/maps/search/38.2575,-85.7655	https://data.globe.gov/system/photos/2019/08/15/1169092/original.jpg	https://data.globe.gov/system/photos/2019/08/15/1169094/original.jpg	https://data.globe.gov/system/photos/2019/08/15/1169093/original.jpg	https://data.globe.gov/system/photos/2019/08/15/1169095/original.jpg	https://data.globe.gov/system/photos/2019/08/15/1169097/original.jpg
GV_2026	38.2569	-85.7631	140.1		https://www.google.com/maps/search/38.2569,-85.7631	https://data.globe.gov/system/photos/2019/08/15/1169098/original.jpg	https://data.globe.gov/system/photos/2019/08/15/1169100/original.jpg	https://data.globe.gov/system/photos/2019/08/15/1169099/original.jpg	https://data.globe.gov/system/photos/2019/08/15/1169101/original.jpg	https://data.globe.gov/system/photos/2019/08/15/1169103/original.jpg
GV_2027	38.2542	-85.7596	140		https://www.google.com/maps/search/38.2542,-85.7596	https://data.globe.gov/system/photos/2019/08/15/1169104/original.jpg	https://data.globe.gov/system/photos/2019/08/15/1169106/original.jpg	https://data.globe.gov/system/photos/2019/08/15/1169105/original.jpg	https://data.globe.gov/system/photos/2019/08/15/1169107/original.jpg	https://data.globe.gov/system/photos/2019/08/15/1169109/original.jpg
GV_2028	38.2588	-85.7631	143.4		https://www.google.com/maps/search/38.2588,-85.7631	https://data.globe.gov/system/photos/2019/08/15/1169110/original.jpg	https://data.globe.gov/system/photos/2019/08/15/1169112/original.jpg	https://data.globe.gov/system/photos/2019/08/15/1169111/original.jpg	https://data.globe.gov/system/photos/2019/08/15/1169113/original.jpg	https://data.globe.gov/system/photos/2019/08/15/1169115/original.jpg
GV_2029	38.259	-85.7616	131.8		https://www.google.com/maps/search/38.259,-85.7616	https://data.globe.gov/system/photos/2019/08/15/1169116/original.jpg	https://data.globe.gov/system/photos/2019/08/15/1169118/original.jpg	https://data.globe.gov/system/photos/2019/08/15/1169117/original.jpg	https://data.globe.gov/system/photos/2019/08/15/1169119/original.jpg	https://data.globe.gov/system/photos/2019/08/15/1169121/original.jpg
GV_2030	38.259	-85.7608	130.9		https://www.google.com/maps/search/38.259,-85.7608	https://data.globe.gov/system/photos/2019/08/15/1169122/original.jpg	https://data.globe.gov/system/photos/2019/08/15/1169124/original.jpg	https://data.globe.gov/system/photos/2019/08/15/1169123/original.jpg	https://data.globe.gov/system/photos/2019/08/15/1169125/original.jpg	https://data.globe.gov/system/photos/2019/08/15/1169127/original.jpg
GV_2031	38.2591	-85.7598	127.9		https://www.google.com/maps/search/38.2591,-85.7598	https://data.globe.gov/system/photos/2019/08/15/1169128/original.jpg	https://data.globe.gov/system/photos/2019/08/15/1169130/original.jpg	https://data.globe.gov/system/photos/2019/08/15/1169129/original.jpg	https://data.globe.gov/system/photos/2019/08/15/1169131/original.jpg	https://data.globe.gov/system/photos/2019/08/15/1169133/original.jpg
GV_2032	38.2547	-85.7601	140.1		https://www.google.com/maps/search/38.2547,-85.7601	https://data.globe.gov/system/photos/2019/08/15/1169134/original.jpg	https://data.globe.gov/system/photos/2019/08/15/1169136/original.jpg	https://data.globe.gov/system/photos/2019/08/15/1169135/original.jpg	https://data.globe.gov/system/photos/2019/08/15/1169137/original.jpg	https://data.globe.gov/system/photos/2019/08/15/1169139/original.jpg
GV_2033	38.2544	-85.7595	140.9		https://www.google.com/maps/search/38.2544,-85.7595	https://data.globe.gov/system/photos/2019/08/15/1169140/original.jpg	https://data.globe.gov/system/photos/2019/08/15/1169142/original.jpg	https://data.globe.gov/system/photos/2019/08/15/1169141/original.jpg	https://data.globe.gov/system/photos/2019/08/15/1169143/original.jpg	https://data.globe.gov/system/photos/2019/08/15/1169145/original.jpg
GV_2034	38.254	-85.7595	140.1		https://www.google.com/maps/search/38.254,-85.7595	https://data.globe.gov/system/photos/2019/08/15/1169146/original.jpg	https://data.globe.gov/system/photos/2019/08/15/1169148/original.jpg	https://data.globe.gov/system/photos/2019/08/15/1169147/original.jpg	https://data.globe.gov/system/photos/2019/08/15/1169149/original.jpg	https://data.globe.gov/system/photos/2019/08/15/1169151/original.jpg
GV_2035	38.2534	-85.76	140.8		https://www.google.com/maps/search/38.2534,-85.76	https://data.globe.gov/system/photos/2019/08/15/1169152/original.jpg	https://data.globe.gov/system/photos/2019/08/15/1169154/original.jpg	https://data.globe.gov/system/photos/2019/08/15/1169153/original.jpg	https://data.globe.gov/system/photos/2019/08/15/1169155/original.jpg	https://data.globe.gov/system/photos/2019/08/15/1169157/original.jpg
GV_2036	38.2544	-85.7597	140.8		https://www.google.com/maps/search/38.2544,-85.7597	https://data.globe.gov/system/photos/2019/08/15/1169158/original.jpg	https://data.globe.gov/system/photos/2019/08/15/1169160/original.jpg	https://data.globe.gov/system/photos/2019/08/15/1169159/original.jpg	https://data.globe.gov/system/photos/2019/08/15/1169161/original.jpg	https://data.globe.gov/system/photos/2019/08/15/1169163/original.jpg
GV_2037	38.254	-85.7571	140.9		https://www.google.com/maps/search/38.254,-85.7571	https://data.globe.gov/system/photos/2019/08/15/1169164/original.jpg	https://data.globe.gov/system/photos/2019/08/15/1169166/original.jpg	https://data.globe.gov/system/photos/2019/08/15/1169165/original.jpg	https://data.globe.gov/system/photos/2019/08/15/1169167/original.jpg	https://data.globe.gov/system/photos/2019/08/15/1169169/original.jpg
GV_2038	38.2548	-85.7527	141.3		https://www.google.com/maps/search/38.2548,-85.7527	https://data.globe.gov/system/photos/2019/08/15/1169170/original.jpg	https://data.globe.gov/system/photos/2019/08/15/1169172/original.jpg	https://data.globe.gov/system/photos/2019/08/15/1169171/original.jpg	https://data.globe.gov/system/photos/2019/08/15/1169173/original.jpg	https://data.globe.gov/system/photos/2019/08/15/1169175/original.jpg
GV_2039	38.2562	-85.7525	141.1		https://www.google.com/maps/search/38.2562,-85.7525	https://data.globe.gov/system/photos/2019/08/15/1169176/original.jpg	https://data.globe.gov/system/photos/2019/08/15/1169178/original.jpg	https://data.globe.gov/system/photos/2019/08/15/1169177/original.jpg	https://data.globe.gov/system/photos/2019/08/15/1169179/original.jpg	https://data.globe.gov/system/photos/2019/08/15/1169181/original.jpg
GV_2040	38.2564	-85.7516	138.8		https://www.google.com/maps/search/38.2564,-85.7516	https://data.globe.gov/system/photos/2019/08/15/1169182/original.jpg	https://data.globe.gov/system/photos/2019/08/15/1169184/original.jpg	https://data.globe.gov/system/photos/2019/08/15/1169183/original.jpg	https://data.globe.gov/system/photos/2019/08/15/1169185/original.jpg	https://data.globe.gov/system/photos/2019/08/15/1169187/original.jpg
GV_2041	38.2561	-85.752	140.8		https://www.google.com/maps/search/38.2561,-85.752	https://data.globe.gov/system/photos/2019/08/15/1169188/original.jpg	https://data.globe.gov/system/photos/2019/08/15/1169190/original.jpg	https://data.globe.gov/system/photos/2019/08/15/1169189/original.jpg	https://data.globe.gov/system/photos/2019/08/15/1169191/original.jpg	https://data.globe.gov/system/photos/2019/08/15/1169193/original.jpg
GV_2042	38.2561	-85.7533	140.9		https://www.google.com/maps/search/38.2561,-85.7533	https://data.globe.gov/system/photos/2019/08/15/1169194/original.jpg	https://data.globe.gov/system/photos/2019/08/15/1169196/original.jpg	https://data.globe.gov/system/photos/2019/08/15/1169195/original.jpg	https://data.globe.gov/system/photos/2019/08/15/1169197/original.jpg	https://data.globe.gov/system/photos/2019/08/15/1169199/original.jpg
GV_2043	38.2621	-85.7409	134.7		https://www.google.com/maps/search/38.2621,-85.7409	https://data.globe.gov/system/photos/2019/08/16/1169358/original.jpg	https://data.globe.gov/system/photos/2019/08/16/1169360/original.jpg	https://data.globe.gov/system/photos/2019/08/16/1169359/original.jpg	https://data.globe.gov/system/photos/2019/08/16/1169361/original.jpg	https://data.globe.gov/system/photos/2019/08/16/1169363/original.jpg
GV_2044	38.264	-85.725	134		https://www.google.com/maps/search/38.264,-85.725	https://data.globe.gov/system/photos/2019/08/16/1169371/original.jpg	https://data.globe.gov/system/photos/2019/08/16/1169373/original.jpg	https://data.globe.gov/system/photos/2019/08/16/1169372/original.jpg	https://data.globe.gov/system/photos/2019/08/16/1169374/original.jpg	https://data.globe.gov/system/photos/2019/08/16/1169376/original.jpg
GV_2045	41.957	-83.4098	186.4		https://www.google.com/maps/search/41.957,-83.4098	https://data.globe.gov/system/photos/2019/08/16/1169377/original.jpg	https://data.globe.gov/system/photos/2019/08/16/1169379/original.jpg	https://data.globe.gov/system/photos/2019/08/16/1169378/original.jpg	https://data.globe.gov/system/photos/2019/08/16/1169380/original.jpg	https://data.globe.gov/system/photos/2019/08/16/1169382/original.jpg
GV_2046	41.9571	-83.4098	186.4		https://www.google.com/maps/search/41.9571,-83.4098	https://data.globe.gov/system/photos/2019/08/16/1169390/original.jpg	https://data.globe.gov/system/photos/2019/08/16/1169392/original.jpg	https://data.globe.gov/system/photos/2019/08/16/1169391/original.jpg	https://data.globe.gov/system/photos/2019/08/16/1169393/original.jpg	https://data.globe.gov/system/photos/2019/08/16/1169395/original.jpg
GV_2047	41.957	-83.4098	186.4		https://www.google.com/maps/search/41.957,-83.4098	https://data.globe.gov/system/photos/2019/08/16/1169396/original.jpg	https://data.globe.gov/system/photos/2019/08/16/1169398/original.jpg	https://data.globe.gov/system/photos/2019/08/16/1169397/original.jpg	https://data.globe.gov/system/photos/2019/08/16/1169399/original.jpg	https://data.globe.gov/system/photos/2019/08/16/1169401/original.jpg
GV_2048	41.9567	-83.4103	186.1		https://www.google.com/maps/search/41.9567,-83.4103	https://data.globe.gov/system/photos/2019/08/16/1169420/original.jpg	https://data.globe.gov/system/photos/2019/08/16/1169422/original.jpg	https://data.globe.gov/system/photos/2019/08/16/1169421/original.jpg	https://data.globe.gov/system/photos/2019/08/16/1169423/original.jpg	https://data.globe.gov/system/photos/2019/08/16/1169425/original.jpg
GV_2049	2.5669	-72.64	184.5		https://www.google.com/maps/search/2.5669,-72.64	https://data.globe.gov/system/photos/2019/08/16/1169495/original.jpg	https://data.globe.gov/system/photos/2019/08/16/1169497/original.jpg	https://data.globe.gov/system/photos/2019/08/16/1169496/original.jpg	https://data.globe.gov/system/photos/2019/08/16/1169498/original.jpg	https://data.globe.gov/system/photos/2019/08/16/1169500/original.jpg
GV_2050	42.1918	-71.3285	38.9		https://www.google.com/maps/search/42.1918,-71.3285	https://data.globe.gov/system/photos/2019/08/13/1169501/original.jpg	https://data.globe.gov/system/photos/2019/08/13/1169503/original.jpg	https://data.globe.gov/system/photos/2019/08/13/1169502/original.jpg	https://data.globe.gov/system/photos/2019/08/13/1169504/original.jpg	https://data.globe.gov/system/photos/2019/08/13/1169506/original.jpg
GV_2051	-34.891	138.7459	317.2		https://www.google.com/maps/search/-34.891,138.7459	https://data.globe.gov/system/photos/2019/08/17/1169681/original.jpg	https://data.globe.gov/system/photos/2019/08/17/1169683/original.jpg	https://data.globe.gov/system/photos/2019/08/17/1169682/original.jpg	https://data.globe.gov/system/photos/2019/08/17/1169684/original.jpg	https://data.globe.gov/system/photos/2019/08/17/1169686/original.jpg
GV_2052	42.2446	-90.2711	195.5		https://www.google.com/maps/search/42.2446,-90.2711	https://data.globe.gov/system/photos/2019/08/17/1169789/original.jpg	https://data.globe.gov/system/photos/2019/08/17/1169791/original.jpg	https://data.globe.gov/system/photos/2019/08/17/1169790/original.jpg	https://data.globe.gov/system/photos/2019/08/17/1169792/original.jpg	https://data.globe.gov/system/photos/2019/08/17/1169794/original.jpg
GV_2053	41.9474	-87.6546	182.7		https://www.google.com/maps/search/41.9474,-87.6546	https://data.globe.gov/system/photos/2019/08/15/1169795/original.jpg	https://data.globe.gov/system/photos/2019/08/15/1169797/original.jpg	https://data.globe.gov/system/photos/2019/08/15/1169796/original.jpg	https://data.globe.gov/system/photos/2019/08/15/1169798/original.jpg	https://data.globe.gov/system/photos/2019/08/15/1169800/original.jpg
GV_2054	42.366	-90.457	181.7		https://www.google.com/maps/search/42.366,-90.457	https://data.globe.gov/system/photos/2019/08/17/1170198/original.jpg	https://data.globe.gov/system/photos/2019/08/17/1170200/original.jpg	https://data.globe.gov/system/photos/2019/08/17/1170199/original.jpg	https://data.globe.gov/system/photos/2019/08/17/1170201/original.jpg	https://data.globe.gov/system/photos/2019/08/17/1170203/original.jpg
GV_2055	42.2876	-90.3329	265		https://www.google.com/maps/search/42.2876,-90.3329	https://data.globe.gov/system/photos/2019/08/18/1170204/original.jpg	https://data.globe.gov/system/photos/2019/08/18/1170206/original.jpg	https://data.globe.gov/system/photos/2019/08/18/1170205/original.jpg	https://data.globe.gov/system/photos/2019/08/18/1170207/original.jpg	https://data.globe.gov/system/photos/2019/08/18/1170209/original.jpg
GV_2056	25.0473	121.5141	9.6		https://www.google.com/maps/search/25.0473,121.5141	https://data.globe.gov/system/photos/2019/08/18/1170312/original.jpg	https://data.globe.gov/system/photos/2019/08/18/1170314/original.jpg	https://data.globe.gov/system/photos/2019/08/18/1170313/original.jpg	https://data.globe.gov/system/photos/2019/08/18/1170315/original.jpg	https://data.globe.gov/system/photos/2019/08/18/1170317/original.jpg
GV_2057	53.4284	-0.6226	26		https://www.google.com/maps/search/53.4284,-0.6226	https://data.globe.gov/system/photos/2019/08/17/1170363/original.jpg	https://data.globe.gov/system/photos/2019/08/17/1170365/original.jpg	https://data.globe.gov/system/photos/2019/08/17/1170364/original.jpg	https://data.globe.gov/system/photos/2019/08/17/1170366/original.jpg	https://data.globe.gov/system/photos/2019/08/17/1170368/original.jpg
GV_2058	31.8763	-106.6009	1145.6		https://www.google.com/maps/search/31.8763,-106.6009	https://data.globe.gov/system/photos/2019/08/18/1170659/original.jpg	https://data.globe.gov/system/photos/2019/08/18/1170662/original.jpg	https://data.globe.gov/system/photos/2019/08/18/1170660/original.jpg	https://data.globe.gov/system/photos/2019/08/18/1170663/original.jpg	https://data.globe.gov/system/photos/2019/08/18/1170669/original.jpg
GV_2059	31.8761	-106.6005	1145.6		https://www.google.com/maps/search/31.8761,-106.6005	https://data.globe.gov/system/photos/2019/08/18/1170693/original.jpg	https://data.globe.gov/system/photos/2019/08/18/1170695/original.jpg	https://data.globe.gov/system/photos/2019/08/18/1170694/original.jpg	https://data.globe.gov/system/photos/2019/08/18/1170696/original.jpg	https://data.globe.gov/system/photos/2019/08/18/1170698/original.jpg
GV_2060	31.8738	-106.5513	1259.2		https://www.google.com/maps/search/31.8738,-106.5513	https://data.globe.gov/system/photos/2019/08/18/1170724/original.jpg	https://data.globe.gov/system/photos/2019/08/18/1170726/original.jpg	https://data.globe.gov/system/photos/2019/08/18/1170725/original.jpg	https://data.globe.gov/system/photos/2019/08/18/1170727/original.jpg	https://data.globe.gov/system/photos/2019/08/18/1170729/original.jpg
GV_2061	53.4284	-0.6226	26		https://www.google.com/maps/search/53.4284,-0.6226	https://data.globe.gov/system/photos/2019/08/18/1170942/original.jpg	https://data.globe.gov/system/photos/2019/08/18/1170944/original.jpg	https://data.globe.gov/system/photos/2019/08/18/1170943/original.jpg	https://data.globe.gov/system/photos/2019/08/18/1170945/original.jpg	https://data.globe.gov/system/photos/2019/08/18/1170947/original.jpg
GV_2062	39.0078	-76.9045	21.7		https://www.google.com/maps/search/39.0078,-76.9045	https://data.globe.gov/system/photos/2019/08/17/1171561/original.jpg	https://data.globe.gov/system/photos/2019/08/17/1171563/original.jpg	https://data.globe.gov/system/photos/2019/08/17/1171562/original.jpg	https://data.globe.gov/system/photos/2019/08/17/1171564/original.jpg	https://data.globe.gov/system/photos/2019/08/17/1171566/original.jpg
GV_2063	2.4933	-72.6384	220.3		https://www.google.com/maps/search/2.4933,-72.6384	https://data.globe.gov/system/photos/2019/08/17/1171578/original.jpg	https://data.globe.gov/system/photos/2019/08/17/1171580/original.jpg	https://data.globe.gov/system/photos/2019/08/17/1171579/original.jpg	https://data.globe.gov/system/photos/2019/08/17/1171581/original.jpg	https://data.globe.gov/system/photos/2019/08/17/1171583/original.jpg

GV_2064	41.5856	-72.0467	20.3		https://www.google.com/maps/search/41.5856,-72.0467	https://data.globe.gov/system/photos/2019/08/17/1171633/original.jpg	https://data.globe.gov/system/photos/2019/08/17/1171635/original.jpg	https://data.globe.gov/system/photos/2019/08/17/1171634/original.jpg	https://data.globe.gov/system/photos/2019/08/17/1171642/original.jpg	https://data.globe.gov/system/photos/2019/08/17/1171644/original.jpg
GV_2065	41.5719	-72.0054	39.8		https://www.google.com/maps/search/41.5719,-72.0054	https://data.globe.gov/system/photos/2019/08/19/1171667/original.jpg	https://data.globe.gov/system/photos/2019/08/19/1171673/original.jpg	https://data.globe.gov/system/photos/2019/08/19/1171672/original.jpg	https://data.globe.gov/system/photos/2019/08/19/1171674/original.jpg	https://data.globe.gov/system/photos/2019/08/19/1171676/original.jpg
GV_2066	45.4971	-122.6678	9.1		https://www.google.com/maps/search/45.4971,-122.6678	https://data.globe.gov/system/photos/2019/08/19/1171935/original.jpg	https://data.globe.gov/system/photos/2019/08/19/1171937/original.jpg	https://data.globe.gov/system/photos/2019/08/19/1171936/original.jpg	https://data.globe.gov/system/photos/2019/08/19/1171938/original.jpg	https://data.globe.gov/system/photos/2019/08/19/1171940/original.jpg
GV_2067	34.0151	-109.4572	2531.1		https://www.google.com/maps/search/34.0151,-109.4572	https://data.globe.gov/system/photos/2019/08/19/1172006/original.jpg	https://data.globe.gov/system/photos/2019/08/19/1172008/original.jpg	https://data.globe.gov/system/photos/2019/08/19/1172007/original.jpg	https://data.globe.gov/system/photos/2019/08/19/1172009/original.jpg	https://data.globe.gov/system/photos/2019/08/19/1172011/original.jpg
GV_2068	-25.3318	127.7211	560.8		https://www.google.com/maps/search/-25.3318,127.7211	https://data.globe.gov/system/photos/2019/08/15/1172185/original.jpg	https://data.globe.gov/system/photos/2019/08/15/1172187/original.jpg	https://data.globe.gov/system/photos/2019/08/15/1172186/original.jpg	https://data.globe.gov/system/photos/2019/08/15/1172188/original.jpg	https://data.globe.gov/system/photos/2019/08/15/1172190/original.jpg
GV_2069	-27.0591	124.5646	437		https://www.google.com/maps/search/-27.0591,124.5646	https://data.globe.gov/system/photos/2019/08/16/1172197/original.jpg	https://data.globe.gov/system/photos/2019/08/16/1172199/original.jpg	https://data.globe.gov/system/photos/2019/08/16/1172198/original.jpg	https://data.globe.gov/system/photos/2019/08/16/1172200/original.jpg	https://data.globe.gov/system/photos/2019/08/16/1172202/original.jpg
GV_2070	-27.0507	124.5642	444.1		https://www.google.com/maps/search/-27.0507,124.5642	https://data.globe.gov/system/photos/2019/08/16/1172203/original.jpg	https://data.globe.gov/system/photos/2019/08/16/1172205/original.jpg	https://data.globe.gov/system/photos/2019/08/16/1172204/original.jpg	https://data.globe.gov/system/photos/2019/08/16/1172206/original.jpg	https://data.globe.gov/system/photos/2019/08/16/1172208/original.jpg
GV_2071	-27.705	124.1757	422.6		https://www.google.com/maps/search/-27.705,124.1757	https://data.globe.gov/system/photos/2019/08/16/1172209/original.jpg	https://data.globe.gov/system/photos/2019/08/16/1172211/original.jpg	https://data.globe.gov/system/photos/2019/08/16/1172210/original.jpg	https://data.globe.gov/system/photos/2019/08/16/1172212/original.jpg	https://data.globe.gov/system/photos/2019/08/16/1172214/original.jpg
GV_2072	-28.041	119.3053	522.9		https://www.google.com/maps/search/-28.041,119.3053	https://data.globe.gov/system/photos/2019/08/20/1172215/original.jpg	https://data.globe.gov/system/photos/2019/08/20/1172217/original.jpg	https://data.globe.gov/system/photos/2019/08/20/1172216/original.jpg	https://data.globe.gov/system/photos/2019/08/20/1172218/original.jpg	https://data.globe.gov/system/photos/2019/08/20/1172220/original.jpg
GV_2073	54.0123	22.9915	177.7		https://www.google.com/maps/search/54.0123,22.9915	https://data.globe.gov/system/photos/2019/08/20/1172647/original.jpg	https://data.globe.gov/system/photos/2019/08/20/1172649/original.jpg	https://data.globe.gov/system/photos/2019/08/20/1172648/original.jpg	https://data.globe.gov/system/photos/2019/08/20/1172650/original.jpg	https://data.globe.gov/system/photos/2019/08/20/1172652/original.jpg
GV_2074	54.0119	22.9923	177.5		https://www.google.com/maps/search/54.0119,22.9923	https://data.globe.gov/system/photos/2019/08/20/1172660/original.jpg	https://data.globe.gov/system/photos/2019/08/20/1172662/original.jpg	https://data.globe.gov/system/photos/2019/08/20/1172661/original.jpg	https://data.globe.gov/system/photos/2019/08/20/1172663/original.jpg	https://data.globe.gov/system/photos/2019/08/20/1172665/original.jpg
GV_2075	35.3061	-106.5755	1537.7		https://www.google.com/maps/search/35.3061,-106.5755	https://data.globe.gov/system/photos/2019/08/18/1172835/original.jpg	https://data.globe.gov/system/photos/2019/08/18/1172840/original.jpg	https://data.globe.gov/system/photos/2019/08/18/1172838/original.jpg	https://data.globe.gov/system/photos/2019/08/18/1172842/original.jpg	https://data.globe.gov/system/photos/2019/08/18/1172845/original.jpg
GV_2076	52.1071	-109.7121	689		https://www.google.com/maps/search/52.1071,-109.7121	https://data.globe.gov/system/photos/2019/08/19/1173268/original.jpg	https://data.globe.gov/system/photos/2019/08/19/1173270/original.jpg	https://data.globe.gov/system/photos/2019/08/19/1173269/original.jpg	https://data.globe.gov/system/photos/2019/08/19/1173271/original.jpg	https://data.globe.gov/system/photos/2019/08/19/1173273/original.jpg
GV_2077	40.6325	-74.0261	26		https://www.google.com/maps/search/40.6325,-74.0261	https://data.globe.gov/system/photos/2019/07/18/1173299/original.jpg	https://data.globe.gov/system/photos/2019/07/18/1173301/original.jpg	https://data.globe.gov/system/photos/2019/07/18/1173300/original.jpg	https://data.globe.gov/system/photos/2019/07/18/1173302/original.jpg	https://data.globe.gov/system/photos/2019/07/18/1173304/original.jpg
GV_2078	43.8535	-101.4833	747.2		https://www.google.com/maps/search/43.8535,-101.4833	https://data.globe.gov/system/photos/2019/08/20/1173700/original.jpg	https://data.globe.gov/system/photos/2019/08/20/1173706/original.jpg	https://data.globe.gov/system/photos/2019/08/20/1173704/original.jpg	https://data.globe.gov/system/photos/2019/08/20/1173707/original.jpg	https://data.globe.gov/system/photos/2019/08/20/1173715/original.jpg
GV_2079	45.564	-107.4177	1005.2		https://www.google.com/maps/search/45.564,-107.4177	https://data.globe.gov/system/photos/2019/08/20/1173718/original.jpg	https://data.globe.gov/system/photos/2019/08/20/1173721/original.jpg	https://data.globe.gov/system/photos/2019/08/20/1173720/original.jpg	https://data.globe.gov/system/photos/2019/08/20/1173724/original.jpg	https://data.globe.gov/system/photos/2019/08/20/1173729/original.jpg
GV_2080	40.8242	-74.2045	87.2		https://www.google.com/maps/search/40.8242,-74.2045	https://data.globe.gov/system/photos/2019/08/21/1173884/original.jpg	https://data.globe.gov/system/photos/2019/08/21/1173886/original.jpg	https://data.globe.gov/system/photos/2019/08/21/1173885/original.jpg	https://data.globe.gov/system/photos/2019/08/21/1173887/original.jpg	https://data.globe.gov/system/photos/2019/08/21/1173889/original.jpg
GV_2081	40.8241	-74.2045	87.4		https://www.google.com/maps/search/40.8241,-74.2045	https://data.globe.gov/system/photos/2019/08/21/1173890/original.jpg	https://data.globe.gov/system/photos/2019/08/21/1173892/original.jpg	https://data.globe.gov/system/photos/2019/08/21/1173891/original.jpg	https://data.globe.gov/system/photos/2019/08/21/1173893/original.jpg	https://data.globe.gov/system/photos/2019/08/21/1173895/original.jpg
GV_2082	53.4284	-0.6226	26		https://www.google.com/maps/search/53.4284,-0.6226	https://data.globe.gov/system/photos/2019/08/21/1174199/original.jpg	https://data.globe.gov/system/photos/2019/08/21/1174201/original.jpg	https://data.globe.gov/system/photos/2019/08/21/1174200/original.jpg	https://data.globe.gov/system/photos/2019/08/21/1174202/original.jpg	https://data.globe.gov/system/photos/2019/08/21/1174204/original.jpg
GV_2083	53.4125	-0.647	22.7		https://www.google.com/maps/search/53.4125,-0.647	https://data.globe.gov/system/photos/2019/08/21/1174205/original.jpg	https://data.globe.gov/system/photos/2019/08/21/1174207/original.jpg	https://data.globe.gov/system/photos/2019/08/21/1174206/original.jpg	https://data.globe.gov/system/photos/2019/08/21/1174208/original.jpg	https://data.globe.gov/system/photos/2019/08/21/1174210/original.jpg
GV_2084	53.4266	-0.6295	24.9		https://www.google.com/maps/search/53.4266,-0.6295	https://data.globe.gov/system/photos/2019/08/19/1174211/original.jpg	https://data.globe.gov/system/photos/2019/08/19/1174213/original.jpg	https://data.globe.gov/system/photos/2019/08/19/1174212/original.jpg	https://data.globe.gov/system/photos/2019/08/19/1174214/original.jpg	https://data.globe.gov/system/photos/2019/08/19/1174216/original.jpg
GV_2085	32.2745	-110.8249	785.7		https://www.google.com/maps/search/32.2745,-110.8249	https://data.globe.gov/system/photos/2019/08/21/1174283/original.jpg	https://data.globe.gov/system/photos/2019/08/21/1174285/original.jpg	https://data.globe.gov/system/photos/2019/08/21/1174284/original.jpg	https://data.globe.gov/system/photos/2019/08/21/1174286/original.jpg	https://data.globe.gov/system/photos/2019/08/21/1174288/original.jpg
GV_2086	45.4965	-122.6714	10.9		https://www.google.com/maps/search/45.4965,-122.6714	https://data.globe.gov/system/photos/2019/08/22/1175052/original.jpg	https://data.globe.gov/system/photos/2019/08/22/1175054/original.jpg	https://data.globe.gov/system/photos/2019/08/22/1175053/original.jpg	https://data.globe.gov/system/photos/2019/08/22/1175055/original.jpg	https://data.globe.gov/system/photos/2019/08/22/1175057/original.jpg
GV_2087	61.604	-149.1383	86.8		https://www.google.com/maps/search/61.604,-149.1383	https://data.globe.gov/system/photos/2019/08/22/1175186/original.jpg	https://data.globe.gov/system/photos/2019/08/22/1175189/original.jpg	https://data.globe.gov/system/photos/2019/08/22/1175187/original.jpg	https://data.globe.gov/system/photos/2019/08/22/1175190/original.jpg	https://data.globe.gov/system/photos/2019/08/22/1175192/original.jpg
GV_2088	-34.5275	139.0097	340.9		https://www.google.com/maps/search/-34.5275,139.0097	https://data.globe.gov/system/photos/2019/08/23/1175534/original.jpg	https://data.globe.gov/system/photos/2019/08/23/1175536/original.jpg	https://data.globe.gov/system/photos/2019/08/23/1175535/original.jpg	https://data.globe.gov/system/photos/2019/08/23/1175537/original.jpg	https://data.globe.gov/system/photos/2019/08/23/1175539/original.jpg
GV_2089	-34.5322	138.9874	282.2		https://www.google.com/maps/search/-34.5322,138.9874	https://data.globe.gov/system/photos/2019/08/23/1175546/original.jpg	https://data.globe.gov/system/photos/2019/08/23/1175548/original.jpg	https://data.globe.gov/system/photos/2019/08/23/1175547/original.jpg	https://data.globe.gov/system/photos/2019/08/23/1175549/original.jpg	https://data.globe.gov/system/photos/2019/08/23/1175551/original.jpg
GV_2090	40.8256	-74.2031	88.6		https://www.google.com/maps/search/40.8256,-74.2031	https://data.globe.gov/system/photos/2019/08/22/1176328/original.jpg	https://data.globe.gov/system/photos/2019/08/22/1176330/original.jpg	https://data.globe.gov/system/photos/2019/08/22/1176329/original.jpg	https://data.globe.gov/system/photos/2019/08/22/1176331/original.jpg	https://data.globe.gov/system/photos/2019/08/22/1176333/original.jpg
GV_2091	40.8273	-74.2023	90		https://www.google.com/maps/search/40.8273,-74.2023	https://data.globe.gov/system/photos/2019/08/22/1176336/original.jpg	https://data.globe.gov/system/photos/2019/08/22/1176338/original.jpg	https://data.globe.gov/system/photos/2019/08/22/1176337/original.jpg	https://data.globe.gov/system/photos/2019/08/22/1176339/original.jpg	https://data.globe.gov/system/photos/2019/08/22/1176341/original.jpg
GV_2092	40.828	-74.2019	88.4		https://www.google.com/maps/search/40.828,-74.2019	https://data.globe.gov/system/photos/2019/08/22/1176349/original.jpg	https://data.globe.gov/system/photos/2019/08/22/1176360/original.jpg	https://data.globe.gov/system/photos/2019/08/22/1176355/original.jpg	https://data.globe.gov/system/photos/2019/08/22/1176361/original.jpg	https://data.globe.gov/system/photos/2019/08/22/1176363/original.jpg
GV_2093	40.8282	-74.2016	88.3		https://www.google.com/maps/search/40.8282,-74.2016	https://data.globe.gov/system/photos/2019/08/22/1176365/original.jpg	https://data.globe.gov/system/photos/2019/08/22/1176367/original.jpg	https://data.globe.gov/system/photos/2019/08/22/1176366/original.jpg	https://data.globe.gov/system/photos/2019/08/22/1176368/original.jpg	https://data.globe.gov/system/photos/2019/08/22/1176370/original.jpg
GV_2094	40.8291	-74.2011	86.9		https://www.google.com/maps/search/40.8291,-74.2011	https://data.globe.gov/system/photos/2019/08/22/1176372/original.jpg	https://data.globe.gov/system/photos/2019/08/22/1176374/original.jpg	https://data.globe.gov/system/photos/2019/08/22/1176373/original.jpg	https://data.globe.gov/system/photos/2019/08/22/1176375/original.jpg	https://data.globe.gov/system/photos/2019/08/22/1176377/original.jpg
GV_2095	40.8295	-74.2008	86.3		https://www.google.com/maps/search/40.8295,-74.2008	https://data.globe.gov/system/photos/2019/08/22/1176378/original.jpg	https://data.globe.gov/system/photos/2019/08/22/1176380/original.jpg	https://data.globe.gov/system/photos/2019/08/22/1176379/original.jpg	https://data.globe.gov/system/photos/2019/08/22/1176381/original.jpg	https://data.globe.gov/system/photos/2019/08/22/1176387/original.jpg
GV_2096	39.2532	-77.196	177.3		https://www.google.com/maps/search/39.2532,-77.196	https://data.globe.gov/system/photos/2019/08/23/1176590/original.jpg	https://data.globe.gov/system/photos/2019/08/23/1176592/original.jpg	https://data.globe.gov/system/photos/2019/08/23/1176591/original.jpg	https://data.globe.gov/system/photos/2019/08/23/1176593/original.jpg	https://data.globe.gov/system/photos/2019/08/23/1176595/original.jpg
GV_2097	-27.3913	117.4759	418		https://www.google.com/maps/search/-27.3913,117.4759	https://data.globe.gov/system/photos/2019/08/24/1176721/original.jpg	https://data.globe.gov/system/photos/2019/08/24/1176723/original.jpg	https://data.globe.gov/system/photos/2019/08/24/1176722/original.jpg	https://data.globe.gov/system/photos/2019/08/24/1176724/original.jpg	https://data.globe.gov/system/photos/2019/08/24/1176726/original.jpg
GV_2098	41.5948	-72.0498	21		https://www.google.com/maps/search/41.5948,-72.0498	https://data.globe.gov/system/photos/2019/08/22/1177195/original.jpg	https://data.globe.gov/system/photos/2019/08/22/1177198/original.jpg	https://data.globe.gov/system/photos/2019/08/22/1177197/original.jpg	https://data.globe.gov/system/photos/2019/08/22/1177199/original.jpg	https://data.globe.gov/system/photos/2019/08/22/1177201/original.jpg
GV_2099	53.414	-0.6052	37.9		https://www.google.com/maps/search/53.414,-0.6052	https://data.globe.gov/system/photos/2019/08/24/1177263/original.jpg	https://data.globe.gov/system/photos/2019/08/24/1177265/original.jpg	https://data.globe.gov/system/photos/2019/08/24/1177264/original.jpg	https://data.globe.gov/system/photos/2019/08/24/1177266/original.jpg	https://data.globe.gov/system/photos/2019/08/24/1177268/original.jpg
GV_2100	-34.4596	138.8203	189.9		https://www.google.com/maps/search/-34.4596,138.8203	https://data.globe.gov/system/photos/2019/08/25/1177551/original.jpg	https://data.globe.gov/system/photos/2019/08/25/1177553/original.jpg	https://data.globe.gov/system/photos/2019/08/25/1177552/original.jpg	https://data.globe.gov/system/photos/2019/08/25/1177554/original.jpg	https://data.globe.gov/system/photos/2019/08/25/1177556/original.jpg
GV_2101	29.9054	-95.6813	42.6		https://www.google.com/maps/search/29.9054,-95.6813	https://data.globe.gov/system/photos/2019/08/21/1177612/original.jpg	https://data.globe.gov/system/photos/2019/08/21/1177614/original.jpg	https://data.globe.gov/system/photos/2019/08/21/1177613/original.jpg	https://data.globe.gov/system/photos/2019/08/21/1177615/original.jpg	https://data.globe.gov/system/photos/2019/08/21/1177617/original.jpg
GV_2102	25.0213	121.3063	77.3		https://www.google.com/maps/search/25.0213,121.3063	https://data.globe.gov/system/photos/2019/08/25/1177642/original.jpg	https://data.globe.gov/system/photos/2019/08/25/1177644/original.jpg	https://data.globe.gov/system/photos/2019/08/25/1177643/original.jpg	https://data.globe.gov/system/photos/2019/08/25/1177645/original.jpg	https://data.globe.gov/system/photos/2019/08/25/1177647/original.jpg
GV_2103	-27.3701	117.7431	416.8		https://www.google.com/maps/search/-27.3701,117.7431	https://data.globe.gov/system/photos/2019/08/25/1177671/original.jpg	https://data.globe.gov/system/photos/2019/08/25/1177673/original.jpg	https://data.globe.gov/system/photos/2019/08/25/1177672/original.jpg	https://data.globe.gov/system/photos/2019/08/25/1177674/original.jpg	https://data.globe.gov/system/photos/2019/08/25/1177676/original.jpg
GV_2104	-27.3975	117.82	427.4		https://www.google.com/maps/search/-27.3975,117.82	https://data.globe.gov/system/photos/2019/08/25/1177707/original.jpg	https://data.globe.gov/system/photos/2019/08/25/1177709/original.jpg	https://data.globe.gov/system/photos/2019/08/25/1177708/original.jpg	https://data.globe.gov/system/photos/2019/08/25/1177710/original.jpg	https://data.globe.gov/system/photos/2019/08/25/1177712/original.jpg
GV_2105	30.5169	-97.7416	240.5		https://www.google.com/maps/search/30.5169,-97.7416	https://data.globe.gov/system/photos/2019/08/25/1177876/original.jpg	https://data.globe.gov/system/photos/2019/08/25/1177878/original.jpg	https://data.globe.gov/system/photos/2019/08/25/1177877/original.jpg	https://data.globe.gov/system/photos/2019/08/25/1177879/original.jpg	https://data.globe.gov/system/photos/2019/08/25/1177881/original.jpg
GV_2106	24.84	121.5399	231.1		https://www.google.com/maps/search/24.84,121.5399	https://data.globe.gov/system/photos/2019/08/26/1178723/original.jpg	https://data.globe.gov/system/photos/2019/08/26/1178725/original.jpg	https://data.globe.gov/system/photos/2019/08/26/1178724/original.jpg	https://data.globe.gov/system/photos/2019/08/26/1178726/original.jpg	https://data.globe.gov/system/photos/2019/08/26/1178728/original.jpg
GV_2107	-34.4345	138.9328	291.8		https://www.google.com/maps/search/-34.4345,138.9328	https://data.globe.gov/system/photos/2019/08/26/1179956/original.jpg	https://data.globe.gov/system/photos/2019/08/26/1179958/original.jpg	https://data.globe.gov/system/photos/2019/08/26/1179957/original.jpg	https://data.globe.gov/system/photos/2019/08/26/1179965/original.jpg	https://data.globe.gov/system/photos/2019/08/26/1179979/original.jpg
GV_2108	52.0997	-109.6886	696.4		https://www.google.com/maps/search/52.0997,-109.6886	https://data.globe.gov/system/photos/2019/08/21/1180017/original.jpg	https://data.globe.gov/system/photos/2019/08/21/1180019/original.jpg	https://data.globe.gov/system/photos/2019/08/21/1180018/original.jpg	https://data.globe.gov/system/photos/2019/08/21/1180020/original.jpg	https://data.globe.gov/system/photos/2019/08/21/1180022/original.jpg
GV_2109	52.2203	-110.0342	651.7		https://www.google.com/maps/search/52.2203,-110.0342	https://data.globe.gov/system/photos/2019/08/26/1180029/original.jpg	https://data.globe.gov/system/photos/2019/08/26/1180031/original.jpg	https://data.globe.gov/system/photos/2019/08/26/1180030/original.jpg	https://data.globe.gov/system/photos/2019/08/26/1180032/original.jpg	https://data.globe.gov/system/photos/2019/08/26/1180034/original.jpg
GV_2110	-34.3541	138.9066	230.9		https://www.google.com/maps/search/-34.3541,138.9066	https://data.globe.gov/system/photos/2019/08/27/1180218/original.jpg	https://data.globe.gov/system/photos/2019/08/27/1180220/original.jpg	https://data.globe.gov/system/photos/2019/08/27/1180219/original.jpg	https://data.globe.gov/system/photos/2019/08/27/1180221/original.jpg	https://data.globe.gov/system/photos/2019/08/27/1180223/original.jpg
GV_2111	-34.4595	138.9281	266		https://www.google.com/maps/search/-34.4595,138.9281	https://data.globe.gov/system/photos/2019/08/27/1180242/original.jpg	https://data.globe.gov/system/photos/2019/08/27/1180244/original.jpg	https://data.globe.gov/system/photos/2019/08/27/1180243/original.jpg	https://data.globe.gov/system/photos/2019/08/27/1180245/original.jpg	https://data.globe.gov/system/photos/2019/08/27/1180247/original.jpg
GV_2112	-34.4777	138.8091	180.4		https://www.google.com/maps/search/-34.4777,138.8091	https://data.globe.gov/system/photos/2019/08/27/1180260/original.jpg	https://data.globe.gov/system/photos/2019/08/27/1180262/original.jpg	https://data.globe.gov/system/photos/2019/08/27/1180261/original.jpg	https://data.globe.gov/system/photos/2019/08/27/1180263/original.jpg	https://data.globe.gov/system/photos/2019/08/27/1180265/original.jpg
GV_2113	53.4109	-0.6008	44.1		https://www.google.com/maps/search/53.4109,-0.6008	https://data.globe.gov/system/photos/2019/08/27/1180278/original.jpg	https://data.globe.gov/system/photos/2019/08/27/1180280/original.jpg	https://data.globe.gov/system/photos/2019/08/27/1180279/original.jpg	https://data.globe.gov/system/photos/2019/08/27/1180281/original.jpg	https://data.globe.gov/system/photos/2019/08/27/1180283/original.jpg
GV_2114	53.4284	-0.6226	26		https://www.google.com/maps/search/53.4284,-0.6226	https://data.globe.gov/system/photos/2019/08/27/1181187/original.jpg	https://data.globe.gov/system/photos/2019/08/27/1181189/original.jpg	https://data.globe.gov/system/photos/2019/08/27/1181188/original.jpg	https://data.globe.gov/system/photos/2019/08/27/1181190/original.jpg	https://data.globe.gov/system/photos/2019/08/27/1181192/original.jpg
GV_2115	45.6112	-122.6061	7.6		https://www.google.com/maps/search/45.6112,-122.6061	https://data.globe.gov/system/photos/2019/08/27/1181405/original.jpg	https://data.globe.gov/system/photos/2019/08/27/1181407/original.jpg	https://data.globe.gov/system/photos/2019/08/27/1181406/original.jpg	https://data.globe.gov/system/photos/2019/08/27/1181408/original.jpg	https://data.globe.gov/system/photos/2019/08/27/1181411/original.jpg
GV_2116	45.6118	-122.6075	7.5		https://www.google.com/maps/search/45.6118,-122.6075	https://data.globe.gov/system/photos/2019/08/27/1181413/original.jpg	https://data.globe.gov/system/photos/2019/08/27/1181415/original.jpg	https://data.globe.gov/system/photos/2019/08/27/1181414/original.jpg	https://data.globe.gov/system/photos/2019/08/27/1181417/original.jpg	https://data.globe.gov/system/photos/2019/08/27/1181419/original.jpg
GV_2117	45.6119	-122.6111	9.6		https://www.google.com/maps/search/45.6119,-122.6111	https://data.globe.gov/system/photos/2019/08/27/1181420/original.jpg	https://data.globe.gov/system/photos/2019/08/27/1181422/original.jpg	https://data.globe.gov/system/photos/2019/08/27/1181421/original.jpg	https://data.globe.gov/system/photos/2019/08/27/1181423/original.jpg	https://data.globe.gov/system/photos/2019/08/27/1181425/original.jpg
GV_2118	45.6114	-122.6116	8.7		https://www.google.com/maps/search/45.6114,-122.6116	https://data.globe.gov/system/photos/2019/08/27/1181426/original.jpg	https://data.globe.gov/system/photos/2019/08/27/1181428/original.jpg	https://data.globe.gov/system/photos/2019/08/27/1181427/original.jpg	https://data.globe.gov/system/photos/2019/08/27/1181429/original.jpg	https://data.globe.gov/system/photos/2019/08/27/1181431/original.jpg
GV_2119	45.611	-122.6121	6		https://www.google.com/maps/search/45.611,-122.6121	https://data.globe.gov/system/photos/2019/08/27/1181432/original.jpg	https://data.globe.gov/system/photos/2019/08/27/1181434/original.jpg	https://data.globe.gov/system/photos/2019/08/27/1181433/original.jpg	https://data.globe.gov/system/photos/2019/08/27/1181435/original.jpg	https://data.globe.gov/system/photos/2019/08/27/1181437/original.jpg
GV_2120	45.6118	-122.6062	12.4		https://www.google.com/maps/search/45.6118,-122.6062	https://data.globe.gov/system/photos/2019/08/27/1181438/original.jpg	https://data.globe.gov/system/photos/2019/08/27/1181440/original.jpg	https://data.globe.gov/system/photos/2019/08/27/1181439/original.jpg	https://data.globe.gov/system/photos/2019/08/27/1181442/original.jpg	https://data.globe.gov/system/photos/2019/08/27/1181444/original.jpg
GV_2121	45.6242	-122.6571	14.2		https://www.google.com/maps/search/45.6242,-122.6571	https://data.globe.gov/system/photos/2019/08/27/1181445/original.jpg	https://data.globe.gov/system/photos/2019/08/27/1181447/original.jpg	https://data.globe.gov/system/photos/2019/08/27/1181446/original.jpg	https://data.globe.gov/system/photos/2019/08/27/1181448/original.jpg	https://data.globe.gov/system/photos/2019/08/27/1181451/original.jpg
GV_2122	45.6245	-122.6599	15.2		https://www.google.com/maps/search/45.6245,-122.6599	https://data.globe.gov/system/photos/2019/08/27/1181452/original.jpg	https://data.globe.gov/system/photos/2019/08/27/1181454/original.jpg	https://data.globe.gov/system/photos/2019/08/27/1181453/original.jpg	https://data.globe.gov/system/photos/2019/08/27/1181456/original.jpg	https://data.globe.gov/system/photos/2019/08/27/1181458/original.jpg
GV_2123	45.6241	-122.6617	13.5		https://www.google.com/maps/search/45.6241,-122.6617	https://data.globe.gov/system/photos/2019/08/27/1181459/original.jpg	https://data.globe.gov/system/photos/2019/08/27/1181461/original.jpg	https://data.globe.gov/system/photos/2019/08/27/1181460/original.jpg	https://data.globe.gov/system/photos/2019/08/27/1181462/original.jpg	https://data.globe.gov/system/photos/2019/08/27/1181464/original.jpg
GV_2124	45.6246	-122.6599	15.2		https://www.google.com/maps/search/45.6246,-122.6599	https://data.globe.gov/system/photos/2019/08/27/1181465/original.jpg	https://data.globe.gov/system/photos/2019/08/27/1181467/original.jpg	https://data.globe.gov/system/photos/2019/08/27/1181466/original.jpg	https://data.globe.gov/system/photos/2019/08/27/1181468/original.jpg	https://data.globe.gov/system/photos/2019/08/27/1181470/original.jpg
GV_2125	45.6248	-122.6584	16.8		https://www.google.com/maps/search/45.6248,-122.6584	https://data.globe.gov/system/photos/2019/08/27/1181472/original.jpg	https://data.globe.gov/system/photos/2019/08/27/1181474/original.jpg	https://data.globe.gov/system/photos/2019/08/27/1181473/original.jpg	https://data.globe.gov/system/photos/2019/08/27/1181475/original.jpg	https://data.globe.gov/system/photos/2019/08/27/1181477/original.jpg
GV_2126	45.6262	-122.6563	28.6		https://www.google.com/maps/search/45.6262,-122.6563	https://data.globe.gov/system/photos/2019/08/27/1181478/original.jpg	https://data.globe.gov/system/photos/2019/08/27/1181480/original.jpg	https://data.globe.gov/system/photos/2019/08/27/1181479/original.jpg	https://data.globe.gov/system/photos/2019/08/27/1181481/original.jpg	https://data.globe.gov/system/photos/2019/08/27/1181483/original.jpg
GV_2127	45.6274	-122.6605	31.1		https://www.google.com/maps/search/45.6274,-122.6605	https://data.globe.gov/system/photos/2019/08/27/1181484/original.jpg	https://data.globe.gov/system/photos/2019/08/27/1181486/original.jpg	https://data.globe.gov/system/photos/2019/08/27/1181485/original.jpg	https://data.globe.gov/system/photos/2019/08/27/1181487/original.jpg	https://data.globe.gov/system/photos/2019/08/27/1181489/original.jpg
GV_2128	45.6201	-122.6693	7.4		https://www.google.com/maps/search/45.6201,-122.6693	https://data.globe.gov/system/photos/2019/08/27/1181490/original.jpg	https://data.globe.gov/system/photos/2019/08/27/1181492/original.jpg	https://data.globe.gov/system/photos/2019/08/27/1181491/original.jpg	https://data.globe.gov/system/photos/2019/08/27/1181493/original.jpg	https://data.globe.gov/system/photos/2019/08/27/1181495/original.jpg
GV_2129	45.6206	-122.6708	8.4		https://www.google.com/maps/search/45.6206,-122.6708	https://data.globe.gov/system/photos/2019/08/27/1181496/original.jpg	https://data.globe.gov/system/photos/2019/08/27/1181498/original.jpg	https://data.globe.gov/system/photos/2019/08/27/1181497/original.jpg	https://data.globe.gov/system/photos/2019/08/27/1181499/original.jpg	https://data.globe.gov/system/photos/2019/08/27/1181502/original.jpg
GV_2130	45.6215	-122.6714	9.7		https://www.google.com/maps/search/45.6215,-122.6714	https://data.globe.gov/system/photos/2019/08/27/1181504/original.jpg	https://data.globe.gov/system/photos/2019/08/27/1181507/original.jpg	https://data.globe.gov/system/photos/2019/08/27/1181505/original.jpg	https://data.globe.gov/system/photos/2019/08/27/1181509/original.jpg	https://data.globe.gov/system/photos/2019/08/27/1181512/original.jpg
GV_2131	45.6212	-122.6724	5.1		https://www.google.com/maps/search/45.6212,-122.6724	https://data.globe.gov/system/photos/2019/08/27/1181513/original.jpg	https://data.globe.gov/system/photos/2019/08/27/1181516/original.jpg	https://data.globe.gov/system/photos/2019/08/27/1181515/original.jpg	https://data.globe.gov/system/photos/2019/08/27/1181517/original.jpg	https://data.globe.gov/system/photos/2019/08/27/1181519/original.jpg
GV_2132	45.6218	-122.6732	13.4		https://www.google.com/maps/search/45.6218,-122.6732	https://data.globe.gov/system/photos/2019/08/27/1181520/original.jpg	https://data.globe.gov/system/photos/2019/08/27/1181522/original.jpg	https://data.globe.gov/system/photos/2019/08/27/1181521/original.jpg	https://data.globe.gov/system/photos/2019/08/27/1181523/original.jpg	https://data.globe.gov/system/photos/2019/08/27/1181525/original.jpg
GV_2133	45.6195	-122.6746	3.4		https://www.google.com/maps/search/45.6195,-122.6746	https://data.globe.gov/system/photos/2019/08/27/1181526/original.jpg	https://data.globe.gov/system/photos/2019/08/27/1181528/original.jpg	https://data.globe.gov/system/photos/2019/08/27/1181527/original.jpg	https://data.globe.gov/system/photos/2019/08/27/1181529/original.jpg	https://data.globe.gov/system/photos/2019/08/27/1181531/original.jpg
GV_2134	45.6178	-122.6756	3.4		https://www.google.com/maps/search/45.6178,-122.6756	https://data.globe.gov/system/photos/2019/08/27/1181532/original.jpg	https://data.globe.gov/system/photos/2019/08/27/1181534/original.jpg	https://data.globe.gov/system/photos/2019/08/27/1181533/original.jpg	https://data.globe.gov/system/photos/2019/08/27/1181535/original.jpg	https://data.globe.gov/system/photos/2019/08/27/1181537/original.jpg
GV_2135	45.6125	-122.634	4.4		https://www.google.com/maps/search/45.6125,-122.634	https://data.globe.gov/system/photos/2019/08/27/1181538/original.jpg	https://data.globe.gov/system/photos/2019/08/27/1181540/original.jpg	https://data.globe.gov/system/photos/2019/08/27/1181539/original.jpg	https://data.globe.gov/system/photos/2019/08/27/1181541/original.jpg	https://data.globe.gov/system/photos/2019/08/27/1181543/original.jpg
GV_2136	45.6146	-122.6273	9.3		https://www.google.com/maps/search/45.6146,-122.6273	https://data.globe.gov/system/photos/2019/08/27/1181544/original.jpg	https://data.globe.gov/system/photos/2019/08/27/1181546/original.jpg	https://data.globe.gov/system/photos/2019/08/27/1181545/original.jpg	https://data.globe.gov/system/photos/2019/08/27/1181547/original.jpg	https://data.globe.gov/system/photos/2019/08/27/1181549/original.jpg
GV_2137	-27.126	117.4619	499.7		https://www.google.com/maps/search/-27.126,117.4619	https://data.globe.gov/system/photos/2019/08/26/1183582/original.jpg	https://data.globe.gov/system/photos/2019/08/26/1183584/original.jpg	https://data.globe.gov/system/photos/2019/08/26/1183583/original.jpg	https://data.globe.gov/system/photos/2019/08/26/1183585/original.jpg	https://data.globe.gov/system/photos/2019/08/26/1183587/original.jpg
GV_2138	-27.1018	116.3504	323.7		https://www.google.com/maps/search/-27.1018,116.3504	https://data.globe.gov/system/photos/2019/08/28/1183588/original.jpg	https://data.globe.gov/system/photos/2019/08/28/1183590/original.jpg	https://data.globe.gov/system/photos/2019/08/28/1183589/original.jpg	https://data.globe.gov/system/photos/2019/08/28/1183591/original.jpg	https://data.globe.gov/system/photos/2019/08/28/1183593/original.jpg
GV_2139	40.0051	-75.0442	4.1		https://www.google.com/maps/search/40.0051,-75.0442	https://data.globe.gov/system/photos/2019/08/29/1183959/original.jpg	https://data.globe.gov/system/photos/2019/08/29/1183963/original.jpg	https://data.globe.gov/system/photos/2019/08/29/1183962/original.jpg	https://data.globe.gov/system/photos/2019/08/29/1183964/original.jpg	https://data.globe.gov/system/photos/2019/08/29/1183966/original.jpg
GV_2140	53.4287	-0.6181	30.9		https://www.google.com/maps/search/53.4287,-0.6181	https://data.globe.gov/system/photos/2019/08/28/1185686/original.jpg	https://data.globe.gov/system/photos/2019/08/28/1185688/original.jpg	https://data.globe.gov/system/photos/2019/08/28/1185687/original.jpg	https://data.globe.gov/system/photos/2019/08/28/1185689/original.jpg	https://data.globe.gov/system/photos/2019/08/28/1185691/original.jpg
GV_2141	53.4283	-0.6227	26		https://www.google.com/maps/search/53.4283,-0.6227	https://data.globe.gov/system/photos/2019/08/30/1185692/original.jpg	https://data.globe.gov/system/photos/2019/08/30/1185694/original.jpg	https://data.globe.gov/system/photos/2019/08/30/1185693/original.jpg	https://data.globe.gov/system/photos/2019/08/30/1185695/original.jpg	https://data.globe.gov/system/photos/2019/08/30/1185698/original.jpg
GV_2142	42.771	-71.1412	78.8		https://www.google.com/maps/search/42.771,-71.1412	https://data.globe.gov/system/photos/2019/08/30/1186039/original.jpg	https://data.globe.gov/system/photos/2019/08/30/1186041/original.jpg	https://data.globe.gov/system/photos/2019/08/30/1186040/original.jpg	https://data.globe.gov/system/photos/2019/08/30/1186042/original.jpg	https://data.globe.gov/system/photos/2019/08/30/1186044/original.jpg
GV_2143	47.5382	-120.7082	671.8		https://www.google.com/maps/search/47.5382,-120.7082	https://data.globe.gov/system/photos/2019/08/26/1186198/original.jpg	https://data.globe.gov/system/photos/2019/08/26/1186200/original.jpg	https://data.globe.gov/system/photos/2019/08/26/1186199/original.jpg	https://data.globe.gov/system/photos/2019/08/26/1186201/original.jpg	https://data.globe.gov/system/photos/2019/08/26/1186203/original.jpg
GV_2144	47.5382	-120.7082	671.8		https://www.google.com/maps/search/47.5382,-120.7082	https://data.globe.gov/system/photos/2019/08/26/1186248/original.jpg	https://data.globe.gov/system/photos/2019/08/26/1186250/original.jpg	https://data.globe.gov/system/photos/2019/08/26/1186249/original.jpg	https://data.globe.gov/system/photos/2019/08/26/1186251/original.jpg	https://data.globe.gov/system/photos/2019/08/26/1186253/original.jpg
GV_2145	47.4978	-120.7343	1508.8		https://www.google.com/maps/search/47.4978,-120.7343	https://data.globe.gov/system/photos/2019/08/27/1186265/original.jpg	https://data.globe.gov/system/photos/2019/08/27/1186267/original.jpg	https://data.globe.gov/system/photos/2019/08/27/1186266/original.jpg	https://data.globe.gov/system/photos/2019/08/27/1186269/original.jpg	https://data.globe.gov/system/photos/2019/08/27/1186271/original.jpg
GV_2146	47.4978	-120.7343	1508.8		https://www.google.com/maps/search/47.4978,-120.7343	https://data.globe.gov/system/photos/2019/08/27/1186272/original.jpg	https://data.globe.gov/system/photos/2019/08/27/1186274/original.jpg	https://data.globe.gov/system/photos/2019/08/27/1186273/original.jpg	https://data.globe.gov/system/photos/2019/08/27/1186275/original.jpg	https://data.globe.gov/system/photos/2019/08/27/1186277/original.jpg
GV_2147	47.4855	-120.7492	1655.8		https://www.google.com/maps/search/47.4855,-120.7492	https://data.globe.gov/system/photos/2019/08/27/1186278/original.jpg	https://data.globe.gov/system/photos/2019/08/27/1186280/original.jpg	https://data.globe.gov/system/photos/2019/08/27/1186279/original.jpg	https://data.globe.gov/system/photos/2019/08/27/1186281/original.jpg	https://data.globe.gov/system/photos/2019/08/27/1186283/original.jpg
GV_2148	47.4806	-120.7846	2102.3		https://www.google.com/maps/search/47.4806,-120.7846	https://data.globe.gov/system/photos/2019/08/28/1186284/original.jpg	https://data.globe.gov/system/photos/2019/08/28/1186286/original.jpg	https://data.globe.gov/system/photos/2019/08/28/1186285/original.jpg	https://data.globe.gov/system/photos/2019/08/28/1186287/original.jpg	https://data.globe.gov/system/photos/2019/08/28/1186289/original.jpg
GV_2149	47.4806	-120.7846	2102.3		https://www.google.com/maps/search/47.4806,-120.7846	https://data.globe.gov/system/photos/2019/08/28/1186290/original.jpg	https://data.globe.gov/system/photos/2019/08/28/1186292/original.jpg	https://data.globe.gov/system/photos/2019/08/28/1186291/original.jpg	https://data.globe.gov/system/photos/2019/08/28/1186293/original.jpg	https://data.globe.gov/system/photos/2019/08/28/1186295/original.jpg
GV_2150	47.4756	-120.8014	2148.2		https://www.google.com/maps/search/47.4756,-120.8014	https://data.globe.gov/system/photos/2019/08/28/1186296/original.jpg	https://data.globe.gov/system/photos/2019/08/28/1186298/original.jpg	https://data.globe.gov/system/photos/2019/08/28/1186297/original.jpg	https://data.globe.gov/system/photos/2019/08/28/1186299/original.jpg	https://data.globe.gov/system/photos/2019/08/28/1186301/original.jpg
GV_2151	47.4756	-120.8014	2148.2		https://www.google.com/maps/search/47.4756,-120.8014	https://data.globe.gov/system/photos/2019/08/28/1186302/original.jpg	https://data.globe.gov/system/photos/2019/08/28/1186304/original.jpg	https://data.globe.gov/system/photos/2019/08/28/1186303/original.jpg	https://data.globe.gov/system/photos/2019/08/28/1186305/original.jpg	https://data.globe.gov/system/photos/2019/08/28/1186307/original.jpg
GV_2152	47.478	-120.8053	2287.8		https://www.google.com/maps/search/47.478,-120.8053	https://data.globe.gov/system/photos/2019/08/28/1186308/original.jpg	https://data.globe.gov/system/photos/2019/08/28/1186310/original.jpg	https://data.globe.gov/system/photos/2019/08/28/1186309/original.jpg	https://data.globe.gov/system/photos/2019/08/28/1186311/original.jpg	https://data.globe.gov/system/photos/2019/08/28/1186313/original.jpg
GV_2153	47.478	-120.8053	2287.8		https://www.google.com/maps/search/47.478,-120.8053	https://data.globe.gov/system/photos/2019/08/28/1186314/original.jpg	https://data.globe.gov/system/photos/2019/08/28/1186316/original.jpg	https://data.globe.gov/system/photos/2019/08/28/1186315/original.jpg	https://data.globe.gov/system/photos/2019/08/28/1186317/original.jpg	https://data.globe.gov/system/photos/2019/08/28/1186319/original.jpg

GV_2154	47.4801	-120.8186	2371.5		https://www.google.com/maps/search/47.4801,-120.8186	https://data.globe.gov/system/photos/2019/08/28/1186320/original.jpg	https://data.globe.gov/system/photos/2019/08/28/1186322/original.jpg	https://data.globe.gov/system/photos/2019/08/28/1186321/original.jpg	https://data.globe.gov/system/photos/2019/08/28/1186323/original.jpg	https://data.globe.gov/system/photos/2019/08/28/1186325/original.jpg
GV_2155	47.4801	-120.8186	2371.5		https://www.google.com/maps/search/47.4801,-120.8186	https://data.globe.gov/system/photos/2019/08/28/1186326/original.jpg	https://data.globe.gov/system/photos/2019/08/28/1186328/original.jpg	https://data.globe.gov/system/photos/2019/08/28/1186327/original.jpg	https://data.globe.gov/system/photos/2019/08/28/1186329/original.jpg	https://data.globe.gov/system/photos/2019/08/28/1186331/original.jpg
GV_2156	45.6059	-121.1864	32.1		https://www.google.com/maps/search/45.6059,-121.1864	https://data.globe.gov/system/photos/2019/08/30/1186332/original.jpg	https://data.globe.gov/system/photos/2019/08/30/1186334/original.jpg	https://data.globe.gov/system/photos/2019/08/30/1186333/original.jpg	https://data.globe.gov/system/photos/2019/08/30/1186335/original.jpg	https://data.globe.gov/system/photos/2019/08/30/1186337/original.jpg
GV_2157	45.6066	-121.1873	32.8		https://www.google.com/maps/search/45.6066,-121.1873	https://data.globe.gov/system/photos/2019/08/30/1186338/original.jpg	https://data.globe.gov/system/photos/2019/08/30/1186340/original.jpg	https://data.globe.gov/system/photos/2019/08/30/1186339/original.jpg	https://data.globe.gov/system/photos/2019/08/30/1186341/original.jpg	https://data.globe.gov/system/photos/2019/08/30/1186343/original.jpg
GV_2158	45.6067	-121.1873	29.5		https://www.google.com/maps/search/45.6067,-121.1873	https://data.globe.gov/system/photos/2019/08/30/1186344/original.jpg	https://data.globe.gov/system/photos/2019/08/30/1186346/original.jpg	https://data.globe.gov/system/photos/2019/08/30/1186345/original.jpg	https://data.globe.gov/system/photos/2019/08/30/1186347/original.jpg	https://data.globe.gov/system/photos/2019/08/30/1186349/original.jpg
GV_2159	45.6068	-121.1878	34.7		https://www.google.com/maps/search/45.6068,-121.1878	https://data.globe.gov/system/photos/2019/08/30/1186351/original.jpg	https://data.globe.gov/system/photos/2019/08/30/1186353/original.jpg	https://data.globe.gov/system/photos/2019/08/30/1186352/original.jpg	https://data.globe.gov/system/photos/2019/08/30/1186354/original.jpg	https://data.globe.gov/system/photos/2019/08/30/1186356/original.jpg
GV_2160	45.6069	-121.1878	34.6		https://www.google.com/maps/search/45.6069,-121.1878	https://data.globe.gov/system/photos/2019/08/31/1186357/original.jpg	https://data.globe.gov/system/photos/2019/08/31/1186359/original.jpg	https://data.globe.gov/system/photos/2019/08/31/1186358/original.jpg	https://data.globe.gov/system/photos/2019/08/31/1186360/original.jpg	https://data.globe.gov/system/photos/2019/08/31/1186362/original.jpg
GV_2161	45.6068	-121.1879	34.6		https://www.google.com/maps/search/45.6068,-121.1879	https://data.globe.gov/system/photos/2019/08/31/1186363/original.jpg	https://data.globe.gov/system/photos/2019/08/31/1186365/original.jpg	https://data.globe.gov/system/photos/2019/08/31/1186364/original.jpg	https://data.globe.gov/system/photos/2019/08/31/1186366/original.jpg	https://data.globe.gov/system/photos/2019/08/31/1186368/original.jpg
GV_2162	-26.0911	113.5795	30.9		https://www.google.com/maps/search/-26.0911,113.5795	https://data.globe.gov/system/photos/2019/08/31/1186405/original.jpg	https://data.globe.gov/system/photos/2019/08/31/1186407/original.jpg	https://data.globe.gov/system/photos/2019/08/31/1186406/original.jpg	https://data.globe.gov/system/photos/2019/08/31/1186408/original.jpg	https://data.globe.gov/system/photos/2019/08/31/1186410/original.jpg
GV_2163	-34.5454	138.6906	54.4		https://www.google.com/maps/search/-34.5454,138.6906	https://data.globe.gov/system/photos/2019/08/31/1186418/original.jpg	https://data.globe.gov/system/photos/2019/08/31/1186420/original.jpg	https://data.globe.gov/system/photos/2019/08/31/1186419/original.jpg	https://data.globe.gov/system/photos/2019/08/31/1186421/original.jpg	https://data.globe.gov/system/photos/2019/08/31/1186423/original.jpg
GV_2164	-34.4496	138.5341	46.1		https://www.google.com/maps/search/-34.4496,138.5341	https://data.globe.gov/system/photos/2019/08/31/1186429/original.jpg	https://data.globe.gov/system/photos/2019/08/31/1186432/original.jpg	https://data.globe.gov/system/photos/2019/08/31/1186430/original.jpg	https://data.globe.gov/system/photos/2019/08/31/1186433/original.jpg	https://data.globe.gov/system/photos/2019/08/31/1186436/original.jpg
GV_2165	24.4358	118.3142	21.7		https://www.google.com/maps/search/24.4358,118.3142	https://data.globe.gov/system/photos/2019/08/31/1186441/original.jpg	https://data.globe.gov/system/photos/2019/08/31/1186443/original.jpg	https://data.globe.gov/system/photos/2019/08/31/1186442/original.jpg	https://data.globe.gov/system/photos/2019/08/31/1186444/original.jpg	https://data.globe.gov/system/photos/2019/08/31/1186446/original.jpg
GV_2166	42.9132	-70.8093	4.5		https://www.google.com/maps/search/42.9132,-70.8093	https://data.globe.gov/system/photos/2019/08/31/1186563/original.jpg	https://data.globe.gov/system/photos/2019/08/31/1186565/original.jpg	https://data.globe.gov/system/photos/2019/08/31/1186564/original.jpg	https://data.globe.gov/system/photos/2019/08/31/1186566/original.jpg	https://data.globe.gov/system/photos/2019/08/31/1186568/original.jpg
GV_2167	42.8848	-71.0678	35.1		https://www.google.com/maps/search/42.8848,-71.0678	https://data.globe.gov/system/photos/2019/08/31/1186686/original.jpg	https://data.globe.gov/system/photos/2019/08/31/1186688/original.jpg	https://data.globe.gov/system/photos/2019/08/31/1186687/original.jpg	https://data.globe.gov/system/photos/2019/08/31/1186689/original.jpg	https://data.globe.gov/system/photos/2019/08/31/1186691/original.jpg
GV_2168	53.4284	-0.6226	26		https://www.google.com/maps/search/53.4284,-0.6226	https://data.globe.gov/system/photos/2019/08/31/1186930/original.jpg	https://data.globe.gov/system/photos/2019/08/31/1186932/original.jpg	https://data.globe.gov/system/photos/2019/08/31/1186931/original.jpg	https://data.globe.gov/system/photos/2019/08/31/1186933/original.jpg	https://data.globe.gov/system/photos/2019/08/31/1186935/original.jpg
GV_2169	-33.928	137.6209	9.8		https://www.google.com/maps/search/-33.928,137.6209	https://data.globe.gov/system/photos/2019/09/01/1187070/original.jpg	https://data.globe.gov/system/photos/2019/09/01/1187072/original.jpg	https://data.globe.gov/system/photos/2019/09/01/1187071/original.jpg	https://data.globe.gov/system/photos/2019/09/01/1187073/original.jpg	https://data.globe.gov/system/photos/2019/09/01/1187075/original.jpg
GV_2170	-33.9261	137.6183	0.1		https://www.google.com/maps/search/-33.9261,137.6183	https://data.globe.gov/system/photos/2019/09/01/1187076/original.jpg	https://data.globe.gov/system/photos/2019/09/01/1187078/original.jpg	https://data.globe.gov/system/photos/2019/09/01/1187077/original.jpg	https://data.globe.gov/system/photos/2019/09/01/1187079/original.jpg	https://data.globe.gov/system/photos/2019/09/01/1187081/original.jpg
GV_2171	-33.9941	137.7301	49.5		https://www.google.com/maps/search/-33.9941,137.7301	https://data.globe.gov/system/photos/2019/09/01/1187208/original.jpg	https://data.globe.gov/system/photos/2019/09/01/1187210/original.jpg	https://data.globe.gov/system/photos/2019/09/01/1187209/original.jpg	https://data.globe.gov/system/photos/2019/09/01/1187211/original.jpg	https://data.globe.gov/system/photos/2019/09/01/1187213/original.jpg
GV_2172	-34.0405	137.9154	137.3		https://www.google.com/maps/search/-34.0405,137.9154	https://data.globe.gov/system/photos/2019/09/01/1187214/original.jpg	https://data.globe.gov/system/photos/2019/09/01/1187216/original.jpg	https://data.globe.gov/system/photos/2019/09/01/1187215/original.jpg	https://data.globe.gov/system/photos/2019/09/01/1187217/original.jpg	https://data.globe.gov/system/photos/2019/09/01/1187219/original.jpg
GV_2173	42.7716	-71.1336	7.5		https://www.google.com/maps/search/42.7716,-71.1336	https://data.globe.gov/system/photos/2019/09/01/1187490/original.jpg	https://data.globe.gov/system/photos/2019/09/01/1187492/original.jpg	https://data.globe.gov/system/photos/2019/09/01/1187491/original.jpg	https://data.globe.gov/system/photos/2019/09/01/1187493/original.jpg	https://data.globe.gov/system/photos/2019/09/01/1187495/original.jpg
GV_2174	42.732	-71.1758	53.1		https://www.google.com/maps/search/42.732,-71.1758	https://data.globe.gov/system/photos/2019/09/01/1187496/original.jpg	https://data.globe.gov/system/photos/2019/09/01/1187498/original.jpg	https://data.globe.gov/system/photos/2019/09/01/1187497/original.jpg	https://data.globe.gov/system/photos/2019/09/01/1187499/original.jpg	https://data.globe.gov/system/photos/2019/09/01/1187501/original.jpg
GV_2175	42.6975	-71.1073	60.6		https://www.google.com/maps/search/42.6975,-71.1073	https://data.globe.gov/system/photos/2019/09/01/1187504/original.jpg	https://data.globe.gov/system/photos/2019/09/01/1187509/original.jpg	https://data.globe.gov/system/photos/2019/09/01/1187508/original.jpg	https://data.globe.gov/system/photos/2019/09/01/1187510/original.jpg	https://data.globe.gov/system/photos/2019/09/01/1187512/original.jpg
GV_2176	52.0248	-109.4512	685		https://www.google.com/maps/search/52.0248,-109.4512	https://data.globe.gov/system/photos/2019/08/31/1187554/original.jpg	https://data.globe.gov/system/photos/2019/08/31/1187556/original.jpg	https://data.globe.gov/system/photos/2019/08/31/1187555/original.jpg	https://data.globe.gov/system/photos/2019/08/31/1187557/original.jpg	https://data.globe.gov/system/photos/2019/08/31/1187559/original.jpg
GV_2177	47.143	-91.468	209.3		https://www.google.com/maps/search/47.143,-91.468	https://data.globe.gov/system/photos/2019/09/01/1188001/original.jpg	https://data.globe.gov/system/photos/2019/09/01/1188003/original.jpg	https://data.globe.gov/system/photos/2019/09/01/1188002/original.jpg	https://data.globe.gov/system/photos/2019/09/01/1188004/original.jpg	https://data.globe.gov/system/photos/2019/09/01/1188006/original.jpg
GV_2178	24.4358	118.3144	21.8		https://www.google.com/maps/search/24.4358,118.3144	https://data.globe.gov/system/photos/2019/09/02/1188203/original.jpg	https://data.globe.gov/system/photos/2019/09/02/1188206/original.jpg	https://data.globe.gov/system/photos/2019/09/02/1188205/original.jpg	https://data.globe.gov/system/photos/2019/09/02/1188209/original.jpg	https://data.globe.gov/system/photos/2019/09/02/1188232/original.jpg
GV_2179	24.4356	118.3139	22		https://www.google.com/maps/search/24.4356,118.3139	https://data.globe.gov/system/photos/2019/09/02/1188245/original.jpg	https://data.globe.gov/system/photos/2019/09/02/1188247/original.jpg	https://data.globe.gov/system/photos/2019/09/02/1188246/original.jpg	https://data.globe.gov/system/photos/2019/09/02/1188248/original.jpg	https://data.globe.gov/system/photos/2019/09/02/1188250/original.jpg
GV_2180	42.0785	-83.2247	178.1		https://www.google.com/maps/search/42.0785,-83.2247	https://data.globe.gov/system/photos/2019/09/02/1188600/original.jpg	https://data.globe.gov/system/photos/2019/09/02/1188602/original.jpg	https://data.globe.gov/system/photos/2019/09/02/1188601/original.jpg	https://data.globe.gov/system/photos/2019/09/02/1188603/original.jpg	https://data.globe.gov/system/photos/2019/09/02/1188605/original.jpg
GV_2181	42.0785	-83.2246	178.6		https://www.google.com/maps/search/42.0785,-83.2246	https://data.globe.gov/system/photos/2019/09/02/1188606/original.jpg	https://data.globe.gov/system/photos/2019/09/02/1188608/original.jpg	https://data.globe.gov/system/photos/2019/09/02/1188607/original.jpg	https://data.globe.gov/system/photos/2019/09/02/1188609/original.jpg	https://data.globe.gov/system/photos/2019/09/02/1188611/original.jpg
GV_2182	38.9934	-76.8385	64.6		https://www.google.com/maps/search/38.9934,-76.8385	https://data.globe.gov/system/photos/2019/08/24/1189467/original.jpg	https://data.globe.gov/system/photos/2019/08/24/1189469/original.jpg	https://data.globe.gov/system/photos/2019/08/24/1189468/original.jpg	https://data.globe.gov/system/photos/2019/08/24/1189470/original.jpg	https://data.globe.gov/system/photos/2019/08/24/1189472/original.jpg
GV_2183	38.9929	-76.8398	62.8		https://www.google.com/maps/search/38.9929,-76.8398	https://data.globe.gov/system/photos/2019/08/24/1189473/original.jpg	https://data.globe.gov/system/photos/2019/08/24/1189475/original.jpg	https://data.globe.gov/system/photos/2019/08/24/1189474/original.jpg	https://data.globe.gov/system/photos/2019/08/24/1189476/original.jpg	https://data.globe.gov/system/photos/2019/08/24/1189478/original.jpg
GV_2184	-34.1665	138.1795	13.8		https://www.google.com/maps/search/-34.1665,138.1795	https://data.globe.gov/system/photos/2019/09/01/1189563/original.jpg	https://data.globe.gov/system/photos/2019/09/01/1189565/original.jpg	https://data.globe.gov/system/photos/2019/09/01/1189564/original.jpg	https://data.globe.gov/system/photos/2019/09/01/1189566/original.jpg	https://data.globe.gov/system/photos/2019/09/01/1189568/original.jpg
GV_2185	46.2781	-124.054	61.5		https://www.google.com/maps/search/46.2781,-124.054	https://data.globe.gov/system/photos/2019/09/02/1189624/original.jpg	https://data.globe.gov/system/photos/2019/09/02/1189626/original.jpg	https://data.globe.gov/system/photos/2019/09/02/1189625/original.jpg	https://data.globe.gov/system/photos/2019/09/02/1189627/original.jpg	https://data.globe.gov/system/photos/2019/09/02/1189629/original.jpg
GV_2186	46.278	-124.0539	62.3		https://www.google.com/maps/search/46.278,-124.0539	https://data.globe.gov/system/photos/2019/09/02/1189641/original.jpg	https://data.globe.gov/system/photos/2019/09/02/1189644/original.jpg	https://data.globe.gov/system/photos/2019/09/02/1189642/original.jpg	https://data.globe.gov/system/photos/2019/09/02/1189645/original.jpg	https://data.globe.gov/system/photos/2019/09/02/1189647/original.jpg
GV_2187	46.226	-124.0129	8.3		https://www.google.com/maps/search/46.226,-124.0129	https://data.globe.gov/system/photos/2019/09/03/1189656/original.jpg	https://data.globe.gov/system/photos/2019/09/03/1189658/original.jpg	https://data.globe.gov/system/photos/2019/09/03/1189657/original.jpg	https://data.globe.gov/system/photos/2019/09/03/1189659/original.jpg	https://data.globe.gov/system/photos/2019/09/03/1189661/original.jpg
GV_2188	46.2271	-124.0137	5.6		https://www.google.com/maps/search/46.2271,-124.0137	https://data.globe.gov/system/photos/2019/09/03/1189662/original.jpg	https://data.globe.gov/system/photos/2019/09/03/1189664/original.jpg	https://data.globe.gov/system/photos/2019/09/03/1189663/original.jpg	https://data.globe.gov/system/photos/2019/09/03/1189665/original.jpg	https://data.globe.gov/system/photos/2019/09/03/1189667/original.jpg
GV_2189	46.2273	-124.0152	4.6		https://www.google.com/maps/search/46.2273,-124.0152	https://data.globe.gov/system/photos/2019/09/03/1189668/original.jpg	https://data.globe.gov/system/photos/2019/09/03/1189670/original.jpg	https://data.globe.gov/system/photos/2019/09/03/1189669/original.jpg	https://data.globe.gov/system/photos/2019/09/03/1189671/original.jpg	https://data.globe.gov/system/photos/2019/09/03/1189673/original.jpg
GV_2190	46.2276	-124.0174	3.6		https://www.google.com/maps/search/46.2276,-124.0174	https://data.globe.gov/system/photos/2019/09/03/1189674/original.jpg	https://data.globe.gov/system/photos/2019/09/03/1189676/original.jpg	https://data.globe.gov/system/photos/2019/09/03/1189675/original.jpg	https://data.globe.gov/system/photos/2019/09/03/1189677/original.jpg	https://data.globe.gov/system/photos/2019/09/03/1189679/original.jpg
GV_2191	46.2786	-124.0529	38.6		https://www.google.com/maps/search/46.2786,-124.0529	https://data.globe.gov/system/photos/2019/09/02/1189689/original.jpg	https://data.globe.gov/system/photos/2019/09/02/1189691/original.jpg	https://data.globe.gov/system/photos/2019/09/02/1189690/original.jpg	https://data.globe.gov/system/photos/2019/09/02/1189692/original.jpg	https://data.globe.gov/system/photos/2019/09/02/1189694/original.jpg
GV_2192	46.2786	-124.053	38.5		https://www.google.com/maps/search/46.2786,-124.053	https://data.globe.gov/system/photos/2019/09/02/1189695/original.jpg	https://data.globe.gov/system/photos/2019/09/02/1189697/original.jpg	https://data.globe.gov/system/photos/2019/09/02/1189696/original.jpg	https://data.globe.gov/system/photos/2019/09/02/1189698/original.jpg	https://data.globe.gov/system/photos/2019/09/02/1189700/original.jpg
GV_2193	46.2782	-124.0537	59.1		https://www.google.com/maps/search/46.2782,-124.0537	https://data.globe.gov/system/photos/2019/09/02/1189701/original.jpg	https://data.globe.gov/system/photos/2019/09/02/1189703/original.jpg	https://data.globe.gov/system/photos/2019/09/02/1189702/original.jpg	https://data.globe.gov/system/photos/2019/09/02/1189704/original.jpg	https://data.globe.gov/system/photos/2019/09/02/1189706/original.jpg
GV_2194	46.1788	-123.9579	7.7		https://www.google.com/maps/search/46.1788,-123.9579	https://data.globe.gov/system/photos/2019/09/03/1189707/original.jpg	https://data.globe.gov/system/photos/2019/09/03/1189709/original.jpg	https://data.globe.gov/system/photos/2019/09/03/1189708/original.jpg	https://data.globe.gov/system/photos/2019/09/03/1189710/original.jpg	https://data.globe.gov/system/photos/2019/09/03/1189712/original.jpg
GV_2195	46.2274	-124.0172	3.4		https://www.google.com/maps/search/46.2274,-124.0172	https://data.globe.gov/system/photos/2019/09/03/1189736/original.jpg	https://data.globe.gov/system/photos/2019/09/03/1189738/original.jpg	https://data.globe.gov/system/photos/2019/09/03/1189737/original.jpg	https://data.globe.gov/system/photos/2019/09/03/1189739/original.jpg	https://data.globe.gov/system/photos/2019/09/03/1189741/original.jpg
GV_2196	46.2249	-123.9871	1.4		https://www.google.com/maps/search/46.2249,-123.9871	https://data.globe.gov/system/photos/2019/09/03/1189742/original.jpg	https://data.globe.gov/system/photos/2019/09/03/1189744/original.jpg	https://data.globe.gov/system/photos/2019/09/03/1189743/original.jpg	https://data.globe.gov/system/photos/2019/09/03/1189745/original.jpg	https://data.globe.gov/system/photos/2019/09/03/1189747/original.jpg
GV_2197	45.5111	-122.6815	40.1		https://www.google.com/maps/search/45.5111,-122.6815	https://data.globe.gov/system/photos/2019/09/02/1189780/original.jpg	https://data.globe.gov/system/photos/2019/09/02/1189782/original.jpg	https://data.globe.gov/system/photos/2019/09/02/1189781/original.jpg	https://data.globe.gov/system/photos/2019/09/02/1189783/original.jpg	https://data.globe.gov/system/photos/2019/09/02/1189785/original.jpg
GV_2198	45.5112	-122.6804	37.2		https://www.google.com/maps/search/45.5112,-122.6804	https://data.globe.gov/system/photos/2019/09/02/1189786/original.jpg	https://data.globe.gov/system/photos/2019/09/02/1189788/original.jpg	https://data.globe.gov/system/photos/2019/09/02/1189787/original.jpg	https://data.globe.gov/system/photos/2019/09/02/1189789/original.jpg	https://data.globe.gov/system/photos/2019/09/02/1189791/original.jpg
GV_2199	46.0492	-123.8512	17.4		https://www.google.com/maps/search/46.0492,-123.8512	https://data.globe.gov/system/photos/2019/09/02/1189792/original.jpg	https://data.globe.gov/system/photos/2019/09/02/1189794/original.jpg	https://data.globe.gov/system/photos/2019/09/02/1189793/original.jpg	https://data.globe.gov/system/photos/2019/09/02/1189795/original.jpg	https://data.globe.gov/system/photos/2019/09/02/1189797/original.jpg
GV_2200	46.0772	-123.8443	2.2		https://www.google.com/maps/search/46.0772,-123.8443	https://data.globe.gov/system/photos/2019/09/02/1189798/original.jpg	https://data.globe.gov/system/photos/2019/09/02/1189800/original.jpg	https://data.globe.gov/system/photos/2019/09/02/1189799/original.jpg	https://data.globe.gov/system/photos/2019/09/02/1189801/original.jpg	https://data.globe.gov/system/photos/2019/09/02/1189803/original.jpg
GV_2201	46.1035	-123.8534	4.6		https://www.google.com/maps/search/46.1035,-123.8534	https://data.globe.gov/system/photos/2019/09/02/1189804/original.jpg	https://data.globe.gov/system/photos/2019/09/02/1189806/original.jpg	https://data.globe.gov/system/photos/2019/09/02/1189805/original.jpg	https://data.globe.gov/system/photos/2019/09/02/1189807/original.jpg	https://data.globe.gov/system/photos/2019/09/02/1189809/original.jpg
GV_2202	46.1755	-123.8489	5.4		https://www.google.com/maps/search/46.1755,-123.8489	https://data.globe.gov/system/photos/2019/09/02/1189810/original.jpg	https://data.globe.gov/system/photos/2019/09/02/1189812/original.jpg	https://data.globe.gov/system/photos/2019/09/02/1189811/original.jpg	https://data.globe.gov/system/photos/2019/09/02/1189813/original.jpg	https://data.globe.gov/system/photos/2019/09/02/1189815/original.jpg
GV_2203	46.2788	-124.053	37.3		https://www.google.com/maps/search/46.2788,-124.053	https://data.globe.gov/system/photos/2019/09/02/1189816/original.jpg	https://data.globe.gov/system/photos/2019/09/02/1189818/original.jpg	https://data.globe.gov/system/photos/2019/09/02/1189817/original.jpg	https://data.globe.gov/system/photos/2019/09/02/1189819/original.jpg	https://data.globe.gov/system/photos/2019/09/02/1189821/original.jpg
GV_2204	46.279	-124.0537	51.6		https://www.google.com/maps/search/46.279,-124.0537	https://data.globe.gov/system/photos/2019/09/02/1189822/original.jpg	https://data.globe.gov/system/photos/2019/09/02/1189824/original.jpg	https://data.globe.gov/system/photos/2019/09/02/1189823/original.jpg	https://data.globe.gov/system/photos/2019/09/02/1189825/original.jpg	https://data.globe.gov/system/photos/2019/09/02/1189827/original.jpg
GV_2205	46.2782	-124.054	61.2		https://www.google.com/maps/search/46.2782,-124.054	https://data.globe.gov/system/photos/2019/09/02/1189828/original.jpg	https://data.globe.gov/system/photos/2019/09/02/1189830/original.jpg	https://data.globe.gov/system/photos/2019/09/02/1189829/original.jpg	https://data.globe.gov/system/photos/2019/09/02/1189831/original.jpg	https://data.globe.gov/system/photos/2019/09/02/1189833/original.jpg
GV_2206	46.278	-124.0539	62.3		https://www.google.com/maps/search/46.278,-124.0539	https://data.globe.gov/system/photos/2019/09/02/1189834/original.jpg	https://data.globe.gov/system/photos/2019/09/02/1189836/original.jpg	https://data.globe.gov/system/photos/2019/09/02/1189835/original.jpg	https://data.globe.gov/system/photos/2019/09/02/1189837/original.jpg	https://data.globe.gov/system/photos/2019/09/02/1189839/original.jpg
GV_2207	46.2777	-124.0537	60.3		https://www.google.com/maps/search/46.2777,-124.0537	https://data.globe.gov/system/photos/2019/09/02/1189840/original.jpg	https://data.globe.gov/system/photos/2019/09/02/1189842/original.jpg	https://data.globe.gov/system/photos/2019/09/02/1189841/original.jpg	https://data.globe.gov/system/photos/2019/09/02/1189843/original.jpg	https://data.globe.gov/system/photos/2019/09/02/1189845/original.jpg
GV_2208	53.4283	-0.6227	26		https://www.google.com/maps/search/53.4283,-0.6227	https://data.globe.gov/system/photos/2019/09/02/1189850/original.jpg	https://data.globe.gov/system/photos/2019/09/02/1189852/original.jpg	https://data.globe.gov/system/photos/2019/09/02/1189851/original.jpg	https://data.globe.gov/system/photos/2019/09/02/1189853/original.jpg	https://data.globe.gov/system/photos/2019/09/02/1189855/original.jpg
GV_2209	53.4284	-0.6226	26		https://www.google.com/maps/search/53.4284,-0.6226	https://data.globe.gov/system/photos/2019/09/03/1191024/original.jpg	https://data.globe.gov/system/photos/2019/09/03/1191026/original.jpg	https://data.globe.gov/system/photos/2019/09/03/1191025/original.jpg	https://data.globe.gov/system/photos/2019/09/03/1191027/original.jpg	https://data.globe.gov/system/photos/2019/09/03/1191029/original.jpg
GV_2210	61.6066	-149.1376	91.6		https://www.google.com/maps/search/61.6066,-149.1376	https://data.globe.gov/system/photos/2019/09/04/1191398/original.jpg	https://data.globe.gov/system/photos/2019/09/04/1191400/original.jpg	https://data.globe.gov/system/photos/2019/09/04/1191399/original.jpg	https://data.globe.gov/system/photos/2019/09/04/1191401/original.jpg	https://data.globe.gov/system/photos/2019/09/04/1191403/original.jpg
GV_2211	-27.5477	-48.4982	8.7		https://www.google.com/maps/search/-27.5477,-48.4982	https://data.globe.gov/system/photos/2019/09/04/1191531/original.jpg	https://data.globe.gov/system/photos/2019/09/04/1191533/original.jpg	https://data.globe.gov/system/photos/2019/09/04/1191532/original.jpg	https://data.globe.gov/system/photos/2019/09/04/1191534/original.jpg	https://data.globe.gov/system/photos/2019/09/04/1191536/original.jpg
GV_2212	-27.5479	-48.4981	8.7		https://www.google.com/maps/search/-27.5479,-48.4981	https://data.globe.gov/system/photos/2019/09/04/1191545/original.jpg	https://data.globe.gov/system/photos/2019/09/04/1191547/original.jpg	https://data.globe.gov/system/photos/2019/09/04/1191546/original.jpg	https://data.globe.gov/system/photos/2019/09/04/1191548/original.jpg	https://data.globe.gov/system/photos/2019/09/04/1191552/original.jpg
GV_2213	-27.5482	-48.4985	8.9		https://www.google.com/maps/search/-27.5482,-48.4985	https://data.globe.gov/system/photos/2019/09/04/1191671/original.jpg	https://data.globe.gov/system/photos/2019/09/04/1191673/original.jpg	https://data.globe.gov/system/photos/2019/09/04/1191672/original.jpg	https://data.globe.gov/system/photos/2019/09/04/1191675/original.jpg	https://data.globe.gov/system/photos/2019/09/04/1191680/original.jpg
GV_2214	-27.5478	-48.498	8.9		https://www.google.com/maps/search/-27.5478,-48.498	https://data.globe.gov/system/photos/2019/09/04/1191674/original.jpg	https://data.globe.gov/system/photos/2019/09/04/1191678/original.jpg	https://data.globe.gov/system/photos/2019/09/04/1191677/original.jpg	https://data.globe.gov/system/photos/2019/09/04/1191679/original.jpg	https://data.globe.gov/system/photos/2019/09/04/1191683/original.jpg
GV_2215	38.9931	-76.846	66.3		https://www.google.com/maps/search/38.9931,-76.846	https://data.globe.gov/system/photos/2019/09/04/1191876/original.jpg	https://data.globe.gov/system/photos/2019/09/04/1191878/original.jpg	https://data.globe.gov/system/photos/2019/09/04/1191877/original.jpg	https://data.globe.gov/system/photos/2019/09/04/1191879/original.jpg	https://data.globe.gov/system/photos/2019/09/04/1191881/original.jpg
GV_2216	61.6054	-149.1383	90.9		https://www.google.com/maps/search/61.6054,-149.1383	https://data.globe.gov/system/photos/2019/09/04/1191923/original.jpg	https://data.globe.gov/system/photos/2019/09/04/1191925/original.jpg	https://data.globe.gov/system/photos/2019/09/04/1191924/original.jpg	https://data.globe.gov/system/photos/2019/09/04/1191926/original.jpg	https://data.globe.gov/system/photos/2019/09/04/1191930/original.jpg
GV_2217	38.9928	-76.8551	55.3		https://www.google.com/maps/search/38.9928,-76.8551	https://data.globe.gov/system/photos/2019/09/04/1191928/original.jpg	https://data.globe.gov/system/photos/2019/09/04/1191931/original.jpg	https://data.globe.gov/system/photos/2019/09/04/1191929/original.jpg	https://data.globe.gov/system/photos/2019/09/04/1191932/original.jpg	https://data.globe.gov/system/photos/2019/09/04/1191934/original.jpg
GV_2218	61.6064	-149.138	91.3		https://www.google.com/maps/search/61.6064,-149.138	https://data.globe.gov/system/photos/2019/09/04/1191946/original.jpg	https://data.globe.gov/system/photos/2019/09/04/1191948/original.jpg	https://data.globe.gov/system/photos/2019/09/04/1191947/original.jpg	https://data.globe.gov/system/photos/2019/09/04/1191949/original.jpg	https://data.globe.gov/system/photos/2019/09/04/1191951/original.jpg
GV_2219	61.6027	-149.1357	81.8		https://www.google.com/maps/search/61.6027,-149.1357	https://data.globe.gov/system/photos/2019/09/04/1191954/original.jpg	https://data.globe.gov/system/photos/2019/09/04/1191956/original.jpg	https://data.globe.gov/system/photos/2019/09/04/1191955/original.jpg	https://data.globe.gov/system/photos/2019/09/04/1191957/original.jpg	https://data.globe.gov/system/photos/2019/09/04/1191959/original.jpg
GV_2220	53.4223	-0.6038	37.7		https://www.google.com/maps/search/53.4223,-0.6038	https://data.globe.gov/system/photos/2019/09/04/1191980/original.jpg	https://data.globe.gov/system/photos/2019/09/04/1191982/original.jpg	https://data.globe.gov/system/photos/2019/09/04/1191981/original.jpg	https://data.globe.gov/system/photos/2019/09/04/1191983/original.jpg	https://data.globe.gov/system/photos/2019/09/04/1191985/original.jpg
GV_2221	45.6772	-111.0578	1473.3		https://www.google.com/maps/search/45.6772,-111.0578	https://data.globe.gov/system/photos/2019/09/04/1192030/original.jpg	https://data.globe.gov/system/photos/2019/09/04/1192032/original.jpg	https://data.globe.gov/system/photos/2019/09/04/1192031/original.jpg	https://data.globe.gov/system/photos/2019/09/04/1192033/original.jpg	https://data.globe.gov/system/photos/2019/09/04/1192035/original.jpg
GV_2222	45.5487	-111.0501	1766.5		https://www.google.com/maps/search/45.5487,-111.0501	https://data.globe.gov/system/photos/2019/09/04/1192036/original.jpg	https://data.globe.gov/system/photos/2019/09/04/1192038/original.jpg	https://data.globe.gov/system/photos/2019/09/04/1192037/original.jpg	https://data.globe.gov/system/photos/2019/09/04/1192039/original.jpg	https://data.globe.gov/system/photos/2019/09/04/1192041/original.jpg
GV_2223	-26.1989	113.2045	91.6		https://www.google.com/maps/search/-26.1989,113.2045	https://data.globe.gov/system/photos/2019/09/03/1192100/original.jpg	https://data.globe.gov/system/photos/2019/09/03/1192102/original.jpg	https://data.globe.gov/system/photos/2019/09/03/1192101/original.jpg	https://data.globe.gov/system/photos/2019/09/03/1192103/original.jpg	https://data.globe.gov/system/photos/2019/09/03/1192105/original.jpg
GV_2224	-34.5283	138.9535	256		https://www.google.com/maps/search/-34.5283,138.9535	https://data.globe.gov/system/photos/2019/09/05/1192345/original.jpg	https://data.globe.gov/system/photos/2019/09/05/1192347/original.jpg	https://data.globe.gov/system/photos/2019/09/05/1192346/original.jpg	https://data.globe.gov/system/photos/2019/09/05/1192348/original.jpg	https://data.globe.gov/system/photos/2019/09/05/1192350/original.jpg
GV_2225	-34.8679	139.3911	6.1		https://www.google.com/maps/search/-34.8679,139.3911	https://data.globe.gov/system/photos/2019/09/05/1192357/original.jpg	https://data.globe.gov/system/photos/2019/09/05/1192359/original.jpg	https://data.globe.gov/system/photos/2019/09/05/1192358/original.jpg	https://data.globe.gov/system/photos/2019/09/05/1192360/original.jpg	https://data.globe.gov/system/photos/2019/09/05/1192362/original.jpg
GV_2226	-34.8679	139.3911	6.1		https://www.google.com/maps/search/-34.8679,139.3911	https://data.globe.gov/system/photos/2019/09/05/1192363/original.jpg	https://data.globe.gov/system/photos/2019/09/05/1192365/original.jpg	https://data.globe.gov/system/photos/2019/09/05/1192364/original.jpg	https://data.globe.gov/system/photos/2019/09/05/1192366/original.jpg	https://data.globe.gov/system/photos/2019/09/05/1192368/original.jpg
GV_2227	42.7711	-71.1412	77.8		https://www.google.com/maps/search/42.7711,-71.1412	https://data.globe.gov/system/photos/2019/09/05/1192439/original.jpg	https://data.globe.gov/system/photos/2019/09/05/1192441/original.jpg	https://data.globe.gov/system/photos/2019/09/05/1192440/original.jpg	https://data.globe.gov/system/photos/2019/09/05/1192442/original.jpg	https://data.globe.gov/system/photos/2019/09/05/1192444/original.jpg
GV_2228	61.6041	-149.1376	84.4		https://www.google.com/maps/search/61.6041,-149.1376	https://data.globe.gov/system/photos/2019/09/05/1193258/original.jpg	https://data.globe.gov/system/photos/2019/09/05/1193260/original.jpg	https://data.globe.gov/system/photos/2019/09/05/1193259/original.jpg	https://data.globe.gov/system/photos/2019/09/05/1193261/original.jpg	https://data.globe.gov/system/photos/2019/09/05/1193263/original.jpg
GV_2229	61.6023	-149.1395	85.3		https://www.google.com/maps/search/61.6023,-149.1395	https://data.globe.gov/system/photos/2019/09/05/1193271/original.jpg	https://data.globe.gov/system/photos/2019/09/05/1193273/original.jpg	https://data.globe.gov/system/photos/2019/09/05/1193272/original.jpg	https://data.globe.gov/system/photos/2019/09/05/1193274/original.jpg	https://data.globe.gov/system/photos/2019/09/05/1193276/original.jpg
GV_2230	-34.8332	139.1272	386.9		https://www.google.com/maps/search/-34.8332,139.1272	https://data.globe.gov/system/photos/2019/09/05/1193423/original.jpg	https://data.globe.gov/system/photos/2019/09/05/1193425/original.jpg	https://data.globe.gov/system/photos/2019/09/05/1193424/original.jpg	https://data.globe.gov/system/photos/2019/09/05/1193426/original.jpg	https://data.globe.gov/system/photos/2019/09/05/1193428/original.jpg
GV_2231	-34.8545	139.1622	182.2		https://www.google.com/maps/search/-34.8545,139.1622	https://data.globe.gov/system/photos/2019/09/05/1193429/original.jpg	https://data.globe.gov/system/photos/2019/09/05/1193431/original.jpg	https://data.globe.gov/system/photos/2019/09/05/1193430/original.jpg	https://data.globe.gov/system/photos/2019/09/05/1193432/original.jpg	https://data.globe.gov/system/photos/2019/09/05/1193434/original.jpg
GV_2232	-34.938	139.2994	3.6		https://www.google.com/maps/search/-34.938,139.2994	https://data.globe.gov/system/photos/2019/09/06/1193659/original.jpg	https://data.globe.gov/system/photos/2019/09/06/1193661/original.jpg	https://data.globe.gov/system/photos/2019/09/06/1193660/original.jpg	https://data.globe.gov/system/photos/2019/09/06/1193662/original.jpg	https://data.globe.gov/system/photos/2019/09/06/1193664/original.jpg
GV_2233	-34.7418	138.9591	410.2		https://www.google.com/maps/search/-34.7418,138.9591	https://data.globe.gov/system/photos/2019/09/06/1193698/original.jpg	https://data.globe.gov/system/photos/2019/09/06/1193700/original.jpg	https://data.globe.gov/system/photos/2019/09/06/1193699/original.jpg	https://data.globe.gov/system/photos/2019/09/06/1193701/original.jpg	https://data.globe.gov/system/photos/2019/09/06/1193703/original.jpg
GV_2234	-34.7052	138.9341	386		https://www.google.com/maps/search/-34.7052,138.9341	https://data.globe.gov/system/photos/2019/09/06/1193704/original.jpg	https://data.globe.gov/system/photos/2019/09/06/1193706/original.jpg	https://data.globe.gov/system/photos/2019/09/06/1193705/original.jpg	https://data.globe.gov/system/photos/2019/09/06/1193707/original.jpg	https://data.globe.gov/system/photos/2019/09/06/1193709/original.jpg
GV_2235	53.4284	-0.6226	25.8		https://www.google.com/maps/search/53.4284,-0.6226	https://data.globe.gov/system/photos/2019/09/05/1193803/original.jpg	https://data.globe.gov/system/photos/2019/09/05/1193805/original.jpg	https://data.globe.gov/system/photos/2019/09/05/1193804/original.jpg	https://data.globe.gov/system/photos/2019/09/05/1193806/original.jpg	https://data.globe.gov/system/photos/2019/09/05/1193808/original.jpg
GV_2236	53.4283	-0.6227	26.4		https://www.google.com/maps/search/53.4283,-0.6227	https://data.globe.gov/system/photos/2019/09/06/1193809/original.jpg	https://data.globe.gov/system/photos/2019/09/06/1193811/original.jpg	https://data.globe.gov/system/photos/2019/09/06/1193810/original.jpg	https://data.globe.gov/system/photos/2019/09/06/1193812/original.jpg	https://data.globe.gov/system/photos/2019/09/06/1193814/original.jpg
GV_2237	54.4102	18.6341	3.9		https://www.google.com/maps/search/54.4102,18.6341	https://data.globe.gov/system/photos/2019/09/06/1194255/original.jpg	https://data.globe.gov/system/photos/2019/09/06/1194257/original.jpg	https://data.globe.gov/system/photos/2019/09/06/1194256/original.jpg	https://data.globe.gov/system/photos/2019/09/06/1194258/original.jpg	https://data.globe.gov/system/photos/2019/09/06/1194260/original.jpg
GV_2238	-15.7875	-47.8988	1131.4		https://www.google.com/maps/search/-15.7875,-47.8988	https://data.globe.gov/system/photos/2019/09/06/1194466/original.jpg	https://data.globe.gov/system/photos/2019/09/06/1194468/original.jpg	https://data.globe.gov/system/photos/2019/09/06/1194467/original.jpg	https://data.globe.gov/system/photos/2019/09/06/1194469/original.jpg	https://data.globe.gov/system/photos/2019/09/06/1194471/original.jpg
GV_2239	61.604	-149.1366	84.6		https://www.google.com/maps/search/61.604,-149.1366	https://data.globe.gov/system/photos/2019/09/04/1194638/original.jpg	https://data.globe.gov/system/photos/2019/09/04/1194640/original.jpg	https://data.globe.gov/system/photos/2019/09/04/1194639/original.jpg	https://data.globe.gov/system/photos/2019/09/04/1194641/original.jpg	https://data.globe.gov/system/photos/2019/09/04/1194643/original.jpg
GV_2240	-34.6138	138.855	203		https://www.google.com/maps/search/-34.6138,138.855	https://data.globe.gov/system/photos/2019/09/07/1194823/original.jpg	https://data.globe.gov/system/photos/2019/09/07/1194825/original.jpg	https://data.globe.gov/system/photos/2019/09/07/1194824/original.jpg	https://data.globe.gov/system/photos/2019/09/07/1194826/original.jpg	https://data.globe.gov/system/photos/2019/09/07/1194828/original.jpg
GV_2241	44.7804	-121.9896	659.9		https://www.google.com/maps/search/44.7804,-121.9896	https://data.globe.gov/system/photos/2019/09/06/1194829/original.jpg	https://data.globe.gov/system/photos/2019/09/06/1194831/original.jpg	https://data.globe.gov/system/photos/2019/09/06/1194830/original.jpg	https://data.globe.gov/system/photos/2019/09/06/1194832/original.jpg	https://data.globe.gov/system/photos/2019/09/06/1194834/original.jpg
GV_2242	44.7801	-121.9902	659.1		https://www.google.com/maps/search/44.7801,-121.9902	https://data.globe.gov/system/photos/2019/09/06/1194835/original.jpg	https://data.globe.gov/system/photos/2019/09/06/1194837/original.jpg	https://data.globe.gov/system/photos/2019/09/06/1194836/original.jpg	https://data.globe.gov/system/photos/2019/09/06/1194838/original.jpg	https://data.globe.gov/system/photos/2019/09/06/1194840/original.jpg
GV_2243	44.7798	-121.9903	655.3		https://www.google.com/maps/search/44.7798,-121.9903	https://data.globe.gov/system/photos/2019/09/06/1194841/original.jpg	https://data.globe.gov/system/photos/2019/09/06/1194843/original.jpg	https://data.globe.gov/system/photos/2019/09/06/1194842/original.jpg	https://data.globe.gov/system/photos/2019/09/06/1194844/original.jpg	https://data.globe.gov/system/photos/2019/09/06/1194846/original.jpg

GV_2244	44.7802	-121.9914	658.5		https://www.google.com/maps/search/44.7802,-121.9914	https://data.globe.gov/system/photos/2019/09/06/1194847/original.jpg	https://data.globe.gov/system/photos/2019/09/06/1194849/original.jpg	https://data.globe.gov/system/photos/2019/09/06/1194848/original.jpg	https://data.globe.gov/system/photos/2019/09/06/1194850/original.jpg	https://data.globe.gov/system/photos/2019/09/06/1194852/original.jpg
GV_2245	44.7796	-121.9915	663.8		https://www.google.com/maps/search/44.7796,-121.9915	https://data.globe.gov/system/photos/2019/09/06/1194853/original.jpg	https://data.globe.gov/system/photos/2019/09/06/1194855/original.jpg	https://data.globe.gov/system/photos/2019/09/06/1194854/original.jpg	https://data.globe.gov/system/photos/2019/09/06/1194856/original.jpg	https://data.globe.gov/system/photos/2019/09/06/1194858/original.jpg
GV_2246	44.7801	-121.9921	653.6		https://www.google.com/maps/search/44.7801,-121.9921	https://data.globe.gov/system/photos/2019/09/06/1194859/original.jpg	https://data.globe.gov/system/photos/2019/09/06/1194861/original.jpg	https://data.globe.gov/system/photos/2019/09/06/1194860/original.jpg	https://data.globe.gov/system/photos/2019/09/06/1194862/original.jpg	https://data.globe.gov/system/photos/2019/09/06/1194864/original.jpg
GV_2247	44.781	-121.9892	668.7		https://www.google.com/maps/search/44.781,-121.9892	https://data.globe.gov/system/photos/2019/09/06/1194865/original.jpg	https://data.globe.gov/system/photos/2019/09/06/1194867/original.jpg	https://data.globe.gov/system/photos/2019/09/06/1194866/original.jpg	https://data.globe.gov/system/photos/2019/09/06/1194868/original.jpg	https://data.globe.gov/system/photos/2019/09/06/1194870/original.jpg
GV_2248	44.7812	-121.9891	669.2		https://www.google.com/maps/search/44.7812,-121.9891	https://data.globe.gov/system/photos/2019/09/06/1194871/original.jpg	https://data.globe.gov/system/photos/2019/09/06/1194873/original.jpg	https://data.globe.gov/system/photos/2019/09/06/1194872/original.jpg	https://data.globe.gov/system/photos/2019/09/06/1194874/original.jpg	https://data.globe.gov/system/photos/2019/09/06/1194876/original.jpg
GV_2249	44.7813	-121.9884	672.4		https://www.google.com/maps/search/44.7813,-121.9884	https://data.globe.gov/system/photos/2019/09/06/1194877/original.jpg	https://data.globe.gov/system/photos/2019/09/06/1194879/original.jpg	https://data.globe.gov/system/photos/2019/09/06/1194878/original.jpg	https://data.globe.gov/system/photos/2019/09/06/1194880/original.jpg	https://data.globe.gov/system/photos/2019/09/06/1194882/original.jpg
GV_2250	44.7816	-121.9892	669		https://www.google.com/maps/search/44.7816,-121.9892	https://data.globe.gov/system/photos/2019/09/06/1194883/original.jpg	https://data.globe.gov/system/photos/2019/09/06/1194885/original.jpg	https://data.globe.gov/system/photos/2019/09/06/1194884/original.jpg	https://data.globe.gov/system/photos/2019/09/06/1194886/original.jpg	https://data.globe.gov/system/photos/2019/09/06/1194888/original.jpg
GV_2251	44.7804	-121.9901	662		https://www.google.com/maps/search/44.7804,-121.9901	https://data.globe.gov/system/photos/2019/09/06/1194889/original.jpg	https://data.globe.gov/system/photos/2019/09/06/1194891/original.jpg	https://data.globe.gov/system/photos/2019/09/06/1194890/original.jpg	https://data.globe.gov/system/photos/2019/09/06/1194892/original.jpg	https://data.globe.gov/system/photos/2019/09/06/1194894/original.jpg
GV_2252	44.7807	-121.99	665.1		https://www.google.com/maps/search/44.7807,-121.99	https://data.globe.gov/system/photos/2019/09/06/1194895/original.jpg	https://data.globe.gov/system/photos/2019/09/06/1194897/original.jpg	https://data.globe.gov/system/photos/2019/09/06/1194896/original.jpg	https://data.globe.gov/system/photos/2019/09/06/1194898/original.jpg	https://data.globe.gov/system/photos/2019/09/06/1194900/original.jpg
GV_2253	44.7771	-121.9971	659.4		https://www.google.com/maps/search/44.7771,-121.9971	https://data.globe.gov/system/photos/2019/09/06/1194901/original.jpg	https://data.globe.gov/system/photos/2019/09/06/1194903/original.jpg	https://data.globe.gov/system/photos/2019/09/06/1194902/original.jpg	https://data.globe.gov/system/photos/2019/09/06/1194904/original.jpg	https://data.globe.gov/system/photos/2019/09/06/1194906/original.jpg
GV_2254	44.7775	-121.9976	648.1		https://www.google.com/maps/search/44.7775,-121.9976	https://data.globe.gov/system/photos/2019/09/06/1194907/original.jpg	https://data.globe.gov/system/photos/2019/09/06/1194909/original.jpg	https://data.globe.gov/system/photos/2019/09/06/1194908/original.jpg	https://data.globe.gov/system/photos/2019/09/06/1194910/original.jpg	https://data.globe.gov/system/photos/2019/09/06/1194912/original.jpg
GV_2255	44.7864	-121.972	722.2		https://www.google.com/maps/search/44.7864,-121.972	https://data.globe.gov/system/photos/2019/09/06/1194913/original.jpg	https://data.globe.gov/system/photos/2019/09/06/1194915/original.jpg	https://data.globe.gov/system/photos/2019/09/06/1194914/original.jpg	https://data.globe.gov/system/photos/2019/09/06/1194916/original.jpg	https://data.globe.gov/system/photos/2019/09/06/1194918/original.jpg
GV_2256	44.8101	-121.8815	1087.9		https://www.google.com/maps/search/44.8101,-121.8815	https://data.globe.gov/system/photos/2019/09/06/1194919/original.jpg	https://data.globe.gov/system/photos/2019/09/06/1194921/original.jpg	https://data.globe.gov/system/photos/2019/09/06/1194920/original.jpg	https://data.globe.gov/system/photos/2019/09/06/1194922/original.jpg	https://data.globe.gov/system/photos/2019/09/06/1194924/original.jpg
GV_2257	44.9657	-121.895	689.1		https://www.google.com/maps/search/44.9657,-121.895	https://data.globe.gov/system/photos/2019/09/06/1194925/original.jpg	https://data.globe.gov/system/photos/2019/09/06/1194927/original.jpg	https://data.globe.gov/system/photos/2019/09/06/1194926/original.jpg	https://data.globe.gov/system/photos/2019/09/06/1194928/original.jpg	https://data.globe.gov/system/photos/2019/09/06/1194930/original.jpg
GV_2258	45.0167	-121.9206	631.8		https://www.google.com/maps/search/45.0167,-121.9206	https://data.globe.gov/system/photos/2019/09/06/1194931/original.jpg	https://data.globe.gov/system/photos/2019/09/06/1194933/original.jpg	https://data.globe.gov/system/photos/2019/09/06/1194932/original.jpg	https://data.globe.gov/system/photos/2019/09/06/1194934/original.jpg	https://data.globe.gov/system/photos/2019/09/06/1194936/original.jpg
GV_2259	45.0279	-121.9659	569.8		https://www.google.com/maps/search/45.0279,-121.9659	https://data.globe.gov/system/photos/2019/09/06/1194937/original.jpg	https://data.globe.gov/system/photos/2019/09/06/1194939/original.jpg	https://data.globe.gov/system/photos/2019/09/06/1194938/original.jpg	https://data.globe.gov/system/photos/2019/09/06/1194940/original.jpg	https://data.globe.gov/system/photos/2019/09/06/1194942/original.jpg
GV_2260	45.0279	-121.9653	571.1		https://www.google.com/maps/search/45.0279,-121.9653	https://data.globe.gov/system/photos/2019/09/06/1194943/original.jpg	https://data.globe.gov/system/photos/2019/09/06/1194945/original.jpg	https://data.globe.gov/system/photos/2019/09/06/1194944/original.jpg	https://data.globe.gov/system/photos/2019/09/06/1194946/original.jpg	https://data.globe.gov/system/photos/2019/09/06/1194948/original.jpg
GV_2261	-34.6176	138.8572	217.2		https://www.google.com/maps/search/-34.6176,138.8572	https://data.globe.gov/system/photos/2019/09/07/1194953/original.jpg	https://data.globe.gov/system/photos/2019/09/07/1194955/original.jpg	https://data.globe.gov/system/photos/2019/09/07/1194954/original.jpg	https://data.globe.gov/system/photos/2019/09/07/1194956/original.jpg	https://data.globe.gov/system/photos/2019/09/07/1194958/original.jpg
GV_2262	45.0241	-122.0181	495.1		https://www.google.com/maps/search/45.0241,-122.0181	https://data.globe.gov/system/photos/2019/09/06/1194959/original.jpg	https://data.globe.gov/system/photos/2019/09/06/1194961/original.jpg	https://data.globe.gov/system/photos/2019/09/06/1194960/original.jpg	https://data.globe.gov/system/photos/2019/09/06/1194962/original.jpg	https://data.globe.gov/system/photos/2019/09/06/1194964/original.jpg
GV_2263	45.0433	-122.0628	441.2		https://www.google.com/maps/search/45.0433,-122.0628	https://data.globe.gov/system/photos/2019/09/06/1194971/original.jpg	https://data.globe.gov/system/photos/2019/09/06/1194973/original.jpg	https://data.globe.gov/system/photos/2019/09/06/1194972/original.jpg	https://data.globe.gov/system/photos/2019/09/06/1194974/original.jpg	https://data.globe.gov/system/photos/2019/09/06/1194976/original.jpg
GV_2264	45.0447	-122.0626	440.6		https://www.google.com/maps/search/45.0447,-122.0626	https://data.globe.gov/system/photos/2019/09/06/1194977/original.jpg	https://data.globe.gov/system/photos/2019/09/06/1194979/original.jpg	https://data.globe.gov/system/photos/2019/09/06/1194978/original.jpg	https://data.globe.gov/system/photos/2019/09/06/1194980/original.jpg	https://data.globe.gov/system/photos/2019/09/06/1194982/original.jpg
GV_2265	45.045	-122.0624	455.7		https://www.google.com/maps/search/45.045,-122.0624	https://data.globe.gov/system/photos/2019/09/06/1194983/original.jpg	https://data.globe.gov/system/photos/2019/09/06/1194985/original.jpg	https://data.globe.gov/system/photos/2019/09/06/1194984/original.jpg	https://data.globe.gov/system/photos/2019/09/06/1194986/original.jpg	https://data.globe.gov/system/photos/2019/09/06/1194988/original.jpg
GV_2266	45.0412	-122.0627	458.4		https://www.google.com/maps/search/45.0412,-122.0627	https://data.globe.gov/system/photos/2019/09/06/1194989/original.jpg	https://data.globe.gov/system/photos/2019/09/06/1194991/original.jpg	https://data.globe.gov/system/photos/2019/09/06/1194990/original.jpg	https://data.globe.gov/system/photos/2019/09/06/1194992/original.jpg	https://data.globe.gov/system/photos/2019/09/06/1194994/original.jpg
GV_2267	45.0419	-122.0627	456.1		https://www.google.com/maps/search/45.0419,-122.0627	https://data.globe.gov/system/photos/2019/09/06/1194995/original.jpg	https://data.globe.gov/system/photos/2019/09/06/1194997/original.jpg	https://data.globe.gov/system/photos/2019/09/06/1194996/original.jpg	https://data.globe.gov/system/photos/2019/09/06/1194998/original.jpg	https://data.globe.gov/system/photos/2019/09/06/1195000/original.jpg
GV_2268	45.0434	-122.0625	455.6		https://www.google.com/maps/search/45.0434,-122.0625	https://data.globe.gov/system/photos/2019/09/06/1195001/original.jpg	https://data.globe.gov/system/photos/2019/09/06/1195003/original.jpg	https://data.globe.gov/system/photos/2019/09/06/1195002/original.jpg	https://data.globe.gov/system/photos/2019/09/06/1195004/original.jpg	https://data.globe.gov/system/photos/2019/09/06/1195006/original.jpg
GV_2269	-28.8676	114.6339	1.4		https://www.google.com/maps/search/-28.8676,114.6339	https://data.globe.gov/system/photos/2019/09/07/1195038/original.jpg	https://data.globe.gov/system/photos/2019/09/07/1195040/original.jpg	https://data.globe.gov/system/photos/2019/09/07/1195039/original.jpg	https://data.globe.gov/system/photos/2019/09/07/1195041/original.jpg	https://data.globe.gov/system/photos/2019/09/07/1195043/original.jpg
GV_2270	24.4074	118.2891	34.4		https://www.google.com/maps/search/24.4074,118.2891	https://data.globe.gov/system/photos/2019/09/07/1195055/original.jpg	https://data.globe.gov/system/photos/2019/09/07/1195057/original.jpg	https://data.globe.gov/system/photos/2019/09/07/1195056/original.jpg	https://data.globe.gov/system/photos/2019/09/07/1195058/original.jpg	https://data.globe.gov/system/photos/2019/09/07/1195060/original.jpg
GV_2271	24.4074	118.2894	32.4		https://www.google.com/maps/search/24.4074,118.2894	https://data.globe.gov/system/photos/2019/09/07/1195087/original.jpg	https://data.globe.gov/system/photos/2019/09/07/1195089/original.jpg	https://data.globe.gov/system/photos/2019/09/07/1195088/original.jpg	https://data.globe.gov/system/photos/2019/09/07/1195090/original.jpg	https://data.globe.gov/system/photos/2019/09/07/1195092/original.jpg
GV_2272	53.4284	-0.6226	25.8		https://www.google.com/maps/search/53.4284,-0.6226	https://data.globe.gov/system/photos/2019/09/07/1195325/original.jpg	https://data.globe.gov/system/photos/2019/09/07/1195331/original.jpg	https://data.globe.gov/system/photos/2019/09/07/1195329/original.jpg	https://data.globe.gov/system/photos/2019/09/07/1195332/original.jpg	https://data.globe.gov/system/photos/2019/09/07/1195334/original.jpg
GV_2273	53.4284	-0.6226	25.8		https://www.google.com/maps/search/53.4284,-0.6226	https://data.globe.gov/system/photos/2019/09/08/1195965/original.jpg	https://data.globe.gov/system/photos/2019/09/08/1195967/original.jpg	https://data.globe.gov/system/photos/2019/09/08/1195966/original.jpg	https://data.globe.gov/system/photos/2019/09/08/1195968/original.jpg	https://data.globe.gov/system/photos/2019/09/08/1195970/original.jpg
GV_2274	-28.7638	114.6122	0		https://www.google.com/maps/search/-28.7638,114.6122	https://data.globe.gov/system/photos/2019/09/08/1196418/original.jpg	https://data.globe.gov/system/photos/2019/09/08/1196420/original.jpg	https://data.globe.gov/system/photos/2019/09/08/1196419/original.jpg	https://data.globe.gov/system/photos/2019/09/08/1196421/original.jpg	https://data.globe.gov/system/photos/2019/09/08/1196423/original.jpg
GV_2275	-34.7522	138.9997	458.1		https://www.google.com/maps/search/-34.7522,138.9997	https://data.globe.gov/system/photos/2019/09/08/1196527/original.jpg	https://data.globe.gov/system/photos/2019/09/08/1196529/original.jpg	https://data.globe.gov/system/photos/2019/09/08/1196528/original.jpg	https://data.globe.gov/system/photos/2019/09/08/1196530/original.jpg	https://data.globe.gov/system/photos/2019/09/08/1196532/original.jpg
GV_2276	-28.4289	113.7443	9.5		https://www.google.com/maps/search/-28.4289,113.7443	https://data.globe.gov/system/photos/2019/09/09/1196705/original.jpg	https://data.globe.gov/system/photos/2019/09/09/1196707/original.jpg	https://data.globe.gov/system/photos/2019/09/09/1196706/original.jpg	https://data.globe.gov/system/photos/2019/09/09/1196708/original.jpg	https://data.globe.gov/system/photos/2019/09/09/1196710/original.jpg
GV_2277	14.4529	103.4316	212.1		https://www.google.com/maps/search/14.4529,103.4316	https://data.globe.gov/system/photos/2019/09/09/1196772/original.jpg	https://data.globe.gov/system/photos/2019/09/09/1196774/original.jpg	https://data.globe.gov/system/photos/2019/09/09/1196773/original.jpg	https://data.globe.gov/system/photos/2019/09/09/1196775/original.jpg	https://data.globe.gov/system/photos/2019/09/09/1196780/original.jpg
GV_2278	14.4531	103.4315	212		https://www.google.com/maps/search/14.4531,103.4315	https://data.globe.gov/system/photos/2019/09/09/1196776/original.jpg	https://data.globe.gov/system/photos/2019/09/09/1196779/original.jpg	https://data.globe.gov/system/photos/2019/09/09/1196778/original.jpg	https://data.globe.gov/system/photos/2019/09/09/1196781/original.jpg	https://data.globe.gov/system/photos/2019/09/09/1196783/original.jpg
GV_2279	14.4528	103.4319	212.4		https://www.google.com/maps/search/14.4528,103.4319	https://data.globe.gov/system/photos/2019/09/09/1196846/original.jpg	https://data.globe.gov/system/photos/2019/09/09/1196853/original.jpg	https://data.globe.gov/system/photos/2019/09/09/1196847/original.jpg	https://data.globe.gov/system/photos/2019/09/09/1196855/original.jpg	https://data.globe.gov/system/photos/2019/09/09/1196858/original.jpg
GV_2280	14.4529	103.4318	212.2		https://www.google.com/maps/search/14.4529,103.4318	https://data.globe.gov/system/photos/2019/09/09/1196861/original.jpg	https://data.globe.gov/system/photos/2019/09/09/1196863/original.jpg	https://data.globe.gov/system/photos/2019/09/09/1196862/original.jpg	https://data.globe.gov/system/photos/2019/09/09/1196864/original.jpg	https://data.globe.gov/system/photos/2019/09/09/1196866/original.jpg
GV_2281	70	1	-3145.5		https://www.google.com/maps/search/70,1	https://data.globe.gov/system/photos/2019/09/09/1196971/original.jpg	https://data.globe.gov/system/photos/2019/09/09/1196973/original.jpg	https://data.globe.gov/system/photos/2019/09/09/1196972/original.jpg	https://data.globe.gov/system/photos/2019/09/09/1196974/original.jpg	https://data.globe.gov/system/photos/2019/09/09/1196976/original.jpg
GV_2282	53.4284	-0.6226	25.8		https://www.google.com/maps/search/53.4284,-0.6226	https://data.globe.gov/system/photos/2019/09/09/1197095/original.jpg	https://data.globe.gov/system/photos/2019/09/09/1197097/original.jpg	https://data.globe.gov/system/photos/2019/09/09/1197096/original.jpg	https://data.globe.gov/system/photos/2019/09/09/1197098/original.jpg	https://data.globe.gov/system/photos/2019/09/09/1197100/original.jpg
GV_2283	-34.6673	138.8443	296.5		https://www.google.com/maps/search/-34.6673,138.8443	https://data.globe.gov/system/photos/2019/09/09/1197593/original.jpg	https://data.globe.gov/system/photos/2019/09/09/1197595/original.jpg	https://data.globe.gov/system/photos/2019/09/09/1197594/original.jpg	https://data.globe.gov/system/photos/2019/09/09/1197596/original.jpg	https://data.globe.gov/system/photos/2019/09/09/1197598/original.jpg
GV_2284	-34.7162	138.8335	315.1		https://www.google.com/maps/search/-34.7162,138.8335	https://data.globe.gov/system/photos/2019/09/09/1197600/original.jpg	https://data.globe.gov/system/photos/2019/09/09/1197602/original.jpg	https://data.globe.gov/system/photos/2019/09/09/1197601/original.jpg	https://data.globe.gov/system/photos/2019/09/09/1197603/original.jpg	https://data.globe.gov/system/photos/2019/09/09/1197605/original.jpg
GV_2285	14.4528	103.4319	212.4		https://www.google.com/maps/search/14.4528,103.4319	https://data.globe.gov/system/photos/2019/09/10/1197737/original.jpg	https://data.globe.gov/system/photos/2019/09/10/1197739/original.jpg	https://data.globe.gov/system/photos/2019/09/10/1197738/original.jpg	https://data.globe.gov/system/photos/2019/09/10/1197740/original.jpg	https://data.globe.gov/system/photos/2019/09/10/1197744/original.jpg
GV_2286	14.453	103.4315	212		https://www.google.com/maps/search/14.453,103.4315	https://data.globe.gov/system/photos/2019/09/10/1197776/original.jpg	https://data.globe.gov/system/photos/2019/09/10/1197781/original.jpg	https://data.globe.gov/system/photos/2019/09/10/1197780/original.jpg	https://data.globe.gov/system/photos/2019/09/10/1197783/original.jpg	https://data.globe.gov/system/photos/2019/09/10/1197788/original.jpg
GV_2287	14.4529	103.4317	212.1		https://www.google.com/maps/search/14.4529,103.4317	https://data.globe.gov/system/photos/2019/09/10/1197928/original.jpg	https://data.globe.gov/system/photos/2019/09/10/1197930/original.jpg	https://data.globe.gov/system/photos/2019/09/10/1197929/original.jpg	https://data.globe.gov/system/photos/2019/09/10/1197931/original.jpg	https://data.globe.gov/system/photos/2019/09/10/1197933/original.jpg
GV_2288	14.4527	103.4318	212.3		https://www.google.com/maps/search/14.4527,103.4318	https://data.globe.gov/system/photos/2019/09/10/1197971/original.jpg	https://data.globe.gov/system/photos/2019/09/10/1197973/original.jpg	https://data.globe.gov/system/photos/2019/09/10/1197972/original.jpg	https://data.globe.gov/system/photos/2019/09/10/1197974/original.jpg	https://data.globe.gov/system/photos/2019/09/10/1197976/original.jpg
GV_2289	14.4528	103.4318	212.3		https://www.google.com/maps/search/14.4528,103.4318	https://data.globe.gov/system/photos/2019/09/10/1198003/original.jpeg	https://data.globe.gov/system/photos/2019/09/10/1198005/original.jpeg	https://data.globe.gov/system/photos/2019/09/10/1198004/original.jpeg	https://data.globe.gov/system/photos/2019/09/10/1198006/original.jpeg	https://data.globe.gov/system/photos/2019/09/10/1198008/original.jpeg
GV_2290	-27.548	-48.4981	8.7		https://www.google.com/maps/search/-27.548,-48.4981	https://data.globe.gov/system/photos/2019/09/04/1198818/original.jpg	https://data.globe.gov/system/photos/2019/09/04/1198820/original.jpg	https://data.globe.gov/system/photos/2019/09/04/1198819/original.jpg	https://data.globe.gov/system/photos/2019/09/04/1198821/original.jpg	https://data.globe.gov/system/photos/2019/09/04/1198823/original.jpg
GV_2291	45.4969	-122.6678	9.1		https://www.google.com/maps/search/45.4969,-122.6678	https://data.globe.gov/system/photos/2019/09/10/1198830/original.jpg	https://data.globe.gov/system/photos/2019/09/10/1198832/original.jpg	https://data.globe.gov/system/photos/2019/09/10/1198831/original.jpg	https://data.globe.gov/system/photos/2019/09/10/1198833/original.jpg	https://data.globe.gov/system/photos/2019/09/10/1198835/original.jpg
GV_2292	42.0236	-87.8607	193.2		https://www.google.com/maps/search/42.0236,-87.8607	https://data.globe.gov/system/photos/2019/09/10/1198872/original.jpg	https://data.globe.gov/system/photos/2019/09/10/1198875/original.jpg	https://data.globe.gov/system/photos/2019/09/10/1198874/original.jpg	https://data.globe.gov/system/photos/2019/09/10/1198876/original.jpg	https://data.globe.gov/system/photos/2019/09/10/1198878/original.jpg
GV_2293	39.186	-86.7784	231.9		https://www.google.com/maps/search/39.186,-86.7784	https://data.globe.gov/system/photos/2019/09/10/1198914/original.jpg	https://data.globe.gov/system/photos/2019/09/10/1198916/original.jpg	https://data.globe.gov/system/photos/2019/09/10/1198915/original.jpg	https://data.globe.gov/system/photos/2019/09/10/1198917/original.jpg	https://data.globe.gov/system/photos/2019/09/10/1198919/original.jpg
GV_2294	53.4283	-0.6226	26.4		https://www.google.com/maps/search/53.4283,-0.6226	https://data.globe.gov/system/photos/2019/09/10/1200106/original.jpg	https://data.globe.gov/system/photos/2019/09/10/1200108/original.jpg	https://data.globe.gov/system/photos/2019/09/10/1200107/original.jpg	https://data.globe.gov/system/photos/2019/09/10/1200109/original.jpg	https://data.globe.gov/system/photos/2019/09/10/1200111/original.jpg
GV_2295	53.4283	-0.6233	25.2		https://www.google.com/maps/search/53.4283,-0.6233	https://data.globe.gov/system/photos/2019/09/11/1200112/original.jpg	https://data.globe.gov/system/photos/2019/09/11/1200114/original.jpg	https://data.globe.gov/system/photos/2019/09/11/1200113/original.jpg	https://data.globe.gov/system/photos/2019/09/11/1200115/original.jpg	https://data.globe.gov/system/photos/2019/09/11/1200117/original.jpg
GV_2296	14.6519	121.0514	51.2		https://www.google.com/maps/search/14.6519,121.0514	https://data.globe.gov/system/photos/2019/09/12/1200695/original.jpg	https://data.globe.gov/system/photos/2019/09/12/1200697/original.jpg	https://data.globe.gov/system/photos/2019/09/12/1200696/original.jpg	https://data.globe.gov/system/photos/2019/09/12/1200698/original.jpg	https://data.globe.gov/system/photos/2019/09/12/1200700/original.jpg
GV_2297	53.4286	-0.6207	27.3		https://www.google.com/maps/search/53.4286,-0.6207	https://data.globe.gov/system/photos/2019/09/12/1200793/original.jpg	https://data.globe.gov/system/photos/2019/09/12/1200796/original.jpg	https://data.globe.gov/system/photos/2019/09/12/1200795/original.jpg	https://data.globe.gov/system/photos/2019/09/12/1200797/original.jpg	https://data.globe.gov/system/photos/2019/09/12/1200799/original.jpg
GV_2298	36.0169	-95.0165	278.5		https://www.google.com/maps/search/36.0169,-95.0165	https://data.globe.gov/system/photos/2019/09/12/1201383/original.jpg	https://data.globe.gov/system/photos/2019/09/12/1201385/original.jpg	https://data.globe.gov/system/photos/2019/09/12/1201384/original.jpg	https://data.globe.gov/system/photos/2019/09/12/1201386/original.jpg	https://data.globe.gov/system/photos/2019/09/12/1201388/original.jpg
GV_2299	53.4283	-0.6226	26.4		https://www.google.com/maps/search/53.4283,-0.6226	https://data.globe.gov/system/photos/2019/09/13/1201924/original.jpg	https://data.globe.gov/system/photos/2019/09/13/1201926/original.jpg	https://data.globe.gov/system/photos/2019/09/13/1201925/original.jpg	https://data.globe.gov/system/photos/2019/09/13/1201927/original.jpg	https://data.globe.gov/system/photos/2019/09/13/1201929/original.jpg
GV_2300	35.9127	-94.9707	241.5		https://www.google.com/maps/search/35.9127,-94.9707	https://data.globe.gov/system/photos/2019/09/13/1202532/original.jpg	https://data.globe.gov/system/photos/2019/09/13/1202534/original.jpg	https://data.globe.gov/system/photos/2019/09/13/1202533/original.jpg	https://data.globe.gov/system/photos/2019/09/13/1202535/original.jpg	https://data.globe.gov/system/photos/2019/09/13/1202537/original.jpg
GV_2301	35.9123	-94.9706	239.6		https://www.google.com/maps/search/35.9123,-94.9706	https://data.globe.gov/system/photos/2019/09/13/1202538/original.jpg	https://data.globe.gov/system/photos/2019/09/13/1202540/original.jpg	https://data.globe.gov/system/photos/2019/09/13/1202539/original.jpg	https://data.globe.gov/system/photos/2019/09/13/1202541/original.jpg	https://data.globe.gov/system/photos/2019/09/13/1202543/original.jpg
GV_2302	53.4285	-0.6184	30.2		https://www.google.com/maps/search/53.4285,-0.6184	https://data.globe.gov/system/photos/2019/09/14/1203378/original.jpg	https://data.globe.gov/system/photos/2019/09/14/1203380/original.jpg	https://data.globe.gov/system/photos/2019/09/14/1203379/original.jpg	https://data.globe.gov/system/photos/2019/09/14/1203381/original.jpg	https://data.globe.gov/system/photos/2019/09/14/1203383/original.jpg
GV_2303	35.8845	-94.9767	248.1		https://www.google.com/maps/search/35.8845,-94.9767	https://data.globe.gov/system/photos/2019/09/14/1203537/original.jpg	https://data.globe.gov/system/photos/2019/09/14/1203540/original.jpg	https://data.globe.gov/system/photos/2019/09/14/1203538/original.jpg	https://data.globe.gov/system/photos/2019/09/14/1203541/original.jpg	https://data.globe.gov/system/photos/2019/09/14/1203543/original.jpg
GV_2304	35.9198	-94.9688	251.6		https://www.google.com/maps/search/35.9198,-94.9688	https://data.globe.gov/system/photos/2019/09/14/1203544/original.jpg	https://data.globe.gov/system/photos/2019/09/14/1203546/original.jpg	https://data.globe.gov/system/photos/2019/09/14/1203545/original.jpg	https://data.globe.gov/system/photos/2019/09/14/1203547/original.jpg	https://data.globe.gov/system/photos/2019/09/14/1203549/original.jpg
GV_2305	35.9187	-94.9693	244.7		https://www.google.com/maps/search/35.9187,-94.9693	https://data.globe.gov/system/photos/2019/09/14/1203550/original.jpg	https://data.globe.gov/system/photos/2019/09/14/1203552/original.jpg	https://data.globe.gov/system/photos/2019/09/14/1203551/original.jpg	https://data.globe.gov/system/photos/2019/09/14/1203553/original.jpg	https://data.globe.gov/system/photos/2019/09/14/1203555/original.jpg
GV_2306	35.9187	-94.9688	243.8		https://www.google.com/maps/search/35.9187,-94.9688	https://data.globe.gov/system/photos/2019/09/14/1203556/original.jpg	https://data.globe.gov/system/photos/2019/09/14/1203558/original.jpg	https://data.globe.gov/system/photos/2019/09/14/1203557/original.jpg	https://data.globe.gov/system/photos/2019/09/14/1203559/original.jpg	https://data.globe.gov/system/photos/2019/09/14/1203561/original.jpg
GV_2307	53.4266	-0.6296	23.4		https://www.google.com/maps/search/53.4266,-0.6296	https://data.globe.gov/system/photos/2019/09/15/1203692/original.jpg	https://data.globe.gov/system/photos/2019/09/15/1203694/original.jpg	https://data.globe.gov/system/photos/2019/09/15/1203693/original.jpg	https://data.globe.gov/system/photos/2019/09/15/1203695/original.jpg	https://data.globe.gov/system/photos/2019/09/15/1203697/original.jpg
GV_2308	52.3305	4.5801	2.9		https://www.google.com/maps/search/52.3305,4.5801	https://data.globe.gov/system/photos/2019/09/15/1203888/original.jpg	https://data.globe.gov/system/photos/2019/09/15/1203890/original.jpg	https://data.globe.gov/system/photos/2019/09/15/1203889/original.jpg	https://data.globe.gov/system/photos/2019/09/15/1203891/original.jpg	https://data.globe.gov/system/photos/2019/09/15/1203893/original.jpg
GV_2309	42.1549	-83.1512	175.5		https://www.google.com/maps/search/42.1549,-83.1512	https://data.globe.gov/system/photos/2019/09/15/1204203/original.jpg	https://data.globe.gov/system/photos/2019/09/15/1204205/original.jpg	https://data.globe.gov/system/photos/2019/09/15/1204204/original.jpg	https://data.globe.gov/system/photos/2019/09/15/1204206/original.jpg	https://data.globe.gov/system/photos/2019/09/15/1204208/original.jpg
GV_2310	51.9998	-109.3865	678		https://www.google.com/maps/search/51.9998,-109.3865	https://data.globe.gov/system/photos/2019/09/15/1204256/original.jpg	https://data.globe.gov/system/photos/2019/09/15/1204258/original.jpg	https://data.globe.gov/system/photos/2019/09/15/1204257/original.jpg	https://data.globe.gov/system/photos/2019/09/15/1204259/original.jpg	https://data.globe.gov/system/photos/2019/09/15/1204261/original.jpg
GV_2311	-25.3646	116.1475	332.5		https://www.google.com/maps/search/-25.3646,116.1475	https://data.globe.gov/system/photos/2019/09/13/1205882/original.jpg	https://data.globe.gov/system/photos/2019/09/13/1205884/original.jpg	https://data.globe.gov/system/photos/2019/09/13/1205883/original.jpg	https://data.globe.gov/system/photos/2019/09/13/1205885/original.jpg	https://data.globe.gov/system/photos/2019/09/13/1205887/original.jpg
GV_2312	-24.8495	116.9311	434.3		https://www.google.com/maps/search/-24.8495,116.9311	https://data.globe.gov/system/photos/2019/09/14/1205888/original.jpg	https://data.globe.gov/system/photos/2019/09/14/1205890/original.jpg	https://data.globe.gov/system/photos/2019/09/14/1205889/original.jpg	https://data.globe.gov/system/photos/2019/09/14/1205891/original.jpg	https://data.globe.gov/system/photos/2019/09/14/1205893/original.jpg
GV_2313	-24.326	116.8431	1061.6		https://www.google.com/maps/search/-24.326,116.8431	https://data.globe.gov/system/photos/2019/09/15/1205894/original.jpg	https://data.globe.gov/system/photos/2019/09/15/1205896/original.jpg	https://data.globe.gov/system/photos/2019/09/15/1205895/original.jpg	https://data.globe.gov/system/photos/2019/09/15/1205897/original.jpg	https://data.globe.gov/system/photos/2019/09/15/1205899/original.jpg
GV_2314	-23.9371	115.592	294.1		https://www.google.com/maps/search/-23.9371,115.592	https://data.globe.gov/system/photos/2019/09/16/1205900/original.jpg	https://data.globe.gov/system/photos/2019/09/16/1205902/original.jpg	https://data.globe.gov/system/photos/2019/09/16/1205901/original.jpg	https://data.globe.gov/system/photos/2019/09/16/1205903/original.jpg	https://data.globe.gov/system/photos/2019/09/16/1205905/original.jpg
GV_2315	-23.4542	113.784	38.1		https://www.google.com/maps/search/-23.4542,113.784	https://data.globe.gov/system/photos/2019/09/17/1205906/original.jpg	https://data.globe.gov/system/photos/2019/09/17/1205908/original.jpg	https://data.globe.gov/system/photos/2019/09/17/1205907/original.jpg	https://data.globe.gov/system/photos/2019/09/17/1205909/original.jpg	https://data.globe.gov/system/photos/2019/09/17/1205911/original.jpg
GV_2316	6.1784	-75.6618	2000.9		https://www.google.com/maps/search/6.1784,-75.6618	https://data.globe.gov/system/photos/2019/09/17/1206818/original.jpg	https://data.globe.gov/system/photos/2019/09/17/1206820/original.jpg	https://data.globe.gov/system/photos/2019/09/17/1206819/original.jpg	https://data.globe.gov/system/photos/2019/09/17/1206821/original.jpg	https://data.globe.gov/system/photos/2019/09/17/1206829/original.jpg
GV_2317	52.9068	1.2597	64.6		https://www.google.com/maps/search/52.9068,1.2597	https://data.globe.gov/system/photos/2019/09/17/1206830/original.jpg	https://data.globe.gov/system/photos/2019/09/17/1206832/original.jpg	https://data.globe.gov/system/photos/2019/09/17/1206831/original.jpg	https://data.globe.gov/system/photos/2019/09/17/1206833/original.jpg	https://data.globe.gov/system/photos/2019/09/17/1206836/original.jpg
GV_2318	40.0325	-75.2305	36.7		https://www.google.com/maps/search/40.0325,-75.2305	https://data.globe.gov/system/photos/2019/09/17/1207081/original.jpg	https://data.globe.gov/system/photos/2019/09/17/1207083/original.jpg	https://data.globe.gov/system/photos/2019/09/17/1207082/original.jpg	https://data.globe.gov/system/photos/2019/09/17/1207084/original.jpg	https://data.globe.gov/system/photos/2019/09/17/1207086/original.jpg
GV_2319	39.1857	-86.7781	235.5		https://www.google.com/maps/search/39.1857,-86.7781	https://data.globe.gov/system/photos/2019/09/17/1207181/original.jpg	https://data.globe.gov/system/photos/2019/09/17/1207183/original.jpg	https://data.globe.gov/system/photos/2019/09/17/1207182/original.jpg	https://data.globe.gov/system/photos/2019/09/17/1207189/original.jpg	https://data.globe.gov/system/photos/2019/09/17/1207192/original.jpg
GV_2320	45.3313	-121.7112	1815.6		https://www.google.com/maps/search/45.3313,-121.7112	https://data.globe.gov/system/photos/2019/09/17/1207194/original.jpg	https://data.globe.gov/system/photos/2019/09/17/1207196/original.jpg	https://data.globe.gov/system/photos/2019/09/17/1207195/original.jpg	https://data.globe.gov/system/photos/2019/09/17/1207197/original.jpg	https://data.globe.gov/system/photos/2019/09/17/1207199/original.jpg
GV_2321	45.3315	-121.7118	1817.1		https://www.google.com/maps/search/45.3315,-121.7118	https://data.globe.gov/system/photos/2019/09/17/1207206/original.jpg	https://data.globe.gov/system/photos/2019/09/17/1207208/original.jpg	https://data.globe.gov/system/photos/2019/09/17/1207207/original.jpg	https://data.globe.gov/system/photos/2019/09/17/1207209/original.jpg	https://data.globe.gov/system/photos/2019/09/17/1207211/original.jpg
GV_2322	45.3295	-121.7086	1784.9		https://www.google.com/maps/search/45.3295,-121.7086	https://data.globe.gov/system/photos/2019/09/17/1207223/original.jpg	https://data.globe.gov/system/photos/2019/09/17/1207225/original.jpg	https://data.globe.gov/system/photos/2019/09/17/1207224/original.jpg	https://data.globe.gov/system/photos/2019/09/17/1207226/original.jpg	https://data.globe.gov/system/photos/2019/09/17/1207228/original.jpg
GV_2323	45.3288	-121.7085	1784.2		https://www.google.com/maps/search/45.3288,-121.7085	https://data.globe.gov/system/photos/2019/09/17/1207229/original.jpg	https://data.globe.gov/system/photos/2019/09/17/1207231/original.jpg	https://data.globe.gov/system/photos/2019/09/17/1207230/original.jpg	https://data.globe.gov/system/photos/2019/09/17/1207233/original.jpg	https://data.globe.gov/system/photos/2019/09/17/1207235/original.jpg
GV_2324	45.1484	-121.7519	988.7		https://www.google.com/maps/search/45.1484,-121.7519	https://data.globe.gov/system/photos/2019/09/18/1207341/original.jpg	https://data.globe.gov/system/photos/2019/09/18/1207344/original.jpg	https://data.globe.gov/system/photos/2019/09/18/1207342/original.jpg	https://data.globe.gov/system/photos/2019/09/18/1207346/original.jpg	https://data.globe.gov/system/photos/2019/09/18/1207350/original.jpg
GV_2325	45.1479	-121.7534	993.4		https://www.google.com/maps/search/45.1479,-121.7534	https://data.globe.gov/system/photos/2019/09/18/1207351/original.jpg	https://data.globe.gov/system/photos/2019/09/18/1207355/original.jpg	https://data.globe.gov/system/photos/2019/09/18/1207353/original.jpg	https://data.globe.gov/system/photos/2019/09/18/1207356/original.jpg	https://data.globe.gov/system/photos/2019/09/18/1207358/original.jpg
GV_2326	45.1456	-121.7524	991.5		https://www.google.com/maps/search/45.1456,-121.7524	https://data.globe.gov/system/photos/2019/09/18/1207359/original.jpg	https://data.globe.gov/system/photos/2019/09/18/1207361/original.jpg	https://data.globe.gov/system/photos/2019/09/18/1207360/original.jpg	https://data.globe.gov/system/photos/2019/09/18/1207362/original.jpg	https://data.globe.gov/system/photos/2019/09/18/1207364/original.jpg
GV_2327	45.1445	-121.7545	989.6		https://www.google.com/maps/search/45.1445,-121.7545	https://data.globe.gov/system/photos/2019/09/18/1207366/original.jpg	https://data.globe.gov/system/photos/2019/09/18/1207373/original.jpg	https://data.globe.gov/system/photos/2019/09/18/1207371/original.jpg	https://data.globe.gov/system/photos/2019/09/18/1207374/original.jpg	https://data.globe.gov/system/photos/2019/09/18/1207376/original.jpg
GV_2328	45.1436	-121.7546	986		https://www.google.com/maps/search/45.1436,-121.7546	https://data.globe.gov/system/photos/2019/09/18/1207377/original.jpg	https://data.globe.gov/system/photos/2019/09/18/1207379/original.jpg	https://data.globe.gov/system/photos/2019/09/18/1207378/original.jpg	https://data.globe.gov/system/photos/2019/09/18/1207380/original.jpg	https://data.globe.gov/system/photos/2019/09/18/1207382/original.jpg
GV_2329	45.1431	-121.7549	989		https://www.google.com/maps/search/45.1431,-121.7549	https://data.globe.gov/system/photos/2019/09/18/1207383/original.jpg	https://data.globe.gov/system/photos/2019/09/18/1207385/original.jpg	https://data.globe.gov/system/photos/2019/09/18/1207384/original.jpg	https://data.globe.gov/system/photos/2019/09/18/1207386/original.jpg	https://data.globe.gov/system/photos/2019/09/18/1207388/original.jpg
GV_2330	45.1421	-121.7547	993.5		https://www.google.com/maps/search/45.1421,-121.7547	https://data.globe.gov/system/photos/2019/09/18/1207389/original.jpg	https://data.globe.gov/system/photos/2019/09/18/1207391/original.jpg	https://data.globe.gov/system/photos/2019/09/18/1207390/original.jpg	https://data.globe.gov/system/photos/2019/09/18/1207392/original.jpg	https://data.globe.gov/system/photos/2019/09/18/1207394/original.jpg
GV_2331	45.1429	-121.7555	990.8		https://www.google.com/maps/search/45.1429,-121.7555	https://data.globe.gov/system/photos/2019/09/18/1207395/original.jpg	https://data.globe.gov/system/photos/2019/09/18/1207397/original.jpg	https://data.globe.gov/system/photos/2019/09/18/1207396/original.jpg	https://data.globe.gov/system/photos/2019/09/18/1207398/original.jpg	https://data.globe.gov/system/photos/2019/09/18/1207400/original.jpg
GV_2332	45.1482	-121.7506	990.4		https://www.google.com/maps/search/45.1482,-121.7506	https://data.globe.gov/system/photos/2019/09/18/1207401/original.jpg	https://data.globe.gov/system/photos/2019/09/18/1207403/original.jpg	https://data.globe.gov/system/photos/2019/09/18/1207402/original.jpg	https://data.globe.gov/system/photos/2019/09/18/1207404/original.jpg	https://data.globe.gov/system/photos/2019/09/18/1207406/original.jpg
GV_2333	45.1478	-121.7472	990		https://www.google.com/maps/search/45.1478,-121.7472	https://data.globe.gov/system/photos/2019/09/18/1207407/original.jpg	https://data.globe.gov/system/photos/2019/09/18/1207409/original.jpg	https://data.globe.gov/system/photos/2019/09/18/1207408/original.jpg	https://data.globe.gov/system/photos/2019/09/18/1207410/original.jpg	https://data.globe.gov/system/photos/2019/09/18/1207412/original.jpg

GV_2334	45.1483	-121.7506	991		https://www.google.com/maps/search/45.1483,-121.7506	https://data.globe.gov/system/photos/2019/09/18/1207421/original.jpg	https://data.globe.gov/system/photos/2019/09/18/1207423/original.jpg	https://data.globe.gov/system/photos/2019/09/18/1207422/original.jpg	https://data.globe.gov/system/photos/2019/09/18/1207424/original.jpg	https://data.globe.gov/system/photos/2019/09/18/1207426/original.jpg
GV_2335	45.1484	-121.7509	988.8		https://www.google.com/maps/search/45.1484,-121.7509	https://data.globe.gov/system/photos/2019/09/18/1207427/original.jpg	https://data.globe.gov/system/photos/2019/09/18/1207429/original.jpg	https://data.globe.gov/system/photos/2019/09/18/1207428/original.jpg	https://data.globe.gov/system/photos/2019/09/18/1207430/original.jpg	https://data.globe.gov/system/photos/2019/09/18/1207432/original.jpg
GV_2336	45.5611	-122.4428	8		https://www.google.com/maps/search/45.5611,-122.4428	https://data.globe.gov/system/photos/2019/09/17/1207444/original.jpg	https://data.globe.gov/system/photos/2019/09/17/1207446/original.jpg	https://data.globe.gov/system/photos/2019/09/17/1207445/original.jpg	https://data.globe.gov/system/photos/2019/09/17/1207447/original.jpg	https://data.globe.gov/system/photos/2019/09/17/1207449/original.jpg
GV_2337	45.5614	-122.4436	3.6		https://www.google.com/maps/search/45.5614,-122.4436	https://data.globe.gov/system/photos/2019/09/17/1207450/original.jpg	https://data.globe.gov/system/photos/2019/09/17/1207452/original.jpg	https://data.globe.gov/system/photos/2019/09/17/1207451/original.jpg	https://data.globe.gov/system/photos/2019/09/17/1207453/original.jpg	https://data.globe.gov/system/photos/2019/09/17/1207455/original.jpg
GV_2338	45.5611	-122.4432	6.8		https://www.google.com/maps/search/45.5611,-122.4432	https://data.globe.gov/system/photos/2019/09/17/1207456/original.jpg	https://data.globe.gov/system/photos/2019/09/17/1207458/original.jpg	https://data.globe.gov/system/photos/2019/09/17/1207457/original.jpg	https://data.globe.gov/system/photos/2019/09/17/1207459/original.jpg	https://data.globe.gov/system/photos/2019/09/17/1207461/original.jpg
GV_2339	45.5604	-122.4426	7.7		https://www.google.com/maps/search/45.5604,-122.4426	https://data.globe.gov/system/photos/2019/09/17/1207462/original.jpg	https://data.globe.gov/system/photos/2019/09/17/1207464/original.jpg	https://data.globe.gov/system/photos/2019/09/17/1207463/original.jpg	https://data.globe.gov/system/photos/2019/09/17/1207465/original.jpg	https://data.globe.gov/system/photos/2019/09/17/1207467/original.jpg
GV_2340	45.5096	-122.1981	426.9		https://www.google.com/maps/search/45.5096,-122.1981	https://data.globe.gov/system/photos/2019/09/17/1207468/original.jpg	https://data.globe.gov/system/photos/2019/09/17/1207470/original.jpg	https://data.globe.gov/system/photos/2019/09/17/1207469/original.jpg	https://data.globe.gov/system/photos/2019/09/17/1207471/original.jpg	https://data.globe.gov/system/photos/2019/09/17/1207473/original.jpg
GV_2341	45.5296	-122.1254	693.9		https://www.google.com/maps/search/45.5296,-122.1254	https://data.globe.gov/system/photos/2019/09/17/1207474/original.jpg	https://data.globe.gov/system/photos/2019/09/17/1207476/original.jpg	https://data.globe.gov/system/photos/2019/09/17/1207475/original.jpg	https://data.globe.gov/system/photos/2019/09/17/1207477/original.jpg	https://data.globe.gov/system/photos/2019/09/17/1207479/original.jpg
GV_2342	45.5467	-122.1015	871.1		https://www.google.com/maps/search/45.5467,-122.1015	https://data.globe.gov/system/photos/2019/09/17/1207480/original.jpg	https://data.globe.gov/system/photos/2019/09/17/1207482/original.jpg	https://data.globe.gov/system/photos/2019/09/17/1207481/original.jpg	https://data.globe.gov/system/photos/2019/09/17/1207483/original.jpg	https://data.globe.gov/system/photos/2019/09/17/1207485/original.jpg
GV_2343	45.5352	-122.1009	1029.4		https://www.google.com/maps/search/45.5352,-122.1009	https://data.globe.gov/system/photos/2019/09/17/1207486/original.jpg	https://data.globe.gov/system/photos/2019/09/17/1207488/original.jpg	https://data.globe.gov/system/photos/2019/09/17/1207487/original.jpg	https://data.globe.gov/system/photos/2019/09/17/1207489/original.jpg	https://data.globe.gov/system/photos/2019/09/17/1207491/original.jpg
GV_2344	45.5354	-122.1002	1041.5		https://www.google.com/maps/search/45.5354,-122.1002	https://data.globe.gov/system/photos/2019/09/17/1207492/original.jpg	https://data.globe.gov/system/photos/2019/09/17/1207494/original.jpg	https://data.globe.gov/system/photos/2019/09/17/1207493/original.jpg	https://data.globe.gov/system/photos/2019/09/17/1207495/original.jpg	https://data.globe.gov/system/photos/2019/09/17/1207497/original.jpg
GV_2345	45.3328	-121.7113	1841.1		https://www.google.com/maps/search/45.3328,-121.7113	https://data.globe.gov/system/photos/2019/09/17/1207498/original.jpg	https://data.globe.gov/system/photos/2019/09/17/1207500/original.jpg	https://data.globe.gov/system/photos/2019/09/17/1207499/original.jpg	https://data.globe.gov/system/photos/2019/09/17/1207501/original.jpg	https://data.globe.gov/system/photos/2019/09/17/1207503/original.jpg
GV_2346	45.3333	-121.7131	1855.1		https://www.google.com/maps/search/45.3333,-121.7131	https://data.globe.gov/system/photos/2019/09/17/1207504/original.jpg	https://data.globe.gov/system/photos/2019/09/17/1207506/original.jpg	https://data.globe.gov/system/photos/2019/09/17/1207505/original.jpg	https://data.globe.gov/system/photos/2019/09/17/1207507/original.jpg	https://data.globe.gov/system/photos/2019/09/17/1207510/original.jpg
GV_2347	45.3324	-121.7141	1837.4		https://www.google.com/maps/search/45.3324,-121.7141	https://data.globe.gov/system/photos/2019/09/17/1207511/original.jpg	https://data.globe.gov/system/photos/2019/09/17/1207514/original.jpg	https://data.globe.gov/system/photos/2019/09/17/1207512/original.jpg	https://data.globe.gov/system/photos/2019/09/17/1207515/original.jpg	https://data.globe.gov/system/photos/2019/09/17/1207518/original.jpg
GV_2348	45.3315	-121.7126	1817		https://www.google.com/maps/search/45.3315,-121.7126	https://data.globe.gov/system/photos/2019/09/17/1207519/original.jpg	https://data.globe.gov/system/photos/2019/09/17/1207521/original.jpg	https://data.globe.gov/system/photos/2019/09/17/1207520/original.jpg	https://data.globe.gov/system/photos/2019/09/17/1207522/original.jpg	https://data.globe.gov/system/photos/2019/09/17/1207525/original.jpg
GV_2349	45.3311	-121.7125	1810.3		https://www.google.com/maps/search/45.3311,-121.7125	https://data.globe.gov/system/photos/2019/09/17/1207526/original.jpg	https://data.globe.gov/system/photos/2019/09/17/1207528/original.jpg	https://data.globe.gov/system/photos/2019/09/17/1207527/original.jpg	https://data.globe.gov/system/photos/2019/09/17/1207529/original.jpg	https://data.globe.gov/system/photos/2019/09/17/1207532/original.jpg
GV_2350	45.3302	-121.7101	1796.9		https://www.google.com/maps/search/45.3302,-121.7101	https://data.globe.gov/system/photos/2019/09/17/1207533/original.jpg	https://data.globe.gov/system/photos/2019/09/17/1207535/original.jpg	https://data.globe.gov/system/photos/2019/09/17/1207534/original.jpg	https://data.globe.gov/system/photos/2019/09/17/1207536/original.jpg	https://data.globe.gov/system/photos/2019/09/17/1207538/original.jpg
GV_2351	45.3214	-121.7051	1660.1		https://www.google.com/maps/search/45.3214,-121.7051	https://data.globe.gov/system/photos/2019/09/17/1207539/original.jpg	https://data.globe.gov/system/photos/2019/09/17/1207542/original.jpg	https://data.globe.gov/system/photos/2019/09/17/1207540/original.jpg	https://data.globe.gov/system/photos/2019/09/17/1207543/original.jpg	https://data.globe.gov/system/photos/2019/09/17/1207545/original.jpg
GV_2352	45.3209	-121.7046	1655.7		https://www.google.com/maps/search/45.3209,-121.7046	https://data.globe.gov/system/photos/2019/09/17/1207546/original.jpg	https://data.globe.gov/system/photos/2019/09/17/1207548/original.jpg	https://data.globe.gov/system/photos/2019/09/17/1207547/original.jpg	https://data.globe.gov/system/photos/2019/09/17/1207549/original.jpg	https://data.globe.gov/system/photos/2019/09/17/1207551/original.jpg
GV_2353	45.3202	-121.7063	1646.1		https://www.google.com/maps/search/45.3202,-121.7063	https://data.globe.gov/system/photos/2019/09/17/1207552/original.jpg	https://data.globe.gov/system/photos/2019/09/17/1207554/original.jpg	https://data.globe.gov/system/photos/2019/09/17/1207553/original.jpg	https://data.globe.gov/system/photos/2019/09/17/1207555/original.jpg	https://data.globe.gov/system/photos/2019/09/17/1207557/original.jpg
GV_2354	45.3202	-121.7046	1653.5		https://www.google.com/maps/search/45.3202,-121.7046	https://data.globe.gov/system/photos/2019/09/17/1207558/original.jpg	https://data.globe.gov/system/photos/2019/09/17/1207560/original.jpg	https://data.globe.gov/system/photos/2019/09/17/1207559/original.jpg	https://data.globe.gov/system/photos/2019/09/17/1207561/original.jpg	https://data.globe.gov/system/photos/2019/09/17/1207563/original.jpg
GV_2355	45.3211	-121.7055	1652.3		https://www.google.com/maps/search/45.3211,-121.7055	https://data.globe.gov/system/photos/2019/09/17/1207564/original.jpg	https://data.globe.gov/system/photos/2019/09/17/1207566/original.jpg	https://data.globe.gov/system/photos/2019/09/17/1207565/original.jpg	https://data.globe.gov/system/photos/2019/09/17/1207567/original.jpg	https://data.globe.gov/system/photos/2019/09/17/1207569/original.jpg
GV_2356	45.3073	-121.7032	1473.6		https://www.google.com/maps/search/45.3073,-121.7032	https://data.globe.gov/system/photos/2019/09/17/1207570/original.jpg	https://data.globe.gov/system/photos/2019/09/17/1207572/original.jpg	https://data.globe.gov/system/photos/2019/09/17/1207571/original.jpg	https://data.globe.gov/system/photos/2019/09/17/1207573/original.jpg	https://data.globe.gov/system/photos/2019/09/17/1207575/original.jpg
GV_2357	45.229	-121.6991	1192.8		https://www.google.com/maps/search/45.229,-121.6991	https://data.globe.gov/system/photos/2019/09/17/1207576/original.jpg	https://data.globe.gov/system/photos/2019/09/17/1207578/original.jpg	https://data.globe.gov/system/photos/2019/09/17/1207577/original.jpg	https://data.globe.gov/system/photos/2019/09/17/1207579/original.jpg	https://data.globe.gov/system/photos/2019/09/17/1207581/original.jpg
GV_2358	45.1481	-121.7499	989.8		https://www.google.com/maps/search/45.1481,-121.7499	https://data.globe.gov/system/photos/2019/09/17/1207582/original.jpg	https://data.globe.gov/system/photos/2019/09/17/1207584/original.jpg	https://data.globe.gov/system/photos/2019/09/17/1207583/original.jpg	https://data.globe.gov/system/photos/2019/09/17/1207585/original.jpg	https://data.globe.gov/system/photos/2019/09/17/1207587/original.jpg
GV_2359	45.1438	-121.6986	1090.3		https://www.google.com/maps/search/45.1438,-121.6986	https://data.globe.gov/system/photos/2019/09/17/1207588/original.jpg	https://data.globe.gov/system/photos/2019/09/17/1207590/original.jpg	https://data.globe.gov/system/photos/2019/09/17/1207589/original.jpg	https://data.globe.gov/system/photos/2019/09/17/1207591/original.jpg	https://data.globe.gov/system/photos/2019/09/17/1207593/original.jpg
GV_2360	45.135	-121.7144	1042.4		https://www.google.com/maps/search/45.135,-121.7144	https://data.globe.gov/system/photos/2019/09/17/1207594/original.jpg	https://data.globe.gov/system/photos/2019/09/17/1207596/original.jpg	https://data.globe.gov/system/photos/2019/09/17/1207595/original.jpg	https://data.globe.gov/system/photos/2019/09/17/1207597/original.jpg	https://data.globe.gov/system/photos/2019/09/17/1207599/original.jpg
GV_2361	45.1401	-121.7268	1024.4		https://www.google.com/maps/search/45.1401,-121.7268	https://data.globe.gov/system/photos/2019/09/17/1207600/original.jpg	https://data.globe.gov/system/photos/2019/09/17/1207602/original.jpg	https://data.globe.gov/system/photos/2019/09/17/1207601/original.jpg	https://data.globe.gov/system/photos/2019/09/17/1207603/original.jpg	https://data.globe.gov/system/photos/2019/09/17/1207605/original.jpg
GV_2362	45.1393	-121.727	1022.7		https://www.google.com/maps/search/45.1393,-121.727	https://data.globe.gov/system/photos/2019/09/17/1207606/original.jpg	https://data.globe.gov/system/photos/2019/09/17/1207608/original.jpg	https://data.globe.gov/system/photos/2019/09/17/1207607/original.jpg	https://data.globe.gov/system/photos/2019/09/17/1207609/original.jpg	https://data.globe.gov/system/photos/2019/09/17/1207611/original.jpg
GV_2363	45.1478	-121.7482	988		https://www.google.com/maps/search/45.1478,-121.7482	https://data.globe.gov/system/photos/2019/09/17/1207612/original.jpg	https://data.globe.gov/system/photos/2019/09/17/1207614/original.jpg	https://data.globe.gov/system/photos/2019/09/17/1207613/original.jpg	https://data.globe.gov/system/photos/2019/09/17/1207615/original.jpg	https://data.globe.gov/system/photos/2019/09/17/1207617/original.jpg
GV_2364	45.1482	-121.7494	988.7		https://www.google.com/maps/search/45.1482,-121.7494	https://data.globe.gov/system/photos/2019/09/17/1207618/original.jpg	https://data.globe.gov/system/photos/2019/09/17/1207620/original.jpg	https://data.globe.gov/system/photos/2019/09/17/1207619/original.jpg	https://data.globe.gov/system/photos/2019/09/17/1207621/original.jpg	https://data.globe.gov/system/photos/2019/09/17/1207623/original.jpg
GV_2365	24.4358	118.314	21.6		https://www.google.com/maps/search/24.4358,118.314	https://data.globe.gov/system/photos/2019/09/18/1207729/original.jpg	https://data.globe.gov/system/photos/2019/09/18/1207731/original.jpg	https://data.globe.gov/system/photos/2019/09/18/1207730/original.jpg	https://data.globe.gov/system/photos/2019/09/18/1207732/original.jpg	https://data.globe.gov/system/photos/2019/09/18/1207734/original.jpg
GV_2366	34.6864	135.526	33		https://www.google.com/maps/search/34.6864,135.526	https://data.globe.gov/system/photos/2019/09/18/1208724/original.jpg	https://data.globe.gov/system/photos/2019/09/18/1208726/original.jpg	https://data.globe.gov/system/photos/2019/09/18/1208725/original.jpg	https://data.globe.gov/system/photos/2019/09/18/1208727/original.jpg	https://data.globe.gov/system/photos/2019/09/18/1208729/original.jpg
GV_2367	42.326	-83.2215	185		https://www.google.com/maps/search/42.326,-83.2215	https://data.globe.gov/system/photos/2019/09/18/1209096/original.jpg	https://data.globe.gov/system/photos/2019/09/18/1209102/original.jpg	https://data.globe.gov/system/photos/2019/09/18/1209098/original.jpg	https://data.globe.gov/system/photos/2019/09/18/1209105/original.jpg	https://data.globe.gov/system/photos/2019/09/18/1209107/original.jpg
GV_2368	42.3271	-83.2224	185.3		https://www.google.com/maps/search/42.3271,-83.2224	https://data.globe.gov/system/photos/2019/09/18/1209209/original.jpg	https://data.globe.gov/system/photos/2019/09/18/1209211/original.jpg	https://data.globe.gov/system/photos/2019/09/18/1209210/original.jpg	https://data.globe.gov/system/photos/2019/09/18/1209212/original.jpg	https://data.globe.gov/system/photos/2019/09/18/1209214/original.jpg
GV_2369	45.7808	-123.432	493.7		https://www.google.com/maps/search/45.7808,-123.432	https://data.globe.gov/system/photos/2019/09/18/1209277/original.jpg	https://data.globe.gov/system/photos/2019/09/18/1209279/original.jpg	https://data.globe.gov/system/photos/2019/09/18/1209278/original.jpg	https://data.globe.gov/system/photos/2019/09/18/1209280/original.jpg	https://data.globe.gov/system/photos/2019/09/18/1209282/original.jpg
GV_2370	45.7781	-123.4316	532		https://www.google.com/maps/search/45.7781,-123.4316	https://data.globe.gov/system/photos/2019/09/18/1209283/original.jpg	https://data.globe.gov/system/photos/2019/09/18/1209285/original.jpg	https://data.globe.gov/system/photos/2019/09/18/1209284/original.jpg	https://data.globe.gov/system/photos/2019/09/18/1209286/original.jpg	https://data.globe.gov/system/photos/2019/09/18/1209288/original.jpg
GV_2371	45.7776	-123.4308	528		https://www.google.com/maps/search/45.7776,-123.4308	https://data.globe.gov/system/photos/2019/09/18/1209289/original.jpg	https://data.globe.gov/system/photos/2019/09/18/1209291/original.jpg	https://data.globe.gov/system/photos/2019/09/18/1209290/original.jpg	https://data.globe.gov/system/photos/2019/09/18/1209292/original.jpg	https://data.globe.gov/system/photos/2019/09/18/1209294/original.jpg
GV_2372	46.1823	-123.9625	8.4		https://www.google.com/maps/search/46.1823,-123.9625	https://data.globe.gov/system/photos/2019/09/18/1209295/original.jpg	https://data.globe.gov/system/photos/2019/09/18/1209297/original.jpg	https://data.globe.gov/system/photos/2019/09/18/1209296/original.jpg	https://data.globe.gov/system/photos/2019/09/18/1209298/original.jpg	https://data.globe.gov/system/photos/2019/09/18/1209300/original.jpg
GV_2373	46.2146	-124.0045	3.8		https://www.google.com/maps/search/46.2146,-124.0045	https://data.globe.gov/system/photos/2019/09/18/1209301/original.jpg	https://data.globe.gov/system/photos/2019/09/18/1209303/original.jpg	https://data.globe.gov/system/photos/2019/09/18/1209302/original.jpg	https://data.globe.gov/system/photos/2019/09/18/1209304/original.jpg	https://data.globe.gov/system/photos/2019/09/18/1209306/original.jpg
GV_2374	46.2145	-124.0037	14.9		https://www.google.com/maps/search/46.2145,-124.0037	https://data.globe.gov/system/photos/2019/09/18/1209307/original.jpg	https://data.globe.gov/system/photos/2019/09/18/1209309/original.jpg	https://data.globe.gov/system/photos/2019/09/18/1209308/original.jpg	https://data.globe.gov/system/photos/2019/09/18/1209310/original.jpg	https://data.globe.gov/system/photos/2019/09/18/1209312/original.jpg
GV_2375	46.2161	-124.0046	10.6		https://www.google.com/maps/search/46.2161,-124.0046	https://data.globe.gov/system/photos/2019/09/18/1209313/original.jpg	https://data.globe.gov/system/photos/2019/09/18/1209315/original.jpg	https://data.globe.gov/system/photos/2019/09/18/1209314/original.jpg	https://data.globe.gov/system/photos/2019/09/18/1209316/original.jpg	https://data.globe.gov/system/photos/2019/09/18/1209318/original.jpg
GV_2376	46.2167	-124.0053	14		https://www.google.com/maps/search/46.2167,-124.0053	https://data.globe.gov/system/photos/2019/09/18/1209319/original.jpg	https://data.globe.gov/system/photos/2019/09/18/1209321/original.jpg	https://data.globe.gov/system/photos/2019/09/18/1209320/original.jpg	https://data.globe.gov/system/photos/2019/09/18/1209322/original.jpg	https://data.globe.gov/system/photos/2019/09/18/1209324/original.jpg
GV_2377	46.2182	-124.0042	4.3		https://www.google.com/maps/search/46.2182,-124.0042	https://data.globe.gov/system/photos/2019/09/18/1209330/original.jpg	https://data.globe.gov/system/photos/2019/09/18/1209332/original.jpg	https://data.globe.gov/system/photos/2019/09/18/1209331/original.jpg	https://data.globe.gov/system/photos/2019/09/18/1209333/original.jpg	https://data.globe.gov/system/photos/2019/09/18/1209335/original.jpg
GV_2378	46.2201	-124.0061	4		https://www.google.com/maps/search/46.2201,-124.0061	https://data.globe.gov/system/photos/2019/09/18/1209336/original.jpg	https://data.globe.gov/system/photos/2019/09/18/1209338/original.jpg	https://data.globe.gov/system/photos/2019/09/18/1209337/original.jpg	https://data.globe.gov/system/photos/2019/09/18/1209339/original.jpg	https://data.globe.gov/system/photos/2019/09/18/1209341/original.jpg
GV_2379	46.2201	-124.0074	10		https://www.google.com/maps/search/46.2201,-124.0074	https://data.globe.gov/system/photos/2019/09/18/1209342/original.jpg	https://data.globe.gov/system/photos/2019/09/18/1209344/original.jpg	https://data.globe.gov/system/photos/2019/09/18/1209343/original.jpg	https://data.globe.gov/system/photos/2019/09/18/1209345/original.jpg	https://data.globe.gov/system/photos/2019/09/18/1209347/original.jpg
GV_2380	46.2168	-124.0029	5.6		https://www.google.com/maps/search/46.2168,-124.0029	https://data.globe.gov/system/photos/2019/09/18/1209348/original.jpg	https://data.globe.gov/system/photos/2019/09/18/1209350/original.jpg	https://data.globe.gov/system/photos/2019/09/18/1209349/original.jpg	https://data.globe.gov/system/photos/2019/09/18/1209351/original.jpg	https://data.globe.gov/system/photos/2019/09/18/1209353/original.jpg
GV_2381	46.2058	-123.9965	4.5		https://www.google.com/maps/search/46.2058,-123.9965	https://data.globe.gov/system/photos/2019/09/18/1209354/original.jpg	https://data.globe.gov/system/photos/2019/09/18/1209356/original.jpg	https://data.globe.gov/system/photos/2019/09/18/1209355/original.jpg	https://data.globe.gov/system/photos/2019/09/18/1209357/original.jpg	https://data.globe.gov/system/photos/2019/09/18/1209359/original.jpg
GV_2382	46.2064	-123.9976	9.3		https://www.google.com/maps/search/46.2064,-123.9976	https://data.globe.gov/system/photos/2019/09/18/1209360/original.jpg	https://data.globe.gov/system/photos/2019/09/18/1209362/original.jpg	https://data.globe.gov/system/photos/2019/09/18/1209361/original.jpg	https://data.globe.gov/system/photos/2019/09/18/1209363/original.jpg	https://data.globe.gov/system/photos/2019/09/18/1209365/original.jpg
GV_2383	46.2046	-123.9953	5.6		https://www.google.com/maps/search/46.2046,-123.9953	https://data.globe.gov/system/photos/2019/09/18/1209366/original.jpg	https://data.globe.gov/system/photos/2019/09/18/1209368/original.jpg	https://data.globe.gov/system/photos/2019/09/18/1209367/original.jpg	https://data.globe.gov/system/photos/2019/09/18/1209369/original.jpg	https://data.globe.gov/system/photos/2019/09/18/1209371/original.jpg
GV_2384	46.015	-123.9116	5.8		https://www.google.com/maps/search/46.015,-123.9116	https://data.globe.gov/system/photos/2019/09/18/1209372/original.jpg	https://data.globe.gov/system/photos/2019/09/18/1209374/original.jpg	https://data.globe.gov/system/photos/2019/09/18/1209373/original.jpg	https://data.globe.gov/system/photos/2019/09/18/1209375/original.jpg	https://data.globe.gov/system/photos/2019/09/18/1209377/original.jpg
GV_2385	46.0148	-123.9125	3		https://www.google.com/maps/search/46.0148,-123.9125	https://data.globe.gov/system/photos/2019/09/18/1209378/original.jpg	https://data.globe.gov/system/photos/2019/09/18/1209380/original.jpg	https://data.globe.gov/system/photos/2019/09/18/1209379/original.jpg	https://data.globe.gov/system/photos/2019/09/18/1209381/original.jpg	https://data.globe.gov/system/photos/2019/09/18/1209383/original.jpg
GV_2386	46.0159	-123.9112	6.6		https://www.google.com/maps/search/46.0159,-123.9112	https://data.globe.gov/system/photos/2019/09/18/1209384/original.jpg	https://data.globe.gov/system/photos/2019/09/18/1209386/original.jpg	https://data.globe.gov/system/photos/2019/09/18/1209385/original.jpg	https://data.globe.gov/system/photos/2019/09/18/1209387/original.jpg	https://data.globe.gov/system/photos/2019/09/18/1209389/original.jpg
GV_2387	46.0158	-123.9135	2.9		https://www.google.com/maps/search/46.0158,-123.9135	https://data.globe.gov/system/photos/2019/09/18/1209390/original.jpg	https://data.globe.gov/system/photos/2019/09/18/1209392/original.jpg	https://data.globe.gov/system/photos/2019/09/18/1209391/original.jpg	https://data.globe.gov/system/photos/2019/09/18/1209393/original.jpg	https://data.globe.gov/system/photos/2019/09/18/1209395/original.jpg
GV_2388	45.9629	-123.6901	503.8		https://www.google.com/maps/search/45.9629,-123.6901	https://data.globe.gov/system/photos/2019/09/18/1209396/original.jpg	https://data.globe.gov/system/photos/2019/09/18/1209398/original.jpg	https://data.globe.gov/system/photos/2019/09/18/1209397/original.jpg	https://data.globe.gov/system/photos/2019/09/18/1209399/original.jpg	https://data.globe.gov/system/photos/2019/09/18/1209401/original.jpg
GV_2389	45.9627	-123.6896	519.3		https://www.google.com/maps/search/45.9627,-123.6896	https://data.globe.gov/system/photos/2019/09/18/1209402/original.jpg	https://data.globe.gov/system/photos/2019/09/18/1209404/original.jpg	https://data.globe.gov/system/photos/2019/09/18/1209403/original.jpg	https://data.globe.gov/system/photos/2019/09/18/1209405/original.jpg	https://data.globe.gov/system/photos/2019/09/18/1209407/original.jpg
GV_2390	45.9615	-123.6881	542.5		https://www.google.com/maps/search/45.9615,-123.6881	https://data.globe.gov/system/photos/2019/09/18/1209408/original.jpg	https://data.globe.gov/system/photos/2019/09/18/1209410/original.jpg	https://data.globe.gov/system/photos/2019/09/18/1209409/original.jpg	https://data.globe.gov/system/photos/2019/09/18/1209411/original.jpg	https://data.globe.gov/system/photos/2019/09/18/1209413/original.jpg
GV_2391	45.9637	-123.6906	501.8		https://www.google.com/maps/search/45.9637,-123.6906	https://data.globe.gov/system/photos/2019/09/18/1209414/original.jpg	https://data.globe.gov/system/photos/2019/09/18/1209416/original.jpg	https://data.globe.gov/system/photos/2019/09/18/1209415/original.jpg	https://data.globe.gov/system/photos/2019/09/18/1209417/original.jpg	https://data.globe.gov/system/photos/2019/09/18/1209419/original.jpg
GV_2392	45.9092	-123.7415	153.6		https://www.google.com/maps/search/45.9092,-123.7415	https://data.globe.gov/system/photos/2019/09/18/1209420/original.jpg	https://data.globe.gov/system/photos/2019/09/18/1209422/original.jpg	https://data.globe.gov/system/photos/2019/09/18/1209421/original.jpg	https://data.globe.gov/system/photos/2019/09/18/1209423/original.jpg	https://data.globe.gov/system/photos/2019/09/18/1209425/original.jpg
GV_2393	45.9089	-123.7414	151		https://www.google.com/maps/search/45.9089,-123.7414	https://data.globe.gov/system/photos/2019/09/18/1209426/original.jpg	https://data.globe.gov/system/photos/2019/09/18/1209428/original.jpg	https://data.globe.gov/system/photos/2019/09/18/1209427/original.jpg	https://data.globe.gov/system/photos/2019/09/18/1209429/original.jpg	https://data.globe.gov/system/photos/2019/09/18/1209431/original.jpg
GV_2394	45.8364	-123.5161	403.9		https://www.google.com/maps/search/45.8364,-123.5161	https://data.globe.gov/system/photos/2019/09/18/1209432/original.jpg	https://data.globe.gov/system/photos/2019/09/18/1209434/original.jpg	https://data.globe.gov/system/photos/2019/09/18/1209433/original.jpg	https://data.globe.gov/system/photos/2019/09/18/1209435/original.jpg	https://data.globe.gov/system/photos/2019/09/18/1209437/original.jpg
GV_2395	45.8322	-123.5177	464.1		https://www.google.com/maps/search/45.8322,-123.5177	https://data.globe.gov/system/photos/2019/09/18/1209438/original.jpg	https://data.globe.gov/system/photos/2019/09/18/1209440/original.jpg	https://data.globe.gov/system/photos/2019/09/18/1209439/original.jpg	https://data.globe.gov/system/photos/2019/09/18/1209441/original.jpg	https://data.globe.gov/system/photos/2019/09/18/1209443/original.jpg
GV_2396	45.8315	-123.5216	495.1		https://www.google.com/maps/search/45.8315,-123.5216	https://data.globe.gov/system/photos/2019/09/18/1209444/original.jpg	https://data.globe.gov/system/photos/2019/09/18/1209446/original.jpg	https://data.globe.gov/system/photos/2019/09/18/1209445/original.jpg	https://data.globe.gov/system/photos/2019/09/18/1209447/original.jpg	https://data.globe.gov/system/photos/2019/09/18/1209449/original.jpg
GV_2397	45.8382	-123.5136	365		https://www.google.com/maps/search/45.8382,-123.5136	https://data.globe.gov/system/photos/2019/09/18/1209450/original.jpg	https://data.globe.gov/system/photos/2019/09/18/1209452/original.jpg	https://data.globe.gov/system/photos/2019/09/18/1209451/original.jpg	https://data.globe.gov/system/photos/2019/09/18/1209453/original.jpg	https://data.globe.gov/system/photos/2019/09/18/1209455/original.jpg
GV_2398	24.4357	118.3141	21.9		https://www.google.com/maps/search/24.4357,118.3141	https://data.globe.gov/system/photos/2019/09/19/1209577/original.jpg	https://data.globe.gov/system/photos/2019/09/19/1209579/original.jpg	https://data.globe.gov/system/photos/2019/09/19/1209578/original.jpg	https://data.globe.gov/system/photos/2019/09/19/1209581/original.jpg	https://data.globe.gov/system/photos/2019/09/19/1209587/original.jpg
GV_2399	52.9453	1.1967	49.5		https://www.google.com/maps/search/52.9453,1.1967	https://data.globe.gov/system/photos/2019/09/19/1209749/original.jpg	https://data.globe.gov/system/photos/2019/09/19/1209751/original.jpg	https://data.globe.gov/system/photos/2019/09/19/1209750/original.jpg	https://data.globe.gov/system/photos/2019/09/19/1209752/original.jpg	https://data.globe.gov/system/photos/2019/09/19/1209754/original.jpg
GV_2400	37.1693	-76.4484	4.3		https://www.google.com/maps/search/37.1693,-76.4484	https://data.globe.gov/system/photos/2019/09/19/1209761/original.jpg	https://data.globe.gov/system/photos/2019/09/19/1209766/original.jpg	https://data.globe.gov/system/photos/2019/09/19/1209764/original.jpg	https://data.globe.gov/system/photos/2019/09/19/1209767/original.jpg	https://data.globe.gov/system/photos/2019/09/19/1209769/original.jpg
GV_2401	39.9266	-75.448	107.7		https://www.google.com/maps/search/39.9266,-75.448	https://data.globe.gov/system/photos/2019/09/19/1209996/original.jpg	https://data.globe.gov/system/photos/2019/09/19/1209998/original.jpg	https://data.globe.gov/system/photos/2019/09/19/1209997/original.jpg	https://data.globe.gov/system/photos/2019/09/19/1210000/original.jpg	https://data.globe.gov/system/photos/2019/09/19/1210006/original.jpg
GV_2402	39.9266	-75.4482	107.9		https://www.google.com/maps/search/39.9266,-75.4482	https://data.globe.gov/system/photos/2019/09/19/1209999/original.jpg	https://data.globe.gov/system/photos/2019/09/19/1210005/original.jpg	https://data.globe.gov/system/photos/2019/09/19/1210004/original.jpg	https://data.globe.gov/system/photos/2019/09/19/1210008/original.jpg	https://data.globe.gov/system/photos/2019/09/19/1210012/original.jpg
GV_2403	39.9269	-75.448	108.4		https://www.google.com/maps/search/39.9269,-75.448	https://data.globe.gov/system/photos/2019/09/19/1210001/original.jpg	https://data.globe.gov/system/photos/2019/09/19/1210007/original.jpg	https://data.globe.gov/system/photos/2019/09/19/1210003/original.jpg	https://data.globe.gov/system/photos/2019/09/19/1210009/original.jpg	https://data.globe.gov/system/photos/2019/09/19/1210013/original.jpg
GV_2404	39.928	-75.448	111.5		https://www.google.com/maps/search/39.928,-75.448	https://data.globe.gov/system/photos/2019/09/19/1210019/original.jpg	https://data.globe.gov/system/photos/2019/09/19/1210021/original.jpg	https://data.globe.gov/system/photos/2019/09/19/1210020/original.jpg	https://data.globe.gov/system/photos/2019/09/19/1210022/original.jpg	https://data.globe.gov/system/photos/2019/09/19/1210024/original.jpg
GV_2405	39.9267	-75.4477	105.2		https://www.google.com/maps/search/39.9267,-75.4477	https://data.globe.gov/system/photos/2019/09/19/1210025/original.jpg	https://data.globe.gov/system/photos/2019/09/19/1210027/original.jpg	https://data.globe.gov/system/photos/2019/09/19/1210026/original.jpg	https://data.globe.gov/system/photos/2019/09/19/1210028/original.jpg	https://data.globe.gov/system/photos/2019/09/19/1210030/original.jpg
GV_2406	39.927	-75.4479	108.3		https://www.google.com/maps/search/39.927,-75.4479	https://data.globe.gov/system/photos/2019/09/19/1210031/original.jpg	https://data.globe.gov/system/photos/2019/09/19/1210033/original.jpg	https://data.globe.gov/system/photos/2019/09/19/1210032/original.jpg	https://data.globe.gov/system/photos/2019/09/19/1210034/original.jpg	https://data.globe.gov/system/photos/2019/09/19/1210036/original.jpg
GV_2407	39.9266	-75.4482	107.9		https://www.google.com/maps/search/39.9266,-75.4482	https://data.globe.gov/system/photos/2019/09/19/1210043/original.jpg	https://data.globe.gov/system/photos/2019/09/19/1210045/original.jpg	https://data.globe.gov/system/photos/2019/09/19/1210044/original.jpg	https://data.globe.gov/system/photos/2019/09/19/1210046/original.jpg	https://data.globe.gov/system/photos/2019/09/19/1210048/original.jpg
GV_2408	39.9266	-75.4481	107.9		https://www.google.com/maps/search/39.9266,-75.4481	https://data.globe.gov/system/photos/2019/09/19/1210331/original.jpg	https://data.globe.gov/system/photos/2019/09/19/1210333/original.jpg	https://data.globe.gov/system/photos/2019/09/19/1210332/original.jpg	https://data.globe.gov/system/photos/2019/09/19/1210334/original.jpg	https://data.globe.gov/system/photos/2019/09/19/1210336/original.jpg
GV_2409	39.9266	-75.4481	107.9		https://www.google.com/maps/search/39.9266,-75.4481	https://data.globe.gov/system/photos/2019/09/19/1210337/original.jpg	https://data.globe.gov/system/photos/2019/09/19/1210339/original.jpg	https://data.globe.gov/system/photos/2019/09/19/1210338/original.jpg	https://data.globe.gov/system/photos/2019/09/19/1210340/original.jpg	https://data.globe.gov/system/photos/2019/09/19/1210342/original.jpg
GV_2410	39.392	-76.8249	187.6		https://www.google.com/maps/search/39.392,-76.8249	https://data.globe.gov/system/photos/2019/09/19/1210366/original.jpg	https://data.globe.gov/system/photos/2019/09/19/1210368/original.jpg	https://data.globe.gov/system/photos/2019/09/19/1210367/original.jpg	https://data.globe.gov/system/photos/2019/09/19/1210369/original.jpg	https://data.globe.gov/system/photos/2019/09/19/1210371/original.jpg
GV_2411	39.3988	-76.8255	183.2		https://www.google.com/maps/search/39.3988,-76.8255	https://data.globe.gov/system/photos/2019/09/19/1210422/original.jpg	https://data.globe.gov/system/photos/2019/09/19/1210425/original.jpg	https://data.globe.gov/system/photos/2019/09/19/1210424/original.jpg	https://data.globe.gov/system/photos/2019/09/19/1210426/original.jpg	https://data.globe.gov/system/photos/2019/09/19/1210436/original.jpg
GV_2412	39.9269	-75.4478	107.4		https://www.google.com/maps/search/39.9269,-75.4478	https://data.globe.gov/system/photos/2019/09/19/1211667/original.jpg	https://data.globe.gov/system/photos/2019/09/19/1211670/original.jpg	https://data.globe.gov/system/photos/2019/09/19/1211669/original.jpg	https://data.globe.gov/system/photos/2019/09/19/1211671/original.jpg	https://data.globe.gov/system/photos/2019/09/19/1211673/original.jpg
GV_2413	39.9271	-75.4476	106.8		https://www.google.com/maps/search/39.9271,-75.4476	https://data.globe.gov/system/photos/2019/09/19/1211674/original.jpg	https://data.globe.gov/system/photos/2019/09/19/1211676/original.jpg	https://data.globe.gov/system/photos/2019/09/19/1211675/original.jpg	https://data.globe.gov/system/photos/2019/09/19/1211677/original.jpg	https://data.globe.gov/system/photos/2019/09/19/1211679/original.jpg
GV_2414	-26.77	-51.025	930.3		https://www.google.com/maps/search/-26.77,-51.025	https://data.globe.gov/system/photos/2019/09/20/1211682/original.jpg	https://data.globe.gov/system/photos/2019/09/20/1211684/original.jpg	https://data.globe.gov/system/photos/2019/09/20/1211683/original.jpg	https://data.globe.gov/system/photos/2019/09/20/1211685/original.jpg	https://data.globe.gov/system/photos/2019/09/20/1211687/original.jpg
GV_2415	26.77	51.025	-17		https://www.google.com/maps/search/26.77,51.025	https://data.globe.gov/system/photos/2019/09/20/1211710/original.jpg	https://data.globe.gov/system/photos/2019/09/20/1211712/original.jpg	https://data.globe.gov/system/photos/2019/09/20/1211711/original.jpg	https://data.globe.gov/system/photos/2019/09/20/1211713/original.jpg	https://data.globe.gov/system/photos/2019/09/20/1211715/original.jpg
GV_2416	-5.5352	-36.8715	25		https://www.google.com/maps/search/-5.5352,-36.8715	https://data.globe.gov/system/photos/2019/09/20/1212029/original.jpg	https://data.globe.gov/system/photos/2019/09/20/1212031/original.jpg	https://data.globe.gov/system/photos/2019/09/20/1212030/original.jpg	https://data.globe.gov/system/photos/2019/09/20/1212032/original.jpg	https://data.globe.gov/system/photos/2019/09/20/1212034/original.jpg
GV_2417	42.0859	-83.2098	177.7		https://www.google.com/maps/search/42.0859,-83.2098	https://data.globe.gov/system/photos/2019/09/20/1212684/original.jpg	https://data.globe.gov/system/photos/2019/09/20/1212686/original.jpg	https://data.globe.gov/system/photos/2019/09/20/1212685/original.jpg	https://data.globe.gov/system/photos/2019/09/20/1212687/original.jpg	https://data.globe.gov/system/photos/2019/09/20/1212689/original.jpg
GV_2418	44.8955	-93.1807	216		https://www.google.com/maps/search/44.8955,-93.1807	https://data.globe.gov/system/photos/2019/09/20/1212802/original.jpg	https://data.globe.gov/system/photos/2019/09/20/1212804/original.jpg	https://data.globe.gov/system/photos/2019/09/20/1212803/original.jpg	https://data.globe.gov/system/photos/2019/09/20/1212805/original.jpg	https://data.globe.gov/system/photos/2019/09/20/1212807/original.jpg
GV_2419	44.8942	-93.1815	210		https://www.google.com/maps/search/44.8942,-93.1815	https://data.globe.gov/system/photos/2019/09/20/1212808/original.jpg	https://data.globe.gov/system/photos/2019/09/20/1212810/original.jpg	https://data.globe.gov/system/photos/2019/09/20/1212809/original.jpg	https://data.globe.gov/system/photos/2019/09/20/1212811/original.jpg	https://data.globe.gov/system/photos/2019/09/20/1212813/original.jpg
GV_2420	44.893	-93.1827	245.7		https://www.google.com/maps/search/44.893,-93.1827	https://data.globe.gov/system/photos/2019/09/20/1212814/original.jpg	https://data.globe.gov/system/photos/2019/09/20/1212816/original.jpg	https://data.globe.gov/system/photos/2019/09/20/1212815/original.jpg	https://data.globe.gov/system/photos/2019/09/20/1212817/original.jpg	https://data.globe.gov/system/photos/2019/09/20/1212819/original.jpg
GV_2421	44.8957	-93.1787	234.9		https://www.google.com/maps/search/44.8957,-93.1787	https://data.globe.gov/system/photos/2019/09/20/1212820/original.jpg	https://data.globe.gov/system/photos/2019/09/20/1212822/original.jpg	https://data.globe.gov/system/photos/2019/09/20/1212821/original.jpg	https://data.globe.gov/system/photos/2019/09/20/1212823/original.jpg	https://data.globe.gov/system/photos/2019/09/20/1212825/original.jpg
GV_2422	44.8958	-93.1787	236.2		https://www.google.com/maps/search/44.8958,-93.1787	https://data.globe.gov/system/photos/2019/09/20/1212826/original.jpg	https://data.globe.gov/system/photos/2019/09/20/1212828/original.jpg	https://data.globe.gov/system/photos/2019/09/20/1212827/original.jpg	https://data.globe.gov/system/photos/2019/09/20/1212829/original.jpg	https://data.globe.gov/system/photos/2019/09/20/1212831/original.jpg
GV_2423	44.8961	-93.1794	235.6		https://www.google.com/maps/search/44.8961,-93.1794	https://data.globe.gov/system/photos/2019/09/20/1212832/original.jpg	https://data.globe.gov/system/photos/2019/09/20/1212834/original.jpg	https://data.globe.gov/system/photos/2019/09/20/1212833/original.jpg	https://data.globe.gov/system/photos/2019/09/20/1212835/original.jpg	https://data.globe.gov/system/photos/2019/09/20/1212837/original.jpg

GV_2424	44.8963	-93.1798	236.8		https://www.google.com/maps/search/44.8963,-93.1798	https://data.globe.gov/system/photos/2019/09/20/1212838/original.jpg	https://data.globe.gov/system/photos/2019/09/20/1212840/original.jpg	https://data.globe.gov/system/photos/2019/09/20/1212839/original.jpg	https://data.globe.gov/system/photos/2019/09/20/1212841/original.jpg	https://data.globe.gov/system/photos/2019/09/20/1212843/original.jpg
GV_2425	42.4912	-96.4184	334.5		https://www.google.com/maps/search/42.4912,-96.4184	https://data.globe.gov/system/photos/2019/06/30/1212912/original.jpg	https://data.globe.gov/system/photos/2019/06/30/1212914/original.jpg	https://data.globe.gov/system/photos/2019/06/30/1212913/original.jpg	https://data.globe.gov/system/photos/2019/06/30/1212915/original.jpg	https://data.globe.gov/system/photos/2019/06/30/1212917/original.jpg
GV_2426	44.8982	-93.1655	215.2		https://www.google.com/maps/search/44.8982,-93.1655	https://data.globe.gov/system/photos/2019/09/20/1213087/original.jpg	https://data.globe.gov/system/photos/2019/09/20/1213089/original.jpg	https://data.globe.gov/system/photos/2019/09/20/1213088/original.jpg	https://data.globe.gov/system/photos/2019/09/20/1213090/original.jpg	https://data.globe.gov/system/photos/2019/09/20/1213093/original.jpg
GV_2427	44.8984	-93.1652	215.6		https://www.google.com/maps/search/44.8984,-93.1652	https://data.globe.gov/system/photos/2019/09/20/1213095/original.jpg	https://data.globe.gov/system/photos/2019/09/20/1213097/original.jpg	https://data.globe.gov/system/photos/2019/09/20/1213096/original.jpg	https://data.globe.gov/system/photos/2019/09/20/1213098/original.jpg	https://data.globe.gov/system/photos/2019/09/20/1213100/original.jpg
GV_2428	44.8983	-93.164	216.1		https://www.google.com/maps/search/44.8983,-93.164	https://data.globe.gov/system/photos/2019/09/20/1213102/original.jpg	https://data.globe.gov/system/photos/2019/09/20/1213104/original.jpg	https://data.globe.gov/system/photos/2019/09/20/1213103/original.jpg	https://data.globe.gov/system/photos/2019/09/20/1213105/original.jpg	https://data.globe.gov/system/photos/2019/09/20/1213107/original.jpg
GV_2429	44.898	-93.1646	214.7		https://www.google.com/maps/search/44.898,-93.1646	https://data.globe.gov/system/photos/2019/09/20/1213108/original.jpg	https://data.globe.gov/system/photos/2019/09/20/1213110/original.jpg	https://data.globe.gov/system/photos/2019/09/20/1213109/original.jpg	https://data.globe.gov/system/photos/2019/09/20/1213111/original.jpg	https://data.globe.gov/system/photos/2019/09/20/1213113/original.jpg
GV_2430	44.897	-93.165	214.6		https://www.google.com/maps/search/44.897,-93.165	https://data.globe.gov/system/photos/2019/09/20/1213114/original.jpg	https://data.globe.gov/system/photos/2019/09/20/1213116/original.jpg	https://data.globe.gov/system/photos/2019/09/20/1213115/original.jpg	https://data.globe.gov/system/photos/2019/09/20/1213117/original.jpg	https://data.globe.gov/system/photos/2019/09/20/1213119/original.jpg
GV_2431	44.8972	-93.1661	213.8		https://www.google.com/maps/search/44.8972,-93.1661	https://data.globe.gov/system/photos/2019/09/20/1213120/original.jpg	https://data.globe.gov/system/photos/2019/09/20/1213122/original.jpg	https://data.globe.gov/system/photos/2019/09/20/1213121/original.jpg	https://data.globe.gov/system/photos/2019/09/20/1213123/original.jpg	https://data.globe.gov/system/photos/2019/09/20/1213125/original.jpg
GV_2432	-35.1239	139.2845	5.9		https://www.google.com/maps/search/-35.1239,139.2845	https://data.globe.gov/system/photos/2019/09/21/1213131/original.jpg	https://data.globe.gov/system/photos/2019/09/21/1213133/original.jpg	https://data.globe.gov/system/photos/2019/09/21/1213132/original.jpg	https://data.globe.gov/system/photos/2019/09/21/1213134/original.jpg	https://data.globe.gov/system/photos/2019/09/21/1213136/original.jpg
GV_2433	6.182	-75.6462	1775.5		https://www.google.com/maps/search/6.182,-75.6462	https://data.globe.gov/system/photos/2019/09/21/1213482/original.jpg	https://data.globe.gov/system/photos/2019/09/21/1213484/original.jpg	https://data.globe.gov/system/photos/2019/09/21/1213483/original.jpg	https://data.globe.gov/system/photos/2019/09/21/1213485/original.jpg	https://data.globe.gov/system/photos/2019/09/21/1213487/original.jpg
GV_2434	45.0289	-92.8648	287.2		https://www.google.com/maps/search/45.0289,-92.8648	https://data.globe.gov/system/photos/2019/09/21/1213756/original.jpg	https://data.globe.gov/system/photos/2019/09/21/1213758/original.jpg	https://data.globe.gov/system/photos/2019/09/21/1213757/original.jpg	https://data.globe.gov/system/photos/2019/09/21/1213759/original.jpg	https://data.globe.gov/system/photos/2019/09/21/1213761/original.jpg
GV_2435	45.0322	-92.8663	287.3		https://www.google.com/maps/search/45.0322,-92.8663	https://data.globe.gov/system/photos/2019/09/21/1213762/original.jpg	https://data.globe.gov/system/photos/2019/09/21/1213764/original.jpg	https://data.globe.gov/system/photos/2019/09/21/1213763/original.jpg	https://data.globe.gov/system/photos/2019/09/21/1213765/original.jpg	https://data.globe.gov/system/photos/2019/09/21/1213767/original.jpg
GV_2436	45.0325	-92.8643	284.3		https://www.google.com/maps/search/45.0325,-92.8643	https://data.globe.gov/system/photos/2019/09/21/1213768/original.jpg	https://data.globe.gov/system/photos/2019/09/21/1213770/original.jpg	https://data.globe.gov/system/photos/2019/09/21/1213769/original.jpg	https://data.globe.gov/system/photos/2019/09/21/1213771/original.jpg	https://data.globe.gov/system/photos/2019/09/21/1213773/original.jpg
GV_2437	45.0336	-92.8632	288.3		https://www.google.com/maps/search/45.0336,-92.8632	https://data.globe.gov/system/photos/2019/09/21/1213774/original.jpg	https://data.globe.gov/system/photos/2019/09/21/1213782/original.jpg	https://data.globe.gov/system/photos/2019/09/21/1213776/original.jpg	https://data.globe.gov/system/photos/2019/09/21/1213786/original.jpg	https://data.globe.gov/system/photos/2019/09/21/1213791/original.jpg
GV_2438	45.0299	-92.8629	283.1		https://www.google.com/maps/search/45.0299,-92.8629	https://data.globe.gov/system/photos/2019/09/21/1213801/original.jpg	https://data.globe.gov/system/photos/2019/09/21/1213805/original.jpg	https://data.globe.gov/system/photos/2019/09/21/1213803/original.jpg	https://data.globe.gov/system/photos/2019/09/21/1213809/original.jpg	https://data.globe.gov/system/photos/2019/09/21/1213814/original.jpg
GV_2439	51.9755	-109.3272	669		https://www.google.com/maps/search/51.9755,-109.3272	https://data.globe.gov/system/photos/2019/09/21/1214031/original.jpg	https://data.globe.gov/system/photos/2019/09/21/1214033/original.jpg	https://data.globe.gov/system/photos/2019/09/21/1214032/original.jpg	https://data.globe.gov/system/photos/2019/09/21/1214034/original.jpg	https://data.globe.gov/system/photos/2019/09/21/1214036/original.jpg
GV_2440	-22.4125	115.0124	39.9		https://www.google.com/maps/search/-22.4125,115.0124	https://data.globe.gov/system/photos/2019/09/21/1214163/original.jpg	https://data.globe.gov/system/photos/2019/09/21/1214165/original.jpg	https://data.globe.gov/system/photos/2019/09/21/1214164/original.jpg	https://data.globe.gov/system/photos/2019/09/21/1214166/original.jpg	https://data.globe.gov/system/photos/2019/09/21/1214168/original.jpg
GV_2441	-21.7183	115.1043	6.9		https://www.google.com/maps/search/-21.7183,115.1043	https://data.globe.gov/system/photos/2019/09/22/1214169/original.jpg	https://data.globe.gov/system/photos/2019/09/22/1214171/original.jpg	https://data.globe.gov/system/photos/2019/09/22/1214170/original.jpg	https://data.globe.gov/system/photos/2019/09/22/1214172/original.jpg	https://data.globe.gov/system/photos/2019/09/22/1214174/original.jpg
GV_2442	-35.0335	139.2059	69.7		https://www.google.com/maps/search/-35.0335,139.2059	https://data.globe.gov/system/photos/2019/09/22/1214213/original.jpg	https://data.globe.gov/system/photos/2019/09/22/1214215/original.jpg	https://data.globe.gov/system/photos/2019/09/22/1214214/original.jpg	https://data.globe.gov/system/photos/2019/09/22/1214216/original.jpg	https://data.globe.gov/system/photos/2019/09/22/1214218/original.jpg
GV_2443	-34.3384	138.913	243.5		https://www.google.com/maps/search/-34.3384,138.913	https://data.globe.gov/system/photos/2019/09/07/1214221/original.jpg	https://data.globe.gov/system/photos/2019/09/07/1214223/original.jpg	https://data.globe.gov/system/photos/2019/09/07/1214222/original.jpg	https://data.globe.gov/system/photos/2019/09/07/1214224/original.jpg	https://data.globe.gov/system/photos/2019/09/07/1214226/original.jpg
GV_2444	-35.0779	139.2307	39.1		https://www.google.com/maps/search/-35.0779,139.2307	https://data.globe.gov/system/photos/2019/09/22/1214227/original.jpg	https://data.globe.gov/system/photos/2019/09/22/1214229/original.jpg	https://data.globe.gov/system/photos/2019/09/22/1214228/original.jpg	https://data.globe.gov/system/photos/2019/09/22/1214230/original.jpg	https://data.globe.gov/system/photos/2019/09/22/1214232/original.jpg
GV_2445	-35.0428	139.2145	57.5		https://www.google.com/maps/search/-35.0428,139.2145	https://data.globe.gov/system/photos/2019/09/22/1214235/original.jpg	https://data.globe.gov/system/photos/2019/09/22/1214237/original.jpg	https://data.globe.gov/system/photos/2019/09/22/1214236/original.jpg	https://data.globe.gov/system/photos/2019/09/22/1214238/original.jpg	https://data.globe.gov/system/photos/2019/09/22/1214240/original.jpg
GV_2446	-21.7749	115.2105	9.5		https://www.google.com/maps/search/-21.7749,115.2105	https://data.globe.gov/system/photos/2019/09/22/1214247/original.jpg	https://data.globe.gov/system/photos/2019/09/22/1214249/original.jpg	https://data.globe.gov/system/photos/2019/09/22/1214248/original.jpg	https://data.globe.gov/system/photos/2019/09/22/1214250/original.jpg	https://data.globe.gov/system/photos/2019/09/22/1214252/original.jpg
GV_2447	42.1551	-83.1513	174.9		https://www.google.com/maps/search/42.1551,-83.1513	https://data.globe.gov/system/photos/2019/09/22/1214699/original.jpg	https://data.globe.gov/system/photos/2019/09/22/1214701/original.jpg	https://data.globe.gov/system/photos/2019/09/22/1214700/original.jpg	https://data.globe.gov/system/photos/2019/09/22/1214702/original.jpg	https://data.globe.gov/system/photos/2019/09/22/1214704/original.jpg
GV_2448	39.1858	-86.7786	234.4		https://www.google.com/maps/search/39.1858,-86.7786	https://data.globe.gov/system/photos/2019/09/22/1215122/original.jpg	https://data.globe.gov/system/photos/2019/09/22/1215124/original.jpg	https://data.globe.gov/system/photos/2019/09/22/1215123/original.jpg	https://data.globe.gov/system/photos/2019/09/22/1215125/original.jpg	https://data.globe.gov/system/photos/2019/09/22/1215127/original.jpg
GV_2449	-34.5208	138.9849	279		https://www.google.com/maps/search/-34.5208,138.9849	https://data.globe.gov/system/photos/2019/09/22/1215291/original.jpg	https://data.globe.gov/system/photos/2019/09/22/1215293/original.jpg	https://data.globe.gov/system/photos/2019/09/22/1215292/original.jpg	https://data.globe.gov/system/photos/2019/09/22/1215294/original.jpg	https://data.globe.gov/system/photos/2019/09/22/1215296/original.jpg
GV_2450	-34.5372	138.9994	409.8		https://www.google.com/maps/search/-34.5372,138.9994	https://data.globe.gov/system/photos/2019/09/22/1215297/original.jpg	https://data.globe.gov/system/photos/2019/09/22/1215299/original.jpg	https://data.globe.gov/system/photos/2019/09/22/1215298/original.jpg	https://data.globe.gov/system/photos/2019/09/22/1215300/original.jpg	https://data.globe.gov/system/photos/2019/09/22/1215302/original.jpg
GV_2451	-34.5824	139.0247	493.2		https://www.google.com/maps/search/-34.5824,139.0247	https://data.globe.gov/system/photos/2019/09/22/1215321/original.jpg	https://data.globe.gov/system/photos/2019/09/22/1215323/original.jpg	https://data.globe.gov/system/photos/2019/09/22/1215322/original.jpg	https://data.globe.gov/system/photos/2019/09/22/1215324/original.jpg	https://data.globe.gov/system/photos/2019/09/22/1215326/original.jpg
GV_2452	-34.614	139.0703	403.7		https://www.google.com/maps/search/-34.614,139.0703	https://data.globe.gov/system/photos/2019/09/22/1215327/original.jpg	https://data.globe.gov/system/photos/2019/09/22/1215329/original.jpg	https://data.globe.gov/system/photos/2019/09/22/1215328/original.jpg	https://data.globe.gov/system/photos/2019/09/22/1215330/original.jpg	https://data.globe.gov/system/photos/2019/09/22/1215332/original.jpg
GV_2453	-34.6788	139.0992	350.3		https://www.google.com/maps/search/-34.6788,139.0992	https://data.globe.gov/system/photos/2019/09/22/1215333/original.jpg	https://data.globe.gov/system/photos/2019/09/22/1215335/original.jpg	https://data.globe.gov/system/photos/2019/09/22/1215334/original.jpg	https://data.globe.gov/system/photos/2019/09/22/1215336/original.jpg	https://data.globe.gov/system/photos/2019/09/22/1215338/original.jpg
GV_2454	-21.6577	115.8763	95.3		https://www.google.com/maps/search/-21.6577,115.8763	https://data.globe.gov/system/photos/2019/09/22/1215432/original.jpg	https://data.globe.gov/system/photos/2019/09/22/1215434/original.jpg	https://data.globe.gov/system/photos/2019/09/22/1215433/original.jpg	https://data.globe.gov/system/photos/2019/09/22/1215435/original.jpg	https://data.globe.gov/system/photos/2019/09/22/1215437/original.jpg
GV_2455	-24.8028	28.9448	990.6		https://www.google.com/maps/search/-24.8028,28.9448	https://data.globe.gov/system/photos/2019/09/22/1215547/original.jpg	https://data.globe.gov/system/photos/2019/09/22/1215549/original.jpg	https://data.globe.gov/system/photos/2019/09/22/1215548/original.jpg	https://data.globe.gov/system/photos/2019/09/22/1215550/original.jpg	https://data.globe.gov/system/photos/2019/09/22/1215552/original.jpg
GV_2456	40.4223	-111.6193	2550.7		https://www.google.com/maps/search/40.4223,-111.6193	https://data.globe.gov/system/photos/2019/09/21/1216118/original.jpg	https://data.globe.gov/system/photos/2019/09/21/1216120/original.jpg	https://data.globe.gov/system/photos/2019/09/21/1216119/original.jpg	https://data.globe.gov/system/photos/2019/09/21/1216121/original.jpg	https://data.globe.gov/system/photos/2019/09/21/1216123/original.jpg
GV_2457	40.4208	-111.6192	2556.3		https://www.google.com/maps/search/40.4208,-111.6192	https://data.globe.gov/system/photos/2019/09/21/1216124/original.jpg	https://data.globe.gov/system/photos/2019/09/21/1216126/original.jpg	https://data.globe.gov/system/photos/2019/09/21/1216125/original.jpg	https://data.globe.gov/system/photos/2019/09/21/1216127/original.jpg	https://data.globe.gov/system/photos/2019/09/21/1216129/original.jpg
GV_2458	40.4188	-111.6167	2559.9		https://www.google.com/maps/search/40.4188,-111.6167	https://data.globe.gov/system/photos/2019/09/21/1216130/original.jpg	https://data.globe.gov/system/photos/2019/09/21/1216132/original.jpg	https://data.globe.gov/system/photos/2019/09/21/1216131/original.jpg	https://data.globe.gov/system/photos/2019/09/21/1216133/original.jpg	https://data.globe.gov/system/photos/2019/09/21/1216135/original.jpg
GV_2459	40.4218	-111.6152	2552.2		https://www.google.com/maps/search/40.4218,-111.6152	https://data.globe.gov/system/photos/2019/09/21/1216138/original.jpg	https://data.globe.gov/system/photos/2019/09/21/1216140/original.jpg	https://data.globe.gov/system/photos/2019/09/21/1216139/original.jpg	https://data.globe.gov/system/photos/2019/09/21/1216141/original.jpg	https://data.globe.gov/system/photos/2019/09/21/1216143/original.jpg
GV_2460	38.8849	-76.8204	44.3		https://www.google.com/maps/search/38.8849,-76.8204	https://data.globe.gov/system/photos/2019/09/23/1216326/original.jpg	https://data.globe.gov/system/photos/2019/09/23/1216328/original.jpg	https://data.globe.gov/system/photos/2019/09/23/1216327/original.jpg	https://data.globe.gov/system/photos/2019/09/23/1216329/original.jpg	https://data.globe.gov/system/photos/2019/09/23/1216331/original.jpg
GV_2461	39.1856	-86.7785	236.3		https://www.google.com/maps/search/39.1856,-86.7785	https://data.globe.gov/system/photos/2019/09/24/1217317/original.jpg	https://data.globe.gov/system/photos/2019/09/24/1217319/original.jpg	https://data.globe.gov/system/photos/2019/09/24/1217318/original.jpg	https://data.globe.gov/system/photos/2019/09/24/1217320/original.jpg	https://data.globe.gov/system/photos/2019/09/24/1217322/original.jpg
GV_2462	42.0856	-83.2096	177.1		https://www.google.com/maps/search/42.0856,-83.2096	https://data.globe.gov/system/photos/2019/09/23/1217347/original.jpg	https://data.globe.gov/system/photos/2019/09/23/1217349/original.jpg	https://data.globe.gov/system/photos/2019/09/23/1217348/original.jpg	https://data.globe.gov/system/photos/2019/09/23/1217350/original.jpg	https://data.globe.gov/system/photos/2019/09/23/1217352/original.jpg
GV_2463	2.9086	-75.2656	453.1		https://www.google.com/maps/search/2.9086,-75.2656	https://data.globe.gov/system/photos/2019/09/24/1217549/original.jpg	https://data.globe.gov/system/photos/2019/09/24/1217551/original.jpg	https://data.globe.gov/system/photos/2019/09/24/1217550/original.jpg	https://data.globe.gov/system/photos/2019/09/24/1217552/original.jpg	https://data.globe.gov/system/photos/2019/09/24/1217554/original.jpg
GV_2464	46.2883	16.3353	171.6		https://www.google.com/maps/search/46.2883,16.3353	https://data.globe.gov/system/photos/2019/09/25/1218059/original.jpg	https://data.globe.gov/system/photos/2019/09/25/1218061/original.jpg	https://data.globe.gov/system/photos/2019/09/25/1218060/original.jpg	https://data.globe.gov/system/photos/2019/09/25/1218062/original.jpg	https://data.globe.gov/system/photos/2019/09/25/1218064/original.jpg
GV_2465	-18.6519	121.9367	48.8		https://www.google.com/maps/search/-18.6519,121.9367	https://data.globe.gov/system/photos/2019/09/24/1218137/original.jpg	https://data.globe.gov/system/photos/2019/09/24/1218139/original.jpg	https://data.globe.gov/system/photos/2019/09/24/1218138/original.jpg	https://data.globe.gov/system/photos/2019/09/24/1218140/original.jpg	https://data.globe.gov/system/photos/2019/09/24/1218142/original.jpg
GV_2466	42.8201	-75.5382	337.6		https://www.google.com/maps/search/42.8201,-75.5382	https://data.globe.gov/system/photos/2019/09/25/1218326/original.jpg	https://data.globe.gov/system/photos/2019/09/25/1218328/original.jpg	https://data.globe.gov/system/photos/2019/09/25/1218327/original.jpg	https://data.globe.gov/system/photos/2019/09/25/1218329/original.jpg	https://data.globe.gov/system/photos/2019/09/25/1218331/original.jpg
GV_2467	42.823	-75.5388	338.6		https://www.google.com/maps/search/42.823,-75.5388	https://data.globe.gov/system/photos/2019/09/25/1218445/original.jpg	https://data.globe.gov/system/photos/2019/09/25/1218448/original.jpg	https://data.globe.gov/system/photos/2019/09/25/1218446/original.jpg	https://data.globe.gov/system/photos/2019/09/25/1218451/original.jpg	https://data.globe.gov/system/photos/2019/09/25/1218455/original.jpg
GV_2468	42.8212	-75.5369	339.3		https://www.google.com/maps/search/42.8212,-75.5369	https://data.globe.gov/system/photos/2019/09/25/1218473/original.jpg	https://data.globe.gov/system/photos/2019/09/25/1218476/original.jpg	https://data.globe.gov/system/photos/2019/09/25/1218474/original.jpg	https://data.globe.gov/system/photos/2019/09/25/1218477/original.jpg	https://data.globe.gov/system/photos/2019/09/25/1218481/original.jpg
GV_2469	-26.7848	-50.9756	996.1		https://www.google.com/maps/search/-26.7848,-50.9756	https://data.globe.gov/system/photos/2019/09/25/1218963/original.jpg	https://data.globe.gov/system/photos/2019/09/25/1218965/original.jpg	https://data.globe.gov/system/photos/2019/09/25/1218964/original.jpg	https://data.globe.gov/system/photos/2019/09/25/1218966/original.jpg	https://data.globe.gov/system/photos/2019/09/25/1218968/original.jpg
GV_2470	42.0857	-83.2096	177.3		https://www.google.com/maps/search/42.0857,-83.2096	https://data.globe.gov/system/photos/2019/09/25/1219069/original.jpg	https://data.globe.gov/system/photos/2019/09/25/1219071/original.jpg	https://data.globe.gov/system/photos/2019/09/25/1219070/original.jpg	https://data.globe.gov/system/photos/2019/09/25/1219072/original.jpg	https://data.globe.gov/system/photos/2019/09/25/1219074/original.jpg
GV_2471	45.0583	-92.8049	207.8		https://www.google.com/maps/search/45.0583,-92.8049	https://data.globe.gov/system/photos/2019/09/25/1219150/original.jpg	https://data.globe.gov/system/photos/2019/09/25/1219158/original.jpg	https://data.globe.gov/system/photos/2019/09/25/1219151/original.jpg	https://data.globe.gov/system/photos/2019/09/25/1219159/original.jpg	https://data.globe.gov/system/photos/2019/09/25/1219161/original.jpg
GV_2472	45.0567	-92.8061	211.8		https://www.google.com/maps/search/45.0567,-92.8061	https://data.globe.gov/system/photos/2019/09/24/1219168/original.jpg	https://data.globe.gov/system/photos/2019/09/24/1219170/original.jpg	https://data.globe.gov/system/photos/2019/09/24/1219169/original.jpg	https://data.globe.gov/system/photos/2019/09/24/1219171/original.jpg	https://data.globe.gov/system/photos/2019/09/24/1219173/original.jpg
GV_2473	45.4906	-93.2481	284.2		https://www.google.com/maps/search/45.4906,-93.2481	https://data.globe.gov/system/photos/2019/09/24/1219174/original.jpg	https://data.globe.gov/system/photos/2019/09/24/1219176/original.jpg	https://data.globe.gov/system/photos/2019/09/24/1219175/original.jpg	https://data.globe.gov/system/photos/2019/09/24/1219177/original.jpg	https://data.globe.gov/system/photos/2019/09/24/1219179/original.jpg
GV_2474	42.8151	-75.5358	396.6		https://www.google.com/maps/search/42.8151,-75.5358	https://data.globe.gov/system/photos/2019/09/25/1219186/original.jpg	https://data.globe.gov/system/photos/2019/09/25/1219188/original.jpg	https://data.globe.gov/system/photos/2019/09/25/1219187/original.jpg	https://data.globe.gov/system/photos/2019/09/25/1219189/original.jpg	https://data.globe.gov/system/photos/2019/09/25/1219191/original.jpg
GV_2475	42.8206	-75.853	555.2		https://www.google.com/maps/search/42.8206,-75.853	https://data.globe.gov/system/photos/2019/09/25/1219282/original.jpg	https://data.globe.gov/system/photos/2019/09/25/1219284/original.jpg	https://data.globe.gov/system/photos/2019/09/25/1219283/original.jpg	https://data.globe.gov/system/photos/2019/09/25/1219285/original.jpg	https://data.globe.gov/system/photos/2019/09/25/1219287/original.jpg
GV_2476	45.0546	-92.8053	211.8		https://www.google.com/maps/search/45.0546,-92.8053	https://data.globe.gov/system/photos/2019/09/25/1219326/original.jpg	https://data.globe.gov/system/photos/2019/09/25/1219328/original.jpg	https://data.globe.gov/system/photos/2019/09/25/1219327/original.jpg	https://data.globe.gov/system/photos/2019/09/25/1219329/original.jpg	https://data.globe.gov/system/photos/2019/09/25/1219331/original.jpg
GV_2477	45.0288	-92.8584	287.9		https://www.google.com/maps/search/45.0288,-92.8584	https://data.globe.gov/system/photos/2019/09/25/1219332/original.jpg	https://data.globe.gov/system/photos/2019/09/25/1219334/original.jpg	https://data.globe.gov/system/photos/2019/09/25/1219333/original.jpg	https://data.globe.gov/system/photos/2019/09/25/1219335/original.jpg	https://data.globe.gov/system/photos/2019/09/25/1219337/original.jpg
GV_2478	-34.5258	138.9534	257.8		https://www.google.com/maps/search/-34.5258,138.9534	https://data.globe.gov/system/photos/2019/09/26/1219630/original.jpg	https://data.globe.gov/system/photos/2019/09/26/1219632/original.jpg	https://data.globe.gov/system/photos/2019/09/26/1219631/original.jpg	https://data.globe.gov/system/photos/2019/09/26/1219633/original.jpg	https://data.globe.gov/system/photos/2019/09/26/1219635/original.jpg
GV_2479	-34.5367	138.9347	274.3		https://www.google.com/maps/search/-34.5367,138.9347	https://data.globe.gov/system/photos/2019/09/26/1219636/original.jpg	https://data.globe.gov/system/photos/2019/09/26/1219638/original.jpg	https://data.globe.gov/system/photos/2019/09/26/1219637/original.jpg	https://data.globe.gov/system/photos/2019/09/26/1219639/original.jpg	https://data.globe.gov/system/photos/2019/09/26/1219641/original.jpg
GV_2480	-34.5348	138.9138	218.6		https://www.google.com/maps/search/-34.5348,138.9138	https://data.globe.gov/system/photos/2019/09/26/1219642/original.jpg	https://data.globe.gov/system/photos/2019/09/26/1219644/original.jpg	https://data.globe.gov/system/photos/2019/09/26/1219643/original.jpg	https://data.globe.gov/system/photos/2019/09/26/1219645/original.jpg	https://data.globe.gov/system/photos/2019/09/26/1219647/original.jpg
GV_2481	50.5254	21.6887	146		https://www.google.com/maps/search/50.5254,21.6887	https://data.globe.gov/system/photos/2019/09/26/1220026/original.jpg	https://data.globe.gov/system/photos/2019/09/26/1220028/original.jpg	https://data.globe.gov/system/photos/2019/09/26/1220027/original.jpg	https://data.globe.gov/system/photos/2019/09/26/1220029/original.jpg	https://data.globe.gov/system/photos/2019/09/26/1220031/original.jpg
GV_2482	50.5261	21.6891	146		https://www.google.com/maps/search/50.5261,21.6891	https://data.globe.gov/system/photos/2019/09/26/1220032/original.jpg	https://data.globe.gov/system/photos/2019/09/26/1220034/original.jpg	https://data.globe.gov/system/photos/2019/09/26/1220033/original.jpg	https://data.globe.gov/system/photos/2019/09/26/1220035/original.jpg	https://data.globe.gov/system/photos/2019/09/26/1220037/original.jpg
GV_2483	50.5276	21.6897	146.2		https://www.google.com/maps/search/50.5276,21.6897	https://data.globe.gov/system/photos/2019/09/26/1220038/original.jpg	https://data.globe.gov/system/photos/2019/09/26/1220040/original.jpg	https://data.globe.gov/system/photos/2019/09/26/1220039/original.jpg	https://data.globe.gov/system/photos/2019/09/26/1220041/original.jpg	https://data.globe.gov/system/photos/2019/09/26/1220043/original.jpg
GV_2484	50.5268	21.6901	146		https://www.google.com/maps/search/50.5268,21.6901	https://data.globe.gov/system/photos/2019/09/26/1220044/original.jpg	https://data.globe.gov/system/photos/2019/09/26/1220046/original.jpg	https://data.globe.gov/system/photos/2019/09/26/1220045/original.jpg	https://data.globe.gov/system/photos/2019/09/26/1220047/original.jpg	https://data.globe.gov/system/photos/2019/09/26/1220049/original.jpg
GV_2485	50.527	21.6893	146.7		https://www.google.com/maps/search/50.527,21.6893	https://data.globe.gov/system/photos/2019/09/26/1220050/original.jpg	https://data.globe.gov/system/photos/2019/09/26/1220052/original.jpg	https://data.globe.gov/system/photos/2019/09/26/1220051/original.jpg	https://data.globe.gov/system/photos/2019/09/26/1220053/original.jpg	https://data.globe.gov/system/photos/2019/09/26/1220055/original.jpg
GV_2486	50.5282	21.69	147		https://www.google.com/maps/search/50.5282,21.69	https://data.globe.gov/system/photos/2019/09/26/1220056/original.jpg	https://data.globe.gov/system/photos/2019/09/26/1220058/original.jpg	https://data.globe.gov/system/photos/2019/09/26/1220057/original.jpg	https://data.globe.gov/system/photos/2019/09/26/1220059/original.jpg	https://data.globe.gov/system/photos/2019/09/26/1220061/original.jpg
GV_2487	50.5282	21.69	147		https://www.google.com/maps/search/50.5282,21.69	https://data.globe.gov/system/photos/2019/09/26/1220062/original.jpg	https://data.globe.gov/system/photos/2019/09/26/1220064/original.jpg	https://data.globe.gov/system/photos/2019/09/26/1220063/original.jpg	https://data.globe.gov/system/photos/2019/09/26/1220065/original.jpg	https://data.globe.gov/system/photos/2019/09/26/1220067/original.jpg
GV_2488	50.532	21.6918	146.5		https://www.google.com/maps/search/50.532,21.6918	https://data.globe.gov/system/photos/2019/09/26/1220068/original.jpg	https://data.globe.gov/system/photos/2019/09/26/1220070/original.jpg	https://data.globe.gov/system/photos/2019/09/26/1220069/original.jpg	https://data.globe.gov/system/photos/2019/09/26/1220071/original.jpg	https://data.globe.gov/system/photos/2019/09/26/1220073/original.jpg
GV_2489	50.532	21.6935	146.3		https://www.google.com/maps/search/50.532,21.6935	https://data.globe.gov/system/photos/2019/09/26/1220086/original.jpg	https://data.globe.gov/system/photos/2019/09/26/1220088/original.jpg	https://data.globe.gov/system/photos/2019/09/26/1220087/original.jpg	https://data.globe.gov/system/photos/2019/09/26/1220089/original.jpg	https://data.globe.gov/system/photos/2019/09/26/1220091/original.jpg
GV_2490	50.533	21.6935	146		https://www.google.com/maps/search/50.533,21.6935	https://data.globe.gov/system/photos/2019/09/26/1220092/original.jpg	https://data.globe.gov/system/photos/2019/09/26/1220094/original.jpg	https://data.globe.gov/system/photos/2019/09/26/1220093/original.jpg	https://data.globe.gov/system/photos/2019/09/26/1220095/original.jpg	https://data.globe.gov/system/photos/2019/09/26/1220097/original.jpg
GV_2491	50.5335	21.6933	146		https://www.google.com/maps/search/50.5335,21.6933	https://data.globe.gov/system/photos/2019/09/26/1220104/original.jpg	https://data.globe.gov/system/photos/2019/09/26/1220106/original.jpg	https://data.globe.gov/system/photos/2019/09/26/1220105/original.jpg	https://data.globe.gov/system/photos/2019/09/26/1220107/original.jpg	https://data.globe.gov/system/photos/2019/09/26/1220109/original.jpg
GV_2492	50.5344	21.6938	147		https://www.google.com/maps/search/50.5344,21.6938	https://data.globe.gov/system/photos/2019/09/26/1220111/original.jpg	https://data.globe.gov/system/photos/2019/09/26/1220113/original.jpg	https://data.globe.gov/system/photos/2019/09/26/1220112/original.jpg	https://data.globe.gov/system/photos/2019/09/26/1220114/original.jpg	https://data.globe.gov/system/photos/2019/09/26/1220116/original.jpg
GV_2493	50.5344	21.6938	147		https://www.google.com/maps/search/50.5344,21.6938	https://data.globe.gov/system/photos/2019/09/26/1220117/original.jpg	https://data.globe.gov/system/photos/2019/09/26/1220119/original.jpg	https://data.globe.gov/system/photos/2019/09/26/1220118/original.jpg	https://data.globe.gov/system/photos/2019/09/26/1220120/original.jpg	https://data.globe.gov/system/photos/2019/09/26/1220122/original.jpg
GV_2494	50.5368	21.6948	146.7		https://www.google.com/maps/search/50.5368,21.6948	https://data.globe.gov/system/photos/2019/09/26/1220123/original.jpg	https://data.globe.gov/system/photos/2019/09/26/1220125/original.jpg	https://data.globe.gov/system/photos/2019/09/26/1220124/original.jpg	https://data.globe.gov/system/photos/2019/09/26/1220126/original.jpg	https://data.globe.gov/system/photos/2019/09/26/1220128/original.jpg
GV_2495	50.5371	21.6963	146.5		https://www.google.com/maps/search/50.5371,21.6963	https://data.globe.gov/system/photos/2019/09/26/1220130/original.jpg	https://data.globe.gov/system/photos/2019/09/26/1220132/original.jpg	https://data.globe.gov/system/photos/2019/09/26/1220131/original.jpg	https://data.globe.gov/system/photos/2019/09/26/1220133/original.jpg	https://data.globe.gov/system/photos/2019/09/26/1220135/original.jpg
GV_2496	50.5371	21.6918	147		https://www.google.com/maps/search/50.5371,21.6918	https://data.globe.gov/system/photos/2019/09/26/1220136/original.jpg	https://data.globe.gov/system/photos/2019/09/26/1220138/original.jpg	https://data.globe.gov/system/photos/2019/09/26/1220137/original.jpg	https://data.globe.gov/system/photos/2019/09/26/1220139/original.jpg	https://data.globe.gov/system/photos/2019/09/26/1220141/original.jpg
GV_2497	50.5365	21.688	149.6		https://www.google.com/maps/search/50.5365,21.688	https://data.globe.gov/system/photos/2019/09/26/1220142/original.jpg	https://data.globe.gov/system/photos/2019/09/26/1220144/original.jpg	https://data.globe.gov/system/photos/2019/09/26/1220143/original.jpg	https://data.globe.gov/system/photos/2019/09/26/1220145/original.jpg	https://data.globe.gov/system/photos/2019/09/26/1220147/original.jpg
GV_2498	50.5379	21.6882	150.3		https://www.google.com/maps/search/50.5379,21.6882	https://data.globe.gov/system/photos/2019/09/26/1220148/original.jpg	https://data.globe.gov/system/photos/2019/09/26/1220154/original.jpg	https://data.globe.gov/system/photos/2019/09/26/1220150/original.jpg	https://data.globe.gov/system/photos/2019/09/26/1220157/original.jpg	https://data.globe.gov/system/photos/2019/09/26/1220159/original.jpg
GV_2499	50.538	21.6883	150.1		https://www.google.com/maps/search/50.538,21.6883	https://data.globe.gov/system/photos/2019/09/26/1220160/original.jpg	https://data.globe.gov/system/photos/2019/09/26/1220162/original.jpg	https://data.globe.gov/system/photos/2019/09/26/1220161/original.jpg	https://data.globe.gov/system/photos/2019/09/26/1220163/original.jpg	https://data.globe.gov/system/photos/2019/09/26/1220165/original.jpg
GV_2500	50.5372	21.6881	149.5		https://www.google.com/maps/search/50.5372,21.6881	https://data.globe.gov/system/photos/2019/09/26/1220166/original.jpg	https://data.globe.gov/system/photos/2019/09/26/1220168/original.jpg	https://data.globe.gov/system/photos/2019/09/26/1220167/original.jpg	https://data.globe.gov/system/photos/2019/09/26/1220169/original.jpg	https://data.globe.gov/system/photos/2019/09/26/1220171/original.jpg
GV_2501	50.5368	21.6874	150.2		https://www.google.com/maps/search/50.5368,21.6874	https://data.globe.gov/system/photos/2019/09/26/1220172/original.jpg	https://data.globe.gov/system/photos/2019/09/26/1220174/original.jpg	https://data.globe.gov/system/photos/2019/09/26/1220173/original.jpg	https://data.globe.gov/system/photos/2019/09/26/1220175/original.jpg	https://data.globe.gov/system/photos/2019/09/26/1220177/original.jpg
GV_2502	40.1118	-105.307	1790.3		https://www.google.com/maps/search/40.1118,-105.307	https://data.globe.gov/system/photos/2019/07/07/1220830/original.jpg	https://data.globe.gov/system/photos/2019/07/07/1220832/original.jpg	https://data.globe.gov/system/photos/2019/07/07/1220831/original.jpg	https://data.globe.gov/system/photos/2019/07/07/1220833/original.jpg	https://data.globe.gov/system/photos/2019/07/07/1220835/original.jpg
GV_2503	40.1111	-105.3068	1794.5		https://www.google.com/maps/search/40.1111,-105.3068	https://data.globe.gov/system/photos/2019/07/07/1220836/original.jpg	https://data.globe.gov/system/photos/2019/07/07/1220838/original.jpg	https://data.globe.gov/system/photos/2019/07/07/1220837/original.jpg	https://data.globe.gov/system/photos/2019/07/07/1220839/original.jpg	https://data.globe.gov/system/photos/2019/07/07/1220841/original.jpg
GV_2504	40.1086	-105.3031	1815.9		https://www.google.com/maps/search/40.1086,-105.3031	https://data.globe.gov/system/photos/2019/07/08/1220842/original.jpg	https://data.globe.gov/system/photos/2019/07/08/1220844/original.jpg	https://data.globe.gov/system/photos/2019/07/08/1220843/original.jpg	https://data.globe.gov/system/photos/2019/07/08/1220845/original.jpg	https://data.globe.gov/system/photos/2019/07/08/1220847/original.jpg
GV_2505	-2.3812	-44.3956	45.8		https://www.google.com/maps/search/-2.3812,-44.3956	https://data.globe.gov/system/photos/2019/09/01/1220854/original.jpg	https://data.globe.gov/system/photos/2019/09/01/1220856/original.jpg	https://data.globe.gov/system/photos/2019/09/01/1220855/original.jpg	https://data.globe.gov/system/photos/2019/09/01/1220857/original.jpg	https://data.globe.gov/system/photos/2019/09/01/1220859/original.jpg
GV_2506	-2.3909	-44.3824	35.2		https://www.google.com/maps/search/-2.3909,-44.3824	https://data.globe.gov/system/photos/2019/09/01/1220860/original.jpg	https://data.globe.gov/system/photos/2019/09/01/1220862/original.jpg	https://data.globe.gov/system/photos/2019/09/01/1220861/original.jpg	https://data.globe.gov/system/photos/2019/09/01/1220863/original.jpg	https://data.globe.gov/system/photos/2019/09/01/1220865/original.jpg
GV_2507	-2.391	-44.3826	36		https://www.google.com/maps/search/-2.391,-44.3826	https://data.globe.gov/system/photos/2019/09/01/1220866/original.jpg	https://data.globe.gov/system/photos/2019/09/01/1220868/original.jpg	https://data.globe.gov/system/photos/2019/09/01/1220867/original.jpg	https://data.globe.gov/system/photos/2019/09/01/1220869/original.jpg	https://data.globe.gov/system/photos/2019/09/01/1220872/original.jpg
GV_2508	-2.3171	-44.3683	40		https://www.google.com/maps/search/-2.3171,-44.3683	https://data.globe.gov/system/photos/2019/09/01/1220873/original.jpg	https://data.globe.gov/system/photos/2019/09/01/1220875/original.jpg	https://data.globe.gov/system/photos/2019/09/01/1220874/original.jpg	https://data.globe.gov/system/photos/2019/09/01/1220876/original.jpg	https://data.globe.gov/system/photos/2019/09/01/1220878/original.jpg
GV_2509	-2.3884	-44.378	37.6		https://www.google.com/maps/search/-2.3884,-44.378	https://data.globe.gov/system/photos/2019/09/01/1220879/original.jpg	https://data.globe.gov/system/photos/2019/09/01/1220881/original.jpg	https://data.globe.gov/system/photos/2019/09/01/1220880/original.jpg	https://data.globe.gov/system/photos/2019/09/01/1220882/original.jpg	https://data.globe.gov/system/photos/2019/09/01/1220884/original.jpg
GV_2510	40.1082	-105.303	1816.6		https://www.google.com/maps/search/40.1082,-105.303	https://data.globe.gov/system/photos/2019/09/26/1220897/original.jpg	https://data.globe.gov/system/photos/2019/09/26/1220899/original.jpg	https://data.globe.gov/system/photos/2019/09/26/1220898/original.jpg	https://data.globe.gov/system/photos/2019/09/26/1220900/original.jpg	https://data.globe.gov/system/photos/2019/09/26/1220902/original.jpg
GV_2511	42.8204	-75.538	337.6		https://www.google.com/maps/search/42.8204,-75.538	https://data.globe.gov/system/photos/2019/09/25/1221020/original.jpg	https://data.globe.gov/system/photos/2019/09/25/1221022/original.jpg	https://data.globe.gov/system/photos/2019/09/25/1221021/original.jpg	https://data.globe.gov/system/photos/2019/09/25/1221023/original.jpg	https://data.globe.gov/system/photos/2019/09/25/1221025/original.jpg
GV_2512	45.4947	15.5517	114.2		https://www.google.com/maps/search/45.4947,15.5517	https://data.globe.gov/system/photos/2019/09/27/1221318/original.jpg	https://data.globe.gov/system/photos/2019/09/27/1221320/original.jpg	https://data.globe.gov/system/photos/2019/09/27/1221319/original.jpg	https://data.globe.gov/system/photos/2019/09/27/1221321/original.jpg	https://data.globe.gov/system/photos/2019/09/27/1221323/original.jpg
GV_2513	3.9583	-76.2687	963.5		https://www.google.com/maps/search/3.9583,-76.2687	https://data.globe.gov/system/photos/2019/09/27/1221644/original.jpg	https://data.globe.gov/system/photos/2019/09/27/1221646/original.jpg	https://data.globe.gov/system/photos/2019/09/27/1221645/original.jpg	https://data.globe.gov/system/photos/2019/09/27/1221647/original.jpg	https://data.globe.gov/system/photos/2019/09/27/1221649/original.jpg

GV_2514	2.9102	-75.266	451.4		https://www.google.com/maps/search/2.9102,-75.266	https://data.globe.gov/system/photos/2019/09/27/1222127/original.jpg	https://data.globe.gov/system/photos/2019/09/27/1222129/original.jpg	https://data.globe.gov/system/photos/2019/09/27/1222128/original.jpg	https://data.globe.gov/system/photos/2019/09/27/1222130/original.jpg	https://data.globe.gov/system/photos/2019/09/27/1222132/original.jpg
GV_2515	-1.2526	36.858	1635.9		https://www.google.com/maps/search/-1.2526,36.858	https://data.globe.gov/system/photos/2019/09/28/1222334/original.jpg	https://data.globe.gov/system/photos/2019/09/28/1222336/original.jpg	https://data.globe.gov/system/photos/2019/09/28/1222335/original.jpg	https://data.globe.gov/system/photos/2019/09/28/1222337/original.jpg	https://data.globe.gov/system/photos/2019/09/28/1222339/original.jpg
GV_2516	-16.4202	123.0326	7.1		https://www.google.com/maps/search/-16.4202,123.0326	https://data.globe.gov/system/photos/2019/09/29/1223231/original.jpg	https://data.globe.gov/system/photos/2019/09/29/1223233/original.jpg	https://data.globe.gov/system/photos/2019/09/29/1223232/original.jpg	https://data.globe.gov/system/photos/2019/09/29/1223234/original.jpg	https://data.globe.gov/system/photos/2019/09/29/1223236/original.jpg
GV_2517	52.9574	1.0525	2.9		https://www.google.com/maps/search/52.9574,1.0525	https://data.globe.gov/system/photos/2019/09/29/1223444/original.jpg	https://data.globe.gov/system/photos/2019/09/29/1223446/original.jpg	https://data.globe.gov/system/photos/2019/09/29/1223445/original.jpg	https://data.globe.gov/system/photos/2019/09/29/1223447/original.jpg	https://data.globe.gov/system/photos/2019/09/29/1223449/original.jpg
GV_2518	39.1862	-86.7785	228.5		https://www.google.com/maps/search/39.1862,-86.7785	https://data.globe.gov/system/photos/2019/09/29/1223656/original.jpg	https://data.globe.gov/system/photos/2019/09/29/1223658/original.jpg	https://data.globe.gov/system/photos/2019/09/29/1223657/original.jpg	https://data.globe.gov/system/photos/2019/09/29/1223659/original.jpg	https://data.globe.gov/system/photos/2019/09/29/1223661/original.jpg
GV_2519	42.8205	-75.538	337.3		https://www.google.com/maps/search/42.8205,-75.538	https://data.globe.gov/system/photos/2019/09/25/1223846/original.jpg	https://data.globe.gov/system/photos/2019/09/25/1223848/original.jpg	https://data.globe.gov/system/photos/2019/09/25/1223847/original.jpg	https://data.globe.gov/system/photos/2019/09/25/1223849/original.jpg	https://data.globe.gov/system/photos/2019/09/25/1223851/original.jpg
GV_2520	47.1058	-92.4049	419		https://www.google.com/maps/search/47.1058,-92.4049	https://data.globe.gov/system/photos/2019/09/28/1223986/original.jpg	https://data.globe.gov/system/photos/2019/09/28/1223994/original.jpg	https://data.globe.gov/system/photos/2019/09/28/1223993/original.jpg	https://data.globe.gov/system/photos/2019/09/28/1223995/original.jpg	https://data.globe.gov/system/photos/2019/09/28/1223997/original.jpg
GV_2521	38.8863	-76.8211	46.7		https://www.google.com/maps/search/38.8863,-76.8211	https://data.globe.gov/system/photos/2019/09/23/1224271/original.jpg	https://data.globe.gov/system/photos/2019/09/23/1224273/original.jpg	https://data.globe.gov/system/photos/2019/09/23/1224272/original.jpg	https://data.globe.gov/system/photos/2019/09/23/1224274/original.jpg	https://data.globe.gov/system/photos/2019/09/23/1224276/original.jpg
GV_2522	39.2444	-77.2013	151.1		https://www.google.com/maps/search/39.2444,-77.2013	https://data.globe.gov/system/photos/2019/09/27/1224755/original.jpg	https://data.globe.gov/system/photos/2019/09/27/1224757/original.jpg	https://data.globe.gov/system/photos/2019/09/27/1224756/original.jpg	https://data.globe.gov/system/photos/2019/09/27/1224758/original.jpg	https://data.globe.gov/system/photos/2019/09/27/1224760/original.jpg
GV_2523	-26.7851	-50.9758	994.5		https://www.google.com/maps/search/-26.7851,-50.9758	https://data.globe.gov/system/photos/2019/09/25/1224835/original.jpg	https://data.globe.gov/system/photos/2019/09/25/1224837/original.jpg	https://data.globe.gov/system/photos/2019/09/25/1224836/original.jpg	https://data.globe.gov/system/photos/2019/09/25/1224838/original.jpg	https://data.globe.gov/system/photos/2019/09/25/1224840/original.jpg
GV_2524	42.8204	-75.5379	337.6		https://www.google.com/maps/search/42.8204,-75.5379	https://data.globe.gov/system/photos/2019/09/25/1224857/original.jpg	https://data.globe.gov/system/photos/2019/09/25/1224859/original.jpg	https://data.globe.gov/system/photos/2019/09/25/1224858/original.jpg	https://data.globe.gov/system/photos/2019/09/25/1224860/original.jpg	https://data.globe.gov/system/photos/2019/09/25/1224862/original.jpg
GV_2525	-17.77	122.2821	19.1		https://www.google.com/maps/search/-17.77,122.2821	https://data.globe.gov/system/photos/2019/09/30/1224882/original.jpg	https://data.globe.gov/system/photos/2019/09/30/1224884/original.jpg	https://data.globe.gov/system/photos/2019/09/30/1224883/original.jpg	https://data.globe.gov/system/photos/2019/09/30/1224885/original.jpg	https://data.globe.gov/system/photos/2019/09/30/1224887/original.jpg
GV_2526	-18.7986	126.5349	260.8		https://www.google.com/maps/search/-18.7986,126.5349	https://data.globe.gov/system/photos/2019/10/01/1224894/original.jpg	https://data.globe.gov/system/photos/2019/10/01/1224896/original.jpg	https://data.globe.gov/system/photos/2019/10/01/1224895/original.jpg	https://data.globe.gov/system/photos/2019/10/01/1224897/original.jpg	https://data.globe.gov/system/photos/2019/10/01/1224899/original.jpg
GV_2527	24.4357	118.314	21.9		https://www.google.com/maps/search/24.4357,118.314	https://data.globe.gov/system/photos/2019/10/01/1225025/original.jpg	https://data.globe.gov/system/photos/2019/10/01/1225027/original.jpg	https://data.globe.gov/system/photos/2019/10/01/1225026/original.jpg	https://data.globe.gov/system/photos/2019/10/01/1225030/original.jpg	https://data.globe.gov/system/photos/2019/10/01/1225035/original.jpg
GV_2528	45.4855	15.5443	109.9		https://www.google.com/maps/search/45.4855,15.5443	https://data.globe.gov/system/photos/2019/10/01/1225227/original.jpg	https://data.globe.gov/system/photos/2019/10/01/1225229/original.jpg	https://data.globe.gov/system/photos/2019/10/01/1225228/original.jpg	https://data.globe.gov/system/photos/2019/10/01/1225230/original.jpg	https://data.globe.gov/system/photos/2019/10/01/1225232/original.jpg
GV_2529	42.8215	-75.5448	338.5		https://www.google.com/maps/search/42.8215,-75.5448	https://data.globe.gov/system/photos/2019/09/25/1226038/original.jpg	https://data.globe.gov/system/photos/2019/09/25/1226040/original.jpg	https://data.globe.gov/system/photos/2019/09/25/1226039/original.jpg	https://data.globe.gov/system/photos/2019/09/25/1226041/original.jpg	https://data.globe.gov/system/photos/2019/09/25/1226043/original.jpg
GV_2530	42.8122	-75.5377	405		https://www.google.com/maps/search/42.8122,-75.5377	https://data.globe.gov/system/photos/2019/10/01/1226044/original.jpg	https://data.globe.gov/system/photos/2019/10/01/1226046/original.jpg	https://data.globe.gov/system/photos/2019/10/01/1226045/original.jpg	https://data.globe.gov/system/photos/2019/10/01/1226047/original.jpg	https://data.globe.gov/system/photos/2019/10/01/1226049/original.jpg
GV_2531	47.5562	-122.1739	108.9		https://www.google.com/maps/search/47.5562,-122.1739	https://data.globe.gov/system/photos/2019/10/01/1226153/original.jpg	https://data.globe.gov/system/photos/2019/10/01/1226155/original.jpg	https://data.globe.gov/system/photos/2019/10/01/1226154/original.jpg	https://data.globe.gov/system/photos/2019/10/01/1226156/original.jpg	https://data.globe.gov/system/photos/2019/10/01/1226158/original.jpg
GV_2532	35.9993	129.2537	16.8		https://www.google.com/maps/search/35.9993,129.2537	https://data.globe.gov/system/photos/2019/10/01/1226315/original.jpg	https://data.globe.gov/system/photos/2019/10/01/1226317/original.jpg	https://data.globe.gov/system/photos/2019/10/01/1226316/original.jpg	https://data.globe.gov/system/photos/2019/10/01/1226318/original.jpg	https://data.globe.gov/system/photos/2019/10/01/1226320/original.jpg
GV_2533	-19.8371	128.0566	388.2		https://www.google.com/maps/search/-19.8371,128.0566	https://data.globe.gov/system/photos/2019/10/01/1226396/original.jpg	https://data.globe.gov/system/photos/2019/10/01/1226398/original.jpg	https://data.globe.gov/system/photos/2019/10/01/1226397/original.jpg	https://data.globe.gov/system/photos/2019/10/01/1226399/original.jpg	https://data.globe.gov/system/photos/2019/10/01/1226401/original.jpg
GV_2534	-19.8842	128.6531	402.6		https://www.google.com/maps/search/-19.8842,128.6531	https://data.globe.gov/system/photos/2019/10/01/1226402/original.jpg	https://data.globe.gov/system/photos/2019/10/01/1226404/original.jpg	https://data.globe.gov/system/photos/2019/10/01/1226403/original.jpg	https://data.globe.gov/system/photos/2019/10/01/1226405/original.jpg	https://data.globe.gov/system/photos/2019/10/01/1226407/original.jpg
GV_2535	-19.8958	129.0012	421		https://www.google.com/maps/search/-19.8958,129.0012	https://data.globe.gov/system/photos/2019/10/01/1226408/original.jpg	https://data.globe.gov/system/photos/2019/10/01/1226410/original.jpg	https://data.globe.gov/system/photos/2019/10/01/1226409/original.jpg	https://data.globe.gov/system/photos/2019/10/01/1226411/original.jpg	https://data.globe.gov/system/photos/2019/10/01/1226413/original.jpg
GV_2536	-20.4138	130.1671	343.6		https://www.google.com/maps/search/-20.4138,130.1671	https://data.globe.gov/system/photos/2019/10/02/1226420/original.jpg	https://data.globe.gov/system/photos/2019/10/02/1226422/original.jpg	https://data.globe.gov/system/photos/2019/10/02/1226421/original.jpg	https://data.globe.gov/system/photos/2019/10/02/1226423/original.jpg	https://data.globe.gov/system/photos/2019/10/02/1226425/original.jpg
GV_2537	-20.7666	130.5247	374.3		https://www.google.com/maps/search/-20.7666,130.5247	https://data.globe.gov/system/photos/2019/10/02/1226426/original.jpg	https://data.globe.gov/system/photos/2019/10/02/1226428/original.jpg	https://data.globe.gov/system/photos/2019/10/02/1226427/original.jpg	https://data.globe.gov/system/photos/2019/10/02/1226429/original.jpg	https://data.globe.gov/system/photos/2019/10/02/1226431/original.jpg
GV_2538	-21.2777	130.8495	420.6		https://www.google.com/maps/search/-21.2777,130.8495	https://data.globe.gov/system/photos/2019/10/02/1226432/original.jpg	https://data.globe.gov/system/photos/2019/10/02/1226434/original.jpg	https://data.globe.gov/system/photos/2019/10/02/1226433/original.jpg	https://data.globe.gov/system/photos/2019/10/02/1226435/original.jpg	https://data.globe.gov/system/photos/2019/10/02/1226437/original.jpg
GV_2539	47.1065	-92.4066	419.8		https://www.google.com/maps/search/47.1065,-92.4066	https://data.globe.gov/system/photos/2019/09/27/1226491/original.jpg	https://data.globe.gov/system/photos/2019/09/27/1226493/original.jpg	https://data.globe.gov/system/photos/2019/09/27/1226492/original.jpg	https://data.globe.gov/system/photos/2019/09/27/1226494/original.jpg	https://data.globe.gov/system/photos/2019/09/27/1226496/original.jpg
GV_2540	47.1087	-92.4069	423.4		https://www.google.com/maps/search/47.1087,-92.4069	https://data.globe.gov/system/photos/2019/09/27/1226497/original.jpg	https://data.globe.gov/system/photos/2019/09/27/1226499/original.jpg	https://data.globe.gov/system/photos/2019/09/27/1226498/original.jpg	https://data.globe.gov/system/photos/2019/09/27/1226500/original.jpg	https://data.globe.gov/system/photos/2019/09/27/1226502/original.jpg
GV_2541	47.109	-92.4062	420.8		https://www.google.com/maps/search/47.109,-92.4062	https://data.globe.gov/system/photos/2019/09/27/1226503/original.jpg	https://data.globe.gov/system/photos/2019/09/27/1226505/original.jpg	https://data.globe.gov/system/photos/2019/09/27/1226504/original.jpg	https://data.globe.gov/system/photos/2019/09/27/1226506/original.jpg	https://data.globe.gov/system/photos/2019/09/27/1226508/original.jpg
GV_2542	47.1099	-92.4046	428.3		https://www.google.com/maps/search/47.1099,-92.4046	https://data.globe.gov/system/photos/2019/09/27/1226509/original.jpg	https://data.globe.gov/system/photos/2019/09/27/1226511/original.jpg	https://data.globe.gov/system/photos/2019/09/27/1226510/original.jpg	https://data.globe.gov/system/photos/2019/09/27/1226512/original.jpg	https://data.globe.gov/system/photos/2019/09/27/1226514/original.jpg
GV_2543	47.1182	-92.4136	428.9		https://www.google.com/maps/search/47.1182,-92.4136	https://data.globe.gov/system/photos/2019/09/27/1226515/original.jpg	https://data.globe.gov/system/photos/2019/09/27/1226517/original.jpg	https://data.globe.gov/system/photos/2019/09/27/1226516/original.jpg	https://data.globe.gov/system/photos/2019/09/27/1226518/original.jpg	https://data.globe.gov/system/photos/2019/09/27/1226520/original.jpg
GV_2544	47.12	-92.4149	425.8		https://www.google.com/maps/search/47.12,-92.4149	https://data.globe.gov/system/photos/2019/09/27/1226521/original.jpg	https://data.globe.gov/system/photos/2019/09/27/1226523/original.jpg	https://data.globe.gov/system/photos/2019/09/27/1226522/original.jpg	https://data.globe.gov/system/photos/2019/09/27/1226524/original.jpg	https://data.globe.gov/system/photos/2019/09/27/1226526/original.jpg
GV_2545	47.1193	-92.415	433.7		https://www.google.com/maps/search/47.1193,-92.415	https://data.globe.gov/system/photos/2019/09/27/1226527/original.jpg	https://data.globe.gov/system/photos/2019/09/27/1226529/original.jpg	https://data.globe.gov/system/photos/2019/09/27/1226528/original.jpg	https://data.globe.gov/system/photos/2019/09/27/1226530/original.jpg	https://data.globe.gov/system/photos/2019/09/27/1226532/original.jpg
GV_2546	47.1187	-92.4156	435.5		https://www.google.com/maps/search/47.1187,-92.4156	https://data.globe.gov/system/photos/2019/09/27/1226533/original.jpg	https://data.globe.gov/system/photos/2019/09/27/1226535/original.jpg	https://data.globe.gov/system/photos/2019/09/27/1226534/original.jpg	https://data.globe.gov/system/photos/2019/09/27/1226536/original.jpg	https://data.globe.gov/system/photos/2019/09/27/1226538/original.jpg
GV_2547	47.1157	-92.4143	431.6		https://www.google.com/maps/search/47.1157,-92.4143	https://data.globe.gov/system/photos/2019/09/27/1226539/original.jpg	https://data.globe.gov/system/photos/2019/09/27/1226541/original.jpg	https://data.globe.gov/system/photos/2019/09/27/1226540/original.jpg	https://data.globe.gov/system/photos/2019/09/27/1226542/original.jpg	https://data.globe.gov/system/photos/2019/09/27/1226544/original.jpg
GV_2548	47.1184	-92.4003	422.6		https://www.google.com/maps/search/47.1184,-92.4003	https://data.globe.gov/system/photos/2019/09/28/1226545/original.jpg	https://data.globe.gov/system/photos/2019/09/28/1226547/original.jpg	https://data.globe.gov/system/photos/2019/09/28/1226546/original.jpg	https://data.globe.gov/system/photos/2019/09/28/1226548/original.jpg	https://data.globe.gov/system/photos/2019/09/28/1226550/original.jpg
GV_2549	47.1185	-92.4003	422.5		https://www.google.com/maps/search/47.1185,-92.4003	https://data.globe.gov/system/photos/2019/09/28/1226551/original.jpg	https://data.globe.gov/system/photos/2019/09/28/1226553/original.jpg	https://data.globe.gov/system/photos/2019/09/28/1226552/original.jpg	https://data.globe.gov/system/photos/2019/09/28/1226554/original.jpg	https://data.globe.gov/system/photos/2019/09/28/1226556/original.jpg
GV_2550	47.1145	-92.4049	424.8		https://www.google.com/maps/search/47.1145,-92.4049	https://data.globe.gov/system/photos/2019/09/28/1226557/original.jpg	https://data.globe.gov/system/photos/2019/09/28/1226559/original.jpg	https://data.globe.gov/system/photos/2019/09/28/1226558/original.jpg	https://data.globe.gov/system/photos/2019/09/28/1226560/original.jpg	https://data.globe.gov/system/photos/2019/09/28/1226562/original.jpg
GV_2551	47.1152	-92.4085	427.3		https://www.google.com/maps/search/47.1152,-92.4085	https://data.globe.gov/system/photos/2019/09/28/1226563/original.jpg	https://data.globe.gov/system/photos/2019/09/28/1226565/original.jpg	https://data.globe.gov/system/photos/2019/09/28/1226564/original.jpg	https://data.globe.gov/system/photos/2019/09/28/1226566/original.jpg	https://data.globe.gov/system/photos/2019/09/28/1226568/original.jpg
GV_2552	47.1173	-92.4122	430.7		https://www.google.com/maps/search/47.1173,-92.4122	https://data.globe.gov/system/photos/2019/09/28/1226569/original.jpg	https://data.globe.gov/system/photos/2019/09/28/1226571/original.jpg	https://data.globe.gov/system/photos/2019/09/28/1226570/original.jpg	https://data.globe.gov/system/photos/2019/09/28/1226572/original.jpg	https://data.globe.gov/system/photos/2019/09/28/1226574/original.jpg
GV_2553	47.1192	-92.4143	431.3		https://www.google.com/maps/search/47.1192,-92.4143	https://data.globe.gov/system/photos/2019/09/28/1226575/original.jpg	https://data.globe.gov/system/photos/2019/09/28/1226577/original.jpg	https://data.globe.gov/system/photos/2019/09/28/1226576/original.jpg	https://data.globe.gov/system/photos/2019/09/28/1226578/original.jpg	https://data.globe.gov/system/photos/2019/09/28/1226580/original.jpg
GV_2554	47.1192	-92.415	432.3		https://www.google.com/maps/search/47.1192,-92.415	https://data.globe.gov/system/photos/2019/09/28/1226581/original.jpg	https://data.globe.gov/system/photos/2019/09/28/1226583/original.jpg	https://data.globe.gov/system/photos/2019/09/28/1226582/original.jpg	https://data.globe.gov/system/photos/2019/09/28/1226584/original.jpg	https://data.globe.gov/system/photos/2019/09/28/1226586/original.jpg
GV_2555	47.119	-92.415	430.3		https://www.google.com/maps/search/47.119,-92.415	https://data.globe.gov/system/photos/2019/09/28/1226587/original.jpg	https://data.globe.gov/system/photos/2019/09/28/1226589/original.jpg	https://data.globe.gov/system/photos/2019/09/28/1226588/original.jpg	https://data.globe.gov/system/photos/2019/09/28/1226590/original.jpg	https://data.globe.gov/system/photos/2019/09/28/1226592/original.jpg
GV_2556	47.1179	-92.4138	427.7		https://www.google.com/maps/search/47.1179,-92.4138	https://data.globe.gov/system/photos/2019/09/28/1226593/original.jpg	https://data.globe.gov/system/photos/2019/09/28/1226595/original.jpg	https://data.globe.gov/system/photos/2019/09/28/1226594/original.jpg	https://data.globe.gov/system/photos/2019/09/28/1226596/original.jpg	https://data.globe.gov/system/photos/2019/09/28/1226598/original.jpg
GV_2557	47.1143	-92.4124	441.4		https://www.google.com/maps/search/47.1143,-92.4124	https://data.globe.gov/system/photos/2019/09/28/1226599/original.jpg	https://data.globe.gov/system/photos/2019/09/28/1226601/original.jpg	https://data.globe.gov/system/photos/2019/09/28/1226600/original.jpg	https://data.globe.gov/system/photos/2019/09/28/1226602/original.jpg	https://data.globe.gov/system/photos/2019/09/28/1226604/original.jpg
GV_2558	47.1116	-92.4061	433.7		https://www.google.com/maps/search/47.1116,-92.4061	https://data.globe.gov/system/photos/2019/09/28/1226605/original.jpg	https://data.globe.gov/system/photos/2019/09/28/1226608/original.jpg	https://data.globe.gov/system/photos/2019/09/28/1226606/original.jpg	https://data.globe.gov/system/photos/2019/09/28/1226609/original.jpg	https://data.globe.gov/system/photos/2019/09/28/1226612/original.jpg
GV_2559	47.1107	-92.4051	436.2		https://www.google.com/maps/search/47.1107,-92.4051	https://data.globe.gov/system/photos/2019/09/28/1226614/original.jpg	https://data.globe.gov/system/photos/2019/09/28/1226616/original.jpg	https://data.globe.gov/system/photos/2019/09/28/1226615/original.jpg	https://data.globe.gov/system/photos/2019/09/28/1226617/original.jpg	https://data.globe.gov/system/photos/2019/09/28/1226619/original.jpg
GV_2560	47.1092	-92.4065	424		https://www.google.com/maps/search/47.1092,-92.4065	https://data.globe.gov/system/photos/2019/09/28/1226620/original.jpg	https://data.globe.gov/system/photos/2019/09/28/1226622/original.jpg	https://data.globe.gov/system/photos/2019/09/28/1226621/original.jpg	https://data.globe.gov/system/photos/2019/09/28/1226623/original.jpg	https://data.globe.gov/system/photos/2019/09/28/1226625/original.jpg
GV_2561	46.7219	-92.4536	370.7		https://www.google.com/maps/search/46.7219,-92.4536	https://data.globe.gov/system/photos/2019/09/28/1226626/original.jpg	https://data.globe.gov/system/photos/2019/09/28/1226628/original.jpg	https://data.globe.gov/system/photos/2019/09/28/1226627/original.jpg	https://data.globe.gov/system/photos/2019/09/28/1226629/original.jpg	https://data.globe.gov/system/photos/2019/09/28/1226632/original.jpg
GV_2562	44.1657	-94.0024	238.9		https://www.google.com/maps/search/44.1657,-94.0024	https://data.globe.gov/system/photos/2019/09/30/1226633/original.jpg	https://data.globe.gov/system/photos/2019/09/30/1226635/original.jpg	https://data.globe.gov/system/photos/2019/09/30/1226634/original.jpg	https://data.globe.gov/system/photos/2019/09/30/1226636/original.jpg	https://data.globe.gov/system/photos/2019/09/30/1226640/original.jpg
GV_2563	44.0503	-95.423	436.1		https://www.google.com/maps/search/44.0503,-95.423	https://data.globe.gov/system/photos/2019/09/30/1226643/original.jpg	https://data.globe.gov/system/photos/2019/09/30/1226647/original.jpg	https://data.globe.gov/system/photos/2019/09/30/1226644/original.jpg	https://data.globe.gov/system/photos/2019/09/30/1226648/original.jpg	https://data.globe.gov/system/photos/2019/09/30/1226650/original.jpg
GV_2564	44.0501	-95.4243	437.7		https://www.google.com/maps/search/44.0501,-95.4243	https://data.globe.gov/system/photos/2019/09/30/1226651/original.jpg	https://data.globe.gov/system/photos/2019/09/30/1226653/original.jpg	https://data.globe.gov/system/photos/2019/09/30/1226652/original.jpg	https://data.globe.gov/system/photos/2019/09/30/1226654/original.jpg	https://data.globe.gov/system/photos/2019/09/30/1226656/original.jpg
GV_2565	44.0368	-95.4361	430.1		https://www.google.com/maps/search/44.0368,-95.4361	https://data.globe.gov/system/photos/2019/09/30/1226657/original.jpg	https://data.globe.gov/system/photos/2019/09/30/1226659/original.jpg	https://data.globe.gov/system/photos/2019/09/30/1226658/original.jpg	https://data.globe.gov/system/photos/2019/09/30/1226660/original.jpg	https://data.globe.gov/system/photos/2019/09/30/1226662/original.jpg
GV_2566	44.0232	-95.3609	424.6		https://www.google.com/maps/search/44.0232,-95.3609	https://data.globe.gov/system/photos/2019/09/30/1226663/original.jpg	https://data.globe.gov/system/photos/2019/09/30/1226665/original.jpg	https://data.globe.gov/system/photos/2019/09/30/1226664/original.jpg	https://data.globe.gov/system/photos/2019/09/30/1226666/original.jpg	https://data.globe.gov/system/photos/2019/09/30/1226668/original.jpg
GV_2567	44.0264	-95.361	425		https://www.google.com/maps/search/44.0264,-95.361	https://data.globe.gov/system/photos/2019/09/30/1226669/original.jpg	https://data.globe.gov/system/photos/2019/09/30/1226671/original.jpg	https://data.globe.gov/system/photos/2019/09/30/1226670/original.jpg	https://data.globe.gov/system/photos/2019/09/30/1226672/original.jpg	https://data.globe.gov/system/photos/2019/09/30/1226674/original.jpg
GV_2568	44.032	-95.3636	421.9		https://www.google.com/maps/search/44.032,-95.3636	https://data.globe.gov/system/photos/2019/09/30/1226675/original.jpg	https://data.globe.gov/system/photos/2019/09/30/1226677/original.jpg	https://data.globe.gov/system/photos/2019/09/30/1226676/original.jpg	https://data.globe.gov/system/photos/2019/09/30/1226678/original.jpg	https://data.globe.gov/system/photos/2019/09/30/1226680/original.jpg
GV_2569	44.032	-95.3608	422.2		https://www.google.com/maps/search/44.032,-95.3608	https://data.globe.gov/system/photos/2019/09/30/1226681/original.jpg	https://data.globe.gov/system/photos/2019/09/30/1226683/original.jpg	https://data.globe.gov/system/photos/2019/09/30/1226682/original.jpg	https://data.globe.gov/system/photos/2019/09/30/1226684/original.jpg	https://data.globe.gov/system/photos/2019/09/30/1226686/original.jpg
GV_2570	44.0301	-95.3587	422.5		https://www.google.com/maps/search/44.0301,-95.3587	https://data.globe.gov/system/photos/2019/09/30/1226687/original.jpg	https://data.globe.gov/system/photos/2019/09/30/1226689/original.jpg	https://data.globe.gov/system/photos/2019/09/30/1226688/original.jpg	https://data.globe.gov/system/photos/2019/09/30/1226690/original.jpg	https://data.globe.gov/system/photos/2019/09/30/1226692/original.jpg
GV_2571	44.0276	-95.3601	426.6		https://www.google.com/maps/search/44.0276,-95.3601	https://data.globe.gov/system/photos/2019/09/30/1226693/original.jpg	https://data.globe.gov/system/photos/2019/09/30/1226695/original.jpg	https://data.globe.gov/system/photos/2019/09/30/1226694/original.jpg	https://data.globe.gov/system/photos/2019/09/30/1226696/original.jpg	https://data.globe.gov/system/photos/2019/09/30/1226698/original.jpg
GV_2572	44.8757	-93.1904	212.9		https://www.google.com/maps/search/44.8757,-93.1904	https://data.globe.gov/system/photos/2019/10/01/1226699/original.jpg	https://data.globe.gov/system/photos/2019/10/01/1226701/original.jpg	https://data.globe.gov/system/photos/2019/10/01/1226700/original.jpg	https://data.globe.gov/system/photos/2019/10/01/1226702/original.jpg	https://data.globe.gov/system/photos/2019/10/01/1226704/original.jpg
GV_2573	50.497	21.6783	146.4		https://www.google.com/maps/search/50.497,21.6783	https://data.globe.gov/system/photos/2019/10/02/1226996/original.jpg	https://data.globe.gov/system/photos/2019/10/02/1226998/original.jpg	https://data.globe.gov/system/photos/2019/10/02/1226997/original.jpg	https://data.globe.gov/system/photos/2019/10/02/1226999/original.jpg	https://data.globe.gov/system/photos/2019/10/02/1227001/original.jpg
GV_2574	50.5085	21.6734	145.7		https://www.google.com/maps/search/50.5085,21.6734	https://data.globe.gov/system/photos/2019/10/02/1227005/original.jpg	https://data.globe.gov/system/photos/2019/10/02/1227007/original.jpg	https://data.globe.gov/system/photos/2019/10/02/1227006/original.jpg	https://data.globe.gov/system/photos/2019/10/02/1227008/original.jpg	https://data.globe.gov/system/photos/2019/10/02/1227010/original.jpg
GV_2575	50.5093	21.6712	146.5		https://www.google.com/maps/search/50.5093,21.6712	https://data.globe.gov/system/photos/2019/10/02/1227011/original.jpg	https://data.globe.gov/system/photos/2019/10/02/1227013/original.jpg	https://data.globe.gov/system/photos/2019/10/02/1227012/original.jpg	https://data.globe.gov/system/photos/2019/10/02/1227014/original.jpg	https://data.globe.gov/system/photos/2019/10/02/1227016/original.jpg
GV_2576	50.5105	21.6679	146		https://www.google.com/maps/search/50.5105,21.6679	https://data.globe.gov/system/photos/2019/10/02/1227017/original.jpg	https://data.globe.gov/system/photos/2019/10/02/1227019/original.jpg	https://data.globe.gov/system/photos/2019/10/02/1227018/original.jpg	https://data.globe.gov/system/photos/2019/10/02/1227020/original.jpg	https://data.globe.gov/system/photos/2019/10/02/1227022/original.jpg
GV_2577	45.4872	15.5432	114.1		https://www.google.com/maps/search/45.4872,15.5432	https://data.globe.gov/system/photos/2019/10/02/1227080/original.jpg	https://data.globe.gov/system/photos/2019/10/02/1227082/original.jpg	https://data.globe.gov/system/photos/2019/10/02/1227081/original.jpg	https://data.globe.gov/system/photos/2019/10/02/1227083/original.jpg	https://data.globe.gov/system/photos/2019/10/02/1227085/original.jpg
GV_2578	39.0027	-76.913	16.8		https://www.google.com/maps/search/39.0027,-76.913	https://data.globe.gov/system/photos/2019/10/02/1227215/original.jpg	https://data.globe.gov/system/photos/2019/10/02/1227217/original.jpg	https://data.globe.gov/system/photos/2019/10/02/1227216/original.jpg	https://data.globe.gov/system/photos/2019/10/02/1227218/original.jpg	https://data.globe.gov/system/photos/2019/10/02/1227220/original.jpg
GV_2579	45.497	-122.6679	9.9		https://www.google.com/maps/search/45.497,-122.6679	https://data.globe.gov/system/photos/2019/10/02/1227323/original.jpg	https://data.globe.gov/system/photos/2019/10/02/1227325/original.jpg	https://data.globe.gov/system/photos/2019/10/02/1227324/original.jpg	https://data.globe.gov/system/photos/2019/10/02/1227326/original.jpg	https://data.globe.gov/system/photos/2019/10/02/1227328/original.jpg
GV_2580	42.0861	-83.2101	177.7		https://www.google.com/maps/search/42.0861,-83.2101	https://data.globe.gov/system/photos/2019/10/02/1227486/original.jpg	https://data.globe.gov/system/photos/2019/10/02/1227488/original.jpg	https://data.globe.gov/system/photos/2019/10/02/1227487/original.jpg	https://data.globe.gov/system/photos/2019/10/02/1227489/original.jpg	https://data.globe.gov/system/photos/2019/10/02/1227491/original.jpg
GV_2581	44.4883	-93.8751	290.9		https://www.google.com/maps/search/44.4883,-93.8751	https://data.globe.gov/system/photos/2019/09/30/1227789/original.jpg	https://data.globe.gov/system/photos/2019/09/30/1227791/original.jpg	https://data.globe.gov/system/photos/2019/09/30/1227790/original.jpg	https://data.globe.gov/system/photos/2019/09/30/1227792/original.jpg	https://data.globe.gov/system/photos/2019/09/30/1227794/original.jpg
GV_2582	-34.5258	138.8153	148.1		https://www.google.com/maps/search/-34.5258,138.8153	https://data.globe.gov/system/photos/2019/09/26/1227815/original.jpg	https://data.globe.gov/system/photos/2019/09/26/1227817/original.jpg	https://data.globe.gov/system/photos/2019/09/26/1227816/original.jpg	https://data.globe.gov/system/photos/2019/09/26/1227818/original.jpg	https://data.globe.gov/system/photos/2019/09/26/1227820/original.jpg
GV_2583	-34.9694	138.6184	72.5		https://www.google.com/maps/search/-34.9694,138.6184	https://data.globe.gov/system/photos/2019/10/03/1227821/original.jpg	https://data.globe.gov/system/photos/2019/10/03/1227823/original.jpg	https://data.globe.gov/system/photos/2019/10/03/1227822/original.jpg	https://data.globe.gov/system/photos/2019/10/03/1227824/original.jpg	https://data.globe.gov/system/photos/2019/10/03/1227826/original.jpg
GV_2584	50.5126	21.6713	146.1		https://www.google.com/maps/search/50.5126,21.6713	https://data.globe.gov/system/photos/2019/10/03/1228043/original.jpg	https://data.globe.gov/system/photos/2019/10/03/1228045/original.jpg	https://data.globe.gov/system/photos/2019/10/03/1228044/original.jpg	https://data.globe.gov/system/photos/2019/10/03/1228046/original.jpg	https://data.globe.gov/system/photos/2019/10/03/1228048/original.jpg
GV_2585	50.5128	21.6658	148.9		https://www.google.com/maps/search/50.5128,21.6658	https://data.globe.gov/system/photos/2019/10/03/1228049/original.jpg	https://data.globe.gov/system/photos/2019/10/03/1228051/original.jpg	https://data.globe.gov/system/photos/2019/10/03/1228050/original.jpg	https://data.globe.gov/system/photos/2019/10/03/1228052/original.jpg	https://data.globe.gov/system/photos/2019/10/03/1228054/original.jpg
GV_2586	52.9445	1.207	15.8		https://www.google.com/maps/search/52.9445,1.207	https://data.globe.gov/system/photos/2019/10/03/1228098/original.jpg	https://data.globe.gov/system/photos/2019/10/03/1228100/original.jpg	https://data.globe.gov/system/photos/2019/10/03/1228099/original.jpg	https://data.globe.gov/system/photos/2019/10/03/1228101/original.jpg	https://data.globe.gov/system/photos/2019/10/03/1228103/original.jpg
GV_2587	45.4856	15.5442	109.7		https://www.google.com/maps/search/45.4856,15.5442	https://data.globe.gov/system/photos/2019/10/03/1228109/original.jpg	https://data.globe.gov/system/photos/2019/10/03/1228111/original.jpg	https://data.globe.gov/system/photos/2019/10/03/1228110/original.jpg	https://data.globe.gov/system/photos/2019/10/03/1228112/original.jpg	https://data.globe.gov/system/photos/2019/10/03/1228114/original.jpg
GV_2588	50.5253	21.6769	151.3		https://www.google.com/maps/search/50.5253,21.6769	https://data.globe.gov/system/photos/2019/10/03/1228177/original.jpg	https://data.globe.gov/system/photos/2019/10/03/1228179/original.jpg	https://data.globe.gov/system/photos/2019/10/03/1228178/original.jpg	https://data.globe.gov/system/photos/2019/10/03/1228180/original.jpg	https://data.globe.gov/system/photos/2019/10/03/1228182/original.jpg
GV_2589	50.5218	21.6741	151.9		https://www.google.com/maps/search/50.5218,21.6741	https://data.globe.gov/system/photos/2019/10/03/1228184/original.jpg	https://data.globe.gov/system/photos/2019/10/03/1228186/original.jpg	https://data.globe.gov/system/photos/2019/10/03/1228185/original.jpg	https://data.globe.gov/system/photos/2019/10/03/1228187/original.jpg	https://data.globe.gov/system/photos/2019/10/03/1228190/original.jpg
GV_2590	50.5458	21.7069	145.7		https://www.google.com/maps/search/50.5458,21.7069	https://data.globe.gov/system/photos/2019/10/03/1228192/original.jpg	https://data.globe.gov/system/photos/2019/10/03/1228194/original.jpg	https://data.globe.gov/system/photos/2019/10/03/1228193/original.jpg	https://data.globe.gov/system/photos/2019/10/03/1228195/original.jpg	https://data.globe.gov/system/photos/2019/10/03/1228197/original.jpg
GV_2591	50.5415	21.6892	151.8		https://www.google.com/maps/search/50.5415,21.6892	https://data.globe.gov/system/photos/2019/10/03/1228378/original.jpg	https://data.globe.gov/system/photos/2019/10/03/1228380/original.jpg	https://data.globe.gov/system/photos/2019/10/03/1228379/original.jpg	https://data.globe.gov/system/photos/2019/10/03/1228381/original.jpg	https://data.globe.gov/system/photos/2019/10/03/1228383/original.jpg
GV_2592	50.5407	21.6891	152		https://www.google.com/maps/search/50.5407,21.6891	https://data.globe.gov/system/photos/2019/10/03/1228384/original.jpg	https://data.globe.gov/system/photos/2019/10/03/1228386/original.jpg	https://data.globe.gov/system/photos/2019/10/03/1228385/original.jpg	https://data.globe.gov/system/photos/2019/10/03/1228387/original.jpg	https://data.globe.gov/system/photos/2019/10/03/1228389/original.jpg
GV_2593	50.5398	21.689	150.9		https://www.google.com/maps/search/50.5398,21.689	https://data.globe.gov/system/photos/2019/10/03/1228390/original.jpg	https://data.globe.gov/system/photos/2019/10/03/1228392/original.jpg	https://data.globe.gov/system/photos/2019/10/03/1228391/original.jpg	https://data.globe.gov/system/photos/2019/10/03/1228393/original.jpg	https://data.globe.gov/system/photos/2019/10/03/1228395/original.jpg
GV_2594	50.539	21.6889	150		https://www.google.com/maps/search/50.539,21.6889	https://data.globe.gov/system/photos/2019/10/03/1228396/original.jpg	https://data.globe.gov/system/photos/2019/10/03/1228398/original.jpg	https://data.globe.gov/system/photos/2019/10/03/1228397/original.jpg	https://data.globe.gov/system/photos/2019/10/03/1228399/original.jpg	https://data.globe.gov/system/photos/2019/10/03/1228401/original.jpg
GV_2595	44.5674	-123.2772	78.1		https://www.google.com/maps/search/44.5674,-123.2772	https://data.globe.gov/system/photos/2019/10/03/1228790/original.jpg	https://data.globe.gov/system/photos/2019/10/03/1228792/original.jpg	https://data.globe.gov/system/photos/2019/10/03/1228791/original.jpg	https://data.globe.gov/system/photos/2019/10/03/1228793/original.jpg	https://data.globe.gov/system/photos/2019/10/03/1228795/original.jpg
GV_2596	44.7074	-123.2055	63.4		https://www.google.com/maps/search/44.7074,-123.2055	https://data.globe.gov/system/photos/2019/08/10/1228796/original.jpg	https://data.globe.gov/system/photos/2019/08/10/1228798/original.jpg	https://data.globe.gov/system/photos/2019/08/10/1228797/original.jpg	https://data.globe.gov/system/photos/2019/08/10/1228799/original.jpg	https://data.globe.gov/system/photos/2019/08/10/1228801/original.jpg
GV_2597	47.2007	-91.3669	217.8		https://www.google.com/maps/search/47.2007,-91.3669	https://data.globe.gov/system/photos/2019/08/27/1228802/original.jpg	https://data.globe.gov/system/photos/2019/08/27/1228804/original.jpg	https://data.globe.gov/system/photos/2019/08/27/1228803/original.jpg	https://data.globe.gov/system/photos/2019/08/27/1228805/original.jpg	https://data.globe.gov/system/photos/2019/08/27/1228807/original.jpg
GV_2598	44.4138	-123.3338	93.1		https://www.google.com/maps/search/44.4138,-123.3338	https://data.globe.gov/system/photos/2019/09/14/1228808/original.jpg	https://data.globe.gov/system/photos/2019/09/14/1228810/original.jpg	https://data.globe.gov/system/photos/2019/09/14/1228809/original.jpg	https://data.globe.gov/system/photos/2019/09/14/1228811/original.jpg	https://data.globe.gov/system/photos/2019/09/14/1228813/original.jpg
GV_2599	-2.6182	35.8945	653		https://www.google.com/maps/search/-2.6182,35.8945	https://data.globe.gov/system/photos/2019/07/04/1228820/original.jpg	https://data.globe.gov/system/photos/2019/07/04/1228822/original.jpg	https://data.globe.gov/system/photos/2019/07/04/1228821/original.jpg	https://data.globe.gov/system/photos/2019/07/04/1228823/original.jpg	https://data.globe.gov/system/photos/2019/07/04/1228825/original.jpg
GV_2600	46.1341	-123.8807	14.6		https://www.google.com/maps/search/46.1341,-123.8807	https://data.globe.gov/system/photos/2019/07/28/1228832/original.jpg	https://data.globe.gov/system/photos/2019/07/28/1228834/original.jpg	https://data.globe.gov/system/photos/2019/07/28/1228833/original.jpg	https://data.globe.gov/system/photos/2019/07/28/1228835/original.jpg	https://data.globe.gov/system/photos/2019/07/28/1228837/original.jpg
GV_2601	45.4862	15.5443	109		https://www.google.com/maps/search/45.4862,15.5443	https://data.globe.gov/system/photos/2019/10/04/1229361/original.jpg	https://data.globe.gov/system/photos/2019/10/04/1229363/original.jpg	https://data.globe.gov/system/photos/2019/10/04/1229362/original.jpg	https://data.globe.gov/system/photos/2019/10/04/1229364/original.jpg	https://data.globe.gov/system/photos/2019/10/04/1229366/original.jpg
GV_2602	40.5117	-105.026	1496.3		https://www.google.com/maps/search/40.5117,-105.026	https://data.globe.gov/system/photos/2019/10/04/1229885/original.jpg	https://data.globe.gov/system/photos/2019/10/04/1229887/original.jpg	https://data.globe.gov/system/photos/2019/10/04/1229886/original.jpg	https://data.globe.gov/system/photos/2019/10/04/1229888/original.jpg	https://data.globe.gov/system/photos/2019/10/04/1229890/original.jpg
GV_2603	39.0338	-97.5979	374.8		https://www.google.com/maps/search/39.0338,-97.5979	https://data.globe.gov/system/photos/2019/10/04/1230079/original.jpg	https://data.globe.gov/system/photos/2019/10/04/1230085/original.jpg	https://data.globe.gov/system/photos/2019/10/04/1230084/original.jpg	https://data.globe.gov/system/photos/2019/10/04/1230088/original.jpg	https://data.globe.gov/system/photos/2019/10/04/1230094/original.jpg

GV_2604	44.4123	-123.3319	90.8		https://www.google.com/maps/search/44.4123,-123.3319	https://data.globe.gov/system/photos/2019/10/04/1230455/original.jpg	https://data.globe.gov/system/photos/2019/10/04/1230457/original.jpg	https://data.globe.gov/system/photos/2019/10/04/1230456/original.jpg	https://data.globe.gov/system/photos/2019/10/04/1230458/original.jpg	https://data.globe.gov/system/photos/2019/10/04/1230460/original.jpg
GV_2605	44.4141	-123.3354	103		https://www.google.com/maps/search/44.4141,-123.3354	https://data.globe.gov/system/photos/2019/10/04/1230461/original.jpg	https://data.globe.gov/system/photos/2019/10/04/1230463/original.jpg	https://data.globe.gov/system/photos/2019/10/04/1230462/original.jpg	https://data.globe.gov/system/photos/2019/10/04/1230464/original.jpg	https://data.globe.gov/system/photos/2019/10/04/1230466/original.jpg
GV_2606	44.4155	-123.336	116.4		https://www.google.com/maps/search/44.4155,-123.336	https://data.globe.gov/system/photos/2019/10/04/1230467/original.jpg	https://data.globe.gov/system/photos/2019/10/04/1230469/original.jpg	https://data.globe.gov/system/photos/2019/10/04/1230468/original.jpg	https://data.globe.gov/system/photos/2019/10/04/1230470/original.jpg	https://data.globe.gov/system/photos/2019/10/04/1230472/original.jpg
GV_2607	45.5259	-122.734	170.3		https://www.google.com/maps/search/45.5259,-122.734	https://data.globe.gov/system/photos/2019/10/02/1230483/original.jpg	https://data.globe.gov/system/photos/2019/10/02/1230486/original.jpg	https://data.globe.gov/system/photos/2019/10/02/1230485/original.jpg	https://data.globe.gov/system/photos/2019/10/02/1230487/original.jpg	https://data.globe.gov/system/photos/2019/10/02/1230490/original.jpg
GV_2608	45.5239	-122.7301	171.2		https://www.google.com/maps/search/45.5239,-122.7301	https://data.globe.gov/system/photos/2019/10/02/1230491/original.jpg	https://data.globe.gov/system/photos/2019/10/02/1230493/original.jpg	https://data.globe.gov/system/photos/2019/10/02/1230492/original.jpg	https://data.globe.gov/system/photos/2019/10/02/1230494/original.jpg	https://data.globe.gov/system/photos/2019/10/02/1230496/original.jpg
GV_2609	45.5273	-122.731	126.8		https://www.google.com/maps/search/45.5273,-122.731	https://data.globe.gov/system/photos/2019/10/02/1230497/original.jpg	https://data.globe.gov/system/photos/2019/10/02/1230499/original.jpg	https://data.globe.gov/system/photos/2019/10/02/1230498/original.jpg	https://data.globe.gov/system/photos/2019/10/02/1230500/original.jpg	https://data.globe.gov/system/photos/2019/10/02/1230502/original.jpg
GV_2610	44.5065	-123.2853	74.1		https://www.google.com/maps/search/44.5065,-123.2853	https://data.globe.gov/system/photos/2019/10/04/1230503/original.jpg	https://data.globe.gov/system/photos/2019/10/04/1230505/original.jpg	https://data.globe.gov/system/photos/2019/10/04/1230504/original.jpg	https://data.globe.gov/system/photos/2019/10/04/1230506/original.jpg	https://data.globe.gov/system/photos/2019/10/04/1230508/original.jpg
GV_2611	44.4976	-123.3156	74.3		https://www.google.com/maps/search/44.4976,-123.3156	https://data.globe.gov/system/photos/2019/10/05/1230509/original.jpg	https://data.globe.gov/system/photos/2019/10/05/1230511/original.jpg	https://data.globe.gov/system/photos/2019/10/05/1230510/original.jpg	https://data.globe.gov/system/photos/2019/10/05/1230512/original.jpg	https://data.globe.gov/system/photos/2019/10/05/1230514/original.jpg
GV_2612	44.7389	-123.0478	62.3		https://www.google.com/maps/search/44.7389,-123.0478	https://data.globe.gov/system/photos/2019/10/05/1230515/original.jpg	https://data.globe.gov/system/photos/2019/10/05/1230517/original.jpg	https://data.globe.gov/system/photos/2019/10/05/1230516/original.jpg	https://data.globe.gov/system/photos/2019/10/05/1230518/original.jpg	https://data.globe.gov/system/photos/2019/10/05/1230520/original.jpg
GV_2613	-34.4626	138.8181	177.4		https://www.google.com/maps/search/-34.4626,138.8181	https://data.globe.gov/system/photos/2019/10/05/1230673/original.jpg	https://data.globe.gov/system/photos/2019/10/05/1230675/original.jpg	https://data.globe.gov/system/photos/2019/10/05/1230674/original.jpg	https://data.globe.gov/system/photos/2019/10/05/1230676/original.jpg	https://data.globe.gov/system/photos/2019/10/05/1230678/original.jpg
GV_2614	50.5493	21.669	158.9		https://www.google.com/maps/search/50.5493,21.669	https://data.globe.gov/system/photos/2019/10/05/1230819/original.jpg	https://data.globe.gov/system/photos/2019/10/05/1230821/original.jpg	https://data.globe.gov/system/photos/2019/10/05/1230820/original.jpg	https://data.globe.gov/system/photos/2019/10/05/1230822/original.jpg	https://data.globe.gov/system/photos/2019/10/05/1230824/original.jpg
GV_2615	50.5359	21.6767	160		https://www.google.com/maps/search/50.5359,21.6767	https://data.globe.gov/system/photos/2019/10/05/1230825/original.jpg	https://data.globe.gov/system/photos/2019/10/05/1230827/original.jpg	https://data.globe.gov/system/photos/2019/10/05/1230826/original.jpg	https://data.globe.gov/system/photos/2019/10/05/1230828/original.jpg	https://data.globe.gov/system/photos/2019/10/05/1230830/original.jpg
GV_2616	50.5356	21.6759	160.8		https://www.google.com/maps/search/50.5356,21.6759	https://data.globe.gov/system/photos/2019/10/05/1230831/original.jpg	https://data.globe.gov/system/photos/2019/10/05/1230833/original.jpg	https://data.globe.gov/system/photos/2019/10/05/1230832/original.jpg	https://data.globe.gov/system/photos/2019/10/05/1230834/original.jpg	https://data.globe.gov/system/photos/2019/10/05/1230836/original.jpg
GV_2617	45.4855	15.5444	109.8		https://www.google.com/maps/search/45.4855,15.5444	https://data.globe.gov/system/photos/2019/10/05/1230896/original.jpg	https://data.globe.gov/system/photos/2019/10/05/1230898/original.jpg	https://data.globe.gov/system/photos/2019/10/05/1230897/original.jpg	https://data.globe.gov/system/photos/2019/10/05/1230899/original.jpg	https://data.globe.gov/system/photos/2019/10/05/1230901/original.jpg
GV_2618	45.6287	15.4245	271.2		https://www.google.com/maps/search/45.6287,15.4245	https://data.globe.gov/system/photos/2019/10/06/1231718/original.jpg	https://data.globe.gov/system/photos/2019/10/06/1231720/original.jpg	https://data.globe.gov/system/photos/2019/10/06/1231719/original.jpg	https://data.globe.gov/system/photos/2019/10/06/1231721/original.jpg	https://data.globe.gov/system/photos/2019/10/06/1231723/original.jpg
GV_2619	50.5116	21.6251	166.4		https://www.google.com/maps/search/50.5116,21.6251	https://data.globe.gov/system/photos/2019/10/06/1231872/original.jpg	https://data.globe.gov/system/photos/2019/10/06/1231874/original.jpg	https://data.globe.gov/system/photos/2019/10/06/1231873/original.jpg	https://data.globe.gov/system/photos/2019/10/06/1231876/original.jpg	https://data.globe.gov/system/photos/2019/10/06/1231880/original.jpg
GV_2620	50.512	21.6255	165.2		https://www.google.com/maps/search/50.512,21.6255	https://data.globe.gov/system/photos/2019/10/06/1231881/original.jpg	https://data.globe.gov/system/photos/2019/10/06/1231883/original.jpg	https://data.globe.gov/system/photos/2019/10/06/1231882/original.jpg	https://data.globe.gov/system/photos/2019/10/06/1231884/original.jpg	https://data.globe.gov/system/photos/2019/10/06/1231886/original.jpg
GV_2621	50.5103	21.6295	166.8		https://www.google.com/maps/search/50.5103,21.6295	https://data.globe.gov/system/photos/2019/10/06/1231888/original.jpg	https://data.globe.gov/system/photos/2019/10/06/1231891/original.jpg	https://data.globe.gov/system/photos/2019/10/06/1231890/original.jpg	https://data.globe.gov/system/photos/2019/10/06/1231892/original.jpg	https://data.globe.gov/system/photos/2019/10/06/1231896/original.jpg
GV_2622	50.5122	21.628	163		https://www.google.com/maps/search/50.5122,21.628	https://data.globe.gov/system/photos/2019/10/06/1231897/original.jpg	https://data.globe.gov/system/photos/2019/10/06/1231900/original.jpg	https://data.globe.gov/system/photos/2019/10/06/1231899/original.jpg	https://data.globe.gov/system/photos/2019/10/06/1231901/original.jpg	https://data.globe.gov/system/photos/2019/10/06/1231903/original.jpg
GV_2623	50.5113	21.6282	164.8		https://www.google.com/maps/search/50.5113,21.6282	https://data.globe.gov/system/photos/2019/10/06/1231907/original.jpg	https://data.globe.gov/system/photos/2019/10/06/1231909/original.jpg	https://data.globe.gov/system/photos/2019/10/06/1231908/original.jpg	https://data.globe.gov/system/photos/2019/10/06/1231910/original.jpg	https://data.globe.gov/system/photos/2019/10/06/1231912/original.jpg
GV_2624	50.5104	21.6305	165.9		https://www.google.com/maps/search/50.5104,21.6305	https://data.globe.gov/system/photos/2019/10/06/1231913/original.jpg	https://data.globe.gov/system/photos/2019/10/06/1231915/original.jpg	https://data.globe.gov/system/photos/2019/10/06/1231914/original.jpg	https://data.globe.gov/system/photos/2019/10/06/1231916/original.jpg	https://data.globe.gov/system/photos/2019/10/06/1231918/original.jpg
GV_2625	50.5104	21.6284	167.8		https://www.google.com/maps/search/50.5104,21.6284	https://data.globe.gov/system/photos/2019/10/06/1231919/original.jpg	https://data.globe.gov/system/photos/2019/10/06/1231921/original.jpg	https://data.globe.gov/system/photos/2019/10/06/1231920/original.jpg	https://data.globe.gov/system/photos/2019/10/06/1231922/original.jpg	https://data.globe.gov/system/photos/2019/10/06/1231924/original.jpg
GV_2626	50.5099	21.6274	169.9		https://www.google.com/maps/search/50.5099,21.6274	https://data.globe.gov/system/photos/2019/10/06/1231925/original.jpg	https://data.globe.gov/system/photos/2019/10/06/1231927/original.jpg	https://data.globe.gov/system/photos/2019/10/06/1231926/original.jpg	https://data.globe.gov/system/photos/2019/10/06/1231928/original.jpg	https://data.globe.gov/system/photos/2019/10/06/1231930/original.jpg
GV_2627	50.5105	21.6253	169.4		https://www.google.com/maps/search/50.5105,21.6253	https://data.globe.gov/system/photos/2019/10/06/1231931/original.jpg	https://data.globe.gov/system/photos/2019/10/06/1231933/original.jpg	https://data.globe.gov/system/photos/2019/10/06/1231932/original.jpg	https://data.globe.gov/system/photos/2019/10/06/1231934/original.jpg	https://data.globe.gov/system/photos/2019/10/06/1231936/original.jpg
GV_2628	44.4126	-123.3323	90.3		https://www.google.com/maps/search/44.4126,-123.3323	https://data.globe.gov/system/photos/2019/10/04/1232089/original.jpg	https://data.globe.gov/system/photos/2019/10/04/1232091/original.jpg	https://data.globe.gov/system/photos/2019/10/04/1232090/original.jpg	https://data.globe.gov/system/photos/2019/10/04/1232092/original.jpg	https://data.globe.gov/system/photos/2019/10/04/1232094/original.jpg
GV_2629	39.1858	-86.7784	234.6		https://www.google.com/maps/search/39.1858,-86.7784	https://data.globe.gov/system/photos/2019/10/06/1232244/original.jpg	https://data.globe.gov/system/photos/2019/10/06/1232254/original.jpg	https://data.globe.gov/system/photos/2019/10/06/1232250/original.jpg	https://data.globe.gov/system/photos/2019/10/06/1232255/original.jpg	https://data.globe.gov/system/photos/2019/10/06/1232257/original.jpg
GV_2630	-33.5925	137.953	18.7		https://www.google.com/maps/search/-33.5925,137.953	https://data.globe.gov/system/photos/2019/10/06/1232486/original.jpg	https://data.globe.gov/system/photos/2019/10/06/1232488/original.jpg	https://data.globe.gov/system/photos/2019/10/06/1232487/original.jpg	https://data.globe.gov/system/photos/2019/10/06/1232489/original.jpg	https://data.globe.gov/system/photos/2019/10/06/1232491/original.jpg
GV_2631	45.4953	-122.6708	11.1		https://www.google.com/maps/search/45.4953,-122.6708	https://data.globe.gov/system/photos/2019/10/05/1232553/original.jpg	https://data.globe.gov/system/photos/2019/10/05/1232555/original.jpg	https://data.globe.gov/system/photos/2019/10/05/1232554/original.jpg	https://data.globe.gov/system/photos/2019/10/05/1232556/original.jpg	https://data.globe.gov/system/photos/2019/10/05/1232558/original.jpg
GV_2632	39.2831	-76.6016	3.4		https://www.google.com/maps/search/39.2831,-76.6016	https://data.globe.gov/system/photos/2019/10/06/1232559/original.jpg	https://data.globe.gov/system/photos/2019/10/06/1232561/original.jpg	https://data.globe.gov/system/photos/2019/10/06/1232560/original.jpg	https://data.globe.gov/system/photos/2019/10/06/1232562/original.jpg	https://data.globe.gov/system/photos/2019/10/06/1232564/original.jpg
GV_2633	39.2828	-76.6031	1.8		https://www.google.com/maps/search/39.2828,-76.6031	https://data.globe.gov/system/photos/2019/10/06/1232571/original.jpg	https://data.globe.gov/system/photos/2019/10/06/1232573/original.jpg	https://data.globe.gov/system/photos/2019/10/06/1232572/original.jpg	https://data.globe.gov/system/photos/2019/10/06/1232574/original.jpg	https://data.globe.gov/system/photos/2019/10/06/1232576/original.jpg
GV_2634	39.2822	-76.6012	1.6		https://www.google.com/maps/search/39.2822,-76.6012	https://data.globe.gov/system/photos/2019/10/06/1232577/original.jpg	https://data.globe.gov/system/photos/2019/10/06/1232579/original.jpg	https://data.globe.gov/system/photos/2019/10/06/1232578/original.jpg	https://data.globe.gov/system/photos/2019/10/06/1232580/original.jpg	https://data.globe.gov/system/photos/2019/10/06/1232582/original.jpg
GV_2635	-28.5549	134.2565	278.2		https://www.google.com/maps/search/-28.5549,134.2565	https://data.globe.gov/system/photos/2019/10/06/1232584/original.jpg	https://data.globe.gov/system/photos/2019/10/06/1232586/original.jpg	https://data.globe.gov/system/photos/2019/10/06/1232585/original.jpg	https://data.globe.gov/system/photos/2019/10/06/1232587/original.jpg	https://data.globe.gov/system/photos/2019/10/06/1232589/original.jpg
GV_2636	-28.5549	134.2565	278.2		https://www.google.com/maps/search/-28.5549,134.2565	https://data.globe.gov/system/photos/2019/10/06/1232590/original.jpg	https://data.globe.gov/system/photos/2019/10/06/1232592/original.jpg	https://data.globe.gov/system/photos/2019/10/06/1232591/original.jpg	https://data.globe.gov/system/photos/2019/10/06/1232593/original.jpg	https://data.globe.gov/system/photos/2019/10/06/1232595/original.jpg
GV_2637	-29.0147	134.7998	209		https://www.google.com/maps/search/-29.0147,134.7998	https://data.globe.gov/system/photos/2019/10/07/1232596/original.jpg	https://data.globe.gov/system/photos/2019/10/07/1232598/original.jpg	https://data.globe.gov/system/photos/2019/10/07/1232597/original.jpg	https://data.globe.gov/system/photos/2019/10/07/1232599/original.jpg	https://data.globe.gov/system/photos/2019/10/07/1232601/original.jpg
GV_2638	44.4193	-123.3213	78		https://www.google.com/maps/search/44.4193,-123.3213	https://data.globe.gov/system/photos/2019/10/04/1232611/original.jpg	https://data.globe.gov/system/photos/2019/10/04/1232613/original.jpg	https://data.globe.gov/system/photos/2019/10/04/1232612/original.jpg	https://data.globe.gov/system/photos/2019/10/04/1232614/original.jpg	https://data.globe.gov/system/photos/2019/10/04/1232616/original.jpg
GV_2639	38.9361	-120.0545	1906		https://www.google.com/maps/search/38.9361,-120.0545	https://data.globe.gov/system/photos/2019/10/05/1232676/original.jpg	https://data.globe.gov/system/photos/2019/10/05/1232678/original.jpg	https://data.globe.gov/system/photos/2019/10/05/1232677/original.jpg	https://data.globe.gov/system/photos/2019/10/05/1232679/original.jpg	https://data.globe.gov/system/photos/2019/10/05/1232681/original.jpg
GV_2640	-35.0418	139.3811	55.4		https://www.google.com/maps/search/-35.0418,139.3811	https://data.globe.gov/system/photos/2019/09/30/1232711/original.jpg	https://data.globe.gov/system/photos/2019/09/30/1232713/original.jpg	https://data.globe.gov/system/photos/2019/09/30/1232712/original.jpg	https://data.globe.gov/system/photos/2019/09/30/1232714/original.jpg	https://data.globe.gov/system/photos/2019/09/30/1232716/original.jpg
GV_2641	-29.1008	137.6983	-6.4		https://www.google.com/maps/search/-29.1008,137.6983	https://data.globe.gov/system/photos/2019/10/07/1232722/original.jpg	https://data.globe.gov/system/photos/2019/10/07/1232724/original.jpg	https://data.globe.gov/system/photos/2019/10/07/1232723/original.jpg	https://data.globe.gov/system/photos/2019/10/07/1232725/original.jpg	https://data.globe.gov/system/photos/2019/10/07/1232727/original.jpg
GV_2642	50.4956	21.6833	149		https://www.google.com/maps/search/50.4956,21.6833	https://data.globe.gov/system/photos/2019/10/06/1232975/original.jpg	https://data.globe.gov/system/photos/2019/10/06/1232977/original.jpg	https://data.globe.gov/system/photos/2019/10/06/1232976/original.jpg	https://data.globe.gov/system/photos/2019/10/06/1232978/original.jpg	https://data.globe.gov/system/photos/2019/10/06/1232980/original.jpg
GV_2643	45.4988	15.5516	111.9		https://www.google.com/maps/search/45.4988,15.5516	https://data.globe.gov/system/photos/2019/10/07/1233029/original.jpg	https://data.globe.gov/system/photos/2019/10/07/1233031/original.jpg	https://data.globe.gov/system/photos/2019/10/07/1233030/original.jpg	https://data.globe.gov/system/photos/2019/10/07/1233032/original.jpg	https://data.globe.gov/system/photos/2019/10/07/1233034/original.jpg
GV_2644	50.4964	21.6881	149.5		https://www.google.com/maps/search/50.4964,21.6881	https://data.globe.gov/system/photos/2019/10/07/1233089/original.jpg	https://data.globe.gov/system/photos/2019/10/07/1233091/original.jpg	https://data.globe.gov/system/photos/2019/10/07/1233090/original.jpg	https://data.globe.gov/system/photos/2019/10/07/1233092/original.jpg	https://data.globe.gov/system/photos/2019/10/07/1233094/original.jpg
GV_2645	39.2851	-76.6042	2		https://www.google.com/maps/search/39.2851,-76.6042	https://data.globe.gov/system/photos/2019/10/07/1233404/original.jpg	https://data.globe.gov/system/photos/2019/10/07/1233407/original.jpg	https://data.globe.gov/system/photos/2019/10/07/1233406/original.jpg	https://data.globe.gov/system/photos/2019/10/07/1233408/original.jpg	https://data.globe.gov/system/photos/2019/10/07/1233410/original.jpg
GV_2646	39.2846	-76.6029	2.6		https://www.google.com/maps/search/39.2846,-76.6029	https://data.globe.gov/system/photos/2019/10/07/1233411/original.jpg	https://data.globe.gov/system/photos/2019/10/07/1233413/original.jpg	https://data.globe.gov/system/photos/2019/10/07/1233412/original.jpg	https://data.globe.gov/system/photos/2019/10/07/1233414/original.jpg	https://data.globe.gov/system/photos/2019/10/07/1233416/original.jpg
GV_2647	39.2831	-76.6032	2.3		https://www.google.com/maps/search/39.2831,-76.6032	https://data.globe.gov/system/photos/2019/10/07/1233417/original.jpg	https://data.globe.gov/system/photos/2019/10/07/1233419/original.jpg	https://data.globe.gov/system/photos/2019/10/07/1233418/original.jpg	https://data.globe.gov/system/photos/2019/10/07/1233420/original.jpg	https://data.globe.gov/system/photos/2019/10/07/1233422/original.jpg
GV_2648	39.2837	-76.6035	2.1		https://www.google.com/maps/search/39.2837,-76.6035	https://data.globe.gov/system/photos/2019/10/07/1233424/original.jpg	https://data.globe.gov/system/photos/2019/10/07/1233430/original.jpg	https://data.globe.gov/system/photos/2019/10/07/1233428/original.jpg	https://data.globe.gov/system/photos/2019/10/07/1233432/original.jpg	https://data.globe.gov/system/photos/2019/10/07/1233434/original.jpg
GV_2649	39.2842	-76.6039	2.3		https://www.google.com/maps/search/39.2842,-76.6039	https://data.globe.gov/system/photos/2019/10/07/1233435/original.jpg	https://data.globe.gov/system/photos/2019/10/07/1233443/original.jpg	https://data.globe.gov/system/photos/2019/10/07/1233441/original.jpg	https://data.globe.gov/system/photos/2019/10/07/1233444/original.jpg	https://data.globe.gov/system/photos/2019/10/07/1233448/original.jpg
GV_2650	39.2823	-76.602	0		https://www.google.com/maps/search/39.2823,-76.602	https://data.globe.gov/system/photos/2019/10/07/1233632/original.jpg	https://data.globe.gov/system/photos/2019/10/07/1233634/original.jpg	https://data.globe.gov/system/photos/2019/10/07/1233633/original.jpg	https://data.globe.gov/system/photos/2019/10/07/1233635/original.jpg	https://data.globe.gov/system/photos/2019/10/07/1233637/original.jpg
GV_2651	44.4127	-123.3317	88		https://www.google.com/maps/search/44.4127,-123.3317	https://data.globe.gov/system/photos/2019/10/04/1233788/original.jpg	https://data.globe.gov/system/photos/2019/10/04/1233790/original.jpg	https://data.globe.gov/system/photos/2019/10/04/1233789/original.jpg	https://data.globe.gov/system/photos/2019/10/04/1233791/original.jpg	https://data.globe.gov/system/photos/2019/10/04/1233793/original.jpg
GV_2652	44.0901	-123.3534	121.9		https://www.google.com/maps/search/44.0901,-123.3534	https://data.globe.gov/system/photos/2019/10/07/1233794/original.jpg	https://data.globe.gov/system/photos/2019/10/07/1233796/original.jpg	https://data.globe.gov/system/photos/2019/10/07/1233795/original.jpg	https://data.globe.gov/system/photos/2019/10/07/1233797/original.jpg	https://data.globe.gov/system/photos/2019/10/07/1233799/original.jpg
GV_2653	44.0899	-123.3534	121.5		https://www.google.com/maps/search/44.0899,-123.3534	https://data.globe.gov/system/photos/2019/10/07/1233800/original.jpg	https://data.globe.gov/system/photos/2019/10/07/1233802/original.jpg	https://data.globe.gov/system/photos/2019/10/07/1233801/original.jpg	https://data.globe.gov/system/photos/2019/10/07/1233803/original.jpg	https://data.globe.gov/system/photos/2019/10/07/1233805/original.jpg
GV_2654	10.5561	-61.3724	14		https://www.google.com/maps/search/10.5561,-61.3724	https://data.globe.gov/system/photos/2019/10/07/1233877/original.jpg	https://data.globe.gov/system/photos/2019/10/07/1233879/original.jpg	https://data.globe.gov/system/photos/2019/10/07/1233878/original.jpg	https://data.globe.gov/system/photos/2019/10/07/1233880/original.jpg	https://data.globe.gov/system/photos/2019/10/07/1233882/original.jpg
GV_2655	-29.0623	138.438	32.9		https://www.google.com/maps/search/-29.0623,138.438	https://data.globe.gov/system/photos/2019/10/07/1234244/original.jpg	https://data.globe.gov/system/photos/2019/10/07/1234246/original.jpg	https://data.globe.gov/system/photos/2019/10/07/1234245/original.jpg	https://data.globe.gov/system/photos/2019/10/07/1234247/original.jpg	https://data.globe.gov/system/photos/2019/10/07/1234249/original.jpg
GV_2656	-28.0195	138.6648	59.5		https://www.google.com/maps/search/-28.0195,138.6648	https://data.globe.gov/system/photos/2019/10/07/1234250/original.jpg	https://data.globe.gov/system/photos/2019/10/07/1234254/original.jpg	https://data.globe.gov/system/photos/2019/10/07/1234251/original.jpg	https://data.globe.gov/system/photos/2019/10/07/1234258/original.jpg	https://data.globe.gov/system/photos/2019/10/07/1234263/original.jpg
GV_2657	-27.6987	138.7463	16		https://www.google.com/maps/search/-27.6987,138.7463	https://data.globe.gov/system/photos/2019/10/08/1234268/original.jpg	https://data.globe.gov/system/photos/2019/10/08/1234270/original.jpg	https://data.globe.gov/system/photos/2019/10/08/1234269/original.jpg	https://data.globe.gov/system/photos/2019/10/08/1234271/original.jpg	https://data.globe.gov/system/photos/2019/10/08/1234273/original.jpg
GV_2658	-27.6987	138.7463	16		https://www.google.com/maps/search/-27.6987,138.7463	https://data.globe.gov/system/photos/2019/10/08/1234274/original.jpg	https://data.globe.gov/system/photos/2019/10/08/1234276/original.jpg	https://data.globe.gov/system/photos/2019/10/08/1234275/original.jpg	https://data.globe.gov/system/photos/2019/10/08/1234277/original.jpg	https://data.globe.gov/system/photos/2019/10/08/1234279/original.jpg
GV_2659	-26.9372	139.4175	37		https://www.google.com/maps/search/-26.9372,139.4175	https://data.globe.gov/system/photos/2019/10/08/1234280/original.jpg	https://data.globe.gov/system/photos/2019/10/08/1234282/original.jpg	https://data.globe.gov/system/photos/2019/10/08/1234281/original.jpg	https://data.globe.gov/system/photos/2019/10/08/1234283/original.jpg	https://data.globe.gov/system/photos/2019/10/08/1234285/original.jpg
GV_2660	-26.9372	139.4175	37		https://www.google.com/maps/search/-26.9372,139.4175	https://data.globe.gov/system/photos/2019/10/08/1234286/original.jpg	https://data.globe.gov/system/photos/2019/10/08/1234288/original.jpg	https://data.globe.gov/system/photos/2019/10/08/1234287/original.jpg	https://data.globe.gov/system/photos/2019/10/08/1234289/original.jpg	https://data.globe.gov/system/photos/2019/10/08/1234291/original.jpg
GV_2661	-25.997	139.3765	39		https://www.google.com/maps/search/-25.997,139.3765	https://data.globe.gov/system/photos/2019/10/08/1234292/original.jpg	https://data.globe.gov/system/photos/2019/10/08/1234294/original.jpg	https://data.globe.gov/system/photos/2019/10/08/1234293/original.jpg	https://data.globe.gov/system/photos/2019/10/08/1234295/original.jpg	https://data.globe.gov/system/photos/2019/10/08/1234297/original.jpg
GV_2662	-25.9094	139.3642	40.2		https://www.google.com/maps/search/-25.9094,139.3642	https://data.globe.gov/system/photos/2019/10/08/1234352/original.jpg	https://data.globe.gov/system/photos/2019/10/08/1234354/original.jpg	https://data.globe.gov/system/photos/2019/10/08/1234353/original.jpg	https://data.globe.gov/system/photos/2019/10/08/1234355/original.jpg	https://data.globe.gov/system/photos/2019/10/08/1234357/original.jpg
GV_2663	50.4964	21.6881	149.5		https://www.google.com/maps/search/50.4964,21.6881	https://data.globe.gov/system/photos/2019/10/08/1234703/original.jpg	https://data.globe.gov/system/photos/2019/10/08/1234705/original.jpg	https://data.globe.gov/system/photos/2019/10/08/1234704/original.jpg	https://data.globe.gov/system/photos/2019/10/08/1234706/original.jpg	https://data.globe.gov/system/photos/2019/10/08/1234708/original.jpg
GV_2664	50.508	21.6693	146.4		https://www.google.com/maps/search/50.508,21.6693	https://data.globe.gov/system/photos/2019/10/08/1235297/original.jpg	https://data.globe.gov/system/photos/2019/10/08/1235299/original.jpg	https://data.globe.gov/system/photos/2019/10/08/1235298/original.jpg	https://data.globe.gov/system/photos/2019/10/08/1235300/original.jpg	https://data.globe.gov/system/photos/2019/10/08/1235302/original.jpg
GV_2665	50.5111	21.6673	146.1		https://www.google.com/maps/search/50.5111,21.6673	https://data.globe.gov/system/photos/2019/10/08/1235309/original.jpg	https://data.globe.gov/system/photos/2019/10/08/1235311/original.jpg	https://data.globe.gov/system/photos/2019/10/08/1235310/original.jpg	https://data.globe.gov/system/photos/2019/10/08/1235312/original.jpg	https://data.globe.gov/system/photos/2019/10/08/1235314/original.jpg
GV_2666	39.1857	-86.7782	235.7		https://www.google.com/maps/search/39.1857,-86.7782	https://data.globe.gov/system/photos/2019/10/08/1235499/original.jpg	https://data.globe.gov/system/photos/2019/10/08/1235507/original.jpg	https://data.globe.gov/system/photos/2019/10/08/1235506/original.jpg	https://data.globe.gov/system/photos/2019/10/08/1235508/original.jpg	https://data.globe.gov/system/photos/2019/10/08/1235515/original.jpg
GV_2667	44.09	-123.3534	122.1		https://www.google.com/maps/search/44.09,-123.3534	https://data.globe.gov/system/photos/2019/10/08/1235767/original.jpg	https://data.globe.gov/system/photos/2019/10/08/1235769/original.jpg	https://data.globe.gov/system/photos/2019/10/08/1235768/original.jpg	https://data.globe.gov/system/photos/2019/10/08/1235770/original.jpg	https://data.globe.gov/system/photos/2019/10/08/1235772/original.jpg
GV_2668	-25.8796	139.055	43.5		https://www.google.com/maps/search/-25.8796,139.055	https://data.globe.gov/system/photos/2019/10/09/1236019/original.jpg	https://data.globe.gov/system/photos/2019/10/09/1236021/original.jpg	https://data.globe.gov/system/photos/2019/10/09/1236020/original.jpg	https://data.globe.gov/system/photos/2019/10/09/1236022/original.jpg	https://data.globe.gov/system/photos/2019/10/09/1236024/original.jpg
GV_2669	40.4778	-3.8666	733.6		https://www.google.com/maps/search/40.4778,-3.8666	https://data.globe.gov/system/photos/2019/10/06/1236121/original.jpg	https://data.globe.gov/system/photos/2019/10/06/1236123/original.jpg	https://data.globe.gov/system/photos/2019/10/06/1236122/original.jpg	https://data.globe.gov/system/photos/2019/10/06/1236124/original.jpg	https://data.globe.gov/system/photos/2019/10/06/1236126/original.jpg
GV_2670	-25.6652	140.0476	64		https://www.google.com/maps/search/-25.6652,140.0476	https://data.globe.gov/system/photos/2019/10/09/1236147/original.jpg	https://data.globe.gov/system/photos/2019/10/09/1236149/original.jpg	https://data.globe.gov/system/photos/2019/10/09/1236148/original.jpg	https://data.globe.gov/system/photos/2019/10/09/1236150/original.jpg	https://data.globe.gov/system/photos/2019/10/09/1236152/original.jpg
GV_2671	-25.9991	140.3679	70.9		https://www.google.com/maps/search/-25.9991,140.3679	https://data.globe.gov/system/photos/2019/10/09/1236153/original.jpg	https://data.globe.gov/system/photos/2019/10/09/1236155/original.jpg	https://data.globe.gov/system/photos/2019/10/09/1236154/original.jpg	https://data.globe.gov/system/photos/2019/10/09/1236156/original.jpg	https://data.globe.gov/system/photos/2019/10/09/1236158/original.jpg
GV_2672	-26.692	140.5274	88.8		https://www.google.com/maps/search/-26.692,140.5274	https://data.globe.gov/system/photos/2019/10/09/1236159/original.jpg	https://data.globe.gov/system/photos/2019/10/09/1236161/original.jpg	https://data.globe.gov/system/photos/2019/10/09/1236160/original.jpg	https://data.globe.gov/system/photos/2019/10/09/1236162/original.jpg	https://data.globe.gov/system/photos/2019/10/09/1236164/original.jpg
GV_2673	-26.9697	140.9683	72		https://www.google.com/maps/search/-26.9697,140.9683	https://data.globe.gov/system/photos/2019/10/09/1236165/original.jpg	https://data.globe.gov/system/photos/2019/10/09/1236167/original.jpg	https://data.globe.gov/system/photos/2019/10/09/1236166/original.jpg	https://data.globe.gov/system/photos/2019/10/09/1236168/original.jpg	https://data.globe.gov/system/photos/2019/10/09/1236170/original.jpg
GV_2674	-29.159	134.9316	215.7		https://www.google.com/maps/search/-29.159,134.9316	https://data.globe.gov/system/photos/2019/10/09/1236310/original.jpg	https://data.globe.gov/system/photos/2019/10/09/1236314/original.jpg	https://data.globe.gov/system/photos/2019/10/09/1236312/original.jpg	https://data.globe.gov/system/photos/2019/10/09/1236318/original.jpg	https://data.globe.gov/system/photos/2019/10/09/1236323/original.jpg
GV_2675	-31.1791	136.1685	162.1		https://www.google.com/maps/search/-31.1791,136.1685	https://data.globe.gov/system/photos/2019/10/09/1236324/original.jpg	https://data.globe.gov/system/photos/2019/10/09/1236326/original.jpg	https://data.globe.gov/system/photos/2019/10/09/1236325/original.jpg	https://data.globe.gov/system/photos/2019/10/09/1236327/original.jpg	https://data.globe.gov/system/photos/2019/10/09/1236329/original.jpg
GV_2676	50.5006	21.6506	146		https://www.google.com/maps/search/50.5006,21.6506	https://data.globe.gov/system/photos/2019/10/09/1236781/original.jpg	https://data.globe.gov/system/photos/2019/10/09/1236783/original.jpg	https://data.globe.gov/system/photos/2019/10/09/1236782/original.jpg	https://data.globe.gov/system/photos/2019/10/09/1236784/original.jpg	https://data.globe.gov/system/photos/2019/10/09/1236786/original.jpg
GV_2677	50.5009	21.6497	146.3		https://www.google.com/maps/search/50.5009,21.6497	https://data.globe.gov/system/photos/2019/10/09/1236787/original.jpg	https://data.globe.gov/system/photos/2019/10/09/1236789/original.jpg	https://data.globe.gov/system/photos/2019/10/09/1236788/original.jpg	https://data.globe.gov/system/photos/2019/10/09/1236790/original.jpg	https://data.globe.gov/system/photos/2019/10/09/1236792/original.jpg
GV_2678	50.4994	21.6517	147		https://www.google.com/maps/search/50.4994,21.6517	https://data.globe.gov/system/photos/2019/10/09/1236793/original.jpg	https://data.globe.gov/system/photos/2019/10/09/1236795/original.jpg	https://data.globe.gov/system/photos/2019/10/09/1236794/original.jpg	https://data.globe.gov/system/photos/2019/10/09/1236796/original.jpg	https://data.globe.gov/system/photos/2019/10/09/1236798/original.jpg
GV_2679	50.4994	21.6496	146.5		https://www.google.com/maps/search/50.4994,21.6496	https://data.globe.gov/system/photos/2019/10/09/1236805/original.jpg	https://data.globe.gov/system/photos/2019/10/09/1236807/original.jpg	https://data.globe.gov/system/photos/2019/10/09/1236806/original.jpg	https://data.globe.gov/system/photos/2019/10/09/1236808/original.jpg	https://data.globe.gov/system/photos/2019/10/09/1236810/original.jpg
GV_2680	50.4872	21.6556	146.5		https://www.google.com/maps/search/50.4872,21.6556	https://data.globe.gov/system/photos/2019/10/09/1236811/original.jpg	https://data.globe.gov/system/photos/2019/10/09/1236813/original.jpg	https://data.globe.gov/system/photos/2019/10/09/1236812/original.jpg	https://data.globe.gov/system/photos/2019/10/09/1236814/original.jpg	https://data.globe.gov/system/photos/2019/10/09/1236816/original.jpg
GV_2681	50.4887	21.6558	152.3		https://www.google.com/maps/search/50.4887,21.6558	https://data.globe.gov/system/photos/2019/10/09/1236817/original.jpg	https://data.globe.gov/system/photos/2019/10/09/1236819/original.jpg	https://data.globe.gov/system/photos/2019/10/09/1236818/original.jpg	https://data.globe.gov/system/photos/2019/10/09/1236820/original.jpg	https://data.globe.gov/system/photos/2019/10/09/1236822/original.jpg
GV_2682	50.4894	21.6552	154.3		https://www.google.com/maps/search/50.4894,21.6552	https://data.globe.gov/system/photos/2019/10/09/1236823/original.jpg	https://data.globe.gov/system/photos/2019/10/09/1236825/original.jpg	https://data.globe.gov/system/photos/2019/10/09/1236824/original.jpg	https://data.globe.gov/system/photos/2019/10/09/1236826/original.jpg	https://data.globe.gov/system/photos/2019/10/09/1236828/original.jpg
GV_2683	50.4894	21.6552	154.3		https://www.google.com/maps/search/50.4894,21.6552	https://data.globe.gov/system/photos/2019/10/09/1236829/original.jpg	https://data.globe.gov/system/photos/2019/10/09/1236831/original.jpg	https://data.globe.gov/system/photos/2019/10/09/1236830/original.jpg	https://data.globe.gov/system/photos/2019/10/09/1236832/original.jpg	https://data.globe.gov/system/photos/2019/10/09/1236834/original.jpg
GV_2684	50.491	21.6553	155		https://www.google.com/maps/search/50.491,21.6553	https://data.globe.gov/system/photos/2019/10/09/1236835/original.jpg	https://data.globe.gov/system/photos/2019/10/09/1236837/original.jpg	https://data.globe.gov/system/photos/2019/10/09/1236836/original.jpg	https://data.globe.gov/system/photos/2019/10/09/1236838/original.jpg	https://data.globe.gov/system/photos/2019/10/09/1236840/original.jpg
GV_2685	50.4888	21.6568	153.2		https://www.google.com/maps/search/50.4888,21.6568	https://data.globe.gov/system/photos/2019/10/09/1236841/original.jpg	https://data.globe.gov/system/photos/2019/10/09/1236843/original.jpg	https://data.globe.gov/system/photos/2019/10/09/1236842/original.jpg	https://data.globe.gov/system/photos/2019/10/09/1236844/original.jpg	https://data.globe.gov/system/photos/2019/10/09/1236846/original.jpg
GV_2686	50.4902	21.6585	147		https://www.google.com/maps/search/50.4902,21.6585	https://data.globe.gov/system/photos/2019/10/09/1236847/original.jpg	https://data.globe.gov/system/photos/2019/10/09/1236849/original.jpg	https://data.globe.gov/system/photos/2019/10/09/1236848/original.jpg	https://data.globe.gov/system/photos/2019/10/09/1236850/original.jpg	https://data.globe.gov/system/photos/2019/10/09/1236852/original.jpg
GV_2687	50.4897	21.6577	149.2		https://www.google.com/maps/search/50.4897,21.6577	https://data.globe.gov/system/photos/2019/10/09/1236853/original.jpg	https://data.globe.gov/system/photos/2019/10/09/1236855/original.jpg	https://data.globe.gov/system/photos/2019/10/09/1236854/original.jpg	https://data.globe.gov/system/photos/2019/10/09/1236856/original.jpg	https://data.globe.gov/system/photos/2019/10/09/1236858/original.jpg
GV_2688	50.489	21.657	153.8		https://www.google.com/maps/search/50.489,21.657	https://data.globe.gov/system/photos/2019/10/09/1236859/original.jpg	https://data.globe.gov/system/photos/2019/10/09/1236861/original.jpg	https://data.globe.gov/system/photos/2019/10/09/1236860/original.jpg	https://data.globe.gov/system/photos/2019/10/09/1236862/original.jpg	https://data.globe.gov/system/photos/2019/10/09/1236864/original.jpg
GV_2689	50.489	21.657	153.8		https://www.google.com/maps/search/50.489,21.657	https://data.globe.gov/system/photos/2019/10/09/1236865/original.jpg	https://data.globe.gov/system/photos/2019/10/09/1236867/original.jpg	https://data.globe.gov/system/photos/2019/10/09/1236866/original.jpg	https://data.globe.gov/system/photos/2019/10/09/1236868/original.jpg	https://data.globe.gov/system/photos/2019/10/09/1236870/original.jpg
GV_2690	50.5002	21.6442	157.4		https://www.google.com/maps/search/50.5002,21.6442	https://data.globe.gov/system/photos/2019/10/09/1236883/original.jpg	https://data.globe.gov/system/photos/2019/10/09/1236885/original.jpg	https://data.globe.gov/system/photos/2019/10/09/1236884/original.jpg	https://data.globe.gov/system/photos/2019/10/09/1236886/original.jpg	https://data.globe.gov/system/photos/2019/10/09/1236888/original.jpg
GV_2691	50.4973	21.6427	153.1		https://www.google.com/maps/search/50.4973,21.6427	https://data.globe.gov/system/photos/2019/10/09/1236889/original.jpg	https://data.globe.gov/system/photos/2019/10/09/1236891/original.jpg	https://data.globe.gov/system/photos/2019/10/09/1236890/original.jpg	https://data.globe.gov/system/photos/2019/10/09/1236892/original.jpg	https://data.globe.gov/system/photos/2019/10/09/1236894/original.jpg
GV_2692	50.4964	21.6418	152.6		https://www.google.com/maps/search/50.4964,21.6418	https://data.globe.gov/system/photos/2019/10/09/1236895/original.jpg	https://data.globe.gov/system/photos/2019/10/09/1236897/original.jpg	https://data.globe.gov/system/photos/2019/10/09/1236896/original.jpg	https://data.globe.gov/system/photos/2019/10/09/1236898/original.jpg	https://data.globe.gov/system/photos/2019/10/09/1236900/original.jpg
GV_2693	50.4964	21.6408	154.6		https://www.google.com/maps/search/50.4964,21.6408	https://data.globe.gov/system/photos/2019/10/09/1236901/original.jpg	https://data.globe.gov/system/photos/2019/10/09/1236903/original.jpg	https://data.globe.gov/system/photos/2019/10/09/1236902/original.jpg	https://data.globe.gov/system/photos/2019/10/09/1236908/original.jpg	https://data.globe.gov/system/photos/2019/10/09/1236917/original.jpg

GV_2694	50.4913	21.6601	147.1		https://www.google.com/maps/search/50.4913,21.6601	https://data.globe.gov/system/photos/2019/10/09/1236928/original.jpg	https://data.globe.gov/system/photos/2019/10/09/1236930/original.jpg	https://data.globe.gov/system/photos/2019/10/09/1236929/original.jpg	https://data.globe.gov/system/photos/2019/10/09/1236931/original.jpg	https://data.globe.gov/system/photos/2019/10/09/1236933/original.jpg
GV_2695	50.4985	21.6438	153.7		https://www.google.com/maps/search/50.4985,21.6438	https://data.globe.gov/system/photos/2019/10/09/1236934/original.jpg	https://data.globe.gov/system/photos/2019/10/09/1236936/original.jpg	https://data.globe.gov/system/photos/2019/10/09/1236935/original.jpg	https://data.globe.gov/system/photos/2019/10/09/1236937/original.jpg	https://data.globe.gov/system/photos/2019/10/09/1236939/original.jpg
GV_2696	50.4994	21.6431	156.1		https://www.google.com/maps/search/50.4994,21.6431	https://data.globe.gov/system/photos/2019/10/09/1236940/original.jpg	https://data.globe.gov/system/photos/2019/10/09/1236942/original.jpg	https://data.globe.gov/system/photos/2019/10/09/1236941/original.jpg	https://data.globe.gov/system/photos/2019/10/09/1236943/original.jpg	https://data.globe.gov/system/photos/2019/10/09/1236945/original.jpg
GV_2697	50.4994	21.6431	156.1		https://www.google.com/maps/search/50.4994,21.6431	https://data.globe.gov/system/photos/2019/10/09/1236946/original.jpg	https://data.globe.gov/system/photos/2019/10/09/1236948/original.jpg	https://data.globe.gov/system/photos/2019/10/09/1236947/original.jpg	https://data.globe.gov/system/photos/2019/10/09/1236949/original.jpg	https://data.globe.gov/system/photos/2019/10/09/1236951/original.jpg
GV_2698	50.5013	21.6433	158		https://www.google.com/maps/search/50.5013,21.6433	https://data.globe.gov/system/photos/2019/10/09/1236952/original.jpg	https://data.globe.gov/system/photos/2019/10/09/1236954/original.jpg	https://data.globe.gov/system/photos/2019/10/09/1236953/original.jpg	https://data.globe.gov/system/photos/2019/10/09/1236955/original.jpg	https://data.globe.gov/system/photos/2019/10/09/1236957/original.jpg
GV_2699	50.4997	21.6421	157.9		https://www.google.com/maps/search/50.4997,21.6421	https://data.globe.gov/system/photos/2019/10/09/1236958/original.jpg	https://data.globe.gov/system/photos/2019/10/09/1236961/original.jpg	https://data.globe.gov/system/photos/2019/10/09/1236959/original.jpg	https://data.globe.gov/system/photos/2019/10/09/1236964/original.jpg	https://data.globe.gov/system/photos/2019/10/09/1236969/original.jpg
GV_2700	50.4977	21.6475	146.9		https://www.google.com/maps/search/50.4977,21.6475	https://data.globe.gov/system/photos/2019/10/09/1237026/original.jpg	https://data.globe.gov/system/photos/2019/10/09/1237028/original.jpg	https://data.globe.gov/system/photos/2019/10/09/1237027/original.jpg	https://data.globe.gov/system/photos/2019/10/09/1237029/original.jpg	https://data.globe.gov/system/photos/2019/10/09/1237031/original.jpg
GV_2701	50.4986	21.6481	147		https://www.google.com/maps/search/50.4986,21.6481	https://data.globe.gov/system/photos/2019/10/09/1237032/original.jpg	https://data.globe.gov/system/photos/2019/10/09/1237037/original.jpg	https://data.globe.gov/system/photos/2019/10/09/1237035/original.jpg	https://data.globe.gov/system/photos/2019/10/09/1237038/original.jpg	https://data.globe.gov/system/photos/2019/10/09/1237040/original.jpg
GV_2702	50.4984	21.647	148.9		https://www.google.com/maps/search/50.4984,21.647	https://data.globe.gov/system/photos/2019/10/09/1237041/original.jpg	https://data.globe.gov/system/photos/2019/10/09/1237043/original.jpg	https://data.globe.gov/system/photos/2019/10/09/1237042/original.jpg	https://data.globe.gov/system/photos/2019/10/09/1237044/original.jpg	https://data.globe.gov/system/photos/2019/10/09/1237046/original.jpg
GV_2703	50.497	21.6464	146.7		https://www.google.com/maps/search/50.497,21.6464	https://data.globe.gov/system/photos/2019/10/09/1237047/original.jpg	https://data.globe.gov/system/photos/2019/10/09/1237049/original.jpg	https://data.globe.gov/system/photos/2019/10/09/1237048/original.jpg	https://data.globe.gov/system/photos/2019/10/09/1237050/original.jpg	https://data.globe.gov/system/photos/2019/10/09/1237052/original.jpg
GV_2704	50.497	21.6447	148.8		https://www.google.com/maps/search/50.497,21.6447	https://data.globe.gov/system/photos/2019/10/09/1237053/original.jpg	https://data.globe.gov/system/photos/2019/10/09/1237055/original.jpg	https://data.globe.gov/system/photos/2019/10/09/1237054/original.jpg	https://data.globe.gov/system/photos/2019/10/09/1237056/original.jpg	https://data.globe.gov/system/photos/2019/10/09/1237058/original.jpg
GV_2705	50.4976	21.6439	151.7		https://www.google.com/maps/search/50.4976,21.6439	https://data.globe.gov/system/photos/2019/10/09/1237059/original.jpg	https://data.globe.gov/system/photos/2019/10/09/1237061/original.jpg	https://data.globe.gov/system/photos/2019/10/09/1237060/original.jpg	https://data.globe.gov/system/photos/2019/10/09/1237062/original.jpg	https://data.globe.gov/system/photos/2019/10/09/1237064/original.jpg
GV_2706	39.2823	-76.603	1		https://www.google.com/maps/search/39.2823,-76.603	https://data.globe.gov/system/photos/2019/10/08/1237362/original.jpg	https://data.globe.gov/system/photos/2019/10/08/1237364/original.jpg	https://data.globe.gov/system/photos/2019/10/08/1237363/original.jpg	https://data.globe.gov/system/photos/2019/10/08/1237365/original.jpg	https://data.globe.gov/system/photos/2019/10/08/1237367/original.jpg
GV_2707	42.0859	-83.2099	177.7		https://www.google.com/maps/search/42.0859,-83.2099	https://data.globe.gov/system/photos/2019/10/09/1237516/original.jpg	https://data.globe.gov/system/photos/2019/10/09/1237518/original.jpg	https://data.globe.gov/system/photos/2019/10/09/1237517/original.jpg	https://data.globe.gov/system/photos/2019/10/09/1237519/original.jpg	https://data.globe.gov/system/photos/2019/10/09/1237521/original.jpg
GV_2708	39.03	-97.5984	372.2		https://www.google.com/maps/search/39.03,-97.5984	https://data.globe.gov/system/photos/2019/10/09/1237694/original.jpg	https://data.globe.gov/system/photos/2019/10/09/1237698/original.jpg	https://data.globe.gov/system/photos/2019/10/09/1237697/original.jpg	https://data.globe.gov/system/photos/2019/10/09/1237699/original.jpg	https://data.globe.gov/system/photos/2019/10/09/1237702/original.jpg
GV_2709	38.569	-121.5021	4.4		https://www.google.com/maps/search/38.569,-121.5021	https://data.globe.gov/system/photos/2019/10/09/1237753/original.jpg	https://data.globe.gov/system/photos/2019/10/09/1237755/original.jpg	https://data.globe.gov/system/photos/2019/10/09/1237754/original.jpg	https://data.globe.gov/system/photos/2019/10/09/1237756/original.jpg	https://data.globe.gov/system/photos/2019/10/09/1237758/original.jpg
GV_2710	38.5692	-121.5015	5		https://www.google.com/maps/search/38.5692,-121.5015	https://data.globe.gov/system/photos/2019/10/09/1237760/original.jpg	https://data.globe.gov/system/photos/2019/10/09/1237762/original.jpg	https://data.globe.gov/system/photos/2019/10/09/1237761/original.jpg	https://data.globe.gov/system/photos/2019/10/09/1237763/original.jpg	https://data.globe.gov/system/photos/2019/10/09/1237765/original.jpg
GV_2711	-31.2471	136.5463	134.1		https://www.google.com/maps/search/-31.2471,136.5463	https://data.globe.gov/system/photos/2019/10/09/1237986/original.jpg	https://data.globe.gov/system/photos/2019/10/09/1237989/original.jpg	https://data.globe.gov/system/photos/2019/10/09/1237987/original.jpg	https://data.globe.gov/system/photos/2019/10/09/1238002/original.jpg	https://data.globe.gov/system/photos/2019/10/09/1238006/original.jpg
GV_2712	-31.2487	136.749	192.7		https://www.google.com/maps/search/-31.2487,136.749	https://data.globe.gov/system/photos/2019/10/09/1238033/original.jpg	https://data.globe.gov/system/photos/2019/10/09/1238035/original.jpg	https://data.globe.gov/system/photos/2019/10/09/1238034/original.jpg	https://data.globe.gov/system/photos/2019/10/09/1238036/original.jpg	https://data.globe.gov/system/photos/2019/10/09/1238038/original.jpg
GV_2713	-31.7735	137.2906	104.5		https://www.google.com/maps/search/-31.7735,137.2906	https://data.globe.gov/system/photos/2019/10/10/1238228/original.jpg	https://data.globe.gov/system/photos/2019/10/10/1238231/original.jpg	https://data.globe.gov/system/photos/2019/10/10/1238230/original.jpg	https://data.globe.gov/system/photos/2019/10/10/1238232/original.jpg	https://data.globe.gov/system/photos/2019/10/10/1238237/original.jpg
GV_2714	-27.7555	140.5998	51.2		https://www.google.com/maps/search/-27.7555,140.5998	https://data.globe.gov/system/photos/2019/10/10/1238347/original.jpg	https://data.globe.gov/system/photos/2019/10/10/1238349/original.jpg	https://data.globe.gov/system/photos/2019/10/10/1238348/original.jpg	https://data.globe.gov/system/photos/2019/10/10/1238350/original.jpg	https://data.globe.gov/system/photos/2019/10/10/1238352/original.jpg
GV_2715	-28.1324	140.2115	46.4		https://www.google.com/maps/search/-28.1324,140.2115	https://data.globe.gov/system/photos/2019/10/10/1238353/original.jpg	https://data.globe.gov/system/photos/2019/10/10/1238355/original.jpg	https://data.globe.gov/system/photos/2019/10/10/1238354/original.jpg	https://data.globe.gov/system/photos/2019/10/10/1238356/original.jpg	https://data.globe.gov/system/photos/2019/10/10/1238358/original.jpg
GV_2716	45.4855	15.5446	109.5		https://www.google.com/maps/search/45.4855,15.5446	https://data.globe.gov/system/photos/2019/10/10/1238639/original.jpg	https://data.globe.gov/system/photos/2019/10/10/1238641/original.jpg	https://data.globe.gov/system/photos/2019/10/10/1238640/original.jpg	https://data.globe.gov/system/photos/2019/10/10/1238642/original.jpg	https://data.globe.gov/system/photos/2019/10/10/1238644/original.jpg
GV_2717	50.4974	21.655	146.8		https://www.google.com/maps/search/50.4974,21.655	https://data.globe.gov/system/photos/2019/10/10/1238741/original.jpg	https://data.globe.gov/system/photos/2019/10/10/1238743/original.jpg	https://data.globe.gov/system/photos/2019/10/10/1238742/original.jpg	https://data.globe.gov/system/photos/2019/10/10/1238744/original.jpg	https://data.globe.gov/system/photos/2019/10/10/1238746/original.jpg
GV_2718	50.4985	21.6544	145.5		https://www.google.com/maps/search/50.4985,21.6544	https://data.globe.gov/system/photos/2019/10/10/1238747/original.jpg	https://data.globe.gov/system/photos/2019/10/10/1238749/original.jpg	https://data.globe.gov/system/photos/2019/10/10/1238748/original.jpg	https://data.globe.gov/system/photos/2019/10/10/1238750/original.jpg	https://data.globe.gov/system/photos/2019/10/10/1238753/original.jpg
GV_2719	50.4948	21.6524	146.4		https://www.google.com/maps/search/50.4948,21.6524	https://data.globe.gov/system/photos/2019/10/10/1238764/original.jpg	https://data.globe.gov/system/photos/2019/10/10/1238766/original.jpg	https://data.globe.gov/system/photos/2019/10/10/1238765/original.jpg	https://data.globe.gov/system/photos/2019/10/10/1238767/original.jpg	https://data.globe.gov/system/photos/2019/10/10/1238769/original.jpg
GV_2720	50.4942	21.6517	146		https://www.google.com/maps/search/50.4942,21.6517	https://data.globe.gov/system/photos/2019/10/10/1238770/original.jpg	https://data.globe.gov/system/photos/2019/10/10/1238772/original.jpg	https://data.globe.gov/system/photos/2019/10/10/1238771/original.jpg	https://data.globe.gov/system/photos/2019/10/10/1238773/original.jpg	https://data.globe.gov/system/photos/2019/10/10/1238776/original.jpg
GV_2721	50.4935	21.6513	146		https://www.google.com/maps/search/50.4935,21.6513	https://data.globe.gov/system/photos/2019/10/10/1238777/original.jpg	https://data.globe.gov/system/photos/2019/10/10/1238780/original.jpg	https://data.globe.gov/system/photos/2019/10/10/1238778/original.jpg	https://data.globe.gov/system/photos/2019/10/10/1238781/original.jpg	https://data.globe.gov/system/photos/2019/10/10/1238784/original.jpg
GV_2722	50.4952	21.6512	146		https://www.google.com/maps/search/50.4952,21.6512	https://data.globe.gov/system/photos/2019/10/10/1238786/original.jpg	https://data.globe.gov/system/photos/2019/10/10/1238789/original.jpg	https://data.globe.gov/system/photos/2019/10/10/1238787/original.jpg	https://data.globe.gov/system/photos/2019/10/10/1238791/original.jpg	https://data.globe.gov/system/photos/2019/10/10/1238794/original.jpg
GV_2723	50.4888	21.6505	148.7		https://www.google.com/maps/search/50.4888,21.6505	https://data.globe.gov/system/photos/2019/10/10/1238796/original.jpg	https://data.globe.gov/system/photos/2019/10/10/1238798/original.jpg	https://data.globe.gov/system/photos/2019/10/10/1238797/original.jpg	https://data.globe.gov/system/photos/2019/10/10/1238799/original.jpg	https://data.globe.gov/system/photos/2019/10/10/1238801/original.jpg
GV_2724	50.4925	21.6542	149.4		https://www.google.com/maps/search/50.4925,21.6542	https://data.globe.gov/system/photos/2019/10/10/1238802/original.jpg	https://data.globe.gov/system/photos/2019/10/10/1238804/original.jpg	https://data.globe.gov/system/photos/2019/10/10/1238803/original.jpg	https://data.globe.gov/system/photos/2019/10/10/1238805/original.jpg	https://data.globe.gov/system/photos/2019/10/10/1238807/original.jpg
GV_2725	50.4925	21.6542	149.4		https://www.google.com/maps/search/50.4925,21.6542	https://data.globe.gov/system/photos/2019/10/10/1238808/original.jpg	https://data.globe.gov/system/photos/2019/10/10/1238810/original.jpg	https://data.globe.gov/system/photos/2019/10/10/1238809/original.jpg	https://data.globe.gov/system/photos/2019/10/10/1238811/original.jpg	https://data.globe.gov/system/photos/2019/10/10/1238813/original.jpg
GV_2726	50.4934	21.6535	147		https://www.google.com/maps/search/50.4934,21.6535	https://data.globe.gov/system/photos/2019/10/10/1238814/original.jpg	https://data.globe.gov/system/photos/2019/10/10/1238816/original.jpg	https://data.globe.gov/system/photos/2019/10/10/1238815/original.jpg	https://data.globe.gov/system/photos/2019/10/10/1238817/original.jpg	https://data.globe.gov/system/photos/2019/10/10/1238822/original.jpg
GV_2727	50.4934	21.6535	147		https://www.google.com/maps/search/50.4934,21.6535	https://data.globe.gov/system/photos/2019/10/10/1238826/original.jpg	https://data.globe.gov/system/photos/2019/10/10/1238828/original.jpg	https://data.globe.gov/system/photos/2019/10/10/1238827/original.jpg	https://data.globe.gov/system/photos/2019/10/10/1238829/original.jpg	https://data.globe.gov/system/photos/2019/10/10/1238831/original.jpg
GV_2728	50.4917	21.6528	147.6		https://www.google.com/maps/search/50.4917,21.6528	https://data.globe.gov/system/photos/2019/10/10/1238832/original.jpg	https://data.globe.gov/system/photos/2019/10/10/1238834/original.jpg	https://data.globe.gov/system/photos/2019/10/10/1238833/original.jpg	https://data.globe.gov/system/photos/2019/10/10/1238835/original.jpg	https://data.globe.gov/system/photos/2019/10/10/1238837/original.jpg
GV_2729	50.4917	21.6528	147.6		https://www.google.com/maps/search/50.4917,21.6528	https://data.globe.gov/system/photos/2019/10/10/1238838/original.jpg	https://data.globe.gov/system/photos/2019/10/10/1238840/original.jpg	https://data.globe.gov/system/photos/2019/10/10/1238839/original.jpg	https://data.globe.gov/system/photos/2019/10/10/1238841/original.jpg	https://data.globe.gov/system/photos/2019/10/10/1238843/original.jpg
GV_2730	50.4907	21.6526	150.9		https://www.google.com/maps/search/50.4907,21.6526	https://data.globe.gov/system/photos/2019/10/10/1238844/original.jpg	https://data.globe.gov/system/photos/2019/10/10/1238846/original.jpg	https://data.globe.gov/system/photos/2019/10/10/1238845/original.jpg	https://data.globe.gov/system/photos/2019/10/10/1238847/original.jpg	https://data.globe.gov/system/photos/2019/10/10/1238849/original.jpg
GV_2731	50.4907	21.6526	150.9		https://www.google.com/maps/search/50.4907,21.6526	https://data.globe.gov/system/photos/2019/10/10/1238850/original.jpg	https://data.globe.gov/system/photos/2019/10/10/1238856/original.jpg	https://data.globe.gov/system/photos/2019/10/10/1238851/original.jpg	https://data.globe.gov/system/photos/2019/10/10/1238859/original.jpg	https://data.globe.gov/system/photos/2019/10/10/1238861/original.jpg
GV_2732	50.4891	21.6516	152.3		https://www.google.com/maps/search/50.4891,21.6516	https://data.globe.gov/system/photos/2019/10/10/1238862/original.jpg	https://data.globe.gov/system/photos/2019/10/10/1238864/original.jpg	https://data.globe.gov/system/photos/2019/10/10/1238863/original.jpg	https://data.globe.gov/system/photos/2019/10/10/1238865/original.jpg	https://data.globe.gov/system/photos/2019/10/10/1238867/original.jpg
GV_2733	50.4943	21.6564	148.3		https://www.google.com/maps/search/50.4943,21.6564	https://data.globe.gov/system/photos/2019/10/10/1238868/original.jpg	https://data.globe.gov/system/photos/2019/10/10/1238870/original.jpg	https://data.globe.gov/system/photos/2019/10/10/1238869/original.jpg	https://data.globe.gov/system/photos/2019/10/10/1238871/original.jpg	https://data.globe.gov/system/photos/2019/10/10/1238873/original.jpg
GV_2734	50.4933	21.656	151.7		https://www.google.com/maps/search/50.4933,21.656	https://data.globe.gov/system/photos/2019/10/10/1238874/original.jpg	https://data.globe.gov/system/photos/2019/10/10/1238876/original.jpg	https://data.globe.gov/system/photos/2019/10/10/1238875/original.jpg	https://data.globe.gov/system/photos/2019/10/10/1238877/original.jpg	https://data.globe.gov/system/photos/2019/10/10/1238879/original.jpg
GV_2735	50.4933	21.656	151.7		https://www.google.com/maps/search/50.4933,21.656	https://data.globe.gov/system/photos/2019/10/10/1238880/original.jpg	https://data.globe.gov/system/photos/2019/10/10/1238882/original.jpg	https://data.globe.gov/system/photos/2019/10/10/1238881/original.jpg	https://data.globe.gov/system/photos/2019/10/10/1238883/original.jpg	https://data.globe.gov/system/photos/2019/10/10/1238885/original.jpg
GV_2736	50.4918	21.6543	152		https://www.google.com/maps/search/50.4918,21.6543	https://data.globe.gov/system/photos/2019/10/10/1238886/original.jpg	https://data.globe.gov/system/photos/2019/10/10/1238888/original.jpg	https://data.globe.gov/system/photos/2019/10/10/1238887/original.jpg	https://data.globe.gov/system/photos/2019/10/10/1238889/original.jpg	https://data.globe.gov/system/photos/2019/10/10/1238891/original.jpg
GV_2737	50.4913	21.6532	149.9		https://www.google.com/maps/search/50.4913,21.6532	https://data.globe.gov/system/photos/2019/10/10/1238892/original.jpg	https://data.globe.gov/system/photos/2019/10/10/1238895/original.jpg	https://data.globe.gov/system/photos/2019/10/10/1238893/original.jpg	https://data.globe.gov/system/photos/2019/10/10/1238897/original.jpg	https://data.globe.gov/system/photos/2019/10/10/1238900/original.jpg
GV_2738	50.4907	21.6566	150		https://www.google.com/maps/search/50.4907,21.6566	https://data.globe.gov/system/photos/2019/10/10/1238901/original.jpg	https://data.globe.gov/system/photos/2019/10/10/1238903/original.jpg	https://data.globe.gov/system/photos/2019/10/10/1238902/original.jpg	https://data.globe.gov/system/photos/2019/10/10/1238904/original.jpg	https://data.globe.gov/system/photos/2019/10/10/1238906/original.jpg
GV_2739	50.4909	21.6565	150.6		https://www.google.com/maps/search/50.4909,21.6565	https://data.globe.gov/system/photos/2019/10/10/1238907/original.jpg	https://data.globe.gov/system/photos/2019/10/10/1238909/original.jpg	https://data.globe.gov/system/photos/2019/10/10/1238908/original.jpg	https://data.globe.gov/system/photos/2019/10/10/1238910/original.jpg	https://data.globe.gov/system/photos/2019/10/10/1238912/original.jpg
GV_2740	50.4917	21.6573	151.9		https://www.google.com/maps/search/50.4917,21.6573	https://data.globe.gov/system/photos/2019/10/10/1238913/original.jpg	https://data.globe.gov/system/photos/2019/10/10/1238915/original.jpg	https://data.globe.gov/system/photos/2019/10/10/1238914/original.jpg	https://data.globe.gov/system/photos/2019/10/10/1238916/original.jpg	https://data.globe.gov/system/photos/2019/10/10/1238918/original.jpg
GV_2741	50.4924	21.6579	152.9		https://www.google.com/maps/search/50.4924,21.6579	https://data.globe.gov/system/photos/2019/10/10/1238919/original.jpg	https://data.globe.gov/system/photos/2019/10/10/1238921/original.jpg	https://data.globe.gov/system/photos/2019/10/10/1238920/original.jpg	https://data.globe.gov/system/photos/2019/10/10/1238922/original.jpg	https://data.globe.gov/system/photos/2019/10/10/1238924/original.jpg
GV_2742	50.4929	21.6589	152.4		https://www.google.com/maps/search/50.4929,21.6589	https://data.globe.gov/system/photos/2019/10/10/1238925/original.jpg	https://data.globe.gov/system/photos/2019/10/10/1238927/original.jpg	https://data.globe.gov/system/photos/2019/10/10/1238926/original.jpg	https://data.globe.gov/system/photos/2019/10/10/1238928/original.jpg	https://data.globe.gov/system/photos/2019/10/10/1238930/original.jpg
GV_2743	50.4934	21.6624	147.2		https://www.google.com/maps/search/50.4934,21.6624	https://data.globe.gov/system/photos/2019/10/10/1238931/original.jpg	https://data.globe.gov/system/photos/2019/10/10/1238933/original.jpg	https://data.globe.gov/system/photos/2019/10/10/1238932/original.jpg	https://data.globe.gov/system/photos/2019/10/10/1238934/original.jpg	https://data.globe.gov/system/photos/2019/10/10/1238936/original.jpg
GV_2744	50.4935	21.6589	153.9		https://www.google.com/maps/search/50.4935,21.6589	https://data.globe.gov/system/photos/2019/10/10/1238937/original.jpg	https://data.globe.gov/system/photos/2019/10/10/1238939/original.jpg	https://data.globe.gov/system/photos/2019/10/10/1238938/original.jpg	https://data.globe.gov/system/photos/2019/10/10/1238940/original.jpg	https://data.globe.gov/system/photos/2019/10/10/1238942/original.jpg
GV_2745	50.4934	21.6584	154.4		https://www.google.com/maps/search/50.4934,21.6584	https://data.globe.gov/system/photos/2019/10/10/1238943/original.jpg	https://data.globe.gov/system/photos/2019/10/10/1238945/original.jpg	https://data.globe.gov/system/photos/2019/10/10/1238944/original.jpg	https://data.globe.gov/system/photos/2019/10/10/1238946/original.jpg	https://data.globe.gov/system/photos/2019/10/10/1238950/original.jpg
GV_2746	50.4932	21.6589	153.9		https://www.google.com/maps/search/50.4932,21.6589	https://data.globe.gov/system/photos/2019/10/10/1238954/original.jpg	https://data.globe.gov/system/photos/2019/10/10/1238957/original.jpg	https://data.globe.gov/system/photos/2019/10/10/1238956/original.jpg	https://data.globe.gov/system/photos/2019/10/10/1238958/original.jpg	https://data.globe.gov/system/photos/2019/10/10/1238960/original.jpg
GV_2747	50.4892	21.6577	150.6		https://www.google.com/maps/search/50.4892,21.6577	https://data.globe.gov/system/photos/2019/10/10/1238961/original.jpg	https://data.globe.gov/system/photos/2019/10/10/1238967/original.jpg	https://data.globe.gov/system/photos/2019/10/10/1238964/original.jpg	https://data.globe.gov/system/photos/2019/10/10/1238970/original.jpg	https://data.globe.gov/system/photos/2019/10/10/1238972/original.jpg
GV_2748	50.4922	21.6566	155.4		https://www.google.com/maps/search/50.4922,21.6566	https://data.globe.gov/system/photos/2019/10/10/1238973/original.jpg	https://data.globe.gov/system/photos/2019/10/10/1238975/original.jpg	https://data.globe.gov/system/photos/2019/10/10/1238974/original.jpg	https://data.globe.gov/system/photos/2019/10/10/1238976/original.jpg	https://data.globe.gov/system/photos/2019/10/10/1238978/original.jpg
GV_2749	44.7405	-123.0542	62.3		https://www.google.com/maps/search/44.7405,-123.0542	https://data.globe.gov/system/photos/2019/10/10/1239444/original.jpg	https://data.globe.gov/system/photos/2019/10/10/1239447/original.jpg	https://data.globe.gov/system/photos/2019/10/10/1239445/original.jpg	https://data.globe.gov/system/photos/2019/10/10/1239452/original.jpg	https://data.globe.gov/system/photos/2019/10/10/1239455/original.jpg
GV_2750	44.5668	-123.2846	75.2		https://www.google.com/maps/search/44.5668,-123.2846	https://data.globe.gov/system/photos/2019/10/10/1239456/original.jpg	https://data.globe.gov/system/photos/2019/10/10/1239458/original.jpg	https://data.globe.gov/system/photos/2019/10/10/1239457/original.jpg	https://data.globe.gov/system/photos/2019/10/10/1239459/original.jpg	https://data.globe.gov/system/photos/2019/10/10/1239461/original.jpg
GV_2751	44.5669	-123.2806	79.2		https://www.google.com/maps/search/44.5669,-123.2806	https://data.globe.gov/system/photos/2019/10/10/1239462/original.jpg	https://data.globe.gov/system/photos/2019/10/10/1239464/original.jpg	https://data.globe.gov/system/photos/2019/10/10/1239463/original.jpg	https://data.globe.gov/system/photos/2019/10/10/1239470/original.jpg	https://data.globe.gov/system/photos/2019/10/10/1239473/original.jpg
GV_2752	44.5668	-123.2793	78.8		https://www.google.com/maps/search/44.5668,-123.2793	https://data.globe.gov/system/photos/2019/10/10/1239474/original.jpg	https://data.globe.gov/system/photos/2019/10/10/1239476/original.jpg	https://data.globe.gov/system/photos/2019/10/10/1239475/original.jpg	https://data.globe.gov/system/photos/2019/10/10/1239477/original.jpg	https://data.globe.gov/system/photos/2019/10/10/1239481/original.jpg
GV_2753	41.5534	-83.6687	179.3		https://www.google.com/maps/search/41.5534,-83.6687	https://data.globe.gov/system/photos/2019/10/10/1239667/original.jpg	https://data.globe.gov/system/photos/2019/10/10/1239674/original.jpg	https://data.globe.gov/system/photos/2019/10/10/1239671/original.jpg	https://data.globe.gov/system/photos/2019/10/10/1239678/original.jpg	https://data.globe.gov/system/photos/2019/10/10/1239682/original.jpg
GV_2754	44.5657	-123.278	78		https://www.google.com/maps/search/44.5657,-123.278	https://data.globe.gov/system/photos/2019/10/10/1239947/original.jpg	https://data.globe.gov/system/photos/2019/10/10/1239949/original.jpg	https://data.globe.gov/system/photos/2019/10/10/1239948/original.jpg	https://data.globe.gov/system/photos/2019/10/10/1239950/original.jpg	https://data.globe.gov/system/photos/2019/10/10/1239952/original.jpg
GV_2755	44.5657	-123.2789	77.9		https://www.google.com/maps/search/44.5657,-123.2789	https://data.globe.gov/system/photos/2019/10/10/1239953/original.jpg	https://data.globe.gov/system/photos/2019/10/10/1239957/original.jpg	https://data.globe.gov/system/photos/2019/10/10/1239954/original.jpg	https://data.globe.gov/system/photos/2019/10/10/1239962/original.jpg	https://data.globe.gov/system/photos/2019/10/10/1239970/original.jpg
GV_2756	44.5657	-123.276	78		https://www.google.com/maps/search/44.5657,-123.276	https://data.globe.gov/system/photos/2019/10/10/1240071/original.jpg	https://data.globe.gov/system/photos/2019/10/10/1240073/original.jpg	https://data.globe.gov/system/photos/2019/10/10/1240072/original.jpg	https://data.globe.gov/system/photos/2019/10/10/1240074/original.jpg	https://data.globe.gov/system/photos/2019/10/10/1240076/original.jpg
GV_2757	-32.3642	140.1421	329.4		https://www.google.com/maps/search/-32.3642,140.1421	https://data.globe.gov/system/photos/2019/10/10/1240133/original.jpg	https://data.globe.gov/system/photos/2019/10/10/1240135/original.jpg	https://data.globe.gov/system/photos/2019/10/10/1240134/original.jpg	https://data.globe.gov/system/photos/2019/10/10/1240141/original.jpg	https://data.globe.gov/system/photos/2019/10/10/1240143/original.jpg
GV_2758	-32.0005	141.3105	298.5		https://www.google.com/maps/search/-32.0005,141.3105	https://data.globe.gov/system/photos/2019/10/11/1240144/original.jpg	https://data.globe.gov/system/photos/2019/10/11/1240146/original.jpg	https://data.globe.gov/system/photos/2019/10/11/1240145/original.jpg	https://data.globe.gov/system/photos/2019/10/11/1240147/original.jpg	https://data.globe.gov/system/photos/2019/10/11/1240149/original.jpg
GV_2759	-32.3642	140.1425	328.6		https://www.google.com/maps/search/-32.3642,140.1425	https://data.globe.gov/system/photos/2019/10/11/1240411/original.jpg	https://data.globe.gov/system/photos/2019/10/11/1240413/original.jpg	https://data.globe.gov/system/photos/2019/10/11/1240412/original.jpg	https://data.globe.gov/system/photos/2019/10/11/1240414/original.jpg	https://data.globe.gov/system/photos/2019/10/11/1240416/original.jpg
GV_2760	-32.1229	141.8273	144.4		https://www.google.com/maps/search/-32.1229,141.8273	https://data.globe.gov/system/photos/2019/10/11/1240423/original.jpg	https://data.globe.gov/system/photos/2019/10/11/1240425/original.jpg	https://data.globe.gov/system/photos/2019/10/11/1240424/original.jpg	https://data.globe.gov/system/photos/2019/10/11/1240426/original.jpg	https://data.globe.gov/system/photos/2019/10/11/1240428/original.jpg
GV_2761	45.4859	15.5451	108.5		https://www.google.com/maps/search/45.4859,15.5451	https://data.globe.gov/system/photos/2019/10/11/1240524/original.jpg	https://data.globe.gov/system/photos/2019/10/11/1240526/original.jpg	https://data.globe.gov/system/photos/2019/10/11/1240525/original.jpg	https://data.globe.gov/system/photos/2019/10/11/1240527/original.jpg	https://data.globe.gov/system/photos/2019/10/11/1240529/original.jpg
GV_2762	50.4972	21.6865	149.1		https://www.google.com/maps/search/50.4972,21.6865	https://data.globe.gov/system/photos/2019/10/11/1240593/original.jpg	https://data.globe.gov/system/photos/2019/10/11/1240595/original.jpg	https://data.globe.gov/system/photos/2019/10/11/1240594/original.jpg	https://data.globe.gov/system/photos/2019/10/11/1240596/original.jpg	https://data.globe.gov/system/photos/2019/10/11/1240598/original.jpg
GV_2763	44.5678	-123.2866	76.1		https://www.google.com/maps/search/44.5678,-123.2866	https://data.globe.gov/system/photos/2019/10/10/1240723/original.jpg	https://data.globe.gov/system/photos/2019/10/10/1240725/original.jpg	https://data.globe.gov/system/photos/2019/10/10/1240724/original.jpg	https://data.globe.gov/system/photos/2019/10/10/1240726/original.jpg	https://data.globe.gov/system/photos/2019/10/10/1240728/original.jpg
GV_2764	44.5679	-123.288	75.9		https://www.google.com/maps/search/44.5679,-123.288	https://data.globe.gov/system/photos/2019/10/10/1240729/original.jpg	https://data.globe.gov/system/photos/2019/10/10/1240731/original.jpg	https://data.globe.gov/system/photos/2019/10/10/1240730/original.jpg	https://data.globe.gov/system/photos/2019/10/10/1240732/original.jpg	https://data.globe.gov/system/photos/2019/10/10/1240734/original.jpg
GV_2765	44.5679	-123.2896	75.9		https://www.google.com/maps/search/44.5679,-123.2896	https://data.globe.gov/system/photos/2019/10/10/1240735/original.jpg	https://data.globe.gov/system/photos/2019/10/10/1240737/original.jpg	https://data.globe.gov/system/photos/2019/10/10/1240736/original.jpg	https://data.globe.gov/system/photos/2019/10/10/1240738/original.jpg	https://data.globe.gov/system/photos/2019/10/10/1240740/original.jpg
GV_2766	43.1172	-77.5378	127.3		https://www.google.com/maps/search/43.1172,-77.5378	https://data.globe.gov/system/photos/2019/10/11/1240831/original.jpg	https://data.globe.gov/system/photos/2019/10/11/1240833/original.jpg	https://data.globe.gov/system/photos/2019/10/11/1240832/original.jpg	https://data.globe.gov/system/photos/2019/10/11/1240834/original.jpg	https://data.globe.gov/system/photos/2019/10/11/1240836/original.jpg
GV_2767	34.2769	-77.8713	5.2		https://www.google.com/maps/search/34.2769,-77.8713	https://data.globe.gov/system/photos/2019/10/11/1241220/original.jpg	https://data.globe.gov/system/photos/2019/10/11/1241227/original.jpg	https://data.globe.gov/system/photos/2019/10/11/1241224/original.jpg	https://data.globe.gov/system/photos/2019/10/11/1241229/original.jpg	https://data.globe.gov/system/photos/2019/10/11/1241240/original.jpg
GV_2768	38.9933	-76.8401	63		https://www.google.com/maps/search/38.9933,-76.8401	https://data.globe.gov/system/photos/2019/10/10/1241370/original.jpg	https://data.globe.gov/system/photos/2019/10/10/1241372/original.jpg	https://data.globe.gov/system/photos/2019/10/10/1241371/original.jpg	https://data.globe.gov/system/photos/2019/10/10/1241373/original.jpg	https://data.globe.gov/system/photos/2019/10/10/1241375/original.jpg
GV_2769	10.6643	-61.5112	21.8		https://www.google.com/maps/search/10.6643,-61.5112	https://data.globe.gov/system/photos/2019/10/11/1241430/original.jpg	https://data.globe.gov/system/photos/2019/10/11/1241432/original.jpg	https://data.globe.gov/system/photos/2019/10/11/1241431/original.jpg	https://data.globe.gov/system/photos/2019/10/11/1241433/original.jpg	https://data.globe.gov/system/photos/2019/10/11/1241435/original.jpg
GV_2770	27.8809	-82.1781	17.5		https://www.google.com/maps/search/27.8809,-82.1781	https://data.globe.gov/system/photos/2019/10/11/1241501/original.jpg	https://data.globe.gov/system/photos/2019/10/11/1241503/original.jpg	https://data.globe.gov/system/photos/2019/10/11/1241502/original.jpg	https://data.globe.gov/system/photos/2019/10/11/1241504/original.jpg	https://data.globe.gov/system/photos/2019/10/11/1241506/original.jpg
GV_2771	-33.3251	144.8322	99		https://www.google.com/maps/search/-33.3251,144.8322	https://data.globe.gov/system/photos/2019/10/11/1241590/original.jpg	https://data.globe.gov/system/photos/2019/10/11/1241592/original.jpg	https://data.globe.gov/system/photos/2019/10/11/1241591/original.jpg	https://data.globe.gov/system/photos/2019/10/11/1241593/original.jpg	https://data.globe.gov/system/photos/2019/10/11/1241598/original.jpg
GV_2772	45.4867	15.5427	112.9		https://www.google.com/maps/search/45.4867,15.5427	https://data.globe.gov/system/photos/2019/10/12/1241913/original.jpg	https://data.globe.gov/system/photos/2019/10/12/1241915/original.jpg	https://data.globe.gov/system/photos/2019/10/12/1241914/original.jpg	https://data.globe.gov/system/photos/2019/10/12/1241916/original.jpg	https://data.globe.gov/system/photos/2019/10/12/1241918/original.jpg
GV_2773	50.5014	21.6592	146		https://www.google.com/maps/search/50.5014,21.6592	https://data.globe.gov/system/photos/2019/10/12/1242190/original.jpg	https://data.globe.gov/system/photos/2019/10/12/1242192/original.jpg	https://data.globe.gov/system/photos/2019/10/12/1242191/original.jpg	https://data.globe.gov/system/photos/2019/10/12/1242193/original.jpg	https://data.globe.gov/system/photos/2019/10/12/1242195/original.jpg
GV_2774	50.4933	21.6499	146.8		https://www.google.com/maps/search/50.4933,21.6499	https://data.globe.gov/system/photos/2019/10/12/1242196/original.jpg	https://data.globe.gov/system/photos/2019/10/12/1242198/original.jpg	https://data.globe.gov/system/photos/2019/10/12/1242197/original.jpg	https://data.globe.gov/system/photos/2019/10/12/1242199/original.jpg	https://data.globe.gov/system/photos/2019/10/12/1242201/original.jpg
GV_2775	50.4925	21.6495	145.4		https://www.google.com/maps/search/50.4925,21.6495	https://data.globe.gov/system/photos/2019/10/12/1242202/original.jpg	https://data.globe.gov/system/photos/2019/10/12/1242204/original.jpg	https://data.globe.gov/system/photos/2019/10/12/1242203/original.jpg	https://data.globe.gov/system/photos/2019/10/12/1242205/original.jpg	https://data.globe.gov/system/photos/2019/10/12/1242207/original.jpg
GV_2776	50.4923	21.6484	145.6		https://www.google.com/maps/search/50.4923,21.6484	https://data.globe.gov/system/photos/2019/10/12/1242208/original.jpg	https://data.globe.gov/system/photos/2019/10/12/1242210/original.jpg	https://data.globe.gov/system/photos/2019/10/12/1242209/original.jpg	https://data.globe.gov/system/photos/2019/10/12/1242211/original.jpg	https://data.globe.gov/system/photos/2019/10/12/1242213/original.jpg
GV_2777	50.4918	21.6476	146		https://www.google.com/maps/search/50.4918,21.6476	https://data.globe.gov/system/photos/2019/10/12/1242214/original.jpg	https://data.globe.gov/system/photos/2019/10/12/1242216/original.jpg	https://data.globe.gov/system/photos/2019/10/12/1242215/original.jpg	https://data.globe.gov/system/photos/2019/10/12/1242217/original.jpg	https://data.globe.gov/system/photos/2019/10/12/1242219/original.jpg
GV_2778	39.257	-76.7141	80.9		https://www.google.com/maps/search/39.257,-76.7141	https://data.globe.gov/system/photos/2019/10/12/1242374/original.jpg	https://data.globe.gov/system/photos/2019/10/12/1242378/original.jpg	https://data.globe.gov/system/photos/2019/10/12/1242376/original.jpg	https://data.globe.gov/system/photos/2019/10/12/1242380/original.jpg	https://data.globe.gov/system/photos/2019/10/12/1242382/original.jpg
GV_2779	10.5248	-61.4133	15.5		https://www.google.com/maps/search/10.5248,-61.4133	https://data.globe.gov/system/photos/2019/10/12/1243071/original.jpg	https://data.globe.gov/system/photos/2019/10/12/1243073/original.jpg	https://data.globe.gov/system/photos/2019/10/12/1243072/original.jpg	https://data.globe.gov/system/photos/2019/10/12/1243074/original.jpg	https://data.globe.gov/system/photos/2019/10/12/1243076/original.jpg
GV_2780	45.4012	-121.6543	1785.3		https://www.google.com/maps/search/45.4012,-121.6543	https://data.globe.gov/system/photos/2019/10/12/1243126/original.jpg	https://data.globe.gov/system/photos/2019/10/12/1243128/original.jpg	https://data.globe.gov/system/photos/2019/10/12/1243127/original.jpg	https://data.globe.gov/system/photos/2019/10/12/1243129/original.jpg	https://data.globe.gov/system/photos/2019/10/12/1243131/original.jpg
GV_2781	45.401	-121.6527	1784.1		https://www.google.com/maps/search/45.401,-121.6527	https://data.globe.gov/system/photos/2019/10/12/1243132/original.jpg	https://data.globe.gov/system/photos/2019/10/12/1243138/original.jpg	https://data.globe.gov/system/photos/2019/10/12/1243137/original.jpg	https://data.globe.gov/system/photos/2019/10/12/1243139/original.jpg	https://data.globe.gov/system/photos/2019/10/12/1243141/original.jpg
GV_2782	45.4006	-121.652	1767.6		https://www.google.com/maps/search/45.4006,-121.652	https://data.globe.gov/system/photos/2019/10/12/1243142/original.jpg	https://data.globe.gov/system/photos/2019/10/12/1243144/original.jpg	https://data.globe.gov/system/photos/2019/10/12/1243143/original.jpg	https://data.globe.gov/system/photos/2019/10/12/1243145/original.jpg	https://data.globe.gov/system/photos/2019/10/12/1243147/original.jpg
GV_2783	45.3991	-121.6474	1739.6		https://www.google.com/maps/search/45.3991,-121.6474	https://data.globe.gov/system/photos/2019/10/12/1243148/original.jpg	https://data.globe.gov/system/photos/2019/10/12/1243150/original.jpg	https://data.globe.gov/system/photos/2019/10/12/1243149/original.jpg	https://data.globe.gov/system/photos/2019/10/12/1243151/original.jpg	https://data.globe.gov/system/photos/2019/10/12/1243153/original.jpg

GV_2784	45.3988	-121.6466	1746.2		https://www.google.com/maps/search/45.3988,-121.6466	https://data.globe.gov/system/photos/2019/10/12/1243154/original.jpg	https://data.globe.gov/system/photos/2019/10/12/1243156/original.jpg	https://data.globe.gov/system/photos/2019/10/12/1243155/original.jpg	https://data.globe.gov/system/photos/2019/10/12/1243157/original.jpg	https://data.globe.gov/system/photos/2019/10/12/1243159/original.jpg
GV_2785	45.3981	-121.6437	1733.4		https://www.google.com/maps/search/45.3981,-121.6437	https://data.globe.gov/system/photos/2019/10/12/1243160/original.jpg	https://data.globe.gov/system/photos/2019/10/12/1243162/original.jpg	https://data.globe.gov/system/photos/2019/10/12/1243161/original.jpg	https://data.globe.gov/system/photos/2019/10/12/1243163/original.jpg	https://data.globe.gov/system/photos/2019/10/12/1243165/original.jpg
GV_2786	45.4015	-121.6345	1630.1		https://www.google.com/maps/search/45.4015,-121.6345	https://data.globe.gov/system/photos/2019/10/12/1243166/original.jpg	https://data.globe.gov/system/photos/2019/10/12/1243168/original.jpg	https://data.globe.gov/system/photos/2019/10/12/1243167/original.jpg	https://data.globe.gov/system/photos/2019/10/12/1243169/original.jpg	https://data.globe.gov/system/photos/2019/10/12/1243171/original.jpg
GV_2787	45.405	-121.6246	1477.7		https://www.google.com/maps/search/45.405,-121.6246	https://data.globe.gov/system/photos/2019/10/12/1243172/original.jpg	https://data.globe.gov/system/photos/2019/10/12/1243174/original.jpg	https://data.globe.gov/system/photos/2019/10/12/1243173/original.jpg	https://data.globe.gov/system/photos/2019/10/12/1243175/original.jpg	https://data.globe.gov/system/photos/2019/10/12/1243177/original.jpg
GV_2788	45.4097	-121.6159	1296.9		https://www.google.com/maps/search/45.4097,-121.6159	https://data.globe.gov/system/photos/2019/10/12/1243178/original.jpg	https://data.globe.gov/system/photos/2019/10/12/1243180/original.jpg	https://data.globe.gov/system/photos/2019/10/12/1243179/original.jpg	https://data.globe.gov/system/photos/2019/10/12/1243181/original.jpg	https://data.globe.gov/system/photos/2019/10/12/1243183/original.jpg
GV_2789	45.6242	-121.5156	221.9		https://www.google.com/maps/search/45.6242,-121.5156	https://data.globe.gov/system/photos/2019/10/12/1243264/original.jpg	https://data.globe.gov/system/photos/2019/10/12/1243266/original.jpg	https://data.globe.gov/system/photos/2019/10/12/1243265/original.jpg	https://data.globe.gov/system/photos/2019/10/12/1243267/original.jpg	https://data.globe.gov/system/photos/2019/10/12/1243269/original.jpg
GV_2790	45.5198	-121.5863	534.3		https://www.google.com/maps/search/45.5198,-121.5863	https://data.globe.gov/system/photos/2019/10/12/1243270/original.jpg	https://data.globe.gov/system/photos/2019/10/12/1243272/original.jpg	https://data.globe.gov/system/photos/2019/10/12/1243271/original.jpg	https://data.globe.gov/system/photos/2019/10/12/1243273/original.jpg	https://data.globe.gov/system/photos/2019/10/12/1243275/original.jpg
GV_2791	45.4144	-121.6026	1162.5		https://www.google.com/maps/search/45.4144,-121.6026	https://data.globe.gov/system/photos/2019/10/12/1243276/original.jpg	https://data.globe.gov/system/photos/2019/10/12/1243278/original.jpg	https://data.globe.gov/system/photos/2019/10/12/1243277/original.jpg	https://data.globe.gov/system/photos/2019/10/12/1243279/original.jpg	https://data.globe.gov/system/photos/2019/10/12/1243281/original.jpg
GV_2792	45.4182	-121.6085	1192.9		https://www.google.com/maps/search/45.4182,-121.6085	https://data.globe.gov/system/photos/2019/10/12/1243282/original.jpg	https://data.globe.gov/system/photos/2019/10/12/1243284/original.jpg	https://data.globe.gov/system/photos/2019/10/12/1243283/original.jpg	https://data.globe.gov/system/photos/2019/10/12/1243285/original.jpg	https://data.globe.gov/system/photos/2019/10/12/1243287/original.jpg
GV_2793	45.4214	-121.6134	1216.4		https://www.google.com/maps/search/45.4214,-121.6134	https://data.globe.gov/system/photos/2019/10/12/1243288/original.jpg	https://data.globe.gov/system/photos/2019/10/12/1243290/original.jpg	https://data.globe.gov/system/photos/2019/10/12/1243289/original.jpg	https://data.globe.gov/system/photos/2019/10/12/1243291/original.jpg	https://data.globe.gov/system/photos/2019/10/12/1243293/original.jpg
GV_2794	45.4236	-121.6327	1336.7		https://www.google.com/maps/search/45.4236,-121.6327	https://data.globe.gov/system/photos/2019/10/12/1243294/original.jpg	https://data.globe.gov/system/photos/2019/10/12/1243296/original.jpg	https://data.globe.gov/system/photos/2019/10/12/1243295/original.jpg	https://data.globe.gov/system/photos/2019/10/12/1243297/original.jpg	https://data.globe.gov/system/photos/2019/10/12/1243299/original.jpg
GV_2795	45.4245	-121.6271	1286.1		https://www.google.com/maps/search/45.4245,-121.6271	https://data.globe.gov/system/photos/2019/10/12/1243300/original.jpg	https://data.globe.gov/system/photos/2019/10/12/1243302/original.jpg	https://data.globe.gov/system/photos/2019/10/12/1243301/original.jpg	https://data.globe.gov/system/photos/2019/10/12/1243303/original.jpg	https://data.globe.gov/system/photos/2019/10/12/1243305/original.jpg
GV_2796	45.42	-121.6387	1411.9		https://www.google.com/maps/search/45.42,-121.6387	https://data.globe.gov/system/photos/2019/10/12/1243306/original.jpg	https://data.globe.gov/system/photos/2019/10/12/1243308/original.jpg	https://data.globe.gov/system/photos/2019/10/12/1243307/original.jpg	https://data.globe.gov/system/photos/2019/10/12/1243309/original.jpg	https://data.globe.gov/system/photos/2019/10/12/1243311/original.jpg
GV_2797	45.4169	-121.6404	1475.4		https://www.google.com/maps/search/45.4169,-121.6404	https://data.globe.gov/system/photos/2019/10/12/1243312/original.jpg	https://data.globe.gov/system/photos/2019/10/12/1243314/original.jpg	https://data.globe.gov/system/photos/2019/10/12/1243313/original.jpg	https://data.globe.gov/system/photos/2019/10/12/1243315/original.jpg	https://data.globe.gov/system/photos/2019/10/12/1243317/original.jpg
GV_2798	45.4151	-121.6465	1502		https://www.google.com/maps/search/45.4151,-121.6465	https://data.globe.gov/system/photos/2019/10/12/1243318/original.jpg	https://data.globe.gov/system/photos/2019/10/12/1243320/original.jpg	https://data.globe.gov/system/photos/2019/10/12/1243319/original.jpg	https://data.globe.gov/system/photos/2019/10/12/1243321/original.jpg	https://data.globe.gov/system/photos/2019/10/12/1243323/original.jpg
GV_2799	45.402	-121.6554	1786		https://www.google.com/maps/search/45.402,-121.6554	https://data.globe.gov/system/photos/2019/10/12/1243324/original.jpg	https://data.globe.gov/system/photos/2019/10/12/1243326/original.jpg	https://data.globe.gov/system/photos/2019/10/12/1243325/original.jpg	https://data.globe.gov/system/photos/2019/10/12/1243327/original.jpg	https://data.globe.gov/system/photos/2019/10/12/1243329/original.jpg
GV_2800	45.398	-121.6423	1716.7		https://www.google.com/maps/search/45.398,-121.6423	https://data.globe.gov/system/photos/2019/10/12/1243330/original.jpg	https://data.globe.gov/system/photos/2019/10/12/1243332/original.jpg	https://data.globe.gov/system/photos/2019/10/12/1243331/original.jpg	https://data.globe.gov/system/photos/2019/10/12/1243333/original.jpg	https://data.globe.gov/system/photos/2019/10/12/1243335/original.jpg
GV_2801	45.4034	-121.6281	1545.7		https://www.google.com/maps/search/45.4034,-121.6281	https://data.globe.gov/system/photos/2019/10/12/1243336/original.jpg	https://data.globe.gov/system/photos/2019/10/12/1243338/original.jpg	https://data.globe.gov/system/photos/2019/10/12/1243337/original.jpg	https://data.globe.gov/system/photos/2019/10/12/1243339/original.jpg	https://data.globe.gov/system/photos/2019/10/12/1243341/original.jpg
GV_2802	45.4062	-121.6203	1417.8		https://www.google.com/maps/search/45.4062,-121.6203	https://data.globe.gov/system/photos/2019/10/12/1243342/original.jpg	https://data.globe.gov/system/photos/2019/10/12/1243344/original.jpg	https://data.globe.gov/system/photos/2019/10/12/1243343/original.jpg	https://data.globe.gov/system/photos/2019/10/12/1243345/original.jpg	https://data.globe.gov/system/photos/2019/10/12/1243347/original.jpg
GV_2803	45.4129	-121.6074	1224.6		https://www.google.com/maps/search/45.4129,-121.6074	https://data.globe.gov/system/photos/2019/10/12/1243348/original.jpg	https://data.globe.gov/system/photos/2019/10/12/1243350/original.jpg	https://data.globe.gov/system/photos/2019/10/12/1243349/original.jpg	https://data.globe.gov/system/photos/2019/10/12/1243351/original.jpg	https://data.globe.gov/system/photos/2019/10/12/1243353/original.jpg
GV_2804	45.3102	-121.6424	1424.5		https://www.google.com/maps/search/45.3102,-121.6424	https://data.globe.gov/system/photos/2019/10/12/1243360/original.jpg	https://data.globe.gov/system/photos/2019/10/12/1243362/original.jpg	https://data.globe.gov/system/photos/2019/10/12/1243361/original.jpg	https://data.globe.gov/system/photos/2019/10/12/1243363/original.jpg	https://data.globe.gov/system/photos/2019/10/12/1243365/original.jpg
GV_2805	45.303	-121.746	1211.2		https://www.google.com/maps/search/45.303,-121.746	https://data.globe.gov/system/photos/2019/10/12/1243366/original.jpg	https://data.globe.gov/system/photos/2019/10/12/1243368/original.jpg	https://data.globe.gov/system/photos/2019/10/12/1243367/original.jpg	https://data.globe.gov/system/photos/2019/10/12/1243369/original.jpg	https://data.globe.gov/system/photos/2019/10/12/1243371/original.jpg
GV_2806	45.4178	-121.5932	1096.5		https://www.google.com/maps/search/45.4178,-121.5932	https://data.globe.gov/system/photos/2019/10/12/1243372/original.jpg	https://data.globe.gov/system/photos/2019/10/12/1243374/original.jpg	https://data.globe.gov/system/photos/2019/10/12/1243373/original.jpg	https://data.globe.gov/system/photos/2019/10/12/1243375/original.jpg	https://data.globe.gov/system/photos/2019/10/12/1243377/original.jpg
GV_2807	45.4145	-121.6041	1168.8		https://www.google.com/maps/search/45.4145,-121.6041	https://data.globe.gov/system/photos/2019/10/12/1243378/original.jpg	https://data.globe.gov/system/photos/2019/10/12/1243380/original.jpg	https://data.globe.gov/system/photos/2019/10/12/1243379/original.jpg	https://data.globe.gov/system/photos/2019/10/12/1243381/original.jpg	https://data.globe.gov/system/photos/2019/10/12/1243383/original.jpg
GV_2808	45.4204	-121.6094	1207.4		https://www.google.com/maps/search/45.4204,-121.6094	https://data.globe.gov/system/photos/2019/10/12/1243384/original.jpg	https://data.globe.gov/system/photos/2019/10/12/1243386/original.jpg	https://data.globe.gov/system/photos/2019/10/12/1243385/original.jpg	https://data.globe.gov/system/photos/2019/10/12/1243387/original.jpg	https://data.globe.gov/system/photos/2019/10/12/1243389/original.jpg
GV_2809	45.4216	-121.6125	1220.1		https://www.google.com/maps/search/45.4216,-121.6125	https://data.globe.gov/system/photos/2019/10/12/1243390/original.jpg	https://data.globe.gov/system/photos/2019/10/12/1243392/original.jpg	https://data.globe.gov/system/photos/2019/10/12/1243391/original.jpg	https://data.globe.gov/system/photos/2019/10/12/1243393/original.jpg	https://data.globe.gov/system/photos/2019/10/12/1243395/original.jpg
GV_2810	45.4227	-121.6146	1224.4		https://www.google.com/maps/search/45.4227,-121.6146	https://data.globe.gov/system/photos/2019/10/12/1243396/original.jpg	https://data.globe.gov/system/photos/2019/10/12/1243398/original.jpg	https://data.globe.gov/system/photos/2019/10/12/1243397/original.jpg	https://data.globe.gov/system/photos/2019/10/12/1243399/original.jpg	https://data.globe.gov/system/photos/2019/10/12/1243401/original.jpg
GV_2811	45.4238	-121.6254	1276.8		https://www.google.com/maps/search/45.4238,-121.6254	https://data.globe.gov/system/photos/2019/10/12/1243402/original.jpg	https://data.globe.gov/system/photos/2019/10/12/1243404/original.jpg	https://data.globe.gov/system/photos/2019/10/12/1243403/original.jpg	https://data.globe.gov/system/photos/2019/10/12/1243405/original.jpg	https://data.globe.gov/system/photos/2019/10/12/1243407/original.jpg
GV_2812	45.4245	-121.6296	1300.6		https://www.google.com/maps/search/45.4245,-121.6296	https://data.globe.gov/system/photos/2019/10/12/1243408/original.jpg	https://data.globe.gov/system/photos/2019/10/12/1243410/original.jpg	https://data.globe.gov/system/photos/2019/10/12/1243409/original.jpg	https://data.globe.gov/system/photos/2019/10/12/1243412/original.jpg	https://data.globe.gov/system/photos/2019/10/12/1243414/original.jpg
GV_2813	45.4247	-121.6321	1310.5		https://www.google.com/maps/search/45.4247,-121.6321	https://data.globe.gov/system/photos/2019/10/12/1243415/original.jpg	https://data.globe.gov/system/photos/2019/10/12/1243418/original.jpg	https://data.globe.gov/system/photos/2019/10/12/1243416/original.jpg	https://data.globe.gov/system/photos/2019/10/12/1243419/original.jpg	https://data.globe.gov/system/photos/2019/10/12/1243421/original.jpg
GV_2814	45.4231	-121.6333	1347.4		https://www.google.com/maps/search/45.4231,-121.6333	https://data.globe.gov/system/photos/2019/10/12/1243422/original.jpg	https://data.globe.gov/system/photos/2019/10/12/1243425/original.jpg	https://data.globe.gov/system/photos/2019/10/12/1243423/original.jpg	https://data.globe.gov/system/photos/2019/10/12/1243426/original.jpg	https://data.globe.gov/system/photos/2019/10/12/1243429/original.jpg
GV_2815	45.4216	-121.6357	1395		https://www.google.com/maps/search/45.4216,-121.6357	https://data.globe.gov/system/photos/2019/10/12/1243432/original.jpg	https://data.globe.gov/system/photos/2019/10/12/1243434/original.jpg	https://data.globe.gov/system/photos/2019/10/12/1243433/original.jpg	https://data.globe.gov/system/photos/2019/10/12/1243435/original.jpg	https://data.globe.gov/system/photos/2019/10/12/1243437/original.jpg
GV_2816	45.4201	-121.6388	1411.7		https://www.google.com/maps/search/45.4201,-121.6388	https://data.globe.gov/system/photos/2019/10/12/1243438/original.jpg	https://data.globe.gov/system/photos/2019/10/12/1243440/original.jpg	https://data.globe.gov/system/photos/2019/10/12/1243439/original.jpg	https://data.globe.gov/system/photos/2019/10/12/1243441/original.jpg	https://data.globe.gov/system/photos/2019/10/12/1243443/original.jpg
GV_2817	45.4188	-121.6417	1442.4		https://www.google.com/maps/search/45.4188,-121.6417	https://data.globe.gov/system/photos/2019/10/12/1243444/original.jpg	https://data.globe.gov/system/photos/2019/10/12/1243446/original.jpg	https://data.globe.gov/system/photos/2019/10/12/1243445/original.jpg	https://data.globe.gov/system/photos/2019/10/12/1243448/original.jpg	https://data.globe.gov/system/photos/2019/10/12/1243450/original.jpg
GV_2818	45.4179	-121.6399	1447.7		https://www.google.com/maps/search/45.4179,-121.6399	https://data.globe.gov/system/photos/2019/10/12/1243452/original.jpg	https://data.globe.gov/system/photos/2019/10/12/1243455/original.jpg	https://data.globe.gov/system/photos/2019/10/12/1243453/original.jpg	https://data.globe.gov/system/photos/2019/10/12/1243456/original.jpg	https://data.globe.gov/system/photos/2019/10/12/1243459/original.jpg
GV_2819	45.4164	-121.6431	1487.6		https://www.google.com/maps/search/45.4164,-121.6431	https://data.globe.gov/system/photos/2019/10/12/1243461/original.jpg	https://data.globe.gov/system/photos/2019/10/12/1243463/original.jpg	https://data.globe.gov/system/photos/2019/10/12/1243462/original.jpg	https://data.globe.gov/system/photos/2019/10/12/1243464/original.jpg	https://data.globe.gov/system/photos/2019/10/12/1243467/original.jpg
GV_2820	45.416	-121.6445	1493.4		https://www.google.com/maps/search/45.416,-121.6445	https://data.globe.gov/system/photos/2019/10/12/1243468/original.jpg	https://data.globe.gov/system/photos/2019/10/12/1243470/original.jpg	https://data.globe.gov/system/photos/2019/10/12/1243469/original.jpg	https://data.globe.gov/system/photos/2019/10/12/1243471/original.jpg	https://data.globe.gov/system/photos/2019/10/12/1243473/original.jpg
GV_2821	45.4161	-121.6502	1513.1		https://www.google.com/maps/search/45.4161,-121.6502	https://data.globe.gov/system/photos/2019/10/12/1243480/original.jpg	https://data.globe.gov/system/photos/2019/10/12/1243482/original.jpg	https://data.globe.gov/system/photos/2019/10/12/1243481/original.jpg	https://data.globe.gov/system/photos/2019/10/12/1243483/original.jpg	https://data.globe.gov/system/photos/2019/10/12/1243485/original.jpg
GV_2822	45.4144	-121.6514	1563.6		https://www.google.com/maps/search/45.4144,-121.6514	https://data.globe.gov/system/photos/2019/10/12/1243486/original.jpg	https://data.globe.gov/system/photos/2019/10/12/1243488/original.jpg	https://data.globe.gov/system/photos/2019/10/12/1243487/original.jpg	https://data.globe.gov/system/photos/2019/10/12/1243489/original.jpg	https://data.globe.gov/system/photos/2019/10/12/1243491/original.jpg
GV_2823	45.4114	-121.6522	1659		https://www.google.com/maps/search/45.4114,-121.6522	https://data.globe.gov/system/photos/2019/10/12/1243492/original.jpg	https://data.globe.gov/system/photos/2019/10/12/1243494/original.jpg	https://data.globe.gov/system/photos/2019/10/12/1243493/original.jpg	https://data.globe.gov/system/photos/2019/10/12/1243495/original.jpg	https://data.globe.gov/system/photos/2019/10/12/1243497/original.jpg
GV_2824	45.4128	-121.6521	1614.1		https://www.google.com/maps/search/45.4128,-121.6521	https://data.globe.gov/system/photos/2019/10/12/1243498/original.jpg	https://data.globe.gov/system/photos/2019/10/12/1243500/original.jpg	https://data.globe.gov/system/photos/2019/10/12/1243499/original.jpg	https://data.globe.gov/system/photos/2019/10/12/1243501/original.jpg	https://data.globe.gov/system/photos/2019/10/12/1243503/original.jpg
GV_2825	45.4119	-121.6522	1644.5		https://www.google.com/maps/search/45.4119,-121.6522	https://data.globe.gov/system/photos/2019/10/12/1243504/original.jpg	https://data.globe.gov/system/photos/2019/10/12/1243506/original.jpg	https://data.globe.gov/system/photos/2019/10/12/1243505/original.jpg	https://data.globe.gov/system/photos/2019/10/12/1243507/original.jpg	https://data.globe.gov/system/photos/2019/10/12/1243509/original.jpg
GV_2826	45.4095	-121.6533	1702.6		https://www.google.com/maps/search/45.4095,-121.6533	https://data.globe.gov/system/photos/2019/10/12/1243510/original.jpg	https://data.globe.gov/system/photos/2019/10/12/1243512/original.jpg	https://data.globe.gov/system/photos/2019/10/12/1243511/original.jpg	https://data.globe.gov/system/photos/2019/10/12/1243513/original.jpg	https://data.globe.gov/system/photos/2019/10/12/1243515/original.jpg
GV_2827	45.4071	-121.6542	1745.5		https://www.google.com/maps/search/45.4071,-121.6542	https://data.globe.gov/system/photos/2019/10/12/1243516/original.jpg	https://data.globe.gov/system/photos/2019/10/12/1243518/original.jpg	https://data.globe.gov/system/photos/2019/10/12/1243517/original.jpg	https://data.globe.gov/system/photos/2019/10/12/1243519/original.jpg	https://data.globe.gov/system/photos/2019/10/12/1243521/original.jpg
GV_2828	45.406	-121.6543	1760		https://www.google.com/maps/search/45.406,-121.6543	https://data.globe.gov/system/photos/2019/10/12/1243522/original.jpg	https://data.globe.gov/system/photos/2019/10/12/1243524/original.jpg	https://data.globe.gov/system/photos/2019/10/12/1243523/original.jpg	https://data.globe.gov/system/photos/2019/10/12/1243525/original.jpg	https://data.globe.gov/system/photos/2019/10/12/1243527/original.jpg
GV_2829	45.4038	-121.6556	1769.1		https://www.google.com/maps/search/45.4038,-121.6556	https://data.globe.gov/system/photos/2019/10/12/1243528/original.jpg	https://data.globe.gov/system/photos/2019/10/12/1243530/original.jpg	https://data.globe.gov/system/photos/2019/10/12/1243529/original.jpg	https://data.globe.gov/system/photos/2019/10/12/1243531/original.jpg	https://data.globe.gov/system/photos/2019/10/12/1243533/original.jpg
GV_2830	45.4024	-121.6548	1783.8		https://www.google.com/maps/search/45.4024,-121.6548	https://data.globe.gov/system/photos/2019/10/12/1243534/original.jpg	https://data.globe.gov/system/photos/2019/10/12/1243536/original.jpg	https://data.globe.gov/system/photos/2019/10/12/1243535/original.jpg	https://data.globe.gov/system/photos/2019/10/12/1243537/original.jpg	https://data.globe.gov/system/photos/2019/10/12/1243539/original.jpg
GV_2831	45.4001	-121.6492	1756.3		https://www.google.com/maps/search/45.4001,-121.6492	https://data.globe.gov/system/photos/2019/10/12/1243540/original.jpg	https://data.globe.gov/system/photos/2019/10/12/1243542/original.jpg	https://data.globe.gov/system/photos/2019/10/12/1243541/original.jpg	https://data.globe.gov/system/photos/2019/10/12/1243543/original.jpg	https://data.globe.gov/system/photos/2019/10/12/1243545/original.jpg
GV_2832	45.398	-121.6423	1716.7		https://www.google.com/maps/search/45.398,-121.6423	https://data.globe.gov/system/photos/2019/10/12/1243546/original.jpg	https://data.globe.gov/system/photos/2019/10/12/1243548/original.jpg	https://data.globe.gov/system/photos/2019/10/12/1243547/original.jpg	https://data.globe.gov/system/photos/2019/10/12/1243549/original.jpg	https://data.globe.gov/system/photos/2019/10/12/1243551/original.jpg
GV_2833	45.4007	-121.6369	1674.5		https://www.google.com/maps/search/45.4007,-121.6369	https://data.globe.gov/system/photos/2019/10/12/1243552/original.jpg	https://data.globe.gov/system/photos/2019/10/12/1243554/original.jpg	https://data.globe.gov/system/photos/2019/10/12/1243553/original.jpg	https://data.globe.gov/system/photos/2019/10/12/1243555/original.jpg	https://data.globe.gov/system/photos/2019/10/12/1243557/original.jpg
GV_2834	45.4022	-121.6326	1588.6		https://www.google.com/maps/search/45.4022,-121.6326	https://data.globe.gov/system/photos/2019/10/12/1243558/original.jpg	https://data.globe.gov/system/photos/2019/10/12/1243560/original.jpg	https://data.globe.gov/system/photos/2019/10/12/1243559/original.jpg	https://data.globe.gov/system/photos/2019/10/12/1243561/original.jpg	https://data.globe.gov/system/photos/2019/10/12/1243563/original.jpg
GV_2835	45.4055	-121.6234	1453.9		https://www.google.com/maps/search/45.4055,-121.6234	https://data.globe.gov/system/photos/2019/10/12/1243564/original.jpg	https://data.globe.gov/system/photos/2019/10/12/1243566/original.jpg	https://data.globe.gov/system/photos/2019/10/12/1243565/original.jpg	https://data.globe.gov/system/photos/2019/10/12/1243567/original.jpg	https://data.globe.gov/system/photos/2019/10/12/1243569/original.jpg
GV_2836	45.4073	-121.6159	1373.6		https://www.google.com/maps/search/45.4073,-121.6159	https://data.globe.gov/system/photos/2019/10/12/1243570/original.jpg	https://data.globe.gov/system/photos/2019/10/12/1243572/original.jpg	https://data.globe.gov/system/photos/2019/10/12/1243571/original.jpg	https://data.globe.gov/system/photos/2019/10/12/1243573/original.jpg	https://data.globe.gov/system/photos/2019/10/12/1243575/original.jpg
GV_2837	45.408	-121.6136	1335		https://www.google.com/maps/search/45.408,-121.6136	https://data.globe.gov/system/photos/2019/10/12/1243576/original.jpg	https://data.globe.gov/system/photos/2019/10/12/1243578/original.jpg	https://data.globe.gov/system/photos/2019/10/12/1243577/original.jpg	https://data.globe.gov/system/photos/2019/10/12/1243579/original.jpg	https://data.globe.gov/system/photos/2019/10/12/1243581/original.jpg
GV_2838	45.4088	-121.6136	1301.1		https://www.google.com/maps/search/45.4088,-121.6136	https://data.globe.gov/system/photos/2019/10/12/1243582/original.jpg	https://data.globe.gov/system/photos/2019/10/12/1243584/original.jpg	https://data.globe.gov/system/photos/2019/10/12/1243583/original.jpg	https://data.globe.gov/system/photos/2019/10/12/1243585/original.jpg	https://data.globe.gov/system/photos/2019/10/12/1243587/original.jpg
GV_2839	45.4119	-121.6104	1251.2		https://www.google.com/maps/search/45.4119,-121.6104	https://data.globe.gov/system/photos/2019/10/12/1243588/original.jpg	https://data.globe.gov/system/photos/2019/10/12/1243590/original.jpg	https://data.globe.gov/system/photos/2019/10/12/1243589/original.jpg	https://data.globe.gov/system/photos/2019/10/12/1243591/original.jpg	https://data.globe.gov/system/photos/2019/10/12/1243593/original.jpg
GV_2840	45.3204	-121.6217	1315.8		https://www.google.com/maps/search/45.3204,-121.6217	https://data.globe.gov/system/photos/2019/10/12/1243594/original.jpg	https://data.globe.gov/system/photos/2019/10/12/1243596/original.jpg	https://data.globe.gov/system/photos/2019/10/12/1243595/original.jpg	https://data.globe.gov/system/photos/2019/10/12/1243597/original.jpg	https://data.globe.gov/system/photos/2019/10/12/1243599/original.jpg
GV_2841	45.31	-121.6419	1427.8		https://www.google.com/maps/search/45.31,-121.6419	https://data.globe.gov/system/photos/2019/10/12/1243600/original.jpg	https://data.globe.gov/system/photos/2019/10/12/1243602/original.jpg	https://data.globe.gov/system/photos/2019/10/12/1243601/original.jpg	https://data.globe.gov/system/photos/2019/10/12/1243603/original.jpg	https://data.globe.gov/system/photos/2019/10/12/1243605/original.jpg
GV_2842	45.63	15.4253	251		https://www.google.com/maps/search/45.63,15.4253	https://data.globe.gov/system/photos/2019/10/13/1243869/original.jpg	https://data.globe.gov/system/photos/2019/10/13/1243871/original.jpg	https://data.globe.gov/system/photos/2019/10/13/1243870/original.jpg	https://data.globe.gov/system/photos/2019/10/13/1243872/original.jpg	https://data.globe.gov/system/photos/2019/10/13/1243874/original.jpg
GV_2843	-25.7537	28.2295	1367.6		https://www.google.com/maps/search/-25.7537,28.2295	https://data.globe.gov/system/photos/2019/10/13/1244025/original.jpg	https://data.globe.gov/system/photos/2019/10/13/1244027/original.jpg	https://data.globe.gov/system/photos/2019/10/13/1244026/original.jpg	https://data.globe.gov/system/photos/2019/10/13/1244029/original.jpg	https://data.globe.gov/system/photos/2019/10/13/1244031/original.jpg
GV_2844	21.2802	81.6884	276.2		https://www.google.com/maps/search/21.2802,81.6884	https://data.globe.gov/system/photos/2019/10/13/1244819/original.jpg	https://data.globe.gov/system/photos/2019/10/13/1244821/original.jpg	https://data.globe.gov/system/photos/2019/10/13/1244820/original.jpg	https://data.globe.gov/system/photos/2019/10/13/1244822/original.jpg	https://data.globe.gov/system/photos/2019/10/13/1244824/original.jpg
GV_2845	39.1857	-86.7781	235.5		https://www.google.com/maps/search/39.1857,-86.7781	https://data.globe.gov/system/photos/2019/10/13/1245216/original.jpg	https://data.globe.gov/system/photos/2019/10/13/1245219/original.jpg	https://data.globe.gov/system/photos/2019/10/13/1245217/original.jpg	https://data.globe.gov/system/photos/2019/10/13/1245227/original.jpg	https://data.globe.gov/system/photos/2019/10/13/1245230/original.jpg
GV_2846	-27.4691	153.1008	5.5		https://www.google.com/maps/search/-27.4691,153.1008	https://data.globe.gov/system/photos/2019/10/14/1245421/original.jpg	https://data.globe.gov/system/photos/2019/10/14/1245423/original.jpg	https://data.globe.gov/system/photos/2019/10/14/1245422/original.jpg	https://data.globe.gov/system/photos/2019/10/14/1245424/original.jpg	https://data.globe.gov/system/photos/2019/10/14/1245426/original.jpg
GV_2847	46.2882	16.3354	171.9		https://www.google.com/maps/search/46.2882,16.3354	https://data.globe.gov/system/photos/2019/10/14/1245946/original.jpg	https://data.globe.gov/system/photos/2019/10/14/1245948/original.jpg	https://data.globe.gov/system/photos/2019/10/14/1245947/original.jpg	https://data.globe.gov/system/photos/2019/10/14/1245949/original.jpg	https://data.globe.gov/system/photos/2019/10/14/1245951/original.jpg
GV_2848	50.4943	21.6445	146		https://www.google.com/maps/search/50.4943,21.6445	https://data.globe.gov/system/photos/2019/10/14/1246665/original.jpg	https://data.globe.gov/system/photos/2019/10/14/1246667/original.jpg	https://data.globe.gov/system/photos/2019/10/14/1246666/original.jpg	https://data.globe.gov/system/photos/2019/10/14/1246668/original.jpg	https://data.globe.gov/system/photos/2019/10/14/1246670/original.jpg
GV_2849	50.4943	21.6445	146		https://www.google.com/maps/search/50.4943,21.6445	https://data.globe.gov/system/photos/2019/10/14/1246671/original.jpg	https://data.globe.gov/system/photos/2019/10/14/1246673/original.jpg	https://data.globe.gov/system/photos/2019/10/14/1246672/original.jpg	https://data.globe.gov/system/photos/2019/10/14/1246674/original.jpg	https://data.globe.gov/system/photos/2019/10/14/1246679/original.jpg
GV_2850	50.4938	21.6412	146.3		https://www.google.com/maps/search/50.4938,21.6412	https://data.globe.gov/system/photos/2019/10/14/1246682/original.jpg	https://data.globe.gov/system/photos/2019/10/14/1246685/original.jpg	https://data.globe.gov/system/photos/2019/10/14/1246684/original.jpg	https://data.globe.gov/system/photos/2019/10/14/1246686/original.jpg	https://data.globe.gov/system/photos/2019/10/14/1246688/original.jpg
GV_2851	50.4952	21.6399	148		https://www.google.com/maps/search/50.4952,21.6399	https://data.globe.gov/system/photos/2019/10/14/1246698/original.jpg	https://data.globe.gov/system/photos/2019/10/14/1246705/original.jpg	https://data.globe.gov/system/photos/2019/10/14/1246700/original.jpg	https://data.globe.gov/system/photos/2019/10/14/1246710/original.jpg	https://data.globe.gov/system/photos/2019/10/14/1246713/original.jpg
GV_2852	50.4928	21.647	146.1		https://www.google.com/maps/search/50.4928,21.647	https://data.globe.gov/system/photos/2019/10/14/1246722/original.jpg	https://data.globe.gov/system/photos/2019/10/14/1246727/original.jpg	https://data.globe.gov/system/photos/2019/10/14/1246723/original.jpg	https://data.globe.gov/system/photos/2019/10/14/1246730/original.jpg	https://data.globe.gov/system/photos/2019/10/14/1246732/original.jpg
GV_2853	50.4924	21.646	146.2		https://www.google.com/maps/search/50.4924,21.646	https://data.globe.gov/system/photos/2019/10/14/1246733/original.jpg	https://data.globe.gov/system/photos/2019/10/14/1246737/original.jpg	https://data.globe.gov/system/photos/2019/10/14/1246734/original.jpg	https://data.globe.gov/system/photos/2019/10/14/1246739/original.jpg	https://data.globe.gov/system/photos/2019/10/14/1246741/original.jpg
GV_2854	50.4922	21.6457	145.6		https://www.google.com/maps/search/50.4922,21.6457	https://data.globe.gov/system/photos/2019/10/14/1246742/original.jpg	https://data.globe.gov/system/photos/2019/10/14/1246744/original.jpg	https://data.globe.gov/system/photos/2019/10/14/1246743/original.jpg	https://data.globe.gov/system/photos/2019/10/14/1246746/original.jpg	https://data.globe.gov/system/photos/2019/10/14/1246749/original.jpg
GV_2855	50.4929	21.6457	145.4		https://www.google.com/maps/search/50.4929,21.6457	https://data.globe.gov/system/photos/2019/10/14/1246750/original.jpg	https://data.globe.gov/system/photos/2019/10/14/1246752/original.jpg	https://data.globe.gov/system/photos/2019/10/14/1246751/original.jpg	https://data.globe.gov/system/photos/2019/10/14/1246753/original.jpg	https://data.globe.gov/system/photos/2019/10/14/1246755/original.jpg
GV_2856	50.4936	21.6452	145.6		https://www.google.com/maps/search/50.4936,21.6452	https://data.globe.gov/system/photos/2019/10/14/1246756/original.jpg	https://data.globe.gov/system/photos/2019/10/14/1246758/original.jpg	https://data.globe.gov/system/photos/2019/10/14/1246757/original.jpg	https://data.globe.gov/system/photos/2019/10/14/1246759/original.jpg	https://data.globe.gov/system/photos/2019/10/14/1246761/original.jpg
GV_2857	50.4926	21.6548	150.7		https://www.google.com/maps/search/50.4926,21.6548	https://data.globe.gov/system/photos/2019/10/14/1246781/original.jpg	https://data.globe.gov/system/photos/2019/10/14/1246783/original.jpg	https://data.globe.gov/system/photos/2019/10/14/1246782/original.jpg	https://data.globe.gov/system/photos/2019/10/14/1246784/original.jpg	https://data.globe.gov/system/photos/2019/10/14/1246786/original.jpg
GV_2858	50.4941	21.65	146		https://www.google.com/maps/search/50.4941,21.65	https://data.globe.gov/system/photos/2019/10/14/1246787/original.jpg	https://data.globe.gov/system/photos/2019/10/14/1246789/original.jpg	https://data.globe.gov/system/photos/2019/10/14/1246788/original.jpg	https://data.globe.gov/system/photos/2019/10/14/1246790/original.jpg	https://data.globe.gov/system/photos/2019/10/14/1246792/original.jpg
GV_2859	50.4941	21.6489	146		https://www.google.com/maps/search/50.4941,21.6489	https://data.globe.gov/system/photos/2019/10/14/1246793/original.jpg	https://data.globe.gov/system/photos/2019/10/14/1246795/original.jpg	https://data.globe.gov/system/photos/2019/10/14/1246794/original.jpg	https://data.globe.gov/system/photos/2019/10/14/1246796/original.jpg	https://data.globe.gov/system/photos/2019/10/14/1246798/original.jpg
GV_2860	50.4944	21.6485	146		https://www.google.com/maps/search/50.4944,21.6485	https://data.globe.gov/system/photos/2019/10/14/1246799/original.jpg	https://data.globe.gov/system/photos/2019/10/14/1246801/original.jpg	https://data.globe.gov/system/photos/2019/10/14/1246800/original.jpg	https://data.globe.gov/system/photos/2019/10/14/1246802/original.jpg	https://data.globe.gov/system/photos/2019/10/14/1246804/original.jpg
GV_2861	50.4929	21.6481	145.9		https://www.google.com/maps/search/50.4929,21.6481	https://data.globe.gov/system/photos/2019/10/14/1246805/original.jpg	https://data.globe.gov/system/photos/2019/10/14/1246807/original.jpg	https://data.globe.gov/system/photos/2019/10/14/1246806/original.jpg	https://data.globe.gov/system/photos/2019/10/14/1246809/original.jpg	https://data.globe.gov/system/photos/2019/10/14/1246815/original.jpg
GV_2862	45.4861	15.5442	109.2		https://www.google.com/maps/search/45.4861,15.5442	https://data.globe.gov/system/photos/2019/10/14/1246909/original.jpg	https://data.globe.gov/system/photos/2019/10/14/1246911/original.jpg	https://data.globe.gov/system/photos/2019/10/14/1246910/original.jpg	https://data.globe.gov/system/photos/2019/10/14/1246912/original.jpg	https://data.globe.gov/system/photos/2019/10/14/1246915/original.jpg
GV_2863	39.1859	-86.7783	233.2		https://www.google.com/maps/search/39.1859,-86.7783	https://data.globe.gov/system/photos/2019/10/14/1248279/original.jpg	https://data.globe.gov/system/photos/2019/10/14/1248281/original.jpg	https://data.globe.gov/system/photos/2019/10/14/1248280/original.jpg	https://data.globe.gov/system/photos/2019/10/14/1248282/original.jpg	https://data.globe.gov/system/photos/2019/10/14/1248284/original.jpg
GV_2864	44.4106	-122.6543	199.5		https://www.google.com/maps/search/44.4106,-122.6543	https://data.globe.gov/system/photos/2019/10/14/1248792/original.jpg	https://data.globe.gov/system/photos/2019/10/14/1248794/original.jpg	https://data.globe.gov/system/photos/2019/10/14/1248793/original.jpg	https://data.globe.gov/system/photos/2019/10/14/1248795/original.jpg	https://data.globe.gov/system/photos/2019/10/14/1248797/original.jpg
GV_2865	44.3914	-122.5107	243		https://www.google.com/maps/search/44.3914,-122.5107	https://data.globe.gov/system/photos/2019/10/14/1248798/original.jpg	https://data.globe.gov/system/photos/2019/10/14/1248800/original.jpg	https://data.globe.gov/system/photos/2019/10/14/1248799/original.jpg	https://data.globe.gov/system/photos/2019/10/14/1248801/original.jpg	https://data.globe.gov/system/photos/2019/10/14/1248803/original.jpg
GV_2866	44.3912	-122.5108	243.2		https://www.google.com/maps/search/44.3912,-122.5108	https://data.globe.gov/system/photos/2019/10/14/1248804/original.jpg	https://data.globe.gov/system/photos/2019/10/14/1248806/original.jpg	https://data.globe.gov/system/photos/2019/10/14/1248805/original.jpg	https://data.globe.gov/system/photos/2019/10/14/1248807/original.jpg	https://data.globe.gov/system/photos/2019/10/14/1248809/original.jpg
GV_2867	44.4033	-122.3795	336.9		https://www.google.com/maps/search/44.4033,-122.3795	https://data.globe.gov/system/photos/2019/10/15/1248810/original.jpg	https://data.globe.gov/system/photos/2019/10/15/1248812/original.jpg	https://data.globe.gov/system/photos/2019/10/15/1248811/original.jpg	https://data.globe.gov/system/photos/2019/10/15/1248813/original.jpg	https://data.globe.gov/system/photos/2019/10/15/1248815/original.jpg
GV_2868	44.399	-122.3475	377.4		https://www.google.com/maps/search/44.399,-122.3475	https://data.globe.gov/system/photos/2019/10/15/1248822/original.jpg	https://data.globe.gov/system/photos/2019/10/15/1248824/original.jpg	https://data.globe.gov/system/photos/2019/10/15/1248823/original.jpg	https://data.globe.gov/system/photos/2019/10/15/1248825/original.jpg	https://data.globe.gov/system/photos/2019/10/15/1248827/original.jpg
GV_2869	44.3933	-122.2455	504.8		https://www.google.com/maps/search/44.3933,-122.2455	https://data.globe.gov/system/photos/2019/10/15/1248828/original.jpg	https://data.globe.gov/system/photos/2019/10/15/1248830/original.jpg	https://data.globe.gov/system/photos/2019/10/15/1248829/original.jpg	https://data.globe.gov/system/photos/2019/10/15/1248831/original.jpg	https://data.globe.gov/system/photos/2019/10/15/1248833/original.jpg
GV_2870	44.3958	-122.1412	1292		https://www.google.com/maps/search/44.3958,-122.1412	https://data.globe.gov/system/photos/2019/10/15/1248834/original.jpg	https://data.globe.gov/system/photos/2019/10/15/1248836/original.jpg	https://data.globe.gov/system/photos/2019/10/15/1248835/original.jpg	https://data.globe.gov/system/photos/2019/10/15/1248837/original.jpg	https://data.globe.gov/system/photos/2019/10/15/1248839/original.jpg
GV_2871	44.4165	-122.012	1053.7		https://www.google.com/maps/search/44.4165,-122.012	https://data.globe.gov/system/photos/2019/10/15/1248841/original.jpg	https://data.globe.gov/system/photos/2019/10/15/1248843/original.jpg	https://data.globe.gov/system/photos/2019/10/15/1248842/original.jpg	https://data.globe.gov/system/photos/2019/10/15/1248844/original.jpg	https://data.globe.gov/system/photos/2019/10/15/1248846/original.jpg
GV_2872	44.4164	-122.0116	1055.5		https://www.google.com/maps/search/44.4164,-122.0116	https://data.globe.gov/system/photos/2019/10/15/1248848/original.jpg	https://data.globe.gov/system/photos/2019/10/15/1248850/original.jpg	https://data.globe.gov/system/photos/2019/10/15/1248849/original.jpg	https://data.globe.gov/system/photos/2019/10/15/1248851/original.jpg	https://data.globe.gov/system/photos/2019/10/15/1248853/original.jpg
GV_2873	44.4161	-122.0034	1010.4		https://www.google.com/maps/search/44.4161,-122.0034	https://data.globe.gov/system/photos/2019/10/15/1248854/original.jpg	https://data.globe.gov/system/photos/2019/10/15/1248856/original.jpg	https://data.globe.gov/system/photos/2019/10/15/1248855/original.jpg	https://data.globe.gov/system/photos/2019/10/15/1248857/original.jpg	https://data.globe.gov/system/photos/2019/10/15/1248859/original.jpg

GV_2874	44.461	-121.9764	1127.1		https://www.google.com/maps/search/44.461,-121.9764	https://data.globe.gov/system/photos/2019/10/15/1248860/original.jpg	https://data.globe.gov/system/photos/2019/10/15/1248862/original.jpg	https://data.globe.gov/system/photos/2019/10/15/1248861/original.jpg	https://data.globe.gov/system/photos/2019/10/15/1248863/original.jpg	https://data.globe.gov/system/photos/2019/10/15/1248865/original.jpg
GV_2875	44.5971	-121.9691	784		https://www.google.com/maps/search/44.5971,-121.9691	https://data.globe.gov/system/photos/2019/10/15/1248866/original.jpg	https://data.globe.gov/system/photos/2019/10/15/1248868/original.jpg	https://data.globe.gov/system/photos/2019/10/15/1248867/original.jpg	https://data.globe.gov/system/photos/2019/10/15/1248869/original.jpg	https://data.globe.gov/system/photos/2019/10/15/1248871/original.jpg
GV_2876	44.6371	-121.9409	721.2		https://www.google.com/maps/search/44.6371,-121.9409	https://data.globe.gov/system/photos/2019/10/15/1248872/original.jpg	https://data.globe.gov/system/photos/2019/10/15/1248874/original.jpg	https://data.globe.gov/system/photos/2019/10/15/1248873/original.jpg	https://data.globe.gov/system/photos/2019/10/15/1248875/original.jpg	https://data.globe.gov/system/photos/2019/10/15/1248877/original.jpg
GV_2877	50.4968	21.6825	147		https://www.google.com/maps/search/50.4968,21.6825	https://data.globe.gov/system/photos/2019/10/12/1250175/original.jpg	https://data.globe.gov/system/photos/2019/10/12/1250178/original.jpg	https://data.globe.gov/system/photos/2019/10/12/1250177/original.jpg	https://data.globe.gov/system/photos/2019/10/12/1250180/original.jpg	https://data.globe.gov/system/photos/2019/10/12/1250183/original.jpg
GV_2878	50.4966	21.6825	147		https://www.google.com/maps/search/50.4966,21.6825	https://data.globe.gov/system/photos/2019/10/12/1250184/original.jpg	https://data.globe.gov/system/photos/2019/10/12/1250186/original.jpg	https://data.globe.gov/system/photos/2019/10/12/1250185/original.jpg	https://data.globe.gov/system/photos/2019/10/12/1250187/original.jpg	https://data.globe.gov/system/photos/2019/10/12/1250189/original.jpg
GV_2879	50.4966	21.6825	147		https://www.google.com/maps/search/50.4966,21.6825	https://data.globe.gov/system/photos/2019/10/13/1250190/original.jpg	https://data.globe.gov/system/photos/2019/10/13/1250192/original.jpg	https://data.globe.gov/system/photos/2019/10/13/1250191/original.jpg	https://data.globe.gov/system/photos/2019/10/13/1250193/original.jpg	https://data.globe.gov/system/photos/2019/10/13/1250195/original.jpg
GV_2880	50.4955	21.6837	149		https://www.google.com/maps/search/50.4955,21.6837	https://data.globe.gov/system/photos/2019/10/14/1250196/original.jpg	https://data.globe.gov/system/photos/2019/10/14/1250199/original.jpg	https://data.globe.gov/system/photos/2019/10/14/1250197/original.jpg	https://data.globe.gov/system/photos/2019/10/14/1250201/original.jpg	https://data.globe.gov/system/photos/2019/10/14/1250205/original.jpg
GV_2881	45.4874	15.5446	110.6		https://www.google.com/maps/search/45.4874,15.5446	https://data.globe.gov/system/photos/2019/10/15/1250227/original.jpg	https://data.globe.gov/system/photos/2019/10/15/1250229/original.jpg	https://data.globe.gov/system/photos/2019/10/15/1250228/original.jpg	https://data.globe.gov/system/photos/2019/10/15/1250230/original.jpg	https://data.globe.gov/system/photos/2019/10/15/1250232/original.jpg
GV_2882	40.0086	-75.0417	3.1		https://www.google.com/maps/search/40.0086,-75.0417	https://data.globe.gov/system/photos/2019/10/15/1250491/original.jpg	https://data.globe.gov/system/photos/2019/10/15/1250493/original.jpg	https://data.globe.gov/system/photos/2019/10/15/1250492/original.jpg	https://data.globe.gov/system/photos/2019/10/15/1250494/original.jpg	https://data.globe.gov/system/photos/2019/10/15/1250496/original.jpg
GV_2883	40.0086	-75.0417	3.1		https://www.google.com/maps/search/40.0086,-75.0417	https://data.globe.gov/system/photos/2019/10/15/1250527/original.jpg	https://data.globe.gov/system/photos/2019/10/15/1250532/original.jpg	https://data.globe.gov/system/photos/2019/10/15/1250528/original.jpg	https://data.globe.gov/system/photos/2019/10/15/1250533/original.jpg	https://data.globe.gov/system/photos/2019/10/15/1250541/original.jpg
GV_2884	38.9933	-76.8398	62.8		https://www.google.com/maps/search/38.9933,-76.8398	https://data.globe.gov/system/photos/2019/10/15/1250695/original.jpg	https://data.globe.gov/system/photos/2019/10/15/1250697/original.jpg	https://data.globe.gov/system/photos/2019/10/15/1250696/original.jpg	https://data.globe.gov/system/photos/2019/10/15/1250698/original.jpg	https://data.globe.gov/system/photos/2019/10/15/1250700/original.jpg
GV_2885	50.4868	21.6645	147.2		https://www.google.com/maps/search/50.4868,21.6645	https://data.globe.gov/system/photos/2019/10/15/1250955/original.jpg	https://data.globe.gov/system/photos/2019/10/15/1250960/original.jpg	https://data.globe.gov/system/photos/2019/10/15/1250956/original.jpg	https://data.globe.gov/system/photos/2019/10/15/1250964/original.jpg	https://data.globe.gov/system/photos/2019/10/15/1250966/original.jpg
GV_2886	50.4871	21.6629	147		https://www.google.com/maps/search/50.4871,21.6629	https://data.globe.gov/system/photos/2019/10/15/1250967/original.jpg	https://data.globe.gov/system/photos/2019/10/15/1250971/original.jpg	https://data.globe.gov/system/photos/2019/10/15/1250968/original.jpg	https://data.globe.gov/system/photos/2019/10/15/1250974/original.jpg	https://data.globe.gov/system/photos/2019/10/15/1250982/original.jpg
GV_2887	50.4861	21.6618	147		https://www.google.com/maps/search/50.4861,21.6618	https://data.globe.gov/system/photos/2019/10/15/1250984/original.jpg	https://data.globe.gov/system/photos/2019/10/15/1250986/original.jpg	https://data.globe.gov/system/photos/2019/10/15/1250985/original.jpg	https://data.globe.gov/system/photos/2019/10/15/1250987/original.jpg	https://data.globe.gov/system/photos/2019/10/15/1250996/original.jpg
GV_2888	50.4864	21.6599	147		https://www.google.com/maps/search/50.4864,21.6599	https://data.globe.gov/system/photos/2019/10/15/1251005/original.jpg	https://data.globe.gov/system/photos/2019/10/15/1251009/original.jpg	https://data.globe.gov/system/photos/2019/10/15/1251008/original.jpg	https://data.globe.gov/system/photos/2019/10/15/1251010/original.jpg	https://data.globe.gov/system/photos/2019/10/15/1251023/original.jpg
GV_2889	50.4867	21.6585	147		https://www.google.com/maps/search/50.4867,21.6585	https://data.globe.gov/system/photos/2019/10/15/1251027/original.jpg	https://data.globe.gov/system/photos/2019/10/15/1251031/original.jpg	https://data.globe.gov/system/photos/2019/10/15/1251030/original.jpg	https://data.globe.gov/system/photos/2019/10/15/1251032/original.jpg	https://data.globe.gov/system/photos/2019/10/15/1251036/original.jpg
GV_2890	50.4871	21.655	146.2		https://www.google.com/maps/search/50.4871,21.655	https://data.globe.gov/system/photos/2019/10/15/1251037/original.jpg	https://data.globe.gov/system/photos/2019/10/15/1251041/original.jpg	https://data.globe.gov/system/photos/2019/10/15/1251039/original.jpg	https://data.globe.gov/system/photos/2019/10/15/1251043/original.jpg	https://data.globe.gov/system/photos/2019/10/15/1251048/original.jpg
GV_2891	50.4865	21.6542	146		https://www.google.com/maps/search/50.4865,21.6542	https://data.globe.gov/system/photos/2019/10/15/1251050/original.jpg	https://data.globe.gov/system/photos/2019/10/15/1251057/original.jpg	https://data.globe.gov/system/photos/2019/10/15/1251053/original.jpg	https://data.globe.gov/system/photos/2019/10/15/1251061/original.jpg	https://data.globe.gov/system/photos/2019/10/15/1251066/original.jpg
GV_2892	50.4857	21.6532	146.9		https://www.google.com/maps/search/50.4857,21.6532	https://data.globe.gov/system/photos/2019/10/15/1251068/original.jpg	https://data.globe.gov/system/photos/2019/10/15/1251077/original.jpg	https://data.globe.gov/system/photos/2019/10/15/1251071/original.jpg	https://data.globe.gov/system/photos/2019/10/15/1251083/original.jpg	https://data.globe.gov/system/photos/2019/10/15/1251086/original.jpg
GV_2893	50.485	21.6523	147		https://www.google.com/maps/search/50.485,21.6523	https://data.globe.gov/system/photos/2019/10/15/1251094/original.jpg	https://data.globe.gov/system/photos/2019/10/15/1251116/original.jpg	https://data.globe.gov/system/photos/2019/10/15/1251115/original.jpg	https://data.globe.gov/system/photos/2019/10/15/1251117/original.jpg	https://data.globe.gov/system/photos/2019/10/15/1251119/original.jpg
GV_2894	50.4845	21.651	147		https://www.google.com/maps/search/50.4845,21.651	https://data.globe.gov/system/photos/2019/10/15/1251120/original.jpg	https://data.globe.gov/system/photos/2019/10/15/1251122/original.jpg	https://data.globe.gov/system/photos/2019/10/15/1251121/original.jpg	https://data.globe.gov/system/photos/2019/10/15/1251123/original.jpg	https://data.globe.gov/system/photos/2019/10/15/1251125/original.jpg
GV_2895	50.484	21.6509	147		https://www.google.com/maps/search/50.484,21.6509	https://data.globe.gov/system/photos/2019/10/15/1251126/original.jpg	https://data.globe.gov/system/photos/2019/10/15/1251128/original.jpg	https://data.globe.gov/system/photos/2019/10/15/1251127/original.jpg	https://data.globe.gov/system/photos/2019/10/15/1251129/original.jpg	https://data.globe.gov/system/photos/2019/10/15/1251131/original.jpg
GV_2896	50.4833	21.649	147		https://www.google.com/maps/search/50.4833,21.649	https://data.globe.gov/system/photos/2019/10/15/1251132/original.jpg	https://data.globe.gov/system/photos/2019/10/15/1251134/original.jpg	https://data.globe.gov/system/photos/2019/10/15/1251133/original.jpg	https://data.globe.gov/system/photos/2019/10/15/1251135/original.jpg	https://data.globe.gov/system/photos/2019/10/15/1251138/original.jpg
GV_2897	50.4839	21.6477	146.9		https://www.google.com/maps/search/50.4839,21.6477	https://data.globe.gov/system/photos/2019/10/15/1251139/original.jpg	https://data.globe.gov/system/photos/2019/10/15/1251145/original.jpg	https://data.globe.gov/system/photos/2019/10/15/1251140/original.jpg	https://data.globe.gov/system/photos/2019/10/15/1251149/original.jpg	https://data.globe.gov/system/photos/2019/10/15/1251152/original.jpg
GV_2898	50.4843	21.6469	146.9		https://www.google.com/maps/search/50.4843,21.6469	https://data.globe.gov/system/photos/2019/10/15/1251153/original.jpg	https://data.globe.gov/system/photos/2019/10/15/1251155/original.jpg	https://data.globe.gov/system/photos/2019/10/15/1251154/original.jpg	https://data.globe.gov/system/photos/2019/10/15/1251156/original.jpg	https://data.globe.gov/system/photos/2019/10/15/1251158/original.jpg
GV_2899	50.485	21.6451	147		https://www.google.com/maps/search/50.485,21.6451	https://data.globe.gov/system/photos/2019/10/15/1251159/original.jpg	https://data.globe.gov/system/photos/2019/10/15/1251161/original.jpg	https://data.globe.gov/system/photos/2019/10/15/1251160/original.jpg	https://data.globe.gov/system/photos/2019/10/15/1251162/original.jpg	https://data.globe.gov/system/photos/2019/10/15/1251164/original.jpg
GV_2900	39.1862	-86.7783	228.1		https://www.google.com/maps/search/39.1862,-86.7783	https://data.globe.gov/system/photos/2019/10/15/1253064/original.jpg	https://data.globe.gov/system/photos/2019/10/15/1253066/original.jpg	https://data.globe.gov/system/photos/2019/10/15/1253065/original.jpg	https://data.globe.gov/system/photos/2019/10/15/1253068/original.jpg	https://data.globe.gov/system/photos/2019/10/15/1253075/original.jpg
GV_2901	42.1544	-83.1512	177.1		https://www.google.com/maps/search/42.1544,-83.1512	https://data.globe.gov/system/photos/2019/10/06/1253293/original.jpg	https://data.globe.gov/system/photos/2019/10/06/1253296/original.jpg	https://data.globe.gov/system/photos/2019/10/06/1253294/original.jpg	https://data.globe.gov/system/photos/2019/10/06/1253298/original.jpg	https://data.globe.gov/system/photos/2019/10/06/1253301/original.jpg
GV_2902	36.8176	-121.731	32.3		https://www.google.com/maps/search/36.8176,-121.731	https://data.globe.gov/system/photos/2019/10/12/1257226/original.jpg	https://data.globe.gov/system/photos/2019/10/12/1257228/original.jpg	https://data.globe.gov/system/photos/2019/10/12/1257227/original.jpg	https://data.globe.gov/system/photos/2019/10/12/1257231/original.jpg	https://data.globe.gov/system/photos/2019/10/12/1257233/original.jpg
GV_2903	36.8176	-121.7308	33.9		https://www.google.com/maps/search/36.8176,-121.7308	https://data.globe.gov/system/photos/2019/10/12/1257234/original.jpg	https://data.globe.gov/system/photos/2019/10/12/1257238/original.jpg	https://data.globe.gov/system/photos/2019/10/12/1257237/original.jpg	https://data.globe.gov/system/photos/2019/10/12/1257239/original.jpg	https://data.globe.gov/system/photos/2019/10/12/1257241/original.jpg
GV_2904	36.8176	-121.7307	34.5		https://www.google.com/maps/search/36.8176,-121.7307	https://data.globe.gov/system/photos/2019/10/12/1257242/original.jpg	https://data.globe.gov/system/photos/2019/10/12/1257246/original.jpg	https://data.globe.gov/system/photos/2019/10/12/1257245/original.jpg	https://data.globe.gov/system/photos/2019/10/12/1257247/original.jpg	https://data.globe.gov/system/photos/2019/10/12/1257249/original.jpg
GV_2905	36.8181	-121.7311	25.9		https://www.google.com/maps/search/36.8181,-121.7311	https://data.globe.gov/system/photos/2019/10/12/1257250/original.jpg	https://data.globe.gov/system/photos/2019/10/12/1257252/original.jpg	https://data.globe.gov/system/photos/2019/10/12/1257251/original.jpg	https://data.globe.gov/system/photos/2019/10/12/1257253/original.jpg	https://data.globe.gov/system/photos/2019/10/12/1257263/original.jpg
GV_2906	39.9958	-82.858	236.3		https://www.google.com/maps/search/39.9958,-82.858	https://data.globe.gov/system/photos/2019/10/06/1274470/original.jpg	https://data.globe.gov/system/photos/2019/10/06/1274472/original.jpg	https://data.globe.gov/system/photos/2019/10/06/1274471/original.jpg	https://data.globe.gov/system/photos/2019/10/06/1274473/original.jpg	https://data.globe.gov/system/photos/2019/10/06/1274476/original.jpg
GV_2907	50.4973	21.6863	148.9		https://www.google.com/maps/search/50.4973,21.6863	https://data.globe.gov/system/photos/2019/10/15/1277513/original.jpg	https://data.globe.gov/system/photos/2019/10/15/1277515/original.jpg	https://data.globe.gov/system/photos/2019/10/15/1277514/original.jpg	https://data.globe.gov/system/photos/2019/10/15/1277516/original.jpg	https://data.globe.gov/system/photos/2019/10/15/1277518/original.jpg
GV_2908	44.4154	-123.3367	121.7		https://www.google.com/maps/search/44.4154,-123.3367	https://data.globe.gov/system/photos/2019/10/04/1289129/original.jpg	https://data.globe.gov/system/photos/2019/10/04/1289131/original.jpg	https://data.globe.gov/system/photos/2019/10/04/1289130/original.jpg	https://data.globe.gov/system/photos/2019/10/04/1289132/original.jpg	https://data.globe.gov/system/photos/2019/10/04/1289134/original.jpg
GV_2909	44.5639	-123.2794	76.1		https://www.google.com/maps/search/44.5639,-123.2794	https://data.globe.gov/system/photos/2019/10/05/1289138/original.jpg	https://data.globe.gov/system/photos/2019/10/05/1289140/original.jpg	https://data.globe.gov/system/photos/2019/10/05/1289139/original.jpg	https://data.globe.gov/system/photos/2019/10/05/1289141/original.jpg	https://data.globe.gov/system/photos/2019/10/05/1289143/original.jpg
GV_2910	38.5696	-121.5022	4.1		https://www.google.com/maps/search/38.5696,-121.5022	https://data.globe.gov/system/photos/2019/09/27/1417051/original.jpg	https://data.globe.gov/system/photos/2019/09/27/1417053/original.jpg	https://data.globe.gov/system/photos/2019/09/27/1417052/original.jpg	https://data.globe.gov/system/photos/2019/09/27/1417056/original.jpg	https://data.globe.gov/system/photos/2019/09/27/1417060/original.jpg
GV_2911	34.6465	-78.9767	43.7		https://www.google.com/maps/search/34.6465,-78.9767	https://data.globe.gov/system/photos/2019/09/24/1419647/original.jpg	https://data.globe.gov/system/photos/2019/09/24/1419649/original.jpg	https://data.globe.gov/system/photos/2019/09/24/1419648/original.jpg	https://data.globe.gov/system/photos/2019/09/24/1419650/original.jpg	https://data.globe.gov/system/photos/2019/09/24/1419652/original.jpg
GV_2912	15.2476	104.8481	127		https://www.google.com/maps/search/15.2476,104.8481	https://data.globe.gov/system/photos/2019/07/01/1466334/original.jpg	https://data.globe.gov/system/photos/2019/07/01/1466336/original.jpg	https://data.globe.gov/system/photos/2019/07/01/1466335/original.jpg	https://data.globe.gov/system/photos/2019/07/01/1466337/original.jpg	https://data.globe.gov/system/photos/2019/07/01/1466339/original.jpg
GV_2913	46.2084	6.1632	372.2		https://www.google.com/maps/search/46.2084,6.1632	https://data.globe.gov/system/photos/2019/06/16/1470567/original.jpg	https://data.globe.gov/system/photos/2019/06/16/1470569/original.jpg	https://data.globe.gov/system/photos/2019/06/16/1470568/original.jpg	https://data.globe.gov/system/photos/2019/06/16/1470570/original.jpg	https://data.globe.gov/system/photos/2019/06/16/1470572/original.jpg
GV_2914	-1.253	36.8571	1636.4		https://www.google.com/maps/search/-1.253,36.8571	https://data.globe.gov/system/photos/2019/07/10/1475434/original.jpg	https://data.globe.gov/system/photos/2019/07/10/1475436/original.jpg	https://data.globe.gov/system/photos/2019/07/10/1475435/original.jpg	https://data.globe.gov/system/photos/2019/07/10/1475439/original.jpg	https://data.globe.gov/system/photos/2019/07/10/1475442/original.jpg
GV_2915	44.4124	-123.3324	91.1		https://www.google.com/maps/search/44.4124,-123.3324	https://data.globe.gov/system/photos/2019/10/04/1485224/original.jpg	https://data.globe.gov/system/photos/2019/10/04/1485226/original.jpg	https://data.globe.gov/system/photos/2019/10/04/1485225/original.jpg	https://data.globe.gov/system/photos/2019/10/04/1485227/original.jpg	https://data.globe.gov/system/photos/2019/10/04/1485229/original.jpg
